# Metabolomic profiles associated with a mouse model of antipsychotic-induced food intake and weight gain

**DOI:** 10.1038/s41598-020-75624-2

**Published:** 2020-10-29

**Authors:** Rizaldy C. Zapata, Sara Brin Rosenthal, Kathleen Fisch, Khoi Dao, Mohit Jain, Olivia Osborn

**Affiliations:** 1 Division of Endocrinology and Metabolism, School of Medicine, University of California San Diego, 9500 Gilman Drive Mail Code 0673, La Jolla, CA 92093 USA; 2 Center for Computational Biology and Bioinformatics, School of Medicine, University of California San Diego, La Jolla, CA USA; 3 Department of Medicine and Pharmacology, University of California San Diego, La Jolla, CA 92093 USA

**Keywords:** Hypothalamus, Obesity, Preclinical research

## Abstract

Antipsychotic drugs (AP) are used to treat a multitude of psychiatric conditions including schizophrenia and bipolar disorder. However, APs also have metabolic side effects including increased food intake and body weight, but the underlying mechanisms remain unknown. We previously reported that minocycline (MINO) co-treatment abrogates olanzapine (OLZ)-induced hyperphagia and weight gain in mice. Using this model, we investigated the changes in the pharmacometabolome in the plasma and hypothalamus associated with OLZ-induced hyperphagia and weight gain. Female C57BL/6 mice were divided into groups and fed either i) control, CON (45% fat diet) ii) CON + MINO, iii) OLZ (45% fat diet with OLZ), iv) OLZ + MINO. We identified one hypothalamic metabolite indoxylsulfuric acid and 389 plasma metabolites (including 19 known metabolites) that were specifically associated with AP-induced hyperphagia and weight gain in mice. We found that plasma citrulline, tricosenoic acid, docosadienoic acid and palmitoleic acid were increased while serine, asparagine and arachidonic acid and its derivatives were decreased in response to OLZ. These changes were specifically blocked by co-treatment with MINO. These pharmacometabolomic profiles associated with AP-induced hyperphagia and weight gain provide candidate biomarkers and mechanistic insights related to the metabolic side effects of these widely used drugs.

## Introduction

Second generation antipsychotic drugs (AP) are used to treat a multitude of conditions including, but not limited to, schizophrenia and bipolar disorder^1–4^. However, while they are effective in treating these psychiatric diseases, they also have prominent side effects including increasing food intake^5–7^ and body weight, leading to obesity and associated cardiometabolic diseases^8–10^. Notably, the incidence of diabetes among AP users is four times higher than matched controls^11^. Olanzapine (OLZ) is regarded as one of the clinically most effective APs^12^ but approximately 20–40% of patients taking OLZ gain a clinically significant amount of weight^13–15^. The mechanisms underlying AP-induced weight gain may involve several different peptides, neurotransmitters and receptors in the appetite and reward systems in the brain^16, 17^. In addition, underlying genetics are believed to play a role in predicting individual susceptibility to AP-induced weight gain but further detailed studies are needed to facilitate prediction tests in larger data sets^18, 19^. Therefore, despite the prominent weight gain side effects, the underlying mechanisms driving AP-induced weight gain remain largely unknown.

To mitigate the adverse metabolic actions of APs, multiple compounds have been investigated as add-on medications to blunt AP-induced weight gain^20, 21^. Adjunctive metformin is widely prescribed with APs^22, 23^ to treat metabolic side effects but has limited efficacy in blunting weight gain^24^. Minocycline (MINO), a second-generation tetracycline antibiotic, has been reported to have neuroprotective and anti-inflammatory properties that maybe beneficial to patients with psychiatric disorders^25–27^. We recently reported that minocycline (MINO) co-treatment blocks OLZ-induced hyperphagia and weight gain in mice^28^. Importantly, we showed that MINO co-treatment did not interfere with the therapeutic efficacy of olanzapine. In addition, we identified a hypothalamic gene expression signature that was specifically associated with OLZ-induced increase in food intake and reversed with MINO co-administration^28^. The use of MINO as a ‘tool’ to block AP-induced side effects enables the identification of AP-induced pathways implicated in hyperphagia and weight gain.

Metabolomics is a rapidly expanding technology based on either NMR or LC/GC-based mass spectrometry which allows for the identification of complex metabolite profiles and establish their correlation with disease. Data derived from metabolomics can provide a deeper mechanistic understanding of drug-induced changes induced by APs at the metabolite level and also identify biomarkers of efficacy and side-effects to further facilitate clinical trials during the evaluation of future AP therapies. Here, using metabolomics, we determined the specific AP-induced biomarkers of weight gain and hyperphagia in the plasma and hypothalamus of OLZ-treated mice.

## Methods

### Mouse studies

All procedures were approved by UCSD IACUC and all experiments were performed in accordance with relevant guidelines and regulations. Female C57BL/6 (stock #000,664) were purchased from Jackson Labs at 9 weeks of age and were acclimatized to the experimental environment (12:12 light/dark, 20–22 °C, 60% humidity) with normal chow until 10 weeks of age. Mice were then randomized to four treatment groups: control (CON), OLZ, MINO, and OLZ + MINO. CON animals were fed a high fat diet (HFD, 45 kcal% from fat, Research Diets, Inc., D09092903, New Brunswick, NJ). OLZ was compounded into the HFD at a concentration of 54 mg/kg of diet^28, 29^. MINO was administered in drinking water at a dose of 0.6 mg/ml^28, 30^. After 2 weeks of treatment, mice were sacrificed and blood collected in heparinized capillaries followed by centrifuge at 12,000 rpm for 10 min at 4 °C to collect plasma. Hypothalami were dissected and snap-frozen in liquid nitrogen. Samples were stored at -80 °C until analysis. Four to five samples per group were processed for metabolomics analysis.

### Untargeted metabolomics

Metabolomics analysis was performed on collected mouse plasma and hypothalamus samples as previously described^31–33^. Briefly, small bioactive lipid metabolites and polar, hydrophilic metabolites were extracted from plasma samples using addition of organic solvent followed by offline solid phase extraction (SPE), as described^31–33^. Metabolites were chromatographically separated using a Thermo Vanquish UPLC system with a Phenomenex Kinetex C18 (1.7um particle size, 100 × 2.1 mm) or Merck-SeQuant ZIC-pHILIC (5 μm particle size, 100 × 2.1 mm) column for measure of bioactive lipids and polar metabolites, respectively. Mass spectra were acquired on a Thermo QExactive orbitrap mass spectrometer with electrospray ionization in negative mode and positive mode, for measure of bioactive lipids and polar metabolites, respectively, as previously described^31–33^. Metabolites were identified by matching accurate mass, retention time, and MS/MS fragmentation patterns to an in-house library of commercially available standards, as described^31–33^.

### Bioinformatic analysis

Differential metabolomic analysis was conducted in R statistical software, using MetaboDiff version 0.9.3^34^ (https://github.com/andreasmock/MetaboDiff/), starting with the table of metabolite measurements. Following the recommended MetaboDiff workflow, K-nearest neighbor imputation was used to impute values for metabolites which were missing in less than 60% of samples. Metabolites that were missing in more than 60% of samples were excluded. Variance stabilizing normalization was then applied. Abundance changes were assessed on the normalized data, with t-tests computed in R, and the Benjamini–Hochberg multiple test correction was applied for each comparison. To identify the metabolites specifically associated with AP-induced hyperphagia, and unchanged by MINO treatment alone, we used the following filtering criteria: Firstly, we identified metabolites that were significantly changed in abundance by OLZ treatment compared with CON. This list was then refined to only include metabolites that were specifically blocked by co-treatment with MINO (OLZ + MINO). Finally, this list was refined to include only metabolites that were not differentially changed in abundance between CON versus MINO-treated samples. Therefore, metabolites were filtered by the following criteria: significant in OLZ vs CON (adj pval < 0.3), AND significant in OLZ + MINO vs OLZ (adj pval < 0.3) and direction of effect opposite in OLZ vs CON compared to OLZ + MINO vs OLZ, and not significant in MINO vs CON (adj pval > 0.3). Heatmaps were generated using ClustVis 2.0 software^35^ (https://biit.cs.ut.ee/clustvis). Two-tailed Pearson correlation analysis between the AP-induced metabolites and food intake, weight gain and gonadal adipose tissue (gWAT) weight were performed using GraphPad Prism with significance set at *p* < 0.05.

Prediction of pathway activity from untargeted mass spectral data was determined using the mummichog algorithm^36^ in Metaboanalyst 4.0, https://www.metaboanalyst.ca^37^. The mummichog algorithm enables pathway analysis from m/z values, bypassing the need for metabolite identification. Metabolites which were significantly changed in OLZ vs CON (adj. pval < 0.3), as well as MINO vs OLZ (adj pval < 0.3), but which were not significantly altered in MINO vs CON (adj pval > 0.3) were included in the analysis. These metabolites were further filtered by those which changed in different directions in OLZ vs CON compared to MINO vs OLZ. This resulted in 389 metabolites for input to Metaboanalyst. The Metaboanalyst MSPeaksToPathways parameters were: Mass Accuracy (ppm) = 0.01, Analytical mode = Negative, P-value Cutoff = 0.05.

## Results

**Figure 1:**
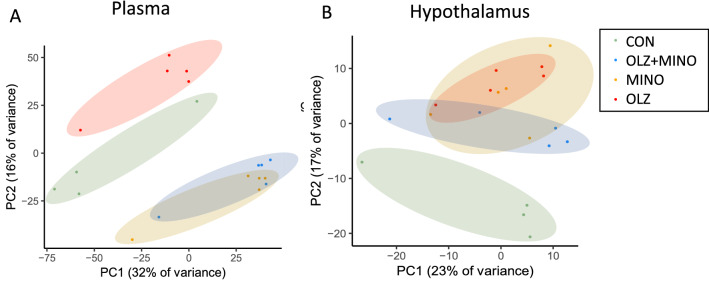
(**A**) Principal components plot of metabolomics data from plasma and (**B**) hypothalamic samples. Web tool Clustvis 2.0^35^ (https://biit.cs.ut.ee/clustvis) used to visualize data.

Principal components analysis (PCA) of the plasma metabolomics (Fig. 1A) revealed that CON and OLZ show clear separation (red vs green). In addition, MINO and OLZ + MINO (yellow and blue) can be clearly differentiated from CON (green) or OLZ (red). MINO and OLZ + MINO groups had clear similarities, as shown by overlapping space in the PCA plot (blue/yellow). PCA analysis of the hypothalamic metabolites (Fig. 1B) revealed that CON (green) and OLZ (red) groups were clearly separated. OLZ, MINO and OLZ + MINO share overlapping space suggesting that differences between these groups were less pronounced.

**Figure 2:**
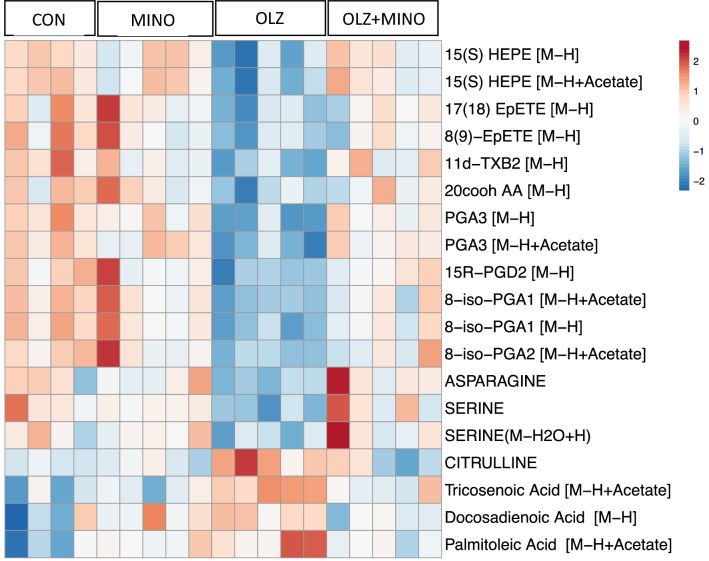
Plasma metabolites associated with antipsychotic-induced hyperphagia. These metabolites were significantly changed in abundance by olanzapine treatment compared with the control treated group and these changes in abundance were specifically blocked by co-treatment with minocycline. Specifically, using this filtering criteria: Significant in OLZ vs CON (adj pval < 0.3), and significant in OLZ + MINO vs OLZ (adj pval < 0.3) and direction of effect opposite in OLZ vs CON compared to OLZ + MINO vs OLZ, and not significant in MINO vs CON (adj pval > 0.3). Web tool Clustvis 2.0^35^ (https://biit.cs.ut.ee/clustvis) used to visualize data.

We detected 7140 metabolites in the plasma, comprising 396 identified metabolites and 6744 unknown metabolites (Supplemental Table 1). Differential analysis revealed that 1188 metabolites were significantly changed between OLZ vs CON adjusted *p* value < 0.3 (Supplemental table 2). To further identify metabolites that were specifically associated with AP-induced hyperphagia and weight gain, we applied another set of filters: metabolites that were significantly changed with OLZ treatment compared with CON and these metabolites should not be differentially changed between CON vs MINO. This revealed 389 plasma metabolites (Supplemental table 3) of which 19 (Supplemental table 4) were ‘known’ metabolites. A summary heatmap of all 19 ‘known’ plasma metabolites associated with AP-induced hyperphagia after shown in the heatmap in (Fig. 2).

**Table 1:** Significant pathways associated with hyperphagia in response to OLZ treatment in mice.

	Pathway total	Hits. total	Hits. sig	Gamma (adjusted *p* value)
Arachidonic acid metabolism	36	10	7	0.00038696
Steroid hormone biosynthesis	72	15	8	0.00038927
Retinol metabolism	16	6	3	0.0010736

**Figure 3:**
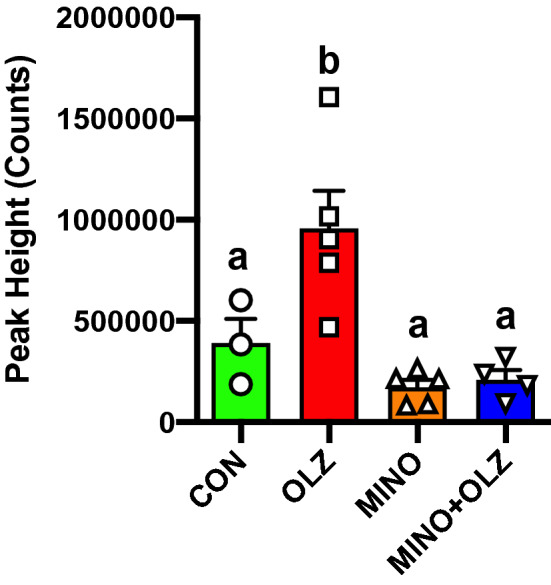
Hypothalamic levels of Indoxylsulfuric acid were increased by olanzapine treatment and blocked by co-treatment with minocycline. Significantly increased in OLZ vs CON (adj pval < 0.3), and significant in OLZ + MINO vs OLZ (adj pval < 0.3) and direction of effect opposite in OLZ vs CON compared to OLZ + MINO vs OLZ, AND not significant in MINO vs CON (adj pval > 0.3). a-b indicate statistical difference between groups.

In the hypothalamus, we detected a total of 414 metabolites of which 92 were identified and 322 unclassified metabolites (Supplemental table 5). Of these, 151 metabolites were significantly changed in OLZ compared with CON treated groups (Supplemental table 6). To identify hypothalamic metabolites that were specifically associated with AP-induced hyperphagia and weight gain, we applied the same filter that we used with plasma metabolites (metabolites that were significantly changed with OLZ treatment compared with CON and these metabolites should not be differentially changed between CON vs MINO). This revealed just one metabolite, indoxylsulfuric acid, that was increased by ~ twofold by OLZ treatment compared with CON and OLZ + MINO normalized levels to CON range (Fig. 3 and Supplemental table 7).

**Figure 4:**
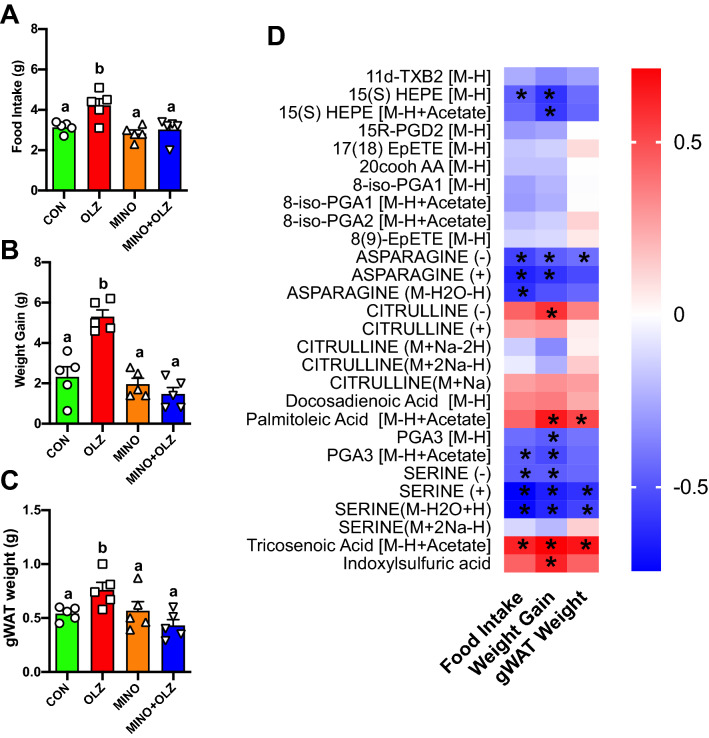
Metabolites that were significantly changed by olanzapine treatment were strongly correlated with food intake, weight gain and gWAT weights. (**A**) Daily food intake average, (**B**) weight gain, (**C**) gonadal adipose tissue weight of mice treated with CON (green bars), OLZ (red bars), MINO (orange bars), and MINO + OLZ (blue bars) for 14-days. (**D**) Heatmap showing r values derived from Pearson correlation analysis. Red indicates positive correlation while blue indicates negative correlation. * indicates significant correlation at *p* < 0.05. Data was expressed as mean ± SEM and was analyzed using one-way ANOVA at *p* < 0.05 followed by two-stage linear step-up procedure of Benjamini, Krieger and Yekutieli with a false discovery rate of 0.10 in Prism (Graphpad V8). a-b indicate statistical difference between groups.

As we previously reported, OLZ increased food intake (Fig. 4A), weight gain (Fig. 4B) and gWAT weight (Fig. 4C) compared to CON while co-treatment MINO reversed these OLZ-induced effects^28^. We then determined whether these metabolites correlate with food intake, weight gain and adipose tissues mass (Fig. 4). Plasma metabolites, citrulline, tricosenoic acid and palmitoleic acid were strongly positively correlated with weight gain, food intake and/or gWAT weight while HEPE, asparagine, PGA3 and serine were strongly negatively correlated with weight gain, food intake and/or gWAT weight (Fig. 4D). In addition, hypothalamic indoxylsufuric acid levels were significantly positively correlated with weight gain and tended to be positively correlated with food intake and gWAT weight (Fig. 4D).

### Pathway analysis

**Figure 5:**
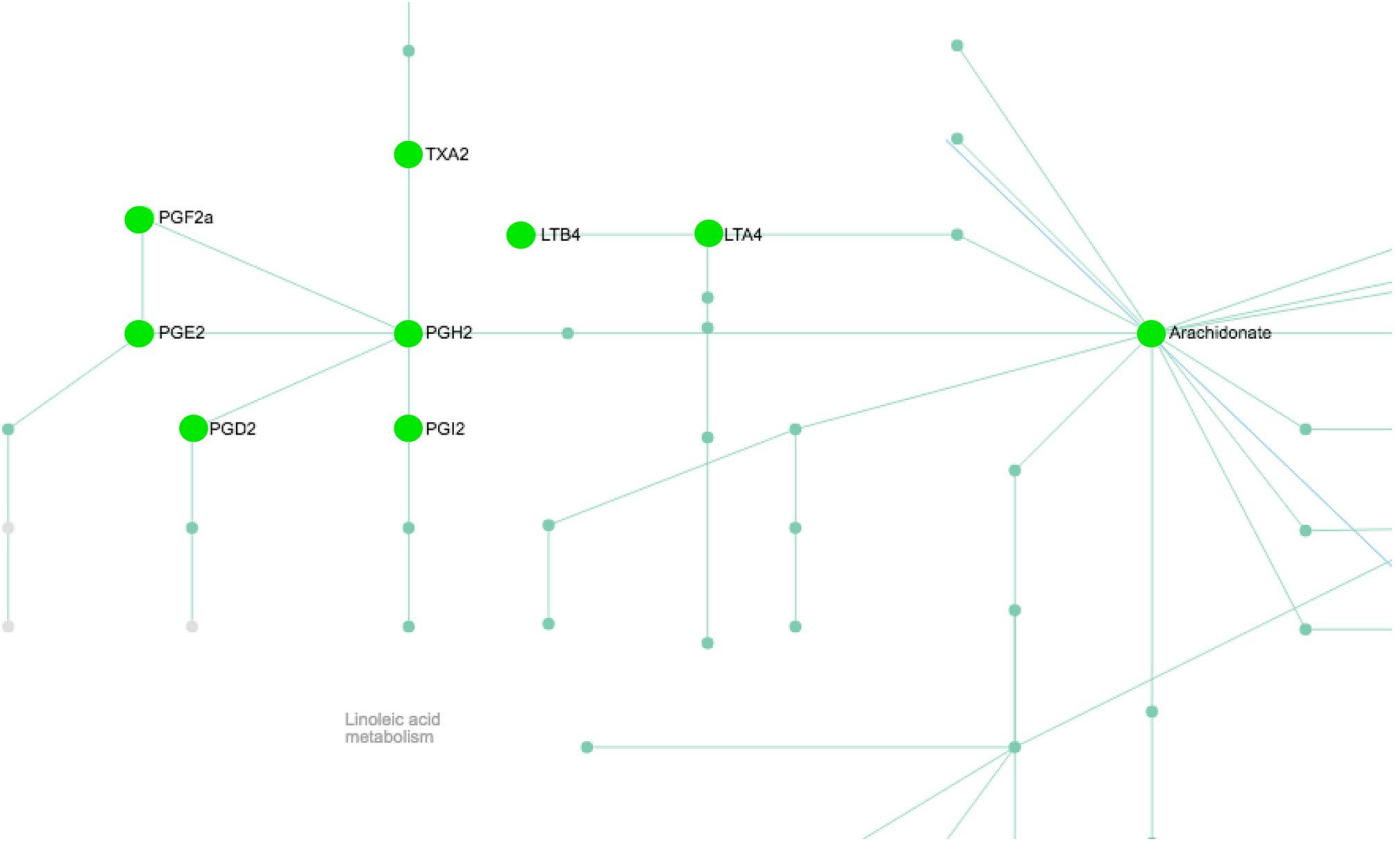
Arachidonic acid metabolism pathway. Metabolites colored green were decreased by OLZ treatment compared with CON, direction of change reversed after MINO co-treatment. Prediction of pathway activity from untargeted mass spectral data was determined using the mummichog algorithm^36^ in Metaboanalyst 4.0, https://www.metaboanalyst.ca^37^.

We then conducted pathway analysis using Metaboanalyst software to determine which pathways the plasma metabolites associated with antipsychotic hyperphagia belong to (Supplemental Fig. 1). There was a significant number of hits related to arachidonic acid metabolism, steroid hormone biosynthesis and retinol metabolism (Table 1). The arachidonic acid metabolism pathway was particularly enriched with significant changes in metabolite abundance after OLZ treatment compared with CON (Table 1, Fig. 5).

## Discussion

Studies in psychiatric patients have highlighted changes in metabolite abundance associated with APs treatment^38–44^. However, these studies are based on relatively small, diverse human populations suffering from psychiatric disease and exposed to various APs with differing lengths of treatment. Therefore, it has been a challenge to unravel the complexity of these datasets and to specifically focus on the food-intake related side effects of AP-treatment. In the present study, we used MINO co-treatment to specifically block OLZ-induced hyperphagia in mice to identify metabolites that were specifically associated with AP-induced hyperphagia and weight gain.

In the hypothalamus, we identified a single metabolite, indoxylsulfuric acid, that was increased by ~ twofold by OLZ treatment compared with CON and this increase was specifically blocked by co-treatment MINO. Indoxylsulfuric acid (also known as Indoxyl sulfate) is a metabolite of the common amino acid tryptophan^45^. Indoxylsulfuric acid has been shown to induce oxidative stress and inflammation in central nervous system cells^46^ and high levels in the brain are associated with neuroinflammation and cerebral dysfunction^47, 48^. Our studies identify a novel potential role of indoxylsulfuric acid in the hypothalamus in food intake and body weight regulation.

In the plasma, we identified 4 known metabolites that increased in concentration in response to OLZ and 19 that decreased in abundance in response to OLZ (Fig. 2). The four metabolites that were increased included the amino acid citrulline and fatty acids tricosenoic acid (C23:0), docosadienoic acid (C22:2) and palmitoleic acid (C16.1). Plasma citrulline is highly induced after AP treatment and specifically blocked by the co-treatment with MINO. Citrulline is a nonessential amino acid synthesized almost exclusively by enterocytes of the small intestine. Serum citrulline levels have previously been linked to changes in food intake in mice^49^ and human studies^50, 51^. Notably, in fasted mice, significantly higher levels of serum citrulline were observed in the morning compared to the evening^49^. In addition, increased plasma citrulline in mice is associated with diet-induced obesity and may predict the development of the metabolic syndrome^52^. Accordingly, in human studies, circulating citrulline levels have been found to be reduced by approximately 10–20% in the post-prandial period^50, 51^ and lowered by 30% after prolonged starvation^53^. These studies provide evidence that serum citrulline has previously been associated with changes in food intake in mouse and human studies, but further studies are required to address the role of serum citrulline in AP-induced food intake. Docosadienoic acid is a natural ω-6 polyunsaturated fatty acid (PUFA) and acts as an agonist of free fatty acid receptor 4 (FFAR4, also known as GPR120). Docosadienoic acid treatment in vitro in mouse gastric cells inhibits the secretion of the pro-feeding hormone ghrelin^54^. Other published studies have suggested ghrelin levels are increased in response to AP-treatment^55^. Therefore, it is possible the AP-induced increase in docosadienoic acid is a response to attempt to block the AP-induced increase in ghrelin. Interestingly, there is no significant change in docosadienoic acid levels when comparing plasma from lean and obese people suggesting this increase may be specific to AP treatment^56^. Tricosenoic acid is an odd chain fatty acid (23:0) that is highly prevalent in human plasma compared with other odd chain fatty acids. However, tricosenoic acid has no known link to food intake and is unchanged in abundance in studies comparing lean and obese human plasma^57^. Finally, palmitoleic acid (palmitoleate 16:1n-7) is one of the most abundant fatty acids in serum and tissues, particularly adipose tissue and liver^58^. A recent study grouped schizophrenia patients treated with APs by quartiles of increasing metabolic impairments (indicated by fasting insulin and BMI) and found palmitoleic acid was significantly elevated with increasing metabolic impairments (*p* =  < 0.0001, fdr 0.02%)^42^. Furthermore, higher palmitoleic acid has also been observed in plasma of the obese children^59^ and adults^60^ compared with control lean groups. However, administration of palmitoleic acid in rats resulted in a lower food intake^61^ and interestingly, this study proposed that the increased circulating concentrations observed in obesity may result in a loss of the metabolic beneficial effects of palmitoleic acid. This phenomenon of obesity-induced elevation of circulating factors and subsequent resistance to these signals is frequently observed in obesity where resistance to both leptin and insulin occurs^62^. Whether obese subjects are resistant to palmitoleic acid effects on food intake should be confirmed in future investigations.

After OLZ treatment, we observed a significant decrease in arachidonic acid (20:4 n-6) as well as the downstream metabolites in the arachidonic acid metabolism pathway including, prostaglandin (PG) D2 and 15R-PGD2, 8-iso-PGA1, 8-iso-PGA2, PGA3, thromboxane 11d-TXB2 and hydroxyeicosapentaenoic acids (15(S) HEPE), and epoxyeicosatetraenoic acid (17(18)EpETE and 8(9)-EpETE (Table 1, Fig. 2). Arachidonic acid (20:4 n-6) is a polyunsaturated fatty acid (PUFA) and serves as a precursor to eicosanoids (prostaglandins, thromboxanes, leukotrienes) which mediate inflammatory responses^63^. Plasma levels of metabolites synthesized from n-6 PUFAs have been implicated in previous metabolomic and lipidomic analyses of AP treated psychiatric patients. For example, AP treatment significantly lowered plasma levels of metabolites synthesized from n-6 PUFAs including reduced prostaglandins PGE1, PGF2a and PGG2 in bipolar individuals compared with controls^64^. The arachidonic acid signaling pathway has been implicated in food intake in a variety of studies^65–67^. Inhibition of food intake has been reported after treatment of rodents with arachidonic acid or PGF2 alpha^65^ or PGE2^65, 66^ through the EP4 receptor^66^. Therefore, it is possible that reduced prostaglandin signaling may contribute to the increase in food intake observed during AP treatment. However, other reports suggest that other members of the prostanoid family, specifically PGD2, stimulate food intake^67, 68^, and thus, further investigation is needed to specifically understand the role of the arachidonic acid pathway in AP-induced hyperphagia. OLZ treatment also resulted in significantly lower levels of amino acids serine and asparagine compared with CON mice. In previous studies, AP treatment was associated with decreased plasma serine in AP treated patients with high BMI patients compared with lower BMI groups^42^. Furthermore, administration of serine significantly reduced food intake^69, 70^ in mouse studies. Therefore, we speculate that lower levels of serine observed in our studies may play a role in potentiating food intake as seen in AP-treatment. In other studies, plasma asparagine levels do not change between lean and obese people but are significantly lower in obese patients with visceral obesity (which has a stronger association with metabolic disturbances and cardiovascular risks) compared with than obesity resulting from increased subcutaneous adipose tissue^71^. Follow-up studies are warranted to investigate the role of the amino acids serine and asparagine in AP-induced food intake and weight gain.

A potential caveat of our study is that we used our mouse model of OLZ-induced hyperphagia and weight gain. Future studies will be necessary to determine if these metabolite changes are associated with hyperphagia in general, or specific to AP-induced hyperphagia. We have related our mouse-based studies to similar human studies measuring changes in the metabolome after AP treatment in various psychiatric disease populations. However, these human studies are highly complex datasets with many confounding elements including length of treatment, medication interactions, dietary variation and complex psychiatric phenotypes that all contribute to changes in the metabolic profiles. To overcome this in the future, it would be ideal to treat a control population without underlying psychiatric disease to specifically investigate the changes in the metabolome associated with AP-induced hyperphagia and weight gain.

## Conclusions

In conclusion, our mouse-based study has identified metabolite biomarkers in the plasma that were associated with AP-induced hyperphagia and weight gain. Some of these AP-induced metabolites have been previously implicated in food intake and body weight regulation based on published literature. In addition, we have related these findings to other human studies investigating the pharmacometabolome during AP treatment. Further studies are warranted to determine whether these AP-induced metabolites play a mechanistic role in energy balance regulation or to investigate their utility as candidate biomarkers during drug evaluation for AP-induced side-effects.

## Supplementary information

Supplementary information

## Data Availability

All data generated or analyzed during this study are included in this published article and its supplementary information files.
