# Supplementary material for: Metabolomic profiles associated with a mouse model of antipsychotic-induced food intake and weight gain
Source: Sci Rep. 2020 Oct 29;10:18581. doi: 10.1038/s41598-020-75624-2 (PMC7596057; doi:10.1038/s41598-020-75624-2)
Supplement: Supplementary file 1 — Supplementary information [file 41598_2020_75624_MOESM1_ESM.pdf]

Metabolomic profiles associated with a mouse model of antipsychotic-induced food intake and weight gain

Rizaldy C. Zapata[1], Sara Brin Rosenthal[2], Kathleen Fisch[2], Khoi Dao[3], Mohit Jain[3], Olivia Osborn*[1]

[1] Division of Endocrinology and Metabolism, School of Medicine,

[2] Center for Computational Biology & Bioinformatics, School of Medicine,

[3] Departments of Medicine & Pharmacology, University of California San Diego, La Jolla, CA

92093, USA.

Supplementary data legends

Supplemental figure 1. Pathway analysis of the plasma metabolites associated with antipsychotic hyperphagia. Pathway analysis conducted using Metaboanalyst software determined a significant number of hits related to arachidonic acid metabolism, steroid hormone biosynthesis and retinol metabolism.

Supplemental Table 1. Plasma metabolites detected in control, olanzapine, minocycline, and co-treated (olanzapine and minocycline) mice. 7140 metabolites were detected in the plasma, comprising 396 identified metabolites and 6744 unknown metabolites.

Supplemental Table 2. Differential analysis of plasma metabolite abundance between groups. Differential analysis revealed that 1188 metabolites were significantly changed between OLZ vs CON adjusted p value < 0.3. Relative abundance is also calculated for these metabolites in OLZ vs MO (minocycline + Olanzapine), and MINO vs CON.

Supplemental Table 3. All plasma metabolites significantly associated with antipsychotic hyperphagia. To identify metabolites that are specifically associated with AP-induced hyperphagia and weight gain, we applied another set of filters: metabolites that were significantly changed with OLZ treatment compared with CON and these metabolites should not be differentially changed between CON vs MINO, which revealed 389 plasma metabolites significantly associated with antipsychotic hyperphagia.

Supplemental Table 4. Known plasma metabolites significantly associated with antipsychotic hyperphagia. Known metabolites that were significantly changed with OLZ treatment compared with CON and these metabolites should not be differentially changed between CON vs MINO. This revealed 19 'known' metabolites.

Supplemental Table 5. Hypothalamic metabolites detected in control, olanzapine, minocycline, and co-treated (olanzapine and minocycline) mice. In the hypothalamus, we detected a total of 414 metabolites of which 92 were identified and 322 unclassified metabolites.

Supplemental Table 6. Differential analysis of hypothalamic metabolite abundance between groups. Differential analysis revealed that 151 metabolites were significantly changed between OLZ vs CON adjusted p value < 0.3. Relative abundance changes is also calculated for these metabolites in OLZ vs MO (minocycline + Olanzapine), and MINO vs CON.

Supplemental Table 7. Hypothalamic metabolites significantly associated with antipsychotic hyperphagia. To identify hypothalamic metabolites that are specifically associated with AP-induced hyperphagia and weight gain, we applied the same filter that we used with plasma metabolites (metabolites that were significantly changed with OLZ treatment compared with CON and these metabolites should not be differentially changed between CON vs MINO). This revealed just one metabolite, indoxylsulfuric acid, that was increased by ~2 fold by OLZ treatment compared with CON and OLZ+MINO normalized levels to CON range.

Supplemental figure 1

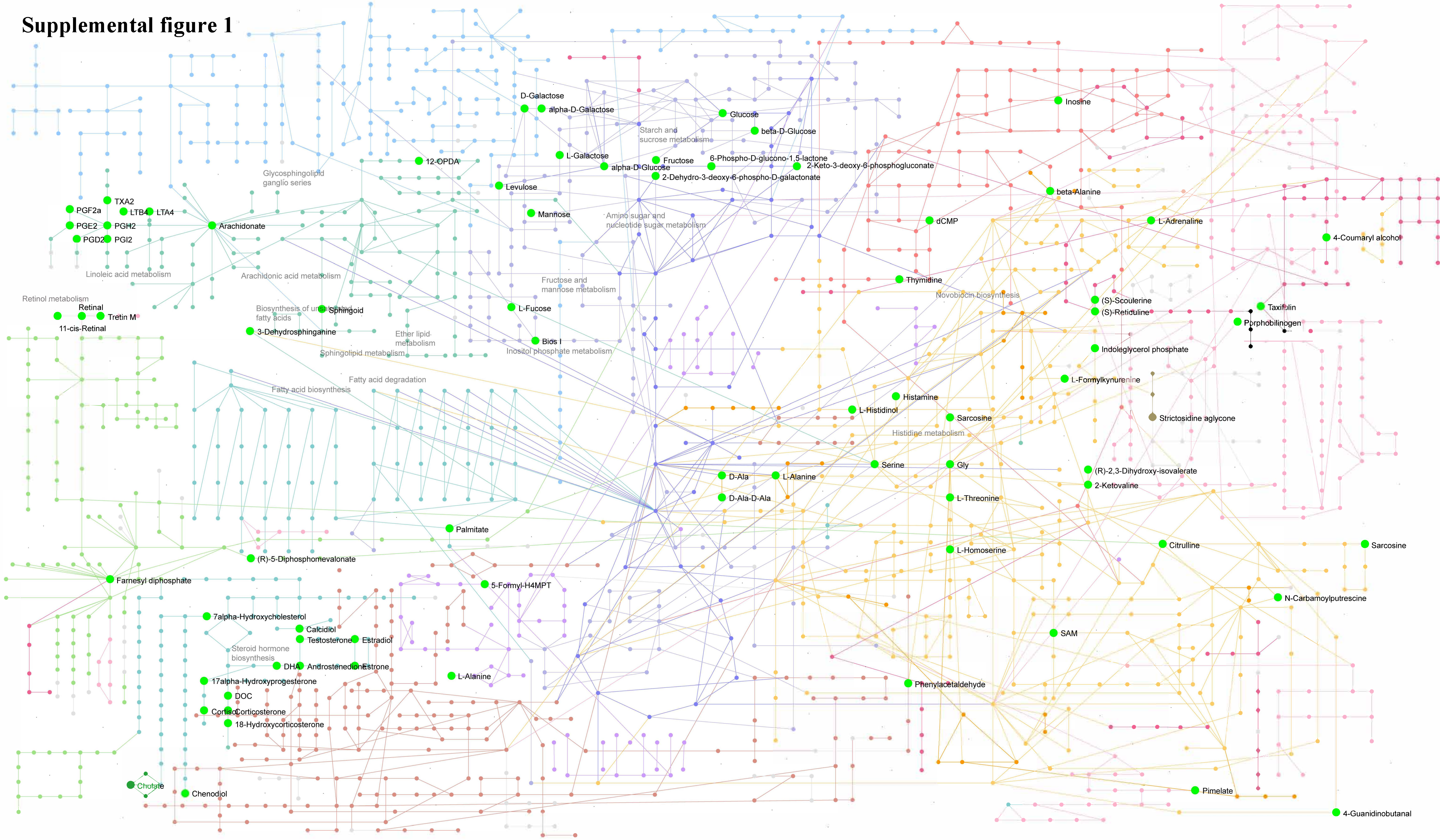

Supplemental Table 1

Method	Polarity	m/z	RT	ID	C1	C3	C5	C8	O1	O3	O4	O7	O8	M3	M4	M5	M7	M8	MO3	MO4	MO6	MO7	MO8
EIC	Negative	361.2390	3.80	10,11-DiHDPA [M-H]	110184	94077	102473	250633	79260	80414	82353	88574	100128	154956	96746	95581	123335	71017	81914	133165	88521	149821	65229
EIC	Negative	343.2279	5.28	10,11-EpDPA [M-H]	307369	304542	167076	384531	228046	277820	243992	221258	520204	227783	182563	261236	309862	114576	233941	458258	348426	201796	292606
EIC	Negative	405.2286	2.00	11-Deoxycortisol [M-H+	0	583097	0	0	159367	222279	459364	0	625694	178216	698963	211385	629796	683482	544959	761257	413262	190151	461931
EIC	Negative	337.2383	3.90	11,12-diHETrE [M-H]	394053	305195	300908	598182	269664	411109	274073	269089	252752	406023	257789	347372	355557	212602	281677	404570	299566	399014	212237
EIC	Negative	397.2579	3.81	11,12-diHETrE [M-H+Ac	246940	169357	237856	137822	122945	261919	122873	86014	247755	141080	135823	242367	262580	192182	79062	572075	425369	437263	200877
EIC	Negative	319.2281	5.45	11,12-EET [M-H]	409944	347010	220438	474101	395948	412782	421246	267554	534136	282133	313681	498086	405410	267669	413398	555876	644020	379165	498565
EIC	Negative	377.2330	4.03	11(S) HEPE [M-H+Acetat	581939	349440	709340	774783	472266	350655	349718	842371	377172	670836	339541	361298	292263	708137	413855	239672	447549	805997	388531
EIC	Negative	353.2327	2.36	11b dhk PGF2a [M-H]	352459	294089	285867	323664	281297	321238	333971	419842	308889	321889	262544	297207	286420	284884	284248	463914	318036	302551	275656
EIC	Negative	367.2122	2.17	11d-TXB2 [M-H]	280279	205823	361691	241957	105891	137845	168911	124117	111229	280946	158659	196841	158053	147621	173522	263293	156342	184688	217451
EIC	Negative	379.2473	4.92	12-HETE [M-H+Acetate]	316550	209749	440310	458570	272391	294330	325413	437362	422860	318987	266955	359057	366044	315267	226561	786899	364161	536981	226755
EIC	Negative	351.2170	3.55	12-OPDA [M-H+Acetate	326032	265838	717338	766239	578074	390240	563329	939637	506509	568493	297563	396430	321213	380111	197433	240935	380275	676547	359027
EIC	Negative	313.2387	3.45	12,13 diHOME [M-H]	2293626	1433159	4154629	10339952	2448478	2156194	3397563	4715300	3806153	3811524	2073877	3709062	3225590	2513523	756373	3453222	2973423	5695206	1554121
EIC	Negative	295.2279	5.17	12,13 EpOME [M-H]	3600487	2097970	3806851	3952462	2430621	2749700	3400897	3265680	5406491	2480827	1836364	3168216	2935760	1749715	1843098	4102327	3456429	3657575	2657606
EIC	Negative	393.2289	3.43	12oxo LTB4 [M-H+Aceta	511034	181441	387946	207588	92947	110817	124642	93555	117542	394254	91218	134938	111296	136804	102034	339775	140645	73454	147798
EIC	Negative	335.2228	5.04	12S-HpETE [M-H]	684173	376716	779392	686146	358103	328528	444355	616736	283003	764117	367760	478575	359353	352320	378257	327851	481707	913556	352238
EIC	Negative	343.2277	4.74	13 HDoHE [M-H]	644289	760434	433657	1096856	977887	647536	934441	493652	1721460	570382	509098	581068	865579	304057	752222	1189581	991742	665905	700153
EIC	Negative	295.2279	4.50	13-HODE [M-H]	1792021	1414617	4299983	5397009	3304522	2306801	2392174	5549218	4517201	2293929	1579536	1900032	1903457	2858088	662475	1821840	2155996	4238027	1721284
EIC	Negative	293.2125	4.59	13-oxoODE [M-H]	1152010	713182	1119246	1396739	1184329	935833	1369207	1086325	1660993	828983	700391	889075	933859	918433	651246	3203894	1130518	1312126	940928
EIC	Negative	361.2390	3.70	13,14-DiHDPA [M-H]	136600	128017	146066	377107	136191	112493	123524	148106	139045	208460	123660	151524	196159	85963	95238	185563	139588	214994	135496
EIC	Negative	393.2288	2.89	13,14-dihydro-15-keto	1692485	944831	1625943	1110748	595533	393628	1566346	868082	1061549	1465596	1154254	1639381	2034402	1088530	947341	1030424	1396181	2003007	1369552
EIC	Negative	353.2328	2.64	13,14-dihydro-15-keto	287184	132597	221672	199242	147278	258989	179873	149545	84361	209498	120197	136911	136800	93851	125393	135349	142443	167362	111133
EIC	Negative	297.1692	1.70	13,14-dihydro-15-keto	0	90745	0	0	41176	167912	27938	0	36148	0	88262	30102	24226	22182	35067	77628	72508	0	84317
EIC	Negative	343.2278	5.17	13,14-EpDPA [M-H]	442502	475915	246608	703643	497242	519568	515253	316876	947797	354759	354724	383966	526645	264319	439964	682563	536793	314393	456932
EIC	Negative	293.2125	3.98	13(S) HOTrE [M-H]	306257	207431	620617	2153305	442437	278021	367266	993831	646225	593477	309508	541801	505702	279223	147801	459789	438611	960926	196983
EIC	Negative	353.2312	4.03	13(S) HOTrE [M-H+Aceta	382256	377393	988953	2277523	764250	542475	638451	1612416	1275105	999081	499698	874345	760474	260008	136296	882683	594087	1385124	272657
EIC	Negative	353.2312	4.14	13(S) HOTrE(y) [M-H+Ac	1542332	1280941	2846920	3188959	2091430	1446516	1734070	3353117	2244691	2299384	1366411	1404617	1401719	2529408	991760	1168974	1923854	3193435	1728637
EIC	Negative	309.2074	4.12	13S-HpOTrE [M-H]	1447036	826542	1933968	790240	704944	1070858	684083	1495917	359340	1432233	643030	832797	528110	1328063	1077536	449221	659903	1593211	1125808
EIC	Negative	395.2439	3.32	14,15-DiHETE [M-H+Ace	2057452	987304	2602963	3154710	599781	653017	947956	1359623	887347	1521060	928264	1450383	1377365	1004703	824984	1266172	1467509	1799418	937305
EIC	Negative	381.2625	5.71	15 oxoEDE [M-H+Acetat	384013	395660	1060273	1009331	759630	492719	693906	1144626	898832	607471	325316	443574	447547	380839	232274	370276	466702	792041	364013
EIC	Negative	395.2441	2.92	15-epi-PGA1 [M-H+Acet	2328182	2725766	1650420	129116	2572673	2242925	136995	2992421	135174	171020	150917	144463	2511359	1947553	2753180	118918	2319562	2760071	2429857
EIC	Negative	379.2472	4.62	15-HETE [M-H+Acetate]	565953	345499	1000039	1134207	555696	403331	591110	1142900	657384	1063251	590804	620984	634938	625082	186682	672206	622146	1117980	511670
EIC	Negative	317.2124	4.14	15(S) HEPE [M-H]	20792812	18380351	21203246	19235856	7055493	6461397	11604355	7836969	10939718	11994421	12324922	19866509	19268476	13022391	17717709	16502979	16657888	13172187	11401453
EIC	Negative	377.2332	4.14	15(S) HEPE [M-H+Acetat	875000	755831	959645	811631	349178	300705	502441	370053	425053	508450	543675	821569	779515	531042	785155	710235	631672	562366	488630
EIC	Negative	315.1967	4.16	15d PGA2 [M-H]	2090173	862850	3082886	2274975	479086	904548	925508	859998	770614	3083649	1463601	1328996	1481537	1103210	473741	1214231	1425080	1648407	1372481
EIC	Negative	393.2288	3.23	15d PGD2 [M-H+Acetat	181896	139276	214516	206588	131807	106397	178664	141971	215728	159677	120657	152183	145289	125204	115362	158750	153253	150537	149280
EIC	Negative	315.1967	4.02	15d PGJ2 [M-H]	16241493	7544069	32205244	18498521	4789559	6035307	10586273	9297297	7402762	12333125	10004375	11555030	15603887	8744897	4215823	11155622	11903671	14782492	21650182
EIC	Negative	351.2180	2.28	15R-PGD2 [M-H]	788133	389797	748197	921068	148577	254640	264825	242077	234271	1259627	333869	411468	386479	413367	310527	418535	432120	625347	583142
EIC	Negative	343.2279	4.64	16 HDoHE [M-H]	1530218	1287601	351523	4471771	1323118	1278769	1033196	1319222	5374942	620487	727055	1611331	4121392	1760111	1641363	3118156	5390500	3685756	1987571
EIC	Negative	379.2477	4.34	16-HETE [M-H+Acetate]	434730	571357	425587	361568	429488	624809	482912	416416	734406	478962	665502	576791	533837	413139	194724	624003	699368	271766	618311
EIC	Negative	361.2390	3.63	16,17-DiHDPA [M-H]	317293	350832	352401	941011	237647	291351	292527	387799	309768	538625	389941	479992	601407	184341	258992	496554	343914	526057	195252
EIC	Negative	319.2279	4.31	17-HETE [M-H]	5056180	1138791	5160809	3479636	669300	876685	1182069	1803452	708298	4899975	1438793	1639692	1723464	1240760	1080644	1392681	1729462	2396035	1237051
EIC	Negative	379.2480	4.40	17-HETE [M-H+Acetate]	336258	301790	289187	290058	235425	306348	265886	300320	460983	333194	364945	322217	330963	283640	194724	432117	355383	252068	358312
EIC	Negative	317.2124	4.54	17(18) EpETE [M-H]	4477568	1554528	6906745	3770332	861125	684392	1593343	1637115	985018	9526239	2919901	2718311	1823770	1732906	1024704	2416139	3238436	2576688	2653506
EIC	Negative	319.2272	4.22	18(+/-)-HETE [M-H]	1518766	1142818	1226528	1550183	1202890	1483788	1518426	1237869	1407786	1289262	1052705	1617115	1364586	828257	1216945	2080683	1516542	1592543	1219304
EIC	Negative	377.2329	3.92	18(S) HEPE [M-H+Acetat	315265	226946	536695	1602586	406112	336741	386108	819468	723944	635491	331939	623298	487749	86420	205277	866389	472769	868773	261434
EIC	Negative	361.2390	3.49	19,20-DiHDPA [M-H]	2368588	1281019	2015220	6871429	1548903	1619817	1470687	2313419	1863821	3509439	1668258	2032127	2527648	906642	1050409	2090204	1605925	3136911	773809
EIC	Negative	343.2277	5.38	19(20) EpDPE [M-H]	208266	240026	140586	337629	223375	212688	253965	156137	567040	191194	148122	201163	239524	97218	217048	382001	303243	187323	231847
EIC	Negative	333.2075	3.90	20cooh AA [M-H]	3101539	1382626	3284775	3210111	1109874	852804	1335440	1684462	1193897	3971872	2412444	2130505	1458885	1494501	1167681	1767577	2824497	1879638	1939140
EIC	Negative	319.2281	5.66	5,6-EET [M-H]	908051	799398	901196	2943936	1145240	908944	1159164	1423802	1659597	1256239	771663	1178211	1261581	527798	631748	1158521	951761	1789793	622769
EIC	Negative	377.2331	4.42	5(S) HEPE [M-H+Acetate	436443	206882	710986	362772	431015	256147	442903	463615	376375	364796	304221	324508	236964	570193	189269	542904	376843	625775	319149
EIC	Negative	321.2441	6.04	5(S) HETrE [M-H]	949184	1145992	1562085	3397183	1360264	1320761	1620851	2108925	1652404	1582179	1198110	1611683	1597430	714210	816612	1216590	1266233	1984555	843447
EIC	Negative	369.2286	1.81	6,15 dk-,dh- PGF1a [M-	472310	237386	63165	0	92746	433600	59336	661651	1984719	72608	378476	1243380	1949200	2248253	365483	400492	4041092	0	291420
EIC	Negative	411.2387	2.56	6S-LXA4 [M-H+Acetate]	589931	304306	325855	444560	143754	169851	211044	171846	143268	337922	187718	325620	312097	301216	207884	273566	294034	110291	271134
EIC	Negative	335.2232	3.16	6t,12epi LTB4 [M-H]	864960	468777	810734	730419	314420	303712	392153	431190	314871	607061	318080	539434	454451	356194	287386	415157	637807	880935	299139
EIC	Negative	343.2279	4.97	7 HDoHE [M-H]	1301534	1729939	1315915	5041132	1624638	1386361	1599910	2066938	2616500	2141024	1607150	1676652	2562332	889324	1217357	2290068	1763851	2223863	966091
EIC	Negative	361.2390	4.01	7,8-DiHDPA [M-H]	253442	226129	200291	321032	251637	274632	234605	211042	237443	246565	233212	236750	260467	245745	265458	297448	255942	257635	241879
EIC	Negative	349.2023	2.25	8-iso-15-keto-PGE2 [M-	2719690	2563826	2715154	2971639	857029	807142	838277	949556	750503	2385524	1274532	1970041	1531743	1466283	1451385	1640176	1371041	1515253	1297949
EIC	Negative	351.2180	1.92	8-iso-15-keto-PGF2alph	2362833	11497799	372474	1684344	4012853	4156470	4778427	0	4618595	1648601	9205156	8790573	6791585	7049727	7607919	12343054	10912720	576960	7076516
EIC	Negative	411.2388	1.99	8-iso-15-keto-PGF2alph	417360	774573	0	0	237518	423595	417652	0	359823	0	725713	678031	435359	472058	460992	1255143	902295	0	637830
EIC	Negative	335.2232	2.62	8-iso-PGA1 [M-H]	9077873	3805691	10273598	6902294	872378	1269535	1809712	940960	1179335	13897328	4379201	3365015	3048083	3906960	2156812	3192471	4366512	2061078	5340675
EIC	Negative	395.2441	2.62	8-iso-PGA1 [M-H+Aceta	1498976	639910	1588168	1280118	139197	207023	241186	0	198451	2704109	817060	549793	465562	701030	309179	528158	753267	279359	1040767
EIC	Negative	393.2288	2.67	8-iso-PGA2 [M-H+Aceta	589875	286688	647550	797547	88793	137155	139748	108076	102295	1568685	389908	228996	213943	267103	0	197714	372596	200351	706173
EIC	Negative	397.2577	4.02	8,9-diHETrE [M-H+Aceta	205588	219171	275799	446257	277658	249823	241368	348971	315835	250168	152524	278379	258574	200464	137212	469824	232999	398850	185809
EIC	Negative	319.2282	5.55	8,9-EET [M-H]	245165	327354	198307	374258	420010	360492	417408	219975	568950	166819	220279	338675	313099	186251	342011	352387	520660	252861	366725
EIC	Negative	317.2124	4.89	8(9)-EpETE [M-H]	10571459	3875951	13330722	8425804	2062735	1714579	3482049	3581874	2288121	14660091	5027729	4524955	3015656	3366206	1984199	4842867	6486258	4672780	4370953
EIC	Negative	377.2330	4.88	8(9)-EpETE [M-H+Acetat	377403	145322	425262	288861	172187	128799	172038	229893	192412	459154	203927	192322	175153	242160	116915	208269	251030	255748	192064
EIC	Negative	321.2444	5.22	8(S) HETrE [M-H]	282502	281261	322659	0	259724	293832	0	294092	28586	300931	258899	0	296973	0	51776	88963	42799	332403	0
EIC	Negative	381.2628	5.25	8(S) HETrE [M-H+Acetat	111098	130882	289492	540777	178175	115307	147633	339669	176052	227206	115170	184316	176890	125054	42653	193970	133418	309308	150485
EIC	Negative	379.2472	5.03	9-HETE [M-H+Acetate]	269206	159110	323520	224396	302260	239290	287639	255032	307204	237546	140599	243096	235570	204332	107905	598256	265331	388508	226504
EIC	Negative	293.2125	4.74	9-oxoODE [M-H]	692460	620142	767636	1044610	911602	753835	948332	804006	1119450	739795	499077	676468	600470	559250	483909	4456715	711944	902371	622661
EIC	Negative	293.2124	3.86	9(S) HOTrE [M-H]	181158	224622	235597	285112	279684	302970	392899	214629	317038	223612	171888	198516	205464	363969	156606	355896	296005	463398	265694
EIC	Negative	277.2173	6.35	a-linolenic Acid [M-H]	160613520	20188613	222880960	424404288	256508320	237275056	254670640	258068512	339142784	14504219	13642614	208679920	18487909	134768288	10796572	14092725	18501212	203615088	153313488
fpHILIC	Positive	268.1037	1.80	ADENOSINE	335378	659476	710422	5755705	567181	526471	959692	3857181	963617	526254	1083570	790041	15584202	3680764	250256	606736	4007969	829005	449684
EIC	Negative	331.2646	6.53	Adrenic Acid [M-H]	21737668	22932082	22154146	39162186	25138727	38255109	38798766	34590759	39709672	32577005	46499531	37282375	42195682	39412218	32753911	36926141	42017662	39460879	28170355
EIC	Negative	331.2646	6.57	Adrenic Acid [M-H]	1337385	1420017	1303179	2582211	1782106	1853613	1977526	2490669	2829265	1878750	2070256	2249690	2488248	1719765	1547614	1921962	2299157	3264069	1818636
fpHILIC	Positive	162.0756	3.75	AMINOADIPATE	4353000	5278841	4033712	3170874	4901958	8112861	4967637	2549225	4314993	2957045	5676261	4493670	5373473	8558064	4380278	4644601	4163364	2701782	4465433
fpHILIC	Negative	160.0615	3.75	AMINOADIPATE	1839431	2137811	1533092	1126173	1630194	2523929	1516010	880281	1225404	1161305	1931661	1402035	1896988	2330058	1563121	1671436	1451515	1145736	1533796
fpHILIC	Positive	144.0656	3.75	AMINOADIPATE(M-H2O	387167	479515	367966	345878	476119	814617	498425	266775	393910	329056	527800	463151	502665	791269	400840	543762	403963	262441	454424

fpHILIC	Positive	241.1292	3.19	ANSERINE	6317116	3952579	3364399	5090608	3165917	5322522	3428434	2837730	3752768	3623189	3991477	4152607	4115541	3929855	3834844	5100988	3256326	4812912	3331111
fpHILIC	Negative	187.0378	2.71	ARABITOL(M+Cl);RIBITO	2917426	1949397	2218371	3444990	2283702	2975423	2433880	2911692	2631314	2484500	1994545	2211263	2382474	1865425	1440727	2556448	2273650	2718796	1995515
EIC	Negative	311.2957	6.72	ARACHIDATE [M-H]	6494117	10616147	8403271	12908594	14284889	9371361	13138705	10290503	8954705	12805066	12373207	16705353	10670212	13623811	13198990	7567970	12934813	15138816	11255206
EIC	Negative	303.2327	6.44	Arachidonic Acid [M-H]	279097984	286350250	74770048	308100898	272181760	240106265	305291285	296875285	273160024	199873177	266454207	273219723	291855651	234738924	241612353	281442482	281256126	232527673	270581709
EIC	Negative	303.2327	6.58	Arachidonic Acid [M-H]	2100065	3063116	1586917	3011277	3780460	3251577	4057548	2929640	5226068	2430767	3943136	4025696	4338848	3511353	3654571	4131778	4952532	3968987	3698437
fpHILIC	Negative	131.0463	3.50	ASPARAGINE	12893349	6797381	8569786	6763466	4573566	4841557	3869839	5573223	3800565	8282816	6820602	7131154	7161180	6778168	12941481	8296191	5358403	9419360	3545159
fpHILIC	Positive	133.0605	3.50	ASPARAGINE	12846199	10654547	11954843	7296091	5490428	6332984	5959176	6741955	6347364	9251476	7288083	7907089	8987410	10829127	14980608	10079335	7439610	10835011	8722231
fpHILIC	Negative	113.0357	3.50	ASPARAGINE (M-H2O-H	1722506	889920	1280494	1053317	662456	760243	625874	876086	637181	1259235	1040394	1110687	1117921	1129987	2045107	1374557	859417	1511322	551525
EIC	Negative	407.2805	2.75	b-Muricholic Acid [M-H	169281174	19474887	190273416	100097636	30051720	39170150	20002242	151853885	37012646	5853435	299348	110109	6630086	447054	113141	90532	65572	3922362	542283
EIC	Negative	467.3020	2.75	b-Muricholic Acid [M-H	77307030	9343046	86292364	44953576	14348556	17484424	9095656	71806776	17342814	2809515	341060	225885	3032147	399553	268196	205585	219834	1954376	534134
EIC	Negative	339.3269	6.86	Behenic Acid [M-H]	128890	163904	136114	192930	158334	171559	208909	163856	157665	245209	221247	350244	231549	253726	216656	167803	246622	333471	224047
fpHILIC	Negative	116.0717	2.28	BETAINE	7882849	3905292	6183611	5480663	4164353	5467874	4392542	5442712	2831511	5429602	3292554	3717558	3354480	2254425	3374708	3574619	3432685	5689481	2423437
fpHILIC	Positive	118.0863	2.28	BETAINE	1197446912	856638080	1077841920	970940992	922274496	1021024640	939321792	1027562496	732770752	1008540224	804361792	844726592	809894336	612896192	746776704	776748416	813261440	977769024	663242944
fpHILIC	Positive	140.0681	2.28	BETAINE(M+Na)	1657798	3112809	3317566	3387178	3094878	3786097	2694902	3458880	1754455	2842474	1921976	2291579	2359401	2214164	2668413	2171347	1660126	3711379	1360877
fpHILIC	Negative	225.0993	3.63	CARNOSINE	2608349	1271185	1697302	2046413	1117139	1413814	1078333	888614	707238	1924403	1585195	1648291	1545421	986367	1044095	1425717	1118335	1002640	1076657
fpHILIC	Positive	227.1134	3.63	CARNOSINE	8446045	3889466	4491539	5913339	2775701	5532052	3759743	2389121	2682727	5258414	4606140	5325266	5081354	3801713	3348933	5622421	3865312	3366945	5501348
fpHILIC	Positive	249.0955	3.63	CARNOSINE(M+Na)	978852	518241	709660	903040	476875	556635	489436	467177	385253	888570	716013	714594	707317	472049	485601	668761	492721	514050	577095
EIC	Negative	391.2859	3.68	Chenodeoxycholic acid	23690433	1137086	36650252	5955779	3258109	1946589	3384821	10523079	1266596	418387	16264	44732	174821	0	0	20084	0	298726	79676
EIC	Negative	451.3070	3.68	Chenodeoxycholic acid	67123118	3270221	106807504	18238944	9991295	5765890	9986023	31003785	3836998	1125647	79134	84425	470817	72409	68122	84759	38790	873505	151506
fpHILIC	Negative	191.0197	4.72	CITRATE	139428784	93197952	96307032	115063464	106547232	143562208	100517312	98689088	119337632	91292784	96558808	98500504	99044648	99422136	88094200	120585760	92102672	103995624	95811264
fpHILIC	Negative	213.0017	4.72	CITRATE (M+Na-2H)	546853	578444	538485	688860	621380	571942	586110	641885	667429	652330	698799	609261	681761	689180	710985	618667	630240	632462	723587
fpHILIC	Positive	236.9983	4.71	CITRATE(M+2Na-H)	878112	1007371	992789	1148327	1131496	1138316	1183990	1254517	1297759	1250876	1265376	1268862	1268858	1399844	1288979	1304413	1365508	1385876	1224407
fpHILIC	Positive	215.0161	4.71	CITRATE(M+Na)	1578021	1086213	1328043	1853349	1933989	2771902	1627818	1419514	1854878	1330146	1624279	1424551	1270457	1420212	972667	2286170	1189397	1558260	1243830
fpHILIC	Positive	176.1026	3.71	CITRULLINE	63703408	56758932	64275536	65868912	72690504	88680928	79811728	63848400	69098224	62754060	56473324	64741752	54878736	44534568	64566720	68590416	49969252	53232748	48261684
fpHILIC	Negative	174.0883	3.71	CITRULLINE	16926856	13622335	13972847	12944427	15362122	17464784	14450564	12923574	11404907	12541884	12894065	12084766	11697424	9591678	14052641	13999857	11236659	12673760	11127372
fpHILIC	Negative	196.0704	3.72	CITRULLINE (M+Na-2H)	628637	706523	666037	723332	697213	653175	634752	657496	619841	683852	692932	675534	730361	614733	701049	703968	641337	641659	553117
fpHILIC	Positive	220.0663	3.72	CITRULLINE(M+2Na-H)	568566	651310	688258	704453	772695	634475	644545	756895	565885	834261	761908	764454	746488	661163	728735	671161	721430	764077	629111
fpHILIC	Positive	198.0856	3.72	CITRULLINE(M+Na)	12874030	13213772	13942743	13801182	14904485	14640567	14517182	13902787	14317192	14106111	13561146	14341663	13759940	12095453	15190483	14277071	12488013	13441811	12132001
EIC	Negative	345.2075	1.87	CORTEXOLONE [M-H]	40254	175719	0	0	58284	100438	137084	0	205386	0	201247	73045	225907	220428	188373	291716	126189	0	204643
EIC	Negative	405.2285	1.87	CORTEXOLONE [M-H+Ac	13827269	47507340	2250432	3559977	11235558	20517681	35246986	2212124	50981536	1737051	55617763	17074820	54872334	58984682	46116788	60480451	34060787	4302583	42522583
EIC	Negative	361.2018	1.45	CORTISOL [M-H]	0	31250	0	0	111611	44387	79700	0	69268	0	40429	0	21692	162454	80463	80398	26488	0	132913
fpHILIC	Negative	130.0622	3.36	CREATINE	8920123	8032879	4421572	3826605	6681534	8688202	6535983	6001655	8051018	3493709	4511170	6260162	7443535	6505364	7459436	9838511	6438643	6410756	3704079
fpHILIC	Positive	132.0768	3.37	CREATINE	905958272	807127872	548336768	505986368	632487040	837678720	603247872	708904704	737437056	477395456	525010016	641353024	736034240	771633856	785265152	868057472	619840640	769910080	481608416
fpHILIC	Positive	154.0596	3.37	CREATINE(M+Na)	1074096	1520606	993972	929638	1401770	1730106	1041304	1362034	1586639	803707	1154215	1287587	1364018	1712766	1405494	1542181	1380042	1370213	1027222
fpHILIC	Negative	242.0786	2.61	CYTIDINE	802499	422705	325070	794899	402394	662700	461389	501664	804632	466125	307714	617814	478013	369214	444571	810671	460941	525590	524559
fpHILIC	Positive	244.0919	2.62	CYTIDINE	12768348	8711554	5861432	12636649	7915307	13371811	7816389	8088312	12292029	8409480	6687174	11965852	9347790	6753963	8054861	12060288	7189578	9517051	7112551
fpHILIC	Negative	278.0548	2.61	CYTIDINE(M+Cl)	2642578	1716414	1206037	2478733	1692305	2560751	1860725	2035956	2432910	2219711	1400997	2505942	2019822	1597483	1874675	2010218	1830753	2071226	1702599
fpHILIC	Positive	266.0732	2.61	CYTIDINE(M+Na)	1774218	1307568	953544	2085053	1397684	2072617	1304759	1434804	2088950	1442631	1102125	1926592	1562198	1094208	1259585	1992065	1293706	1513412	1366336
fpHILIC	Positive	112.0507	2.22	CYTOSINE	10664462	8099069	10767831	11903500	9125440	11568850	8319314	9596635	12043338	10684265	10344922	10357358	9872386	8229079	6563972	8982356	7642570	8397223	8369661
fpHILIC	Positive	159.0274	2.05	D-GLUCURONOLACTON	2633330	1184890	3218257	527901	7017837	5985480	2556776	617601	4641355	836051	38670	48402	4530093	4977515	0	54319	5994378	66166	0
fpHILIC	Positive	162.0756	3.18	D-MANNOSAMINE(M+H	914632	735359	972185	914546	806629	1043300	974304	797687	771152	788298	743711	704526	749777	615699	731183	688680	695389	891618	668631
fpHILIC	Negative	229.0117	4.03	D-RIBOSE 5-PHOSPHATE	1135162	373902	219271	300856	663912	824540	527757	298857	1069034	204798	229450	540808	460068	702979	467262	570991	1191701	510996	715395
EIC	Negative	367.2120	1.32	d17 6k PGF1a [M-H]	469748	101249	0	0	24449	36054	51034	0	28425	179986	108102	75121	64373	104465	46655	89819	109064	0	110851
EIC	Negative	391.2859	3.85	Deoxycholic Acid [M-H]	180539560	32030821	166167883	125196938	52062801	40074616	54557476	122977693	15999598	58831555	5953162	3491762	5037061	160435	1029400	1790295	839702	13387791	9083240
EIC	Negative	451.3069	3.85	Deoxycholic Acid [M-H	43479769	8118852	42206422	31015428	12812877	10288544	14228485	29109905	3886423	14561470	1446379	857047	1202152	42920	270302	428044	210408	3268937	2216234
fpHILIC	Positive	228.0975	2.22	DEOXYCYTIDINE	4994171	3470974	4779005	5706101	4239762	4990879	3988089	4557044	5430646	4709176	4657575	4798408	4577710	3972859	3141544	3919635	3198617	3698075	3547043
fpHILIC	Negative	226.0834	2.24	DEOXYCYTIDINE	1011605	672144	974067	1092339	700406	904001	613211	831262	891413	938394	713289	772602	781461	551456	527649	676594	677281	758303	591848
fpHILIC	Positive	250.0793	2.24	DEOXYCYTIDINE(M+Na)	557186	1227073	1306137	1261305	1131851	1149467	1174602	1128242	1376187	1043261	1133154	1334641	1211794	1270466	1142485	1058378	935513	1093381	1048239
EIC	Negative	227.0648	0.55	DEOXYURIDINE [M-H]	110933	134578	123336	115002	76376	73490	88758	105069	108885	94032	113487	104437	110927	53363	102813	110082	110442	128721	74199
EIC	Negative	305.2487	6.57	Dihomo-g-linolenic [M	1378194	2051087	1536906	2179837	2052642	2078455	2453165	2262057	3085022	1983707	2739245	2501767	2892138	1925583	2096819	1910253	2777385	2862349	2289608
EIC	Negative	335.2958	6.64	Docosadienoic Acid [M	771940	957320	995903	2033394	1644603	1726952	1348241	1613245	1511924	1328328	1134212	2033249	1230615	1328582	791149	1302023	1287486	1252652	1030314
EIC	Negative	327.2330	6.41	Docosahexaenoic Acid	278867044	455554070	381745169	620596859	461391635	413257613	415287951	460199024	523511230	364661504	357356704	393838144	461969810	260919831	312788257	406689835	440744936	378807710	307991998
EIC	Negative	329.2489	6.49	Docosapentaenoic Acid	56914520	35017944	79558608	109935760	55491240	67752376	50474204	82923696	24842540	57664772	49700820	86927640	41133828	29600356	44843252	40633992	51460056	40673848	46475108
EIC	Negative	333.2803	6.59	Docosatrienoic Acid [M	1595969	1745552	1903148	2516336	1971848	2643280	2908025	2697731	2395222	2593080	2355986	2368967	2479216	2194692	1736342	1716669	2756080	2893896	2330104
EIC	Negative	267.1967	3.56	EIC_1	1932522	1047775	3745698	3310006	1562893	1581620	1240120	3088235	1218414	2859198	1223213	1399318	1053043	2543341	512420	509519	1316459	3129895	1462316
EIC	Negative	267.1966	3.44	EIC_1;EIC_2	1772828	1344146	4550873	4308110	2990315	1987971	2318621	4550110	2453476	2804643	1317527	1914732	1935908	2325296	576737	1022291	2033406	3380849	1498176
EIC	Negative	293.2125	5.02	EIC_11;EIC_12	2151142	2214167	2328131	6994520	3039109	2960183	3644950	3365477	3995446	3108760	2288234	3613836	3341643	1478199	1920775	2929748	2675747	4169981	1648073
EIC	Negative	321.2444	5.38	EIC_111	333422	348267	485534	839780	372436	450356	394611	588222	537695	444145	330078	414693	438020	235088	302611	299519	550591	651465	329599
EIC	Negative	327.2181	3.24	EIC_121	509675	207450	582171	441725	314108	309534	330765	537747	142578	419483	206089	209654	188614	135733	156595	210287	194555	420821	193481
EIC	Negative	329.2336	2.30	EIC_125;EIC_126;EIC_1	2143153	375635	1254234	523032	421634	692963	515663	527093	457306	689571	275589	391784	461203	602021	339735	1779629	452957	676908	548125
EIC	Negative	329.2335	2.55	EIC_128	844321	160210	419895	266297	159550	279779	246466	232152	244170	400246	191287	233426	246229	297086	157122	437442	251450	348008	250329
EIC	Negative	331.1918	2.57	EIC_131	3124982	3345311	3107362	1910997	1270294	1279217	1861504	626930	1186292	4617913	3611528	4035721	2769716	2587732	1631638	2719539	3178582	2430087	757130
EIC	Negative	331.1918	2.67	EIC_131	25327644	20290655	25222001	22525581	9431742	11655254	20327052	12922500	16088187	18306008	15846062	26938440	25504130	21212058	16335276	19859296	20908047	21577331	15752476
EIC	Negative	331.1918	3.24	EIC_134	3708245	2198024	5298520	4006295	912715	923698	1404964	988451	848896	2971435	1452911	1934048	1544516	1060403	1255792	1966384	1482522	2466425	1462796
EIC	Negative	333.2075	2.32	EIC_138	57263328	23798868	41817758	58145034	6257322	7625557	11706353	10232286	7544203	99330153	29955980	21812904	17450912	28769870	13831205	21622470	27870457	30355222	26049854
EIC	Negative	333.2075	2.44	EIC_138;EIC_139;EIC_1	66654874	15862703	72271537	43200284	3841043	4943190	8067218	6316310	6298962	116974094	24532434	15769427	11677105	23681940	7212886	20264235	24102027	22448064	20969295
EIC	Negative	333.2074	2.36	EIC_138;EIC_140	17026608	5736584	20977041	17183842	1933169	2182906	2979450	4789629	1992924	31409608	5731537	4735332	3881261	5319242	2975392	6384874	6203553	7268170	5410387
EIC	Negative	293.2125	4.32	EIC_14	277930	155489	329456	379056	213345	210642	239873	303208	229255	268375	125636	180358	170283	146670	149132	172378	175590	315119	146940
EIC	Negative	293.2125	4.38	EIC_14;EIC_16	303039	323876	544461	784873	465698	317507	707836	666006	692634	353169	262734	277199	454057	261377	153614	439051	520242	671306	215660
EIC	Negative	333.2074	2.80	EIC_141;EIC_146;EIC_1	1539079	1048820	2244698	988983	640856	717646	1352063	751184	749390	2728924	1233428	1179148	1343193	1029346	990030	1133072	1499258	1676243	1391501
EIC	Negative	333.2074	3.53	EIC_143	1726195	949779	2822542	1318036	478872	496582	915272	741014	484053	914200	634882	830048	1028781	559449	690041	845414	803524	1021415	1015575
EIC	Negative	333.2074	3.04	EIC_144;EIC_148;EIC_1	4601574	2358299	5681654	3872929	930337	1014218	1270594	1572547	769431	3393112	1835500	2838733	1933012	2291566	1633697	2411183	2302664	2229140	2189985
EIC	Negative	333.2074	2.90	EIC_146;EIC_149;EIC_1	12409216	6695662	11738369	8081221	4208052	2884371	11065781	6554916	7853572	10905785	8710048	11978158	14353024	7920336	7116420	7348229	9610439	14266127	9660572
EIC	Negative	333.2074	3.15	EIC_148	2398697	1231363	3007968	2831974	522924	581595	915477	1022783	595999	2061633	1002715	1132096	1294911	901449	900556	1207641	1313491	1364779	1054214
EIC	Negative	335.2232	3.02	EIC_156;EIC_159;EIC_1	3277912	1142088	3579324	2284255	640640	986978	1075301	1123540	1045668	2854134	1096740	1332901	1541148	972777	927134	1393143	1154205	1711947	1726603
EIC	Negative	293.2125	4.45	EIC_16;EIC_18	866020	584112	933110	1168888	1008396	803429	1258887	961825	1492233	693004	519044	687173	682271	720906	514181	2901189	890730	1083997	730763
EIC	Negative	293.2125	4.53	EIC_16;EIC_18	361263	209170	338014	319896	287662	286438	375917	312184	436705	264862	208686	251491	260496	263960	216686	2209384	315360	319513	267327
EIC	Negative	335.2231	3.88	EIC_166	272179	284275	206332	751229	235267	288531	228045	425250	1291363	211131	192916	436707	860535	696857	233351	571077	1568686	998132	545308
EIC	Negative	335.2231	3.62	EIC_170;EIC_172;EIC_1	2049363	395096	2046279	1106708	325253	324129	444785	426998	438269	1486982	356800	504715	495713	422435	328669	720484	602129	732641	414723

EIC	Negative	335.2231	3.32	EIC_171	2673538	1169098	3137382	2480717	530521	606435	957268	1011401	474746	1877509	994787	1368732	1263349	1223011	1050985	1240207	1621477	1614961	1174766
EIC	Negative	337.2383	3.73	EIC_181	351590	237810	347843	686234	328855	316747	292905	386339	287076	464120	256601	404361	351858	163986	206038	399163	287459	496863	176921
EIC	Negative	337.2382	3.54	EIC_182	634861	408395	677888	1751911	524664	530552	569619	736105	692998	827198	519749	939062	762831	250328	330776	629317	632937	1006354	285654
EIC	Negative	341.2334	3.69	EIC_184	976933	402623	989957	642891	546527	608659	816788	1068580	168493	757431	372692	469362	319598	183500	259528	263785	219583	788424	342401
EIC	Negative	343.2277	4.42	EIC_193;EIC_195	542395	602545	319821	710064	562665	475637	616113	335029	1126065	469053	462388	565900	671010	215074	620983	978298	742474	459560	611652
EIC	Negative	343.2277	4.20	EIC_198;EIC_202	1800899	1707986	1626050	4858218	1707811	1669193	1454534	2022361	2631937	2670654	1649230	1833258	2496957	971435	1301969	2420291	2031041	2572346	1300091
EIC	Negative	295.2279	4.37	EIC_20;EIC_21	7600328	5319601	11868138	9516957	8132087	7033492	7916484	10062917	7850821	6645544	5086729	5449024	5462019	10927753	3305389	9499134	7599666	10238852	6700080
EIC	Negative	295.2279	4.43	EIC_20;EIC_21	5868100	3741927	10522118	5635086	5031159	4414555	6123265	6380718	5475786	4830318	3883324	3941239	3338253	8185164	2355467	7277939	5308954	7836805	4331562
EIC	Negative	349.2023	1.73	EIC_219	4661361	1121679	2623269	2818422	203349	345970	408317	330478	365585	5717463	1145128	885217	614021	992223	569677	972411	854037	350156	835096
EIC	Negative	295.2279	4.60	EIC_22	1814263	1242153	2792388	2098844	1476053	1505184	2403587	1803080	1783972	1459831	1056914	1237674	1127736	1850811	842572	3085360	2050788	2107796	1259174
EIC	Negative	351.2180	2.69	EIC_232;EIC_237;EIC_2	1269165	592287	1742809	707783	323741	357170	542365	447053	342040	826993	541355	704648	719023	567057	647001	539880	521454	903129	692049
EIC	Negative	351.2180	1.99	EIC_233;EIC_249	1240954	2002709	883624	450273	577744	823106	956532	0	826293	960124	1907250	1767383	1199103	1336586	1247557	2136947	2166815	0	1667854
EIC	Negative	295.2279	4.87	EIC_24	1758142	1223342	2681576	2048385	1850019	1634064	1810269	2745009	2479887	1434098	1183958	1268345	1477835	2777201	775516	1427006	1695083	2398377	1532683
EIC	Negative	295.2279	4.23	EIC_25	1149653	773847	1771176	2506718	1065347	941476	1106316	1623156	1364437	1236935	759763	1075993	949548	1072596	575745	1821045	1032673	1841128	756012
EIC	Negative	353.2343	3.25	EIC_251;EIC_256;EIC_2	82265	50695	122171	55776	75020	118529	55370	66294	101434	54678	40748	42777	46989	39443	35463	3033583	55351	95609	50039
EIC	Negative	353.2322	3.31	EIC_251;EIC_265	184205	159102	180669	190372	186517	178831	176301	159165	157424	166780	164965	155937	164252	153508	150369	3083900	182311	163674	154416
EIC	Negative	357.2055	4.76	EIC_277	24162	0	0	358595	0	0	0	181549	0	0	77689	208603	0	328747	110199	35556	0	622842	0
EIC	Negative	295.2279	3.92	EIC_29	4355107	2833948	7837475	32728570	5934848	4237401	5177897	13505552	10547096	9029549	4894902	10786102	8421024	2089140	2453830	12700316	6715084	14604257	3229498
EIC	Negative	297.2436	4.72	EIC_31	6765881	5291685	14916543	18029994	11225066	8268788	9321963	16933909	13253614	9886977	5680328	8179057	8162908	8436309	3488445	10952324	8526313	13389325	5843028
EIC	Negative	363.2530	3.78	EIC_312	188964	72611	152430	59842	133174	54967	33222	11516	46190	285773	41548	174571	0	38304	46964	188949	40551	71855	69873
EIC	Negative	363.2553	3.79	EIC_312	208443	138793	196069	564609	185477	163088	144547	227530	214969	310168	152295	182090	212987	112316	92886	206691	186210	296295	85317
EIC	Negative	365.1975	2.01	EIC_315	0	82273	0	0	40656	90526	86331	0	50553	0	81057	108820	71661	52858	101426	155415	63672	0	105486
EIC	Negative	299.1878	2.57	EIC_32	883040	77085	604590	1048908	73587	98743	106217	663709	53721	724641	85247	88049	80991	56353	100727	78715	73440	737070	71629
EIC	Negative	299.1880	2.49	EIC_32	79673	499498	43582	89403	547183	651773	591208	60944	517940	73529	666815	648291	808057	330754	611587	714156	646607	78154	407780
EIC	Negative	375.2173	2.91	EIC_328;EIC_332;EIC_3	7457627	4877275	9229228	6705362	2905285	2570058	4312582	2578429	3050684	7207802	4969892	5839924	5067730	5126322	4100040	5829202	5441222	4707553	3238359
EIC	Negative	299.2591	5.44	EIC_33	1842977	1539795	1350476	1451152	2487107	2496631	1916251	1877615	1710715	1233811	1185905	1069009	1407122	1462059	1577666	2192676	1360027	1826091	1797439
EIC	Negative	299.2591	5.50	EIC_33	1497490	1181801	3633108	3046581	2311495	1759553	2158629	4027523	2801719	2048264	1288801	1297498	1667995	1678430	822133	1423725	1587704	2543997	1396947
EIC	Negative	299.2592	5.37	EIC_33	5103601	3825379	10326756	13335019	5078186	3473148	5002087	10435433	6876145	5357375	3041570	3386209	3931515	6054609	2994770	2893625	4110643	9156559	4742954
EIC	Negative	299.2591	5.36	EIC_33	1986408	1754053	10927021	14024105	2384818	1989892	5002087	10740308	7390457	5357375	3041570	1579739	3931515	2465127	2994770	2893625	1994961	9534463	4742954
EIC	Negative	375.2172	3.76	EIC_340	451381	292059	621130	337972	203589	212671	332867	296710	227275	275984	196848	310042	297781	303902	210327	321854	298258	423708	186000
EIC	Negative	377.2333	2.64	EIC_346	0	77908	0	0	102364	89578	137957	0	99497	0	60080	68314	61960	47794	70078	89804	72938	76446	75423
EIC	Negative	391.2135	2.15	EIC_352	1353176	376558	1429942	594509	318174	722010	105904	131491	311314	406329	590108	379105	599793	505039	608404	310377	723011	121660	324769
EIC	Negative	391.2136	2.48	EIC_356	331987	891958	0	1141773	875013	910392	1081200	0	915759	623557	891712	915469	983075	998739	964810	889005	1032952	377117	1073529
EIC	Negative	289.1810	3.77	EIC_4	284245	194640	393113	238032	216737	193256	296053	306614	64155	365190	289301	280445	184479	168938	195875	79683	149056	263116	210349
EIC	Negative	307.1936	2.26	EIC_41;EIC_42	0	210838	449762	0	109843	0	155992	205643	97801	309343	0	200301	177395	31486	19914	167443	38214	236113	30758
EIC	Negative	309.2074	3.68	EIC_51;EIC_58	922156	938972	922363	1593730	1292200	834852	1491311	1394720	1706642	968164	680727	996371	878243	797690	726278	1008581	1074276	1178738	1000156
EIC	Negative	309.2073	3.61	EIC_51;EIC_59	964863	876440	1056768	1578506	1209746	868226	1385745	1391639	1543824	856580	630802	979297	794792	728626	711329	875442	1052981	1009288	906185
EIC	Negative	309.2072	3.37	EIC_55	303533	245577	295801	481708	351291	282196	366121	442105	523621	239205	207988	334926	245905	222028	212668	333580	288451	324968	259191
EIC	Negative	309.2074	3.80	EIC_58	12694305	3365429	16848273	11980314	3717569	3801183	3728948	10912164	3076735	19543624	3408964	4346705	3433061	8241886	6748073	2706653	4362170	14394207	5825451
EIC	Negative	291.1967	3.99	EIC_6	102125	68735	101414	97142	94893	68820	103292	96269	156016	82525	68071	71347	78055	72935	56800	102475	95332	120955	82195
EIC	Negative	311.2230	3.23	EIC_61;EIC_64;EIC_66	1133502	996137	1372220	1572841	1426964	962420	1830818	1367103	2251638	1038948	835820	1169337	1032442	917867	842874	1355161	1210615	1265325	1210227
EIC	Negative	311.2229	3.30	EIC_61;EIC_64;EIC_67	1972991	1540887	2116138	2288030	2286455	1828469	2704432	1941396	3504684	1498612	1273156	1905604	1736700	1621262	1229432	6502351	2122896	2393402	2085420
EIC	Negative	311.2229	2.92	EIC_62	1434912	512646	1621077	2267288	669030	680872	1509019	1176049	818334	1606756	464462	656091	889260	670382	277282	743279	907958	1564755	474824
EIC	Negative	311.2229	3.08	EIC_62;EIC_66	734475	508125	739498	752113	691001	607976	923544	718108	979489	561308	428711	646795	525544	454392	390387	1338620	617975	654694	561849
EIC	Negative	311.2229	4.35	EIC_63;EIC_68	14278944	4139812	17807795	18188107	6024277	4593753	6260993	15952954	5280500	23451307	4671745	5877099	5814179	8153851	7981772	4160074	6495203	24070419	6091534
EIC	Negative	313.2388	3.32	EIC_71;EIC_73;EIC_74	3766900	2024915	7914021	31227329	4761730	3620259	5564348	11363710	8363841	8597089	3986055	8600092	7539058	2068100	899658	7399676	5778599	12024925	1746547
EIC	Negative	313.2385	3.14	EIC_76	878304	318929	846428	724049	349046	592679	463988	562645	552003	559371	336979	497316	494960	490254	212438	1081169	702701	973683	436976
EIC	Negative	313.2388	5.04	EIC_77	7590056	3669148	10324633	10401596	4741711	3917630	5366310	8316609	3487243	9369914	4020801	5220777	4303042	4703322	4151169	3631986	5414801	12947573	3853966
EIC	Negative	315.1967	3.09	EIC_79	5332861	2792241	7864288	5200098	1387095	1252003	2376423	1539917	1366708	4686934	2697245	3449609	2886524	2693724	2175626	3184796	3155735	2780313	1967792
EIC	Negative	315.2543	4.04	EIC_80	145910	2930788	231840	9909477	4145376	3989322	4524935	5532583	4949757	115863	96268	4907862	121640	3548043	1950952	7212833	3779236	7016879	162488
EIC	Negative	315.2525	4.03	EIC_80	2737159	2735264	157118	5986337	756666	1450413	4212505	2125283	1899794	3664970	2526167	4326987	3988616	3251527	1817402	2239796	4003300	6624879	2954583
EIC	Negative	317.2124	3.77	EIC_83;EIC_93;EIC_94	16358843	7367768	26763403	28329633	2301736	2536305	4720163	3361996	2804969	42891606	13726938	9484208	9349423	9547816	3289185	7114184	12830717	5710528	25379654
EIC	Negative	317.2124	4.28	EIC_87	23518059	6139952	29310659	17476286	3603596	2785088	6265681	8842436	4018640	28291730	10138914	10673523	10528879	8051028	4954950	6903263	10257232	9789621	8473357
EIC	Negative	317.2124	3.91	EIC_89;EIC_90	4566251	2031980	7558944	3450766	1347138	1179134	3242579	2854440	1229449	3142377	2466327	3433489	3930617	2236265	1207059	2681566	2842798	4347828	1641856
EIC	Negative	319.2280	4.76	EIC_96;EIC_98;EIC_100	26691716	11237066	2071542	77324363	7893637	14095109	7050101	40061722	75096942	5504253	14181317	44912760	97362878	70420759	23937698	18641293	163443204	125060715	20287617
EIC	Negative	319.2280	4.49	EIC_99;EIC_104	1204344	509647	1266972	1099835	581805	703609	528539	752587	1437396	1196576	655187	1023902	1269782	1268831	645503	1278405	2370811	1817986	607841
EIC	Negative	307.2645	6.57	Eicosadienoic Acid [M-	18775061	21690965	21878430	28116185	27338247	23422385	30794152	32425968	41288199	26265447	30821655	36231249	31522981	26284568	22766182	22899204	30229282	48751329	29240356
EIC	Negative	301.2173	6.25	Eicosapentaenoic Acid	41756245	123820505	48871225	144447396	139206165	70897225	105798543	75401452	261927717	64914222	69781691	55135227	130236180	78663375	68396952	117501857	111826855	61278183	50468310
EIC	Negative	305.2487	6.50	Eicosatrienoic Acid [M-	22085023	38605194	33676376	48334428	36263986	33723118	43922401	41887023	44541974	32756841	49390172	40142737	45342049	36066088	36587527	35963685	51209355	40800002	38655973
EIC	Negative	281.2489	6.56	ELAIDATE [M-H]	123822641	133953869	161118761	178739429	176108187	136146440	188193155	192997960	228788657	158468382	283090764	222226358	354288303	217234469	237696421	191654035	230417364	345862939	234545748
EIC	Negative	301.2173	6.36	EPA [M-H]	39339235	27594240	50877846	25636115	22988268	21856164	28620254	25235191	24294591	36202763	24110060	18867677	24878477	20731410	18738434	27737440	27215447	35938122	32173649
EIC	Negative	361.2384	6.36	EPA [M-H+Acetate]	804165	516366	979642	355714	476659	518416	540827	528322	377075	817580	470472	366784	495890	416877	397306	526435	492441	784877	669483
EIC	Negative	337.3115	6.71	ERUCATE [M-H]	3021349	4680388	3932775	5969322	7242599	6608979	5319723	4830498	5032102	4567166	7084696	9929202	7227016	5525580	7042019	5733482	5700752	6992980	4855427
EIC	Negative	329.1762	2.52	Estrone [M-H+Acetate]	627990	562496	825581	906728	280585	212078	255885	349863	176191	508051	335855	411356	424708	362245	361528	412511	379065	487385	396429
fpHILIC	Negative	140.0118	3.98	ETHANOLAMINE PHOSP	787491	431958	261908	514232	394318	808365	384886	386965	577785	404147	333838	599314	483852	396427	446942	745105	732857	714862	571788
fpHILIC	Positive	142.0262	3.98	ETHANOLAMINEPHOSP	754722	432575	270466	539107	392394	858666	389758	392047	637286	337256	346978	571197	476671	350264	386970	751272	723678	704875	535380
EIC	Negative	449.3634	6.55	FAHFA_449.363085	4287549	2360767	3066729	3042427	4088707	2323853	3754654	3250982	4210909	4858239	5650856	2754224	4818778	5457227	10143098	8029775	3598004	3650052	6928517
EIC	Negative	451.3798	5.79	FAHFA_451.378735	254785	731106	1623907	8756428	3124830	665478	2397125	1719011	2245894	895324	305137	1872637	817665	88125	61721	477812	606828	1289389	183668
EIC	Negative	499.3801	6.57	FAHFA_499.378735	695051	669708	600444	698087	1133914	634177	1171572	1151383	1358039	771964	1177727	781609	928986	948035	1458779	1774705	810903	751081	1249124
EIC	Negative	499.3800	5.58	FAHFA_499.378735	90939	148949	214244	2145377	350717	126102	257813	508832	1005252	213330	114990	257124	314708	36370	41157	211773	212217	443586	69195
EIC	Negative	501.3955	6.59	FAHFA_501.394385	3077153	3107917	2939571	3103138	4585185	3156127	5960514	4671606	6220535	3981057	3900124	2945105	2862383	4308751	5337158	7008870	3834420	2982158	5541877
EIC	Negative	501.3955	5.80	FAHFA_501.394385	55510	259647	214267	2191701	712999	163326	398702	381068	955288	179408	96322	202484	313892	21296	30869	165575	251960	193933	58873
EIC	Negative	503.4104	6.63	FAHFA_503.410035	1323625	996233	1038657	1238382	1617442	971030	2330952	1671641	1490417	1376540	1331610	1345991	982458	1457348	2243054	3031931	1271273	894016	1918247
EIC	Negative	503.4109	6.06	FAHFA_503.410035	327105	477342	1253389	3826653	1046353	264910	780327	1231508	1680181	578422	242312	462797	666886	175764	167617	341017	510371	1183217	211709
EIC	Negative	505.4270	6.67	FAHFA_505.425685	288608	259069	316865	375033	408958	366459	487310	452820	520187	419298	317636	296776	313340	342458	389687	521526	278120	237927	515233
EIC	Negative	505.4266	6.34	FAHFA_505.425685	929645	1483406	2026148	3419898	2193560	1535234	1894702	2376174	2185128	1271896	907201	1176546	1388389	603374	740916	902189	1001723	1636647	828817
EIC	Negative	525.3930	6.06	FAHFA_525.394385	157636	197323	412185	1042783	397423	125057	263530	391971	507177	216891	109839	173863	271012	80842	76722	171873	179792	416997	99797
EIC	Negative	529.4263	6.63	FAHFA_529.425685	6416608	3585791	3474106	4427364	6502825	3761830	8856759	8130187	5364792	5490745	4274973	4581562	3266108	4876123	7178748	8541270	5134146	3664787	7359674
EIC	Negative	529.4263	6.70	FAHFA_529.425685	485829	408175	465676	504693	630321	440942	600776	556748	545914	455560	532317	452321	367501	525779	656601	567370	286437	393676	511152

EIC	Negative	535.4738	6.53	FAHFA_535.472635	828676	964767	1561079	1908255	1336773	975744	1521206	1640030	2110758	1152335	1708533	1262644	2028019	1739100	1125314	1904472	1802115	1475008	1231771
EIC	Negative	551.5055	6.58	FAHFA_551.503935	348927	524916	539367	708087	653040	514298	758482	651422	1223786	485612	515138	433020	728929	640579	562100	804136	826015	593567	598012
EIC	Negative	555.4425	6.70	FAHFA_555.441335	459393	422278	359720	444455	633171	434503	598725	509544	406697	350863	538837	482345	414509	505207	617052	398058	285350	395287	514046
EIC	Negative	555.4419	6.29	FAHFA_555.441335	9570693	14766058	24793610	39685485	26034000	12257607	19370277	24675616	29981149	13357126	9023753	11752323	21188987	8992769	5180990	12589050	17023208	18779303	9731787
EIC	Negative	555.4425	6.63	FAHFA_555.441335	879748	498814	579175	737430	934985	675398	1186601	1025358	561384	830534	723595	778572	564951	757882	1077681	1113880	778226	692438	1057696
EIC	Negative	557.4580	6.67	FAHFA_557.456985	5172547	4565304	4977899	5432464	6700649	6638626	7714838	8654638	5298701	6521293	5203234	4966325	4906250	4825792	6014084	6348631	4942807	5661293	8667804
EIC	Negative	559.4731	6.70	FAHFA_559.472635	3216574	2574719	2834293	3248738	4670858	2996951	4401367	3996244	3130072	2557578	3739868	3208551	2556596	3552833	4213877	2901938	1996239	3024509	3373329
EIC	Negative	559.4716	6.47	FAHFA_559.472635	41032510	50348298	49668698	51463355	52743432	40329612	50976624	50027661	70087557	56875709	43680081	49236849	50771921	42528403	43455701	52571024	52975365	65588014	45727432
EIC	Negative	563.5049	6.65	FAHFA_563.503935	431548	784583	1301943	1609598	1053029	1195930	1154639	1293432	1750446	737555	597523	853328	1421735	780299	512477	1026099	886230	905020	581317
EIC	Negative	563.5048	6.55	FAHFA_563.503935	7407676	6651239	9529378	10826528	10132700	6314883	11101477	9853144	12991891	8238243	20056592	13422782	25674191	12498565	12365450	12413109	15129949	22940103	10340876
EIC	Negative	581.4579	6.41	FAHFA_581.456985	1484208	2619907	4246049	6677966	4119208	2411683	5194352	4796085	6315357	2105610	1976146	4191010	3507951	1459217	1052427	3176067	3117396	3682277	1374520
EIC	Negative	583.4726	6.46	FAHFA_583.472635	2463625	3803153	2281561	5603873	4358645	3333092	4613225	3762936	5208319	3335669	3504930	4127379	4177896	3191943	3274958	4480087	4342154	3707727	3590445
EIC	Negative	587.5083	6.84	FAHFA_587.503935	95017	94614	59194	74095	84711	62697	51805	44766	354664	35813	68807	73281	87691	62514	79633	84335	238089	80406	66056
EIC	Negative	589.5218	6.57	FAHFA_589.519585	1521514	2151945	2052888	3926272	2398655	1952980	2966923	3987116	3536102	1689063	4104044	3005855	2829055	1848439	2325312	3955778	2614369	4678196	2295317
EIC	Negative	593.5530	6.71	FAHFA_593.550885	191490	386715	343781	516015	425962	299724	486250	416041	385223	91478	308920	364027	304265	280377	205951	334626	290869	326187	203447
EIC	Negative	603.4430	6.37	FAHFA_603.441335	298415	41626	407401	125925	49428	0	136693	65580	124786	221597	124101	59088	75083	52244	0	132910	165296	135959	119929
EIC	Negative	607.4730	6.44	FAHFA_607.472635	30935076	33399766	1476807	39029194	32408575	23624528	36103507	29308851	33218461	16356016	27480407	29955877	35515719	20215258	19746415	32567700	31083886	24077143	28365746
EIC	Negative	619.5682	6.64	FAHFA_619.566535	749063	805070	1083774	1471349	1410092	1107370	1100432	1320474	1629012	1193292	1135888	2646068	1276311	946485	1072160	1724097	1170612	2270736	681024
EIC	Negative	631.4727	6.44	FAHFA_631.472635	2842897	5503640	2319215	8121231	4453821	2613036	4346910	4566163	6538985	2317104	2809748	2468181	5091809	1574338	1705640	4903486	3066673	2855624	2463544
EIC	Negative	277.2174	6.29	GAMMA-LINOLENATE [M	259445398	334155311	491897270	677875711	486466456	292689418	386815542	479804996	563344058	312079288	239928283	281701676	428415830	232686808	167898290	306540235	377969995	406482534	245664416
EIC	Negative	337.2327	6.29	GAMMA-LINOLENATE [M	0	547758	0	0	0	558734	0	881101	961921	0	434250	0	592705	441030	0	522010	765232	0	0
fpHILIC	Negative	195.0510	3.11	GLUCONATE	21164068	17612336	12994808	16134800	25703544	34287940	28567742	14126324	29128876	15589673	21883266	25153206	19660910	20165750	22397164	24640040	23256956	12348842	17327818
fpHILIC	Negative	259.0231	4.35	GLUCOSE 6-PHOSPHATI	504939	224863	108276	236685	235381	481234	174557	154529	304693	177435	143146	284670	206700	241928	181199	512457	365611	283296	322143
fpHILIC	Negative	193.0351	3.92	GLUCURONATE	2035132	1500301	1334746	1690109	1684470	2293761	1287790	1174372	1678493	1597437	1508221	1594563	1191904	1270611	1491711	1929126	1475601	1855687	1786952
fpHILIC	Negative	229.0117	3.90	GLUCURONATE (M+Cl)	3052424	1411931	1890019	1716775	1975485	2467613	1964698	1231983	1640188	989641	887682	1002781	2278989	1910830	395260	846986	3774681	1083310	708185
fpHILIC	Positive	130.0498	3.47	GLUTAMATE(M-H2O+H)	135620608	99550528	117003168	117488536	98255928	103656328	96969312	98774168	106264952	106611888	106944576	93763224	93868256	84208632	113740040	90700560	99826344	107863928	92925176
fpHILIC	Negative	145.0619	3.47	GLUTAMINE	175012512	115482760	145489584	126490824	106152768	113358816	110111424	109731248	100659616	124580104	110586216	111500136	110135104	87360104	140132304	104941240	107288560	137916528	79360640
fpHILIC	Positive	147.0763	3.47	GLUTAMINE	524928832	393783168	470703008	465122080	395777184	410887680	394788320	382990272	429661632	419949472	424219168	392478816	386596320	348605344	447700768	367112576	447433376	434528896	373632832
fpHILIC	Negative	127.0513	3.47	GLUTAMINE (M-H2O-H)	5399409	3609923	4212449	4140890	3356536	3525554	3405654	3579921	3049477	3871420	3565726	3483246	3405531	2703501	4443388	3219687	3619082	4027338	2407960
fpHILIC	Negative	167.0442	3.47	GLUTAMINE (M+Na-2H)	1438585	1256255	1405155	1286944	1179280	1199714	1309834	1188582	1168504	1347333	1267121	1210429	1191924	1034656	1532426	1232476	1233553	1355284	921714
fpHILIC	Positive	169.0581	3.45	GLUTAMINE(M+Na)	10063963	10013392	12825250	12456639	11412187	11650433	11092460	12818326	10502452	11655612	12209981	11606783	11109990	10248242	11837445	11476983	10992460	12239636	7957108
EIC	Negative	448.3079	2.97	Glycochenodeoxycholi	1052932	103950	1032205	472139	23652	0	84146	611602	33783	0	0	0	0	0	0	0	0	60605	14262
EIC	Negative	464.3024	2.13	GLYCOCHOLATE [M-H]	13292092	805388	52118404	18816324	1193151	1020663	1055534	37266458	2197983	6079248	108149	64892	401875	773353	200504	62248	388789	11790119	336516
EIC	Negative	448.3081	2.09	Glycoursodeoxycholic	859183	79394	1975707	871635	97805	83078	118191	1897538	124144	0	0	0	0	0	0	0	0	0	0
EIC	Negative	309.2799	6.64	Gondolic Acid [M-H]	13345937	23594155	25508340	36772030	34978594	26221225	30794298	30884370	34412069	26623116	27129561	52549012	36397494	29732540	27473345	50939931	36492277	42637035	27101759
EIC	Negative	369.3010	6.64	Gondolic Acid [M-H+Ac	377966	504170	350250	685591	492408	624594	595280	761126	640961	526063	407551	532420	443310	539629	468592	725358	568109	528691	708990
fpHILIC	Positive	118.0612	3.65	GUANIDINOACETATE	19563672	8084588	15277096	29573390	7837655	14806788	9242879	18837918	7166400	18005528	8917783	9524854	8751004	6561233	9395342	9028141	10539670	25728484	9076664
EIC	Negative	267.2332	6.50	Heptadecaenoic Acid [	5612460	6618146	9586990	11463825	9845984	7597134	10026950	13227517	13147013	7993157	9203369	10734016	12312832	10532607	8985185	12926012	12443561	13040542	8591502
EIC	Negative	269.2488	6.58	HEPTADECANOATE [M-H	5976396	5951186	5753221	8181426	8428891	6793088	8656190	8844242	13203050	8039376	9387029	8053889	10108695	11299862	7758679	12025890	12230719	9824412	11631162
fpHILIC	Positive	156.0766	3.32	HISTIDINE	167834416	82345752	167047376	113778480	58880908	74912208	67887792	97924728	59043308	141947648	84814224	95999840	76073120	91765128	119627408	75880568	71227472	143647840	92484080
fpHILIC	Positive	178.0583	3.41	HISTIDINE(M+Na)	2211689	2052228	3990285	2623440	2050784	1840138	1695165	2508613	1619538	3588623	2281382	2260962	1832663	3012580	2706375	1866584	2029244	3066961	2714810
EIC	Negative	267.0462	0.57	HOMOCYSTINE [M-H]	49561	48608	139705	368897	99190	64124	109830	128030	677510	251785	90062	103822	231868	16147	54498	245947	87308	164586	48122
EIC	Negative	335.2231	3.94	HXA3 [M-H]	367595	282910	206013	713880	244162	302185	245909	420049	1417039	188934	199163	390488	848331	674479	241887	559702	1437350	948048	633912
fpHILIC	Negative	108.0125	3.39	HYPOTAURINE	2147646	744667	1075875	1603066	694960	2030282	447721	1473318	299650	1247462	330925	796184	324125	388616	224971	1081192	757012	1356274	1029079
fpHILIC	Positive	110.0273	3.38	HYPOTAURINE	3924614	1265200	2371702	2921466	1483628	3957472	830969	2903738	592244	2521085	700340	1624660	724761	505757	485446	2089498	1340959	3003506	2027647
fpHILIC	Positive	137.0455	2.07	HYPOXANTHINE	19971278	2461274	6536722	1280505	18761872	14942368	5889717	1106009	12278198	1498630	67017	52960	9868556	13755456	0	50214	18839282	101648	57781
fpHILIC	Negative	212.0024	0.97	Indoxylsulfuric Acid	400179392	331106304	270907296	121961936	467704960	649193088	343881632	234388736	226679616	2895514	2672727	2789161	3234862	2428179	2683284	3647053	1966605	1448449	2494309
fpHILIC	Positive	269.0871	2.43	INOSINE	4572844	848428	6372614	555548	3054350	2428219	1145725	427946	2376666	661908	38753	0	3580128	3005004	0	0	5740045	0	0
fpHILIC	Negative	267.0735	2.43	INOSINE	2229808	489801	4013476	326955	1777279	1473377	691692	254056	1544821	402172	45381	0	2104407	2028126	0	0	4230770	54102	0
fpHILIC	Positive	291.0691	2.43	INOSINE(M+Na)	4642139	2380533	9191775	1186520	5110212	4384009	2451871	823972	4341678	1604413	88752	63953	6212806	6291816	0	41312	8385979	150729	0
fpHILIC	Positive	132.1019	2.40	ISOLEUCINE	212473664	197085856	171798048	122198760	159795456	230227168	180192512	95230672	172949600	180113568	203316720	213930256	180333840	234901120	365617664	239221600	152758512	151806832	216472256
fpHILIC	Positive	209.0917	2.38	KYNURENINE	4932654	3283268	2727064	2392839	2679455	3513602	2614968	1215777	2161748	2527697	4152203	3790687	4595338	3425043	2894244	2887405	2403557	2107267	2878326
fpHILIC	Positive	162.1120	2.92	L-CARNITINE	680463552	670778368	580165504	406705248	403863104	619458816	522052320	422297216	315354944	453968512	582961984	554792000	447513920	474739776	536488736	415163360	402604416	449884896	541036800
fpHILIC	Negative	196.0735	2.92	L-CARNITINE (M+Cl)	8044624	9020647	7968243	6724665	6121044	9280957	7480825	6484891	4827061	7132252	9990031	9014350	7030552	5173123	6440314	6315610	6118177	6510699	6475555
fpHILIC	Negative	130.0875	2.30	LEUCINE;NORLEUCINE	100817776	73640960	80406168	46763624	63805056	87313664	77411960	33925976	64321240	67339680	78148856	87649672	66400936	95186032	174464320	77416856	60244352	58991216	82817672
fpHILIC	Positive	132.1019	2.30	LEUCINE;NORLEUCINE	189195072	193054112	143600640	85001112	136738976	187177008	170453408	57385856	147117584	127061976	187542352	201406624	154855776	291023552	414691520	173306800	142601584	105729080	225531392
EIC	Negative	367.3584	6.89	Lignoceric Acid [M-H]	2292560	2234902	2447240	3118392	2711304	2708598	2718374	3514273	2880267	3608506	2942395	4637756	2666275	3595721	2539266	1979291	3238577	6454556	3066719
EIC	Negative	279.2322	6.47	LINOLEATE [M-H]	419138530	480330856	444173183	452102032	452447730	393252479	460647990	493068305	555038210	499453434	457664665	497197371	511311374	482739296	448491718	511588501	540439220	598534999	451139698
EIC	Negative	279.2322	6.57	Linoleic Acid [M-H]	9466635	9415235	12076428	14972692	12892256	8953025	12278650	15206481	20505597	12322063	13266326	13097177	17271528	12749131	11705334	15257724	16229754	19320359	12632035
EIC	Negative	375.2912	5.17	Lithocholic Acid [M-H]	3760450	245453	3388562	1498100	490143	208826	371094	1484969	160135	1206213	142763	52834	56873	0	30517	36934	0	298537	160009
EIC	Negative	435.3115	5.17	Lithocholic Acid [M-H+	7755957	460908	7137458	3282981	1116882	490151	764298	3115121	325427	2663038	248114	130028	132934	0	52251	75782	26856	631253	400982
EIC	Negative	241.0726	0.95	LUMICHROME [M-H]	133481	124088	118721	122744	103334	149905	134477	139415	131371	121604	101298	102040	98517	114270	112617	123005	108398	121234	118470
fpHILIC	Negative	133.0142	4.15	MALATE	147217760	121517544	117291376	121322752	127057128	123160792	113773992	104976336	83203528	141552800	115420600	106854472	72676920	147859712	171690736	93308928	114360488	144084240	117876416
fpHILIC	Negative	115.0039	4.15	MALATE (M-H2O-H)	7834171	6595945	6412463	6785534	6875038	6816782	5914278	5508622	4625488	7679748	6099419	5644621	4030681	8150081	9433924	4973251	6116099	7894203	6539173
fpHILIC	Negative	181.0716	3.05	MANNITOL;SORBITOL	1759013	1535790	2247218	1390612	1560312	1504567	2266146	2021234	1320549	1894087	1812976	1541185	1451003	2317358	1939412	1485505	1672476	2095428	2053595
fpHILIC	Negative	148.0439	2.46	METHIONINE	30814452	34770572	24975906	13179981	16624087	29237228	15325382	15892671	17580436	19779004	22829032	29832180	20655888	53727740	42433720	22132632	24495526	29426216	45137336
fpHILIC	Positive	150.0579	2.46	METHIONINE	321271904	338419712	263415248	134470704	179196400	268819200	188026144	174592928	195986576	191512256	254459072	290544320	232019200	470762272	356072640	219794464	238744496	278088544	429641120
fpHILIC	Negative	184.0205	2.48	METHIONINE (M+Cl)	201621	378662	216951	111555	75740	187061	57486	150946	93815	255982	336134	437936	133314	743924	915942	153546	137013	405637	934835
fpHILIC	Positive	194.0219	2.46	METHIONINE(M+2Na-H	312260	752732	518512	331613	322326	503189	425626	428535	361959	433485	438900	502614	449246	675047	679540	391170	428279	491705	768041
EIC	Negative	227.2013	6.34	MYRISTATE [M-H]	99700096	2773346	203616144	290925152	150816736	131959240	159054960	230953424	211029552	124123104	80106296	131266992	122356032	75645864	1082205	100000976	118229328	146851920	100134816
EIC	Negative	287.2231	6.35	MYRISTATE [M-H+Aceta	1305521	1577198	2951709	3580980	2405772	1818485	2190374	2798363	2768792	1605471	1088247	1591766	1794609	994639	747656	1423769	1499417	2226140	1340641
EIC	Negative	227.2013	6.36	Myristic Acid [M-H]	99700096	128685616	203616144	290925152	150816736	131959240	159054960	2355701	211029552	124123104	80106296	131266992	1982376	1091888	745480	100000976	118229328	146851920	1221439
fpHILIC	Negative	188.0564	3.70	N-ACETYLGLUTAMATE	2057953	2095932	1251863	1241113	1580760	2645010	1420891	1162091	1228970	1247345	1657342	1450395	1478541	2295514	2163534	2194199	1775303	1391865	1956625
fpHILIC	Positive	190.0708	3.70	N-ACETYLGLUTAMATE	556985	801129	583007	655017	756809	997196	837927	585298	702847	670896	767885	754758	545853	1089671	1046638	951978	710994	562304	790150
fpHILIC	Positive	189.1229	3.42	N-ALPHA-ACETYLLYSINE	471508	317326	380016	519680	475935	639334	436120	286507	359819	644048	588740	624782	556827	406709	445679	635512	398276	791753	653455
EIC	Negative	438.2940	6.20	N-arachidonoyl Dopam	4795786	1775128	3103110	7084799	1407471	1482476	1537226	3616953	2399708	2643256	1565128	867360	1598061	1698477	1607044	647971	1573249	2018730	936437
EIC	Negative	416.3090	6.44	N-oleoyl Dopamine [M-	12033258	6873018	2369025	5549458	4211950	6778318	7579473	7149797	3480159	5682243	7536126	3795083	5201259	7521781	8210380	2624512	7053814	3801398	4408776
EIC	Negative	365.3427	6.79	NERVONATE [M-H]	2729727	4092264	3399459	5141612	2921509	4447843	3427885	3492834	2932940	4481603	5485359	8262884	5575702	3214600	3070176	3817639	2917679	5917300	3710149
EIC	Negative	365.3427	6.85	Nervonic Acid [M-H]	188840	310786	250881	438803	215359	323298	319405	270951	248052	463772	421728	709221	463890	257329	269534	334163	313690	427032	267799

EIC	Negative	269.2125	4.75	Novel EIC_1	1291290	1234596	2067090	1857813	1985678	1589034	1848629	2322468	2345999	1366703	1129953	1314313	1333454	1374252	1084035	1828301	1297793	1645224	1353187
EIC	Negative	345.2438	4.94	Novel EIC_13;Novel EIC	371544	197414	176989	704750	178427	240874	186492	275767	906272	209630	145543	311177	620743	322658	230608	379604	813626	611084	257155
EIC	Negative	345.2435	5.29	Novel EIC_14;Novel EIC	548980	113311	117573	955957	127000	281852	181527	277789	479592	121618	106081	342845	413020	171075	240350	206246	572502	686051	230942
EIC	Negative	347.2595	5.22	Novel EIC_23	232017	62335	149776	184975	77674	93585	82179	141122	125091	196414	79551	148194	109084	39709	84805	178740	174882	159312	109184
EIC	Negative	347.2594	5.60	Novel EIC_24;Novel EIC	460451	202150	172781	1097916	254289	323128	240676	437815	943785	186183	178330	568068	816027	460585	327151	451150	1235621	1166548	374008
EIC	Negative	365.2694	5.67	Novel EIC_33	404818	214271	420429	367242	246161	186780	263095	321122	233187	345389	189944	181237	188451	157162	239517	234308	176039	305574	180932
EIC	Negative	295.1541	5.18	Novel EIC_4	213562	168075	0	357414	267998	0	288191	280447	0	208267	0	212066	259846	0	0	187376	187115	0	0
EIC	Negative	295.1588	5.18	Novel EIC_4	224982	185609	295589	442528	284477	234441	321056	315431	338980	212707	204841	224929	313688	155859	147271	184679	229434	315427	172292
EIC	Negative	309.1710	2.73	Novel EIC_6	2801515	3941745	1932157	2478474	3291824	3150306	3580132	2759325	3185264	3306864	3851194	3413713	4055231	3443620	4128913	2871559	3869595	3956003	4156219
EIC	Negative	325.2371	4.92	Novel EIC_9	278205	127213	371415	374752	190353	144152	166749	351869	126609	301364	148418	179422	177294	166996	142326	178112	211806	498298	123790
fpHILIC	Positive	204.1226	2.32	O-ACETYLCARNITINE	252386896	289114848	337102784	504982528	291623424	369159808	385057824	324496576	625402816	304302816	401348608	391777440	434942464	168504864	198548672	523925248	267362464	338750656	230938064
fpHILIC	Negative	202.1086	2.31	O-ACETYLCARNITINE	296554	323650	353225	509159	296454	349652	393416	367303	595019	331915	418960	413682	472251	198048	232442	499507	271154	363682	220963
EIC	Negative	384.3127	6.36	Oleoyl Ethanolamide [M	3255840	2913322	2436172	3994120	3300164	3542223	3513855	3344328	2379535	1995769	2316541	2505368	2372227	1492576	2023047	2258012	2047602	2328619	1612233
EIC	Negative	355.2853	6.39	OLEOYL-GLYCEROL [M-H	937275	683770	1220901	986196	335144	306059	153402	141159	0	484771	80254	403895	459765	200380	200679	425228	392000	710840	209625
EIC	Negative	415.3060	6.44	OLEOYL-GLYCEROL [M-H	45278836	27232203	8769027	21627335	17309883	26429824	28771337	25698067	13492916	21996242	29327089	15208911	19861602	29931830	30916522	10037265	26329033	14399644	17030251
fpHILIC	Negative	145.0141	3.88	OXOGLUTARATE	1598343	1213601	1875250	2581616	944016	1407106	1347403	2246129	904006	2507589	1227150	717747	900463	1991018	2082764	754681	1224245	2067082	1229374
fpHILIC	Positive	176.0690	3.50	P-OCTOPAMINE(M+Na)	2216851	1155050	2046980	3253482	3672249	3130153	2668658	3618098	1026305	3848465	3978365	4024449	3934593	0	2982839	3759838	3990629	3053717	684946
fpHILIC	Negative	341.1091	3.53	PALATINOSE;SUCROSE	2391539	49419260	29723630	3460413	4510216	5428136	18158210	8550205	37537600	2343553	1545857	4437346	4998860	44063472	4079867	3333763	11599565	4607508	187939216
fpHILIC	Negative	377.0860	3.53	PALATINOSE(M+Cl);SUC	145414	1790201	1233869	251128	341053	488085	999855	404065	1403898	156745	139472	347326	483139	1398442	332628	232346	738638	301909	4360407
fpHILIC	Negative	363.0904	3.55	PALATINOSE(M+Na-2H)	75374	710722	619765	127855	154053	164232	466583	247988	695076	90194	62272	138063	161892	754228	134184	107009	314061	159064	0
fpHILIC	Positive	365.1048	3.53	PALATINOSE(M+Na);SU	2589430	14167190	12673654	3409331	3750652	4091804	10273056	6903117	13452089	2373143	1666290	3791091	3891149	0	3798218	3190543	7905948	4469640	9856480
EIC	Negative	253.2170	6.42	PALMITOLEATE [M-H]	145406976	152334531	303893616	329755900	250528675	173611384	313063040	284474688	339027817	194282115	146740833	198662525	236811715	123373036	125394296	202712497	224613497	236853067	109395604
EIC	Negative	313.2385	6.39	PALMITOLEATE [M-H+A	37307512	17588604	36869444	29376092	24460200	18469254	1711714	1965191	2021416	25644950	14404268	1254815	13456887	11830691	682320	13913844	15027454	29157098	13006392
EIC	Negative	313.2386	6.52	Palmitoleic Acid [M-H+	941549	1112505	1133023	1857017	1779123	1653452	1678865	2819674	2618564	1802108	1479744	1472840	1473803	1822854	1277631	1600124	1590344	1294627	1365255
EIC	Negative	358.2962	6.24	Palmitoyl Ethanolamid	2907898	2082838	2409014	3163632	2624664	3436454	2616987	2866191	1502490	2277217	2649762	2554176	2018348	1591867	1942082	2130220	2260016	2405308	1939961
EIC	Negative	331.1919	2.41	PGA3 [M-H]	7488365	5176897	9741816	6505689	2203213	2369159	3909225	2245822	2185794	5537973	4383962	6842155	4117065	5000011	5852902	4561861	4838942	4537378	4808204
EIC	Negative	391.2135	2.43	PGA3 [M-H+Acetate]	1910363	1233439	2036905	1685753	559219	689205	984069	681275	519111	1136659	963560	1722210	1569279	1168605	1454926	1072904	1170708	1481619	1175442
EIC	Negative	353.2326	2.23	PGD1 [M-H]	220087	104675	184486	236104	105140	215575	130647	181303	156377	199443	91344	122820	161670	186972	115342	134228	272914	292522	98999
EIC	Negative	351.2181	1.48	PGD2 [M-H]	884669	1359779	946410	282933	534848	658474	1096461	0	712584	387119	1148232	1341157	1357411	1196976	1310044	1743687	1364113	271618	1089511
EIC	Negative	411.2388	1.48	PGD2 [M-H+Acetate]	1095958	2042671	789835	783201	711296	951546	1384320	0	855863	630457	1406978	1729666	1710621	1514575	1682807	2232767	1716306	293958	1316432
EIC	Negative	367.2128	2.37	PGG2 [M-H]	251774	91300	269945	150401	63264	87475	111811	134678	75979	169727	90914	118224	135777	98422	90026	134959	118891	66318	106685
fpHILIC	Negative	164.0716	2.06	PHENYLALANINE	85071552	47952824	61537572	36959576	34888080	51645988	39199472	32726562	40509432	51417020	41358944	47102824	39774052	57090140	65162700	44499320	35708948	52378744	46773232
fpHILIC	Positive	166.0859	2.06	PHENYLALANINE	483868864	276771392	378964288	246366176	225403120	283886752	243844768	237151472	263669648	317115232	267492752	285804192	233358352	374641152	409788192	290270880	211712800	341046816	270318464
fpHILIC	Negative	210.0284	3.97	PHOSPHOCREATINE	1096835	290313	239993	427946	288192	576263	439691	350858	369557	310137	348611	495988	351163	402097	330438	635983	432423	590768	382772
fpHILIC	Positive	212.0427	3.97	PHOSPHOCREATINE	2115027	803229	483350	994406	761085	1499832	1040752	759217	870967	664650	822870	1172110	832951	982397	774445	1490461	1028219	1364952	909059
fpHILIC	Positive	130.0863	2.71	PIPECOLATE	12485032	14812108	9729421	16489573	14302058	18409794	13856738	7896523	12165471	8890542	14140082	12425613	15618387	7732377	12165973	17617362	13546453	9852160	9256333
fpHILIC	Negative	114.0560	2.79	PROLINE	16625191	16468868	12510500	6167800	7480513	11201413	6004681	6682575	5735266	9525520	9375717	9655059	6822012	16774652	27086518	8479343	8111995	12125195	13828244
fpHILIC	Positive	116.0707	2.79	PROLINE	873386112	791868224	752803968	549392576	609965184	775746624	528468352	575783680	538394816	629582080	637772864	673619456	559630336	824745280	1015801984	607442432	588987776	725174848	769967488
fpHILIC	Positive	160.0341	2.79	PROLINE(M+2Na-H)	1085123	1182008	1125899	596818	686697	808188	459256	603082	541400	861602	797780	889712	733006	1384994	1538610	856477	751182	1173358	1138538
fpHILIC	Positive	138.0521	2.79	PROLINE(M+Na)	1722691	2145443	2101572	1371169	1518684	1711814	1017405	1338748	1175473	1844835	1890256	2100958	1334042	2282664	2436060	1420410	1579286	1913806	2109335
fpHILIC	Positive	151.0866	0.98	PYRIDOXAMINE(M-H2C	632753	607725	276421	271643	899055	992000	567797	339780	460282	30346	0	46020	32503	0	43319	52487	0	46065	0
fpHILIC	Negative	104.0354	3.62	SERINE	37373420	21773034	26605544	23226048	13815662	14502344	12576777	16295856	13025245	23399998	18428720	21213318	20555774	18819268	30305386	23516894	17125850	30442232	15207386
fpHILIC	Positive	106.0501	3.61	SERINE	18959366	15950545	15708095	11685174	10542176	12937495	11277379	9379645	12121649	16083645	14856634	15435173	15046096	16322972	23937090	17707608	14837737	14561895	15038502
fpHILIC	Positive	88.0398	3.61	SERINE(M-H2O+H)	524336	512821	495055	401543	266442	366862	351177	297888	349804	423298	396457	431337	407715	454470	682642	471354	347926	432837	424585
fpHILIC	Positive	150.0134	3.62	SERINE(M+2Na-H)	481058	508272	612491	614079	599608	438510	464714	695908	416534	708712	681953	570692	624607	491290	642456	625602	552053	599434	270999
fpHILIC	Negative	217.0478	2.99	SORBITOL(M+Cl)	7716562	6974347	11152418	5655548	2921522	6149001	8146462	2553073	2144267	3447275	4928614	5288219	5014271	1715157	3248586	4106533	3640542	5068689	4169903
fpHILIC	Positive	205.0676	3.05	SORBITOL(M+Na);MANI	1158928	1286454	1790387	1148972	1509625	1126031	2067502	1541995	1111669	1350300	1611191	1368652	1267850	1725804	1513791	1025190	1341896	1667430	1410248
EIC	Negative	343.2852	6.63	STEARATE [M-H+Acetate	431586	612919	614867	856464	863259	786408	823303	880576	1078842	914433	844954	1059522	892992	1316544	1067848	1034694	1302202	991036	908518
EIC	Negative	401.1253	0.49	SUCROSE [M-H+Acetate	133747	0	0	52731	113379	122142	321194	0	100602	73058	0	78034	0	526977	243681	65682	491037	178908	57165
fpHILIC	Negative	149.0091	4.37	TARTRATE	15094111	12592219	7521687	1146187	3639588	15697190	2514490	6309494	2702928	2014278	2527289	5751695	1305763	39707440	26669154	3359713	4252911	5511224	15485424
fpHILIC	Negative	124.0073	3.22	TAURINE	258183008	197679120	179003600	206087488	184434896	208790448	170824288	168347040	184636960	181709440	164633456	193226672	187087296	152999936	184855968	210262240	185004944	205769616	178442032
EIC	Negative	498.2901	1.97	Tauroursodeoxycholic	122996336	9643191	102523088	105989272	10911137	15098188	14037996	141358208	30051989	55336680	20381027	18152706	29951088	39358658	34049475	21753200	26128414	75484752	16138727
EIC	Negative	365.2338	1.67	Tetrahydrocortisol [M-I	0	248342	0	163566	1652490	276408	1075120	580654	1772166	57386	322268	239646	488558	480289	691880	1188089	472974	919754	291931
EIC	Negative	265.1811	3.53	tetranor 12(R) HETE [M-	158866	103541	222446	260781	160579	146593	157529	188173	132462	184493	135372	170222	136638	130641	82201	108921	109940	157168	114770
fpHILIC	Negative	118.0510	3.16	THREONINE	33793156	50245308	34712672	27450788	32884336	41754540	44443584	31506460	35991532	35341800	63704420	61249444	53131320	61623588	70678384	54169600	43706292	39196456	59294076
fpHILIC	Positive	120.0654	3.18	THREONINE	137991152	165095632	114081232	103380296	119591984	156780288	137402064	91196944	118778776	102946184	166958512	168263712	178191776	176975008	191936016	157594320	140810208	117237144	171110976
fpHILIC	Positive	102.0552	3.18	THREONINE(M-H2O+H)	10350449	13309226	8768889	7915801	9182767	12698375	10547496	6682692	9056430	7870430	12619431	13276970	13375280	13252612	14961969	12141030	10430202	9286115	12996557
fpHILIC	Negative	130.0511	3.32	TRANS-4-HYDROXY-L-PI	1337594	1439963	913794	1686418	1289865	2269285	1094004	1122085	1659672	1398412	1456878	1376644	1471440	831404	1161313	1656835	1663187	1345600	1224877
EIC	Negative	341.1090	0.50	TREHALOSE [M-H]	79854	929769	456368	91328	83046	81924	237205	81143	585541	59500	109953	39925	67164	336060	129851	79503	340989	389211	4628662
EIC	Negative	401.1295	0.49	TREHALOSE [M-H+Aceta	165170	1236192	742523	156203	206494	190069	284754	128405	861722	53987	196223	158991	130376	600969	264772	204417	483446	593766	6710650
EIC	Negative	353.3429	6.87	TRICOSANOATE [M-H]	1176554	61603	1223277	1619000	1854487	1394603	64111	64233	35595	65373	1951513	61296	39611	1782436	2002764	34337	47156	3875827	2218271
EIC	Negative	351.3270	6.74	Tricosenoic Acid [M-H]	652907	657019	747031	879207	803112	707320	683611	648991	448861	818155	763737	1047177	601893	534302	767907	585923	621444	1161343	539416
EIC	Negative	411.3483	6.71	Tricosenoic Acid [M-H+	1495064	2058437	1566435	2069301	2550407	2596265	3248681	3131461	2893886	2169838	1729334	1386542	1815812	1952075	1930580	1909982	1791248	1912991	2586609
fpHILIC	Positive	138.0545	2.52	TRIGONELLINE	2243477	3090821	937584	876096	3598855	6972140	5344891	1223616	4000295	0	4683905	2863152	4493678	3488959	2959934	3676845	2774016	985777	2352519
fpHILIC	Positive	205.0966	2.61	TRYPTOPHAN	169330816	216850640	176159696	111787056	151924768	193383072	188801376	118217176	136080704	146939456	199528624	184579760	163600256	228490384	263022240	192165344	165153312	171862176	187099904
fpHILIC	Negative	203.0825	2.61	TRYPTOPHAN	38410576	49019852	39281436	24386946	32677300	38420228	43678500	27813150	29295216	32818950	44422708	40227972	36892656	50566224	60606316	46188224	37696000	40306144	47348176
fpHILIC	Negative	239.0594	2.61	TRYPTOPHAN (M+Cl)	4778495	7804908	5930238	2944603	5144357	4950353	6674915	4367458	3973439	5158175	7361476	6319299	5702740	7913453	9832168	5414606	5624881	5564319	6563450
fpHILIC	Positive	227.0779	2.61	TRYPTOPHAN(M+Na)	3144598	4614440	3951445	2359004	3738939	3849385	4227772	2695222	3180142	3264314	4989011	3904520	4113263	4878282	5520578	3852674	4010646	3856957	4560105
EIC	Negative	429.2417	1.87	TxB2 [M-H+Acetate]	39061	104809	0	0	30523	67934	115835	0	105505	0	92114	54394	119422	108866	109794	101395	89541	0	94313
fpHILIC	Negative	180.0665	2.95	TYROSINE	30394498	20312728	24337992	15140713	13538243	16142976	12283363	16159501	13824405	20502740	12170524	14254570	13524697	24368836	25387902	14396082	18026562	27611408	18837422
fpHILIC	Positive	182.0807	2.95	TYROSINE	230140304	170193312	221434208	144805424	128753424	142894032	111237472	130915936	126962416	187487328	115070896	124603928	122874064	200748240	211754896	129851664	154960640	220843920	161902656
fpHILIC	Negative	216.0436	2.95	TYROSINE (M+Cl)	5446017	6739824	8383933	5983675	5427262	5599152	4624394	5886806	5286254	7711224	5005251	5737778	5539008	4242703	5724974	5550070	6920339	9137155	5437062
fpHILIC	Positive	226.0443	2.94	TYROSINE(M+2Na-H)	1489005	1467631	1766602	1477343	1295986	1159550	927374	1277485	1226155	1711774	1108955	1218197	1323095	1442302	1772916	1303435	1437530	1777585	1584425
fpHILIC	Positive	204.0627	2.94	TYROSINE(M+Na)	1770643	1870355	1911830	1253850	1338618	1415729	997217	1266394	1524943	2160026	990801	937616	1119163	1846153	1836454	1508210	1416169	1865383	1281541
fpHILIC	Negative	243.0621	2.01	URIDINE	1701353	536051	536314	1850434	515366	311461	904884	1144438	2370339	857828	743261	705988	1737848	1602319	1036942	453095	2065433	1004570	592852
EIC	Negative	403.0000	0.49	URIDINE 5'-DIPHOSPHA	7232632	5439470	5869649	6265993	12048508	6695817	9622559	7359401	5424203	7360442	5383773	4397513	5840342	32266246	7812848	4860264	5588727	6054403	6601883
EIC	Negative	391.2859	2.76	Ursodeoxycholic acid [I	7953512	2510581	10690048	10155870	4300151	2599524	5925052	3170363	1264712	670318	155337	0	168944	0	0	0	0	298545	176184
EIC	Negative	451.3070	2.76	Ursodeoxycholic acid [I	24620742	8071600	34537406	32301030	14234662	8166187	18371694	10226477	3977137	2166104	352456	237478	474683	75687	99930	83082	84505	927878	588253
fpHILIC	Negative	116.0717	2.70	VALINE	31434512	33715200	27266172	16517518	23109110	35432828	26726842	16217185	20355786	24054754	32012696	34389180	26988792	44046552	56781680	31068916	28118066	23783496	40257356

fpHILIC	Positive	118.0862	2.70	VALINE	189461264	226060384	198742560	151577840	173969760	235383408	196479616	146329952	158744288	182998896	246969584	242085520	209265616	310406752	333916032	209335728	184898144	178683760	248191360
fpHILIC	Negative	152.0484	2.71	VALINE (M+Cl)	291197	411555	425810	318034	364498	436629	401274	370351	343034	403326	459022	432626	394596	545982	603897	313273	430525	306001	479412
EIC	Negative	297.1348	1.14	unknown	44817	24121	30517	20623	73496	45322	55743	53792	56405	53842	87820	32767	37656	68449	43605	43338	37573	37503	48432
EIC	Negative	298.2834	6.67	unknown	310342	411479	365462	689813	619642	643453	617651	710669	856333	685383	512170	720710	635797	581158	468894	627413	639032	697075	708500
EIC	Negative	297.1347	1.03	unknown	87124	56778	77038	56965	163798	128783	131215	117848	140897	112032	156592	85086	87795	139771	113751	107190	87570	101410	109547
EIC	Negative	357.1557	0.90	unknown	567901	389588	488026	377744	937670	851545	886401	739437	924738	589158	778447	613416	519264	845386	722030	717758	552978	650286	792702
EIC	Negative	439.3798	6.79	unknown	550877	963413	698823	885118	975374	1438666	1130949	1341690	1233746	816359	584061	569079	795904	612891	614414	749354	653043	1029061	1149428
EIC	Negative	434.3358	5.79	unknown	445626	695775	708697	492492	1244558	1098772	1241566	1112405	1155192	436862	808239	513443	332272	986310	898089	835259	1090498	1521321	872584
EIC	Negative	358.1596	0.90	unknown	104960	73868	93951	75297	184849	153509	170960	132324	175611	113593	157134	112555	100384	163742	141380	135191	111527	122373	149121
EIC	Negative	433.3328	5.79	unknown	1583630	2326125	2336340	1781906	4214336	3577431	4115998	3929269	3979814	1456982	2691434	1802762	1206482	3491957	2998686	2943842	3550478	4960632	2894699
EIC	Negative	253.1235	3.23	unknown	242207	276492	241764	314555	330613	358311	371491	408318	381550	394232	229109	259075	266084	264442	289546	297060	293337	335569	343861
EIC	Negative	449.1739	3.46	unknown	269602	112711	130500	109947	386293	282496	308589	243479	264330	213506	286817	201133	162221	344813	242804	238747	160080	249357	339472
EIC	Negative	450.1772	3.29	unknown	167144	93168	95123	76036	307049	214250	236930	187912	215815	133581	187466	139167	118741	244907	174667	188585	128997	196649	253251
EIC	Negative	297.1349	0.92	unknown	348958	235376	290142	212882	829628	678397	681519	449399	736623	499535	814729	364594	415116	711425	438497	509354	403444	504860	625722
EIC	Negative	440.3828	6.79	unknown	218040	291600	216367	267094	292259	422296	347018	411531	407552	264878	187150	198071	266865	245503	322983	225615	208647	352255	355173
EIC	Negative	425.2560	1.21	unknown	50388	93664	52623	74391	177441	275554	164419	116947	241329	49650	57165	62713	56595	61136	137012	90857	124916	144529	130288
EIC	Negative	367.9042	3.33	unknown	0	0	0	0	145138	96728	87924	99760	55476	0	0	0	0	10600	76726	43883	73214	101221	67029
EIC	Negative	451.1724	3.29	unknown	272552	134292	148730	128648	414553	309816	320498	242597	299713	208223	281714	205837	176643	340106	241360	266369	169547	268021	330357
EIC	Negative	297.2797	6.67	unknown	1425990	2063365	1742492	3427826	2995072	2976513	2919126	3338736	3985260	3211956	2329386	3555199	2913269	2758294	2203087	2930341	3121178	3551428	3119455
EIC	Negative	449.1741	3.35	unknown	316616	121778	201866	116145	636707	465631	501947	347716	461162	262035	453514	264710	238831	513117	372274	437726	262617	426587	564712
EIC	Negative	370.8956	3.33	unknown	60682	29876	48972	18117	397943	254767	233031	265439	139058	25194	34459	18165	19835	43429	207508	105210	183621	240087	175983
EIC	Negative	487.2727	1.20	unknown	24073	56248	28341	42926	103249	159730	113802	59661	157322	30812	33737	38250	28741	40610	84435	63310	78635	74717	74852
EIC	Negative	451.1724	3.15	unknown	158511	74953	97449	81987	244783	175414	176914	140669	175522	118427	170737	136486	116173	237508	136407	173422	123025	158495	236070
EIC	Negative	258.9080	0.58	unknown	5360	26315	0	39183	47204	79182	103269	140091	111125	69678	102611	107574	107969	46479	84514	86125	109646	125073	65630
EIC	Negative	369.9016	3.33	unknown	0	0	0	0	157307	102152	86210	107800	52969	17664	10438	0	8885	11283	86233	30100	75974	88287	69482
fpHILIC	Positive	117.1021	2.66	unknown	78003	0	0	0	1464803	1701790	1142943	828640	625021	0	36457	0	57283	0	893334	865980	1234075	621188	1172382
EIC	Negative	474.2210	3.46	unknown	547207	293078	277763	194629	1013486	750924	724637	531614	677824	408825	709475	462981	371255	846730	572806	653689	424882	532219	801572
EIC	Negative	368.8983	3.33	unknown	161663	83352	157363	42474	1141081	697532	655745	733733	380168	78788	86445	77749	75887	82450	612661	333974	536727	685926	482111
EIC	Negative	366.9008	3.33	unknown	154426	85258	134751	49793	1151696	725309	644409	737878	385469	87366	88266	76755	68922	88305	603531	288513	515394	668832	513205
EIC	Negative	449.1738	3.29	unknown	746965	349250	405718	314595	1098012	857868	908396	648518	845494	546828	730880	572597	460762	936447	672305	716809	499251	743430	933182
EIC	Negative	643.4252	2.78	unknown	264464	396317	337658	388153	1432100	757724	1253936	1379386	1049419	114791	235705	164848	90405	1293461	67429	1094874	328165	1937919	895743
EIC	Negative	473.2178	3.46	unknown	1856939	978686	989293	658275	3562719	2465774	2543202	1773437	2327480	1436736	2353159	1590296	1334003	3017065	2000820	2162635	1333114	1852770	2718015
EIC	Negative	282.2520	6.66	unknown	3610920	4580718	6244073	8259208	5879463	7125767	6948838	7849973	10411226	5730108	5470785	6741416	9498906	6676482	5779968	7885359	7387244	7780951	5562788
EIC	Negative	450.1774	3.15	unknown	106602	70568	61238	46565	184381	133308	135579	98918	131020	86159	130027	105076	84128	162981	105580	138534	76197	117369	153482
fpHILIC	Negative	242.0864	1.03	unknown	965303	877885	810327	1086155	1203151	1066996	1037232	933433	1189144	941746	819817	765896	908262	666869	734063	886794	929692	819743	494187
EIC	Negative	349.2387	2.79	unknown	2352812	2789278	2260139	2035511	8227691	4215752	6623732	8401478	7013402	2608388	3769332	1129067	4492956	7292433	3779759	7541147	2302727	9939283	4310180
EIC	Negative	473.2178	3.41	unknown	2573063	1297341	1372053	905324	4703404	3358315	3255297	2298853	2911043	1682169	3050796	1993696	1718233	3822745	2571406	2843149	1807246	2215280	3679061
EIC	Negative	402.3054	2.67	unknown	1019253	420462	352327	363331	1063520	533036	527293	349740	536030	367907	539862	152076	535053	402390	310011	440051	928499	789036	340204
EIC	Negative	474.2212	3.15	unknown	785033	499957	444604	389002	1523194	1150196	1099806	803542	1112706	632534	1054257	798183	666300	1339064	863764	1029797	678246	841930	1291451
EIC	Negative	379.2129	0.84	unknown	25373	28410	31046	28000	93920	68929	78864	107409	59579	20159	26117	27624	22372	22133	61933	49945	34876	102469	43070
EIC	Negative	529.2768	4.82	unknown	412812	408709	345031	312278	707256	681528	487209	552083	467224	357268	514656	552579	391243	589791	553046	553697	514063	406346	774206
EIC	Negative	343.1702	4.42	unknown	368154	514061	352962	359527	609837	591275	628083	668466	553007	411374	414353	405284	415034	379958	486134	507891	474016	529062	607120
EIC	Negative	474.2210	3.41	unknown	716368	368775	387105	249750	1373996	941044	950213	647287	853829	499075	841922	586326	479841	1090544	736256	813710	529018	630244	1023449
EIC	Negative	430.3048	4.48	unknown	57791	142073	121484	73692	187560	318421	183406	236229	196355	49742	0	64731	51415	49381	229794	134169	48279	298881	154312
EIC	Negative	350.2424	2.79	unknown	499455	673848	601951	503115	1863994	976283	1561568	2001440	1614501	614147	901925	246418	999071	1689800	865918	1774141	532381	2415531	974702
EIC	Negative	295.1539	2.93	unknown	195202	193508	205488	791717	473012	276204	397830	513182	960203	361955	219404	323919	432851	129065	140337	369898	79680	504098	128634
EIC	Negative	412.3521	6.71	unknown	411204	589261	435756	556876	671937	686679	868292	870296	822134	584899	481199	408973	552098	914210	548583	524939	551211	568791	727114
EIC	Negative	473.2178	3.35	unknown	2251903	1513364	1010576	864201	6111656	4341282	4078255	2940219	4089386	1189504	3360721	2130367	1994661	4261489	3349682	3258445	2043142	3051688	4120821
EIC	Negative	474.2210	3.35	unknown	643164	426915	312084	244562	1775937	1268113	1173336	858042	1181005	382219	938552	647712	555899	1188994	928571	917126	578684	894181	1213894
EIC	Negative	473.2178	3.28	unknown	6977765	3528346	3715391	2976935	11782218	9254784	7803326	6035783	8198348	5329442	8044385	5780573	4765390	9916510	7042238	7667030	5314427	7297149	9643412
EIC	Negative	482.3154	6.33	unknown	2942917	3698195	3284554	3501657	4535577	4606208	3751341	4080343	4257339	2568361	2807799	2328966	2294325	2915850	2158890	2072130	3100627	2516716	3006896
EIC	Negative	261.1505	2.78	unknown	219119	184581	385031	375527	288936	278753	503349	482723	601399	502975	257053	191640	184692	114600	260471	266320	349594	218140	144658
EIC	Negative	473.2178	3.15	unknown	2629386	1700655	1548910	1336544	5315577	4017705	3773804	2770852	3870426	2172538	3656107	2722572	2272421	4652092	3019253	3486996	2400669	2918449	4408843
EIC	Negative	474.2210	3.28	unknown	1953924	1021548	1087372	878221	3325740	2682325	2237555	1721323	2369405	1565098	2280480	1680051	1385934	2855059	2013754	2191027	1523249	2124939	2759457
EIC	Negative	487.2693	1.20	unknown	25485	19970	26711	37967	95775	180109	143178	58405	131076	21878	23993	23780	21003	32150	85531	15148	20921	51157	39292
EIC	Negative	433.3176	4.66	unknown	396634	369450	395450	571174	678420	635146	514061	759855	1056405	699528	357559	450948	496229	454955	477323	778669	369640	444567	606372
EIC	Negative	449.1739	3.15	unknown	372060	216406	219880	145824	653066	503713	540478	357178	479368	338964	480248	360137	288424	591227	399098	477246	304585	406446	603251
EIC	Negative	644.4261	2.78	unknown	114785	0	79754	0	623123	63328	545136	599214	563787	119099	293346	0	340209	711893	337666	625731	131430	806018	0
EIC	Negative	322.1779	5.05	unknown	890684	647021	640078	988851	1114654	1464457	1284218	1299761	1465865	2177946	1607627	1174406	1112099	834872	841280	734070	947327	989036	922469
EIC	Negative	431.3020	4.73	unknown	1746423	1589688	1687832	2674560	2774909	3188263	2003704	3265575	3492864	2483085	1328856	1769514	1890765	2174116	2313168	2187917	1435773	1707121	3009200
EIC	Negative	449.3285	4.20	unknown	53181	197146	210951	137695	502751	294668	524486	366531	381038	165788	241386	153891	91101	108454	310433	335256	268940	380818	331287
EIC	Negative	527.2981	3.08	unknown	0	118293	0	0	146457	182325	133609	282884	289987	0	72452	122364	98351	228076	166140	120456	109930	0	104603
EIC	Negative	480.3096	6.33	unknown	63473052	81467377	69132214	75507564	91346273	94805735	77068097	90219483	85625189	55784117	60825155	49817259	46435861	60738013	46167199	42756143	65667583	52661707	61800524
EIC	Negative	481.3127	6.22	unknown	3100731	4194284	3146355	3780319	4672110	4699877	3619419	4454129	4584192	2630390	2925091	2513216	2285346	3167794	2138909	1930060	3129492	2958501	3243360
EIC	Negative	412.9560	0.47	unknown	111596	56717	97663	64866	148824	86640	100572	115544	97811	45512	44587	38621	105692	338809	120829	55971	76171	83039	92902
EIC	Negative	269.1403	2.01	unknown	155043	254463	124635	208536	544805	830284	954107	535248	652823	493452	249281	248275	223065	225751	345287	512294	639585	262923	785152
EIC	Negative	321.1745	5.05	unknown	5245799	3849363	3721452	5926476	6226685	8105267	7500056	7391079	8214102	12809406	9111524	6564500	6272250	5048599	4771841	4215103	5613316	5489640	5067952
EIC	Negative	433.3176	4.97	unknown	420777	401525	378955	620703	624401	698632	453076	716540	729625	607883	335095	405888	425849	455177	493674	490759	342812	391504	631879
EIC	Negative	481.3127	6.33	unknown	16576052	21303165	18186828	20135204	24190997	25127725	20287550	23369131	22705206	14787020	15669655	12952226	12160627	16076605	12234028	11122119	17448796	13851037	16504696
EIC	Negative	473.2178	3.00	unknown	2007962	1908692	1384334	1750146	2703818	3385536	3381739	2650420	3449341	2925556	2081071	1958942	2182907	3070908	2564978	2666652	2617116	2665030	3539978
EIC	Negative	403.2710	4.11	unknown	259711	234219	219853	326417	358317	445352	258773	428370	470301	353574	187449	208233	242826	281958	300065	257224	205189	212288	386796
EIC	Negative	432.3053	5.03	unknown	345368	307555	291280	522913	518033	656381	389435	621375	667825	465432	276496	369378	347558	495518	426501	451924	339169	329314	626870
EIC	Negative	431.3018	5.02	unknown	1324408	1266296	1220335	2071676	2056276	2574874	1563391	2376078	2590408	1905552	1103456	1397697	1442415	1870140	1707306	1673143	1368776	1357503	2416095
fpHILIC	Negative	872.5959	0.42	unknown	1148319	1062180	986548	1277832	1679924	1627052	1525717	1686314	1460364	1156409	1193277	1236051	1353998	1175891	1435644	1288042	1585217	1333469	1376578
EIC	Negative	451.1721	3.35	unknown	127303	79207	43471	63906	220393	175714	187421	121067	177998	63784	159922	95325	105284	191259	133372	158840	98430	153547	218285
EIC	Negative	480.3096	6.22	unknown	11782783	15844481	11973493	14140323	17407358	17391253	13375235	16541636	17635310	9354983	10717150	9268371	8413076	11806761	7674203	7259773	11518037	10201969	11876947
fpHILIC	Positive	178.1070	3.71	unknown	413111	371743	443054	384190	511398	506454	467666	435953	466197	360950	379517	371252	334409	291292	431494	424338	327603	366423	331109
EIC	Negative	613.5427	6.66	unknown	130869	74584	192626	425967	315500	441429	245770	506203	692918	322280	203313	232596	318713	337461	316134	316204	238049	226382	373365
EIC	Negative	255.0693	1.82	unknown	87238	50161	34288	0	250122	359260	331646	144136	214797	31603	51736	60643	59471	58880	99763	197038	249046	0	225060

fpHILIC	Negative	241.0831	1.03	unknown	8425455	7002569	7247959	9926228	10845545	10312342	8634412	7633372	10515620	8486437	6307129	6600940	8477054	6235940	6150014	7145732	8450771	7575480	4495982
EIC	Negative	433.3171	4.74	unknown	202846	172810	186476	267517	286396	317776	221647	356761	311816	269064	137607	136977	185150	180664	215987	192642	156712	164801	293156
EIC	Negative	492.3423	3.24	unknown	84188	123837	80590	68268	192458	182427	159709	138797	248210	73615	111547	125925	116996	174343	206127	167612	136759	89503	118591
EIC	Negative	482.3155	6.22	unknown	566266	809099	572451	699062	858316	862047	685992	856833	856432	497044	542559	515026	442908	606805	446620	397699	575975	598336	629148
fpHILIC	Negative	345.1542	0.47	unknown	767033	360526	676315	713951	1079394	852738	797951	674015	1047348	468039	690017	767909	494901	253173	638728	1030785	596763	544020	305064
EIC	Negative	270.2159	4.30	unknown	175940	127574	193564	197656	211424	171646	270452	226357	207090	172483	138674	127778	155847	151571	116532	235805	178840	200675	127185
EIC	Negative	475.2680	1.71	unknown	296710	243281	218559	67808	672139	255158	699001	555917	380624	75193	25087	27517	31395	87021	65871	79671	32686	101521	79226
EIC	Negative	433.3180	4.84	unknown	289675	241572	218503	315583	396379	491528	281658	365171	503551	331381	206726	245272	250350	336408	334823	336824	188956	188331	503880
EIC	Negative	423.4205	7.14	unknown	49751	68370	40295	58438	86827	76182	68324	67887	112808	82000	37133	86030	49540	54189	61656	67564	60138	67804	44296
EIC	Negative	483.3076	2.67	unknown	136851	0	0	0	214813	113059	92150	38963	86200	37524	106407	19965	94439	107186	55104	64863	150708	131704	55406
EIC	Negative	300.2626	5.44	unknown	336724	322810	302587	308004	493376	488936	375269	360170	340711	210389	219933	212588	277788	311257	292809	438972	239624	349225	301042
EIC	Negative	424.2436	0.85	unknown	94844	149349	97368	84753	208103	225240	128096	127835	110657	109725	133684	56458	204438	105213	120107	200912	98030	184210	77609
EIC	Negative	491.3381	3.24	unknown	291777	392944	233596	209463	610650	572800	461014	427331	784138	249865	338896	399032	411189	557609	628879	517579	422834	286304	382361
EIC	Negative	269.0877	2.27	unknown	33158	64956	0	80796	274575	381001	316866	134223	237863	98224	18081	63587	61210	64798	99545	194130	243869	119093	259632
fpHILIC	Positive	175.0221	2.48	unknown	357879	270611	251745	57399	490923	618274	324501	188993	430198	212688	0	0	353164	698082	0	0	503581	63410	0
EIC	Negative	558.4613	6.67	unknown	2062532	1886065	2013027	2140354	2698981	2732807	3134347	3426294	2148027	2616862	2112174	1978836	1938538	1955448	2354703	2547882	2030488	2261232	3530302
EIC	Negative	628.5624	6.59	unknown	169442	138633	165467	223934	251710	215411	329529	308321	555539	335377	155105	55941	206884	542306	229849	327722	277318	224667	351575
fpHILIC	Negative	304.1041	2.30	unknown	486130	461344	453857	400551	740913	716280	606711	551314	605336	413745	398824	598653	500989	380588	716390	688154	465581	700713	352032
fpHILIC	Positive	508.3765	0.58	unknown	928887	1196932	954278	1169703	1294071	1308563	1262092	917124	1262240	1055219	1318057	1215010	1283151	706131	1120238	1037529	1068480	1040042	1077527
EIC	Negative	474.2212	3.00	unknown	552608	560428	376639	523124	792703	968522	932238	698754	1007387	867261	596125	525776	624797	884287	747825	790406	751944	791781	1071917
EIC	Negative	641.5725	6.55	unknown	0	1095154	1291131	2106608	2141553	1913819	1821906	2141897	3613518	3022172	0	0	0	4588105	0	3342823	3607571	0	0
EIC	Negative	295.2643	6.60	unknown	2987501	3492194	4121720	6518134	4730565	4146649	6178406	5306557	7495468	4579134	4303901	5105181	5721077	4237567	3548520	6375736	6265473	6456755	4716108
EIC	Negative	627.5574	6.58	unknown	309166	233770	265227	488885	572264	543540	508904	732397	1379179	509596	377633	273267	377626	701705	378748	558843	530187	453959	855383
EIC	Negative	604.2968	2.20	unknown	0	0	0	43512	145191	146300	240772	0	186874	257269	0	0	151179	125469	109404	143719	185107	0	218019
EIC	Negative	269.1403	2.11	unknown	166134	174418	195229	251024	233498	322352	361755	345889	257787	302094	153676	150881	160607	160267	179325	203411	251981	374300	245242
EIC	Negative	325.1127	0.71	unknown	0	0	0	0	68980	95478	100126	215581	39119	0	0	0	0	0	70210	46879	54975	196416	48205
EIC	Negative	335.2233	1.54	unknown	0	56780	150681	0	161422	402296	162853	319210	182117	0	31518	9181	10891	52269	53834	60470	129262	468387	99268
EIC	Negative	441.2527	1.11	unknown	13932	41514	0	13635	77312	179544	75078	26298	75256	0	13096	5492	0	5321	27676	0	22261	24657	31940
EIC	Negative	494.2167	0.76	unknown	374463	293295	562325	353659	421253	400170	747151	643561	392021	522007	455344	339280	179454	183460	379550	312485	423438	205235	231100
EIC	Negative	283.1011	2.74	unknown	126697	96501	65478	92530	381987	656597	489643	217639	368018	201703	98475	93520	96670	93848	154794	283683	387021	118978	377802
fpHILIC	Positive	142.9384	4.50	unknown	249947	351580	414680	393764	378965	440944	437981	446667	417017	580184	477743	429752	415380	445692	434094	411831	392614	436897	377389
EIC	Negative	327.1632	3.41	unknown	151913	190031	171732	546611	743898	1174654	933099	638516	680985	523772	218943	219148	205356	237410	312151	581964	716120	771526	763405
EIC	Negative	308.2679	6.57	unknown	4154468	4764432	4910728	6355316	6326979	5202188	6933265	7105234	9292802	5656531	6851932	7854833	7077267	6009453	4962911	5087147	6533697	10823704	6553695
EIC	Negative	394.2685	1.71	unknown	583477	298802	412156	89670	918299	273275	926273	850986	551230	174551	42323	36681	40223	92825	95009	70084	55705	288593	77037
EIC	Negative	454.2903	1.71	unknown	844799	431736	642988	108243	1285799	395837	1322530	1218921	824577	189976	65556	64725	61094	153027	105716	100378	83421	368104	105669
fpHILIC	Positive	836.6094	0.43	unknown	2315147	2387643	1681636	2049399	3098427	2897617	2744378	2266204	2734689	1862664	2242515	2459309	2302543	2461317	2311593	2926059	2680374	2128861	2331968
fpHILIC	Positive	155.0096	2.73	unknown	624238	639966	648769	1120430	761119	926390	1153905	983410	1186315	665749	768135	633103	813175	489590	609686	1382887	675647	764022	500227
EIC	Negative	301.1804	3.58	unknown	276996	235247	632923	791845	442841	283730	690980	674380	891921	738497	340948	252579	223377	166200	334305	377963	539331	231932	156440
EIC	Negative	433.3175	4.60	unknown	407366	298837	321092	437642	477383	550790	406265	563737	635717	478055	260456	346920	385668	464856	457351	426024	278466	185641	591144
EIC	Negative	401.3024	2.67	unknown	4441873	1744123	1374558	1560241	4505651	2294653	2281833	1238885	2026616	1586168	2329544	633089	2318878	1764560	1294854	1877168	3916607	3235268	1391951
fpHILIC	Negative	117.0710	0.54	unknown	801822	266786	961311	1156444	2224838	437311	1875210	822605	2554298	149987	0	168753	172993	0	0	357443	37281	223831	0
fpHILIC	Positive	199.0890	3.71	unknown	797912	807854	878428	866408	975426	1004134	1015300	856329	893302	889172	907359	954199	832160	786218	967897	932738	852754	819563	818732
fpHILIC	Negative	133.9917	0.88	unknown	500513	1174177	1773910	2740799	2607836	2487714	2444281	2291897	3130496	3513646	2015935	3857520	1174558	2529063	712339	1750771	2111169	1483313	2626527
EIC	Negative	324.1432	3.43	unknown	95467	98589	122803	187157	158483	117073	138066	171112	207690	174896	125309	187188	160146	70247	68534	134422	128069	168892	84609
EIC	Negative	449.3279	3.34	unknown	2150111	3707054	2038289	1641088	4901364	4374010	4847195	3478544	4973871	1361162	2833382	2231553	2455168	1934439	4145682	4488349	4405302	2976159	3761762
EIC	Negative	509.7611	6.23	unknown	0	31086	59416	0	82811	112384	160704	422613	218372	154722	179028	127042	123046	80170	90756	102378	135456	196692	174351
fpHILIC	Negative	105.0442	0.96	unknown	137208	174336	312738	578133	392428	331084	295527	348384	862507	278826	647196	328613	314792	259329	363132	464983	195917	334149	176725
EIC	Negative	393.2656	1.71	unknown	2297125	1225465	1472910	314042	3410311	1135632	3626913	3363471	1800788	449125	143395	152639	125289	461394	354356	225968	220793	637327	292928
EIC	Negative	403.3059	6.51	unknown	386808	244100	431918	762600	527304	550551	475663	778013	889756	576128	164850	445663	533568	703827	609139	638085	582028	633021	738608
EIC	Negative	379.2130	0.97	unknown	74069	63029	106252	80753	413213	184772	162771	139470	236519	45920	59265	59737	70328	45108	98012	88597	70402	116759	60331
EIC	Negative	403.2129	1.74	unknown	509024	1183065	523216	709596	1192685	1127682	1311382	1335391	1044030	737818	1222572	451415	1066201	1052580	971896	1716917	1058553	301247	1241937
EIC	Negative	296.2677	6.60	unknown	652839	760288	856582	1414443	1006787	867937	1301286	1141419	1604437	967949	922938	1115241	1188529	955622	806828	1386914	1257519	1369285	1015129
EIC	Negative	399.1948	0.72	unknown	123265	229641	181235	122371	568192	334405	370123	263568	402073	241533	237449	50503	177243	185606	234541	335585	214170	216253	206248
EIC	Negative	554.3473	5.09	unknown	216230418	223861634	212470132	217061608	217357772	226467349	225479865	231195420	227725358	210009563	233478603	222809045	211511421	241897868	224297424	225621812	215991157	208907397	225053274
EIC	Negative	561.4786	6.52	unknown	581492	0	1098394	0	823768	816851	1221202	1135382	1358695	854619	0	0	1373918	0	864318	1190970	0	207870	173232
EIC	Negative	297.1532	4.41	unknown	384921	303911	536179	1005673	1230978	2452717	1330659	1135404	1422053	977553	453500	562917	439993	545260	681748	1204456	1156117	1389555	1060027
EIC	Negative	623.5739	7.08	unknown	139536	155509	83923	93575	155030	181263	210085	262940	163307	148059	101372	116318	137783	122051	98990	129954	122332	190263	184217
EIC	Negative	335.1093	0.90	unknown	68104	28854	65455	49036	90098	88208	80727	69387	87041	95072	84933	64924	50222	135311	75890	67915	49934	67041	111753
EIC	Negative	445.2965	4.16	unknown	0	0	0	17142	131717	72558	94884	112060	0	19583	16505	0	18507	0	22264	24429	0	150697	0
fpHILIC	Positive	812.6087	0.43	unknown	6676597	7422587	6673830	6807080	9181877	8533770	8878042	8577598	6567132	6851481	7067591	7186848	7027906	7784239	6698149	7475902	9197598	7359222	7241490
EIC	Negative	557.3553	2.11	unknown	116049	21265	0	0	444983	99084	649295	357433	268831	0	0	0	0	0	0	35646	0	0	0
EIC	Negative	254.9187	0.65	unknown	56308	51452	52331	56059	58306	87162	81794	78673	62220	68076	45934	36865	38670	22388	84065	53597	70066	80157	68200
fpHILIC	Negative	146.9659	1.15	unknown	632781	712277	620761	1234380	1896870	1323862	1535392	980868	1884638	1547078	760216	1321300	2473103	1073876	546362	1475826	1130458	854864	1046905
EIC	Negative	460.2767	3.01	unknown	200277	117582	130313	0	501220	76144	405378	328274	143984	40316	28073	16602	32709	37720	7768	36603	12491	94596	53045
EIC	Negative	453.2200	2.15	unknown	185631	162869	121863	200929	236580	204682	292136	393997	282175	268127	159632	307667	175392	238366	182380	292170	211126	252323	307757
fpHILIC	Negative	304.0788	1.10	unknown	318077	423397	455007	748730	1013740	727808	692968	589920	886642	765628	346739	553485	909714	482392	344595	539797	588764	425067	402846
EIC	Negative	555.3503	5.09	unknown	63954897	65395579	62909856	63802869	63942832	66412689	66001923	68432729	67563002	61834370	69050476	65784349	61931937	71658680	66610250	66327216	63713490	62225435	66594150
EIC	Negative	534.2458	1.17	unknown	80315	54924	100184	67981	81137	95664	152410	118477	81561	126474	92335	63953	30792	51830	82948	77126	86408	0	51153
EIC	Negative	297.1167	3.26	unknown	107191	91830	112941	298956	402985	583308	454097	297213	320041	266516	88154	97473	95065	107067	160292	298942	383376	503148	376727
fpHILIC	Negative	871.5917	0.43	unknown	3022847	3143618	2268277	2637315	4321573	3657168	4177496	3632230	3055796	2943209	2630848	3374534	3411505	2908204	3892134	3589734	4308514	3398078	3251964
EIC	Negative	450.3314	3.35	unknown	589914	1128023	209284	499865	1433003	1304589	1425703	1032584	1482570	419612	852767	673035	715997	585099	1233823	1354262	1337747	902812	1102514
EIC	Negative	394.2800	6.66	unknown	128467	122316	216127	265204	290182	442174	215246	362193	376502	225513	106119	89371	140572	215118	65198	96403	118465	142134	253834
fpHILIC	Negative	119.0351	1.65	unknown	1785384	10041285	3416751	15417782	9983278	12142559	11764849	4009252	15913300	5448537	14261968	12945168	0	3538171	10808453	20200188	10584473	6561121	0
EIC	Negative	377.2703	2.12	unknown	1640435	1860555	1453689	786922	7581044	1589772	10079428	6001095	4259724	1112604	502181	241673	357547	378491	466613	585767	607497	1454667	784897
EIC	Negative	393.2653	2.12	unknown	127288	250635	216844	120750	762203	183315	1729994	653260	1233234	0	20892	19076	12823	0	33491	0	0	36802	211235
EIC	Negative	450.1778	3.35	unknown	84457	50784	0	31830	158751	121493	119296	48252	126370	41469	132583	59221	51767	139331	132838	101791	76276	110486	135526
EIC	Negative	584.9784	0.48	unknown	0	0	0	0	182774	71167	126120	64746	0	0	0	0	0	2019766	72305	0	0	0	58058
EIC	Negative	474.3672	5.78	unknown	64308	0	298709	66428	413969	132820	395114	305372	334672	139355	31463	312866	160104	22670	0	90319	131308	57202	23516
EIC	Negative	438.2946	2.12	unknown	420926	315154	234824	149557	1376512	289356	1835900	1065636	768515	252930	25899	27954	68171	67157	26625	134472	101776	361920	144921

EIC	Negative	425.3637	6.58	unknown	1275780	1077950	1162607	1646768	1592219	1121332	1516492	1659036	2316612	1561913	1236298	873332	1330456	758872	965636	1303202	928093	955526	920480
EIC	Negative	461.1948	3.11	unknown	82132	63323	31860	62614	96075	67603	75032	82009	82312	71787	76983	70184	55745	86915	77360	110321	61258	52287	79840
EIC	Negative	511.3645	2.67	unknown	115475	108936	130917	105643	191136	109959	169961	187212	124201	104864	130734	70900	92013	134392	146729	141786	104552	152033	118377
fpHILIC	Negative	273.0761	1.15	unknown	174274	169661	144707	246754	408552	280902	311596	189004	364129	298929	181428	265964	610145	248045	128009	240729	262606	159234	201021
EIC	Negative	459.2725	3.01	unknown	650266	371070	625867	0	1790543	239848	1507431	1306508	430238	202440	97408	95082	109851	153032	130424	128949	120156	311510	152410
EIC	Negative	378.2739	2.27	unknown	1411847	1041275	1287752	489356	4236674	1189404	3421125	3571150	843220	260690	38679	161045	0	0	230783	0	0	487270	601562
EIC	Negative	377.2703	2.27	unknown	5516179	4147737	5126436	1971411	16282239	4607660	13720251	14385437	3349279	1117366	552622	602455	579021	252835	826064	761694	470905	1786652	2122685
EIC	Negative	393.1729	5.49	unknown	231889	174488	205036	289954	312696	405674	261556	262232	348285	244692	186213	193759	155124	196384	182655	355000	281517	342350	296667
EIC	Negative	524.3312	2.24	unknown	0	34805	0	0	117957	22211	92473	96534	47503	35567	25075	31902	15983	33208	40833	70237	26330	143032	56452
EIC	Negative	439.2975	2.27	unknown	237897	162894	264522	43224	526670	150525	428053	467921	134105	0	17449	24444	26296	7767	32304	27354	16485	59747	78746
EIC	Negative	460.2768	3.28	unknown	134705	145970	250878	104268	454605	74013	675833	382584	427502	0	0	0	0	0	0	13559	0	0	72754
EIC	Negative	437.2912	2.12	unknown	1056065	1179731	908504	480016	4772489	1017231	6576887	3617362	2729694	733001	268965	176858	245568	164325	272861	372935	382360	827751	520380
EIC	Negative	616.3425	1.06	unknown	166068	37329	79596	18996	165025	131634	167133	159047	36583	7105	0	0	0	0	0	0	0	17624	0
EIC	Negative	333.1115	0.90	unknown	193230	91536	168595	117481	257195	261076	233435	162690	285681	286304	244953	170039	153775	400451	207965	212727	151449	192773	343879
EIC	Negative	378.2738	2.12	unknown	407648	489959	384422	236256	1918773	389727	2588453	1521970	1048453	255005	0	49231	31412	0	0	0	44136	400942	46293
EIC	Negative	423.2394	0.85	unknown	281939	576948	372661	327317	689786	735557	463499	517365	350694	356247	469579	169840	608727	462495	475530	673537	354878	621024	321001
fpHILIC	Positive	244.1100	2.71	unknown	538522	658065	386262	427007	807816	695765	602195	609676	634492	339373	502102	621162	699021	761848	576926	582844	652210	582674	624372
EIC	Negative	438.2946	2.27	unknown	969613	729595	954799	381947	2980136	844348	2412219	2670397	588541	205149	118822	126385	106862	49428	176249	135594	94519	340781	368710
EIC	Negative	299.1880	3.71	unknown	233460	123363	210280	403653	276701	219218	230855	355843	518029	282135	169653	277543	263432	199236	170199	306545	222016	370054	154417
EIC	Negative	297.1532	4.30	unknown	313605	228779	274437	623303	828272	1647339	807210	692792	846585	536538	298592	370297	270852	338482	405575	899330	806993	887343	681105
fpHILIC	Negative	103.0401	0.96	unknown	34496960	29620530	43569808	90194496	65964784	36133388	53039560	47299372	133893896	51053232	98013040	55152292	61082688	37286716	51293472	79769840	28481346	51765216	26546764
EIC	Negative	437.2910	2.27	unknown	3434961	2679597	3368096	1385287	10714078	3000278	8621206	9444734	2139348	730315	341715	370831	357191	188936	507271	505663	294163	1199254	1363498
EIC	Negative	319.1908	1.26	unknown	26921	17947	36755	24887	110620	60296	92841	32194	21865	27658	18577	0	3686	7955	74393	23359	32725	26951	19394
EIC	Negative	404.2162	1.74	unknown	147512	307158	135134	177117	309723	271347	326622	344718	268317	178161	318942	119709	263306	268980	247306	458194	275119	113393	320901
EIC	Negative	459.2729	3.28	unknown	530144	534876	742371	353926	1572671	247430	2495508	1443844	1475116	0	50927	59464	62416	0	12460	102703	0	13723	258695
EIC	Negative	467.2965	5.91	unknown	415473	640173	465909	556539	667334	737122	487187	604208	638104	403246	435816	405329	368390	509862	344620	361653	471581	399252	428319
EIC	Negative	338.2361	2.15	unknown	408976	416985	327323	398993	448109	428363	493363	534452	450260	461281	430042	476644	442978	451881	437315	427947	460249	412867	453015
EIC	Negative	534.2823	4.98	unknown	545001	669549	439117	691970	633155	647106	554516	628348	850366	515335	478150	625606	432531	776808	544046	483029	593521	503270	554821
EIC	Negative	453.2864	1.71	unknown	2861503	1921251	1875061	525018	4569004	1447723	5825011	5446328	2834310	687274	185753	206436	226889	464566	444294	364728	288864	1231351	465877
fpHILIC	Negative	131.0463	2.86	unknown	2307393	1695397	1891302	0	2607461	3900726	2099285	2130163	2485000	3057351	2151260	2479317	2164535	2270467	3683748	2629267	2793797	2114628	1413011
fpHILIC	Positive	810.6008	0.43	unknown	32552266	38707156	30792644	34803348	48487276	42860000	44526092	46632872	33289980	35937588	36803888	42384604	38008792	38813240	37453892	38702408	50427196	39910196	38745068
fpHILIC	Negative	123.9888	1.15	unknown	170135	273382	242594	348172	618646	477638	513941	281603	546342	481017	315111	479394	966519	360735	215617	365814	424492	264600	372048
fpHILIC	Negative	125.0247	0.95	unknown	644226	604782	626840	1405458	583351	894460	955142	968499	1898872	1040526	785815	615600	910073	1270500	578975	1455189	833907	1037623	520087
EIC	Negative	404.9993	0.49	unknown	662453	463941	549663	541653	1185044	614439	941587	707007	524492	670427	474082	378513	568416	2942241	749014	448999	518939	510761	576994
EIC	Negative	403.1721	3.43	unknown	359920	373063	388603	782391	559127	455215	549472	657938	780756	616556	515101	742608	630302	236099	277924	568961	555794	680974	289551
EIC	Negative	486.0073	0.49	unknown	961082	715634	781326	774562	1481149	895698	1307971	1011427	722451	952818	651493	595406	766239	3202322	1053887	628452	772561	784558	812512
fpHILIC	Negative	125.0357	1.03	unknown	21238222	17519168	17204456	24143984	22964984	24525250	21252382	17454582	24363378	19776170	15267138	17223208	18518026	14248706	14482947	17009698	18626038	15996444	11709621
fpHILIC	Negative	870.5852	0.42	unknown	5918061	5961727	4638980	5354569	8967381	7562357	7635710	7083609	5002703	6544508	6236603	7507766	7103513	5851599	7479993	6342959	9141950	6179635	6493559
EIC	Negative	453.2822	5.23	unknown	18434413	23135709	16727334	23729381	25125802	24255678	21022296	20827748	29572252	15873500	18478169	18854923	16525653	23381001	16093798	18218251	20074659	15839216	18490951
EIC	Negative	375.2558	3.13	unknown	1249217	1811620	1812463	734197	7897756	872547	14623296	6436491	7305688	48484	153669	145891	176182	42650	63900	339206	50795	71236	1055100
EIC	Negative	542.2741	1.70	unknown	0	142849	60497	41576	100380	139305	199150	162876	107704	207455	97561	135810	102910	89642	99321	99750	95223	93433	114045
EIC	Negative	615.3389	1.06	unknown	467328	95751	232451	58451	452483	362097	476092	444305	103471	24220	6283	0	8022	0	0	0	0	51240	11479
fpHILIC	Positive	905.5927	0.37	unknown	1120162	1150890	1011129	1439938	1521059	1161421	1063877	1412798	1660962	1705004	1466784	1273440	1360333	1215693	1184966	1344975	1372290	1194079	1594545
fpHILIC	Positive	813.6127	0.43	unknown	1753435	2155829	1663357	1803253	2478508	2313835	2444178	2547359	1754953	2114692	2034153	1963068	2092197	2256096	1899110	1697170	2731549	2001443	1928570
EIC	Negative	334.2834	6.59	unknown	388604	440153	485167	572091	477138	646260	802930	661947	555730	628155	609031	577546	654515	552562	416676	427924	685389	719520	571107
fpHILIC	Negative	104.0435	0.96	unknown	1554028	1358201	1937374	4089907	3066058	1615396	2490736	2179955	6279208	2245743	4755354	2673963	2789012	1861166	2300777	3776796	1360800	2398625	1147310
EIC	Negative	508.3387	2.83	unknown	48471	225614	85601	0	614762	138764	561685	395333	317025	80868	197524	253136	133781	235946	275166	281464	248385	259680	233925
EIC	Negative	586.3626	1.24	unknown	0	0	56308	25621	135267	0	127975	67952	251892	0	0	6572	0	125535	0	7102	0	0	0
EIC	Negative	452.2790	5.23	unknown	75981857	97309791	68773881	99270563	105257987	101784565	87272884	86915695	123126830	66672154	76747969	78601547	68737979	97835751	66955909	76760843	84027097	65800678	76811398
EIC	Negative	366.2372	1.67	unknown	0	66420	0	0	389588	70674	261579	105581	424882	0	93864	62024	92862	113014	156673	270936	107786	219045	72347
EIC	Negative	377.2703	3.28	unknown	7614459	6995165	11222102	5018312	21621696	3001234	37756243	20070892	19302844	572514	669906	741183	632136	19449	325613	1321468	29642	571159	3317359
fpHILIC	Positive	115.0502	2.86	unknown	0	4149072	0	7445305	6229299	10959295	5673048	4431022	6696568	6530626	5479784	6022466	5426612	6499634	11706038	5985321	5530158	5969804	4041732
EIC	Negative	379.2776	2.27	unknown	199204	172041	266950	72460	625795	172775	536773	547520	117063	0	22069	20749	0	0	36163	23785	15776	62305	88592
fpHILIC	Positive	153.0657	0.95	unknown	19142370	22683466	10169691	14251565	34959404	43477056	28727220	15065889	32535756	21253164	45548820	24051086	30751856	20257442	34923076	29068982	42860560	12091295	34825956
EIC	Negative	378.2740	3.28	unknown	1903816	1798539	2895744	1256586	5399157	738271	9621417	5068428	4892240	66873	0	38687	87069	0	0	70689	0	67007	817668
EIC	Negative	347.1575	2.02	unknown	0	118052	0	0	316040	284298	306541	0	360105	0	167145	45256	149832	125118	383249	342131	143739	0	82327
EIC	Negative	413.2466	3.28	unknown	165420	166333	264861	105062	514865	68485	963944	454672	486743	0	17637	15848	0	0	0	24961	0	0	85923
fpHILIC	Negative	393.2653	0.61	unknown	3435777	918685	1639243	307246	3296323	4121560	3439892	4977369	1240322	152546	0	0	57643	66243	125780	87012	95720	533779	322018
EIC	Negative	475.2243	3.29	unknown	404967	221913	228272	202072	714772	539456	350388	257209	573367	314396	464904	334675	284030	578632	419845	472114	296146	431407	570659
EIC	Negative	365.2456	6.43	unknown	11583697	15628413	0	0	14660385	16118652	16593581	22578896	15843575	0	6651518	13549175	14814797	12316582	1509035	13228591	11149733	14425994	15910209
EIC	Negative	313.1499	2.87	unknown	0	46363	0	89946	297298	376792	398592	125495	242634	165172	76233	62049	0	61299	107445	212750	337282	168074	340105
EIC	Negative	361.1390	4.08	unknown	41785	61814	43068	314364	140611	120069	200107	191851	298149	295955	193731	247268	238901	63308	99899	197388	226241	328072	82180
EIC	Negative	319.1541	2.87	unknown	92228	107698	64175	348674	202913	130050	174939	157063	431419	186102	115701	149881	265482	71764	79863	185851	174399	277559	102476
EIC	Negative	323.2956	6.67	unknown	272117	402847	414394	805458	802631	634836	642756	803811	722806	639994	564095	894441	671531	394050	500446	705694	721746	745146	494740
EIC	Negative	445.2966	2.25	unknown	0	38739	0	0	74766	22848	92246	220427	82688	0	21721	38116	12897	74692	52278	30475	47126	144473	45639
EIC	Negative	379.2773	3.28	unknown	282014	263099	411245	0	804426	67828	1480533	777564	723472	0	0	0	0	0	0	0	0	0	82782
EIC	Negative	311.1333	3.84	unknown	123080	52925	89380	241079	293353	438506	304792	219379	231835	190638	84966	79864	74720	86709	130772	201224	256978	278812	239888
EIC	Negative	376.2596	3.14	unknown	327855	441199	470537	178728	2035368	209290	3747380	1426150	1761940	0	25812	35947	30984	0	0	34698	0	0	252737
EIC	Negative	362.2065	1.63	unknown	718986	669038	369904	0	817384	2541272	814048	757905	997074	468658	738379	218066	963293	1466183	785396	1968767	378107	1125811	736527
fpHILIC	Positive	175.0014	2.05	unknown	525428	320646	777688	125774	1637325	1414971	663614	179546	978378	164934	0	0	941879	1253739	0	0	1314772	0	0
fpHILIC	Positive	811.6016	0.43	unknown	16073088	18446922	15397663	18379466	25583320	22209210	22890606	23164900	16557123	17305352	18593472	21505066	19391664	20050956	18571804	19348506	25362252	20506448	19826742
EIC	Negative	471.2300	1.81	unknown	68511	77943	99058	0	145850	141081	143502	101658	166851	128550	149433	141381	152735	144870	145671	161563	163184	0	146619
EIC	Negative	579.3501	3.65	unknown	0	0	0	210405	969546	978287	87298	0	864913	877846	0	135128	0	758837	1199560	0	0	0	1305730
EIC	Negative	473.4013	6.79	unknown	207778	277661	146804	118536	235664	257416	261062	239487	254301	295654	276943	259125	283300	204211	228768	258095	168614	308005	237034
fpHILIC	Positive	906.5973	0.37	unknown	396229	395821	330272	547476	468973	388146	365338	454390	571451	507918	490816	346041	449259	426097	430545	393602	427661	396495	494990
EIC	Negative	322.0009	0.49	unknown	420320	333418	308928	343021	789265	415649	581173	482028	334005	457555	349825	246392	348394	2389747	495579	322218	382755	380288	382279
fpHILIC	Positive	113.0709	3.71	unknown	1499007	1359693	1445104	1442383	1750242	2100163	1727383	1516991	1487421	1444906	1253362	1557406	1273474	1022497	1530116	1639224	1178013	1585452	1209816
fpHILIC	Positive	177.1061	3.71	unknown	4206967	3777876	4373218	4373200	4883157	5948112	5218771	4265168	4910891	4325234	3651255	4345697	3557978	2929329	4548829	4671578	3488724	3662319	3415323
EIC	Negative	325.2751	6.57	unknown	850567	703234	965910	1501051	1431005	1108882	1116186	1865819	1399387	1623155	1288509	1317676	1491718	760469	1177625	1207154	1305961	1781987	867575

fpHILIC	Positive	387.1901	3.71	unknown	500686	407569	362602	499121	576472	724222	557336	456886	548805	414230	462735	460829	468963	412762	488307	486421	557597	455564	425039
fpHILIC	Negative	110.9758	0.94	unknown	2085331	1027813	951693	611976	4641911	2393880	4301840	1209754	2209361	451075	464428	646637	698788	832940	773699	1797980	786169	903697	1067197
EIC	Negative	383.2996	5.63	unknown	0	0	0	0	305751	264185	284187	0	0	0	0	0	0	121327	203788	0	256446	0	0
EIC	Negative	322.9959	0.49	unknown	399888	318852	333429	358873	758076	397409	540237	443882	364095	400347	320788	242331	331787	2381938	431451	256212	350561	323213	350581
fpHILIC	Positive	197.0892	3.07	unknown	435727	255203	258134	249408	669108	1052916	419187	270136	679542	179473	246504	216177	199453	195132	428120	562862	276867	622432	241737
EIC	Negative	446.3787	6.70	unknown	250461	141085	186697	344919	260182	254640	309264	286454	521150	220152	352103	301602	429822	265784	283064	276958	276411	294621	370493
EIC	Negative	463.2555	2.72	unknown	57406	147560	68487	247283	215772	226376	182520	159272	294029	128423	171588	262945	210760	195793	232819	216865	199113	144833	200886
EIC	Negative	425.2561	0.73	unknown	103961	178415	144064	245078	401179	283969	304778	412045	186096	150920	226007	181947	318863	118079	252304	323880	231102	414694	126633
EIC	Negative	333.2709	6.53	unknown	690982	695141	659132	1252795	779217	1169540	1203389	1951647	1284251	1053621	1471590	1175562	1405619	1192816	989070	1240180	1347199	1272220	874027
EIC	Negative	361.2022	1.63	unknown	3095854	2999375	1627736	923135	3586685	11064485	3517744	3252118	4230294	1985623	3245554	980899	4271500	6276229	3365524	8376153	1390005	5060395	3156525
EIC	Negative	535.1625	1.63	unknown	0	82263	0	0	74329	199900	69416	0	65090	0	61585	0	83638	84882	46180	100819	17503	42753	45997
fpHILIC	Positive	308.0907	3.62	unknown	200315	401079	306706	465493	882569	1654612	1137146	477071	656303	415025	582274	812300	815148	642673	528362	663650	986304	709651	783729
EIC	Negative	520.2324	0.98	unknown	161014	137870	208329	166569	170293	165958	280434	204402	231568	257251	234305	119136	70340	99420	163787	149655	180949	64325	115670
EIC	Negative	527.2379	2.14	unknown	0	251761	214650	136586	343765	287599	406576	213101	302487	0	0	0	0	0	0	36321	0	0	0
EIC	Negative	459.2735	2.12	unknown	62753	281710	155115	0	874819	224713	1051712	379122	538437	43675	72924	52183	49818	62492	73924	107600	112309	172285	109573
EIC	Negative	360.2994	6.60	unknown	478534	451998	338974	450305	539475	578145	825143	613585	583731	706336	536437	743032	596218	422929	340049	465798	692686	897046	564473
EIC	Negative	329.1521	3.08	unknown	0	0	0	0	0	415471	457964	601996	0	0	0	386903	316919	419224	570774	430633	0	0	234339
EIC	Negative	423.2394	0.73	unknown	138474	609519	212682	207153	1258778	490006	932298	718313	529080	220345	418039	81224	295764	220043	393061	753636	581980	695537	264079
EIC	Negative	338.2365	3.54	unknown	0	84878	0	0	106086	108450	0	173190	138490	174683	108610	177817	136913	57017	73594	0	0	0	65303
EIC	Negative	337.2327	3.11	unknown	0	156600	0	0	158758	154474	154293	165860	163112	198283	161631	151163	0	155363	0	0	151264	207621	0
EIC	Negative	507.3332	2.83	unknown	255263	693127	379332	108893	1921207	379882	1802562	1047124	914029	276711	598367	776526	385827	777066	850414	899107	835675	815173	660461
fpHILIC	Positive	200.0536	2.05	unknown	233476	158872	372086	57845	748842	601156	252626	132977	614352	55887	0	0	492162	535391	0	0	653954	0	0
EIC	Negative	378.2743	3.01	unknown	1707579	709068	2069289	344616	4191438	534392	3306106	3328961	784756	398713	0	81033	121698	131159	97515	93724	108954	566297	135613
EIC	Negative	454.2848	5.23	unknown	3184827	4011670	2858506	4183840	4411043	4236575	3599649	3592098	5011890	2777718	3189828	3346182	2891455	4030556	2798744	3211227	3555431	2765467	3174188
EIC	Negative	337.2328	4.99	unknown	0	270735	0	0	261531	173743	271297	413144	250294	0	273737	0	0	306044	421617	0	252523	0	251367
EIC	Negative	393.2652	1.97	unknown	0	158507	0	0	477045	615392	815029	0	327226	0	0	0	11044	29378	18603	41461	0	0	51401
EIC	Negative	454.1863	0.69	unknown	42048	46803	80263	58030	58666	60910	108471	110679	56711	60281	74482	58888	31755	21848	64339	47518	70162	40840	25907
EIC	Negative	538.2430	1.14	unknown	51353	50420	41456	31231	50416	68487	98991	88709	43716	74922	57764	83809	62984	50693	43429	56903	63724	44711	63816
EIC	Negative	377.2705	3.01	unknown	6849964	2881137	8117411	1506075	16371780	1792073	13253134	13197395	3325833	1661760	603727	606734	556875	1004339	795625	731276	703626	2424224	1038171
EIC	Negative	319.2786	4.00	unknown	323457	314222	297382	277888	337209	316912	295021	309696	294220	255893	294761	289017	305043	291022	292634	301833	339664	271976	292969
EIC	Negative	495.3111	6.17	unknown	0	376841	63947	109851	419159	355624	502268	256729	416016	124529	381055	200695	346628	301642	474380	405441	251389	323236	329529
EIC	Negative	428.3172	6.53	unknown	613845	397716	620979	675712	419442	1350908	1138891	977741	1348997	1055130	853229	764520	595914	659270	584820	1138974	713209	985080	402008
EIC	Negative	457.2581	3.14	unknown	100998	156053	137224	51352	558478	73212	1019207	344426	487751	0	10268	0	0	0	0	41861	0	0	80936
EIC	Negative	431.3018	4.91	unknown	913001	596012	561035	855603	832107	1123035	666726	1116962	1151229	892744	513045	582166	678028	844613	819160	796119	694305	629579	1033978
EIC	Negative	251.1323	3.91	unknown	2497912	973497	771710	434638	3216230	870636	2354783	599554	1189255	781525	960747	197584	1615136	823646	599534	646262	2106821	1036295	2174182
EIC	Negative	322.9959	0.55	unknown	26714	85949	70947	92836	148254	84628	144889	175028	91288	26115	68416	89810	96073	209832	80785	63867	93472	104772	24676
EIC	Negative	503.2872	4.46	unknown	491327	842254	514506	500285	846907	1202725	771624	591171	646242	469736	657597	642296	573605	764897	758666	537821	705698	493609	762268
EIC	Negative	343.1388	1.56	unknown	37872	74239	90650	0	88301	78479	103354	76717	95050	0	215970	124708	97314	297897	53703	108847	51026	0	74578
EIC	Negative	588.3332	4.33	unknown	330729	416828	323162	366144	475741	551889	571578	452667	378698	359537	413928	472717	448754	366889	456657	508891	493016	366576	440507
EIC	Negative	437.2911	3.01	unknown	4506119	1984833	5590438	1014100	11320256	1227311	8736390	8738373	2252081	1122958	424028	440607	389506	684041	553538	494570	498490	1625851	687229
EIC	Negative	438.2945	3.01	unknown	1255267	541575	1545418	292138	3159235	327775	2394370	2400798	651818	318406	118885	119891	113462	183548	171714	143333	137508	460115	208532
EIC	Negative	376.1823	2.58	unknown	0	0	0	0	505138	0	519997	0	265731	0	0	0	0	0	0	0	938714	0	0
EIC	Negative	238.9941	0.49	unknown	641722	454165	446566	472503	1134672	614208	811934	582457	435104	579662	459560	356984	484397	4518316	642564	385286	464416	473408	522733
EIC	Negative	584.3837	6.12	unknown	6109743	8079850	6309920	6804799	7444001	8279997	8044376	7521628	6787180	5821712	7244483	7641064	7330095	7232816	6887556	6592108	7558472	6290032	7353689
EIC	Negative	437.2913	3.28	unknown	54451	56567	74917	34747	160160	11054	200530	153625	90668	10151	0	0	0	0	0	0	0	13319	25994
EIC	Negative	602.3293	3.03	unknown	109999	116905	108997	152341	126739	113704	223225	128756	224672	158151	115849	98357	112564	101775	97634	122062	252205	174563	82396
EIC	Negative	485.0032	0.49	unknown	6529242	5186458	5551518	6046197	11135987	6596298	9034341	7018673	5420401	7105714	5362132	4223011	5842623	24212895	7537644	4760690	5739450	6047762	6084054
EIC	Negative	466.2939	5.91	unknown	1625335	2394845	1940009	2232153	2561360	2834383	1948290	2289270	2424908	1482911	1626885	1465290	1326325	1978845	1330836	1347065	1821298	1469103	1715826
EIC	Negative	363.3271	6.71	unknown	571819	610901	545225	1111120	866872	964431	922773	935665	947106	944659	1145765	1225819	1108081	732986	836764	852844	870949	1052194	675115
EIC	Negative	602.3469	4.33	unknown	88928706	108341821	83277891	93864055	109980137	126042556	125153093	103487644	99204798	92079987	106571907	111037316	110210115	95323913	106862189	110605837	120935029	93753580	106371504
EIC	Negative	520.2323	0.89	unknown	51810	56504	54768	60838	67547	71272	98340	67511	83687	75757	89704	64529	39539	39011	66330	77924	76608	24555	55157
fpHILIC	Positive	337.1712	4.14	unknown	376089	663941	360255	421748	450185	638802	778859	521662	643881	325892	401088	318751	599105	393458	908161	436119	400102	325600	416251
fpHILIC	Positive	837.6144	0.43	unknown	757583	784945	716329	837241	1070329	1009823	940049	893482	716508	703284	816691	768351	611192	790454	754591	875557	913953	766965	802792
EIC	Negative	244.1556	1.82	unknown	101950	52311	51196	0	159797	179104	173780	183327	486758	81600	122778	154582	186414	21982	58016	295703	123294	0	28489
EIC	Negative	605.3560	4.33	unknown	1004791	1364135	1086853	1132507	1334089	1505694	1680663	1254142	1185036	1067311	1295365	1343480	1316502	1220887	1353834	1446280	1434845	1148910	1334436
EIC	Negative	603.3501	4.33	unknown	30003381	37036988	28280383	31952122	37865913	42727569	42598816	35358818	33577161	31284645	36522020	38222573	37440713	32220138	36603307	37622156	41284908	32244421	35698693
fpHILIC	Positive	214.0585	3.71	unknown	1926220	2245317	2357659	2317321	2623219	2392504	2506686	2435656	2305909	2289964	2261133	2306886	2092908	2012668	2576066	2239723	2021012	2330013	1982378
EIC	Negative	435.2735	5.41	unknown	0	435036	0	503544	295476	273579	284416	444031	985555	307069	348649	0	417969	501577	404146	192536	475883	69203	177435
fpHILIC	Negative	227.0385	0.38	unknown	802002	2747424	1731626	1238990	3918361	2574168	5730083	2545809	2974570	1034102	3592689	4555590	4644477	3414551	1307828	4554887	3130691	2423087	2654830
EIC	Negative	522.2480	1.41	unknown	0	68049	0	0	84568	172365	98523	0	60631	0	107358	109641	31833	113296	85919	81890	128172	0	66724
EIC	Negative	339.1576	4.08	unknown	132320	114373	206178	884252	417894	321479	531923	469056	774590	608982	450754	666399	666282	144959	227753	603995	562825	877974	192897
EIC	Negative	366.2373	1.43	unknown	0	49934	0	0	93317	89039	93714	0	81776	0	81046	38015	91139	79058	111459	129411	54517	0	40530
EIC	Negative	274.1903	5.44	unknown	155964	175361	97345	296716	227787	166361	170602	177206	359145	151669	90885	114817	223933	162631	169655	242585	223087	183852	239931
EIC	Negative	379.1716	1.74	unknown	81372	115157	59234	85918	121093	91379	108887	147715	237312	110086	122153	71362	114999	112190	104350	170903	111854	0	122219
fpHILIC	Positive	295.0661	2.05	unknown	836848	67946	543369	0	2162038	1923881	349569	0	1287963	0	0	0	920371	1254100	0	0	1890807	0	0
EIC	Negative	606.2812	4.39	unknown	605474	854505	397438	631888	928723	776399	809413	665261	1025536	540562	582779	566437	637852	712271	796073	732113	850727	556611	626898
EIC	Negative	357.2588	2.48	unknown	531661	701120	433483	535525	741166	722442	728108	689902	724047	700427	700615	716602	734753	728662	743088	679305	203693	194572	714137
EIC	Negative	594.4843	6.56	unknown	736570	519401	464637	557424	720952	787461	703139	754478	1072423	549990	997403	684175	817310	740625	833964	677495	563828	781405	817500
fpHILIC	Negative	135.0214	2.95	unknown	552246	512256	200858	299417	756891	778437	674638	489043	879020	302984	354594	673223	505460	876271	968894	578829	653726	595148	759162
EIC	Negative	311.1691	5.29	unknown	2695035	16504190	17914546	35406276	40333728	88095098	42933749	29801568	38883912	23253424	19514750	20743156	16705388	20775536	21919876	40321448	34715330	35276812	38110216
EIC	Negative	364.2214	1.96	unknown	0	530148	0	0	1201250	1373336	609746	0	465381	0	647716	377455	969022	438335	925987	1016450	632063	312271	280981
EIC	Negative	308.2679	6.65	unknown	141689	203551	263356	413131	292274	355112	298692	343205	374272	269367	379205	345678	330486	283256	191193	237666	286573	316052	252107
EIC	Negative	298.2469	5.78	unknown	604980	507410	621781	550423	623468	631758	591577	616515	671729	529209	476971	648910	553749	634888	489295	788892	536748	667088	595400
EIC	Negative	439.2975	3.01	unknown	207623	99790	327752	60841	580855	66798	426024	384906	116512	59856	14791	10782	25353	36167	26733	23894	16605	81179	39843
fpHILIC	Negative	161.0818	0.97	unknown	329670	283958	363213	323346	864756	1098208	464427	577495	335469	222508	229919	380156	317158	232597	579921	414448	493842	346068	313610
EIC	Negative	405.1873	4.08	unknown	448056	436874	533382	989529	742127	587110	730749	719242	939344	941833	735306	808268	776770	242874	408725	699787	731997	902165	345064
EIC	Negative	404.0032	0.49	unknown	746753	635320	685781	664855	1316691	696430	1067248	822930	613960	825352	600278	520160	576275	3756254	919923	546946	633427	656353	733143
EIC	Negative	363.2178	1.96	unknown	689073	1838553	1004931	1195855	4639711	5023822	2392598	1100992	1815775	947725	2707398	1602491	4177272	1993778	4001941	4228144	2657353	1375103	1179115
EIC	Negative	523.3282	2.24	unknown	159346	120685	156262	125114	362561	58793	292663	366065	172841	79739	71676	52819	52913	76045	129813	186134	108137	455277	119966

EIC	Negative	370.1592	2.11	unknown	1094857	800845	805091	1285042	1387322	1150012	1327201	968951	1407524	917265	957009	1084190	1065439	438828	1200538	1647736	866756	993983	395293
fpHILIC	Positive	146.0268	1.03	unknown	375937	3517176	1696518	1752012	3441121	3640029	5478687	2009130	3845618	1804480	3695265	2928015	4753615	2127038	2548747	2770600	3638161	2176939	2364707
EIC	Negative	455.2875	5.23	unknown	396920	495894	374220	509329	588087	527267	457163	491859	686121	334144	427643	384079	361478	537291	385290	412037	506388	352559	405206
EIC	Negative	415.1975	2.89	unknown	0	0	0	0	239197	177139	0	0	463462	0	0	515905	0	0	362162	0	0	0	0
EIC	Negative	320.9970	0.49	unknown	4733616	3809739	3886146	3976500	8398567	4424558	6692016	4830681	3634116	4966277	3803217	3016742	4016251	27384709	5515885	3359795	3629625	4103557	4268437
fpHILIC	Negative	196.0542	3.11	unknown	1324192	1133757	828906	1058113	1660901	2242641	1850733	936487	1832921	919031	1487761	1609063	1291601	1268338	1485683	1669954	1536924	811216	1142051
EIC	Negative	443.2056	1.63	unknown	173618	212886	0	0	272661	830358	262480	0	322290	155698	251034	90554	337873	486786	268503	661718	115981	230385	268367
EIC	Negative	526.2844	4.37	unknown	2659742	6186625	2207172	3986108	6328279	7185615	5597193	3652738	6663082	2431384	4228424	3877369	3862809	5354701	4660582	4754657	5293862	2575758	3813282
EIC	Negative	365.2338	1.44	unknown	0	232635	0	0	404555	344561	319396	0	344240	0	238899	165527	410823	312074	473091	524164	205936	0	167764
EIC	Negative	649.0093	0.48	unknown	5036310	3894609	4044930	4473738	7987371	4592730	6907966	5215624	3961649	5179914	3800988	3139792	4326680	10611934	5069045	3598414	4052668	4562260	4395955
fpHILIC	Positive	222.0787	3.71	unknown	269544	222558	153149	518493	384790	421525	341963	209106	1097047	245936	311454	345153	504965	355051	336252	609004	438535	354059	196688
EIC	Negative	525.2818	4.37	unknown	12727054	32444282	11429691	20076195	33347350	36667705	28445022	19142019	34024119	12154491	22611950	20149233	19361871	28676850	23532987	24271693	27273141	13354632	19701748
EIC	Negative	494.9585	0.47	unknown	260116	187766	241075	183880	426209	227652	289106	234167	183435	211667	184477	124903	170477	783760	205604	111257	204196	214419	218289
EIC	Negative	535.2865	4.98	unknown	140016	167387	134712	163691	191250	171020	153419	169286	250278	127277	152624	156769	128351	190014	160582	145336	157027	140691	143413
EIC	Negative	473.2184	2.64	unknown	236393	140277	0	93442	330987	263951	288340	103005	249299	144157	213268	180012	168980	327417	194424	265871	169049	162168	334078
fpHILIC	Positive	503.2950	0.50	unknown	1691180	2222823	1267385	1238801	2458437	2520004	2303689	1647601	1978691	1013182	1734500	1904250	1452682	1975191	1960079	1254125	2382362	1153198	1756575
EIC	Negative	311.1690	5.48	unknown	11399861	9491800	10649245	17346935	20057202	34548104	19936697	14758089	18791899	11846524	10555757	10896945	8047154	10981613	11435744	18635097	16470278	16943772	18023339
EIC	Negative	568.0091	0.48	unknown	989478	768582	826579	902810	1659783	960761	1413898	1077643	760119	1046679	797758	599768	862872	2684463	1085089	609438	841197	928149	966262
EIC	Negative	500.9785	0.49	unknown	316109	225444	172454	215139	1100065	338448	617238	362728	180606	411343	209823	118454	205190	11333609	446785	152239	196978	229875	268533
EIC	Negative	542.3738	5.61	unknown	653195	875253	638694	798108	798705	867232	687307	676617	877475	639771	797080	728634	694140	681055	672752	626458	671386	603075	761452
EIC	Negative	502.2846	4.46	unknown	3534810	5995362	3367633	3804632	6266185	7477712	5877047	4072281	4985928	3241271	4788391	4701069	3625904	6028193	5147115	4057467	5431092	3485999	4917880
EIC	Negative	306.9825	0.55	unknown	0	69055	63792	95967	143220	54237	144468	184932	88179	14215	71764	73526	93276	234759	79481	58114	94852	98259	0
EIC	Negative	353.3072	6.65	unknown	180605	222534	270743	517089	368198	610868	438300	445193	399083	265539	277524	384841	438120	339870	299415	416933	369539	341039	241435
EIC	Negative	649.3909	5.89	unknown	228407	314267	190074	217601	299041	370538	303575	215780	353335	186097	304314	262971	286733	237097	290799	282154	267787	174262	310696
fpHILIC	Positive	414.1385	3.11	unknown	796088	835811	817454	955561	949469	877651	1030511	1047530	925006	858190	1036869	1024746	858508	642651	958837	860444	719800	876663	591395
fpHILIC	Positive	205.1263	2.32	unknown	24324086	27911430	31926762	50164092	28999138	36491964	37189420	31323462	61243804	28700964	38534020	38805920	42880560	16208464	20023408	51288516	25479196	33909488	22722120
EIC	Negative	393.2653	1.66	unknown	392350	253210	0	0	383160	778099	811298	234369	285406	0	24612	12757	20648	7603	35685	27587	20343	101693	31699
fpHILIC	Negative	361.2026	0.47	unknown	412751	380293	450626	181503	547290	1297929	393108	461634	469305	267517	358917	72649	427100	527246	224327	728832	212512	595105	394337
fpHILIC	Positive	432.2375	0.41	unknown	33501580	36296516	31011192	38184436	49437532	40101400	37070592	36385024	47517988	40068284	32112678	36757996	41035968	33884696	23296266	30581062	32750244	41050500	25641078
EIC	Negative	281.1738	5.18	unknown	1656099	1657657	2331547	2873234	2176252	2067704	2302244	2332535	2888522	1594748	1779625	1880368	2167425	1632844	1572558	1705674	1807066	2503997	1683306
EIC	Negative	524.2786	4.37	unknown	41230109	105453348	37090907	64944913	108409312	118480387	93356993	61960846	110825963	39833373	74947054	66365034	63293849	92057818	78634118	78773167	88513012	43910551	64451733
fpHILIC	Negative	306.0764	3.63	unknown	311887	325143	260819	422260	732629	1350575	890221	394312	413256	350090	473434	814350	646231	413465	443038	555863	833212	690719	671656
EIC	Negative	530.4295	6.63	unknown	2379366	1328149	1332752	1697122	2498690	1366549	3418928	3190487	2035619	2168784	1641394	1700907	1200198	1799772	2699064	3230665	1814166	1410078	2779795
EIC	Negative	413.2464	3.01	unknown	157013	90198	169232	34816	389729	41762	298662	275578	47042	26166	0	0	13568	15843	0	11689	0	45940	0
EIC	Negative	425.2561	1.11	unknown	207794	241030	237340	466048	877579	560578	579841	482110	316606	314401	351163	139017	541446	304537	648048	658204	419408	641309	320019
EIC	Negative	564.3668	5.84	unknown	269085	335547	250111	297203	353892	407803	331374	275419	365626	185417	335776	253362	248261	219153	274097	287523	264291	205855	258111
EIC	Negative	312.1722	5.48	unknown	2311927	1826693	2165265	3420252	3898933	6838911	3936300	2923457	3743519	2323447	2073078	2117527	1592631	2202052	2258732	3611049	3205863	3264612	3496652
EIC	Negative	356.2472	2.15	unknown	2123656	2700530	2012861	2430376	2635146	2666252	2750882	2431550	2643788	2693285	2557101	2787783	2586492	2673806	2537200	2634774	2677749	2380722	2736500
EIC	Negative	241.1808	3.87	unknown	740358	999176	1345225	7062012	2700491	1431021	2579788	2815313	6709451	2366213	1304831	2248111	2761230	485112	471378	2677645	1618882	3387283	565023
EIC	Negative	388.9840	0.55	unknown	16886	82795	63797	118535	174009	77381	167685	278943	93567	53867	90259	74979	104566	247641	107185	54284	114421	125353	49239
fpHILIC	Positive	236.0717	3.14	unknown	1037307	1127721	1149903	982140	1285725	1130042	1127469	1238332	1071328	1119095	1119947	1094063	1286208	1245829	1172961	1146423	1047461	1195596	1140772
fpHILIC	Negative	401.1996	0.35	unknown	204132	325123	358128	584487	1024468	714773	657451	531142	669602	665007	602618	987566	383339	634365	424213	441872	860037	316042	556611
EIC	Negative	426.2597	1.11	unknown	59903	60318	61216	116705	224160	139470	144769	119470	81225	82386	93735	45825	146863	85650	154832	173589	104442	156381	86563
fpHILIC	Negative	375.1144	3.12	unknown	445010	311651	230691	294158	466802	618123	486539	282270	596417	301900	405433	508577	320079	324955	411429	475722	413228	198440	314866
EIC	Negative	501.2819	4.46	unknown	18384070	32084121	17749572	20499614	33415594	38856194	31735745	21797471	26429737	17006889	24909689	23429952	19652651	30196366	27732639	21271783	29125493	18390573	26333345
EIC	Negative	313.1668	5.48	unknown	608327	511560	596685	944864	1049779	1852346	1063056	770282	1011693	637395	543630	587741	442921	622274	613934	1005034	894086	884581	957232
EIC	Negative	480.2021	0.70	unknown	77225	97547	101512	97123	90369	84053	155659	135244	114459	101074	143952	92825	61417	55297	89572	103373	106609	62555	61676
EIC	Negative	312.1722	5.29	unknown	3536023	3244222	3438527	6724480	7750916	17411070	8422861	5853145	7571106	4456317	3842915	4032095	3369460	4028550	4249613	7952513	6826987	6831392	7539144
EIC	Negative	539.3550	5.56	unknown	901968	1231214	770438	944389	1322179	1210238	1112159	1028342	1218491	790400	1188781	963936	957956	944745	1045707	1013107	1011941	777205	1084428
EIC	Negative	297.9900	1.19	unknown	401948	425537	386463	374813	411745	425742	431815	415883	402609	381991	413402	402248	391714	398758	404226	375579	408850	357415	415694
fpHILIC	Negative	297.1531	0.35	unknown	3619511	7997273	8746359	15464388	20201684	24632936	14170428	11878794	16347859	15288359	13782422	21768274	6893903	15346815	7987355	9253263	15628039	8428193	10997056
EIC	Negative	530.4300	6.70	unknown	181168	148323	166158	145614	224803	158858	239849	216654	215779	175103	198195	207321	173481	200179	242607	201471	107046	138828	199547
EIC	Negative	286.0391	1.19	unknown	66179	70678	64859	74117	69471	73607	77635	72820	76083	71378	71769	76545	72634	74282	67749	68472	77825	66017	68825
EIC	Negative	596.3582	4.30	unknown	209129	247446	184216	210340	315897	227748	353703	325582	659931	128866	234905	300434	202734	291786	300054	252396	218194	243139	214320
EIC	Negative	491.3381	3.59	unknown	566682	461379	880934	271848	1183249	232370	1114248	1595536	729862	166056	216472	386596	203125	801035	440882	375856	495229	453889	440979
EIC	Negative	582.9804	0.49	unknown	368062	235254	237359	322167	1354822	441320	689473	436364	264563	433600	265125	144919	296408	10783580	460126	183124	224123	297704	339925
EIC	Negative	567.0061	0.49	unknown	6072449	5068988	5099661	5879781	10515870	6193401	8606422	6719036	4858889	6619766	5071733	3859707	5548278	18036419	6813287	4320183	5057465	5645518	5810989
EIC	Negative	522.2481	0.88	unknown	104735	119145	93944	88452	120990	135152	189961	138301	109517	100867	93759	115255	139817	93694	97658	104813	133433	143916	119204
fpHILIC	Positive	433.2413	0.41	unknown	9354367	10007559	8334273	10189725	12990510	10650035	9842373	10018212	12629628	10574104	8805306	10037457	11064354	9109960	6293037	8142025	9075561	10607168	7284849
EIC	Negative	638.2853	3.90	unknown	116102	299311	178165	235298	364129	246770	364688	282528	681471	177176	295886	230854	436287	548634	306130	481665	462875	244369	348248
EIC	Negative	444.2102	1.63	unknown	64162	51154	0	0	76066	211226	72777	0	85226	0	68426	19991	78311	131066	66947	162637	30573	0	70822
EIC	Negative	418.9732	0.49	unknown	250436	107970	158545	173442	818137	267647	481328	282508	93897	262697	132543	76503	169601	10959640	357442	111094	164693	190391	228190
EIC	Negative	383.3549	6.82	unknown	51057	73106	79829	153874	87803	97675	120771	176655	132762	148044	76301	165892	114999	51319	60452	97502	96690	152700	69551
EIC	Negative	500.2789	4.46	unknown	65420928	112107204	63627153	72244387	118362771	136500533	110509244	76577906	92453379	59232459	89090121	81805941	69129861	105898123	96437639	74753055	103928367	66239413	92259662
EIC	Negative	593.3321	3.24	unknown	150744	243531	109864	198996	301163	315306	382990	196814	587062	91554	230076	384987	276050	270015	287111	305580	240055	130873	218645
EIC	Negative	409.2606	2.03	unknown	591585	1236927	991016	647698	2362452	1128887	1987473	940205	1220776	251228	1903830	461491	1672370	1512152	1206554	2110969	912023	1134629	1570066
fpHILIC	Negative	298.1562	0.36	unknown	722100	1443523	1614229	3087988	3662466	4039051	2690532	2104040	3127096	2953355	2051205	3823485	1226628	2900519	1473378	1598673	2921227	1537591	2070469
fpHILIC	Positive	159.0762	3.71	unknown	14728789	13231008	15282189	16182106	16520295	20779284	18996960	14989923	15648171	15202091	12843781	15318749	12212595	10022874	16131740	16318083	11796353	12405899	10940152
EIC	Negative	403.2339	2.14	unknown	98261	120329	73063	288545	172442	262112	267834	217997	227631	100855	123420	170995	194021	82860	115782	172030	122567	180194	81975
EIC	Negative	569.0059	0.49	unknown	656830	555258	579711	623699	1058631	679551	872309	738290	511576	696549	522881	479952	569074	1995162	746180	435020	534263	600610	608418
EIC	Negative	538.3518	5.56	unknown	3096374	4242957	2787426	3159033	4431594	4035022	3844907	3610207	4312346	2652843	3971786	3251125	3253655	3377631	3767500	3499396	3569815	2527057	3733121
EIC	Negative	387.1862	1.57	unknown	0	112418	0	0	640970	0	513481	103637	234025	0	53141	20919	31226	196133	417407	157263	136609	118399	132429
EIC	Negative	238.9941	0.54	unknown	84695	170709	144570	222397	329382	157546	300534	359490	175143	95821	144279	137454	220138	645658	192282	114033	176996	199152	77435
EIC	Negative	582.3779	6.12	unknown	91938046	119375740	92842022	102473337	111955483	123269573	123005508	111155002	100763231	87639778	107868119	115206601	109493638	109669883	100574674	99298656	111874494	93814481	109984379
EIC	Negative	323.1843	3.87	unknown	107044	134898	127134	400934	236456	144519	220162	200718	619108	232765	133903	222065	247803	83549	63216	271961	168178	333598	74712
fpHILIC	Positive	527.2948	0.50	unknown	1125558	1938151	795865	1104959	1958356	2125084	2016661	1208676	2761016	807587	1297573	1346129	1419168	1749485	1367134	1194381	1699337	612920	1175057
EIC	Negative	583.3810	6.12	unknown	29505465	37534253	29501575	32845944	35407981	38738250	39011329	35334774	32152440	27881507	34570195	36575279	34816510	34761472	31691227	31433286	35210290	29818921	34921042
EIC	Negative	583.3716	5.40	unknown	1815213	1784337	1750860	1739329	1799001	2040962	1907489	1934987	1729860	1671263	1814898	1896203	1745017	1758020	1761738	1714156	1885929	1664402	1822769
EIC	Negative	317.1060	1.96	unknown	195447	1367491	205050	0	4135509	4254749	2481188	232717	1196190	0	1536224	401888	363280	259070	1265469	1176744	417107	0	249777

fpHILIC	Positive	524.3714	0.55	unknown	149534192	195521696	162359776	181950000	223913008	243475072	204715216	190427968	162722656	188444192	184146528	218472624	212826496	192437216	208379152	179028048	217347568	203409312	194948544
fpHILIC	Negative	207.0137	0.49	unknown	909668	2123044	1832319	4473416	4423875	3657822	3734070	3065500	4756280	4887753	2858153	5938354	1480988	3479010	1513421	1751661	3768916	1887931	4127364
EIC	Negative	638.2853	3.73	unknown	17428	108168	54751	60426	129633	101883	113852	85841	242094	60078	118505	69618	144575	221723	104144	211121	159806	68873	109658
EIC	Negative	336.2264	1.95	unknown	0	62607	0	0	87476	135974	128264	0	288337	0	0	0	14989	16347	17572	33683	17661	0	20597
EIC	Negative	337.2051	6.42	unknown	6404301	4638684	4121401	4806718	11924909	3641673	12198550	6360689	6316986	4638766	4852518	2087454	5465609	3026057	2333023	4315623	11908274	8129798	4985256
EIC	Negative	492.3420	3.59	unknown	172055	157429	294382	98439	358789	86536	348464	504589	240743	51514	78241	128112	68111	258748	143448	124169	169990	146606	142596
EIC	Negative	335.2232	1.95	unknown	0	289635	0	0	293014	571553	585455	0	1263675	0	113029	108538	65887	135590	77527	158390	86741	0	78869
EIC	Negative	556.3526	5.09	unknown	13075369	13362885	12845200	13041709	13142451	13395571	13511564	13763957	13728679	12662217	14191777	13453088	12496695	14770850	13616654	13634195	13034472	12882681	13656200
EIC	Negative	459.2731	2.27	unknown	350311	228676	338722	188584	752225	204903	594850	1159365	193905	153750	16866	29927	33929	14927	45086	42667	29179	221579	141954
EIC	Negative	307.1561	3.00	unknown	923155	699707	838462	612009	833779	945425	926468	891744	705166	740237	209569	704001	202609	0	716918	247136	0	0	422896
EIC	Negative	458.1687	0.69	unknown	76494	55936	51986	27691	65773	80202	59887	161483	65075	85912	23257	126419	109422	46010	64427	62935	94061	79932	90825
EIC	Negative	242.1841	3.87	unknown	114516	167488	207846	1080636	393766	224329	428544	431194	1033071	361551	199692	339245	437245	77350	88308	409662	255027	542213	91007
EIC	Negative	346.1591	2.02	unknown	1182670	1286640	567859	854595	2113953	1948228	2000421	869332	2167183	510333	1486482	1740793	1447561	691865	1908395	2363239	1335157	297939	712997
fpHILIC	Positive	766.5734	0.43	unknown	774581	832976	936399	625556	1067608	949687	831582	1388805	868587	669337	791951	622468	662335	844928	796391	878471	728483	1055914	943004
fpHILIC	Positive	175.1070	3.04	unknown	1064657	541892	628366	688283	1322931	2652286	872706	593647	1398650	367179	364363	383409	403416	512928	835241	1261834	549037	1690448	575133
EIC	Negative	438.2491	2.06	unknown	72475	329956	0	0	354490	335539	357594	204461	302890	75726	288648	378870	259510	262494	303510	240534	300441	128245	325267
fpHILIC	Positive	174.1125	2.06	unknown	423826	688743	386976	512288	839043	756521	696842	587376	485555	494518	627813	656061	567324	715432	470111	495922	665165	403246	660642
EIC	Negative	543.2963	6.12	unknown	4445392	4853533	4020551	4299446	4797397	4877168	4939279	4400985	4388710	4111998	4478675	4861135	4636863	4557878	3792734	4914352	4504373	4782952	5277198
EIC	Negative	453.2821	4.98	unknown	3151125	4720736	3043823	3908579	4436783	4896485	3547418	3427585	5162858	2683319	3188957	3459998	2807245	4659546	3150931	2833784	3512875	2751539	3496518
EIC	Negative	592.3282	3.24	unknown	472819	828663	325450	592775	963107	1007551	1198268	623164	1894589	308070	722763	1279574	815355	866694	971273	949439	751112	438261	721165
fpHILIC	Negative	257.2393	0.38	unknown	3901992	6310427	7302804	9706385	6593283	6394289	7325652	7916905	10490113	5242444	5078171	6458009	6857800	4754760	4428446	6241790	5955368	6274052	5630088
EIC	Negative	354.1752	3.46	unknown	92127	135833	70547	264150	220932	98446	239260	134497	552380	168344	99541	121150	265215	62873	92386	353886	163522	132782	95009
EIC	Negative	318.1098	1.96	unknown	0	224893	0	0	675766	674661	414506	0	213790	0	265449	73680	77136	56062	202124	196318	74985	0	44860
EIC	Negative	561.4788	6.88	unknown	0	0	444602	0	773888	485384	0	577826	521452	0	0	0	0	0	0	459954	438268	0	0
EIC	Negative	595.4957	6.59	unknown	393357	403765	345392	421272	494199	381054	679385	528158	874476	405494	492037	317095	415891	492562	485110	635376	454643	348441	569648
EIC	Negative	487.0023	0.49	unknown	725809	524371	585221	629382	1141479	632451	963247	692348	521472	707811	528641	421527	507379	2402985	721448	487072	575210	569536	609682
EIC	Negative	409.2610	1.77	unknown	68393	46752	371723	113268	306554	484058	230869	673818	154275	41768	259696	226660	324130	301661	187560	324934	219391	0	215147
fpHILIC	Negative	142.0509	1.05	unknown	230363	106040	200109	1679591	481091	480207	448447	370984	1528634	536670	320735	331804	560086	96996	119141	698217	219705	270988	86719
EIC	Negative	255.0664	1.82	unknown	0	8405	14418	0	239193	0	329494	28254	207821	0	53352	59061	63776	61048	0	189151	244760	0	211983
fpHILIC	Negative	126.0391	1.03	unknown	1293124	1055995	920054	1370583	1180503	1424354	1319440	1037206	1405818	1190847	961513	992098	1165230	892272	835594	1171862	1060361	903161	706408
fpHILIC	Negative	525.2823	0.50	unknown	1319827	2901201	775166	1216648	3375705	3274367	3109190	1175318	3606417	744433	2087454	2223711	2028837	2262249	2538161	2064342	2564800	774906	1978745
EIC	Negative	423.2393	1.44	unknown	0	144648	0	0	411156	318442	272130	0	93785	0	155335	28354	267399	159257	232987	237014	182720	0	159931
EIC	Negative	379.2475	1.65	unknown	85482	66080	0	0	168916	111027	279458	0	206206	0	10592	13448	10479	18562	20448	26611	15306	0	20427
EIC	Negative	515.2436	1.03	unknown	234581	435253	220444	0	276313	420338	610128	476225	311162	331371	395335	550431	435398	421871	308947	299545	365581	337178	528431
fpHILIC	Negative	233.9844	0.98	unknown	2605982	2633926	1246123	1403593	3902001	3682260	2831574	1861567	2320615	0	0	0	32626	0	0	48991	0	0	35018
EIC	Negative	319.2273	3.12	unknown	102277	113718	564508	125345	244165	207397	436432	751359	624288	95009	129919	82415	121211	347640	175150	309093	127909	447238	121530
EIC	Negative	570.3686	5.76	unknown	2023489	3029713	1981237	2058558	2813254	3253256	2671543	2435884	2273243	1767875	2535766	2537063	2483249	2535213	2530108	2428751	2599533	1943074	2574365
EIC	Negative	413.1944	3.35	unknown	0	0	0	0	747179	779240	1388322	188946	848337	0	0	2169299	0	0	1029191	0	0	1855019	0
EIC	Negative	527.2865	4.38	unknown	356854	971311	365160	542412	968073	1056639	817708	577314	1028150	389638	671513	750085	510922	886395	698569	699133	756237	451934	519105
EIC	Negative	339.1453	1.47	unknown	0	31380	0	0	83672	77902	66390	0	76768	0	41207	32561	17680	63250	67600	63888	45758	0	86591
EIC	Negative	452.2790	4.98	unknown	13206764	19482518	12735922	16421329	18425810	20355324	15396755	14374611	21391489	11221015	13583142	14293331	11499112	19484311	12852157	11597264	14923598	11363505	14276414
EIC	Negative	536.4780	6.53	unknown	304875	414553	575926	730877	507913	366914	603693	662410	811547	680441	628983	567466	1124916	664677	405701	701630	686525	630026	418160
EIC	Negative	595.3459	3.60	unknown	145230	255309	140492	174978	287821	256444	296772	231511	563098	106992	238622	257529	229096	308554	251275	288288	239775	161163	212001
EIC	Negative	504.3067	4.93	unknown	213019	419517	273843	133259	424041	492158	360090	278267	281257	172071	317092	209588	258549	408626	340263	184767	332676	243278	342436
EIC	Negative	310.0651	1.56	unknown	64417	109019	0	0	132236	142445	132075	72594	29997	0	125200	97679	85221	88003	153609	74256	106628	0	132621
EIC	Negative	246.1137	2.13	unknown	0	0	0	0	0	0	302891	333258	24717	0	0	25554	12945	45244	0	10521	6985	0	82624
EIC	Negative	300.1606	2.09	unknown	228227	175526	211907	204604	248429	203711	227687	307129	189242	137372	123125	138692	147945	153389	145177	201672	187641	234202	132033
EIC	Negative	366.2372	1.86	unknown	251800	221089	0	0	548908	419455	318860	0	232136	0	305448	154048	424681	252149	423221	485700	237742	0	174417
fpHILIC	Positive	904.5886	0.37	unknown	2150223	2190362	1956724	2623361	2800214	2019882	2035265	2475884	2961497	3058277	2584756	2156465	2408391	2218260	2216062	2361685	2515551	2202434	2733838
EIC	Negative	336.9715	0.49	unknown	77653	49166	0	55719	375392	78319	154457	78590	39806	73745	46836	0	53795	5731844	119966	41644	55379	69251	102948
EIC	Negative	451.3345	3.34	unknown	0	186905	0	0	281297	256055	274641	0	326036	0	158577	126589	139084	100629	212084	249437	228673	140935	189814
EIC	Negative	498.2095	0.76	unknown	311749	371704	224323	168423	279712	398157	377660	683063	252910	363396	296126	322548	437211	212096	241117	261190	222195	317711	310498
EIC	Negative	321.1690	2.06	unknown	53733	292629	289214	0	78869	701915	352478	144229	0	112635	145273	134387	53855	0	123486	83893	0	0	0
EIC	Negative	524.2276	0.96	unknown	115650	173711	85675	75355	110136	156864	213628	191114	145361	185485	150208	194662	170253	119806	108326	129237	154825	96745	162990
EIC	Negative	243.1221	2.58	unknown	0	6334	0	0	23579	7076	891505	1298084	80989	0	0	44002	44507	74087	11003	26847	14165	0	143891
EIC	Negative	423.2761	1.87	unknown	916006	508399	494589	0	2601706	171248	1021692	598587	1111948	0	24672	33211	39216	0	0	72418	0	0	113972
fpHILIC	Positive	835.6038	0.43	unknown	3961000	7074057	4169033	5080627	8130569	6475577	6442692	5084275	7372683	4527092	6867563	6137223	5717088	6476354	5078442	7126273	7480097	4759896	6797752
EIC	Negative	527.3593	1.55	unknown	42134	79070	0	0	274041	63512	157204	82542	133080	72920	63767	37143	56463	120193	121123	116811	110638	106093	76773
EIC	Negative	320.9970	0.55	unknown	404285	934246	713543	1049836	1665146	725196	1489970	2151811	829681	364418	696979	811087	1155030	2492408	896706	564048	1085001	1168113	320988
EIC	Negative	231.1756	3.83	unknown	99715	119918	165694	81141	171608	121898	181534	181785	74641	72492	63251	51930	48082	50584	82965	80706	61220	160385	89792
EIC	Negative	312.2264	3.23	unknown	226420	198945	264620	339301	293364	190607	382572	298926	437270	215281	168215	236168	200083	175809	168382	271432	253422	264589	244403
EIC	Negative	242.1186	2.58	unknown	0	7217	0	0	104501	20991	5232651	7350988	395633	0	22874	223920	201143	407163	46471	78702	101536	0	840786
EIC	Negative	371.1708	0.89	unknown	62442	78722	71536	76102	76074	78346	91825	97282	70255	60600	57350	68746	73984	61551	73637	73601	79015	85293	64273
fpHILIC	Negative	584.3575	0.54	unknown	27914916	40596708	28080208	25224328	49214912	46418488	41937024	29725914	33814456	27915794	39467524	39497856	37993460	36894744	42049472	37071092	39958460	24356200	37225748
fpHILIC	Negative	197.0550	3.11	unknown	288333	290153	192055	240759	367945	528924	420792	195556	394633	215942	318278	324140	315528	275858	305117	323514	314827	178380	235084
fpHILIC	Negative	174.0556	1.70	unknown	0	630172	659094	476821	1413759	832709	1193913	518005	535872	478911	426696	517883	397128	644998	551667	616472	431724	577197	455549
fpHILIC	Negative	240.0852	1.65	unknown	0	1404038	417351	710774	0	1064592	1201698	800667	1507590	983117	1166243	997366	0	1077941	1206738	1295563	1056887	979553	0
EIC	Negative	375.2553	1.43	unknown	0	135132	0	0	127866	139901	0	0	129476	0	20708	129751	0	0	143549	0	0	0	131073
EIC	Negative	357.1354	1.65	unknown	0	105813	0	0	263478	138916	208562	0	187180	48682	90032	229608	133194	129768	53719	149570	109424	120376	155770
EIC	Negative	374.2913	3.07	unknown	111093	119087	77701	108750	147800	136751	121633	109294	119360	96450	105237	99446	112927	116042	101369	122629	119759	106186	121220
EIC	Negative	399.1096	1.96	unknown	0	97015	0	0	306361	308744	193336	0	80674	0	121300	35357	35826	14289	92336	90720	30932	0	23208
EIC	Negative	427.1571	1.92	unknown	0	92140	0	0	184884	161093	170494	0	153562	0	106874	140639	111140	109263	184237	181838	156086	0	100434
EIC	Negative	270.2160	4.75	unknown	255136	192138	366904	350929	327649	265232	298165	416692	421117	249012	204117	247469	237870	254547	201362	327085	238584	320422	247626
EIC	Negative	369.2047	4.14	unknown	70908	44355	139234	235772	157771	96220	149565	318833	207666	217308	117926	139638	140604	255559	109923	119051	191776	343825	158462
EIC	Negative	425.1647	1.41	unknown	0	32665	0	0	365591	11977	159568	0	214633	0	0	0	0	0	59796	65333	103966	0	22608
EIC	Negative	365.2339	1.85	unknown	983381	941291	402915	0	2248889	1794644	1347530	0	1035570	0	1289912	776485	2106536	1072367	1837850	2070089	1038540	0	781941
fpHILIC	Negative	308.0659	2.25	unknown	606849	803300	505700	618789	1038189	886845	962035	717554	974770	448425	593022	826843	894201	728064	864690	1066509	871452	799238	798937
EIC	Negative	258.1139	2.04	unknown	0	0	0	0	24792	0	942058	842067	0	0	0	0	0	0	0	0	0	0	171509
EIC	Negative	594.3425	3.60	unknown	504759	810824	472650	598581	924558	799945	936373	713140	1862600	322060	788160	856858	746893	948298	849788	937670	758609	447644	750736

fpHILIC	Positive	144.1016	2.10	unknown	866735	643038	778540	565869	1859341	3366187	8279463	1050442	1177826	381451	455987	520080	773139	621883	463347	598920	1077104	1275684	519865
fpHILIC	Positive	144.1016	3.00	unknown	1655641	1773822	1104174	1600947	1789661	2426044	1751238	1420567	4512995	1076385	1514348	1611948	1615705	1164045	1091435	2220345	1441565	1062186	998014
fpHILIC	Negative	325.1842	0.35	unknown	10890265	20989900	25090110	39847396	63849424	60168364	40264660	32499598	43473452	42945208	37887652	61419744	20582350	43088256	25569990	27849688	47561652	22909424	32511324
fpHILIC	Negative	253.2171	0.38	unknown	28209720	47890892	92818904	139741312	86220272	65544596	90715776	111944384	143237024	55477444	38575316	59299696	95214960	29340732	31988336	70352568	71100120	74723824	38948352
fpHILIC	Negative	326.1876	0.35	unknown	2145656	4216097	5203436	8304714	12401732	12094673	7713679	6784144	9038379	8930438	7818347	12306834	4082622	8746384	5429261	5766744	9010042	4624766	6653352
EIC	Negative	567.3864	5.89	unknown	2388359	3745179	2506985	2824560	3899423	4666247	3564306	2514849	3812336	2171152	3849702	3364189	3140128	2680357	3363116	3176394	2927955	2008803	3447187
EIC	Negative	320.2311	3.11	unknown	20968	13642	135334	19446	50433	53541	89260	177632	128236	20232	32443	13347	34815	81077	45932	71938	33292	109995	31460
EIC	Negative	600.2958	2.20	unknown	117207	148005	64149	172265	187209	180523	309650	160862	262649	390327	249294	148447	76148	128928	158997	161942	208624	179581	153049
fpHILIC	Positive	834.5991	0.43	unknown	7814408	14662769	7801957	9153496	15125794	13061650	11838332	10009036	14690269	8942419	12756032	11330644	11450972	12676594	10793742	15092683	15276289	9668831	12391036
EIC	Negative	527.4474	6.98	unknown	2365644	1813124	605879	808235	958765	4391109	1768550	3913740	1877842	1071612	993140	1812370	948627	1352868	644623	1138913	959692	670233	1602740
EIC	Negative	281.0218	1.19	unknown	54882	47329	43186	48495	64508	61416	68573	44007	50177	51898	57537	53621	52442	119421	58814	46572	51197	42502	60031
EIC	Negative	364.2216	1.89	unknown	0	81539	0	0	142909	438405	140548	0	145951	0	140208	158605	163416	96048	156612	261225	130606	0	80706
EIC	Negative	228.0412	1.72	unknown	8749	87054	0	0	101681	72268	157919	36419	98345	12457	119188	124008	135939	101427	46976	142840	69501	0	64937
fpHILIC	Positive	417.2171	0.41	unknown	656116	639080	527841	642451	1085620	901873	871334	850683	2067090	672943	728317	710917	836610	777015	393495	642966	618158	781105	510426
fpHILIC	Negative	311.1685	0.35	unknown	11229351	21398570	23812776	41248672	60364592	61647952	39688244	33075618	42592540	42220156	37972248	61027464	19841994	42212736	24205786	25884614	50726496	23443148	31055502
EIC	Negative	429.3014	3.73	unknown	0	0	0	0	0	0	0	344517	334375	423492	0	127323	0	0	0	0	0	0	213377
EIC	Negative	361.2024	5.18	unknown	0	0	0	0	0	1281453	1321320	0	0	0	0	0	1321367	0	1398971	0	0	0	0
EIC	Negative	525.4891	6.58	unknown	131776	166619	47952	171627	162610	126387	263032	192500	449189	135711	185584	107703	181853	310044	110093	268720	240465	163934	295374
EIC	Negative	528.4509	6.98	unknown	902692	699613	236326	305176	354901	1685273	676126	1515983	721573	399283	381102	679172	360209	513800	245452	439871	380234	251464	628872
EIC	Negative	501.2819	1.98	unknown	0	0	0	0	0	311374	291745	0	0	0	374826	0	575855	0	0	433596	0	0	0
EIC	Negative	375.2483	3.14	unknown	0	0	0	0	0	27710	0	5579766	6583254	0	37643	143205	162194	36795	51636	58918	81060	75116	0
EIC	Negative	527.2869	4.54	unknown	924317	1717597	795017	1300530	1703015	1794756	1748650	1111631	1803140	832348	1181527	1389857	1206116	1490787	1445704	1014042	1376220	1011481	1294984
EIC	Negative	390.2942	6.43	unknown	8665932	7770323	7556979	10608422	10163407	10703957	8726439	8342089	10915191	10362621	6926441	7611077	8023737	10125748	9267149	8439479	7824517	7117495	12353582
EIC	Negative	365.2337	5.83	unknown	0	0	0	0	0	754170	0	0	643816	0	0	0	0	0	0	279635	0	342821	0
EIC	Negative	438.2983	5.76	unknown	668850	979506	660183	731288	997790	1148421	721194	727791	1088617	607189	770121	946092	695714	964359	773275	787286	859135	671573	897081
EIC	Negative	295.1544	3.00	unknown	295365	291820	276697	293367	306286	288855	345261	338011	286433	279962	264210	272960	293199	252098	261001	288572	308244	331647	308761
EIC	Negative	258.1137	2.12	unknown	0	0	0	0	9383	0	669527	1177060	46239	0	0	24710	33126	42594	5143	6070	7505	0	125745
fpHILIC	Negative	103.0038	3.85	unknown	862316	774458	644634	1490553	895848	1169112	2547152	668436	2199545	730136	653999	772804	723961	676704	607569	1561161	605315	829671	802938
EIC	Negative	265.0484	1.19	unknown	3583527	3858131	3294645	3175797	3835220	4456336	4525391	3353399	3845506	3705512	4196476	4197813	3738877	4150466	4057655	3642225	4222870	2893797	4326639
EIC	Negative	256.1927	2.20	unknown	186771	207680	74062	234585	250479	264415	236404	176425	244856	278333	198339	182468	260827	183439	180471	301297	196357	258279	224237
EIC	Negative	372.3122	6.35	unknown	1086028	1339834	743364	740661	1194010	1143056	1131656	1316737	933699	593400	897331	769754	834176	859076	903933	672104	931591	860178	1156907
EIC	Negative	345.1560	2.02	unknown	6496006	7221511	3345364	3882965	11763705	11008357	11093533	3919908	11773897	3180342	8422583	9663647	7445208	4322179	10587979	12852656	7544275	2007953	3953908
EIC	Negative	526.2845	4.54	unknown	6397483	12192401	5487845	7813658	12062564	11958100	10791407	7974443	12295288	5940202	7923728	8224097	8607026	10342967	8694065	7286364	9612543	6287783	8859122
EIC	Negative	368.1431	1.78	unknown	62074	44474	64995	0	64600	67853	63713	63235	57559	81080	50770	63681	53967	28218	63915	78513	53630	0	34802
EIC	Negative	243.1965	3.43	unknown	1155120	794911	2597578	2403120	2481387	1628154	2064941	3369180	2448902	1550597	880080	1319830	1105351	673591	322862	884523	1109173	1737549	818288
EIC	Negative	356.2518	2.35	unknown	796470	1062847	930588	921887	1006651	1038126	1153077	977998	1029521	996668	1058028	1008002	1055780	856213	850739	1033101	1017018	881748	1157726
EIC	Negative	266.0518	1.19	unknown	568327	593013	506152	480953	589196	700595	703868	509792	608691	569076	657572	640904	567868	636669	613338	568545	632743	447709	660028
EIC	Negative	423.2762	1.97	unknown	0	112395	0	0	248941	237281	383723	0	308452	0	0	0	0	0	0	0	0	0	12050
fpHILIC	Positive	526.2928	0.50	unknown	3102482	6834279	2165262	3558148	6593205	6604423	6585852	3663643	8652269	2602180	4736509	4906140	4672911	5893375	4285418	4623471	6200251	2094555	4045648
EIC	Negative	569.3659	5.76	unknown	9812948	14501108	9616552	10223184	13807365	16068931	12938095	11848191	11088371	8656153	12405393	12913050	12040072	12767551	12182862	11660641	12764583	9269263	12573793
EIC	Negative	369.1558	2.11	unknown	5309901	3936425	3794018	6435860	6848214	5661137	6586625	4497074	6824088	4326506	4755458	5379787	5191014	2178799	6014019	7936649	4385874	4351308	1988238
EIC	Negative	618.3423	3.74	unknown	129015	190479	126263	132636	187705	219163	210576	161655	243823	94502	178177	247234	207030	211102	178090	219011	195695	135494	185961
fpHILIC	Negative	255.0630	0.43	unknown	0	0	0	0	0	833109	1128043	0	0	514015	41287	903023	61570	69142	0	62197	0	749947	107696
EIC	Negative	498.2628	3.82	unknown	0	1909982	0	847227	1662632	1352867	1103474	806850	3367152	539669	875380	505095	822072	2379016	1036989	1153171	1354630	656355	864475
EIC	Negative	648.3869	5.89	unknown	675287	987542	689528	784845	1006947	1194639	973240	672504	1187229	649675	994475	899764	921627	758742	934032	932357	849830	578864	1035393
EIC	Negative	475.2244	3.00	unknown	132514	113864	39143	102659	211937	185145	266600	90975	115215	156913	133993	128923	121555	188409	153449	159377	151048	108959	219434
EIC	Negative	616.3027	5.23	unknown	0	0	0	0	0	169980	0	0	235212	274333	205227	0	0	246844	0	0	0	307194	0
EIC	Negative	571.3706	5.76	unknown	326973	430978	298649	318642	409146	523880	415445	328387	347923	260247	395770	405344	373829	381839	422533	391995	412420	309845	374867
fpHILIC	Negative	279.1264	0.97	unknown	1094199	487849	506680	511142	1106946	1511628	957208	703738	636523	708344	857668	824639	798438	1009413	796063	859226	1016535	994781	579212
EIC	Negative	516.3233	5.61	unknown	326761	424061	331682	413753	386730	431661	312996	345404	471302	338379	394358	372194	367759	365119	326204	342295	381283	316333	409115
EIC	Negative	409.0670	1.19	unknown	82134	106137	81398	70841	94115	125719	118997	78896	104801	92300	115641	114259	98268	104740	113306	92664	117891	59798	119587
EIC	Negative	394.2684	1.66	unknown	72422	70774	0	0	97437	209977	203194	0	88144	0	0	7906	0	0	0	9713	7181	0	0
EIC	Negative	244.1999	3.43	unknown	178272	130087	419856	363431	394988	257945	315694	559532	394061	249102	130047	219180	171111	115061	51260	140643	158063	269907	131050
fpHILIC	Positive	525.3741	0.55	unknown	43759944	57361644	48748768	53957612	65948476	71287248	58138580	56238024	46118308	54015728	52120552	64567848	61463828	57526676	61484408	52243608	61208332	57120020	57742164
EIC	Negative	287.1972	0.81	unknown	95419	104747	77477	93131	89384	108189	144162	118519	103792	74262	99424	121269	100244	72150	85444	83635	107280	107768	96437
EIC	Negative	595.5680	6.72	unknown	197051	362899	255059	375206	467013	370679	456819	363720	281189	277418	335772	339804	306818	331613	213411	211049	270790	280743	296484
EIC	Negative	473.2585	2.27	unknown	245526	107290	233609	93272	405051	107302	304933	592404	91614	0	7957	15933	9877	0	26404	23561	0	114361	63611
EIC	Negative	326.2713	5.83	unknown	0	0	0	0	318972	212732	0	0	0	0	0	270342	0	0	0	184494	0	0	0
EIC	Negative	648.3878	5.55	unknown	111071	264503	145850	196546	250236	344157	235286	154095	239565	89654	206116	119269	133394	150209	180644	204308	201621	80935	156463
EIC	Negative	509.3493	3.58	unknown	237061	566434	251671	196763	577175	577781	460036	216207	725191	176044	448868	317639	440203	500591	738713	581099	388158	223216	362716
fpHILIC	Negative	303.1269	0.95	unknown	2758113	2224603	2891657	2379672	3042169	2173108	2953394	1933185	2396298	0	0	1875653	0	2158711	0	3285827	0	0	0
EIC	Negative	326.1588	4.08	unknown	134153	116876	153214	242671	201791	156942	213272	190554	274160	251323	201498	253752	236544	0	129964	209932	208588	234782	0
EIC	Negative	298.2470	4.34	unknown	1665284	1843387	1576102	1572656	1686721	1791924	1789883	1716871	1693413	1720647	1800372	1616320	1748252	1611498	1565162	1605498	1665042	1781954	1709510
fpHILIC	Positive	150.1124	1.28	unknown	1234060	1072690	1185899	996177	1513847	5063113	1583027	1070567	1841991	1059052	1529656	2596382	1740684	992355	1352499	1062272	1606676	1402767	1119160
EIC	Negative	493.1842	2.72	unknown	267992	299980	326231	393199	342654	359037	393726	329375	362269	309352	407329	335494	400475	392588	413756	338555	311512	287275	380777
EIC	Negative	590.5243	6.57	unknown	1077776	857362	809124	1088994	973166	759144	1219936	1584138	1478699	707702	1489820	1177870	1106270	974903	976771	855444	1073722	1873970	712155
EIC	Negative	246.1169	1.93	unknown	0	102365	0	0	109377	266875	127327	0	41819	0	73338	65269	43356	17033	84435	97639	20971	0	16054
EIC	Negative	568.3626	5.76	unknown	31915865	48247738	31162052	33521056	44890806	52640824	42458647	38416559	36112871	28504687	40616660	41615233	39165900	41190198	40009117	38143227	42115085	30259853	41269012
EIC	Negative	496.3732	3.37	unknown	170527	128018	111044	189906	180560	169267	120652	254839	184776	65275	139537	142393	163359	142761	245246	187101	81266	176735	108488
EIC	Negative	310.2108	3.61	unknown	195069	163464	222851	328908	233071	161543	272544	283678	313973	173911	131875	192734	157966	147171	151447	183847	204350	214352	181490
EIC	Negative	529.4551	6.98	unknown	203503	161931	67325	85231	82481	472237	151617	339658	164951	88655	116937	146934	93257	132229	65921	133244	100335	79719	153490
EIC	Negative	313.1435	3.38	unknown	0	0	0	234805	117279	67697	113096	98964	143336	105217	38710	89952	75809	15397	12303	0	88432	138342	0
EIC	Negative	432.2217	5.66	unknown	629810	2282612	475510	1651174	2458518	1389406	2134778	1108616	3124719	982411	903830	978089	2162068	1808346	1107688	2321364	1514036	1273682	687250
EIC	Negative	495.2211	0.76	unknown	110890	100068	137551	114948	111944	138680	184796	170979	94552	128303	116235	102339	55738	52133	122786	100595	103801	70622	63958
EIC	Negative	481.2959	2.62	unknown	0	0	0	0	115349	91874	103729	0	468997	0	89331	72836	97143	109270	199142	85225	275713	0	147700
fpHILIC	Positive	281.1277	1.80	unknown	285678	344706	428096	573646	604821	780251	593889	497125	535138	531800	755789	611346	830171	509471	485228	564549	605980	474667	574086
EIC	Negative	407.0066	0.57	unknown	0	9593	77745	135243	53312	14347	66129	55354	294447	102714	61742	31938	83871	0	20863	101736	25186	62223	0
EIC	Negative	403.0000	0.55	unknown	344987	1018797	645951	1091908	1670396	715841	1425135	2312960	782202	301658	782702	864642	1198856	2043042	882673	562488	1055340	1185129	257709
EIC	Negative	251.1269	2.09	unknown	0	84601	0	23764	283949	122497	85191	110571	198788	55225	80919	223516	102056	47513	98124	290007	151727	63364	124968

EIC	Negative	225.0917	2.86	unknown	115268	94754	193237	120642	305785	247085	115645	216499	299071	207395	148369	373620	348488	138381	93336	155159	304695	328480	229041
fpHILIC	Negative	303.0595	2.48	unknown	827308	166733	300147	39304	606817	704282	417082	149647	508485	154654	0	0	431560	483236	0	0	691262	0	0
fpHILIC	Positive	160.0794	3.71	unknown	1042268	868677	978052	1010501	1137205	1420102	1295650	965511	1012902	1019647	903561	1147225	852348	712882	960096	996772	774311	929765	755457
EIC	Negative	309.1711	5.20	unknown	4101150	0	2469894	0	4134445	0	3724174	2356477	0	2734720	0	2847721	0	0	0	0	4159772	0	2386656
fpHILIC	Negative	165.0403	2.96	unknown	4728657	3402449	2660990	3417830	4638569	5473687	5520055	3135269	4958819	3269227	3920512	4228516	3688422	3907628	4488581	5094485	4967342	3527293	3895335
EIC	Negative	226.1446	2.00	unknown	0	139801	0	0	273185	353201	248121	0	153058	0	263279	528191	254069	134141	282936	260591	286521	0	136862
EIC	Negative	441.2131	1.81	unknown	142101	141351	86162	0	139528	205325	171135	96775	67805	0	114298	104398	124288	80372	116618	76742	124488	0	100894
EIC	Negative	363.2553	1.97	unknown	0	184569	0	0	310661	508545	1123480	0	730333	0	17964	29603	0	50234	0	0	0	0	68596
EIC	Negative	419.1912	3.74	unknown	22486	105597	200947	50094	121243	29901	335545	264004	61849	69579	207465	33415	13727	48140	30472	0	14102	16731	0
EIC	Negative	227.0380	1.71	unknown	242685	644158	187933	139524	799317	546878	1100871	281652	691987	215572	868475	946158	1066336	738924	287075	1084626	546035	69116	498870
EIC	Negative	541.3712	5.61	unknown	3205364	4652329	3247572	3861459	3950772	4284620	3658232	3563776	4386187	3215523	3974259	3600201	3652832	3540045	3633517	3377516	3509930	3178143	4019765
fpHILIC	Negative	213.9981	0.97	unknown	17174702	14047676	10495868	4877611	19738434	28264534	14425637	10198725	9830976	117433	113821	110357	176781	96121	103723	143722	104286	77363	84603
EIC	Negative	343.1401	1.49	unknown	0	73422	0	0	83749	99798	126049	0	140578	0	406693	237921	110118	501235	54801	155760	68229	0	128180
EIC	Negative	319.1903	3.31	unknown	532895	149770	176877	148910	363299	417649	743946	833927	107402	26956	24623	28489	27056	25857	22007	18492	18319	32796	13123
EIC	Negative	585.3867	6.12	unknown	928510	1246821	937027	1123830	1156754	1262724	1262327	1224389	1058114	947508	1138146	1179346	1146360	1141956	1026838	1048469	1203270	1000933	1119362
EIC	Negative	509.3503	3.28	unknown	113854	307161	137901	70718	326507	281103	236477	136220	422738	101596	257004	177058	233822	303939	487417	322822	247678	134327	201663
EIC	Negative	288.1632	1.35	unknown	26434	192151	0	0	272101	260324	239492	0	343564	0	264410	210310	290318	82164	266008	398367	245991	0	107069
fpHILIC	Negative	894.5845	0.42	unknown	1337398	2234315	993378	1348792	2639508	2145219	1991389	1296658	2204014	1447962	1832254	1907673	2059288	1924032	1927664	2133800	2307001	1302937	1859114
EIC	Negative	271.2281	4.51	unknown	1104974	451073	1114827	1083510	1271445	1191294	868727	1447736	1184881	701364	510637	604003	583785	738941	405945	683888	662999	1060890	647571
EIC	Negative	338.2366	3.12	unknown	0	218223	0	0	220387	210924	0	210981	199378	230470	198348	221727	203384	217969	226893	223717	12591	0	219359
EIC	Negative	473.2830	3.86	unknown	0	0	0	0	0	2659583	0	0	1392527	107676	486465	308789	107517	0	99792	180964	78011	270913	783196
fpHILIC	Negative	158.1183	2.32	unknown	286711	285569	298301	420092	269941	413809	402074	293891	549864	283804	378065	365272	396853	144854	247743	489880	283422	288281	208793
fpHILIC	Negative	192.0667	0.89	unknown	7791774	9165460	3883374	2927496	14074583	25208370	14703781	4936683	5796495	913443	1919042	2460571	1362037	1978947	1978198	4696941	1855170	1049104	1750115
EIC	Negative	566.3829	5.89	unknown	7719086	11993544	8089133	8926926	12290574	14779503	11310417	7560335	12131741	6975717	12268668	10475917	9796877	8405901	10656633	10026082	9227258	6253226	10845128
fpHILIC	Negative	149.0457	2.80	unknown	1979957	2093331	1535408	1636512	2289356	3917101	3546074	1579202	2284234	2233968	3200617	3522280	3050156	3031222	2233513	2743113	3161558	2084868	2879950
EIC	Negative	359.1840	3.65	unknown	7483842	7808994	7367314	7225555	7627823	7500806	7887815	8043012	8170018	7471960	7311819	7728826	7067062	7353540	7264443	7348474	6763233	7490357	8055134
EIC	Negative	299.1877	1.98	unknown	0	112010	0	0	103487	147525	117639	0	167879	0	201794	139835	175549	141758	117735	217041	108730	0	88152
EIC	Negative	438.2941	5.75	unknown	500275	606314	455295	714013	903103	1058743	684122	479418	1085711	496670	613059	434406	377333	925245	432755	458303	793846	594301	533126
fpHILIC	Negative	312.1721	0.35	unknown	2281535	4234609	4655136	9366607	12159847	12069089	7875151	6314243	8449711	8280721	7494640	12191054	4296798	8588574	4862063	5064599	9483851	4732670	6526260
EIC	Negative	378.9949	1.19	unknown	130664	135767	136805	123642	129547	140590	133111	142050	137274	125653	131831	133776	136879	124572	132952	126531	133480	120656	131016
fpHILIC	Negative	387.1850	0.35	unknown	372011	657194	651445	1095533	1960520	1634860	1140849	921635	1087951	1309775	1075742	1734168	601649	1333536	746897	833092	1337760	669241	1080727
EIC	Negative	491.2430	1.82	unknown	480736	124289	239636	0	519470	979736	648599	40183	136045	0	0	7639	9706	18801	0	0	22736	0	27988
EIC	Negative	453.2822	1.71	unknown	238420	1530183	0	421067	0	0	4720206	4284700	2316549	556061	0	163063	177205	6532	360559	297240	238478	890196	369378
EIC	Negative	257.2271	6.24	unknown	273771	816985	399064	1007818	1492119	494746	1036981	496335	2021082	227044	427481	80740	189831	535623	456567	1133918	1150999	442967	247544
EIC	Negative	582.4618	6.41	unknown	641640	937272	1694677	2613081	1647340	973390	1461593	1761603	3730133	921099	790391	1523297	1321120	956041	615552	1161089	1285896	1409450	652817
fpHILIC	Negative	183.0121	0.35	unknown	131401	335312	347940	510565	828947	774615	549962	449985	544748	657077	632822	836362	300975	550472	364365	359857	668110	298273	416249
EIC	Negative	241.1078	0.82	unknown	250302	299677	259488	238540	259618	263436	323876	338475	273830	217892	222070	231530	250877	226594	302674	275811	259348	263149	255681
fpHILIC	Positive	161.0915	2.96	unknown	977704	1372089	675451	745728	1262980	1654675	1489281	738516	1557414	524384	563382	714827	658040	886014	861733	1067079	929798	761464	965024
EIC	Negative	367.2393	6.53	unknown	866606	1079107	786447	826211	721420	1800836	1416084	886088	928673	1281299	1256372	884258	855453	563803	578285	1019459	546476	577539	503456
fpHILIC	Positive	138.0489	2.07	unknown	1085639	79631	333825	0	1027518	734290	283241	35271	704331	79629	0	0	489090	707594	0	0	1081396	0	0
EIC	Negative	321.0442	1.43	unknown	0	71697	0	0	78176	275412	84579	0	46679	0	81112	94401	93908	92857	116817	67182	73680	0	76920
fpHILIC	Positive	136.0421	2.47	unknown	346664	280029	241394	377704	546898	290597	416203	268735	920067	317349	393191	341063	462845	230160	306379	622317	348261	402968	211648
fpHILIC	Positive	131.0813	3.72	unknown	553018	340886	441600	460170	518276	675586	470209	506841	496798	421016	373184	433354	425261	361064	559320	515488	433808	619788	455581
EIC	Negative	337.1415	3.43	unknown	135953	116902	193843	927629	358977	276391	402901	519954	724035	571237	341868	691598	559948	155581	175425	497173	457679	775679	184263
EIC	Negative	495.3696	2.83	unknown	261506	420585	362383	214760	475245	256072	510777	445810	400523	262273	329141	339895	234319	611530	479201	357546	428145	401794	364375
EIC	Negative	347.0515	1.19	unknown	537875	596525	521819	485583	593043	689911	677982	498308	589623	558370	631576	621741	591389	621451	619264	575754	634982	420931	648071
EIC	Negative	495.3696	3.37	unknown	510003	367812	378337	623641	544490	510954	417157	788744	572764	277106	459112	471491	499840	382214	760137	589491	295298	495411	377046
EIC	Negative	461.2908	2.47	unknown	84808	28914	125305	35816	192036	29068	66403	355282	133667	72104	38377	34621	29763	106129	114414	77400	67783	391040	91890
EIC	Negative	462.1128	1.48	unknown	0	56614	0	0	139657	335218	1060641	0	70346	0	181260	102035	81261	77483	23870	99356	131444	0	36812
EIC	Negative	393.2653	2.62	unknown	256145	34587	0	0	127906	0	331355	278955	218097	0	0	0	11036	14492	0	11918	22731	0	29427
EIC	Negative	428.1831	1.54	unknown	0	113809	0	0	1159466	66125	364927	0	333571	0	47358	0	43372	172709	132063	126584	149807	0	59724
EIC	Negative	528.3069	5.23	unknown	128869	198849	343615	79854	99392	459354	356500	362823	65765	256279	271421	412722	165270	42254	306000	71065	243466	277191	265801
EIC	Negative	525.2818	4.54	unknown	33367846	65727904	28184129	42365915	62599858	61076610	56180010	42264658	63911806	29322094	41212946	41700780	44185928	50892808	46346878	38043631	49356154	33494103	46036016
fpHILIC	Positive	191.1390	4.76	unknown	1485981	1404213	742296	676400	1223776	2261401	1518362	866324	1818006	717563	1028211	1175330	1178028	1579578	1488920	1659751	1025731	534992	1330397
EIC	Negative	427.1799	1.54	unknown	0	464319	0	0	4798710	3111230	1543938	0	1323812	0	135838	39394	158363	636376	543731	489487	628643	0	234467
EIC	Negative	461.1084	1.48	unknown	202228	238657	838805	0	557333	1408394	4377177	0	200219	0	775757	434472	263313	292203	69603	440348	522151	727275	162331
EIC	Negative	560.4763	6.70	unknown	1290928	999960	1082560	1317903	1810733	1273086	1687754	1502600	1180259	999068	1483745	1277666	1495429	1796503	2207745	1108492	859249	1589290	1565201
EIC	Negative	363.1213	0.69	unknown	77842	69325	56084	56324	67197	71498	74352	88104	69256	66422	66831	75406	65580	72866	74739	72317	75443	67049	69593
fpHILIC	Negative	313.1639	0.35	unknown	454163	839684	973250	1588646	2096438	2157019	1301091	1120690	1923806	1513266	1431949	2259962	803642	1520817	960162	912532	1783186	858924	1255060
EIC	Negative	404.0034	0.55	unknown	21018	123619	67447	123534	184209	73433	155324	272527	90465	19652	92971	102447	140083	243654	78275	64067	110829	119622	25161
EIC	Negative	583.3724	5.17	unknown	812502	981394	865659	815295	956221	1153246	1177691	994337	857949	721991	1045904	1050732	1028383	1056889	1067893	964954	1055934	891925	1087785
fpHILIC	Positive	119.0859	0.41	unknown	676965	671265	593575	715894	948599	1011597	684500	800751	1822344	710256	697153	770426	893418	823888	414011	577989	746010	805779	534884
EIC	Negative	348.0548	1.19	unknown	100361	102750	90115	85437	102457	121969	125615	93532	112381	98238	119332	110832	105615	115488	112726	103202	111767	78890	117017
fpHILIC	Positive	793.5942	0.43	unknown	334467	393230	387925	444633	408253	404927	339218	435657	502569	401288	334090	335463	307942	354389	271557	431773	339924	339739	400308
EIC	Negative	328.1570	1.67	unknown	77851	212755	69976	458055	309166	356237	359762	108820	622468	293953	383101	317397	501740	94572	317395	508312	353998	352266	157453
fpHILIC	Negative	193.0703	0.89	unknown	780749	974925	415890	314536	1484049	2816992	1463365	520272	589041	112789	186035	285867	149357	220927	208309	499165	193507	118710	187315
fpHILIC	Negative	254.2207	0.38	unknown	5103215	8047665	17398340	23115460	14314234	10061844	15228707	19445210	24899282	9802944	7018013	9921626	16740341	5002529	5327146	11412320	11811810	12545309	6663076
fpHILIC	Positive	167.0925	1.00	unknown	389063	186019	318093	265622	308014	449127	291918	428835	527107	207913	338796	342296	292251	412412	269238	263666	703864	282612	366552
EIC	Negative	363.2553	2.20	unknown	177537	60492	36868	0	185389	137065	336975	23859	352563	0	0	0	0	0	0	0	0	0	22670
fpHILIC	Positive	147.0466	2.73	unknown	442038	212731	207928	458706	367382	280032	292707	249944	551930	294473	200935	166092	249625	100157	108420	356654	201578	202654	105976
EIC	Negative	436.2836	5.74	unknown	4542647	5792397	4323202	5356007	5542866	6814347	4361465	4981348	5348878	3875993	4590543	4332097	4258796	5185915	4097086	4089110	5120655	4335176	5279373
fpHILIC	Positive	134.0599	0.97	unknown	9247834	8005093	3942168	3368837	11701146	14620760	8634849	4625756	6323130	117743	62594	37965	121205	42497	55865	120452	0	41461	46781
EIC	Negative	271.2281	4.39	unknown	2035960	1702109	6968843	6297930	5467908	3111730	4718559	9005470	7643968	3587992	1779899	2417188	3340150	1822823	971515	2807518	3146875	5321403	1881840
fpHILIC	Positive	705.5813	0.48	unknown	487137	444620	442766	492724	650021	404873	419707	470744	604897	506889	482304	391199	438301	573841	502463	465910	438115	419181	428503
EIC	Negative	578.3468	4.02	unknown	259186	465400	600669	3408660	2944031	653747	3793449	696184	2858452	2347593	3633645	952582	791446	779922	3237993	906891	957389	867802	754779
EIC	Negative	548.2013	1.12	unknown	44103	17527	92642	61368	35711	52237	88809	88860	70938	115899	80182	52100	18603	50365	53038	53526	66614	38464	42397
fpHILIC	Positive	76.0762	2.09	unknown	19182790	21395458	18220426	8784554	23873840	86186680	46472112	6191088	20249666	6719033	44250632	27729960	65554360	951481	12919300	15916816	12236522	6267220	25312972
EIC	Negative	514.2410	1.03	unknown	1101276	1833963	962492	469548	1177840	1709662	2623210	1949959	1290349	1738837	1904090	2315693	1778341	1773732	1256013	1280345	1501547	1399400	2218723
EIC	Negative	301.1117	1.52	unknown	0	368378	0	0	819773	590801	578845	0	383638	0	436762	93740	33628	0	541958	411365	109448	0	91722

EIC	Negative	463.3425	6.61	unknown	1174838	666470	190939	320252	3557657	2073331	2525574	1645817	3881024	183262	621670	1456345	2493568	3472684	1843942	2110988	3425171	1205273	2385444
EIC	Negative	415.2164	1.56	unknown	37620	603514	0	122837	1044150	33475	890479	44671	1935928	0	0	0	15292	0	0	0	0	84867	0
EIC	Negative	593.4788	6.57	unknown	1958444	1239978	1172356	1370726	1697193	1873787	1902613	1814319	2730658	1434787	2409126	1911489	1969460	1886520	1977405	1770269	1590780	1828987	2122254
EIC	Negative	564.5084	6.65	unknown	180310	309880	542779	645590	428303	466531	435510	491388	697931	284157	235011	355176	613243	346469	210455	473058	318445	372710	226825
fpHILIC	Positive	112.0870	4.47	unknown	2030117	4911722	1885583	5163625	3243265	7171675	2137201	5019560	8655113	2335992	3446974	4415662	6331440	4470103	4737413	6481293	4291787	5713219	4697952
fpHILIC	Positive	222.0583	0.57	unknown	2364999	2682288	1000008	1364640	2882960	5099809	2152651	1663048	4031535	0	0	0	0	0	0	141528	0	0	0
fpHILIC	Negative	524.2783	0.50	unknown	4484048	10223448	2263319	3552335	10046935	9491353	9649853	3929237	12428929	2441886	6487890	7063633	6443088	7286598	7169715	6562376	8408240	2226860	5990923
EIC	Negative	524.2786	4.54	unknown	109056373	215185054	92208702	139615952	206036691	198834741	185581365	137855306	210726939	96596188	134395955	136818376	146850131	169174770	152307114	126425230	162983274	112124707	151037626
EIC	Negative	629.3659	4.26	unknown	0	0	0	1503199	1692067	1931390	0	1237061	1711719	1165881	1855731	1699059	0	0	1619430	1960973	0	1120050	1731945
EIC	Negative	272.2315	4.51	unknown	183821	54738	187900	165594	213129	214785	134288	241697	193888	142042	53229	58368	117508	138006	0	126753	75696	187680	28328
EIC	Negative	510.3589	3.57	unknown	29489	180438	31694	0	144576	183863	136239	31082	237977	0	145004	113931	131990	144460	184002	166365	117094	29330	98061
EIC	Negative	604.2911	2.20	unknown	0	207491	0	43963	168314	175707	281729	62890	224522	314181	174786	201069	181434	141488	127925	164373	219219	106066	252828
EIC	Negative	569.3659	5.50	unknown	1347852	1979071	1259985	1517012	1847162	2077740	1745909	1634276	1495378	1175649	1613136	1669895	1637469	1610869	1526627	1380003	1524603	1177854	1579615
EIC	Negative	291.1600	2.53	unknown	56847	45408	77103	28521	59860	53386	81161	99264	33155	32522	44620	34614	45662	46177	29519	45518	32973	108611	53668
fpHILIC	Negative	224.1292	0.45	unknown	495565	514146	594541	416029	592128	818066	603688	344874	506108	489554	1092905	991725	667597	1076302	411391	476749	598954	241156	590356
fpHILIC	Negative	339.1997	0.35	unknown	5238671	10699347	12432930	20114202	32788824	27584660	19510650	16248549	22808396	21111794	19338822	32193374	10055731	21759554	13185659	12550427	28617324	10646271	15882926
EIC	Negative	483.0908	1.48	unknown	0	12802	0	0	48307	105060	348367	0	0	0	63271	34163	0	27243	0	32921	45748	0	9069
EIC	Negative	437.2458	2.06	unknown	361242	1027361	0	0	1107011	1012695	1075807	420442	907026	249477	879661	1045238	753201	755512	816467	756693	904950	306708	948032
EIC	Negative	272.1693	2.16	unknown	1738858	1257628	1209277	3347512	1990317	1285814	2070951	2130777	3016797	1922326	1204817	1265048	1901811	386882	1085223	2763940	1324195	2937204	646841
EIC	Negative	567.0064	0.55	unknown	149904	470495	266413	519136	693456	293221	650525	1198758	336524	142519	387959	448288	566058	704830	362455	342667	510427	534407	104429
EIC	Negative	379.2480	1.74	unknown	499511	155221	178103	0	344129	682095	663403	58258	399902	0	15174	15534	21755	18789	16562	19204	15297	0	16921
EIC	Negative	327.1458	1.33	unknown	84277	95738	0	0	133294	129491	131212	0	88312	0	391532	233786	150012	521371	70219	156260	70424	0	112540
EIC	Negative	649.0095	0.55	unknown	95698	283785	175238	255874	374316	159049	396774	813886	195812	143502	249232	295014	364216	401041	205317	141313	318109	293744	70235
fpHILIC	Positive	528.3037	0.50	unknown	543331	527754	388132	426183	611985	631348	587457	352691	655405	228521	466478	590597	408821	452317	409967	326898	516735	227683	339025
EIC	Negative	337.2327	3.73	unknown	0	216274	0	0	291744	298720	0	343397	0	411533	244931	0	343853	161097	0	367791	271689	0	178253
EIC	Negative	303.2333	4.96	unknown	197255	143722	176827	352407	221031	277966	263055	256832	291994	184345	214775	198533	248423	99604	161504	249131	155266	193566	146141
fpHILIC	Positive	147.0220	2.99	unknown	529272	923623	782691	1064974	1694410	7049561	1909346	477340	1143408	586888	1019879	1536343	1362219	586892	1258743	1865514	1739155	1174352	1475320
EIC	Negative	250.1120	1.48	unknown	0	24090	0	0	31936	41713	47672	0	70339	0	32786	38820	50322	16168	27451	57247	37588	0	12539
EIC	Negative	560.4752	6.47	unknown	16100565	20244144	20007801	21031247	21231151	16915104	21075544	19993470	28746470	22659715	17296264	19740478	20476921	16602435	17269345	21252793	21216175	26265733	18359967
EIC	Negative	442.0726	2.09	unknown	47416	111686	0	0	177008	178471	190186	64243	217179	0	234935	0	31575	95601	196208	314975	0	0	150338
EIC	Negative	321.2913	3.99	unknown	554374	562570	572962	537672	595921	593738	561151	574515	529589	543660	577206	529404	524748	511473	581162	561876	560357	539267	559600
EIC	Negative	500.9273	4.20	unknown	208784	274965	193207	236077	276105	295671	239820	201636	271218	181186	263869	230796	246245	275987	265870	253391	226877	192172	214425
EIC	Negative	283.1023	2.25	unknown	0	112188	0	18771	145255	89929	177567	52037	91127	0	109421	38578	28340	23888	135859	117333	38633	0	37501
EIC	Negative	269.1402	2.24	unknown	163342	148376	192762	284122	188451	210506	240552	395036	236385	312722	119716	148597	138535	126927	142503	182720	163453	371041	208511
EIC	Negative	343.1780	6.05	unknown	129665	98900	164278	122597	146437	133552	222418	222975	78794	185061	125643	18899	18620	0	105462	216280	0	430925	0
EIC	Negative	227.1651	2.82	unknown	114300	120204	241340	122338	176397	226175	196446	236115	146395	146542	156692	272387	218951	103195	145308	175316	162420	193024	122988
EIC	Negative	349.0490	0.57	unknown	46951	76229	159754	408293	129757	59428	131827	158607	726588	238364	99791	112738	236510	12695	68526	240703	55041	214361	68642
fpHILIC	Positive	526.3767	0.55	unknown	8916989	10690752	9517607	11133795	13683256	13506429	11115681	10898899	9274754	10926128	10795809	12597934	11471977	10902567	11898953	9343181	12500886	11288419	11050815
EIC	Negative	371.1397	2.85	unknown	0	87019	0	93657	123347	87641	87371	62731	159708	136984	129543	122243	117634	55290	43376	94205	99886	169547	49736
EIC	Negative	250.0259	1.93	unknown	0	78751	0	0	109726	86715	99953	0	79442	0	163951	142209	143013	160197	44936	85681	137829	0	101416
EIC	Negative	453.2865	1.66	unknown	316976	200943	117639	0	261112	548565	636276	0	226116	0	14390	0	0	0	24620	40128	25668	74759	19037
EIC	Negative	346.2029	3.39	unknown	545550	390441	585717	409566	552808	533000	600255	730062	360581	522887	473463	413463	406860	403204	488418	422373	440440	537389	379185
EIC	Negative	295.2643	6.66	unknown	190984	248613	365105	536588	341685	395934	337010	394628	496752	314247	294578	443937	440439	342442	288774	398435	365868	490129	300787
EIC	Negative	416.2659	1.90	unknown	0	36659	0	0	61391	59081	68755	0	146789	0	59279	56481	64028	22805	34607	85764	51587	0	28606
fpHILIC	Negative	213.0059	0.97	unknown	34677116	27642664	22345810	10184652	38861712	54237204	28975176	19796590	19152020	242055	239877	259304	280972	208402	268795	298967	156033	108467	235553
EIC	Negative	299.2531	5.99	unknown	0	0	0	0	0	0	751591	0	225831	581260	260933	599595	564833	0	89397	180834	0	0	687063
EIC	Negative	546.3176	5.77	unknown	362501	555864	347492	411792	498340	546690	482992	450822	402266	330424	456707	434743	444935	434345	426375	406247	466475	364782	434071
EIC	Negative	527.2963	4.72	unknown	241788	581926	265759	305671	619573	614299	426635	356026	770480	274918	435764	381008	364756	814632	569764	434811	496225	287398	424280
EIC	Negative	373.3152	6.35	unknown	239138	451568	200745	271104	450161	302618	433019	455863	285770	121139	275560	287259	379704	245239	238364	304464	352216	318641	443691
fpHILIC	Negative	181.0870	0.61	unknown	2213339	2376599	4427739	2623586	3871170	2651816	3505635	4225128	3064823	8934986	8366548	7530375	4814309	5978010	1105312	3567067	1978202	2252232	2081183
EIC	Negative	506.3540	4.97	unknown	0	164557	128927	0	125041	182669	361927	0	502093	20617	104462	0	355394	37035	124369	396150	238752	292155	90613
fpHILIC	Negative	304.2362	0.37	unknown	8381163	16747248	10906407	17820446	15114603	17070092	18860218	12036409	19378396	10310608	12941742	18878382	15411417	9915997	13095561	13323429	18121652	13592811	14617021
fpHILIC	Negative	383.0993	3.13	unknown	443946	319818	198516	239501	421155	701705	546179	220562	398086	230152	407256	383843	392520	201526	323257	416704	339033	175095	274445
EIC	Negative	500.2679	3.98	unknown	103670	267744	0	66739	260103	193355	188024	94243	584376	0	107165	83856	146944	340208	146719	158742	221276	75904	164333
fpHILIC	Negative	586.3633	0.54	unknown	1878576	2904567	1889519	1388061	3034273	2916144	2582393	1829184	2187414	1788077	2335287	2543704	2392906	2420873	2558368	2388865	2450509	1484428	2464747
EIC	Negative	365.2338	1.53	unknown	101605	96472	51236	202125	90326	237094	116364	307651	134594	73487	116862	220691	175419	61694	139960	246489	122479	215864	56358
fpHILIC	Positive	136.0480	1.80	unknown	1097241	2310207	2227014	2657193	2423238	2509471	2480195	2462907	2714539	2752625	2578964	2547438	3078635	2217494	2440267	2456039	2404836	2374339	0
EIC	Negative	285.0359	1.19	unknown	528145	579769	585224	548749	570980	585958	594449	575289	595571	550364	587626	590643	583081	554313	588474	563475	602916	477662	587332
fpHILIC	Negative	202.0722	1.78	unknown	419785	439732	421011	589530	736305	836202	672429	484864	604443	488336	652956	773199	633076	462642	614302	740604	573517	576216	472790
EIC	Negative	439.3797	6.84	unknown	30422	34445	40018	38630	108695	352625	58391	37473	61406	39998	0	44785	13902	8926	27112	38318	15475	26789	25034
EIC	Negative	377.1557	2.85	unknown	0	91267	0	111692	164578	120525	136003	45705	264886	134894	123736	191267	188988	91513	56822	168321	156604	186406	93828
EIC	Negative	356.2472	2.35	unknown	52814	877524	823906	801572	822077	852188	909283	889800	871227	817802	852787	818494	879155	91450	124536	846469	859358	760082	940866
EIC	Negative	485.0032	0.55	unknown	226546	703259	465482	781331	1089155	350600	1024392	1685594	526251	194397	523815	612786	834697	1143733	579244	384086	702700	830636	165798
EIC	Negative	603.3501	4.72	unknown	6405364	7742920	5904066	6647734	8000460	8732431	9651335	7449530	6512283	5896993	6959759	8281182	7810401	6309378	6985567	6624856	7593013	5495445	6819657
fpHILIC	Negative	340.2031	0.35	unknown	1185772	2179808	2587195	4246572	6878557	5818965	4625841	3278087	4671338	4710349	4445605	6816482	2185830	4683231	2941901	2996227	6195466	2365835	3649657
EIC	Negative	267.0544	1.19	unknown	75317	76315	69462	57539	71650	86579	80983	70569	72725	70803	82019	79603	70470	77181	75720	71568	79706	50544	74524
fpHILIC	Positive	415.2114	0.41	unknown	14151370	15104911	12588681	15604924	21098928	19562964	14930810	15639228	45643708	15232915	13688559	15896759	19199078	17147710	9339141	13345355	14033202	16358121	10763515
EIC	Negative	446.3006	3.08	unknown	824051	337465	445669	333352	511620	619624	466342	885263	795672	332819	216846	406865	345343	639430	528030	399231	378351	665678	327964
fpHILIC	Positive	85.0290	2.32	unknown	800180	956930	1003372	1425630	952462	1097240	1076947	922266	2050307	885448	1273760	1133586	1385937	547422	757960	1648055	832159	1074273	823704
EIC	Negative	540.3677	5.61	unknown	11131341	15722989	11185061	13292223	13470151	14605386	12208680	11884463	15113567	10959272	13470761	12357666	12402472	12071227	12298221	11818346	12099315	10684103	13657858
fpHILIC	Negative	190.1183	3.41	unknown	438441	985954	383447	364175	924706	1631234	1461704	869494	605907	434646	1192124	1281229	1342666	1452426	894874	761590	1275271	672375	1099345
EIC	Negative	255.0665	2.14	unknown	0	102635	0	70275	109085	99253	92618	0	411334	89846	78863	110494	242207	150089	73090	188881	167126	120796	106712
EIC	Negative	570.3686	5.62	unknown	358757	551501	323528	355093	470442	665136	465495	413918	419532	306347	411179	441373	498202	453036	541220	422094	464065	241281	414386
EIC	Negative	272.2315	4.39	unknown	361147	285516	1250359	1103558	947214	567759	792179	1623651	1307284	649117	299854	414895	605776	344809	179003	526686	503106	893934	366472
EIC	Negative	291.0075	0.55	unknown	598037	1516420	1502331	1671541	1448815	1059526	1796948	3110363	1698788	881957	1495024	1653796	1693795	675172	1348565	1266484	1884846	2164440	794737
EIC	Negative	376.2218	5.02	unknown	39537	44537	83517	202155	94727	91592	119505	67717	143044	96999	48501	131884	110451	92735	75880	109932	105973	127619	41464
EIC	Negative	418.1868	3.74	unknown	142939	463761	827291	225664	486303	99263	1341064	1053897	243722	299920	798250	114931	30790	130415	125173	0	30433	49267	46094
fpHILIC	Negative	115.0765	0.53	unknown	5106730	3563046	6579814	6543977	5780708	8863869	4845259	6004012	10582782	7691272	4681995	6474183	6501751	8436244	3859156	5054209	9697968	6216674	4160652
EIC	Negative	235.1339	2.36	unknown	291856	145934	353622	399987	244252	215467	418065	397289	386197	435441	208466	201754	129404	142331	256826	206620	317100	230036	153063

fpHILIC	Positive	151.1438	2.08	unknown	7008461	6000321	5632095	1056206	6673479	52756648	17974854	731279	4632397	1174339	13974185	7544043	38264616	0	3524041	3655692	2370514	904789	7360060
fpHILIC	Positive	351.1978	3.71	unknown	2083180	1293539	1342842	1098926	1717485	2299828	1928289	1226869	1234795	1046736	1172478	1295907	1219933	852276	1402210	1544628	1093321	1114492	974260
fpHILIC	Positive	416.2150	0.41	unknown	3645518	3765876	3326833	3684636	5492656	5260463	3709966	3963732	11635628	3797517	3594067	3951653	4806115	4635632	2272238	3414819	3846859	4175442	2742695
EIC	Negative	544.3217	5.77	unknown	922905	1399607	974983	1069452	1327874	1400754	1277599	1101402	1115557	872361	1163090	1140276	1125823	1085507	1035641	1107566	1120243	957489	1110662
EIC	Negative	305.1748	3.63	unknown	229900	199018	257670	294579	183323	221068	302095	425632	336216	320061	119794	221718	286082	131752	168938	209456	229481	248947	152885
EIC	Negative	602.3468	4.72	unknown	18940376	22873485	17057184	19131752	23584018	25604522	27822043	21586397	19071469	17418108	20866380	24343343	23159678	18416937	20572309	19203991	22171982	16201820	20558627
EIC	Negative	292.0114	0.55	unknown	26912	123428	116553	129950	110492	88099	151451	234111	112977	61447	109883	127459	121448	50018	93145	113302	151079	157128	64892
EIC	Negative	460.2752	5.12	unknown	52150	0	0	0	0	0	173458	243133	0	0	0	0	0	0	0	161219	0	0	0
fpHILIC	Positive	152.1473	2.08	unknown	480584	331982	362130	132701	436770	3437984	1246765	30009	354404	47418	858646	531770	2500296	0	265695	228208	151123	0	605337
fpHILIC	Negative	132.0452	0.96	unknown	1094599	826614	690212	397326	1128446	2006386	866245	740732	666296	0	0	0	0	0	0	30129	0	0	0
EIC	Negative	363.2180	1.89	unknown	359386	317220	0	0	600764	1631485	502862	0	542520	0	532100	645436	687414	387255	642156	1087950	575556	0	330219
EIC	Negative	429.1953	1.92	unknown	0	121320	0	0	382781	83636	187338	0	187244	0	106313	100883	120670	122163	150179	126548	121652	0	106248
EIC	Negative	455.0134	0.55	unknown	272790	749033	684307	798323	663814	512543	839781	1777566	848684	420269	769501	848478	856301	294249	641012	557962	988698	1007622	335972
fpHILIC	Positive	370.2947	0.60	unknown	2557553	2074874	3105647	6384294	3192759	3576829	3638179	3730537	5979772	2962094	2215117	2493397	3008383	794845	1458648	3731710	1828952	2818931	1886718
fpHILIC	Negative	278.2206	0.38	unknown	2900248	4054886	4932572	6442316	4611038	3619718	4232610	6002612	7771099	3550254	3066009	3667761	4839971	2203612	3237473	2947332	4068507	3607646	3456177
EIC	Negative	564.3663	4.99	unknown	398732	547712	367989	464080	634160	613428	519491	335436	573779	385957	509978	501949	511256	363656	558956	529216	461655	341075	461769
fpHILIC	Negative	500.2784	0.51	unknown	8016202	12662830	5866781	5534536	12596603	13936532	12009148	6759629	10373419	4302321	9200402	9275060	8177341	11144386	11811210	6974667	11661153	4668868	10819332
fpHILIC	Negative	618.3403	0.62	unknown	722136	729513	513409	720058	684420	1276304	920209	983028	749431	690069	608516	924069	875674	672688	655850	690018	827938	560631	935639
fpHILIC	Negative	142.0509	0.44	unknown	791217	1098603	1497147	1372914	902739	1079683	1652739	1406199	1286379	1772814	1269120	1279448	1331626	897642	824728	932003	1350353	1076676	1159034
EIC	Negative	502.2942	4.93	unknown	3397511	6302075	3935557	3471065	6446460	8132025	5781460	3856844	4278283	2667020	4790394	3341963	3417472	5680494	5111848	3103578	5009039	3181928	4556497
fpHILIC	Positive	77.0796	2.09	unknown	744399	679794	648990	312731	796179	2905760	1443193	172295	761079	230925	1808669	983666	2222004	52539	458357	578495	442246	245843	951027
EIC	Negative	385.1877	2.17	unknown	45173	175751	36045	101572	137603	86585	175539	106994	263920	38501	124602	103370	119111	156891	159369	191282	104683	125260	106008
EIC	Negative	540.2733	2.82	unknown	338500	675609	304833	291597	550440	855758	461300	364853	1068007	416915	438439	622897	453400	406003	657463	771874	830252	257487	737511
fpHILIC	Positive	155.0536	2.44	unknown	1181348	674440	2225072	2093743	1593411	2505393	1066860	2791478	5634541	1654410	1059607	3406558	3581137	2848346	762451	2034580	2262077	1496138	839619
EIC	Negative	401.2184	1.99	unknown	0	541643	0	0	562640	860495	984542	0	820694	0	630388	681518	490300	280733	629353	566195	444989	0	336184
EIC	Negative	318.2672	3.43	unknown	614268	651709	567532	523806	607497	631977	615191	608407	581238	530413	614209	635342	600047	583270	616616	558772	596078	527801	617658
EIC	Negative	445.2958	3.08	unknown	2801659	1173773	1482294	1016546	1687249	1964528	1536452	3060325	2790479	1131012	734134	1387658	1157494	2021547	1669163	1305018	1275548	2278630	1132540
EIC	Negative	564.2584	1.64	unknown	232341	253655	0	0	186530	287828	378087	228417	292944	251013	283220	353753	306613	188096	145070	0	287317	49487	312677
EIC	Negative	557.3570	6.10	unknown	63020	1210460	175583	0	909678	1751435	0	0	0	0	0	0	0	417490	0	0	0	132374	691803
fpHILIC	Negative	263.0650	1.04	unknown	626069	627094	441686	878303	742094	729731	607501	515031	717011	643542	514585	514147	583172	434320	463226	490166	590469	528167	392758
fpHILIC	Positive	281.1383	0.41	unknown	510261	536795	430286	503719	755710	722478	502097	637664	1899439	575111	493515	714698	710089	626782	384343	511210	463334	579001	362404
fpHILIC	Positive	219.0261	3.13	unknown	531110	706973	628175	739255	771245	825916	784694	750161	618875	601801	735374	694074	622419	427020	566636	774043	558925	596869	519117
EIC	Negative	283.1111	3.08	unknown	332533	438464	374005	418517	657282	508794	508835	373012	472029	382164	371982	368548	415932	419421	443840	442114	480371	319812	482922
fpHILIC	Positive	221.1211	1.65	unknown	0	5277987	2349047	2963015	2352119	6195674	3642545	1608064	3787849	2383514	3957465	3862241	0	3435193	5397758	5043238	1526264	2627239	0
EIC	Negative	602.3469	4.89	unknown	3953987	5152724	4057117	4103957	4986301	5769757	6202547	4842156	3806874	3781695	4541854	5691035	5217371	4338065	4470760	4311711	5402458	3997668	5357797
fpHILIC	Positive	192.0736	3.10	unknown	220104	379467	301240	389260	359915	424169	290319	255357	704175	286944	346306	350190	352298	284797	375635	398343	328542	254434	226254
EIC	Negative	509.3498	1.86	unknown	69313	65906	0	0	98749	81326	75871	0	93268	0	68408	58871	81918	60197	97568	93335	63834	0	60512
EIC	Negative	454.2847	4.98	unknown	564762	827055	535711	729831	752860	838536	607105	593138	905115	489482	556106	614882	495503	864460	512991	467572	631591	486663	597026
EIC	Negative	499.2650	3.99	unknown	334223	1422246	0	376598	1288020	826980	803885	463192	2737447	270528	81430	39635	702909	1693019	89892	674843	970157	333839	727554
EIC	Negative	253.1062	1.37	unknown	0	148391	0	0	247421	182847	316480	0	390948	0	189551	210883	217749	122322	153927	199612	233145	0	163123
EIC	Negative	255.2230	6.42	unknown	2676693	3020414	5620203	6290844	5375410	4063736	5675892	5453652	6040881	4354675	3379719	5180920	5506747	2457747	2420632	4820357	5657438	4377734	2614218
EIC	Negative	299.0957	2.76	unknown	68128	288481	51342	42404	361540	268021	437294	85543	217243	15720	275486	109258	91675	112309	295008	232700	115482	39224	78448
EIC	Negative	423.0376	1.19	unknown	145102	156756	138921	151446	173192	189621	186667	136781	160737	156419	245961	173760	161956	174194	171296	149761	173216	147578	198945
EIC	Negative	391.1975	1.97	unknown	0	350823	0	0	593684	469562	588153	0	363276	0	403913	313581	337990	126246	298829	461339	37386	0	243913
fpHILIC	Negative	79.9573	0.97	unknown	3233037	2836972	2324265	1178047	4018140	5576484	2798867	1853253	1753216	115226	110111	143041	117103	73841	118641	138096	96322	140006	136688
EIC	Negative	262.1123	2.05	unknown	0	195219	0	0	204297	196568	213497	0	221461	0	182348	183019	193982	246153	209051	203250	234771	0	178855
EIC	Negative	302.2206	6.25	unknown	9146689	27076286	10889743	31751814	30852609	15827001	23294888	16702742	57509691	14384853	15378779	12299084	28939707	17066151	15034262	26006435	24582045	13433720	11042575
EIC	Negative	246.1533	1.67	unknown	782930	992206	665297	2490615	1329447	1661345	1567104	796811	2699791	1473954	1634728	1269538	2106069	371963	1339735	2224197	1454481	1726432	584093
EIC	Negative	534.2277	2.72	unknown	418935	543850	517534	415284	474516	546062	528229	465221	595710	444644	553239	536986	564993	586268	581641	468216	524455	425838	488094
fpHILIC	Positive	319.2271	0.37	unknown	303278	353876	297253	375693	324487	385323	344482	251351	1405866	202645	194199	270520	448978	361686	110525	190404	897962	410045	580366
EIC	Negative	431.2108	1.01	unknown	191665	891664	401315	318866	793806	37133	868381	1121997	1606723	155393	755103	756351	796078	448410	1314133	1112713	454291	1146611	232707
fpHILIC	Negative	413.1971	0.42	unknown	613549	943348	546672	378386	1038283	1309592	775800	801688	3449706	659208	613355	892490	1286693	1018380	691193	642455	686541	858581	766711
EIC	Negative	429.0547	1.19	unknown	120155	147169	133433	118972	148734	164998	147884	129835	153809	126863	158273	147592	154318	153870	152936	138938	159164	94290	151070
EIC	Negative	272.1693	2.07	unknown	935487	921554	692852	1847903	1227828	1195347	1266817	900190	1776188	979412	1264337	1042883	1547215	334012	998139	1703098	1224864	1416998	552468
EIC	Negative	584.3161	1.42	unknown	0	48043	0	0	63477	62719	127835	0	48027	0	0	0	0	0	0	0	0	0	10004
EIC	Negative	244.1377	2.16	unknown	31486	59246	0	0	49695	63739	89082	0	124073	19476	52145	52357	50848	50781	50697	51089	41219	0	44387
EIC	Negative	568.3626	5.62	unknown	5250073	8604823	5269538	5787550	8044668	10721167	7528334	6572801	6730166	4476035	6291730	6348694	7934166	7159682	8355305	6897036	6994808	3984699	6730413
EIC	Negative	456.0163	0.55	unknown	26687	99138	90595	98776	115628	45418	116434	207583	100110	70365	93571	101002	110598	49241	84150	50799	139149	120644	54444
EIC	Negative	601.2997	2.20	unknown	0	61289	0	0	71462	62815	111828	0	99462	137038	81873	59404	20245	44616	52601	69613	74759	0	52687
EIC	Negative	429.1954	1.55	unknown	98745	308365	169014	61946	1111742	103766	513962	185764	296035	54640	217153	141914	200342	205990	394536	346807	232741	228285	166557
EIC	Negative	239.1077	3.07	unknown	109476	129905	124733	160064	132813	138259	150907	162865	132510	155877	119293	115116	138340	124193	122340	135673	127565	148662	133111
EIC	Negative	318.2671	3.31	unknown	1456548	1581296	1518112	1458653	1598797	1571721	1542809	1474337	1494593	1438677	1541137	1497390	1530481	1499038	1506115	1475534	1574212	1425729	1540669
EIC	Negative	416.2014	1.45	unknown	0	109564	0	0	135733	141674	81091	0	169911	0	80887	87716	127671	43590	59646	148605	102647	0	29274
EIC	Negative	303.2237	6.25	unknown	1035115	3325551	1302300	4769657	4873133	1933141	2922996	1956554	6997088	1724880	1868074	1571848	3541926	2334080	2431581	3189968	2954770	2116047	1360187
EIC	Negative	373.0103	0.55	unknown	480627	1367478	1261579	1497215	1232843	876287	1543828	2961122	1489425	737788	1328040	1496192	1496505	525997	1166314	995006	1635419	1867108	627306
EIC	Negative	499.2650	3.82	unknown	0	519762	0	144339	470199	388351	301605	0	939996	57060	252936	139648	247324	668561	289614	323419	363529	0	238112
EIC	Negative	299.2530	5.60	unknown	0	0	0	772974	930335	1067252	6938177	0	0	2586689	2948205	0	2197240	0	1254854	2119918	2421981	0	2553937
EIC	Negative	603.3502	4.89	unknown	1342921	1742535	1365278	1371221	1775657	1983007	2206672	1596316	1247178	1304390	1520735	1896334	1732613	1490211	1537630	1454991	1849833	1336097	1893948
EIC	Negative	395.2755	1.83	unknown	84314	169725	0	0	47657	80794	585801	142481	104505	0	0	0	0	0	0	0	0	0	30248
EIC	Negative	381.2622	4.32	unknown	31752	0	378309	60752	428247	413102	38210	401526	460183	442987	40912	0	57299	0	21230	86364	53385	506352	20208
EIC	Negative	377.1977	5.27	unknown	266219	197925	217194	250282	192058	372441	295797	272667	263675	336030	234017	239272	165506	182960	185512	168765	220934	271648	226756
EIC	Negative	503.2979	4.93	unknown	934464	1716491	1290281	1026796	1798807	2225230	1618996	1058593	1245367	754769	1345006	899797	960238	1635464	1379563	870420	1511274	879954	1209114
EIC	Negative	243.0331	2.17	unknown	44175	116113	93620	36132	116632	74433	115839	74729	123212	71549	203745	246409	115884	203919	44085	143617	76278	26178	74155
EIC	Negative	346.2245	1.45	unknown	0	61655	0	0	77591	102053	114033	0	107890	0	64985	108551	81459	21328	33987	99717	61752	0	63712
EIC	Negative	367.1583	1.95	unknown	0	97485	0	0	357395	0	200085	0	144046	0	32008	0	0	32582	101963	85851	32620	0	0
EIC	Negative	568.3626	5.50	unknown	4459175	6794955	4395154	4772042	5893933	6600476	5555365	5285321	4872651	3924440	5334935	5292093	5406765	5441968	5157689	4584316	5102533	3943738	5312026
fpHILIC	Negative	311.1296	3.36	unknown	1663910	1205131	796566	799691	1247057	1471865	1353912	1104319	1437142	648980	766327	1172345	1328651	932195	1212835	1468140	1054949	1149933	653256
EIC	Negative	584.2968	4.93	unknown	156500	274596	167111	156528	313638	278831	225249	172612	203691	135719	188098	134537	143644	259679	230909	154488	217724	161777	188316
EIC	Negative	594.5563	6.72	unknown	77834	151634	148664	189742	197606	129956	207092	174728	160327	133221	150227	148568	162479	103450	121905	121196	112625	145861	84826

EIC	Negative	487.1907	4.08	unknown	132987	147809	123787	198179	181289	153012	197091	165346	251112	233339	158059	197411	187699	83995	99965	200196	183565	209023	100814
EIC	Negative	359.1902	3.65	unknown	7243091	0	7208673	7314741	7100793	7034373	7557623	7550868	8214069	0	0	0	0	0	0	0	0	0	7512612
fpHILIC	Negative	277.0595	1.04	unknown	1207305	854213	914206	1096207	1221454	1370408	1033018	779227	959717	811055	694498	640932	642298	629354	584294	632877	691172	636900	438967
EIC	Negative	233.1185	2.23	unknown	80208	51586	34308	80609	59357	50917	52425	96800	104410	69729	38580	53387	51543	36455	43189	54616	60722	80630	71098
fpHILIC	Negative	405.0291	4.72	unknown	933852	583210	807527	887693	895472	1234838	793901	879403	1046066	866277	729744	778288	854908	915162	767548	1005958	824790	931898	729075
EIC	Negative	244.1999	3.59	unknown	171450	48996	88894	89554	142368	163777	91781	140194	90106	71357	59678	54892	58115	62812	30727	55899	59296	83051	60704
EIC	Negative	352.2193	3.55	unknown	70003	58960	179964	52056	143687	87432	112295	188725	94652	126211	65245	73807	64462	97893	48160	53559	88568	147617	69365
fpHILIC	Negative	351.0565	4.50	unknown	578170	411902	233057	421216	565818	875029	533615	327766	1156348	223430	476568	725597	759094	731445	713556	761106	861819	277684	402342
EIC	Negative	303.0913	1.97	unknown	0	107737	0	0	172735	106793	159223	0	110618	0	139685	89663	84040	72353	144220	131827	77200	0	52594
fpHILIC	Negative	243.0334	0.50	unknown	0	282775	454903	189865	394283	262076	550804	372840	140446	192539	763894	407972	315034	520184	0	204100	273599	186563	346525
fpHILIC	Positive	130.0608	2.51	unknown	248649	246867	148517	267696	276779	450523	283411	159172	558473	198718	336977	432976	407910	281766	329123	535937	311183	183267	261929
EIC	Negative	241.1439	3.24	unknown	112268	113249	91688	89083	120891	114290	118467	97684	112377	105577	110055	118109	103980	100600	132375	153934	109766	0	123643
EIC	Negative	569.3659	5.62	unknown	1554370	2600049	1617061	1767795	2405183	3173852	2248569	2049628	2038309	1295033	1944056	1964418	2466315	2190293	2561541	2075987	2172656	1172410	2047520
fpHILIC	Positive	129.0657	2.57	unknown	1100822	448137	922473	1157077	981530	881008	840109	736810	1217876	1335094	744766	677640	698080	750931	764058	716691	1091812	1108603	628688
fpHILIC	Positive	638.3359	0.40	unknown	854881	1221673	858487	949492	1007456	1176613	882492	1233060	2091222	881799	958706	933027	1286261	1271474	580799	883539	963448	816608	1066065
EIC	Negative	633.5094	6.43	unknown	4057194	5682982	3123209	7211298	6190308	4068446	4977802	5073348	7439062	2369934	2321529	2756146	4354600	2545044	1920969	2326202	3407727	3343091	3491278
fpHILIC	Positive	249.1069	1.80	unknown	451535	1262769	1101538	1636933	1214463	1528489	1397251	1214520	1560159	1398595	1441500	1243128	1667228	1035206	1255753	1414913	1167956	1167040	1033973
EIC	Negative	379.1561	6.46	unknown	494880	958146	541296	925360	703204	601879	1056534	639505	1683603	506886	449452	781703	559226	788591	606385	658768	572382	600683	579702
EIC	Negative	381.2296	0.79	unknown	189668	177579	216739	599489	316770	762789	327054	1182752	155104	202365	296129	223126	121361	117401	667105	292507	214117	1135282	269574
EIC	Negative	301.2656	5.59	unknown	412514	29991	157690	114936	332243	453641	172146	218340	147011	68987	82572	43814	74289	79702	25529	30446	69781	126057	84968
EIC	Negative	402.2225	1.99	unknown	0	136315	0	0	123743	183073	216827	0	182195	0	145650	155445	80880	89755	123679	145874	128612	0	96612
EIC	Negative	506.3231	5.17	unknown	582616	573592	505182	530532	596862	772424	721332	578978	627209	502373	742289	585979	571053	595489	670204	632660	652694	477688	657035
EIC	Negative	602.3390	3.73	unknown	159780	761606	247640	286868	637390	480487	527974	372236	1010599	257389	465473	282920	537359	866171	428435	776921	596731	264554	392141
EIC	Negative	251.1292	1.99	unknown	0	239153	0	0	233078	300708	204131	0	261387	0	224500	235147	253776	183563	176204	322564	255027	0	164535
EIC	Negative	357.2523	2.15	unknown	398925	757464	474746	669771	652342	655366	750298	628321	634791	650368	614418	650899	527385	622930	619935	556400	633131	480380	575745
fpHILIC	Negative	301.2173	0.38	unknown	2187440	4995062	3595959	4644469	5399841	2842588	4747932	2777947	12655842	2764541	2799136	2472343	5474768	2512716	2104977	4047261	4331247	2515934	2511284
EIC	Negative	430.9688	0.55	unknown	170111	261436	325113	298251	253791	193115	323480	514834	335711	189923	294074	301424	326903	54546	245897	256075	348594	400334	159022
EIC	Negative	509.3497	1.94	unknown	0	195873	0	0	376622	76434	346337	0	230732	0	103604	162835	79503	130617	267928	258588	137306	0	151393
EIC	Negative	522.3805	6.49	unknown	1524403	2020791	1723769	1847741	2677131	1642823	1861441	2198421	1720120	1328664	1891777	1815489	1910067	1415573	1482796	1626083	2305937	2592496	2009566
EIC	Negative	473.2830	3.70	unknown	0	0	0	61002	24594	636532	0	0	452775	37512	10653	21411	11196	23718	20636	24059	0	96499	19678
EIC	Negative	443.8600	1.34	unknown	12462	72679	0	0	59332	85151	73986	0	99238	0	67727	56888	80603	77869	61782	80123	56226	0	48353
EIC	Negative	316.1224	1.97	unknown	0	290890	0	0	231161	204161	190988	0	634927	0	234938	240770	557728	165296	224722	692761	242631	0	69264
EIC	Negative	497.3065	5.57	unknown	434912	411910	411735	321683	423322	433920	421820	503850	313839	398357	324836	249756	258459	274249	294364	258343	357611	406064	351939
EIC	Negative	323.1842	4.08	unknown	3349607	3073628	3879343	7289798	4856024	3938474	5306632	4857537	6866171	6242639	4696922	6044049	5654808	1784792	2603589	5144051	5028383	6486756	2242031
EIC	Negative	564.2695	1.63	unknown	0	32764	0	0	29551	234786	304354	0	240875	0	239379	287019	233230	29298	36154	0	39222	0	259263
fpHILIC	Positive	168.0766	3.17	unknown	357916	183522	201166	135576	189600	875576	408030	163830	193444	145486	167513	247491	185844	213762	175366	622585	307071	240819	297760
EIC	Negative	247.1552	0.97	unknown	19262	13711	62219	10018	54262	11338	28172	58994	52588	16166	28905	15984	17164	13166	16174	23102	17910	9572	17082
fpHILIC	Negative	197.0819	2.78	unknown	447877	425269	434112	1953924	834613	1040550	1406233	815441	1771238	1104798	1113650	1517206	1736445	169889	490470	1994650	724258	722046	237010
EIC	Negative	312.2264	3.08	unknown	165309	99608	146156	160853	145109	136907	186853	146071	184154	106114	83064	126637	103512	96897	77328	272699	124694	131710	117334
fpHILIC	Negative	213.0227	0.38	unknown	1622968	975157	2650580	1834539	2056719	4427563	2284377	2644766	1189675	1796103	1536091	1955338	2173834	1657221	1475813	1692824	1798205	1569405	5684279
EIC	Negative	383.2553	1.58	unknown	800983	1004920	666726	700190	996218	968311	946243	744172	983411	780097	950041	951284	1100666	916095	922515	972559	974630	550969	948474
EIC	Negative	321.1689	3.43	unknown	2818558	2579776	2995099	6668811	4271056	3293595	4095156	4537305	5770246	4618405	3343717	5293397	4607333	1563065	2012797	3954610	3721753	5421596	1938416
fpHILIC	Positive	602.1880	3.15	unknown	929431	935928	997919	858202	949698	927607	941609	1044439	977232	1196585	1004238	1027437	1073805	880318	834912	894710	823706	830759	829492
EIC	Negative	301.0726	2.33	unknown	39668	141875	100285	0	98805	77146	133837	122107	40795	0	94215	0	16648	15022	58696	290177	0	0	53779
EIC	Negative	330.2077	3.98	unknown	436695	267487	433686	382485	444811	299226	415131	459575	376505	368547	306759	321992	292883	280839	312684	321412	293954	370211	264195
EIC	Negative	324.2186	4.00	unknown	171073	178813	137540	111428	176802	145969	168310	200800	133501	154017	143520	124971	133804	114826	161963	126957	164106	183198	152312
EIC	Negative	619.0206	0.55	unknown	66997	283992	203584	242612	187742	140020	280697	687981	268982	126168	271599	294899	307274	47985	190796	158506	351906	358814	90323
EIC	Negative	423.3495	6.72	unknown	83574	127566	169761	137128	232169	742469	173765	132026	96843	147008	157847	218115	81736	70636	123798	129394	0	266823	134548
EIC	Negative	581.3654	4.34	unknown	990224	1784120	707576	581369	837487	1691545	727778	1621992	985798	1493364	871659	1631196	887788	812837	824343	868137	1529281	1478209	784398
fpHILIC	Negative	383.0997	3.32	unknown	451573	230826	257563	319054	335638	523284	2199286	290917	315210	278899	0	334080	268240	327383	347106	424484	263714	773275	405825
fpHILIC	Positive	206.1278	2.33	unknown	2645387	3034607	3643101	5111136	2870065	3798107	3848280	3133889	6905976	2986509	4206600	3517904	4375296	1797513	2156603	5562578	2925588	3600214	2184067
EIC	Negative	475.2679	1.82	unknown	2146714	628532	655667	0	1719649	2723458	1839242	263147	394936	0	25712	0	59386	25708	54555	28745	26772	0	117643
EIC	Negative	633.5086	6.49	unknown	186914	0	0	0	0	1808831	0	1577666	0	151080	159729	0	0	0	239929	1149237	0	0	0
EIC	Negative	267.1435	2.17	unknown	104972	238599	58729	178280	145745	147595	177291	116921	691215	116100	175578	148897	260613	223086	175455	450653	285166	48764	98136
EIC	Negative	369.1558	2.04	unknown	3039275	3174184	1962757	2382601	4226748	5142840	4625506	1731382	4145867	1877274	3877264	4027524	3792895	1765058	4496853	6157952	3275174	1357013	1842176
EIC	Negative	358.2052	5.82	unknown	114585	282581	14546	111937	336258	169522	145317	309385	105841	171398	89362	150793	322129	24273	0	233150	43592	174923	41557
fpHILIC	Positive	374.1833	3.71	unknown	622081	565011	585938	484818	657339	653411	642404	495649	422100	583106	489610	454437	482106	358112	654473	528426	464462	529228	417720
EIC	Negative	481.2731	3.08	unknown	117977	29425	59988	37531	62303	78286	61036	104467	99240	56264	36854	59126	46266	81685	67245	57471	62688	98695	39809
EIC	Negative	538.0189	0.55	unknown	27079	74267	29781	99203	75624	39523	85193	178213	67298	29663	80840	64403	102158	0	77449	21122	96357	88210	29834
EIC	Negative	247.1562	1.67	unknown	85756	138872	0	360478	182003	221549	232491	89064	378956	224186	240076	187022	295901	68967	190750	324720	208194	245691	84048
EIC	Negative	368.2810	3.47	unknown	107989	96948	108431	233233	127488	168968	158248	136629	224228	111906	111721	146156	128603	49673	73044	141853	87989	102148	74517
EIC	Negative	429.3049	3.48	unknown	152233	133389	153387	336859	188371	217480	231702	189507	298942	147371	141404	195573	167185	62029	107848	213189	116137	161906	88385
fpHILIC	Negative	151.0261	2.48	unknown	52703112	25809458	30089962	9058243	34470980	45634952	28972302	18748400	35841744	19522364	1130492	723658	28742688	60417200	301527	1219102	46115852	6719166	2334273
EIC	Negative	280.1629	2.69	unknown	132271	133622	115288	96637	122849	113821	159324	143881	122256	112310	112217	103047	130927	114909	154832	133791	114599	81847	117931
EIC	Negative	326.2720	4.01	unknown	2492671	2645340	2270716	2358548	2483407	2609777	2470532	2584320	2306251	2237363	2363657	2312224	2284734	2408833	2338259	0	0	2411303	2375119
EIC	Negative	527.2965	4.90	unknown	511657	846213	429756	507136	911454	687896	639078	550729	1098501	324088	530550	485018	460853	925737	536035	398845	607137	453266	603867
EIC	Negative	627.3504	4.26	unknown	26254080	51679542	27902498	37355483	44605345	49777274	46815531	33287832	45255445	31142756	49300754	44229224	45398707	41199654	41682870	49464630	46472577	29147353	46687767
EIC	Negative	629.3660	4.43	unknown	2113385	0	0	2069788	2387607	2293702	0	2077262	2410695	2029343	2510879	2293461	0	0	2221694	2278461	0	1976561	2371897
EIC	Negative	626.3473	4.26	unknown	72984669	143305934	77314400	102449536	124908988	137809549	129279935	91917373	125455624	87513347	136569745	122200310	127406552	114295926	116493192	137226761	129389158	81303250	128531979
fpHILIC	Negative	283.1136	3.36	unknown	987611	824137	582946	463683	812598	880874	649014	733944	925219	464722	410548	739305	662568	716283	751255	991936	678148	810484	383970
EIC	Negative	251.1287	3.25	unknown	66588	38331	740225	220583	49916	176012	841751	683426	70581	495388	34706	0	91465	387108	306490	53907	36765	620721	56245
EIC	Negative	430.1997	1.77	unknown	0	37141	0	0	138640	7324	69454	18578	55258	37545	25452	17709	17718	29501	35178	41322	50412	0	31803
EIC	Negative	251.1269	3.32	unknown	85735	109116	144772	277373	188324	126074	191280	181375	221481	125802	86036	138090	131920	71956	60169	120920	165988	159763	77387
EIC	Negative	453.2865	1.82	unknown	14631329	3806185	4575116	0	9764663	17129469	12406864	3010153	2275930	40541	163106	138411	263801	206281	228548	202602	200085	0	500773
EIC	Negative	302.1760	1.99	unknown	0	172087	0	0	255778	198360	244729	0	224647	0	159730	133372	181397	175740	144825	154348	188608	0	150699
EIC	Negative	329.1761	1.92	unknown	298109	537425	0	0	713203	317155	677654	0	1404302	295140	192252	139681	278675	263636	291438	436017	228294	0	359912
fpHILIC	Negative	161.0455	3.35	unknown	15627901	13731492	15882382	16418002	15026591	17108074	17953902	16372770	14998609	16221601	18332254	15836476	16786786	10476907	14925538	14676331	15879607	15574112	14514243
EIC	Negative	498.2625	3.98	unknown	1850658	5149247	1779234	2145468	4542181	3108009	2922249	2146296	9619401	1493671	2170523	1500442	2534181	6263393	2311384	2528200	3521182	1930438	2527465
EIC	Negative	557.2713	1.83	unknown	300224	106660	105064	0	272359	395703	299211	0	99069	0	0	0	7253	0	8454	0	0	0	0

EIC	Negative	300.2626	5.59	unknown	3545022	416905	1385387	825175	2742888	3606904	1386978	1906266	1128087	506975	643317	418974	452202	607476	214534	407795	486860	876697	533685
EIC	Negative	291.0368	1.59	unknown	383855	827493	75855	55036	1056563	1929961	1020317	89595	365620	54711	867276	165041	73536	36956	460031	567975	54099	45521	42446
EIC	Negative	429.1956	1.77	unknown	168048	123210	126828	0	563273	38421	299928	118958	212232	178479	93357	65604	70552	121603	134396	144597	209569	0	115457
fpHILIC	Positive	249.1514	2.46	unknown	486541	305470	336460	1051141	297025	415111	518610	288430	1657247	495303	509240	470445	493720	109253	239148	735893	245243	480698	237699
fpHILIC	Positive	548.2982	0.40	unknown	408550	478618	224445	268881	561132	757549	471481	507418	4158072	318558	338465	609499	837223	862597	306883	418444	381832	485224	527501
EIC	Negative	299.2591	5.59	unknown	17852944	1954890	6935775	3958205	13509001	17969868	6970958	9717642	5652751	2853229	3100761	1960907	2242045	2974110	1409563	2184621	2561464	4365512	2567720
fpHILIC	Negative	191.0566	2.94	unknown	50655	180438	99370	36147	293790	193822	15058769	265362	187445	113344	40967	69371	76959	191104	63304	169599	79052	422433	132382
fpHILIC	Negative	131.0596	0.49	unknown	2015014	1315599	1331683	1672666	2214389	2297034	1885818	995647	2091430	1787495	1910629	2273986	1348751	842080	2160962	1598714	1071853	1357161	975460
fpHILIC	Negative	160.0981	2.31	unknown	358959	307407	418509	575424	325379	450475	448133	346970	712498	359535	441464	452185	543407	187714	267631	634308	281174	357475	281820
fpHILIC	Negative	152.0294	2.48	unknown	2715577	1532931	1737705	543494	1830380	2446486	1601239	960388	1994208	1098338	80278	0	1602501	3472449	0	67926	2540269	351226	116734
EIC	Negative	308.1598	3.00	unknown	201279	126358	140490	104175	156266	182823	155653	161405	135201	145658	131905	133846	113827	87307	124957	125321	21722	116222	91784
EIC	Negative	310.2108	3.68	unknown	204307	192851	165860	316445	259928	166597	288133	249958	364794	214779	148690	207560	173881	156748	157552	204194	238827	240885	195576
fpHILIC	Positive	134.0639	2.86	unknown	776651	393674	466193	608000	635291	977532	601601	397845	555886	459720	486825	543743	457449	601420	1038757	526158	491785	511100	294450
EIC	Negative	634.3347	4.17	unknown	2368520	2955800	2530424	2214730	2697809	2740611	2985458	2585531	3089408	2251161	2631872	2616052	2866481	2960897	2639975	2839619	2519585	2256977	2500108
fpHILIC	Positive	248.1487	2.46	unknown	4057309	2260916	2876578	8978147	2752974	3563200	3687251	2534627	12953754	3802728	3921356	3596028	4174721	1130765	1889564	6448228	2374494	4172708	1836218
EIC	Negative	411.2255	3.14	unknown	0	50331	23764	14663	0	0	374621	0	190769	0	0	0	15710	0	0	28423	16113	0	0
EIC	Negative	406.1895	4.08	unknown	118081	95664	97351	199306	138293	134108	161152	140510	191809	172245	134978	170218	167150	50438	81102	159557	151418	187952	68974
EIC	Negative	295.1544	2.85	unknown	1894611	1118567	1157028	2137799	1834281	1487975	1741141	1787073	1989921	1803227	1188658	1825686	1588849	793275	872936	1370041	1473631	2615182	810411
EIC	Negative	432.3194	4.17	unknown	112888	80894	138508	106414	100132	147565	119441	183730	97050	103318	108090	111102	84679	104707	110080	94316	106220	180828	62776
EIC	Negative	303.0877	1.96	unknown	0	0	0	0	160526	96666	0	0	110574	0	124505	84231	82495	0	131214	124611	15085	0	52447
fpHILIC	Positive	796.5815	0.44	unknown	955995	1193986	808262	1069377	1242498	1190109	1009887	1152882	902580	892519	858947	937517	858659	1018262	933146	909492	1234287	952393	949773
EIC	Negative	243.1965	3.59	unknown	1048239	314895	571965	543702	850042	1055424	512581	885865	548761	345951	370251	337571	321441	397217	250804	317067	362735	568221	367077
fpHILIC	Negative	821.5733	0.44	unknown	1459053	1364261	1257458	1119257	1484598	1157921	1089656	1209811	1632923	1243494	1363822	1559266	1251095	1327677	1511329	1049237	1502355	1055670	1323109
EIC	Negative	580.3623	5.40	unknown	164405787	167785930	169021492	155175970	166586720	185259651	171487030	172462465	160323384	149057615	174945315	179454923	172978005	163447155	170214195	160831664	173372745	159809650	180156772
fpHILIC	Negative	264.1088	2.31	unknown	415266	350970	489405	644485	356322	532288	533214	404774	793153	404119	535315	543043	556745	196900	311455	729877	347599	477665	347732
EIC	Negative	580.2681	3.98	unknown	129562	300437	94581	130382	289026	173479	168633	124352	602194	100783	149166	95238	152159	351239	152905	162893	215903	0	166460
EIC	Negative	494.2596	1.98	unknown	321719	306143	446497	278646	206719	410921	346150	647815	466440	268132	289640	295619	290869	963832	430934	163220	322443	283159	314863
EIC	Negative	266.1400	2.17	unknown	702665	1579514	487707	1015619	1016497	1022558	971945	847874	4502216	757646	1021133	997999	1776384	1547067	1121999	3043772	1898254	872236	638787
EIC	Negative	356.1904	4.11	unknown	865537	942725	553258	1835942	1194219	1165803	1674605	989255	1696227	1004161	948887	1325119	1536203	428503	899948	1839784	1142423	1136490	661767
EIC	Negative	601.3347	3.73	unknown	647443	3140014	803628	1138985	2520637	1806199	2137078	1251588	4252529	919915	1806408	1100333	2195464	3569080	1553926	3096294	2495184	924062	1587027
fpHILIC	Negative	249.1857	0.38	unknown	866605	1144756	1800622	4189845	1738177	1197448	2091835	2973043	3434432	1727242	1028409	1984000	1975744	792752	710856	1179101	2291891	2351164	1113872
EIC	Negative	587.3582	4.08	unknown	0	0	80233	355998	158300	111746	160634	148418	263769	210862	121437	210066	188471	0	20245	158307	156972	275828	15282
EIC	Negative	261.0744	2.09	unknown	166728	322395	136106	36368	305347	298812	325794	200216	322802	159100	309191	34816	171306	223013	287838	346279	91062	35793	260965
fpHILIC	Negative	303.2336	0.37	unknown	38868348	73059488	48898488	77960808	68095424	71616920	82647336	52972260	87542496	45958124	55500844	77274912	69511088	43888116	58164776	55731528	78535176	58446548	63663628
EIC	Negative	537.0172	0.55	unknown	158305	498512	433541	467294	374910	270266	511431	1169722	463423	220283	485499	526482	559054	204657	385679	314986	610608	638799	187314
fpHILIC	Positive	805.6237	0.46	unknown	535795	270106	358359	424067	446085	506788	473373	480432	331846	601124	343284	479514	262145	259055	418838	375472	398758	467007	528766
EIC	Negative	453.2821	1.83	unknown	143661	3192022	0	0	0	0	10382678	2373001	1914239	0	0	114681	221086	0	200029	168596	164644	0	424817
fpHILIC	Negative	187.0030	2.48	unknown	1214483	595461	787223	207493	822598	957110	739306	444546	775673	579676	0	0	804795	1608548	0	0	1005026	186285	70126
EIC	Negative	512.9719	0.55	unknown	131561	208812	222343	231690	174345	140056	214375	450577	282843	114244	215539	249484	256161	20515	191365	204432	255950	300966	110874
EIC	Negative	600.3314	3.73	unknown	1923634	9414003	2441818	3417572	7398003	5299382	6345658	3792002	12522052	2696545	5272640	3324457	6624765	10680561	4696836	9263756	7167185	2634721	4713979
EIC	Negative	278.1843	3.46	unknown	0	207118	0	0	0	237682	0	0	0	0	179163	0	0	0	0	0	0	219622	0
EIC	Negative	321.1743	3.45	unknown	2597461	0	0	0	4030478	0	0	0	0	0	0	0	0	124707	1880615	210805	189315	0	263402
EIC	Negative	240.1604	2.91	unknown	0	0	0	0	0	782240	0	0	0	0	345885	0	0	0	440864	0	0	0	0
EIC	Negative	325.1946	4.65	unknown	0	0	0	0	0	0	0	0	273995	314494	0	0	0	0	0	183778	0	0	0
EIC	Negative	555.3682	4.17	unknown	0	0	0	0	0	0	691106	0	0	389098	72426	0	647625	664396	548083	0	0	0	0
EIC	Negative	555.3682	4.24	unknown	0	0	0	0	0	0	406377	0	0	263355	0	0	338170	336639	343094	0	0	0	0
EIC	Negative	609.3879	4.04	unknown	0	0	0	0	0	0	0	0	108465	266054	0	0	21983	0	101150	0	0	0	0
EIC	Negative	415.2113	6.04	unknown	0	0	0	0	0	440666	0	0	0	648175	44703	0	639418	46038	49134	0	0	0	380821
EIC	Negative	229.0538	1.91	unknown	0	345241	0	0	302212	208546	326385	0	351235	0	441292	356165	319026	365948	264684	362509	274330	0	218886
fpHILIC	Positive	171.0760	1.60	unknown	134457	270519	920691	0	1484855	0	243027	0	2694365	1087995	0	0	1918735	1606922	0	0	1375496	962429	1328720
fpHILIC	Positive	204.1336	2.61	unknown	941148	855701	668396	883022	995093	2081116	966128	784396	1262859	1030893	1608998	1360256	1362107	1203858	831403	1379858	1616160	638380	1373722
EIC	Negative	284.1054	0.57	unknown	0	0	51998	65983	21161	9294	26255	15696	136911	96414	12625	0	47397	0	0	51096	0	28869	0
EIC	Negative	249.0227	1.93	unknown	471718	565250	205490	0	779517	677216	792799	0	639264	248560	1121272	1127629	1112988	1152686	289974	632430	1037422	0	768648
fpHILIC	Negative	195.0511	3.32	unknown	4339781	1775544	1986010	3082518	3036166	3716336	20836118	2179841	2715209	2448265	2316080	2871049	2125110	2334508	3252758	4067053	2496347	6438788	3423860
fpHILIC	Positive	531.2722	0.39	unknown	146604	210891	126219	132054	274825	289327	214435	186344	1313291	115458	200366	193355	309774	365705	154224	177333	201319	226070	198723
EIC	Negative	441.2529	5.71	unknown	723817	332775	404432	722813	763011	336482	1086944	441339	775748	468791	325575	646467	363280	277601	211770	537352	636010	615289	403210
EIC	Negative	429.2425	2.25	unknown	242137	55769	298196	0	243183	40827	173203	999216	153426	0	0	0	0	6029	8250	0	0	35121	7859
fpHILIC	Positive	502.2931	0.50	unknown	5594747	8644411	4747069	4688943	7644544	8911328	8476384	5385861	6315157	3995127	6694372	6117899	5307561	7523691	6912797	4222385	8629168	4090243	6849437
fpHILIC	Positive	370.0735	4.30	unknown	144699	339768	99374	155562	320324	551560	412499	116114	349053	129068	353642	289379	399499	238452	298009	371141	305256	103605	248372
fpHILIC	Negative	425.0807	4.29	unknown	1203467	1751933	422772	812928	1710296	2582184	1702815	630996	1728779	662684	1518697	1805983	1667072	1524961	1833179	1702256	1797024	663885	1719548
EIC	Negative	228.1601	2.25	unknown	109524	56859	285436	22597	173977	252366	99726	194887	195344	140316	57645	36812	56484	44366	129621	229409	41957	185470	164939
EIC	Negative	499.3105	3.17	unknown	0	122316	0	0	328367	396956	212532	0	330233	0	112046	53755	59060	164578	98918	50508	79510	0	81463
EIC	Negative	301.1447	3.07	unknown	182212	16082	35200	64898	96611	87782	109712	157782	63180	15278	35423	64440	35115	0	53963	13730	15434	167694	14280
EIC	Negative	393.2654	2.25	unknown	7742889	1234086	10443853	444835	8443174	671830	5691181	32890240	5137031	191494	0	33564	238064	9920	125034	169628	58103	2114227	463888
EIC	Negative	394.2686	2.25	unknown	1986414	320415	2604915	132271	2152014	168362	1430348	8367936	1304639	92668	0	0	66473	39909	35782	28953	6522	592282	125333
EIC	Negative	348.1438	2.17	unknown	0	109998	0	0	66594	33264	62878	0	335657	0	29894	67904	40031	59189	22172	194180	56051	0	61693
EIC	Negative	274.1487	2.58	unknown	0	53489	0	104588	80406	68688	101359	0	154630	48974	109114	67817	102896	14152	66776	106752	102018	57268	33423
EIC	Negative	399.2659	3.31	unknown	382988	477257	414716	404908	540710	464091	425100	415673	437097	380140	461858	447462	456885	451470	478878	417827	448934	415022	478506
EIC	Negative	343.1403	1.56	unknown	46385	64497	129122	0	88105	40642	99950	80080	97402	0	207099	122920	91395	280898	53703	101922	39966	0	76359
EIC	Negative	225.1857	5.57	unknown	1708855	2704935	7361372	12763017	7317002	3360859	5550152	9211488	12103748	3940391	2004672	3524821	5412110	1222826	866672	4233696	4496369	6331097	1919855
fpHILIC	Positive	263.0127	3.87	unknown	106296	384931	55147	90163	223058	348322	369142	130142	614194	89142	386056	328340	469274	335169	542893	345890	303999	121935	325043
EIC	Negative	398.8930	0.66	unknown	6052	43867	47963	43674	33491	23275	42029	91566	51162	24350	36715	39220	56254	0	26298	30062	54500	65044	21391
fpHILIC	Positive	434.2442	0.41	unknown	1634400	1764569	1667612	1697313	2186686	1860353	1746397	1578393	2234952	1968609	1493549	1546765	1959681	1603244	1121956	1528282	1568555	1850343	1301886
EIC	Negative	289.1448	2.97	unknown	71016	53771	104844	90801	127222	78295	107755	169001	30366	52819	58456	52594	43958	24517	48709	45177	49506	113359	37767
EIC	Negative	385.1870	1.82	unknown	507874	406374	346627	0	396733	334842	369450	536914	809197	309827	243860	199342	365117	86057	201365	619622	335298	0	113414
EIC	Negative	379.9847	3.76	unknown	85489	43090	73863	112435	50505	112885	141566	501726	17871	46626	16398	124949	0	0	7854	108892	85276	33251	82808
fpHILIC	Negative	605.3279	0.57	unknown	8231463	8476320	6722785	6702363	8209030	10012406	9926159	7824604	7432355	6016401	7690282	8525099	8543288	7020143	7403769	7635689	8752851	5679890	8499203
EIC	Negative	356.0319	2.09	unknown	175147	217562	178153	48705	218783	220318	222429	221941	215507	125591	217516	0	107049	164538	170010	253221	72868	62377	173081
EIC	Negative	284.2455	6.41	unknown	886829	1489888	1274436	1966212	1621396	1377223	1782260	1570514	1648943	1220390	1140533	1324294	1519352	765635	994530	1329028	1480369	1188024	1049320

EIC	Negative	328.2820	4.45	unknown	2264003	2161904	2058505	2262709	2275172	2181542	2167777	2227375	2178363	2164088	2254487	2291895	2223291	1907753	2066365	2190814	2136381	2365425	2018738
fpHILIC	Negative	361.1251	3.34	unknown	782067	675652	831387	948227	794417	919268	920736	841116	866218	844425	922949	712881	913164	445338	717910	606519	881518	700901	615532
fpHILIC	Positive	141.0656	1.84	unknown	10848311	5165684	5810342	7830783	8280533	13117774	8818261	7997405	7188232	6809500	6786098	6772473	5939592	4882633	7440140	11650471	6532104	7197580	5468628
EIC	Negative	489.2834	2.13	unknown	2742446	5060488	2297304	3379176	4673415	4347905	4919498	2982642	3657036	1309567	114029	120658	241655	24286	28791	285373	28514	941109	146166
fpHILIC	Positive	314.1231	3.89	unknown	670574	52051	0	0	285848	1115191	258819	242879	0	0	0	40811	0	258223	0	62946	0	322667	0
fpHILIC	Negative	344.1535	0.37	unknown	754729	996053	546709	1668259	626789	1052121	1003221	717997	2439471	561956	997742	1360703	1359591	717729	1175321	2320701	788694	423777	645220
EIC	Negative	581.3655	5.40	unknown	51853750	52855053	53320517	49250268	53259488	58724173	54429519	54314852	50538363	47115723	55686310	56762243	54735441	52046034	53478183	50830622	54877312	50721316	57129381
fpHILIC	Negative	611.1453	4.51	unknown	559387	244321	125375	270778	462671	735830	308046	238411	410469	217687	211845	390772	275834	351162	323920	381178	472528	362014	489241
EIC	Negative	255.1138	1.46	unknown	0	395289	0	0	345076	355019	360056	0	303929	0	373220	420038	361902	195596	250254	431733	283356	0	242412
EIC	Negative	315.2282	1.61	unknown	474496	405246	139353	0	390498	355776	622793	260581	395290	267966	364904	346399	442021	353235	369370	442593	368196	110848	603524
fpHILIC	Positive	173.0206	3.86	unknown	934874	997581	624468	686452	960144	1486161	874650	470991	744088	493448	685727	689335	636806	571049	754836	998917	850850	703636	838853
EIC	Negative	226.1891	5.57	unknown	450663	695604	1841256	3054334	1732640	862137	1385671	2199806	2957908	924264	478878	837817	1349869	316589	200881	1028260	1113769	1532239	459056
EIC	Negative	370.1685	2.21	unknown	67679	148148	64348	25505	121324	132034	118041	66610	118169	42321	107421	101354	107856	93172	134354	165459	106298	47087	93017
EIC	Negative	415.1975	1.45	unknown	0	520137	0	0	412205	563670	335536	0	524522	0	345409	314109	452902	121077	257662	645503	344739	0	186582
EIC	Negative	612.3316	4.26	unknown	324638	609198	310116	364579	468223	573059	449647	401545	551379	394023	597667	473553	484278	466373	454523	559991	585210	329174	481322
EIC	Negative	327.2181	2.10	unknown	152418	68899	113919	62314	53991	110531	1305398	57287	99148	53153	50946	93104	86043	69787	52203	264368	82007	60671	74961
EIC	Negative	333.1314	3.23	unknown	76376	163310	124128	276388	233179	131098	203656	164089	265881	103350	116940	193759	154911	99193	58493	128518	149058	164126	129183
EIC	Negative	487.1900	1.43	unknown	0	10451	0	0	99905	101334	46217	0	80251	0	24181	24752	78727	67673	81308	42664	41082	0	69574
fpHILIC	Negative	229.0538	0.37	unknown	633482	1044695	646910	216966	596009	693002	947002	673241	1063745	542555	936371	845708	667074	1135248	683040	730178	774736	598327	572651
EIC	Negative	552.2736	2.62	unknown	0	450140	0	0	402638	50287	45944	305334	401967	0	78428	385866	492024	409172	496620	81287	516017	0	84955
fpHILIC	Positive	711.0675	2.94	unknown	4659810	4794773	4649117	4850314	4854071	4844978	5135498	4540428	5479438	5035383	5092741	5431904	5364586	4473423	4752016	5226715	4688497	4784358	4905318
fpHILIC	Negative	82.9542	0.95	unknown	3190927	5884038	9807159	13411159	12332045	72513504	7751550	6625725	10283822	21767332	10717677	35815624	70673536	36444096	9588975	13851277	11477009	25417052	16854372
fpHILIC	Positive	427.0948	4.29	unknown	1191189	1612359	477766	863345	1692050	2275476	1790083	664279	1869072	774075	1440845	1880609	1666582	1707561	1858801	1771089	1710037	773106	1558291
EIC	Negative	552.5000	6.35	unknown	419006	453461	443639	623111	512088	584611	630543	566692	456089	213055	236034	340359	334999	174292	224609	288150	295605	287340	267416
EIC	Negative	227.1285	1.20	unknown	97272	99590	116953	135425	105773	82837	134996	130715	137727	87548	71029	110793	125749	64883	74401	86153	101996	130492	100013
EIC	Negative	462.9630	3.75	unknown	946419	1344295	842851	1075622	1178596	1350878	1178712	930952	1199561	771247	1187305	979836	1065588	1008930	1205746	1200190	1018042	936937	920342
fpHILIC	Negative	102.0561	2.58	unknown	872482	398869	820329	1058869	827304	985553	772092	714349	924309	965880	629290	584261	623782	585661	646963	656864	896219	813286	539480
fpHILIC	Negative	164.0644	2.45	unknown	666041	871046	530418	324888	1012178	958312	1209538	501028	1191532	496026	1181932	1182918	1172634	929676	923582	1001644	989708	651288	1042632
EIC	Negative	648.3173	1.94	unknown	0	183974	0	0	202511	222556	267615	0	181276	0	173324	242727	143745	162991	182594	191422	205664	0	221711
fpHILIC	Positive	373.1799	3.72	unknown	4276864	3604983	3932783	3565204	4314885	4266759	4519786	3657245	3109041	3275847	3552808	3542490	3173350	2540342	4038480	3803150	3150555	3639507	2897155
EIC	Negative	499.9343	4.20	unknown	403012	529427	359084	431086	490870	495029	472028	379589	482789	340358	446849	412270	439030	470987	446463	461647	414057	364897	387614
EIC	Negative	243.1603	2.96	unknown	231287	217858	394743	329754	215965	252820	359658	549575	335994	378674	165115	355932	336670	158016	164093	248185	272637	350153	210397
EIC	Negative	317.1635	1.87	unknown	171895	86285	0	0	112018	166458	82283	0	432159	0	118909	132232	171855	130139	142156	272512	101666	0	57738
EIC	Negative	256.1637	3.03	unknown	78781	87418	100448	76343	57482	68350	113805	169529	108173	115617	53716	93134	78609	55502	45285	60396	75129	97534	63772
fpHILIC	Negative	84.9513	0.95	unknown	1274905	2134074	3539754	4505086	4287086	25684282	2731812	2204958	3462504	7490303	3596014	12904656	25296660	12902903	3240653	4895754	3901222	8892982	5885043
EIC	Negative	461.2902	2.12	unknown	55654	24456	68001	102086	59617	70951	24463	155487	96624	97909	31759	42646	49912	92938	62453	29834	43611	152843	37793
fpHILIC	Negative	177.0411	0.95	unknown	318800	284612	260858	431251	362494	338046	398567	245500	445038	367706	403742	315697	263473	261094	384164	506872	291883	261697	241933
fpHILIC	Positive	104.0709	2.57	unknown	59679132	34281272	52371348	81902936	59924872	79993992	57030504	58425608	62400652	48814148	34315392	37925816	36201380	30238928	34569156	44572024	39291648	43208116	44657628
EIC	Negative	594.9786	0.55	unknown	54780	160334	183757	146461	131499	77393	169723	324586	174983	54120	176926	183384	179144	0	122119	119972	223360	226514	54120
fpHILIC	Positive	105.0740	2.57	unknown	2712785	1481819	2456009	3695168	2834150	3793448	2762434	2582275	2671824	2307060	1575189	1768593	1504330	1374602	1519373	1938941	1884491	1942344	2042516
EIC	Negative	475.2672	2.25	unknown	370793	61314	452332	0	322183	22101	199030	1855695	227797	0	0	0	0	0	0	0	0	177299	26998
EIC	Negative	353.2314	4.39	unknown	132334	60800	412832	329496	270818	157499	239467	515163	356414	242632	95225	165349	218793	91362	47887	187135	178046	315945	109584
EIC	Negative	382.9622	0.57	unknown	14158	0	38618	68619	10167	13172	19652	22533	176383	87682	14708	9208	48049	0	7934	62571	0	23008	15527
EIC	Negative	567.3174	2.81	unknown	1246740	575339	1213340	453738	1540801	705933	1622879	775717	231884	265758	104114	47193	86875	0	14960	33459	16551	92729	206468
EIC	Negative	379.2478	6.20	unknown	272753	249957	93288	83701	155225	221015	166577	501904	157612	408925	144028	242070	208021	187156	194256	183463	177005	123858	237393
EIC	Negative	324.1876	4.08	unknown	626104	552977	711801	1309782	893890	713023	948856	859510	1181117	1054408	829652	1046924	980919	320720	479266	928315	936094	1131983	392819
EIC	Negative	374.0133	0.55	unknown	53491	125246	133341	176462	118100	86898	151042	263714	136627	87937	129661	144271	143013	67561	123796	98760	161736	183353	69565
EIC	Negative	225.1857	3.47	unknown	218855	217451	253755	546019	299365	377140	347401	314967	486285	238555	271275	314890	287559	104031	178030	302572	194945	230005	159892
fpHILIC	Positive	142.0690	1.84	unknown	703144	325540	384614	461241	516728	889946	530897	489802	446853	442485	395417	421829	357347	303143	475536	719685	397165	457614	368070
EIC	Negative	560.3313	6.11	unknown	955052	1078475	965335	966548	1008677	1024732	1090444	1029080	953587	942855	1040021	1092657	1014193	1024868	943721	991346	1065867	951419	1030659
EIC	Negative	348.9661	0.55	unknown	189308	361194	457411	455287	338732	252913	419701	675848	476981	334715	397880	373738	389918	139241	348490	329911	464209	571889	262049
EIC	Negative	540.2734	3.31	unknown	203528	206268	73746	133263	155131	257264	120111	152627	329054	152260	181388	201388	139756	114389	189563	231619	253432	64790	241856
fpHILIC	Positive	241.0306	3.87	unknown	594505	1892337	346526	503998	1245461	1983309	2102219	518720	2938206	423883	1955153	1555640	2560328	1714855	2525364	1673351	1721027	509378	1642317
EIC	Negative	393.2656	1.82	unknown	20073554	4450813	6354147	0	13022954	23604879	14083466	3589210	2675083	19007	213731	169731	317783	339710	313650	239306	284921	0	596792
fpHILIC	Positive	576.1922	3.54	unknown	581108	1081682	0	857412	1034356	940763	1427050	1242928	0	661240	482154	837341	995671	0	868606	636638	1204914	1016807	358031
fpHILIC	Negative	230.9969	4.20	unknown	1687759	821113	522366	285591	1544123	3485716	1215775	288860	652681	132306	197779	386261	178805	1110330	218139	362445	110776	498859	144610
EIC	Negative	337.2327	3.90	unknown	0	283178	0	0	268818	367817	0	239913	0	397291	237250	0	305075	196462	0	374323	270529	0	204249
EIC	Negative	359.2664	2.01	unknown	0	640500	130874	0	647244	655772	685488	0	504028	0	598369	619208	492530	445560	587282	629687	615535	0	584132
fpHILIC	Negative	165.0555	1.66	unknown	218254	580813	0	584599	0	4028801	1555162	0	0	406330	680358	705742	0	84193	602611	513376	0	0	327136
EIC	Negative	375.2598	1.81	unknown	177559	196381	190287	0	179993	180160	195189	157266	194136	98624	157916	181314	171834	182027	181704	172398	182090	0	188405
EIC	Negative	539.2445	1.27	unknown	104273	107790	0	17041	113764	149493	184491	0	90865	156042	95862	135943	125588	83452	80045	115664	117826	0	111388
EIC	Negative	394.2684	1.82	unknown	5134403	1157940	1638187	0	3301062	5989393	3559342	886439	812072	0	71016	39204	92377	86314	84434	69744	70299	0	145916
fpHILIC	Positive	300.1556	3.48	unknown	429045	399185	447954	581976	564055	400853	667580	528302	437659	544946	528761	596918	569910	362572	471054	647167	440452	434569	357696
EIC	Negative	428.3020	3.48	unknown	551831	469907	566836	1269114	676819	829811	775113	713944	1140632	526090	577626	704607	682021	218064	380271	750221	418677	502529	351267
EIC	Negative	440.2665	2.01	unknown	0	317432	0	0	338070	310115	335174	0	284884	0	273147	262596	226421	201951	362273	270716	259116	0	298153
fpHILIC	Positive	305.1454	3.87	unknown	624755	254365	409828	497627	521326	664569	625710	459489	448477	413844	444776	241305	400239	239359	307332	140667	443671	215244	212697
EIC	Negative	298.9881	1.19	unknown	122744	126922	114443	89704	121812	145322	133393	99838	114016	116286	111728	121494	98856	108523	109096	106643	114384	90673	126311
fpHILIC	Negative	131.0713	0.54	unknown	7861265	4292202	3678281	3501111	6323131	13807962	5062526	2432177	5328061	3907140	3678496	3754047	3369757	2700785	5149789	4759752	3397245	3019252	3149080
fpHILIC	Negative	368.0589	4.30	unknown	149193	388480	82697	165556	330164	637908	420167	106649	397492	130288	367714	324580	485899	238047	343076	391145	337741	97824	297297
EIC	Negative	374.2571	2.19	unknown	28147	102456	0	199044	118588	114298	122779	43037	206557	99300	81950	130732	16040	56761	0	117194	70458	0	71854
EIC	Negative	581.3655	5.17	unknown	23734671	30607175	25883579	23411997	27618426	33628750	33784549	26824140	24542635	21654306	31720702	31645571	28862380	30798117	31379962	28433028	30653066	23364689	30120065
EIC	Negative	384.2756	6.41	unknown	1558426	2250592	2512027	2726959	2702620	2533905	2732150	2665665	2243695	2087027	2498250	1216615	2509777	2503375	1877454	2401197	2549299	2051781	1858571
EIC	Negative	628.3531	4.26	unknown	5866938	11698515	6380456	8542777	9964636	11243171	10583005	7494732	10097704	7263270	11102929	10341152	10262852	9193889	9427757	11129151	10216821	6757633	10538224
EIC	Negative	379.9932	1.19	unknown	145231	169308	163323	143470	154229	167777	155168	157658	160515	148990	160704	148776	153154	143798	162197	150337	153473	138137	141625
EIC	Negative	603.3421	3.90	unknown	116547	512063	174166	195817	393166	256767	300872	225718	568568	179228	240824	199561	336610	487650	228437	340470	336047	172780	292301
EIC	Negative	439.2616	2.01	unknown	0	987753	0	0	1062490	958223	1033880	0	610495	0	817826	840747	554032	452815	755785	784990	790977	0	787443
EIC	Negative	271.1920	2.36	unknown	74855	57438	64198	0	43218	197622	49152	60133	19356	87830	58536	76209	44547	34632	46506	51334	63765	32814	56702
EIC	Negative	431.3176	4.17	unknown	373320	289627	448133	456229	379297	466811	410750	629272	323088	457913	352659	296934	311825	351709	368609	371679	308722	587331	261697
EIC	Negative	629.4634	6.39	unknown	0	425258	180861	570134	405637	265790	490445	215425	549168	93283	150974	184109	270676	0	124472	209478	220598	161825	130048

EIC	Negative	602.3467	4.14	unknown	193096	534655	294220	260761	389216	444047	432444	360702	461220	255086	475466	339401	405774	517827	335628	489640	409552	223530	356693
EIC	Negative	227.1066	1.56	unknown	63481	121492	56077	0	126258	139629	141305	48811	0	47650	118714	107955	100449	80806	143746	71243	100857	0	126417
EIC	Negative	454.2901	1.82	unknown	4033873	887470	1622645	0	2722819	4800875	2873631	776939	656267	0	38013	40743	76578	63208	65223	48965	33578	0	122193
EIC	Negative	360.0064	1.19	unknown	166543	171876	139349	129530	159935	184677	187899	141684	144992	145724	169966	161028	140463	157832	158285	139997	160915	126150	166951
EIC	Negative	600.3314	3.90	unknown	10299395	36786816	11115332	13623679	28152415	18869326	23773838	15680550	43890719	11237700	19306115	13236980	25918955	37259168	16433892	26583596	26100522	11984872	18793115
EIC	Negative	357.2589	2.01	unknown	5052043	12557884	4643284	3676245	12713129	12517393	12943660	3973445	9820778	4174520	11950117	11812527	10170425	9325685	11530209	11781635	11561091	3587872	11524728
EIC	Negative	496.3049	5.57	unknown	1600706	1500709	1585424	1424082	1607282	1665964	1490057	1959071	1335723	1425155	1296587	999372	1114806	1182406	1194055	1086645	1425804	1527697	1305472
fpHILIC	Negative	511.4732	0.38	unknown	268878	798059	1294969	2323192	1088091	884657	1380061	1558978	1781079	642844	319848	723987	1026075	285275	268956	654052	1092669	1082934	415175
EIC	Negative	420.9404	0.67	unknown	11766	59039	55718	55284	44786	29097	52160	114107	54065	32951	46321	61492	66229	5069	38063	43577	66970	76034	24615
EIC	Negative	356.2472	2.06	unknown	2793733	3791437	2559609	2235857	3657704	3698427	3738355	2456239	3369418	2509254	3493033	3581914	3391603	3286313	3451518	3588392	3448338	2044934	3496574
EIC	Negative	635.3419	2.65	unknown	0	75463	0	0	77959	81459	135667	0	72764	0	68559	80812	80141	77262	53136	82762	101535	0	120389
EIC	Negative	540.3315	5.10	unknown	862636	1008367	896130	889821	960611	981583	959340	915933	904281	923543	957049	993213	916835	965588	946876	923252	901619	898081	936641
fpHILIC	Negative	144.0666	0.95	unknown	2038691	1462775	1487917	7173666	2654207	4517630	3493536	2154787	6275974	2236695	2578878	2873847	3370614	878705	1696735	5079379	2451859	1172695	901628
EIC	Negative	581.3572	4.35	unknown	1542826	416518	1714937	1487986	1518091	1688697	1632695	1663069	1659003	1590500	1817697	1604589	1598117	1902802	1499710	1527847	1635430	1481282	1871740
fpHILIC	Negative	101.0608	0.49	unknown	666174	543475	439593	583127	662028	847327	702515	357208	870394	628426	734715	976761	511829	329086	928107	720303	454701	336731	426730
fpHILIC	Positive	267.0082	1.61	unknown	122278	288821	0	451863	782503	441296	533462	0	737643	0	327425	562038	661310	710749	560339	519294	0	673090	0
EIC	Negative	535.2500	1.30	unknown	176729	98689	0	0	176744	171579	292456	0	150769	249664	150851	125949	53156	98536	125528	114376	154722	0	97569
EIC	Negative	338.1743	4.08	unknown	89182	65486	102668	270248	132179	112703	168436	149043	215562	162849	133597	179919	167819	7629	0	152583	136406	183909	0
EIC	Negative	298.1723	3.38	unknown	77605	131078	156937	423202	247835	160615	232955	221807	307514	166541	129279	254108	185155	73033	61489	130986	138561	303560	84446
EIC	Negative	249.0380	0.89	unknown	75944	93689	29642	26902	130217	59912	98171	67534	204555	32289	63829	5580	70148	73964	77028	170882	37813	54760	147247
EIC	Negative	325.2658	4.73	unknown	561210	591779	493203	638334	599729	596478	560120	566766	574524	558684	580763	531958	536072	511846	550500	582076	526356	555564	546466
EIC	Negative	437.2868	5.74	unknown	1166510	1392593	1152380	1238128	1365567	1651062	1058181	1214800	1287702	921592	1096088	1062305	1083251	1249549	960043	1009252	1205142	1032306	1233438
fpHILIC	Positive	764.5218	0.38	unknown	346781	359035	269986	301127	388279	201850	243317	434390	527816	260126	210524	241988	213674	507802	209717	170254	267488	229190	200609
EIC	Negative	601.3347	3.90	unknown	3536528	12347248	3744896	4678882	9548784	6281375	7972347	5199631	14634124	3737860	6437402	4412593	8640206	12529423	5515038	8970729	8805138	4040625	6405299
EIC	Negative	552.3320	2.72	unknown	577033	0	0	0	337928	0	438952	0	281993	0	457159	306647	296516	0	0	0	285966	0	0
EIC	Negative	328.2932	6.65	unknown	158617	203671	233384	477960	273290	360382	302648	420960	265550	246275	249836	387084	385040	272195	289850	256978	276220	320580	159808
EIC	Negative	498.9309	4.20	unknown	4129565	5522543	3790409	4708942	5107177	5404461	4756296	3876262	5043648	3652856	4933178	4566893	5052048	4806941	4948635	4830183	4368435	3777879	4096476
EIC	Negative	554.2740	1.77	unknown	809318	795605	745976	493145	551321	789081	1070137	1095786	619656	1177988	793055	1011015	723184	868985	491407	558112	631741	0	1020510
fpHILIC	Negative	327.2330	0.37	unknown	32716214	73663672	40384904	107444320	65692220	50759880	72651832	54733804	107106344	51203184	42856004	46553164	73541752	28933730	43375664	64539172	61572868	53455152	43472300
fpHILIC	Positive	156.0420	2.30	unknown	0	564953	557269	652568	606334	628207	548851	802720	240189	513560	453806	439732	465547	365669	548662	243853	130447	516955	179825
EIC	Negative	420.1147	1.67	unknown	0	91764	0	96738	87160	109965	88556	0	142759	40237	88896	75031	125736	17093	69499	95094	68522	62822	39596
EIC	Negative	363.2540	3.11	unknown	420065	95211	197688	27867	278074	106902	556948	79543	407798	17748	21949	18825	24804	23492	22532	13348	17994	17399	26785
fpHILIC	Negative	155.0098	1.97	unknown	1914468	1604034	1676728	2023921	2038056	2902587	2554059	2016012	1282826	1866582	1783512	1838974	1676489	1899891	2063912	1666795	2282886	2040576	1839645
EIC	Negative	630.3782	4.27	unknown	0	0	0	0	0	0	246355	184175	0	0	0	0	0	0	0	0	0	174982	220815
fpHILIC	Positive	215.1390	1.66	unknown	0	259436	204916	406882	226245	574410	297621	159051	244755	317016	301133	340206	199444	198926	256112	341248	225436	193461	167681
fpHILIC	Negative	289.0698	2.77	unknown	278918	134209	160904	238940	299043	749101	215165	125314	100105	248586	295269	403522	121625	0	190513	146737	175410	191286	97761
fpHILIC	Negative	130.0590	0.49	unknown	21108112	16502648	16286704	16856402	20212794	24157010	22492532	10863980	25279060	19086146	20322368	24014904	15641117	10733741	25054636	17858858	12921660	11761450	12188534
EIC	Negative	297.1688	3.38	unknown	523210	867835	1100935	2671085	1628236	997515	1412932	1534321	1955034	1096139	793576	1352498	1142253	465218	373235	912797	955651	1962504	517008
fpHILIC	Positive	127.0500	2.74	unknown	1476074	945387	1127197	1399874	992050	1677308	1236365	1880755	1371388	1241210	893881	1294636	1115399	1196435	835098	1565417	1438289	1517482	1048038
EIC	Negative	476.2718	1.82	unknown	692953	208024	230978	0	469759	744730	543095	34899	133042	0	6739	0	16025	7098	15612	9844	16207	0	35403
EIC	Negative	516.2472	1.03	unknown	0	105455	0	0	69494	101367	139818	0	70721	89238	95271	128960	92043	95251	79219	71048	81699	0	108917
fpHILIC	Negative	192.0666	0.61	unknown	17130956	20580484	9008398	6725455	24873676	51069000	21091468	9284894	11339575	1396118	3978957	3820710	2383723	3574037	2858575	4592358	3897922	1852020	3351004
EIC	Negative	251.1269	3.23	unknown	377956	581093	750216	1262746	959742	559323	867818	736534	1117590	596400	495122	821916	712471	430276	351193	572606	706390	742279	541290
EIC	Negative	381.1648	3.46	unknown	187038	64750	144086	91322	118616	130858	162825	273644	59975	167450	70641	102728	88645	83178	102569	104799	104801	221087	63101
EIC	Negative	602.3382	3.90	unknown	804096	2858097	879758	1022739	2129067	1452834	1777923	1231952	3287505	926276	1604663	1059522	1997369	2791377	1342976	2077322	2054345	1035913	1439404
EIC	Negative	540.2733	3.10	unknown	485054	483838	263908	372672	458975	586661	388188	324652	813360	356850	355807	468015	385000	330485	517332	511814	562810	124906	524172
EIC	Negative	283.1030	0.57	unknown	145736	74489	308605	557961	156416	131193	192198	183864	1185292	775811	161249	133021	304171	57129	72367	369732	100741	285731	162144
EIC	Negative	580.3623	5.17	unknown	75991180	97449969	81619353	73764012	87580101	106620533	105893560	84366132	78079349	67927230	99042912	99250348	91175401	96785742	98787692	89224813	96645968	72919833	94974114
fpHILIC	Positive	115.0636	1.80	unknown	1747090	1407393	1418091	1674235	1599030	2088228	1814173	1405467	1802035	1520780	1604736	1692142	1775355	1303612	1456363	1883423	1428239	1461273	1344801
EIC	Negative	502.2845	4.64	unknown	8837653	11315426	8381734	8736319	10827912	11007438	10411469	9014315	9350964	6763128	8781395	8239099	8142273	10266623	9331236	6154898	9829852	8104803	9894973
EIC	Negative	316.1673	0.76	unknown	83837	231546	75511	54573	174878	205033	199227	163973	100749	115386	294689	238262	299673	230366	272863	97314	90365	47241	134829
EIC	Negative	540.2732	3.20	unknown	267668	317900	151900	205284	242123	361802	211031	200985	477306	205582	201616	288863	212941	178386	265398	322612	339865	122742	311386
EIC	Negative	586.4939	6.50	unknown	421600	811658	992526	1463898	1015972	679005	1098782	1118840	1314637	685528	968900	723176	905031	612833	723697	724371	925119	854557	666396
fpHILIC	Positive	175.1440	4.34	unknown	4319927	3074654	2611725	4678087	3945482	4979705	3757306	2540183	4338689	3813484	3571681	3674980	3155570	2336082	3680980	4609964	3351260	2707816	2876934
EIC	Negative	430.3331	6.59	unknown	308786	215703	298748	279122	242639	357376	360780	518716	229412	386111	230193	243733	267753	240260	205204	301856	279059	563799	322686
fpHILIC	Positive	105.1106	4.07	unknown	15842534	11247876	11706324	10549075	11778864	14591261	13815348	11776338	11411388	10404877	7808865	10660041	8898124	7679478	11107107	12011185	9373973	11976732	10846486
EIC	Negative	383.1814	5.78	unknown	159169	0	0	0	0	171654	278943	0	0	0	0	0	0	0	0	20425	198344	0	118276
EIC	Negative	606.3694	4.80	unknown	1455644	3147461	2189780	1731939	2625771	3233433	2940183	1953430	2058903	1761407	3211897	2414512	2651048	2428828	2531059	2583214	2929922	1370362	2661519
fpHILIC	Positive	409.1718	3.72	unknown	771101	874422	904564	964617	1094169	872642	860487	1111244	813762	970171	975992	939915	896832	844835	992016	889116	851451	822925	753887
EIC	Negative	357.2517	2.06	unknown	493459	971424	476389	526397	830424	893010	898999	551858	657333	294232	833231	927637	817001	669960	745288	820761	699709	403709	764673
fpHILIC	Negative	129.0559	0.49	unknown	338040800	260871632	263259744	268070880	324825664	380490464	345036608	174886672	408426368	293130944	340309472	398039488	263546656	175517200	409676832	288492704	217432624	189838352	201977120
EIC	Negative	327.2181	2.28	unknown	337896	182046	365465	242026	211358	393586	369425	315698	519085	217344	207950	461547	405966	245529	118759	354885	383490	302197	393542
EIC	Negative	395.2713	1.82	unknown	816470	201963	349301	0	519951	958319	708968	33145	129239	0	0	0	9194	0	8817	0	0	0	21608
EIC	Negative	375.2749	6.24	unknown	835549	401416	576705	913424	706697	971011	610436	931826	696797	707377	454927	487562	450967	624500	562053	562274	466803	596660	800233
fpHILIC	Positive	304.1606	4.34	unknown	673155	523108	270550	389561	473308	890814	620878	309449	488957	294801	386435	407047	438479	363391	642478	472354	408632	284786	374037
EIC	Negative	358.2627	2.01	unknown	1545940	3807454	1363142	972619	3802297	3755051	3982266	892977	2944505	1228682	3659127	3606082	3093921	2801459	3464976	3555625	3526221	866035	3530003
fpHILIC	Negative	283.0684	2.93	unknown	480461	287088	279788	325920	390064	594742	379732	217743	415334	283536	248943	263251	255071	347820	342177	326788	465585	307897	267273
EIC	Negative	416.2010	1.87	unknown	174938	507648	0	0	366561	169587	425783	0	1059246	0	278410	255382	396651	298609	443590	741872	274660	0	195545
EIC	Negative	255.1604	3.03	unknown	505437	381642	773688	562891	370377	376707	686195	1068304	695178	742039	267034	560055	538811	289231	263844	373217	451315	634198	332282
EIC	Negative	381.2624	4.40	unknown	0	0	531906	48111	511355	407905	0	456140	431815	599101	40912	0	0	0	48682	0	0	558969	0
EIC	Negative	365.2067	6.41	unknown	7455779	7824569	7930316	6756290	7106777	6647490	9058796	9567282	7572562	5236904	5566481	6223843	5622695	4176441	5823246	5567595	5957333	4670650	4833685
EIC	Negative	242.1841	4.39	unknown	591537	132286	393383	556438	500123	786966	364899	508423	389900	324491	144221	213132	244317	126828	56631	106730	182219	430829	113069
EIC	Negative	489.2534	1.82	unknown	1068513	262983	427045	0	717281	1200316	754687	123507	152216	0	0	0	21637	0	23759	0	0	0	21821
EIC	Negative	354.1749	4.15	unknown	493530	610718	373209	1307264	1010378	810602	1373449	717515	793907	785123	806333	1031117	1198441	252321	706297	889484	774872	920349	403528
fpHILIC	Negative	291.0355	0.43	unknown	906858	1915074	895945	271602	2084637	3067452	1725306	606103	746689	368754	1389030	240657	172283	87566	631819	798199	193852	146656	160286
EIC	Negative	420.2443	2.73	unknown	0	0	624095	270445	126115	202113	123521	167824	1010116	94493	115496	84715	1259733	0	117231	87652	108186	87492	107588
EIC	Negative	409.2814	4.97	unknown	489918	529894	434455	496032	506000	466253	485292	554260	548132	531019	461082	450822	436244	450195	481749	498496	429060	471975	471848
EIC	Negative	243.1233	0.74	unknown	88598	121560	114889	92514	100923	96028	117017	137168	101300	85154	95661	99277	105754	85480	108553	103235	97628	112435	102904
EIC	Negative	498.2527	0.77	unknown	213120	274999	236860	225332	197978	276426	460093	263338	205963	208610	235843	423235	252011	200290	194219	295717	205495	336562	270355

EIC	Negative	328.2360	6.41	unknown	66443948	111560428	92281229	149884830	109969306	99632978	123468896	111028148	126869670	88049030	85779607	93168312	110545863	62591953	74818460	97712562	106570536	91240134	73916854
fpHILIC	Positive	233.1579	1.83	unknown	1345933	2256905	1709549	1677123	1745295	3095813	2370206	1568914	1582392	1070840	2136833	2065942	1860629	3151748	1556038	2257825	1695897	1151990	1615629
fpHILIC	Positive	190.1630	4.65	unknown	1079502	523766	408501	935699	870535	892660	789419	662088	1112411	611417	670595	808396	780733	731270	1029787	875605	846375	557618	744041
EIC	Negative	540.2736	3.44	unknown	211069	224657	80183	122827	177646	266551	155712	112080	349505	153964	138649	205092	137573	138397	199139	218148	257750	79396	241320
EIC	Negative	529.3010	4.17	unknown	0	0	81566	0	0	0	0	391317	0	365574	0	0	0	0	0	0	0	345794	0
EIC	Negative	608.9910	0.55	unknown	312023	1625031	1275242	1767896	1020568	857238	1415951	2787886	1463069	465676	1416646	1770434	1762296	57220	947916	1034572	1680902	1768146	399357
fpHILIC	Negative	277.2172	0.38	unknown	12885396	18894370	27203476	41722568	26918814	17876900	24131362	26718798	39995308	18517540	14645418	19787326	23569852	11557058	14177434	15589179	21949066	21848226	16567714
EIC	Negative	358.2080	5.81	unknown	116527	311260	186000	696781	388827	181094	269829	340510	660787	175690	202702	162239	314521	134509	140358	236376	356031	191329	106343
fpHILIC	Negative	239.0165	3.87	unknown	947505	2528010	473297	731700	1507339	2784722	2646857	623941	3484681	495143	2377188	1883571	3619751	1982680	3321791	1897944	2378304	678400	1953505
EIC	Negative	570.3687	5.50	unknown	286052	391821	279649	316670	359928	401384	343291	328330	296100	243458	348330	352359	329471	343004	314517	290450	318233	249698	352189
EIC	Negative	321.2061	2.88	unknown	406750	140031	128347	130418	218690	433958	303451	253300	103959	32762	37771	26825	26936	24778	33550	31630	32965	32525	27814
fpHILIC	Negative	628.3253	0.54	unknown	6855852	13574518	6247125	7124412	10887976	11446018	11086338	6612682	12953935	6938522	12529831	9889809	11320655	9039957	9499987	12551374	11994434	5430054	12199889
EIC	Negative	291.0368	2.17	unknown	52424	97671	126492	31296	68537	181334	119005	41223	24380	30857	235033	64129	0	0	84212	117707	0	0	0
EIC	Negative	388.8248	0.52	unknown	186787	117700	162441	151059	253329	143513	177440	151057	128797	188040	157191	119267	157976	370378	187658	112188	118662	147375	153664
fpHILIC	Negative	115.0402	0.44	unknown	2107383	3224022	5602420	5431533	2974323	3661563	5413852	4704476	4224624	5532155	4313629	4353094	4487072	2876362	2873756	3806124	4662326	3834824	3730652
EIC	Negative	315.1249	2.05	unknown	0	80683	83988	0	135309	118040	170059	0	13025	0	36069	126391	0	0	183342	112221	44851	0	90008
fpHILIC	Positive	729.5887	0.47	unknown	425370	470084	361749	376669	552283	348905	411124	319058	626799	442577	442745	345062	358863	501505	438841	461189	522826	416716	396942
EIC	Negative	271.2281	4.66	unknown	3598476	2972024	10692136	10551480	8108157	5196468	6417696	12152642	9454848	6228982	3330599	4966433	4404419	4193839	2037044	3855850	4748938	7609810	3889833
EIC	Negative	473.2831	6.83	unknown	95598	107435	99283	111502	94067	108153	148721	118914	81282	58771	87851	62946	74990	65768	23042	62709	26662	52300	29113
EIC	Negative	253.1446	1.92	unknown	0	283785	0	0	217801	123149	279356	0	379870	0	211603	175612	303036	172511	199573	269816	218973	0	140667
EIC	Negative	280.1197	3.22	unknown	61765	63652	118975	51205	134782	58082	71496	137847	50952	85098	53114	14289	9896	34545	148085	9137	46908	87420	32075
fpHILIC	Positive	104.1073	4.07	unknown	285827552	193999568	214999376	180053920	203876448	259605792	238174832	216200768	204526016	182276992	144960384	183762304	153304512	138076864	201599920	215368480	166120896	211435216	195510384
EIC	Negative	391.1513	0.74	unknown	174857	131289	88614	94049	131017	118826	132216	150434	145494	123551	108290	133159	120095	105847	138919	119360	129632	105595	123266
EIC	Negative	523.2661	4.03	unknown	114848	534604	121301	203406	361404	411645	306130	163735	437028	187740	378876	322564	388746	508075	369759	272712	378679	22285	370020
EIC	Negative	538.2425	1.27	unknown	371649	371695	241507	105141	397748	498853	647239	45742	326525	497942	311986	499104	430645	299289	255804	374899	371936	0	383117
EIC	Negative	357.2051	5.81	unknown	527571	1365528	857419	3050116	1492626	815253	1232093	1396851	2918604	892865	795287	687869	1489241	604807	726661	1094695	1418671	817965	517470
EIC	Negative	315.2328	6.39	unknown	1747166	6351818	2855933	6464028	4665169	4456841	5755302	2595237	15787898	3949779	4525350	2385556	5532704	3475289	2581644	4661508	4637287	2810698	3480185
EIC	Negative	415.1975	1.87	unknown	701856	2440865	0	0	1560410	731604	1945265	0	4586470	0	1215242	1070837	1775141	1402198	1936410	3365738	1223937	0	1003219
EIC	Negative	379.2482	3.12	unknown	141268	68975	160935	85447	82483	94311	121211	169075	175702	312619	90851	95178	98831	118977	92098	137507	101317	136336	103999
EIC	Negative	605.3660	4.80	unknown	6723319	14600435	10286573	7845575	12207987	14648471	13598401	9079758	9346243	8133968	15171725	11328466	12305378	11233473	11221435	12306022	13928386	6192010	11997372
EIC	Negative	381.2627	5.59	unknown	1190229	136199	456823	201813	753101	1037296	404462	582722	317164	185818	204832	162495	147611	201492	107656	127204	163595	259956	164188
fpHILIC	Positive	362.1192	2.08	unknown	593051	45244	201909	41586	297611	453535	230641	0	327565	0	0	0	279972	300494	0	0	346424	0	0
EIC	Negative	231.1758	2.65	unknown	172040	228269	273393	245329	286970	197965	268480	348108	152678	261322	229527	186830	155733	177472	168671	138950	152902	366729	327024
EIC	Negative	372.2401	1.87	unknown	133847	101810	0	0	131234	143036	129947	0	189991	0	121839	148659	127582	63762	58682	98673	96699	0	93823
EIC	Negative	285.2075	3.47	unknown	0	102385	280577	182968	47511	193917	243952	341981	73910	227943	117400	168872	60474	110696	135769	69646	141394	268960	75789
EIC	Negative	347.1870	2.44	unknown	584344	2090083	597294	883526	1179478	925309	2223605	1708344	1231203	891230	265827	2505859	2158591	1782644	2284116	2224883	1759130	288719	293194
EIC	Negative	399.1701	4.08	unknown	198644	177090	220053	478854	300722	248506	307746	316832	415510	396883	328016	387167	335173	98577	153983	328574	324809	493120	139163
EIC	Negative	310.0722	0.69	unknown	71472	5306	0	4064	0	11805	0	154765	73825	4147	15489	0	20289	74705	17510	31810	42933	24927	17031
EIC	Negative	307.0315	1.26	unknown	461536	729079	70394	35853	846478	1040800	666251	88626	228456	382904	550627	118317	51121	18429	311816	232860	42778	0	34982
fpHILIC	Positive	133.0730	2.45	unknown	554762	203135	584765	490164	337584	642005	200291	670821	1234712	472300	330369	730452	734674	746327	191005	465249	706464	412068	342070
EIC	Negative	374.2572	1.81	unknown	1034407	1075520	962694	0	957634	1037986	1084294	740073	951299	555871	979350	992596	1035985	973040	970480	1010170	1032182	0	976787
EIC	Negative	241.1808	4.39	unknown	3745139	830441	2427949	3456933	3255855	4199601	2188552	3331216	2342939	2041783	1046609	1309073	1347810	659119	443874	954289	1277762	2786624	710648
fpHILIC	Positive	639.3408	0.40	unknown	349096	391444	405992	375074	338969	452474	364962	393552	702350	373122	326751	345747	475867	486026	242918	336245	398244	338723	401018
EIC	Negative	296.1580	2.85	unknown	307760	190088	211035	301351	290276	237996	272123	250911	311801	311710	197169	298173	262671	125992	148142	210848	238859	406298	117611
EIC	Negative	320.1566	4.90	unknown	78061	91453	0	0	191311	93345	21645	0	183132	0	0	0	0	0	0	91493	0	0	0
fpHILIC	Negative	308.0982	3.20	unknown	652808	466331	532480	697371	650967	885741	645100	438788	615188	528460	639394	499291	501081	408719	533999	563581	545286	555651	610096
fpHILIC	Positive	259.1134	3.32	unknown	4899200	11222589	4979366	4387583	8001451	9896221	11001331	4850632	7966432	3544609	8611073	9931514	7432874	7259803	7361684	9877002	7881716	5285608	10226306
fpHILIC	Positive	544.3386	0.56	unknown	144401184	142307840	145467184	148229184	144076160	169291632	188346416	163894768	114308776	133892392	132433536	164431488	150639904	121547464	129724608	128941096	154946096	145421680	141910944
EIC	Negative	594.3777	5.75	unknown	369661	470646	378707	350200	446076	554978	549037	406072	376442	239131	407042	442263	402936	376176	437312	399595	392514	296520	423237
fpHILIC	Negative	174.0407	3.81	unknown	4546487	2969823	2657319	3221612	2662184	5887366	2071746	2103222	10517291	2415041	2567889	4680669	3403483	2709389	2267548	2826324	3176490	3161676	2472104
fpHILIC	Positive	545.3431	0.57	unknown	49702632	47365536	46192840	49215572	48202820	55683132	64181484	56230204	37044052	43518604	46278292	53256120	50322252	40765008	43186848	42793628	52168440	50072248	47072224
EIC	Negative	601.3237	3.03	unknown	556822	341668	218865	398243	338022	244893	636551	353612	542619	404558	266819	208824	246206	227970	235961	265878	797211	461305	209175
EIC	Negative	539.4322	6.51	unknown	642221	542217	779608	730389	699620	430286	937123	861250	617395	489509	472427	428973	448036	364647	407742	516914	469675	381050	452140
fpHILIC	Positive	259.0921	3.14	unknown	1133898	788497	734195	1061443	872057	1939940	1338044	953062	641279	938518	1011350	997536	901690	594756	1028530	1059666	873309	638135	754199
fpHILIC	Negative	116.0799	0.53	unknown	157748	367830	456349	543705	395708	576985	332326	368918	493646	455063	387802	432835	431200	594252	256053	400543	618851	196097	395855
fpHILIC	Negative	131.0826	3.71	unknown	6380872	5385574	5401931	4843568	5985982	6613244	5774110	4983482	4371732	4809633	4912920	4597782	4588258	3810968	5283311	5087681	4297673	5042478	4132427
EIC	Negative	304.2358	6.44	unknown	61629084	62479799	16282958	68442225	59207663	53669018	67476175	65787503	60737456	44030903	58546074	61026997	64821204	52465786	53485426	62718103	62045110	51822312	60164931
EIC	Negative	534.2450	1.30	unknown	672460	362603	0	0	515215	610822	1073059	0	559923	726812	542851	476762	137166	315104	518026	436395	583395	0	307660
EIC	Negative	383.2211	6.24	unknown	614813	1025830	642460	1445411	1025169	491266	923375	786038	2101497	774036	582257	507091	1084088	759331	596367	1348869	919292	707476	525400
EIC	Negative	631.3817	5.28	unknown	993322	1121001	961364	1014657	1246802	1682384	1526007	1055161	800126	832639	1438221	1312723	965609	795001	1178252	966685	1264787	835443	1165586
EIC	Negative	282.1781	5.18	unknown	252897	334995	353389	477134	355386	287639	310108	427506	472999	251221	278842	294335	348752	294714	211851	228617	271940	438888	225645
EIC	Negative	507.3338	3.38	unknown	315052	173451	324361	230619	292513	265525	245383	453046	170532	137091	118936	152025	133231	153687	206054	153826	130977	330645	147083
EIC	Negative	572.4724	6.35	unknown	1613546	1724067	2172448	1934185	1941024	1775348	2072265	1998140	1713221	1135187	1005782	1232642	1239326	1026040	694613	1198803	991076	1240390	1101287
EIC	Negative	389.2911	6.43	unknown	0	34703508	0	45834044	43112692	45549276	36073268	0	45022372	44029220	29528754	34153096	33038382	42381208	38333440	36841880	33061908	0	53675936
EIC	Negative	322.1722	5.79	unknown	0	0	0	0	0	0	387694	0	327207	0	0	0	274339	95643	0	192567	0	0	0
EIC	Negative	249.1130	2.10	unknown	262548	226680	201394	270467	220929	233577	224509	235591	313278	279458	216574	262715	272751	245001	223136	261377	279786	199434	184206
EIC	Negative	324.1796	1.85	unknown	850690	645781	0	0	655533	770793	1009103	0	591805	0	535354	584441	621942	503332	572966	947307	570721	0	733030
EIC	Negative	254.1481	2.36	unknown	143082	111491	144209	178179	117270	128998	167188	217756	138647	214147	104302	105562	101156	103025	107664	116904	119102	195821	138563
fpHILIC	Positive	972.2524	3.35	unknown	513459	401381	566726	573125	367299	657685	707458	494307	827916	410984	597735	667366	508066	764490	701249	157270	741136	335133	534718
EIC	Negative	430.1403	0.69	unknown	68914	53244	96565	51659	73870	73831	115375	64453	65121	107795	73472	57545	37104	56248	65313	63335	64645	37025	64481
EIC	Negative	388.0295	0.54	unknown	582381	1565839	1311982	1738872	1127402	963265	1394165	2515873	1378491	673350	1268068	1606308	1680190	634579	1023454	983984	1535228	1730727	584547
EIC	Negative	350.2471	3.22	unknown	107039	118388	107265	146504	113711	125878	113132	167532	117531	131197	114153	113856	120781	92260	100773	162289	107332	111204	118884
EIC	Negative	604.3629	4.80	unknown	20374659	43561643	30291955	23990873	36215692	43315607	40369351	27166154	28322102	24373466	44834939	33599113	36540097	33496094	33355204	36773078	41239630	18552423	36348775
fpHILIC	Positive	568.3383	0.55	unknown	45765904	74008000	44759352	61709880	57352444	66977368	59782988	51038692	69051648	56103844	65321916	62673888	71797232	53693444	49524052	61873900	66921368	49985352	71203976
EIC	Negative	228.2047	6.34	unknown	17702088	477777	36604516	50178216	26593544	23199766	28128822	41529508	36955340	22104340	14228451	23394400	21285470	12814467	160505	17271148	20500512	26119702	17257182
fpHILIC	Negative	116.0434	0.57	unknown	7422796	6467201	8791174	8459330	7002148	8394876	8848806	8616244	8175773	7892443	8415071	9664849	7228773	4453915	5621198	6526374	6115107	7012573	6085745
EIC	Negative	600.3314	3.99	unknown	1025877	2515799	1137151	1107616	1799444	1792044	1970812	1302112	2379288	892398	1746912	1494157	1927970	2153511	1399083	1761688	1779151	935179	1502184
EIC	Negative	271.2281	4.22	unknown	1869498	1683744	5278215	5045374	3850299	2784260	3092545	6127939	4372447	2631131	1678370	2039609	2201340	2474839	1105170	2050417	2433567	3853722	2073320
EIC	Negative	346.2601	3.52	unknown	201272	205553	168277	529265	263100	321544	428742	466932	333596	345645	226306	160392	232897	195587	197501	244940	241397	263956	348425

EIC	Negative	304.1173	1.81	unknown	85763	51953	0	0	64131	144221	71100	0	35764	0	55680	89227	77382	40467	86668	79076	92634	0	38878
fpHILIC	Negative	305.0681	4.52	unknown	452090	238038	121176	205175	308904	558818	242259	198256	280659	178929	149419	307654	0	239925	225544	283970	385435	264157	354632
fpHILIC	Positive	114.0988	1.80	unknown	486195	423762	398617	403928	360775	541417	459563	361367	468025	384128	415236	442732	449934	306575	336823	459282	390133	334711	320500
EIC	Negative	397.1551	3.43	unknown	159103	171402	197457	507408	277542	219963	274884	331640	396806	318553	281001	402340	328810	108649	151701	269630	269742	440039	138088
fpHILIC	Negative	310.1288	2.70	unknown	238550	262295	592104	164487	414588	275658	335000	421351	260142	312105	526849	369634	305562	432779	108528	277590	186660	175569	223477
EIC	Negative	327.2907	6.65	unknown	743919	822423	1111322	2243462	1218435	1601832	1347830	1607366	1278177	1104917	1116639	1667153	1703491	949688	985368	1165443	1242882	1354002	772797
EIC	Negative	500.2789	4.64	unknown	168351504	213584596	158801883	165319160	199871990	208721496	193056103	171062162	173252379	129589405	164398571	157099119	152766540	197209026	175662575	116630141	187964440	153552312	184786715
EIC	Negative	442.0091	1.19	unknown	123164	126380	107881	97030	122166	131958	126470	101141	113173	112587	122442	121215	118792	119266	115339	102664	117913	90869	127083
EIC	Negative	272.2315	4.66	unknown	605832	584911	1944406	1924343	1462117	917323	1050735	2254454	1739771	1095389	603854	938648	742561	692995	367452	668035	858140	1339821	623757
EIC	Negative	269.1402	2.34	unknown	373823	207239	43374	88596	266542	213055	244372	130039	337145	127101	153117	49813	219984	95401	108075	199520	40817	89482	66925
EIC	Negative	583.3811	5.40	unknown	0	0	0	1677999	0	1941029	0	1851288	0	1578695	0	0	0	1753281	0	1637309	0	0	0
fpHILIC	Positive	288.1915	2.29	unknown	541522	520765	447639	707175	652750	549538	522406	507350	569403	578660	400079	480352	676200	386746	231457	429252	467400	664984	434686
fpHILIC	Negative	841.5428	0.44	unknown	159392	325545	212724	140934	296993	287894	393963	150926	682797	209240	309231	317909	342951	328313	314225	346888	296925	89552	263056
EIC	Negative	255.1604	1.94	unknown	66587	178223	0	0	165913	243452	165910	0	176297	0	177651	267665	283123	149141	203850	221641	151844	0	133382
fpHILIC	Positive	115.0697	1.80	unknown	6901747	5718647	5540251	6289859	5729195	7698963	6595799	5620990	6831304	5707622	6457380	6114718	6789690	5071783	5757405	6960401	5302193	5604775	5181598
fpHILIC	Positive	828.5493	0.43	unknown	73689	571508	260739	355594	515073	359054	456886	233947	571486	333424	382807	479583	606406	438967	395288	480529	401081	296303	414852
EIC	Negative	604.3628	4.90	unknown	2957451	4221865	3724941	2610157	3645775	5377367	4301868	3845592	2291949	2393688	4108623	2963556	3107437	3317190	3454458	3039017	3594698	2182743	3522837
EIC	Negative	438.2490	3.17	unknown	170336	161771	174617	172386	173363	174390	171191	173661	163307	163739	197536	177420	172450	160357	170570	164859	169801	170035	170672
EIC	Negative	269.1374	2.12	unknown	56412	77687	170117	0	132092	99819	0	314305	0	260615	0	0	44658	0	0	0	0	342216	0
fpHILIC	Positive	164.0731	2.73	unknown	690136	281391	242907	780470	496270	497328	472898	356533	801038	513178	423163	316833	395136	177116	320595	665274	322356	521176	237955
EIC	Negative	548.2644	1.28	unknown	66089	106343	0	0	90549	78378	131040	0	105854	72698	92148	86164	106287	75373	80363	89248	108762	0	111879
fpHILIC	Negative	233.9133	2.09	unknown	602298	1133172	585704	285520	578571	1839105	965341	182451	383523	299802	1272379	834751	1293214	45991	533547	174851	366149	148708	759561
EIC	Negative	578.2732	1.87	unknown	458599	371055	0	0	318740	438013	683396	0	369718	0	416289	487249	431834	312444	347261	309864	419520	0	517522
EIC	Negative	430.1989	1.41	unknown	0	353216	0	0	365834	26476	360433	0	139657	0	200355	144242	183975	115544	336399	286564	99693	0	71369
EIC	Negative	263.1294	2.04	unknown	131546	145381	338867	94924	217678	270170	263427	249356	31833	171925	244342	220339	176162	105736	200039	154744	164719	97837	112350
EIC	Negative	372.1855	2.64	unknown	71748	619744	147298	492565	584141	859542	647278	118445	403183	452875	567794	624742	561388	508294	663287	708325	515968	80960	437738
EIC	Negative	464.3102	6.03	unknown	385896	549922	376222	462854	576841	590054	424469	400584	432106	110947	430715	435024	384227	428290	445437	396318	458807	365158	519323
EIC	Negative	272.2314	5.12	unknown	121005	138165	315147	313783	236777	158297	210350	338311	274055	170947	71129	119859	179636	194135	66668	207405	144282	288458	119319
EIC	Negative	522.2627	4.02	unknown	485434	1704906	408535	737581	1193830	1331869	943122	526827	1473935	556614	1312919	976495	1247422	1605223	1172245	951509	1295071	545122	1142122
EIC	Negative	503.2995	4.45	unknown	0	59850	0	0	0	997073	0	0	598056	422724	0	609346	550788	0	0	60546	0	43120	696969
EIC	Negative	607.3717	4.80	unknown	272870	516370	362956	267431	454458	520592	468683	320806	319535	296143	568595	400827	430288	398634	403963	419689	520513	227266	418124
EIC	Negative	630.3782	4.93	unknown	0	695224	1112507	0	0	0	1602415	1107577	0	0	1052665	1406859	0	484867	0	529973	0	0	999569
fpHILIC	Negative	302.2201	0.38	unknown	379707	1181445	1027392	1395901	973454	583866	1141396	373775	3424782	720645	677629	644741	1173195	551082	630351	1059593	906988	710124	551581
EIC	Negative	365.0326	2.04	unknown	324016	316427	487156	109417	182817	693960	990252	0	109638	265492	516579	253295	85308	156408	30425	272929	270329	134204	475336
EIC	Negative	254.2203	6.42	unknown	19000073	27846856	53621314	59120330	42855439	31022594	42421449	50869456	60501767	33920239	25577959	35541974	41957820	21988831	19658806	35890008	39095290	41844635	19381106
fpHILIC	Positive	151.0477	3.46	unknown	554078	574961	594411	601187	597234	569675	695085	666418	563374	593104	620112	654578	653172	418603	547491	611029	638688	630211	0
fpHILIC	Positive	321.2012	2.30	unknown	5968768	4163284	7871962	10376188	5509790	6554985	7507921	8702761	7866764	5678970	4286457	4200576	5832563	2368789	4844713	5872572	2439492	6847630	2084633
EIC	Negative	335.1536	3.43	unknown	287085	238545	333461	876588	446305	320145	437369	543776	657993	543844	339126	545660	493770	152798	199933	431157	381400	560450	169715
EIC	Negative	322.1723	3.43	unknown	521296	473108	580448	1219833	688795	605638	730302	807539	1022418	802333	667699	956361	866287	282592	364628	710290	648407	1086491	356178
EIC	Negative	387.0262	0.54	unknown	5298966	14012276	11737952	16002640	10039294	8288553	12550915	23312081	12187588	6186052	11119787	14301235	15468548	5762143	9104292	8986876	13908680	15022746	5154523
EIC	Negative	353.2002	5.50	unknown	2351757	1117471	1368252	1468766	2246094	856602	2336127	1182209	1528378	1333916	872961	1707518	749181	786005	648665	1344517	2108101	1494632	1195360
EIC	Negative	299.2527	4.62	unknown	471116	193559	179274	312971	162858	308747	227882	1033953	189254	667962	408153	441347	463823	552145	201126	372204	497091	174756	388967
EIC	Negative	392.2639	6.44	unknown	4547887	4187834	1327688	3485930	2792533	3903842	4874795	7117486	2094461	3151301	4029412	2059468	2833983	3408227	3298360	1520637	3313500	1982761	2212883
EIC	Negative	501.2819	4.64	unknown	47290073	60593287	44499575	47176474	56577468	59609138	55405847	48348014	48543791	37465543	46606758	44916815	42935516	56390632	49639248	33269753	53220722	43916833	52565898
EIC	Negative	300.2626	5.71	unknown	1019786	973594	2956000	2840350	2028774	1385296	2018703	3272635	2365274	1530572	924504	1188281	1330225	994904	498927	937925	1232801	2201723	987979
EIC	Negative	375.2481	5.49	unknown	0	0	275792	25350	0	145454	0	239970	183934	275999	0	173156	0	0	0	210606	0	253704	0
EIC	Negative	271.2282	5.12	unknown	718495	655073	1749804	1640711	1170037	936211	1091660	1861301	1507779	919312	657656	751234	867556	1032054	610696	955960	808725	1523470	820854
EIC	Negative	231.2108	6.16	unknown	48026	45056	36418	336344	157971	71711	145308	177468	165935	60072	36826	130909	96320	0	0	144670	76619	171207	42914
EIC	Negative	272.2314	4.22	unknown	304129	311874	917352	931698	703494	468071	555376	1096284	758703	458518	289879	364230	387641	432708	182266	376118	435332	679667	380202
fpHILIC	Negative	261.0427	1.77	unknown	269764	164893	146982	179824	145715	504836	210217	174181	140826	178108	224142	228144	232565	172423	231059	257250	159632	195370	155076
EIC	Negative	439.2080	5.81	unknown	106520	254558	164674	520113	254578	133072	220307	244146	526198	171032	145741	110030	298240	108654	120123	199490	276626	142382	93098
fpHILIC	Negative	310.1286	3.36	unknown	15506638	11675103	7148470	6820901	10122821	12666546	11100387	9271402	11652078	5769552	7562769	10151815	10970731	8088541	10379587	14136117	9387973	9359999	5333733
EIC	Negative	528.2728	5.82	unknown	708630	1245355	683820	800669	1186373	933314	1275291	814886	859883	592306	880946	705526	655007	983160	701720	570573	1018898	553553	803261
EIC	Negative	275.2016	6.16	unknown	1112061	1203460	1596247	5494939	2651045	1851914	2961265	3107132	3043708	1497678	961492	1840871	1908356	601148	784520	1761908	1653762	2447711	924008
EIC	Negative	589.2286	3.26	unknown	96899	0	44773	0	93707	0	65011	70380	36928	56121	79231	0	81304	69974	57795	61347	47936	18879	51705
EIC	Negative	540.2733	2.65	unknown	361455	428850	233899	342528	282491	536059	231193	255962	718176	254302	280021	450127	317615	210957	362181	501528	555795	83142	482253
EIC	Negative	541.3352	3.92	unknown	543576	43358	0	0	18853	0	0	1508322	51830	0	0	20091	26078	69718	61947	0	18640	897079	0
EIC	Negative	488.2766	2.48	unknown	0	342946	738426	0	0	243957	523677	571468	135112	0	0	0	0	0	0	0	0	57374	0
EIC	Negative	339.2538	3.11	unknown	607746	14413	79427	0	13562	72276	628874	619074	0	75387	39929	0	627447	638199	628534	51576	0	64193	46439
EIC	Negative	287.2016	6.21	unknown	388820	363669	455805	811197	617018	367869	672467	554817	626978	495148	384120	492775	485829	142886	270308	599045	441348	641460	287777
EIC	Negative	299.2592	5.71	unknown	4947385	4790455	14804000	14057185	9999869	6927199	10181549	15984173	11651714	7734725	4457557	5924808	6138244	4904976	2679522	4702487	6144004	10891387	5073123
EIC	Negative	489.2308	0.71	unknown	74995	92830	116460	68946	75895	86241	104851	114901	72943	71727	103976	96589	85150	60325	80306	65630	77550	93473	92937
EIC	Negative	396.8957	0.66	unknown	5172	44323	50169	51421	30857	16134	38431	91036	50322	19985	44074	45961	57504	0	31540	32063	50862	60252	17064
fpHILIC	Negative	176.0387	0.94	unknown	5889839	4469523	3390352	6988263	5962787	4578169	5217844	3878571	7521839	5084056	5996751	4562634	4291093	3333672	5805957	7115603	4691350	4077964	3711307
EIC	Negative	280.9787	0.68	unknown	82865	200349	194315	235924	143866	118378	177325	433926	170050	152403	197273	216730	235336	71595	134379	139284	223278	313427	115041
fpHILIC	Positive	923.2365	3.18	unknown	254496	411428	231954	391800	675025	504410	437763	380680	411379	453535	417966	417369	328811	323719	483858	428203	408233	317728	575462
fpHILIC	Positive	72.0563	3.36	unknown	712231	506890	316147	346952	393037	688988	529586	363542	628930	344332	409189	391044	502651	512398	625205	0	321838	583010	277903
EIC	Negative	299.1390	4.08	unknown	213394	182881	238345	540812	311535	235752	341911	310910	433042	393378	267097	365073	375908	108490	159532	328130	344924	397684	133862
EIC	Negative	607.3820	5.50	unknown	1788541	2973973	2350821	1983370	2631989	4076741	2642944	2344415	1670240	1675917	3000730	2602987	2254972	2491829	2350434	1992006	2451626	1603406	2779022
fpHILIC	Positive	282.0479	2.61	unknown	266311	327522	251082	556301	268283	491595	408363	305376	386967	348834	205134	429809	353093	225041	289350	432625	322303	470158	282648
EIC	Negative	391.2609	6.44	unknown	19234007	17787312	6895251	15344254	12753824	16504580	20980884	28737202	9488680	13975363	17333400	8332849	12031734	15582002	14203746	6633993	13987027	8565329	9270529
EIC	Negative	292.1251	3.36	unknown	39958	23574	50567	102565	66559	74430	80381	87011	35567	0	28750	63455	68544	22884	56575	69526	20150	85777	0
EIC	Negative	553.2739	1.58	unknown	0	0	62298	378274	0	49045	419342	0	209547	80736	21218	0	0	0	10429	0	10312	55507	51835
fpHILIC	Negative	226.1897	0.39	unknown	742250	858096	1477879	3025319	1868688	856720	1547485	1818383	2134083	1059237	666970	1448366	1314352	457515	568775	1260903	1123511	1292696	846369
fpHILIC	Positive	228.1294	1.80	unknown	2175515	1527801	1564367	2011209	1725211	2635022	1917471	1688599	2023601	1956343	1747355	1770121	2106803	1303034	1763168	2224796	1690825	1774555	1394694
EIC	Negative	337.2327	3.54	unknown	0	386334	0	0	487576	489553	0	0	614249	786305	488202	845231	687991	219054	311038	0	565132	0	271892
EIC	Negative	305.1604	1.79	unknown	0	129208	584845	0	124431	142282	199888	553038	170425	303380	590087	307047	137877	448810	40890	220113	91343	0	147236
fpHILIC	Positive	189.1596	4.65	unknown	11132787	5578001	4299760	9673751	8207628	9252723	8083922	5934840	12251175	6422646	6989313	8249875	8202962	7442594	10595869	9469946	8566663	5477491	7723053
EIC	Negative	605.3660	4.90	unknown	943296	1380545	1213688	855549	1153282	1778842	1423967	1236011	732387	776096	1279645	957708	1019948	1049521	1068446	982394	1176710	695461	1167988

fpHILIC	Negative	175.0441	3.81	unknown	340281	226858	226932	232029	203024	396261	151317	156305	705531	176119	181567	326443	214159	194523	181622	202523	225762	255035	193201
fpHILIC	Positive	87.0796	3.36	unknown	586463	623077	278389	253698	395818	664077	514194	347888	438946	322939	315096	453144	498030	644184	487258	536737	352827	479054	287045
EIC	Negative	299.2593	6.48	unknown	4576471	10410487	11985808	11778183	11443757	11421478	7304999	16494675	8013258	6995091	4964486	5690196	5046449	8039051	5422724	3052924	4963356	9302209	8294645
EIC	Negative	353.2312	4.22	unknown	168597	162171	409930	409287	321683	221271	262364	477617	324309	210466	147694	179157	188644	225516	111386	163675	193990	306539	163859
EIC	Negative	503.2870	4.64	unknown	1287923	1706554	1106546	1206433	1483592	1608012	1538374	1229325	1345167	973401	1336683	1130856	1115831	1440913	1267356	852546	1363491	1127407	1304720
EIC	Negative	337.1693	4.08	unknown	413316	355942	488907	1250406	653332	508963	730667	674682	1010173	814010	640747	892582	826117	219257	290312	734248	681864	900295	258164
EIC	Negative	469.0291	0.54	unknown	2849583	7132862	5595733	8021409	4880786	4193618	6098606	11974744	5994530	3303280	5636015	7005141	7564093	3103898	4273237	4261993	6958979	7361554	2595041
EIC	Negative	371.1891	5.81	unknown	113615	284433	157563	662915	303713	159239	235724	255705	621181	189185	149844	144604	363598	114942	146975	240782	297588	157934	104126
fpHILIC	Positive	455.1799	2.62	unknown	1906427	1249114	1023832	2414848	1545558	1731731	1380878	1600878	2188059	1532351	1061293	1766301	1668622	1046914	1053294	2036889	1090055	1619662	1078819
fpHILIC	Positive	307.0831	4.52	unknown	388058	151509	81704	197222	291094	620941	203035	146292	276598	151020	178495	311350	174629	272149	189444	278450	281817	249197	325648
EIC	Negative	349.2385	2.25	unknown	170144	0	188317	171210	103083	120818	80536	484629	49790	110412	0	0	0	0	0	0	0	469441	0
EIC	Negative	629.3659	4.78	unknown	2748733	4206098	2400651	2638354	3634729	3042706	3312382	2832031	4372278	2383256	3670554	2758344	2794956	2854993	2531987	2685441	3151188	2130227	3108117
EIC	Negative	251.2015	6.28	unknown	5014556	7119835	8628276	16011932	9833643	7853688	10864865	10816582	12432189	7948753	5704132	9283970	10609802	4338826	4414317	6754360	7542501	10003519	5311264
fpHILIC	Positive	190.1429	2.82	unknown	323355	488868	293199	405470	354902	631523	282963	162243	804380	378506	351031	409906	462702	303861	357130	512859	368125	315407	308666
fpHILIC	Positive	534.2936	0.90	unknown	282980	453273	430779	321419	409963	505104	640019	224670	467391	248238	373047	406750	379131	355821	387664	395756	300588	196329	338165
fpHILIC	Negative	165.0555	0.57	unknown	10805295	2189300	2419653	1688705	5056176	19908718	2370850	1522517	2164094	2321714	1964072	2114897	1809671	2986075	2799424	1542405	1566311	2253822	1848958
fpHILIC	Negative	132.0748	0.54	unknown	654394	287353	190641	341797	440851	991426	321348	117755	442978	177515	305888	310258	264825	248153	429256	211294	184197	118549	254172
fpHILIC	Negative	271.2278	0.39	unknown	0	1821260	4592427	5860128	3006040	3121304	2895988	4610733	4452203	3548310	2186911	3225104	2779212	2094645	1320882	2804115	2462310	4214241	2445479
fpHILIC	Positive	781.5547	0.44	unknown	1034233	2311756	1550764	1667620	1639500	1703105	2148785	1503208	2854769	1234385	1658464	1567938	2000318	2616721	1747443	2003759	1753726	1202124	1606075
EIC	Negative	300.2627	5.51	unknown	311460	259435	682333	622914	469186	325951	389908	852334	578131	384920	246172	274770	284426	330674	167979	264766	296932	524641	255596
EIC	Negative	598.3732	5.46	unknown	8106720	7698278	8551115	7619898	8726361	8302334	8601255	10001342	5742072	7837681	7448362	6463620	6992082	6842384	7536317	5840779	7205882	7815508	6431246
fpHILIC	Negative	299.0984	3.34	unknown	705201	539757	597051	624976	682999	657064	695674	594171	605097	571882	596217	644084	571136	348745	538341	575112	559377	540851	494385
EIC	Negative	327.1633	1.83	unknown	0	23209	0	0	165116	134398	0	0	259893	0	0	0	0	124527	0	0	0	0	69842
EIC	Negative	397.2048	6.23	unknown	97890	216426	155315	258321	161760	123075	149589	185276	486235	210901	163028	94903	223091	131819	114726	394913	230437	136709	112731
EIC	Negative	435.2327	4.13	unknown	240744	211608	421239	502062	370906	254804	371837	575600	370423	402517	228511	239675	218043	423798	168460	189818	310366	526798	297795
EIC	Negative	301.2659	5.71	unknown	118932	111687	351496	340545	230301	156496	229169	391936	287635	179076	106859	140130	134914	121002	60093	112324	150553	263999	138160
fpHILIC	Negative	310.1134	3.32	unknown	998686	1148569	820117	2491503	1404915	2508662	1119451	1269654	1428733	1521372	1760381	1572582	1570971	732966	1005314	1515448	1794785	1512609	1409216
EIC	Negative	632.4754	6.44	unknown	1353601	1837760	826942	3804821	2064410	1229640	2226569	2064754	2965753	1157854	1348482	1125010	2452533	837703	846790	2236455	1486936	1323125	1079672
fpHILIC	Positive	190.9925	4.01	unknown	276818	421143	505438	371387	386247	252174	428729	657008	376831	546377	529237	225039	254213	685672	713264	198111	271649	394041	358066
EIC	Negative	389.0306	0.54	unknown	143505	356878	295356	388464	249997	209337	290574	601967	286698	144774	278946	337000	381076	145485	211921	225761	351636	380448	113518
EIC	Negative	599.3781	3.16	unknown	407327	350592	378427	424354	394657	503631	416120	484193	272015	382175	352936	435384	352026	239146	327791	327328	401310	478860	342153
fpHILIC	Negative	295.2274	0.39	unknown	1856855	1797972	3389772	4491974	3099130	2236323	3076267	3286371	3487290	2493409	1619219	2415189	1816752	2354699	1474531	2707508	2380704	4215946	1798394
fpHILIC	Negative	320.1239	3.47	unknown	977715	700456	783886	1029105	1139238	1085606	944578	1068772	767706	1095938	1101348	1179967	1058825	892436	1023237	1024567	933721	1105193	477829
EIC	Negative	417.2042	1.87	unknown	28623	116101	0	0	69517	39130	37724	0	197694	0	65161	54058	63240	95651	84814	127485	46862	0	42195
EIC	Negative	288.2260	6.35	unknown	236220	256450	536852	674061	391922	506224	378605	509622	528083	436660	250346	413357	309679	228655	173543	301945	400695	449278	231612
fpHILIC	Positive	371.2992	0.60	unknown	677761	250093	908426	1315930	647322	864176	730178	940744	1276702	623611	639294	535568	907638	0	235278	735231	561695	582381	151577
EIC	Negative	351.2163	5.06	unknown	87555	61442	101708	247767	120012	97142	107969	139123	169490	114491	54632	72127	98166	51804	34835	123003	139752	140359	43942
EIC	Negative	391.1749	2.85	unknown	500423	1280480	276313	550607	643067	965906	652162	799259	381476	1243919	1236695	814437	439499	600172	762630	574061	716814	940718	326135
EIC	Negative	605.3555	5.31	unknown	0	1938334	0	0	1581428	0	2198200	0	0	0	0	0	0	0	0	912899	0	0	0
fpHILIC	Positive	220.1174	1.65	unknown	0	51940016	22024240	28259054	23277452	57460720	36245272	0	38888924	23364996	38507824	37610184	32350848	33341476	51341040	50996372	15798013	23301850	0
EIC	Negative	606.3784	5.50	unknown	5254512	8588420	6933758	5693985	7609802	11874125	7868372	6711212	4842624	4840239	8667315	7500210	6707696	6968040	6632922	5946835	7435700	4550279	8178127
EIC	Negative	630.3782	5.28	unknown	2901278	3134337	2732899	2794510	3623278	4548473	4139793	2888780	2137950	2160115	3924800	3828800	2775714	2316232	3233923	2672015	3458887	2311088	3296479
fpHILIC	Positive	273.1206	2.29	unknown	0	520438	925816	688675	524200	618571	525195	851556	363923	274188	406477	200076	324060	160275	233491	225417	0	572080	0
EIC	Negative	316.2570	4.03	unknown	548505	554505	28682	1896055	855129	765427	844609	1149590	971721	761395	524732	898625	838074	671471	360109	1513009	726416	1304147	22114
fpHILIC	Positive	257.1508	2.29	unknown	2106079	5608421	7440330	10328407	10178366	9395215	6050910	9152170	3696466	4160075	4201882	4380364	8659962	3912639	9518342	6828265	1232834	6332949	1548887
EIC	Negative	526.2845	4.71	unknown	853224	1904408	830096	214103	2129345	2116661	321243	272787	2388664	760717	1526523	164480	1101294	2325761	1612519	1206133	1635825	830056	1348011
EIC	Negative	608.3942	5.49	unknown	341109	605186	240029	424342	488214	852964	559049	374599	351345	387512	430232	35806	366021	545260	344660	197051	162810	182079	582677
EIC	Negative	508.3380	3.38	unknown	100955	58365	125188	80672	92805	100604	82805	142110	55727	21679	42178	44444	48348	60115	66243	51855	41662	113708	33682
fpHILIC	Positive	133.0738	3.36	unknown	10805508	8698263	6065598	5416841	7484735	9298935	7463904	7989678	8636757	5631092	5820047	7422906	7946172	8697155	8748635	10161920	7317190	8839452	5351865
EIC	Negative	305.1435	2.73	unknown	4331948	4544004	2102526	1305609	4302205	4247174	4995058	2060889	3520932	362013	902183	1009356	1037013	901508	1041473	1208233	945117	90787	1270272
fpHILIC	Negative	237.9090	2.09	unknown	545405	1116809	607355	336401	495059	1923659	887355	175796	360395	291909	1134636	753299	1121948	44543	545830	140174	327415	143918	668663
EIC	Negative	632.3941	5.90	unknown	132202	155184	152131	108460	155134	287216	199938	135385	83513	96355	156880	143104	91369	88666	129104	95751	125654	70740	124964
EIC	Negative	323.2503	5.03	unknown	82836	0	143707	0	70109	29132	0	217161	155108	0	22928	0	0	0	0	122660	33745	269470	0
fpHILIC	Positive	192.1052	2.06	unknown	330383	276297	351882	559220	422254	471635	409141	270775	654509	363127	375317	336670	400467	195210	260623	458980	252780	385782	151695
EIC	Negative	287.2016	5.92	unknown	96564	407277	159565	879315	459902	187654	282913	292097	875203	251893	167628	126373	524666	132833	122621	328043	408753	223165	135432
fpHILIC	Negative	359.1195	3.18	unknown	14866715	11271100	14324599	14982511	14052484	13839744	17600454	14492178	13203548	14386454	14892760	12083961	13147460	6389868	10341661	11317844	13379823	12111816	10245517
EIC	Negative	308.1604	3.17	unknown	174037	83089	159227	157747	151981	94091	155007	241933	120212	171432	68621	71430	74688	75602	89200	81616	95870	133065	68478
fpHILIC	Negative	167.0473	0.86	unknown	359947	71378072	130827	43598	51239724	48846460	929183	312834	71067	0	0	20687558	955308	0	16490147	0	685854	33220490	35159320
EIC	Negative	305.2389	6.44	unknown	6140092	7777911	2030209	8648067	7200678	6419197	8137084	8187117	7239265	5340681	7254822	7396195	8039067	6435386	6555933	7437997	7578497	7988704	7168876
fpHILIC	Positive	341.1725	2.71	unknown	1484601	1487446	886926	1812922	1750279	1818803	1613058	872317	1523734	978162	1258618	1240689	1686020	741052	876459	1931975	1490640	986409	868419
EIC	Negative	306.1462	2.73	unknown	690070	740370	362925	253496	675839	626241	875587	374762	558282	40930	121950	141338	128824	100859	130752	137728	105768	0	190156
fpHILIC	Negative	272.0539	2.98	unknown	834520	594319	508943	647044	604421	824247	666904	530083	728858	703034	632158	575269	640298	488447	582932	623151	680251	657721	671528
fpHILIC	Negative	111.0088	4.59	unknown	1849580	1535579	1478439	1771143	1664985	2321385	1712903	1434117	1564720	1315602	1581191	1540711	1472637	1470275	1363595	1720586	1558326	1583338	1434369
EIC	Negative	609.3968	5.73	unknown	0	0	0	295724	0	521992	0	85390	0	61457	67803	366724	0	0	0	0	343920	259899	0
EIC	Negative	329.2389	6.41	unknown	8759750	14308720	11928951	19644786	14715996	13096988	13069400	14442713	16439969	11765833	10951256	12430829	14908739	8134439	10011183	12686098	13900195	12049519	9953934
EIC	Negative	412.9672	3.17	unknown	79854	155989	88610	78356	151471	139508	120785	72532	134778	74023	147491	105751	132236	121486	132793	121129	117359	67424	106183
EIC	Negative	630.4829	6.16	unknown	236796	285124	300944	1087748	324334	459154	728377	563264	589899	275470	194335	260139	446910	94353	144472	167066	405716	332326	118919
EIC	Negative	464.3092	6.25	unknown	529442	920786	63552	958502	616578	942131	720691	559264	704496	651404	1258793	473311	585338	1437069	670596	365857	1023216	435801	679971
EIC	Negative	248.1739	4.79	unknown	176440	372090	321644	1615009	577536	272113	422573	569324	1324001	432965	219243	240062	679153	148232	127371	507369	501500	498392	179326
fpHILIC	Positive	307.0255	3.39	unknown	296842	324063	513467	608259	617581	246729	690732	521851	393923	677725	464759	440105	382217	446898	354310	475918	400435	485247	435282
EIC	Negative	317.2489	6.49	unknown	381755	460282	716003	1078609	617625	808421	612939	553245	694880	367652	328788	562496	698893	456179	444816	286804	657425	398537	298502
fpHILIC	Negative	436.1599	3.32	unknown	587546	1335973	524626	536879	1141449	1372338	1499569	456232	931737	419794	1124888	1337974	1064247	846505	921537	1072714	1091570	537449	1327415
fpHILIC	Negative	118.0591	0.78	unknown	514926	313737	407383	488424	444081	740675	379968	350902	440641	448835	391447	385713	439133	335056	336832	165772	365203	285593	361701
EIC	Negative	381.3384	6.72	unknown	514901	613784	751720	1243443	659691	846838	1043780	1023598	653774	791679	649738	960449	949080	408155	715244	834875	861201	999019	474527
EIC	Negative	595.3814	5.75	unknown	122938	141524	113584	117791	128287	170641	172265	124996	122282	84485	120761	145230	59431	119949	126880	151553	114881	90260	145111
EIC	Negative	526.2845	4.90	unknown	0	2680439	1455341	1641116	2796369	2251247	0	1713253	3323337	1032116	1766441	1491450	1463271	3006339	1693117	1178684	1936021	1441417	1944266
EIC	Negative	300.2566	4.34	unknown	1037480	1272096	994942	1038475	1108227	1120802	1106730	1089451	1123366	1123094	1120840	1048158	1057385	1066561	1037828	1066067	1127901	1056948	1069622
EIC	Negative	601.3347	3.99	unknown	339937	883641	390823	391346	641781	578032	636795	415932	782851	316912	585835	517409	643111	732272	449789	574743	620684	336119	491769
fpHILIC	Positive	105.1042	4.07	unknown	1358715	890980	783054	806975	936007	1095023	1035069	909231	758596	717776	562677	747206	562963	554889	814694	832053	587596	819186	731650
EIC	Negative	299.1228	3.43	unknown	49307	55974	59494	148209	87085	69036	78324	98568	101697	104525	72643	95930	117313	38064	45183	88227	67562	98401	40194

EIC	Negative	526.2947	5.07	unknown	3912706	2312995	3612867	4026577	4436174	7292780	4788923	3670326	1349348	2654057	3630547	4983947	1699049	2686066	4540498	1474576	3103281	2587937	3069811
EIC	Negative	243.1965	3.23	unknown	412051	346555	1068403	1008448	752552	559346	611417	1167675	843740	605854	385486	428619	414156	535570	172474	354491	475106	841483	437735
EIC	Negative	560.3314	6.23	unknown	665804	705196	731194	772925	807657	788785	757803	722345	701776	715641	787999	762306	728566	745312	701507	716349	730366	744766	682238
EIC	Negative	271.2281	4.29	unknown	751764	579830	1119721	1418132	1019253	814762	842749	1410385	1047283	828801	600454	656840	760795	736959	536271	930739	741589	1121218	674585
EIC	Negative	308.0679	1.55	unknown	2066907	2286928	1447118	772827	2575625	2899852	2682661	1041727	682100	1285582	2543182	2093307	1679659	1765155	2698125	1512417	2077209	975249	2536639
EIC	Negative	606.3776	4.79	unknown	296750	250403	0	316665	2575872	3155720	0	0	372466	169321	0	0	0	279496	0	0	269265	0	0
EIC	Negative	325.2010	3.09	unknown	346711	336955	499064	644883	549629	362758	445288	620161	505319	446810	311870	371462	383369	516875	235769	324630	408944	552781	365459
EIC	Negative	387.1084	1.39	unknown	321293	361455	0	0	213792	245077	473223	0	241924	0	95081	226840	286915	88593	58221	218610	171719	0	275697
EIC	Negative	426.1904	0.69	unknown	100197	114942	75828	62642	64433	79202	103607	157189	80960	75933	102052	133175	96953	80300	88711	77601	79645	98797	90803
EIC	Negative	558.3329	6.11	unknown	2632310	2907580	2570041	2820660	2648695	2768914	2930133	2794947	2645323	2646075	2653574	2873135	2837558	2672979	2516776	2567671	2756627	2591107	2812137
fpHILIC	Negative	163.0402	1.77	unknown	759283	369924	350128	194633	401505	708585	659080	208422	152279	260371	445898	252892	229672	291636	302278	198812	256475	820897	249149
fpHILIC	Positive	163.0797	3.75	unknown	279967	339593	279426	207589	314134	632042	317665	135034	254926	172903	343786	268004	325285	625340	258360	309611	280427	179427	251955
EIC	Negative	507.3332	1.99	unknown	0	220958	0	0	287839	570520	147785	0	132380	0	215040	301889	177337	202887	259238	158080	203912	0	108488
EIC	Negative	516.2734	3.30	unknown	561188	356669	394194	0	297500	598664	261711	412976	395055	487476	242079	380172	227784	238059	370565	346536	525262	0	545898
fpHILIC	Positive	381.0783	3.53	unknown	105331	702467	715959	165990	261390	246404	577079	391886	628518	194437	136022	235739	260435	716689	216336	181607	425894	234499	632342
fpHILIC	Negative	189.0349	2.71	unknown	940958	582462	734893	1180989	755197	882010	866688	941464	970505	938670	744057	795773	860670	685402	451387	846029	848038	928277	623340
EIC	Negative	296.2313	4.49	unknown	395429	290819	875772	1151800	681224	446230	492599	1148296	917314	487945	331350	409187	361754	570151	127588	401846	389730	853102	340302
EIC	Negative	425.3074	6.58	unknown	239207	670290	1147811	439265	982930	294573	354777	941459	974348	1519883	785445	272582	907635	206643	339118	602545	514440	489894	198632
EIC	Negative	627.3504	4.43	unknown	53603895	69128127	53774986	55055661	62967034	59941779	64465307	55287518	63111057	53384643	66527980	60053884	60896149	58590223	58179703	60258750	57831881	51590502	59737895
EIC	Negative	382.3418	6.72	unknown	125545	164654	201710	337708	194139	219081	271403	270735	164663	187597	169442	268700	248678	140124	194008	211232	229475	268236	140309
fpHILIC	Positive	158.0268	1.02	unknown	351113	200557	376339	222610	249866	494871	157386	313133	601339	161200	272681	280483	263193	376302	220424	227204	408054	257516	204120
EIC	Negative	320.2260	5.66	unknown	0	0	0	0	0	203766	228701	0	0	0	162834	258080	0	0	0	0	205718	0	0
fpHILIC	Positive	202.1181	2.40	unknown	4430570	3151245	3683662	3995291	3891399	6002427	3337912	3969827	3112547	3700112	4797456	3695187	3475055	2606365	2906107	3324090	4100800	2506121	3479177
EIC	Negative	327.2255	6.22	unknown	108225	352079	111212	472072	324090	245342	211832	160564	470716	173919	318960	185102	441796	161273	194726	269943	309355	212956	184194
fpHILIC	Negative	242.1844	0.38	unknown	607831	640635	495553	3276068	889301	789321	1390342	1134131	2514766	1382961	808699	1208493	1443015	180368	298122	1045468	1323825	2193017	274778
EIC	Negative	275.1269	3.25	unknown	81936	90332	86335	173286	123232	86206	97757	100928	156880	109314	77094	95862	114998	73212	75021	118480	120266	98384	75553
fpHILIC	Negative	217.0175	0.77	unknown	1070744	304852	428675	214716	425736	1132002	386112	384891	530991	86824	32478	82541	105279	226933	141879	173970	81736	170433	0
EIC	Negative	473.2881	2.76	unknown	603128	619171	555738	714370	1006149	539788	1168180	295541	257108	227319	0	0	0	0	0	0	0	90840	83502
EIC	Negative	523.3841	5.80	unknown	0	169746	0	0	348159	0	0	0	0	0	0	98239	193523	0	0	84280	0	121287	18539
EIC	Negative	283.2427	6.41	unknown	3707619	6666150	5160749	8576713	6482801	5819981	5806074	6631986	7284998	5072646	4970955	5825570	6666388	4606377	4233841	5856070	6377789	5295271	4156870
fpHILIC	Negative	429.3003	0.40	unknown	1177682	467443	1406895	519394	668587	670684	843328	1426897	1067858	524566	473745	438094	393402	1204866	629832	438354	576029	737286	568226
EIC	Negative	377.1798	1.90	unknown	193906	455474	0	0	260620	320292	367832	0	191473	0	307512	217541	99028	0	320017	396330	199483	0	149678
fpHILIC	Negative	222.0263	0.59	unknown	627285	816196	207068	468783	337204	1465459	516152	278462	522512	175865	596696	1465520	611717	560741	499893	308412	537955	0	1021434
EIC	Negative	253.1446	2.00	unknown	267244	453703	200372	122842	436391	347840	404046	0	500534	155944	332533	321973	400950	277947	330245	615175	408395	0	233680
EIC	Negative	326.2720	5.20	unknown	261619	228361	225599	255932	255400	261844	228276	264524	215569	241599	253325	236627	235935	94250	266339	243722	44399	261531	30208
EIC	Negative	602.3388	4.14	unknown	43694	95037	114433	165052	142658	431673	127074	0	0	0	0	328433	388461	475809	98698	0	0	96229	0
EIC	Negative	232.8893	0.66	unknown	26348	72525	93648	85375	66835	45235	67756	119081	77165	60113	68422	75367	89450	28316	56453	54476	81751	96657	53105
fpHILIC	Positive	232.1539	1.83	unknown	11887471	17966620	14221863	13440903	14361327	24781862	18844606	11738198	12835019	0	17721650	16927182	15470689	26557990	13363095	16930370	13372223	9541811	12945118
fpHILIC	Positive	127.0502	1.03	unknown	1053972	561811	842749	1017017	938172	1012050	688995	665823	968758	635014	438519	459929	490552	396734	432972	524787	509253	455914	275667
fpHILIC	Negative	619.2891	0.50	unknown	3318748	3658057	2727293	2678036	3386982	4468000	4254425	2584801	3938606	2155868	4110283	4449310	3967200	3093531	3512078	3975872	4015624	2046850	4362257
EIC	Negative	291.1220	3.08	unknown	79283	186636	135109	183295	194857	87751	137567	102212	234659	129389	89696	69543	170382	66206	78219	247867	115705	121613	47012
EIC	Negative	276.2050	6.16	unknown	208482	228776	302540	1154153	532141	373570	583112	592811	608178	310943	205660	375427	397605	70044	189968	337603	308600	473986	180777
EIC	Negative	299.2590	6.00	unknown	600814	613935	849458	801409	729020	708063	762678	804810	643899	550121	566981	624277	610403	740405	505440	698564	638388	742542	746065
fpHILIC	Negative	200.1738	0.40	unknown	813214	578562	1196426	1761179	1035744	743938	724340	1071245	1871612	740883	506294	620189	797447	512149	384549	691005	722213	949128	665028
EIC	Negative	253.1446	2.36	unknown	956402	677001	1002381	1164983	753854	848175	1000029	1302574	887292	1362592	644800	660033	631820	614618	691074	774666	735879	1214145	838570
EIC	Negative	484.3647	6.02	unknown	1360397	1368879	1304501	2900951	1604246	1764859	1822209	1577936	2443061	1245168	1543802	1680716	1954888	668888	1203799	2148324	1192237	1379285	992583
EIC	Negative	316.8898	0.66	unknown	19949	94757	113377	103825	74499	19703	80540	199578	97980	62123	86897	95064	121222	21732	57625	68355	109445	136209	52199
EIC	Negative	552.3321	4.04	unknown	4432121	8149139	4549633	1281205	6017666	7560236	8204842	4614307	3902642	4424311	6711250	7399179	6344512	7420451	7184957	5683027	6832183	4017399	5904306
fpHILIC	Positive	804.5516	0.44	unknown	1006671	2818905	1325505	1625346	1828698	2190775	2469627	1467867	2477540	1310029	2044274	2334019	3044197	2158476	2055255	2483500	2359396	1275589	1863864
EIC	Negative	635.3377	4.17	unknown	762238	978782	753900	695833	830965	825915	912957	775426	968496	688864	805359	805144	914239	934507	823628	878892	820189	723436	756712
EIC	Negative	322.2441	6.04	unknown	0	0	364670	714081	313802	282928	374106	448363	324264	334715	255626	0	135637	89292	0	266441	74635	18878	0
fpHILIC	Negative	256.2364	0.38	unknown	41458624	61967972	83286496	103629992	72620256	63750544	68950704	78952120	95734656	50876824	52819452	64826540	70554008	51929652	47381120	62098728	64518180	64786748	58474320
EIC	Negative	247.1703	4.63	unknown	34243	241939	205256	1307542	654818	88832	337581	358099	1016373	319140	103960	111959	744881	40725	38040	514654	843386	212157	54482
EIC	Negative	628.3626	4.78	unknown	7700722	13220047	6759928	7432951	10082356	8568617	9250600	7667119	13381107	6836304	10441892	7782151	8581563	8174578	7329019	7690725	8847820	5782445	9188280
EIC	Negative	285.0770	2.32	unknown	167218	41977	65647	46143	47334	131001	68610	116728	194812	105751	110973	195644	88700	49274	14037	46938	94793	246045	90669
EIC	Negative	432.1024	1.41	unknown	221013	67399	0	0	29548	237906	115773	0	54409	0	112241	72725	53498	33438	0	68035	167672	0	73912
fpHILIC	Positive	196.0970	2.48	unknown	812172	740309	694593	1061665	822910	642598	941848	926859	881026	913939	935337	961237	912777	692834	795337	796960	779487	847163	776171
EIC	Negative	473.2762	2.61	unknown	141674	397743	292559	217693	400657	225723	385296	128067	397529	213574	378716	343374	366436	295633	301815	290910	322566	0	373307
EIC	Negative	326.2710	6.38	unknown	286200	365823	393553	831662	351551	288465	440400	513441	776474	328672	353970	447330	440715	217461	193184	654619	329884	653737	224278
fpHILIC	Negative	111.0088	4.72	unknown	4397925	2817147	3018086	3390482	3447908	4614283	3304783	3231975	3657804	2952538	3144224	3041255	2958237	2994522	2750348	3766938	2998673	3335215	3018244
EIC	Negative	475.2338	3.28	unknown	399479	0	20130	202950	0	0	522664	432432	0	0	0	0	0	0	0	0	302376	0	0
EIC	Negative	296.2313	4.87	unknown	333568	240189	557337	385639	343967	318286	361898	518542	476284	262457	253696	255710	279771	537572	152874	281248	324163	479307	281768
EIC	Negative	485.3687	6.02	unknown	404337	422266	384927	887119	471852	505602	554836	472204	748089	356019	458688	504845	605058	188093	337191	620925	357532	406225	297416
EIC	Negative	629.3559	4.43	unknown	2241465	2554362	2006726	2184029	2436963	2241370	2445664	2191126	2410573	2217088	2712114	2467481	2235132	2227213	2288363	2226804	2198711	1931367	2413305
EIC	Negative	593.3715	3.30	unknown	203855	29081	292763	163497	176663	216493	101961	368906	19014	111862	56223	66494	43013	18404	50579	0	56183	347722	14515
EIC	Negative	608.3891	5.50	unknown	409912	626792	531987	445327	621135	868109	620407	481457	370844	354119	675917	555855	480557	601967	529627	520090	584605	364061	608265
fpHILIC	Negative	192.0230	4.72	unknown	9280441	6106737	6196729	7614522	7275564	9644101	6846556	6788624	7976696	6141169	6344529	6728533	6357970	6675093	5805201	8093116	6202052	7021049	6316357
fpHILIC	Negative	380.1346	2.03	unknown	296419	301957	384483	342388	359963	555008	334420	214511	183811	373939	331785	323902	206952	121491	316489	480894	148829	248161	250462
EIC	Negative	552.2198	0.71	unknown	138829	292156	331441	208110	171765	202578	282955	422940	174594	165560	299414	277078	243613	97577	224302	169086	238878	290448	140497
fpHILIC	Negative	235.9113	2.09	unknown	1333048	2292676	1246749	572116	1081657	3584573	1787288	428220	716322	609123	2559775	1740636	2435170	87824	1154507	291240	679044	259733	1447606
fpHILIC	Negative	494.1893	3.52	unknown	475600	4757677	3486600	812968	1253588	1285621	2953280	1762183	4823937	575080	387857	1006369	1246599	4759405	727793	658574	2292762	939111	0
fpHILIC	Negative	307.1544	0.93	unknown	513140	238051	482535	370564	414553	299133	380123	725636	250657	423492	209066	218911	236190	178584	345016	333634	277271	589020	226388
fpHILIC	Negative	620.2903	0.51	unknown	1155136	1266390	1012593	920810	1270499	1572564	1404757	737476	1254963	726450	1331696	1362638	1275125	1073470	1059549	1391963	1262818	803756	1491719
fpHILIC	Positive	114.0663	1.80	unknown	153068176	119071608	123695992	146612288	125144368	167256384	148443008	125842040	145605328	124927776	142163264	146664096	148254704	108291424	132689928	151871824	117070792	123975752	108107440
fpHILIC	Positive	146.0809	2.82	unknown	4803964	3523883	2862224	4705490	4212707	5438630	3730484	2955459	4290673	3646411	3388758	3414549	4076088	2918126	3233511	4472917	3851590	3186097	2790057
EIC	Negative	598.3734	3.16	unknown	1239150	1180871	1201287	1393071	1301964	1629642	1279539	1494245	828504	1178038	1106348	1264798	1109121	813583	1066419	1057147	1252694	1444492	1065814
EIC	Negative	470.0325	0.54	unknown	366956	966858	760355	1078076	657551	515242	805576	1618353	791941	421031	728433	1007095	1059734	434331	585494	566966	888530	990086	348850
fpHILIC	Positive	332.0952	2.43	unknown	442301	179830	758462	119277	557461	457720	235794	99509	411859	120830	0	0	560494	576529	0	0	785159	0	0
EIC	Negative	626.3473	4.43	unknown	149730013	192802373	151466709	152195665	174665106	165784706	180511223	152136102	174523819	148538333	185510597	166260510	169122547	164558993	163234526	167935138	161795522	143080010	164772847

fpHILIC	Negative	328.2363	0.37	unknown	7922750	19612596	9110410	26857354	16137965	11911823	15675947	14667008	24501590	12067491	10263071	10524747	18225682	7106261	10562353	15391818	14585811	12543941	9745140
fpHILIC	Negative	92.0434	1.74	unknown	334641	1017071	1080497	1367485	971187	1090733	1035062	875393	1219955	925427	787752	1031351	966754	640695	625208	1171554	864401	1118340	800011
fpHILIC	Negative	270.1712	0.41	unknown	1130315	641358	1081558	765838	674198	958793	914027	864467	1253372	1269061	950029	1021577	840741	564928	612950	756136	839214	1156566	833710
EIC	Negative	595.3806	6.00	unknown	1445098	1918826	1434819	1317622	1591762	2450783	2227714	1468507	1044802	1060218	1327100	1493218	1282735	1171281	1482536	1089877	1291877	906312	1284991
EIC	Negative	335.1865	2.49	unknown	1357761	330497	581428	382090	748859	643272	892834	1403031	212175	1220409	273267	362610	243008	295001	374945	271544	351247	1123100	431068
EIC	Negative	418.9730	3.75	unknown	272027	390095	238753	292204	307613	348520	327380	258861	326357	219495	327426	266645	313038	289687	325356	332400	288362	257623	251062
EIC	Negative	463.3059	6.24	unknown	1455745	2343160	1182381	2907336	1620161	2975225	2072795	1893583	2163035	2025764	3797076	1469982	1856192	4625532	2306899	1124923	3440924	1259679	2174716
fpHILIC	Negative	539.5033	0.38	unknown	317987	474242	1307522	1679989	923598	876024	931100	1292300	1173442	617907	468761	726254	674468	250977	181687	292185	914812	1165572	796032
EIC	Negative	527.2984	5.06	unknown	1234218	666362	1105381	1208140	1168912	2204986	1353069	1095584	392208	754635	1057427	1524818	531679	849428	1279829	404318	895677	812769	862341
EIC	Negative	310.1746	5.20	unknown	0	320994	421471	0	713230	0	0	396308	0	448353	0	0	225145	0	0	0	0	0	416030
fpHILIC	Positive	150.0545	0.97	unknown	302988	388564	144313	158972	422399	463982	400716	116569	282267	255953	586434	488734	386235	372752	536865	341200	363403	215615	0
fpHILIC	Positive	763.5924	0.44	unknown	452005	394265	381702	259719	427584	385932	271888	407881	435558	377819	538934	386438	352572	543590	356393	354540	310830	303636	301320
fpHILIC	Positive	245.0955	2.61	unknown	1062792	778425	565356	1363642	791337	1263764	770259	794468	1201528	820770	584933	1083046	935538	717522	669995	1154466	671904	923748	740065
fpHILIC	Negative	369.1558	0.47	unknown	713288	422405	466940	1038662	677201	798506	727429	559201	675533	554123	571249	570692	562737	223983	554420	849765	509050	653419	224810
EIC	Negative	431.3180	5.26	unknown	299308	61517	507409	198940	277105	144465	266784	501259	163872	116544	77501	101934	99822	112146	142986	105361	110186	236193	127151
EIC	Negative	301.1658	1.60	unknown	92435	168418	168548	0	138968	47146	142823	191058	166853	126487	102393	73260	73117	77277	69780	61684	78430	0	56859
EIC	Negative	276.2050	5.81	unknown	1729428	4308784	2786722	12465517	5350691	2595227	4176416	4682624	10150539	2772047	2432684	2224274	5052000	1883443	2158347	3550825	4793661	2729687	1569854
fpHILIC	Negative	147.0300	3.97	unknown	16165833	8599044	10026224	16745281	12970774	17355014	13016778	11232698	11837419	9846015	8823574	8915153	9389089	8373961	9627336	10556342	10661743	12034780	9359498
fpHILIC	Positive	197.1011	5.40	unknown	1229763	1254003	927320	975404	1125046	1256403	1172874	976980	1204999	887478	1198021	1186068	1217534	1111966	1514881	1150182	887485	972028	1096365
fpHILIC	Positive	227.1250	1.80	unknown	27517252	20187306	20602202	26651410	21628300	31516220	26115820	21011746	25940060	24711712	22829586	22966058	26505310	17985766	22432112	29635742	22127286	21649574	18387006
EIC	Negative	463.9667	3.75	unknown	102899	130662	70284	103198	122965	116649	109140	84374	106847	78095	118441	97669	91469	99377	111023	127425	90818	95941	77048
fpHILIC	Positive	264.1497	3.37	unknown	1908170	1207218	439536	287117	674720	1092122	822019	965538	1325364	281576	484010	628529	810610	1135203	1125624	1308762	609435	878693	368313
EIC	Negative	568.3623	4.78	unknown	0	0	503960	339148	423534	0	0	397741	296595	367076	0	0	0	456058	0	454496	0	0	417557
EIC	Negative	366.9415	3.64	unknown	327090	565307	354714	448510	520358	524867	485180	345132	480442	325273	492721	459970	565598	487643	547179	317123	407546	363731	448497
fpHILIC	Negative	279.9955	0.98	unknown	1210841	1157642	972763	878935	1509110	1844447	1689220	524302	329699	0	0	0	0	0	0	36181	0	0	0
EIC	Negative	244.1999	3.23	unknown	68059	52943	178832	158528	120534	92436	103793	178376	124741	104181	71997	71583	72464	88028	27449	58151	85678	124901	78015
EIC	Negative	435.2326	4.03	unknown	57383	21893	107255	240698	105784	63455	69187	184469	144110	120369	31927	88973	90960	30024	14774	106088	76215	187729	0
EIC	Negative	290.8484	0.66	unknown	43916	112302	162836	143366	116180	60322	106875	203823	139639	100122	114793	123325	157794	42870	83991	92028	136095	169815	94727
EIC	Negative	307.1565	3.16	unknown	936800	446595	923156	765667	854035	462841	792348	1200363	590884	864394	357765	348752	412052	341370	467035	488829	550201	214495	321787
EIC	Negative	373.2058	3.94	unknown	60237	0	412139	26623	44838	336968	198908	28053	45183	35804	32973	336321	365151	251401	86803	235998	54581	73611	162021
EIC	Negative	379.2472	4.72	unknown	515700	394253	1019793	1140966	833136	574253	716253	1112737	882449	756800	476813	617834	573213	646313	167983	736317	595800	848888	470496
EIC	Negative	275.2016	5.81	unknown	8717824	21739927	14025785	62472529	27006028	13059648	21116356	23034657	50943855	14009608	12231552	11392167	25121787	9356231	10804597	17635606	24188286	13859154	7762502
fpHILIC	Negative	165.0555	1.62	unknown	149814	0	535174	511472	1220665	240676	0	613437	234262	413317	0	0	300346	0	602611	0	485944	708174	478788
EIC	Negative	247.1703	4.79	unknown	1008459	2053129	1962818	8904323	3030126	1368430	2327742	3127158	7522922	2331713	1166623	1443760	3777195	821012	672852	2885866	2962766	2839906	1026656
fpHILIC	Negative	174.0123	4.59	unknown	2130206	1670039	1668406	1825211	1772846	2345632	1926923	1525790	1884933	1367945	1792432	1672900	1687812	1522652	1467284	1776646	1490688	1683409	1702485
EIC	Negative	299.2234	2.87	unknown	70836	72704	109597	101992	117011	84273	67915	141347	52783	127944	71679	60674	63961	55201	45411	52643	68727	134647	66437
EIC	Negative	305.1749	3.44	unknown	1057150	370011	772495	268305	659435	602449	801860	648718	203953	316634	173470	495350	170901	336807	481772	182546	400329	273056	144310
EIC	Negative	252.1306	3.23	unknown	54808	79625	108333	187433	137656	74055	122126	92716	142831	69240	68599	116351	88718	50906	48340	81954	90313	80385	86194
EIC	Negative	571.2871	2.74	unknown	419443	297190	271644	327436	326019	349312	261233	362170	394750	0	0	0	80705	0	0	0	0	73246	0
EIC	Negative	458.3494	5.67	unknown	756978	565430	587854	1178804	615490	723391	576143	728592	1102443	573931	626144	586000	696933	305107	500740	706387	381450	510939	349126
EIC	Negative	227.0900	1.25	unknown	27326	35554	28092	60090	37311	48209	52318	29099	30101	38824	34701	66594	45864	46662	35197	32556	40380	33655	26365
EIC	Negative	609.3876	5.74	unknown	352429	447281	395416	288535	393965	530796	461852	390512	215432	282764	368989	361480	282612	269961	283523	233468	348495	276598	363642
EIC	Negative	286.2103	5.79	unknown	28432	36072	76263	167642	125720	32343	85415	54153	91183	60356	24203	69907	56473	12183	0	35321	33696	78388	20913
EIC	Negative	628.3531	4.43	unknown	12240520	15323859	12033404	12565585	13971551	13665235	14200904	12199214	14060299	12156844	14948833	13656960	13805658	13212215	13254149	13633678	13446956	11700829	13374312
EIC	Negative	361.1659	1.76	unknown	324378	408958	319110	323269	364210	334620	410079	335954	344472	329184	355695	495223	592609	416197	411281	467974	393660	0	366322
fpHILIC	Positive	789.6135	0.44	unknown	1826080	1626748	2050454	1838191	1928201	1900507	1716363	2453090	1333472	2065130	2159518	1850662	1440687	2299464	1960002	1424825	1883888	2104289	1760149
EIC	Negative	367.2436	6.43	unknown	4125254	0	5068332	6476867	5112098	5740210	0	7733875	5358982	6023207	3555268	4686516	5369749	3983709	3487248	4611122	4002111	4542637	5360547
EIC	Negative	283.1921	2.18	unknown	398963	272323	271366	321763	300734	423315	336230	285943	232070	390398	199124	260593	213393	184692	255115	246149	245129	369760	233188
fpHILIC	Negative	148.0335	3.97	unknown	856914	506949	520462	936637	702609	954234	700960	615358	622924	574738	439677	488031	484818	447446	536356	555396	607850	640607	513566
EIC	Negative	356.2471	2.04	unknown	3631420	212430	3153234	2660461	197596	4537063	211678	3105309	4118913	3285863	191090	186962	4370650	3909017	4296938	182270	4420609	2467856	176498
fpHILIC	Negative	198.1138	0.47	unknown	962438	807541	719398	1712725	1028286	2102947	1311976	893292	988586	1063326	1160599	1382601	1265896	462526	981901	1153981	1096609	902162	794735
EIC	Negative	567.2265	1.83	unknown	549957	148651	148430	0	294689	510523	286879	0	71292	0	0	0	0	0	0	7254	0	0	0
EIC	Negative	323.1843	3.96	unknown	233706	219605	164830	163807	201087	163292	171204	300206	146711	228673	74616	160326	160241	137451	162969	180545	163631	169908	206014
EIC	Negative	255.0664	2.42	unknown	846725	167107	2610656	375213	4217198	392751	378080	380966	354648	7961285	603959	952698	363828	355997	70872	239078	352921	973399	439143
fpHILIC	Negative	295.2638	0.37	unknown	1092548	1177049	2196912	2398004	2136441	1673586	1562498	2183981	1555150	1185273	970846	1457011	1435257	673001	918704	1262850	1314190	1670483	1264900
fpHILIC	Positive	174.0872	3.28	unknown	1981839	952263	1081935	1083583	1512776	1724153	1306117	1150537	772233	1171937	870581	900955	739896	1138415	896554	740559	811072	832497	1027248
EIC	Negative	447.3129	3.38	unknown	433233	150351	452905	209508	268163	255266	260957	590299	173812	186277	99817	125006	121084	160089	191293	162832	149046	387083	120749
EIC	Negative	385.3166	6.36	unknown	784024	682295	559561	943668	812422	885531	841053	844913	538211	450701	568824	580921	585510	361108	500795	539099	514307	568917	409775
EIC	Negative	612.3317	4.43	unknown	629727	865775	581649	642878	685753	700380	734885	595613	797648	626020	715895	722894	692414	741003	686354	722224	674830	617093	713008
fpHILIC	Positive	303.1272	2.07	unknown	2524415	227662	721009	158340	1082149	1403657	539449	126399	1137947	192162	0	0	883661	1415314	0	0	1273764	0	0
EIC	Negative	296.2313	5.17	unknown	697511	430082	740619	782255	459617	567347	708653	692843	1089648	477080	377974	646164	606758	362223	358438	778175	608127	731700	495349
fpHILIC	Negative	242.0436	2.72	unknown	372390	482227	622068	643151	496658	474829	623440	577741	478567	763321	542271	423066	525763	326380	400615	498344	563928	435542	433895
fpHILIC	Positive	185.0324	3.44	unknown	808030	870899	1048632	1146979	934453	949271	920783	1292990	838039	979406	927610	824139	907911	810576	957945	920719	965902	1198340	633929
EIC	Negative	269.1403	1.50	unknown	141037	203999	0	114731	192252	142376	171624	0	201712	0	110512	131006	164728	120542	160507	240104	154382	0	128150
fpHILIC	Positive	366.1080	3.52	unknown	301924	1963231	1743662	493036	581789	537887	1431633	834293	1983364	344187	300359	538641	551520	2033979	545786	432993	1100590	594546	0
fpHILIC	Positive	314.2321	0.61	unknown	1057983	452923	749165	1886236	862500	770492	1344967	1044376	1315177	682145	677926	796866	679379	135374	534653	669511	237752	1432454	635794
EIC	Negative	277.2076	5.81	unknown	200432	477592	320103	1388837	565335	305853	470378	525875	1112652	322956	283670	276194	556423	213715	256400	418070	538792	309383	173416
EIC	Negative	283.2283	4.30	unknown	112727	85894	218155	319870	200124	109679	131554	282975	226786	145581	85175	111092	161296	56587	0	160752	158772	184793	85063
EIC	Negative	577.3757	5.17	unknown	1712376	823658	1203130	1133449	1749827	1319336	850918	1700752	786123	1072341	946750	607697	824635	436845	584814	532860	823324	1163991	789212
EIC	Negative	381.1715	3.45	unknown	169902	43411	76478	89093	34406	137585	33269	260001	24810	11504	14832	25973	0	19302	17732	0	12885	31890	23317
EIC	Negative	244.1817	1.85	unknown	203814	136216	0	0	136010	140720	135363	0	109576	0	92627	94908	125458	90088	106031	160750	99472	0	112065
EIC	Negative	490.2876	2.74	unknown	1063582	548852	988351	807763	821173	894719	552883	1044425	1048252	173463	0	0	226863	0	0	0	0	180820	0
EIC	Negative	387.1760	4.91	unknown	217102	244202	383009	928127	558837	203479	446022	469946	576775	337712	156756	348890	314707	155741	54789	286245	273281	482376	155037
EIC	Negative	353.2312	6.04	unknown	351757	687439	673370	735208	633673	496349	547429	789202	612391	557627	403955	461197	470921	483590	312018	406047	416037	630676	485879
fpHILIC	Positive	302.1242	2.07	unknown	16709109	1766341	4908093	852513	7327323	9483466	3319666	602482	7556174	1175148	0	50946	5667178	9397835	0	69438	8687089	59090	48222
fpHILIC	Negative	368.9951	1.08	unknown	453728	456787	547677	308124	357233	627274	387138	358995	346515	420846	129050	402167	323633	309240	194452	212579	358281	359178	180948
fpHILIC	Negative	240.1691	0.38	unknown	572737	291122	492727	2392334	1004175	664814	761914	980642	1589750	1137920	689434	1017133	1118158	0	160281	767149	720230	1516770	0
EIC	Negative	390.2942	6.43	unknown	8665932	7770323	7556979	10608422	10163407	10703957	8726439	4833768	10915191	10362621	6926441	7611077	8023737	10125748	9267149	8439479	7824517	5818301	12353582
EIC	Negative	352.2211	3.55	unknown	76619	58960	146168	153169	118032	80410	96945	193134	90623	117767	66257	73219	61855	90589	48160	51699	65230	146030	52690

EIC	Negative	409.2865	4.44	unknown	0	387679	442122	512907	0	243069	472088	516401	547360	0	31659	37376	0	0	0	0	0	194941	94611
EIC	Negative	435.0356	2.09	unknown	77818	151675	0	0	120054	114611	111436	0	100450	0	89986	12575	44397	57128	72323	83075	0	0	56701
EIC	Negative	305.1747	3.46	unknown	1015241	340605	739778	433531	627585	545138	739338	1002850	240045	597788	270416	462756	314473	318649	425894	355666	355168	752188	268386
EIC	Negative	603.4632	5.82	unknown	67936	209444	122636	510841	204366	154031	260850	226408	381187	89704	102681	62167	171364	107095	132424	62338	196379	0	52501
EIC	Negative	278.1841	3.32	unknown	0	199535	0	324387	0	369261	269632	0	10337	0	262658	382407	0	0	0	0	0	284714	0
EIC	Negative	256.0698	2.42	unknown	130631	37830	455786	68927	690309	65602	65089	68096	73399	1314362	103180	167371	72705	74854	8886	46270	67800	173321	84577
fpHILIC	Negative	305.2492	0.37	unknown	7308564	13572176	13399585	13122711	11086642	11200185	13286101	11991991	13170069	10157987	10704064	10849193	10930309	7101118	8490821	7641659	11975655	10668283	9916221
fpHILIC	Negative	337.2050	0.35	unknown	860952	401571	313747	412602	1113748	274242	662088	244052	110279	751580	427776	364946	629585	340310	304004	401722	522149	455089	432364
EIC	Negative	559.3376	6.11	unknown	745501	865678	747936	908381	817009	827867	900394	796657	774453	805239	815133	917415	833021	865448	821880	764032	853197	715268	812946
EIC	Negative	360.1536	2.09	unknown	2765737	1926263	1163496	232557	1823306	1878469	1979918	1702638	1954405	1298068	2038880	12104	474055	1052062	1399884	3724687	73974	418236	1052447
EIC	Negative	288.2052	6.21	unknown	75928	108223	111637	192633	148171	111842	120530	158768	114056	146971	88071	107241	129164	20733	56633	127179	109250	128464	69401
fpHILIC	Positive	511.3583	0.59	unknown	1320609	1505558	1215357	1239707	1375860	1765273	1589925	1296178	1351212	1249651	1376730	1313628	1709398	1211149	1500155	1323790	1500109	1379825	1499304
EIC	Negative	268.1272	2.93	unknown	170028	239481	88927	77371	149263	218444	178720	85925	115387	136181	152353	135748	129652	160079	192525	158975	130058	88896	104192
fpHILIC	Positive	112.0507	2.61	unknown	6033146	4267182	3035410	6500643	4050499	5820295	3938005	4316576	6112507	4367403	2825918	5407199	4608123	3463926	3786644	6408438	3594985	4364396	3470995
EIC	Negative	306.2413	6.44	unknown	648209	821298	126879	1151781	836825	581274	597092	705824	885004	590935	625173	700576	712658	607965	596501	650491	705774	740625	633682
EIC	Negative	556.3526	4.84	unknown	8555075	10767773	8452339	8686026	9081617	9126343	9278696	8705943	10307848	8300008	9788665	9517261	9460787	9766580	9732586	9470855	8896957	7542648	9690848
EIC	Negative	634.3356	4.04	unknown	253231	526879	239142	132695	380503	472520	450271	250955	248179	275638	416680	434335	367074	431140	475314	303485	415534	240473	354622
EIC	Negative	300.2627	6.48	unknown	963423	1985599	2243544	2417378	2307256	1328855	1468930	3263085	1637794	1366129	987357	1053232	1684334	1569463	786281	622213	993159	1863300	1715483
EIC	Negative	630.3690	4.93	unknown	1308539	752222	1156808	1314074	1110008	2013725	1656642	1220049	301310	775609	1104550	1402509	702372	517258	1132145	548002	961677	856321	1002434
fpHILIC	Negative	280.0519	2.61	unknown	857099	559376	415284	815305	555724	828052	538484	639163	785694	720698	478049	856342	782419	484003	606480	658350	622731	663338	567479
EIC	Negative	359.1503	2.09	unknown	12655604	8519135	5192382	1184771	8108874	8289235	8818078	7721607	8871301	5734228	9033383	113131	2071811	4789466	6376304	16692639	605401	1572012	4486005
EIC	Negative	266.1842	3.31	unknown	459354	158304	390231	0	283825	272112	293256	358657	108373	450037	233808	364339	283193	130865	188895	260534	175914	444820	158990
fpHILIC	Negative	160.0332	3.84	unknown	461562	355844	442713	364806	387527	697680	397546	414921	248157	284292	548437	309120	296262	1493281	426282	304204	314104	290358	523903
EIC	Negative	272.0649	1.64	unknown	160255	25928	0	0	24202	115556	64519	0	177771	0	206454	202354	77175	33885	0	30259	57930	27261	88818
EIC	Negative	311.1861	2.50	unknown	138196	101261	119812	217347	110041	99893	163233	164328	173671	138098	74562	89387	136275	82249	67084	194693	125162	190890	77628
EIC	Negative	504.3153	5.65	unknown	842331	1386339	1064142	727003	1157744	2076242	1134342	968772	519956	429374	0	922029	599522	1502661	1143099	592752	914726	726659	1368838
EIC	Negative	287.0567	1.04	unknown	136047	45089	172540	46068	160863	181722	101327	49494	322695	112505	201882	354767	108945	56548	14498	36351	77120	115310	116572
EIC	Negative	298.2470	4.72	unknown	1314416	1050748	2864168	3641175	2241317	1628712	1872121	3394946	2642843	2022327	1169606	1753706	1644684	1773209	727892	2110515	1728482	2665408	1170109
EIC	Negative	594.3777	6.00	unknown	4432370	5919117	4513978	3973612	4773324	7335721	6730006	4516931	3215180	3278757	4156943	4685033	4018301	3638836	4690287	3323219	3939447	2817517	4020675
fpHILIC	Negative	225.1863	0.39	unknown	4694878	5759202	10387520	20633700	12423045	6028466	11576698	9834046	13122639	7383522	3277902	8073364	7723040	2813954	2319590	6451487	7123730	8971966	4800954
fpHILIC	Positive	571.4280	0.41	unknown	69019	139752	133521	272529	695000	182718	302048	205319	419515	403697	397519	752671	170493	379609	343321	290908	568399	208096	287476
fpHILIC	Positive	201.0865	2.59	unknown	829102	465882	748815	818707	626344	1027845	695330	877297	415857	650700	654215	665361	524404	625517	568198	775154	531089	596767	563908
fpHILIC	Negative	495.1908	3.51	unknown	91000	796103	471478	137838	218764	203113	412921	280091	699748	138186	191006	182561	212391	666079	182408	167697	328020	153017	949399
EIC	Negative	456.4143	6.35	unknown	944332	861150	1833639	2375145	1363787	878838	1874374	1914826	1469064	1317905	613572	1242292	779618	477516	430497	595084	903603	1188443	531772
EIC	Negative	435.2698	5.41	unknown	208367	409941	203001	482163	58841	227646	194278	370670	783412	294535	307883	135433	363904	454684	401099	65962	414329	159262	172333
EIC	Negative	390.1955	5.79	unknown	102446	147938	214744	374359	255421	136201	230754	217858	230976	175927	91546	260953	175674	81629	30051	118186	138736	188501	94982
fpHILIC	Negative	153.0444	0.52	unknown	3388743	2078635	1438495	366728	2023286	1865122	2072128	1921300	2476144	1611340	2071233	0	597100	1128028	1263917	3711376	284354	692188	1500274
EIC	Negative	331.1902	5.58	unknown	1238970	1526302	2154846	5298512	2334070	1434194	2166011	2809626	4092930	2128167	1207133	2105080	2499774	937558	1008313	1983728	1934648	2682013	1234796
fpHILIC	Negative	149.0279	0.78	unknown	496004	324909	314944	180701	219609	981189	139802	208612	160344	181624	225344	288612	222892	580945	411990	98300	200410	191854	430446
EIC	Negative	552.3342	4.04	unknown	4503874	7865543	4681447	3731500	6544401	7705164	8895311	4689239	4295048	4630478	6937214	7297927	6605395	7337967	7645255	5568080	7240078	4238681	5900819
EIC	Negative	319.2711	3.30	unknown	235747	271612	241548	236884	245014	247859	271451	232309	237023	244448	230072	273469	249383	229167	256971	236920	242835	218058	246267
EIC	Negative	569.3659	5.55	unknown	1613104	1930896	1484670	1584614	1841090	2224983	2037336	1656705	1415150	1261280	1491253	1622431	1691209	1423852	1864006	1446332	1529784	1076007	1558928
EIC	Negative	630.3691	4.78	unknown	703264	1151278	566521	642699	851365	693065	890862	633956	1028355	561100	828860	692765	611480	768900	577630	682216	723678	490279	675091
EIC	Negative	273.1245	3.38	unknown	19464	50136	88585	179970	97943	58734	77478	93084	103700	72118	44600	78573	63725	26325	12283	71315	52010	110294	21447
EIC	Negative	322.1776	5.79	unknown	139218	253897	319977	579801	390168	214946	387708	341997	366239	249728	145405	343255	291334	94273	84391	220689	196952	286730	113702
EIC	Negative	325.2026	3.00	unknown	246062	148394	319593	500958	288998	219121	239514	421398	349028	352163	174995	318088	266935	116441	89326	240443	261503	442829	128071
fpHILIC	Negative	327.1453	0.57	unknown	357783	316182	987650	148256	349146	490796	365820	562164	405889	207416	976439	665899	417903	991197	108272	522559	272802	188428	523160
EIC	Negative	319.2785	3.76	unknown	261953	314220	249418	285448	289645	300739	277452	262612	274537	286472	278048	281202	267631	278033	258436	282630	284063	277491	291379
EIC	Negative	255.1969	3.69	unknown	397636	379252	500171	1290753	856679	454845	789724	794503	699043	691438	446753	676572	664600	143313	148659	590970	585911	772404	199396
EIC	Negative	392.2237	2.67	unknown	0	89917	109992	0	0	0	122692	0	103723	0	0	0	147590	125090	0	0	78970	0	59765
fpHILIC	Positive	571.9316	0.41	unknown	107418	114143	49882	194773	493480	127224	209819	153104	319469	312780	362467	613801	101221	279175	157172	144177	357959	169538	197743
EIC	Negative	516.2860	3.57	unknown	0	218307	0	223108	24678	0	29105	236427	220822	198069	43940	20387	0	178774	26226	0	311323	0	0
EIC	Negative	471.1964	5.79	unknown	143777	192416	232242	308915	240015	168306	215570	238499	250245	216058	140953	225650	189965	92347	68617	151819	154665	213719	118621
EIC	Negative	326.2720	4.72	unknown	3004597	2771667	2948132	3070538	3100579	2886210	2831625	2949688	2914218	2982961	2827685	2871577	2963432	2820039	2910936	2982994	2774362	2841270	2638933
EIC	Negative	301.0720	2.37	unknown	103103	23796	0	0	57145	9023	32632	52536	78728	0	19071	0	0	7253	9232	79285	0	0	17611
fpHILIC	Positive	704.5779	0.48	unknown	1935401	2083215	1650443	1735920	1857104	1536670	1563446	1915904	2223737	1536991	1749640	1348452	1357158	1890048	1811861	1528716	1676796	1414095	1484597
fpHILIC	Negative	267.1428	0.36	unknown	352426	587293	1140875	840185	1408736	618310	570879	400987	285850	885214	752776	551745	876063	477710	431606	735050	1150015	364243	429453
EIC	Negative	433.0135	0.55	unknown	153204	102744	78074	218038	70757	215021	186478	119092	65424	43286	86921	108000	87006	286526	82780	74650	81283	98237	52994
EIC	Negative	322.2440	6.22	unknown	0	0	310157	507854	181597	183836	123810	245971	178091	321875	142320	0	0	0	0	144143	0	0	0
fpHILIC	Positive	188.1275	2.57	unknown	5415737	2828866	1881810	4919850	3398730	3635143	2504813	1696467	6972638	3784611	2364576	2153665	2581145	830100	2634068	4179124	2914170	1660717	1401063
EIC	Negative	553.2244	0.71	unknown	39920	77142	91871	69315	51288	64297	76844	100505	49107	48315	77590	73128	73017	24232	55119	48783	60512	72176	46077
fpHILIC	Positive	780.5511	0.44	unknown	2449996	4450988	3130384	3113300	3087098	3221901	3885291	2773357	5303017	2077442	3116587	3256210	4008215	4948201	2989374	3342481	3670673	2408275	3137442
EIC	Negative	249.1496	5.30	unknown	630268	624953	1139337	687409	864023	651699	970785	575235	697106	890648	1017729	799443	680222	599710	958707	668954	732642	615315	565355
EIC	Negative	631.3720	4.93	unknown	232364	142657	197233	246144	208446	355294	280972	196286	53214	146627	196729	224234	116958	80798	201517	96064	180327	151995	166256
EIC	Negative	270.2159	3.55	unknown	705388	486097	1620508	1719649	1215323	773356	986061	1975545	1027114	1126149	593288	782447	667199	791220	363237	464798	811686	1454890	669208
EIC	Negative	629.3660	4.93	unknown	5794908	3069650	4805040	5720663	4925934	8650451	6922573	4704164	1254781	3363149	4708432	6283646	3144051	2172561	4888089	2328272	4247096	3659664	4246263
fpHILIC	Negative	869.5726	0.42	unknown	1874405	2034154	1402944	1492236	1882152	2142300	2147543	1782967	1313787	1690965	1543807	2042662	1867941	1474725	2240546	1592023	1936874	1377653	1537388
fpHILIC	Negative	117.0558	0.78	unknown	9106353	6425947	7543286	10491907	8102831	13367199	7201719	5871845	8394170	7550115	7335254	7237963	7924507	6360451	7142967	3260037	6845374	4922096	7308368
EIC	Negative	563.3887	2.71	unknown	363745	140345	277736	218872	227833	422903	207713	227068	171176	200454	160375	192329	225444	256304	141545	167591	212761	219826	205473
EIC	Negative	517.2761	3.42	unknown	151861	84294	92660	0	63871	122216	56575	114049	98419	110937	65626	92727	75948	75452	93061	81459	127608	0	128948
EIC	Negative	227.1286	2.64	unknown	378088	262537	204846	64938	243329	589727	221731	46152	36184	160658	172020	147381	90887	120678	217233	89609	76655	45576	146241
EIC	Negative	517.2378	4.13	unknown	74493	70306	102217	134043	99051	82047	66392	149221	106051	102332	73309	69863	61032	128282	0	60202	87576	103189	68182
EIC	Negative	283.1531	2.67	unknown	66379	132686	0	0	79112	128884	0	0	122353	74183	0	0	218062	135090	0	0	171216	0	147576
fpHILIC	Negative	191.0056	4.72	unknown	1109975	499733	700023	884880	666721	972353	728230	760641	749006	677027	575720	644363	749067	710584	558852	793807	584651	700693	627181
EIC	Negative	361.1568	2.09	unknown	486172	319116	252276	0	304016	304361	409708	293974	334753	260740	413712	0	102129	183973	309454	594825	0	0	215585
EIC	Negative	242.1761	1.85	unknown	13027709	6968882	2829873	635882	7219720	8913218	8808337	657492	6372070	767828	5634335	5473288	6605111	5359763	5926036	10193772	6074738	789098	7320646
EIC	Negative	628.3627	4.93	unknown	16142427	9008286	13362935	15909545	13654774	24492866	19121588	13356318	3430208	9431233	13190442	17392625	9476884	6008493	13482501	6504003	11993504	10278301	12163756
EIC	Negative	249.1861	5.31	unknown	117550	663913	712633	3490007	1607636	368722	777420	1160806	2391253	747721	374411	354149	1645379	161721	90507	1126132	1660170	697681	234628
EIC	Negative	431.0982	1.42	unknown	1225732	272048	0	0	100524	1047572	517015	0	204303	0	448038	306447	191051	159125	40043	277779	724618	0	299441

fpHILIC	Positive	323.1264	4.19	unknown	1305656	341995	254451	122859	587646	1324803	450194	101814	240068	50840	92198	132083	67463	437848	85710	147916	55706	227801	49295
EIC	Negative	309.1526	3.78	unknown	789828	263684	687741	307625	474578	467400	499556	1021169	116096	0	357865	274977	230911	147381	308295	236428	293370	645679	365420
EIC	Negative	235.1705	3.05	unknown	360808	380108	345921	338341	375054	334276	394037	361926	321028	353132	335775	340925	335652	342395	321274	400288	346941	356327	348692
fpHILIC	Positive	395.1617	3.72	unknown	669572	592082	644442	575492	738076	597465	589591	577667	560768	616153	580082	568632	551488	397155	669587	558120	552338	590104	410072
EIC	Negative	303.2249	4.96	unknown	190393	0	0	0	0	0	0	237543	0	171893	193192	174600	212763	86876	0	0	0	0	146980
fpHILIC	Negative	136.0165	0.52	unknown	1072258	712508	667593	129336	646850	703752	925806	600881	973843	740544	770475	0	220632	394937	384924	1179612	59991	135717	562927
EIC	Negative	503.2984	5.13	unknown	1922339	2587377	1757499	1479969	2384649	2560550	2117175	1684759	1637608	1095740	1807960	1387358	1373045	2302040	1524745	767536	1698377	1213711	1766624
EIC	Negative	588.3326	4.52	unknown	802901	870121	743662	743271	802477	833632	737828	785382	849429	740082	863332	810234	860065	796085	797797	815465	740429	768179	816387
EIC	Negative	352.2207	3.47	unknown	55480	21148	68255	214185	80262	58418	59826	146897	121525	85145	57819	84966	77480	21846	21463	76279	56706	137578	23453
EIC	Negative	285.2073	2.37	unknown	528563	261926	392838	421670	379782	420619	391545	545289	218638	370819	209781	290280	223218	180065	194759	244674	223586	471056	205039
EIC	Negative	562.3844	2.71	unknown	1023529	397515	710575	570990	622641	1266604	572197	564809	444251	547111	470842	519101	632565	653703	430634	486825	590962	666883	500649
EIC	Negative	252.2046	6.28	unknown	904534	1260894	1983430	2763649	1758442	1412543	1941373	1811577	2222888	1574161	978817	1645331	2037580	791115	819114	1148987	1372734	1839947	961354
EIC	Negative	490.1993	1.03	unknown	96381	62526	75439	38725	57668	77639	100461	83736	69046	161812	70733	82783	68150	116182	52921	56781	52064	69028	139187
fpHILIC	Negative	129.0193	4.58	unknown	10697994	8248063	8294852	9614712	8999072	11609105	9246608	7341168	9320008	7134938	8858671	8278941	8185928	7898191	7671312	9417301	7974378	8501163	7434358
EIC	Negative	375.2168	5.02	unknown	239779	319864	269792	720993	392296	362778	418513	381446	500706	376511	315324	468804	406230	218295	235490	388131	346973	470635	187021
EIC	Negative	609.3650	4.04	unknown	449010	39887	615716	207752	174222	106563	93167	1047962	108589	293256	16284	52565	77457	107169	115039	21942	108282	348693	98059
fpHILIC	Positive	292.0725	2.43	unknown	504402	238432	1062424	143221	722033	586334	236891	55145	477573	143267	0	0	701844	740074	0	0	1015190	0	0
EIC	Negative	629.4762	6.17	unknown	516164	554326	604630	2459497	825900	768937	1487543	1114737	1142931	487565	443207	499909	662312	240937	361701	340293	655838	569409	252268
EIC	Negative	459.1509	1.48	unknown	0	842973	0	0	370839	378783	390254	0	338008	0	353376	288685	354804	382512	416704	293631	330105	0	226469
EIC	Negative	460.1545	1.48	unknown	0	185541	0	0	83754	84681	84558	0	77859	0	79085	64190	86239	81931	90250	67191	81217	0	55012
EIC	Negative	243.1794	1.85	unknown	1900683	989935	492209	0	1053644	1309841	1286769	0	927351	0	846147	800475	952315	781813	862083	1493873	874015	0	1069766
EIC	Negative	367.1401	1.78	unknown	261323	186435	206867	314189	276165	331779	299459	74238	274102	264597	235228	280019	283048	150813	283291	385836	249148	0	128100
EIC	Negative	322.1442	2.51	unknown	221221	112962	197036	170244	176183	133935	165300	276063	118908	176813	95882	112682	104249	100563	121828	118700	108594	192518	87463
fpHILIC	Negative	193.0242	4.72	unknown	2098778	1460524	1397997	1743133	1542582	2090956	1522782	1455647	1786144	1422226	1432553	1470109	1436515	1493447	1253158	1892401	1397819	1520139	1407218
EIC	Negative	568.3626	5.55	unknown	5391718	6267623	5128172	5235829	6013102	7206981	6636191	5634432	4831953	4084208	4984648	5499284	5459920	4623975	6056321	4701260	4966584	3572438	4866386
EIC	Negative	394.2622	6.44	unknown	2089748	1331919	527310	1084556	947629	1329386	1591038	1532871	1068298	1130731	1212768	613649	939227	1121367	1165755	651258	1192743	704563	714546
fpHILIC	Negative	227.2016	0.39	unknown	21927522	25714652	59758540	75810808	42874628	29223602	43582996	59401480	54174992	34852992	20557826	30622552	35871612	18030686	13975875	25999374	34000880	47306868	24544862
fpHILIC	Positive	654.3314	0.40	unknown	2719790	3096371	2096640	2222331	2707703	2168952	2456173	2782865	3171588	1614628	2357421	2523043	4435697	3710014	1012041	2721140	2277825	1819031	4675252
EIC	Negative	283.1919	3.46	unknown	14850843	4282461	11490539	7647764	8725350	7910534	10146010	15038661	3616473	9391998	3971768	6485776	4515368	4200027	5398993	5286911	5193366	11326929	3442740
EIC	Negative	293.2125	3.46	unknown	135065	105171	206662	392497	165957	120938	180174	248385	345821	208804	120050	245560	194997	80915	90559	336150	185518	249677	124091
EIC	Negative	256.2000	3.69	unknown	81937	58222	41081	235042	139419	73717	134935	123897	122634	129301	69759	120839	107465	35054	21702	102738	107165	138277	36162
fpHILIC	Negative	255.2329	0.38	unknown	224955440	343397216	461329568	582365440	376129408	343023264	379646080	426973824	522734688	286097248	284759136	353196928	389244576	279387104	256241216	335271232	342267328	357533696	325501408
EIC	Negative	366.2665	5.93	unknown	211386	0	0	0	0	20956	0	224870	0	0	167586	0	0	0	0	205033	0	0	0
fpHILIC	Positive	131.0892	2.71	unknown	820460	975098	702653	1071078	956182	1239589	953106	628912	828286	642062	1124279	810955	927312	490503	670446	1020415	1017817	740370	641668
EIC	Negative	301.2601	4.40	unknown	239177	168163	215653	211488	221000	212677	211054	225392	170299	213335	172287	187026	217557	185973	204220	230765	159153	216017	184649
EIC	Negative	311.1648	3.67	unknown	290698	276145	340192	301336	119814	839038	203856	169009	77264	282372	285833	389671	489707	292628	136112	338332	241454	352376	297837
EIC	Negative	284.1954	3.46	unknown	2605401	759963	2097452	1357173	1560380	1412548	1772074	2677272	651280	1707431	724401	1152366	797863	760282	965113	922543	935076	2027722	605658
fpHILIC	Negative	848.5942	0.43	unknown	1560601	1199881	1382080	1301425	1409889	1506118	1035654	1617082	977075	1313012	1211482	1395721	1050190	1349291	1415712	1004547	1266047	1251355	1398279
EIC	Negative	485.3033	2.88	unknown	260682	23623	229081	38124	67053	37005	62861	412600	52107	94615	19418	30870	22752	56836	32319	53288	14815	225622	24011
fpHILIC	Positive	309.1285	3.48	unknown	1420191	1481713	1517223	1418616	1238152	1356688	1610547	1337293	1313183	1511502	1297967	948948	1352794	689436	1223215	1065984	1426746	1568816	1110897
EIC	Negative	330.2522	6.49	unknown	13907329	3819816	11912751	27235358	13153525	13468292	12453039	20042810	11590786	14607104	12202137	11081306	10133475	7209274	10922170	10055406	9480219	22310220	11804713
EIC	Negative	351.2166	5.19	unknown	205677	364795	332160	1076041	521429	317530	482193	494827	617448	535553	357058	669776	446761	172026	168691	382666	366743	664980	192709
EIC	Negative	489.2839	2.74	unknown	3645199	2016451	3215548	2931090	2893557	3397146	1756222	3454630	3295870	582821	0	0	727710	47535	0	0	0	431341	49004
EIC	Negative	252.2049	6.31	unknown	490874	840104	1420327	3102911	1438438	557005	1332676	1303799	1456002	917542	675660	1116787	0	352283	0	560269	926501	1549720	0
EIC	Negative	427.2711	0.91	unknown	143047	42100	128143	248096	69272	177724	61101	298009	42515	154172	63806	59095	74251	43088	53607	111630	64645	250513	51149
EIC	Negative	393.1727	3.20	unknown	124290	51141	76340	96393	62176	115964	87942	108276	41808	44368	47177	0	0	0	0	40053	0	41082	0
EIC	Negative	251.1322	3.39	unknown	112539	165948	0	0	0	0	240830	0	301066	0	145922	0	0	0	0	0	0	0	154204
fpHILIC	Negative	153.0307	2.64	unknown	417264	370354	315127	501927	395318	534620	397468	308198	348706	417783	506053	391824	406727	265111	389303	341351	411863	323901	316387
EIC	Negative	271.0615	1.64	unknown	840151	160966	425041	0	131143	653877	358922	92864	1110947	318740	1215961	1179548	400724	226700	53181	157849	267947	255876	447741
EIC	Negative	228.2047	6.35	unknown	13297570	16906260	29975079	36971892	23291498	18313098	21404143	31834751	26809374	16657985	10781891	16837426	17972260	10151074	7897365	13555992	14898719	22970012	13035393
EIC	Negative	475.3780	5.58	unknown	0	31651	126241	699740	173452	0	127210	257495	455942	109403	0	79234	123978	0	0	95117	95289	168160	0
EIC	Negative	389.1926	5.79	unknown	544686	758516	1090575	1848501	1266401	647672	1084636	1111462	1203770	902014	469291	1091490	816941	268580	247900	630348	688990	941798	402262
EIC	Negative	419.2818	4.23	unknown	404340	110513	150812	85203	164195	126650	170846	346560	150081	0	0	0	0	0	0	0	0	29288	0
EIC	Negative	306.1770	4.91	unknown	294709	364129	564392	1292432	768787	349558	614036	624005	748904	481974	254931	530665	445828	164825	170349	363292	382626	611573	221333
EIC	Negative	359.0079	1.19	unknown	214383	208141	170245	164358	190428	210934	218672	159666	167551	179804	193176	193610	175773	185834	185780	165927	194876	162665	193799
EIC	Negative	464.3022	1.59	unknown	2315525	105513	4664085	307003	196439	143011	90042	7583866	268912	168134	0	0	47489	8696	0	0	0	159764	0
EIC	Negative	405.2865	3.37	unknown	219243	88734	220374	87001	128528	187900	120243	273842	101672	183419	179440	57285	65929	405931	219186	0	189200	178789	84898
fpHILIC	Positive	218.1125	2.59	unknown	2207917	1090626	1725555	2148611	1890361	2122894	1730989	2223297	1071364	1662808	1360528	1335735	1321314	1593012	1490573	1680633	1062156	1593699	1220953
EIC	Negative	321.1745	5.79	unknown	792551	1221398	1668261	2794665	1945475	1054554	1782981	1786681	1697218	1265099	741444	1702007	1180966	414533	386811	1003159	1060026	1374354	601753
EIC	Negative	355.2432	2.27	unknown	244782	313358	267877	224564	298343	254403	290674	266242	243434	244773	288549	244481	270654	266255	278245	282032	251872	222131	264598
EIC	Negative	325.2183	6.37	unknown	477931	933820	401284	1213513	912229	723329	1099544	407754	822156	600473	409504	553600	1211213	170493	335129	916988	573902	435915	305809
EIC	Negative	265.2175	6.36	unknown	4873425	7138557	9605124	13903788	8604327	6893661	8030275	9673558	11067946	5073840	4734046	5700996	7161836	4540034	3791668	5548880	6264181	6333549	4273269
EIC	Negative	263.2017	5.88	unknown	424985	465963	486435	1018478	608728	392256	611183	547593	775612	400368	519983	267743	605289	324424	296659	366947	569612	543039	293659
EIC	Negative	332.1944	5.58	unknown	231627	294680	403038	1064539	497714	257107	387435	539572	767326	394939	233156	416144	468265	196125	193628	392596	388892	503102	248878
fpHILIC	Negative	256.0943	2.61	unknown	371717	388047	432204	253389	365752	338402	506202	242427	306654	368583	498693	456094	298281	272997	354697	465590	280313	247128	309299
fpHILIC	Negative	113.0245	3.34	unknown	22128474	18165736	20625830	21783878	20241188	21137812	22387114	20419540	19351668	20694332	22995754	18857554	21527254	14293142	18726930	19417742	19904960	19234128	18421806
EIC	Negative	553.3351	4.04	unknown	1384251	2356080	1314841	1134773	1766285	2261421	2439086	1298381	1186809	1311932	1935852	2107555	1947827	2060455	2047903	1700554	2114392	1223234	1738014
EIC	Negative	269.2125	3.55	unknown	3789925	2783026	9156641	9621161	6632047	4309650	5419230	10806390	5672702	6332091	3372987	4269302	3780458	4425022	2074876	2643303	4455872	8130135	3821058
EIC	Negative	349.2136	6.38	unknown	378812	255864	573115	639551	314522	248096	342083	992404	271613	426151	282858	310693	276365	201949	342879	376457	247784	599880	282818
EIC	Negative	249.1860	5.58	unknown	18114191	24253856	32486502	98157136	39307802	22865894	34414507	46833838	67923486	31136572	18716318	32589491	37668684	14212798	14825444	29947249	30491008	42654547	18597419
fpHILIC	Positive	262.0901	2.78	unknown	362839	508309	337545	306432	376641	558794	440352	196203	268632	331682	464714	426248	307357	333874	457696	411356	308737	148429	303229
EIC	Negative	354.3025	6.49	unknown	336895	450564	1758175	2590487	642230	1288868	1258243	1337059	1427252	695559	506410	750925	1621130	595422	362146	868372	944231	1294943	406233
EIC	Negative	501.3223	3.43	unknown	55189	415651	0	4790244	971273	764732	976075	1653614	2234738	1750004	602875	1776702	1210284	154593	0	1239585	776086	1963863	231729
EIC	Negative	509.3494	2.87	unknown	2618570	193746	2130113	700508	547485	187519	571823	4251698	467610	869263	175945	215316	177747	387193	322711	237342	254940	2172157	232484
EIC	Negative	427.2624	0.91	unknown	122905	0	107370	217019	70073	144897	0	254357	0	0	0	0	0	14509	0	0	0	203205	50975
fpHILIC	Positive	703.5744	0.48	unknown	4314790	4417350	3893940	4162506	4476205	3479720	3439063	4221370	4954265	3201798	3691711	3111008	3261853	3787821	4135401	3324324	3851315	3286618	3665585
EIC	Negative	364.2138	3.59	unknown	104786	55059	142210	57205	70425	220310	60661	66949	37485	91689	81665	57029	64429	51294	77384	87248	96542	88527	81369
fpHILIC	Negative	159.0298	3.84	unknown	7056182	5609422	6782764	5342307	6099224	10010646	5504646	6036354	3683196	4301599	8331512	4956720	4336268	21899966	5946670	4864178	5110476	4282786	8026395
fpHILIC	Positive	392.1047	3.16	unknown	765272	857201	832638	886416	843372	931595	774066	857122	855038	976863	882248	903221	974713	719597	800043	771663	727209	839870	749606

EIC	Negative	359.2960	6.24	unknown	0	446170	473199	775580	586743	805407	591699	0	331195	507053	619924	602141	0	0	481563	489794	512898	0	0
EIC	Negative	396.3123	5.83	unknown	137740	167785	135850	162109	147537	171413	153010	139179	137320	122059	144507	146246	150658	181404	155161	146619	153068	129089	140560
fpHILIC	Positive	383.1152	3.16	unknown	846746	896177	917222	1029707	947121	957899	895443	946792	943368	973785	820753	952063	953202	742287	905795	863294	874087	775951	817775
EIC	Negative	227.2014	6.35	unknown	75122258	97313091	170436732	212991060	133726823	103908780	121232610	178442943	152849058	94235738	61413931	95063444	103112096	58829834	45634266	78815298	85481503	131038409	74253104
EIC	Negative	631.3817	5.49	unknown	3068160	2743447	2574129	2701057	2958308	3350334	3154232	2697365	2178534	1890750	2865810	2636429	1964995	1731694	1956475	1538424	2166731	1924086	2603279
fpHILIC	Negative	267.2328	0.38	unknown	3001711	4265727	6365380	7951091	4568555	3750683	5121410	6737265	6692151	3394876	3294624	3936634	5871614	2882715	2818701	4333353	4157062	4914749	3473405
EIC	Negative	251.1921	5.58	unknown	338008	499315	609985	1767484	720870	421408	612382	871556	1287058	567156	311133	597055	678813	283920	269456	544665	588189	788695	355584
EIC	Negative	630.3685	4.43	unknown	0	0	0	0	0	336824	0	0	0	287572	0	0	0	0	0	0	323250	0	0
EIC	Negative	305.1738	4.91	unknown	1657124	1996239	3076396	7417007	4225469	1844093	3582401	3510820	4132667	2474169	1374589	2960522	2552787	996808	748631	2020257	2213693	3517995	1253833
EIC	Negative	303.1574	4.34	unknown	261187	155021	277176	497795	319924	229218	438445	305274	337860	299860	211455	368622	394231	45822	118740	259441	222428	423985	100314
fpHILIC	Negative	488.1795	3.51	unknown	492842	3059485	2557427	571691	708203	802929	1830731	1096155	2771035	467606	439549	721489	757723	0	666096	511557	1194321	693121	0
fpHILIC	Negative	181.0870	0.96	unknown	672176	604114	1644321	513484	1080363	527606	1016599	780078	602427	1400534	1882137	1449445	941731	1132645	236788	626566	289019	432902	399301
fpHILIC	Negative	501.3952	0.36	unknown	1293307	1236913	1473555	996732	1571965	948103	1613088	1120297	1259681	931573	1164929	988855	937774	1009132	1438107	1370195	722138	992776	916513
fpHILIC	Negative	112.0516	1.80	unknown	8541685	6244391	6566967	8151410	6402431	9351515	7369960	6304341	7709298	6959581	7659322	7016937	7598294	5645068	7330535	8224784	7192747	6684468	6150990
fpHILIC	Negative	487.1782	3.52	unknown	2048647	15741919	12420957	2459468	2991668	3526757	9124012	4987148	13798916	1458823	1049384	3057774	3304245	0	2770541	1818093	5844300	3033769	0
fpHILIC	Negative	281.2486	0.37	unknown	288683040	424136544	594543616	699828864	445140288	412217824	530359776	524745984	621440000	362279616	311728544	437793824	461788352	269496000	309970912	397594048	426658944	507640928	323202400
EIC	Negative	249.1860	3.42	unknown	623525	241251	590678	542262	428202	289126	465251	655075	489193	458436	275990	333873	285278	176997	281134	342137	241905	420682	225850
EIC	Negative	555.3503	4.84	unknown	41855302	52799354	42548495	42129997	44251470	43640436	45375732	41824832	49011031	40288270	47797308	47384194	46138110	47627906	47217785	45831674	43321572	36933167	47887776
EIC	Negative	542.3118	1.80	unknown	141044	90801	138672	0	107941	113729	94933	103319	69912	96239	94452	140444	93956	110291	107588	71374	89314	0	91016
EIC	Negative	359.3002	6.24	unknown	670984	631322	533498	970479	726525	1053128	694267	657174	374574	644938	811182	686968	452492	349375	613782	585309	579130	543900	447728
fpHILIC	Negative	282.2513	0.37	unknown	58672376	81310880	123478520	137908144	89889352	80722936	107374968	104277064	118960416	72192112	61252992	89170688	91303080	53521692	63540040	75255176	87267664	100519376	66019748
EIC	Negative	323.2502	6.37	unknown	0	0	1894834	3264384	1515910	1574234	0	2233026	0	1597780	0	0	1090746	0	0	964137	0	1667114	0
EIC	Negative	573.4754	6.35	unknown	623388	705865	881064	767026	732686	685524	835662	778299	723270	488788	383378	561205	763750	417733	391191	467644	534275	462192	753338
EIC	Negative	510.3536	2.87	unknown	934214	58646	677348	176026	184709	62387	178579	1380806	144997	233372	56660	66586	43827	107046	109483	67580	73249	697751	80114
EIC	Negative	630.3781	5.49	unknown	8123222	7637954	6952851	7473354	8410756	8889299	8596182	7472397	5608590	5188123	7904729	7470716	5527911	4833224	5484692	4358234	6274288	5250177	6904647
EIC	Negative	495.2792	3.58	unknown	365487	746258	486348	332149	1177146	161484	629470	347691	666877	359354	618769	270072	591207	678498	688654	462932	565609	686228	408185
fpHILIC	Positive	203.0522	3.16	unknown	19468550	21818064	21204866	24619702	22499134	20621178	21611102	22629064	21272504	21789562	20923934	22364814	22253742	17296014	20921160	20420638	20151646	18482602	17927294
EIC	Negative	230.2102	6.35	unknown	179235	117741	251025	380661	213281	191025	113108	256327	341250	257004	54954	171469	119537	60393	48510	124065	70465	185186	55805
EIC	Negative	243.1965	3.09	unknown	3794450	3842358	6797768	7966820	6273283	4003464	4853859	7824335	5388636	5607525	3494755	4091473	4097297	5591145	2305139	3298715	4629489	6509321	4198169
EIC	Negative	474.2629	3.93	unknown	662728	873068	702743	901484	788031	924624	826590	665273	643796	424285	716627	644738	473608	1129901	909413	536057	763715	646632	734872
fpHILIC	Negative	140.9194	2.12	unknown	2420671	2877814	2214243	2938649	2545394	3070934	2701372	2507026	2600388	2338544	2971125	2756788	2608102	2481934	2412268	2313417	2665607	2328842	2595314
EIC	Negative	285.2350	3.57	unknown	75402	77889	66912	80577	73005	71329	72594	89628	64925	72094	78906	71771	83172	78290	76035	70748	73702	77364	64803
EIC	Negative	257.2272	6.38	unknown	801418	1075787	561981	1396745	920124	1578368	1083400	783066	906212	944011	836640	1490528	1280847	424491	910724	960798	1167223	1056498	819660
EIC	Negative	345.1745	5.58	unknown	312788	401836	482843	1200754	586637	308386	492791	647802	929796	565879	305431	559639	635670	225771	229863	530667	433331	644716	313329
EIC	Negative	319.1589	4.91	unknown	401677	431608	682992	1618640	929037	433329	774342	746749	900896	578381	302711	659549	527115	231547	181503	423737	471638	763765	278764
EIC	Negative	239.1652	3.77	unknown	724209	701122	738495	2762974	1374967	632823	1322313	1241059	1765606	1147898	991475	1306194	1462624	333584	472568	1224840	903833	1736023	371798
EIC	Negative	548.4692	5.78	unknown	206433	301920	443264	1176138	676784	337257	598189	550384	544634	348192	203473	469525	343646	115663	82470	255186	303279	468425	150424
EIC	Negative	242.1841	4.08	unknown	5258257	4483187	6848687	24757514	9353713	7342978	10720525	9892652	15114658	11991761	8398033	12933921	12125980	2661992	3886998	10167453	9888732	15314828	3305893
EIC	Negative	325.2009	3.00	unknown	135515	165987	268179	635176	256723	238944	236854	477468	301853	378901	197118	311912	248126	113740	66378	263100	207712	462439	113542
EIC	Negative	303.1589	3.22	unknown	326507	110489	231530	160192	193875	184323	254548	292810	54534	249007	108926	146434	114162	115250	190738	138968	155796	261938	114964
fpHILIC	Negative	149.0244	0.57	unknown	1011627	673104	699064	360010	588333	1059412	683774	624896	592259	461675	458563	485124	297919	837222	709233	363967	396749	496362	704303
EIC	Negative	587.3455	3.23	unknown	170175	180924	169034	180598	192719	159552	168466	182231	168711	144781	171742	157663	166218	159676	171521	162597	169036	159500	183615
fpHILIC	Positive	322.2047	2.30	unknown	1009488	718517	1281526	2013638	729430	1094071	1069757	1583550	1161097	1047617	532953	739248	940993	384052	698808	905722	393941	1014430	360688
fpHILIC	Negative	130.0228	4.58	unknown	541158	479673	481334	567933	489798	626754	566746	408782	467537	386636	455831	490686	472334	424414	410946	589837	458208	466669	443573
EIC	Negative	237.1132	1.80	unknown	152849	106706	127994	0	121079	97850	109106	90032	101133	90249	85726	78435	97673	43310	102062	142373	75754	0	51623
EIC	Negative	434.2370	6.30	unknown	3400060	1493662	2122415	3805133	1944733	3911814	3117253	2966240	997549	1823142	1203254	2888060	1837895	1066097	2221508	1354603	1662142	3017740	1266269
EIC	Negative	263.0020	1.91	unknown	10426559	6309526	1803424	1217312	6219383	4734497	6205173	1389813	6072760	1575664	7288097	7342114	9182516	8371327	2621256	6697547	7969514	530482	6619089
fpHILIC	Negative	142.9171	2.12	unknown	2276529	2858803	2166973	2849068	2356354	3169033	2658719	2325226	2532748	2195158	2884305	2773098	2640691	2487619	2373161	2353394	2640784	2298652	2556789
EIC	Negative	250.1893	5.58	unknown	3280577	4300865	5822422	17752224	6956998	4034688	6038950	8565481	11901242	5480603	3312991	5842526	6736402	2597742	2706654	5415091	5457100	7640480	3272178
fpHILIC	Negative	92.0265	0.52	unknown	2816332	1294798	1209737	145558	1351122	986027	1679122	1556497	1883999	1215740	1668576	0	494134	764747	903801	2775775	184160	496473	1166614
fpHILIC	Negative	175.0916	3.72	unknown	1101877	897073	903146	845309	1023138	1049919	947125	791026	739223	776061	831200	759422	752555	615322	951897	867879	709303	823445	703256
EIC	Negative	555.3428	4.17	unknown	486732	677714	599005	459223	534416	587265	744630	482248	611645	381625	514330	530639	668727	741592	573262	566081	490278	437260	476530
EIC	Negative	523.3753	5.80	unknown	55387	188518	136763	862667	429148	79570	193149	197865	530954	98615	53908	99143	206602	0	19490	86786	102254	125514	25007
EIC	Negative	278.2205	6.29	unknown	51133581	65738991	96931832	134089928	96100134	57519558	76676506	94382974	111015361	60756461	47367283	55533121	83893142	46133471	33028311	60114548	74229641	80176070	48376024
EIC	Negative	285.2074	5.79	unknown	160509	261143	334173	802876	476666	215906	427969	341426	433874	254992	163216	357991	262266	91707	73423	192525	215506	289699	132310
EIC	Negative	363.1783	3.07	unknown	399140	0	294836	184473	202624	196001	276008	223589	38891	287497	95574	0	0	0	108782	0	127359	245328	0
EIC	Negative	375.2556	1.81	unknown	158578	167227	172561	0	153376	164116	0	16428	165355	0	0	164173	0	0	159068	0	0	0	163127
fpHILIC	Positive	290.1592	2.91	unknown	1436436	686982	866524	1547342	729604	785820	678682	537785	2522024	1234349	735066	781961	856347	394572	963125	1433034	605292	712101	0
EIC	Negative	323.0596	1.59	unknown	71004	387954	55903	0	111753	55255	154633	0	334483	0	29163	45767	39057	10907	51255	46063	38314	0	311336
EIC	Negative	336.1900	2.49	unknown	314465	78962	109647	133068	143926	143190	180284	309793	42496	258244	67710	81920	12583	71742	75561	71504	73538	260182	95574
EIC	Negative	269.2125	4.05	unknown	2240174	2365512	5254516	9246333	4277066	3509417	3210811	7305196	4835602	3794640	2556976	3375648	3460798	3136955	1412146	3525179	3338660	5591001	2478148
EIC	Negative	347.2231	5.28	unknown	138469	219442	147186	248343	174205	226775	254727	193261	96766	198314	155513	34065	128663	190434	163100	24460	162658	125408	76994
EIC	Negative	279.1599	3.67	unknown	199167	230325	261073	549507	256244	183860	328237	402580	255146	247510	181174	170958	189048	71664	105809	328326	209371	289390	56696
EIC	Negative	344.0454	2.53	unknown	95178	190206	97178	60142	131765	93463	119677	124228	82479	92407	123398	0	0	0	97186	125360	0	87338	0
fpHILIC	Positive	456.1810	2.61	unknown	433890	308579	203975	510445	276304	404294	259545	349187	483774	314301	266410	369819	364998	220733	274513	503104	280286	396580	235556
fpHILIC	Positive	133.0690	3.31	unknown	1130019	974056	794301	1709269	838189	1661734	782865	1048327	1004107	1035225	998908	1080812	979110	485488	679764	1128763	1099976	1102552	805366
EIC	Negative	541.3347	4.22	unknown	806683	954902	796296	735900	737048	889050	726071	674769	674374	679879	807777	830921	807695	850524	756806	808117	694665	658207	783967
EIC	Negative	615.2470	1.12	unknown	433416	224476	671257	489596	194141	119301	302900	347384	141412	318549	364522	307600	376637	100418	187029	206760	184267	435732	91810
EIC	Negative	247.0952	2.41	unknown	402730	87560	210497	233097	80153	72155	85426	185293	60749	259793	73917	77911	68091	175629	72830	79349	68661	215629	47511
EIC	Negative	362.2424	3.49	unknown	610940	312492	534728	1675298	365341	385690	379811	646517	457513	828780	427578	552375	668225	222266	257556	547253	373091	826952	231107
EIC	Negative	296.2313	4.02	unknown	1671929	862072	2527616	2773314	1335731	1048245	1130877	2864004	1135017	2085437	954439	1131170	899718	2063678	1165985	686229	1370897	2947654	1347551
EIC	Negative	273.1246	0.92	unknown	304719	191069	364962	349140	164215	129210	216378	296050	126148	305217	220258	208284	228307	81740	133598	209033	136650	330743	122572
EIC	Negative	437.2461	6.00	unknown	2253728	3760352	2126360	5329482	2032608	3582435	2578106	2674536	3674511	4169312	3988542	1785119	3140345	3568423	3628586	1678180	4239178	2069338	2936409
EIC	Negative	301.2034	3.00	unknown	654721	409980	346403	530729	401259	562845	510953	477277	373172	459732	474008	460395	602125	234403	399633	522073	447114	684123	274534
EIC	Negative	440.2471	6.00	unknown	228354	329842	193404	502570	195283	331981	247150	259134	323259	408655	383454	162237	307156	321950	332753	149583	399221	200956	275569
EIC	Negative	356.2173	3.32	unknown	200473	54879	222287	129063	56805	69352	86978	133835	0	168599	16760	73660	39328	31824	54363	0	42230	192959	57816
EIC	Negative	425.2536	3.95	unknown	134256	137385	175875	68285	104291	142334	131386	150655	49818	54132	125307	134559	101855	241015	114895	52862	116340	135090	160056
fpHILIC	Positive	191.0572	3.00	unknown	529854	374010	300185	496167	332037	378880	391683	249221	417036	336841	432013	358069	315680	272102	408915	349841	347090	311841	353499
EIC	Negative	648.4923	6.36	unknown	523789	298584	599054	118317	310755	234868	330763	213374	142632	287467	235817	0	198901	217992	175925	190919	226355	315214	325768

EIC	Negative	309.2716	6.57	unknown	941549	1032386	584111	813658	1042676	676755	830670	809611	1136368	1159386	1269488	944322	802755	788927	922834	529598	852816	1330908	866945
fpHILIC	Positive	494.3237	0.61	unknown	12558132	13374523	13519752	10623611	10638983	11511624	15583880	10203929	9215732	9662322	12716031	13597259	14911869	12740216	12848489	11510890	12990120	10145471	9752411
EIC	Negative	530.3027	4.84	unknown	3011856	3109271	2940147	3056672	2893738	2759387	2921292	2953777	3140580	2867333	2870394	2939058	3048752	2924031	2997453	3117862	2887977	2648247	3042575
EIC	Negative	228.9854	0.55	unknown	486578	118993	147603	320720	104783	363650	177718	77335	104189	238347	162910	173337	116441	93169	139893	227174	79593	170478	212132
EIC	Negative	254.1480	2.27	unknown	1160791	691104	913390	1467820	713354	629751	552575	807175	587887	1148424	429724	507385	639005	412471	481590	779509	574909	1083301	377918
EIC	Negative	236.1373	4.12	unknown	66163	48538	67930	148172	49904	35044	50153	77970	36967	75055	38179	34871	71465	7879	30718	75429	40124	72413	31866
EIC	Negative	236.1375	4.23	unknown	225273	107933	195736	310890	84652	101597	93886	154467	67001	161351	78772	125251	125415	15827	88871	107217	78007	160284	68305
EIC	Negative	238.1529	4.60	unknown	235162	63186	341797	646073	67469	146497	131030	225671	82327	258961	75321	235487	220891	0	59810	135525	116587	289879	52669
EIC	Negative	621.4017	6.22	unknown	2396633	775130	2200423	1577074	1083580	534530	689865	2439941	777730	1839172	799374	841654	728709	686067	848483	958084	747121	2365736	618804
EIC	Negative	470.0325	0.48	unknown	7537529	7769606	8407015	8542689	6794546	7237543	7711572	7734563	8375282	7979871	7733839	7598014	8149986	2861459	7399864	7784801	7740623	7810850	7205103
fpHILIC	Negative	548.1718	3.35	unknown	1978329	1694149	2053814	1916817	1614428	1682243	1516597	1917917	1704250	2030011	1803357	1755334	1600877	1486168	1691567	1521039	1626430	1798087	1820444
EIC	Negative	576.5632	0.64	unknown	133977	55571	131684	103245	106869	80687	71383	49733	80561	210309	51455	30053	63816	127680	60060	60272	44126	89806	136042
fpHILIC	Positive	262.1645	2.26	unknown	2403175	2180602	2207504	2009000	1942072	2806617	2305985	1623381	2218345	2167810	2435450	2455747	2396874	1779958	2317761	2388815	1618583	2270776	2332068
EIC	Negative	542.6502	0.52	unknown	279199	0	236787	181731	225885	137457	147925	0	121479	275975	65960	28048	90589	288761	116213	102143	43553	163805	208873
fpHILIC	Negative	311.2957	0.37	unknown	1160201	1286144	2015993	2087278	1513435	816033	1059770	822938	861104	1387020	947264	1866820	846417	1242413	719953	755833	878028	1685821	1048499
fpHILIC	Negative	126.0031	3.22	unknown	11878660	8707358	8136858	9729491	8941034	10052182	7919053	7670858	8693443	8328052	7514225	8764977	9028724	7119384	7852020	9441520	8721170	9377237	8020119
fpHILIC	Positive	148.0037	3.23	unknown	2043724	2305235	2326571	2473480	2076614	2427761	2023926	1934737	2377688	1966396	1860117	2265561	2117944	2007163	2006824	2367309	2315101	2111019	2083757
EIC	Negative	366.7784	0.52	unknown	213989	87080	166476	187344	224835	163553	168806	48144	172060	279416	72594	73349	143645	651295	157416	133095	100196	148352	241592
EIC	Negative	285.2073	3.87	unknown	31835648	9057790	39934108	38550908	12635130	12018841	14166488	32738961	12603487	42346903	10920967	13335245	12113989	13643782	10999529	10414569	12680231	40439754	9984270
EIC	Negative	325.2658	4.47	unknown	660716	524243	600991	705762	569002	541925	569078	625113	570692	589881	495850	646128	607095	504973	567925	597607	553773	636933	531797
EIC	Negative	271.1748	2.44	unknown	1233852	626868	926504	978470	800896	867976	764550	914789	921394	1171051	961533	909620	792305	772488	826556	782018	929405	1141771	608027
EIC	Negative	295.2279	4.02	unknown	8204428	4320367	12772488	13904183	6799708	5261201	5563977	13661754	5743983	10059401	4961641	5397202	4442584	10704805	5707158	3393130	6842866	14596553	6648856
fpHILIC	Negative	386.0329	4.81	unknown	1714584	198195	989293	1004661	241382	277957	178834	929194	214604	1209432	171235	229612	186731	290146	202499	241730	228422	886924	290550
EIC	Negative	388.0295	0.48	unknown	8734527	9042173	10018986	9975367	7970347	8612997	9317661	9081584	9396183	9427590	8995641	8746114	9290789	4506052	8733353	9251539	9104069	8865442	8624028
EIC	Negative	608.3942	6.23	unknown	1295248	1015416	1277070	1030206	874713	1228792	1063029	999104	816325	863291	997474	1209614	866943	930575	1276510	982821	907984	865313	973108
EIC	Negative	578.3467	4.35	unknown	151756064	176065269	165496008	145053712	152612678	164204309	149169611	156790082	153235615	147270092	180785899	162307888	158743125	185084309	153585928	152253779	150577003	150191950	173352908
fpHILIC	Negative	250.1448	0.41	unknown	866061	435059	279806	1086193	664828	917207	664470	407172	295388	644185	750611	1048235	710053	346017	657815	737790	599876	412476	556713
EIC	Negative	528.2853	4.04	unknown	135426	255159	145487	158440	175377	222947	218853	148744	127654	113767	194342	222746	207700	201775	209543	165809	216069	124865	182075
EIC	Negative	367.2125	3.22	unknown	165443	239658	169051	150926	219865	165597	159342	179348	147563	143006	156916	150964	158363	143503	179145	165148	169689	163603	199070
EIC	Negative	253.1445	2.06	unknown	288842	314224	211702	200998	251839	222392	292314	173321	274393	241507	181480	230986	280516	235984	323765	328838	299015	124830	144819
EIC	Negative	387.0262	0.48	unknown	79293181	81029760	90198001	89641813	71665741	77587249	81718424	82077852	84401501	86181970	81459505	78419184	83872180	39853981	77759097	82583882	80536198	80947481	77990813
fpHILIC	Negative	305.0737	3.21	unknown	3687637	2960637	2786754	3343888	2864183	3172453	2714924	2700421	3057208	3300512	2865652	2888259	2740743	2459044	2928467	2830124	2710066	3411695	2720794
EIC	Negative	339.2539	3.96	unknown	59352	48768	73150	156601	47798	56488	51882	85224	75462	76144	72929	85950	104313	31105	21302	94224	55070	101605	46853
EIC	Negative	286.2108	3.87	unknown	5504873	1626494	7251502	6930687	2312605	2138384	2558127	5896048	2212473	7629281	1918030	2417755	2122928	2454032	1975893	1835946	2305443	7317748	1789756
EIC	Negative	299.2530	4.40	unknown	8841917	8381730	8687390	9141525	8470441	7010709	7301344	8284397	7426822	8680614	7069345	7101331	7537428	7261755	7536247	7349369	7446959	9392817	7049185
EIC	Negative	429.2860	4.32	unknown	1198963	445805	576065	785875	219479	519614	367097	559334	589751	851783	499505	328180	370602	313996	648035	259262	719400	174507	470808
EIC	Negative	469.0291	0.48	unknown	55263884	58361996	64032983	63963320	49182861	54329926	58225470	57832579	60566334	60617400	58015747	56369156	59802382	21743349	54707786	58563663	57365081	57159893	54447740
fpHILIC	Negative	146.0458	2.32	unknown	4166872	2335396	2297644	3109304	2661257	3721694	2130634	2063714	2722313	3243351	3355884	3448551	2734160	2317547	3067251	2865583	2487378	2368786	2610976
EIC	Negative	287.2232	3.21	unknown	353372	169797	443577	701951	278763	317104	234055	407173	270133	387351	249912	330088	271652	417661	137383	347252	281389	504207	248595
EIC	Negative	337.2327	2.06	unknown	638962	652400	562891	444258	597219	585942	650379	450982	572908	539766	638206	643589	540227	541113	575039	641485	643593	440193	579171
EIC	Negative	504.3067	4.35	unknown	1091989	1293007	1179627	1108530	1017141	1185034	1178624	1145734	1045753	1240383	1376690	1162404	1119542	1451948	1092535	1098474	1153460	930461	1341012
EIC	Negative	373.1418	1.82	unknown	720152	415183	721408	0	355733	230515	378069	433708	269062	0	469482	452711	499800	205237	265354	693334	439453	0	282326
EIC	Negative	254.1762	3.07	unknown	170067	121891	140183	207869	127158	169841	152643	124118	102877	155587	140690	179958	142908	111784	139139	163046	144645	133966	101438
EIC	Negative	295.1818	3.71	unknown	962060	0	1033923	0	0	0	0	1107349	0	833058	474911	0	499903	457020	547601	569357	0	0	0
EIC	Negative	235.1705	2.95	unknown	242579	223826	219010	221354	229188	202832	246422	218409	205890	220158	217191	210515	221151	199955	217281	236336	222261	208970	227773
EIC	Negative	551.0325	0.48	unknown	40416975	43578156	48758948	47556003	35665960	40647501	42304302	43362618	44919504	44974243	43184916	41483122	45133129	12436091	39685627	43930813	42105713	42630777	40461420
EIC	Negative	266.1483	2.62	unknown	739306	527975	1502725	404646	521485	826569	376029	725065	180304	1098732	614456	413722	232713	810563	650350	250692	361946	855562	535814
fpHILIC	Positive	655.3351	0.40	unknown	1029318	1182507	661418	887260	1021714	803532	847977	1038465	1167290	692206	884764	907646	1735627	1362302	385901	1113186	873567	681312	1872577
fpHILIC	Positive	331.1719	3.70	unknown	619172	315367	360784	297965	273191	360484	271892	267871	203913	374577	240173	281846	241218	218855	383548	292574	213972	310078	190632
EIC	Negative	270.1713	2.16	unknown	394933	171405	216917	264839	193926	320999	196601	231403	246575	382802	274595	317117	222857	220930	233172	225509	275057	252795	215282
fpHILIC	Negative	842.5544	0.43	unknown	14241516	13907804	11812369	12203273	12408530	11783708	11101660	10829115	14312426	10287711	12134653	13043783	13193169	13353946	12471879	12014149	13200739	9492448	13884384
fpHILIC	Positive	119.0645	3.65	unknown	651087	277359	491413	946768	284868	512328	321103	654398	257897	596683	275773	331754	306767	209686	329996	289955	320081	818323	294013
EIC	Negative	579.3498	4.35	unknown	48068720	55720155	52289133	45952053	47764350	52600055	47183304	49248095	48179894	46899712	56563301	51285504	50250561	58978914	48611021	48150898	47810858	47663509	55392277
EIC	Negative	251.1654	2.43	unknown	302624	99570	363739	147380	123679	106724	108581	224165	100651	292775	111486	89712	104375	105193	103085	118914	99409	252289	97313
EIC	Negative	558.3149	5.17	unknown	713342	762989	737401	650933	672837	729551	813005	767286	640847	638712	785382	809322	668211	730370	761817	690469	832533	650558	727663
fpHILIC	Positive	149.0803	3.47	unknown	3113894	2296024	2527333	2406044	2223677	2340065	2392753	2278250	2393780	2644972	2648892	2273888	2369811	2126818	2746956	2064529	2348897	2545745	2049968
EIC	Negative	243.1965	4.13	unknown	398502	414567	499096	456498	404448	405167	416907	412949	403292	392865	387239	461388	391288	408530	365158	441997	363368	565395	329732
EIC	Negative	375.2481	5.77	unknown	233363	14350	257845	0	163096	0	0	212234	0	273551	0	154859	0	0	166035	0	0	198117	15494
fpHILIC	Positive	247.0572	3.05	unknown	517241	376018	270663	324378	378575	401785	408470	285530	226219	311553	306789	371187	317114	235177	373831	360568	312582	432225	240933
EIC	Negative	430.8093	0.52	unknown	592029	411869	678235	581793	403936	429898	347242	432565	529019	608379	392534	372556	394515	0	349831	508313	343682	447638	518008
EIC	Negative	465.1879	0.71	unknown	60918	59353	69394	33544	37101	67280	54939	26747	48546	70220	57473	49452	48478	74716	48583	41496	39603	23746	83903
fpHILIC	Negative	883.5339	0.38	unknown	1786298	1331539	1877836	1510630	1359580	1322708	1144094	1099470	932796	1266724	1099490	888563	1100332	1237119	1291331	1093866	1448978	1159365	933807
EIC	Negative	561.4785	6.46	unknown	3179647	7293353	4279006	4397328	4395022	3341046	4430154	4238729	6032095	4692564	4373303	5541617	4408701	5079338	3631183	4510941	4448694	5455816	4653967
EIC	Negative	253.1647	2.64	unknown	924347	550220	252166	817887	642026	1184485	658492	271218	381781	561043	795286	808439	551816	221036	989658	712461	865639	219016	388185
EIC	Negative	296.2313	3.92	unknown	887875	554093	1600072	6685443	1213502	875196	1028995	2676124	2084844	1748497	974964	2125392	1682246	394191	510090	2564661	1297527	2933743	665516
fpHILIC	Positive	77.0392	2.06	unknown	518517	334375	404300	321056	280782	364246	311914	317281	248549	319088	376775	329321	239831	416719	447470	284304	202920	361218	244859
fpHILIC	Positive	784.5823	0.43	unknown	37330244	37316156	40130580	32454048	27351150	30082318	28831730	32538372	26643736	30333894	31625470	30579840	27747284	39276104	30218416	25882102	28250256	30323956	32524258
EIC	Negative	566.3474	5.40	unknown	721271	727992	692697	644849	778642	695851	699917	737467	667854	727576	739665	784932	678148	655853	721764	743210	651741	695795	811084
EIC	Negative	327.2545	3.77	unknown	72422	55465	201193	92975	149321	77429	57186	146938	44685	89980	72220	70448	81310	70689	76016	64987	88294	194958	57139
EIC	Negative	393.2290	5.86	unknown	247123	156585	241631	238163	120176	195378	167140	237250	292802	228093	133254	247373	197128	79979	129080	342969	210180	174734	168219
EIC	Negative	298.2469	5.86	unknown	2181161	1496287	2220954	2299011	1173732	1978493	1592570	2025065	2726339	1947853	1260880	2106067	1883599	824282	1198440	3134004	2037936	1671962	1571564
EIC	Negative	614.4052	5.80	unknown	145573	108039	189364	131197	98036	84621	79388	114202	72927	93799	111432	61108	78192	109360	120418	60001	75251	83312	115792
fpHILIC	Negative	103.0149	1.93	unknown	11081678	9557350	10159111	10749795	9551591	13695137	9875218	7924089	7589110	9990811	11802040	10433482	8771236	11298631	10480949	9579708	10478109	7688759	11014527
EIC	Negative	580.3530	4.35	unknown	10218006	11765657	10939197	9708579	10143466	11015000	10161895	10409009	10170559	9783482	11845269	10628133	10181210	12292320	10174550	10322927	10262816	10141920	11665244
fpHILIC	Positive	171.0760	3.47	unknown	6578966	4500617	6027115	5534652	4530195	5010071	4881054	4542012	5130207	5086949	4741858	4720228	4749002	4162434	5009067	4516445	4534381	5033459	5319372
EIC	Negative	596.2904	5.16	unknown	746023	1099010	955841	823670	891037	1564976	865159	753593	902008	810054	1315079	1247510	935435	1608936	857023	1106029	864178	734693	1481215
EIC	Negative	338.2416	3.90	unknown	97212	78057	84729	147884	63657	94788	62380	60381	69735	75211	62449	74746	69183	47612	74917	75107	73395	81829	50406
EIC	Negative	266.8039	0.63	unknown	949294	654032	1454303	1061985	736395	830200	680992	536762	1004527	1884446	674218	607281	781783	517745	432066	583116	573815	849133	1401843
EIC	Negative	571.3460	2.42	unknown	1539373	1139142	1194443	1323980	943259	1203227	1008545	1242328	905564	1210959	1186447	1182811	973052	855711	1001154	982373	1010390	1320099	1062198

EIC	Negative	574.7641	0.52	unknown	444764	405148	656346	630428	339062	425655	347079	390989	551595	413633	422206	395428	440192	59554	362329	515053	372908	486082	346679
EIC	Negative	283.2283	6.22	unknown	391319	290270	405760	633424	252353	209087	273386	279716	322363	278184	278302	402152	471452	210806	210965	359828	257241	297976	229607
EIC	Negative	275.1655	0.89	unknown	65741	0	83045	69536	0	74752	0	77541	0	0	0	82654	0	0	0	99860	0	54617	0
EIC	Negative	341.2699	4.28	unknown	66599	35911	144073	482779	61401	75094	79090	203077	105577	125552	98260	172711	137242	38017	48157	130573	87544	223486	42124
EIC	Negative	373.0102	0.48	unknown	626836	626964	685994	629688	574383	636477	592235	581893	724458	696039	632950	683056	569683	314876	624995	609056	684098	687697	550618
EIC	Negative	458.8488	0.52	unknown	405883	475812	594340	646052	391003	482579	444364	420668	530824	417053	496008	466796	486208	107387	419619	522563	462338	520653	378580
EIC	Negative	578.3467	4.89	unknown	13903598	18679352	18709044	12980088	13645377	17591921	16947030	15094941	11957886	13066879	18214431	18179663	15445291	20100508	14887064	12696106	14267273	13833080	20723213
EIC	Negative	325.2660	4.98	unknown	1453191	1433745	1425588	1639498	1476363	1351685	1384792	1494212	1418128	1449513	1390591	1420046	1457528	1308854	1487821	1482044	1441983	1486149	1338607
EIC	Negative	317.1146	0.92	unknown	784423	502269	973849	911422	422455	332333	576246	760398	317387	790519	670631	546796	624745	247326	356765	545412	387583	905924	250886
EIC	Negative	295.1552	2.43	unknown	1598828	1473643	1523829	1546905	1493526	1331050	1401652	1556248	1504313	1537362	1447733	1454310	1636610	1363688	1345789	1743568	1416023	1482378	1414097
EIC	Negative	256.1638	2.37	unknown	169732	78533	142936	147427	73552	103625	76684	108720	47001	141394	62781	82456	72362	58550	84108	71303	58408	142105	60241
EIC	Negative	263.1291	2.28	unknown	1003656	331766	1222294	352754	235175	432393	235978	624090	87333	1149762	269219	301386	129753	393228	362932	134911	179783	629882	333683
EIC	Negative	502.6319	0.63	unknown	272023	198590	565195	382477	213414	249388	184387	139160	314100	688228	193684	173486	221254	97938	166933	240472	164996	267242	278963
EIC	Negative	329.2877	4.97	unknown	366183	378284	347583	394258	370745	358287	363130	368527	355826	372045	357214	366943	375358	334633	365148	387208	375136	383205	335931
EIC	Negative	637.3536	5.27	unknown	809516	757919	693311	730538	665721	556801	680146	684934	694173	744471	673738	709725	722320	669968	720112	668468	638138	632699	677434
EIC	Negative	312.2630	6.50	unknown	194335	174493	392380	877853	217456	149369	249311	245294	213933	338615	437997	318492	318161	61011	122927	294753	253365	301344	121036
EIC	Negative	286.7722	0.52	unknown	262724	65563	206804	134917	258964	158171	117687	76738	125806	406210	78585	68681	96566	1408565	116356	140649	42429	125048	325358
fpHILIC	Positive	193.0949	3.13	unknown	631616	873378	845230	747946	760734	812812	703157	836781	746511	772882	752363	901211	665839	929866	795932	782054	725464	766072	855942
fpHILIC	Positive	499.3497	0.60	unknown	2332852	3173672	2357815	2554551	2519797	2530184	2893930	2028613	2754934	2165598	3082441	2899088	3389052	3085156	2813907	3019523	2296226	2068909	2748635
fpHILIC	Positive	148.0794	3.47	unknown	30663828	22273982	25872270	26871026	21599718	22741054	22731554	22576062	25747088	23615502	23266796	21382192	21824688	19508372	24905168	21168046	25762802	24118190	21440794
fpHILIC	Negative	182.0538	1.76	unknown	2098955	885205	1044764	1434885	842607	1511066	847920	867310	857241	1470811	1024859	1085504	969380	1262584	923982	950261	991180	1268122	879278
EIC	Negative	347.2593	6.22	unknown	330555	155115	221959	354301	135633	227037	135421	227490	310736	256772	123339	271654	186097	67187	154977	243619	257218	215299	183447
EIC	Negative	339.9407	0.52	unknown	249165	259829	279252	334289	229744	236049	285300	324787	268983	379024	263425	316105	294850	75620	236064	275690	304987	296417	255770
EIC	Negative	557.3563	5.09	unknown	1940142	2000536	1888977	2031327	1881032	1940998	1950109	2034164	2085724	1829034	2201889	2062463	1897535	2184051	2053777	2064718	1842544	1912544	2196296
fpHILIC	Negative	369.1096	3.20	unknown	1107723	1172848	1330493	1303715	1049058	1051115	998229	1124452	1126794	1245484	1193490	1170582	977361	969016	1228179	1002847	986516	1189443	1039454
EIC	Negative	319.1897	5.15	unknown	598883	348278	1051326	1319715	175767	58396	124029	240452	148318	636835	159734	1360458	202099	171079	99896	132101	90818	209030	228825
EIC	Negative	251.1288	2.74	unknown	346038	355307	190681	508513	331800	255621	194247	169391	197998	507500	164746	187661	233426	107189	200482	271091	181452	411055	106115
EIC	Negative	370.9448	0.55	unknown	662449	155946	260530	469092	155866	474513	213995	91319	152646	350979	220555	207267	168288	75885	189611	297459	109200	203832	308387
EIC	Negative	555.2726	5.62	unknown	2868821	2277168	2275283	3055926	2081660	1643951	1917729	2024765	1570796	2503869	1487157	1430121	1921882	1545591	1599621	1571877	1938596	2871828	1430231
EIC	Negative	265.1087	2.22	unknown	83558	60090	69636	81652	56230	53288	57327	92208	53559	101777	56299	65823	48300	46139	52361	68925	47596	84545	65071
fpHILIC	Negative	124.0150	2.98	unknown	488720	458978	447018	549582	338323	531295	357396	270322	300945	417798	331796	283165	423945	156414	298054	403360	409066	393850	327958
EIC	Negative	252.1606	2.64	unknown	6757879	4565252	3457509	7616593	5360087	8557234	5373789	3922453	3709610	4726606	6096739	6424270	4992008	2290439	7422372	6295179	6707878	3938940	3070077
fpHILIC	Positive	495.3270	0.61	unknown	3862228	3432957	3350190	2909829	3305700	2954329	3742574	2569212	2700818	2659423	2874009	3845052	3513110	3281184	3338094	3154038	3947265	2720700	2402743
EIC	Negative	464.2785	4.89	unknown	891521	1003133	1126265	637070	733819	1045278	812212	850659	414291	643417	739177	733861	561906	1278746	849589	442667	707074	851182	1079167
fpHILIC	Positive	273.0462	2.70	unknown	310144	316759	238552	394951	324099	347655	319513	175136	288161	283404	113033	318257	158340	251587	294768	229582	201419	238769	517855
fpHILIC	Positive	785.5869	0.43	unknown	16461353	15852007	17753570	13994723	11979065	13241882	12971873	14005408	10590179	13894232	13543558	12997641	11499192	17261466	13103127	11269436	11416118	14262029	13941263
EIC	Negative	349.2011	4.37	unknown	302710	223983	498245	1400415	345484	178534	339472	430268	449752	537899	292514	528399	406244	81657	91169	365096	308282	864844	147942
EIC	Negative	440.2470	6.09	unknown	117603	127396	103551	251239	77729	69178	85022	131895	144174	177660	134859	51025	120906	167836	95893	55513	146484	139486	80518
EIC	Negative	579.3498	4.89	unknown	4425173	5814786	6010027	4091145	4359847	5587532	5319955	4654125	3788645	4048850	5751012	5813791	4940528	6328526	4679377	4017670	4521834	4372257	6581287
EIC	Negative	508.9095	0.55	unknown	297038	75063	187331	237281	91945	231758	132926	13145	113577	179935	108686	98143	103941	0	92719	153204	72928	105978	169915
EIC	Negative	616.2505	1.03	unknown	143727	78952	222583	162634	71298	43185	103712	132346	55435	113544	128838	100973	130649	42566	69349	82963	68506	154279	38820
EIC	Negative	363.2549	5.88	unknown	1724510	1066615	1985681	1841208	1172676	703597	880059	1703826	914798	2309898	1165533	793032	902700	909707	1137400	944338	901495	1663043	834505
EIC	Negative	323.2501	5.21	unknown	3721538	3635866	3488901	3722521	3554376	3523852	3252366	3628280	3266178	3487599	3422186	3266726	3438761	3060643	3458513	3713837	3417338	3568193	3409155
EIC	Negative	381.2567	4.40	unknown	609371	523623	537230	426032	559163	425163	453723	525735	475207	660371	482590	468373	531132	481924	494460	497533	460122	584406	450893
fpHILIC	Negative	819.5577	0.43	unknown	9442158	9017557	7922106	6323034	7140362	6310956	5747288	6563615	8891174	6685028	8128024	7258093	6506452	9839019	8114044	6031667	6001800	5893849	9300685
fpHILIC	Positive	733.5567	0.44	unknown	482551	512852	748722	494172	384107	427257	345995	496784	603134	475727	507278	511522	445111	810525	431805	347238	542197	543889	437216
fpHILIC	Negative	844.5689	0.43	unknown	6724470	5848757	6695599	5089928	5031394	5307939	4826395	4939410	4342763	5198301	5024956	5186035	4915164	5728442	5507340	4291978	4690753	5031779	5418590
EIC	Negative	566.3472	5.16	unknown	380813	428715	365678	367876	447700	433667	437408	381584	349538	359043	445374	464384	412046	461159	476370	424605	402987	341037	446976
EIC	Negative	374.8485	0.52	unknown	1337694	1042600	1688306	1630878	1093350	1194661	1087572	847512	1319711	1468715	1085431	1040811	1149624	331177	994017	1257304	988517	1234341	1183226
fpHILIC	Positive	637.4687	0.41	unknown	124412	164034	52656	314495	476987	172010	171651	207237	285873	277578	379395	470020	72577	430696	334028	165927	357058	195279	288842
EIC	Negative	633.0356	0.48	unknown	28810874	31402516	35257415	34587510	24617813	28815642	29814854	31246371	32396534	32343731	31458533	30231935	32565431	7331145	28213488	31447632	30816169	30870542	28666928
EIC	Negative	644.3525	3.56	unknown	188953	56806	35639	69824	63167	111038	40284	69650	39901	89030	67722	139663	38642	26049	61845	60811	65119	40705	102496
EIC	Negative	253.0959	0.75	unknown	388907	181882	136855	521867	144272	585521	396625	0	115989	187094	254397	445055	346962	494475	216602	353796	211259	157488	330127
fpHILIC	Negative	215.9734	2.08	unknown	348984	512601	585204	340241	326359	580663	394700	315900	300530	554577	456722	382338	398672	505020	623810	254572	295171	366989	419678
fpHILIC	Positive	233.0626	3.13	unknown	553257	287572	242042	417537	244246	429607	197354	192554	260161	289842	327516	435175	225453	202763	229543	371451	191033	243989	317313
fpHILIC	Positive	285.1193	3.76	unknown	619349	256824	366315	350625	244269	387782	214210	214415	206260	316152	267706	246678	234648	286163	272428	252479	252021	281191	237280
EIC	Negative	379.2469	5.86	unknown	757246	550329	829160	831839	463199	781488	581265	748159	995170	762712	457277	825597	727051	327907	412867	1186622	773235	672920	604244
EIC	Negative	347.1874	4.07	unknown	139938	70910	213003	90802	75558	78447	82115	107116	22644	111281	59052	98941	56258	57466	62740	63610	44892	142354	60599
fpHILIC	Positive	101.0713	3.48	unknown	6633031	3745487	5149396	4839141	4159823	3963478	4170954	3965607	3966179	4601563	4017557	4303701	3856776	3691630	4541394	3876667	3834815	5279967	3153566
EIC	Negative	538.3211	3.73	unknown	1991159	278832	5514005	3130057	312846	214221	204066	4030522	392182	2843853	342124	361916	264519	531450	541869	320677	373187	3685337	350333
EIC	Negative	287.2130	3.87	unknown	714032	203576	944004	884068	274895	277421	325030	687339	296724	955969	245902	305291	278846	310026	260613	230943	288379	912833	211387
EIC	Negative	439.2440	6.09	unknown	421202	401518	390582	1025235	178624	264832	226361	427702	483724	659538	499340	165856	517880	497709	345861	129679	433940	434089	274691
EIC	Negative	455.0135	0.48	unknown	490128	511927	566970	481723	355760	495982	486196	459285	542654	531439	515863	487168	483147	176495	464748	443199	518522	504506	464149
fpHILIC	Negative	281.1751	0.61	unknown	2801421	751161	2618198	1242738	863407	937903	1115994	1591634	429043	1540231	559487	769493	745401	694860	719366	632409	910068	2163891	630287
EIC	Negative	239.1287	2.11	unknown	193530	164201	249811	379850	210274	158300	293071	212998	260159	294569	173587	205888	247026	112236	210212	305275	227062	280501	139085
EIC	Negative	297.2436	5.86	unknown	10941339	7282706	11444433	11306362	5920765	9763762	7961399	10088547	13341507	9573694	6232438	10709087	9433823	3984797	5914855	15664555	10350081	8508457	7775153
EIC	Negative	554.3018	4.36	unknown	2287351	2185123	2355575	2287589	2003704	1834972	1906689	2398794	2133761	2099391	2042347	2177580	2185933	2298494	2096719	2212316	1877642	2232880	2107754
fpHILIC	Negative	578.3085	0.60	unknown	992739	1031493	954957	1111435	727370	894875	988085	895875	885879	782397	939520	924930	985300	958947	900093	927062	939025	784514	948280
EIC	Negative	300.2003	3.00	unknown	3422954	2120651	2324051	4338493	1918669	2476346	2509553	2351198	1914511	2817428	2322760	2247767	3131953	1048140	2022058	2586087	2165549	3334681	1336353
EIC	Negative	450.2631	4.27	unknown	3883204	4994452	4775520	3261971	3791944	5234970	3736132	3437735	2747971	2653807	3308255	3655588	3024546	5721637	3632607	4127245	2965365	3923178	4769177
EIC	Negative	268.1556	2.33	unknown	652924	417130	312934	618018	374355	508565	372039	307638	402774	526075	433035	475448	426222	261119	479311	523096	429832	319942	261924
EIC	Negative	324.0578	2.39	unknown	107656	48121	89527	103866	40180	35877	40110	95444	38326	119428	43515	45381	39073	37664	39186	45131	30875	105066	35879
EIC	Negative	379.1773	2.16	unknown	286400	152250	242766	188995	76666	95654	169375	130825	82474	135706	93485	155498	147662	101311	97616	131049	101606	133280	105353
fpHILIC	Positive	807.5699	0.43	unknown	22282306	39240392	21364566	25896428	26019968	25343928	25369792	19310518	38638884	21139326	32544148	29577514	32463462	31144776	25603474	34812872	29331984	17984916	30407022
EIC	Negative	578.3468	4.49	unknown	23570300	27794504	32447020	22283316	21784969	24053541	22397088	22923805	21574729	22450740	28700423	24563568	22745435	32637131	22196838	22593498	20337162	22534999	27978494
EIC	Negative	479.2972	5.54	unknown	21845087	26332535	22831860	18371197	20933743	27119354	20084310	20068174	13744427	15039125	16902804	20390397	13321071	24835321	19092632	21747713	17878527	20972702	24890565
EIC	Negative	239.1652	2.67	unknown	804756	487218	776588	856191	448660	637639	615358	690993	554096	726577	557458	821278	861945	590659	435935	619267	660525	794807	512563
EIC	Negative	321.2913	3.75	unknown	593859	602107	536959	537150	557409	576063	567131	555055	511834	533316	544818	518799	542570	536301	540367	563125	578723	581253	576943
fpHILIC	Positive	189.0865	2.48	unknown	5269962	3631043	3159507	5832462	3874885	4209256	3135719	3632476	3938476	3829725	4262077	2966430	3503538	3090485	4318250	4929823	3380690	3255378	3403221

EIC	Negative	406.2534	5.66	unknown	124568	146051	118744	187212	127398	132984	138803	119160	111916	135874	133768	145398	165241	113380	137287	135409	127394	145761	101085
EIC	Negative	319.1556	3.15	unknown	524942	138649	544246	448634	171204	199456	246201	388138	94856	507086	176053	248993	169457	116515	152712	136086	177080	484596	178846
EIC	Negative	501.3013	3.81	unknown	480914	180871	512308	1105082	233890	346918	421638	603031	566627	863015	351109	338487	636161	31632	205078	426132	431463	1167720	0
EIC	Negative	297.1718	2.53	unknown	473390	191349	406940	212029	103577	164683	143101	314024	71527	350106	177705	118551	118528	156632	106468	83690	86289	381658	160325
EIC	Negative	374.2913	3.22	unknown	556427	671549	572542	759689	687749	677359	566574	623136	649921	672901	679574	645208	649230	556920	585840	828778	610032	612585	654277
fpHILIC	Negative	160.0615	1.66	unknown	158126	3004156	0	3751126	0	4304793	2808077	0	0	0	4363097	4766060	0	0	3095085	4211376	0	0	0
fpHILIC	Positive	240.1014	1.67	unknown	613457	693216	720367	745652	457871	578105	647946	550950	653014	658758	712152	675302	646710	507356	621920	746077	445149	585717	568706
fpHILIC	Negative	195.9262	4.55	unknown	1192816	1003057	1006201	1094570	824163	1136359	916965	931982	1031313	1267355	966433	1031377	1018739	935505	1132326	1031687	853702	935293	1022341
EIC	Negative	263.1654	3.90	unknown	191137	93182	332122	153842	104744	86058	122750	174394	78130	223167	134944	116610	109611	106644	96873	70751	86266	216241	111724
EIC	Negative	504.3134	4.00	unknown	0	0	323808	706393	15614	0	0	0	0	593648	0	0	0	0	0	16050	0	0	0
EIC	Negative	313.2027	2.41	unknown	198612	71585	210124	114906	65824	83701	69251	144439	48731	165044	74631	73263	59037	56695	68917	69915	68323	141921	74501
EIC	Negative	352.1751	2.44	unknown	312751	133682	160114	234796	177668	176790	148832	261387	194574	196322	162715	178304	144947	141822	167248	136853	154430	410351	117835
EIC	Negative	319.2640	5.88	unknown	836974	519072	933253	881320	569075	343715	452739	807072	462846	1121298	593151	396242	463359	452055	591106	467535	445296	799170	407924
EIC	Negative	326.2001	3.09	unknown	266717	114235	185013	332038	164749	199508	145444	215918	131597	235246	94434	230386	176240	222577	161418	159206	181207	238832	141298
EIC	Negative	480.8962	0.52	unknown	1086799	1274974	1203691	1431196	798095	1012915	1099617	1331154	1197005	951871	1207052	1371088	1248701	161422	970054	1080872	1172785	1250395	1122480
fpHILIC	Negative	594.3404	0.62	unknown	2059432	2597943	2918702	1838329	1704206	2720560	2039387	2066176	1547004	1623707	2316047	2224835	2070491	2336195	1865490	1657817	1632563	2161396	3052824
EIC	Negative	278.1839	3.13	unknown	675875	347757	581242	478240	0	471358	433959	498332	0	565150	499582	555223	394927	0	518161	0	432104	494169	0
EIC	Negative	255.1604	2.45	unknown	512691	291748	525664	593296	303481	333183	303637	415972	300174	499381	247417	337208	356281	259156	272962	343053	287254	566863	234636
EIC	Negative	327.2727	4.01	unknown	382116	324556	301314	316862	329552	337768	345895	316073	303825	292992	316544	326754	307146	333994	308632	355717	332777	356702	326987
fpHILIC	Negative	146.0651	3.47	unknown	9257249	5925570	7639285	6729111	5707486	6262063	6108204	6077079	5795110	6860115	6029740	6287811	5693778	4535227	7357454	5732126	6019150	7407164	4323179
EIC	Negative	636.3501	5.27	unknown	2301463	2241621	2132761	2077572	1976310	1750952	2010747	2070603	2181598	2159809	2176032	2277798	2238818	2084484	2241206	2177548	2011782	1809049	2052299
fpHILIC	Positive	229.1546	2.03	unknown	3993114	3128676	2243111	3865372	2524413	3644919	2870750	1682911	3062717	2800184	2890989	3324907	3050500	2120547	2731292	4412447	2260559	2605097	2192951
fpHILIC	Positive	175.1190	5.38	unknown	54862160	52207740	37747520	40414576	44586832	57423480	46470476	36142392	42173936	33076006	43138776	48689676	50216416	44944368	65776228	56984972	35129420	34685776	41088288
EIC	Negative	579.3498	4.49	unknown	7582616	8806299	10334847	7146087	6913476	7781443	7067400	7346153	6853409	7101495	8958405	7915550	7263904	10456002	7164884	7375416	6459795	7284063	8777202
fpHILIC	Positive	343.1889	2.39	unknown	1007906	806349	822116	755562	829248	838390	946968	695717	719265	871999	957966	833051	881852	876423	881079	819916	781813	980790	689596
fpHILIC	Positive	247.1279	3.02	unknown	1679955	720430	772216	867321	708465	1450768	695079	438890	578320	764072	900821	926341	796284	776995	885993	913542	688508	616505	886449
fpHILIC	Positive	148.0734	3.48	unknown	3828256	3268924	3461595	3592235	2787761	3445419	2853660	2878994	3576112	3413552	3532607	2826839	3058265	2423909	3800380	3047880	3357592	3228874	2593501
EIC	Negative	296.2313	4.60	unknown	365489	227512	563107	404413	316659	320588	503091	346135	353633	301219	214075	243203	231903	362804	200140	618206	407592	436443	235371
EIC	Negative	478.2843	4.68	unknown	9523400	10109178	10148086	8018106	7687468	9468654	7403413	8172865	5778246	7114843	8230344	7505998	6248732	10806849	8679091	4394815	7553552	9262907	9832776
EIC	Negative	241.0506	2.39	unknown	5509545	4126674	5158407	5774095	3691900	3951830	4124509	5122375	3565658	5337915	3786147	4053182	3966248	4089158	3973475	3964881	3927933	5330594	3909157
EIC	Negative	313.1197	1.06	unknown	373386	162628	226698	258361	137749	140227	162159	313302	153017	318730	155086	212829	190611	152376	152015	198053	175299	279877	130098
EIC	Negative	637.3536	5.04	unknown	708466	497787	603111	598294	463755	411899	556333	621342	606764	709660	475708	547856	509541	522083	554702	539176	528551	658357	465471
fpHILIC	Positive	787.6059	0.43	unknown	20134682	19738960	24368056	19585688	20083122	18671686	17544124	24263556	15452994	20336776	20788256	18493914	16185466	23103242	19775330	16479713	19430524	22369636	21087972
EIC	Negative	327.1601	1.83	unknown	375149	477258	283789	0	184439	155985	209944	26574	306589	0	139516	168860	147576	138903	215990	405890	218455	0	72045
EIC	Negative	417.2278	4.56	unknown	738052	572793	851394	656514	487752	481929	331528	508941	578767	687324	404187	659884	427135	441860	351019	581360	505578	643995	384999
fpHILIC	Positive	330.1760	2.70	unknown	312355	406882	396712	276009	333287	335858	390468	292495	349646	315426	337533	345835	373967	541637	325178	326573	321705	363514	420662
fpHILIC	Positive	311.1229	3.54	unknown	1139469	726153	826907	740143	562070	957720	564813	499615	515637	902584	483029	513935	479198	740319	613635	557480	610020	833484	587214
EIC	Negative	288.1637	2.64	unknown	483504	134958	0	37474	56390	186254	291138	0	111975	42234	192197	203619	189346	111148	191354	153728	235058	0	86924
EIC	Negative	429.2862	3.34	unknown	649545	258688	361358	524887	175294	273688	173748	427313	358403	401225	284262	224935	262416	282971	407446	148821	457227	221380	221025
EIC	Negative	331.1193	3.46	unknown	1604121	1527714	1666496	1636756	1269377	1108789	1447857	1495426	1325384	1759184	1455056	1465765	1400081	1292698	1684984	1618335	1114081	1434557	1019043
fpHILIC	Negative	274.1409	4.35	unknown	536791	477231	296732	337790	394047	700859	350944	207995	279527	322847	487580	568940	456731	376101	472870	500863	358330	290068	376617
fpHILIC	Negative	207.0144	4.21	unknown	3703655	1207904	452390	1843927	1990607	2841979	1650892	761485	2187214	537982	1005837	2684197	1820429	1309785	1700346	2923384	2238518	1279910	1609044
fpHILIC	Positive	496.2795	0.60	unknown	2019528	2521235	1915187	1947212	2116079	1945734	1976332	2102332	2617117	1969951	2428735	2291321	2677977	2448165	2491657	2531084	2346691	1541971	2210894
fpHILIC	Positive	302.2149	0.61	unknown	2369099	683112	1327203	2248761	807822	886314	1256742	1436084	803519	1284986	1108937	976509	971143	190961	702566	1056523	897078	2211766	499463
EIC	Negative	376.8464	0.52	unknown	615695	531268	835412	824825	529318	605410	513599	354323	617017	682338	523447	498545	542890	167919	472546	609387	500020	578189	548822
EIC	Negative	476.2785	4.68	unknown	205372816	208704751	219450424	172033686	160200475	203634827	154771155	175795069	120219310	156547436	173991869	157927214	134334018	227747963	185281078	91559791	164337636	191756651	209486576
EIC	Negative	450.9511	0.55	unknown	674472	233974	339100	582373	167321	570953	290480	93194	182701	327152	284962	302428	201786	74374	230423	380316	162216	261858	314793
EIC	Negative	478.2943	5.54	unknown	84125669	100424174	86610179	69395287	80373890	102621218	75913593	76212527	51500469	57397689	64351059	77756132	50110887	94601805	73893032	83425499	67644431	80462442	94673134
EIC	Negative	595.2892	5.16	unknown	2367242	3572771	3215668	2751294	2919660	5002684	2774758	2339325	3132269	2802020	4555043	4023607	3190922	4820178	3100590	3660843	2916465	2287063	4770530
EIC	Negative	428.8733	3.47	unknown	116442	105005	96396	152825	116762	87223	107832	58418	110875	70070	117535	92901	94235	94000	108969	112279	100442	91891	84398
EIC	Negative	477.2814	4.68	unknown	53850511	54966438	57044468	45168399	41750131	52787353	40458051	45600686	31399408	41238384	45313207	41359061	35086627	59949472	48043767	24089754	42477214	50548292	54323284
fpHILIC	Positive	689.5593	0.48	unknown	736684	654562	548487	520173	478228	357902	367222	458192	606292	497726	533784	453548	449414	534787	494913	532922	430951	454070	435460
EIC	Negative	556.3008	4.60	unknown	999755	878463	924784	938310	899579	765113	948077	868262	874643	945603	867395	857339	983962	921115	934220	965897	826763	923925	859464
fpHILIC	Negative	176.9169	4.55	unknown	843101	668109	655082	752463	433390	678423	550940	609269	659421	742792	588838	602207	662792	536626	613265	657059	564733	564261	636391
EIC	Negative	298.1848	2.51	unknown	4674863	2196803	3036415	5904575	2455732	2016362	2508433	3556430	2805676	3582786	2198192	2951297	2658815	1673637	2169358	3222706	2158135	3959871	2310037
fpHILIC	Negative	146.0591	3.48	unknown	1211368	771692	1070872	965852	861140	842601	857863	853496	778782	920963	977288	779969	854100	614748	1070057	776789	853598	1034732	609753
EIC	Negative	242.0539	2.39	unknown	818198	638159	791142	906787	588597	612894	665911	770129	553752	842525	595657	628656	615881	634603	629514	619934	610964	841971	604452
fpHILIC	Negative	222.0982	2.92	unknown	583826	542661	466095	280217	263844	514300	406519	286666	240570	369146	535014	379426	328007	398334	433674	290803	272113	386916	390678
EIC	Negative	364.2581	5.88	unknown	419197	252737	487703	436989	313048	154860	219588	402739	216736	578110	286864	188361	220468	233474	289724	234338	226249	407248	197389
fpHILIC	Positive	498.3454	0.60	unknown	18936696	21486664	18034140	18004744	18996550	19428674	18704432	15679245	22202024	17686530	24031080	21779008	22225926	21263720	21406188	21215248	19334576	16860508	19682612
EIC	Negative	437.2453	6.09	unknown	952311	1003218	127365	2559802	434284	633989	510154	1194008	1388147	1573851	1265820	447941	1430177	1169047	830006	393160	1044881	1257467	789730
EIC	Negative	296.1581	2.43	unknown	247410	250781	285119	277327	251423	235422	249198	287787	229861	286227	259529	234101	247097	253238	229810	314191	251425	248501	256525
EIC	Negative	322.2965	3.99	unknown	163314	145237	162421	156215	154995	154746	155982	150117	147511	145830	158227	134352	157776	153566	161653	159242	181707	144333	144908
EIC	Negative	586.6330	0.52	unknown	505672	215339	837076	554548	259345	366541	240863	77838	496259	719282	203977	163188	305043	0	168824	405202	142857	190165	588168
EIC	Negative	344.9033	0.66	unknown	39452	52579	44406	82464	22103	52038	47306	37375	26795	26885	54756	63204	44451	4578	31555	50652	40555	43607	26259
fpHILIC	Positive	183.1391	0.42	unknown	3046309	1725258	2776559	4013527	1451018	1698883	1370507	1799395	1080614	2406315	1263937	2335184	2455286	631932	1217368	1620301	1314693	2514800	1017597
fpHILIC	Negative	96.9602	4.56	unknown	76850384	61867800	63793652	69943728	50476180	70887400	54193052	54515536	60928672	75648048	57406316	63284340	65757484	55629776	63819964	70793744	51088424	57097620	59423924
EIC	Negative	239.0768	0.50	unknown	2235159	1628871	1900837	1827997	1450278	1903572	1644056	1302770	1813953	2189496	1790407	1550797	1709125	518801	1429900	1912994	1513692	1559012	2012278
EIC	Negative	319.2222	3.97	unknown	405234	205161	558189	0	450975	336971	407721	212819	178427	632533	208772	634574	512234	155142	159991	178943	218598	939066	151803
EIC	Negative	463.2974	6.09	unknown	493426	649296	229791	1814090	245660	416956	314362	321536	619701	614158	603654	213526	573609	602852	438554	187501	607679	411297	347521
EIC	Negative	334.2530	6.23	unknown	243652	152372	204977	338109	165035	203893	229458	239782	187735	254082	167982	172335	180253	218584	252779	198413	191451	253567	169465
fpHILIC	Positive	512.3338	0.61	unknown	3262734	1879344	2614705	2736978	2184429	2120530	2009714	2665489	1688817	3259889	2670044	2818321	2699109	1752451	2185819	2454026	2344898	2138091	1961053
EIC	Negative	332.1226	3.46	unknown	326031	300969	313058	341820	266278	211589	280137	285380	278257	327825	276185	279088	285676	265356	346575	320135	213762	276960	215885
EIC	Negative	479.2028	3.76	unknown	423910	279990	454097	302647	166814	240469	354887	279048	230184	221510	243598	323758	392398	283805	213228	282085	296488	367503	161926
fpHILIC	Positive	496.3386	0.60	unknown	368306112	422779872	348824192	335452288	377072896	379792512	370486048	311930208	424530400	338563648	440926720	427441152	454886080	415331008	398985504	418023200	395688512	329033344	396010496
EIC	Negative	408.2743	4.98	unknown	329799	309091	305459	296006	333654	276289	272340	298814	274320	336230	275306	305804	305396	278971	296971	335897	286401	322207	271710
EIC	Negative	615.2470	1.03	unknown	371615	203800	584307	432011	170915	112961	267042	331114	133920	302886	309502	276604	332981	94934	172866	198692	170831	428111	86201
fpHILIC	Positive	972.1405	3.32	unknown	910235	447444	499537	374122	312282	406202	414737	242064	548245	311827	253746	668845	486924	539881	286920	409233	330403	429214	544950
EIC	Negative	480.2999	5.54	unknown	4087836	4775320	4144874	3388686	3794941	4830098	3707484	3493824	2577804	2715817	3076222	3803704	2432746	4544484	3561122	3951154	3310660	3791175	4540588

EIC	Negative	541.3347	4.46	unknown	2630817	2270438	2159754	2194357	1957249	2050256	1838791	1870242	2242105	2240327	2129713	2219234	2112580	2120189	2049360	2492385	1829375	2009385	1986004
EIC	Negative	406.2604	5.21	unknown	507806	426017	424370	416032	447098	466719	431636	469646	372165	465921	476539	428693	469613	417232	469364	463251	421101	464425	459259
fpHILIC	Positive	144.0805	2.07	unknown	731933	314911	486786	342203	315387	278367	304519	275158	282020	361808	354056	302266	279175	505039	463306	264433	257616	363814	166632
fpHILIC	Positive	263.1687	2.25	unknown	234919	430467	247586	310099	340086	279377	360292	265683	281197	248928	310681	381505	277651	228687	260134	351474	260451	387242	330663
fpHILIC	Negative	172.0250	3.88	unknown	570228	420444	627932	867274	310827	427700	486696	692161	300948	870276	431309	242531	289896	659559	763084	0	468898	674605	405634
EIC	Negative	229.1442	2.10	unknown	224328	204785	250650	208132	182599	189749	222117	200285	207121	309802	259206	244127	363528	116720	217164	229370	275221	168813	153714
EIC	Negative	275.1620	0.89	unknown	68459	110656	92297	71184	58612	80041	71752	83478	46275	58432	57584	85241	68721	50759	62042	104370	63699	58370	66089
EIC	Negative	288.9420	0.55	unknown	546104	100409	172649	306810	91020	346706	145002	32542	151147	284646	138120	129781	56196	90496	129360	197947	78564	127797	265222
fpHILIC	Positive	205.0816	3.85	unknown	2054918	716866	890604	1903973	751185	1275998	754835	777663	751551	914289	840029	953140	966977	599767	716067	1128738	986737	881836	780847
fpHILIC	Negative	144.1112	0.45	unknown	437019	283538	564020	555001	395854	391380	319651	385623	336465	397846	280053	396339	307541	310388	300033	375572	404949	513383	312693
EIC	Negative	380.8154	0.65	unknown	245014	92535	131107	249837	58750	177972	96418	46134	66353	143728	102624	118341	89828	33057	73078	115986	60514	84958	130295
fpHILIC	Positive	149.0456	2.48	unknown	683622	691615	737077	351952	380350	589068	377312	344847	340344	495853	503136	515464	375069	719760	479056	329257	412604	433413	465422
fpHILIC	Positive	520.3403	0.60	unknown	383323168	394942528	478174720	355023648	308324064	378886464	362608896	369770560	269561568	315178016	418279584	398481536	364397472	432863744	313779456	304237568	339842368	386536480	418069600
EIC	Negative	281.1763	3.03	unknown	1089555	345752	880932	900255	327177	393318	433933	660455	287503	1170034	341414	487818	405001	347293	372338	399569	329565	820681	272574
EIC	Negative	265.1449	2.62	unknown	4381507	3669588	8533514	2640378	3748459	4998491	2627988	3695371	1099045	6121300	3642555	2987307	1469264	4831987	3936227	1816127	2134855	5210852	3781549
fpHILIC	Positive	786.5998	0.43	unknown	42646264	42913056	50660840	40849884	40872592	41489220	35732548	53250992	31655394	45369568	43645212	39972444	34432196	52140028	42958136	35096156	38668760	48383100	46903976
fpHILIC	Negative	98.9559	4.56	unknown	3955875	3138924	3398721	3618799	2690449	3595319	2868681	3042052	3528826	4068587	3042351	3337558	3618174	2851810	3649809	3747586	2674763	3134571	3221437
fpHILIC	Negative	211.1705	0.39	unknown	1509351	871057	1714589	2924184	1227728	1068639	1224144	1311917	1131093	1775628	816990	1547104	1790917	572541	788750	1229985	1267408	2268891	675505
EIC	Negative	325.2745	5.83	unknown	1137906	1143003	2309572	2564048	1582879	951782	1145782	1812983	1176161	1186133	1297162	1257092	1265017	1672282	1385233	808451	1224455	2322858	1228078
fpHILIC	Negative	200.1292	0.45	unknown	3112916	1748013	2159471	3290297	1674920	2369819	2195872	1602598	1932933	2609108	1922236	2323457	1937368	875708	1968948	2819329	1939084	2505984	984774
EIC	Negative	318.1789	1.78	unknown	199267	107340	185313	82755	52779	82151	117384	66258	37961	125963	133017	173616	172581	82909	72089	140138	101490	0	59198
fpHILIC	Positive	163.1153	2.92	unknown	53660316	53508776	47381936	32754484	30817130	49173984	41359608	31680352	25115956	36206436	45152368	44980592	35758012	36586228	42020368	33268290	31533284	34650228	44914288
EIC	Negative	553.0388	0.49	unknown	1520411	1652953	1921429	1822714	1376473	1514835	1582546	1640203	1690982	1669542	1737491	1562996	1773582	110695	1531876	1745417	1655459	1674429	1559887
EIC	Negative	285.2075	5.12	unknown	351665	164650	620249	292126	162611	178707	234879	439173	47619	330021	189223	185975	164342	209587	176682	103363	171259	419349	241931
fpHILIC	Positive	221.0911	2.61	unknown	1597110	1873872	1729694	1114255	1584665	1680455	1777836	1212756	1316716	1344593	1793162	1584863	1608889	1976125	1970694	1686570	1252555	1497676	1520833
fpHILIC	Negative	181.0507	1.76	unknown	20142916	9158250	11131978	15206214	8424294	16350900	8197744	8503857	8081664	14714568	10329509	11163426	9861841	12447211	9329286	8903216	10849468	13351408	9457785
EIC	Negative	318.1182	0.92	unknown	149921	98911	177631	167331	77468	68933	98878	136102	72573	141008	113630	101775	108283	45137	82436	98791	85524	156226	52396
EIC	Negative	540.3312	4.46	unknown	9302815	8260009	7717826	7551285	6822036	6922773	6609199	6798309	7528062	7648048	7520437	7645928	7793271	7305010	7512231	8923693	6306177	6879503	6708900
fpHILIC	Negative	194.9274	4.55	unknown	84022248	70252064	72543080	78855672	57682008	74823952	62112668	65833844	70490512	85447184	63962320	70830632	77033424	65640920	75902560	79096824	59253536	63976876	68510096
fpHILIC	Positive	293.1452	3.47	unknown	1121845	666057	808635	794836	737260	741760	726134	770673	748496	876102	836831	679380	797376	690223	979590	689498	761355	928716	544762
EIC	Negative	378.8184	0.65	unknown	148939	54733	77503	145503	34223	113288	56799	16532	43710	94669	60869	72361	51597	20339	43649	71828	34258	50855	76774
EIC	Negative	247.1703	3.84	unknown	203046	244327	137976	147136	195360	176479	186579	173278	161756	140166	205060	180911	203518	148753	178174	153802	179894	193081	231963
EIC	Negative	372.8511	0.52	unknown	1043796	837733	1348957	1244933	806377	899152	790924	628677	1074369	1132619	825363	830028	853793	198146	796599	998840	747128	974899	887556
fpHILIC	Negative	196.9233	4.55	unknown	7445866	6162219	6332557	6915964	5076767	6391476	5364101	5776647	6208617	7416736	5297311	6197246	6585361	5975611	6599829	6981664	5039400	5614366	5994781
EIC	Negative	345.2104	6.31	unknown	4179148	13122685	15331457	11138244	5199947	12352622	6143649	11101238	9920617	11342802	20451748	15849263	5495648	8860131	15412079	8180920	9572243	6224978	7525863
fpHILIC	Negative	97.9595	4.56	unknown	624266	561410	562030	609397	475067	630525	525965	503185	528714	702998	530258	502422	568155	462340	576184	647344	496989	522067	552295
EIC	Negative	257.1760	2.83	unknown	287350	202088	271247	290814	219759	269670	232082	275336	156871	331566	182746	317346	299329	137524	183533	216557	218096	396364	196261
fpHILIC	Positive	806.5683	0.43	unknown	42863012	76773720	41393884	51206724	50775044	50160716	47898896	39010360	74052000	43375764	65420536	58861048	63355756	60544148	50604868	69423384	58319928	35497268	61112780
EIC	Negative	450.2632	4.06	unknown	871974	1322233	1179081	751477	1041766	1633022	1028592	867526	724273	728789	1038222	932719	726943	1900677	1268886	1048995	893767	951744	1397267
fpHILIC	Negative	166.0132	2.98	unknown	734693	409194	663101	637145	435547	472526	359980	389385	368888	571303	449372	464605	498822	256147	396093	358918	485981	498887	388613
fpHILIC	Negative	304.1302	0.93	unknown	514136	402953	317585	385284	455785	243315	488384	318200	432233	358937	404239	364762	326800	351265	410703	544138	328049	421888	330858
fpHILIC	Negative	818.5558	0.44	unknown	18943132	18255506	17402424	13360236	15265879	13307121	11234166	12746728	18403352	14599579	16859006	14533113	14291114	19891282	16544258	13481533	13708244	11799348	19034628
EIC	Negative	556.3009	4.36	unknown	839384	797631	883302	873798	802018	696816	751624	817043	796978	877899	854021	861308	815885	796944	830273	865947	712722	863696	788548
fpHILIC	Positive	279.1000	2.97	unknown	5256625	2679299	4529694	2588100	2189403	2670938	2782004	2478035	2644615	3678742	4295438	2550224	3495867	5053840	4791288	628979	3783678	1735814	2478180
EIC	Negative	466.2209	0.88	unknown	169150	231307	117911	84703	108420	127296	133536	156694	111892	116018	194649	175834	151444	155469	121241	146755	103124	116359	183117
EIC	Negative	478.8991	0.52	unknown	1129949	1283362	1215254	1495708	862881	1022407	1070729	1302788	1225588	932865	1250181	1384976	1295866	117341	957446	1128606	1266119	1223141	1192291
EIC	Negative	232.8893	0.52	unknown	969488	676527	1131882	1002526	785007	723775	652278	522004	859046	1139831	647411	627680	668521	557785	622076	779470	615277	758389	950208
EIC	Negative	238.1893	5.15	unknown	846436	447403	1673813	2459842	162593	147465	147311	333107	186383	879568	217086	1766301	285058	216423	105910	145049	103794	352316	320501
EIC	Negative	268.8011	0.63	unknown	1542198	1033997	2341999	1724282	1230990	1296474	1096110	903533	1622529	2975805	1101988	961703	1220488	852895	675355	1329271	794263	1408438	2261712
fpHILIC	Negative	845.5735	0.43	unknown	2994355	2620983	3148633	2326448	2082677	2397910	2263395	2247568	1662310	2226380	2280396	2467174	2129964	2826459	2540085	1903102	2032165	2378092	2354420
EIC	Negative	355.2133	3.25	unknown	1884018	339193	2134743	940215	390949	553444	593640	1225095	154513	1288152	292533	582279	304900	335156	361761	112066	323066	1824335	448632
EIC	Negative	548.3011	3.23	unknown	889658	967393	785611	1038358	835414	823978	1101207	767220	692860	870669	921340	1171332	990505	940846	1041563	1003137	848173	747695	801462
EIC	Negative	606.6841	0.52	unknown	565725	379985	684526	750989	443514	373816	448640	290034	499779	598988	401334	382098	531419	0	360839	421178	349697	528526	504512
EIC	Negative	473.2702	2.98	unknown	1887631	658526	1981508	3886898	887227	1284211	1762474	3608739	2210977	3876535	1334236	1169790	2454350	179423	720238	1387466	1978101	5772461	413903
EIC	Negative	234.8864	0.52	unknown	1041815	684387	1140618	1031356	769337	771130	720772	570340	866344	1186057	673765	660736	688172	663400	674687	819825	612673	821122	966653
fpHILIC	Positive	521.3439	0.60	unknown	112195776	116988704	140081232	105653080	89023600	111678704	105468408	106236704	79477248	92893376	121639328	118737856	107307792	126084576	92295272	88953032	99477128	113651016	123760904
fpHILIC	Positive	788.6080	0.44	unknown	6384669	5907388	8025358	6227159	6527480	6401377	5288499	8179143	4913506	7256315	6800047	6282826	5744911	8386257	6212504	5315092	6184481	7785984	7485042
EIC	Negative	246.0770	0.87	unknown	179770	211689	215457	107435	118971	229941	104983	126744	93559	265779	202785	147988	126698	271860	122661	157359	105747	223342	101487
fpHILIC	Positive	497.3421	0.60	unknown	96513864	113725960	94226704	91118504	100847952	104263088	97937440	83530240	112569280	90449136	120563128	113423848	120753152	111954128	113694744	112953968	105254216	90256872	104059288
fpHILIC	Positive	198.1235	3.34	unknown	549795	164467	225150	253771	201342	244573	341528	304197	356323	299594	263275	197887	255523	345871	338550	237800	353601	251017	345593
EIC	Negative	317.1758	1.78	unknown	1003204	442371	884026	382663	211899	320706	457941	277472	192823	526618	535080	795805	803361	348669	292534	577651	425357	0	246048
fpHILIC	Negative	335.0489	3.00	unknown	13219220	9131560	12020490	11304754	6796860	9361474	7261211	7759750	8287643	10930273	7680436	7348715	7671704	7583018	7602816	6603633	9331285	10459888	7665003
fpHILIC	Negative	846.5872	0.43	unknown	7728279	6855270	8160090	6084187	7352179	6486872	5568205	7975406	4748126	7471055	6702705	6089188	6188066	7651152	7182981	5706176	6234338	7566175	7232813
EIC	Negative	362.2076	4.91	unknown	299657	588980	704510	515862	313338	433514	369683	552750	515710	612821	861348	769893	353736	413424	678694	386434	499852	382989	359020
fpHILIC	Negative	324.9865	2.05	unknown	4364574	2757174	2967643	5898776	2004095	1195531	3416323	4853357	6354835	4219674	3100783	2583778	5966967	7181051	4994659	1162639	6137735	3629923	2904672
EIC	Negative	636.3501	5.14	unknown	1275071	824232	1105354	1187264	971884	696222	790951	1148427	1067843	1468229	910789	846894	982823	919723	885419	964318	923680	1306885	860306
EIC	Negative	549.3044	3.23	unknown	271737	292493	219991	313271	255529	251151	314565	223151	205878	261971	278658	360185	287956	271774	315853	291460	243886	230459	239740
EIC	Negative	333.2802	5.91	unknown	152575	103510	149210	150468	94324	79155	91770	126971	82168	145770	101120	97492	96315	87719	109249	116312	88002	114229	73615
fpHILIC	Negative	278.0892	2.96	unknown	432768	257651	320916	251845	207567	227436	270053	195849	205770	377293	359495	237854	280340	525263	417712	47769	293474	182126	228688
fpHILIC	Positive	276.1549	4.35	unknown	995791	1023596	648822	753638	832143	1643071	783344	489466	598882	706918	963332	1239944	1004530	879237	1101455	1091971	803195	648128	949995
fpHILIC	Negative	172.1426	0.42	unknown	232486	391562	698700	366115	411285	404618	187079	416317	340024	393470	149622	360965	531471	393168	283333	460416	334130	423383	358934
EIC	Negative	361.2055	4.91	unknown	1422183	2742058	3516081	2601076	1714384	2167374	1744624	2627975	2537053	3038121	4303360	3580787	1691150	2115094	3243468	1918161	2497270	1797163	1957710
EIC	Negative	234.1496	4.61	unknown	572975	814502	461193	447146	612716	653083	748216	504919	606830	430510	978251	748128	622116	734949	824833	735229	707566	435696	603426
fpHILIC	Positive	229.1542	2.85	unknown	1992944	1302788	1020798	1726351	1026047	1594876	1250800	749512	1378519	1275698	1327647	1428386	1394071	980884	1215556	1968710	1021366	1082707	1058842
EIC	Negative	298.1848	2.51	unknown	4674863	2707981	3036415	5904575	3082765	3460727	3252394	3556430	2805676	3582786	3520499	3386686	4186012	1701314	3383357	3911550	3537610	3959871	2310037
EIC	Negative	317.1146	0.84	unknown	1149076	856558	1740570	1517271	720215	477417	979809	1089793	505439	1167290	1099877	860052	1056979	383400	600034	883292	661320	1459048	389543
fpHILIC	Positive	261.1434	2.69	unknown	2823309	2007599	1674944	1133804	1800414	3310158	1514868	740125	1403006	1342105	1888585	1966054	1558204	2183517	3452024	1817196	1187048	1116059	1715445
fpHILIC	Positive	230.1583	2.03	unknown	496689	408817	294854	457903	291156	465631	335166	229361	318308	318507	345192	386569	363478	259857	286145	527462	303116	298530	321723
fpHILIC	Negative	323.9917	2.05	unknown	453725	301191	278318	613593	286079	142647	369893	526506	676048	434430	374129	270200	631383	721568	611855	143448	679756	394876	290359

EIC	Negative	448.1698	0.71	unknown	682479	580772	798567	321002	463919	564141	586763	341325	484805	760895	619657	482297	486349	821789	507352	469851	404172	285909	920934
EIC	Negative	436.6609	0.62	unknown	85113	65686	114849	85953	75666	66850	61881	46310	85908	131396	62251	59734	60983	44846	55694	72662	48514	67099	113209
fpHILIC	Negative	554.3087	0.60	unknown	2094129	2500717	1932844	1790738	1675369	1838551	2100386	1683861	1735862	1869012	1845295	2297722	2143183	1984091	2178790	1991790	1912780	1514767	1988602
EIC	Negative	440.7698	0.65	unknown	95458	47057	55063	100901	22357	73374	42603	28299	29767	62515	52750	55068	40624	8590	31227	55451	27188	40854	53730
EIC	Negative	371.1402	3.94	unknown	206499	167223	269775	149331	106301	261949	188455	158800	143272	121419	187437	258245	263639	223826	92282	226379	186437	268270	138920
EIC	Negative	531.2732	5.75	unknown	2434145	3181633	1998810	2321195	3197289	1633180	3139883	2199014	1375588	1692457	2170244	2382078	2243130	2673895	2383815	1413349	3120663	3228443	2129700
EIC	Negative	249.1126	3.07	unknown	14203	55169	32287	112335	62324	67636	17560	15860	68081	74707	45600	89731	44059	41687	26152	58068	73788	68316	19895
EIC	Negative	263.1654	3.31	unknown	260410	242686	270097	301924	199429	185847	206478	214166	155224	254794	229033	212765	238114	164328	172313	198836	184632	219095	247716
fpHILIC	Negative	196.9316	4.56	unknown	1355634	1085519	1161172	1197046	936210	1159413	911691	1068496	1132181	1411472	1099599	1133078	1265411	1023444	1215731	1214119	1006522	985650	1119714
fpHILIC	Positive	343.1641	3.39	unknown	1695127	1636039	1079391	1030365	1307720	1418310	1143639	1172056	1406606	936253	1068717	1439742	1431713	1792042	1525428	1464750	1233801	1698780	942058
EIC	Negative	250.1448	2.24	unknown	1136666	611596	549780	1535696	669980	1184918	695745	578164	532395	852011	795618	910246	682311	294761	1079109	939959	877592	603492	399285
EIC	Negative	619.0205	0.48	unknown	242947	306383	284062	314669	253807	272163	261669	307143	322894	308944	288123	329413	289212	49838	239216	315104	352822	329158	273702
EIC	Negative	318.7466	0.61	unknown	104993	70031	112950	104199	87270	81184	67206	50937	93639	135934	68071	59783	80978	74179	64173	71610	72458	82062	114770
EIC	Negative	446.1721	0.71	unknown	1954194	1662116	2275655	946577	1306276	1650947	1673510	934702	1402974	2164649	1768025	1403701	1402885	2344531	1463771	1315878	1141250	885248	2620462
EIC	Negative	447.1756	0.71	unknown	391455	321807	442072	178771	249724	320977	327429	181300	268796	422834	333015	276025	267646	450196	288362	252463	218475	171926	506330
EIC	Negative	519.1675	1.87	unknown	43329	365089	0	0	65758	144810	149437	0	207587	0	228211	86027	221432	172730	166995	179976	112726	0	145918
EIC	Negative	619.3472	2.85	unknown	332807	964480	1075252	686010	366471	720018	475704	605402	266979	972764	881040	605987	277314	97125	496529	519803	573692	565840	441919
fpHILIC	Positive	167.0483	3.24	unknown	6010418	5450413	4464935	4633675	4083561	5024510	3538748	3140111	4684075	3594710	3385319	3932692	4299980	3547298	4042542	4860725	4333110	4552029	4060657
EIC	Negative	366.2728	6.01	unknown	201904	140223	222689	161158	134717	83202	141434	168909	123336	201718	137767	117283	104859	131740	161039	178801	103057	134743	118041
EIC	Negative	505.2087	1.15	unknown	164496	131033	121032	67381	80853	117984	164198	45878	112146	122181	88829	146292	99021	75998	96198	142069	172629	80104	65459
EIC	Negative	274.1848	2.48	unknown	1163381	200957	649458	1538850	280092	230945	351391	1149665	500284	1069554	221411	358888	286019	74642	189842	352488	261242	1680835	198680
fpHILIC	Negative	147.0663	3.47	unknown	1056363	673873	868267	764074	623113	674118	606061	582503	567338	839896	671037	699661	651975	533703	832364	540248	588325	869795	430025
fpHILIC	Negative	291.0385	2.69	unknown	1115317	247244	132522	131411	187872	387486	0	173095	343315	73143	82831	203659	182198	161465	82684	306350	249605	344758	182810
EIC	Negative	635.0414	0.48	unknown	1359243	1445440	1644945	1622353	1134278	1305670	1327648	1379160	1469255	1554348	1477313	1430074	1513552	84216	1335949	1538709	1345493	1551355	1296673
EIC	Negative	260.7875	0.61	unknown	172515	133390	200817	176501	180589	139905	142163	103124	175966	239139	127049	113381	137179	204453	136467	134869	134016	154696	232683
fpHILIC	Negative	309.1087	3.54	unknown	726318	363274	426283	397090	279590	418719	245975	331874	234201	450999	268747	234200	240293	312463	318232	244025	317105	486731	248864
EIC	Negative	325.2384	5.94	unknown	570127	337041	1441177	601833	344692	0	432455	908474	0	645908	432223	530011	354464	338002	273968	0	322572	738870	466956
EIC	Negative	242.1841	3.53	unknown	238615	162683	324371	305066	151322	179553	179518	211152	152806	245448	190701	282224	266488	176612	149495	193496	179371	278364	134356
fpHILIC	Negative	357.0292	3.01	unknown	559048	493372	549956	613589	399990	414505	403609	434307	445469	667474	427004	384097	457831	407593	391331	404628	474040	533885	439852
EIC	Negative	299.2592	4.34	unknown	0	5600672	5056496	4970579	5236493	0	0	0	0	0	0	0	0	0	0	0	0	5600907	0
EIC	Negative	475.2665	3.99	unknown	1320997	1592166	1750083	906464	1078938	1715215	965830	1226881	646415	759142	1311871	862809	731300	2633706	1307135	400878	1094949	1284861	1938276
EIC	Negative	476.2695	3.99	unknown	235219	313351	362057	192494	226940	341540	205544	233122	130501	170565	276649	186372	177819	497074	253373	85089	243881	253890	380871
EIC	Negative	570.3424	2.89	unknown	182052	228067	165117	130506	140070	276703	155547	107012	0	136229	122693	156170	137270	343036	163953	183464	138031	0	241379
EIC	Negative	570.3417	3.94	unknown	400416	330554	337203	292325	311502	401531	345189	340789	231287	389452	450777	393939	280770	425351	433335	311148	271325	304255	426894
fpHILIC	Positive	173.0916	2.59	unknown	1513751	1374785	1018829	901548	943802	1705187	1126775	560219	852228	1038198	1156341	1345487	823914	1177730	1683896	1215563	624314	1181364	855168
fpHILIC	Negative	203.0678	3.86	unknown	723789	274992	317226	562244	242231	447358	230733	260960	250200	320800	278083	278142	287513	192162	256415	383469	306508	331519	256734
EIC	Negative	315.1602	2.01	unknown	979369	816613	860948	459411	408148	603987	969807	270217	398040	0	866899	1264457	1135640	489649	541617	1062471	702870	0	535160
fpHILIC	Negative	358.1694	0.93	unknown	890381	328198	640306	924704	377795	353237	389052	822870	745073	537768	268241	435592	416647	400375	436414	615559	559446	908908	362468
EIC	Negative	380.6992	0.62	unknown	99898	82809	141209	96883	95421	74400	81782	52559	101435	155762	73493	66602	79443	90716	71463	75299	68663	84184	135433
fpHILIC	Negative	571.3445	0.62	unknown	878809	944589	593678	661869	564093	930218	738109	610465	1024185	700095	832649	769137	893144	787851	1005429	918603	782779	571128	870590
fpHILIC	Negative	125.0066	3.22	unknown	1605081	1134842	961849	1334921	1016021	1290270	1040987	1041359	1189156	1073775	1098425	1230662	1133656	1042547	1100754	1475015	1148033	1185318	1075672
fpHILIC	Positive	328.1602	2.78	unknown	6021232	5481088	6308594	4498547	5143956	5483098	4425707	5081931	4496992	5206993	5978399	5599274	5182233	6098532	5093180	5012627	4965361	5597993	5766962
fpHILIC	Positive	792.5925	0.43	unknown	677792	764690	1032787	860168	850039	803136	611989	727549	828069	630324	760748	833107	757103	799603	645412	1028152	840756	817306	770922
EIC	Negative	301.2385	3.28	unknown	148379	81960	320489	139747	218636	147183	75970	175055	63287	152633	118386	102268	113326	108795	91731	191368	130133	256723	60578
EIC	Negative	345.1714	1.53	unknown	289583	241424	0	289492	64462	184795	121089	0	69177	0	139055	213606	119207	163416	119813	161390	72324	0	118669
EIC	Negative	299.2592	4.40	unknown	0	7626982	8054837	7985151	7889285	0	0	0	0	0	0	0	0	0	0	0	0	8475975	0
fpHILIC	Negative	409.2351	0.61	unknown	3786327	5375044	3097774	4150198	3757244	4001725	3629729	3096207	4944257	3719511	5449861	4484941	4777755	4623173	4771780	4307461	3994326	3285252	4526963
fpHILIC	Positive	344.1325	2.99	unknown	458060	415715	672440	402656	274597	367545	258971	193298	155164	304946	259260	251205	279970	201332	276759	216942	279154	358049	212422
EIC	Negative	424.2475	3.95	unknown	592535	656321	815449	522289	460278	763807	514279	591676	345617	478558	566057	608025	368804	1222221	516970	323783	539273	675927	820494
EIC	Negative	363.2544	4.04	unknown	62956	28972	91834	207788	41349	64394	57928	73478	55484	99305	53539	81380	65362	43005	36874	56095	47700	137579	36420
EIC	Negative	368.9473	0.55	unknown	1003205	248033	394934	753425	214485	785456	321370	52361	249650	513454	386369	335368	255357	134942	322081	482691	171792	306496	463823
EIC	Negative	322.7401	0.62	unknown	150121	109188	175112	143815	135079	111695	113902	96202	147798	216944	105972	87046	113682	127904	106195	115784	102595	125763	200622
EIC	Negative	560.5910	0.63	unknown	245699	155367	455662	333157	158094	199456	152905	121258	273360	525351	158378	140811	187688	41862	134593	204420	135725	234624	265338
EIC	Negative	403.0807	0.50	unknown	1855441	1310713	1356686	1280536	1081374	1379101	1304631	954469	1417053	1723698	1413938	1165455	1309324	0	1097415	1515245	1247806	1154008	1596773
EIC	Negative	479.2975	5.31	unknown	5544042	7732261	6967702	4845319	6297257	8684303	5312841	5493158	3240845	4187185	5869769	5343902	3617982	10171927	7091609	5070174	5667720	5809270	7637767
fpHILIC	Negative	125.0107	3.22	unknown	5716196	4149964	3777384	4113197	4043682	4509047	3708628	3667909	3825316	4013220	3668430	4249928	4539084	3087093	4279999	4712538	3916966	4655529	3724056
EIC	Negative	318.1181	0.84	unknown	199918	149847	309991	275274	127528	92357	171346	193913	89323	213949	181834	155281	192258	71626	107748	167544	114973	267450	69479
fpHILIC	Positive	148.0423	2.48	unknown	12416931	13170928	11725690	6507676	7629873	10771512	6799351	6890212	6870754	9801472	8382051	9952264	7172460	14570954	10890050	6866848	6670004	7372257	10210211
EIC	Negative	608.3202	2.26	unknown	533612	496342	412543	393939	344439	448585	409784	443488	327958	529084	413882	565885	264043	515610	384979	365409	373118	321931	683328
fpHILIC	Negative	325.9897	2.05	unknown	465152	275847	279728	620576	223723	145042	350008	496867	675560	422348	367955	275503	540282	796012	516654	111111	633400	481147	297056
fpHILIC	Positive	483.3626	0.60	unknown	636678	665924	844396	782814	881030	507498	657503	580777	633619	584026	574950	802727	543461	458848	725158	633601	551112	802762	803721
fpHILIC	Negative	847.5889	0.43	unknown	3817415	3376891	3666013	2850987	3391424	3304820	2980161	4383803	2543390	3602130	3253063	2989891	2838802	3780986	3744579	2647960	3436952	4111999	3966328
EIC	Negative	270.7983	0.63	unknown	997532	500952	1511460	1142189	780198	861333	718339	560630	1023035	1971081	713371	620911	788734	543347	438080	876008	526247	921204	1492036
EIC	Negative	557.3628	4.83	unknown	1268962	355492	1313735	391863	569188	469071	1347410	386027	1476364	387232	394288	401912	394131	455494	1400534	1357065	1320718	252051	252017
fpHILIC	Negative	176.9356	4.07	unknown	827279	324156	156355	1692421	292567	1147237	304384	114604	99112	398421	484913	1066234	429216	156936	125593	825416	468706	423377	268930
EIC	Negative	542.3378	4.46	unknown	513704	520393	438420	401593	398183	371059	361347	365780	439596	407435	379979	391649	423473	418842	441799	489403	352909	407743	396916
fpHILIC	Positive	875.5262	3.33	unknown	535864	736679	691002	463066	401161	974341	606194	396185	525107	384352	443839	911442	611845	690630	808095	711439	676603	541315	395819
fpHILIC	Negative	557.3289	0.60	unknown	17417998	21248108	14722768	15463185	16847012	17287144	17047906	13328837	21738530	16651415	19920080	19536980	21157342	19276690	21143672	20144590	18009338	13927077	19900502
EIC	Negative	264.8976	0.62	unknown	355905	54097	135681	276835	80892	142256	59152	63571	37032	162206	72026	158848	53261	48532	63646	80171	82394	37514	149732
fpHILIC	Negative	228.0809	3.34	unknown	617987	485722	432847	478061	443038	553408	531181	424352	564363	506966	411874	423066	498015	581046	649610	457462	559263	449901	601767
EIC	Negative	226.1446	2.45	unknown	278867	173868	222367	920332	126950	183597	143074	249931	340641	384031	234756	308322	265655	106076	177090	306088	205284	310175	129561
fpHILIC	Negative	252.1603	0.40	unknown	1469480	818777	470514	1619881	1009938	1417786	1053722	871464	495869	1057202	1007084	1179624	1385887	509155	1263291	1201832	1245477	882311	646965
fpHILIC	Positive	416.1833	2.61	unknown	6974112	9303790	8161866	5234695	7690156	6757284	8762947	5768936	6102512	6927569	9093968	8253120	8017883	9684957	9750061	7953260	5940601	7307926	6964245
EIC	Negative	478.2943	5.31	unknown	20675739	28725342	25928867	18096817	21607381	33286939	19702506	20424591	11654637	15519433	21987722	19993135	13329606	38220248	26721217	18577490	21085463	20716941	28221527
fpHILIC	Positive	126.0145	3.23	unknown	730108	588474	487401	519131	369480	610310	435434	356111	505166	396748	424117	544761	524563	429102	451688	665065	479767	472132	393290
fpHILIC	Negative	255.1602	0.96	unknown	734328	243233	781843	560801	230271	216713	304304	740297	240179	988441	177030	208798	208813	218537	411958	248094	250588	847018	235855
fpHILIC	Positive	387.2163	0.35	unknown	397605	487162	486516	500996	378901	340149	372750	412624	333928	520270	436749	464797	502395	447328	374314	410937	371163	434361	398000
EIC	Negative	283.1921	2.67	unknown	505146	208658	448982	558711	225956	358200	268173	325386	257313	562888	278625	569268	445826	346536	257628	304464	260002	459817	270720
fpHILIC	Positive	146.0267	2.47	unknown	1792807	1747773	1450198	644051	828841	1027494	676008	767692	630427	799260	967638	919907	800833	1280914	1134832	643429	651838	781710	1041344

EIC	Negative	410.1712	3.94	unknown	466382	386923	728564	343519	212671	677039	423414	323370	277248	249680	460279	626771	688572	513047	189936	463675	476584	633973	306904
EIC	Negative	578.3460	3.68	unknown	0	5433857	3448319	127807	118890	111653	222527	5125620	0	91333	103201	4199488	4578946	3756500	0	75739	114267	5769784	77960
EIC	Negative	286.9446	0.55	unknown	872706	160036	269481	459512	142388	559090	202412	71622	155034	459750	210041	209405	153290	138381	230917	314143	72506	182523	423600
EIC	Negative	282.1798	3.03	unknown	185771	64003	149960	165722	61431	73849	71302	109301	56213	222262	61466	100575	72907	66421	70334	69066	67312	146956	49128
EIC	Negative	257.1760	2.36	unknown	357316	166432	339556	338619	194389	239278	244942	283551	219289	292854	221167	293002	283195	183115	228235	304699	206206	366490	173377
fpHILIC	Positive	128.0176	3.23	unknown	4752617	4177390	3220444	3559559	2911449	4033249	2614989	2376250	3473753	2608844	2491417	3041849	3238444	2794687	3127300	3924745	3209017	3558128	2869557
EIC	Negative	293.1741	5.18	unknown	208256	144739	169835	505321	207411	161801	180305	205462	256739	235848	181166	213376	348422	62729	133171	403003	199283	257065	103289
fpHILIC	Negative	161.0563	2.94	unknown	744071	286936	190410	275421	192751	542840	178104	197605	266207	256121	238244	348686	244002	214869	200874	322558	349947	286983	332677
EIC	Negative	476.2785	4.47	unknown	76972350	91444216	87094888	66126309	69381394	102600825	66585162	74772189	48274252	62754981	81783098	65642012	53945263	116117014	96436954	47370388	74207780	78288018	95809161
fpHILIC	Negative	245.1137	3.03	unknown	531653	342121	334954	255216	287065	566659	287564	244432	255993	294524	369429	375772	309313	348203	464464	333747	336850	242287	403067
EIC	Negative	255.0929	0.75	unknown	144227	68972	53498	179352	63657	206370	175995	0	46418	81186	116101	155943	121413	164663	97000	146990	93954	73721	122423
EIC	Negative	620.5429	0.64	unknown	101920	52592	156641	113362	51907	69283	57349	44671	85191	176203	53575	37055	65124	0	35640	71487	31075	80573	115729
EIC	Negative	477.2815	4.47	unknown	20271228	23487179	22825407	17297280	18293321	26819409	17546386	19517860	12441627	16790980	21346072	17196054	14283766	30466156	25081037	12405469	19353272	20607557	25195161
fpHILIC	Positive	318.1296	3.39	unknown	298936	270352	454440	408731	244756	239499	377091	279736	156463	558553	389226	270313	374533	145180	337957	136757	299598	315149	318153
fpHILIC	Positive	177.1232	3.39	unknown	794317	386772	758269	667439	387030	519317	464018	488393	365496	517824	416161	561729	396053	439120	445134	484069	405302	628588	450910
EIC	Negative	478.2844	4.47	unknown	3592097	4755542	4120563	3171947	3306923	4970058	3173660	3509472	2382874	2949177	3786161	3207870	2523711	5410651	4478987	2295541	3752781	3821669	4474125
fpHILIC	Negative	595.3438	0.62	unknown	732414	709430	976122	566452	634857	816268	608554	724225	442398	612732	796126	732683	589767	840723	603818	625217	683566	676867	914615
fpHILIC	Positive	149.0595	2.06	unknown	2094140	1165616	1665184	1168837	1113898	1173865	1088996	1112606	1199278	1341338	1190345	1391463	1001016	1670561	1624907	1236882	858393	1398501	1061944
EIC	Negative	251.0924	2.53	unknown	194894	166818	140286	216400	162380	153718	165435	148609	141124	194216	152020	139803	162634	152758	169862	146172	161942	209588	150628
EIC	Negative	322.2440	5.22	unknown	1401733	1264063	1285769	1384223	1259788	1154342	1173245	1327292	1129492	1325275	1299268	1239134	1174118	1190423	1232866	1399177	1277424	1337158	1322728
EIC	Negative	490.7658	0.52	unknown	813239	495034	952611	843264	508288	580772	458931	463958	681897	835459	466604	426793	515099	57045	432216	651583	535755	599741	709127
EIC	Negative	464.1822	0.71	unknown	313126	269255	376280	151428	212089	263020	271184	151667	222218	340504	279086	227857	224740	379316	239495	214858	194112	144698	425310
EIC	Negative	320.7431	0.62	unknown	178095	126620	212647	176623	174225	131428	134837	92322	184898	257831	130653	106253	137151	141411	123978	140389	125593	152306	238741
EIC	Negative	373.2022	3.94	unknown	231244	196332	466340	226534	116908	388742	202220	184184	146948	136532	205714	361187	398229	278250	90439	280245	261734	323846	174956
EIC	Negative	330.2523	6.50	unknown	13907329	8399113	19176256	27235358	570727	17055422	12453039	986951	11590786	14607104	12202137	871720	10133475	703937	836327	10055406	12548342	22310220	756638
fpHILIC	Negative	336.0522	2.99	unknown	1629343	1064299	1343894	1365054	865533	1073075	879471	917993	1006358	1433724	930845	888016	932479	920235	924450	842515	1206283	1311299	886405
EIC	Negative	344.2176	3.63	unknown	338356	84867	470008	70781	38274	112352	59811	220840	33135	211974	54219	134935	42457	112144	60710	19323	87211	170076	169256
fpHILIC	Positive	127.0211	3.23	unknown	647376	558716	445517	484507	375084	492557	377959	356869	431391	314164	339519	445467	434594	433689	426577	565731	392254	461772	372405
EIC	Negative	550.7204	0.52	unknown	545766	310873	729242	788081	401736	408259	472247	283961	519282	638030	426963	446834	600749	0	393259	470720	410006	531161	471940
fpHILIC	Negative	126.0112	3.23	unknown	1702345	1174844	1182775	1362503	1011988	1319166	1044969	1054243	1195539	1090432	1120345	1131258	1115599	938543	1211034	1228400	1156130	1461052	1086246
EIC	Negative	438.6577	0.62	unknown	85924	77094	134361	97824	85677	72025	72409	53591	94428	154665	66122	59247	74796	52723	67461	73576	62803	81278	130614
EIC	Negative	582.6385	0.52	unknown	534548	226970	537682	632954	316309	448205	275389	95714	480782	701173	217150	220020	267441	0	163730	430722	239193	388138	544661
EIC	Negative	523.3650	3.15	unknown	152706	114656	126288	108204	104663	100436	101708	151410	110570	161058	118689	105317	84372	73518	112966	145685	106821	154470	101755
EIC	Negative	253.1446	2.68	unknown	3976801	6490650	3686580	4945227	4374985	2265114	4555817	2660394	7727679	3409150	3739124	3811627	5546613	5422439	5496144	7799518	3563939	3399774	3334005
fpHILIC	Positive	210.0951	2.38	unknown	602246	335620	220020	288390	303938	462154	359175	149786	212433	300842	494942	478515	464598	370497	401706	358821	281908	241075	342568
EIC	Negative	365.2693	5.92	unknown	899656	687093	1153004	824204	637184	483319	730255	916409	642376	1062548	633779	590226	547252	613782	914136	858946	518369	817425	620597
EIC	Negative	346.2138	6.31	unknown	931035	2786047	3112032	2282156	1063291	2500540	1179220	2211522	2032920	2422912	4176276	3192900	1195227	1811827	3176297	1683598	2028878	1300936	1643309
fpHILIC	Positive	248.1116	3.18	unknown	681801	248154	221069	434691	279201	403315	264635	207446	450260	249609	332968	320324	347496	375359	249301	542875	301060	358503	304320
fpHILIC	Negative	857.5180	0.38	unknown	1939142	1387752	1212475	2275543	1265861	956944	768889	1432116	2119183	1635352	1557055	1441506	833767	1542719	1288966	1420457	1557109	1294419	1601626
EIC	Negative	234.8865	0.59	unknown	263451	211816	322927	257632	240061	207890	212432	171427	265390	393832	216898	203766	227017	227891	202803	237490	184084	232944	343880
EIC	Negative	253.1810	3.45	unknown	143638	88064	182500	134926	72423	93038	113094	114369	97004	117295	77954	139879	143105	84192	68263	98574	103022	177743	89172
fpHILIC	Positive	875.1299	3.32	unknown	1111313	788963	1084933	1138570	1016413	954685	609338	786197	890603	907140	664475	977487	878841	986540	1214430	1007692	564938	783053	1094718
EIC	Negative	511.3641	3.13	unknown	143605	65362	79015	110479	55996	93281	59991	101596	23218	97665	57121	36303	33996	46986	65014	50254	55839	143235	51839
fpHILIC	Positive	244.0790	3.16	unknown	3732045	4534697	4858377	5062489	4428119	4194749	0	4607154	4002664	4283765	4215127	4181502	4361154	3479620	3816796	3820484	3748540	4062444	3443292
EIC	Negative	492.7622	0.52	unknown	587302	359600	755214	633503	432167	452306	362978	343266	596562	678472	385229	381861	402824	73929	383691	516164	426018	483196	589379
EIC	Negative	241.1808	3.52	unknown	1604395	1007679	2069910	2004960	968841	1157117	1138297	1335263	1004875	1503401	1222903	1744613	1655258	1129778	996079	1232532	1201256	1820666	896488
EIC	Negative	366.3461	6.85	unknown	51441	86231	69466	127773	55766	90832	76001	79796	83787	122756	118231	190766	118900	70142	77083	92063	82133	104279	68115
fpHILIC	Positive	923.5700	3.18	unknown	845977	598610	908108	951370	700514	772005	616989	774587	558449	811730	729525	634821	772500	1201696	833073	752895	771249	677792	719904
EIC	Negative	347.1458	3.72	unknown	355797	155440	270870	368882	160785	185528	210162	211371	93185	275754	159415	261136	241669	76609	178586	185238	148175	326478	106354
fpHILIC	Positive	203.1385	3.13	unknown	691722	503621	407201	509359	417873	581522	455422	238970	376566	394457	396593	332936	406282	254578	424668	554584	449641	371151	472067
EIC	Negative	487.2862	3.46	unknown	150416	69548	182738	294681	79073	116021	138429	198929	136694	339761	120843	132360	158996	0	0	122400	143250	429889	38831
fpHILIC	Negative	131.0463	2.88	unknown	2307393	1670446	2394713	2828712	0	3900726	2099285	0	0	2508390	2151260	2479317	2164535	0	0	2629267	2704302	0	1252229
EIC	Negative	323.2227	3.73	unknown	61640	48110	140507	179533	66702	59580	71327	99966	94215	67563	46220	55046	68187	41925	45642	77761	63815	108998	133835
EIC	Negative	531.3325	4.99	unknown	385013	376899	365384	778832	353258	417621	482960	391838	383784	659076	493251	440990	497602	0	296508	406682	514361	730312	280726
fpHILIC	Negative	209.1183	0.40	unknown	1349406	667457	1420765	2253078	872659	698489	860200	1144560	979811	1426658	782495	979668	1124771	338800	754984	1098427	1083590	1725286	342104
EIC	Negative	266.7785	0.61	unknown	71776	54979	83482	73195	66742	56078	64869	34695	66332	106946	51060	44029	52443	79805	57716	62512	49742	58879	88301
fpHILIC	Positive	128.0256	3.23	unknown	612925	548421	389397	520514	388264	497603	382652	320015	471087	342443	332205	437543	478300	415331	445936	495806	432403	480145	409845
EIC	Negative	264.7814	0.62	unknown	196486	143070	228038	195985	190856	154412	156562	95448	191052	274021	137919	119848	148920	217020	141387	154349	141848	165222	263918
fpHILIC	Negative	213.9751	2.08	unknown	432727	515648	481951	371448	335801	468185	423224	350377	294598	502418	438074	441050	344010	496125	549805	250397	365547	396196	496461
fpHILIC	Positive	126.0219	3.23	unknown	100083104	90105536	67376360	74551384	61123088	87493848	55477372	50969656	73158920	56848900	53587752	67693960	67789088	62488540	65454016	87594952	68093032	76467080	63322452
EIC	Negative	633.4077	5.67	unknown	308539	23520	326958	165616	110316	34870	120861	0	105293	166665	41852	0	0	22175	0	0	29872	253559	124918
EIC	Negative	308.1928	3.87	unknown	244863	136042	350666	365288	132938	146712	209376	316738	162986	495465	150375	173229	169014	175596	169573	137512	165282	473338	119691
EIC	Negative	249.1498	2.46	unknown	95744	77792	118999	70691	69521	75331	72533	99840	66312	107615	61770	55353	60889	67381	73021	77969	63290	129919	77719
EIC	Negative	555.3687	2.91	unknown	3722007	1387242	2832430	6203472	710330	1602968	1293747	769888	406562	2205910	1406915	3938916	2969838	677426	1460791	1162852	1693299	0	945324
fpHILIC	Positive	482.3601	0.61	unknown	2764963	2137822	2763593	3013656	2669899	2331714	2229774	2321575	2458594	2484556	1959022	2407840	2303857	2506547	2677633	2796523	2400540	2993771	2350527
EIC	Negative	474.2629	3.99	unknown	5144556	5898524	6571662	3559899	4087559	6413136	3789168	4721756	2288359	3053421	5142887	3504547	2870408	9909535	5125979	1663956	4274943	4903816	7483232
EIC	Negative	409.1690	3.94	unknown	1861501	1417416	2759362	1349631	785577	2509845	1539819	1273919	1008380	863506	1637776	2344968	2705357	1880467	713548	1779999	1823535	2313182	1208181
EIC	Negative	333.1384	4.23	unknown	332027	375231	408477	452824	277693	109298	278086	242377	322465	304843	245118	253372	344692	185966	265089	398834	223327	349202	118170
EIC	Negative	301.0942	1.19	unknown	91988	48189	81972	72068	57724	76693	67103	36011	69336	127433	58272	53176	56390	48159	56556	63448	63040	55826	99188
EIC	Negative	396.1871	3.94	unknown	981798	794733	1134994	647772	494420	1109930	825627	688352	591826	497240	812633	1112317	1199048	945887	435977	950022	890792	1130959	679981
EIC	Negative	494.7604	0.52	unknown	164272	224537	306638	264997	175245	201868	250977	128991	234628	292916	252799	211112	237326	48991	216780	261436	181304	162804	270657
EIC	Negative	548.7246	0.52	unknown	597926	475469	705007	624882	448793	437316	492951	287900	542652	666807	325840	428025	500430	0	387383	476960	496282	559110	515869
EIC	Negative	295.1895	5.75	unknown	228946	304405	295677	1030814	267213	199932	262421	282960	429919	321039	270111	307171	534683	124617	145722	547441	417775	359469	119951
EIC	Negative	636.3501	5.04	unknown	2172612	1542918	1937450	1826883	1628292	1367462	1615958	1851564	1763992	2178262	1442414	1742095	1692698	1636792	1824225	1803837	1681342	2199505	1570927
EIC	Negative	365.2693	6.01	unknown	749078	568126	867206	696243	560127	347935	585194	660268	482512	760964	561658	484365	448374	478912	691607	640064	434687	620941	522386
EIC	Negative	273.1246	0.85	unknown	295009	219313	407423	349810	168968	116662	227257	297819	121561	296710	253945	231038	252445	95918	148873	211319	177168	394788	97288
EIC	Negative	247.1307	0.78	unknown	154339	122476	89869	273827	93066	236202	261376	71823	52476	93112	205027	382382	215443	154927	158939	193317	206714	132889	97773
EIC	Negative	331.1585	3.08	unknown	191539	77402	159751	0	0	0	0	192824	54602	0	0	0	0	0	101536	0	0	152930	18817
EIC	Negative	363.1795	2.45	unknown	181628	65077	116328	101126	67756	74009	85999	109836	46750	136019	50596	56746	64013	61699	65159	53475	59731	187031	59130

fpHILIC	Positive	508.3393	0.60	unknown	1131688	1233239	1470111	984678	1283024	1406390	1153126	1107553	825301	1057764	1346359	1261745	1140753	0	1216605	1161521	1567949	1163705	1276408
fpHILIC	Positive	194.0441	2.98	unknown	1551524	1076680	1196618	754981	762322	681816	721683	1135852	697666	963382	554828	590551	656867	986281	1196841	673785	709419	999010	843434
fpHILIC	Positive	417.1870	2.61	unknown	1665075	1958495	1976842	1224989	1563671	1452235	1733148	1306282	1282881	1470281	2068540	2005741	1740996	2002832	2141436	1671614	1235884	1513174	1558908
EIC	Negative	360.2468	3.28	unknown	136473	56335	100515	45091	47484	63714	67979	126868	61683	91716	68474	77444	48375	279764	77221	16812	92738	160344	59992
EIC	Negative	307.1895	3.88	unknown	1533217	659115	2107263	1934334	845055	763040	1022824	1836228	975263	2853840	783237	1052485	875647	871922	767525	773972	953015	2824814	707575
fpHILIC	Positive	191.1025	3.25	unknown	1726001	419744	882705	1356888	613630	611987	396708	562432	661987	1541502	604305	615918	544840	349254	381302	763680	543670	1096522	369886
EIC	Negative	272.7955	0.64	unknown	321443	158153	492607	354451	253885	287948	247482	180711	334449	618557	229990	196601	251269	181762	210061	278443	196167	286252	475484
EIC	Negative	462.1661	0.68	unknown	92610	74830	56528	26844	41091	57174	63053	58211	63403	74437	61482	73358	63341	85964	59650	56404	50015	47687	100023
fpHILIC	Negative	322.9883	2.05	unknown	4476081	2774474	2620344	5978185	2083586	1181813	3348709	4601259	6098998	4554316	3132922	2809122	5792207	7269472	5429274	1251722	6048429	3674417	2807193
EIC	Negative	305.2483	5.87	unknown	128083	123136	144031	141347	99663	93957	103151	115904	84144	143983	108279	126105	113302	104340	119918	105489	119518	113805	92533
EIC	Negative	332.7515	0.64	unknown	71674	41972	85490	64916	45212	55332	45377	36303	62982	111816	47696	38648	46605	25062	41799	47881	35769	61471	85184
EIC	Negative	317.1757	3.22	unknown	138184	120302	151579	174165	108006	103466	123305	120660	148477	145388	119158	118507	148684	96192	97098	234540	122051	130304	86193
EIC	Negative	327.2545	5.67	unknown	844582	628093	1170639	1019514	776782	690481	628732	1046239	580825	823607	650055	837564	718363	518520	484630	1114587	761517	1265656	506001
EIC	Negative	272.7954	0.64	unknown	389492	209216	474230	346502	252894	275039	232210	174828	327184	581550	220614	197659	245455	211493	201127	273263	191465	285158	450188
EIC	Negative	283.2282	5.95	unknown	706965	670522	725691	837027	457786	391875	425232	425214	341486	375927	377204	544988	552630	425859	544527	524911	391274	413194	392708
EIC	Negative	294.8425	0.52	unknown	1341485	732983	1492741	1235306	911070	932232	763916	504472	1089750	1567108	793733	702556	815017	576952	782020	990610	619941	974184	1298251
EIC	Negative	614.4147	2.73	unknown	162688	144060	268732	609212	340449	246376	229963	249723	158916	764928	187839	239105	216287	427508	105417	0	282297	634267	338854
fpHILIC	Negative	233.0777	2.69	unknown	859828	674829	504844	935379	588723	823985	727865	468340	435720	523083	495685	576455	426293	467631	758825	516563	468265	503223	1134617
EIC	Negative	427.1798	2.32	unknown	449812	1013390	323171	0	397418	32044	741740	0	763861	1421213	22735	0	1143129	32879	1061005	74569	10662	73456	929609
EIC	Negative	414.1987	3.34	unknown	133586	631442	188254	0	196028	234389	77974	208452	230067	204076	770166	114153	503427	57195	51060	673533	0	143819	183419
EIC	Negative	254.1762	3.02	unknown	312722	239000	226919	310999	247226	290103	286902	225359	172568	282757	271504	356147	291526	224657	306862	302628	305340	231110	211907
EIC	Negative	359.2441	3.28	unknown	677109	252400	522588	270359	273991	323211	316915	611141	293506	412889	274345	322428	275997	1255544	341717	136801	474752	794136	274145
EIC	Negative	271.1921	4.38	unknown	750801	391755	1159372	546791	352886	297457	388432	754908	196162	669027	393626	449691	324412	563336	397436	274947	327398	689591	522395
EIC	Negative	477.3592	5.96	unknown	0	62689	47932	59920	60857	30182	54460	16866	45329	44878	89345	55743	67236	55701	38128	64927	38557	0	89384
EIC	Negative	378.7017	0.62	unknown	97493	90248	139978	100419	98319	77853	79918	54443	103256	160380	75699	67008	79036	69185	75846	83886	68385	91217	141240
EIC	Negative	243.1602	4.11	unknown	428350	394385	577786	326375	207490	275842	377443	281227	271545	250935	318655	433389	508440	280809	266419	340975	336537	374993	254453
fpHILIC	Negative	182.0319	3.17	unknown	541680	252475	424956	431020	202941	257942	287796	311644	320282	461176	294300	229236	284585	293228	289381	214030	400566	348562	284707
fpHILIC	Positive	201.0652	2.61	unknown	387933	505098	453868	259266	308910	503132	370456	212217	255675	281840	417190	353074	338295	422818	415515	503956	300535	281456	338189
fpHILIC	Negative	237.1497	0.39	unknown	2377974	1618267	2439035	3800571	1830260	1451239	2216771	1579232	2414953	2527282	1579603	1814834	2751832	1023368	1986851	2550877	2332869	2686800	1024615
EIC	Negative	298.1850	2.57	unknown	4658480	74545	2633011	5516733	69646	75624	81647	3388090	55934	3235961	77288	142354	127592	37673	96339	83391	89943	3770257	52557
EIC	Negative	262.7844	0.61	unknown	289605	228364	346131	291966	294736	223743	232422	143804	288576	408125	205664	191584	233991	325543	225268	223717	218405	259640	392157
fpHILIC	Negative	78.9589	4.07	unknown	1268871	615852	450933	1630183	555934	1230729	556854	380320	306249	776830	691325	1162656	662691	361680	368491	1077277	724698	720204	488161
EIC	Negative	385.2382	5.88	unknown	255050	140526	253158	256250	141647	107486	130366	220126	146855	356880	163848	123389	143411	124477	169479	152842	145125	239936	125267
EIC	Negative	286.2108	4.43	unknown	205345	238668	219662	227831	110719	131745	192211	183015	178488	132972	197063	218163	204427	169861	225896	207630	148433	181893	125128
EIC	Negative	561.3006	5.54	unknown	763827	635874	696738	615349	626921	660347	552794	689053	520575	600318	576145	596102	517458	762720	622169	707432	649800	712617	714137
EIC	Negative	564.5813	0.63	unknown	130767	65764	187842	139575	65668	79050	64977	31398	107831	221078	66128	60534	75871	0	58040	64991	17870	97646	151191
fpHILIC	Positive	223.1066	2.95	unknown	1479198	1140170	1165037	994511	1029050	1107191	743533	827831	967316	1142565	879769	695955	792282	1161706	1168447	816072	989502	1381638	867477
fpHILIC	Negative	277.0859	2.96	unknown	3063384	2032265	3144457	1813448	1439783	1959974	1786747	2021396	1979628	2890128	3127527	1613299	2609799	4134942	3732766	388676	2742289	1380360	2021405
EIC	Negative	352.8014	0.52	unknown	830399	350121	831970	661415	445599	503890	440314	288730	566907	1012962	361319	443820	564213	255705	407630	546807	286632	447282	821562
EIC	Negative	366.2728	5.92	unknown	249089	173406	254430	191809	156187	99857	182073	236031	149010	245537	184165	165553	134054	141151	225631	215645	126380	206664	145824
EIC	Negative	290.8484	0.59	unknown	440602	371239	558831	432430	415250	343285	345424	308869	472468	774671	379697	339362	401018	372445	335352	407780	306587	427869	629862
EIC	Negative	245.1580	5.24	unknown	878099	195929	1064522	838870	110228	289423	185485	567106	102778	612687	216799	213231	97042	0	200094	329531	29673	722635	120986
EIC	Negative	227.1652	3.97	unknown	129952	89375	151037	544080	96545	100829	112192	128222	127559	179946	89958	130805	282744	43214	69907	218128	115427	185878	42666
fpHILIC	Positive	270.0726	2.74	unknown	1237890	1178765	1001550	640415	751629	1445100	958051	345148	576294	617498	827276	722346	653966	637202	1211470	1026123	650467	893214	776322
fpHILIC	Negative	185.1183	0.40	unknown	2107239	1430751	2114604	1728744	1124966	1872273	922662	1382605	1600537	1659357	1038891	1525592	1515945	1583903	1752341	1598047	1146702	1679499	1542034
EIC	Negative	644.3571	3.22	unknown	283261	84737	143739	137155	83923	184351	113264	110259	60563	147021	105086	199361	76423	49461	119663	99392	142469	80645	175588
EIC	Negative	292.8455	0.52	unknown	2727474	1616943	3123892	2655679	1940243	1889370	1587615	1130681	2192491	3224585	1604387	1472529	1710354	1174970	1591120	2061569	1441112	2018161	2668034
EIC	Negative	395.1842	3.94	unknown	3964317	3143141	4997580	2678113	1946904	4564775	3344199	2825471	2509524	2133509	3442631	4500560	4906347	3932598	1776470	3859107	3737499	4820965	2823904
EIC	Negative	313.1813	3.94	unknown	55113004	42331097	88084235	43233224	23570039	77847217	46715934	38570341	30425135	27295970	49976162	70946284	82663067	57177510	20760496	54421628	56780225	74540943	36796299
EIC	Negative	265.1810	4.34	unknown	436108	162907	541251	640396	172792	138090	160168	340377	146029	447171	190909	280376	216450	88670	124976	172658	183749	536388	55176
EIC	Negative	335.1491	2.27	unknown	382890	163561	297675	603712	130818	131311	115110	384035	132464	503021	89183	131521	115481	81515	92905	201672	133786	537204	35995
EIC	Negative	236.8835	0.52	unknown	349243	243083	379955	341882	255246	244396	210662	162479	290868	390529	208244	209435	214659	179363	223245	274988	191995	266137	347677
EIC	Negative	290.8484	0.52	unknown	2233897	1235548	2351099	2024186	1500018	1494343	1261863	876168	1750621	2487016	1242674	1156700	1272213	929494	1308716	1665137	1120070	1510163	2113925
EIC	Negative	310.2047	3.08	unknown	98441	125868	76581	74572	95002	81423	98064	108937	81081	78913	110313	92680	114732	95596	114932	94299	96835	97830	117047
EIC	Negative	530.3360	4.99	unknown	1204930	0	1200290	2506750	0	1370226	1395312	1399358	1119209	1983698	1422716	1206868	1544634	0	960871	1222825	1503329	2239476	0
EIC	Negative	424.2476	3.74	unknown	128315	183442	204885	117043	133628	250921	146593	151323	76232	138363	168201	150555	108979	361709	170838	94214	175012	164405	246593
EIC	Negative	254.1481	2.68	unknown	639981	1033121	556162	744754	686912	361806	684756	390595	1190128	536380	596766	584091	868154	831902	879243	1246130	532386	534653	529026
EIC	Negative	330.7541	0.64	unknown	309821	164124	395488	285645	196075	200252	170058	134138	274121	500179	180340	157534	198265	121920	155575	208780	154334	224219	352073
EIC	Negative	284.2315	5.95	unknown	132932	130667	134782	152565	79516	74620	84614	81346	68855	73519	67007	112879	112173	74338	99784	103229	78333	81702	74078
EIC	Negative	305.1750	3.10	unknown	415312	122756	328244	362929	111396	130719	143533	262632	109075	483620	120463	202929	160681	115616	129631	141923	118001	342815	119020
EIC	Negative	279.1245	2.54	unknown	7840713	4995038	4997108	4393189	3947462	3803970	3823940	3325363	2655999	5856004	4427966	4689552	4292849	3931685	4660926	5585021	3515264	3290852	2687152
EIC	Negative	443.2079	4.91	unknown	168872	304798	333100	209339	169233	233211	174234	284075	269692	331346	468908	369453	199578	251331	392810	211430	274975	180825	211030
EIC	Negative	602.4508	5.67	unknown	241674	58187	210987	175533	52890	65751	94836	113670	97547	101141	69197	56736	139132	95872	88224	60807	100846	96211	131478
fpHILIC	Negative	146.0824	2.65	unknown	260604	592351	523406	523299	545850	434543	568103	326264	433187	586165	637108	591791	562850	550460	656279	511514	434363	517632	475632
EIC	Negative	255.1969	3.51	unknown	440810	425287	477383	376048	340891	436045	333110	432150	362032	521897	499610	482483	473999	245421	344132	575745	489370	382589	349660
fpHILIC	Positive	127.0252	3.23	unknown	2283033	1915144	1310872	1693357	1323001	1951375	1201304	1028475	1589400	1216250	1243217	1467595	1503665	1390710	1502352	1926633	1518833	1663767	1370703
EIC	Negative	314.1845	3.94	unknown	12016749	9218429	19249698	9415931	5267386	16952353	10142966	8425079	6699497	5974706	10956022	15481017	17895052	12525807	4622109	11901233	12370719	16474631	8077415
EIC	Negative	397.2050	6.36	unknown	576926	380409	697526	236149	252585	266660	282554	250057	204300	466980	352980	181274	279633	212908	201625	275640	293789	345692	297191
EIC	Negative	412.1745	1.00	unknown	152228	127450	118773	76606	96660	113700	110442	152676	59301	147596	76824	107920	82506	148968	123479	62264	88622	150629	174725
EIC	Negative	516.2853	4.46	unknown	161189	164573	208505	210959	195743	79867	138333	157033	122855	174154	86934	161677	172837	157747	136071	248262	82117	215203	99413
EIC	Negative	281.1763	3.39	unknown	4304745	1170284	4355364	1453959	1160422	2210054	1629549	2351344	355131	3319119	1183590	1727212	1138090	2227542	1607319	701232	1454442	2785928	2126772
fpHILIC	Negative	372.1043	2.03	unknown	3518036	454141	404336	579703	447109	452499	369350	568064	1291862	465985	257217	653648	667973	895957	434949	660020	870714	1137865	418922
fpHILIC	Positive	359.2027	5.22	unknown	1678896	2026857	1667441	1634598	1643695	1910213	1827448	1305360	1638117	1746654	1988520	2050028	1850029	1939411	2103765	2163406	1621602	1620614	1863293
EIC	Negative	274.7927	0.64	unknown	63026	34081	73198	62417	40006	45078	36318	29878	56756	95013	34506	31326	43648	33291	31561	42829	31341	53122	75381
EIC	Negative	366.3460	6.79	unknown	731919	1081489	909293	1328927	767002	1252274	923776	931680	758603	1212171	1414765	2214805	1480909	837884	826846	1032886	783743	1596513	977338
fpHILIC	Negative	180.8421	2.10	unknown	304466	366839	237122	321173	232969	371762	245963	344342	318530	411856	385633	410055	286631	521513	397803	199181	397342	338452	371563
EIC	Negative	247.0952	2.46	unknown	1146135	302494	379779	640993	344521	361698	354410	516865	160613	770738	242723	354426	305331	165878	318775	307014	258390	869904	203600
EIC	Negative	365.2693	6.15	unknown	986845	460407	975773	833556	508696	433784	507492	744400	460596	1147578	505068	449873	410417	458998	721988	592384	450963	741876	483932
EIC	Negative	644.3572	3.56	unknown	217343	51358	100390	93180	61709	127671	82490	77843	44572	90211	67307	153475	49315	26350	86189	70102	101220	73067	114058

EIC	Negative	323.0540	2.39	unknown	586950	220158	470472	645395	190583	201102	216795	493563	183489	641974	190734	202576	195502	199797	194099	208428	197886	643129	207069
EIC	Negative	278.1766	3.13	unknown	764219	369120	617126	532570	390675	514172	455967	520456	198210	598499	525779	593192	426769	439521	580732	331650	486790	516539	430892
EIC	Negative	280.1278	2.54	unknown	1249748	831742	851548	757781	636597	583321	612708	546041	437174	961956	715323	756841	680853	623762	762787	882158	567069	548175	420262
EIC	Negative	552.2846	3.83	unknown	241436	493792	352099	402068	314359	378778	432356	331655	401998	273856	500679	539522	428053	445996	454705	466613	391604	342392	445011
EIC	Negative	584.3053	4.00	unknown	153088	75737	88936	230534	85265	130769	137466	159357	113329	298269	118564	146826	157135	0	73262	110527	169011	325292	29469
EIC	Negative	305.1384	3.83	unknown	454366	600947	541391	764448	489121	412728	628665	396088	642529	594346	549320	663423	772110	356118	653389	772197	522053	647875	307974
EIC	Negative	237.1495	4.23	unknown	13946833	14262919	17299296	18728529	11681281	6242858	11459658	10102679	11731973	12597162	9905694	9940764	14234586	7672797	11401439	15804678	9366003	15388275	5934869
fpHILIC	Positive	176.1276	2.47	unknown	633646	596201	751423	555968	505675	508035	567122	468367	579093	575858	784871	736425	665873	560436	586980	750923	502022	509058	622956
EIC	Negative	314.2419	5.99	unknown	146025	107140	245577	147705	50277	90859	53488	54292	126145	70303	69429	125856	111313	62582	68104	76787	83688	137972	57652
EIC	Negative	593.2872	3.10	unknown	251406	47615	192907	192550	88394	162715	115664	182895	0	158196	79163	117853	105866	50213	133885	98303	68282	195402	125928
EIC	Negative	505.4998	7.44	unknown	61017	29687	79421	106179	60083	39226	46413	81737	46797	46077	25363	89154	54531	40755	63285	67705	46689	96120	36606
fpHILIC	Negative	152.9958	2.94	unknown	590887	446452	540158	480089	429852	617729	458052	396729	374589	659261	739883	658972	519819	387526	528556	545546	535985	523462	444433
EIC	Negative	305.1746	2.36	unknown	1047888	499026	1130777	928195	239958	375582	565668	603104	282381	547748	275947	405117	823973	226708	616968	379988	203865	692273	448650
EIC	Negative	331.1309	1.28	unknown	281261	111241	97835	118736	123404	70370	141615	9411	104429	153235	152984	177040	194493	93458	99918	183693	139562	0	85909
EIC	Negative	435.1702	3.65	unknown	524238	632129	514072	560443	534286	589064	580989	616771	560224	599114	705657	637351	662606	626463	701985	596722	562280	761083	643367
EIC	Negative	278.1765	3.46	unknown	381887	222688	274311	154788	203062	272878	200745	287945	64339	260309	189838	254696	168717	155216	316735	126830	260741	232766	229178
EIC	Negative	319.1532	4.23	unknown	1347694	1370783	1620855	1760063	1116406	620489	973012	978074	1220664	1212640	978633	941157	1320187	764404	1109409	1482514	896407	1488663	586681
EIC	Negative	631.2418	2.47	unknown	2807499	899710	1790133	3080267	1198508	716795	1482404	2012489	1376609	1613080	1434882	1416753	1813921	713766	951376	1786210	1320348	2594122	1044991
fpHILIC	Negative	473.3634	0.37	unknown	1448733	1241979	1278726	1224273	1071144	892253	1355940	1065940	989829	1333701	850914	968548	1113241	975256	1560679	1649922	858077	1145827	882292
EIC	Negative	344.7309	0.52	unknown	275774	27680	192028	89160	162670	90468	77356	22687	93909	323776	32676	31918	40326	818725	83926	65100	0	87107	271195
EIC	Negative	268.8010	0.64	unknown	1906842	1027411	2269122	1691051	1221264	1270155	1087185	860712	1567147	2891131	1061715	968314	1220781	1039847	985988	1315840	921348	1414463	2178691
EIC	Negative	475.2667	3.81	unknown	326529	498610	461811	274725	352169	650004	319144	339005	204094	253374	488785	289919	234545	967075	572688	202497	390846	352301	598078
fpHILIC	Positive	197.0893	3.11	unknown	435727	300027	291978	249408	0	581683	302591	222692	357450	221633	285596	284284	228617	251825	335325	397850	281103	345622	303122
fpHILIC	Positive	203.1020	2.71	unknown	611717	346647	234686	175444	174500	393403	91661	59391	144992	190135	241071	289012	177478	419157	277046	195161	157208	96199	255524
EIC	Negative	350.8041	0.52	unknown	1295684	572472	1291466	958659	719845	773265	587716	505499	885717	1564773	574944	488231	585395	377466	581883	846584	444142	750771	1320825
EIC	Negative	266.1399	2.25	unknown	241620	363368	55871	776304	309357	364198	225246	177110	517629	370451	255665	218682	454405	164161	316790	661837	536945	210010	86219
EIC	Negative	397.1997	3.76	unknown	2696147	1637469	2920251	1845865	1030965	1359104	1985788	1653060	1294708	1336030	1302985	1964517	2244688	1627472	1286764	1895859	1777665	2304421	1063206
EIC	Negative	236.1050	2.90	unknown	194514	130799	78117	88402	129784	86350	91367	105699	107857	83709	140798	95916	143492	89013	143082	90427	138493	86696	134117
EIC	Negative	440.6813	0.64	unknown	149105	77970	201375	136303	75280	97677	73768	61757	126497	235628	75459	71302	90227	34373	62919	100135	61535	108566	161939
fpHILIC	Positive	169.0944	5.22	unknown	3563220	4288105	3288131	2787788	2889760	3853950	3187785	2341128	3346854	2722581	3843034	3846731	3651063	3537702	4374931	3601981	3247555	3048960	3506373
EIC	Negative	534.9526	0.55	unknown	257662	140783	163774	301334	69504	280467	132388	93011	66333	130336	166542	198248	127747	0	155575	195739	106218	155014	118169
EIC	Negative	526.2444	0.69	unknown	78446	133998	85195	65769	53045	75874	86826	107296	42356	47948	105165	114357	104866	50950	78657	64056	66123	128910	78364
fpHILIC	Positive	189.1228	2.90	unknown	5667066	3160201	2826510	2789911	2846456	3517961	2263601	1851733	2445375	3052352	2754679	2765944	2191515	2482409	3738962	3208473	2550104	2914105	2307570
EIC	Negative	279.1581	4.08	unknown	18574	79774	120331	468047	16266	112951	170956	164741	16621	21394	144729	22869	16995	57316	24941	178869	19593	258861	66143
EIC	Negative	448.6673	0.64	unknown	104429	54130	147990	92555	57801	69708	53816	36533	80555	174118	52406	40623	64436	17937	47101	66638	45817	70994	109333
EIC	Negative	637.3536	5.14	unknown	402081	239278	341116	330900	290856	197217	225788	368619	341667	463562	305855	234564	291786	273820	281226	303390	316004	414549	307300
EIC	Negative	329.1762	2.32	unknown	577212	360777	962520	267231	153686	378904	394398	362593	220264	436621	315592	580060	239880	153644	119649	390797	288181	625299	296171
fpHILIC	Positive	129.0658	3.40	unknown	2905380	1864601	3213195	5378559	2054299	1981235	2088558	2033705	2122270	2905241	2050794	1793518	1898133	1220460	2233234	1982620	2156755	2789994	2549987
fpHILIC	Negative	242.9908	4.05	unknown	917165	380737	348063	1125876	386021	608411	411448	332717	93975	709088	468177	433181	340751	330815	234615	428414	357237	710683	357336
EIC	Negative	292.8456	0.59	unknown	554843	470892	721820	536172	499832	406869	438145	354512	603256	971731	463416	392230	461811	461842	404045	494601	382054	516881	826880
EIC	Negative	240.1604	2.66	unknown	5330573	2035338	2663904	7730030	2444782	5788366	3319157	3058972	1366614	4126014	3122649	4967764	4392834	935938	4022454	3900040	4507989	4236707	1549318
fpHILIC	Negative	285.1021	3.62	unknown	501717	313239	400254	442576	260294	250622	251240	347252	258939	373943	324756	329174	305692	175898	369695	334045	257411	428831	170103
fpHILIC	Positive	247.1740	1.65	unknown	567974	301514	397333	342881	199985	418008	218124	175537	248036	266163	385794	382595	236338	271847	484172	301511	166411	181283	302954
fpHILIC	Negative	191.0579	0.86	unknown	749082	546710	533327	636192	379271	523319	445422	481435	424109	587103	652449	724420	475763	763220	918480	0	415556	663348	572331
EIC	Negative	592.3280	2.68	unknown	484947	469579	405890	291631	411206	478437	491849	320510	434671	377469	487214	579245	0	504558	417568	526410	456064	255821	781253
EIC	Negative	266.8039	0.64	unknown	1190986	644634	1415428	1049273	741716	796660	674556	526279	973469	1769042	672914	596024	765136	631921	606424	811862	577114	869768	1370935
EIC	Negative	238.1529	4.23	unknown	2174249	2263536	2714915	2954970	1846604	999032	1768689	1549851	1808855	1972903	1531516	1562503	2198773	1222691	1755018	2503946	1534049	2399578	905453
EIC	Negative	398.2030	3.76	unknown	609673	391174	725983	484668	252735	349429	496004	415202	307668	338691	300844	455371	546426	429755	319352	467048	418827	601802	272138
fpHILIC	Positive	117.0568	2.61	unknown	440286	497111	373414	298339	378938	444685	474764	271269	360854	308900	446579	374914	443053	446441	622659	418584	420082	347136	395837
EIC	Negative	261.1342	0.83	unknown	220709	113112	90707	105723	127650	89761	114050	117264	100705	142494	200835	120728	118981	99942	115882	84005	233216	99041	95377
EIC	Negative	560.2976	5.54	unknown	2611543	2323188	2435259	2128673	2198922	2179602	2099000	2285719	1905474	2160032	2041662	2281305	1784139	2493390	2302369	2490562	2263167	2550119	2579547
fpHILIC	Positive	358.2004	5.22	unknown	11637167	13162529	10826332	10111454	11179119	11818420	11242493	8515635	10023287	10830443	11763142	13245801	12674746	12576627	13852021	13253548	11130109	10206779	11865427
EIC	Negative	370.2026	3.52	unknown	324627	185915	403748	394099	156306	206753	192953	302700	166400	365239	247041	263843	222992	189376	237032	267310	183963	312146	171163
EIC	Negative	324.7629	0.64	unknown	461052	256708	593527	418379	277107	289463	259507	196251	400931	728276	259318	235718	293306	177718	224615	319001	219574	335146	528359
EIC	Negative	594.3426	3.19	unknown	414767	353309	303293	216379	270835	409168	339061	292567	331980	285226	324392	474924	280137	394135	360103	348566	353865	240449	575904
EIC	Negative	291.1602	3.11	unknown	187946	158349	140840	111441	148367	109091	153331	154611	129687	114360	122932	133803	147275	154613	173650	119403	157166	165689	178980
EIC	Negative	328.2576	5.67	unknown	177876	147767	262577	208256	159526	145268	120211	226535	113107	151786	128323	176405	154045	115787	86514	232214	166252	270292	119189
EIC	Negative	477.1876	3.94	unknown	567768	450231	637008	394083	292626	579414	483267	446017	381471	326539	479230	661045	610836	525350	278053	555095	572913	671827	418464
EIC	Negative	317.1757	1.98	unknown	0	404943	156062	0	162357	228512	325056	0	131179	0	158926	154186	353700	92167	247675	233609	133313	0	354232
fpHILIC	Positive	226.1157	3.59	unknown	610504	428626	529461	617719	460712	429243	358477	530917	411372	630179	520633	493493	499193	295657	510134	516178	406961	696065	378104
EIC	Negative	313.2027	2.35	unknown	174667	80928	156615	111875	84945	86695	83056	106728	40139	157221	73739	78999	56307	66267	83491	74997	96220	136602	68349
fpHILIC	Negative	105.0193	2.74	unknown	2119394	1987958	2066780	1989861	1780130	1866111	2349689	1794584	1570260	2156783	2062148	1994070	1916296	2083039	1837759	2723755	2070623	2024498	2063344
EIC	Negative	314.2420	5.04	unknown	1512465	785152	2071543	2099146	957750	854921	1046147	1669835	750729	1858066	857508	1064456	812455	992650	832872	753129	1069245	2627011	764148
fpHILIC	Positive	448.1803	2.61	unknown	777826	621964	404547	497071	388159	601177	564842	376721	593345	387610	453076	721483	704630	510415	590079	730101	462472	613744	495630
fpHILIC	Positive	418.1816	2.62	unknown	463021	455342	475843	330002	358198	356584	442630	348443	362282	358601	517870	359259	400319	465506	530208	412113	345997	409045	443369
EIC	Negative	391.0702	0.55	unknown	51886	87262	119158	84072	51422	64800	52575	95208	82346	56265	76958	77219	93572	0	71982	59342	86089	120481	59072
EIC	Negative	524.2276	0.76	unknown	85682	158866	84077	60789	72696	100210	118103	100674	54635	65282	110395	137266	150792	91752	92851	75186	77790	112805	153321
EIC	Negative	270.7981	0.64	unknown	1233169	661282	1481828	1104335	782724	832871	700886	536631	1018277	1821825	692171	623003	790066	667017	640207	851245	607325	917230	1424806
EIC	Negative	252.1606	2.87	unknown	217908	157983	207512	466609	105717	158885	115059	137230	185039	224630	183389	230724	179985	75207	139481	208073	160285	205760	91725
EIC	Negative	632.2445	2.47	unknown	948865	347132	648661	1151266	456195	256990	559520	755384	535456	645224	502077	531384	677993	274268	341132	685005	511384	1015241	388641
fpHILIC	Negative	308.0666	0.43	unknown	1681440	988601	1806963	946938	995842	925041	1008799	1079482	221187	1267985	898308	864606	877968	631371	1024081	677476	833277	1500624	818668
EIC	Negative	384.7186	0.64	unknown	518370	278361	674300	487331	319220	335412	282208	211189	426341	844080	295374	262942	328352	172778	250103	343703	233783	379734	599213
EIC	Negative	498.6373	0.64	unknown	98399	53243	154203	101781	58809	59193	54055	46212	84361	168705	55157	54137	65172	30496	49991	65875	38110	75612	117777
fpHILIC	Negative	204.0304	0.93	unknown	557467	137606	485320	259851	230549	263267	195577	391353	34141	495280	301866	321719	167941	220749	272767	186410	275006	342721	202792
EIC	Negative	381.1875	6.31	unknown	422879	924843	1529723	1265533	434762	1143800	584397	1069619	1597111	1375517	2125945	1620562	536719	970349	1801329	894804	1286525	779654	785312
EIC	Negative	504.6260	0.64	unknown	250166	135508	400150	264318	141490	168507	127801	91878	217152	485775	132653	116225	152041	25640	105965	164260	102255	190410	294986
EIC	Negative	326.7598	0.64	unknown	890018	481932	1129963	812650	561114	593309	504663	388981	771948	1406883	503894	457989	587912	355028	446704	627567	434979	671561	1059369
fpHILIC	Positive	235.0924	3.94	unknown	701575	567785	426178	417754	399428	670836	351669	356810	366757	480329	532985	481709	431412	434391	604610	483005	482934	493463	402800
EIC	Negative	328.7569	0.64	unknown	716127	394469	913043	662347	448664	468995	406200	309136	615866	1143807	408746	369247	461968	281845	342623	494820	348624	537660	862626

fpHILIC	Negative	134.0176	4.15	unknown	6520198	5588658	5158013	5221971	5572376	5260573	5045346	4800073	3582166	6340485	5125540	4862573	3300504	6392188	7655927	3962708	4935217	6113941	5239745
fpHILIC	Negative	269.0546	2.69	unknown	6787334	5484466	4522731	6741536	4940711	6955863	6218804	3438450	3310141	4090377	2993949	5118482	3205588	3956012	6092520	3601045	4029370	3842719	11037411
EIC	Negative	580.2925	2.63	unknown	4412231	60781	3898989	3847091	78436	106083	96545	4356648	437719	4291971	170167	186212	290822	404620	230904	122799	156016	5175907	153648
EIC	Negative	313.1455	2.22	unknown	532891	227604	587499	373644	167837	345725	375831	413031	452483	407306	196837	609027	355102	331394	131712	348582	323259	728637	487353
EIC	Negative	297.1692	2.52	unknown	393172	157725	369462	0	0	147680	56669	92492	73247	140221	139414	0	100973	38987	102500	61381	39171	94142	128755
EIC	Negative	504.1308	0.71	unknown	178131	117803	187411	61955	111198	129564	108570	43523	123412	215636	125628	91742	106570	218683	99770	104229	74490	49534	266327
EIC	Negative	528.6739	0.52	unknown	431932	206200	664965	420335	237497	310658	227420	107862	443094	587727	138253	198679	112443	0	175085	379358	165694	351963	455123
fpHILIC	Negative	259.0634	0.37	unknown	1402923	3181938	2060936	1076368	1363760	1441049	2590890	2140900	1250205	1294611	3738248	4228248	2185096	4001812	3190978	1913802	1368470	1228341	2415073
EIC	Negative	279.1583	4.44	unknown	2731759	613412	1478442	2185605	710723	690877	890876	1354286	321832	1378955	641141	1344710	1082736	345185	756221	926016	664388	1746496	476249
EIC	Negative	388.7126	0.64	unknown	262365	138908	356918	239412	148015	169747	139885	105974	221206	419890	143466	122619	161216	74370	123488	170723	116616	187221	306061
EIC	Negative	338.2363	2.06	unknown	250406	271373	133331	131405	179512	197962	194623	101559	178417	110490	195673	189477	274377	256185	222257	195449	228059	122048	214068
EIC	Negative	358.2516	5.78	unknown	347255	266985	322772	367133	262181	247356	301839	351402	261542	383416	320603	274357	291271	245876	310833	266750	326401	409278	328506
fpHILIC	Positive	146.1174	2.05	unknown	806584	761129	913705	886091	612391	549948	877911	455640	909256	610898	871115	954680	1050841	679477	551807	1003104	903032	910622	557777
fpHILIC	Negative	163.1127	0.40	unknown	1883328	970633	1319041	2070864	656415	1086336	876793	1368616	495431	1520759	685438	1172390	863512	477203	1217393	1094936	765725	1450485	643310
EIC	Negative	512.2735	2.08	unknown	12513956	2817486	9728611	0	1418204	1649946	1962511	0	1115823	4619764	2444656	223697	1396761	1864994	1736196	3566820	524921	0	1267004
fpHILIC	Negative	369.0614	1.99	unknown	1814556	740266	749300	2112518	616245	503032	976014	1160383	2467917	1437596	1206499	1157188	2296977	1754236	1685300	909291	2002347	1255535	756628
EIC	Negative	259.1915	2.62	unknown	289328	204338	528575	848386	272840	381743	309378	385588	359383	484395	337635	453753	470138	541779	173565	362345	381535	669997	386583
EIC	Negative	352.3297	6.74	unknown	174195	160870	176116	228616	210920	178540	165800	147813	124487	219185	205317	280947	148160	133694	195213	136043	174572	304149	147056
fpHILIC	Negative	137.0356	2.67	unknown	59039376	8551389	6531750	6814504	7992450	18954404	2723071	11781235	20284924	7008732	5325468	17875974	12261237	15055651	5368623	15795938	16812818	21672856	11746658
EIC	Negative	386.7160	0.64	unknown	491235	270733	648315	452557	295450	321684	264540	195079	421890	804912	281952	246092	312891	149481	232332	331811	219588	367993	583231
EIC	Negative	412.7570	0.52	unknown	293994	135796	389423	296411	195950	226370	175658	82833	261555	390986	176118	181286	229749	65031	160560	224755	152489	247939	305276
fpHILIC	Positive	217.1294	3.42	unknown	986468	789169	825908	780295	773744	989213	704434	740634	475297	791835	769891	851129	615752	998871	1066462	887592	591809	924644	794607
EIC	Negative	442.6774	0.64	unknown	359425	195361	525031	371803	215961	242161	198971	156549	317941	629098	209336	187603	239884	105283	173857	250406	164483	284439	438028
EIC	Negative	298.2470	4.92	unknown	740987	525931	1207699	1400523	719544	769564	769560	1111546	1212012	669481	564388	902060	941051	629775	333129	2190694	881621	1331278	574672
fpHILIC	Positive	300.1992	0.98	unknown	2789896	1416914	1941097	2940159	1369741	1589940	1711964	1656357	1354762	2164196	1577374	1726064	2066797	699483	1615396	1772053	1871024	2607482	1040555
EIC	Negative	382.7227	0.64	unknown	234950	121632	291042	202979	129850	141743	123162	91973	185807	356638	123668	106665	139367	69616	101435	155772	100333	152126	270432
EIC	Negative	357.1346	3.17	unknown	381519	168515	113699	15561	131628	102444	142177	86491	87158	72062	161438	6965	20306	46330	82857	496475	7750	10449	41002
EIC	Negative	517.2774	3.11	unknown	179565	116668	0	0	75406	146212	63493	0	96755	0	68685	111508	72759	88122	100676	75555	142672	0	124787
EIC	Negative	310.2107	4.12	unknown	306415	153700	386157	170284	139912	219836	141911	288091	59403	286353	129904	164480	96884	250603	219806	83330	134252	312370	236916
fpHILIC	Positive	253.0318	3.23	unknown	814883	530784	346119	572074	392852	627091	328974	313286	488678	318377	328129	437981	479223	339088	456056	596070	437584	575398	435453
EIC	Negative	419.1128	0.46	unknown	160322	147138	63740	72720	77905	142631	101868	93127	71755	87855	114906	88192	131660	83992	178948	147565	62097	79011	99020
fpHILIC	Positive	204.1337	3.59	unknown	759071	471930	548839	567048	545753	634654	442733	466082	518368	606042	639600	603728	582085	431489	641864	702655	551355	596722	539909
EIC	Negative	526.6770	0.52	unknown	682330	310537	623653	761627	419886	559800	381090	147141	564165	879080	271341	272568	423396	0	337703	600782	230471	507206	734238
EIC	Negative	506.1271	0.71	unknown	116388	76734	127408	46295	70906	85510	80968	33615	79760	152457	87192	61855	73329	155294	69399	64308	54259	35056	185020
EIC	Negative	510.9076	0.55	unknown	307107	67122	171447	246888	95751	220432	99859	0	83068	196301	123493	124990	62221	0	83452	191944	34551	95722	174969
EIC	Negative	444.6737	0.64	unknown	414050	218009	577902	402285	241548	259588	220238	158248	353618	704123	222784	207190	263505	111182	184588	276986	182197	309719	481947
EIC	Negative	309.1709	3.49	unknown	175380	144124	149505	227500	137246	115344	145727	186527	145720	126357	124953	153532	201151	130822	154456	163844	143416	224043	164646
EIC	Negative	446.6702	0.64	unknown	254669	136821	358492	251403	146332	173609	137446	98674	218832	426043	138475	126857	161168	59838	116273	173251	114161	189386	309770
EIC	Negative	524.6803	0.52	unknown	613017	266314	640606	719160	334772	427683	304838	106891	504681	816370	235310	251013	300953	0	243867	430518	143683	472440	665956
fpHILIC	Positive	143.0485	3.24	unknown	3552471	3623280	2868696	2665348	2112811	3150838	2023846	1761860	2778689	2188829	1952131	2473694	2883542	2285428	2569062	3285168	2566570	2917861	2445311
EIC	Negative	297.2436	4.92	unknown	3809228	2567482	5920274	6991476	3594677	3891166	3985702	5442212	5968173	3518987	2726353	4340260	4555462	3221326	1748960	10918691	4417483	6880287	2704240
fpHILIC	Positive	488.1764	3.18	unknown	1035160	1035500	853215	796916	899523	879178	887976	714827	835431	760685	1184742	1142366	1130784	1012024	1129626	994722	940306	802968	1047399
EIC	Negative	551.2511	1.11	unknown	176093	128770	196493	151032	81818	75315	137387	62497	102554	160183	130434	147975	126316	77269	87210	145326	118627	107228	77279
EIC	Negative	392.2226	2.15	unknown	322152	101922	558586	231310	101479	213047	120508	213320	87614	105687	169755	122024	212528	152331	233809	133104	246175	124587	120583
fpHILIC	Negative	93.0455	2.67	unknown	1051229	166921	120556	127623	131755	382107	53387	189647	403343	128535	100996	354759	232777	329451	109706	305405	306748	429628	201744
fpHILIC	Positive	188.1029	3.17	unknown	1629320	1675443	2127972	1712996	1421283	1315152	1908357	1727489	1526642	2221193	1355269	1260079	1494355	1528393	1962601	1507287	1730858	2205594	1806903
EIC	Negative	411.1082	0.69	unknown	174684	113386	106356	83672	83061	131304	86296	75462	75427	127527	107954	101493	90802	123886	117025	158727	74409	77167	99369
EIC	Negative	257.1760	3.11	unknown	341778	215745	315157	203742	189429	198664	230312	202249	198912	205683	207869	311275	379273	250637	195445	246749	241311	273862	232408
EIC	Negative	343.2495	3.84	unknown	130959	90037	110396	205975	99399	96631	99122	132996	111820	130492	104444	117560	107224	84594	88838	134804	99551	232551	74581
fpHILIC	Positive	194.9465	4.07	unknown	3722803	1412632	622015	7436978	1298178	4603651	1217740	430981	419291	1693258	2124266	4903596	1941870	616385	486912	3724075	2124892	1909219	1060431
fpHILIC	Negative	286.0603	3.59	unknown	1108459	961151	671326	1016446	718359	1071895	814556	617584	704395	811338	757220	786043	1000037	518972	877311	1177962	1061666	901108	566945
fpHILIC	Negative	377.1480	2.93	unknown	1277915	999552	894372	483326	481094	708394	503771	457230	343347	674228	575013	555736	455600	883222	732065	401758	497094	677758	603964
fpHILIC	Positive	599.6146	3.77	unknown	1300645	252319	129253	2662862	220466	891430	58054	2194946	1849742	424993	688434	1158222	3184460	1754492	444537	506410	2799317	1783552	557044
fpHILIC	Negative	175.0091	2.67	unknown	1695949	240640	207928	235801	243426	530775	70549	389210	591260	196966	160507	503434	389340	436901	150967	466659	535716	649597	329714
fpHILIC	Positive	242.1328	3.19	unknown	608646	486838	372352	595123	342955	544128	376726	296842	415249	414130	434297	474721	411568	524691	483641	583790	360787	493815	388651
EIC	Negative	314.1485	2.22	unknown	143119	58437	141601	106382	37365	78368	71158	122887	96239	105380	52155	103661	92233	63016	20210	76637	83484	195794	98772
EIC	Negative	226.1446	2.32	unknown	2000705	591368	698539	1359391	574773	1506929	605933	811219	284713	1140700	622793	965743	680677	330867	1182632	681345	989619	975386	597130
fpHILIC	Negative	271.0514	2.69	unknown	2333814	1723865	1562294	2375318	1533846	2353409	1932683	1043811	1093602	1411214	1024869	1684719	1042613	1296652	1881324	1145924	1388773	1259208	3643661
EIC	Negative	320.1567	4.23	unknown	254238	207087	300190	304640	205366	117284	177715	163787	186806	201447	172441	176199	228894	139986	196373	268264	162434	257641	111871
EIC	Negative	326.2777	5.83	unknown	234368	258399	535310	578229	323011	76935	274328	379240	233140	275867	275601	263374	288370	352754	296467	200503	248376	491313	270229
EIC	Negative	289.1812	3.05	unknown	325040	198882	512032	271605	123039	137959	229409	171926	213204	214834	163080	280418	265503	162669	173236	247419	225385	241939	137446
EIC	Negative	581.2972	2.62	unknown	1490955	20779	1243968	1294290	33860	53434	28787	1426015	141341	1325660	57776	51053	88776	127030	71720	42666	51940	1755466	51495
EIC	Negative	546.7264	0.52	unknown	225300	189003	278295	270660	0	208281	165017	143897	344230	252224	212471	152956	167671	0	184179	224162	181367	215903	204402
EIC	Negative	302.2206	6.36	unknown	8649848	6010680	11417677	5639026	5150150	4645852	6215437	5528601	5199625	8045485	5355786	4168826	5427770	4537359	4179516	6086020	6017297	7924932	7153098
fpHILIC	Negative	313.1826	0.40	unknown	1749087	1400017	3977710	1734395	639253	2511715	2305122	1310715	1073795	1112111	1417535	2290436	3119733	1686058	896250	1694760	2383926	2480435	1743592
fpHILIC	Positive	501.2998	0.38	unknown	534924	167676	474319	1160625	259584	375954	374377	636656	419350	950133	431171	413650	500447	87655	185858	310271	525344	1215164	187906
EIC	Negative	285.2076	6.09	unknown	157297	149395	209122	155488	89037	115385	135496	106455	96992	100633	121483	167263	175586	124985	150479	123938	113386	145724	104424
fpHILIC	Positive	235.0918	2.69	unknown	327662	284153	293289	505802	282164	347213	309996	223056	267213	246526	191662	278813	236681	226131	281951	357981	236642	258497	538284
fpHILIC	Positive	501.0645	3.23	unknown	614667	434965	457715	528211	331233	443866	301732	319944	380220	340695	252798	399460	393127	335752	355624	479441	390998	524489	336674
EIC	Negative	318.1182	0.78	unknown	201095	176420	210757	217454	144812	87659	182085	144497	91993	201455	199686	185937	139485	81576	109198	189371	135754	257110	73524
EIC	Negative	330.9502	0.52	unknown	168825	28846	76472	87777	76804	93027	33732	0	95642	244921	29918	31245	68803	56173	28618	66295	22220	39419	195323
EIC	Negative	366.2102	4.74	unknown	551210	124868	413921	538050	104109	130408	118408	386932	99578	493234	118331	134187	103102	107938	114068	86973	107192	560666	118505
EIC	Negative	285.2434	6.22	unknown	305357	353450	321545	332977	147760	145869	207879	206471	217083	154323	167758	311287	248188	177000	284959	253878	204823	199037	146014
fpHILIC	Positive	168.0902	2.32	unknown	684926	628505	429228	252780	544988	265147	496617	399929	634946	406476	708867	667714	566223	947195	795122	516572	415009	398000	632066
EIC	Negative	339.2189	4.25	unknown	276140	242988	212430	188352	252380	191345	217309	267257	168976	238366	235451	199407	246305	231545	273271	204393	265628	270329	278188
EIC	Negative	265.1424	3.72	unknown	1417066	610688	1056257	1416410	629998	737245	771838	740575	321419	1163408	669166	984648	871185	289019	702868	739576	569809	1371252	387187
EIC	Negative	426.9053	0.55	unknown	412264	61244	150971	236153	90560	248426	107390	12391	123698	247941	135273	81584	77309	9516	87408	167710	80514	104146	246769
EIC	Negative	627.3692	3.94	unknown	1703000	1029743	4658272	1023991	469282	3648709	1299098	873082	526889	449994	1466270	2985685	4088806	1916174	292879	1674023	2005637	3241774	825834
EIC	Negative	291.2102	0.63	unknown	63452	36487	60839	48145	52225	47589	44262	28942	52134	103878	44125	34496	38021	85699	45686	45995	36137	51969	85965

EIC	Negative	266.1454	3.72	unknown	215106	86101	163231	200693	97111	112033	121656	112790	49923	172818	87948	154197	143055	42530	108489	113781	96941	198101	65616
fpHILIC	Negative	233.0777	3.94	unknown	545883	351609	289041	285325	253106	409938	230327	217018	253640	306072	301374	306500	308845	291063	362086	344566	321059	285708	298176
fpHILIC	Positive	158.0443	2.71	unknown	817141	557463	765961	837399	420455	613739	333879	697111	313602	669530	551999	718784	597327	589770	550265	643239	412245	692860	512841
EIC	Negative	562.5862	0.64	unknown	239235	121945	379683	268351	123350	142869	119261	98847	199164	413259	121836	112750	140966	0	97875	153013	95094	185323	276568
EIC	Negative	341.2112	5.01	unknown	215517	140741	238690	287645	144468	136268	164297	256162	46637	228234	172657	171611	125179	112756	180512	161300	146892	256115	139395
EIC	Negative	285.2073	4.43	unknown	1013363	1327958	1169340	1268679	787159	719964	967986	978863	860896	963436	944773	1235970	1180349	963297	1201152	1131899	945988	1015720	812382
EIC	Negative	556.3723	2.91	unknown	209058	532546	692861	1681510	349193	881459	454723	0	115311	0	557983	1281410	981658	142113	646703	484282	580546	0	420030
fpHILIC	Positive	257.0730	2.69	unknown	1000010	756972	599799	1067302	743116	858311	770906	513682	476135	696994	433011	788006	543803	614248	726682	586850	719130	554103	1394673
EIC	Negative	315.1563	3.65	unknown	673070	622712	604707	508240	585324	543951	530707	672978	490822	436497	627860	509827	595780	499044	583836	594883	652929	786276	726294
fpHILIC	Negative	135.0184	4.15	unknown	1490830	1219728	1294532	1201338	1301973	1170926	1237529	1135434	866878	1372122	1194915	1165278	815690	1529946	1775850	977257	1199667	1627089	1186436
fpHILIC	Positive	262.1275	3.12	unknown	917749	774980	325342	548429	615024	810961	529537	393309	574885	365558	414543	627050	479170	478835	505435	1119679	547626	458452	833039
EIC	Negative	502.3032	4.00	unknown	6005134	3146342	5076528	11069661	3309122	5417799	6247853	6805017	4960546	9800415	5928620	6339447	7274126	1317771	3948740	5343995	7324424	12772777	2306049
EIC	Negative	549.3048	2.34	unknown	312496	104284	410878	131475	54129	87811	90633	235730	25785	262401	93406	87373	57989	130094	86585	39126	38924	296220	125045
EIC	Negative	448.2571	2.48	unknown	264629	93738	386646	965934	128587	252162	237774	672837	143038	743023	198705	320865	404695	49755	124709	65371	397889	1006078	107074
EIC	Negative	354.7988	0.52	unknown	277708	155303	251123	242146	139648	180619	195808	95433	247624	325108	149268	131491	170547	76555	168358	247438	129736	202631	271123
EIC	Negative	317.1146	0.78	unknown	847285	716161	1203223	1214782	599858	376833	755538	847363	386236	884923	1117346	735278	846048	340237	462684	789532	576555	1368561	322335
fpHILIC	Negative	270.0583	2.70	unknown	602517	462787	385252	632214	419998	656853	500092	300039	312972	423827	273370	513922	298459	379698	507110	316316	366900	349627	1061530
fpHILIC	Positive	207.1123	2.07	unknown	3518824	2362070	3033268	2089468	1980703	2199452	2342326	2437441	2327531	2980481	2237479	2369864	2098931	3069240	3502454	2543018	1951472	3000049	2414010
EIC	Negative	273.1722	1.84	unknown	95352	61198	50286	0	54819	102043	41660	0	31250	0	43833	62520	68518	69922	67549	76112	62098	0	67270
fpHILIC	Positive	302.2146	0.97	unknown	2238108	1038722	1704438	2610299	1032109	1108192	1367973	1376003	1110115	1619928	1316573	1503020	1630165	518339	1076186	1605653	1427839	2290443	724597
EIC	Negative	258.2158	6.09	unknown	127064	80358	135882	110414	26494	81369	78743	80743	34192	64641	51463	88293	93602	59439	82242	92319	73594	83026	27340
EIC	Negative	299.1873	2.31	unknown	259819	108338	267254	208473	93003	159474	136064	231270	80440	344840	134131	141997	156973	98448	125179	126302	130793	326631	123858
EIC	Negative	310.1664	2.40	unknown	286126	173882	223002	211830	159606	136596	151798	256078	150848	237189	183386	155254	187362	138169	189895	140811	188052	315465	182535
EIC	Negative	313.2027	2.66	unknown	738943	337821	758435	364583	259568	382811	198158	344458	105114	675004	248466	474312	216565	266719	229930	161883	257837	547222	339296
EIC	Negative	464.7238	0.52	unknown	348669	138151	457717	338815	232293	243462	99670	90473	359382	462101	173838	73257	205237	0	161077	251189	145570	294521	368530
EIC	Negative	500.6365	0.64	unknown	283938	152710	421212	288912	153143	180872	148186	114573	241378	505240	157541	141324	168921	59436	119548	191168	120567	219340	347526
fpHILIC	Positive	138.0390	2.67	unknown	3755719	617303	448570	438946	522430	1253876	163602	717472	1292214	438884	361265	1168772	759376	1085091	358873	1036316	1093605	1469340	746063
EIC	Negative	470.7155	0.52	unknown	525761	174468	394060	414419	248738	259468	248205	108243	342363	512802	194152	182589	245624	56007	186536	336822	205350	278722	430718
EIC	Negative	558.5952	0.64	unknown	199720	108309	315371	211643	108971	133895	106516	84554	183150	358615	107868	98733	128694	38820	90271	145236	96248	159633	243958
EIC	Negative	464.3102	3.11	unknown	228033	16000	276027	179994	37349	34133	12881	404102	39833	311831	41756	35002	35157	51306	35328	16197	42633	535398	0
EIC	Negative	466.7210	0.52	unknown	798471	408336	785108	871667	525205	555584	346476	232854	487850	1037723	466853	395429	463280	0	383868	422881	354854	578680	820234
EIC	Negative	317.1146	1.05	unknown	141333	84746	151919	170122	81668	57725	89706	111588	53639	114538	93712	86165	100391	49911	59031	98260	73064	192993	47422
fpHILIC	Negative	147.0452	2.06	unknown	1341896	637047	833538	508764	485266	815575	597854	470725	645342	735000	589638	669034	545223	917154	924701	629717	539684	788325	717757
EIC	Negative	464.2058	0.76	unknown	191345	477463	229644	167955	211181	299917	302054	300875	142673	180943	298840	400017	449940	274001	263160	218777	221964	343568	453461
EIC	Negative	385.2016	4.90	unknown	420477	564317	561809	613626	560308	482697	514899	500766	419238	582817	628206	629669	633317	688064	641047	628776	607937	564882	517360
fpHILIC	Positive	157.0969	2.79	unknown	2938967	2374955	2436268	1987523	2068224	2163835	1905761	1950708	1778113	1969419	1897777	2159473	1755620	2292073	2726065	2206750	2139793	2292343	2428260
fpHILIC	Positive	175.0705	2.70	unknown	2949365	2177094	2936973	3086846	1825591	2035325	1401498	2661011	1255235	2515417	2432730	2004079	1904132	2411499	2085550	2649710	1729740	2297363	2217920
EIC	Negative	343.2116	3.64	unknown	1958893	406365	2483634	590642	257585	696408	472120	1020414	150541	1123324	311874	622155	278423	668327	472015	195235	425005	1304507	711726
EIC	Negative	529.3324	4.98	unknown	3051735	1745573	2839537	7360093	1553481	2879664	3167724	3452374	2793951	5868984	3132753	3037821	4066239	505314	2095662	2903461	3674814	7003981	906742
fpHILIC	Positive	480.3079	0.50	unknown	2175710	2584604	2180749	1464627	2030197	2443072	2152899	1667082	1197330	1259102	1966829	2435605	1364753	2918801	2182355	2553368	2205312	1646326	2782819
fpHILIC	Positive	376.0506	3.23	unknown	1545034	797300	622440	915291	575566	987676	488227	463213	890491	556408	457856	736153	755419	589020	643597	1111120	791368	911353	622591
EIC	Negative	501.3010	4.00	unknown	18979268	9331346	16082186	36016684	10085624	17148976	19974188	21512897	15720848	32135195	19318099	20097191	23844962	3206403	11335872	16962295	23980147	42336538	6585401
fpHILIC	Positive	367.1644	3.62	unknown	1824534	1000211	1218379	1184165	711389	800322	634659	944279	626472	1274592	893340	920502	742485	854562	1109690	870492	672408	1235191	567169
EIC	Negative	331.1541	3.27	unknown	276678	200542	411435	138399	124708	190826	107868	196025	68784	307623	174253	172548	103265	216979	221394	80378	129772	211969	181375
EIC	Negative	474.2736	2.97	unknown	538433	200502	599220	1075880	231291	345757	451099	870364	612843	1036400	357619	322790	667387	53030	224394	368816	566801	1722809	127673
EIC	Negative	227.1478	2.33	unknown	294412	91927	122473	219571	81973	250731	98428	108796	50891	195427	120226	152582	99159	59236	192043	105980	158072	160158	91995
EIC	Negative	502.6316	0.64	unknown	352624	193225	538151	359223	196635	226132	179449	135500	305266	655139	194125	169232	215125	73733	158177	237576	157056	273864	424149
fpHILIC	Positive	117.0739	2.79	unknown	48807904	44935664	42474920	31303790	35048736	43493384	31510628	31859312	30281012	35458324	36719388	38313480	31547514	46167368	57224404	34223612	31903996	40545672	42642560
EIC	Negative	294.1792	2.90	unknown	803363	754203	633837	546721	685971	483463	694998	542542	502709	488809	634492	563673	641009	524502	713777	620799	694410	747818	713493
EIC	Negative	362.1892	5.18	unknown	325066	364721	326024	355363	372620	328841	397644	258012	323972	309629	420594	384560	350057	410805	378483	451767	334250	368457	483615
EIC	Negative	488.7677	0.52	unknown	379246	352322	540016	387037	111057	328515	355748	228923	361159	413605	257692	295544	433882	0	257191	322894	324141	392441	365240
EIC	Negative	229.1810	3.64	unknown	245014	184787	363143	287150	94847	181395	204606	132404	157566	176045	171404	379861	417319	171442	238627	237561	137699	172221	138805
fpHILIC	Negative	507.2726	0.38	unknown	5234736	6893685	5297031	4496890	5708206	3072324	5504573	5218864	2746876	3715350	4692074	4922677	4117918	6067162	6017777	2628883	5460377	7290520	5303139
EIC	Negative	317.1145	1.20	unknown	133860	69547	124180	148975	62662	50107	78539	98455	50300	110526	70411	71158	92345	51124	54732	80062	64101	161389	47789
EIC	Negative	447.2537	2.48	unknown	1076987	387350	1477852	3680298	430146	1007301	753180	2790501	544206	2987058	791374	1272394	1576213	188164	417933	216853	1614668	4006056	377580
EIC	Negative	396.1863	2.42	unknown	1415352	164322	1950288	592198	122529	295878	70826	953984	71135	1121735	127797	125660	116879	529133	187523	53664	178949	1064771	333325
EIC	Negative	325.1128	3.11	unknown	72092	95973	86311	40566	22837	50530	69163	56116	52630	78220	64132	65546	70652	67062	72229	91927	43555	37461	56611
EIC	Negative	345.1711	2.63	unknown	835193	768776	1021349	724040	388028	1069163	718542	450571	399696	695007	669417	1112553	1068976	709482	791203	855278	698785	696158	593792
EIC	Negative	330.1795	2.18	unknown	1863291	1667585	2069930	1891168	699938	2192652	1781704	1230079	1669384	1822257	1231474	2607796	2176528	1398005	752375	1969138	1525389	3031520	1821681
EIC	Negative	361.2392	4.95	unknown	1184871	295038	884586	461352	413175	364038	300221	601102	186860	1287142	453216	245495	303257	246292	288936	273415	268855	979757	430763
EIC	Negative	560.2978	5.31	unknown	795714	740346	847600	555846	560862	708549	476341	617927	357790	567214	528406	516734	399245	972871	691892	528022	621180	720804	710235
EIC	Negative	532.3517	5.81	unknown	412369	168652	364532	889697	153443	300534	351279	421603	368132	700078	379706	281138	494025	66598	274330	315207	382095	836302	110743
EIC	Negative	344.2174	3.70	unknown	160699	32253	195045	39839	16852	59458	44354	92211	0	91198	28324	44682	27234	66447	38268	10413	42096	95339	70039
EIC	Negative	533.3521	5.81	unknown	104355	63133	120360	270691	66506	74979	117020	135038	124387	210949	114999	91552	167035	0	84485	95758	128484	290436	24094
EIC	Negative	240.1685	2.53	unknown	369727	160358	363638	584808	165888	195422	201613	307932	190485	413074	223337	336048	317202	167978	175943	264805	195301	504554	136720
EIC	Negative	257.1541	3.92	unknown	263459	244733	211092	199126	240717	207797	209493	238629	174177	181398	216828	196718	226065	173841	247984	232825	271359	265063	271661
fpHILIC	Negative	311.2226	0.48	unknown	2571486	952174	3137756	2776475	1046174	1144111	1195595	2274276	900346	3330370	792692	1200519	987719	1289672	1217832	925223	1258320	3326720	1169342
EIC	Negative	618.5484	0.64	unknown	110082	62592	174900	121243	65046	66185	58517	36393	98128	187463	60470	58810	70763	16562	43661	77538	45821	89479	133921
EIC	Negative	293.1762	2.90	unknown	4541058	4101774	3466921	3089194	3538535	2707362	3598928	3169191	2652280	3104713	3572179	3299544	3434216	2774507	3761077	3542735	3807955	3763842	3997447
fpHILIC	Positive	898.9157	3.78	unknown	957537	262559	88092	1727198	170843	659774	46114	1411315	1403434	269927	608389	774822	2343610	1216434	377081	307252	2050137	1243302	469348
EIC	Negative	282.1798	3.39	unknown	797465	234916	810973	254428	203782	400663	304640	436493	78028	584556	213120	299576	222942	387286	287110	148079	330423	514887	435562
fpHILIC	Negative	473.1625	3.53	unknown	562494	3115421	3138703	541774	560823	598052	1099429	1015421	2151981	470396	306127	655606	719604	3337490	1014602	586260	1316467	883542	2740130
EIC	Negative	562.8986	0.54	unknown	597502	900405	1007809	1157658	670150	271349	890450	377583	930701	578144	903022	1001480	423087	84982	642084	960017	919798	881372	382493
EIC	Negative	377.2703	6.23	unknown	802042	327267	798845	726744	352574	219660	377522	640428	377054	705304	453565	414099	344942	360878	456971	495881	349228	689948	380600
fpHILIC	Positive	252.0772	3.34	unknown	578520	662119	801920	771641	743965	633127	731415	694759	790363	887445	691914	797204	661597	1122058	878424	643324	797031	940643	1075444
fpHILIC	Positive	167.0893	2.06	unknown	48647976	27682000	38083700	24598982	22031620	27318106	23567762	23236158	26318114	31673636	26770352	28574198	23045112	37025828	39226756	27842946	20996094	33119980	26643598
EIC	Negative	306.1414	3.83	unknown	71310	105496	82390	118605	68064	68524	114047	70075	89536	90772	95121	108642	116941	54573	100859	130795	87596	117155	58939
EIC	Negative	250.1530	3.27	unknown	835584	490813	1492277	391213	298505	557920	286246	633885	181193	943292	532385	569258	304498	714820	561881	234961	390342	678251	498975
fpHILIC	Positive	228.1165	3.62	unknown	819813	331620	380603	553464	251480	474490	360512	212430	229865	468303	457244	537271	434967	309809	284792	448891	343779	298407	470542

fpHILIC	Positive	251.0363	3.23	unknown	9917971	6401878	4575019	6401041	4488959	7218657	3742967	3308875	5430356	3898008	3600665	5538111	5438614	4632097	4890155	7565218	5161616	6898893	4700111
fpHILIC	Positive	168.0914	2.06	unknown	2423598	1434803	1929147	1272830	1215780	1545093	1253155	1208111	1393257	1663274	1545206	1518409	1232548	1875078	1996868	1495563	1243268	1842209	1412380
fpHILIC	Positive	188.0699	2.61	unknown	53747232	68844856	55036104	35632484	47491884	65621600	61678004	37736504	41584944	45783840	62640648	59080784	52875616	72353456	85041424	61047680	50423716	52206280	58231456
EIC	Negative	385.2606	3.72	unknown	231224	103504	164297	98412	91074	103798	111686	194527	89724	129076	124279	88290	87689	502433	152986	36341	183413	230419	110701
EIC	Negative	405.2532	5.21	unknown	235474	192444	247315	187317	158949	171309	191281	218687	144176	202601	214010	198236	203714	184970	234748	224636	197180	214476	197578
EIC	Negative	493.3544	3.74	unknown	177297	232009	144180	127810	161242	231200	153548	152795	125514	152609	320572	167077	197209	216644	242069	215847	204209	128893	204988
EIC	Negative	373.2374	6.09	unknown	367806	405578	327847	277433	377102	324504	319357	343897	267417	277599	352342	274966	353774	289453	377850	367210	398095	406761	423252
fpHILIC	Negative	287.0521	2.94	unknown	1820334	905848	866540	1168827	792903	1489359	743203	747893	1013147	967785	725456	1231716	1079896	1191455	1186861	940312	1360876	1181680	1118406
EIC	Negative	323.2587	4.92	unknown	149730	74686	225375	267319	84462	113969	78177	182670	175634	181704	70940	150428	109089	60723	86455	235598	149970	176804	119080
EIC	Negative	556.2656	3.99	unknown	272371	340598	312595	167287	219644	347627	182892	238144	74063	168197	269471	169956	155516	469593	278048	97470	241376	278195	389890
fpHILIC	Positive	118.0651	2.06	unknown	1710203	911938	1439428	875743	746705	1021297	845392	796535	872679	1086579	952397	1067100	768843	1280365	1395840	943647	787742	1172256	880678
EIC	Negative	343.2123	3.53	unknown	390946	346829	317799	252362	165119	358025	285001	259504	343168	211417	270350	374979	341003	378341	359605	312577	228038	386589	420226
EIC	Negative	329.1762	2.18	unknown	8490063	7498679	9579260	8632289	3210235	9817426	8140541	5603969	7598570	8128563	5506637	11906276	9777581	6245439	3462465	9037196	6944857	13866629	8282892
EIC	Negative	383.2442	3.27	unknown	92775	46025	94022	43171	33904	51419	34014	71045	29680	63709	42444	33571	36815	126981	48152	34427	49567	98901	35323
EIC	Negative	498.2527	0.99	unknown	187585	147585	167294	0	120945	0	0	191576	107594	156312	128453	0	119313	103718	158251	0	120267	103646	126584
fpHILIC	Positive	130.0647	2.61	unknown	844168	1041102	987926	577431	720532	967717	830213	637132	702796	753908	908407	872223	816108	1077316	1194279	919255	769424	753536	1038323
fpHILIC	Negative	190.0541	0.86	unknown	11391603	7726395	7186241	7435453	4946636	7914984	5814306	6495255	4847815	8129984	9403721	8864499	6573986	10310047	12408937	0	6238936	8908524	8818914
EIC	Negative	475.2851	3.40	unknown	2234329	957869	2124579	5886702	1290885	2200780	2325319	3687797	1973061	5735036	2234257	2341515	3226011	336012	1036336	1493510	3173224	7464278	742350
EIC	Negative	503.3020	4.00	unknown	1941306	1188164	1546717	3391041	1060402	1654700	1942769	2085207	1508137	3040947	1870145	1976784	2265006	485770	1191629	1664067	2370051	4035322	748040
EIC	Negative	476.2887	3.40	unknown	567806	262917	562336	1698610	385394	602987	632229	977817	547642	1555391	614000	647841	974613	96184	306245	440923	852975	2018379	198452
EIC	Negative	445.2960	3.15	unknown	735365	145129	740152	810057	139214	202573	152043	509732	172289	792980	139046	179679	167399	132652	146277	151117	159347	836362	133954
EIC	Negative	548.3014	2.34	unknown	988250	266371	1556318	487455	184516	245681	225400	755534	84817	845267	296587	262892	147391	455865	295373	143960	175479	790120	410611
EIC	Negative	390.0345	0.48	unknown	158395	160836	127203	213252	177564	150788	133535	168182	138054	206032	178993	180352	205883	66855	184756	225601	173064	189889	158920
EIC	Negative	428.1827	2.67	unknown	203010	182966	237366	169416	87358	118502	194299	116553	142729	136217	139746	229564	206825	167479	155273	171679	164553	177689	131187
fpHILIC	Positive	503.3145	0.37	unknown	953042	441236	912515	2943862	594169	854423	1132963	1275550	1025920	2231121	1064857	1008864	1489950	130544	461308	831210	1280217	2984203	375284
EIC	Negative	618.3421	3.52	unknown	1890765	1303780	1236456	1124732	1039922	1867475	1297177	1268602	1188188	1379807	1227231	1998010	1329784	778382	1347821	1726234	1983374	852560	2185086
EIC	Negative	582.3779	5.40	unknown	11009165	11319746	11281464	10569416	11388373	12786521	0	11695239	10868501	9974565	11743905	11938786	11650252	11132011	11495170	10628412	11802729	10672930	12201275
EIC	Negative	299.2235	5.09	unknown	1223040	1244859	1178314	1418097	812817	754180	852089	977895	823597	824202	938481	1212749	1082105	1105545	1244120	1077962	1011397	953880	842468
EIC	Negative	621.4015	5.65	unknown	0	0	393952	0	23353	36144	105537	32188	0	0	0	0	0	23377	0	239739	0	0	0
fpHILIC	Negative	263.0422	2.61	unknown	405082	515092	508861	304627	343100	282838	456461	195921	186291	307476	528950	281232	416028	328774	623462	277987	285059	271247	322964
EIC	Negative	314.2056	2.66	unknown	80068	74007	91264	0	55590	102511	39061	63944	35951	91339	48533	92288	49809	58884	69466	37771	60036	108379	85668
EIC	Negative	349.2389	3.57	unknown	84034	47794	79518	97324	18900	25356	17322	20644	18975	58332	24354	29783	19345	25913	15675	24069	25561	39521	18321
fpHILIC	Positive	189.1343	4.87	unknown	893557	655075	372894	502903	410424	457623	357224	345428	813191	587303	456936	508517	413444	605114	933607	709506	434853	318792	507861
EIC	Negative	412.3436	6.03	unknown	1286790	543081	561981	981757	608815	430537	995773	496296	631723	456956	587227	412451	490310	453132	593711	775407	453563	443203	1590186
EIC	Negative	333.2075	4.12	unknown	475737	124048	621934	346432	205110	207138	198967	353237	112457	563685	219991	221238	197670	243003	207595	181954	234403	444627	244297
EIC	Negative	608.6791	0.52	unknown	597556	438567	807916	882699	363981	436002	528660	228596	558563	680054	393587	493669	516934	49481	418831	497431	404583	673241	585570
EIC	Negative	616.5513	0.64	unknown	69744	36188	113872	81648	40449	47396	36047	22855	66434	139156	39767	38621	46446	0	30359	51535	33066	60362	84634
EIC	Negative	319.2278	5.99	unknown	242401	163957	536799	222382	128585	144160	150159	249769	70607	281857	194362	202355	140767	226095	124545	116180	169999	293221	202821
EIC	Negative	611.4305	4.14	unknown	179992	140215	399196	461666	119819	88207	151050	196671	176215	185678	110849	170948	150193	193614	137067	140643	188813	256245	170331
fpHILIC	Positive	190.0862	2.06	unknown	1410368	738153	1219365	781055	748487	733193	714273	660563	653814	1086590	848511	775213	683530	1110752	1088764	764092	627183	1014198	789656
EIC	Negative	390.2942	6.39	unknown	23490398	8814691	16698076	11612293	6997074	12304046	0	7805044	0	13674160	10925420	10025642	0	15514145	12851578	4906484	9470733	0	5873667
EIC	Negative	289.1448	1.81	unknown	395502	239851	473349	0	82498	189305	237336	99226	176654	139540	218216	324816	292208	200525	222642	288962	203189	0	244637
EIC	Negative	425.2195	2.22	unknown	305314	96015	217565	280044	85409	148984	109300	266897	26724	248451	64538	127133	81002	78114	121843	69804	148750	295615	143024
fpHILIC	Negative	292.9235	4.07	unknown	1372972	447480	163502	3088254	424568	1940933	377705	92802	77266	684395	830801	2219060	695366	157474	115090	1629616	767127	736216	315795
EIC	Negative	401.1570	4.23	unknown	233649	165948	237302	287371	156086	72727	146251	174214	173449	221120	129541	131210	212793	103736	184256	226363	123232	249285	101072
fpHILIC	Negative	215.0671	2.63	unknown	1606093	277019	181486	281322	332401	790836	116506	259509	586712	176527	236377	602705	443213	557636	227629	650235	505348	747478	421629
EIC	Negative	280.1888	3.65	unknown	1092191	1183023	938855	1087079	1091070	921353	907777	1060071	805815	992156	1111710	1025115	1204532	936808	1160590	1031320	1058526	1326242	1280158
fpHILIC	Positive	70.0657	2.79	unknown	13522982	12863025	11985989	8782025	9822693	12188199	8762266	9130400	8443968	9708024	10562862	10977069	8939799	12768138	15892755	9860055	9638961	11407807	12373809
fpHILIC	Negative	132.0302	3.77	unknown	9908896	4859592	3984853	4539940	3927334	8371346	3455937	3427331	3337647	3651960	2982059	5173532	3849869	3795397	3950262	5953286	5717046	6172571	5784894
fpHILIC	Positive	121.0396	1.00	unknown	1020382	422744	362982	302388	478255	566743	181562	508718	792293	698302	500921	551305	548752	664177	358880	596804	870545	937457	334752
fpHILIC	Positive	202.0498	0.96	unknown	296919	794786	973280	435077	201655	376763	196781	284410	195720	594197	498481	263040	372537	394854	355730	357282	249365	418886	157663
EIC	Negative	346.1743	3.10	unknown	829300	720750	1541535	1004675	387872	345823	500641	517571	296849	560890	400240	617914	417485	345381	532503	613869	382647	640431	343471
EIC	Negative	591.3554	3.46	unknown	262916	206044	318881	375065	202359	99951	184546	302482	215448	309734	239893	93441	234894	247994	226299	191113	80433	493316	241739
EIC	Negative	271.1919	4.73	unknown	151688	79159	247716	120966	78943	51595	139049	125457	45040	127407	52691	116228	116292	100395	107900	74444	83859	196001	77870
EIC	Negative	557.3630	6.10	unknown	2246394	1280909	1981899	3791162	984522	2012146	2096748	1899874	1795313	3097004	2081491	1906596	2936254	449649	1729750	1978000	2096692	4205664	779703
fpHILIC	Positive	118.0320	2.47	unknown	1507810	1548800	1420417	957588	930525	1216554	795076	952254	887433	1032263	1096106	1257976	1059683	1765944	1392764	1013768	1021423	1041823	1402112
fpHILIC	Negative	305.0486	0.38	unknown	3841317	2263670	7016657	3661839	1896704	3220401	3065390	3019614	1534037	3840719	2408154	2866120	2385695	2319915	3355935	2387107	2544929	4500829	2851431
EIC	Negative	272.1952	3.80	unknown	127656	109695	137014	157747	77669	78083	77651	109578	66946	131920	80283	108122	107219	105099	110546	111215	83464	122520	75711
EIC	Negative	267.1143	2.20	unknown	357473	102356	0	244494	131697	116278	132035	277613	191417	183766	80911	145040	111515	185752	153091	156387	193912	312071	228796
fpHILIC	Positive	102.0665	1.93	unknown	3097446	2706156	2918685	2985802	2861433	3626539	0	2591604	2441683	2738225	3163101	2898391	2919110	3172654	2972026	2869584	2710964	2430148	3188431
EIC	Negative	239.1652	2.53	unknown	2221448	992026	2174442	3511625	1085545	1238486	1248699	1915578	1184083	2840957	1451368	2195096	2017090	1161340	1169815	1680291	1222702	3293657	849082
EIC	Negative	249.1496	3.27	unknown	4991244	2938478	8823883	2344692	1833679	3228907	1661336	3723318	1116753	5541555	3184110	3380241	1813481	4247938	3442279	1361181	2300975	4105398	3029066
EIC	Negative	560.5911	0.64	unknown	275141	149922	441875	310331	150804	188909	138693	110201	252887	513381	154406	135897	187032	41862	126918	198070	126036	236504	348700
EIC	Negative	343.2118	3.70	unknown	780513	214011	1009306	244146	134079	281137	193470	460194	67560	530629	133723	251540	164253	331374	210948	90373	213261	511967	372993
fpHILIC	Positive	73.0845	2.71	unknown	703751	917888	727197	544974	762639	848761	628280	586300	545437	657106	734507	730903	718586	975033	1253243	735135	671578	537261	955223
fpHILIC	Positive	120.0809	2.06	unknown	97556712	53430084	76650088	50566652	45308316	54557508	47238264	47217476	51509512	61788480	53437580	57558696	46523380	77275016	83311624	56117008	41734096	68267184	53546756
fpHILIC	Positive	438.2001	3.63	unknown	770657	238448	404291	496080	244069	335895	232550	215374	164777	493095	338471	396802	388047	225658	243853	425167	235249	276866	289592
EIC	Negative	337.1693	3.22	unknown	0	76947	230737	108037	111007	56764	137374	69703	83284	0	88706	190459	239695	89080	134843	68161	97293	94121	170736
EIC	Negative	493.3543	3.80	unknown	215021	355066	224909	221771	307376	236313	287196	212105	285880	207936	297361	267838	263064	288568	415394	413250	253780	231486	325894
EIC	Negative	412.1853	3.12	unknown	90412	103097	108900	89084	45046	87793	65857	15432	36314	84382	98073	142503	39643	93763	28423	82717	89374	55815	52730
EIC	Negative	644.3524	3.06	unknown	297301	184160	164981	87433	139622	257558	169949	148079	203808	187021	190201	257318	168530	97571	203635	226758	265914	113846	324045
EIC	Negative	573.2659	3.47	unknown	127409	60451	224084	40996	37557	37606	71473	111961	35749	118284	62815	49782	34939	53234	50536	40097	33857	173801	65069
EIC	Negative	358.2340	3.84	unknown	433766	93162	580034	174242	66404	133200	84349	272118	0	275652	65350	124888	53176	96201	88860	30466	80466	351861	134804
EIC	Negative	345.1711	3.10	unknown	3587258	3163832	6130632	4564039	1689623	1629957	2189294	2411173	1148404	2519854	1814312	3042742	1929900	1560352	2296846	2731748	1754935	2901032	1511688
EIC	Negative	606.1190	2.33	unknown	126567	171021	160303	371473	163056	131719	176210	166541	109615	235521	162676	172887	183977	89599	173917	183876	136853	266007	161826
fpHILIC	Negative	474.1645	3.52	unknown	111530	614164	615661	129554	121961	114202	228882	217330	418012	117546	62518	157742	176808	610912	210090	141483	285722	187144	535612
fpHILIC	Positive	121.0842	2.06	unknown	8136748	4436830	6427900	4628484	3791030	4578884	3852417	3999110	4499603	4726391	4690128	4902675	3972200	6500207	7101129	4742106	3625513	5516652	4641748
fpHILIC	Negative	284.0985	3.62	unknown	4680171	2706238	3672943	3719051	2373133	2129576	2224885	2763705	2123008	3439165	2940391	2856204	3037528	1789566	3336043	3045117	2498911	3889373	1601609
EIC	Negative	368.2808	6.27	unknown	276617	208477	357556	511156	160244	182331	240799	242361	248105	332373	198899	307079	302669	185812	187795	295559	248216	412875	185393

EIC	Negative	411.1846	3.76	unknown	1229231	714690	1498377	840668	438141	606560	835148	662881	599479	594079	582176	877973	1009429	703116	538735	922051	789033	1157909	484065
EIC	Negative	366.2492	6.43	unknown	445787	0	0	3938703	3533099	0	0	0	2831033	0	0	2947288	3072767	2928843	2261603	2728921	0	2892896	0
EIC	Negative	348.8067	0.52	unknown	736386	343875	809424	582840	438675	494401	345627	293664	539613	986763	332559	382311	431406	183858	388039	551398	421550	414890	840359
fpHILIC	Positive	74.0607	3.18	unknown	4881388	6608911	4335106	3545065	4247158	6258499	4904559	3293108	4295677	3717385	6005735	6036134	6295798	6340630	7476275	5760403	5000292	4272037	5984036
EIC	Negative	412.1859	3.76	unknown	346337	178556	406320	216474	131050	167854	235813	194871	143953	167191	150532	242191	273833	205414	146600	257557	217810	331154	129494
EIC	Negative	257.2124	6.09	unknown	766069	466230	750843	684475	256928	332257	378187	447806	251949	379017	310722	473257	553588	353673	505618	401698	404332	483837	270723
EIC	Negative	366.2502	6.39	unknown	3261268	1440407	3986759	3111232	1042797	2115541	0	2538655	0	2681061	1933144	1787969	718847	2252777	2015104	1190056	1867400	977997	1019893
EIC	Negative	390.2944	6.44	unknown	2987253	685338	7556979	10608422	398202	370983	8726439	13732025	497201	344110	6926441	0	0	10125748	9267149	0	7824517	0	12353582
fpHILIC	Negative	261.0416	2.61	unknown	1305480	1636488	1239462	855076	1136277	1197466	1429393	912613	929625	1101360	1387404	1431531	1220709	1617496	1834153	1151600	1184452	1324368	1463215
EIC	Negative	271.1919	3.81	unknown	776051	650187	869149	986542	447720	462076	485336	684656	398092	801996	506424	643676	639206	569342	707673	618550	493895	791173	466283
EIC	Negative	464.2785	4.67	unknown	107628	179357	167764	110108	135968	195330	158230	136512	41770	109167	142289	176737	119548	252639	216413	125562	190506	108108	188954
fpHILIC	Negative	165.0751	2.06	unknown	8470731	4667219	5913404	3694527	3405368	5037692	3786232	3375125	4361287	4966282	4046909	4584747	3944481	5682982	6605959	4247598	3663485	5458199	4827150
fpHILIC	Positive	352.1695	2.70	unknown	1128244	1107992	1080662	1218451	1223265	1554136	1189598	513757	534354	882671	680206	1083117	691964	1066104	1562526	639585	739749	786960	2506049
EIC	Negative	378.1743	3.31	unknown	819273	190969	1559600	613997	64941	262390	114274	785151	37071	853624	206595	270083	218577	505922	139389	65791	302263	530123	533890
EIC	Negative	457.3663	4.94	unknown	364743	89518	145452	135316	249283	97878	160919	100639	131277	112203	126687	82941	121472	117379	82925	113232	436997	193070	93907
EIC	Negative	344.9032	0.56	unknown	618876	75777	231532	255197	101066	292747	102579	25677	117152	356126	106084	90610	100277	96552	112528	178795	59066	113377	331160
fpHILIC	Negative	447.3481	0.37	unknown	1092529	716867	772877	783196	704914	660503	929176	691501	564215	721871	938333	673437	682205	733246	1084547	1237042	618852	713120	761774
EIC	Negative	291.1603	1.64	unknown	932715	485677	906104	199191	190676	506676	542680	286408	400050	616513	428454	731437	618817	383062	259796	529432	379304	711933	515462
EIC	Negative	504.3064	4.00	unknown	357755	186046	100328	643036	200907	321062	81127	457273	317930	358155	392138	406157	460931	11882	0	353336	447073	811490	102798
EIC	Negative	300.2261	5.09	unknown	245434	268202	197356	258959	167163	140579	156193	188355	169245	159132	177305	200641	249784	196027	239809	237060	163899	191662	189672
fpHILIC	Negative	240.1600	0.40	unknown	1219301	587181	243150	1374052	418053	1652721	439272	492357	113041	719944	602356	915297	793364	0	665390	860161	1175654	901259	274556
EIC	Negative	285.2075	5.00	unknown	401640	363618	355124	258290	378364	279038	298660	389067	267651	339144	307499	299950	331948	248624	371349	367151	381408	478187	408984
fpHILIC	Positive	219.0971	3.60	unknown	2883774	3130248	1728629	1730198	2293610	3277131	2106867	1319767	2188662	2402636	2711357	2256554	2695405	2662600	4091315	2126245	2495724	1573817	3640690
fpHILIC	Negative	251.0176	3.23	unknown	957695	590648	373640	547792	355653	634403	341527	323048	438426	412367	289663	542008	503545	371612	517339	623516	456997	580993	427415
fpHILIC	Positive	208.1162	2.07	unknown	569984	313842	262998	305852	301644	283774	246230	316088	264058	351947	300057	288689	244532	346767	388025	311303	205711	506846	347535
EIC	Negative	518.2965	2.91	unknown	348908	193643	399878	1070146	236353	231917	330259	487596	342907	959723	364759	388684	458435	0	338293	239718	421422	982233	49421
EIC	Negative	368.1480	0.71	unknown	266628	236579	315039	140439	154071	203301	170736	217827	115768	226527	207160	168192	129615	262358	261389	132031	174391	237446	269408
EIC	Negative	635.3810	6.16	unknown	689837	408727	742824	567134	484492	285237	557779	578776	387403	634814	528999	538018	412042	610981	612738	678680	428476	687195	456233
fpHILIC	Positive	189.0730	2.61	unknown	6575481	7946746	6285214	4140833	5193336	7081296	7346361	4447943	4987314	5172600	6981593	6941935	6332786	8424554	9662155	7272630	5866294	6635428	6846285
fpHILIC	Positive	103.0544	2.06	unknown	4075781	2123376	3142789	1948592	1706153	2182311	1966044	1771297	2199047	2525656	2084307	2306576	1823962	3020556	3413596	2308323	1764220	2683601	2098880
EIC	Negative	233.1545	3.87	unknown	838169	1920389	1127489	989823	1712542	859455	1839309	915267	1541950	867675	1466999	1340859	1974428	1625780	1412611	1872696	1419702	1603354	2269681
fpHILIC	Positive	131.0491	2.06	unknown	1530703	819158	1396489	811861	680860	773817	672173	750730	753408	1016064	840435	871500	661777	1286324	1161989	802680	626614	1117491	827656
EIC	Negative	582.3779	5.17	unknown	5169292	6651469	5602451	4926176	6086826	7203235	0	5726749	5159622	4597187	6516755	6808780	6200480	6459735	6601338	5918091	6429750	4966005	6303279
fpHILIC	Positive	157.0604	2.58	unknown	2108202	2253376	1851823	2136919	1382911	3766544	1671809	1406901	1040623	2097477	2694796	2821563	2459981	2741512	2358989	1930644	2346142	1743041	3209434
fpHILIC	Positive	206.1001	2.61	unknown	19316124	25797492	20392010	12618395	17708398	21732432	21386632	13348977	14852347	16655352	22539092	21385334	18404618	26058946	29907802	21925998	18502970	20065206	20953612
EIC	Negative	340.2568	5.50	unknown	231517	129458	338482	289332	144287	133365	150953	217432	130981	223032	148498	177993	182865	162952	135701	143882	189156	372952	130379
fpHILIC	Positive	146.0596	2.61	unknown	1932966	2387415	1853998	1083467	1557373	2349464	2021740	1146909	1322650	1446645	2253061	1800000	1676548	2378795	2755492	2236996	1820841	1664827	2030817
EIC	Negative	408.7626	0.52	unknown	871325	490483	867340	622049	429023	483677	525775	284639	598681	1125768	436546	490647	646234	151063	343434	537676	427884	635098	963642
EIC	Negative	255.1067	0.46	unknown	201678	191989	61508	79051	115074	139098	98597	94059	80376	84744	132247	111539	121758	128729	222745	167931	71005	98316	100379
fpHILIC	Positive	161.0916	3.18	unknown	776395	1019787	702180	679355	626590	865816	820232	478194	738644	613986	1039113	1018033	1152551	1061744	962290	936503	913798	643188	966127
EIC	Negative	584.6361	0.52	unknown	656221	337182	1041375	761712	395358	443665	344977	0	666242	958158	289307	252111	418898	0	275660	578305	171904	508436	783807
fpHILIC	Positive	252.1071	3.17	unknown	1793534	1929164	1377179	1219787	1067647	1226119	1519944	751863	921195	1553123	1387027	1041588	1585237	870760	1781016	1253943	1331530	1319373	1191991
fpHILIC	Positive	86.0969	2.40	unknown	16473507	15210351	13797540	9331021	12432841	19638938	14757697	7148772	13114822	13102543	15589304	15656770	14624502	18255806	29061708	17279738	11347014	10611682	15817623
fpHILIC	Positive	72.0814	2.71	unknown	16138675	18541480	17309728	12862034	14191101	19367876	15962148	11975274	12764370	14820449	19845528	19889494	17729670	24586230	28374696	16738744	14354303	13856609	19843862
EIC	Negative	511.2706	2.08	unknown	19714873	8248542	15302173	3409371	3235455	4079895	5604198	2642314	3022586	8868461	6019001	551551	3021245	5410636	5148176	10688014	1413392	2379039	3693789
fpHILIC	Negative	166.0770	2.06	unknown	551917	254335	300419	199057	183210	260133	222829	183275	230413	262840	211797	241314	172341	306899	336919	217870	222851	316351	261302
fpHILIC	Negative	353.0898	3.20	unknown	528760	492098	411408	718157	421839	687867	563515	362761	528983	447334	540036	507708	552218	982132	624570	811390	534639	616127	632727
fpHILIC	Positive	377.1735	2.06	unknown	1490349	728164	0	807788	605338	603816	574979	706058	736427	826749	771157	803049	754756	750434	1063912	822281	451574	909019	803670
EIC	Negative	412.3436	5.84	unknown	273317	156134	141761	275455	167250	126362	248149	111369	173950	105534	162599	110714	141611	121020	147993	241642	111678	140118	396874
EIC	Negative	363.1809	1.62	unknown	241735	224680	0	33610	92854	97586	151151	62299	95022	109844	144380	323621	243887	181755	148902	149668	199430	0	128392
EIC	Negative	329.1762	2.34	unknown	517882	309437	824430	669423	135563	335043	334910	303225	210592	373451	269613	501093	608235	259486	171514	359376	279832	585801	260394
fpHILIC	Negative	408.1415	2.02	unknown	550464	206212	350872	345860	136634	166978	279003	262027	433018	346315	235640	244306	328710	451296	484102	196716	344981	404800	173994
EIC	Negative	649.3500	3.94	unknown	513480	311386	1175900	293059	101132	1002723	400194	281101	19324	135988	417067	882610	1062205	563582	87421	478653	535747	919477	247163
EIC	Negative	265.1420	3.80	unknown	142064	98796	142218	203829	94732	105985	96156	81312	75167	132062	106461	119247	145885	12606	106607	133144	104472	156588	69202
EIC	Negative	315.0996	0.85	unknown	84460	46504	76000	86714	38496	30390	57810	75175	35374	67094	65641	59901	71545	31095	43017	71629	57507	81364	44833
fpHILIC	Negative	217.0828	3.61	unknown	1089886	1021194	608587	647998	625128	1073455	492697	429018	462638	813491	879675	645682	773351	702721	1116879	648699	790035	688140	633336
EIC	Negative	531.3485	5.81	unknown	1291701	543628	1203559	2899289	448346	883522	1111397	1333407	1112423	2226445	1173691	820117	1565864	155318	886263	1045300	1172474	2712478	334961
EIC	Negative	513.2773	2.08	unknown	2913459	602762	0	0	241251	322355	495307	0	279347	944846	439627	72845	237514	398450	462282	806793	128456	0	286611
EIC	Negative	550.3181	2.65	unknown	1140675	578322	1004495	644339	449100	134603	159730	190199	257483	159386	160818	271056	249916	353285	291489	342686	393883	0	471540
EIC	Negative	527.3176	4.24	unknown	1480488	948253	1403002	4112452	893515	1693851	1402284	1821767	1635870	3280041	1767867	1687362	2300899	318727	973194	1486399	2238522	4067938	610787
fpHILIC	Positive	149.1168	5.22	unknown	390971	495132	407010	333609	355021	535769	414776	205297	367473	347636	454738	553078	538546	480129	660781	484547	395516	372914	451723
EIC	Negative	278.1769	3.20	unknown	170575	90045	138130	141540	81047	108014	105273	108449	48067	119490	103389	130477	92921	91940	126927	74732	136277	134472	95769
fpHILIC	Positive	504.3177	0.37	unknown	275297	204302	324361	908745	203857	235202	319750	392563	341177	804032	319791	280712	445598	84905	165790	259924	462713	850700	141996
EIC	Negative	375.1186	1.24	unknown	66525	32590	51976	97012	38923	15900	37643	71284	37177	40436	37265	60094	82307	30424	33730	68926	60722	60068	29795
fpHILIC	Positive	599.9490	3.77	unknown	1229356	260861	95138	2565481	221281	619468	51606	1653347	1721274	395174	726417	987247	2911037	1464750	351069	361134	2376071	1696204	594732
fpHILIC	Positive	144.0803	2.61	unknown	877443	1156681	852295	551274	816495	974328	986332	582940	738591	753668	1022825	858483	778891	1172349	1405475	1167203	781564	894592	919073
fpHILIC	Positive	103.0585	3.18	unknown	561763	591894	431413	323710	426274	570757	467211	294772	481690	390816	640503	701808	582039	574773	609439	617427	516843	448733	633029
fpHILIC	Negative	192.0505	0.86	unknown	562809	311033	309272	351742	232729	320083	238749	281508	224434	334794	395051	426868	293805	450239	541778	0	300407	402775	391256
EIC	Negative	284.2226	5.07	unknown	448285	218463	366075	565965	185933	118528	196840	146215	255553	358017	224811	263229	133365	199794	190562	255175	216413	362214	114564
EIC	Negative	338.1429	1.15	unknown	38771	30685	89792	15832	20944	32028	13862	75162	19466	42466	26417	35818	17443	74061	27951	10972	36898	68933	58896
EIC	Negative	473.2700	2.88	unknown	1682518	969230	1862649	4970860	1126694	1776210	1640166	3687841	1567726	4966666	1816409	1905884	2567498	342043	884869	1187694	2863260	6666564	757925
EIC	Negative	314.2419	3.14	unknown	180210	57022	166542	151670	75607	127786	91320	121023	114495	108929	66773	94897	98679	91514	31665	219480	137147	193175	87458
EIC	Negative	471.1697	3.94	unknown	238858	200686	324604	174128	130160	285278	221977	192335	161562	157700	297632	329199	353683	270108	128810	261543	268660	391730	199975
fpHILIC	Positive	121.0689	3.18	unknown	6693310	7533442	5212613	4666310	5522097	7028341	6410186	4114430	5774836	4721956	7979188	8278399	8260599	7929541	9417630	7122012	6400449	5449737	8055588
EIC	Negative	343.2124	3.47	unknown	140418	216535	126684	81119	168122	195005	102408	72982	87878	84748	69173	101301	279136	122861	66011	388488	94861	123597	119319
EIC	Negative	432.2776	2.91	unknown	272912	194268	166373	172906	128268	75770	132388	69922	115006	161366	152608	162624	214255	232857	164411	161719	112092	140815	80530
fpHILIC	Positive	147.1127	5.21	unknown	107851640	143464032	106800432	87213704	97212416	122002544	104685032	66192604	99839512	86635160	130607632	149716992	131740096	123515440	170078288	133384600	100192608	97894408	118284072
EIC	Negative	533.3856	4.98	unknown	222619	119765	278606	199024	123186	89510	115154	232569	35565	242654	190860	200748	91518	138039	127750	128425	140491	190396	167425
EIC	Negative	337.1112	0.46	unknown	150062	163334	103463	81016	90635	93803	89792	73021	76615	71570	113210	99911	120696	95848	177679	121116	54916	78055	104967

EIC	Negative	299.1879	2.81	unknown	124913	123463	126507	109080	89023	144580	124651	89695	96349	215214	131911	209985	224205	128844	140877	130788	157431	146532	113767
fpHILIC	Positive	118.0742	2.78	unknown	3392169	3399640	3169609	2213202	2404110	2906126	2248364	2182342	2425086	2710949	2627613	2525499	2388927	3448961	4214834	2367504	2335923	2902098	3590963
EIC	Negative	257.1759	2.16	unknown	335225	152741	282678	199052	129295	194095	124218	171084	121379	298784	122032	226849	154436	303145	176023	139930	162726	233162	226387
fpHILIC	Positive	117.0670	2.79	unknown	3269522	2988318	2783624	2121545	2230126	2889536	1919521	2004023	2042665	2418545	2355210	2632436	2037477	3163464	4121928	2350579	2095154	2576497	2914245
EIC	Negative	507.3690	4.41	unknown	673269	457968	615157	575854	499363	288255	395611	587298	407892	743596	606694	540398	455623	328988	548602	631583	479587	664822	438766
EIC	Negative	493.3542	4.09	unknown	292231	532615	330339	183245	433834	236848	424453	253873	393488	238465	384847	306242	336096	391748	617728	511118	325574	257749	498922
EIC	Negative	318.2156	3.91	unknown	1001243	428225	1665968	782927	306893	252621	751704	619100	260024	670208	543823	747732	900640	491345	249235	598966	620683	943694	368988
EIC	Negative	618.3422	3.06	unknown	2451730	1696941	1536868	1433407	1151620	2576785	1571488	1713918	1724993	1883242	1579142	2822650	1768574	1001763	1813106	2531449	2826448	1166977	2760857
fpHILIC	Positive	190.0763	2.61	unknown	436386	648974	448911	285471	411989	479261	499368	279591	349627	321553	477542	478391	498555	532193	774751	485050	390266	419499	496603
fpHILIC	Negative	249.0217	3.23	unknown	10785753	6503601	4461743	6774648	4204925	7509266	4043331	3531277	5453608	4432945	3473224	5776985	5795756	4506027	5238161	8079462	5442288	7487202	5190281
EIC	Negative	624.3313	3.94	unknown	2073473	7872412	1652445	2958694	4172769	4477691	4044116	1944429	4374541	3159605	7721329	5093304	6018205	5167570	5243912	5107033	5300322	2194394	6315381
EIC	Negative	594.3423	2.53	unknown	321854	270950	351960	197891	269090	335263	326472	181607	299684	269988	354509	332331	228002	312212	323876	382845	349200	213461	527532
fpHILIC	Positive	159.1124	2.70	unknown	1886595	2282745	1985220	1525349	1957689	2024771	1975769	1580511	1559859	1917389	2867630	2315197	2180300	3178881	2968436	2180522	1986580	1786903	2657154
EIC	Negative	296.1873	3.28	unknown	183286	23226	87114	124987	39738	108501	20201	86102	17161	95396	63201	87683	55468	33606	85811	41088	69237	96644	53085
EIC	Negative	628.3727	3.94	unknown	754447	468227	2109291	469485	185025	1573654	559326	404371	243587	210969	663332	1323234	2222760	902985	146709	916786	873093	1461527	378673
EIC	Negative	383.1827	3.87	unknown	161711	63440	211023	303511	81844	125114	147691	285451	174826	457671	125048	166501	148758	146488	121298	126617	134459	524533	130980
EIC	Negative	411.1846	2.91	unknown	5736494	5467617	5510453	5551879	3745877	3461044	5249789	3563069	3976291	6018114	5612840	6020397	5598664	5363574	4945273	5614477	5669039	4909981	4335652
EIC	Negative	333.2075	3.80	unknown	2340001	1093963	2714023	2097720	995188	1161449	1281192	1639378	1131542	2805860	1185981	1453173	1715988	1141985	1150570	1306304	1437830	2600129	1415354
fpHILIC	Positive	207.1028	2.61	unknown	1422974	1797505	1456682	858629	1197167	1449839	1744150	951512	978209	1250857	1572449	1546883	1331247	1820655	2316489	1598206	1187037	1344080	1611699
fpHILIC	Positive	509.2871	0.37	unknown	1092154	1083482	901834	770649	1039358	469086	854303	882900	406114	713190	934376	806978	705357	1072331	1003270	398068	1050037	1188531	1015700
EIC	Negative	346.9010	0.56	unknown	579033	76005	169173	247163	104031	296150	71960	11913	83841	318944	75827	69650	85461	67853	101716	154673	38800	123037	305800
EIC	Negative	507.2727	5.84	unknown	25837857	25124210	21815747	18856338	20199813	11106348	17454791	21680803	8593398	16355489	18367100	16243942	15282856	25922884	23421761	9580070	19614475	29332307	18815663
EIC	Negative	312.2264	4.35	unknown	2847522	809498	3501335	3712247	1307874	949387	1255450	3117793	1085878	4764946	970166	1215443	1182803	1581634	1602017	825215	1291551	4949136	1178164
fpHILIC	Positive	198.0865	2.36	unknown	3209424	2657987	2856004	2619971	1972818	2988556	1734257	1964640	1762505	2626629	3114742	2253643	2597913	4306440	2474117	2757069	2770563	1906668	3393106
fpHILIC	Positive	171.0953	2.83	unknown	1829455	1630978	1150643	1119234	1295770	1489236	1069716	922136	1864306	1130135	1243318	1233358	1354996	1559490	2388291	1882299	1503750	1287498	1413881
fpHILIC	Negative	168.0277	3.66	unknown	459463	406143	369984	431205	354665	456356	327989	396385	369078	485992	391334	479372	385214	429294	447251	519556	534687	523254	406220
EIC	Negative	508.2763	5.84	unknown	7034995	6822871	5886169	5042392	5469493	3016441	4698799	5963833	2346119	4383629	4825907	4365832	4168160	7100184	6338411	2592580	5323487	7989406	5196502
fpHILIC	Positive	170.0919	2.83	unknown	24571946	23760600	14458396	15762170	17795866	20465716	16166531	11762041	24748676	15351748	16722984	17482172	19144630	20062026	32211332	27583356	19788942	17668324	18841936
fpHILIC	Positive	526.3135	0.37	unknown	640996	596896	460932	431882	557753	268962	484365	502726	229983	387367	526147	451330	433400	624760	556409	266465	597712	627623	561419
EIC	Negative	551.2846	3.47	unknown	1407543	633555	2340919	685092	526382	410213	703262	1154013	284931	1276821	715248	661337	392914	474576	541747	441456	493330	1845761	612129
EIC	Negative	394.2321	2.90	unknown	434235	208812	468965	311994	165726	100383	372380	339734	262474	438874	307258	410923	480671	255716	230365	258190	314595	469873	312716
EIC	Negative	365.2693	6.19	unknown	907816	535639	1078556	773183	583655	288549	675204	854992	517804	1146638	642718	475097	462451	602975	913276	803391	581933	769945	665023
EIC	Negative	339.2536	5.50	unknown	1068242	692984	1589467	1311620	693034	599949	663881	1083229	516933	1080112	694713	909247	779066	703395	661356	610082	844911	1786559	645809
EIC	Negative	330.1794	2.34	unknown	121495	65835	189856	167439	36848	78240	80168	75552	51814	59285	55810	114869	117241	64222	46529	79692	63157	169016	54372
fpHILIC	Positive	371.1465	2.47	unknown	706034	457227	325505	331175	280061	520189	246399	200116	298231	309923	531106	555652	328464	315947	498298	380156	299692	345894	452651
fpHILIC	Negative	112.9373	5.36	unknown	1189308	288615	1034115	350088	421906	567950	305750	509611	136807	376468	106540	232746	79672	194990	184258	1466810	92425	450470	291740
fpHILIC	Positive	119.0894	2.71	unknown	9897502	12208442	10384224	7822695	9127781	12502308	10642647	7837961	8518285	9569256	13362746	12944861	10970619	16746202	18234240	11017250	9977321	9915728	13125149
fpHILIC	Positive	218.0914	2.62	unknown	682884	839556	727148	516826	625878	717335	709467	446570	539158	611662	710037	681659	606603	920187	1065634	834698	625485	639179	727064
fpHILIC	Negative	263.0918	2.06	unknown	1475462	337918	296968	167528	223495	591989	207983	174327	260186	244056	205889	413204	163567	250651	232384	443595	218895	661974	310799
EIC	Negative	642.3007	5.54	unknown	386647	307018	364424	297837	313771	293788	266788	333560	304494	397944	337390	335889	284744	378953	367068	434535	375692	404822	355597
fpHILIC	Positive	309.1467	2.78	unknown	847964	801881	690131	543556	548961	654293	476689	539414	564262	746624	689760	881721	576102	723932	763355	712233	613818	641142	842266
EIC	Negative	589.2761	5.84	unknown	1016457	966933	865948	748033	831908	440863	686856	848888	333240	662205	699408	612375	653569	1044842	923836	369497	816613	1161798	762710
fpHILIC	Negative	509.2879	0.38	unknown	2030234	2187889	2097045	1946418	1463363	1682428	1617095	1656675	1010312	1234699	1273388	1720034	1037292	2145402	2079111	1270016	1715929	2581553	1896934
EIC	Negative	570.2976	3.10	unknown	138866	98281	152579	92812	51586	125118	54187	130871	114050	141764	104240	148263	104899	72893	113222	137257	110159	68087	182506
EIC	Negative	316.1998	3.76	unknown	7038017	3971962	9168600	4850001	2433068	3424207	4785168	3942233	2967400	3360325	3186558	5219576	5667253	3992586	2961714	5212620	4497316	6966138	2926326
fpHILIC	Positive	148.1097	5.22	unknown	922529	1057852	873396	599467	689798	995125	799132	489689	786347	675872	1076867	1154514	1066745	961531	1321959	985056	832253	774120	921614
EIC	Negative	299.2235	4.44	unknown	1294257	538953	1639853	1315066	669643	788031	698857	1262250	576476	1430722	662424	817730	619719	816378	655235	1264218	821473	1724157	673781
EIC	Negative	555.3685	3.65	unknown	2477193	3532911	1463476	1784666	2603221	2125545	2039022	2217231	1393258	1592802	2015475	2024258	2415478	1713061	2308520	2173006	3266437	2754959	2847927
EIC	Negative	412.1851	2.91	unknown	1591152	1479320	1475910	1468254	974497	901670	1424123	943969	1129023	1619233	1433959	1680330	1472479	1450122	1307054	1593664	1496738	1325061	1195144
EIC	Negative	337.2387	4.99	unknown	481105	304248	514248	428553	262249	185603	280404	418902	278905	608503	309422	291775	258732	326855	448385	364924	290568	471647	261875
fpHILIC	Negative	149.1336	0.42	unknown	859399	417955	641938	832973	454236	389817	393561	447656	183751	455370	363767	528406	647875	224628	435515	471679	566035	568871	366746
EIC	Negative	315.1967	3.76	unknown	31914265	18063798	42050296	22023445	10930664	15456765	21515421	18000984	13369565	15115258	14383795	23555011	25415787	18019315	13439867	23532496	20581798	31807439	12872785
fpHILIC	Negative	196.0731	2.35	unknown	860284	584660	631236	577271	542360	824756	441202	515476	501848	791012	703660	581043	561033	1162214	943659	654717	702594	504776	839550
EIC	Negative	619.3475	3.25	unknown	789607	534368	516704	457276	379618	856156	496170	571828	495177	657987	488616	912360	571486	302318	611685	832148	898998	358287	910035
EIC	Negative	499.2853	3.41	unknown	8524029	3348888	7741404	18969810	4077846	6248113	6635898	10479050	6536284	17284510	7435789	6795094	9193525	1277936	4716707	6168763	9452789	20971798	2526730
EIC	Negative	393.3738	6.87	unknown	223192	415511	326886	608921	332294	293509	323433	348585	235662	389632	439561	645663	487669	291069	342029	317921	362850	669975	286083
fpHILIC	Positive	118.0649	2.61	unknown	1076728	1362326	1024720	684010	871430	1080310	1227600	716493	800838	973017	1308994	1187353	1142137	1359738	1652313	1241070	969512	997727	1200612
EIC	Negative	492.2740	2.67	unknown	185206	197480	174783	100904	173733	235227	160512	120961	137025	120980	187965	166991	101958	402292	238378	114004	199438	165257	351686
fpHILIC	Positive	148.1159	5.21	unknown	6921942	8830591	6885750	5656925	5657067	7791827	6395011	4142452	6392055	5631632	8185664	9747097	8418890	7594812	10734442	8720811	6277760	6102317	7427943
fpHILIC	Negative	227.1039	0.97	unknown	1568953	243392	75849	142296	243168	297927	62754	172193	349699	127226	144400	364100	214584	326721	106186	342625	256182	491925	258465
fpHILIC	Negative	321.1415	3.69	unknown	654192	276027	273402	244157	206809	427822	203261	218419	196007	246156	152746	266549	175295	160672	273023	322953	333834	421553	268026
fpHILIC	Positive	430.3790	0.39	unknown	819574	515422	548517	461531	597995	465785	545625	597742	531992	695457	617323	710900	595179	553408	473872	775288	677062	659580	956826
fpHILIC	Negative	434.2393	0.61	unknown	1057631	1039899	971274	721978	0	1003308	970573	837132	773633	867369	1070182	1146566	865650	1124184	804206	673257	837663	1012968	1308201
EIC	Negative	376.2207	3.61	unknown	110828	74499	239825	132051	62578	85566	117402	69672	66221	231871	99841	153609	127517	102185	67656	106546	128160	117288	99910
EIC	Negative	272.1952	3.36	unknown	495400	162719	495491	324801	186919	251300	162708	295222	174115	488915	162075	210929	183839	166582	155330	581108	192084	314390	142498
EIC	Negative	328.9559	0.69	unknown	72143	89068	67000	45002	41378	43431	45937	30213	29533	55076	54150	46366	52878	46977	54729	61960	40616	47726	41597
EIC	Negative	307.1089	1.71	unknown	122783	198887	283123	237936	170169	177686	212936	262129	189089	184751	214956	218663	259118	412800	293509	219819	240581	346281	211619
EIC	Negative	475.2688	2.88	unknown	173328	105899	203137	378520	68363	150064	165487	251803	142950	411397	151744	181342	254302	24252	100706	114731	249622	544586	0
fpHILIC	Positive	184.0603	0.47	unknown	614475	312291	373328	802868	536876	1005059	739890	217080	279365	788635	699812	1075889	664614	246602	504052	464643	799435	489094	1345496
EIC	Negative	474.2728	2.88	unknown	588601	269942	595219	1437158	298543	516916	445111	780567	500944	1317627	506456	541830	669014	95779	237522	315549	808685	1726958	207938
EIC	Negative	278.1766	3.31	unknown	448247	206615	280920	344909	221423	398288	288704	283640	77471	304480	271851	406606	268022	216047	428324	244279	406518	302317	265506
EIC	Negative	330.1792	4.79	unknown	30326	84885	57789	148985	23670	25321	0	0	141718	69486	29349	36517	20814	0	39236	69947	88027	24714	25481
fpHILIC	Negative	322.0601	2.41	unknown	418150	516809	320970	222108	274318	412339	236173	298141	339017	241182	303248	386342	284580	455351	367906	534226	288577	449423	386628
fpHILIC	Positive	315.1959	0.47	unknown	2701080	1138767	3463207	2819750	650231	717035	1489915	1300089	738722	1808852	910357	1335607	1701589	806263	841102	1236248	1129375	2247928	908009
EIC	Negative	503.3173	4.54	unknown	2351605	1057938	2454885	6563297	1272325	2402367	2508130	3598865	2612834	6386190	2596644	2295231	3451770	414764	1273873	2087677	3287981	8726587	733072
EIC	Negative	495.3696	3.81	unknown	231942	288177	195238	195092	270406	284442	246555	172645	187095	259221	383949	281362	245395	282605	343829	340158	294454	246273	284762
EIC	Negative	425.1647	2.18	unknown	291534	182250	292077	276196	102854	165713	189062	154394	216527	298322	91826	273286	146620	130115	70245	227670	144673	385147	249593
fpHILIC	Positive	129.1021	5.22	unknown	1006472	1161803	901205	738334	869028	1071401	929112	530495	695808	783116	1094622	1368193	1158858	1021357	1328079	1104358	946391	925072	1022091
EIC	Negative	583.3037	4.00	unknown	454291	213056	344121	786650	220359	371617	374396	451491	382444	939339	389503	347085	453120	43624	236575	357041	534805	1085045	127585

fpHILIC	Negative	218.0406	2.94	unknown	2070859	2372125	2884668	2078749	1881462	2056395	1658468	2132988	1791050	2639520	1776201	2183593	1967203	1963907	2638600	2001690	2557135	3233660	1951042
EIC	Negative	293.2487	4.99	unknown	259379	112842	259690	181674	116837	78806	123753	197681	137184	346390	123984	144703	122926	138791	197657	192053	122326	231129	108520
fpHILIC	Negative	275.0215	4.15	unknown	628859	183923	230860	357468	251678	330890	169808	121518	46415	337943	244190	198789	75454	226852	290957	144062	159503	434558	169143
EIC	Negative	357.2060	4.05	unknown	531937	291448	509454	740780	370189	298454	258907	414850	347641	1017463	310313	315697	285373	302060	335364	305477	472260	721999	365771
EIC	Negative	625.3344	3.94	unknown	725660	2865248	612709	1032568	1466690	1599372	1459768	645556	1621921	1133727	2754193	1851430	2097084	1834433	1886222	1926546	1956415	783460	2301210
EIC	Negative	509.2789	5.84	unknown	1364576	1354292	1185703	1024443	1089832	594527	948502	1135500	489272	901234	1004904	936797	850520	1439220	1229887	525643	1102652	1573988	1118334
EIC	Negative	618.3422	3.25	unknown	2302528	1613612	1525599	1366536	1158499	2519267	1434433	1691820	1563542	2016026	1545961	2709363	1689058	969317	1808673	2547297	2685892	1090023	2784342
fpHILIC	Negative	300.1188	3.16	unknown	1003827	1384234	896768	770008	975502	1105432	1097592	922539	902423	1046732	1726843	1431757	1350475	1411668	1695152	1335867	1036429	1093872	1366429
fpHILIC	Positive	144.1016	2.19	unknown	589618	510941	390335	569804	788397	0	0	421167	964058	446810	481986	628891	652362	388750	502763	857156	649086	457347	370468
EIC	Negative	285.2073	4.37	unknown	497783	320779	571971	583435	243969	222129	398661	335449	333578	351287	338327	545053	595648	391882	383487	457861	391289	432390	336821
fpHILIC	Positive	122.0698	3.18	unknown	884145	1092683	797048	761070	817369	1040221	871915	624653	858380	714419	1088124	963131	1171767	1121228	1339970	1104871	959837	803896	1292315
EIC	Negative	318.1428	2.35	unknown	326059	152458	476105	463795	88872	139373	159294	357548	88132	280438	137981	251316	211478	178560	289110	205506	178407	297122	117934
EIC	Negative	363.2008	3.74	unknown	2689993	231159	563425	6502531	121471	315405	186982	118860	99004	0	1190697	226350	97195	49642	129446	297322	167047	305768	199792
fpHILIC	Positive	133.1049	2.40	unknown	14457123	14122018	12267772	8633252	11134102	16466432	13037744	6800494	12246001	12847524	14240488	14703525	13079928	16855024	25617728	16260124	10313482	10332789	15505828
EIC	Negative	619.3471	3.06	unknown	795142	572347	507700	465960	372892	843294	525985	551070	546533	652447	518201	926248	565710	337018	617713	845010	942748	395991	914309
EIC	Negative	495.1974	2.67	unknown	103880	172017	127490	153877	117672	103485	161014	107819	142795	130120	146133	222812	208363	181128	147229	153250	180131	187805	160142
EIC	Negative	315.1250	2.15	unknown	249001	215786	276821	292574	133725	137803	153268	228828	92740	176065	120654	270791	226957	227481	273872	225685	173544	167126	137736
EIC	Negative	507.2727	5.59	unknown	10194286	14172119	10098083	9185049	13407117	7053370	11295298	11529177	5333440	8671492	11753157	9551509	7872443	15764723	16855773	7103653	14093389	14003944	11684361
EIC	Negative	332.1952	2.67	unknown	5453347	4452023	5506200	5027146	2043969	2579197	4544706	2920461	3618287	3992139	3529574	5974381	5694484	4782401	3575397	4372973	4564525	4646580	3436410
EIC	Negative	367.1685	2.67	unknown	256082	245374	227143	181769	98426	129937	208509	144099	147158	208541	191682	236231	256529	220735	165580	216208	197364	186033	189610
fpHILIC	Positive	157.0798	3.32	unknown	11282682	5375450	11040516	7432561	4097376	5112137	4744253	6505331	4167064	9654556	5417475	6566560	5322983	6240166	7904535	5173464	4176313	9194046	5854390
fpHILIC	Negative	174.0599	2.71	unknown	663942	485624	533792	480975	365873	434728	308487	487304	226379	551890	424885	466693	356759	556595	547873	562373	329867	502881	448235
EIC	Negative	479.2028	3.61	unknown	160822	167845	299922	206180	80628	116988	161123	129379	99414	148238	183033	228550	257904	186506	106379	169103	167046	177762	150782
EIC	Negative	528.1782	3.19	unknown	787239	237009	347465	595287	317061	199325	249600	716548	249302	459989	338540	354347	387078	156847	233389	647878	442898	664207	285030
fpHILIC	Negative	258.0747	2.52	unknown	795053	697458	466486	630660	493564	903803	473632	448874	657173	610627	812902	858044	596989	648546	805927	807115	569079	815123	911034
EIC	Negative	519.3686	6.70	unknown	426490	797203	446281	334397	396811	482306	437218	209410	289590	235697	1312761	807161	738135	896732	771366	416006	308181	388017	500106
EIC	Negative	422.2299	5.92	unknown	3564531	3298966	2715958	2295244	2753935	2388673	1749619	1879877	1429815	1509649	3241217	2737907	3056995	2771885	3311100	2881494	2702457	2478059	2028339
fpHILIC	Positive	130.0864	5.21	unknown	16150545	21310190	15246231	13074269	13989051	18061788	15211785	8828678	12390357	12744620	18698702	22166256	19202362	17541428	24827352	19444786	14314744	14296635	17176858
fpHILIC	Positive	361.1444	2.47	unknown	3096188	2322049	2309296	1323850	1654816	2147005	1451043	1587888	1692970	1601460	2265857	2411059	2227498	2858928	2709830	2092856	1676480	2247348	2500263
EIC	Negative	355.1400	1.62	unknown	1646296	1406479	993107	172646	507129	1109314	559504	1083247	397306	992644	836627	912813	502237	2057097	1340431	492933	843820	926196	1215501
fpHILIC	Positive	107.0494	2.06	unknown	828713	455675	751717	385474	353885	473292	396053	416791	462505	596219	410424	492472	350966	626973	740371	587717	401766	632436	409906
EIC	Negative	332.9485	0.52	unknown	153677	0	92655	0	48131	67653	0	0	73163	219476	51480	0	0	58222	0	72012	0	0	177139
EIC	Negative	258.1799	2.09	unknown	191796	149601	205627	154072	83434	153273	124722	70969	92952	145487	135913	214529	225916	146349	156154	212288	146731	72659	104940
EIC	Negative	410.7598	0.52	unknown	690112	393641	671362	743016	327711	398367	459580	222152	456442	893370	396812	389611	492737	119593	373434	444765	334429	564544	743297
EIC	Negative	384.1853	3.19	unknown	409911	77602	575962	382017	76231	93354	94131	394826	57003	411300	92688	209647	137619	137912	144739	98010	141971	412126	147969
EIC	Negative	357.2301	3.84	unknown	2171566	360247	2755811	794329	310532	550536	405013	1189697	63948	1374084	284983	595179	324791	461398	412961	148930	390338	1724129	651199
EIC	Negative	289.1449	1.88	unknown	753854	582721	0	0	264213	526336	518836	0	366613	0	549685	693462	799419	664357	349243	699192	610012	0	555861
EIC	Negative	421.2265	5.92	unknown	15324260	13994598	11840955	10173110	11709975	10173548	7403877	8079392	6231468	6733419	14289429	11883451	13129763	12038986	14274506	12328968	11477516	10644066	8881610
EIC	Negative	508.2763	5.59	unknown	2723325	3955939	2780397	2416660	3638687	1945690	3076907	3064401	1406822	2305701	3196385	2572330	2121509	4257537	4551497	1891196	3816701	3913103	3184442
EIC	Negative	313.2386	5.99	unknown	776460	551454	1314955	712360	213394	388449	245093	181556	605630	294308	366487	630694	521158	359236	317878	380725	504856	712668	244489
fpHILIC	Negative	157.0504	0.97	unknown	769377	997618	1093558	564133	716692	609537	613905	403172	393139	771278	1821126	490844	633727	476970	404106	539907	391489	1906653	377483
fpHILIC	Negative	298.1141	3.16	unknown	42170100	56314656	40682136	35806804	39045728	45999044	52713700	39701372	39342520	42943132	74104760	68688928	56525800	62823572	71295064	60688756	46876224	42605516	65376096
fpHILIC	Negative	173.0567	2.71	unknown	9663102	7000558	7715302	7514074	5526219	6352408	4291455	7234836	3177674	7015775	6569377	6457585	5940073	7784485	7649646	7928032	5292831	7259098	7042361
fpHILIC	Positive	383.2015	3.22	unknown	403779	297983	367707	506663	205064	410904	212546	181929	251190	380269	375790	426180	420610	466016	253689	353257	247380	412164	402893
EIC	Negative	259.0647	1.77	unknown	1005996	2766650	811960	87410	952652	1010428	1969202	1136513	618060	0	3314819	3590202	1583888	3327758	2798420	1656761	1118263	0	1959603
fpHILIC	Positive	123.0438	2.95	unknown	5456581	4339021	5243826	3282703	2943476	3465632	2753725	3185871	3076147	4386672	3027268	2896752	2979519	4368035	4592880	3351176	3631238	5413628	3471536
EIC	Negative	500.2888	3.41	unknown	2323666	1131527	2214450	5012789	1362207	1842114	1935956	3050682	1917113	4345390	2226049	2052722	2989131	433971	1433398	2131746	3108294	5966738	769804
EIC	Negative	433.2212	1.78	unknown	196403	105025	174913	0	38374	67667	85482	23860	64987	228574	92847	116020	88496	93111	71231	99583	88395	0	112954
fpHILIC	Positive	76.0399	3.61	unknown	5432676	3576790	3770803	5864300	2787662	3865845	3005363	3114953	3656231	4602506	3495233	3740519	4119936	3129093	4193116	5090956	4373282	4161868	4001309
EIC	Negative	390.2943	6.39	unknown	23490398	8814691	16698076	11612293	6997074	12304046	0	7805044	0	13674160	10925420	10025642	0	15514145	12851578	4906484	9470733	2902307	5873667
EIC	Negative	463.3071	3.11	unknown	586938	32785	697942	563623	96899	218541	41768	1160013	111044	984239	112283	86454	89504	119121	108080	67009	106259	1819241	63001
EIC	Negative	317.1398	2.35	unknown	1850767	801129	2286905	2410239	500808	763787	818912	1693145	467199	1395607	700330	1283856	1066258	930099	1460728	1090393	897817	1596880	597231
fpHILIC	Positive	121.0625	3.19	unknown	464333	681484	517399	377613	470043	539368	416521	351428	452609	383978	700813	649011	680787	574647	730135	622195	493699	449498	670527
fpHILIC	Positive	147.0436	2.94	unknown	2548095	1853564	2236005	1812409	1401549	1686811	1278408	1547040	1220744	1783442	1288330	1291964	1399304	1883495	2416508	1380321	1654131	2116817	1929199
fpHILIC	Negative	144.0302	2.05	unknown	1595925	1076057	682023	622629	636598	1570830	818937	519310	602139	715628	587494	799736	499709	729962	774088	1307181	914397	1160037	1367585
EIC	Negative	345.2643	3.77	unknown	286108	491081	1160904	755060	270199	209536	284221	525946	395723	310739	367978	463785	484590	424657	376221	286487	412174	990889	178973
fpHILIC	Positive	137.0786	2.94	unknown	1615636	1244385	1559789	1142286	1005194	909944	930964	902261	1118746	1258604	873561	892751	901805	1350934	1296430	1043201	1222269	1702055	1219250
EIC	Negative	595.3458	2.43	unknown	353316	260552	500802	322176	172200	253111	211270	371003	114797	220991	290277	280657	205015	509690	262907	161777	233148	414950	422698
fpHILIC	Negative	241.0564	2.61	unknown	1621125	2598577	2087143	1064205	1801737	1760238	2276316	1613620	1349187	1866474	2603642	2106472	2005904	2817242	3449872	1878146	2120418	2025409	2258591
EIC	Negative	316.1624	2.19	unknown	285168	207186	233530	318322	118421	97675	144360	73399	115823	203255	94915	89562	169969	56381	105630	136192	80706	242777	167944
EIC	Negative	529.1820	3.19	unknown	244059	84308	103343	193768	107431	55960	87347	226150	86401	168621	109640	123861	127918	58071	79088	218104	133204	224474	96627
EIC	Negative	330.7538	0.52	unknown	513652	149729	646382	369057	248396	290376	228478	83782	336777	829637	160355	135752	219808	219429	207339	271533	148556	248906	708429
EIC	Negative	317.2340	2.82	unknown	0	137407	363878	341487	98202	95084	111519	259432	131944	96253	133918	150742	177495	135885	119672	119760	207391	408350	74291
EIC	Negative	413.1052	0.69	unknown	63723	43531	44026	33285	36553	50048	29317	13508	29837	42694	37011	36972	29026	49405	45844	55501	33061	28135	50108
EIC	Negative	271.1919	3.36	unknown	2799307	991160	2928543	2029726	1063665	1403388	1104107	1778904	1040578	2855186	1045511	1273642	1103467	901695	945467	3668159	1081035	1887184	960091
EIC	Negative	557.2874	4.36	unknown	258856	0	0	267125	221155	0	0	0	0	0	0	0	0	0	0	0	0	295907	0
EIC	Negative	356.2316	6.29	unknown	577728	307105	298307	362362	239270	203981	205492	339451	104996	233621	243732	389146	257506	194229	363019	321152	258705	294684	225701
EIC	Negative	552.2888	3.47	unknown	479467	207004	765448	211832	173695	142375	267333	375384	0	406487	217191	238128	0	150541	152181	156498	154171	613044	205533
EIC	Negative	260.0679	1.77	unknown	144412	356873	104312	21831	116965	127770	253769	138573	83162	14579	423149	453089	207968	435943	355378	213076	138933	0	257158
fpHILIC	Negative	332.1953	0.55	unknown	2126117	879551	2441785	1689604	470988	677849	931447	599043	673817	1318760	709088	947089	991943	741527	768593	1052488	714858	1308028	652340
EIC	Negative	343.2126	3.02	unknown	207286	166239	172396	131498	69538	201306	66011	104043	156621	87868	111739	210085	189475	208918	140008	164472	111102	203969	181719
EIC	Negative	369.1734	3.65	unknown	1047382	431343	832975	1010664	572314	495924	454614	1001698	587291	1076699	553268	575830	565364	436813	788207	869191	567614	1521846	428338
EIC	Negative	558.9958	0.55	unknown	128555	117108	114417	266615	0	191859	92116	0	84477	66345	146794	133653	99637	0	87146	150215	76592	101217	79393
EIC	Negative	431.3020	4.26	unknown	0	122636	0	341024	197849	174707	135833	0	28291	226148	94806	137311	75187	260828	187490	0	92691	313382	127018
fpHILIC	Positive	184.0856	2.94	unknown	1634728	1315525	1560812	1034507	889667	1082155	899314	1220464	1017150	1437344	860758	1029842	987555	1776114	1650540	966911	1243243	1751465	1248358
fpHILIC	Positive	343.2961	0.61	unknown	1693762	457313	727977	209239	541270	716798	636256	574028	127333	933943	1080804	658154	571591	566641	629873	559536	962928	740333	593799
EIC	Negative	373.2372	5.78	unknown	310699	319352	216313	200531	292161	265566	222568	261565	189526	219327	265397	236268	236405	202557	295354	335523	308144	360655	355049
EIC	Negative	643.3459	3.39	unknown	251077	333995	180041	165345	165076	333645	194870	169119	331694	249034	284233	325815	252479	149509	234589	403485	411033	129835	426269
fpHILIC	Negative	223.0757	0.95	unknown	402715	401698	506758	346314	292783	389647	332956	290791	384294	383465	439757	393825	357442	498010	636605	456673	310200	287662	581036

fpHILIC	Negative	200.0154	2.96	unknown	371030	512237	327769	165929	220642	443973	328545	246901	320385	209427	336794	408070	369916	731195	470138	235069	409197	350380	632857
fpHILIC	Negative	190.0541	0.61	unknown	32952584	21256540	24283652	23005138	14074455	19650720	16224852	15585232	12556415	16800270	22868370	20044974	14167829	19991208	27822568	14584969	18040304	22319860	22225066
fpHILIC	Positive	87.1002	2.40	unknown	1017756	819863	679283	537298	720786	1106128	763319	378662	704845	808194	863075	899601	860573	937315	1885665	931798	624383	612427	885475
fpHILIC	Positive	119.0835	2.71	unknown	678470	771751	752360	503821	724178	844766	703751	508817	558355	695308	986437	1000378	779518	1188875	1248330	875946	811368	597389	960281
fpHILIC	Negative	335.1296	3.34	unknown	1325716	753262	1174269	889513	558336	655296	602596	780806	526119	928977	863008	869519	764020	952517	1083803	725495	589848	986101	817516
fpHILIC	Positive	156.0767	3.61	unknown	93859464	46294484	60937708	58217524	29960410	41817528	31366394	39735924	35248428	67092692	38823240	41439996	35946296	40705552	56917880	43154040	34558944	61408680	43664988
EIC	Negative	392.2169	2.67	unknown	176292	119930	117772	143906	74484	71350	144796	47972	115079	116470	100591	172083	170214	65844	109705	124304	140757	136164	99703
EIC	Negative	310.2108	3.80	unknown	2540688	685739	3385677	2415253	736044	761914	751607	2165469	629855	4083446	704792	869826	678477	1619922	1329378	556232	882910	2873165	1151116
EIC	Negative	227.1652	2.62	unknown	182628	273634	182834	196136	183119	299643	181189	122447	196679	331812	354263	363390	291919	155773	219210	359742	345180	173723	226180
EIC	Negative	589.4474	4.14	unknown	198540	167034	453452	440429	94227	75186	134099	187550	143781	218703	103650	179508	183227	230283	142155	124105	192718	273702	122563
EIC	Negative	423.2266	5.92	unknown	1162244	1059003	898789	756597	850340	747495	561972	618414	451674	505243	1054195	880468	987446	878163	1084321	950543	889602	778313	649870
fpHILIC	Negative	201.1131	0.61	unknown	1003294	1176996	848137	1834515	601377	1878809	904107	1064463	1405181	1683819	2088929	1953082	2965516	1175332	1584119	2409929	1298102	1494489	1105536
fpHILIC	Positive	111.0748	3.33	unknown	503575	223938	601936	398142	237816	237576	364619	324173	185092	572796	340848	275125	325410	305118	438741	227909	243987	455200	453833
EIC	Negative	406.7654	0.52	unknown	439044	229310	431144	305650	271475	342440	171703	70880	395299	585930	296751	237403	261167	0	280777	363611	182572	374816	486780
EIC	Negative	320.9970	0.68	unknown	129009	166759	187337	118954	107761	104467	116777	127388	99894	158940	137589	128072	162292	155792	140325	168634	129348	168910	143173
fpHILIC	Positive	165.0542	2.95	unknown	23106582	17161544	23791170	14762781	12948536	14154799	12001328	13008197	13343301	19272474	11353236	12170436	12431084	19324486	19277438	13192857	16693209	22286650	16947332
EIC	Negative	643.3460	3.15	unknown	286185	400603	189372	229755	213719	389322	222424	215011	407397	295009	331498	424829	339898	184579	306741	527674	455300	157382	509327
EIC	Negative	369.1736	2.90	unknown	0	0	445710	0	0	0	351100	0	0	0	0	388701	442131	0	0	0	0	474493	0
fpHILIC	Negative	278.1247	2.57	unknown	327074	703243	624346	310969	308118	528932	831648	257818	208514	523810	662800	572502	498976	561001	893341	384463	498095	456285	653483
fpHILIC	Positive	102.0373	2.47	unknown	1404219	1758962	1430231	725208	935441	1412174	826806	840426	880693	919438	1172495	1239280	1060573	2194214	1457672	1003351	1249295	1205961	1704967
fpHILIC	Negative	137.0356	1.01	unknown	1527715	660345	455231	518597	745615	787036	315711	700460	1172980	1167666	721381	816528	862409	1143276	713084	885328	1476203	1441824	517581
EIC	Negative	283.1531	3.31	unknown	379871	441892	237164	200338	375512	313072	314112	311217	238531	252014	338624	353225	387934	337452	432014	344242	447053	371877	505126
fpHILIC	Negative	204.0860	2.61	unknown	4517851	5719065	4655108	2888145	3916412	4627120	5163000	3233347	3541776	3773305	5339578	4905185	4287693	6034595	7441778	5525132	4399416	4814835	5539436
fpHILIC	Positive	253.1146	2.79	unknown	723867	751424	903538	841672	664382	720082	471960	644968	720485	702201	795668	854590	616048	829106	1103895	648802	687232	961151	960269
EIC	Negative	594.3423	2.43	unknown	1041070	856688	1482650	848846	479630	791327	659023	1126684	392857	737288	888894	899404	668827	1683839	825271	489903	697594	1308637	1348394
EIC	Negative	330.1794	2.42	unknown	238173	249440	373397	195345	158409	151915	122697	91004	150896	143613	173434	192100	182804	178561	173574	181875	168357	187681	202602
EIC	Negative	570.2952	3.10	unknown	135754	92518	153747	96963	51586	118926	65910	130871	95629	142348	104838	149533	104899	76539	98292	127133	106684	68087	227203
fpHILIC	Negative	896.9057	3.78	unknown	702476	204232	37687	1063657	119705	328773	0	821423	769838	146371	372064	500263	1442680	777375	206066	191123	1351077	743646	273543
EIC	Negative	618.3423	2.71	unknown	2823477	2355167	1534413	1801657	1401188	3400554	2212233	1886550	2461851	3366092	2526025	4037739	2655564	1332134	2585707	3333704	3787092	1713410	4002015
EIC	Negative	427.1797	3.24	unknown	84630	46865	90432	73615	20079	13183	32446	27328	20893	58251	33919	36655	39305	28164	26475	26330	30084	43587	28299
fpHILIC	Negative	155.0463	2.59	unknown	1305434	1215486	1109403	1238334	711582	1765602	938085	809196	552460	1194482	1644080	1617443	1677045	1544387	1274072	965197	1602740	920673	1730460
fpHILIC	Negative	131.0906	2.30	unknown	5814940	4348916	4809612	2856842	3874862	5437142	4633048	2111818	3843015	4084167	4836933	5215613	3859519	5629208	10350652	4773115	3627779	3439764	4858705
fpHILIC	Positive	131.0896	5.22	unknown	1125161	1578045	1056194	862539	857965	1200854	992492	592363	867376	803580	1262517	1527152	1344811	1198912	1682860	1398275	920839	974187	1157902
fpHILIC	Negative	299.1175	3.16	unknown	4919415	6419202	4796674	4030048	4483212	5247518	5983895	4325711	4732878	4874781	8596126	8220688	6805421	7331079	8412997	6976083	5544522	5246221	7531583
EIC	Negative	239.1287	2.33	unknown	425816	206582	302614	510330	211063	244113	267258	72425	169294	274690	191470	245399	313600	127480	259272	321420	196553	397378	137759
EIC	Negative	332.1952	2.17	unknown	1244631	789601	1328909	1225137	401386	372620	598771	497053	457186	1091404	517817	778947	568070	513389	487367	794021	541254	656431	688515
EIC	Negative	409.2248	2.48	unknown	173321	172770	0	211368	47165	134978	68590	113828	81391	0	81654	160841	134914	123798	100862	137496	139651	116891	113007
fpHILIC	Negative	155.0655	3.34	unknown	2442274	1438174	2407172	1502629	1182408	1303005	1052325	1252411	987193	1673650	1339391	1382530	1208383	1769108	2135646	1328386	1100042	1762070	1550180
EIC	Negative	378.9556	0.67	unknown	87866	101169	130390	84098	71424	68918	71289	81074	69076	112815	85408	85000	102792	84352	88212	113462	78531	110114	104109
fpHILIC	Positive	317.2113	0.39	unknown	1194342	800486	1696550	1072410	359856	646497	728534	647607	492697	929251	744719	981311	1009799	680230	470098	888420	935671	943083	692318
fpHILIC	Positive	120.0300	3.18	unknown	400302	504713	296935	287640	278124	473451	341217	271574	391958	307908	481834	507791	573036	548210	541721	503956	439710	368119	548777
EIC	Negative	345.1712	3.77	unknown	456962	611403	579489	364527	190856	233246	423670	174779	204253	277264	380184	522615	514300	249855	342166	396575	313354	247440	379080
EIC	Negative	373.2960	4.92	unknown	82593	244931	524608	318017	83060	26363	69936	154779	106776	67156	102833	151315	196878	136991	114048	66162	107693	263035	52451
fpHILIC	Positive	157.0796	3.61	unknown	6208549	3036894	4210267	3910353	1955823	2580641	2093879	2676814	2270257	4327655	2716276	2737625	2391271	2675667	3723554	2953554	2290827	3894399	2992016
EIC	Negative	389.1923	3.88	unknown	88651	65012	101031	119927	71053	93360	78574	145247	109189	251611	82644	139707	95015	110393	82511	96423	101213	307224	93843
EIC	Negative	581.2886	3.41	unknown	279442	134908	270246	457057	139250	232059	230172	330268	245313	531078	271581	242314	334879	55329	174651	277574	345486	714489	101741
EIC	Negative	374.2059	3.94	unknown	71077	62870	119061	79182	21094	97518	55055	28589	25631	27795	62574	87389	101520	75249	21829	75761	79777	91244	43878
fpHILIC	Positive	119.0490	2.94	unknown	2749857	2188461	2612335	1866572	1325451	1523512	1374958	1523066	1553814	2250620	1402285	1455885	1437170	2553528	2429768	1517847	1908033	2286581	1874088
EIC	Negative	642.3422	3.15	unknown	844327	1107836	496771	637263	581479	1173777	622153	574748	1109134	838519	926280	1204284	918912	508984	845609	1495503	1312806	432900	1486012
EIC	Negative	257.1760	2.09	unknown	1248605	833979	1275896	738290	525313	885369	850203	505400	492302	671570	785243	1452969	1463255	910496	1008198	1245465	942661	642361	632814
EIC	Negative	596.3582	3.42	unknown	593884	351721	387557	207097	189526	564904	274417	373042	316553	471863	355476	562480	321320	207209	469913	598636	509094	202631	577892
EIC	Negative	324.7624	0.52	unknown	782767	254336	925506	563184	378877	438413	282673	110183	517587	1222468	221909	191549	376727	297568	265129	462854	186947	417871	1039689
EIC	Negative	595.3456	2.86	unknown	242085	232278	209984	149873	147854	275087	180229	194973	223751	259810	266423	326835	169904	203018	248192	345564	282945	131329	396597
fpHILIC	Positive	136.0753	2.95	unknown	19666962	14814464	19224146	12904474	11169176	11894615	9789823	11123063	11195895	15027277	10222739	10273452	10210540	17600452	17637198	11409148	12933813	19362738	14501477
fpHILIC	Positive	293.2175	5.21	unknown	1652198	2014339	1488452	1259736	1280417	1716231	1602332	760952	1093947	1309664	1299521	1917938	1929703	1835370	2499830	2008867	1482521	1279463	1602117
EIC	Negative	397.1997	3.61	unknown	1106925	901926	1905540	1195059	420176	663127	1089088	795853	566210	959750	1125054	1550006	1597920	1111527	546389	1029627	1066883	1255986	961961
fpHILIC	Negative	238.0252	2.95	unknown	1247254	1006998	1192024	732915	868127	669450	604186	822071	690371	936811	556354	686748	681589	1130213	1275034	627831	884247	1342546	901431
EIC	Negative	357.1692	2.54	unknown	327841	359961	380642	441876	177568	190503	262064	247850	172799	243252	239959	369340	379806	273708	352020	301227	323845	216796	253002
EIC	Negative	261.1134	2.87	unknown	2976444	1016469	6444949	1922856	739751	1516323	1149806	2652425	313131	4290785	1581729	2121325	1154312	1945989	1456354	698946	1464405	3570579	1586376
fpHILIC	Negative	410.1888	2.61	unknown	2062394	2745917	1878258	809246	1636129	1672542	2183247	1049598	1275891	1358535	2536512	1914554	1778559	2623668	3471906	2065092	1500326	1637616	2103335
EIC	Negative	274.1847	2.51	unknown	1156582	47076	581098	1429360	36975	69534	57698	1064817	37428	1034922	34407	48142	40092	9496	47286	31884	36897	1595887	34410
EIC	Negative	292.1636	1.64	unknown	222871	112208	0	0	44457	114977	120627	0	87949	193024	104585	183657	120589	74695	59033	103830	88668	155079	101101
EIC	Negative	558.2820	4.47	unknown	846352	605135	895666	627518	548950	454157	489243	706803	462051	827561	628851	532146	528144	869534	877206	498421	610984	1033907	653016
EIC	Negative	282.2079	4.06	unknown	100454	123332	83999	279290	75585	80344	78232	109017	86988	106632	58139	55655	142038	36793	91813	163674	149917	151548	37075
fpHILIC	Positive	120.0477	2.48	unknown	1233865	1362296	1142439	563115	745119	1077125	722853	669755	667791	885068	918533	1059102	822674	0	1311323	795643	870444	905262	1482263
EIC	Negative	596.3514	3.00	unknown	406276	250451	304194	90125	177313	360663	166871	104247	209435	0	270707	408859	235276	317505	241272	386307	338472	0	441716
EIC	Negative	289.1812	3.25	unknown	2689014	1427236	3756231	1941991	784859	787356	1586265	1291131	1083862	1243430	941126	1980325	1813014	1173582	1293128	1847424	1499066	2007427	1008837
fpHILIC	Negative	231.0601	3.61	unknown	960747	650283	976377	775275	611933	506055	449537	729238	417700	989077	890841	785977	740022	681791	1078521	786794	607564	1000074	287028
fpHILIC	Negative	201.1132	0.94	unknown	276156	306453	199039	546428	238655	483625	289819	233528	427227	441854	572704	530949	779706	295293	386491	824132	405319	378041	323664
fpHILIC	Negative	334.1253	3.34	unknown	9850893	5355895	8908578	5733099	4005369	4663017	4321368	5239675	3974521	7176717	5372980	5928144	4949108	6171615	8448818	4903715	4594090	7253582	5580530
EIC	Negative	371.1890	3.24	unknown	232873	103517	356507	131843	27274	0	144698	104720	103100	0	34880	185526	150303	40049	126383	147198	122884	35535	94501
EIC	Negative	514.2856	0.89	unknown	210573	19566	434619	234197	92097	29716	0	590070	94368	594827	119236	16005	17617	16026	21677	0	18253	987441	90597
EIC	Negative	373.2023	2.89	unknown	232825	178772	291053	275240	147340	74927	135543	105053	128257	158999	95887	202815	132710	122590	170469	214733	194378	148463	91673
EIC	Negative	631.4006	3.93	unknown	165280	68731	419269	563335	65554	148897	141041	228079	139783	164015	133237	374070	336809	68799	0	257212	198204	484945	62792
EIC	Negative	290.1846	3.25	unknown	527088	275677	758292	360321	153328	147466	311832	263336	204366	246140	186402	394547	346766	232117	265928	355628	280176	397112	200059
EIC	Negative	561.3106	2.31	unknown	1449998	0	3772378	2851774	0	0	0	1703140	35057	2166704	0	0	30000	0	0	0	0	2413538	0
fpHILIC	Positive	338.1364	2.47	unknown	753724	568537	561208	559051	480747	653454	361603	522780	544949	431631	617212	633462	445167	765487	949548	683165	508018	574202	845056
EIC	Negative	383.1477	2.31	unknown	368649	197430	309406	197339	81498	119763	171517	186267	164138	188397	164498	275771	221349	219971	211793	197779	208700	222658	163374
EIC	Negative	300.2268	4.44	unknown	258402	82364	327661	270469	101286	167279	191772	193892	80615	292203	98296	148500	120968	175698	118697	262951	177578	320706	141160

fpHILIC	Positive	104.0529	2.46	unknown	10317424	10552874	8745997	4061318	5496007	9099711	6087006	5438370	6163786	5796204	7895153	9193613	7102168	15201949	10817158	6791311	7000285	8393424	11861309
EIC	Negative	413.1947	2.67	unknown	1406271	1229648	1146796	1321639	623298	744967	1239856	756593	994675	1080866	1029370	1558045	1766526	1332923	1022514	1211762	1255484	1307409	1262907
EIC	Negative	262.1166	2.87	unknown	494549	183389	969533	311475	136299	234227	199026	439311	68586	722323	261279	366536	182345	321767	238165	122205	251461	612495	273731
EIC	Negative	407.2439	1.51	unknown	682929	1703482	731346	1073336	928033	1191230	1123497	508529	1121043	769930	2254952	734658	1899613	2032097	1111762	2339374	763990	1227840	1333130
EIC	Negative	516.2735	2.60	unknown	836949	489449	843798	0	341151	771030	355624	0	515919	0	363968	499884	393831	280253	519703	519255	911517	0	808402
EIC	Negative	329.1762	2.42	unknown	1016986	1122400	1795251	860693	631949	656140	550487	364089	660360	628268	736728	900225	845604	817811	769419	834840	719928	795355	863545
EIC	Negative	642.3422	3.06	unknown	1276430	1676811	879146	995476	1114447	1658523	1241700	783655	1808250	1277982	1589348	1879246	1393987	859110	1510720	2227316	2216185	728053	2514473
EIC	Negative	379.1777	3.31	unknown	187726	39178	377995	129589	21325	52608	16687	179667	13271	196509	48684	77033	63396	138321	36116	17763	75575	134350	130969
fpHILIC	Positive	158.0813	3.32	unknown	691168	372336	749844	388196	250560	276985	316856	452172	228400	630297	438309	426500	335133	412088	476949	315638	329650	564108	436981
EIC	Negative	258.1798	2.24	unknown	257486	74789	199404	283853	67700	120355	63493	122707	97857	283566	99421	133471	114550	149566	106667	114793	95223	226653	114151
EIC	Negative	362.1685	2.59	unknown	78153	224816	0	68637	99210	108058	172940	0	128793	70841	230183	328025	406670	210720	265714	214724	27395	0	201279
fpHILIC	Positive	360.1966	5.21	unknown	510951	646193	456302	439105	457859	519511	493535	401565	0	477753	578755	575994	533621	495958	558315	559167	568061	459407	466858
EIC	Negative	334.2109	3.53	unknown	388484	211374	625315	323818	109127	112463	204717	174620	109518	220646	144689	189500	229722	122295	164034	189944	174352	242258	220583
EIC	Negative	375.2175	3.61	unknown	402707	238074	826939	389812	113931	192572	302323	230376	148662	837345	333760	411517	480426	328006	136669	270337	334736	347157	290154
EIC	Negative	237.0224	1.77	unknown	182043	177319	174619	92463	137787	181634	161803	86794	161827	145147	165875	221923	300283	182126	146044	229608	485995	0	157235
EIC	Negative	642.3422	3.20	unknown	711667	783492	409210	466033	378156	819307	409105	443341	805463	729391	640430	936283	656771	327581	589428	1046621	952504	322293	1077855
EIC	Negative	643.3458	2.68	unknown	474488	541639	289754	334675	305066	603833	370150	313491	632120	448308	509668	641608	474748	264306	459366	807580	736569	232371	872528
EIC	Negative	345.1709	2.45	unknown	1095505	2515818	1115093	955855	1167245	2071300	1858656	500526	1248937	763650	2522945	3048013	3667397	2644142	3381911	2421194	2473250	774472	519475
fpHILIC	Negative	181.0712	2.95	unknown	2725116	1904407	2374904	1537298	1319724	1542349	1424242	1599968	1440106	2162293	1212314	1469413	1285457	2512447	2571169	1355613	1675819	2666862	1976336
EIC	Negative	333.2075	3.68	unknown	1507431	1100494	1601440	1976988	624525	449064	811442	684241	365024	1424900	787627	446056	889218	352534	669622	951766	625451	1054778	802750
fpHILIC	Positive	134.1003	4.83	unknown	652268	517566	507520	441866	446842	545707	528157	397935	545258	521012	442159	478709	487584	581832	885471	538490	834773	613454	575471
EIC	Negative	303.1603	3.97	unknown	104362	89653	132138	107443	44532	89210	62381	49625	39969	88563	90774	139229	120811	75231	72351	116399	69390	71262	70645
EIC	Negative	257.1760	2.24	unknown	1564549	368180	1292471	1798952	429643	709692	398472	810180	562294	1791161	506524	846140	776535	923223	646191	713575	589429	1432833	693656
EIC	Negative	329.2332	3.49	unknown	226734	139174	238622	172562	101968	155521	85682	183473	124078	307946	158650	167480	117703	108660	106503	195020	245550	221188	143083
EIC	Negative	427.1797	2.67	unknown	728764	637222	634241	579484	291223	349689	606961	450751	491655	546728	564797	817882	773192	661533	520918	605377	702269	719718	520356
EIC	Negative	643.3461	3.20	unknown	262756	291859	152444	170190	142548	281946	153647	163167	291357	256913	232868	325956	230804	112246	214048	383892	331792	122649	394728
fpHILIC	Positive	106.0490	2.46	unknown	419296	571463	404759	195812	350322	474472	279925	248259	291625	256313	300836	466754	318444	696932	490003	395723	389603	481912	548919
EIC	Negative	468.7190	0.52	unknown	745899	385895	758767	803085	347416	375852	449769	251495	502911	987724	377475	331906	450622	131738	353067	635813	311172	574372	829594
EIC	Negative	352.2214	1.83	unknown	363376	112913	298013	0	37831	63264	76626	78146	59761	0	109954	151121	108408	110759	91015	121540	110096	0	120219
fpHILIC	Positive	409.1855	2.61	unknown	7572457	10573501	7453878	3080569	6604584	6504635	7994075	4179901	4914646	4990663	10082755	7510446	7200840	10128518	13139208	7973568	5890716	7244097	8253022
EIC	Negative	336.2265	3.16	unknown	187022	96601	172761	153666	73521	68142	84993	100275	77656	131434	78050	128698	107651	83027	69455	82543	148016	192669	76910
EIC	Negative	319.1910	3.21	unknown	189261	375704	208860	410512	158207	94137	119661	177631	109864	143014	98632	173900	137355	124899	271261	223873	134943	161781	137535
fpHILIC	Positive	247.1072	1.07	unknown	3678982	1964889	3049326	3395347	1913109	1887104	2158799	2575631	1363304	2396630	2219373	2101574	1847442	2607508	3592381	3293257	1834517	3470760	1760734
EIC	Negative	543.2982	2.55	unknown	749116	0	1463705	505375	0	51201	0	274398	6466	417504	0	0	0	0	0	0	0	468071	0
EIC	Negative	517.2963	2.91	unknown	1276126	537385	1652974	3522350	750194	669591	1102733	1562259	1149360	3219416	1206830	1251708	1594046	184748	841317	894485	1701302	3545768	395934
fpHILIC	Negative	89.0438	3.35	unknown	479658	492653	352118	306736	326571	424116	344291	270913	365998	334191	421951	451152	409652	532011	636981	495192	406088	517823	385825
EIC	Negative	414.1981	2.67	unknown	322538	309676	279129	266848	146420	183126	304735	151276	252551	241540	257407	381513	392683	390348	253088	306714	294475	344764	265428
EIC	Negative	261.0593	1.77	unknown	42879	130846	21906	34933	42966	44634	90428	49244	7711	18916	146770	159984	82733	148384	122104	74911	46881	0	87796
EIC	Negative	367.0876	0.70	unknown	36411	115449	97530	94033	54989	57844	76392	125575	47504	51416	100412	99639	99465	31341	89263	103315	100852	211487	6567
fpHILIC	Positive	133.0971	4.83	unknown	12483398	9398154	9165286	8061620	8065170	9748197	9695656	7632147	9934539	9545827	7963622	8514269	9321136	10709811	17390548	9987741	14781347	11305517	10272433
EIC	Negative	429.1957	2.89	unknown	253515	223888	186586	205376	129779	71653	335981	184690	268752	300698	249864	334892	384319	249558	214164	200416	255680	422727	308877
fpHILIC	Positive	183.0841	2.95	unknown	22742172	16587837	21762190	13853333	12287987	13711382	10499830	12643647	12094608	16948474	11081804	11509106	11353022	18864368	21428618	12958029	14874262	21421134	15959496
fpHILIC	Positive	102.0915	5.23	unknown	866490	1395501	1357173	934437	1077248	1372437	1330677	670513	925565	1081922	1518557	1973675	1420149	1539061	2072647	1717164	1179254	1114999	1526465
fpHILIC	Positive	222.1223	1.65	unknown	0	725552	309811	343343	335424	0	459071	213914	527658	337688	493082	481083	409412	406865	615135	620702	325012	300956	313108
EIC	Negative	413.2001	4.28	unknown	720828	166188	923488	451360	91385	76973	173984	237451	123608	847562	261838	276523	276226	209912	119248	179170	256374	244921	196442
fpHILIC	Positive	110.0714	3.32	unknown	10838697	4769521	11115893	6820697	3533283	4264527	4645719	5258302	3514273	9238358	5331017	5140220	4889230	5498199	6619391	4826844	4145191	8554849	5908110
fpHILIC	Positive	197.1014	3.14	unknown	915499	915747	1461463	810586	522782	763884	890684	763267	580652	1154938	744893	816682	801469	1032068	971033	816764	871170	1238195	1093367
EIC	Negative	596.3579	3.57	unknown	2333951	1586738	1742715	1045964	781425	1985871	1060526	1388542	1212662	2035710	1354572	2413114	1395067	826413	1638287	2173704	2054710	977052	2276948
EIC	Negative	331.1919	2.17	unknown	5806725	3548670	6167305	5527962	1771378	1688993	2757176	2079294	2102637	4649414	2367065	3497345	2541513	2334110	2269066	3626898	2487647	3225050	3159042
EIC	Negative	537.2708	1.89	unknown	635408	681110	0	0	458415	282730	672808	0	461596	0	732586	728511	751851	552704	526240	904756	778238	0	454228
EIC	Negative	289.1812	3.39	unknown	211301	144529	223560	175075	71492	145882	104701	97888	81865	123790	116553	168942	175779	108820	164447	167260	137107	128816	115096
EIC	Negative	411.2388	1.78	unknown	542514	222960	399460	57524	63424	158110	147541	73032	115034	407211	202546	260356	180405	192069	126300	240347	196059	0	229058
EIC	Negative	502.1971	0.69	unknown	111316	111649	89376	53504	58355	67259	81573	47555	44296	83497	83612	96247	94040	107701	74063	69563	56443	75470	149673
EIC	Negative	336.2264	3.02	unknown	753946	257382	748140	472968	127042	215545	252248	245735	233852	646710	237003	280114	325182	210387	213773	279292	259985	400747	374215
EIC	Negative	594.3427	2.86	unknown	577461	645419	585029	380129	418631	897919	540119	494677	695573	710817	807582	978927	584068	647076	795170	1032281	861398	400370	1244266
EIC	Negative	463.3062	6.01	unknown	0	0	0	2276891	0	116772	0	1054102	0	0	0	0	0	0	0	0	1665885	0	0
EIC	Negative	343.1545	2.95	unknown	428942	262816	311257	69454	133669	47800	61665	109663	37935	80410	96502	154013	106682	119646	125329	126453	98378	108386	97540
EIC	Negative	276.1688	2.55	unknown	269436	109598	232962	183207	75776	77583	112154	134754	76389	217892	131890	165491	180558	107743	127872	124172	152720	150540	123580
EIC	Negative	281.2130	4.33	unknown	526682	236509	543167	291061	115402	301459	279495	232528	259373	307623	243476	493060	429505	352016	215342	284721	399362	454689	338975
EIC	Negative	398.2029	3.61	unknown	287940	251988	468381	305632	102105	155567	263084	187612	148301	240847	278459	377490	409747	262221	136980	256154	263658	328694	235266
EIC	Negative	357.2300	3.90	unknown	1027160	198531	1445693	470694	170785	310999	206505	551995	18653	733936	167329	362033	190500	270518	227775	90357	224451	903373	348121
fpHILIC	Negative	163.0401	0.51	unknown	8831344	5103199	5444973	2644200	3088506	5997099	3895148	2972560	3746668	5644854	6035277	5067003	4273355	11513563	9757940	2706443	4398907	4963466	6249614
EIC	Negative	382.2084	5.65	unknown	707192	541614	952056	581926	513603	515448	528440	595149	559184	691038	657065	594597	769787	547943	502778	777950	779616	954761	850544
fpHILIC	Positive	115.0868	4.83	unknown	1633019	1152738	1233274	1005982	988292	1211371	1246205	923530	1287439	1274004	995633	1100451	1213317	1450919	2134985	1320897	1784883	1436607	1388230
fpHILIC	Positive	86.0969	2.30	unknown	12770517	14896505	8842845	5765116	8641168	14646875	10744476	3413809	9835978	8724543	12327525	12619575	9871074	17218924	28626466	10625298	8230108	6227865	13769380
fpHILIC	Positive	458.1667	3.33	unknown	917312	968517	831437	713801	724447	606728	555960	556642	539958	869108	756456	718751	849036	820833	968868	730722	695545	780440	1083289
fpHILIC	Negative	445.3321	0.37	unknown	1503128	1251430	1251028	1349966	920449	721390	968096	853954	817942	1437046	1164963	1004991	838245	1033372	1290721	1646877	1026337	1044325	1104164
EIC	Negative	314.1226	1.69	unknown	223187	107155	50248	182123	83208	63721	110822	147305	81963	154704	116156	130475	127010	87458	86352	165066	121112	238474	84274
EIC	Negative	391.1749	2.92	unknown	725011	406640	0	383358	150918	322034	171313	464796	107196	780097	281501	360495	190754	0	457124	132232	203914	571039	373360
EIC	Negative	381.2050	5.65	unknown	3119871	2316391	3821441	2342159	2119942	2017779	2250052	2494335	2388913	2724073	2581323	2504584	3122620	2355650	2208723	3191726	3078502	4080373	3555076
EIC	Negative	320.2308	4.31	unknown	1012859	245808	1092205	737791	211238	187503	244179	406208	170641	1021588	323992	399045	396684	292978	251097	271676	354138	587831	284422
EIC	Negative	336.2266	4.30	unknown	188558	28953	171540	151322	31488	0	72778	152157	0	193064	31424	0	0	0	35152	114989	0	217703	0
EIC	Negative	642.3422	3.27	unknown	773318	853075	427690	539173	403786	861623	431862	464210	866711	707539	704223	993694	701061	347190	644488	1109904	1063286	355654	1170799
EIC	Negative	305.1748	2.22	unknown	2510945	1501199	2189073	1983567	533991	823966	1182470	971551	814860	1606321	923757	1888119	1697723	1141832	1183378	1406884	1147326	1431859	1042771
EIC	Negative	595.3463	3.10	unknown	1615197	1126521	1347009	853369	561520	1411657	708188	1089915	1038757	1352891	1161000	1701659	1051454	868542	1100565	1446161	1376629	807566	2176477
EIC	Negative	428.9044	0.55	unknown	420519	73578	155927	197290	83619	246045	86961	15859	101456	237622	102321	138863	113226	56079	121038	139879	80202	132056	293701
EIC	Negative	325.2016	4.33	unknown	331496	159692	420801	228326	65533	201672	202289	159282	199744	213937	229577	355025	353689	273789	150228	205018	268571	351187	215483
fpHILIC	Positive	170.0592	2.61	unknown	346348	428939	454384	254033	355106	434925	439981	242661	279061	270893	470792	417797	392258	493544	559044	619780	416324	444797	477138
EIC	Negative	444.6736	0.52	unknown	811131	178932	1002881	599417	358944	418887	236692	47477	531782	1358728	180549	185430	300117	193116	218995	417665	129058	422056	1102574

EIC	Negative	352.2213	1.78	unknown	410375	170801	286131	44778	57093	99835	99392	78939	91000	398460	159509	194626	151613	159441	102916	172834	159205	0	177609
fpHILIC	Negative	465.3042	0.34	unknown	14548842	11850517	14316771	11402863	11475429	7275297	6942948	10185777	10834840	13573942	10208086	11330406	8703099	15284959	14756198	13506718	11039815	15458611	12425493
EIC	Negative	463.2080	5.66	unknown	460895	345292	578519	381587	312647	281976	329209	410719	356394	475532	367988	373216	489081	356290	332723	488899	473574	609305	527691
EIC	Negative	315.1967	3.62	unknown	15516278	10734086	27972466	16676917	5081582	7956736	13260849	9693171	6196153	13308006	13466495	18730269	21136405	13506136	6481261	12377548	13476448	16308586	12096468
fpHILIC	Positive	231.1331	2.79	unknown	6995822	7139894	6000397	4038578	4174322	6112032	3482097	3126848	3512357	4978355	5412944	5356872	4092707	6949891	8317959	4264395	4658766	5999985	6143332
EIC	Negative	286.0891	0.70	unknown	25689	75625	60302	44839	33809	36558	49976	72681	33482	31546	60507	62112	64658	25535	59863	66860	60982	109266	29192
EIC	Negative	594.3426	3.10	unknown	5224935	3541869	4326624	2699506	1712083	4581376	2189480	3350364	3336905	4304860	3701640	5434082	3241717	2757471	3671168	4624803	4257462	2506334	6800082
fpHILIC	Negative	217.0176	0.40	unknown	652503	801612	366897	0	302648	785208	0	314058	218401	535238	477822	652971	0	1146081	988383	129113	239445	266330	712443
EIC	Negative	297.2074	5.11	unknown	558749	216571	597372	369702	127555	192750	286786	287941	276296	302245	261566	470741	358367	321082	216178	230909	379457	598956	263703
EIC	Negative	631.2416	2.38	unknown	184619	111711	295653	385291	103546	55128	150738	122998	136490	166037	137971	144863	180363	84518	85271	173347	114616	350728	97096
fpHILIC	Positive	91.0588	3.33	unknown	1835877	2186132	1479488	1279489	1265591	1718722	1312747	1304319	1335974	1743952	1901452	1840395	1888632	2238586	2673734	1952959	1643164	1877655	1872018
EIC	Negative	396.2463	3.32	unknown	518991	239040	607323	724308	155062	156125	250956	311996	216731	394397	252706	343142	346864	244828	228983	318738	361614	434452	231814
EIC	Negative	316.1998	3.62	unknown	3431782	2427397	6235827	3593001	1087996	1728641	2961773	2149572	1338547	2900469	2982196	4012671	4621015	3026159	1476526	2755337	2948390	3569426	2635276
EIC	Negative	257.1760	3.23	unknown	826728	441523	993379	824818	273785	349277	453799	453414	280047	634121	429311	736009	764572	472715	505549	562430	472949	641704	433051
EIC	Negative	362.1978	4.14	unknown	401402	319914	532501	336020	294599	212483	266245	232681	213218	255348	261505	234218	256567	213395	252937	378139	411013	473429	246279
EIC	Negative	262.0687	0.56	unknown	103397	88550	117842	78211	56543	62160	60240	66966	66149	92195	57736	82651	67433	53686	87725	74859	76762	128118	83575
fpHILIC	Positive	299.2002	0.38	unknown	485363	441055	781684	529837	215809	221928	414964	226973	328905	312338	426673	582933	483885	314062	291061	407687	462501	359730	515801
fpHILIC	Negative	245.0931	1.07	unknown	5153748	3281992	4144338	5108986	2877553	2911032	3388431	4017551	2158194	3823472	3468141	3175663	2664779	3624911	5685609	5368348	2998158	5243213	2906986
EIC	Negative	383.1477	3.52	unknown	1030221	1006686	1226322	1022871	315734	532519	628800	467052	474711	557844	802828	1303889	1102279	640262	778413	870436	605998	625073	618861
fpHILIC	Negative	164.0386	2.96	unknown	2165568	3138649	2306873	918782	1794910	2576285	1734137	1709798	2343038	1729770	2225330	3035701	1748150	5628061	3638523	1794666	2536740	2349137	4376514
fpHILIC	Positive	149.0265	2.97	unknown	750258	640967	406668	198790	388524	538667	399919	397455	435645	279164	434681	470666	381793	1299216	997770	279836	504853	489607	854672
fpHILIC	Positive	166.0526	2.98	unknown	24710502	22171280	16290550	7041184	13576360	21062820	12701570	11101663	16263117	12703321	13518002	19487796	11918234	40846680	29325310	12631277	18923036	17868500	29430894
fpHILIC	Positive	90.0554	3.33	unknown	55668472	67962784	44409136	39852328	38119412	53567964	38800588	37774268	37454852	49223032	57441928	56729648	51167140	62142264	82118528	55359716	47366012	56257200	56862368
EIC	Negative	364.2133	2.78	unknown	258400	134657	257947	80664	121434	150092	147862	199546	189128	186213	155335	164827	142649	141739	107848	226463	323663	330855	182494
EIC	Negative	348.1904	2.46	unknown	822874	415115	963550	592756	233094	164423	410225	384185	237308	521448	305043	471700	412752	335253	422879	424413	339737	564541	320957
fpHILIC	Positive	105.0563	2.46	unknown	561395	538489	454267	178600	268760	427825	258762	236627	300707	277207	412039	362660	339749	666349	469256	339275	311553	393506	652415
EIC	Negative	297.1863	5.49	unknown	1639192	651412	1960740	936956	768075	491758	735988	591468	613741	897329	921081	1028552	1278686	781082	734887	1256446	367992	1018710	1270128
fpHILIC	Positive	120.0900	2.70	unknown	667122	1087283	793044	582611	679726	829902	764536	531542	583458	842571	1044057	939742	794772	1159799	1414387	853849	823117	689571	1143711
EIC	Negative	557.3835	4.07	unknown	62083	34063	129986	251634	44520	21199	61780	26564	70915	87383	57021	66558	76684	0	0	84280	73410	137914	31558
fpHILIC	Negative	577.3083	0.80	unknown	227677	216794	235054	109301	198573	173817	265877	176453	139318	148376	228165	223979	137484	187434	214357	0	259020	668386	245735
EIC	Negative	277.1807	2.33	unknown	0	90144	171252	286583	33319	62463	44622	58727	36228	147948	140024	279465	95635	65078	82995	82960	92173	22090	62279
fpHILIC	Positive	134.1059	2.30	unknown	818616	799555	661071	568659	724140	843094	862895	172148	613866	481687	772636	777282	713335	972586	1809451	654824	581849	510671	1124123
EIC	Negative	406.1955	2.31	unknown	216505	415627	227722	305733	187521	250003	351946	243126	279621	285568	371939	572322	540403	417271	430838	375055	411017	362996	330510
fpHILIC	Positive	394.0711	2.97	unknown	295047	332601	525669	273880	207978	132529	179533	301293	262853	389917	263414	231592	218802	298180	342519	208691	323011	407007	298390
EIC	Negative	359.2213	5.65	unknown	838855	595570	1000319	611420	489504	516442	580824	574349	521999	645974	580112	548538	707656	496513	505020	707755	748611	1114743	834100
EIC	Negative	381.1467	2.31	unknown	963304	631784	837594	695884	222237	361851	474833	423150	426559	613486	522871	839167	688797	576875	625857	529294	565907	545991	514539
EIC	Negative	508.2275	1.33	unknown	1210299	543161	296458	222513	435102	433198	581492	0	323247	755544	399158	570936	525520	543886	533988	735141	818858	0	497756
EIC	Negative	371.1996	3.32	unknown	146145	62381	170887	238185	43064	52164	70404	97668	65512	128702	67803	85604	87259	59528	61470	124945	106439	128946	57874
EIC	Negative	243.1601	3.46	unknown	57177	84746	84114	42542	78512	81271	47085	60758	42320	70940	52621	74027	62779	55423	70920	100885	50340	133901	96171
EIC	Negative	411.1844	3.62	unknown	551267	418177	1032179	640888	182252	325027	487505	346086	261492	430982	498285	753417	843227	549684	261224	479742	512663	626552	458517
fpHILIC	Negative	74.0247	3.17	unknown	534545	926038	606094	470782	523326	689233	624266	510213	580800	575261	1224981	1177789	964115	1118443	1038094	975272	758283	606515	900563
fpHILIC	Negative	218.1029	1.64	unknown	1179071	36099700	17843038	18025330	17279318	0	23934602	13328595	29917450	18858536	26152116	26157310	23282960	23829958	31139520	33670852	21244700	18128276	19267592
EIC	Negative	266.8039	0.52	unknown	2302886	561549	1794888	1195783	962931	935918	645241	468077	1095886	3391659	557943	856764	638581	1435559	731918	1044473	462141	871394	2895362
EIC	Negative	316.1633	3.49	unknown	58780	59565	72715	68667	24604	18970	24472	22222	13995	33035	30045	43088	31606	7454	36517	56508	26644	11855	20970
EIC	Negative	343.0446	0.69	unknown	67109	138899	141058	102341	73802	74868	88020	111610	70163	84761	113240	117228	132033	63606	113159	127155	120957	181292	69669
EIC	Negative	502.2845	4.93	unknown	3254720	6222376	3887451	0	6300551	0	5610891	0	0	0	4574587	0	3176987	0	4987060	0	4940611	3080177	4419638
EIC	Negative	253.1444	2.67	unknown	4562135	7624035	4301495	5552380	4985860	534470	5131374	2978629	2399058	4057136	4220684	4483646	1367463	6233072	6300538	8769911	917664	3686337	3781391
fpHILIC	Negative	580.3256	0.60	unknown	57012828	62773876	65509536	48844288	0	0	58530432	54542788	44722756	48791196	63411580	0	54350132	67691784	52455724	0	53575556	51561640	73346264
EIC	Negative	507.2242	1.62	unknown	1989171	835140	1574570	603177	558131	474149	1101314	425782	644779	1564403	776270	1115850	1281975	1160885	1078707	1049679	1353685	398916	809734
EIC	Negative	386.7154	0.52	unknown	955335	228156	1198793	621736	421688	419436	268239	125301	562200	1451699	257786	217909	347874	74239	260275	452616	206805	485089	1226215
EIC	Negative	367.2128	2.25	unknown	250420	132687	198389	247586	54909	92310	95307	92044	79983	244042	93216	132276	118089	100369	98493	138251	116302	140578	113411
EIC	Negative	425.1638	3.12	unknown	143901	152416	97425	132820	60980	154490	90371	34318	79633	105119	157742	209763	217289	153284	172073	123052	141496	45126	133226
fpHILIC	Negative	466.3076	0.34	unknown	4505396	3539885	4029752	3451057	3467426	2147170	2157206	3310778	3230262	4381844	3170879	3635722	2571848	4655995	4498205	4216517	3477909	5039993	3749312
EIC	Negative	594.3423	2.36	unknown	4396155	2495863	3457115	2472054	1197985	3247447	1592858	3061532	2226664	4240238	2666591	4068500	2195526	1791104	2486315	3438297	3241481	2511843	4929907
fpHILIC	Positive	102.0551	2.46	unknown	1453309	1761344	1320361	648410	766885	1321585	815944	760676	898462	995966	1163713	1233069	899509	2093335	1634187	1035118	1048982	1165071	1813880
fpHILIC	Positive	329.1499	3.65	unknown	2153107	687381	1279091	1951816	467076	845422	564824	1186621	377818	1231647	629205	669553	674023	395796	679695	834292	791445	2137155	616041
EIC	Negative	334.2108	2.90	unknown	2653750	1455547	2512452	1642660	940978	607201	2492452	1415577	1716378	2369939	1980071	2664567	3140941	1740425	1547949	1716958	2055658	3084209	2140241
fpHILIC	Positive	245.0806	3.19	unknown	1268274	1923581	1009999	1021564	1170940	1643651	1007429	878046	1223630	1031512	1556604	2006892	1707204	2128273	2065232	1956849	1567426	1269184	1870732
EIC	Negative	492.2743	3.04	unknown	601482	245109	397116	247198	109227	326851	132517	299103	146950	415808	204409	276025	154456	215704	290640	173927	300229	261449	469230
EIC	Negative	396.1925	5.65	unknown	406943	332654	537284	329627	242015	274449	324096	323953	271288	356424	323867	284056	391863	295496	253852	440386	449063	535792	438027
EIC	Negative	507.2241	1.32	unknown	4032574	1881707	1224976	779479	1440555	1482206	2031811	0	1099427	2453157	1338197	1960554	1803888	1867139	1864348	2585466	2833687	0	1648782
fpHILIC	Positive	188.1388	5.16	unknown	250493	689983	517539	343209	303066	437260	556598	247712	338641	480996	562661	533309	591977	649081	656219	508222	524245	472292	620681
EIC	Negative	356.1439	1.62	unknown	316979	262112	149961	0	109386	203344	114676	203207	83788	203092	162040	173234	100496	393162	250588	88668	164566	314394	237057
EIC	Negative	477.2868	3.03	unknown	95314	205934	108510	198618	58729	144075	224316	78071	139272	194656	161738	457117	370157	263874	303403	213704	163830	44354	227123
fpHILIC	Negative	407.1725	2.61	unknown	724967	1197928	842826	315676	596153	667669	1061063	473562	509353	653689	913446	846905	726292	1350706	1656255	788195	721773	745240	977748
EIC	Negative	271.2280	5.65	unknown	449270	414479	526866	459291	260663	213049	299734	289102	279609	280360	315272	506178	492755	343253	500146	442859	346291	382938	312492
EIC	Negative	410.2218	1.73	unknown	88841	178182	146831	539256	19923	21675	67355	26201	63106	1138953	131455	147983	87965	48903	7675	142777	50296	0	92677
fpHILIC	Positive	246.1222	2.61	unknown	463587	443930	365940	245823	262206	318691	415839	223211	413539	378164	477357	323366	316928	493965	624842	476821	452742	432941	430602
EIC	Negative	334.2110	3.68	unknown	334273	250154	385710	450790	156260	95864	176004	144592	84087	334806	167796	110641	194859	83385	151700	230971	128435	283744	177580
EIC	Negative	437.1418	1.62	unknown	64827	93826	0	0	48917	72151	40453	0	31617	41731	68408	61241	44576	146486	96959	33691	64370	0	91154
fpHILIC	Negative	206.0821	0.58	unknown	12525871	8060355	8210244	4715988	4585390	9264429	5114000	3819825	5210478	7198016	8082937	8408434	6077119	10968769	13092097	7394738	5912172	7103193	7983188
EIC	Negative	283.1921	4.44	unknown	618009	291638	697306	444629	238409	240547	378243	351075	280683	370787	356273	548824	542043	402246	313837	341556	473571	766172	313090
fpHILIC	Negative	126.0560	0.94	unknown	463051	389033	412707	397261	232197	316572	348782	339619	278479	556110	491308	362151	286447	653830	666068	457980	264934	409738	451255
EIC	Negative	268.8010	0.52	unknown	3760739	943180	2998322	1986734	1520223	1461381	985794	865894	1712353	5478582	943839	785602	1095797	2232155	1186257	1723173	789648	1342476	4680033
EIC	Negative	227.0648	0.49	unknown	408977	280996	385498	261882	163458	218103	170527	162857	239618	355667	200144	232128	234597	102558	281500	295362	197639	308037	335261
EIC	Negative	288.0913	1.74	unknown	227462	141498	165706	0	91702	229712	118391	35849	38466	25884	160512	193843	114204	302379	232573	77998	164982	0	288062
fpHILIC	Negative	102.0561	3.66	unknown	746365	391691	407345	431412	298665	513999	300227	343128	357768	415268	303610	429943	346056	408420	433222	510138	529308	703683	518752
EIC	Negative	326.7596	0.52	unknown	1543748	454772	1165948	982257	527183	557833	540263	218887	977185	2353737	402979	403153	611079	585881	338989	605843	414303	790944	2042526
EIC	Negative	396.1872	5.65	unknown	389295	297828	0	300139	0	257767	301966	0	0	0	0	0	21973	287718	0	418821	413313	0	0

fpHILIC	Positive	292.1486	2.70	unknown	514994	531969	637623	616807	426861	570625	313299	414777	214155	508332	593064	492806	433985	593695	687518	632032	386310	598821	579934
EIC	Negative	300.2052	5.65	unknown	9028866	6472232	11325960	6888062	5543756	5622327	6286150	6757603	6190320	7114101	6851752	6560993	8667847	6004670	5667299	8582242	8796750	12325333	9843256
EIC	Negative	259.0647	2.14	unknown	355931	976075	486003	52522	499285	619385	578990	232175	326464	273311	810421	995907	438400	1890613	1255235	700073	486557	159954	754285
EIC	Negative	643.3461	3.27	unknown	292815	302343	171437	188460	135965	316919	152052	147520	309846	273448	245991	350743	257049	128475	234196	419304	386719	122860	418030
fpHILIC	Negative	91.0554	0.52	unknown	908954	659361	681430	148161	317428	676948	388124	203091	319885	571028	513185	631583	439426	1031649	936416	355927	423595	500623	620772
fpHILIC	Positive	393.1673	2.97	unknown	1719068	835989	1048659	529799	700050	676662	366105	1401733	832256	1335306	464577	548849	521528	2449447	1766739	469448	865862	1645948	1174000
EIC	Negative	351.2179	1.79	unknown	1820465	762702	1361496	327824	262302	470547	484061	283994	381961	1768292	721691	885972	671779	713155	456515	818516	705691	0	824248
EIC	Negative	306.1773	2.22	unknown	553690	302347	431178	407437	111934	165382	228178	199501	166944	320389	177704	380012	331767	237548	236793	293789	238777	318762	211559
EIC	Negative	299.2019	5.65	unknown	41033974	29618836	53336445	31703879	25321600	25479531	28692071	30931264	28097608	33083752	31380743	29855725	39288400	27435060	25907008	39845383	40019635	55914068	44831466
EIC	Negative	362.1978	3.76	unknown	522358	491968	1083930	498546	348285	230427	296151	233033	185961	286405	371890	321966	236120	219636	307203	437233	407635	558191	219450
EIC	Negative	405.1922	2.31	unknown	914962	1690966	818161	1296335	786579	1053914	1366230	725400	1286294	1276754	1506050	2375536	2091688	1712656	1796061	1503550	1677801	1421179	1385887
fpHILIC	Negative	246.0969	1.07	unknown	689175	433470	593247	721954	396705	380891	453890	521345	282323	538828	473091	435724	326538	543637	751548	700277	456074	748681	379892
EIC	Negative	316.1998	2.91	unknown	66505910	44173621	84795032	64701862	26376957	24018790	40899243	27085406	27744585	63389361	45382700	56570830	48826544	46707070	38361687	51501217	51276979	43852895	33158736
EIC	Negative	313.1815	3.38	unknown	425761	1519172	1078373	1158284	1094539	352025	285420	511521	413252	412384	511506	448742	419382	607211	1098443	1703214	633432	287762	245167
EIC	Negative	391.2134	2.05	unknown	6512031	3864239	7456786	4217550	2033329	2443378	2888960	1530501	1349595	4412341	2604045	2808485	2036997	2559908	2915848	4016836	2859463	2465237	3056613
fpHILIC	Positive	107.0534	3.61	unknown	684605	505476	565793	368711	366433	469232	388101	289257	423502	539202	452148	541622	451695	520962	819701	575921	489585	400106	609527
EIC	Negative	303.1518	3.52	unknown	299940	266024	357578	322069	88164	148421	184517	137857	118140	179863	201035	383207	321540	200002	217688	250109	178243	211110	156008
EIC	Negative	595.3458	2.36	unknown	1355594	800665	1146332	774377	377317	1035584	499401	927273	713214	1336646	815412	1279859	668993	552313	801331	1111285	1033419	799240	1575259
EIC	Negative	270.7981	0.52	unknown	2333292	630677	1961096	1251404	992306	995391	673044	559310	1072067	3457821	620108	483737	684221	1481895	695075	1132401	732100	853277	3017160
EIC	Negative	315.1967	2.91	unknown	305203592	202280964	384623799	293705531	119500671	109388359	186701809	122551384	125670487	289042175	209664515	257723055	223467150	211930706	175028041	234140018	235001105	199872724	150442920
EIC	Negative	311.1861	3.72	unknown	193297	116050	245235	131216	69311	109217	136347	77053	100953	113660	148204	246171	235699	164797	101915	163861	160378	162031	150409
EIC	Negative	516.2738	2.74	unknown	1050011	653133	951044	0	473000	965974	408927	0	683538	0	622669	726651	260825	547228	720540	798583	1207072	0	1071455
EIC	Negative	411.1799	2.18	unknown	421839	530271	401187	636884	205206	359962	413941	366061	535516	622713	208178	726521	296576	289951	139462	672978	322469	962087	725861
fpHILIC	Positive	243.1031	1.64	unknown	0	533090	243482	308614	234063	0	337695	161155	396123	255701	442557	418022	370040	326115	480110	486943	257288	236384	233981
fpHILIC	Positive	84.0814	5.24	unknown	5530722	7893586	7046519	4622084	5404844	6933182	5518727	3335459	4806953	5286650	7333780	9483593	7097356	7436557	10465010	8955888	6024316	5749705	7831831
EIC	Negative	392.2169	2.06	unknown	1532473	861306	1882299	1000150	415030	557125	652753	338377	366790	983593	579169	812824	627039	664945	700426	797677	669088	660813	597379
EIC	Negative	345.1711	2.31	unknown	21195100	36357254	17303411	28953113	15739835	23588875	30514941	16940022	25858273	28681537	31191636	50689996	44399947	36568531	38951015	32894403	35157678	32608117	29661510
EIC	Negative	376.2209	2.91	unknown	1858919	1197178	2173928	1830249	720538	710568	1092367	684149	745833	1793548	1201961	1562446	1329559	1228937	1010115	1426477	1335537	1287192	884226
EIC	Negative	347.1869	2.46	unknown	3498304	1840765	3977461	2606614	1001089	757358	1898344	1407464	1025080	2049097	1355934	2123242	1805297	1525502	1861747	1936795	1503983	2386131	1469204
fpHILIC	Negative	74.0247	3.61	unknown	3029175	1613786	1867527	2352099	1088728	1372526	1209708	1422778	1236570	1767249	1435116	1758791	1773623	1337098	1930581	1996211	1656943	2363011	1577833
fpHILIC	Negative	117.0751	2.70	unknown	1724916	1950396	1534267	932664	1310483	1811285	1438577	948197	1096109	1266878	1677615	1790398	1468654	2428779	3073864	1672748	1466996	1378657	2348411
EIC	Negative	285.0856	0.70	unknown	182926	469595	368253	327545	225805	227176	288010	456759	183614	203917	378655	404969	404419	155232	380734	416624	399754	727684	154683
EIC	Negative	435.2030	1.14	unknown	46565	58167	44638	90287	26743	32746	48984	19769	56000	31324	48071	75332	83666	38380	50841	71353	78249	27597	49352
EIC	Negative	356.1540	2.53	unknown	582576	305307	697990	482129	315749	417224	402774	471530	248568	717617	456320	553873	503907	618711	625412	512260	605816	566219	485351
EIC	Negative	315.1602	3.36	unknown	469816	601169	438873	465149	265245	276887	420401	319783	317124	276487	350084	635704	595721	430095	594084	635517	436108	382027	365188
fpHILIC	Negative	180.0336	0.56	unknown	507612	277313	454060	397529	340899	479406	374981	199059	429953	180182	367547	384563	525537	358825	304449	731341	461041	739846	515556
EIC	Negative	251.1269	2.75	unknown	96467	298478	43352	507956	242103	100867	76796	0	42835	133386	85750	146257	126097	69063	157713	98946	231596	103268	106501
EIC	Negative	346.1742	2.31	unknown	4601210	8149260	3665425	6571831	3488000	5218125	6786521	3688931	5653912	6624321	6919884	11163866	9896708	8136190	8526691	7298988	7782460	7208288	6661020
fpHILIC	Positive	346.1540	2.61	unknown	688486	623890	564233	258419	432826	451417	504150	285217	358161	435830	616085	658937	395867	692515	882832	615587	455457	515686	596991
EIC	Negative	334.2108	2.81	unknown	324377	244526	608054	241531	175527	201268	330212	189394	197519	627174	306131	314694	305931	268988	260770	279704	371207	396027	343911
EIC	Negative	311.1657	3.47	unknown	98669	111435	156674	169710	65442	86194	62774	33452	73317	55301	82022	115528	125236	83121	98540	122392	98092	70216	95911
EIC	Negative	388.7124	0.52	unknown	487399	145363	574361	296678	223558	274300	123652	28337	305161	774284	106907	95695	144450	226438	151295	295274	118171	226052	652886
fpHILIC	Negative	147.0493	3.66	unknown	1553566	844769	722765	841154	536450	1018369	494737	607622	646272	762194	483185	792032	624460	613185	837729	1009252	1048281	1276542	825610
EIC	Negative	594.3426	3.00	unknown	4230798	2922281	3168885	2143428	1242625	3882251	1640409	2630442	2668346	3858636	3010371	4516623	2665642	2059635	2921130	3992040	3631008	2109461	5600025
EIC	Negative	316.1999	2.26	unknown	0	270813	0	318806	299246	281928	0	0	0	0	0	353583	385843	146503	349417	0	322941	0	207307
fpHILIC	Negative	146.0459	3.67	unknown	28093212	13839049	12876397	14339232	9528889	17244656	9499015	11109562	11271459	14261254	8448241	14964627	11139966	11495973	15198157	17870262	18641996	22417784	14720120
fpHILIC	Positive	87.1003	2.31	unknown	757829	1049626	610001	231107	676812	719052	563930	0	525403	524285	616612	918562	665418	888540	1701738	648939	451634	177815	785302
EIC	Negative	395.1897	5.65	unknown	1489391	1109253	1983105	1106558	963683	969057	1110233	1160990	1151714	1360993	1263586	1282634	1626753	1065692	1017121	1568100	1612143	2094450	1833790
EIC	Negative	539.3046	4.31	unknown	277496	226853	502045	462970	268163	150819	392719	296605	361164	499453	273328	620129	467184	443320	384909	491127	361936	683316	308209
EIC	Negative	596.3517	3.10	unknown	433696	360840	446664	80000	184496	439650	285629	0	306224	366344	340275	471134	284188	355868	329912	486800	381712	87516	559217
EIC	Negative	256.1638	3.22	unknown	516761	388518	658671	359422	253551	236848	361612	350092	228509	346012	320056	491361	662172	377549	319955	489348	389711	543933	428268
EIC	Negative	301.1448	3.52	unknown	11312284	10218546	13342708	10941224	3153848	5686216	6599585	4576347	4660195	5964718	8054676	14565449	11995225	6978507	8239245	9326797	6977026	6676573	6246150
fpHILIC	Positive	131.0814	3.33	unknown	981818	1037365	878397	701456	704297	851008	747872	676252	839353	1043151	1135327	1189202	819191	1188661	1466015	1219643	899710	1062397	1155054
fpHILIC	Negative	146.1015	5.22	unknown	2242038	3240899	1698080	1537390	1549283	2451977	1840653	990458	1475004	1715174	2554668	2961051	2553768	2720106	3999276	2675739	1870195	1629893	2459140
fpHILIC	Positive	237.0866	2.07	unknown	967966	471122	305318	271085	441903	421135	478858	198859	512256	226960	354395	574320	393042	646329	1047205	429057	534156	454231	658430
EIC	Negative	317.2028	2.91	unknown	8824436	6667606	12173067	8586092	3603484	3151063	5434747	3890632	3696269	8824232	6093945	7344518	6873869	7085567	5434704	7205353	6813139	5771878	4918008
EIC	Negative	316.1998	4.16	unknown	460083	190818	673304	514311	98216	190103	209013	201058	174706	675593	309790	297193	324828	224573	104924	255325	311696	365534	294127
fpHILIC	Positive	229.1548	0.92	unknown	1872026	1269605	1275401	352268	1047368	1216340	370580	393719	534819	871184	999913	1294774	493001	1877823	2105558	706348	698356	797173	1129128
fpHILIC	Positive	145.0984	5.22	unknown	33563576	47677572	26613460	23410816	23319370	36342276	28587464	15174750	20931998	25096868	37902168	44480128	38716948	40641648	58625476	40045856	28608368	24943036	37675636
EIC	Negative	262.0447	0.69	unknown	37139	71629	67070	50454	38674	41284	43794	44121	36028	44869	55058	61877	68273	44832	64910	70941	57967	78416	39424
EIC	Negative	255.1604	3.22	unknown	3317438	2472172	4115988	2463858	1573573	1514905	2275462	2100826	1497324	2190839	2044121	3202842	4178682	2369757	1951161	3101196	2555701	3341453	2760854
fpHILIC	Negative	268.1033	3.34	unknown	2283826	2505332	1676069	1131466	1606897	1965474	1274450	971974	1248621	1578408	2039596	1975140	1840164	1961170	3378917	2122657	1757126	1791635	1763942
EIC	Negative	355.2287	6.29	unknown	1585105	775984	1194402	772342	553464	532669	631292	1066563	355279	911836	942123	779772	481162	745814	1309455	956223	699908	900951	938416
EIC	Negative	595.3461	3.00	unknown	1352845	953896	1088189	679858	390279	1215975	540352	810315	817069	1219310	938631	1469361	827415	677168	912583	1266238	1171640	662407	1763681
EIC	Negative	315.1602	2.19	unknown	944384	905660	1014021	1336934	302478	357836	583336	346337	376789	798774	379165	337959	696574	139275	421080	549829	243774	1057902	738662
fpHILIC	Positive	242.0998	1.64	unknown	81186	5043338	2167271	2838881	2396302	0	3449537	1544107	3965142	2271904	4076622	4029037	3254503	3448649	5011923	5095504	2449201	2408530	2422278
fpHILIC	Negative	340.0709	2.42	unknown	1034810	1447739	817937	486929	585116	976603	619173	657776	726833	625985	759899	1160555	698952	1187985	913246	1417786	712173	1231782	1192873
EIC	Negative	348.1906	2.17	unknown	331694	262933	289977	278445	77904	138084	141281	117575	107549	274656	155895	244340	157261	178115	123618	197408	143721	250573	178046
EIC	Negative	331.1913	1.72	unknown	789981	397132	1092476	855789	252972	303997	634200	346718	279801	561274	427755	668229	893220	643909	308012	588185	558224	706958	629495
EIC	Negative	391.2134	2.06	unknown	6097010	3483927	7383293	3939019	1797317	1986655	2457775	1221514	1423841	4174537	2307819	3256936	2525315	2669505	2660208	3331922	2709953	2326317	2620512
EIC	Negative	477.2598	2.47	unknown	310731	130326	243023	380809	101436	111802	101297	228435	107405	308319	103649	138378	154948	167858	124461	174271	153007	435989	112577
EIC	Negative	331.1913	4.23	unknown	1083494	339263	1064987	572159	246285	191081	468936	316151	369422	528991	336460	438040	477150	341842	505182	397748	522751	678183	344719
EIC	Negative	313.1197	1.85	unknown	3205681	1665401	818677	0	1531781	898862	1498389	0	1198309	0	2144295	2282620	2183777	1331849	1254884	3171263	2149560	0	1320324
EIC	Negative	305.1749	2.10	unknown	1371770	814064	1454798	964837	321361	493763	663498	499761	349990	761974	603184	1355035	1227836	624454	739725	831661	740733	671370	602220
EIC	Negative	525.1792	1.91	unknown	0	274946	0	0	73555	100156	79125	0	82745	0	140165	113005	93589	98781	118168	142320	162923	0	93405
EIC	Negative	318.2156	4.28	unknown	5287338	1355744	6402502	3898191	819199	630896	1406841	1955418	895723	6178917	2247854	2261929	2377933	1806341	1120115	1501946	2254159	2091244	1823726
fpHILIC	Positive	152.0536	2.47	unknown	15683950	15725711	12987609	6355703	8345511	12998307	8548394	8211966	9254678	9003371	12452570	14024053	10782277	22088622	16846656	10396195	10911937	13212869	21590148
fpHILIC	Negative	315.2541	0.41	unknown	417586	309533	789956	820163	352279	684369	512268	377421	295930	351673	290222	533583	414641	406413	257426	822893	767730	1069159	506346
EIC	Negative	330.2368	2.30	unknown	487926	71849	282372	70591	84567	136388	94411	84097	82395	141360	55616	76551	90383	117252	62309	358282	81273	136319	104161

EIC	Negative	384.7182	0.52	unknown	959013	218263	1090168	641837	337434	506364	315798	112144	505036	1534107	258272	261375	317869	290531	303266	541474	170600	458417	1266033
EIC	Negative	397.1322	3.52	unknown	322527	320571	391214	318792	91709	168114	190256	133342	142099	175810	238484	433173	359800	197503	248228	284021	197772	197307	193049
EIC	Negative	327.2545	5.91	unknown	381162	187798	676872	419080	87727	192845	91837	150380	298550	168116	154412	397035	346255	170036	318341	246286	260628	341861	101233
fpHILIC	Negative	222.0772	1.66	unknown	0	3111345	4623249	5104309	1692394	2867389	1549584	3241383	1200621	5440330	1778183	1630095	1636285	3198443	4271205	2323171	2349419	5247539	2128823
EIC	Negative	328.7566	0.52	unknown	1250012	330969	916227	927489	431900	670591	422608	201112	516204	1906105	398309	313002	494896	496640	401991	482423	298728	629024	1656853
EIC	Negative	301.2082	5.65	unknown	1106013	804885	1400539	821287	647476	690456	755076	785038	731540	874311	842272	767671	1026436	665088	704001	1055519	1133711	1506620	1187288
EIC	Negative	478.2942	4.47	unknown	0	4271364	0	0	0	0	0	0	2260376	0	0	0	0	0	0	0	3499686	0	0
fpHILIC	Negative	88.0405	3.35	unknown	13390293	14754238	9503386	6698692	8741776	12033700	8270515	6776628	9334867	9056281	10723665	12722251	11135240	14702920	19016698	14435862	10645272	13999637	11852456
fpHILIC	Positive	168.0487	2.97	unknown	1067044	1106046	910003	359754	649975	974130	534156	432237	708462	640623	721075	833265	494337	1690789	1378713	688389	844491	834878	1366623
fpHILIC	Negative	345.1717	0.43	unknown	2099654	2163273	2234216	2380885	948853	1348540	1492750	985430	1443236	1400261	1771266	2799986	2968080	1825513	1945434	1821123	2196617	1897181	1791543
EIC	Negative	302.1481	3.52	unknown	2278188	1998559	2666157	2134726	616798	1114465	1277521	891626	897467	1187389	1570924	2885567	2338914	1358316	1635099	1843711	1368570	1344437	1255523
fpHILIC	Positive	121.0644	2.95	unknown	473385	375833	492411	327600	284472	305073	212667	295149	340067	354359	268421	318310	336041	314262	477988	340192	423462	637119	355358
fpHILIC	Negative	142.0510	1.66	unknown	0	997823	725531	614364	827316	0	834080	495814	438214	604805	1132045	1052293	747819	1186795	901503	1087300	624155	665775	756394
fpHILIC	Negative	219.1068	1.64	unknown	0	3796944	1845689	1909446	1612076	0	1989452	1467439	2951579	1899377	2897564	2815153	2239618	2326998	3101475	3566888	2125063	1830608	1865776
fpHILIC	Positive	133.1049	2.30	unknown	14245406	13383800	11020977	6252206	9255211	13513545	10759890	4267698	11059766	9043719	14171335	13076236	10877273	19419616	31219054	11916366	10041112	6989872	15919696
EIC	Negative	585.4164	3.76	unknown	63772	29998	176983	119789	0	29859	35851	46830	10724	151383	31571	36010	18281	16931	11003	19528	36436	66550	58539
fpHILIC	Positive	308.1141	3.18	unknown	634696	751802	477982	472165	439470	550557	445950	398054	499465	472801	722749	788638	709297	856165	910745	684612	639607	512355	891034
EIC	Negative	642.3422	3.02	unknown	814548	1464270	547539	852465	635820	1243727	722609	511826	1203362	979063	1264546	1560115	1335864	858266	1152663	1831848	1457373	568432	1724746
EIC	Negative	236.0651	0.47	unknown	114965	65023	84728	64274	32343	60746	32894	31129	57963	99871	35874	76660	50250	60133	73596	66261	34522	86848	74396
EIC	Negative	275.1656	2.55	unknown	1182164	632953	1081043	945793	346959	348115	558568	569557	336405	837606	669531	875413	901244	548800	634896	644087	759346	665609	666527
EIC	Negative	313.1197	1.69	unknown	763626	556394	506252	1051816	454877	324536	481955	607134	385764	864601	594925	691711	633959	397156	407140	831366	646459	1257816	384082
EIC	Negative	495.1973	2.24	unknown	414634	104062	367900	316771	46279	29693	71804	194013	65408	421673	47115	95685	59863	75685	59703	83220	68215	344922	81105
EIC	Negative	329.1761	3.30	unknown	768123	438561	1092974	730888	217200	351057	424648	377811	244589	596800	562275	760995	898215	509174	379681	497580	557115	618045	475506
EIC	Negative	562.3144	2.30	unknown	180211	0	1168110	923113	0	0	0	493577	0	701554	0	0	0	0	0	0	0	773014	0
EIC	Negative	397.2001	4.03	unknown	1020648	477328	1745215	883345	317223	405492	623779	548890	548292	759903	606487	644609	863141	562003	299619	673380	712918	823387	1323938
fpHILIC	Positive	151.0615	2.46	unknown	16125750	17418898	13467339	7027089	8575195	14006917	9295621	8945233	9506837	9871456	12553959	14714678	11341183	24229104	18735154	11448090	12777681	14921122	21266850
EIC	Negative	379.1946	2.07	unknown	431509	268360	632805	1681378	65540	75651	140655	61925	112912	898396	402994	107998	0	0	130849	256643	33432	230485	66773
EIC	Negative	446.6708	0.52	unknown	557003	101725	720351	336311	208275	256926	146125	0	310873	875985	130657	110863	166540	93404	98522	278033	95700	224712	754416
EIC	Negative	512.2689	1.26	unknown	1021202	33913	9189348	5384922	88752	53184	45588	9816737	128044	840795	89858	50476	57084	137340	133170	75450	74770	15592876	80533
EIC	Negative	313.1196	1.84	unknown	3861145	2062850	983843	84318	1858301	435828	1773845	553342	1426044	304197	2592231	2774187	2552387	471000	1504715	3862742	2624944	0	1532898
fpHILIC	Negative	206.0821	0.74	unknown	1721701	714152	952486	700048	492412	969215	511573	538528	552975	937179	788548	932254	621046	1291936	1723331	889013	643899	939084	635450
EIC	Negative	367.2439	6.39	unknown	5150994	2400854	6449790	0	1592782	3645664	1707454	503106	0	3973069	3146991	2707873	1336298	3626454	3317328	1873800	3306120	1666877	1577708
EIC	Negative	347.1869	2.17	unknown	1453395	1176755	1261366	1151945	302601	577889	624913	421916	448976	1210717	643558	1015113	711199	690165	502809	816167	588348	1032585	806194
fpHILIC	Positive	117.0659	3.12	unknown	1968669	1711819	2265795	2977909	1159329	1578295	1911683	1740821	1278644	2569811	1759956	1575659	2132812	1712391	1580207	2621538	2615456	2995290	2281800
fpHILIC	Positive	135.0273	2.46	unknown	1922248	1987569	1596364	785665	988385	1394121	987891	946862	1138559	1121061	1491411	1680150	1141440	2751645	2062520	1202174	1225706	1529703	2590013
EIC	Negative	507.2243	0.91	unknown	103190	51870	89491	35799	18691	23408	67104	27771	43082	69193	37072	64918	49368	49275	50279	67986	80086	39698	46111
fpHILIC	Negative	207.0858	0.58	unknown	1507053	850993	996491	522613	536907	1275137	612687	342708	665284	860324	1029682	995565	833439	1384349	1613703	1026875	655888	1192467	931130
fpHILIC	Positive	150.0481	2.46	unknown	2425476	2267209	1987422	963678	971993	2007156	1452256	1001336	1435995	1257171	1836320	2222120	1590581	3558360	2677564	1621701	1592966	2167538	2786830
EIC	Negative	336.2266	2.06	unknown	677170	233131	779399	397172	100827	141391	167804	218606	148329	903821	232364	254769	237171	323674	193213	214659	338237	167966	314222
EIC	Negative	492.2743	3.15	unknown	614278	273631	465153	245470	119956	388449	127511	292203	184998	420997	235737	329607	184703	282096	346189	191430	357641	315228	549582
fpHILIC	Positive	388.1803	3.54	unknown	126163	2601891	1443698	173542	251288	235895	926436	413855	1729658	151371	47983	241347	202081	1957953	192240	153611	523468	216246	4542514
EIC	Negative	258.1799	3.23	unknown	121400	74654	151697	136413	37923	51506	74692	61808	44396	97646	62071	114104	115067	63703	91580	92438	75632	102638	66154
EIC	Negative	362.2373	3.50	unknown	575771	0	481185	0	0	0	345732	553806	0	0	388721	494553	614576	44842	0	485504	0	743861	196341
EIC	Negative	643.3459	3.01	unknown	277861	517997	172937	277795	221147	441885	232914	199804	417778	356878	440759	550650	475563	314878	415401	635893	528934	224100	594626
EIC	Negative	491.3381	3.82	unknown	715531	576043	816682	326013	348329	403092	402033	416248	280969	935012	614759	758470	593278	867103	717923	565991	569520	719188	361476
EIC	Negative	261.0411	0.69	unknown	309284	575262	513350	343379	284211	302763	330916	323257	247546	370062	438139	446769	488095	344020	472235	553806	421492	596097	316935
EIC	Negative	263.0378	0.69	unknown	102424	180622	160702	109943	89386	97649	109419	100349	85298	114141	142634	142508	167080	111720	157748	180912	132080	190896	102914
fpHILIC	Positive	133.0314	2.46	unknown	41567892	43026852	34255160	16582389	21207120	34208232	23639334	20236146	23181256	23986258	32374096	37427668	28741206	59253740	45145188	26905014	28887524	34938064	58540068
EIC	Negative	327.1605	4.23	unknown	527448	725410	612282	764851	276720	278367	337410	300122	229683	264606	263695	550366	457865	326514	502786	605971	369084	428938	351252
EIC	Negative	343.1541	2.45	unknown	896165	479164	848742	125171	269775	587940	490086	403449	42780	518438	458311	678550	734312	454638	585379	490021	472247	767950	539479
EIC	Negative	299.2235	5.01	unknown	399964	310987	451102	328887	163675	174312	232887	158059	201731	248977	322718	469928	492088	332567	269488	365898	305250	260077	281256
EIC	Negative	631.4007	3.76	unknown	643538	203468	1076108	327993	85798	159291	303582	208096	145413	169128	144102	362290	436028	232415	118988	335575	258812	607466	116440
EIC	Negative	484.2766	6.19	unknown	511111	692155	605101	497928	533985	398320	521371	548514	287068	602981	688830	607996	433292	1009648	1006127	342277	582472	852573	864826
EIC	Negative	241.0983	1.82	unknown	183615	102817	147366	0	75159	24334	79867	0	65103	0	132995	122932	114130	29127	62661	155704	101283	0	70059
EIC	Negative	334.2109	3.90	unknown	680727	312744	745414	698950	243901	177801	293996	392986	259660	889992	539998	471490	329272	310959	255003	424117	641420	417571	443889
EIC	Negative	331.1908	3.79	unknown	1132038	183250	1504259	1064514	368725	146436	191077	989333	126405	2019058	343769	225227	338509	686614	639082	264146	201142	1281185	527647
EIC	Negative	399.2155	4.28	unknown	1483133	507413	1743995	1112202	283913	258377	484445	588864	288144	1798724	718186	778821	808499	545839	425130	558399	792192	657968	614519
EIC	Negative	492.2742	2.38	unknown	699776	238803	519488	229937	114067	330005	116882	315313	148374	451698	212476	302904	158013	223494	325546	174330	327083	300320	513891
fpHILIC	Positive	134.0641	3.50	unknown	599702	446036	588121	309602	264283	292816	306513	359579	334880	413794	362226	410484	441037	538785	713771	553764	309009	538474	381906
EIC	Negative	357.2801	6.55	unknown	4461701	1665170	2821907	4048793	1962605	4592565	2257703	2917423	2586997	4521117	5411825	3979352	6557895	4266885	3604873	2126730	3766918	7180044	6268858
EIC	Negative	491.1763	3.35	unknown	265227	161102	197238	145255	44951	42832	86646	38208	45011	214654	122419	84280	96306	102637	46784	82438	98137	70568	115124
fpHILIC	Positive	248.1231	2.83	unknown	788536	405394	397748	242913	348195	492227	82484	362222	285820	469377	246013	220288	178605	769796	1286458	210587	296418	388245	336912
EIC	Negative	301.1803	2.63	unknown	1274868	1050002	2361393	1467894	620100	899692	1275820	290012	534933	979321	1205810	2211276	1513165	1430687	1147231	1508087	1739638	563663	849035
EIC	Negative	350.2428	3.22	unknown	98271	108818	93974	136710	18684	0	0	136173	0	120197	0	106326	103568	12248	0	144751	0	103687	0
EIC	Negative	554.3161	3.09	unknown	773011	0	3505582	3310776	0	0	0	2534703	100564	2598801	132926	0	157837	201113	183407	0	121370	3817891	105302
fpHILIC	Positive	271.0742	1.16	unknown	9704004	689514	10900171	8708090	522571	1369520	248899	8247055	78920	13637296	520992	1072933	182883	220936	3408633	3521288	183513	8253645	1450755
fpHILIC	Negative	132.0496	3.50	unknown	554030	318643	427846	325549	170106	230516	188816	321408	158784	373712	356469	344160	397796	300637	563513	368006	214521	396613	176302
EIC	Negative	344.1872	3.91	unknown	281454	184321	513020	277372	124357	85943	103729	90264	78211	125374	105598	155088	176055	114664	107304	178201	241366	145339	105203
EIC	Negative	360.2259	5.65	unknown	200522	155869	300778	167327	121741	123897	162925	149960	119467	205613	176431	150648	224569	142863	137487	225994	200984	269886	258211
EIC	Negative	273.1494	2.41	unknown	624314	238658	1254260	576601	125399	268991	369192	297009	295932	574269	387814	567280	364374	378069	357620	408899	501391	612632	305787
EIC	Negative	414.2030	4.14	unknown	233147	201392	214820	195340	80361	82216	152786	104294	105024	137389	141028	202868	204831	144379	182283	199930	182385	148896	131918
EIC	Negative	301.2173	6.14	unknown	1988146	1311249	2703437	1419456	1042527	1210226	1032165	1264826	1184414	1845436	1277357	1443377	1678792	1097416	1060003	1857608	1725088	3058146	1561931
EIC	Negative	492.3420	2.99	unknown	518860	564214	541623	319617	292751	438281	353529	335442	295654	833148	565073	654877	387713	390898	877586	796764	362894	480437	254212
EIC	Negative	429.1696	2.24	unknown	715123	143654	685793	0	93443	107974	216646	347095	150857	0	179277	348288	222789	236267	183686	70149	194173	842116	190005
EIC	Negative	441.1592	2.31	unknown	418150	588395	321789	787745	241159	346643	442555	466212	422144	735467	478879	740784	671510	542566	577329	549883	536245	925126	512042
EIC	Negative	334.2109	3.15	unknown	522342	272652	638191	622392	119792	135406	202247	229156	128697	438992	221115	248309	293364	207225	193419	271520	294153	319978	239095
EIC	Negative	364.2135	2.98	unknown	384151	170721	727091	368358	116332	232190	183960	321840	326709	312970	294491	410032	370401	266440	100273	313834	430741	744869	321173
EIC	Negative	272.7951	0.52	unknown	751133	257679	609336	681803	305958	323596	346297	164474	337104	1143980	250194	270778	358110	461878	283036	479961	196847	434621	996439
EIC	Negative	629.5139	6.77	unknown	132793	152428	85192	93812	80816	67138	95966	25239	140607	97452	158578	79718	131381	152102	108032	125778	126447	67444	235469

fpHILIC	Positive	153.0567	2.46	unknown	812444	840836	643140	334156	481609	668183	461161	437342	494442	504704	596830	757584	576437	1323984	1022791	665235	651460	721630	1054752
EIC	Negative	491.3379	2.99	unknown	1590198	1689786	1612088	1201103	887744	1168696	1063485	1020122	903609	2438156	1643418	1888719	1159125	1100794	2515157	2188184	1103932	1519900	843456
EIC	Negative	492.3419	2.99	unknown	523294	540317	529405	371977	317825	403470	350585	319930	259348	772408	540199	583857	373252	367606	815214	709095	394645	473824	282385
EIC	Negative	291.0700	2.85	unknown	8453470	10213803	9900042	2876951	7107255	6434407	9402505	6198033	2721048	8747647	11962118	10683457	7486333	16455828	15135460	5772177	7020670	8314395	15418763
EIC	Negative	318.2063	2.91	unknown	973957	617316	1137629	788566	330954	279570	498887	328115	363069	850153	517172	778648	631589	718352	470501	686473	687945	622746	452002
EIC	Negative	292.0732	2.85	unknown	1413126	1795724	1629051	495512	1216820	1124594	1604082	1003311	477885	1500663	2045657	1818068	1265135	2786464	2587586	982291	1208345	1431133	2597740
EIC	Negative	282.2073	4.35	unknown	260992	64075	165622	748419	47182	59029	69251	105255	178642	198878	150367	216851	164853	0	66455	236819	119004	283046	40363
fpHILIC	Positive	312.1102	2.50	unknown	570093	661868	492071	273472	284288	550660	436390	372116	341880	472676	460049	501901	409283	922807	789824	368509	520089	674912	870226
EIC	Negative	413.1739	2.91	unknown	1220367	1054777	1543953	1844396	939703	963109	1710501	1160645	1329647	2390686	1934268	2411444	2168216	2100337	1718498	2334814	2297355	1978436	1582124
EIC	Negative	639.4113	3.82	unknown	470391	0	504476	418334	12095	0	0	392085	0	514589	35224	27039	0	0	0	19083	0	637238	0
EIC	Negative	319.1219	2.38	unknown	161776	51722	96657	26803	33074	61250	23181	41998	33879	83020	46011	56539	49312	115849	89880	35410	61255	61030	66615
EIC	Negative	375.1200	1.11	unknown	39086	23446	41280	67236	25696	13038	25393	41674	17680	23060	34257	42696	58375	19177	26345	54167	36944	61517	21928
EIC	Negative	314.1230	1.85	unknown	594592	317394	146837	0	275393	172029	266901	0	223685	0	425914	422476	418271	248865	248046	611649	416186	0	250620
fpHILIC	Positive	389.1837	3.54	unknown	0	452263	225847	0	0	33220	145188	48568	309320	0	0	0	0	347992	0	60194	107645	0	705309
EIC	Negative	414.1808	2.91	unknown	237603	215002	345318	410772	205071	186366	375806	288314	275841	561897	470728	544163	464795	458549	402016	496787	497201	406828	367107
EIC	Negative	233.1058	1.08	unknown	57575	50407	144655	74494	71899	60895	70200	107064	54025	150324	72210	67232	161288	114561	106131	73998	116047	167898	129771
EIC	Negative	348.1907	1.38	unknown	493195	396910	0	0	131124	242657	250182	0	137593	0	329090	437366	386233	294554	310113	403054	270555	0	259238
EIC	Negative	332.1952	2.24	unknown	12801968	4605775	13261539	7757880	1996767	1904714	4089429	3426634	2318384	8848340	3840937	5833570	5364093	4146316	3734459	4170788	3524834	8155786	2861833
EIC	Negative	302.1841	2.63	unknown	208001	220364	552072	287755	138421	200653	272972	0	110383	261560	253501	431873	337071	300603	260584	322564	347392	73037	177285
EIC	Negative	483.2725	6.19	unknown	2098376	2858716	2446926	2010470	2268764	1598948	1949249	2272302	776179	2196793	2994662	2372633	1658626	4153109	4191456	1313955	2480147	3359680	3159293
fpHILIC	Negative	250.0929	3.17	unknown	299327	536849	397021	276155	224913	406943	393206	226149	269951	484205	377564	368707	391546	380636	702451	443884	437340	449262	459103
EIC	Negative	595.3822	4.13	unknown	359578	171840	245027	353301	203585	142546	101256	749150	164781	872785	710703	264932	470212	231083	321931	277211	518091	793813	319379
fpHILIC	Negative	263.1138	0.95	unknown	1819144	905301	2199687	720237	709435	1826762	1013222	1655550	675705	1361400	1407178	2222267	1427479	1354699	382453	2331483	452929	1293700	5181090
EIC	Negative	330.1794	3.12	unknown	738218	858749	581626	755147	266333	903204	393855	176583	333799	532521	821435	1302687	1329282	808084	929833	655609	712572	449779	656756
fpHILIC	Positive	134.0349	2.46	unknown	2052056	2291992	1690819	942003	1068430	1870734	1187547	958852	1253303	1359670	1680310	1776904	1371508	3099548	2613093	1501983	1489162	1721046	3089916
EIC	Negative	329.1761	3.24	unknown	417483	630202	565259	892121	168784	132479	140597	265826	93028	360748	278878	209318	182242	100564	303143	433740	170007	157054	252471
fpHILIC	Negative	438.2166	1.64	unknown	0	622207	217581	241380	263767	0	193987	126829	486363	226722	442790	432214	322476	346392	621105	532336	265273	155415	186829
fpHILIC	Negative	329.2324	0.48	unknown	711523	190838	602973	111458	139786	434674	366871	158849	345248	398972	120382	281669	202361	262475	107611	1219164	307551	416193	326598
EIC	Negative	492.3425	3.82	unknown	217318	181650	224453	98203	121890	132206	125685	119689	88578	278429	202183	247986	184788	276689	232423	181639	192812	237014	123505
EIC	Negative	331.1919	2.24	unknown	57488586	20796503	60682694	34192816	8924176	8507416	18379029	15122975	10362656	39721957	17431980	26393464	23815838	18585788	16884552	18589865	15826769	36629970	12908238
EIC	Negative	273.2074	2.43	unknown	101296	33303	139301	91163	57198	61309	27236	100186	24663	101502	33586	33746	32999	22813	38800	223591	46576	107686	29629
EIC	Negative	374.1442	1.82	unknown	196496	86186	152366	0	100080	48212	52308	0	52955	0	100969	117684	107013	47357	74619	157015	106883	0	79857
EIC	Negative	594.3424	2.27	unknown	481929	377774	482696	343854	213477	487466	237818	364480	405101	609797	419914	623942	309244	292041	411258	675005	521083	362188	852726
EIC	Negative	313.1816	3.61	unknown	346057	357841	932549	1250208	203180	152158	288411	316328	321752	275420	224956	293269	303466	209713	142585	288827	276834	263322	1149626
fpHILIC	Negative	291.0695	0.37	unknown	6067893	3939994	9044914	3373955	2645363	2432552	3858733	4255118	1627502	5118732	4222731	4708580	3336328	5858170	5720192	2182175	2909359	6982773	6729107
EIC	Negative	500.6353	0.52	unknown	692497	122373	820668	452223	214648	354358	150820	0	407658	1183343	149780	142887	203866	0	135320	338886	102813	301162	991829
fpHILIC	Positive	152.0622	2.46	unknown	1099288	1099422	951578	437273	617791	876121	621160	629947	653098	729969	931547	1087900	805154	1659321	1378839	839669	836673	964671	1616950
EIC	Negative	347.1869	1.38	unknown	1841990	1821449	0	0	608851	1085443	1114596	0	618953	0	1525180	2032555	1714600	1323432	1462495	1812992	1273132	0	1138253
EIC	Negative	371.2017	2.52	unknown	497358	217640	422670	397739	113981	110956	197244	111278	120339	485328	205797	286752	247204	228944	161634	228122	215972	270217	209213
EIC	Negative	427.1796	2.46	unknown	375812	150615	358182	437236	57557	110366	113799	86836	82799	545814	184497	201217	213175	168841	168550	151188	173108	67849	188484
EIC	Negative	409.1691	2.88	unknown	405241	389316	397183	426798	182986	138599	239775	175954	153832	310179	204598	285923	268324	265012	308045	363628	383217	254318	170255
EIC	Negative	328.2574	5.91	unknown	97890	46042	132543	83771	23579	43165	37896	18450	65680	39758	40210	81593	70388	37874	61197	56114	71215	83408	41907
EIC	Negative	370.0164	0.50	unknown	418761	221858	208414	274037	130998	152905	135082	138103	139102	318988	148437	179517	127637	48827	142715	303593	274027	197397	240030
EIC	Negative	533.1669	0.85	unknown	51672	41794	507703	16444	40847	58479	89074	49220	73851	120975	72349	101536	74176	109494	115700	26558	309975	41982	23867
EIC	Negative	316.1999	4.02	unknown	3523306	1617225	7088185	4045862	1017640	1346772	2245407	2058955	1684573	2746849	2186417	2554277	3392531	1987689	958659	2423814	2584251	3189978	4738687
fpHILIC	Negative	312.1122	2.43	unknown	589040	987082	488722	391174	533563	661491	700499	624749	714417	637258	1385532	1464207	735661	1446732	1027690	1273330	652342	860266	1568999
EIC	Negative	391.1070	0.47	unknown	526273	791718	460496	309804	282930	625715	356126	189999	244939	496331	634978	758879	690874	466759	982033	722271	441180	413399	270268
fpHILIC	Positive	215.0695	3.30	unknown	758461	665809	351820	327188	287913	422112	217883	135843	290530	299320	343789	475433	382310	366971	511029	506340	264736	542889	430431
EIC	Negative	317.1757	1.82	unknown	260925	101172	284850	0	20670	62667	50469	0	24968	0	44532	80065	62328	56076	72203	68372	50749	0	73651
fpHILIC	Positive	166.0526	2.47	unknown	595904	498898	392650	175064	223776	296522	272867	199981	220526	271047	314256	411187	233902	652376	544648	302431	334107	388303	456227
EIC	Negative	475.2013	4.14	unknown	122092	102732	107902	104100	62615	35264	72953	43502	67587	71899	98052	135525	108202	68977	116702	113643	87391	85904	66955
EIC	Negative	329.1761	3.12	unknown	3087862	3965114	2465871	3324376	1177132	4012266	1779917	726991	1361741	2374985	3755521	5841608	5879935	3592765	4250503	2825240	3210632	1942188	2917400
EIC	Negative	417.2271	3.02	unknown	190292	107326	284449	153695	76649	0	119822	82860	91981	247620	103431	110316	184795	102321	88070	129985	263740	139323	0
EIC	Negative	369.1860	2.90	unknown	405346	241783	558885	249791	116401	102484	403148	180814	236000	408194	253887	461876	504393	341050	189325	316738	301370	597150	337347
EIC	Negative	353.2327	1.71	unknown	289135	146740	346534	267625	60542	159098	101985	121599	83219	235128	139403	174612	159393	126032	139546	213332	190452	209309	130607
EIC	Negative	239.0591	0.69	unknown	206002	217730	218777	121032	138358	143358	135951	83991	124944	223027	171649	164857	202446	347452	222692	264098	169526	165736	229974
EIC	Negative	513.2269	4.12	unknown	230934	317226	228256	335156	81057	93457	161447	66572	123629	177442	208304	251479	246598	199252	208947	233531	203554	75288	162093
EIC	Negative	352.2211	2.69	unknown	297633	126443	420951	142332	70095	87451	117099	103076	80305	189364	132725	134959	155034	145323	153327	126983	129487	205564	154334
EIC	Negative	350.2056	2.25	unknown	632472	603268	648245	663500	191508	181456	180849	218094	186889	526872	274539	447056	356002	334589	317893	365259	313253	334882	281825
EIC	Negative	235.1023	1.08	unknown	27928	18532	53315	28725	27556	21877	28539	39988	20475	56080	22721	31140	62817	40255	43349	30488	42398	70145	46885
EIC	Negative	502.6326	0.52	unknown	902776	153298	1126184	603976	318477	401105	222331	33142	467749	1541666	166936	66994	253754	0	182331	430993	98914	401182	1317829
EIC	Negative	561.3106	2.45	unknown	1259204	0	3376246	2537082	0	0	0	1451409	40335	2276964	0	0	41728	24846	0	0	0	2514809	0
EIC	Negative	595.3459	2.27	unknown	201457	117316	199175	132552	72877	155100	62912	117033	128633	242260	135662	192736	122850	84755	127229	211606	168648	128835	268563
fpHILIC	Negative	150.0396	2.46	unknown	1449791	1586199	1065855	575802	750774	1398496	661118	666058	754525	847892	1014071	1353404	972154	2312204	1821856	968965	1047785	1263679	2041808
EIC	Negative	361.1661	2.60	unknown	679895	947544	511123	435913	469762	407213	762071	283931	506611	448025	1019310	1502391	1812335	951723	1174065	830949	940472	243050	917970
fpHILIC	Negative	105.0386	3.61	unknown	1343375	798658	927685	827840	440534	457795	460115	571518	440001	778575	631202	786997	706536	613308	1019746	817505	577523	1056811	539886
EIC	Negative	319.0005	0.68	unknown	183079	236111	269854	141297	124531	135252	137508	101339	130954	230842	188285	171432	210061	194824	202241	249502	179007	214766	221040
EIC	Negative	347.1870	2.68	unknown	1425391	771985	1573003	1101409	375158	714655	780901	508178	451166	1055687	738500	1072323	1200923	878959	1261801	910120	824714	1034632	763838
EIC	Negative	367.1689	2.24	unknown	617080	195567	539437	362272	91632	85763	167075	139156	121053	426352	151387	243392	203415	172500	143248	212852	141377	375936	151129
EIC	Negative	412.3435	5.74	unknown	384847	178773	187336	336153	207698	125771	351154	156246	175564	113903	194361	132349	148571	135176	226726	348578	187842	176102	783523
EIC	Negative	300.1685	2.99	unknown	1178103	583089	2057069	1307964	273549	416263	547535	365653	369474	833262	723733	992980	770336	629427	526582	885358	801428	676635	467966
fpHILIC	Negative	315.0806	3.66	unknown	532999	241041	195726	223149	153895	313829	116511	195841	213221	265929	120071	300226	177036	212142	239259	355271	392200	451238	253350
EIC	Negative	431.2047	1.74	unknown	576938	214546	453213	387330	71386	43495	57843	101339	72305	897989	198050	171737	96899	203729	114233	40832	158641	125941	150528
fpHILIC	Negative	114.0196	3.50	unknown	733748	422873	501436	434633	279924	283042	282221	337527	247287	493100	454984	373433	401205	417856	817389	469562	317574	600462	235994
EIC	Negative	378.1384	2.49	unknown	464678	148986	720328	305759	68063	159465	84673	378431	45380	498228	157317	306758	176241	485765	128829	31513	286520	406947	402922
fpHILIC	Positive	190.1261	2.91	unknown	564149	250455	303138	239869	0	267784	171184	176388	224131	313445	196615	235540	160302	192987	364297	310654	236256	252213	270384
EIC	Negative	569.2634	0.99	unknown	57685	42036	64905	97317	22136	31642	39984	15478	34668	67107	57837	47738	49268	33879	41391	47451	41120	38033	78304
EIC	Negative	413.2001	4.14	unknown	853258	687693	852251	718331	323642	259623	454165	270742	342220	520447	504954	745615	705861	469693	703476	606256	579913	486268	449986
EIC	Negative	318.2156	4.14	unknown	4554641	4008170	4642201	4220426	1565905	1403381	2584088	1749971	2487641	2608876	2727754	4323409	4237012	2835315	3952883	3721565	3710552	2881407	2505178

EIC	Negative	332.1952	2.57	unknown	678088	751925	713821	475104	289811	287810	411782	113813	247135	1009077	786932	886274	591511	576369	355083	594427	716346	511519	138287
fpHILIC	Positive	297.1848	0.50	unknown	692761	184801	858570	401424	51301	66287	158794	140996	156789	338871	96439	220531	230568	115769	166277	83176	186174	378495	171228
EIC	Negative	241.0873	2.50	unknown	156770	111839	196385	127992	58247	141524	93579	95865	225098	143229	127643	217423	228464	136059	56234	163831	231756	441308	163492
EIC	Negative	604.3629	4.32	unknown	6615367	8078800	6352122	6997652	8239092	1630522	1176116	7673590	1032589	765693	7906507	1004661	8088646	7022885	7979388	8206085	9106481	764615	7950123
EIC	Negative	362.1979	2.18	unknown	1509307	2541154	1845119	1414161	1061035	370140	979976	572089	1036666	1286456	626902	1553579	910609	632450	1065233	2122905	1516466	1579292	637363
EIC	Negative	504.6292	0.52	unknown	663519	97208	733765	410739	208437	323647	145494	0	356302	1135753	136457	34966	152028	123929	138404	292169	99724	294755	955753
EIC	Negative	396.2471	2.52	unknown	617950	298742	537852	576935	127926	145484	228739	186262	175749	627133	296296	382913	355814	324510	199722	340846	282046	394489	271574
EIC	Negative	269.1762	3.81	unknown	530097	262179	565832	321685	161861	163022	305119	249648	176876	314148	258589	462403	515286	329200	236662	315357	352690	592229	323989
fpHILIC	Negative	347.1862	0.61	unknown	2140757	1108121	1632466	2516052	707604	867915	764665	735105	909411	2229096	1261594	1648246	1258968	924758	1260584	1989293	1261760	1290715	1067879
EIC	Negative	334.2108	1.80	unknown	432237	123141	283125	0	39171	29529	70860	27423	37598	438096	95404	102370	54545	94293	38617	108601	113737	0	92291
EIC	Negative	440.6790	0.52	unknown	282061	36804	332575	242050	97791	118770	31983	0	174862	484781	29775	81738	62973	0	33243	167326	40056	124280	366745
EIC	Negative	299.1653	2.99	unknown	5511847	2940737	9794825	6173154	1275353	1989662	2734594	1672650	1779063	3960767	3628947	4870494	3905599	3088958	2692748	4289608	3788619	3357501	2209133
fpHILIC	Positive	274.0890	3.15	unknown	639991	1579869	917742	633916	612691	886710	854295	613996	588857	1101125	1034786	1059433	964733	1197053	1685125	983886	1109377	1108206	1269716
EIC	Negative	332.1952	3.24	unknown	838979	475465	1160051	891887	198201	190644	307307	205189	178292	636536	295201	430156	360531	232622	263107	426516	329971	536888	313741
EIC	Negative	275.0601	2.33	unknown	71774	169797	269299	38294	94082	227254	108574	59725	72579	195865	455680	439061	199818	451061	342952	191436	132418	92968	214671
EIC	Negative	258.2426	6.54	unknown	207253	217915	158474	329650	168760	186437	197599	158994	306171	368983	406519	285063	400308	273880	231594	410331	401691	347319	377112
fpHILIC	Negative	164.0433	0.51	unknown	722660	440872	539080	381674	253895	753430	326288	182900	356435	578965	507317	571624	400843	1248778	1018973	443520	496345	582382	712530
fpHILIC	Positive	344.1849	4.83	unknown	493370	376712	193827	140629	152675	184254	165087	155184	443127	280294	207877	165603	173755	525221	788868	362891	296183	147978	320512
EIC	Negative	642.5975	0.52	unknown	275880	80483	438670	331676	165538	224011	101258	0	0	427383	100949	78164	122909	0	78166	172148	75646	197439	331580
EIC	Negative	348.1904	2.68	unknown	308578	187260	375778	252909	94071	126930	168807	136575	109412	247416	158504	237804	260110	192481	287160	211208	196290	233846	179704
EIC	Negative	336.2265	2.51	unknown	13094569	6216816	11192925	11151577	2550733	2848984	5086822	3703638	3402459	11667212	5854750	8071036	7087726	6723008	4165927	6819405	5703070	8423032	5595070
EIC	Negative	398.2028	4.02	unknown	232181	107697	424094	186882	77740	84782	150796	116521	104582	172109	145481	168487	207070	142161	85927	178193	155968	220276	292765
EIC	Negative	501.3012	3.76	unknown	441575	42226	156999	415490	73810	188551	108844	252247	196426	449712	219251	159758	286173	31632	101441	206867	306974	817692	0
EIC	Negative	632.2962	3.10	unknown	84366	56970	97538	65655	36577	86375	34852	86346	80205	134198	91849	109212	78271	73091	91563	123502	112111	72022	167954
EIC	Negative	392.2172	2.43	unknown	448182	303201	471920	407785	135569	185532	329352	182065	138793	315768	235428	435915	395758	357837	360976	281071	312141	365096	379499
EIC	Negative	491.3379	2.90	unknown	2764365	2072413	2862636	1778917	2214720	3115955	1092205	1367018	939707	4780111	2896812	4722451	2908559	4717952	4184104	2319768	3420280	3604194	1741785
EIC	Negative	275.1656	2.14	unknown	1141625	905261	2651059	2112626	350968	690615	776405	498624	759096	2516863	1098194	2666536	1037201	1037681	707269	1121263	1296847	1270281	984539
EIC	Negative	348.1902	1.78	unknown	292501	96619	236142	87540	24540	40433	44905	32281	32961	332242	102442	90240	63204	78298	59934	70763	59563	0	116116
EIC	Negative	492.2327	1.93	unknown	1135930	1674452	1717524	552694	1437654	1171804	2117886	0	1159425	1143574	1607359	2343320	1976256	2238491	1881711	2625974	3692941	0	2128655
EIC	Negative	560.5912	0.52	unknown	889054	154682	1143048	629359	200582	390898	167621	0	556280	1490302	144701	122175	180501	0	181719	385297	98966	306428	1337423
EIC	Negative	335.2232	2.06	unknown	3208558	940789	3393971	1882465	401188	682408	703919	826015	711775	4205317	1077360	1062370	1069698	1350686	756339	935420	1617103	1087670	1450685
EIC	Negative	634.4110	2.91	unknown	842600	348231	1127691	669080	181982	107056	320774	142724	183171	833536	343932	473014	499038	384296	241918	449104	448580	345778	160395
EIC	Negative	296.1852	3.28	unknown	172382	0	0	0	0	97066	0	0	0	84625	0	76965	0	0	83168	0	0	87715	0
EIC	Negative	492.3420	2.90	unknown	836372	635020	828573	514073	676060	972516	303524	429411	311384	1448771	906156	1415998	889685	1500203	1338635	673778	1091505	1124903	515035
fpHILIC	Negative	172.0979	0.59	unknown	10766249	9983647	7206208	5281037	5526636	11126112	6167706	2938585	5534425	7790104	11585799	9052722	7106318	10388216	24339886	9942607	5509938	5345439	10017599
EIC	Negative	318.2157	4.89	unknown	2326293	832679	2910198	1819115	463822	339379	727578	791397	485002	3268180	1091816	936752	650567	710295	442511	1085766	1427150	1035645	960265
fpHILIC	Negative	150.0126	4.37	unknown	717313	597301	353734	50097	167862	711629	110366	287699	119822	107330	128611	273202	63890	1808713	1206309	148348	196528	240051	674248
EIC	Negative	411.1853	4.02	unknown	298425	132979	591973	311056	92656	110035	200442	153924	123558	225154	178094	191444	286246	158412	76579	214140	220646	285186	406025
EIC	Negative	448.3515	6.54	unknown	1514345	817049	1111464	1244553	1201635	864292	1324778	1263059	1241499	1974385	2038698	1041895	1648961	1867098	3032683	2526061	1331350	1474620	2057576
EIC	Negative	335.2232	2.51	unknown	59014202	27961316	50851402	50501125	11570085	12789344	22887651	16927765	15279563	52392291	26573302	36664648	32298966	30226777	18823069	31027752	25914558	39093680	25686981
EIC	Negative	345.1711	2.46	unknown	2235302	2122511	1963752	2026742	1021542	1790684	1662403	1091433	1141449	2161355	2086468	2669193	3330146	2333259	2920890	2077097	2164089	2625343	2092054
EIC	Negative	367.1687	2.43	unknown	278237	150594	276808	210145	92422	82647	119951	86560	63987	178894	127899	190601	165215	155618	172004	166638	138735	137724	175213
EIC	Negative	315.1602	3.49	unknown	230034	233869	325524	270922	91373	86462	114050	68367	83921	163404	126691	204602	152778	116673	155682	240500	151519	119660	120823
EIC	Negative	427.1739	2.31	unknown	490797	1205394	330747	1152354	461104	587072	887994	753564	853489	1810536	804279	1424784	1364123	983528	1221465	974404	948940	2037923	1108384
EIC	Negative	395.2441	2.52	unknown	2659770	1210226	2223060	2231245	532343	609718	923506	733894	695929	2549396	1165092	1575968	1360929	1359201	823550	1427710	1169437	1645776	1140871
EIC	Negative	273.1495	2.64	unknown	168817	88359	185668	87352	44664	64283	99767	0	65938	103643	92772	137879	169365	114096	59272	96965	115180	143831	72928
fpHILIC	Positive	334.0916	2.49	unknown	280837	386853	189451	109867	131578	301549	199950	112685	157215	237463	306432	247243	218761	554968	414598	199290	234988	308455	447743
EIC	Negative	325.1619	0.50	unknown	288737	333447	142143	107840	155387	218304	95506	66237	156024	156221	283316	307606	254709	79514	388541	358035	152151	131892	199801
EIC	Negative	400.2190	4.14	unknown	434229	361446	385658	358456	139352	126342	233709	160316	210743	256832	281674	392249	375930	283503	393297	360377	328780	246640	221206
EIC	Negative	336.2264	2.96	unknown	317908	114906	337945	299327	58192	77041	72993	100368	62768	297278	150708	124375	116493	108572	113135	122886	148796	133222	143402
EIC	Negative	335.2232	2.18	unknown	1166122	865470	1909052	1262678	585724	613654	948071	777498	1167641	5794513	1016237	1178328	1537883	3215192	1053218	999925	1176910	2100602	1971741
EIC	Negative	509.3496	2.30	unknown	89113	385099	60486	349793	229162	464947	186503	137927	88212	392950	313237	317223	399434	387751	592881	470576	246657	323717	341779
fpHILIC	Positive	167.0563	2.98	unknown	1197444	1370471	953938	505146	794046	1208997	666221	0	809164	655367	821546	879104	684449	0	1563111	806314	1030520	1101775	1716354
EIC	Negative	432.2126	2.52	unknown	553804	235592	559839	417101	131096	104619	208371	155804	134368	477987	210462	312683	258744	257381	165494	255148	232840	432535	227027
EIC	Negative	358.2335	3.90	unknown	213759	18322	274589	101176	25237	56770	28405	110520	0	147699	46070	79746	47417	67789	52781	17955	64723	182383	77369
EIC	Negative	297.1518	2.19	unknown	412985	0	657761	399845	25784	55079	60743	410860	0	743523	27923	0	0	0	19633	0	0	968647	0
EIC	Negative	399.2154	4.14	unknown	1818717	1511689	1576739	1417328	618092	539079	899802	624153	912414	1033067	1073890	1632361	1558303	1068306	1548117	1455003	1375072	1030820	1025431
EIC	Negative	489.1799	2.24	unknown	205209	63993	161870	198573	18005	24145	42188	70124	31394	217173	7417	87205	58195	0	9171	52229	30876	240998	0
EIC	Negative	321.2064	3.12	unknown	431657	237134	420141	441070	104885	119944	150244	193884	90256	305298	172038	251781	248202	172516	238264	206365	281990	257959	198092
EIC	Negative	309.0679	0.49	unknown	717733	406048	448825	249560	153179	232924	209924	141881	240162	416415	265284	251638	314602	142280	341980	486407	253450	332007	340986
EIC	Negative	329.1761	2.83	unknown	527407	957289	636231	933278	264366	283687	273485	260565	236313	569545	375089	544646	362592	351183	318593	583024	606074	500730	359401
EIC	Negative	428.1821	2.24	unknown	270077	75450	270555	212491	36895	34745	62679	83121	49288	203342	53179	92504	58842	81355	51063	64881	60144	241720	62016
fpHILIC	Negative	315.1965	0.44	unknown	11974501	6357471	17892518	11512459	2799059	2785622	4692311	3351863	3551320	10337831	6247365	8798301	6605752	5949741	4433249	7286870	7226825	6879159	5216158
EIC	Negative	484.2762	6.36	unknown	929906	977974	1212347	1166570	970500	767107	1054504	1502692	607308	1438681	1461111	1582503	1454092	2225311	1817130	797977	1256975	2941133	2045301
EIC	Negative	562.5884	0.52	unknown	739737	88590	926444	448265	213679	251604	160839	0	376211	1295397	82625	88973	181321	0	150346	280734	0	287583	1093760
EIC	Negative	509.2296	1.29	unknown	396115	155924	116765	0	106631	113930	166019	0	74574	358543	92089	162723	120214	146071	183802	230846	234208	0	185232
EIC	Negative	329.1761	2.68	unknown	326497	318637	628501	311783	170799	150537	165420	152228	137680	312876	262699	322238	292439	235777	265739	412635	286812	256379	184791
EIC	Negative	361.2058	3.53	unknown	333850	102447	113454	0	63277	52645	51931	75413	0	88786	0	600312	0	0	81958	181822	0	0	177558
EIC	Negative	501.2821	3.41	unknown	553320	0	551925	1559791	398459	549457	527137	0	524930	0	666852	20575	701690	0	404753	552138	772936	1739422	161724
EIC	Negative	313.1814	3.79	unknown	429033	256542	528418	448444	116938	131781	232680	188537	151149	282085	243169	303987	350993	242653	329751	311028	293397	306243	250955
EIC	Negative	491.2297	1.93	unknown	4821799	5740177	4776038	2356988	4918061	4116114	7420101	665603	4102430	4303556	5645953	8226619	6863776	7769377	6399584	9030598	12897108	2737440	7493414
EIC	Negative	412.2424	1.48	unknown	169581	431894	0	128791	172852	202056	347797	0	215730	0	344469	431096	422317	343041	414986	548348	425831	0	320749
EIC	Negative	373.1413	1.69	unknown	401738	336160	282289	559777	263444	150825	253131	281897	181728	366920	369137	415631	373739	203001	205672	552996	386424	710403	206504
EIC	Negative	303.1633	2.50	unknown	82665	0	0	124600	0	24340	0	109208	0	237691	32496	0	0	0	0	31411	0	212165	0
fpHILIC	Negative	172.0979	0.74	unknown	1248547	967714	907431	488525	583008	1088153	646934	298775	526608	745755	1019126	928385	663879	1149403	2647250	1007034	627276	509074	965745
EIC	Negative	451.1965	1.22	unknown	114758	53252	308301	170023	37938	92324	105906	49657	61045	243061	122408	114743	92512	85646	81056	131092	265337	74913	83579
EIC	Negative	625.3747	2.77	unknown	250683	262403	223195	213093	147164	202142	334347	149474	145818	288067	365102	384084	475707	288456	301041	272614	280690	493469	448019
EIC	Negative	427.1798	2.24	unknown	984370	281509	939405	694900	125117	100001	203968	281340	151441	797366	202779	383235	227290	226236	156934	239283	185080	809722	190694
EIC	Negative	394.2318	2.31	unknown	178507	1147308	2179111	336195	328352	360483	589681	476664	341946	939214	1627801	1070321	901569	1327787	651058	140211	1273951	1579241	209783

fpHILIC	Negative	383.1218	2.95	unknown	648705	394655	500009	270954	236832	197734	214952	295515	254374	438305	169689	279845	245771	623637	658498	215333	314613	606044	413192
fpHILIC	Negative	316.1998	0.44	unknown	2556892	1272537	4117182	2881375	520963	689405	1065322	687881	845880	2163283	1370154	1822527	1678748	1248702	967679	1528549	1645298	1567938	1303208
EIC	Negative	407.2439	2.20	unknown	0	2121517	0	4049376	1393998	922606	2366843	0	2414104	3508462	2958079	1213691	2554852	2218908	1668811	3046200	1496691	4498817	2393224
EIC	Negative	336.2265	3.32	unknown	586711	250621	693174	569095	119432	133162	216937	224069	103939	400275	217597	281275	281534	257120	235738	288485	355617	331462	262116
EIC	Negative	628.5039	6.76	unknown	154720	133714	120677	167767	149672	121126	155581	131895	161622	254336	374310	216617	282909	342840	291445	251887	319731	149797	318416
EIC	Negative	395.1842	2.88	unknown	647453	701803	490755	562735	377386	256385	368540	318908	248051	480911	392347	497935	499432	546329	705022	674103	675614	453622	395156
EIC	Negative	335.2153	2.90	unknown	653884	299110	571626	404574	174381	0	394776	294335	305743	0	411797	503719	600354	372959	283519	454182	398008	582217	445708
EIC	Negative	507.2244	2.08	unknown	241339	145474	333067	112508	94555	149657	216458	0	126354	171408	196150	417277	398260	192980	243202	232614	266069	110356	234153
fpHILIC	Negative	305.1428	0.60	unknown	4566534	2405595	3827408	4035873	2909376	2931965	3407495	4326452	2852468	6336068	5124681	6176995	5046346	5555996	4259402	4664017	6222604	11538601	3747571
EIC	Negative	451.1516	2.24	unknown	43476	14134	182527	166097	15133	9990	40100	94512	30370	203156	31563	49209	39130	43265	35723	46447	33533	206229	30301
EIC	Negative	411.1805	3.12	unknown	402264	476210	341684	407448	138666	372899	221904	99704	150007	364312	433712	606382	563640	469988	477015	375690	425404	253204	291200
EIC	Negative	546.2809	2.55	unknown	609738	0	1022007	491032	0	0	0	382536	22219	253086	0	0	9685	0	0	0	10157	740196	0
EIC	Negative	399.2156	4.88	unknown	881828	333996	1100042	628107	168731	108922	299547	282779	190670	1227605	432012	371825	272957	282591	160126	461727	528644	386813	410771
EIC	Negative	481.2183	4.14	unknown	344207	289298	276653	246739	102187	114169	179561	108389	152882	190470	199936	298991	280787	209118	317032	275828	259849	178607	190773
EIC	Negative	442.6767	0.52	unknown	697418	159821	1009455	519466	31247	345263	235214	38999	462861	1216843	163302	146691	269012	0	163876	412367	126362	340174	1031498
EIC	Negative	313.1814	2.89	unknown	17723829	13500599	19370701	16257297	6365153	4070485	7633011	6518614	4599663	11922725	7224893	10587671	9680088	8799320	10319117	13660542	13305171	10288593	6818524
EIC	Negative	332.1952	2.41	unknown	1581371	1169700	2176411	1405815	462710	531920	918119	497802	506780	1220875	955043	1564920	922728	1113937	1310161	1011033	1065171	984316	1075637
EIC	Negative	400.2189	4.28	unknown	398569	169134	409419	303500	68322	47342	117481	165767	73471	450569	213794	193182	223216	131627	111274	165653	208502	206507	189988
EIC	Negative	314.1846	2.89	unknown	3933189	3012204	4187821	3563445	1388392	885617	1675583	1392801	1008888	2619928	1611899	2286720	2091220	1962098	2283225	2973308	2882403	2243855	1480080
EIC	Negative	347.1869	1.59	unknown	928835	428851	918421	694990	163136	208714	311326	195319	185696	532949	312285	433346	396445	332111	352940	409251	340352	251247	636660
EIC	Negative	415.1206	2.91	unknown	139386	97585	196564	195753	47072	49458	79859	49460	63106	92286	189431	236177	143844	119296	110327	166667	96148	101575	66644
EIC	Negative	493.3468	2.90	unknown	134390	176788	226775	48148	137806	220708	63905	123776	85807	308988	187728	319476	153457	291640	357724	180465	264330	253248	129436
fpHILIC	Positive	256.0806	3.00	unknown	362114	239552	224482	159338	279653	289775	257938	217772	56174	274660	283072	219133	175838	342885	550285	296485	361894	291630	565549
EIC	Negative	329.1761	4.71	unknown	8250871	6335363	9281332	7361538	3079908	2864187	4679138	3481954	4008401	5001494	5121459	8905134	8097420	6029255	8395595	8516619	5978987	5316056	5773969
EIC	Negative	431.2100	2.52	unknown	2188498	879841	1594725	1534141	362919	398254	694579	528463	466451	1812850	806420	1096071	987371	938488	601280	954062	776240	1475821	792603
EIC	Negative	315.1967	3.31	unknown	4734872	3994118	12148226	9287713	3101155	1603888	1404386	1437630	2116127	6506621	5753933	5898298	2390419	3538217	2623351	4284150	5724928	2638325	2912387
EIC	Negative	241.0558	0.68	unknown	73092	62359	68545	35059	40997	44023	33999	19561	44167	73510	47644	57798	63731	113483	65732	90291	52423	53562	81963
EIC	Negative	387.1805	2.10	unknown	121224	104841	40931	43041	34428	66051	100119	0	50082	47076	76515	150954	141054	78229	101837	133390	97729	55357	83857
EIC	Negative	633.4074	2.91	unknown	3904710	1824757	6026683	3620201	920682	780893	1607178	945807	719989	3825605	2011542	2828035	2231026	2130565	1409823	2616423	2324059	1969218	1050919
fpHILIC	Negative	627.4995	0.36	unknown	952949	1929736	1469361	2062511	789196	799022	964389	828775	822532	1632923	2804694	1271306	1751303	3693857	1261168	1256415	2371840	864963	2176933
EIC	Negative	249.1498	2.53	unknown	753315	43141	307241	56404	35899	41275	44968	45489	33624	79616	49362	46818	59030	59935	43706	43393	153715	72144	66777
EIC	Negative	267.1614	4.37	unknown	665420	516503	790747	294423	121005	263601	396861	341401	167196	342306	497933	655327	757502	463294	722041	543187	413288	349237	414605
EIC	Negative	361.1442	3.98	unknown	74442	217286	104126	236903	84271	18802	69133	111228	78584	76282	89447	105342	85477	101981	180703	203088	121731	106765	74143
EIC	Negative	459.1865	3.92	unknown	638768	176534	501929	1429399	45843	63530	64622	63297	63236	1196855	270587	53933	0	0	123762	238607	0	185017	22484
EIC	Negative	309.1036	0.47	unknown	422838	638308	343324	266947	254727	412002	331239	148762	239722	338016	483161	583364	562312	408288	841049	633222	370048	359166	426860
fpHILIC	Positive	363.1534	2.94	unknown	1213443	734983	1262637	515520	386331	499347	327455	408691	357968	804133	381973	373720	417536	844084	962106	434716	625471	1041288	692944
EIC	Negative	557.2967	0.95	unknown	244249	71946	260045	227511	60407	35043	62807	51401	41877	386108	87525	41714	56835	68093	45779	83660	143861	92105	111917
fpHILIC	Negative	187.0723	2.48	unknown	2590279	2603202	1720143	2002145	1209446	2147625	974350	1744907	1518503	2116532	2449133	2295106	1340031	3463562	4907559	1605101	1584373	2298843	4029348
EIC	Negative	499.2283	2.52	unknown	305126	174937	0	151054	95257	96195	148940	0	109106	329929	169663	202404	200475	194577	128204	202668	162613	200854	159418
fpHILIC	Negative	351.2181	0.97	unknown	1495092	858499	1613503	1403264	311709	431756	693063	596968	536620	1301975	789318	794263	943581	691788	736734	1171720	985315	1107325	882283
EIC	Negative	524.2944	4.21	unknown	880317	703177	1342470	635614	321444	215487	639813	350075	360125	1370886	938067	685204	900038	734655	412384	726267	988407	359762	1104636
EIC	Negative	417.2263	2.52	unknown	3295371	1403006	2191128	2283717	609869	659754	1165867	889023	806230	3165764	1325364	1758512	1570977	1485653	1002030	1482974	1295492	2706547	1375172
fpHILIC	Negative	73.0296	1.68	unknown	0	2908138	1998916	2695115	1708235	0	1940689	1552307	2435162	2456248	2999275	2478775	2404442	2907341	4667172	3200344	1819352	2644663	2266548
EIC	Negative	500.2049	3.21	unknown	124987	63950	108135	51165	60449	47131	50609	60877	31501	116280	67898	120498	66231	76714	53173	77406	67117	209533	72035
EIC	Negative	303.1603	2.48	unknown	214624	256982	218047	158661	95180	197576	152199	145435	110146	263250	282353	437883	441805	218706	350956	287046	239156	253330	210307
fpHILIC	Positive	191.0818	1.85	unknown	886105	0	0	285529	46587	78925	0	273101	743363	37708	35031	329403	382485	823626	127762	98008	1234628	443825	286169
EIC	Negative	545.2771	2.57	unknown	2101130	27810	3322688	1514155	24359	28577	31367	1216746	63240	824666	12204	28719	46135	44534	49007	52879	32494	2452593	31814
EIC	Negative	595.2744	2.48	unknown	179988	41834	330929	197512	36309	37549	20560	123576	59161	289406	39101	26056	50887	57489	33860	86966	57617	302339	52408
EIC	Negative	362.1975	3.63	unknown	148599	87942	503089	199227	48931	65072	81972	67214	72138	72484	52173	89672	93141	88745	33212	113637	342105	134257	22397
fpHILIC	Negative	194.0832	2.48	unknown	468855	573091	405935	311118	238952	353095	308979	385451	256166	460805	518042	652168	331700	769213	868130	325329	359251	550789	869561
EIC	Negative	273.1494	2.34	unknown	151357	64365	192990	130972	9862	26491	37121	18245	31628	67666	60247	74491	71120	38050	42627	49552	78202	29294	38389
EIC	Negative	247.1338	0.79	unknown	0	20701	79919	260072	0	3207	249337	0	0	91116	0	353965	27232	17935	150656	189641	23340	29602	94252
EIC	Negative	315.2539	3.81	unknown	2656754	2362936	3343419	2365339	2127591	3788640	1320413	1753697	3259806	1662205	1987149	3075691	3433031	2593988	1274052	7722573	5658268	6222554	2815708
EIC	Negative	401.2308	3.83	unknown	0	983504	285773	556404	0	862077	0	533753	48161	79836	932372	0	0	0	0	0	934100	948234	915144
EIC	Negative	411.1779	4.71	unknown	401642	322246	420454	339419	185078	120447	227341	193325	209772	400809	253465	481449	453546	298994	490107	474581	296383	283000	270788
EIC	Negative	303.1633	2.90	unknown	271506	0	0	115162	0	66773	173695	178685	0	248900	0	0	147142	0	188171	193562	0	432047	0
EIC	Negative	523.2919	4.21	unknown	2919606	2201542	4346371	1987393	1028255	744127	1840388	963226	1046835	4348711	3054896	2204704	2872720	2298809	1202450	2101516	3049309	1097825	3470431
EIC	Negative	336.1277	1.53	unknown	91856	90904	154913	0	60637	118981	66084	96270	32608	0	124741	273122	146360	370991	73033	54171	265982	0	335392
EIC	Negative	401.2194	3.36	unknown	253100	192994	298520	226926	86959	82931	128255	94644	94390	264281	236075	185881	179448	193659	126745	197316	185423	180923	258465
EIC	Negative	553.2663	1.27	unknown	330443	0	298146	0	0	50683	135535	0	0	287644	148259	149231	144592	140640	79247	138522	144981	0	0
EIC	Negative	329.1761	2.89	unknown	386875	1063008	357751	1180020	255023	193135	166425	384706	198105	378501	268231	342246	227415	187033	671341	766210	394979	251322	249333
EIC	Negative	395.2443	2.17	unknown	40279	66734	202479	197623	55091	31568	74853	44006	135340	108045	129897	158360	161768	404334	135137	68680	112456	209689	138964
EIC	Negative	415.2105	2.31	unknown	301667	1033722	262744	716065	289652	304965	512502	815187	307566	2175379	172994	146840	684517	1049004	529779	893862	188903	2341317	397294
EIC	Negative	317.1757	2.49	unknown	247621	180243	206334	149441	19385	98834	125286	134290	94257	201492	139475	266820	234327	148724	182945	200565	165155	254050	118803
EIC	Negative	351.2179	1.69	unknown	991788	490600	738106	731966	158440	208558	306676	192128	190632	1071568	351701	414493	326779	298840	309645	500315	421889	302908	529248
EIC	Negative	358.2833	6.55	unknown	1153948	440363	664058	1124841	567081	1298374	620757	750344	659111	1132233	1391332	1011426	1722069	1072761	1364080	650559	1003722	1960148	2637106
EIC	Negative	629.4019	2.69	unknown	47371	105960	43770	92869	90774	52728	85409	0	123140	22150	63975	79015	139004	136425	143894	240618	126886	74686	102944
EIC	Negative	330.1793	4.71	unknown	1844108	1404052	1982780	1654308	671845	645325	955935	734030	845773	1107679	1140201	2001306	1850550	1319299	1826074	1878525	1365701	1232745	1242186
fpHILIC	Positive	224.0914	1.66	unknown	38313	448983	819316	1024943	290337	0	258982	586472	207533	861828	360255	373956	297336	582006	724328	425267	285249	848733	350156
EIC	Negative	499.2460	4.21	unknown	366350	263211	598926	279878	169665	93844	268188	94663	140710	632965	447900	298195	412993	335426	202064	290716	458822	93723	462565
EIC	Negative	525.2828	4.28	unknown	369937	0	436668	202317	0	0	0	383150	0	0	192529	132742	122534	0	180194	0	312190	261563	0
EIC	Negative	481.2182	3.35	unknown	563211	600983	521353	509265	239415	250503	382630	238409	267767	603950	644651	563342	528105	669168	332448	567804	614943	474989	723194
EIC	Negative	628.5039	6.85	unknown	40454	131947	49403	144228	57969	49414	121148	47295	74833	131649	313495	138230	220223	435824	117769	116477	191272	62126	202591
EIC	Negative	346.1741	2.46	unknown	549948	448579	427364	613585	237271	414126	375052	238425	259965	531026	476281	598076	741247	507784	674505	458981	466113	942620	461613
EIC	Negative	395.1842	3.69	unknown	108564	153056	104682	344061	96791	84230	170228	95250	94968	272796	196584	223464	302216	148341	101628	193123	193493	209242	369214
EIC	Negative	306.1774	2.10	unknown	286460	167700	312659	219698	38335	102936	137752	0	11181	169481	117280	268358	271451	137393	146118	176063	137580	0	112008
fpHILIC	Negative	375.1841	0.50	unknown	2004470	822216	3293782	1130226	417727	598491	474209	1831958	149273	4303394	990279	1395157	975766	1317316	1178738	660555	668327	3189980	1162078
EIC	Negative	305.0495	2.47	unknown	3523263	1961819	10613817	1958430	666403	792646	1191697	1752238	1897669	4798590	1817346	2231507	2284414	2604623	2194060	2890286	2955594	2809153	1627962
EIC	Negative	345.1710	3.60	unknown	186059	211590	322918	218713	92390	87509	131702	86351	54004	129649	141513	234729	139065	127484	178976	264879	146991	182478	122418

EIC	Negative	266.1482	1.73	unknown	225792	211906	338964	32039	79104	152511	50656	141479	73361	218172	138473	165145	78394	540061	266034	63912	187361	122972	345127
fpHILIC	Positive	188.1390	4.89	unknown	608610	1681511	844982	750557	582034	746097	983606	646096	857372	1469691	1297926	1156658	1029522	2420338	2369755	1267222	1540805	792188	1604855
EIC	Negative	414.1986	2.24	unknown	930836	216354	807440	642038	352792	75040	243512	0	234962	686191	137584	245130	139958	171635	221878	217354	195576	875903	303954
EIC	Negative	493.2444	3.08	unknown	665996	219692	745286	345359	103007	121698	190946	180299	80214	294179	123814	94290	336090	115128	304454	189351	154620	328326	379393
EIC	Negative	339.1361	1.32	unknown	55862	0	0	0	0	0	0	0	69371	0	0	43665	93964	102598	0	0	139142	0	0
EIC	Negative	316.2572	3.81	unknown	785681	434829	850208	455522	379525	730433	373047	290186	679871	440099	364589	626239	690636	523858	265165	1581879	1167971	1294727	613097
fpHILIC	Negative	115.0594	2.78	unknown	967624	884766	698569	334744	449243	619612	313836	396761	314815	581755	550222	610425	378005	907270	1673636	503828	513470	681914	830671
EIC	Negative	334.2109	3.04	unknown	1044303	527734	1258995	868421	196531	229557	282872	326171	163710	775671	409851	618982	434986	517890	357946	554279	507653	480706	505979
EIC	Negative	627.5000	6.76	unknown	245005	311420	261665	368251	257070	258779	292514	300253	386361	560470	836908	511737	633328	801288	628375	581907	710522	364555	717112
fpHILIC	Positive	191.0836	2.23	unknown	266816	524906	385380	211417	268055	422842	312731	272734	346755	382475	354792	493235	379928	1406658	700437	402888	471524	482292	1204279
EIC	Negative	481.3126	5.55	unknown	294438	209631	616169	122436	544116	0	176550	0	55499	0	90778	0	121589	644605	485261	0	479071	0	595894
EIC	Negative	227.1007	0.47	unknown	364991	522278	283656	210738	196198	373493	239364	132616	174807	280860	421666	535374	509565	425180	706659	533797	319701	313922	371858
EIC	Negative	376.1779	2.24	unknown	119623	144677	109623	89496	66152	21244	43263	0	61806	81228	52972	92543	84579	50359	72961	123359	108528	34748	48771
EIC	Negative	241.1556	1.52	unknown	7457609	6164595	34809275	15670420	11280370	7844997	10338987	23256634	5893934	25849287	9302933	7555219	35893832	12486696	19370552	7038325	26004970	50508467	15374320
EIC	Negative	242.1590	1.52	unknown	1073447	845962	4688577	2225857	1545501	1103100	1413113	3296123	813330	3581751	1331828	1042265	5004859	1748057	2781458	969795	3674934	6950018	2131340
EIC	Negative	314.1845	3.68	unknown	563567	399285	992076	1168161	228656	213148	447027	432004	223442	723601	559935	647193	915759	433939	244398	525263	546954	601357	1202850
EIC	Negative	533.3860	3.37	unknown	162722	99966	612532	2268621	69439	50928	115878	59837	72507	910744	126355	220799	112430	77220	0	135850	202485	230654	176683
EIC	Negative	393.2287	2.55	unknown	1120254	577781	733420	917198	195636	253645	351715	218434	273250	800997	417711	648096	463706	557768	421926	623132	589335	455792	526521
EIC	Negative	280.1178	1.32	unknown	55127	6099	0	0	0	13824	0	0	74032	0	17003	45313	104855	106141	0	7701	152311	0	17958
EIC	Negative	374.2996	4.92	unknown	0	68222	139252	79995	15957	0	0	22481	16612	10204	18561	19718	53251	24044	22927	0	18199	70563	0
EIC	Negative	401.1247	1.87	unknown	4520722	2638102	5615559	0	1393919	1797855	3030148	0	1861051	550751	1968193	3593408	3836243	2788794	1573694	4686723	4331126	0	5862232
EIC	Negative	554.3160	2.97	unknown	641979	0	3163203	3161247	0	0	0	2335101	0	2599154	120834	0	137644	180506	168358	0	0	4522838	79470
EIC	Negative	440.1471	1.63	unknown	211523	76656	0	0	92561	38790	101640	0	77033	64411	124116	126348	139626	54278	68085	198628	117545	160302	88884
EIC	Negative	499.2017	3.21	unknown	445319	256834	374253	169334	233558	157094	167224	230571	126318	449305	266650	445250	230160	295612	178669	304484	268147	832623	287240
EIC	Negative	291.1966	3.68	unknown	277555	176464	211274	427852	81834	100335	117060	95145	77689	344738	241467	227507	184579	160140	141842	196467	176767	248713	203287
EIC	Negative	402.1282	1.87	unknown	1045855	660723	1297474	0	327306	446318	694088	0	428944	0	435572	874320	879292	632027	377189	1117541	1009500	0	1383917
EIC	Negative	445.3329	5.52	unknown	223941	72850	549864	113667	38854	48027	40404	168647	34342	251754	96392	85501	73718	121515	30916	92871	74308	442435	36855
EIC	Negative	413.1948	2.24	unknown	3221345	850324	3114585	2579121	1427426	267871	1007690	0	996121	2818424	556733	939211	665875	737369	905541	860824	832605	3633518	1357826
EIC	Negative	355.1402	1.38	unknown	342352	192597	0	0	85209	301340	39561	0	71673	0	138713	147546	79653	782468	481659	88108	140889	0	310878
fpHILIC	Negative	252.0875	1.74	unknown	421217	237934	145925	141394	114812	229402	74625	83219	87091	195489	180599	119901	87688	406404	550498	112648	170414	191381	185261
EIC	Negative	314.1845	3.16	unknown	0	204541	291205	318262	123051	56534	0	0	78109	174291	257773	246141	0	103988	145481	205432	178529	0	0
EIC	Negative	301.1802	3.15	unknown	613935	259813	926449	707639	78606	122598	172496	201589	101574	884365	332770	276775	336337	264645	123174	325964	443251	226737	273216
EIC	Negative	306.0526	2.47	unknown	636057	330407	1802316	329783	102700	130936	198953	299267	326496	804847	308958	381932	404276	426438	397618	496691	513778	469629	304562
EIC	Negative	494.2489	3.08	unknown	180848	59993	196647	127403	28798	12674	60111	61019	10007	99220	28109	11890	90069	28183	81504	38582	31604	101471	103186
EIC	Negative	339.2654	6.07	unknown	0	0	96851	0	0	115040	0	0	0	0	0	0	129907	0	0	0	0	237583	0
EIC	Negative	309.1744	3.48	unknown	0	0	0	224376	0	0	0	0	142048	0	112859	0	0	0	136936	156570	0	0	0
EIC	Negative	332.1952	2.75	unknown	1484796	3404662	1001128	2518412	1716179	2490304	3810791	692592	1706209	4551371	3765791	4986059	5593116	4034315	3805278	3520856	3164430	6639558	4429053
EIC	Negative	313.1815	3.68	unknown	2655918	1854396	4741684	5381589	1112702	957514	2070199	1542200	1058717	3417125	2441790	2982481	3871008	1819372	933992	2393952	2611934	2681392	5333793
EIC	Negative	265.1449	1.73	unknown	1290256	1423355	2083526	365874	455513	940935	324962	756728	447501	1296751	839545	1166707	498334	3180043	1642763	431607	1108210	482945	2408991
EIC	Negative	364.2135	3.05	unknown	856451	390082	2015773	664947	241297	281014	352735	388567	342267	710287	447951	630245	519779	418560	219944	502101	1337286	801087	481385
EIC	Negative	363.2004	3.92	unknown	15119669	4405569	11886795	34504401	1075702	1494115	1978481	1869547	1152444	26080128	6315584	1096760	654351	141966	2222556	6931487	673799	5078543	851340
EIC	Negative	390.2400	3.40	unknown	343517	1716191	2703354	2783240	972213	1449354	1147458	1282658	565753	1946554	2070434	1166339	1343225	677455	1218156	3150383	1576249	3930289	1402771
EIC	Negative	394.2319	2.55	unknown	291567	137711	194504	229874	46224	64222	89852	55826	73291	201739	108094	156995	109583	150098	119690	151115	146236	133554	137122
EIC	Negative	403.1322	1.86	unknown	120151	112346	29414	0	63971	77740	119652	0	82180	0	102743	156812	151156	118034	73399	195026	186993	0	263063
EIC	Negative	331.1918	2.75	unknown	6282374	15350519	4465971	11012341	7530009	10946140	16917638	3234694	7513409	20567497	16787887	22404854	24627294	17657829	17094842	15704144	14045718	30209789	19461128
EIC	Negative	323.2502	4.92	unknown	0	0	188648	0	0	0	83216	153197	0	0	0	0	0	0	0	212558	0	167421	115341
fpHILIC	Negative	351.2179	0.61	unknown	6088249	3042124	4992715	6570985	1071411	1737145	1842690	2118660	1790046	6948437	3265446	3231605	3306362	2656928	2778732	3927876	4059087	4049601	3127161
EIC	Negative	449.2568	2.48	unknown	0	15489	63326	248919	0	58130	61142	92028	42601	228441	64764	104387	136505	17798	52635	9059	127256	305848	37909
EIC	Negative	365.1989	3.92	unknown	994719	288615	796322	2341097	67046	124789	92065	136404	72738	1755329	443920	80860	70501	0	167698	466222	16270	327054	58753
EIC	Negative	461.3280	3.41	unknown	132151	36114	327626	27552	23767	0	0	151974	9708	164348	59131	23199	9422	25745	38667	83999	35024	219244	13301
EIC	Negative	315.1967	4.30	unknown	1066732	700762	1741897	1699262	242367	185496	317589	319447	225673	1283891	580755	475536	502119	273962	323075	655124	414798	776635	546796
EIC	Negative	381.2565	5.37	unknown	440863	290572	0	0	0	0	400543	0	0	0	0	0	0	0	251709	254192	0	0	343466
EIC	Negative	356.1249	2.08	unknown	176235	122585	118558	0	131146	62918	96835	0	60617	81048	211769	170313	127074	170238	198844	120772	144947	45956	237995
EIC	Negative	287.1292	2.83	unknown	0	126750	74499	0	14386	66074	72629	0	35421	96621	102097	190153	152108	76252	96980	93211	99041	25679	86506
EIC	Negative	230.0462	0.56	unknown	68082	89718	70281	28074	52499	57716	18640	30428	52397	78648	59878	73926	59693	129121	85578	60672	71091	79047	153378
fpHILIC	Negative	188.0928	0.89	unknown	1125329	314419	167238	130233	177080	327711	135594	138909	189468	207360	296742	274659	209763	541520	702325	467808	267910	282872	343146
EIC	Negative	363.2005	3.71	unknown	2392142	530800	1412275	6502531	147260	315405	145314	110021	92102	6245696	1083232	203057	84532	39904	163829	816550	167047	374397	205163
EIC	Negative	319.0606	0.46	unknown	155608	187533	113228	79643	75765	117861	74384	35755	50137	115855	123568	174262	170962	114014	206724	157039	168850	97791	124704
EIC	Negative	329.1762	2.08	unknown	1774883	830485	1830850	1354350	377258	305760	404114	323289	280497	764105	379112	629801	599533	551260	704470	823227	886492	537746	666448
EIC	Negative	411.2390	1.92	unknown	1028774	6331461	0	669834	2230412	2294670	2540592	0	2458851	630835	4889884	4916899	3802940	3816582	4102286	6510632	5994988	0	3798622
EIC	Negative	352.2213	1.69	unknown	223082	108599	167896	166097	39875	50196	74965	19307	45796	200645	78974	86617	78615	70246	68953	123728	94587	87301	118841
EIC	Negative	414.2027	3.36	unknown	1212967	698503	1401550	969848	210350	272916	388502	216450	252062	1614162	871352	567110	536559	647694	285861	728145	642916	463941	759241
fpHILIC	Negative	221.0671	2.57	unknown	93204	408688	283637	513864	177085	847865	803770	481198	647333	613495	740098	548731	421895	276709	753184	647119	2526903	1588133	863961
EIC	Negative	473.1104	0.47	unknown	494411	751915	434945	335814	263592	490496	350347	199632	289532	398470	662074	707499	650213	345292	1092710	753747	542797	440081	609355
EIC	Negative	631.4007	2.91	unknown	36528349	17913319	55201055	35271749	6692085	5675349	15061057	6803526	6785619	35645252	18874029	27218779	20448634	19791431	13189799	24600271	22362763	17986065	10412870
EIC	Negative	632.4045	2.91	unknown	16117695	7757071	24556179	15520405	2984095	2468609	6538018	3022579	3008215	15661380	8355274	11990392	9018276	8646264	5801228	10616473	10052239	7909320	4618058
EIC	Negative	330.1795	2.08	unknown	385431	180693	386163	272253	77086	78358	83156	62259	60413	99484	86252	151068	143526	126115	155261	191867	186273	91926	157989
EIC	Negative	493.2446	3.63	unknown	218356	32152	391020	86602	0	0	86054	164625	22598	112545	68001	124063	141959	76759	29067	160124	159899	201452	42199
EIC	Negative	649.4111	2.90	unknown	7343987	2522127	8641433	4289253	891975	557036	3593267	1398631	1606454	5832186	3297937	5650879	5802408	2993860	2340881	3272248	4329913	5157087	2419826
EIC	Negative	352.2213	1.92	unknown	573606	2516877	0	0	869779	922054	1058375	0	1013248	468349	2044625	1902983	1490275	1530029	1689682	2721030	2431525	0	1576339
EIC	Negative	279.1142	1.32	unknown	230533	39932	0	138800	0	59119	0	0	359270	0	67691	222150	520561	544158	30182	37941	784313	0	60039
EIC	Negative	412.2425	1.92	unknown	410993	1533171	0	0	531127	546958	607119	0	577124	0	1194590	1165670	905159	920582	958528	1609673	1470160	0	903017
EIC	Negative	483.1282	1.87	unknown	237608	216442	0	0	95938	124207	199996	0	139838	0	160194	265647	278015	208729	113456	348599	301638	0	460924
fpHILIC	Negative	236.0932	0.59	unknown	1276028	875743	554615	390866	344586	634362	291018	183003	257527	573537	529978	440523	330037	1322236	1779499	344405	415969	668644	539762
EIC	Negative	413.2003	3.36	unknown	4393239	2133360	5157551	3546516	799247	881280	1581621	825486	905507	6037895	2828127	2411076	1820757	2524064	1122541	2412361	2445205	2052597	2916431
EIC	Negative	347.1869	1.78	unknown	1315847	440543	1069086	389865	83130	147135	142001	110170	107468	1511255	446659	403091	250575	343614	218648	306627	274776	0	495243
EIC	Negative	313.1814	2.82	unknown	760541	1629211	1286148	2894322	562589	456886	686525	567933	529549	1196678	946313	1297179	1085992	933679	1334115	1229354	1196572	745157	1665525
EIC	Negative	364.2134	2.87	unknown	466482	136057	549335	317172	98233	102045	130766	108810	94627	186643	128866	171587	172859	141511	86105	174184	525070	261930	130427
EIC	Negative	647.3954	2.89	unknown	792193	329547	830880	519929	113670	49634	382128	115103	149611	524964	217725	444231	453611	273931	259363	333111	451636	455961	286943

EIC	Negative	521.2758	3.58	unknown	101485	85936	229525	146837	47641	30215	79816	63735	53111	143884	128411	115092	155718	98614	54777	83200	133331	113008	221319
EIC	Negative	533.3024	2.79	unknown	2158825	39480	2983523	2259615	0	0	33980	1169330	79183	956805	0	0	51841	73019	0	0	0	2830604	0
EIC	Negative	382.7211	0.52	unknown	400148	104330	458774	269692	0	155312	110436	34891	243924	656561	101195	97489	109894	0	115368	241508	76917	202592	566374
EIC	Negative	350.2055	1.73	unknown	1062123	244300	590215	646501	50204	86051	90113	74271	90862	1223309	246397	197211	140488	234020	132568	217172	200111	129030	187469
fpHILIC	Positive	163.0597	2.91	unknown	474941	184246	277951	63148	85399	114431	225874	67474	0	106564	68748	116025	0	703638	439681	69763	76522	208260	297399
EIC	Negative	399.2155	3.35	unknown	6030361	4007671	6551669	5147542	1373251	1487227	2631123	1477910	1670335	7965181	4873822	3864261	3545139	4400441	2115893	4284228	4341917	3360092	5066198
EIC	Negative	459.1873	3.71	unknown	110419	26012	62687	284728	0	25407	0	15097	0	289190	56684	11439	0	0	21026	42160	11958	15390	0
EIC	Negative	632.2974	3.00	unknown	72455	41015	0	62763	22399	71078	13440	25094	68510	94245	71566	112917	73870	70078	75352	91037	94969	66567	120767
EIC	Negative	493.2794	3.03	unknown	163033	32758	70084	48486	0	86511	0	0	20414	52255	25757	78184	23572	20091	68208	21504	0	29233	120477
EIC	Negative	389.2911	6.44	unknown	36037224	34703508	31677198	45834044	43112692	1716724	36073268	2006466	2030173	1659776	29528754	34153096	33038382	1988467	38333440	36841880	33061908	28463296	53675936
EIC	Negative	273.1493	2.16	unknown	2159297	1225343	4117040	2997803	125312	673679	531134	462452	290644	2100717	1174666	2739157	1114061	896364	776650	877767	1511481	436964	1072958
EIC	Negative	418.2307	2.52	unknown	823180	342427	561189	534109	154570	150824	276392	74587	195351	776219	329222	405227	374535	373800	242309	381501	307658	672282	309715
EIC	Negative	265.1480	1.73	unknown	0	1245112	49170	323795	392475	0	283634	24880	412545	0	26399	977718	437458	0	0	367346	44913	0	2094644
EIC	Negative	400.2192	3.36	unknown	1463945	968827	1505977	1322867	329404	379273	621786	345391	394678	1919109	1158907	942528	841026	1088705	508164	1045939	1025462	823391	1265444
EIC	Negative	334.2082	2.01	unknown	187055	245053	0	0	36868	94562	204167	0	51777	174088	302876	365812	138087	74357	209744	174242	209401	100897	179670
EIC	Negative	378.2377	3.35	unknown	1172742	418021	1382032	921163	177099	220723	356428	252879	240337	1979043	687558	558745	475791	591837	311668	599994	680132	491547	738729
EIC	Negative	243.1604	1.52	unknown	99600	105367	560288	234833	169513	122543	172047	299805	84203	295904	152941	121492	568141	205923	317897	117960	398738	842260	244405
EIC	Negative	557.3350	2.28	unknown	377298	0	1499109	1134560	0	0	0	1093407	9761	1347831	0	0	43200	50260	53284	21571	0	2436518	0
EIC	Negative	317.2123	2.91	unknown	8632346	5773508	11217143	0	3407450	0	0	3630454	0	5044236	0	0	6497301	6137784	5039640	6721895	0	0	4319357
fpHILIC	Positive	85.0288	2.92	unknown	690048	424691	378737	147067	128756	167032	284731	290765	98506	294502	256155	267898	278842	884399	770361	264726	262061	296230	625913
EIC	Negative	423.2219	3.92	unknown	1340476	393348	1039818	3027263	84514	132040	166725	183974	79260	2190626	590120	108048	59480	0	213823	658761	67225	453524	89756
EIC	Negative	393.2288	2.32	unknown	11681634	4777645	8814762	1208464	1243972	1560286	2367613	1892833	1523369	3825813	6211485	4422935	3699167	5420332	2690919	4396785	5349431	6464436	857137
EIC	Negative	433.2002	2.51	unknown	606683	229008	501694	695817	149739	174499	344065	265621	238976	1040894	440926	618216	563569	528527	330573	569891	444782	924507	428864
EIC	Negative	314.1846	2.82	unknown	154998	353620	290509	623850	122772	102461	145511	100497	101952	270142	204947	290272	241442	201514	282691	262286	261000	161022	365345
EIC	Negative	364.2036	3.92	unknown	3529997	1035798	2833536	8277669	225006	352906	405731	421952	229466	6249043	1569315	246730	140601	19807	538781	1774737	161475	1109290	223624
EIC	Negative	519.2860	2.54	unknown	680776	0	1026846	549277	0	0	0	278895	0	318290	0	0	0	0	0	0	0	650248	0
EIC	Negative	429.1955	2.32	unknown	1482853	466737	1003110	1581961	196211	157577	299528	366164	199183	2437112	526914	429897	420343	542221	302480	436898	525749	1028098	549883
EIC	Negative	522.3520	2.33	unknown	144610	59070	173079	572942	15751	11847	25301	25640	33787	367602	52880	52420	45196	24210	23518	45680	52180	122380	18975
EIC	Negative	316.1997	3.30	unknown	1066998	847652	2644292	2000281	672474	330024	175030	323475	200294	1429680	1285231	1294408	533636	771019	575896	950084	1239292	594698	621981
EIC	Negative	239.2015	4.44	unknown	301231	68685	334595	180605	27263	61900	126978	110517	87081	168914	133614	230901	227026	186195	119387	140561	209488	358833	140304
EIC	Negative	494.2490	2.18	unknown	648536	193191	0	0	108267	91623	245192	0	99680	1933221	198031	162756	122270	228511	90690	315434	403488	0	465732
EIC	Negative	534.3020	2.79	unknown	455797	10421	501617	460529	0	0	0	274931	26035	221840	11866	0	0	0	15267	0	15777	659411	14406
EIC	Negative	531.2997	2.79	unknown	22240738	53926	27900599	21458019	59907	83994	109685	11906674	416931	9396633	111432	88355	253002	285952	181089	78731	89662	29077542	123205
EIC	Negative	342.2732	6.06	unknown	0	0	224098	0	0	0	160920	0	0	0	0	0	0	0	0	177988	0	200232	0
EIC	Negative	511.3643	2.86	unknown	178187	69287	0	56373	74739	68761	64348	0	14557	133981	61116	55001	47543	62442	63235	87730	71938	244027	56130
EIC	Negative	627.4999	6.86	unknown	89478	287294	116815	283135	105809	108112	187582	89108	155082	291708	701595	267747	451272	965636	268501	247297	430451	101876	472923
EIC	Negative	531.2832	0.89	unknown	161770	5371	188985	179601	33149	27534	15022	346212	25857	590456	5260	0	13941	12480	14648	5585	11103	1019434	9183
EIC	Negative	364.2134	3.22	unknown	568641	192237	1956237	721078	111385	170484	181596	186884	195081	209760	191426	270683	264870	206670	115272	264640	1047017	366414	209612
fpHILIC	Negative	202.1086	0.57	unknown	890251	885429	164761	141914	353106	797020	255960	0	398267	484053	617297	486472	317641	1017792	2245586	473875	466036	463330	640133
EIC	Negative	583.3806	5.19	unknown	158161	0	247465	796449	0	332239	0	867261	191293	196201	0	0	0	0	194609	975924	294263	814690	1033503
EIC	Negative	565.3028	3.69	unknown	87853	135768	354143	222745	122774	85736	256380	238234	80479	515285	293355	240196	179870	177751	328422	233026	264597	778133	266065
EIC	Negative	552.2981	2.43	unknown	87643	0	229883	74586	0	0	0	146874	0	161842	0	0	0	0	0	0	0	350561	0
EIC	Negative	409.2225	1.73	unknown	3832646	905438	2069217	2083733	90619	140763	218103	150553	210282	4651915	807830	588970	351197	684077	303346	571976	556937	0	508246
EIC	Negative	479.3745	5.33	unknown	2057292	1406788	1637182	2024314	704653	963479	699844	1055773	815642	2866550	2890776	1989160	1731886	1682916	1853289	2432313	1908843	2375915	1587208
EIC	Negative	503.2983	4.03	unknown	1842698	997167	160727	631921	181675	273992	55575	320665	34291	173819	0	0	67716	485770	1127566	0	92074	143504	712331
EIC	Negative	507.1702	2.44	unknown	341773	70701	42023	164932	9309	24044	29112	20819	30314	281857	57189	57095	31170	56690	24739	43603	58032	101908	43980
fpHILIC	Negative	317.2124	0.44	unknown	4788285	1711637	7606033	4637844	574754	594832	1258896	892430	858011	8350926	2137096	2259541	1687500	1620230	891552	1676145	2736486	1756921	3010144
EIC	Negative	593.3695	4.04	unknown	112951	60386	89148	76547	55571	47835	43780	120388	60225	199289	161108	119927	126675	77911	103383	132391	138861	319168	96340
EIC	Negative	427.1984	3.84	unknown	0	91701	277402	158398	0	91482	183947	249306	0	285385	100068	154799	190907	191684	201595	253858	111338	513439	185533
EIC	Negative	343.2132	3.10	unknown	512015	187133	205957	157181	260193	227521	260029	158590	233213	183062	192173	683106	408656	248398	201285	1181459	969994	225697	183661
EIC	Negative	533.2901	2.51	unknown	319373	92380	190980	47401	57473	36821	90644	0	57084	208157	103115	102146	104916	90949	68874	115793	92538	195618	114234
EIC	Negative	373.0721	2.85	unknown	46981	94143	43940	0	75649	56950	78126	0	35688	76383	136574	118496	85360	218828	189919	64001	71167	82883	189855
EIC	Negative	621.3946	5.65	unknown	230350	114200	413681	154264	78075	84549	135149	146605	114486	166402	158095	133923	211849	83282	82616	259783	240686	485154	287683
EIC	Negative	532.3029	2.79	unknown	6754940	50135	8499154	6576941	0	46534	46572	3558794	102922	2805613	52679	18216	92352	115040	60873	52246	74359	8903649	26522
EIC	Negative	280.1888	3.54	unknown	491465	0	0	0	0	444440	0	0	0	0	433356	0	0	0	405432	371457	0	0	303302
fpHILIC	Negative	318.2152	0.44	unknown	1385141	411294	1784447	1239408	173643	134422	300918	157055	164836	2059000	604000	554950	472081	440322	231214	426197	586287	311761	707750
fpHILIC	Positive	308.2793	0.43	unknown	310200	194080	305679	249302	373960	224765	230532	104572	141901	107832	90957	131319	266074	302630	136337	1672443	250134	285083	272123
fpHILIC	Negative	447.1556	3.59	unknown	80498	947613	800788	88975	77753	64957	242292	160130	646042	49698	32413	111195	77638	871763	113676	80398	187091	158175	2363192
EIC	Negative	318.2156	4.54	unknown	992293	338824	1553248	813557	154654	89356	317103	335574	156554	2068325	590300	597859	340192	376855	218103	575892	730686	514290	531922
EIC	Negative	478.3619	5.26	unknown	334428	500070	319184	309302	277006	406130	330061	269462	242112	502325	1179630	653649	664972	727515	910082	778159	865330	516430	675966
EIC	Negative	445.2036	3.92	unknown	1094059	416489	931241	2328052	51404	122847	117022	163408	85985	2200257	470773	81522	51503	0	207562	596766	51370	389674	83730
EIC	Negative	351.2180	1.60	unknown	5031925	1589276	2161779	678971	301738	500998	581352	396285	372490	4093346	1202229	1019953	690889	1022182	757317	1271390	1401757	407991	1461992
EIC	Negative	554.3160	2.27	unknown	3521466	299835	14425867	8527899	221431	158624	56142	8353928	331471	14572640	756343	597698	620028	904630	1042171	523821	604720	19850178	467767
EIC	Negative	322.2101	3.12	unknown	81018	55969	82718	97251	14573	25984	35995	21841	17813	68195	37338	51857	54044	40484	59806	48900	68213	65446	44326
EIC	Negative	530.2796	0.89	unknown	525439	25973	637360	587225	86255	47521	47464	1167131	64811	1950764	20923	0	34652	32592	43411	17146	25103	3380962	24726
EIC	Negative	555.3197	2.27	unknown	1226848	106614	4830391	2813025	71493	60631	17364	2780179	113568	4761051	245091	205315	220689	285407	366947	165655	201422	6643732	154178
EIC	Negative	477.3598	5.26	unknown	1123319	1710135	998518	1020207	845758	1303946	1018666	883353	765394	1636579	4009542	2222155	2010466	2355139	2794143	2553513	2656900	1703563	2215858
fpHILIC	Positive	434.2663	0.35	unknown	207119	604162	83323	44167	274555	505888	39938	100732	128710	68008	52124	576464	66532	134339	44286	1025413	96313	722976	711014
EIC	Negative	321.2341	5.22	unknown	199950	0	0	339956	0	0	0	0	253201	0	0	0	0	249089	312267	0	315472	0	0
fpHILIC	Negative	446.1517	3.59	unknown	449616	5928653	4667445	460555	419755	390469	1299258	903747	3736496	296185	194934	528004	515648	5060622	664672	391734	1056823	777512	13847998
EIC	Negative	493.3539	3.31	unknown	215024	338392	197717	210047	175317	217422	180632	135526	156484	342901	730498	632796	438849	471150	419604	547174	430195	259998	501562
EIC	Negative	457.2602	4.68	unknown	322762	145858	376925	413232	75211	63541	116634	66838	148253	246206	136728	417831	327972	171954	235954	363336	138900	245336	208631
EIC	Negative	499.2932	3.41	unknown	7861406	0	7014761	17353770	0	0	0	9550565	0	14868156	0	0	0	0	4265272	0	0	19998390	0
EIC	Negative	375.2173	3.46	unknown	211297	91587	272192	347884	36392	33568	61483	51912	55247	523196	167049	115893	106299	102259	38099	105916	154888	73271	237746
EIC	Negative	415.2106	2.32	unknown	2271495	832687	1497377	2826565	289652	248643	512502	815187	307566	4338171	915178	690471	684517	1049004	529779	893862	853468	2341317	967992
fpHILIC	Positive	301.2162	0.48	unknown	623786	189820	954173	542264	0	62679	275806	80356	66291	1562461	385167	299592	265572	227613	52629	223972	334729	335072	301038
EIC	Negative	480.3784	5.33	unknown	601697	428216	537394	633595	163465	296955	204265	296832	280887	915221	910649	669273	494349	509095	591070	773398	604654	698643	529302
EIC	Negative	301.1802	2.81	unknown	518755	250843	495634	335958	155727	124607	245283	194868	0	303131	246430	371915	367386	261006	273411	314159	698241	321500	252084
EIC	Negative	481.2182	3.77	unknown	208409	119452	269678	366570	41610	66278	81429	58261	52034	554661	204946	135487	142521	153207	77456	104963	203986	78439	315624

EIC	Negative	319.2189	3.36	unknown	4145495	1496297	5505340	3527075	461160	557132	916500	570305	607783	7208026	2259302	1678176	1503866	1944440	720851	1762966	1975625	1520985	2135190
EIC	Negative	371.2440	4.04	unknown	168080	96542	92012	80503	148085	92418	115064	62287	106864	91581	96529	386344	198554	106948	68303	618639	483109	111890	92554
EIC	Negative	556.3316	2.28	unknown	936629	23924	4826355	3470246	0	0	0	3142117	64525	4534195	120894	73431	151028	192734	186122	75362	97651	7971598	72161
EIC	Negative	352.2213	1.60	unknown	1120260	368193	520965	152564	74102	117209	128321	0	95431	921698	265409	213870	146820	223458	169533	273886	320243	0	324506
EIC	Negative	400.2191	3.77	unknown	401505	158356	515195	694909	73960	88655	108692	122316	83607	860115	313750	237189	224068	207529	103956	193682	277845	197936	480117
EIC	Negative	593.3857	3.63	unknown	1014857	556083	1384815	1033905	229210	285836	473730	436238	139875	1966217	1236376	898297	998783	682416	251210	600430	992485	831357	1475329
EIC	Negative	317.2124	3.36	unknown	133065984	53155091	177553786	107588994	15730004	17476157	32955433	17783407	18794620	251039758	73891464	55161274	46228487	62470846	24598967	61410410	66855315	46116072	74405608
EIC	Negative	318.2156	3.36	unknown	29454914	11776013	39317866	23570309	3475234	3847270	7302487	3937756	4137000	54895049	16150368	12174842	10192811	13666595	5374465	13636419	14902720	10101839	16388836
EIC	Negative	444.9849	0.49	unknown	7176350	6115632	8270621	7883594	283136	333742	352215	320997	8931442	8169813	5975416	6914850	8293524	2342659	5422472	314359	6209151	7941434	7350455
EIC	Negative	437.1702	3.36	unknown	194535	119367	224853	267729	44387	71587	109172	75092	83518	583334	265583	187932	192954	229488	101107	239491	232196	177800	273811
fpHILIC	Negative	401.1302	3.53	unknown	65269	1176524	711074	78709	132481	132035	384288	197149	933016	55474	0	93220	109093	987799	113005	81146	279590	95345	4249991
EIC	Negative	377.2334	3.36	unknown	4443342	1603505	5739765	3599706	509064	555108	1039391	578205	577611	7692979	2318087	1799558	1420045	1968674	779650	1915532	2214407	1489039	2503229
EIC	Negative	577.3015	5.25	unknown	389775	1207284	295406	651554	421019	675426	495230	311546	386913	701653	1520210	1227333	2349904	1013297	1175276	1162396	1311805	933607	1671864
EIC	Negative	307.1944	3.52	unknown	0	294418	643079	0	0	0	0	0	216159	734097	275007	270786	0	0	0	103726	0	486844	0
fpHILIC	Negative	343.1139	3.52	unknown	69132	1512180	875759	125214	165002	193953	526241	268159	1255192	85942	56217	164663	152030	1350423	142560	109693	375797	183661	5772100
EIC	Negative	417.2266	2.62	unknown	371813	284241	422639	298276	71727	100450	143436	0	83517	714790	300129	219932	224554	265210	144206	207059	354745	0	388875
EIC	Negative	318.2155	3.05	unknown	379114	106613	861680	327497	77975	54986	87860	98827	72051	976050	315975	121751	158157	162318	92621	206551	269670	323614	185091
EIC	Negative	557.3354	2.39	unknown	459689	25608	2411920	3161525	15499	8765	0	1402135	36294	3181297	107212	97277	115741	202452	155144	71476	70790	3670986	59020
EIC	Negative	334.2109	2.32	unknown	12532996	5265640	9282483	12610844	1370369	1657307	2626669	2236417	1682535	21710495	6597181	4792480	3801336	6260234	3027137	4786893	6115751	6654404	5778729
EIC	Negative	294.1792	6.03	unknown	0	676944	0	909916	0	709954	0	0	0	0	673121	0	698102	605910	0	966847	0	0	991396
EIC	Negative	320.2215	3.36	unknown	420706	143540	506299	300725	37397	46843	83167	45051	56712	726129	191480	156374	116210	159253	61956	190772	175050	119108	198379
EIC	Negative	556.3324	2.39	unknown	1401055	87272	7216068	9464196	41830	32442	25484	4117973	109194	9353239	339522	260851	365959	620716	446554	184193	236461	10995895	171767
EIC	Negative	418.2306	2.56	unknown	452588	203915	176023	324995	57342	72907	87607	74587	57499	697611	238203	167906	149176	232601	107418	183616	250755	101062	330898
EIC	Negative	394.2317	2.32	unknown	2356084	1002981	1773670	2447566	272504	304963	488417	425430	298389	4227455	1345522	937130	722920	1182408	571099	923697	1133833	1284943	1084529
fpHILIC	Negative	196.0285	1.65	unknown	0	1145388	0	738515	0	0	930748	0	0	0	1314312	978079	0	1963276	1480126	1119332	0	0	0
EIC	Negative	298.1897	5.23	unknown	1989241	734497	2848051	1556023	421934	283591	586919	541874	317103	2413936	1315053	1257879	1639458	938190	461359	1129501	1345220	1001525	2097499
EIC	Negative	393.2286	2.32	unknown	9792298	4062238	7413516	10023741	1083550	1281572	2026913	1654430	1271201	17152084	5288002	3781332	3040465	4813064	2300021	3670972	4692837	5376029	4511147
EIC	Negative	416.2288	6.31	unknown	503860	1607918	223916	89780	810653	1341184	78614	373994	353807	177217	156261	1599981	254595	356913	191013	3640298	325204	2188686	1989814
EIC	Negative	392.2172	2.48	unknown	319786	274850	179915	350946	277433	14535	0	0	23540	205774	275075	283352	0	0	294223	273586	304983	0	16724
EIC	Negative	415.2256	6.31	unknown	2156457	7147602	1127041	734511	3654924	5511862	453050	1557153	1872704	841464	695306	7079029	1272665	1473746	773709	15638416	1261286	9815596	9315981
EIC	Negative	364.2038	3.72	unknown	538734	135867	347127	1407586	0	69944	35099	0	29221	1455436	254035	46305	0	0	40224	203641	0	79995	56002
EIC	Negative	381.2565	5.44	unknown	164350	118693	0	0	0	0	165134	0	0	0	0	0	0	0	112519	203519	0	0	151380
EIC	Negative	297.1865	5.23	unknown	9352001	3582844	13002719	6649572	1613882	1313690	2944177	2304619	1375002	10990205	6023978	5758637	7347202	4298937	2104112	5030012	6383456	4198167	9340088
fpHILIC	Negative	342.1124	3.53	unknown	331284	6949079	4239270	471153	728478	791619	2505636	1167524	5196321	345728	227588	629058	621502	6237731	565510	466857	1536621	668371	26198768
EIC	Negative	333.1356	2.96	unknown	322726	25367	396131	45095	12955	21285	10756	45201	26713	66040	36432	0	65812	0	259483	0	40496	0	31284
EIC	Negative	297.1720	3.84	unknown	115983	77681	116210	77649	20231	33590	53821	0	52557	84300	96023	117954	162665	103583	151309	101181	80999	51374	69550
EIC	Negative	385.2383	5.69	unknown	902730	309776	745539	469160	254171	184615	274215	501470	136787	860037	780855	490884	336014	389516	1012502	718956	591289	953551	590377
EIC	Negative	471.3488	5.84	unknown	301353	106497	520615	318083	26117	84214	66701	114604	58114	398483	167770	211476	134244	69933	409957	168555	157144	100263	165482
EIC	Negative	433.2210	1.60	unknown	359463	136649	0	0	25810	57372	47638	0	31759	274071	108607	109188	68733	96462	73458	105451	146179	0	140654
EIC	Negative	534.3884	3.37	unknown	66888	51103	206847	800469	0	12737	20863	33613	37397	315125	73423	98909	36412	15531	0	51976	71234	105436	71096
EIC	Negative	554.3158	2.79	unknown	1663266	0	3786176	7863643	120092	131700	0	4357974	328163	4723282	526450	443310	371720	701076	610641	371160	474607	12294836	448439
EIC	Negative	317.2122	3.05	unknown	1493576	582539	3918054	1528109	263587	296903	382293	354452	325170	4301725	1443349	582194	790967	711557	429143	869334	1188153	1302010	882061
EIC	Negative	318.2157	4.53	unknown	1124233	357302	1554061	909948	235376	100874	317103	147840	128235	2116299	582344	672675	193426	400884	136228	599955	789076	569888	589351
EIC	Negative	629.3853	2.89	unknown	1565274	815366	2111879	1452715	301522	89719	413202	268650	172253	1063966	493237	774204	631783	657962	548137	1023147	1006631	642489	379081
EIC	Negative	555.3184	2.79	unknown	605721	15087	1322960	2793058	29256	34247	0	1514896	123376	1604210	178412	170189	142433	243830	204781	143404	184983	4249154	157952
EIC	Negative	546.3193	3.25	unknown	1236729	0	2786954	1367831	0	0	0	830655	28839	1687445	11356	0	30093	33682	0	0	0	2492023	9705
EIC	Negative	336.2265	2.62	unknown	2080878	844068	2206113	1444343	188021	290204	391324	175814	250902	3037514	969417	743153	656248	866275	489465	717793	960584	440586	1164718
EIC	Negative	415.1919	3.36	unknown	586961	81688	792802	674142	132006	138185	273900	175223	177470	1873072	661531	517626	445106	634721	239629	615315	631481	418883	714999
EIC	Negative	309.0626	0.49	unknown	628240	326521	0	0	0	238508	0	0	0	0	0	0	236951	0	0	367221	0	0	330574
EIC	Negative	635.4327	4.14	unknown	451170	309122	495547	343052	46601	42963	131897	74800	77237	178807	135868	404888	337205	141860	306225	252312	218252	177098	150306
EIC	Negative	415.2109	2.44	unknown	3156794	750498	2814752	2409655	231880	301311	479367	523546	394864	4754524	1117167	709340	685498	1149275	401420	1073209	1123001	2145737	993337
EIC	Negative	545.3164	3.26	unknown	3908421	0	8737261	4143546	0	32721	0	2500667	48399	5332976	32168	11161	35638	37261	36987	0	12677	7607535	29113
EIC	Negative	362.1976	3.12	unknown	1195001	269821	5784176	1957148	171969	154508	235208	330644	96167	268870	95035	174198	119424	135045	143133	155609	2065495	401571	181029
EIC	Negative	446.2042	3.92	unknown	322386	27548	177495	565424	14236	16981	39258	0	16487	499065	142008	0	0	0	38917	126001	0	94382	0
fpHILIC	Positive	361.1530	3.53	unknown	81430	3119103	1731115	226750	333928	371761	1263413	478691	2576972	171488	59093	254096	305984	2867521	219780	227077	607500	305252	13733135
EIC	Negative	315.1600	3.68	unknown	84609	0	441264	0	85607	56125	29587	41405	22412	0	593630	23053	555964	0	14177	46159	647679	0	0
EIC	Negative	601.3346	3.03	unknown	495204	0	0	0	0	0	0	308240	0	0	0	0	0	0	0	0	752127	0	177065
EIC	Negative	627.3178	3.26	unknown	132014	0	288390	132800	0	0	0	96034	0	207910	0	0	0	0	0	0	0	289980	0
fpHILIC	Negative	217.0638	3.62	unknown	609887	203059	249884	135282	80564	108406	65394	50107	51227	171068	128958	148594	121998	124358	405774	205130	132278	252822	80003
EIC	Negative	402.1895	3.35	unknown	136819	60448	160146	112214	15648	0	37948	20300	17593	163699	38706	70335	42538	56257	36101	59510	72505	29564	79080
fpHILIC	Positive	145.0492	2.92	unknown	1344438	568688	375217	73919	97107	132221	373982	226175	95137	212195	80293	91186	0	1748539	1429646	105813	211078	245873	807144
EIC	Negative	558.3339	2.38	unknown	150642	0	813917	1041608	0	0	0	432761	0	996527	38004	8273	0	63637	50721	22798	23439	1191522	21867
EIC	Negative	521.3493	2.33	unknown	329858	126474	492979	1579854	69650	25302	41145	130025	38890	956264	187981	151751	122167	81336	58701	215091	138849	401420	112276
EIC	Negative	477.2484	4.24	unknown	960714	199443	1713383	694304	274015	103518	395463	298292	217121	2907751	432444	252974	509756	677057	281437	554033	1047968	1102984	928354
EIC	Negative	416.1941	3.36	unknown	91360	0	191419	88790	0	31879	82729	19680	38705	451945	165911	106637	110164	151356	32346	132840	131188	78535	151041
EIC	Negative	416.2145	2.44	unknown	782268	176017	642496	598422	57338	61790	115841	80890	88687	1160704	276503	186265	132018	254615	93743	228551	248816	423882	239285
EIC	Negative	301.1807	3.45	unknown	107311	59239	133803	91938	35834	29063	51176	0	0	63963	67672	73210	74023	49904	65873	85978	65606	79741	57070
EIC	Negative	335.0600	2.35	unknown	833696	173601	1372999	543898	51612	71246	114696	141032	93192	591408	153073	250811	244808	212983	269332	333442	240910	440148	163812
EIC	Negative	380.2083	2.98	unknown	165608	55793	541979	194641	24916	36291	43732	28751	25153	65649	55538	60367	61365	42952	16806	44523	306712	75930	44658
EIC	Negative	315.2537	3.33	unknown	166464	0	189801	2553310	0	0	0	0	2227390	171108	0	2252667	2375267	2220900	0	0	2379158	2312055	2216844
EIC	Negative	423.2269	3.71	unknown	145614	49481	95792	99643	0	26080	0	0	0	420179	71994	10101	0	0	9222	54577	0	17634	0
EIC	Negative	314.1848	3.42	unknown	151884	110398	269422	245660	18728	32870	67557	60509	20495	493412	238782	140613	72964	152824	68973	114824	137778	46472	262076
EIC	Negative	376.1879	2.77	unknown	377508	56756	179075	182057	0	716101	0	73333	0	729633	994171	0	899266	0	954974	740294	0	792362	0
EIC	Negative	379.1896	5.23	unknown	557667	165121	791610	359434	96234	53359	138414	158068	62847	815991	357410	345023	433943	201141	155575	287631	356692	239482	565867
EIC	Negative	475.2337	3.60	unknown	1073953	216091	1644613	859448	153484	142387	325172	398141	173124	1338230	417681	346325	555053	672892	222132	398115	931058	949208	1262833
EIC	Negative	399.2155	3.77	unknown	1224793	652744	1837328	2169612	196925	209774	425671	297338	253274	3299248	1134046	833434	816643	795675	315286	572852	1080613	447761	1976835
EIC	Negative	328.1905	2.20	unknown	65258	118923	91230	253442	79958	121498	55047	0	18396	183565	91172	164024	145838	73314	89758	130583	335464	180776	138193
EIC	Negative	444.0835	0.52	unknown	91362	56739	83046	29294	19606	77808	24269	0	25880	97812	61809	47382	31846	48808	64601	180169	74731	20930	129535
EIC	Negative	430.1993	2.44	unknown	541405	123595	580488	366037	37761	46205	44817	52353	54422	941032	173284	100107	26586	155557	48570	141966	158334	255839	146886

EIC	Negative	393.2286	2.44	unknown	9955067	2385420	10599369	6452590	556385	766513	1197225	935004	918612	17463809	3599208	2364954	1772343	3416825	1015307	2881799	3496602	3433995	3161406
EIC	Negative	334.2109	2.44	unknown	14797171	3491393	15763421	9489561	859059	1054326	1792861	1401598	1382805	25683097	5388526	3433050	2557040	5163482	1565924	4398188	5339182	4926429	4638357
fpHILIC	Positive	360.1497	3.53	unknown	794857	22525682	13514578	1255584	1835191	2240935	8260221	3176757	17786320	905324	568344	1964193	2023313	20852010	1706093	1468073	4455295	1737724	98346760
EIC	Negative	315.2538	3.45	unknown	192801	0	299552	953131	0	0	0	0	901781	79606	167920	1015629	967489	1012069	0	0	985508	923893	1013525
EIC	Negative	445.2007	3.92	unknown	1110018	356665	786502	2240266	13863	118535	24859	82256	87414	2065974	322072	75336	25363	0	108515	519752	16967	363868	57615
EIC	Negative	630.3858	2.89	unknown	778953	422187	983330	733733	117146	17448	203571	115102	78938	539602	184815	375064	282873	236017	255086	502618	506418	310594	166959
EIC	Negative	354.2352	3.31	unknown	63774	55238	70667	53839	46113	72131	58045	59388	57832	51513	69396	69971	63878	14829	53298	724393	62975	55928	65085
EIC	Negative	314.2420	6.40	unknown	7431503	3443869	7254149	409892	286087	3804534	443805	428038	415640	5070975	2997314	2989659	2680032	136083	3553015	2741176	2924481	5798679	2666562
EIC	Negative	413.2003	3.77	unknown	551277	244183	960064	968055	77631	99799	150887	109841	114652	1556200	500596	357601	342293	355576	129826	132709	460773	178917	918142
EIC	Negative	394.2318	2.44	unknown	2419040	587221	2656593	1561386	135945	182605	291508	227396	218911	4213260	872418	582755	424740	845042	239921	714332	883303	883384	783226
EIC	Negative	314.1845	1.88	unknown	0	326213	0	0	0	454586	0	0	0	0	0	0	0	0	503875	487640	518504	0	0
EIC	Negative	469.3320	5.49	unknown	790758	255987	1161561	833690	114948	121034	121983	181203	108064	1264931	410543	528821	335768	254580	923187	337008	309928	194437	412776
EIC	Negative	469.2961	6.00	unknown	1106541	1090291	1368720	1480701	215321	107731	239994	206994	143217	835913	555139	683243	251186	389268	580729	933045	237111	987966	338862
fpHILIC	Positive	599.2869	0.35	unknown	80857	135985	408483	249959	86870	70197	106033	206045	61356	446051	225587	249247	122840	230268	64565	130349	256460	1291636	46127
fpHILIC	Positive	171.0760	1.64	unknown	174647	1470022	0	1622308	0	0	1704657	0	0	0	1919947	1994448	0	1606922	2201375	2818002	734943	0	0
EIC	Negative	516.3085	3.75	unknown	3584329	15939	4492251	2759386	0	35090	29408	1076937	59613	1379306	38109	11569	64252	96775	50473	0	13522	3953753	54578
EIC	Negative	397.2513	2.56	unknown	418572	203265	353215	330110	35096	49958	64153	26675	28861	716576	169780	108455	122469	139460	95816	134430	168633	56470	241650
EIC	Negative	429.1960	2.44	unknown	2078783	424324	1997862	1397869	106954	120432	121639	216841	197078	3249886	632098	214193	258276	586774	117917	509305	617665	832330	521261
EIC	Negative	633.4078	3.76	unknown	888959	0	114773	1113458	0	0	188576	0	0	0	0	0	15303	47267	0	298048	0	345073	0
EIC	Negative	313.1815	3.43	unknown	713647	407150	1145333	1148133	131743	158658	174532	181524	167798	2293685	1092304	667588	369170	572653	294189	444485	645117	173814	1221217
EIC	Negative	407.2061	1.92	unknown	0	138895	0	0	82387	39516	0	0	49971	0	138520	164645	265630	74811	162078	133989	171741	0	123758
EIC	Negative	318.2156	3.77	unknown	3548826	1616349	5819517	6285135	503648	560432	995138	739925	620268	9393547	2985526	2084626	1996597	2067352	732901	1519407	2801821	1229340	5579801
EIC	Negative	322.1778	3.43	unknown	480669	453569	525062	0	0	574817	0	0	0	0	612925	0	0	0	338350	0	633562	982394	51762
EIC	Negative	431.1845	2.44	unknown	604861	109046	665043	586269	54975	65695	101443	147149	85811	2670571	331437	216633	168379	338870	127803	319129	363104	479239	301831
EIC	Negative	335.2232	2.56	unknown	37198661	12423423	24884391	26558503	2513936	3677927	4955763	2561336	2953817	57790513	14998578	9929221	8849383	12917015	6553237	10949034	14454893	6257207	20427009
EIC	Negative	628.3532	4.93	unknown	0	8576817	0	0	0	0	0	0	3422330	0	0	0	8760829	0	0	0	0	0	12094239
EIC	Negative	336.2265	2.56	unknown	8312192	2742705	5459568	5925994	543857	815325	1117306	544226	650798	12849892	3307182	2154759	1953429	2862810	1441381	2415213	3214406	1401549	4506035
EIC	Negative	335.0233	2.14	unknown	123132	72890	156386	0	27226	279921	42618	0	10179	96425	634417	319838	158423	142556	68098	164341	837073	80443	125466
EIC	Negative	634.4181	3.76	unknown	367518	107372	1070902	555712	17365	39047	75063	50995	40460	500818	118061	158398	165969	145123	58583	141093	199666	139964	255599
EIC	Negative	397.1999	6.36	unknown	0	131135	571390	0	228999	0	0	0	41532	392233	286283	0	241587	0	171509	256662	216734	320044	0
EIC	Negative	495.3694	3.73	unknown	88128	83467	94288	164273	47197	85067	50902	75855	39323	508645	298485	132217	96952	138726	156659	259379	125796	395898	130064
EIC	Negative	410.2258	1.73	unknown	963749	210667	488960	689843	21791	24816	12595	64758	31087	1321625	192172	180013	109684	178060	78301	169519	151846	0	156072
EIC	Negative	334.2108	2.26	unknown	555174	155736	2894221	3516130	256543	70140	542987	277364	83741	8616442	171952	1030414	661589	1434798	493961	166349	146466	1365081	2276579
EIC	Negative	316.1999	3.46	unknown	1657681	760786	2240968	2648608	190394	183177	382385	257296	304180	4629709	1333763	970240	765699	874029	339685	769310	1116823	482441	2066216
EIC	Negative	633.4164	3.76	unknown	1007460	239782	1891035	1319819	40631	59323	201184	123125	76158	1041382	314506	399900	407955	295533	71683	317121	446077	406567	576584
EIC	Negative	559.4022	3.36	unknown	184400	56850	199575	120317	0	0	18112	20141	0	139249	64207	56368	24496	55131	0	31750	80461	0	27139
EIC	Negative	369.1855	2.33	unknown	804883	319947	673176	787430	41315	86906	34228	58261	75660	1615130	294696	79264	52426	368057	93356	81634	64201	540920	300862
EIC	Negative	315.1967	3.45	unknown	7399665	3385179	10144624	12471082	845875	859785	1688468	1089782	1286812	20897710	6043273	4287382	3565041	4124357	1510201	3314311	5178036	2012171	9506921
EIC	Negative	509.2386	1.59	unknown	1556164	395343	1457695	633651	272179	0	453491	215547	288245	4242346	633687	470659	344181	780185	256996	909277	1480439	956282	1012803
EIC	Negative	395.2441	2.56	unknown	10737298	3595226	6748250	7713670	684603	1012327	1323534	633586	809796	16746074	4274874	2672243	2437056	3573652	1831234	3153613	4093872	1788873	5948379
EIC	Negative	417.2264	2.56	unknown	1809515	809290	635148	1075700	184813	251949	333575	108535	209273	2793282	973289	666531	618647	875529	462038	726618	996962	523085	1401743
EIC	Negative	479.2650	2.33	unknown	1322025	296563	2719707	2854078	75554	97575	127891	157929	89274	4594125	646262	386967	217542	310747	107711	389384	441367	470125	669885
EIC	Negative	528.3011	2.36	unknown	1412204	7399	2330039	5369256	23297	32373	0	2719472	78976	5309966	123444	77232	168848	310851	158112	88228	147454	10358379	105076
EIC	Negative	431.3015	3.03	unknown	0	141630	0	0	0	0	0	0	143372	0	59041	134862	131942	0	0	142839	127752	145374	130529
EIC	Negative	397.2000	6.23	unknown	0	206377	166105	0	194668	0	0	0	0	0	127430	0	219950	0	100907	290803	194546	163394	0
EIC	Negative	315.1825	2.36	unknown	615076	112535	92454	89700	155936	98806	163923	101693	109717	86483	155843	294461	52750	227961	215640	465986	1604869	99665	97596
EIC	Negative	396.2471	2.56	unknown	2624333	869739	1579205	1859322	166805	254332	308656	108315	196542	4155022	1059679	660307	614440	853201	435790	768067	1009416	434491	1442181
EIC	Negative	470.2991	6.00	unknown	351607	345360	457284	488919	74068	0	89249	65930	26174	285043	167788	225853	76714	107066	185449	314917	101099	314258	99241
EIC	Negative	397.2000	3.46	unknown	555977	307054	742696	807019	74511	70792	141930	85747	99578	1570166	495704	348891	287922	363591	136489	273123	445055	163963	863049
EIC	Negative	335.2230	2.32	unknown	1839443	186328	1409057	1815244	100430	129032	142289	377141	254190	0	969460	691944	560589	0	495277	719732	890217	1082461	859241
EIC	Negative	377.2337	3.77	unknown	784192	170802	1382020	1465741	130383	94736	230414	59086	153542	2252823	689597	490639	501399	462486	89816	355752	620975	283294	1347532
EIC	Negative	427.1740	2.24	unknown	61043	0	67265	646110	59118	93010	49610	0	33866	0	178863	325233	22816	187917	0	60445	170810	691863	28701
EIC	Negative	299.2017	6.10	unknown	7723765	1122817	14532295	5323031	449796	285143	1164023	489945	468888	21680212	3519210	2898628	2701833	3019180	395964	2862070	0	2224609	6207180
EIC	Negative	346.2029	4.98	unknown	2031043	515109	8992914	2198396	240620	201579	351091	266018	201225	770530	356008	603927	540803	389890	407121	695764	2700692	799692	560746
EIC	Negative	432.1882	2.44	unknown	140030	27102	146671	152859	18231	12769	26595	23425	24737	580269	97363	74962	34097	80174	27944	89214	90120	146394	80090
EIC	Negative	433.2001	2.56	unknown	305923	101841	278675	354640	0	65988	84148	43314	44573	1090415	234523	182930	148818	222820	111127	204470	243992	72533	359455
EIC	Negative	289.1812	2.51	unknown	127306	52350	226312	196379	7354	14815	43012	0	10572	220083	130586	107347	90458	46834	68426	73222	64793	30164	81506
EIC	Negative	332.1924	3.58	unknown	133209	62999	0	154881	0	0	18973	0	0	0	27393	0	27760	0	0	0	80758	0	0
EIC	Negative	628.3534	5.13	unknown	0	2410764	0	0	0	0	0	0	835182	0	0	0	2473812	0	0	185165	0	0	3381042
EIC	Negative	299.1999	6.10	unknown	7958637	2250668	15703605	5460479	514201	328153	1164023	603020	979184	22119754	4351029	2906858	2934200	3375036	402912	3222646	3419161	2224609	6257785
EIC	Negative	414.2031	4.28	unknown	200189	25456	231995	135562	0	0	0	44245	23399	239007	80793	38789	48613	38315	37275	42517	108600	70289	37667
EIC	Negative	495.3695	2.68	unknown	108180	44871	117756	113336	0	77777	16600	0	15845	237481	154376	95559	76296	80532	98790	89478	72809	140502	82320
EIC	Negative	477.2491	4.30	unknown	417765	80044	794857	303778	109510	0	56062	107303	25454	1689884	143166	83990	129385	163951	66434	139002	279267	354259	502033
EIC	Negative	445.2027	3.71	unknown	201912	51688	124677	503996	0	31573	9546	0	0	560804	106883	13951	9895	0	22639	83507	9639	40237	30697
EIC	Negative	476.2348	3.60	unknown	306630	60748	446168	245589	26077	26483	59312	107182	0	380462	111807	103143	182124	196685	48378	115397	253149	271883	346709
EIC	Negative	487.3077	2.77	unknown	213399	119330	79956	0	34440	87358	0	0	156998	192134	269443	312662	318264	710835	272452	186107	463872	190437	210888
EIC	Negative	483.1344	0.49	unknown	67264	843522	535018	71049	68657	85358	263068	39916	578785	51673	141513	0	0	279235	154227	59100	305143	410165	4019640
EIC	Negative	431.2101	2.56	unknown	1076626	411149	0	704714	58296	94028	105156	0	97778	1577774	493987	317951	267920	411238	215902	243041	456521	197935	638059
EIC	Negative	461.1816	3.92	unknown	150908	24955	126293	416466	15252	20751	0	0	17165	442155	81817	0	0	0	40747	122935	0	91967	17166
EIC	Negative	398.2029	3.45	unknown	144425	75536	202690	208467	11967	16417	43629	18451	0	414330	91024	45481	84325	78127	38018	84971	99448	44479	198895
EIC	Negative	532.2784	0.88	unknown	33466	0	59961	60025	0	0	0	56465	5058	183178	0	0	0	0	5795	0	0	320909	0
EIC	Negative	266.1482	3.72	unknown	196165	0	0	80185	91620	0	0	0	0	150030	82259	142730	125211	12625	101554	109992	93396	171895	12357
EIC	Negative	639.4114	3.91	unknown	312076	0	426623	360155	0	0	0	200657	0	643776	0	0	21066	18957	17173	20716	23715	1016804	0
EIC	Negative	300.2044	6.10	unknown	1656240	491652	3197527	1165912	103950	84824	236022	0	113984	4608831	1000050	675444	593002	673237	114322	602943	725609	305576	1359821
EIC	Negative	399.2155	3.05	unknown	133877	37835	213556	56023	32982	0	16797	21474	0	305080	126722	72966	75441	65123	42624	86130	89535	109079	86459
EIC	Negative	517.2875	1.49	unknown	22836036	320579	25459110	22883406	518845	608163	252035	0	3399005	16091876	912086	455281	4306520	2546201	1356658	623275	1009866	25144694	746742
EIC	Negative	509.2388	1.01	unknown	243907	53609	348072	236737	14381	29332	43094	0	29901	1095872	137265	51152	37918	95436	23925	145718	166897	105853	278201
EIC	Negative	470.3353	5.49	unknown	289570	112340	355845	298901	0	31945	26417	69977	0	418970	143036	193623	104634	100375	313803	149010	103127	77064	171774
EIC	Negative	274.1524	2.16	unknown	403182	275238	765150	527749	0	139845	0	0	0	378926	210970	513718	194162	176430	168863	214442	285796	51769	167343

EIC	Negative	311.1654	3.30	unknown	44371	206479	1454443	1573671	46412	219690	78047	90756	64320	88846	178829	101828	93159	136549	50910	206240	245384	104386	2576610
EIC	Negative	480.2679	2.33	unknown	397575	105141	764824	809841	22932	23314	36683	0	9892	1294583	177722	115321	72339	109682	35130	125199	120690	105586	206730
EIC	Negative	299.1924	5.23	unknown	258283	68548	337652	179440	27751	28971	44004	0	0	299646	157982	193436	210336	109640	44082	132783	139397	95345	257185
fpHILIC	Positive	164.0914	2.36	unknown	541911	467074	2175624	298999	801101	378507	659552	532393	734621	546981	525112	1101553	471299	591063	743478	433558	878436	1194269	17517050
EIC	Negative	395.2353	2.44	unknown	459346	131246	575623	310538	35126	0	65385	0	0	810335	165982	136303	0	165944	0	172709	172804	179573	151399
EIC	Negative	429.1672	2.24	unknown	511608	120166	789379	555694	93443	91423	48775	0	34381	852754	220014	444420	260963	226078	219227	286675	216083	860654	226826
EIC	Negative	381.2046	6.10	unknown	582545	198040	1070906	404396	0	0	49516	0	103786	1465964	420154	260855	273238	241843	0	208070	284207	98429	463591
EIC	Negative	636.4360	3.36	unknown	4771382	694694	7750630	3284675	71732	113295	285984	118212	51036	15479494	1362283	773332	509984	1059828	196605	942390	1267370	533012	1468497
EIC	Negative	640.4653	4.76	unknown	130565	0	0	964687	0	0	0	235937	852936	0	35781	282157	1513264	800369	105139	73020	4632652	2611231	96357
EIC	Negative	251.1323	2.74	unknown	285317	0	0	0	0	0	0	0	56986	0	0	0	208922	0	0	210607	185328	0	0
EIC	Negative	635.4326	3.36	unknown	10785813	1556868	17780805	7395964	148786	222268	655300	183588	191692	34951945	3072615	1791238	1129651	2255281	332888	2147443	2783725	1187820	3343195
EIC	Negative	347.2064	4.98	unknown	420418	101802	1930871	463461	29444	19912	77831	0	31646	147874	78237	123256	108604	79132	78205	174921	576552	172343	115889
EIC	Negative	411.1844	3.46	unknown	247711	120925	353781	357939	0	0	50516	0	38899	705710	231930	159399	0	154038	58749	66437	172773	60025	347202
EIC	Negative	251.1323	2.07	unknown	224376	86186	56812	0	0	0	32450	0	0	0	0	34227	244711	0	0	0	258836	0	0
EIC	Negative	424.2248	3.91	unknown	342281	68400	271015	817406	0	0	16852	0	16840	618616	129726	0	0	0	0	154968	0	116136	0
EIC	Negative	282.1740	3.22	unknown	0	311799	0	0	0	0	0	0	116447	0	265083	0	0	0	0	320138	407883	0	260126
EIC	Negative	571.3145	1.71	unknown	146130	0	671807	841929	0	0	0	415131	0	1193967	0	0	0	0	0	0	0	3570907	0
EIC	Negative	570.3111	1.72	unknown	400136	0	2084357	2515706	0	0	0	1207706	20647	3710364	0	0	0	0	0	0	17590	10593937	0
EIC	Negative	572.3196	1.71	unknown	30625	0	249350	255554	0	0	0	237063	0	427091	0	0	0	0	0	0	0	2073869	0
EIC	Negative	479.3743	5.49	unknown	0	26805	37077	119490	49072	64263	37167	0	60655	536310	476321	243724	165381	0	380184	571678	248381	492409	213723
fpHILIC	Negative	684.2285	3.54	unknown	0	515462	206059	0	0	0	112661	30251	326849	0	0	0	0	437582	0	0	53364	0	4256775
EIC	Negative	580.3621	4.34	unknown	0	0	0	9519199	0	0	0	0	2501849	0	0	0	10282499	0	0	10248067	2555014	10203502	0
EIC	Negative	412.1857	3.46	unknown	81222	38191	110605	103890	0	0	12976	13861	0	204634	63906	46329	40440	30535	7262	57460	63879	9464	109205
EIC	Negative	526.2845	5.23	unknown	0	2894490	0	6038035	0	0	0	0	1410062	3729245	4196077	0	0	0	4641069	1334551	3288131	0	4296532
EIC	Negative	265.1446	3.73	unknown	102667	179101	101905	133184	0	69066	57337	39564	45999	1006092	623286	0	60630	38028	675534	61970	53265	1249379	45160
EIC	Negative	581.2404	3.93	unknown	68999	62010	272546	364309	38106	0	0	0	0	46360	168494	0	73259	0	0	50997	0	326764	0
fpHILIC	Negative	683.2262	3.54	unknown	0	1640576	696453	0	0	45395	264106	69795	1118891	0	0	0	35964	1387108	0	0	149379	0	14822663
EIC	Negative	479.2651	2.27	unknown	372268	127524	427989	3450639	24845	0	41088	0	45650	141963	34852	14160	0	14745	0	59245	22800	41720	1001316
EIC	Negative	501.2932	2.64	unknown	7429882	51946	0	0	98607	0	93843	0	408494	0	143212	170278	267469	333777	198970	117022	151142	5879353	131711
EIC	Negative	632.4044	6.10	unknown	0	463533	0	0	0	0	80280	0	0	268216	139632	0	271063	265006	301579	39312	312325	229857	0
EIC	Negative	428.2039	4.98	unknown	131099	0	547785	121488	0	0	0	19755	0	51017	0	26883	0	0	0	29499	148241	52309	0
EIC	Negative	364.2134	3.65	unknown	889980	154237	3437602	1161847	30939	88544	0	0	33555	219311	47997	19360	118914	19990	128155	0	1539579	70251	141590
EIC	Negative	282.1740	3.39	unknown	0	215921	0	0	0	0	0	0	61428	0	197105	0	0	0	0	126931	277117	0	394739
EIC	Negative	637.4394	3.35	unknown	1171744	161534	1897394	785403	0	0	59577	0	15150	3767330	328836	206386	162363	270781	42112	306401	310630	24490	390123
EIC	Negative	636.4358	3.77	unknown	177626	49081	400010	458791	0	0	18116	0	14956	1022872	121574	92145	72345	10967	0	0	92283	0	388379
EIC	Negative	464.2060	5.66	unknown	128261	29403	139373	103429	26863	0	0	0	0	92820	0	108762	139704	27599	81359	16687	124570	164823	19175
EIC	Negative	428.1829	2.30	unknown	0	257780	0	0	11306	0	0	0	24030	333898	220664	329004	283839	254134	247775	35432	220925	34369	0
EIC	Negative	555.3503	2.91	unknown	3257812	0	2233871	5130930	24979	0	0	0	25697	0	0	3437878	0	16893	13288	0	0	0	766834
EIC	Negative	599.4099	5.65	unknown	0	0	204267	0	34510	0	0	0	0	109893	31042	0	152985	0	0	124363	134976	251339	139411
fpHILIC	Positive	166.1069	2.60	unknown	1245583	257179	4332727	535492	747177	494652	461366	650208	711665	1252206	349637	1386975	383162	578347	2186671	414530	1361437	6572551	48006396
EIC	Negative	473.2701	2.62	unknown	0	343596	0	0	12931	0	0	0	16411	0	11027	283369	310510	22267	9361	245410	0	0	318433
fpHILIC	Positive	210.1333	1.72	unknown	1475327	507375	11335790	1236704	1459450	714531	607022	899924	941189	2342490	600903	2463277	704853	733811	2238216	679658	1923422	4193150	82626032
fpHILIC	Positive	211.1369	1.72	unknown	122351	43894	1027259	106291	127476	59914	52987	73094	88410	211800	63161	236831	56954	63808	217136	64108	147165	386601	7509643
EIC	Negative	575.3950	3.76	unknown	0	157114	0	0	0	60269	0	0	0	0	0	112222	0	0	0	216028	0	400083	671963
fpHILIC	Negative	200.0696	2.61	unknown	242369	33461	834871	89726	150561	74821	67261	116340	118236	250956	88876	237566	65090	112318	408304	102625	300398	1142479	9357333
fpHILIC	Positive	329.1724	3.58	unknown	145281	0	210214	87001	92033	82708	0	0	0	1373037	456670	704120	323289	0	433902	614073	299915	2402738	517755
EIC	Negative	635.4325	3.77	unknown	409602	105095	1058645	1088863	0	0	44625	0	0	2346988	254056	154323	145276	139639	13679	120732	249606	28490	933056
EIC	Negative	381.2563	5.72	unknown	0	338187	0	0	0	0	17424	0	0	85708	0	0	26128	0	223722	12251	0	0	351692
EIC	Negative	414.2029	3.77	unknown	199186	50066	289534	282716	0	9896	0	0	0	440338	140534	86450	91283	106243	32318	59300	141081	0	268760
EIC	Negative	477.2484	4.13	unknown	174448	0	405060	317520	0	0	15140	0	0	1596892	101010	0	52202	100046	0	129968	210069	219188	266854
EIC	Negative	241.0507	0.69	unknown	0	68769	70669	0	0	0	0	4240	0	0	59396	53309	65901	2697	70343	88797	0	0	77540
EIC	Negative	282.1739	5.18	unknown	0	295300	0	0	19701	0	0	0	0	0	0	0	361806	239419	204333	219789	265068	400181	222881
EIC	Negative	501.2845	2.85	unknown	0	0	0	0	0	372142	562479	0	0	0	0	0	198392	0	0	0	0	0	0
EIC	Negative	362.1918	3.76	unknown	0	0	0	0	303712	0	259776	222180	0	0	0	0	0	0	0	0	0	0	0
EIC	Negative	362.1918	4.14	unknown	0	0	0	0	266208	0	236855	226576	0	0	0	0	0	0	0	0	0	0	0
EIC	Negative	225.1246	1.74	unknown	0	0	0	0	214836	97391	0	0	0	0	0	0	2271300	432684	0	0	0	0	0
EIC	Negative	307.1277	1.74	unknown	0	0	0	0	128975	0	0	0	0	0	0	0	1285992	248159	0	0	0	0	0
EIC	Negative	335.1324	3.21	unknown	0	0	0	14276	115743	52333	129634	75271	0	0	27361	0	0	0	0	0	0	0	0
EIC	Negative	364.2130	1.89	unknown	0	62343	0	0	130925	394807	118082	0	132386	0	0	0	0	0	0	0	0	0	0
EIC	Negative	373.2373	2.85	unknown	0	0	0	0	118593	25561	221524	0	119528	0	0	0	0	0	0	0	0	0	0
EIC	Negative	364.2130	1.96	unknown	0	448882	0	0	1106372	1194057	527088	0	415554	0	0	0	0	0	0	0	0	0	0
EIC	Negative	313.1668	3.06	unknown	0	0	0	0	0	0	0	0	546874	0	236196	0	0	0	0	0	0	0	0
EIC	Negative	419.0655	2.78	unknown	65578	0	0	0	0	59448	0	55650	423836	171819	51567	187921	33581	0	0	0	0	0	0
EIC	Negative	451.3434	3.34	unknown	0	0	0	0	251698	223758	234590	0	283336	0	0	0	0	0	0	0	0	0	0
EIC	Negative	483.2969	1.92	unknown	0	100023	0	0	285899	226568	121837	0	427422	0	0	0	91168	0	0	0	0	0	0
EIC	Negative	394.2683	1.96	unknown	0	49117	0	0	120196	160741	212099	0	102857	0	0	0	0	0	0	0	0	0	0
EIC	Negative	489.2837	3.85	unknown	0	357096	0	0	886128	0	1028503	0	0	0	0	0	0	0	0	0	0	0	0
EIC	Negative	416.2200	1.55	unknown	0	156638	0	0	254674	0	214299	0	469095	0	0	0	0	0	0	0	0	0	0
EIC	Negative	395.2718	2.25	unknown	244738	38454	162223	0	341745	20448	267294	1327749	239998	0	0	0	0	0	0	0	0	0	0
EIC	Negative	375.2552	2.03	unknown	0	117108	0	0	326463	186604	429293	0	187101	0	0	17805	0	0	0	0	0	0	0
EIC	Negative	394.3042	3.85	unknown	0	0	0	0	0	194078	278673	0	0	0	0	0	0	0	0	0	0	0	0
EIC	Negative	449.2911	2.33	unknown	222239	248210	338121	0	743672	110655	1159013	244233	211877	0	0	0	0	0	0	0	0	0	0
EIC	Negative	539.3232	1.91	unknown	0	139344	0	0	170908	115574	325835	0	84744	0	0	0	0	0	0	0	0	0	0
EIC	Negative	557.3365	1.96	unknown	0	188877	0	0	570418	466943	145189	0	170737	0	0	0	0	0	0	0	0	0	0
EIC	Negative	300.2626	4.40	unknown	0	1507548	0	0	1618176	0	0	0	1535505	0	0	0	0	0	0	0	0	0	0
EIC	Negative	300.2626	4.34	unknown	0	1148326	0	0	1070274	0	0	0	1028224	0	0	0	0	0	0	0	0	0	0
EIC	Negative	582.2831	5.01	unknown	0	0	747386	0	0	0	738142	0	610997	0	0	0	0	0	0	0	0	0	0
fpHILIC	Negative	291.9594	0.97	unknown	780896	330042	203438	79283	808884	1368545	376660	248809	261440	0	0	0	0	0	0	0	0	0	0
EIC	Negative	437.1816	1.32	unknown	66446	38377	0	0	75656	94297	61246	0	92799	0	0	0	0	0	0	0	0	0	0

fpHILIC	Negative	188.0715	1.07	unknown	162091	57401	519792	193238	37280	1130982	0	196300	240263	0	0	0	0	0	0	0	0	0	0
EIC	Negative	319.2219	5.66	unknown	0	715912	0	0	1074175	876804	1104752	0	0	0	0	0	0	456587	0	0	0	0	0
fpHILIC	Negative	425.0124	0.97	unknown	2016472	1321106	897732	295273	2664513	4039983	1319720	722922	774286	0	0	0	0	0	0	0	0	0	0
EIC	Negative	409.2870	6.18	unknown	519924	0	0	0	518711	316479	0	0	0	0	0	0	0	0	0	0	0	0	0
fpHILIC	Positive	248.0697	0.98	unknown	411282	379521	171705	147506	678537	764192	348114	183432	318631	0	0	0	0	0	0	0	0	0	0
fpHILIC	Negative	735.3657	0.97	unknown	445742	616409	224303	240881	1024892	1090364	667043	270759	303699	0	0	0	0	0	0	0	0	0	0
fpHILIC	Negative	454.0927	0.99	unknown	1569527	746739	773414	420898	1542722	2329246	1016676	514677	723184	0	0	0	0	0	0	0	0	0	0
fpHILIC	Negative	470.0693	0.97	unknown	966137	348419	268064	0	886013	1161320	327072	221329	220176	0	0	0	0	0	0	0	0	0	0
EIC	Negative	451.2700	2.04	unknown	654818	483780	611292	0	343967	1607071	1065256	156731	205922	0	0	0	0	0	0	0	0	0	0
fpHILIC	Negative	446.9943	0.98	unknown	1089477	572647	473164	148088	1120179	1480609	589403	339443	416457	0	0	0	0	0	0	0	0	0	0
EIC	Negative	490.2561	1.82	unknown	309243	89482	114231	0	229197	357282	250589	0	53235	0	0	0	0	0	0	0	0	0	0
fpHILIC	Negative	755.3365	0.97	unknown	435933	461831	246782	228911	573476	820938	550797	304348	278174	0	0	0	0	0	0	0	0	0	0
fpHILIC	Negative	215.0017	0.97	unknown	1548013	1158280	1167793	526990	1780565	2696276	1336677	970516	878990	0	0	0	0	0	0	0	0	0	0
EIC	Negative	527.2984	4.54	unknown	0	0	0	1198323	0	0	1571533	0	0	0	1142976	1203928	0	0	0	0	0	0	0
fpHILIC	Negative	707.3353	0.97	unknown	1179326	1636013	776130	494673	1740082	2272194	1352665	686985	1178212	0	0	0	0	0	0	0	0	0	0
EIC	Negative	283.1110	0.57	unknown	0	74493	0	0	0	0	177051	182236	0	0	0	0	0	0	0	0	0	0	0
EIC	Negative	319.2219	5.12	unknown	537711	424609	0	0	337547	532930	332618	0	0	386601	0	0	0	0	0	0	0	0	0
EIC	Negative	297.2080	5.49	unknown	371103	0	0	412955	238272	272444	230216	565814	0	0	0	192736	0	0	0	0	0	0	0
fpHILIC	Negative	708.3371	0.97	unknown	495680	682422	318798	224763	782639	758935	488805	241479	511940	0	0	0	0	0	0	0	0	0	0
fpHILIC	Negative	733.3467	0.97	unknown	477978	533924	341484	73445	537300	817502	540467	248699	236181	0	0	0	0	0	0	0	0	0	0
fpHILIC	Negative	731.3349	0.97	unknown	1044112	1398537	891925	429560	1334808	2266004	1115106	689743	722997	0	0	0	0	0	0	0	0	0	0
EIC	Negative	526.2946	4.38	unknown	2439510	0	0	3754009	0	7142120	0	0	0	0	0	0	175600	0	0	0	0	0	0
EIC	Negative	478.3179	1.75	unknown	284743	0	325692	72577	33815	9127	7233	712063	31377	43297	0	0	0	0	0	0	0	0	0
fpHILIC	Negative	732.3379	0.97	unknown	393054	612489	419945	207185	617272	782699	585904	322709	272654	0	0	0	0	0	0	0	0	0	0
EIC	Negative	556.3328	1.96	unknown	0	578950	0	0	1748190	1397556	448317	0	433187	0	0	0	0	0	0	0	0	0	12529
fpHILIC	Negative	214.0079	0.97	unknown	3753700	2718456	2317408	1061175	4435770	5502451	3153459	2076824	1924300	41251	0	0	32823	0	0	0	0	77175	0
EIC	Negative	429.1956	1.40	unknown	344630	1398037	0	0	1576446	97541	1508939	0	588259	0	813973	598002	761273	466376	1371596	1203647	421931	481130	274426
EIC	Negative	273.1861	5.44	unknown	775520	787235	485430	1517303	979339	878398	902979	861309	1768992	786011	551521	734347	1145593	734768	887772	1315611	1181341	829436	1150508
fpHILIC	Negative	885.5502	0.38	unknown	8747524	9005883	8467745	9994691	10120240	8404496	8510388	10616023	13999922	10714879	9011098	9829254	10296980	9155928	10312525	11386012	9852259	9645703	10204021
EIC	Negative	307.0648	1.55	unknown	11784644	13234363	8171138	4925682	15053554	16947991	15453279	5751986	4072280	6666522	14661040	12061522	9918794	10200942	15653564	8776335	12123583	5735013	14725586
EIC	Negative	445.2220	1.96	unknown	0	171151	0	0	348445	351348	136036	0	58483	0	202745	112164	335234	150460	294915	303976	197162	0	94482
EIC	Negative	566.5243	6.63	unknown	327889	581285	1000741	900843	873374	810842	1093379	1162797	1280063	771490	887445	1127027	1066724	1058171	835532	1221488	1266693	1265576	610665
EIC	Negative	421.2253	0.80	unknown	151965	360289	176188	171236	455088	304423	532402	517791	462331	169409	465884	107653	382284	359331	371662	631193	364698	486083	409500
EIC	Negative	328.2820	4.71	unknown	2058875	1902085	1926354	2109650	2108141	1920370	1869183	1943055	2025775	2091416	1883314	1869342	1932271	1897678	1980729	2107319	1988627	1880454	1874117
EIC	Negative	441.2646	3.28	unknown	168136	24714	184224	148330	86460	150645	88871	390568	116938	140713	77878	81521	73388	143865	133020	80508	0	503190	113811
EIC	Negative	422.2290	0.80	unknown	39021	95348	52281	52793	126820	77486	135311	132172	120032	48703	123744	32900	91505	96455	98949	162276	101656	133156	93707
EIC	Negative	369.1557	1.87	unknown	231758	243823	0	0	300159	346711	346577	0	233636	0	275984	296615	321823	155693	331790	450996	275212	0	165091
EIC	Negative	299.1504	1.82	unknown	95795	76532	47017	0	81280	91564	97502	54504	73003	37580	87110	75055	85190	66906	94686	75066	135552	0	91345
EIC	Negative	606.4074	6.66	unknown	224461	320457	300418	344142	790261	709322	955937	948695	1094309	338886	550620	952331	961237	1475008	851142	1268910	669612	508888	1188978
EIC	Negative	236.1738	3.04	unknown	42933	70810	70773	70562	65445	68616	65619	68059	59474	60907	61662	54385	57862	61006	63443	77349	65570	60064	60015
fpHILIC	Positive	175.0860	2.62	unknown	945565	1040908	1116714	962544	1181396	1230564	1324312	1037914	1036538	1063974	1214533	1235889	1144423	1107256	1215178	1171529	1179033	1181393	1051065
EIC	Negative	260.1650	4.60	unknown	67817	140682	38224	43969	209603	91785	154837	60924	203197	48164	121713	56270	135306	58418	115198	130838	100510	59228	313085
EIC	Negative	555.3503	5.47	unknown	1815163	2741271	1814914	2159273	2360217	2460680	2263126	1919137	3098889	2035767	2764151	2652680	2677011	2775607	2575238	2590714	2358196	1767218	2786531
EIC	Negative	313.3023	6.72	unknown	248648	302407	225670	351022	385667	357281	372291	285073	242872	333410	334168	456353	454307	384360	358632	224681	335211	418131	303272
EIC	Negative	522.2628	3.88	unknown	142414	618222	173319	255112	457326	590155	382443	157290	519305	259813	490389	370130	413874	616700	494733	444543	552764	239986	372520
fpHILIC	Positive	154.0497	0.96	unknown	997091	1158037	550094	703947	1901977	1991289	1484104	709059	1576272	1078488	1586639	1165807	1518936	912694	1645392	1544670	1864614	650573	1950632
EIC	Negative	338.2364	2.47	unknown	458796	524990	369925	429165	561874	541728	564702	540758	693326	547221	566374	552473	580761	597942	577745	548830	554952	628174	591996
EIC	Negative	605.4044	6.66	unknown	570739	821886	774504	895241	2074666	1846468	2461961	2532818	2545087	867180	1373368	2362657	2462346	3559842	2236331	3089405	1826385	1344176	2962833
EIC	Negative	552.1963	1.03	unknown	64195	23712	62018	32159	58105	90614	137905	146706	82363	136236	113414	133853	117301	121045	80004	88213	97688	104920	144850
EIC	Negative	422.2288	0.86	unknown	89271	293208	105180	104248	376967	150056	430374	307038	460341	109938	348445	50106	184599	316706	229198	481932	298089	299207	416668
EIC	Negative	254.1039	1.28	unknown	53483	54450	0	0	48736	44400	51586	0	57559	54264	54335	53181	55443	40003	53914	39239	58516	0	50849
fpHILIC	Positive	569.3421	0.55	unknown	15538443	25495506	14991079	20409050	20569132	22700966	20367986	17159904	24013824	17795850	22900948	21510890	23429990	18173634	17199008	22371612	23968116	16567287	24841926
EIC	Negative	316.2572	3.31	unknown	6147072	6532642	6199246	6078511	6423408	6479353	6351693	6016901	5972154	6006831	6315609	6118809	6329818	6170769	6202136	6257703	6615277	5954845	6255651
EIC	Negative	644.3313	5.74	unknown	171238	198517	257195	229736	235978	315953	283390	269542	173301	238691	276305	263521	266160	242196	254406	205645	257800	252092	310218
fpHILIC	Negative	129.0193	0.76	unknown	1340957	13511811	1764374	1404657	7790143	11472012	1511310	1351415	1080932	1269509	1101135	5967933	1650920	1364330	5107836	5644515	1467856	5156101	5866448
EIC	Negative	367.0393	1.18	unknown	124395	140164	148636	139616	145177	141630	146339	144579	159317	134809	140394	149475	174817	147054	158393	143703	167596	117144	151389
fpHILIC	Positive	627.5348	0.37	unknown	397870	359642	394598	389868	441584	320988	345105	436603	559954	500937	426401	362689	385665	372163	419529	398879	433100	391384	464735
fpHILIC	Positive	133.0604	2.86	unknown	16610735	7864526	9494477	0	11441256	21943786	10965369	8426356	12974772	12467145	10577238	12609197	10046653	12716989	24985766	11531316	10404560	11254737	7682482
EIC	Negative	528.3085	5.42	unknown	1244922	1297690	1265679	1079143	1503559	2494200	1637799	1323893	970902	1079147	1297822	1402418	1152065	1730077	1809544	1239516	1599245	1596370	1701448
fpHILIC	Positive	748.5269	0.38	unknown	254828	408961	298637	244009	436082	383942	280748	450473	543045	364983	375150	375477	313822	615910	415401	486729	440056	309879	446466
fpHILIC	Negative	171.0064	3.87	unknown	3536211	3063360	1903204	2170678	2627679	4315397	2705118	1585747	1994801	1520217	1933210	2191966	1983937	1659378	2498562	3245398	2610504	2536825	2369026
EIC	Negative	307.2547	6.50	unknown	588229	1054983	978918	1293428	948833	949918	1216496	1107735	1202232	865928	1330696	1060325	1224114	1058120	980440	972240	1320224	1111685	1054145
EIC	Negative	389.1035	3.30	unknown	112235	131664	133536	141996	137674	132445	135597	130185	132792	129418	128341	135661	142300	148248	132166	129572	144936	135698	128180
EIC	Negative	588.2946	1.86	unknown	77171	151595	0	0	124822	103309	144451	0	146153	0	118330	106047	118553	78880	126069	122423	109523	0	162197
fpHILIC	Positive	713.0630	2.94	unknown	4748955	5709871	5474812	5638782	5229017	5582021	5805226	5609525	5684749	5596970	5859373	6132220	6060887	5043611	5123588	5647916	5638162	5464127	6120661
EIC	Negative	629.3559	4.26	unknown	972442	1970683	1066630	1518164	1763824	1928898	1694021	1274736	1845585	1213020	1901332	1788052	1729530	1622202	1761672	1995451	1681535	1178637	1918237
EIC	Negative	297.2436	5.78	unknown	3024275	2565005	2991780	2812978	2992654	3055452	2904949	2954540	3406917	2654424	2525915	3114758	2750171	3124420	2344943	3884314	2697800	3446452	2993524
EIC	Negative	604.3532	4.33	unknown	6909162	8194166	7004224	7502595	8477112	9180105	9337805	7871769	7253310	6869736	8800235	8511450	8273603	7284261	9033736	8801363	9451573	6866815	8129374
EIC	Negative	387.2381	5.37	unknown	0	0	0	200041	149644	191359	0	185469	0	205787	0	0	0	0	176033	0	160876	0	192295
EIC	Negative	505.0385	1.19	unknown	46474	64849	53728	56219	66536	69846	62302	51788	61214	59201	86004	56795	66338	62659	60450	58308	68807	53291	72478
fpHILIC	Positive	105.0740	3.03	unknown	321910	462747	315897	575522	490299	372153	350849	335920	525909	308463	450137	365621	346557	301953	346409	440700	502333	486102	311188
EIC	Negative	636.3501	4.87	unknown	2212521	2170619	2133275	2064617	2224834	1997740	2166047	2221597	2514494	2371817	2060272	2176818	2281199	2115460	2217797	2422937	2175501	2328158	2043430
EIC	Negative	389.0681	1.56	unknown	205306	406123	92431	59752	500435	486774	482842	62476	111208	106213	456832	405782	260539	297682	460131	306425	382513	0	504086
fpHILIC	Positive	113.0903	4.47	unknown	109711	297193	117366	299769	176128	380212	109569	278248	515652	132811	188442	251235	346110	241917	273947	352443	258275	309376	274496
EIC	Negative	265.1811	2.50	unknown	58545	64738	48557	29378	82016	66296	70522	86234	63736	75515	69184	56336	69261	65944	67032	61630	69775	98937	74023
fpHILIC	Positive	134.0447	3.77	unknown	1576142	998574	720400	827102	854477	1982655	953009	773598	865598	670938	614990	1248645	823898	868759	760832	1184359	1111970	1208531	1202008
EIC	Negative	332.2680	6.57	unknown	311190	340007	297387	623096	412193	421058	474651	581897	712769	560526	504478	517849	645202	382048	351098	460599	551206	840191	418925
fpHILIC	Positive	329.1723	2.70	unknown	1837629	2217156	2370516	2025814	2180045	2527369	2314580	1941812	2016574	2172918	2494372	2391185	2451632	2430548	2236799	2252102	2055405	2119174	2399017
EIC	Negative	382.2326	0.78	unknown	52222	42888	63769	133048	74454	184687	96335	269235	39699	57247	76728	66407	30587	20311	154642	93156	64294	269635	87938

EIC	Negative	403.2491	3.78	unknown	204517	242218	240241	234920	226074	240747	248200	245503	240905	210241	183784	217686	230532	227021	244251	240247	216866	253950	255669
EIC	Negative	355.2432	2.35	unknown	469998	503350	421434	130457	446269	478152	497944	466272	464457	451041	465683	489186	478385	461440	489189	491959	485982	411927	492298
EIC	Negative	398.9367	3.09	unknown	118035	193145	112766	107032	154043	156857	158100	95646	171065	107977	186166	150103	175816	146878	179208	174887	143816	110267	133502
EIC	Negative	369.1915	2.71	unknown	535412	398617	248013	326213	354394	401109	378544	579824	387547	494152	344999	369168	384822	395449	430354	303860	447211	447956	491593
EIC	Negative	306.2521	6.50	unknown	4880112	8442364	7383492	10686355	8155410	7427013	9970169	9277789	9738399	7279401	10916329	8913060	9855206	8203963	8224504	8001896	11224788	9039869	8494449
EIC	Negative	356.2472	2.48	unknown	2919432	3657704	2201597	2772219	3763772	3534958	3619616	3728545	3624573	3624689	3685622	3645485	3679958	3839868	3778243	3538998	3701712	3618272	3809354
fpHILIC	Positive	302.0705	3.39	unknown	319848	490514	545422	552980	513990	545264	525426	530263	467509	675879	496039	474411	512714	466556	541630	528161	579932	439348	518579
fpHILIC	Negative	178.0752	0.94	unknown	1909941	1960316	1750585	1651470	1976757	1710699	1843465	1946185	1973417	1818610	1939493	1764616	1664812	1956027	2027543	2050752	1841743	1685003	1937854
fpHILIC	Positive	177.0659	0.96	unknown	593175	543624	306060	395975	1047908	1243529	728312	381797	957125	624584	1048830	706943	893577	538549	1012042	920485	1230778	306836	931927
EIC	Negative	540.2730	2.71	unknown	551618	526597	303541	348400	422408	723976	365543	378241	954488	452292	392107	563860	387430	332013	495227	689278	780498	231641	676759
EIC	Negative	358.2625	2.35	unknown	3294868	3593779	3121402	3282894	3410718	3428078	3738474	3439263	3474158	3181630	3614800	3541473	3519684	3580548	3564247	3483023	3670496	3227242	3730292
EIC	Negative	535.2869	5.23	unknown	483301	468141	436578	510771	518173	402466	457955	577674	567439	528164	424002	507842	440186	522057	487727	511322	501489	555394	494547
fpHILIC	Positive	133.0799	3.37	unknown	40373180	36694776	24584342	23011768	29421860	37514816	26654468	31839122	32885296	21552510	24215452	29311344	33118436	35060372	35510652	38773112	27787442	36303444	21634336
EIC	Negative	283.0614	2.17	unknown	303226	325358	271978	316079	296432	287532	308717	359096	303867	351789	251922	324577	295171	339381	271110	335511	270790	338472	356416
EIC	Negative	320.2856	4.00	unknown	617045	631603	595160	564092	633441	605727	600369	590498	590511	578579	599871	579822	586605	588165	597251	610539	658353	600728	587950
EIC	Negative	441.1884	3.65	unknown	612700	672834	510961	615671	641629	602922	576829	665511	669227	647599	618411	627788	601042	611953	610959	613370	577931	723874	666904
EIC	Negative	309.0628	1.55	unknown	608366	708220	444652	317446	819379	929412	796617	340834	204104	442045	810567	653342	528724	534345	855257	466955	649901	341936	813826
EIC	Negative	502.3992	6.59	unknown	1125002	1132656	1045180	1112392	1597405	1073207	2147885	1678609	2245181	1433207	1365838	1108547	1057233	1570580	1877124	2479978	1388476	1059080	2044185
EIC	Negative	365.2334	0.91	unknown	36114	59639	19770	32754	78362	84605	54850	44655	56633	26898	68440	20309	69773	80975	85437	106023	31796	55862	43957
fpHILIC	Positive	235.0798	3.14	unknown	1726674	2184128	1836542	1721070	1933748	1706723	1897362	2056193	2018237	2039225	1867807	2008466	1988947	2218270	2144213	2051796	1746407	1898184	1892203
EIC	Negative	552.2709	4.72	unknown	1298600	1449582	1081564	1214879	2490362	2488743	1834251	1868554	2836012	1429475	2216046	2100600	1664929	3013664	2347148	2804238	1885102	1596327	3230623
fpHILIC	Positive	136.0392	0.95	unknown	571695	633921	274518	302265	985454	1325769	720842	341102	983004	501440	918237	739443	823700	479875	957244	921658	1222594	343437	967479
EIC	Negative	320.2856	3.75	unknown	586924	597312	567036	558752	606633	585336	557815	586026	548620	565198	589780	560216	560983	557862	582481	583416	556550	600009	599478
EIC	Negative	647.2346	1.85	unknown	174770	97105	0	0	91100	62546	153347	0	77220	0	119343	119382	130596	73333	95181	135942	93670	0	64546
EIC	Negative	554.3473	5.47	unknown	6146279	9460952	6181041	7203029	7990235	8228526	7662010	6538423	10389907	6940574	9622117	9106951	9325528	9604708	8861363	8696197	8149799	5963677	9686904
EIC	Negative	337.2328	2.48	unknown	1837162	1849785	1557664	1847368	1967312	1803494	1985965	1804628	1837201	1927169	1894327	1934034	1915998	1998385	1929799	1837563	1924348	1793497	2043521
fpHILIC	Positive	142.9384	4.62	unknown	365826	399502	410322	434914	458970	484291	513941	471992	510451	503324	633213	470201	489057	456442	511208	473242	517473	484143	487493
fpHILIC	Positive	134.0808	3.15	unknown	802907	634638	513206	397649	717444	989233	804279	354082	382164	516185	715223	600114	616185	472352	793912	810037	552368	704191	432514
EIC	Negative	528.2733	4.82	unknown	1277112	1227834	1007509	1035417	2341952	2442946	1720422	1860475	1574281	1191761	1782451	1860189	1318272	2041256	1961475	1954623	1762484	1602713	2800887
fpHILIC	Positive	134.0808	3.36	unknown	3649530	3528567	2154352	1933583	2511526	3588422	2737924	2823033	3281621	2038559	2194832	2835810	3041785	3162081	3556322	3636540	2684881	3384119	1896436
fpHILIC	Negative	192.0338	2.68	unknown	482024	556879	428909	561328	557306	686046	549727	482265	409577	498690	525975	552215	503032	558655	515828	594973	615117	461506	536449
EIC	Negative	341.0343	1.19	unknown	300220	319595	269542	280570	301632	371484	349203	259564	293111	288008	465958	336342	292201	332192	324253	268136	324210	307153	374887
EIC	Negative	280.0222	1.19	unknown	81447	82431	83392	89431	91830	94639	90292	87572	86210	79594	123893	97103	87794	88744	91347	82038	88868	95042	100177
EIC	Negative	506.3231	5.40	unknown	1535961	1627914	1516306	1407811	1579975	1690566	1438392	1671046	1474916	1414643	1470221	1662891	1602294	1419630	1567162	1544059	1603593	1577165	1686532
EIC	Negative	421.2253	0.86	unknown	344345	1144708	399255	318992	1480257	565869	1659632	1169993	1781193	432098	1360473	169271	733560	1222807	898568	1945373	1158634	1205465	1554146
fpHILIC	Negative	221.0819	0.42	unknown	206289	631098	168215	294125	443051	372192	184580	118091	1410657	149701	216381	372758	909372	333031	335648	861320	271802	205309	895385
EIC	Negative	332.2679	6.53	unknown	5419706	5664689	5430692	9568074	6045562	9323355	9383389	8572974	9464156	8106695	11103261	9038858	10361017	9368268	7985988	8895760	10366753	9496993	6764454
EIC	Negative	427.2713	1.07	unknown	37678	67726	60971	96205	81677	108861	59638	68221	52358	82200	78436	43989	94821	60416	77205	101105	55741	108497	34494
EIC	Negative	395.2441	2.93	unknown	1990925	2288617	1490033	1544406	2042422	1956387	1852259	2491718	1983268	1952931	2312821	1767541	2118549	1656523	2279135	1644787	2039467	2412749	2133657
fpHILIC	Negative	449.0766	4.29	unknown	371695	514154	198553	300454	543713	629583	532817	249455	585970	280651	465085	558305	611664	623337	604121	618964	566538	282810	515497
EIC	Negative	534.2823	5.23	unknown	1910342	1593220	1642525	2042657	1974953	1617661	1688967	2042021	2063805	1907277	1690047	1839556	1675319	1962203	1818907	1911064	1878281	2111476	1843928
fpHILIC	Negative	558.3308	0.60	unknown	3612882	4131781	2848237	3138096	3810978	3432246	3373626	3122035	4530729	3253028	4092870	3860268	4094957	3757005	3951279	4203561	3568589	2538432	4355756
EIC	Negative	315.2513	3.44	unknown	970424	1032004	957804	921851	1042707	1030095	1007526	1016040	996309	822552	946372	1037452	1150261	1151473	1066640	914418	1128476	1020293	1062022
fpHILIC	Positive	234.0768	3.14	unknown	21679124	26734658	24904478	23128458	26447202	22876672	24560252	25980778	24925550	26086018	27080294	26129484	26106356	28372716	27950452	25139918	24360114	24046254	25758180
EIC	Negative	297.2436	5.66	unknown	2754088	1984092	2314170	2004018	2482510	2702477	2165871	2296231	2588568	1926010	1994340	2212092	2219515	2505516	1804810	3211764	2331348	2725301	2406918
EIC	Negative	237.0766	1.86	unknown	133038	143699	0	0	135643	126593	125067	0	123156	0	115459	121870	112419	126398	130133	129489	129035	0	132138
EIC	Negative	226.1193	1.81	unknown	138445	163496	413506	0	213486	168073	195726	350132	122761	334110	190440	185575	435537	222675	278265	165922	378368	0	249300
fpHILIC	Positive	258.1095	3.32	unknown	47834332	120580872	52427632	47881280	86920016	106509896	118455256	51161432	89509232	40849868	91861728	107316792	84572592	83668488	86184456	112262360	88605472	66151252	109420224
fpHILIC	Negative	171.0064	3.31	unknown	550360	1768233	591314	387024	1367672	1727871	1646373	578297	1470155	468725	1228432	1523132	1240143	1465440	1471909	1712136	1257337	707728	1794603
EIC	Negative	355.1683	3.11	unknown	141584	116440	152016	123250	153584	184301	156017	114714	168569	133638	142088	128583	130222	207652	155388	183380	129181	146640	180726
fpHILIC	Positive	161.1285	4.90	unknown	5985268	3948688	5254484	5420361	6159994	6169953	5088344	4868722	7311443	5213913	4958760	4917081	4822599	6181910	6255687	5802201	7329243	5710827	5193113
EIC	Negative	267.0878	1.83	unknown	149948	78606	24855	0	118436	137198	116300	0	97331	0	58452	118370	80823	89791	125576	89184	155338	0	108584
EIC	Negative	279.0189	1.19	unknown	707588	739764	696987	753359	732150	777983	766494	708781	720157	713579	1011272	754742	712035	731972	757412	690656	746032	799934	799489
EIC	Negative	359.2659	2.35	unknown	475147	533941	424028	508486	500854	524680	544661	472382	499717	483572	505673	518073	514343	518201	539960	530900	537241	434215	560921
fpHILIC	Positive	154.0690	0.96	unknown	1675075	1853202	929831	1104388	2604897	3303540	2108061	1127484	2500553	1637846	3228285	1769151	2208864	1670171	2613015	2334831	3389963	845065	2741021
fpHILIC	Negative	163.0612	2.44	unknown	9347799	12835882	7761461	5006197	15213601	13945312	18772084	7252172	17247942	7941809	16931078	17534540	16275668	15256801	15849533	15714631	16063383	9853009	16699533
fpHILIC	Negative	598.3729	0.62	unknown	732223	984782	812821	872541	1103619	1257976	955030	911885	786639	784080	791898	948441	1039899	1073312	1021243	831772	1124584	1091860	1071780
EIC	Negative	473.3854	6.70	unknown	500573	442577	479695	873471	725851	624691	642312	673194	1542010	1043436	969590	885934	670517	1110819	1037255	899296	558788	796006	1022655
EIC	Negative	553.2746	4.72	unknown	387529	511365	351467	407649	753574	820937	569893	576039	893167	433858	642297	593570	513833	975320	767968	843636	575263	487877	1031446
EIC	Negative	342.0381	1.19	unknown	55138	63989	51111	55208	59874	63191	68037	32871	57316	52446	78231	62241	49575	54979	60395	52871	58328	50791	66176
EIC	Negative	253.1446	2.57	unknown	868285	1105626	748848	896834	952532	507093	848231	516991	1555025	734924	668993	792201	1324604	755407	786682	1634328	896018	795930	382516
EIC	Negative	620.2925	5.34	unknown	7612781	13312340	10167699	13670526	15957345	20706915	19255873	13618301	17344063	10144978	19153536	20251414	21259070	20136033	16767842	18829651	18176476	13800377	21695751
EIC	Negative	450.2586	2.72	unknown	212525	244484	69632	264214	202766	232042	210747	182052	284881	299741	295733	188597	283401	267729	285603	256778	148309	225302	227842
EIC	Negative	396.2470	2.93	unknown	470330	542804	351412	400152	523804	495388	442259	598168	452227	445535	554817	424361	536129	388566	521600	408786	540457	626790	485973
EIC	Negative	540.2735	2.93	unknown	295888	337128	0	197329	265715	376784	237215	119359	573637	89787	153467	247390	208274	265484	336279	338772	297234	162913	482867
fpHILIC	Negative	211.0012	2.96	unknown	580355	389380	411255	401188	379527	604544	391854	415982	452353	448117	506297	540874	355936	418037	482018	486881	461740	463910	414440
EIC	Negative	373.1660	3.65	unknown	953058	1081878	827613	801807	1046920	957983	917052	985483	854496	865456	924903	870972	983984	945234	936742	960421	977691	1044068	980825
EIC	Negative	263.0021	1.93	unknown	1230281	6099868	1341992	885165	5864103	4650972	6161095	753506	6162485	1063417	7098382	7331819	8916683	8287693	2609055	6517411	8020069	544961	6588114
EIC	Negative	304.2358	6.57	unknown	445873	708445	362806	677854	819772	712260	892076	643345	1174081	521343	951752	911430	1004025	795842	729162	891129	1118057	844241	782668
EIC	Negative	271.2281	5.48	unknown	735089	790833	875151	917621	819623	847476	814577	749679	815337	674693	662208	956759	782569	743379	742742	1088906	707988	959192	669796
EIC	Negative	338.3148	6.71	unknown	743307	1156463	949819	1487887	1801862	1590983	1311664	1188202	1228994	1105557	1697902	2440317	1759355	1335946	1731747	1409862	1367564	1673168	1158695
EIC	Negative	262.1927	2.12	unknown	195973	239657	132116	148780	227314	195408	220306	252902	198469	226503	221387	206208	202357	180113	240900	202110	217630	220617	247141
EIC	Negative	493.3541	3.26	unknown	184623	151780	134091	181534	197612	169244	147641	178042	183008	178386	177012	147242	157620	166566	261218	205289	128673	149841	158310
EIC	Negative	604.2966	4.26	unknown	653704	1042592	797540	896990	889463	955481	870213	779551	991458	840640	1024409	895194	897026	880681	912364	1009621	938133	751654	1018088
EIC	Negative	390.0708	1.56	unknown	0	81170	0	0	92503	95337	93438	0	29364	0	90194	79634	50816	61492	89679	65730	70313	0	94919
EIC	Negative	619.2895	5.34	unknown	24060132	41529757	30638846	41294892	49321109	64175110	58268468	41673110	53135674	30992719	59333736	61426993	65448160	60943504	51137362	57537622	56509639	43378491	66765810
fpHILIC	Positive	162.1314	4.91	unknown	501645	318300	393469	443194	450552	464091	379482	391925	549983	405335	371605	385014	364889	494847	476726	414230	545033	461517	414041
EIC	Negative	303.1276	2.62	unknown	2597936	2370276	1403019	2718875	3470172	2754187	4362536	2672301	3610157	3125601	4034880	3279854	3534351	3815169	4996553	5160235	2827046	1607817	2847539
fpHILIC	Positive	137.0708	4.68	unknown	2259768	2679276	473101	1652555	3943323	3888171	3360051	1287207	3923171	1914426	3475820	3776663	3093894	2766442	5030103	3620078	3691323	1029875	3585034

fpHILIC	Positive	184.0737	3.68	unknown	7261435	4802447	3296431	5441937	5718998	8983337	4868197	4000154	5428157	3487946	3624435	6731425	4060351	3970699	5173772	7038304	5565527	6335233	5870453
fpHILIC	Positive	224.1119	2.53	unknown	473528	334682	259696	373274	346938	463257	311868	263999	501387	278791	367468	423659	421540	304593	397976	480854	261641	365910	445715
fpHILIC	Positive	148.0964	2.65	unknown	1708708	1976781	1931575	2219460	2416507	2519712	1916066	1288946	2377836	2383952	2286855	2506709	2268743	2066422	2547426	2491573	1799640	2013595	2028424
EIC	Negative	621.2952	5.33	unknown	399372	3129505	2309198	3015751	3794866	4836397	4342038	3198467	4001666	2356982	4447630	4651535	5001878	4710357	3885822	4392439	4313940	3195079	5089961
fpHILIC	Negative	130.0228	0.76	unknown	79496	730195	119277	51859	426682	576652	111086	83742	33574	74513	67899	319057	90725	72689	292100	304345	64308	282871	332072
EIC	Negative	361.1953	6.22	unknown	28205	298088	0	328711	172256	178088	190661	59576	303759	145185	205776	183238	237341	137005	160677	200337	219091	177590	179733
EIC	Negative	346.1591	1.92	unknown	0	211035	0	0	356963	358780	391950	0	293838	362080	262169	360254	264801	271235	448551	396171	375243	0	233893
EIC	Negative	319.1539	4.91	unknown	351437	0	0	0	0	400239	0	0	0	0	0	0	0	0	0	415217	0	0	0
EIC	Negative	382.2967	5.78	unknown	1693562	1713796	1708568	2240685	1903362	2079502	2024991	2200074	1775253	1935792	2046580	1889085	1989127	1589473	1985251	1892088	2242490	2380633	1857609
fpHILIC	Negative	165.0403	3.19	unknown	5391668	4015530	3272984	5300110	4546126	6450810	6284696	3826049	5588175	3890095	5472910	5742929	4925308	6635170	4771767	6594855	6032729	4729394	5585442
EIC	Negative	492.1835	2.72	unknown	823054	1022228	1004201	879785	926659	1017905	1163145	893056	913626	888901	1059286	992477	1146669	1107506	1118371	1078949	1080700	788148	1036144
EIC	Negative	427.3207	6.62	unknown	821602	400102	681415	893328	507849	584907	1236597	989144	508340	593270	706026	860362	531387	618652	640304	1043671	650310	649729	989994
fpHILIC	Positive	389.1795	3.34	unknown	355133	1227486	297745	229735	723020	968365	798729	260743	779812	121520	487862	675849	641857	962053	721201	1046850	643025	430169	825932
EIC	Negative	480.3082	5.30	unknown	783562	1230851	1472147	1117814	905571	1879551	1189757	1209357	1080916	1045496	918347	1232526	870760	2148259	1633966	1212602	1316853	657949	1688567
EIC	Negative	298.2469	5.66	unknown	553569	410742	454713	429566	501885	537216	393575	485512	518499	386663	399997	445380	478977	502571	377113	629184	464170	576587	485567
EIC	Negative	268.1556	2.28	unknown	478081	369343	355648	572447	423290	432696	520118	354239	534173	615651	432424	496281	469065	304898	551913	594741	387485	542108	277727
EIC	Negative	250.1448	2.30	unknown	1650521	1405725	728812	1671599	1650215	2470614	1666445	819544	980727	1717763	1612404	1941089	1516230	916192	1862790	1990176	1587987	1377878	1070197
EIC	Negative	245.1757	3.08	unknown	68894	74396	122910	79298	108690	66306	99626	117291	109500	97976	92089	122471	91732	98047	118065	94835	127680	105356	75458
EIC	Negative	344.2315	4.74	unknown	141073	155163	80513	253253	223410	136556	206179	111791	398072	133518	132682	143638	190500	46304	197630	296552	254399	160534	209905
EIC	Negative	443.1565	2.05	unknown	1509082	4916774	1435811	1153794	3875641	4269225	4343424	921446	3366378	1429006	4127528	3462396	4028022	4687860	5300197	4035094	4237946	958206	2915336
fpHILIC	Positive	104.0709	3.03	unknown	7143600	10056730	7203646	14000275	10718520	8881030	7340426	7065207	12295511	6794799	10616970	7224964	6840526	7609128	8443105	10564196	10650668	11208530	7286316
EIC	Negative	334.1637	2.64	unknown	248167	212735	168135	252114	260022	377547	225207	139870	157319	273391	277048	283585	215157	114237	342402	290657	298991	131586	142897
EIC	Negative	306.2521	6.57	unknown	280998	413874	292924	471358	457882	462138	549269	479099	602291	426985	598033	517238	702618	380502	477120	420694	626520	599868	528918
EIC	Negative	437.2459	2.15	unknown	248541	923473	235005	466723	846231	346456	808962	415796	783735	648363	305433	932357	282158	649204	402965	865603	448042	582657	1030749
EIC	Negative	239.1288	2.81	unknown	2953803	3802920	2246064	2274588	3595228	4068086	4016281	2491985	3279630	3482752	5004553	3506552	3406302	3750339	3763467	3902095	3667514	3000113	3824536
EIC	Negative	410.2640	1.88	unknown	193880	181886	0	0	178346	356874	134318	0	120265	0	270161	228137	290517	182605	177129	235973	165907	0	243833
EIC	Negative	289.1236	3.79	unknown	280961	325430	267580	299758	286761	299581	299084	296308	288611	275034	285922	293989	308461	303965	305220	312753	304321	305527	304787
EIC	Negative	409.2607	1.29	unknown	147603	85881	23996	0	111415	418402	84228	0	84729	219347	154415	161714	101186	137403	200133	86251	146148	0	295882
fpHILIC	Negative	135.0298	2.95	unknown	140463200	82146112	48396156	67554736	117796296	113023080	116406760	87830176	143457056	49557732	65862744	103952752	89533784	130326384	149534128	100333192	126608944	99949616	126732792
EIC	Negative	528.3073	4.33	unknown	689759	796208	582289	567127	646262	811974	832156	657975	668241	625329	639315	740250	767366	586247	695135	746067	1084745	679262	566274
EIC	Negative	619.3466	3.37	unknown	574167	486788	210998	190515	341684	818949	445466	531483	470335	117437	421753	752152	488602	101961	548876	670325	687759	91829	721978
EIC	Negative	444.1593	2.05	unknown	347508	1119666	331318	216541	870854	966707	977578	120242	785793	276061	928129	754268	981822	1038698	1272578	903655	922812	147373	638688
fpHILIC	Negative	303.0586	2.99	unknown	1050187	541418	271302	463291	781122	547002	736739	545590	1023496	322124	339756	536607	493702	507948	663571	699437	991487	643565	796425
EIC	Negative	374.2572	1.43	unknown	0	764781	0	0	750288	760822	758395	0	669424	0	745869	734668	759145	786135	786265	737543	798017	0	748030
EIC	Negative	265.1481	4.60	unknown	12337519	8145373	6458934	12988198	22164619	8161774	10681223	5385874	9968019	7589326	4645069	7922360	6832678	5739859	5144700	15376665	19898174	8875343	9596780
fpHILIC	Negative	158.0823	0.61	unknown	3458972	3508020	2748970	3434231	3390595	6979323	4188167	1627036	4077304	2124621	2887031	3389327	3751124	2824404	7921915	4941993	3598590	1950964	2783579
EIC	Negative	449.3279	2.93	unknown	1358900	2366466	1159259	940018	2563606	1849400	2481693	1632462	2084851	992465	1916438	1781697	1787328	1878676	2780658	2391970	2324750	1706649	1899580
EIC	Negative	637.3537	4.87	unknown	728588	688128	645418	653001	672705	597602	681885	683280	733980	739351	683032	697258	713422	666892	714615	759813	709314	687696	656103
EIC	Negative	582.3779	6.26	unknown	95421077	92907853	98832937	98993128	100315507	97942784	97916488	98987186	101195688	101713056	105426004	111116738	100772512	99095223	99683887	108682237	102229619	106722595	102556630
fpHILIC	Negative	75.0088	0.99	unknown	5215589	3609970	2293433	3527811	6286309	5067443	6727734	4534932	8632596	2406899	3412118	5774811	5114117	6602071	7496547	5554930	7606300	4973549	7106667
EIC	Negative	279.1252	1.97	unknown	0	39945	0	0	57106	49019	64015	0	53693	0	33968	47554	65579	44232	40660	90750	60597	0	42564
EIC	Negative	409.2608	1.88	unknown	532750	732306	0	0	727198	1295075	577272	0	471748	0	1029073	899459	1146438	754386	678789	914280	678010	0	949195
EIC	Negative	588.4014	2.85	unknown	0	225726	199050	688996	484277	334991	302923	222970	519864	196573	177774	348193	478545	177207	151153	559528	495841	566390	182626
EIC	Negative	347.1274	0.89	unknown	71947	59850	66103	75530	65622	81117	73951	57955	71057	86705	55344	60241	65601	80123	65600	67984	69894	78643	84643
EIC	Negative	582.2826	4.47	unknown	556167	491016	489197	524465	532572	403886	477941	507910	672346	564439	467676	485073	481084	573625	546096	604787	558939	584836	430113
EIC	Negative	327.1812	2.28	unknown	313770	201499	360303	205459	233185	266248	223413	405105	129995	377170	159524	291497	148883	324797	203210	189343	186221	558829	183469
EIC	Negative	268.2365	6.50	unknown	1093488	1251044	1862749	2195886	1854436	1396507	2020360	2453819	2476953	1535064	1752137	2005491	2316187	1955542	1651744	2405131	2402580	2506031	1748864
fpHILIC	Positive	243.0518	2.63	unknown	745603	2124634	1891247	1225443	1515682	1684938	1862163	1331561	1345830	1358901	1821944	1636347	1484931	1726519	2017725	1484851	1479901	1501060	1647410
fpHILIC	Negative	205.0352	0.96	unknown	847652	540541	436609	529887	943418	728506	1147668	788154	1269771	419162	468541	715015	835337	909135	942984	880907	1246366	914084	1140898
EIC	Negative	455.2571	1.43	unknown	0	199507	0	0	210074	226855	222542	0	202229	0	192497	215014	215211	227200	230215	223916	238355	0	213204
fpHILIC	Negative	318.0960	3.31	unknown	912649	3176361	1054768	765299	2222094	3079107	3023857	1027837	2382593	972176	2168234	2849001	2088234	2550597	2490656	3089337	2280858	1230339	3250369
EIC	Negative	439.2969	5.75	unknown	152775	122107	75954	175946	214349	122432	23189	164290	65964	53993	195974	96685	50264	89162	48915	105672	190755	48817	226567
EIC	Negative	551.3950	3.43	unknown	15636	15749	26757	185333	65476	49749	70660	92292	149278	51461	35998	77246	48598	0	0	273549	70847	91945	13507
EIC	Negative	312.2992	6.72	unknown	1459469	2390680	1867118	2822757	3192214	2108012	2991281	2331140	2035197	2840056	2763381	3786414	2401350	2999669	2912971	1676749	2907767	3358490	2474066
EIC	Negative	315.2512	3.32	unknown	2286748	2318182	2148210	2585717	2291011	2298156	2338586	2329625	2391480	2250952	2189345	2598590	2754669	2482056	2192235	2288213	2622926	2684683	2496282
fpHILIC	Positive	613.1577	4.51	unknown	423241	202200	119617	251260	366624	597786	219960	216786	369576	207188	165761	359552	235048	341034	314388	382929	475169	302755	392623
EIC	Negative	577.3347	3.83	unknown	2106974	4583901	2832248	3922517	3451914	4035706	4527951	3102544	3579077	2703984	4948396	5104750	3929055	4497800	4500566	4293965	4011571	2693815	4230531
EIC	Negative	583.3810	6.26	unknown	30148814	29409961	31961469	31721231	31593705	31083479	31475774	31502087	31666058	32601714	33973810	35387120	32213944	31247244	31786367	34686513	32477538	34243790	32884201
fpHILIC	Positive	263.1463	3.37	unknown	18425074	14150215	4876684	3325070	8135856	11712719	7366908	9294295	12386396	3233943	4065738	8078447	7729719	13255338	12302201	18605484	6719924	10254757	3756938
fpHILIC	Positive	226.0818	2.08	unknown	2185819	1451530	2036056	2388572	1824818	2889985	1975045	2173898	1975537	2435500	2504896	2496509	1973749	2081197	1964934	2527569	1857271	3217130	1884390
fpHILIC	Positive	260.1143	3.32	unknown	779211	1820652	838771	694528	1294809	1538742	1912412	798364	1274308	630222	1453127	1528495	1324799	1278098	1331991	1721282	1319233	1017409	1815087
fpHILIC	Positive	422.2028	2.60	unknown	606082	428762	521912	453992	530463	699129	690645	427473	229852	400898	559803	416036	417860	573524	599188	659697	455090	517028	492970
fpHILIC	Positive	168.1028	0.94	unknown	1273368	1500430	1464051	1457065	1515798	1472047	1648585	1663120	1490321	1642258	1593181	1553402	1360925	1656934	1607867	1641830	1398908	2104426	1480605
EIC	Negative	383.3000	5.78	unknown	401397	430493	408775	564740	467807	514000	516710	540578	443747	457977	527707	464075	487653	420196	510532	475322	583269	615576	440810
EIC	Negative	233.1545	4.31	unknown	322171	272131	182261	286100	376408	304526	335742	287246	567965	235017	252746	296787	346501	316412	372921	475244	406724	269091	455638
fpHILIC	Negative	556.3255	0.60	unknown	61367140	73006936	51210696	56488872	59800268	60830424	59796664	49886660	78271920	58203500	70925944	71466216	72883984	68177656	71411144	71338376	62529368	49173528	71965648
EIC	Negative	576.3314	3.83	unknown	6821032	14557815	8826743	12267980	10732553	12350424	14173661	9829978	11388440	8433279	15637802	15981819	12351801	14099885	14170114	13448502	12523128	8500132	13245617
EIC	Negative	425.0807	0.64	unknown	26870	61020	17193	28848	61501	77847	70090	28366	59494	36465	65647	73477	82686	58939	70114	70318	69445	41307	63466
EIC	Negative	252.0735	2.05	unknown	0	153412	0	0	205127	187635	185555	0	165650	0	145138	157333	122267	225912	267769	151263	170790	0	198121
EIC	Negative	398.2026	2.91	unknown	1075470	1352870	953847	1091799	1206946	1129540	1302291	1092538	1222451	1087518	1287898	1315275	1241660	1392747	1337352	1216303	1198500	1285108	1275917
EIC	Negative	291.2333	5.52	unknown	207548	289213	216507	248108	310748	237813	300666	196379	255907	190564	352215	259215	247979	288802	302415	327016	302720	204417	243767
EIC	Negative	431.1393	4.47	unknown	447796	433993	424320	498870	502576	527887	436827	506270	557103	557327	526779	489425	542294	532457	581470	537527	553074	532781	480578
fpHILIC	Negative	188.0354	0.96	unknown	1869110	1463270	715537	563171	1228175	2298127	1348394	565071	428907	632328	1104820	1136139	824000	1063812	1854156	1140842	1267680	788615	1278447
EIC	Negative	359.2080	1.96	unknown	0	621242	0	0	517729	448310	498737	0	481146	0	522734	513003	511716	462368	515654	469205	530713	0	550075
EIC	Negative	264.0054	1.92	unknown	580225	863103	0	0	792090	647805	821694	0	860156	0	1004953	1027429	1232200	1143875	368069	932283	1100339	0	913887
EIC	Negative	286.9447	4.27	unknown	204504	283809	201022	231764	325736	273063	263894	167918	267257	180837	306784	267544	256245	186755	231352	306252	237483	282629	320814
EIC	Negative	585.3865	6.26	unknown	1058658	926284	1068454	1061467	1005968	1048706	1075822	1013721	1070374	1061891	1068494	1195398	1074313	1069232	1050846	1129562	1099981	1186217	1074113
EIC	Negative	584.3837	6.26	unknown	6465406	6146303	6756030	6631117	6714570	6472316	6998064	6476701	6673901	6844941	7031443	7509544	6751181	6623669	6785484	7618425	6777973	7301116	6939369
fpHILIC	Negative	142.9986	2.43	unknown	660154	1001504	674982	533464	813953	1082462	686325	641203	823377	485769	759621	878531	789858	788798	872931	1022469	691811	920183	793566

EIC	Negative	227.1285	1.00	unknown	70342	73985	67210	71647	68743	71841	71279	73172	64692	66369	72493	75387	68980	68419	70834	75334	76494	80413	68893
EIC	Negative	240.1323	2.81	unknown	381527	552496	298453	392385	527598	594521	582909	358920	476845	497778	729961	542045	516682	542807	543786	591092	556654	470107	542900
fpHILIC	Negative	160.0615	1.61	unknown	185581	0	2275990	0	2826103	199532	541132	2019171	2616208	2909964	342969	0	3064907	3185108	0	0	3068474	2372492	3291270
EIC	Negative	422.1593	3.11	unknown	532409	389224	479359	388205	523284	498383	540307	440404	576412	475050	494934	455662	473709	651750	495217	668654	468651	479772	633502
EIC	Negative	528.3216	3.87	unknown	997927	2143147	1014573	1139420	1570430	2010704	1821382	1297698	1498956	1434631	1964594	2087354	1892959	2195358	1997996	1759032	1674192	1603446	1698029
EIC	Negative	266.1517	4.60	unknown	1693230	1179456	893313	1835537	3192292	1089044	1492697	767506	1387740	1021299	642716	1171013	957664	809993	733042	2116450	2906201	1328357	1363097
EIC	Negative	424.1563	3.11	unknown	178885	138525	162171	132187	173056	179052	190817	152453	210383	147772	160873	150640	161178	221037	194887	239652	156989	156356	217130
EIC	Negative	412.2708	4.04	unknown	614595	722145	430994	451494	608361	577788	574356	717288	576701	577539	679429	585109	635523	483880	713012	487585	681178	677393	697730
EIC	Negative	269.1406	2.72	unknown	547118	573097	368846	424400	546508	563979	614543	546392	532647	692762	516189	464497	538892	511158	614417	493845	589217	632156	665522
fpHILIC	Negative	182.0456	0.47	unknown	2513888	1246067	1538800	2496021	2308880	3998720	3717276	1053442	1014981	2752395	2471384	3784726	1837967	675216	1576716	1864364	2298103	1468923	5715281
fpHILIC	Negative	259.1298	2.69	unknown	2428954	2601414	2123303	1214919	2307529	3563528	2209252	981829	1465929	1779272	2534564	2463927	1789634	2844575	4380984	1754469	1602960	1224116	2293120
fpHILIC	Negative	867.5579	0.43	unknown	3847182	5497277	3277674	3258144	4818583	4355643	4029582	3275627	6327088	3803357	5557441	5217264	5720858	4876459	5234667	6050984	4863610	2907742	5339962
fpHILIC	Negative	391.2860	0.38	unknown	759852	334500	568586	667633	761464	722716	651971	421866	265411	799648	568936	672835	266218	430069	613811	602630	386840	962360	458617
EIC	Negative	429.3342	3.69	unknown	1282693	536040	318850	422608	1453507	740084	690743	324056	561191	518821	673482	292564	607927	536202	359833	572468	1768562	882793	454829
EIC	Negative	382.3773	6.94	unknown	72705	73857	71011	76010	102970	71217	72233	96812	108163	109889	62758	169400	73268	82981	68264	66482	84831	182647	81698
EIC	Negative	511.3642	1.91	unknown	0	124509	0	0	176210	117151	142663	0	148616	0	116512	86344	117394	113408	206423	217812	94031	0	108698
EIC	Negative	431.2953	3.03	unknown	109436	173824	121329	123248	137610	131214	127470	149654	167632	115152	151150	139530	151776	138716	133326	148368	155571	166687	161494
EIC	Negative	329.1520	2.78	unknown	1861990	1637052	1484456	1324779	1700201	1539415	2118947	1621025	1785102	1479222	2019627	1767770	1944261	1681694	2048256	1639872	2016676	1663330	2038013
EIC	Negative	383.1834	6.31	unknown	143520	0	636840	501131	190278	425868	227189	540377	481420	521424	823862	601111	303535	501715	632045	400359	340652	334194	295438
EIC	Negative	421.1558	3.11	unknown	2510752	1898289	2158030	1840777	2500023	2452731	2585367	2063054	2794566	2125566	2306691	2113042	2246298	3006958	2525638	3252089	2208832	2299212	3024699
fpHILIC	Negative	177.0226	0.89	unknown	1636219	1592723	1344040	1345558	1585303	2961206	1759044	1145990	1508866	1549832	2048850	1924600	1968310	1925335	1837709	2652948	1731431	1454245	1945715
EIC	Negative	489.3230	3.69	unknown	156031	156528	72800	189939	143536	234300	128143	185033	163448	259875	191478	160643	184264	131097	199374	206558	147221	173286	191219
EIC	Negative	642.3423	3.47	unknown	168472	292503	119877	151479	235606	259459	267039	124218	286673	134715	257858	234022	210569	162269	215556	292430	280076	109970	361784
EIC	Negative	245.0819	1.02	unknown	53329	56837	56753	47028	53078	52286	62131	56334	47005	47596	65398	61434	56578	54148	60167	61825	57160	56879	55032
EIC	Negative	610.3375	2.01	unknown	316609	677041	348542	0	572428	621874	700829	124693	439819	0	740606	817758	342940	463873	505086	665409	595645	0	877728
fpHILIC	Negative	103.0038	0.99	unknown	3859386	2596895	1538897	2468622	4212023	3825045	4220743	3416659	5679790	1703628	2522246	3907104	3884282	4705401	5143336	3858386	5312848	3624978	5020869
fpHILIC	Negative	143.0714	0.43	unknown	663858	495239	572907	828020	623921	571976	744616	479700	578265	602182	628420	824097	999024	495925	437406	964670	856125	538039	428741
EIC	Negative	301.0720	1.80	unknown	230412	816059	503090	0	548516	523252	768179	373859	456872	193733	712255	147947	117694	155156	383352	1982615	63490	0	443596
EIC	Negative	345.1713	5.57	unknown	277969	363909	171428	165832	521128	277982	0	262955	152578	490860	29118	86713	593314	0	0	479226	183281	644328	0
EIC	Negative	264.9975	1.92	unknown	0	268620	0	0	252061	205278	271827	0	281755	0	332056	322191	386341	395086	114026	294417	372823	0	306652
EIC	Negative	413.3013	1.97	unknown	0	147915	0	0	152834	126278	102273	0	117604	0	176374	103291	125666	107055	136217	131732	134865	0	134205
EIC	Negative	299.1143	1.43	unknown	0	222824	0	0	216541	192354	209481	0	200721	0	232970	199223	240910	188791	237105	182791	237652	0	224626
EIC	Negative	357.1686	3.72	unknown	240500	418256	170863	159758	407296	164356	271545	267499	310716	118279	167852	223295	350243	143306	267203	287423	229931	254322	492082
fpHILIC	Positive	399.1436	3.78	unknown	756610	487532	398489	578145	517285	879750	518514	412589	541271	561508	584071	721205	517046	573193	560899	608373	624820	664917	632813
EIC	Negative	325.3112	6.75	unknown	468754	522166	458199	659475	969010	641472	686632	758140	568410	612722	694444	805958	624842	501365	844784	730236	684577	1118788	528005
EIC	Negative	281.2383	6.47	unknown	9442809	10838308	10036869	10102782	10065027	9015881	10664561	11184995	12574848	11474180	10312832	11540411	11496745	10930411	10258377	11784624	11968313	13535157	10154943
EIC	Negative	381.3747	6.94	unknown	242789	243915	261299	273718	387743	261954	274322	344589	345613	404637	220010	609309	241857	300702	258143	242675	307649	692753	240080
EIC	Negative	280.2353	6.47	unknown	83921490	95825064	89129946	89896461	89950140	78626823	92630181	99208223	110253731	100021111	91137397	98827568	101743676	97105613	88996322	102840268	106510196	120504908	88875262
fpHILIC	Negative	152.0546	1.01	unknown	385779	473713	197891	270672	714214	697657	627054	233071	574646	377177	585287	514357	576837	412441	699949	579165	801810	227094	763072
EIC	Negative	275.2316	3.84	unknown	203807	228566	195379	199928	243381	227254	197583	198786	210391	203238	224392	225069	221671	205418	246992	225520	222910	239806	227254
EIC	Negative	397.1998	2.91	unknown	4493853	5423076	4073533	4656338	4774366	4672853	5569470	4403647	5137868	4731237	5436612	5363106	5293742	5675691	5351216	5293226	5325277	5240542	5291452
EIC	Negative	293.2489	6.52	unknown	465897	556197	766945	1114838	668525	645618	732416	927255	1044850	754735	874509	815267	1033014	893053	773566	873796	1031085	941030	718201
EIC	Negative	239.1077	3.33	unknown	118210	132966	125783	136921	133226	154150	135512	135308	121407	111923	140474	133842	164022	145096	155134	141890	157762	131873	147934
EIC	Negative	320.2261	3.84	unknown	2839718	3979601	2620787	3870695	3857850	3980031	3768637	3193376	4219859	3550047	4007226	3733466	3793133	4377327	4341839	4371864	3794619	3829560	4226442
EIC	Negative	359.1244	0.84	unknown	200708	127421	105727	179726	150844	171254	164145	217003	177525	128408	115606	163037	192713	200124	169894	159003	229722	212417	182042
fpHILIC	Positive	216.0652	3.63	unknown	717573	692845	454691	541319	637230	943812	861091	525233	1012903	483523	529863	699860	667850	895286	740654	746396	926811	824735	1068744
EIC	Negative	326.3143	6.75	unknown	111767	126466	117226	148166	228846	144393	149566	178852	139303	137516	155003	198652	146102	120455	198667	151868	159774	266338	133921
EIC	Negative	252.1718	1.14	unknown	258259	255511	210942	181680	215372	193715	250729	288011	223171	213876	275343	243324	283399	207725	278136	199628	272505	264641	253294
EIC	Negative	321.2307	3.84	unknown	488148	621534	383096	485813	562814	635053	576222	497141	573417	505027	623695	567877	584977	565897	613749	604139	644442	592164	632255
fpHILIC	Positive	115.0868	1.60	unknown	1035458	773115	1054740	808270	892190	1468123	450798	1139613	2376163	835833	925721	1428657	1467149	1869575	881225	1108067	1979175	2275379	626301
EIC	Negative	526.3154	3.87	unknown	18948658	38561029	17989455	20864749	29108215	36913750	34671797	22159994	27742914	25655451	35241706	38371862	35076142	40751444	37700465	32471740	31361014	29504482	32519574
EIC	Negative	236.0961	1.66	unknown	85081	97715	0	0	155627	184300	137088	50031	156497	46814	79977	70381	81004	97465	189101	272899	144029	16412	120267
fpHILIC	Negative	866.5534	0.43	unknown	7067893	10342195	6510126	7002798	8674053	8308939	7537003	5905194	11219417	7495468	9963472	9326928	10427991	9126725	9184041	11064950	9592172	5755175	9666687
EIC	Negative	268.1557	2.05	unknown	382405	229992	62610	155578	204152	445512	242942	139231	198872	131992	297038	333300	207348	157482	465716	302644	306050	55862	207524
EIC	Negative	527.3187	3.87	unknown	5126942	10485089	4870321	5536652	7947644	10175816	9282655	6006801	7545998	7021925	9578304	10449823	9564495	11025881	10109383	8912001	8511456	8062143	8931447
EIC	Negative	531.2732	5.54	unknown	918295	1730623	855754	1186039	1869424	1037180	1928405	1080964	783390	1015378	1368976	1315805	1105951	1540408	1783151	901138	2056826	1395101	1151530
fpHILIC	Positive	190.1183	3.52	unknown	767722	817322	636790	1021421	1105341	1504119	783994	466053	936262	750456	1306347	1063329	967827	543214	1075694	1620287	1082305	766844	675241
EIC	Negative	302.0752	1.80	unknown	61264	136099	100214	0	107584	95822	133865	80840	73564	18287	150777	27869	20232	29708	89685	341058	5883	0	98881
fpHILIC	Negative	886.5531	0.38	unknown	4397458	4786626	4749186	6339997	5067540	4451905	4457498	5264317	7524156	5539912	5070652	6058144	4664808	4755343	6942689	5393284	5695668	5036691	6089289
EIC	Negative	295.2028	2.11	unknown	3958698	4736000	2800036	3111164	4297763	3749313	4263682	4464646	3863234	3624138	4695694	4041524	4586474	3827435	4722470	3613075	4634701	4364641	5154934
EIC	Negative	381.2291	1.32	unknown	21367	118656	0	0	278922	400299	172266	0	119848	0	234640	143815	80982	75270	481380	154376	136240	0	287120
EIC	Negative	358.1513	1.41	unknown	0	156543	0	0	114119	120361	144443	0	67593	0	174164	178157	144495	219276	141471	121822	90602	0	133233
EIC	Negative	446.2034	3.11	unknown	2452349	2239319	1874036	1801195	2911595	2822223	2987094	2010102	3205452	1997321	2652408	2415990	2326015	3547664	2798414	3813168	2466818	2456275	3674529
EIC	Negative	596.2459	1.03	unknown	0	167335	0	0	110132	166801	243960	19086	120920	0	156320	211316	158165	155399	120280	115756	135500	149019	200930
EIC	Negative	377.2333	5.17	unknown	223454	107994	236532	195638	141286	207987	114685	206045	289481	165255	123026	204686	187197	156956	166114	249622	213951	231508	187782
fpHILIC	Negative	136.0331	2.95	unknown	6493614	3825367	2283328	3247291	5774338	5199921	5181347	4029545	6669290	2444058	3000779	4849545	4307145	0	7692337	4483057	6310333	4448103	6429284
EIC	Negative	280.2353	6.58	unknown	1898122	1825198	2442738	3011830	2513597	1751716	2471281	2948253	4026713	2445060	2753605	2720637	3558377	2596014	2362522	3073064	3236760	3851782	2470883
EIC	Negative	445.2001	3.11	unknown	10469213	9701529	8056386	7758299	12615178	12175620	12839827	8430381	13764644	8809245	11288738	10398166	10066518	15091445	12071577	16300268	10886797	10578445	15601575
fpHILIC	Positive	211.0684	2.47	unknown	562156	694451	531748	863826	710787	813650	502660	683194	720181	553033	721665	629807	640258	554155	879999	933732	512789	689817	736404
fpHILIC	Negative	260.1336	2.69	unknown	347147	304079	223806	156814	277179	440433	242656	122603	191922	186122	284578	322072	224810	325901	528160	235908	200755	138268	291805
EIC	Negative	619.3468	2.93	unknown	905568	630917	331663	380193	645807	1060855	759581	616834	808005	452366	513204	884985	567267	436606	829714	787280	837851	470315	1332768
fpHILIC	Positive	148.0965	0.94	unknown	1299031	874456	866791	974005	1884828	1091051	1254393	462013	1029968	1075649	1383251	1553893	814198	1130626	1113993	965471	1457239	1672968	1052759
EIC	Negative	568.5395	6.63	unknown	1404458	2947647	2713083	3812555	4877032	3405206	4229012	5413693	5736567	4067484	4207014	4987909	4132912	6482496	5294244	4954116	6796717	4606263	4244015
fpHILIC	Positive	243.0519	2.61	unknown	750612	2124634	1891247	1120736	1571659	1596841	1862163	1331561	1345830	1439968	1722562	1513833	1555660	1880283	2017725	1575501	1479901	1829931	1535063
fpHILIC	Positive	176.1276	3.11	unknown	605485	1235968	373753	268486	941786	1105885	928798	430439	670840	546909	1645825	916518	1187516	933564	948529	1058084	816657	350359	1291441
EIC	Negative	613.8356	0.86	unknown	57239	202186	118419	114601	146391	226700	97545	103146	88010	81780	161021	127025	149217	117870	209048	141514	187444	100830	86105
EIC	Negative	296.2060	2.11	unknown	696061	848523	543084	583504	765017	680373	773836	806927	708603	663750	858421	725159	819463	690048	854771	637891	838665	822656	937207
EIC	Negative	340.1615	4.08	unknown	21219	17098	22137	146470	50919	13037	93799	80595	129777	87666	59733	109113	109747	32176	20215	113987	94338	137602	37266
EIC	Negative	311.2869	6.63	unknown	358096	669709	747177	1056601	915527	761129	914209	1064695	918066	730062	753718	1460922	1025103	869557	787845	1347902	981029	1170711	727457

EIC	Negative	491.3380	2.82	unknown	376569	869218	341617	538040	807479	825988	605386	602765	827247	677097	919003	697713	831045	908844	867484	833371	691399	767079	868106
fpHILIC	Negative	137.0346	2.95	unknown	1403565	847080	554171	821322	1373384	1248259	1229135	948084	1523401	540008	628627	1212411	1042461	1570311	1692854	1308766	1328621	1125971	1486675
EIC	Negative	253.1566	0.98	unknown	157362	180092	137767	128350	143868	132843	166990	198668	153235	144730	172672	161173	193916	151126	193659	135086	186406	185229	173962
EIC	Negative	248.1326	0.78	unknown	147700	159029	140119	137397	161337	221490	208766	123226	120130	153587	201956	336876	260204	193804	123156	239349	178582	125343	251242
EIC	Negative	339.1792	2.09	unknown	57410	84510	0	41399	61115	85188	98044	0	67684	51357	57234	144145	150340	86464	77321	103184	86861	0	76044
EIC	Negative	380.2489	5.86	unknown	166259	122952	176069	187341	102968	177361	146652	189213	241451	189103	133780	198788	160178	94240	128811	286225	205856	163650	160047
EIC	Negative	567.5357	6.63	unknown	3425895	7246630	6958497	9400867	12264689	8392431	10525522	13175753	14398281	10569310	10876396	12926961	9807582	16560722	12929684	12924800	16902453	11519102	10448066
EIC	Negative	450.3312	2.93	unknown	364096	702043	347725	300357	747843	531629	689262	480405	599986	299586	566569	543901	553359	550200	832671	722756	717464	496336	589882
EIC	Negative	412.2709	4.09	unknown	283317	317917	220364	209229	268473	262657	281594	331799	244437	288927	345502	263858	267620	234660	332020	224809	344263	285090	344638
fpHILIC	Positive	235.1283	3.70	unknown	389990	447822	311356	294798	453976	467555	364535	294617	325647	348612	459772	403341	317426	566639	493717	462616	396212	310141	439531
EIC	Negative	407.1470	2.85	unknown	92201	152645	209355	239676	133044	206357	171932	194226	102067	306175	288262	205450	123129	100514	184775	173297	212862	248800	71310
EIC	Negative	430.1744	2.03	unknown	278895	340665	338339	224221	301533	402797	566330	134827	278998	266856	468058	710111	422187	333395	380266	573384	475251	127552	302562
fpHILIC	Positive	219.0756	2.62	unknown	691775	750178	746033	508798	638913	765295	830419	561312	693749	589233	766725	741490	818700	914504	968566	772690	611059	726956	772112
EIC	Negative	430.3375	3.69	unknown	344764	127429	35551	105392	367212	203221	161059	76750	151297	136822	169558	80907	157907	131581	91988	164849	460043	226859	115369
fpHILIC	Positive	248.0945	3.40	unknown	272265	217389	50654	658971	347296	379976	256948	172735	410331	468734	299382	266246	417880	92747	291938	491597	501806	271488	175302
EIC	Negative	612.8336	0.86	unknown	472696	1967955	860435	815571	1346835	2155132	891498	967696	696902	601468	1534006	1138599	1440936	1122158	1936371	1324529	1781253	978789	677585
EIC	Negative	276.1688	3.72	unknown	788816	2137861	501098	628144	2146931	773888	1421041	872777	1910549	406440	778325	1023146	1646592	779090	1322753	1552111	1264279	1045262	2695711
EIC	Negative	423.1532	3.11	unknown	910632	661828	701809	622631	827377	804922	861875	707434	956864	750882	809587	724330	753287	1024039	889765	1129883	754397	776509	1048571
EIC	Negative	385.1784	3.11	unknown	116619	91887	89484	114115	152174	127791	131940	88784	170585	97571	108982	108796	143891	156711	133919	164280	125651	142886	176101
fpHILIC	Negative	175.0726	2.74	unknown	314399	254129	176624	273869	251421	585985	274088	224390	189983	217380	423511	393153	343424	496318	270204	396304	322457	197210	502516
fpHILIC	Negative	230.1036	2.74	unknown	764598	658667	440684	576551	506951	927502	538819	385313	758559	493950	666210	654459	613355	550261	710187	874854	708170	453388	703727
EIC	Negative	251.1483	2.30	unknown	198385	199997	72972	230942	247336	354215	217914	93508	144313	248160	226315	274693	221441	137645	265606	285088	236811	219743	163563
EIC	Negative	425.2561	0.86	unknown	93596	117939	100121	147684	221471	303134	140902	220118	130593	108064	152804	91325	148120	118057	187655	184023	157733	428434	168655
EIC	Negative	371.2440	5.69	unknown	255735	263490	202860	191740	205517	198390	224904	272210	237009	240637	268869	229944	275939	224857	248062	191359	272850	286291	263284
fpHILIC	Positive	159.0911	2.61	unknown	11171388	13886672	12809254	9971223	11736143	14578389	13850490	10252377	10208445	11632699	14172910	13741187	12475249	15238026	15497219	14367478	11900292	12693830	12818812
EIC	Negative	613.3353	0.86	unknown	178289	757878	333929	309583	509581	809584	337464	356582	266159	239048	607741	440247	528293	411958	728447	497801	679560	359683	267901
EIC	Negative	365.1978	2.58	unknown	216303	142862	0	111314	87462	180631	206074	20021	158721	88814	141424	272423	247637	143300	80778	198522	150110	84621	212570
EIC	Negative	389.1975	2.18	unknown	0	54559	41652	52111	37744	83445	63242	34663	73257	30413	37411	85867	74024	57051	26079	74147	62445	104257	58449
fpHILIC	Positive	124.0587	0.92	unknown	1234051	1674336	342471	963290	2164961	2293291	1875094	646957	2784287	746297	2051522	2248737	1811505	1840132	2605095	2614869	2127197	1244180	2281217
EIC	Negative	502.2714	3.87	unknown	567739	1004175	509869	604843	784526	952200	938509	556486	748706	711765	947538	1035487	964184	1083968	997785	863486	854261	854829	862183
EIC	Negative	396.3926	7.00	unknown	90206	82331	83368	97671	145989	72641	75336	102835	123788	124826	74421	157668	78302	117685	112762	88351	110403	183376	84830
EIC	Negative	310.1663	2.77	unknown	475156	531427	252216	304675	606810	498405	484582	506052	475749	493710	579293	505139	708229	450200	576761	458149	605421	584675	638861
EIC	Negative	344.2887	6.63	unknown	86437	157362	125272	192065	175816	179146	263155	222575	255308	196850	204165	244927	241255	289586	225077	190957	310638	274845	219945
fpHILIC	Negative	178.0510	0.93	unknown	3311755	2907221	2938052	983269	2339728	5039052	2811913	2001641	2055605	1388990	3222163	2842388	2651647	3684211	4150551	2407635	2398180	2618808	4311122
EIC	Negative	598.2549	4.64	unknown	274248	387278	374112	520054	600715	578015	558683	651440	711110	579677	574533	585811	663259	677354	765480	565731	754231	728730	642390
EIC	Negative	237.1088	2.78	unknown	535853	648806	251878	329121	666430	568634	771684	498262	636210	505562	746519	691785	695717	626493	746791	634669	748177	607916	768915
EIC	Negative	327.2180	2.23	unknown	425427	225833	382277	256046	244608	489781	409759	302561	689537	295983	316551	621148	619533	378275	167754	605994	587022	531268	493092
EIC	Negative	270.2159	5.73	unknown	123685	129439	116509	148550	153251	146339	139007	119443	116513	129616	112983	117147	124118	109484	107957	224616	147752	133612	139610
EIC	Negative	339.3181	6.71	unknown	146866	215589	147929	257254	300317	216200	215347	213641	240124	199323	342734	296437	297806	235788	296255	280576	279728	297628	171406
EIC	Negative	275.1656	3.72	unknown	4304593	11327164	2587285	3409496	11303664	4048755	7466679	4547668	10193116	2241536	4260343	5278033	8893228	4141503	7207107	8218080	6708718	5636736	14278294
EIC	Negative	415.1976	6.06	unknown	0	0	0	0	759735	0	0	0	0	109498	476585	519339	0	0	361427	489661	0	0	0
EIC	Negative	569.5419	6.63	unknown	289001	615370	556133	1224966	1071064	705057	859799	1075332	1176914	888866	851008	1172160	800291	1547566	1014173	1084281	1449081	1033710	895701
EIC	Negative	447.2058	3.11	unknown	449976	400192	316152	313975	523889	488038	519678	357445	584078	374492	476885	447397	443379	613598	494852	685866	460693	448363	681562
EIC	Negative	416.3009	2.64	unknown	420581	515902	419040	584036	570035	659626	673570	490840	586245	532318	726629	695528	756218	1053181	706345	739940	723009	483567	687624
fpHILIC	Negative	125.0179	3.34	unknown	719989	491915	471331	500808	536143	596781	630333	466233	538518	500616	522857	493347	547072	690312	691014	482743	669668	660749	598465
fpHILIC	Positive	125.0710	2.84	unknown	290722	415605	273028	274379	318188	350294	340535	268553	533803	263831	348791	324307	458355	266572	491204	461204	397764	316051	364359
fpHILIC	Positive	190.0505	0.96	unknown	1430821	1054814	802727	670161	1018684	1859217	1193237	553919	437284	741601	1141582	973381	758911	952814	1637796	990719	1037109	818712	1193401
EIC	Negative	380.2083	2.28	unknown	223223	181609	162162	232392	164919	145041	166992	324069	166386	269657	176561	138848	183849	138819	196359	164446	197253	318777	209083
fpHILIC	Negative	478.2937	0.51	unknown	2944274	3567916	2382294	1454782	3173763	3754482	2896105	1964009	2150903	1432308	2809048	2963261	2054166	3891047	3357090	3394272	2465716	2431259	4000736
EIC	Negative	310.1747	2.73	unknown	535511	746282	330809	482293	660580	589367	637007	552400	594226	586125	772907	664186	786517	643021	683895	557888	688952	692854	784003
EIC	Negative	295.1818	2.78	unknown	2194066	2579599	1370299	1384050	2477079	2320791	3095828	2039067	2468265	2118690	3124447	2618086	2918033	2491668	2927246	2440383	2954756	2454952	3173001
EIC	Negative	433.3331	4.01	unknown	144397	97081	159590	152899	143830	112945	133527	156531	104999	142761	109197	104119	94407	99053	96748	180656	102644	255068	98399
EIC	Negative	618.3421	2.93	unknown	2653051	1992950	985581	1083078	1830428	3105808	2222778	1836908	2412685	1425248	1688460	2655163	1761727	1433517	2410030	2472099	2736385	1333971	3886554
fpHILIC	Negative	152.9958	2.73	unknown	7802886	9832937	6606544	5981656	7861930	9994991	8695239	6412825	7280757	6772407	8106717	7494707	7486561	7989744	12565312	8954062	7774917	7707793	8315699
fpHILIC	Negative	138.9707	4.51	unknown	1804421	1828417	1616312	1792790	1776695	1892761	1428655	1687626	2084602	1875299	1735426	1684649	1819856	1487876	2048625	2147216	2019721	1880735	1892423
EIC	Negative	378.2005	0.90	unknown	34294	56609	31750	24918	54930	32052	35224	29552	29739	31211	42326	10886	60397	74806	58619	51265	23812	26457	44286
EIC	Negative	360.2223	5.66	unknown	194330	15393	258335	21786	121978	122268	152369	142069	135730	181214	162034	147784	202919	138839	133452	198873	198742	17223	211423
fpHILIC	Positive	123.0554	0.92	unknown	18987636	28159014	4852611	15391011	35126280	35126588	27121816	11217645	41810372	11686977	33438002	34783780	28214928	26645240	40355924	39682820	36702520	19646614	33085924
EIC	Negative	407.2439	2.03	unknown	0	279685	393991	0	360459	218564	274388	322162	171663	464244	352847	141929	402580	269558	211357	344951	177263	579299	206032
EIC	Negative	346.2138	2.52	unknown	0	98796	0	0	0	84525	218646	0	116986	0	116484	51723	187719	236169	162683	190808	0	0	120219
fpHILIC	Negative	264.0052	0.38	unknown	295115	412085	1020743	920567	329933	308278	1026381	354632	1182763	890245	634994	861229	1428540	803580	298556	708728	979645	730122	893194
fpHILIC	Negative	203.0545	1.02	unknown	3009924	2997126	3379163	1978025	2582817	3632716	2476425	2569338	2942582	2991569	3028356	2572334	2353063	4003648	3450986	3359942	3183350	3030195	2991528
EIC	Negative	366.2372	1.77	unknown	66275	85532	60167	140462	178844	67769	142796	165287	124374	88064	113408	66500	119900	454259	255274	235008	116122	0	159741
EIC	Negative	612.3324	0.86	unknown	691386	2970042	1231922	1223881	1991433	3152445	1295940	1352275	974860	894558	2303083	1678134	2037311	1592585	2833575	1946712	2661803	1467706	986607
EIC	Negative	366.2659	5.08	unknown	211753	220767	167404	168906	210535	220951	168088	240905	197344	203770	213625	163656	238609	201859	210744	216972	248037	244179	253015
EIC	Negative	614.2851	4.01	unknown	371030	460010	467852	617553	502595	566583	704684	664644	634882	509102	749609	840988	640674	787681	711727	701308	704919	600481	756054
fpHILIC	Negative	498.9304	0.34	unknown	1953871	2324336	1737281	1919226	2179937	2264467	2080173	1604537	2220140	1762232	2561578	2301867	2195756	2280176	2476838	2497411	2358800	1917565	2449729
EIC	Negative	618.3422	3.37	unknown	1664002	1516034	993604	930315	1112660	2435949	1370041	1642395	1504517	998146	1228426	2348437	1527513	856542	1686272	2041533	2090853	1052753	2251563
EIC	Negative	508.1727	0.71	unknown	88680	96386	140953	105275	109174	145796	191666	184127	144021	157826	219654	201086	175080	152895	192009	150045	175800	178984	180811
EIC	Negative	286.2735	6.63	unknown	121549	213859	171832	216522	264814	229515	240900	299177	352077	256551	270141	340507	244443	366545	318296	370055	363949	272863	245699
EIC	Negative	251.1323	3.23	unknown	367452	0	0	0	0	537702	0	0	0	0	0	0	662432	0	0	0	609596	0	0
fpHILIC	Negative	181.0372	2.74	unknown	633306	452520	394586	544979	536998	944721	589759	440947	569806	586941	684428	677689	619640	363985	689496	795846	775189	708586	525948
EIC	Negative	251.1403	0.99	unknown	492684	481651	397373	370418	409057	404218	474849	563974	404286	437700	495149	462547	582145	433397	552117	414106	526661	541185	525377
EIC	Negative	433.3331	3.50	unknown	106191	58942	133725	111818	72137	73316	79667	240193	73696	121382	72442	108238	59007	40013	117754	138222	45603	263998	46044
EIC	Negative	415.1976	2.31	unknown	0	0	0	0	243324	0	100498	61728	0	0	0	0	554789	901275	460271	0	0	0	0
EIC	Negative	625.3607	2.34	unknown	120586	229251	134694	55354	201555	144308	268099	173785	270959	65689	199883	184869	263983	140751	273361	328864	258478	104516	236714
fpHILIC	Positive	272.0881	2.71	unknown	1103219	1436008	1043169	751103	1254449	1641742	1143736	851650	1204039	916266	1157238	1180308	1031659	1330489	1879796	1417796	1097247	1284635	1241519
EIC	Negative	239.0928	1.43	unknown	0	151681	0	0	150880	128142	141638	0	149187	0	168772	143094	170270	133598	170073	131471	179490	0	166205
EIC	Negative	294.1792	2.78	unknown	17177943	19396666	10218463	11211859	19901680	17383069	22809950	15207634	19147568	16032582	23757187	19628594	22148213	19023986	22778400	18767362	22484781	19041349	24265422

EIC	Negative	293.1541	4.05	unknown	362398	458669	268011	257802	405318	346258	304535	343340	283768	267913	302582	265283	327115	250008	368142	380039	359911	403659	401710
EIC	Negative	236.1050	2.78	unknown	3479044	4103413	2104311	2409339	3948777	3570630	4958731	3192833	3837984	3223684	4891683	4044469	4550858	3934670	4771411	3845527	4723863	3843368	4993880
EIC	Negative	269.1761	2.66	unknown	87153	147158	91384	107327	98406	165288	105597	122116	107484	158394	168804	181840	186267	77004	117986	154815	164614	114267	129168
EIC	Negative	293.1762	2.78	unknown	88530877	104552788	53886893	59624776	104910929	92817219	123366257	79855168	99993757	85213213	126581390	104240113	117810089	100643480	120820425	99217064	119570247	100315475	129823247
EIC	Negative	474.2630	3.81	unknown	0	1923851	1691276	1039515	1315247	2519471	1262415	1376548	719321	1080321	1866113	1144405	944354	3711928	2153413	719556	1492010	1392829	2426449
EIC	Negative	298.2470	5.03	unknown	539977	373234	760522	567274	625525	579643	591038	583258	738307	423068	353032	508773	521351	411640	257530	1505600	580897	841779	362331
EIC	Negative	377.1970	0.90	unknown	145411	224186	130776	97593	244928	141365	124782	116961	136066	127055	164095	52136	214785	311866	220058	228827	106957	131080	183673
fpHILIC	Positive	242.6414	4.20	unknown	603597	234525	154162	259294	602190	611149	473682	438010	688635	230224	263498	522256	440191	611743	1036271	470982	609095	466938	623643
EIC	Negative	297.2436	5.03	unknown	2701722	1777803	3550406	2866475	3295723	2805048	3091653	3018870	3618199	2119251	1600772	2666808	2437892	2165461	1385058	7600901	2809038	4261514	1989372
EIC	Negative	579.3586	3.65	unknown	1074108	1216278	754723	735485	1070760	1018970	939056	1161291	1012980	937365	1024913	938640	1083383	769668	1202767	1076359	1078992	1253528	1321015
EIC	Negative	395.3892	7.00	unknown	236451	213470	244617	258139	324182	255884	232891	326484	408366	454776	198877	545700	221901	338735	309307	236691	357340	626644	236520
fpHILIC	Negative	279.0540	2.95	unknown	399114	193545	46801	174578	387466	332958	382793	244166	593792	91522	135836	334857	276441	477049	594721	340532	492194	368778	419642
EIC	Negative	365.2340	1.77	unknown	251995	328169	185448	631604	793689	277693	588761	715810	539005	358189	484687	262887	569605	1936896	1178957	981315	512613	0	657178
fpHILIC	Negative	102.0561	3.03	unknown	995406	834269	890474	1188240	1331371	934242	1000386	738105	1959903	961742	1560366	1007100	1021189	932627	1119033	1662347	1576210	1589297	867715
fpHILIC	Positive	160.1329	2.96	unknown	69678944	59590404	51109288	45771852	70402216	91207784	74919728	49400124	63519296	58867008	121679432	79574968	95540744	71634432	79901440	94308968	78789952	55010992	91357400
EIC	Negative	541.2760	2.82	unknown	119650	207518	0	98304	164855	265817	149483	0	324184	124260	124621	191382	153425	118446	209024	223469	255094	127221	218945
EIC	Negative	533.4012	4.98	unknown	0	0	277826	0	114507	0	0	210444	0	0	0	193704	0	122428	0	106907	115760	0	149117
EIC	Negative	259.1666	0.85	unknown	124459	141938	136221	104369	126445	116395	137623	194166	102648	120766	158831	127268	188762	124634	164743	131785	177380	169802	131368
EIC	Negative	370.1880	2.77	unknown	160961	257543	73688	127324	247892	257513	232161	274160	256147	242309	273705	243454	271377	251861	280989	219628	313446	294247	343065
EIC	Negative	640.2980	4.32	unknown	308502	232130	135966	493365	449672	463315	541365	504612	544620	559814	483604	550981	570961	495296	592557	577746	580225	617258	498159
fpHILIC	Positive	280.0917	3.31	unknown	1828990	7688727	3530649	2574935	6051391	6716171	7280412	3509166	6565693	2667947	6263389	8365438	5889917	8736132	6307947	7751131	6334162	4801709	9296371
fpHILIC	Positive	288.0740	3.58	unknown	623574	597577	398343	606209	434099	711176	574023	376560	534657	514220	467788	462783	710723	386213	477216	718878	680300	625855	509971
fpHILIC	Negative	202.0510	1.03	unknown	25569276	24330660	26155692	15394558	20784154	29745900	20532988	20190474	23595128	24762142	23889098	20813462	20344502	34302128	28423072	26981586	27491546	24224116	24670082
fpHILIC	Negative	168.0778	2.84	unknown	1135649	1946963	1483562	1293748	1764220	1893253	1580465	1190956	2179448	1540078	1768467	1779921	1766701	1068440	1514139	2482481	2421226	1795204	1666598
EIC	Negative	273.1246	1.43	unknown	0	94596	0	0	97535	71545	92962	0	87572	0	113025	102686	96912	139232	92800	96750	108512	0	103645
fpHILIC	Negative	302.0519	2.25	unknown	738132	201962	132356	96588	272006	516661	154836	154489	347233	61580	108125	325939	207120	201956	161550	535790	294010	395045	274927
EIC	Negative	331.1305	1.43	unknown	0	55607	0	0	71132	53885	73649	0	64089	0	75097	84921	102766	53392	62858	99218	79237	0	60773
fpHILIC	Positive	161.1360	2.96	unknown	6002888	5349173	4632056	4254888	6247912	7166512	6647505	4179520	5649813	4963571	10139296	6934616	8314632	6219475	6769250	7515028	7370375	5063092	7682221
EIC	Negative	235.1705	5.07	unknown	692792	403625	388529	607879	649926	621595	732964	684559	755830	413083	379027	626780	705592	550452	750008	850065	847271	703671	818832
EIC	Negative	340.1526	1.85	unknown	147303	88738	0	0	160973	215588	213237	0	159117	0	162845	145272	187212	154898	173083	298898	179214	0	212909
EIC	Negative	287.2232	2.83	unknown	136301	147810	452326	273578	342708	320992	172341	441604	171033	363449	181805	185414	228888	194600	218624	507815	256553	523235	166082
fpHILIC	Negative	151.0513	1.01	unknown	4712569	5881986	2147374	3419208	7850953	9464513	7172906	3113918	7881182	5241257	8783524	6703786	7939764	5758775	9165814	7805479	11318270	2993506	9697990
fpHILIC	Negative	137.0243	0.52	unknown	1878485	1298251	2924232	960734	1273132	2345046	2094430	1293729	1280555	1235711	2070000	1551434	2035206	1422302	1252276	1429656	2219653	2878873	1797740
EIC	Negative	509.3496	2.42	unknown	118664	67665	197657	132942	313449	271625	227210	122950	248833	270594	169573	164108	56213	250028	347809	281069	165853	506972	68626
EIC	Negative	590.2848	4.24	unknown	211995	238674	306317	261737	291453	351669	421524	328818	280232	316513	362902	466898	470915	428081	452089	362525	381735	373096	368030
EIC	Negative	270.2523	6.58	unknown	1150140	1179017	1111880	1542923	1589348	1324657	1639095	1697475	2431089	1511491	1841915	1447589	1884660	2063753	1437435	2205519	2221481	1944109	2247749
EIC	Negative	558.2816	4.68	unknown	1928944	1455264	1645128	1979123	1861589	1352169	1678177	1897627	2091519	2368480	1822094	1973221	2044330	1626402	2133886	1994370	2073400	2234545	1860128
EIC	Negative	527.2021	3.11	unknown	146043	175209	105223	144114	218594	212073	216908	121337	250856	130518	196750	176546	176993	287757	222204	281262	205407	182668	291170
EIC	Negative	366.2659	4.91	unknown	220879	187858	143340	152121	213568	175661	188387	213026	152339	156029	191842	173273	203272	159554	213497	190531	227497	239821	223032
EIC	Negative	294.2156	4.59	unknown	220756	141479	200033	243911	218027	211418	251315	183294	323472	164680	148665	165808	163112	174254	75526	657810	213717	254578	176793
EIC	Negative	267.2331	6.57	unknown	183244	198488	253385	306070	267127	212139	317253	338892	454824	264495	324483	334562	427650	321574	281852	400574	418334	455656	290624
EIC	Negative	459.1807	1.63	unknown	34664	0	0	0	79891	227614	88361	51320	113824	0	100421	44104	111790	173753	90100	271807	55206	146043	88619
fpHILIC	Positive	281.0949	3.31	unknown	220817	786723	331818	252880	639170	598052	655399	321053	559797	224796	491125	725475	537948	836811	595374	744080	587606	425576	870772
EIC	Negative	333.1558	2.04	unknown	0	194726	0	149209	117886	230119	187448	150182	394356	205376	501800	315691	177762	496013	252826	400831	250020	0	353481
EIC	Negative	505.3170	2.27	unknown	108862	98335	102794	92423	104521	168130	60903	214540	93659	110059	117537	136452	39399	152068	147609	142574	108379	257389	91446
EIC	Negative	310.2833	6.64	unknown	3063909	5291063	5813677	8145237	7678589	5762476	7023043	6883004	7873752	5837475	6017682	11762746	8186138	6411381	6119472	11300504	8238980	9405354	5966802
EIC	Negative	312.2264	3.30	unknown	377736	306759	422468	459992	473618	363211	560895	372940	746810	297465	253506	377849	347171	335651	249985	1302587	442493	492293	449189
EIC	Negative	582.2821	4.64	unknown	1562323	1240036	1287822	1338525	1310384	1087009	1260807	1293899	1733767	1615752	1425223	1398375	1687458	1228006	1639449	1657482	1610474	1532615	1362398
EIC	Negative	367.3493	6.79	unknown	155710	217224	143354	181142	139368	280589	192157	178575	138324	230308	211235	330805	318041	147126	159065	190025	150910	348563	235601
EIC	Negative	595.3461	2.64	unknown	247008	450959	320151	214237	425417	460885	502474	179557	445954	227550	536557	535311	315827	522088	461290	521228	511646	119814	737110
EIC	Negative	245.1396	2.47	unknown	108613	128337	140485	92592	126290	107602	138964	143414	128520	145757	139620	144675	146848	122885	156774	120163	151026	162323	162394
EIC	Negative	555.2726	5.42	unknown	820737	937276	791935	1145968	971400	965644	1017975	957363	710093	1329032	982012	797854	893878	850577	1041395	934521	1375229	1270085	775121
EIC	Negative	343.2092	3.53	unknown	0	0	0	0	0	325573	264302	0	0	0	0	0	0	0	331404	0	0	0	357509
fpHILIC	Negative	217.1085	2.74	unknown	329213	303642	240406	282856	287040	486553	267655	172410	304753	238249	256617	558531	452292	263830	216158	669929	234655	357482	295655
EIC	Negative	346.1524	2.88	unknown	128659	250419	118421	165469	207567	282593	264211	181103	460266	271888	283660	409579	393188	243372	410863	676205	261062	143784	138787
EIC	Negative	389.1630	2.78	unknown	294934	372449	176249	130233	366962	349087	424643	231027	319415	370342	458408	435232	464059	368382	429243	367727	393066	334427	454117
EIC	Negative	642.3147	5.00	unknown	330639	384487	539204	533746	529233	578098	595356	602050	619505	580646	635775	710716	643999	695076	682770	653744	726695	630007	735540
EIC	Negative	447.3127	2.53	unknown	81153	169024	74165	85573	150737	126426	196945	100752	147372	71999	150125	126027	133237	116987	213837	188819	152037	140356	152182
EIC	Negative	608.3203	2.15	unknown	133514	134854	66039	0	106409	130499	128251	101098	103951	124270	124136	211213	72796	172076	123397	157109	122043	52724	213626
EIC	Negative	415.2114	2.89	unknown	404306	316941	307017	270076	272858	209788	639008	370771	540724	498628	419382	643144	677614	410527	398626	351148	445067	580760	610326
EIC	Negative	523.3645	3.86	unknown	26761	168682	39508	19604	269907	214605	226819	171220	297968	62826	229707	121062	158684	140229	385146	355110	201374	227198	217997
EIC	Negative	240.9888	1.19	unknown	199078	200591	219231	304239	357317	456592	471793	453901	482227	459021	528643	543609	531542	537372	543940	523439	564475	423878	554927
EIC	Negative	379.3583	6.83	unknown	144531	190861	144970	347675	214365	223408	229711	236671	153518	225185	215940	311398	197039	147495	206675	247554	244229	379790	164889
EIC	Negative	358.2397	5.63	unknown	190827	217618	193396	334568	218137	257553	223773	238113	236808	342365	313023	348027	369570	243888	294133	307237	283867	257403	238272
EIC	Negative	368.3617	6.89	unknown	609337	596807	650979	817500	705662	728590	742429	945659	778915	986561	792285	1187544	691005	948471	656340	543414	850000	1711281	827030
EIC	Negative	578.3552	3.65	unknown	4717623	5397364	3607877	3490334	4856597	4354325	4294037	5199000	3915935	3991971	4883156	4111859	4615786	3840218	5378173	4743041	4621951	6161340	5725758
EIC	Negative	429.1704	2.03	unknown	1099512	1538515	458355	920965	1244592	1749936	2527053	423806	1600548	1182894	2507221	3209791	2120594	1396755	2039054	3014966	1975568	639779	1231120
EIC	Negative	631.8200	0.86	unknown	99483	152611	108971	90683	150987	217907	85010	73974	80125	111669	131242	98472	124566	204128	184757	145799	142294	112545	132193
EIC	Negative	505.3216	2.27	unknown	94785	129675	91505	90211	123543	165330	82272	139334	99790	200417	54171	102716	112796	84541	135645	124631	103852	267255	89220
EIC	Negative	363.2097	4.91	unknown	96333	187089	236491	0	91772	137823	89048	117231	158377	212658	269594	211882	32642	124843	199366	124175	159461	115149	103677
EIC	Negative	397.2238	4.23	unknown	863158	857348	623641	643170	863536	644594	759988	954731	734179	687169	814163	762330	894836	691269	922869	758075	1069697	984108	943846
EIC	Negative	322.9914	1.19	unknown	66426	65645	81817	116429	120915	139920	154892	154141	178082	150199	189192	178643	196105	170245	188186	192089	187986	139970	176178
EIC	Negative	504.4149	6.63	unknown	455044	338489	334727	365174	605580	321898	781808	540938	447869	463231	436517	463447	344883	458721	743206	1074504	389447	322975	661883
EIC	Negative	358.2631	1.99	unknown	1877429	4904844	682752	1012481	75633	4803893	71548	0	3813021	1535389	79539	82616	78951	3551054	76160	4563852	81121	866035	4781814
fpHILIC	Positive	192.0243	3.66	unknown	419718	384674	384779	395487	423979	470960	277927	419375	320908	471392	326569	450031	357197	427555	468816	509729	473033	517537	295842
EIC	Negative	364.3308	6.71	unknown	200962	213283	131252	434692	223111	345350	301023	329347	233098	335559	295464	328435	362923	294763	340138	330474	338436	412281	273918
EIC	Negative	594.3423	2.64	unknown	1046402	1505979	855141	791219	1394566	1507026	1632394	668551	1451100	737326	1755810	1649853	1123293	1710582	1462241	1712474	1563290	746328	2409138
fpHILIC	Negative	326.0761	2.04	unknown	563192	332709	254479	254445	490822	610757	432977	358156	455252	235084	321229	524800	383961	461171	555159	720618	366328	613876	531278
EIC	Negative	295.2028	5.91	unknown	133778	0	0	0	0	0	158899	0	0	0	0	0	0	0	0	0	0	188681	0

fpHILIC	Negative	738.5213	0.35	unknown	243210	452466	375896	608779	700483	306376	427517	349013	881468	483530	648656	590081	578582	2092521	435763	736816	801508	518397	671900
fpHILIC	Negative	326.0761	2.24	unknown	689375	564215	360385	563766	1059957	831517	987630	723733	1259995	424240	582654	895817	940096	1020694	1189372	1484636	1021901	947686	1133833
EIC	Negative	242.0515	0.53	unknown	273416	133066	202660	269726	355339	324334	208274	171363	250216	522464	172410	143779	175342	86478	337676	206274	369982	232163	410161
EIC	Negative	293.1765	3.74	unknown	266700	194535	112939	109361	205447	187083	183750	324348	183219	165004	139912	221638	259582	195445	205595	169423	209200	246118	457746
EIC	Negative	580.2931	1.98	unknown	242611	354358	0	239021	423520	559472	520113	278512	1148892	0	795117	679092	1032188	1424516	1215050	766043	882610	0	620619
EIC	Negative	444.2244	1.99	unknown	0	195750	0	0	158497	144060	162714	0	152357	0	214121	166342	178902	156516	184299	142645	193068	0	214437
EIC	Negative	421.2517	2.72	unknown	177017	284321	179537	351735	361463	321471	247233	308676	342369	258069	334765	329838	348006	355809	395189	397643	359118	375340	353654
fpHILIC	Positive	446.1584	3.31	unknown	470318	986180	484387	376599	692570	815517	752940	517376	634815	421309	723717	834552	667491	837784	686047	1062468	727549	624253	963825
EIC	Negative	288.1490	1.13	unknown	142449	98145	110739	94430	107553	110513	121090	130063	125885	161909	140363	115331	148353	156106	143831	114904	135123	123738	191455
EIC	Negative	577.3509	3.65	unknown	13622942	15175267	8686909	9261835	12917508	11483964	10937667	13611069	10323362	10647762	12082689	11654826	12441993	9976374	14229483	12754132	12930092	15260990	15482294
EIC	Negative	287.1175	0.99	unknown	87842	65712	75831	66633	70602	64094	72372	85253	71747	92816	80352	70434	84754	93544	82187	68717	83516	84855	115323
EIC	Negative	323.1842	5.94	unknown	0	0	0	0	0	0	0	230745	0	0	396157	0	432133	0	0	275493	0	0	0
EIC	Negative	397.1797	1.63	unknown	0	0	0	0	28753	85902	12673	0	31240	0	32325	0	37556	53969	24885	67802	13828	43404	39539
EIC	Negative	301.1660	5.18	unknown	442884	559422	435725	461828	541251	501817	556842	406403	502131	448830	553457	547247	516878	620344	620400	661023	528291	534993	655552
EIC	Negative	307.1926	3.02	unknown	72840	55117	0	52084	67205	67143	90615	68180	95972	63490	46358	63420	103721	94342	44232	202074	73300	80793	66340
EIC	Negative	642.3149	4.79	unknown	105901	178970	230785	212079	252138	290178	321123	260835	254220	242685	350433	307021	373201	264261	306124	392738	395113	231493	332024
EIC	Negative	345.1572	2.89	unknown	201050	866631	66627	442993	620466	879481	727029	211040	1414840	352997	873740	1272365	1279106	633981	1230366	1978183	748799	245045	431001
fpHILIC	Positive	132.0803	2.61	unknown	1122011	1453912	1190387	823679	1087233	1442931	1596090	757495	916788	1040080	1574553	1293471	1180382	1721904	1800316	1257516	1243686	1254470	1424837
EIC	Negative	594.5562	6.63	unknown	568075	1452767	1640367	2577898	2454357	1583452	1946751	2026789	2247885	1621617	1691509	3163220	2043456	1824609	1802360	2822197	3703644	2555943	1464000
EIC	Negative	630.3207	0.86	unknown	240470	534209	383792	307209	481959	778399	256866	210074	266925	412717	437647	303983	423329	771887	626401	486745	510278	315262	461818
EIC	Negative	344.1539	2.89	unknown	478461	4399266	715230	1243139	3325648	4747619	3986064	1270060	7337554	1944404	4671449	6404119	6871068	3538842	6620265	10649119	4130122	1255268	2263546
EIC	Negative	642.3422	2.89	unknown	548495	723583	137336	269339	475083	824780	506734	244051	1095402	296529	429637	519754	483105	505232	623588	723451	695422	300260	1455165
EIC	Negative	631.3212	0.86	unknown	148091	311571	211072	170147	279596	441406	152039	132664	147285	215883	251021	182888	254873	449670	370480	267275	299534	183273	271529
EIC	Negative	255.1604	3.91	unknown	499063	508596	377364	302859	486035	405879	388974	460162	319499	359554	410492	394630	448582	340195	488150	443641	462498	554745	540268
EIC	Negative	417.1195	1.61	unknown	523727	456680	830770	280497	295736	415428	815156	550912	550798	354466	549678	846269	673136	620022	504311	820736	762097	550136	537885
fpHILIC	Negative	230.0740	2.95	unknown	240409	330617	268134	192311	318600	432557	376027	221980	303329	301346	663401	401283	526355	302787	324031	515707	409939	281528	466145
EIC	Negative	313.1197	1.99	unknown	990483	1779875	739040	803557	1667361	1028580	1826420	729418	1306324	0	2180419	2147002	2275086	1005819	1201925	3200474	1943796	276790	1307844
fpHILIC	Negative	119.9763	0.81	unknown	460499	449318	451509	514823	539080	507679	540816	580451	541268	483951	492887	552868	547711	504628	583610	0	727902	1453905	512841
EIC	Negative	339.2366	4.38	unknown	359457	347686	251365	206312	332972	256041	332966	318912	228360	277242	355057	256309	360563	269635	336931	310776	351818	391187	389616
EIC	Negative	610.3377	1.92	unknown	0	289176	0	0	240372	269745	303354	0	253036	0	326665	359191	191338	306761	258798	307147	288042	0	439223
EIC	Negative	375.1795	2.78	unknown	1956628	3920463	1256421	1194764	3692983	3465669	4192217	2117043	3612621	3856169	4491795	4391741	4158092	3852708	4601066	3615821	4459609	3465350	4591715
EIC	Negative	455.2564	1.81	unknown	50653	396393	255903	0	337763	0	366044	232930	461037	253468	399285	405213	435816	404998	406632	428192	435629	0	427076
fpHILIC	Negative	125.9949	1.94	unknown	430069	449076	400664	623648	452120	568447	431092	378022	597563	656050	743988	656615	645274	556558	701628	666928	608961	446341	526267
EIC	Negative	373.2372	5.83	unknown	511446	547388	389633	371072	520541	467204	399564	521962	357311	355420	468478	403071	456896	370119	524498	522050	550136	598675	560393
fpHILIC	Positive	738.5325	0.34	unknown	57898	113747	89076	119080	157231	53569	99202	99622	283664	120762	183323	156269	178237	589443	94189	159343	223662	173536	192267
EIC	Negative	397.1795	0.86	unknown	85389	188081	96587	81750	303461	117840	323467	163675	398042	138292	247401	37981	149113	406617	182988	422453	204916	235519	543267
EIC	Negative	276.1970	2.86	unknown	282841	352907	175598	259948	294634	305616	264174	338559	287951	285724	355521	264771	358976	301686	402248	282233	393015	368615	367774
EIC	Negative	592.2995	4.85	unknown	441521	454430	599234	698053	646362	656510	766271	748232	765297	803543	726369	778709	859005	826214	892481	878613	853013	910683	825996
EIC	Negative	587.3596	2.78	unknown	9629680	9502912	4951276	5221207	10459740	8104980	14542450	8563795	9221536	6855018	14206900	10066965	13175524	9895793	14032741	9388798	13579329	9530429	15414803
EIC	Negative	321.0442	2.18	unknown	920046	1223314	1340488	288224	710868	732728	1399184	445417	1295039	437163	945897	1747396	1263553	1178276	1159711	1697249	1230921	419232	1076193
fpHILIC	Positive	221.0912	2.42	unknown	1093790	548066	231837	249098	833021	686982	807454	342484	603634	219603	470210	689368	580737	936566	1219364	608990	690418	539548	931185
EIC	Negative	588.3629	2.78	unknown	3852533	3693490	1863569	1973403	3867076	3058232	5457418	3219323	3490235	2543716	5398814	3867783	4873035	3734346	5322822	3508797	5061850	3588978	5801504
EIC	Negative	340.3303	6.80	unknown	516343	628944	649068	619411	670526	762728	620845	766303	556197	734735	732271	934422	625004	828667	839928	525503	832542	1085597	834986
fpHILIC	Negative	171.0413	2.73	unknown	261841	463871	267684	174704	361361	637703	316141	205119	221635	323215	551993	307910	330295	909277	617942	362339	281555	289053	574687
EIC	Negative	592.3278	2.83	unknown	311303	582789	223471	227034	476663	517125	554062	358656	473843	411336	599206	719939	385933	707371	575975	603348	559677	278080	889202
EIC	Negative	339.3269	6.80	unknown	2109256	2616782	2630257	2554710	2733306	3109760	2525741	3181319	2266433	3013215	3014039	3906675	2575094	3409960	3375467	2222616	3399566	4493645	3424995
EIC	Negative	401.2250	3.84	unknown	356999	1041212	318712	717902	835574	981180	739226	593881	951454	770463	1005488	904947	928983	932645	934657	966798	1065334	1058148	998221
EIC	Negative	614.2851	3.83	unknown	73889	169715	132190	193315	172074	188553	249313	161671	226825	173120	305410	323542	243273	278574	272062	278182	227475	186990	258237
EIC	Negative	278.1838	3.65	unknown	97906233	105484845	78874286	68893516	96290802	86280139	83697934	98361753	71414603	76518609	92432581	84570417	91431105	75923348	104359494	96920097	103071100	111113799	119250267
EIC	Negative	277.1808	3.65	unknown	528233545	568419249	421515547	372547474	515928747	459550406	447299262	528252952	381795838	414789828	497304952	450697626	483921171	406463346	553973804	518176372	556693967	595680404	636226712
fpHILIC	Negative	553.3074	0.80	unknown	170853	288405	223288	178983	248680	219172	317019	211545	222929	156018	265856	245644	227829	195387	287721	0	332085	623679	253224
EIC	Negative	338.2342	4.17	unknown	1774149	1706270	1347896	1187062	1695420	1407583	1526660	1824220	1378023	1448647	1794171	1505670	1847290	1469047	1811624	1645835	1965952	2066866	2127622
EIC	Negative	237.1132	2.07	unknown	263059	224098	111205	89084	353205	295019	315528	339038	275968	284239	318136	322430	294660	275372	529102	301230	384691	218218	505866
EIC	Negative	234.1578	3.87	unknown	144507	354326	170537	178032	306083	160599	313274	165808	263647	165583	245138	221272	340321	277509	262830	315858	243601	300059	363586
EIC	Negative	399.2156	2.26	unknown	0	0	0	0	0	0	0	0	173994	0	0	0	253781	0	0	0	213854	0	0
EIC	Negative	253.1445	3.47	unknown	152356	334147	369933	144139	344740	116439	320726	322071	324512	370345	368876	377382	334095	395640	409703	286820	369684	401530	289056
EIC	Negative	361.1878	5.18	unknown	1153654	1382006	1015100	1217077	1329497	1290194	1379250	981860	1130941	1112781	1367097	1333900	1353790	1506626	1518296	1549225	1372386	1407562	1669267
EIC	Negative	271.1557	2.99	unknown	132253	151943	110486	99492	122013	105720	131517	177171	120287	135645	159076	121641	166390	141433	162308	126728	176289	179452	163213
EIC	Negative	294.2158	4.45	unknown	165210	74933	187273	236898	166314	110037	257174	200298	279413	133903	125230	100656	127978	150772	99959	613482	155226	221907	156090
EIC	Negative	346.2104	2.18	unknown	66132	145327	44595	64413	224950	251272	332483	555239	464765	84104	191149	12346	196336	681536	286486	730669	95119	602120	540785
EIC	Negative	618.3152	5.44	unknown	308835	327544	447800	452805	480708	448332	513516	504944	525879	582933	631611	620650	571840	614067	608975	590276	609457	661845	580551
EIC	Negative	619.3470	3.53	unknown	653253	419001	56216	0	327652	610280	410895	404522	389669	480878	392685	654896	434366	253425	430976	569310	657642	301384	685745
EIC	Negative	455.2358	2.35	unknown	215086	124647	238095	297525	132474	168923	177293	378683	187290	358727	248079	203426	192888	240804	205211	202651	220220	448294	214952
EIC	Negative	237.0766	2.30	unknown	73370	272992	76756	255698	247941	280061	267042	189143	251911	347886	251287	252687	240293	243477	253617	295513	250240	438527	290837
fpHILIC	Negative	224.0327	1.04	unknown	689323	649977	636277	471437	621425	921543	611803	547282	744537	849388	750125	591550	606661	1093355	853468	958778	914640	770885	766120
fpHILIC	Negative	104.0435	1.69	unknown	545013	2150811	1472465	2195712	1215809	2591610	1427265	1162302	2020808	1818974	2315716	1848517	1761919	2070892	3400407	2347810	1182336	1887356	1604102
EIC	Negative	630.8214	0.86	unknown	160030	357505	258353	213830	310511	530962	177739	127039	170971	273579	319703	205502	277668	514821	444024	340483	329587	201606	316200
EIC	Negative	495.3696	3.31	unknown	364899	681323	395556	415919	741222	499083	524495	352834	654906	378073	930484	611335	805268	767901	927799	816939	501339	508954	681508
EIC	Negative	618.3422	3.12	unknown	523278	516186	329175	381930	510873	724427	641567	378879	486439	396139	514783	704707	547959	376485	622101	806416	804707	325892	844247
EIC	Negative	271.1556	3.11	unknown	231904	243466	166087	150714	214896	177175	208432	243344	195506	216738	262627	219505	271889	228221	276564	206233	262803	267290	278007
EIC	Negative	583.2844	4.64	unknown	439064	359346	396992	378606	388608	363735	390839	415390	481350	499564	437173	499687	481557	377621	537285	551587	514981	518651	411933
EIC	Negative	305.1747	1.93	unknown	0	404951	0	0	173827	301638	362730	0	180978	0	254510	486764	452819	265122	290303	423166	284377	0	270743
EIC	Negative	376.1826	2.78	unknown	507581	856682	194967	315391	815096	778523	878993	401239	801725	817785	1039291	889406	992783	834247	1018301	782497	958051	860088	959275
EIC	Negative	338.2342	4.38	unknown	1682544	1713359	1263928	1214200	1533798	1293891	1451199	1688794	1205303	1432569	1535649	1442642	1566702	1299458	1739758	1449427	1862182	2028260	1876662
fpHILIC	Positive	148.0602	3.66	unknown	17804072	10875805	10695426	11953979	10952586	19704150	10166576	10199833	12699750	12883453	9341679	14211427	10964727	12459789	12790812	16583051	17467652	16710475	16036450
EIC	Negative	602.3465	3.91	unknown	0	2725673	167484	197123	261774	260231	295531	1199846	3223247	883222	1522184	233563	293289	381367	1255980	2017922	1969215	1001877	303553
fpHILIC	Negative	322.1137	2.32	unknown	416608	360586	287924	223176	606352	489900	467300	283442	524596	277993	337717	523018	425483	548593	865615	706741	462970	443994	493542
EIC	Negative	287.1289	1.92	unknown	0	450297	0	0	180469	584095	482122	0	393928	0	422442	725767	771737	562949	400173	668369	506891	0	478861
EIC	Negative	438.2494	2.48	unknown	162241	120307	0	0	146991	146985	105910	152982	133687	272884	141393	130266	125589	146807	142393	135736	117795	312306	152249

EIC	Negative	424.2713	4.25	unknown	757114	699286	584707	506969	668336	591325	663295	798334	569642	612450	731065	643909	764988	660287	812636	669337	885774	900311	858334
fpHILIC	Negative	103.0401	1.68	unknown	11561584	46563808	32439944	43695680	26382714	53564352	31397194	25560862	45362272	40336460	47401992	39575096	38806200	44402772	72425280	51761516	26778844	41478344	36144556
EIC	Negative	279.0642	2.50	unknown	442312	337575	149318	224403	324498	315146	307271	380948	307221	579079	332451	307941	331347	362431	360952	344477	335496	670798	342427
EIC	Negative	424.2713	4.18	unknown	802097	933428	651504	594954	792773	732095	790815	1017853	708838	742897	920504	768517	998327	766934	965136	784877	1063298	1183911	1081659
EIC	Negative	643.3456	2.89	unknown	175779	329495	0	155046	208895	285319	181652	82258	376552	122852	64443	170533	173948	211544	250564	264947	253286	157409	500614
EIC	Negative	552.2827	2.60	unknown	417736	457392	269958	245760	404507	345972	405211	345346	398206	378852	495788	391513	498868	404763	552736	359390	547235	443633	485774
EIC	Negative	272.0965	1.61	unknown	137227	252151	0	114747	221857	273281	211052	0	147766	33524	231525	323714	224420	160140	360046	329403	202377	0	182473
fpHILIC	Positive	249.1071	3.69	unknown	465543	742105	299737	387710	498942	961698	551933	348580	414219	350298	1003898	986745	807815	761320	727688	912040	672979	441424	742417
EIC	Negative	516.2732	2.89	unknown	319030	276048	0	0	231131	401072	211157	0	281750	0	173673	257081	197559	195276	304729	269926	369784	0	473444
EIC	Negative	288.1318	1.92	unknown	0	113112	0	0	54509	125138	109614	0	93999	0	94823	166328	148216	109738	120395	143150	107274	0	112770
EIC	Negative	345.2073	2.18	unknown	281858	537555	257408	360638	985991	1163838	1304694	1979165	1739760	397340	737362	89858	793360	2578581	1168581	2772243	425597	2414282	2280914
EIC	Negative	592.2995	5.05	unknown	402012	414184	571180	654297	506119	559682	618073	730313	732479	738337	651128	783405	752253	802480	724006	784883	804107	917631	747289
EIC	Negative	477.7391	0.84	unknown	218807	507414	177671	229767	304573	772648	173833	185030	151576	166546	278862	238689	260219	275506	632564	592868	316941	263536	201680
EIC	Negative	314.1796	1.88	unknown	431847	371407	0	0	426462	540186	426912	0	316160	0	468377	440313	544303	192884	659835	608606	579809	0	314881
EIC	Negative	279.1865	3.65	unknown	11712725	12456188	9458281	8352586	11535332	10210918	9709312	11879803	8579323	9441220	10902669	10119145	11023189	8997899	12570797	11575483	13264737	14190221	14134728
EIC	Negative	628.3532	4.78	unknown	0	11944372	0	0	0	0	0	0	12406813	6520055	0	0	7807093	0	7152326	0	0	0	8567123
fpHILIC	Negative	124.9914	1.93	unknown	18408396	19497436	18159146	29049690	19651032	25553762	20668342	17615270	25793236	30763636	32927038	29634750	29221876	25775922	34812348	30719384	26374882	21625344	24966936
EIC	Negative	457.1821	2.78	unknown	103863	464940	0	0	447403	401916	452842	183956	449011	379519	542413	454953	514022	456150	577448	424624	516461	395551	539201
EIC	Negative	338.2342	4.07	unknown	1673921	1773899	1303038	1009037	1659277	1458113	1495890	1758110	1244520	1355120	1725002	1449662	1743397	1530786	2025148	1515465	1882219	1935451	2297070
fpHILIC	Positive	169.0686	0.96	unknown	221161	235454	175237	208728	353819	362694	320949	191685	295031	196353	379319	377961	243865	314133	413132	302756	275554	254948	688271
EIC	Negative	309.1710	2.94	unknown	547497	789884	534542	445385	827874	765558	728930	614624	568201	511262	919713	831167	908783	809693	925126	817276	1026281	632480	1021681
EIC	Negative	322.0472	2.18	unknown	135945	213264	209088	43233	127022	126531	247537	63043	230757	61152	169265	315120	227475	199384	200614	281428	225363	90806	211283
fpHILIC	Negative	121.0296	0.90	unknown	2091073	1229347	1644048	1428918	1920246	2173081	1098374	1797903	1909938	1807238	984873	1812408	1941381	1615267	1646611	3298500	2276541	2288055	1807964
fpHILIC	Negative	126.9873	1.93	unknown	825333	856524	776099	1294434	877932	1181807	1004673	780336	1121020	1373258	1599666	1391419	1385821	1202256	1647942	1434094	1179172	984132	1074532
EIC	Negative	398.2266	4.23	unknown	204428	207998	140952	155235	191888	154188	167038	205890	187921	161108	209591	184116	230696	162537	205452	173278	277076	275771	223218
EIC	Negative	608.4777	6.44	unknown	13574221	21815452	306710	16271358	14262255	10756504	15440462	12551470	19914846	7136556	20992352	12595702	15599622	15665798	15104970	23392012	22960728	10310151	21139480
EIC	Negative	437.2459	2.48	unknown	371798	380825	150414	128997	411297	372866	313749	539267	414809	793620	421435	415018	411951	450426	425403	419388	418400	918556	433010
EIC	Negative	364.1840	1.92	unknown	0	117282	0	0	65277	153157	157596	0	109276	0	162573	268615	262840	180052	175199	147378	173431	0	122875
EIC	Negative	478.2413	0.84	unknown	172084	288254	98767	139568	174002	397882	96424	121255	114043	128812	192974	135948	147727	141988	334604	308890	186082	214164	110036
EIC	Negative	556.3526	3.65	unknown	762086	0	527688	0	942385	799711	0	0	527318	576096	742084	0	0	552533	0	941188	951055	1006033	0
EIC	Negative	435.3486	6.56	unknown	711229	347450	416374	651503	504785	534155	474295	588395	928428	722124	1086420	572474	741741	647643	796641	810903	558764	901571	821761
fpHILIC	Negative	121.0409	0.92	unknown	198759	271286	44030	168766	377610	438498	332655	114233	537626	143878	373377	467368	375627	300968	477858	611807	454437	292821	476604
EIC	Negative	311.2231	2.49	unknown	168964	71618	139391	81119	83838	138052	157707	104209	144286	81951	72481	89623	88362	97654	73872	366481	150986	116731	100696
EIC	Negative	420.2511	2.72	unknown	561122	1091462	714051	1264413	1184626	1093517	1034766	1096012	1136021	885234	1102545	1297830	1317373	1368989	1342726	1419996	1482254	1344895	1559084
EIC	Negative	434.2664	2.73	unknown	825144	245246096	798153	266955520	264950832	252985152	250338016	1605137	241320672	778238	244475888	251994128	321506528	298873184	318576768	317784640	326280800	0	344295744
EIC	Negative	463.3072	2.14	unknown	86565	17991	119430	90324	93867	27881	12375	494597	87180	227557	8890	31537	26766	62407	59554	41984	42758	732295	48980
EIC	Negative	605.4810	6.84	unknown	209056	146587	145737	131531	0	191402	158218	250287	284957	251763	195647	203221	279043	233389	233244	212597	228512	261100	208597
EIC	Negative	431.1394	3.80	unknown	172298	186651	154379	185077	196644	247544	171259	230124	162610	136266	145176	210072	236699	259854	209746	277683	277646	275016	263634
fpHILIC	Negative	401.1116	2.69	unknown	178295	597216	479194	256121	353076	419706	719025	283680	257431	472460	635628	348272	463595	503669	725470	443703	568020	396940	496820
EIC	Negative	282.1717	2.50	unknown	346885	400874	201788	219956	334032	302487	334308	404714	292363	394127	394755	343274	356282	384901	378864	338301	436129	538304	467340
EIC	Negative	402.2300	3.84	unknown	191106	334130	110303	178116	251643	264118	253430	176582	358058	247071	367595	333819	289329	319703	345112	373676	345007	321021	304444
fpHILIC	Negative	247.0933	3.69	unknown	325552	451586	223184	231640	358375	549171	307396	210969	221506	197022	513140	579214	471021	465284	434061	551520	407317	293622	449712
fpHILIC	Negative	346.1615	2.97	unknown	804488	783926	1046203	510368	1627695	1545341	1251341	1203010	373900	710328	1089116	1298676	1141777	991045	1132876	1481778	1430063	1529937	2217343
EIC	Negative	229.1809	2.65	unknown	156757	185035	120581	143806	199244	160911	185849	157708	182275	152327	180465	186847	215945	148022	151784	399415	209638	210662	179785
EIC	Negative	581.2969	1.98	unknown	0	119250	0	0	134728	167616	180259	0	381638	0	229416	207080	317485	448378	374229	246085	294368	0	208599
EIC	Negative	298.1664	2.15	unknown	187379	190905	158202	109145	228597	316092	194329	163869	170576	145196	425906	275909	375750	411273	408063	329960	340148	107024	210203
fpHILIC	Positive	282.1183	3.18	unknown	1243799	1772028	1142150	1032556	1027641	1655261	1971803	813001	998982	1155234	1933389	1488544	2051314	1318084	1933264	1776593	1742432	1276038	1688025
EIC	Negative	328.8034	0.65	unknown	56248	49518	76711	90102	83467	99248	100686	95067	145611	160724	121381	121463	141442	62715	111077	153325	118035	144368	155680
EIC	Negative	370.1593	1.87	unknown	73602	25343	0	0	59816	77755	79508	0	0	0	86771	68175	62255	28821	80817	91803	69771	0	40439
EIC	Negative	643.2896	5.24	unknown	386160	2827434	898725	1352965	1722589	2232255	1749167	833892	2520390	978620	2571319	2760209	3123715	3761567	1982689	3047413	2414271	1144325	3217538
EIC	Negative	545.4206	6.56	unknown	459558	177823	221915	278909	315265	332975	304287	398725	407964	393592	433001	349369	517938	327657	545512	351908	313926	577484	504376
EIC	Negative	339.2366	4.17	unknown	351692	362883	255118	238860	329176	301194	285037	349130	268481	293744	378829	295849	363093	278431	380093	349015	407209	409495	452874
fpHILIC	Positive	756.5594	0.34	unknown	96980	253374	161492	261478	375225	76020	232135	169577	591260	293641	438274	340532	372163	1409586	183850	450001	471974	350203	427922
EIC	Negative	270.8424	0.65	unknown	65937	49590	81517	111584	100490	113255	127823	114293	165267	182425	148277	138955	165910	96618	137081	177381	137832	168847	189664
EIC	Negative	272.8412	0.65	unknown	67382	52297	75244	94043	95929	115623	117540	108847	160930	177924	138625	139819	166183	88360	139704	161934	140256	159915	180310
fpHILIC	Negative	199.0378	2.46	unknown	5966402	9816911	6312911	3575225	9049005	8603791	10398423	5669551	9688413	6843564	14644081	14751145	10485161	12592997	14201818	9008169	9196578	8713278	15580312
EIC	Negative	330.8014	0.64	unknown	73270	62673	91144	118793	109446	130722	132099	122756	185749	216917	164119	160903	189930	82408	147260	193257	155531	189268	204861
fpHILIC	Positive	280.1377	2.54	unknown	299874	407460	404972	203191	261606	419290	599202	233796	259334	351278	424428	350274	357801	470119	821444	355482	372935	329066	440761
EIC	Negative	597.3623	3.57	unknown	227145	487348	85869	354988	230715	621086	323780	429738	387923	609760	406519	757781	433298	261700	498683	675350	633012	80278	720416
EIC	Negative	306.1717	2.49	unknown	326113	280263	209761	167277	253223	218523	236898	328768	235885	341447	311865	240952	315427	248484	296050	257062	310379	482645	321898
EIC	Negative	339.2368	4.07	unknown	341826	375239	241645	184631	350285	277217	276732	351733	234115	286797	353823	279252	350788	274349	399581	300179	393963	404620	455578
fpHILIC	Negative	240.0853	1.63	unknown	0	167608	1156957	621268	915047	157604	0	943072	1554561	1023255	1154694	236806	1295454	1077941	1206738	181736	1171571	1093815	1038611
fpHILIC	Positive	755.5597	0.34	unknown	264365	481826	382197	492462	725767	131110	494943	380469	1133971	600392	785040	695191	665283	2851161	375208	812727	1071141	735041	773406
fpHILIC	Positive	149.0638	3.66	unknown	938331	591737	511988	644694	639138	1112471	498017	582655	700368	636585	439988	808106	597602	665179	763482	950041	1016048	1005357	909171
EIC	Negative	539.2317	1.39	unknown	0	69227	0	0	106743	84027	150177	0	135823	0	83399	128153	94613	86142	193719	172378	124361	0	136574
EIC	Negative	229.1442	3.18	unknown	217068	358640	179996	155821	342387	270522	194174	213846	166153	179501	219721	224967	230486	174332	237314	377271	233240	338284	377677
EIC	Negative	239.0332	0.58	unknown	69751	169471	188611	214883	219542	173124	301262	207916	156012	205900	185212	136494	137032	245856	290564	174310	185439	224062	520947
EIC	Negative	441.2033	6.54	unknown	1442083	986372	1291658	1558134	1916465	1592201	1334170	1227553	1290743	3535474	2096435	1250250	1805614	1942390	2644843	2089898	1827388	1367045	1783327
EIC	Negative	290.1483	1.88	unknown	167815	103662	0	0	62282	108605	123039	0	85028	0	127653	126548	150432	116717	77214	168533	139055	0	116979
EIC	Negative	313.2387	2.99	unknown	240889	65497	220659	187771	152845	305375	231334	198127	293724	182664	165708	310806	255944	214321	128383	702440	254223	248741	232966
EIC	Negative	473.2248	2.72	unknown	277856	404066	136539	727709	715507	902320	928644	629893	876452	855460	836861	846914	1048054	926693	1040272	1185557	968377	1065507	1117134
EIC	Negative	243.1604	2.49	unknown	274590	248412	167303	174245	222399	273867	180829	253527	187308	319503	292627	242062	312547	158089	271283	554958	255931	195058	209536
EIC	Negative	463.3074	3.10	unknown	177211	31940	168247	99323	102868	224849	41768	1258860	111044	1076149	112283	90040	97714	125012	104550	67009	123059	1969228	49897
EIC	Negative	411.1596	3.94	unknown	282741	212161	507419	301419	172788	578488	387341	317246	268142	245373	460602	706587	760886	539984	207851	538174	516225	690882	345283
fpHILIC	Negative	201.0351	2.46	unknown	1900800	3140813	2110725	1237201	2830424	2601917	3625484	1758286	3097879	2147988	4956303	4863275	3362471	3864163	4758683	2995400	3018519	2866393	4914210
fpHILIC	Positive	146.0809	1.69	unknown	62921	450180	593465	844422	724571	1228182	706429	519394	620954	751666	886860	961051	654540	583272	1375637	1346687	630656	879805	836225
EIC	Negative	283.2549	6.56	unknown	2919671	2991409	3831099	4152367	3967177	3164462	4238868	4423962	5070838	3594800	6407980	5108640	7940929	5064992	5324642	4394034	5112326	7739979	5340263
fpHILIC	Negative	116.0505	2.61	unknown	360766	506037	403181	260923	330486	424088	478988	349289	325286	375425	513150	457094	373680	555148	719177	452387	384412	465782	533741
EIC	Negative	388.7614	0.65	unknown	62149	44696	67361	85741	69144	88134	90159	78916	129630	143735	106462	107374	128655	47529	99140	135343	104140	132967	140044

fpHILIC	Positive	126.1026	3.89	unknown	3181328	2232678	1097932	4052905	2122772	4190363	1193658	3492738	4174806	1923593	2136961	3749901	5651532	4938377	2271021	3838248	6843486	4742020	2689885
EIC	Negative	635.3386	2.65	unknown	0	93831	0	0	69675	79203	122050	0	40852	0	78177	94354	75042	68700	88818	90303	101535	0	138515
EIC	Negative	642.3421	3.39	unknown	708570	851698	435626	450524	480600	896707	538491	476106	938773	662392	771747	934108	727230	431991	681911	1162592	1031413	350225	1252446
EIC	Negative	320.2261	4.30	unknown	0	0	113730	106697	163980	0	233033	0	25720	131419	291145	367067	29647	0	0	0	55284	513674	0
fpHILIC	Negative	240.0628	2.61	unknown	536346	871897	687345	338833	598052	661421	796078	535823	475235	622663	908551	720884	717638	957541	1291234	728201	760152	668749	680872
EIC	Negative	284.2588	6.56	unknown	236128	236819	321772	351913	335126	265265	369463	330485	483468	278656	555864	437785	699079	465007	486711	356994	487185	615895	456767
EIC	Negative	282.2520	6.56	unknown	24968769	26475446	32478072	35683096	35299490	27647978	37842875	38711124	45527017	32038612	56869405	44508145	70343444	44006256	48056773	38211224	46622550	69353886	47330978
EIC	Negative	293.1762	6.03	unknown	0	0	0	0	0	0	8526442	4558476	6699389	0	0	6672143	4466310	3904507	3114212	5844810	17743115	0	0
EIC	Negative	362.2373	4.96	unknown	0	0	0	0	0	0	102294	182929	0	0	121034	101870	97345	0	0	0	0	256627	128953
fpHILIC	Negative	737.5231	0.35	unknown	445161	1184838	1073375	1307866	1660180	339177	1267487	874562	2310669	1225706	1741414	1641852	1600879	6281102	1267639	1978308	2227817	1636229	1623052
fpHILIC	Negative	163.0247	2.90	unknown	1057741	813850	527014	584635	769504	863703	979767	552282	1111216	453111	472528	835426	795098	1102606	1202140	1160951	1279501	893903	1276465
EIC	Negative	534.7828	1.29	unknown	81633	268323	0	0	204076	307391	107682	0	98993	125720	190886	214254	213840	176036	362527	219157	282909	0	113504
EIC	Negative	307.0648	1.95	unknown	3418020	14621570	3209874	522052	12134324	10537693	14707507	999324	5502674	2978548	14715839	16073362	16242425	14746399	22437186	12641999	12379693	675007	11635288
EIC	Negative	565.5146	6.55	unknown	621759	550419	791775	1179338	1522087	592049	1267213	1175259	1038768	782096	2395920	1104882	3604448	986842	1467806	985165	1604053	2718631	853565
EIC	Negative	363.1810	1.92	unknown	0	537659	0	0	216094	727626	652685	0	516011	0	702263	1143333	1159330	843470	791970	702443	778141	0	608777
EIC	Negative	436.2718	2.72	unknown	5755334	9685663	5555507	10599610	10949602	10468911	10079299	7724508	9473294	7865903	9489212	10242341	12637502	12583882	12786648	13528707	13771651	12826952	13623886
EIC	Negative	509.3497	3.04	unknown	97857	120351	97994	67600	194983	152872	218874	196802	202092	144280	199215	66416	127242	156558	271810	352301	243387	177949	274158
fpHILIC	Negative	119.0542	3.17	unknown	1462325	2199777	1472834	1230312	1484934	1950358	2036709	1593453	1708227	1742277	2541093	2717900	2522635	2964572	3183688	2317384	1887707	1836782	2768402
EIC	Negative	624.3314	3.79	unknown	376601	1869984	359948	691533	1034020	1530503	1099516	453644	1128878	763665	2162221	1458499	1828809	1454156	1553186	1815400	1673993	539103	1605569
EIC	Negative	308.0680	1.95	unknown	580892	2491218	381483	0	2134568	1800175	2514732	0	946846	0	2547298	2788167	2788537	2571784	3829535	2168989	2170448	0	1987158
EIC	Negative	642.3422	2.68	unknown	1242532	1478269	777652	893328	908478	1676606	1075365	844919	1875247	1327881	1504699	1762483	1313591	717469	1331417	2312538	2033705	618550	2485573
EIC	Negative	625.3343	3.79	unknown	135233	708962	135852	250613	360373	578287	389227	173979	412255	269917	769635	544721	668690	502674	578938	649127	606789	222104	578288
EIC	Negative	608.3592	5.27	unknown	360593	266709	129216	220090	280326	251065	348434	285841	443463	174132	193001	296121	401302	379849	428834	413795	498430	317450	557794
EIC	Negative	305.1432	3.83	unknown	413567	26774	489523	729502	434480	343331	590636	35191	573293	533200	522228	611524	782166	328842	592076	754915	457071	624949	294040
EIC	Negative	619.3462	2.71	unknown	932457	769139	517445	568461	459784	1099061	782405	681851	839907	1109306	811258	1324093	926168	478499	889294	1177325	1327380	588923	1360793
fpHILIC	Negative	530.3031	0.59	unknown	389437	723065	125106	195247	472245	417950	378763	408141	539921	0	516649	494836	582206	567915	295921	542177	343242	1462684	426898
EIC	Negative	412.1849	2.18	unknown	45402	112099	44769	191095	55072	81117	99728	100135	140544	0	61567	152809	65382	11419	50059	130218	65692	233088	181300
fpHILIC	Negative	216.0308	0.94	unknown	89795	42244	0	39398	1564380	2596727	1587934	789690	687055	0	0	92710	57383	106558	3133112	1832484	1664996	1516971	1869455
EIC	Negative	435.2692	2.72	unknown	35600416	59501464	33881568	64543632	66163013	62962940	61079633	46925835	56553559	48977085	56967452	63249067	75836000	75164021	78894481	82380067	85026665	77287807	83719727
EIC	Negative	434.2662	2.72	unknown	133715964	224162548	127945177	244092204	250169495	238566553	230604004	177227416	214337834	183736556	216561506	236283591	286733634	286587986	299545211	311000377	323583378	293434789	314379058
EIC	Negative	358.2161	2.72	unknown	198197	202312	89250	259785	266147	262688	297048	197832	245468	203753	231851	242613	370382	372191	320716	328655	425330	326541	361518
EIC	Negative	642.3422	2.79	unknown	821719	2025509	634904	735636	1104360	1999628	1157952	677493	2132293	1449274	1677282	2124173	1446220	874306	1541780	2469255	2393449	710895	2710783
EIC	Negative	376.2513	2.72	unknown	2190708	3684685	2001982	4134713	4092047	3895656	4008643	2877501	3626578	2950598	3598888	3837603	5007510	4679871	4894725	5111537	5452019	5050020	5199671
fpHILIC	Negative	200.0414	2.46	unknown	396955	679818	463872	255675	566603	589217	726332	372542	659535	480558	964909	1014001	697988	828602	1035204	691642	665693	620703	1038129
EIC	Negative	375.2481	2.72	unknown	15659271	26260095	14552206	29167356	30101434	27879104	27243394	21332206	25529812	21564809	25605075	27822525	34761403	33843518	35768642	37419513	38771519	35266262	36955342
fpHILIC	Negative	488.1621	3.66	unknown	67524	203720	246555	40237	44946	66818	262023	83274	584093	0	0	88930	40058	305060	53947	51575	98088	135353	1113400
EIC	Negative	618.3422	2.63	unknown	450806	519379	297149	321348	416844	880980	556146	349575	522239	467315	557837	861780	563215	323210	675151	974246	960289	225079	967589
fpHILIC	Negative	136.0331	2.83	unknown	1006636	800263	555984	584412	726043	1075012	630414	654040	747272	719588	697014	753641	698858	1590074	1119702	1141869	1045826	869322	1174477
EIC	Negative	505.3170	2.62	unknown	0	151099	0	171736	119794	162244	113428	0	75319	164044	123791	173731	157565	300512	162359	146074	143514	172152	33446
EIC	Negative	643.3459	3.06	unknown	451825	577608	322331	360765	407070	574738	463929	275350	631347	440206	567175	669438	505557	299494	544831	798489	801951	256050	885240
EIC	Negative	608.3191	3.87	unknown	619626	1808765	531143	621008	1243429	1676052	1459834	724891	1523119	1109235	2025057	2086790	2017134	2321198	2133870	1922890	1815439	1561695	1835565
EIC	Negative	271.1096	1.46	unknown	0	64914	0	0	86241	93111	95658	0	107967	0	78385	85703	100841	88635	96648	238219	106720	0	95191
EIC	Negative	539.2318	1.91	unknown	0	125432	0	0	116010	125236	126460	0	115758	0	127998	127598	94231	90011	164704	193491	179811	0	141044
EIC	Negative	491.3382	2.67	unknown	117236	105377	51349	36981	96681	120009	109976	83173	60497	250708	155718	155462	102982	504365	177234	112088	133221	117950	120241
EIC	Negative	384.1854	1.87	unknown	76290	207117	0	0	49023	115884	177767	0	217955	0	233360	84661	242471	228852	196383	237649	153715	0	200214
EIC	Negative	390.0709	1.95	unknown	0	120682	0	0	107475	81078	120991	0	40160	0	120465	131547	138752	124038	195150	99589	92054	0	104831
EIC	Negative	374.2452	2.72	unknown	64973897	110174015	60818535	120982160	122963251	116903392	115204140	87490531	105882244	91101432	106582625	117042910	144144242	143566576	150858238	156370851	162354174	145076560	156981388
EIC	Negative	481.3329	6.52	unknown	369862	661846	420028	714573	1370275	877741	1841059	804119	1166854	446321	1152141	767031	846162	1262741	1813031	1385230	1385452	497382	3448718
EIC	Negative	614.5445	6.54	unknown	3533906	2133393	1466118	4248585	2628706	2462858	3149728	6083157	4291940	4480181	4321853	4061381	7262665	6834624	4369404	5013032	6609662	4332521	5889956
fpHILIC	Negative	147.0300	3.78	unknown	1591656	1782585	1418957	1993767	1491707	2696638	1863466	1467242	2589457	1848209	2235516	1518724	3138374	1846248	2684523	3547072	2705168	3316270	2007564
fpHILIC	Negative	736.5190	0.35	unknown	1120886	2352482	1749502	2688496	3017415	709081	2319589	1791027	4681106	2567263	3659788	3086426	2992909	12843426	2402781	4009632	4653066	3319261	3279737
EIC	Negative	375.1585	3.65	unknown	601727	715651	627034	816647	1065389	1161742	1174693	1419332	1177271	1209679	1384425	1425776	1463358	1319690	1639212	1578276	1579062	1808939	1863561
EIC	Negative	312.2264	3.52	unknown	101682	66310	89120	113905	84760	95755	96489	99325	130378	71738	59485	83517	69939	68718	55184	428094	78437	84506	70152
EIC	Negative	619.3467	2.58	unknown	736637	637437	174980	279884	410656	994363	550423	430087	638338	618902	629333	1127413	696678	312602	715875	1016138	1125421	306736	1119247
EIC	Negative	415.1975	2.44	unknown	0	0	0	0	0	248510	0	0	0	0	0	0	588536	1057743	352348	0	0	0	0
EIC	Negative	512.3675	2.14	unknown	0	307008	166969	213929	648606	161476	386563	228175	245691	423289	325419	153603	212932	625424	437528	396220	386614	713248	435218
EIC	Negative	469.1632	1.93	unknown	0	221262	0	0	203618	140237	244292	0	185597	0	282585	272800	337010	145352	176225	344004	407155	0	169816
EIC	Negative	310.0652	1.95	unknown	0	124534	0	0	106669	87925	119344	0	40693	0	132302	150706	134115	134362	182631	109613	107431	0	103319
EIC	Negative	410.9653	0.55	unknown	81766	25407	0	71452	61742	72429	81876	0	23001	42473	89733	51136	20897	77217	201162	69659	28945	0	40327
fpHILIC	Positive	411.1913	2.62	unknown	333921	0	313460	127319	274764	274106	373486	202575	221529	226952	441016	234391	263332	416221	572889	394472	323971	294766	333067
EIC	Negative	492.2742	2.77	unknown	86469	244374	81736	0	154987	252894	197510	0	105529	169097	206743	166447	119769	613414	194565	54019	190873	226978	346880
EIC	Negative	596.3579	2.37	unknown	320709	0	0	15082	0	225030	0	125632	151721	83984	8275	265061	38695	112512	167479	232467	221214	0	95635
EIC	Negative	618.3422	2.58	unknown	2143517	1974701	872639	979821	1255564	2930706	1628815	1244735	1922208	1903725	1807677	3432817	2121938	947800	2154470	3038290	3350689	948094	3345185
fpHILIC	Positive	157.0969	2.44	unknown	886013	840674	732514	803970	741781	796739	988306	875817	1034652	926176	788457	928969	961019	824862	1153515	1001038	1659523	1350828	1183187
EIC	Negative	609.3221	3.87	unknown	196136	512019	110852	158632	355281	474419	420095	195079	441070	326015	572804	623167	590193	643515	603125	547338	541423	438553	569348
EIC	Negative	594.3424	2.73	unknown	302530	984263	208094	416244	747594	810077	987575	104193	784341	724531	1321469	1052590	803228	1380949	969751	959392	1019105	560826	1414111
fpHILIC	Positive	295.2266	0.38	unknown	406171	345431	496204	518930	359677	527546	545925	373211	567406	297826	331859	351302	352058	387788	209449	1862437	384761	543897	408788
fpHILIC	Negative	165.0322	0.92	unknown	0	4507062	0	0	3445214	2683123	42503	0	0	0	0	1094268	58253	0	999236	3722038	32553	1700705	2408620
EIC	Negative	285.2073	2.75	unknown	188437	148802	84676	417350	169129	298190	190834	113607	294519	588918	239431	289220	277892	355605	164272	282088	295052	501173	289174
EIC	Negative	564.5083	6.55	unknown	2932604	2731898	3771203	4385459	4097301	2638607	4451097	3964719	5139244	3337385	7930933	5261647	10556093	4991269	4705635	4914439	6078668	9363459	4088618
EIC	Negative	314.1230	1.99	unknown	243184	326176	0	176065	301710	205386	356619	0	262977	0	382393	425446	416735	168268	207096	615333	370443	188789	237571
EIC	Negative	391.1518	2.78	unknown	225538	518824	0	185396	889228	845254	1228315	622332	1041298	862524	1395432	1204380	1361224	1118413	1514044	1158624	1409361	1090802	1489495
fpHILIC	Positive	131.0812	2.79	unknown	159129	553531	453954	379656	357433	515970	512355	351143	392205	483378	457476	669923	524905	726022	601395	599882	596440	619059	649655
EIC	Negative	412.2942	5.65	unknown	0	153439	0	0	337222	383151	315122	0	117005	0	0	0	0	0	829685	114346	221303	37226	458225
EIC	Negative	309.0627	1.95	unknown	328578	782529	0	0	646766	550735	771292	0	288484	0	818997	904796	885603	798113	1261452	685336	672581	0	636015
fpHILIC	Negative	135.0299	2.83	unknown	19969358	17290748	12221747	12862883	16354940	23700312	13392734	14225133	16112272	16631914	16775242	15806435	15811929	33285818	26951824	24512204	23314490	20136076	26076224
EIC	Negative	346.2138	2.18	unknown	0	133401	0	0	0	233693	300276	0	404093	0	167451	0	182685	591586	244647	638032	0	0	475554
EIC	Negative	511.3642	2.14	unknown	368181	988079	348215	632146	1964512	487271	1209905	808974	787954	1220152	1040223	459739	702504	1883460	1367265	1221110	1234229	2427645	1371191
EIC	Negative	574.2558	4.68	unknown	499324	390863	553165	614861	526791	627054	502018	689513	541938	721313	729671	760013	668258	919353	856900	536390	837640	1043154	921124

EIC	Negative	251.1268	2.54	unknown	0	0	0	74675	632486	0	0	0	40522	0	481837	442611	0	0	0	495953	46623	0	435735
EIC	Negative	466.2442	2.44	unknown	0	355505	0	0	313590	267327	288396	302328	303755	422018	427034	324909	430028	328181	429940	320893	419067	555493	426644
EIC	Negative	381.2291	0.86	unknown	47018	59486	75295	72595	170924	196760	146496	265630	71038	75010	110209	95763	45130	79198	289499	194951	187132	380015	189513
EIC	Negative	500.2791	3.42	unknown	328369	1001409	2059447	0	1206879	0	0	2779900	0	0	0	1940231	2738091	30837	1297972	1814305	2716995	0	0
EIC	Negative	292.1566	2.36	unknown	42802	101194	0	135253	71906	122093	73461	71492	53259	147863	114812	122994	147838	78777	126856	129941	129417	103757	83745
EIC	Negative	618.3422	2.48	unknown	300397	339405	102638	154585	246633	575865	294449	341987	339939	466747	348548	601132	361524	243750	437902	623077	628145	293318	650203
EIC	Negative	322.9961	1.19	unknown	0	64653	81189	105072	6710	0	154275	143689	166091	0	181302	169349	184113	16841	184556	180164	186761	138882	0
EIC	Negative	285.0723	0.98	unknown	21982	0	10133	38987	35446	28538	54872	64391	53257	41056	16364	53853	54952	38214	77063	64704	100711	51803	53101
EIC	Negative	376.1604	3.65	unknown	106081	97908	310180	143632	233250	236793	176431	271161	267099	149996	297252	239871	336444	252483	373771	332212	351328	305685	377328
EIC	Negative	431.3176	3.86	unknown	141416	218465	143287	123749	313885	360527	337963	424632	278572	310568	394501	297259	215653	213683	565335	652292	293926	692233	317148
EIC	Negative	237.1085	2.07	unknown	0	0	0	0	0	0	0	309850	0	0	0	0	0	0	455754	0	0	0	0
EIC	Negative	487.2319	1.87	unknown	172398	722088	0	0	215197	331103	556769	0	803052	0	836465	297277	850160	887617	723394	832041	568517	0	687368
EIC	Negative	434.2662	2.88	unknown	1779740	3545382	2113371	3686670	4516726	3982953	3311969	3094940	2939446	2041317	2811776	2933678	4431483	4663929	4786771	5570035	5935589	4640186	5414900
EIC	Negative	375.2167	4.74	unknown	42847	38840	45831	53597	61778	37478	61121	77472	52935	55656	0	38115	0	28738	27432	275821	40851	47109	39397
EIC	Negative	327.1636	4.22	unknown	0	0	0	0	252561	261856	79961	0	206527	258981	0	0	0	0	0	133143	351345	407417	298318
EIC	Negative	374.2452	2.88	unknown	846529	1645777	1014240	1722905	2133880	1881370	1557963	1455449	1359888	974848	1341879	1387424	2126060	2199655	2322409	2707549	2790590	2081929	2566484
fpHILIC	Positive	155.0789	4.84	unknown	320950	337349	272935	203617	258495	260367	359467	211003	342201	380318	281059	295921	354328	360841	574273	336623	531249	314301	379543
EIC	Negative	389.0681	1.95	unknown	0	636466	0	0	495700	417753	572657	0	235212	0	657039	693998	707899	634456	968088	530622	524609	0	546796
EIC	Negative	618.3421	2.85	unknown	426402	674662	318774	245058	571117	959579	664409	347823	677786	490539	681918	990897	642493	497991	900339	1051243	1116309	364515	1378561
EIC	Negative	349.0388	1.99	unknown	0	253789	243047	0	115529	500708	233702	0	121786	0	773146	319338	269211	184094	69545	299966	682632	0	401672
EIC	Negative	363.2096	3.91	unknown	0	0	0	0	969106	0	1751989	0	1033025	0	0	1063242	0	138632	0	0	0	4781601	840111
fpHILIC	Negative	280.1039	3.18	unknown	637391	1257517	752475	594822	872196	1200154	1215668	585537	622864	672250	1469753	1394827	1474871	1107393	1826859	1254648	1245210	956047	1459888
EIC	Negative	436.2719	2.87	unknown	75747	155318	132007	153830	183168	178858	142862	120378	121621	93380	119304	136552	191352	193835	190410	237016	264224	200328	229347
EIC	Negative	304.1591	1.88	unknown	193123	123888	0	0	85469	152077	113218	0	63351	0	135842	207438	134495	249908	153395	164327	136010	0	168541
EIC	Negative	407.2347	1.87	unknown	515051	2032393	0	0	470965	1036682	1499448	0	2167898	0	2711502	835964	2405136	2481069	1966574	2570727	1418296	0	1822917
EIC	Negative	331.1913	1.47	unknown	0	441337	0	202207	368763	192098	434843	0	423902	0	326059	426172	476160	587270	571518	708806	431720	0	421813
EIC	Negative	472.2214	2.72	unknown	1046785	1626475	1272307	2592332	2705769	2887287	3225798	2412125	3241916	3058212	3540543	3570873	4196787	4100441	4386137	4351435	4424499	4350746	4276423
EIC	Negative	510.2909	6.36	unknown	936773	1213600	1449595	1253817	1325456	1322730	1488951	1822506	1110992	1334311	1725455	1715441	1416574	3045515	2801045	1213333	1824272	2700941	2120872
EIC	Negative	435.2692	2.88	unknown	475825	899973	579073	985529	1169715	1039321	908076	774303	744480	574928	744423	822623	1187070	1180923	1272190	1501797	1573279	1182119	1464106
EIC	Negative	395.1842	5.66	unknown	0	1064905	0	1109095	954418	76728	1066162	0	1068942	1249230	1248283	1161712	1478274	1021540	0	1527502	1553923	0	1695144
EIC	Negative	241.0506	1.40	unknown	0	0	0	0	0	0	128309	0	101985	0	5944	4100	0	0	50136	88783	114817	0	94319
EIC	Negative	294.1821	3.30	unknown	0	0	0	0	726980	0	0	0	0	0	0	0	0	0	0	0	450306	649114	0
EIC	Negative	289.1812	5.08	unknown	448503	481946	533586	446418	746562	378887	646975	602880	692999	348957	620256	479668	887008	408323	854671	1008161	1214625	617625	958073
fpHILIC	Negative	233.0570	0.96	unknown	436994	193139	67482	76660	324168	141253	322605	87074	366849	110504	129903	292553	218773	476071	663572	218349	322149	221763	459673
EIC	Negative	406.2318	1.87	unknown	3507397	12097781	0	575203	2859547	5145967	8991646	539064	13102389	868451	14147563	4286540	13999305	15194804	11714465	15307017	8683955	0	10867956
EIC	Negative	330.2368	2.01	unknown	409030	196370	204622	0	209526	663319	424295	137098	299127	0	176037	285281	239623	167360	151629	1920624	304300	0	258407
EIC	Negative	383.1813	1.87	unknown	248144	846895	0	0	297607	405696	694341	0	947343	0	1006608	424613	1023842	1102546	893924	1109077	647186	0	919865
fpHILIC	Negative	264.9424	4.61	unknown	655704	269548	59967	151824	420532	703901	233957	212434	475207	107468	105828	530442	371801	543451	343057	948255	657506	531187	635355
fpHILIC	Negative	197.0314	1.73	unknown	0	584761	657704	930659	916556	0	954179	911492	1088989	886836	584848	748333	742004	983776	971392	851923	1341664	1289013	1446119
fpHILIC	Negative	144.0456	0.57	unknown	7385458	2136413	1275941	1610257	3744880	3687505	4756926	2991814	3803665	1259104	2298237	5112258	3091370	6517663	6332166	4165937	6624647	5402616	6447134
EIC	Negative	463.1444	1.87	unknown	0	208800	0	0	42562	100437	164621	0	206344	0	270216	94144	281287	290222	214937	233313	174647	0	162422
EIC	Negative	642.3422	2.59	unknown	881901	1442099	353087	604513	766024	1649651	776293	472540	1601464	984176	1349147	1766897	1276639	637693	1201366	2239635	1959519	440000	2230326
EIC	Negative	382.1876	1.87	unknown	176083	623579	0	0	180435	282717	461516	0	640034	0	649789	335594	722156	817108	610644	747704	455932	0	593944
EIC	Negative	381.1842	1.87	unknown	655419	2568102	0	0	706721	1224863	2016770	0	2810822	0	2938518	1099278	3050475	3367008	2694181	3238515	1941405	0	2537691
EIC	Negative	408.2476	1.51	unknown	0	420780	0	375781	210949	313618	289393	0	289631	0	585479	206382	487341	553724	268978	565937	185185	345921	336193
fpHILIC	Positive	228.1958	0.49	unknown	1101888	876240	1134405	1335718	891391	832901	1105003	923080	1121317	1565922	629143	1686555	2625708	529581	1326362	1125521	1938113	1894444	1275171
EIC	Negative	344.2315	4.64	unknown	408386	323571	55486	1090903	307487	304633	281229	301599	1227978	173068	176231	405412	960537	432000	401377	730709	1243683	869612	520792
EIC	Negative	388.2249	4.60	unknown	465908	784841	393776	517548	638523	685291	591305	515984	571641	320711	590385	634131	938568	888512	913922	1019515	1041902	633092	1075603
EIC	Negative	294.0968	1.08	unknown	22619	12264	0	50518	3920	21366	3341	62432	64944	0	22811	43046	92519	81413	5373	20036	134281	64342	19437
EIC	Negative	370.2319	1.81	unknown	113989	60941	0	0	27013	131529	0	167738	437453	0	88591	278296	437188	514727	85220	94302	936608	0	83387
EIC	Negative	425.2765	1.67	unknown	25650	72506	0	0	80407	65938	69254	20354	75450	49678	79677	66227	74895	66053	74361	201023	89937	52040	72567
EIC	Negative	511.4731	6.54	unknown	3269671	4255423	4199197	7008315	5371197	3304241	5507724	6480638	7765070	6470664	10782695	6476554	11445426	12255312	11669157	9978900	9880175	6388477	6840047
EIC	Negative	435.2692	2.94	unknown	412140	618896	469879	734333	870437	722156	718604	682350	573203	443054	553809	684858	864276	935133	976565	1146822	1232831	1049042	1210740
EIC	Negative	285.0771	2.60	unknown	565725	732886	713660	489248	377669	625637	1341985	276012	845905	504199	1085012	2278073	1799802	991830	1205509	986495	1696399	629191	950413
fpHILIC	Positive	429.3737	0.40	unknown	787648	300030	444337	381393	802507	438717	741955	756502	735149	554799	495745	1019808	715851	856912	816331	984475	1084538	807966	1798381
EIC	Negative	348.1907	1.92	unknown	0	178876	0	0	62881	80749	120439	0	65634	0	121530	171269	162199	119692	140892	143285	124763	0	112411
EIC	Negative	475.3782	6.56	unknown	1389599	933040	852929	1135228	1338358	1033231	1331404	1327239	1577888	1561763	2277072	1138184	1564902	1861636	3161442	2117689	1175518	1702545	2352727
EIC	Negative	343.1915	1.98	unknown	0	2269079	0	0	238432	1123635	2063558	1434169	3224871	301880	2245397	534849	3824766	5884425	3644685	4440897	2503188	0	2290280
EIC	Negative	401.2308	4.76	unknown	2670476	1293885	251664	5178725	906530	1622215	777387	3420314	5363311	619107	1653997	4204064	5896931	4987924	2493970	1967894	6347959	6271281	2196645
EIC	Negative	227.1066	1.96	unknown	0	84340	0	0	55382	56882	73279	0	8152	0	80924	65459	83375	79442	128147	54567	67089	0	59732
EIC	Negative	402.2349	4.76	unknown	708994	344547	76492	1269601	242045	363236	183039	840919	1322440	170672	412935	996307	1490153	1197763	588829	481695	1594155	1495446	584773
fpHILIC	Negative	196.0285	1.62	unknown	0	0	580417	770658	842677	0	169083	794499	878412	807169	0	543888	1104847	1963276	0	0	1680307	908785	1731639
EIC	Negative	379.2128	0.89	unknown	45101	47940	45411	58335	84106	91527	66843	85072	47060	52582	62825	54556	46958	82101	113996	92789	141758	124695	132352
EIC	Negative	489.3234	2.87	unknown	0	285965	0	186840	273306	394793	235214	0	240818	561707	338872	348400	266576	351563	456007	340722	340224	398196	314968
EIC	Negative	473.3640	6.55	unknown	7518345	3883011	4879482	5513515	7410950	5368554	6158084	7311893	7911787	9157241	11745033	5272052	10026068	10477885	15821661	10892525	7189731	9514901	11971103
fpHILIC	Negative	213.0538	2.83	unknown	49838	241420	0	0	158912	233273	349013	0	334522	0	505625	711104	296567	323385	320922	545834	397852	68734	411219
EIC	Negative	474.3676	6.55	unknown	2392017	1236708	1627591	1850669	2448704	1782516	2030279	2526730	2587516	3030196	3965991	1809645	3284586	3485372	5315799	3566658	2232448	3252661	4155230
EIC	Negative	410.2008	3.52	unknown	256166	96334	243135	401354	169691	190074	251890	441917	165966	347311	128741	477878	379169	454189	236372	255493	399321	877173	218920
EIC	Negative	641.5642	6.55	unknown	2280022	1470747	2055150	2571136	2979715	2367302	2365325	2605599	4110596	3461599	4363860	2776528	5035516	5596824	4391850	4142085	4745088	5053256	5218423
EIC	Negative	282.1741	2.45	unknown	0	392016	0	367493	406643	88577	29410	634123	298334	179990	405889	374449	408317	396457	513688	353072	461276	610552	442088
EIC	Negative	488.2350	1.87	unknown	28101	243920	0	0	74578	110587	185843	0	243606	0	309221	109730	285846	287674	250450	295799	198536	0	262096
EIC	Negative	347.1774	2.31	unknown	806856	1346182	223791	0	591440	868164	1156090	235559	1051732	0	1174042	1848542	1577500	1354595	1388475	1190350	1276035	1420323	1124889
EIC	Negative	232.1089	0.72	unknown	44144	16307	2937	110055	17718	37412	18919	201704	115263	6009	36730	195722	175181	184297	69568	38610	268301	204906	61041
EIC	Negative	435.2405	1.87	unknown	0	83223	0	0	13519	81867	92939	0	95114	0	119492	56138	87230	103456	108962	163129	69211	0	126813
EIC	Negative	305.0494	1.99	unknown	257195	854099	236436	0	590947	578152	727447	219678	237509	193589	943001	1041346	990578	1515101	1414284	693085	869875	0	915472
EIC	Negative	511.3643	2.56	unknown	79488	53532	41738	23471	72053	75502	70386	97629	46941	110728	136069	55247	53069	121937	103421	146023	93752	166752	89799
EIC	Negative	286.0805	2.60	unknown	62213	131471	0	86727	69330	109090	232366	0	159100	74065	192544	380495	310471	182810	211522	155986	299346	111407	166272
fpHILIC	Negative	437.2131	1.65	unknown	0	0	1092595	1087191	955331	0	1450279	506720	2027634	1035572	1976567	1806870	1400189	1744013	2407404	2619821	1102523	911330	1147700
EIC	Negative	293.0937	1.08	unknown	129600	64611	21779	253402	29827	91671	13569	287778	352379	25794	117109	205792	472151	448459	65700	111719	696034	319869	92026

EIC	Negative	374.2452	2.94	unknown	664734	1086153	791576	1366484	1469565	1294358	1081973	1160563	1059364	760274	946037	1256409	1585648	1650658	1775035	2058147	2245278	1845954	2145064
EIC	Negative	613.5415	6.54	unknown	5134043	4835236	6890793	10563427	6552653	5784589	7930283	8607938	10253212	22088720	10321956	9125815	16417277	16128293	10225443	12504376	16428257	11228442	14625204
EIC	Negative	518.2877	2.94	unknown	84980	79392	102248	62047	78809	210660	41876	445933	63677	76829	0	63672	55266	24540	266750	79783	63534	908944	83943
EIC	Negative	434.2663	2.94	unknown	1445662	2215782	1687816	2785439	3143090	2698536	2244287	2318580	2116593	1615530	2096260	2519320	3346752	3434922	3739064	4366996	4567448	3829452	4392720
EIC	Negative	389.1982	3.11	unknown	0	91921	0	18221	29879	105767	42247	0	44581	15767	80916	157347	123628	74532	100146	81648	83272	20723	86394
EIC	Negative	294.2156	4.74	unknown	105197	127919	147518	214804	166403	145959	180576	157536	221558	135643	100467	127505	113252	102907	112705	874303	159116	184570	130680
EIC	Negative	408.2472	2.20	unknown	0	408369	0	1126323	469342	293364	598605	147504	527183	881493	748780	320658	656705	589524	431026	784029	389196	1194361	614783
fpHILIC	Positive	188.0345	2.98	unknown	2012126	2189684	1533622	435089	1997702	2047720	1007860	1754348	1977676	1349605	1403059	1666020	1332005	7196896	5134088	1315959	2063937	2177690	4069890
EIC	Negative	443.1672	2.59	unknown	0	0	0	0	56055	0	65813	0	51211	0	75244	94844	139893	82974	88085	52138	81620	0	69498
EIC	Negative	573.2321	1.93	unknown	0	426877	0	0	377748	321425	568997	0	323687	0	408431	601601	523522	562086	503097	630249	974279	0	579962
EIC	Negative	408.2395	1.87	unknown	57040	141682	0	0	0	84649	148385	0	232260	0	204438	0	246827	249699	221320	275465	140904	0	147965
EIC	Negative	471.3480	6.54	unknown	977287	549675	754995	1105692	877531	663085	1008137	1078650	1008619	1907781	1480890	760709	1018805	1568463	1913229	2276041	1024892	1180683	1436033
EIC	Negative	391.1740	2.73	unknown	237042	280540	108676	136792	263585	214062	325161	159557	193673	420510	254948	280487	304634	322419	426208	227203	418411	479028	401986
fpHILIC	Positive	304.0993	3.16	unknown	269193	989773	417730	369171	440624	664877	699282	365565	468504	534943	874877	1006101	943030	1002246	1091288	784186	775119	689399	1117869
EIC	Negative	435.2409	4.03	unknown	0	0	0	0	97765	0	0	0	0	123136	0	0	0	0	0	0	0	165500	0
EIC	Negative	346.1740	2.45	unknown	256707	530579	264663	220058	255570	466822	418706	290551	291739	185578	580789	677287	828003	564321	759614	556907	544595	942620	114153
EIC	Negative	335.1405	2.27	unknown	0	0	0	0	0	0	0	275430	0	0	0	110475	0	0	0	0	0	467174	0
EIC	Negative	411.1997	2.60	unknown	88376	63442	0	0	63568	134998	30519	0	47930	0	81066	59131	41071	170235	132406	38303	135272	0	163884
EIC	Negative	329.2337	2.01	unknown	2621228	1046127	874101	611334	1085834	2825485	2199322	521837	1625242	758981	789420	1495374	1191844	1009416	905883	9403001	1901566	607386	1221001
EIC	Negative	433.2213	1.48	unknown	0	255528	0	0	129429	117234	206420	0	171196	0	212724	269824	244775	220338	260439	340488	248011	0	214773
EIC	Negative	472.3509	6.54	unknown	340574	235210	277445	355827	337912	247802	331919	374002	359605	552837	520058	253845	383752	431310	736651	795055	357960	459331	471120
EIC	Negative	490.3260	2.87	unknown	0	75275	0	182615	55780	145200	86417	0	61235	201224	114650	112466	90302	105774	156440	108766	105575	123305	102244
EIC	Negative	628.3973	2.68	unknown	101159	243677	139809	201887	228485	126126	199484	90413	292493	79149	144383	181127	316045	330721	327955	597594	273184	174337	235442
EIC	Negative	528.3215	5.42	unknown	0	0	0	0	0	0	1594783	0	0	0	0	0	0	0	0	1152011	0	0	1602242
EIC	Negative	447.3483	6.54	unknown	4878675	2555609	3544045	3696406	4168949	2846396	4419189	4140887	4009104	6041930	6556937	3389765	5393858	6218828	9412820	8620355	4551196	4958159	6309058
EIC	Negative	428.1767	2.31	unknown	52492	306793	64245	223953	122317	151647	188064	226070	190661	489260	246920	403629	328247	304921	304786	253711	243744	482222	235169
EIC	Negative	301.0598	0.70	unknown	18542	42225	32856	35243	37161	41357	56149	53624	33409	49435	69941	71963	73724	78438	82133	85807	81387	97963	36324
fpHILIC	Positive	303.2315	0.37	unknown	295106	143736	103231	980821	120703	196687	109774	443742	967468	110744	185188	466579	1046864	592865	170806	209523	1455667	980814	366615
EIC	Negative	451.2654	4.06	unknown	0	284706	0	0	102705	362127	0	99675	169382	0	246564	209041	178821	465173	284410	236083	229338	207293	314381
EIC	Negative	379.1412	0.60	unknown	11926	0	0	37686	0	7242	0	94981	104073	0	0	66884	114886	63157	0	0	255403	102734	0
fpHILIC	Positive	192.1596	0.59	unknown	3495045	1719963	1795606	3654699	7115196	2129285	2399805	2698491	4122164	1934989	4600083	4802553	3231586	2169515	12475827	3736803	10551953	2673543	2685182
fpHILIC	Positive	251.1025	0.50	unknown	556533	105945	62694	196677	359919	261667	597223	316415	466788	110884	87704	367982	433721	711362	858721	233748	802821	571440	1037889
EIC	Negative	417.3226	6.55	unknown	15724501	8889110	10888284	16040801	17038663	15999903	14673391	20657738	25579516	26082590	29355190	20794314	30056995	33854257	33888290	26090991	25964213	34240454	45026711
EIC	Negative	387.1464	2.97	unknown	32559	43019	0	24582	64073	52350	53565	57280	51490	65880	109535	112511	103108	128536	105244	105657	120437	87349	74472
EIC	Negative	348.2631	5.60	unknown	120578	32961	40155	246810	83964	77491	40682	96764	222127	51604	20401	124824	204231	127697	116818	114371	314802	301844	78228
EIC	Negative	574.2347	1.93	unknown	0	133873	0	0	120928	94183	171763	0	90461	0	138032	206056	156066	194396	150487	207086	313095	0	179168
EIC	Negative	598.3077	6.32	unknown	63362	169774	302569	248951	254108	476778	351651	449359	307517	395159	553488	773924	526430	593427	421621	981185	386265	612495	877046
fpHILIC	Negative	223.0750	2.81	unknown	199540	182988	232479	196067	245641	560978	264387	203086	384795	197518	224203	322983	615671	197098	106592	2064897	263787	311949	213388
fpHILIC	Negative	133.0508	1.00	unknown	308027	341809	336063	369489	418922	484341	267236	355527	293170	389382	501918	442099	398206	1072694	534035	437305	722824	574000	979425
EIC	Negative	333.2200	5.72	unknown	0	406800	0	0	573047	464062	488544	0	465005	0	263793	294354	418642	397231	316164	1862640	312130	642557	427871
EIC	Negative	300.1685	1.92	unknown	0	215829	0	0	103548	149970	195621	0	90125	0	269885	345798	302234	228895	276025	257972	237817	0	193532
EIC	Negative	639.5575	6.55	unknown	21079025	9773642	12650727	19383170	20118860	17911856	17016775	19484049	35501663	28073512	39664156	25276385	46857760	43845019	39826672	29979489	32219340	46247394	49112033
EIC	Negative	409.2608	1.16	unknown	23291	13882	30010	19949	46243	69312	41229	68838	50853	44437	31920	24237	15871	37308	84740	48007	73716	230078	63241
EIC	Negative	443.1837	1.87	unknown	196674	518832	0	0	214239	419868	763625	0	1162105	0	1295190	400408	1450850	1426583	1150372	1543454	868319	0	1074431
EIC	Negative	418.3260	6.55	unknown	3974656	2333256	2675285	4068136	4293480	3986187	3651262	5195810	6260261	6728263	7578637	5329722	7715182	8658105	8649115	6740887	6711584	8656772	11674210
EIC	Negative	449.1948	1.48	unknown	0	49369	0	0	47286	64699	101255	0	64880	0	106167	111668	147093	98206	123985	162041	119653	0	99969
EIC	Negative	640.5612	6.55	unknown	9056136	4270666	5652907	8423234	8576747	7552535	7448495	8394838	15461905	12515420	17488586	10946545	20579354	19303543	17612710	13238147	14084629	20332066	21104589
EIC	Negative	409.2609	1.41	unknown	0	41279	0	0	266334	237083	183733	0	187051	0	161505	162403	54956	220531	472738	301013	535665	0	287947
fpHILIC	Negative	305.1432	0.90	unknown	1556632	1027879	1328494	1340222	1385544	1126028	1296336	1498992	1133297	1913079	1575661	1880030	1521712	1753319	1510931	3036757	1895032	3989654	1336672
EIC	Negative	329.2389	6.06	unknown	0	359600	0	0	0	0	0	867887	0	0	368949	0	0	0	427789	244132	276408	638642	0
EIC	Negative	512.2635	2.38	unknown	0	73657	0	0	32272	30074	58146	0	124081	460671	65023	34922	66835	80976	221388	95315	64980	0	66642
EIC	Negative	429.1960	1.16	unknown	30906	79440	109072	41398	239842	25972	160570	544797	118553	70005	33446	22281	17322	40234	0	62930	55273	1819815	68446
EIC	Negative	357.1376	1.97	unknown	0	207189	0	0	184203	126354	168530	0	93921	0	369740	322285	237876	282889	315024	221197	180969	0	338014
EIC	Negative	475.3442	5.14	unknown	282993	1957307	203194	970001	937738	809945	1281144	649075	2530652	579963	2839938	462801	3133754	3895672	3027154	3182246	1559877	638890	3041342
EIC	Negative	260.0679	2.14	unknown	0	132872	0	0	54323	71131	96799	0	33403	24963	100589	138990	59282	244021	164128	89769	62995	42891	113999
EIC	Negative	450.3670	6.55	unknown	1263365	676485	887448	886252	1273209	701197	1166545	970596	1280485	1394823	1810558	878176	1539718	1701758	3225217	2303882	1168808	1108315	2236530
EIC	Negative	308.1988	3.87	unknown	0	0	0	0	0	0	165448	0	0	0	0	0	158097	0	140823	0	167376	0	0
EIC	Negative	352.2213	1.48	unknown	0	307704	0	0	119748	142632	236285	0	164068	0	252566	294959	313097	268347	297762	383608	310136	0	245476
EIC	Negative	299.1653	1.92	unknown	314782	1034446	0	0	430247	678337	855863	0	509286	0	1085847	1564129	1436024	1093823	1336509	1269128	1118598	0	926309
EIC	Negative	555.2246	1.95	unknown	0	36010	0	0	66464	0	82247	0	50882	0	18965	47501	38035	35937	119835	118972	52667	0	84914
EIC	Negative	388.2609	3.02	unknown	81288	127190	50167	140898	139153	124472	123970	136560	100547	104516	100419	132998	177458	180634	204488	263109	242428	243085	226046
EIC	Negative	597.3047	6.33	unknown	358898	486678	748385	658466	728321	1546358	1048844	1355952	1021900	1255841	1844922	2486610	1666193	2094799	1473133	3266880	1147467	2126533	2885954
EIC	Negative	323.0411	0.70	unknown	0	4889	0	0	27943	32312	53590	62729	29400	38769	72871	67078	75404	32963	79821	84016	71078	121908	38173
fpHILIC	Negative	188.1007	2.81	unknown	196076	148328	187732	106522	199049	455499	222435	158580	320213	132731	160106	301148	515414	220493	111173	1751729	252242	293804	201506
EIC	Negative	642.5651	6.55	unknown	441002	232602	370069	333619	511085	423068	319086	388603	529949	639238	1074169	453672	1511968	815655	686335	780887	599663	1055819	1074572
EIC	Negative	451.2317	1.81	unknown	79979	35776	0	0	8373	44885	8508	35869	212955	0	49191	128468	213722	229797	46325	61195	443265	0	50275
EIC	Negative	493.2367	1.93	unknown	0	383701	0	0	297950	254216	470357	0	239610	0	434146	486488	451676	477183	377276	597221	896629	0	574528
EIC	Negative	476.3468	5.14	unknown	94084	583417	107973	273401	270809	255199	387574	204336	785754	188120	863073	156466	958958	1217798	933264	1025828	499319	226228	1009036
EIC	Negative	319.2640	3.31	unknown	0	0	0	0	0	0	248354	0	0	0	0	240603	0	0	241393	0	0	0	241527
EIC	Negative	344.1950	1.99	unknown	0	522530	0	0	52970	270233	477905	0	744192	0	505389	112916	859253	1352471	857766	1044155	574141	0	533268
fpHILIC	Positive	144.0656	0.95	unknown	1219395	2521505	1833929	1305085	2060199	2268498	2473573	1834317	1917682	1915005	4845863	3480243	3333248	6661366	4736805	3850879	4008797	3157244	4864507
EIC	Negative	315.1966	2.62	unknown	76575	166660	0	0	145930	85630	244754	299842	315448	82613	268083	0	224925	913663	430809	614646	161322	355061	572064
EIC	Negative	419.3267	6.55	unknown	841705	447267	570097	809034	806456	739375	822302	975260	1860504	1694161	1820690	1363044	1960824	1766513	2250866	1500814	1349677	2435414	2639913
EIC	Negative	367.1687	2.75	unknown	0	179472	0	90904	117025	159781	196715	53526	103905	289586	217661	310637	346075	216195	223699	226665	150748	370367	268752
EIC	Negative	512.4768	6.54	unknown	2258999	1571118	1480887	2434836	2038398	1169588	2054909	2396011	2687772	2385479	8274031	4528764	4359501	9829991	4109118	5384037	3637774	2294476	4992031
EIC	Negative	356.1438	1.38	unknown	0	50682	0	0	18303	60767	5906	0	17354	0	24065	34136	9550	160209	91915	18905	31961	0	60141
fpHILIC	Negative	187.0976	2.81	unknown	1718853	1641529	2121663	1192542	2025719	4124723	2045521	1551156	3130935	1647144	1712529	2890288	5145447	1777693	932179	17259566	2396499	2958350	2027082
EIC	Negative	439.2346	0.89	unknown	36633	51290	26690	35255	156207	156705	100459	67123	100890	63888	103639	103075	62959	130776	199107	174195	249167	232569	304666
fpHILIC	Negative	149.0606	0.54	unknown	175157	577082	162879	121033	634737	1005004	359148	88510	305770	346265	575364	1085297	334640	303127	483275	1548837	1050376	1090750	699270

EIC	Negative	447.2058	1.92	unknown	0	470244	0	0	179823	184577	200555	0	201141	0	380337	362165	269156	297899	304621	528359	444801	0	290211
EIC	Negative	306.1462	2.97	unknown	250776	252826	273284	279366	389950	318220	387684	289714	416738	484237	741345	866952	721521	943939	699682	789498	979398	738013	496493
EIC	Negative	433.2214	1.98	unknown	0	246378	0	0	85498	107404	158155	0	104789	0	214418	221426	137080	148497	168728	271621	287162	0	214550
EIC	Negative	305.1431	2.97	unknown	1554841	1544463	1597431	1720644	2410061	1994085	2368572	1653166	2462162	3104566	4599261	5267326	4489725	5640429	4450312	4748770	5802576	4522711	3088531
EIC	Negative	434.2237	1.92	unknown	0	364517	0	0	143199	137507	156923	0	164267	0	314695	285904	225261	239713	242882	395672	366624	0	246133
EIC	Negative	423.2260	3.91	unknown	46435	27813	0	0	91548	138827	52462	165214	88611	0	531786	84829	24874	0	0	593092	82231	421358	19537
EIC	Negative	483.2365	4.76	unknown	161238	41267	0	366668	32653	49466	0	205968	375075	21791	95440	239535	387424	294825	166674	106180	482054	526082	107477
EIC	Negative	251.1322	3.31	unknown	0	0	0	0	0	115025	0	0	0	0	0	0	123306	0	0	101465	139485	0	0
EIC	Negative	412.2424	1.99	unknown	0	189704	0	0	49945	97827	93954	0	107667	0	191921	179470	113734	83475	103195	278756	197884	0	159535
EIC	Negative	448.2731	3.02	unknown	162583	87134	162700	331670	140365	115343	270135	232776	259256	172227	223876	308248	434195	61978	54892	555175	536080	485113	547048
fpHILIC	Positive	404.1744	4.66	unknown	385110	63649	0	214123	42850	110152	0	253461	729497	0	49017	335581	454840	780583	117324	100502	1288681	607589	326937
EIC	Negative	325.2658	5.83	unknown	1053251	0	0	0	1457354	0	0	0	1102166	1186517	1228175	0	0	0	1361452	775840	0	2173302	1202837
EIC	Negative	361.2013	3.54	unknown	345490	169162	142475	629698	76136	247968	46791	436146	1026191	41968	213714	620702	961800	1080850	220619	162873	1800057	1568262	201384
EIC	Negative	448.2797	3.02	unknown	0	266515	151551	376493	350554	271611	305453	110362	308833	0	244390	354586	495980	435420	439924	657712	612453	570892	634508
EIC	Negative	433.2212	1.92	unknown	0	1543926	0	0	562336	569821	656399	0	649588	0	1256685	1202000	917056	1029001	1083358	1682439	1508239	0	1039432
EIC	Negative	580.3622	4.53	unknown	2461359	2308647	1865130	2255878	15055338	1549282	2020427	1584922	2095975	1616980	1809578	2211171	2231565	1906737	15277646	2022195	14796695	14717400	1877729
EIC	Negative	526.2845	5.06	unknown	0	0	0	0	3822612	0	0	0	1326455	2351558	3552434	0	0	0	4254710	1285772	2872855	0	3041508
fpHILIC	Negative	319.2276	0.38	unknown	1533081	884822	754092	5223019	1095040	1490434	917547	2443092	4891384	662254	1245090	2963014	6224401	3916574	1652489	1562688	11370218	7262106	2335896
fpHILIC	Negative	115.0039	0.83	unknown	842564	1748616	1164794	951208	1362030	1743370	878961	871459	853775	736588	834214	1176760	1168168	1057974	1163126	1887228	1625811	2119593	5946747
EIC	Negative	319.1590	3.13	unknown	0	14323	0	0	161069	54016	67223	80218	94430	464988	0	16966	50243	113567	139630	124672	159564	430757	165866
EIC	Negative	319.2280	3.84	unknown	0	0	0	11536961	0	0	0	10742769	12211002	0	13254241	0	0	0	12807031	13063435	13073225	0	12557610
EIC	Negative	261.0220	1.95	unknown	0	87357	0	0	68274	84071	75412	0	79343	0	81927	99682	152679	88843	74692	115079	415765	0	96913
EIC	Negative	644.3514	2.67	unknown	0	0	0	0	103479	96263	0	0	0	72427	47154	173417	131464	0	0	0	213269	0	247169
EIC	Negative	439.2258	6.22	unknown	228398	445575	96245	126551	509791	934904	136885	310040	392946	209372	184768	1004289	333142	237674	219992	2576868	312395	1158751	1023199
EIC	Negative	440.2297	6.22	unknown	42124	103859	0	36867	137997	225718	36114	73718	115387	55138	0	264681	83116	60410	55422	677402	83546	300167	251660
EIC	Negative	492.3421	3.19	unknown	78208	63892	79868	53809	73239	83718	70175	88099	68477	259107	174527	196853	101984	79767	241648	252990	109846	221376	68297
EIC	Negative	491.3381	3.19	unknown	280406	206200	266665	177343	223410	272877	205844	242695	217040	761556	513650	603105	329115	254401	755012	781770	309244	689318	231597
EIC	Negative	321.2342	4.76	unknown	809634	290549	0	2337982	222674	447446	259568	1229778	2173270	0	393931	1347914	2881081	2078143	700177	560718	4852779	3611969	596987
EIC	Negative	279.1968	3.54	unknown	2773068	1360117	298375	8478730	495603	2736846	211330	5168821	11227278	613202	2518799	7242601	11069322	12647701	2119106	1981807	22883990	18713170	1599416
EIC	Negative	441.2524	0.88	unknown	21271	52716	30714	25923	164287	152557	108026	69758	108624	62095	109187	77710	53280	84532	216751	271322	316796	344487	290839
EIC	Negative	280.2000	3.54	unknown	507709	263825	76218	1370252	104815	496487	47693	897996	2137974	135444	489398	1320344	2117518	2360894	471210	407491	4345839	3464838	329259
EIC	Negative	328.1846	2.19	unknown	32331	0	0	0	0	106384	0	0	51941	0	0	0	0	0	0	0	276950	0	111439
fpHILIC	Negative	259.0906	3.16	unknown	67290	267825	39311	0	129846	209286	126221	63285	99852	74623	304169	375648	272409	557380	494640	324948	188837	150183	384792
EIC	Negative	320.2312	4.76	unknown	5832006	2297050	507973	17278146	1627731	3117386	1586759	8857965	16462577	1124978	3491684	9968528	21638369	15773773	5348404	4169274	36098360	27919501	4466747
EIC	Negative	449.1948	1.92	unknown	0	262761	0	0	150465	169347	239078	0	221741	0	438257	436279	347874	361475	396780	618625	567470	0	362029
EIC	Negative	577.3016	5.05	unknown	122847	577339	128716	329880	332943	560852	347511	217125	331828	525961	1209937	814954	1356474	693173	919412	955471	1118496	557812	936205
EIC	Negative	429.1684	2.75	unknown	0	0	0	0	107668	152155	258085	0	127842	268044	253449	363591	428705	358245	269684	275815	234853	504958	335282
EIC	Negative	309.1546	3.58	unknown	0	0	0	0	0	0	47702	0	87623	0	0	0	0	0	0	0	107216	141621	91727
EIC	Negative	307.1587	4.43	unknown	185968	493947	122469	162522	447474	598138	185982	173427	3522699	240609	141661	81246	199640	286388	395238	117489	5254598	6467779	271727
EIC	Negative	322.2371	4.76	unknown	80452	27814	0	263168	0	47738	0	156172	219867	0	43203	152188	332314	233152	93842	52758	520933	325147	90861
EIC	Negative	355.2054	4.76	unknown	403668	131461	30606	1094524	114814	192090	105300	528892	971905	95236	175696	611783	1339336	911642	296222	275749	2204411	1808491	323171
EIC	Negative	432.2108	1.02	unknown	22330	0	17887	20726	0	11288	210155	22407	0	28852	0	183700	197359	0	307143	268659	16024	21850	16465
EIC	Negative	299.2530	5.50	unknown	0	0	0	0	0	0	1961860	0	0	1880055	1234660	0	1462121	0	795826	1374076	1571242	0	1354752
EIC	Negative	290.1433	1.87	unknown	0	10091	0	0	0	88226	23010	0	0	0	31925	26775	35562	12405	0	140458	119623	0	30868
EIC	Negative	417.2053	4.76	unknown	29073	0	0	302992	25025	29909	0	295588	410120	0	85424	239658	550192	379771	155829	119286	858088	738767	122300
EIC	Negative	345.2103	2.19	unknown	0	21326	0	0	0	1047406	0	134626	0	352308	0	0	30112	0	1017276	60762	0	0	2067407
EIC	Negative	427.1798	2.75	unknown	81114	366115	0	111010	179080	209037	355434	0	191820	506174	464988	554464	586342	399239	404158	369762	417050	869134	450904
EIC	Negative	539.3045	3.73	unknown	0	0	0	0	0	0	61767	0	0	0	0	117442	0	0	169187	0	0	0	0
fpHILIC	Negative	185.0277	0.47	unknown	160864	234250	129845	107249	234184	527284	493347	70603	360960	68645	0	254130	1100454	223801	139907	533730	3025044	205418	716879
fpHILIC	Positive	399.2507	0.40	unknown	213800	162517	269662	507152	454615	283301	432553	1751394	625944	329717	3240050	1262312	547583	313482	675568	510372	635166	3064443	5034909
EIC	Negative	555.3913	6.54	unknown	520736	287894	241037	566993	256892	485878	404606	661993	501661	508373	871877	402879	853781	979515	958410	1507106	901536	2212869	1653183
EIC	Negative	493.2447	1.92	unknown	0	354461	0	0	26035	68672	448509	0	51932	0	433372	93432	197996	203823	112142	420685	640322	0	500347
EIC	Negative	619.3459	2.48	unknown	0	68543	0	0	0	41657	102240	76059	38092	0	121596	0	0	49328	35223	205867	190878	85426	211110
fpHILIC	Positive	127.0388	3.53	unknown	56861	735491	439324	41040	97949	55737	339639	125746	714298	50837	0	56899	103407	748750	36266	51411	194119	98475	3388559
EIC	Negative	413.1939	2.76	unknown	141646	883175	241777	193347	468901	533984	970009	97550	366422	1322555	1133550	1595446	1912087	1467703	1259820	1098702	1080802	2064046	1389838
EIC	Negative	308.1616	4.42	unknown	0	55978	0	0	65184	80560	0	0	553133	31347	0	38160	25826	40092	53413	19480	844053	1024990	35028
EIC	Negative	249.1495	4.49	unknown	140597	473664	33563	45073	398823	371715	110551	43349	212322	39788	102542	0	0	29187	86870	2740089	0	37566	370246
fpHILIC	Negative	277.1112	1.03	unknown	356933	471787	517294	381638	571460	603644	459821	401270	625337	1135589	1806844	1356926	1560343	2052329	1185078	1334256	2353900	1530257	1217066
EIC	Negative	556.3383	2.41	unknown	0	66903	0	0	44519	38301	33962	0	70382	0	0	255111	351484	577617	66044	180936	218352	0	84025
EIC	Negative	306.1464	3.24	unknown	323824	287012	338917	326631	453140	421027	433549	376358	397644	820296	1263868	1403430	1069019	1306611	1127262	1205866	1357817	1486627	934405
EIC	Negative	421.1821	3.76	unknown	0	237978	0	0	183238	242333	0	0	0	0	0	136806	0	0	18154	338508	0	596266	296750
fpHILIC	Positive	175.0707	3.68	unknown	556321	433098	535259	553071	741659	551097	440694	457182	841103	803870	1354615	947885	603621	1073594	1076943	1636465	3493989	1127075	1643731
EIC	Negative	299.2530	5.25	unknown	0	0	0	0	0	0	2626330	0	0	3150709	1836600	0	2742177	0	951825	2850025	2154881	0	1866278
EIC	Negative	489.3231	2.67	unknown	0	55259	0	0	44318	50681	49814	0	0	115820	55956	83540	77374	57799	77992	64006	67523	162497	61324
EIC	Negative	305.1432	3.24	unknown	2003948	1853334	2098034	1960441	2693976	2479179	2626183	2305518	2388343	4916435	7633141	8616504	6552531	7947389	7039990	7271370	8330924	8924704	5818984
EIC	Negative	279.1121	1.53	unknown	0	0	0	85167	11694	46876	47659	164558	31571	111807	58425	58271	257778	90100	135565	25932	181438	458214	107758
EIC	Negative	299.2530	5.38	unknown	0	0	0	0	0	0	4768762	0	0	5219021	3022751	0	3725811	0	2841321	2861252	4015895	0	4685619
fpHILIC	Positive	410.2664	0.35	unknown	136133	599083	42001	53880	335165	410674	0	77710	196780	47068	39088	662263	77390	94901	43412	1642064	92440	640813	678278
EIC	Negative	414.1978	2.75	unknown	0	231904	0	0	100605	160459	239606	0	83422	323418	268172	400020	399032	334650	309510	302493	318525	523603	330171
EIC	Negative	320.2313	3.84	unknown	0	0	0	3437093	0	0	0	0	3742916	0	0	0	0	3792678	3858395	3856026	0	0	3765633
EIC	Negative	427.1738	1.54	unknown	0	0	0	0	0	205128	0	0	0	0	125132	0	0	0	0	0	630583	0	0
EIC	Negative	402.2348	3.83	unknown	18263	0	0	0	0	22916	60632	0	310664	37514	310534	299050	68756	292646	303045	332377	282027	299492	0
EIC	Negative	435.2411	4.13	unknown	0	0	0	0	0	0	310333	0	0	380150	0	228870	0	0	165122	0	0	508602	290496
EIC	Negative	305.1735	3.12	unknown	0	0	0	0	0	0	0	78038	101375	0	38507	44109	38591	47475	42893	47567	35640	331156	107565
EIC	Negative	556.3956	6.54	unknown	273446	154327	109242	214475	234278	265913	182087	99831	138307	177934	257293	255903	290288	370656	711807	329072	421532	515616	927191
EIC	Negative	340.1825	3.76	unknown	0	250584	0	0	196377	229401	0	0	0	0	0	142947	0	0	0	349724	0	678722	317567
EIC	Negative	339.1792	3.76	unknown	0	1366443	0	0	1098862	1267175	0	0	0	0	0	860830	87820	0	157024	2027560	0	3799427	1815098
fpHILIC	Negative	145.0506	3.69	unknown	150597	832789	129820	0	465164	654539	91061	103282	131187	0	0	281377	0	0	280226	3398074	0	376725	751507
EIC	Negative	322.2438	4.76	unknown	72265	0	36584	28198	0	0	0	144464	0	22855	0	141135	302069	0	0	0	489425	0	0

EIC	Negative	299.2530	5.44	unknown	0	0	0	0	0	0	1829173	0	0	1201852	1094773	0	1364912	0	1497038	2157756	1293294	0	1678463
EIC	Negative	258.1796	3.76	unknown	177004	2432171	24120	22057	1921761	2332915	91198	60841	30454	18324	68646	1628712	136645	31500	252467	3987210	93085	8260824	3562147
EIC	Negative	257.1760	3.76	unknown	1090033	15737708	216034	208610	12326718	14561214	567910	371698	200612	176590	384553	10539708	893686	154909	1620743	25622957	579450	52825975	22230225
fpHILIC	Positive	148.0965	3.19	unknown	770835	890873	469792	543174	778773	977879	876010	477466	1466531	1826204	3737082	5642697	2091261	376540	1303127	6128623	655697	6670909	2242626
EIC	Negative	259.1813	3.76	unknown	18722	293808	0	0	234455	274221	0	0	0	0	0	188871	0	0	33608	504548	0	973287	431847
fpHILIC	Negative	530.2781	0.61	unknown	569098	48133	1382275	904363	35947	34120	43802	2821245	83971	1001463	43879	0	0	251343	102395	95070	0	11527478	0
EIC	Negative	410.2636	1.10	unknown	19967	13447	27377	5776	47815	35420	20231	14796	31231	57613	67261	54101	30013	101682	102467	70484	130544	145704	163910
EIC	Negative	409.2607	1.10	unknown	65855	50615	85074	30620	179913	125003	69533	70191	123972	187169	242284	193849	116966	385808	419791	220356	510054	609583	648323
EIC	Negative	532.2819	1.96	unknown	0	0	0	0	0	0	0	0	83539	0	55194	47071	81703	211329	126955	78236	90614	0	70760
EIC	Negative	582.2815	1.99	unknown	0	0	0	0	0	0	54447	0	0	0	0	72462	0	0	121763	85741	0	0	63408
EIC	Negative	355.2051	3.32	unknown	0	71314	0	0	70928	58774	0	0	69571	0	0	0	181154	63973	0	144935	63349	995837	0
EIC	Negative	573.3794	3.76	unknown	17980	227294	0	0	129628	183931	0	0	0	0	0	188775	0	0	0	513929	0	1283758	255712
EIC	Negative	639.4646	4.76	unknown	239053	39605	0	2086575	0	43315	0	624644	1836961	0	101258	683756	3378542	1800215	192828	127448	10436623	5691403	123652
EIC	Negative	415.2259	2.44	unknown	0	0	0	0	0	0	0	415211	0	0	0	0	0	0	0	915039	0	1859408	0
EIC	Negative	367.1592	2.13	unknown	0	0	0	0	123552	0	0	0	8567	0	66687	0	0	0	35281	40214	0	62311	761954
EIC	Negative	530.2798	1.95	unknown	0	29014	0	0	57964	32308	41967	311363	228980	349639	426197	379608	717632	2167504	1242339	713394	746387	1443527	654082
fpHILIC	Negative	155.0351	3.75	unknown	463669	122772	160474	168237	396588	97226	205192	93318	185988	815476	275763	105888	135907	224310	696706	152222	4426145	706458	1096642
EIC	Negative	464.3097	2.14	unknown	0	0	0	0	0	60689	0	0	0	0	97392	0	0	664461	172432	0	0	0	279268
EIC	Negative	325.2018	2.58	unknown	296430	334395	105078	281030	476931	638276	287676	158902	451012	232403	261095	200149	215383	200832	280233	15061403	193466	213009	269235
EIC	Negative	612.2817	1.59	unknown	0	0	0	0	54507	0	8483	0	105883	0	42066	80370	45467	407459	394051	459028	246312	0	302423
EIC	Negative	532.2813	1.26	unknown	86105	0	228548	112726	0	0	0	455878	16666	349731	0	0	0	0	0	0	0	4280321	0
EIC	Negative	345.2105	2.51	unknown	0	0	0	0	18635	0	31306	0	75755	0	0	192356	0	0	689198	0	0	0	450340
EIC	Negative	455.2407	2.34	unknown	0	0	0	0	0	8701	33080	0	0	45477	178907	19658	0	194168	8892	0	14211	373016	0
EIC	Negative	321.2340	6.21	unknown	0	0	0	0	0	0	0	0	151112	0	0	0	0	0	442248	0	595967	0	464245
EIC	Negative	613.2883	1.59	unknown	0	0	0	0	10614	0	0	0	26178	0	0	9403	10200	122759	130514	157622	82215	0	92591
fpHILIC	Positive	101.0600	3.56	unknown	36658	0	157025	36528	91680	95688	62801	0	0	972154	454745	635665	411123	0	554628	462163	402688	1519414	874135
EIC	Negative	531.2847	1.95	unknown	0	30833	0	0	0	0	0	0	56626	0	198068	132738	222632	656806	427538	246638	268985	0	219656
EIC	Negative	486.1887	0.73	unknown	0	0	0	0	0	5099	0	0	0	67360	127777	168378	126806	97411	35204	107539	155735	97115	59832
EIC	Negative	337.2297	2.15	unknown	1293341	0	1142007	1330533	0	0	0	0	0	0	0	0	0	0	0	0	0	0	0
EIC	Negative	577.3347	3.65	unknown	12547811	14623181	8661424	0	0	0	0	0	0	0	0	0	12454176	0	0	0	0	0	0
EIC	Negative	636.4365	4.28	unknown	174510	0	280580	113906	0	0	0	0	0	273489	47633	0	39551	0	0	0	0	0	0
EIC	Negative	531.3323	4.13	unknown	220405	0	139615	43813	0	0	0	0	0	72234	0	0	0	0	0	0	0	0	0
EIC	Negative	507.3280	2.44	unknown	528136	273202	53447	0	0	0	0	0	0	0	248226	0	0	0	0	0	0	0	0
EIC	Negative	499.2853	1.98	unknown	0	2923784	0	0	0	0	0	0	0	0	0	0	8953628	0	0	0	0	0	0
EIC	Negative	416.2011	5.67	unknown	0	100262	0	0	0	0	0	0	0	0	0	110472	0	0	0	0	0	0	0
EIC	Negative	430.3048	5.65	unknown	0	498303	341950	0	0	0	0	0	0	0	493104	0	0	0	0	0	0	0	0
EIC	Negative	597.3053	1.50	unknown	0	55901	0	0	0	0	0	0	0	0	430756	285714	1652890	1169784	0	0	0	0	0
EIC	Negative	280.2002	3.65	unknown	0	0	0	1063057	0	0	0	0	0	0	0	0	1131901	851993	0	0	0	0	0
EIC	Negative	412.2786	4.04	unknown	0	0	0	410406	0	0	0	0	0	535430	646822	541877	0	0	0	0	0	0	0
EIC	Negative	251.1322	2.40	unknown	141766	81572	0	0	0	0	0	0	0	0	0	0	0	0	0	79134	0	0	0
EIC	Negative	426.1848	0.69	unknown	93945	0	0	0	0	0	0	0	0	0	0	0	0	80523	0	0	0	0	92117
EIC	Negative	359.2205	6.10	unknown	98531	0	180400	33248	0	0	0	0	0	298552	26729	19639	0	0	0	0	0	29839	65270
EIC	Negative	313.1547	2.22	unknown	0	0	0	0	0	0	0	0	0	348664	0	487043	0	257762	102434	0	0	0	0
EIC	Negative	280.2001	3.79	unknown	0	0	0	231836	0	0	0	0	0	0	0	0	116630	82300	0	0	147196	0	0
EIC	Negative	357.1702	5.65	unknown	0	0	0	0	0	0	0	0	0	121401	0	0	135701	0	0	168920	0	0	0
EIC	Negative	634.4106	3.76	unknown	0	0	818605	481990	0	0	0	0	0	0	128457	0	0	130783	0	125443	0	45966	0
EIC	Negative	460.1901	3.92	unknown	190950	55118	119550	411150	0	0	0	0	0	325603	88266	0	0	0	19912	84883	0	72418	0
EIC	Negative	293.1019	2.56	unknown	0	0	0	0	0	0	0	0	0	0	0	115332	120565	0	98534	90853	0	0	0
EIC	Negative	357.1675	5.65	unknown	0	0	169021	109221	0	0	0	0	0	0	0	0	0	0	0	0	0	193897	0
EIC	Negative	488.2276	2.70	unknown	0	0	0	0	0	0	0	0	0	0	97698	0	99205	0	0	95039	0	0	114531
EIC	Negative	371.1814	2.44	unknown	210914	42984	214285	154035	0	0	0	0	0	400930	13656	0	8696	91595	0	57048	76653	0	81952
EIC	Negative	299.1877	5.23	unknown	0	0	0	0	0	0	0	0	0	288179	0	150968	180380	0	22521	115287	0	93541	0
EIC	Negative	401.2309	3.36	unknown	0	0	257595	207248	0	0	0	0	0	0	222478	170154	0	0	0	0	0	0	240498
EIC	Negative	396.1867	0.71	unknown	0	159929	183609	0	0	0	0	0	0	149225	160392	140434	0	146930	0	0	103317	0	161099
EIC	Negative	345.1558	5.58	unknown	0	0	0	0	0	0	0	0	0	0	0	532095	0	213748	0	0	0	0	283129
EIC	Negative	268.1926	3.44	unknown	316578	0	0	0	0	0	0	0	0	0	0	0	0	0	103847	200833	0	0	0
EIC	Negative	422.2188	3.11	unknown	138494	0	694971	200376	0	0	0	0	0	0	0	0	0	0	0	0	264055	60987	0
EIC	Negative	395.1842	6.09	unknown	281411	0	557227	196428	0	0	0	0	0	745879	0	124376	0	0	0	94977	116426	0	117678
EIC	Negative	345.1559	3.76	unknown	0	0	0	0	0	0	0	0	0	0	0	486823	490381	232229	0	0	0	0	343778
EIC	Negative	320.2313	3.36	unknown	0	0	0	307126	0	0	0	0	0	705222	0	0	0	154431	0	160049	0	0	188440
EIC	Negative	451.1650	3.14	unknown	0	77280	0	0	0	0	0	0	0	0	0	13951	107611	0	0	0	0	141256	227359
EIC	Negative	329.2390	3.80	unknown	0	0	595889	429457	0	0	0	0	0	0	0	0	0	0	0	163334	214375	0	0
EIC	Negative	251.1322	2.56	unknown	1321653	609978	0	158672	0	0	0	0	0	44256	0	0	450442	0	0	0	425730	0	0
EIC	Negative	476.2786	3.41	unknown	0	0	0	1576744	0	0	0	0	0	0	0	0	882671	0	0	429892	0	0	12636
EIC	Negative	393.2764	1.71	unknown	0	0	0	0	0	0	0	0	0	0	0	131227	0	368890	283015	0	186706	0	0
EIC	Negative	358.2160	5.81	unknown	0	0	0	0	0	0	0	0	0	0	176390	0	0	119369	141912	0	329062	0	0
EIC	Negative	519.3338	4.08	unknown	68245	20572	58941	0	0	0	0	0	0	211016	76106	138455	95031	139081	95167	79756	121844	199110	53004
EIC	Negative	451.1648	3.29	unknown	0	129031	0	0	0	0	0	0	0	0	0	0	164847	0	0	15258	0	268556	327785
EIC	Negative	359.2444	3.17	unknown	0	0	0	0	0	0	0	0	0	195153	152338	103684	104095	478108	154377	10335	206349	250547	0
EIC	Negative	335.1414	2.68	unknown	0	0	0	0	0	0	0	0	0	0	0	210121	0	313716	0	0	223303	197353	212494
EIC	Negative	337.2328	2.85	unknown	0	125399	0	0	0	0	0	0	0	0	114966	0	116777	112194	116961	108339	130066	166764	114452
EIC	Negative	329.2389	2.30	unknown	0	0	1150778	475134	0	0	0	0	0	0	272097	0	0	0	337733	0	421212	0	0
EIC	Negative	424.2315	3.64	unknown	506550	0	2061208	770582	0	0	0	0	0	92376	0	32170	26750	0	0	0	799686	0	0
EIC	Negative	321.2342	5.39	unknown	0	0	0	0	0	0	0	0	0	0	0	0	0	214979	0	284563	530561	0	0
EIC	Negative	347.1785	6.06	unknown	667852	0	0	0	0	0	0	0	0	837437	0	0	0	0	0	0	828835	0	0
EIC	Negative	281.1738	2.45	unknown	0	0	75271	1662375	0	0	0	0	0	0	1194977	0	0	0	0	1003246	0	0	0
EIC	Negative	413.2005	2.67	unknown	1350349	0	0	1171628	0	0	0	0	0	0	0	0	1524156	0	0	0	0	0	1079402
EIC	Negative	642.3156	5.54	unknown	0	0	0	0	0	0	0	0	0	0	0	0	0	0	0	415233	328145	380099	0
EIC	Negative	325.2658	6.50	unknown	0	0	0	0	0	0	0	0	0	721941	0	0	0	0	403118	695433	0	0	517040

EIC	Negative	397.2288	4.22	unknown	90611	0	0	0	0	0	0	0	0	0	0	689975	0	0	0	723987	965409	0	0
EIC	Negative	325.2658	6.57	unknown	0	0	0	0	0	0	0	0	0	1200968	0	0	0	0	808337	933862	0	0	0
EIC	Negative	509.2879	5.59	unknown	0	0	569037	0	0	0	0	0	0	484114	0	531811	0	0	0	403399	749592	0	667523
EIC	Negative	329.2389	2.02	unknown	0	907066	698824	159913	0	0	0	0	0	0	0	0	0	0	788830	0	1568324	0	0
EIC	Negative	605.3658	4.53	unknown	2314930	0	0	2142147	0	0	0	0	0	2126917	2266800	0	0	2125100	2374463	0	75482	0	50045
EIC	Negative	555.3508	3.67	unknown	2152348	2758302	0	1511815	0	0	0	0	0	32233	0	28148	29203	28157	0	26677	2701593	0	0
EIC	Negative	363.2181	5.82	unknown	0	0	0	0	0	0	0	0	0	0	0	26272	1531609	0	2230502	572445	0	0	0
EIC	Negative	307.1946	5.80	unknown	0	0	0	0	0	0	0	0	0	0	3090797	0	5071357	0	0	0	4476422	0	0
EIC	Negative	506.2598	2.19	unknown	1018061	35893	1458518	350271	89951	83280	63025	1515354	56133	118350	0	0	6963	0	0	0	0	0	10073
EIC	Negative	453.2864	2.13	unknown	561585	235480	537911	270696	233372	241473	291230	424521	179129	42254	0	0	0	0	0	0	0	0	8009
EIC	Negative	391.2858	2.57	unknown	8317398	721161	5938501	2017803	895343	835863	1583445	2295353	452289	227316	22983	0	38042	0	0	0	0	0	38517
EIC	Negative	612.4276	2.30	unknown	12271202	196988	13325769	4193435	895706	373916	378242	8788113	132971	748884	0	0	0	0	0	0	0	73123	0
EIC	Negative	611.4254	2.30	unknown	35888157	819482	37454611	11775928	2016230	1082764	1027889	25727662	323806	1664780	0	0	0	0	0	0	0	215757	0
EIC	Negative	392.2893	2.76	unknown	2189804	679045	2774346	2691174	1171839	695698	1544848	818529	319927	206829	41063	14929	51240	0	0	0	0	0	36516
EIC	Negative	389.2699	3.55	unknown	7497199	434335	7736712	1794301	361667	630193	900081	2634754	171254	175672	34752	0	11099	0	0	0	0	39479	0
EIC	Negative	391.2858	2.84	unknown	15197560	1855257	9587468	5892071	1509169	3436431	3937205	6819188	1588914	651251	53613	14494	43458	14659	0	30341	0	71863	44127
EIC	Negative	443.2572	2.75	unknown	14857804	977251	16796502	6339174	1525799	1976265	998272	12128232	1843474	278819	0	0	293594	0	0	0	0	161209	0
EIC	Negative	611.9264	2.30	unknown	28675707	543661	30339342	9588164	1642893	825916	883441	20580809	324159	1052213	0	0	0	0	0	0	0	228369	0
EIC	Negative	452.3108	2.84	unknown	12109022	1589133	7302146	4169935	1231005	2826186	3333602	4939025	1320688	550624	17090	0	69449	0	0	11847	0	86143	32979
EIC	Negative	505.2574	2.19	unknown	3647603	111375	5426427	1461844	304112	206839	205234	5506215	307124	376108	0	0	31240	0	0	8523	0	62555	0
EIC	Negative	451.3070	2.84	unknown	41313466	5468787	24678449	14646778	4331729	9903939	11567450	17144377	4614635	1962150	95956	56858	229273	36919	40146	88598	38604	259446	96637
EIC	Negative	489.2629	3.69	unknown	915544	21576	1694991	366557	253092	128468	228547	842427	102762	20408	0	0	0	0	0	0	0	17713	0
EIC	Negative	571.3403	1.67	unknown	1051579	266375	1056523	354167	392447	255720	456015	303828	162806	51152	0	0	0	0	0	18852	0	0	0
EIC	Negative	467.3018	2.19	unknown	150389348	8488248	171904562	47842512	15161490	10666960	8995796	128103669	10905954	11591098	238344	157174	1481799	170669	0	0	89155	1578051	495889
EIC	Negative	468.3051	2.19	unknown	43412424	2429949	49569430	13969022	4430553	3147152	2598237	36799000	3113060	3400336	61897	47161	427956	33859	30861	48113	21280	420332	114902
EIC	Negative	505.2572	2.75	unknown	3637672	386348	5291874	3104879	935296	1200896	728875	6080232	1326564	217620	0	0	275048	0	0	0	0	133658	0
EIC	Negative	587.3592	2.12	unknown	3794956	527056	1845254	186743	476437	531195	570001	1005559	378045	101066	18611	0	0	0	0	38581	0	0	0
EIC	Negative	453.3138	3.68	unknown	3477208	158895	5682471	957507	494706	282660	524809	1612881	213608	49894	0	0	0	0	0	0	0	41374	0
EIC	Negative	392.2891	3.68	unknown	6309472	315833	9701711	1612405	866905	553213	876698	2694933	342226	116156	0	0	54206	0	0	0	0	62925	10680
EIC	Negative	584.3162	1.54	unknown	1394657	90062	1224185	367774	312602	109750	272177	341773	97810	0	12040	0	0	0	0	0	0	0	16585
EIC	Negative	469.3078	2.75	unknown	4152374	556690	4711389	2508475	763060	929391	477931	4105825	939748	157828	15220	0	218798	0	0	0	0	106456	0
EIC	Negative	570.3369	1.67	unknown	5114514	1597337	5045620	2737268	1688613	948037	2912950	1836529	755668	76969	26500	8420	52942	0	0	106169	0	0	15895
fpHILIC	Negative	227.9983	0.94	unknown	2045051	696966	1361974	763819	952350	1122646	1123790	1226245	582485	68731	0	120580	58195	0	0	43622	37157	0	0
EIC	Negative	452.3108	3.69	unknown	19264402	920056	30391945	5252187	2923044	1656278	2911396	8831586	1108029	327794	10337	27787	117593	0	0	0	0	242158	44725
fpHILIC	Negative	408.2839	0.97	unknown	14981975	690277	20906678	3192723	920766	831341	888566	8529488	682628	282886	32009	0	109819	0	0	0	0	197330	0
EIC	Negative	451.3069	2.57	unknown	25109854	2144771	17705214	6211418	2682746	2492933	4608524	6702705	1368226	510410	61155	45832	115338	26445	37292	10106	15327	147539	88596
EIC	Negative	408.2838	2.75	unknown	45530553	5089195	50488532	26681336	8020844	10185554	5219781	41129427	9897766	1548192	98398	0	1747226	99850	0	0	0	1084110	195787
EIC	Negative	405.2649	2.82	unknown	1225441	102851	1810677	527432	197770	165350	242158	577655	79639	322805	0	0	0	0	0	0	0	22174	0
EIC	Negative	409.2866	2.75	unknown	7526056	866120	8316849	4294932	1271937	1600472	855617	6717396	1600998	279644	0	0	306772	0	0	0	0	215345	0
EIC	Negative	441.2419	2.48	unknown	5737349	1068857	10131687	7563487	414632	707931	1712839	1432049	332150	111210	0	0	13848	0	0	0	0	82631	0
EIC	Negative	453.3134	2.84	unknown	2121073	282949	1448416	837354	222734	478308	583993	868062	228958	106413	0	0	15625	0	0	0	0	33002	10320
EIC	Negative	406.2682	2.48	unknown	5192828	1087774	7419820	5828875	417972	710383	1624003	1674389	385089	123150	13034	0	30597	0	0	0	0	90393	0
fpHILIC	Negative	407.2808	0.96	unknown	56413820	2496060	80127344	12295755	3370492	3518617	3269180	33779484	2604484	1498810	52867	40029	334647	0	0	50236	0	728524	113088
EIC	Negative	469.3078	2.19	unknown	8319040	453094	9610812	2659642	824935	573459	485577	7017133	602028	596965	10826	0	94081	0	0	32010	0	126497	25233
EIC	Negative	405.2649	2.36	unknown	7903674	274403	11224076	2192369	290720	724718	350453	3723698	211558	206235	0	0	0	0	0	0	0	107170	0
EIC	Negative	405.2647	3.16	unknown	570875	70849	572637	41487	108018	146306	104481	107390	54532	0	0	0	0	0	0	0	0	11153	0
EIC	Negative	487.2737	3.68	unknown	2523259	159279	3087148	793057	554281	289409	489610	1268231	196481	38399	0	0	15594	0	0	0	0	60182	0
EIC	Negative	485.2577	3.61	unknown	577696	21297	485620	231274	109657	41024	118028	225330	18267	69732	0	0	0	0	0	0	0	0	11377
EIC	Negative	406.2683	2.36	unknown	2103338	81451	2999913	612392	99341	198286	98895	976812	65468	73834	0	0	0	0	0	0	0	32436	0
EIC	Negative	487.2736	2.55	unknown	1158526	53532	574401	209131	163388	0	164784	399959	28774	40991	11534	0	12378	18844	0	0	0	0	17108
EIC	Negative	453.3135	2.76	unknown	1152240	427276	1697515	1666401	740026	402300	940555	646525	182873	113808	20378	0	27284	0	0	0	0	41278	24626
EIC	Negative	468.3053	2.75	unknown	22448242	2682939	24961859	12629273	4222771	5015671	2608519	20554834	5101658	819383	85148	58376	895186	116600	77490	73003	62662	571334	119424
EIC	Negative	405.2649	2.48	unknown	19593464	3862286	28925411	22407279	1597537	2595296	6094507	6111431	1254674	417115	8091	16262	78805	26465	31231	43894	0	316886	34824
EIC	Negative	474.2924	3.69	unknown	1277127	122910	1227360	504535	295152	180547	307045	768340	150369	51377	0	0	0	0	0	8028	0	33778	0
EIC	Negative	615.4297	3.83	unknown	561568	75172	573848	563711	111601	64053	151982	234898	0	133335	0	0	0	0	0	13845	0	0	0
EIC	Negative	473.2883	3.69	unknown	4424508	360707	4158897	1672849	995362	663338	1048869	2695217	507830	141292	0	20460	83997	21410	0	0	8956	102350	34427
fpHILIC	Negative	408.2837	0.61	unknown	58200876	2582746	86094616	14380351	3145856	3927112	2898808	36344900	2235792	2048560	83438	0	271978	0	67535	0	0	955739	188774
EIC	Negative	583.3139	1.54	unknown	3895700	284533	3824392	1178212	954651	308582	832052	1283458	315307	168941	50489	0	0	15826	0	11577	0	0	81184
fpHILIC	Negative	407.2801	0.61	unknown	226533504	8894142	325337344	52012024	12090444	14562818	11115387	139489664	8980296	7518800	378802	215495	1145968	0	199010	247064	59397	3534238	773258
EIC	Negative	430.2637	3.85	unknown	1353652	121866	1157536	655242	176400	159714	203031	581746	56333	210582	0	0	0	0	0	0	0	33500	0
EIC	Negative	389.2699	3.20	unknown	5138077	353718	6687070	3123249	764396	399341	1056668	2815132	281193	773707	20126	0	0	0	0	0	0	133519	27715
EIC	Negative	462.3235	3.25	unknown	385128	32529	543620	66212	33303	41142	66672	280396	58385	15967	0	0	0	0	0	0	0	14624	0
EIC	Negative	503.2406	2.48	unknown	2721022	358811	4553490	4569324	219260	406734	1058949	1050357	227944	27500	0	0	0	0	8978	0	0	82155	0
EIC	Negative	425.2475	3.61	unknown	820488	51192	782365	320494	93571	45610	145585	308540	14315	103637	0	0	0	0	0	0	0	0	19614
EIC	Negative	406.2683	2.21	unknown	3215625	64004	3864538	914327	217443	271719	178327	2400561	123953	279341	0	0	26579	0	0	0	0	104810	0
EIC	Negative	409.2868	2.13	unknown	2251478	838291	2025687	1208151	808706	892162	972661	1455632	529265	237661	0	0	0	0	0	0	0	153428	0
EIC	Negative	452.3112	2.76	unknown	6983811	2348035	9697324	9000881	4074844	2342622	5243187	2846173	1138120	621419	111070	57275	150420	16263	22272	0	0	274403	220679
EIC	Negative	588.4371	3.87	unknown	337463	29896	647805	852114	126036	89791	141635	370124	32194	128439	0	0	0	0	0	0	0	26138	0
EIC	Negative	488.2712	2.48	unknown	1284731	387373	845327	924629	162386	287324	556549	669802	149705	47132	0	0	10746	0	0	0	0	65020	0
EIC	Negative	446.2576	2.74	unknown	1248516	110348	1697988	542072	204540	209785	154171	1207537	232993	0	0	0	48967	0	34629	38368	0	0	0
EIC	Negative	443.2570	2.13	unknown	6881665	1646350	6284159	2613761	1639665	1833253	2135873	3534825	1099882	426641	30354	28280	111582	0	22923	0	0	329874	30615
EIC	Negative	629.3563	1.67	unknown	1191572	215877	715573	419333	292473	216803	363277	482518	115528	0	13544	0	18669	0	7333	31468	7546	0	10735
EIC	Negative	471.2724	3.61	unknown	1505375	71327	1344846	697698	228869	74455	313581	628150	25103	232589	15515	0	0	0	0	0	0	50876	0
EIC	Negative	444.2609	2.14	unknown	1813358	448438	1499731	697797	388783	476625	530201	935531	271444	143952	7669	0	27571	0	0	0	0	105994	0
EIC	Negative	487.2683	2.49	unknown	4356176	1264544	2725001	3192182	535761	819080	1793294	2311735	453403	272701	39957	0	28045	0	0	54437	8168	172430	11950
EIC	Negative	603.3333	2.49	unknown	1586256	320108	1481352	1453052	126274	223476	452867	461152	120956	54744	0	0	0	0	0	0	0	57863	0
EIC	Negative	405.2652	2.21	unknown	12078686	247350	14115378	3532733	812032	985338	644426	8849682	399892	1067818	0	0	33738	0	0	0	0	444442	45178
EIC	Negative	521.3210	1.81	unknown	435332	8902	110683	0	93945	108952	8786	218284	38935	33064	0	0	0	0	0	12204	0	0	7625
EIC	Negative	445.2548	2.13	unknown	2452770	562181	2142274	927671	544157	619590	754638	1294951	399117	184928	21510	0	37372	0	0	27572	0	119692	12467

EIC	Negative	452.3106	3.22	unknown	511707	32858	412860	255059	34993	82955	50245	202706	10596	139273	0	0	0	0	0	0	0	17275	0
EIC	Negative	452.3115	3.85	unknown	12500025	2288886	12071434	9023591	3761123	2913166	4059911	8337524	988900	4220950	62995	150691	119280	0	0	0	0	876856	38218
EIC	Negative	390.2735	3.20	unknown	1331477	107528	1718424	848651	216802	114989	280540	737630	78709	224722	8105	0	6935	0	0	0	0	41088	24092
EIC	Negative	505.2573	2.13	unknown	4517902	1363523	4957453	3025177	2108840	2262137	3279751	4969178	1736904	621617	34920	37477	107804	0	27326	116602	8183	452754	63237
EIC	Negative	449.2907	3.00	unknown	3728261	111259	8828782	2801637	258695	346692	351927	2079708	37209	0	0	0	0	0	0	0	0	0	143990
fpHILIC	Negative	189.0117	0.43	unknown	2317818	1062387	2584827	1095841	367495	152526	1473387	1982875	1164991	0	0	0	0	89892	79930	73205	0	0	88366
EIC	Negative	394.2957	3.85	unknown	825451	158964	821490	619122	232991	215941	322793	600481	78635	302465	0	46725	0	0	0	0	0	42944	25429
EIC	Negative	633.4763	3.86	unknown	378403	18975	616282	1955681	284501	107275	301982	813646	179298	262887	0	0	22338	0	0	0	0	80026	0
EIC	Negative	506.2605	2.14	unknown	1166052	337414	1250285	844740	572227	618273	850437	1393039	475055	190434	0	0	29298	0	0	32470	0	144701	10073
EIC	Negative	469.3078	2.13	unknown	8738159	3191672	8203709	4795084	3045323	3567016	3992949	5548080	2105167	875341	85263	37026	213419	11544	26756	129580	19553	677115	25624
EIC	Negative	389.2697	3.00	unknown	1637571	0	3564997	1137765	128244	140166	72768	859345	41334	0	0	0	0	0	0	0	0	59959	0
fpHILIC	Negative	107.0501	0.43	unknown	2359583	1110629	2184612	856397	166960	145468	1727719	2162534	1109957	0	78425	49621	0	68138	67138	71138	43438	0	76461
EIC	Negative	389.2700	3.28	unknown	2650544	121720	2249488	908816	112136	238699	243500	595523	37387	137413	36238	9188	32876	34040	29223	0	0	0	30890
EIC	Negative	468.3050	2.13	unknown	46385934	16879676	42053915	25044284	16426741	18480050	20803842	31047572	10948082	4743522	420796	262868	841661	84846	228007	678673	109447	3582029	383444
EIC	Negative	467.3017	2.13	unknown	161573266	57965065	148575193	87525683	56998722	63405424	72553886	106614434	38078761	16446815	1482122	956138	2992697	322190	848977	2358111	423833	12262732	1383108
EIC	Negative	407.2806	2.13	unknown	53859988	18767360	47326297	28157673	17835885	20455829	23424661	34817585	12275413	5408037	506948	261210	934243	13357	244804	773056	103504	4056079	428311
EIC	Negative	587.4309	3.87	unknown	730098	101839	1472740	2119626	355392	156043	356114	986427	156842	330569	19984	0	20472	0	0	0	0	88114	15997
EIC	Negative	391.2858	3.22	unknown	841356	45074	886313	466602	85122	97335	89101	360720	33273	208776	11463	0	0	0	0	0	0	35216	0
EIC	Negative	408.2841	2.13	unknown	14258769	4874563	12205035	7391329	4772419	5345361	6089321	9202571	3196412	1392157	136289	92061	224607	29250	76350	219861	31571	1086124	109198
EIC	Negative	569.3340	1.67	unknown	15126073	3719507	12282905	6496923	3808124	2817640	6305168	6092245	1685427	562651	95030	37397	146587	0	28133	273857	7038	790839	60452
EIC	Negative	429.2602	3.85	unknown	5168500	378018	4706647	2612358	735012	525094	790866	2388095	186698	783789	51640	55962	67852	0	0	0	0	155827	106784
EIC	Negative	410.2906	2.30	unknown	302963	84248	347610	154561	124737	89960	99368	252868	48725	41831	0	0	10576	0	0	0	0	37019	0
EIC	Negative	390.2737	3.61	unknown	6382509	276667	5479240	2562767	873187	419281	1061778	2383261	199815	782959	72959	16271	68367	0	0	0	0	166262	131612
EIC	Negative	425.2917	2.30	unknown	227793	44582	277929	153481	94156	70587	66165	191408	46553	53621	0	0	0	0	0	0	0	28363	0
fpHILIC	Negative	189.0034	0.43	unknown	10188801	5060900	10299471	5112439	1181807	574858	7292280	9677816	5084169	274396	305528	376419	165271	361416	463343	348948	207401	73393	360598
EIC	Negative	405.2645	2.94	unknown	2385080	111608	1472340	190503	115784	288599	241392	605046	91566	33357	0	14065	0	0	11389	8645	0	20132	43472
EIC	Negative	464.3023	1.65	unknown	6241626	273197	16244964	2167296	582115	314935	187057	17689399	708136	758081	16979	0	138942	71919	37468	0	54010	1142047	29859
EIC	Negative	428.2648	3.85	unknown	3945084	278904	3465666	1956676	548774	379871	586961	1772820	195710	620678	63966	29953	63985	0	0	20467	0	113315	94234
EIC	Negative	427.2624	3.85	unknown	14998076	1099466	13192025	6955917	2038305	1543807	2183354	6842139	561495	2363138	229840	133541	202749	0	60217	76011	0	437862	304125
EIC	Negative	357.1743	2.68	unknown	1572825	746932	1146318	749466	603536	605602	801240	979839	213071	0	0	0	0	0	0	0	0	0	213828
EIC	Negative	389.2697	3.61	unknown	24042126	1123512	20780898	9782324	3146585	1650262	3967566	8889829	762984	3019598	242612	40872	221646	0	0	109895	0	617329	509802
fpHILIC	Negative	187.0069	0.42	unknown	245826224	124481496	231948112	119652992	21432316	16225096	177414000	244030656	123695200	4917355	5169553	6737707	5398946	10105297	11089251	8124074	5334265	4488520	10331717
EIC	Negative	453.3139	3.85	unknown	2276258	422333	2128907	1590066	618683	491137	733072	1512197	186683	778553	42489	0	0	0	0	0	0	172944	68039
EIC	Negative	449.2416	3.85	unknown	670008	154315	524469	607507	252180	181256	224563	561197	97068	310090	48564	14954	15292	0	0	0	0	69666	21470
fpHILIC	Negative	188.0105	0.43	unknown	18924908	9386558	16923106	8388057	1453195	1430539	12819858	17217400	8911777	321153	491645	504116	406920	678208	656664	684274	382148	396945	777022
EIC	Negative	360.1931	3.02	unknown	13333759	6250074	13579536	6409525	5921184	4719570	7157975	10003009	2041855	104081	47463	56014	105696	40447	40402	53858	0	15290	2021295
EIC	Negative	359.1901	3.02	unknown	64168303	29916887	64516997	30791673	28174638	22673059	34148850	47791510	9634197	482435	165989	192022	442292	192007	133265	114740	162222	211461	9584873
EIC	Negative	467.3021	2.52	unknown	14290631	930929	14836712	3507963	1202172	1493778	1232534	6249345	758729	1001445	0	163105	0	0	0	160990	0	625592	0
EIC	Negative	444.2608	2.30	unknown	3011075	384357	3008276	1262836	599853	438742	437345	1992564	285026	520008	32142	22936	59737	0	10440	0	0	187621	75129
EIC	Negative	361.1888	3.27	unknown	3661187	0	629431	394041	1292204	0	2000348	1038301	100545	323384	126470	0	118014	259418	0	0	109008	219180	0
EIC	Negative	385.1327	3.33	unknown	231800	119607	339503	310283	50128	64653	77101	180961	75353	0	0	0	0	0	0	0	0	0	34215
EIC	Negative	361.1891	3.02	unknown	4109345	1913386	4328612	2052387	1785370	1492459	2192094	3122757	617235	84542	0	13509	37048	30842	47765	9642	30341	22002	598689
EIC	Negative	445.2547	2.30	unknown	3856037	452995	3963417	1650179	795088	564674	578181	2618639	383419	686740	41186	26331	102967	0	0	0	0	291354	93486
EIC	Negative	443.2570	2.30	unknown	10793837	1368687	11314085	4733263	2256162	1592265	1745368	7455653	1116876	1831052	87896	73336	242191	27097	45527	49753	20244	757610	233387
EIC	Negative	478.3183	2.36	unknown	1301318	30763	3095141	85400	57288	61670	55183	2447401	119036	236410	0	0	12045	0	0	9864	0	222429	0
EIC	Negative	359.1905	2.78	unknown	8661351	0	11551195	3702420	4852799	3811523	5161948	4211735	1413923	0	0	0	0	0	0	0	0	0	1683709
EIC	Negative	360.1936	2.79	unknown	1865485	992451	2298942	844367	896776	772293	992331	986192	264225	0	0	0	0	0	0	0	0	0	344437
EIC	Negative	427.2626	3.05	unknown	812763	83091	893260	500831	87721	67412	99192	265597	0	274607	0	0	0	0	0	0	0	37381	8760
EIC	Negative	453.3131	3.05	unknown	436415	44415	509256	280050	73326	38143	72510	154820	10955	143377	0	0	0	0	0	0	0	31466	0
EIC	Negative	452.3111	3.05	unknown	2375387	233071	2926857	1588443	293338	183100	392321	927234	130650	823033	39912	0	7748	0	0	0	0	127165	46315
EIC	Negative	392.2891	3.85	unknown	47216682	8424179	44185560	33078257	13711111	10605208	14604074	32284433	4222137	15844051	1558705	915829	1366051	50336	253144	495213	215638	3543068	2373898
EIC	Negative	393.2925	3.85	unknown	7671077	1287537	7003386	5078497	2237718	1679359	2287313	5184662	653230	2467640	253652	154234	198463	0	48482	66627	17465	616981	379501
EIC	Negative	393.2921	3.05	unknown	871488	85679	1043550	553287	99381	63756	114496	286498	29043	309104	0	0	0	0	0	0	0	56371	0
EIC	Negative	362.1979	3.28	unknown	893938	0	1074566	0	45023	0	486393	0	0	0	0	0	0	15203	0	0	50660	0	0
EIC	Negative	613.3605	2.07	unknown	341889	115419	392996	358076	154404	85160	206847	202571	25370	0	0	0	0	0	30208	34500	0	0	0
EIC	Negative	360.1929	3.13	unknown	3253954	1542248	3860778	1334662	1316190	1099745	1428320	1743470	534349	54209	0	13919	12206	33504	0	0	60461	0	539398
fpHILIC	Negative	360.1933	0.47	unknown	2361033	992262	3164064	1181473	781332	661747	967299	1811025	347153	0	0	0	0	0	0	61982	0	86812	298993
EIC	Negative	489.2634	3.85	unknown	1959472	380015	2288261	2147078	964886	743329	1120178	2621680	313517	1207380	113476	108120	115356	0	17637	50481	17341	308512	179188
EIC	Negative	451.3070	3.06	unknown	8332017	835548	10133527	5449888	981726	668610	1319607	3201907	411129	2856285	122000	11974	38065	0	33870	36333	12189	451351	122466
EIC	Negative	381.1715	3.02	unknown	2084870	1160553	1852495	1143800	982869	841798	1267053	1561523	386374	19182	8048	47433	31201	57598	40328	27882	37404	0	397299
EIC	Negative	391.2858	3.10	unknown	5351426	354099	4992869	1875751	627288	665893	647111	2500922	193944	660067	82822	33003	121796	0	32220	43154	18894	158042	210650
EIC	Negative	469.3077	2.30	unknown	11319739	2471594	12342138	6941904	3942895	2862760	2861544	9214395	1852770	3084577	162440	170362	484490	83013	92265	74135	58155	1521176	364250
EIC	Negative	408.2842	2.30	unknown	16340037	3454244	17109952	9579790	5509235	4072173	4125529	13551150	2690516	4444060	271182	267592	598668	103713	59151	109859	28616	2183089	670199
EIC	Negative	321.2062	2.32	unknown	483683	124757	377840	257326	27764	142908	132651	329604	40399	28344	11481	11643	9630	11951	10302	7924	10504	26541	13783
EIC	Negative	468.3050	2.30	unknown	60740448	12597468	64752523	37005098	20757086	14842607	15081043	49704888	9795307	16417128	864665	801178	2034661	370515	465292	364303	284356	8249807	1953699
EIC	Negative	467.3017	2.30	unknown	208487946	44338106	223363166	127250812	72014771	51243668	52387919	170010501	33455789	56322163	2892739	2799644	6978367	1288390	1618109	1294550	1020156	28300057	6763119
EIC	Negative	359.1901	3.13	unknown	15994823	7272372	18410629	6341100	6315422	5244894	6697033	8306772	2531255	264345	79839	106257	142469	152100	93769	40983	84031	138836	2680381
EIC	Negative	502.2932	2.83	unknown	1522469	0	1266418	1570754	55603	57472	119244	978069	86914	314990	18004	16799	41109	0	0	0	0	119093	16775
EIC	Negative	487.2733	3.85	unknown	3173794	1194603	2928659	3178552	1977761	1493468	1897453	3284140	701571	2041809	254880	135015	215951	0	22903	79331	0	517999	377070
EIC	Negative	407.2806	2.30	unknown	62397462	12928736	65512174	36574111	21084026	15381167	15782065	50669976	10041393	16242897	1079308	1007035	2262613	377451	536662	450681	318721	8163231	2605308
EIC	Negative	391.2858	3.06	unknown	21188485	2034435	25406317	13028950	2429455	1477900	3102433	7685918	1010811	7489852	323837	33003	52407	0	82871	105706	18894	1209148	339829
fpHILIC	Negative	359.1891	0.47	unknown	11544434	4215765	13727558	5925507	3878121	3311927	4572734	8017721	1582512	211838	92393	0	139246	181590	142063	234421	47722	420461	1586956
EIC	Negative	469.3076	6.01	unknown	1092063	958501	0	0	0	0	0	201020	0	0	0	0	0	0	0	0	23104	0	0
EIC	Negative	392.2891	3.05	unknown	5603293	533483	6562187	3548723	653915	395248	784123	2053189	250945	1954133	92181	0	21900	0	27374	31972	0	324359	99966
EIC	Negative	468.3051	2.52	unknown	3767643	231861	4026439	911448	329187	420401	313451	1737580	211412	212542	48247	43532	60620	48355	51438	43327	39422	155730	63660
EIC	Negative	498.2902	2.23	unknown	22644011	1681207	47493276	35978175	1719201	1103068	934669	28050081	1695576	19092160	598811	216585	351963	333276	402761	311048	393920	1935263	914361
EIC	Negative	487.2731	2.50	unknown	317082	0	348714	3006353	0	0	0	1960653	28774	219158	26572	16988	38866	18844	0	30269	0	171882	34503
EIC	Negative	319.2220	4.55	unknown	0	685456	254820	0	0	730631	919796	0	0	605444	107724	0	0	0	0	0	0	0	197020
EIC	Negative	501.2930	2.83	unknown	9722766	0	7455324	9582116	43995	409772	612606	5850767	547082	2137478	168178	87048	197356	27376	40538	41771	29211	737915	45307

EIC	Negative	499.2933	2.78	unknown	147629554	38694	112910139	144732587	40699	88854	74338	91229074	196091	29397292	57102	69820	103706	152787	98364	20047	69112	10830334	55997
EIC	Negative	634.4336	2.31	unknown	1356868	29852	897079	529842	54078	98403	54225	472724	38030	132480	0	0	0	0	0	0	0	86757	0
EIC	Negative	505.2574	2.30	unknown	5426199	507545	7060413	4693724	1363491	951315	1099755	8160051	766879	2572212	62445	74549	155472	33652	29596	40310	27997	1232045	172968
EIC	Negative	409.2868	2.30	unknown	2649951	543956	2862269	1578440	938048	662307	667211	2120727	438460	914683	56747	40091	90409	11490	25150	12593	7469	405792	139539
EIC	Negative	478.3182	1.70	unknown	361461	107967	245227	52245	114216	103991	78712	332418	70638	69443	0	0	0	0	0	0	0	67363	25670
EIC	Negative	485.2577	3.12	unknown	466270	10773	405664	308891	39810	0	23662	332818	0	558761	0	0	0	0	0	0	0	53683	0
EIC	Negative	506.2599	2.29	unknown	1554548	138724	1906043	1250131	343434	266975	277944	2169404	213859	726749	22812	14127	45451	43400	43431	0	38393	325564	50766
EIC	Negative	446.2576	2.15	unknown	578650	145904	681524	253363	208257	213772	37495	310159	63401	32040	23984	32324	29461	22906	15467	36676	10067	43240	12171
EIC	Negative	500.2912	2.83	unknown	46306892	0	35033798	45972172	0	0	2680315	28742867	2513794	9542520	887883	415053	0	129126	214926	225196	0	3403042	1046190
EIC	Negative	413.1952	4.15	unknown	113500	0	823245	0	283038	256218	0	0	329213	466459	0	28164	0	33791	0	129408	0	0	0
EIC	Negative	473.2884	3.85	unknown	4528160	2263328	4024433	4324164	3338120	2838557	3327449	4286427	1625346	3646960	545328	336491	464365	0	112206	192321	105842	1065293	847797
EIC	Negative	446.2584	2.30	unknown	902312	102865	990983	392771	215693	163891	163659	732537	113894	211416	29483	28359	45033	23161	28909	31005	24148	100185	36196
EIC	Negative	499.2933	2.23	unknown	6695365	521097	13884617	10627995	539972	347044	300914	8484642	510053	5579617	174779	62625	106513	139787	129474	90631	77847	1079884	272489
fpHILIC	Negative	172.9913	0.47	unknown	75228528	6266413	8492568	1410112	4105731	54134684	2855738	5140785	4616784	1490997	1070834	2680031	2907292	1518505	1222298	4685797	1600545	1766243	2259279
EIC	Negative	359.1901	3.33	unknown	8400778	3681088	10570663	2818109	2600905	2285329	2368902	3811578	729305	357292	141185	133711	120082	147536	74388	47986	62496	199390	1585311
EIC	Negative	436.3143	5.17	unknown	2180382	149681	2065843	1000704	300566	135783	199676	897603	90751	760856	53567	0	28920	0	0	0	0	181415	108010
EIC	Negative	502.3990	5.80	unknown	0	94892	70723	759447	223435	0	137713	136154	326197	95905	23650	101996	126879	0	0	0	110516	38233	0
EIC	Negative	474.2631	2.88	unknown	0	0	0	1232186	0	0	0	0	429816	0	0	0	0	0	48347	29766	0	0	0
EIC	Negative	319.1535	3.13	unknown	88882	133147	51563	417124	0	0	0	363856	79450	56488	29695	227237	161730	0	44602	0	0	0	36100
EIC	Negative	407.2442	1.89	unknown	0	104422	0	0	33827	845313	19336	0	20492	0	2372924	706658	21500	0	61373	0	84075	0	24254
EIC	Negative	376.2939	5.17	unknown	994644	54676	861711	447254	135724	58626	61648	385260	0	313565	30053	31048	0	0	0	0	0	79303	39268
fpHILIC	Negative	171.0564	0.95	unknown	1368533	251960	981431	832684	737647	879303	652596	1229190	443821	0	48554	128970	95401	112093	282096	146244	110560	43615	148111
fpHILIC	Positive	251.0481	0.95	unknown	1687993	209792	1107172	1122825	775978	1007360	724184	1115601	431310	0	53022	141042	59940	74883	300859	117044	150991	50980	134763
EIC	Negative	507.2612	2.30	unknown	198962	0	313854	240987	81266	64512	17863	358349	38914	146854	23341	7815	0	15819	0	15417	19991	69655	0
EIC	Negative	389.2696	3.12	unknown	7642161	266298	7568494	5090620	496813	377332	405644	4483526	189523	9549560	228261	0	181257	0	0	0	0	962729	210662
EIC	Negative	449.2899	3.12	unknown	10040036	307819	10380785	6650140	623710	423085	650500	5852950	252327	12869751	322009	99675	222919	0	0	0	0	1262525	288233
EIC	Negative	390.2733	3.12	unknown	2038765	87959	2044388	1351812	137661	109887	124725	1151117	53504	2528086	80712	31671	61407	0	0	0	0	256069	66894
EIC	Negative	450.2942	3.12	unknown	2911634	107256	3037188	1887566	194793	118417	155034	1707678	73061	3798522	84406	17411	69440	0	0	0	0	367613	98320
EIC	Negative	375.1848	2.77	unknown	1693507	218730	1023196	1065422	0	0	0	1738526	0	3483853	0	4103770	185389	379106	0	0	0	375289	0
EIC	Negative	480.2782	3.61	unknown	2768038	54835	1503176	649435	53177	43840	62667	1190946	52178	640077	0	0	0	0	0	0	0	306129	0
EIC	Negative	477.2820	3.02	unknown	356812	0	275110	154853	0	91056	180547	24111	131825	203177	0	421634	307489	0	0	0	0	94112	0
EIC	Negative	596.5278	6.36	unknown	328841	279959	209205	780922	289151	339928	257498	132807	195859	138917	136144	156640	97555	0	0	96004	0	172145	0
EIC	Negative	339.2654	5.50	unknown	0	629665	1545002	1325282	681822	569683	0	1009763	506936	0	0	0	0	0	0	619196	0	0	0
EIC	Negative	362.1883	3.03	unknown	0	339524	0	392115	0	262698	0	134918	111621	0	0	0	0	0	0	0	0	0	120263
EIC	Negative	406.2678	5.20	unknown	435728	0	0	363485	0	0	411138	0	0	0	0	0	0	0	0	0	0	0	98277
EIC	Negative	630.3781	4.79	unknown	639648	1018689	558227	111890	0	147083	792287	629321	1002253	155844	807935	628749	0	686068	0	627148	0	0	0
EIC	Negative	462.3162	3.06	unknown	95456	14896	341460	375792	42346	82779	30957	106649	20539	105750	26096	7760	0	0	0	0	0	69285	0
EIC	Negative	472.2765	3.12	unknown	286645	0	276925	214827	14880	31707	15473	190792	0	402680	0	0	0	0	0	0	0	62280	0
EIC	Negative	390.2735	3.28	unknown	715771	66014	627828	243622	60055	77344	84079	176438	33481	52072	23470	12683	30227	25376	25995	17326	14134	15353	35444
EIC	Negative	452.3821	5.79	unknown	51160	245979	518849	2658440	958335	41234	736133	524839	692610	257555	94956	598124	244524	25543	0	50429	243300	394046	72260
fpHILIC	Positive	166.0718	2.68	unknown	126575	400910	432213	600592	395985	386184	367049	456886	334369	210727	252942	523694	356747	224614	210403	62133	149690	0	78720
EIC	Negative	448.3512	4.91	unknown	16814	15779	114494	675857	150361	0	101956	126784	159853	60552	0	79768	52538	0	0	14490	17137	108935	0
EIC	Negative	333.2074	6.06	unknown	0	0	4958356	7641276	4458263	2433231	3786455	4673007	0	3397865	2359798	2923676	3626416	182485	1916841	0	140040	0	2056184
EIC	Negative	553.4480	5.81	unknown	169720	324955	390350	935760	493827	323252	708730	409082	377637	227424	194327	204151	223078	125466	87115	68750	243270	140225	81925
EIC	Negative	381.1717	3.35	unknown	473104	200123	597460	188127	149110	129140	132074	236095	66220	0	0	15947	17200	10723	12555	26722	24959	23053	107076
EIC	Negative	481.2810	3.61	unknown	847585	11657	482894	204628	15558	0	22099	356733	14062	193901	0	0	0	0	0	0	0	111944	0
EIC	Negative	446.2576	2.19	unknown	499299	36024	636761	170125	51486	41994	41765	340904	46044	48340	23984	32324	21154	24680	16336	36676	21756	43240	26624
EIC	Negative	524.2948	3.29	unknown	401502	0	349012	385209	41101	45791	60693	303008	296008	331819	43397	37547	30240	346783	35067	39006	47241	46088	42757
EIC	Negative	483.2979	3.75	unknown	2391076	7916	2133441	1275484	79911	58667	58008	1270067	71439	881318	28235	16887	19720	0	12448	12919	0	372136	35505
EIC	Negative	471.2731	3.12	unknown	1065057	26473	978399	772873	0	0	12932	703179	16431	1422058	54747	8987	49669	0	0	0	0	167024	44049
EIC	Negative	473.2827	3.06	unknown	158919	238297	1857170	182630	265314	180561	72829	217477	72783	154572	51909	0	0	0	0	14573	0	172971	45864
EIC	Negative	482.2943	3.76	unknown	7987796	127091	6926436	4149599	234670	174594	169493	4151586	202614	2881332	86465	63090	58590	45851	63716	59662	49269	1196636	68546
EIC	Negative	365.2463	6.39	unknown	0	7030993	19074970	14292722	0	9818884	5439546	0	0	0	9545785	7934966	0	0	0	0	0	0	4646216
EIC	Negative	500.2289	0.71	unknown	165060	38112	69742	89829	20465	123501	38919	20972	21694	58505	23693	18976	12937	23854	5732	0	0	20182	44549
fpHILIC	Positive	502.1553	2.42	unknown	782077	193401	804178	146622	532983	421670	241512	61466	396040	158690	0	0	590641	482924	0	0	524987	0	0
EIC	Negative	473.3632	5.79	unknown	210993	485622	934705	2892004	1400734	401854	1133490	881672	1068289	546045	190184	1091120	473015	75910	28184	361391	417997	631018	143089
EIC	Negative	474.3638	5.79	unknown	64308	157048	395927	924845	413537	131465	426530	339249	363115	176432	58371	361648	206504	22670	0	148657	137501	203762	54543
EIC	Negative	510.2534	1.12	unknown	1271100	0	2146949	932350	0	0	0	1497770	0	0	0	0	0	0	0	0	0	488473	0
EIC	Negative	447.3487	4.91	unknown	70652	108385	272921	2219914	527892	93598	313087	323386	484836	152779	53670	214343	159714	18948	0	90464	117326	339556	57182
EIC	Negative	437.2909	5.73	unknown	1128638	1352930	1102169	0	0	1616218	0	0	1256971	0	0	0	1037660	1203380	0	0	0	998075	0
EIC	Negative	505.3152	5.65	unknown	230623	347009	289322	239803	342974	595896	286444	161125	0	96683	262711	285678	97967	149963	74147	156315	80731	104645	67636
EIC	Negative	468.3051	5.90	unknown	0	132848	99310	53824	54232	48750	55060	23219	111118	14998	48388	0	56057	99094	0	39776	0	42140	21339
EIC	Negative	461.3133	3.06	unknown	592152	77003	1098924	1364226	164033	276592	108240	239775	74366	425784	80862	55766	0	0	20980	0	11481	231182	42428
EIC	Negative	470.3330	4.91	unknown	19221	0	86516	415515	133465	22563	114542	107832	133143	67299	27112	65201	70746	0	0	25130	50353	108080	0
EIC	Negative	490.2872	4.73	unknown	0	196454	157680	0	0	172255	148812	0	0	0	0	0	0	0	0	0	0	115878	0
EIC	Negative	335.0995	3.02	unknown	374577	66671	246140	106121	49867	54356	19580	104913	0	141712	27136	32723	33533	27058	14683	0	0	54218	13681
EIC	Negative	484.2957	3.75	unknown	731294	18383	653559	391380	36294	38673	42131	378290	43453	291089	31029	0	29477	12389	29526	19163	18294	115176	13455
EIC	Negative	394.1779	1.27	unknown	212739	143855	225329	119437	103465	89311	148557	47706	84773	88107	58483	16496	6422	0	103404	68818	0	0	0
EIC	Negative	510.2534	0.98	unknown	212020	0	403286	184143	0	0	0	284293	0	0	0	0	0	0	0	0	0	103455	0
EIC	Negative	393.1740	1.27	unknown	873948	568933	905628	430378	438472	357931	672612	172800	336800	415938	253981	65626	52602	0	405138	279532	16321	0	25309
EIC	Negative	454.2847	1.71	unknown	0	0	541527	0	0	0	0	0	651960	157785	0	0	0	126086	0	0	0	242937	0
EIC	Negative	593.4784	6.18	unknown	273807	546829	833302	2146831	895320	547893	850653	1029363	1267709	425644	448035	327040	767714	260464	229558	344536	537208	399183	205368
EIC	Negative	581.4803	6.35	unknown	1084446	1080835	975211	1343536	487283	633332	783581	975934	987561	665023	445215	280454	457680	459739	414041	0	375013	371691	288522
EIC	Negative	291.1222	2.05	unknown	0	233580	264149	0	23650	37273	41845	0	185071	0	197830	202698	0	0	20913	39702	25019	0	22298
EIC	Negative	502.2845	5.44	unknown	0	408888	389380	0	381355	0	418637	0	0	0	260267	0	209755	0	0	0	302837	0	0
EIC	Negative	357.1737	2.83	unknown	298716	248254	382416	186933	141989	187899	217468	178252	77276	0	36001	0	20868	0	18298	0	48838	32165	205707
EIC	Negative	319.2218	3.35	unknown	98299	126063	4990464	698083	92036	465684	193274	190262	316404	0	96220	78121	1346215	1752554	37211	103253	136244	100517	104250
EIC	Negative	594.4833	6.17	unknown	92974	330979	375848	899196	335511	249488	360886	329454	441119	214343	252315	0	305368	72449	0	145818	271470	249936	0
EIC	Negative	493.3533	5.44	unknown	600120	144338	1174883	447030	429743	0	227362	776952	240351	468367	174256	0	146547	0	0	0	0	660621	0

EIC	Negative	269.1576	5.06	unknown	294481	184392	224820	242858	163597	293678	173268	242195	109702	123127	82490	0	0	0	32039	131579	0	224480	0
EIC	Negative	323.2502	5.67	unknown	0	119812	163790	143779	0	119787	102155	114061	0	93780	0	0	0	0	0	0	0	136354	0
EIC	Negative	481.2096	1.78	unknown	314875	23163	342453	181959	64417	85513	75654	362402	26331	234651	49977	96113	37840	90815	105146	48628	73961	0	21596
EIC	Negative	351.1806	2.26	unknown	587086	83895	222995	112072	127279	142206	174076	450506	45204	285230	56821	74915	52619	47578	40482	54618	70248	126873	95396
EIC	Negative	403.3227	4.01	unknown	961736	0	507142	79321	0	0	0	208782	0	137191	0	0	0	0	0	0	0	86594	0
EIC	Negative	341.2698	6.42	unknown	2007707	1940136	2802397	1777000	1286560	1085571	1023786	1308046	1433558	928944	700937	879620	1104310	498865	684602	455541	889608	295056	258196
EIC	Negative	245.1579	4.23	unknown	118528	0	0	349505	66894	81345	87465	96413	0	156011	0	0	0	0	0	0	0	141218	0
EIC	Negative	601.4479	5.58	unknown	107558	166217	183483	1289648	141868	94174	272788	321144	398458	229834	184458	103593	235314	105199	145575	0	236128	97439	43562
EIC	Negative	641.4795	6.39	unknown	983529	1334280	1518898	2048167	1527632	1132740	1277345	1797434	923589	855694	529503	534626	739967	312113	404461	553276	536470	821630	540688
EIC	Negative	239.0672	2.98	unknown	479189	251731	106777	283082	255634	247924	145228	131319	377816	174528	244274	110241	109086	147125	112442	127577	86174	81776	90159
EIC	Negative	469.3308	4.91	unknown	91055	106390	252396	1558558	450487	111417	295524	291988	396132	172065	24616	227353	167641	0	14226	103148	123168	385674	39974
EIC	Negative	342.2016	3.13	unknown	586305	22524	426544	59958	26056	0	0	321701	0	298304	0	120706	39204	96744	49804	0	53262	46969	0
EIC	Negative	642.4849	6.39	unknown	449143	607669	679401	1389645	673334	374703	780229	780328	376707	447080	300476	214865	527138	117136	192466	308673	266007	340317	202416
EIC	Negative	227.1909	5.79	unknown	373547	591131	834332	2214817	1176627	478532	1010148	909816	1009683	612248	367888	904212	588950	0	0	489548	555464	684345	290639
EIC	Negative	605.4798	6.17	unknown	1294998	2257296	2643172	6107035	2121613	1961583	2916430	2779970	2674634	1305204	1351055	1087730	1882871	1151237	1148406	718573	1686096	1208912	750997
EIC	Negative	469.3283	4.91	unknown	91055	114783	264006	1558558	450209	106093	303601	268449	210672	155588	52705	237795	192088	20916	14226	102236	145448	291001	51093
EIC	Negative	471.2729	3.44	unknown	228290	39876	230152	216307	56953	61113	91393	117175	32605	104430	25535	63569	69814	0	0	0	49310	114613	0
EIC	Negative	226.1891	5.94	unknown	142248	249910	474285	1504952	622940	254387	465540	438375	576920	268415	158256	479659	295518	95866	65339	215164	272024	395869	128882
EIC	Negative	298.2469	6.42	unknown	1700483	1071276	1828017	5094482	1449870	1080719	1410817	2353891	2382319	1572077	1011734	1512688	1101617	342882	301860	1180671	722127	1309063	463828
EIC	Negative	482.2797	3.60	unknown	259124	24258	159611	78848	11915	14243	13841	117595	0	72715	20715	9114	20931	21937	9391	10196	9575	43431	0
EIC	Negative	226.1891	5.79	unknown	3059192	5075105	7488186	19783076	10555027	4543795	9300046	7745911	9065627	5249470	2957636	8224434	5160415	1596821	1467054	3978999	4619154	6470186	2481009
fpHILIC	Positive	345.1765	2.25	unknown	3549333	1463595	2844755	4001458	2090220	2429479	2204358	2943961	2498146	2338248	1428711	1684702	1940109	758111	797062	1207575	963343	2157793	489617
EIC	Negative	225.1857	5.79	unknown	12054969	19949845	30048293	80136298	42479812	18074450	36654392	30994634	35674628	21096274	12106214	32902549	20539649	6463513	5783072	15837768	18354927	25893450	9854983
EIC	Negative	354.0363	2.09	unknown	261539	311787	110778	57761	229400	212345	182890	156981	163911	82110	169111	16059	85440	117370	137258	159726	37002	0	107467
EIC	Negative	551.3218	2.65	unknown	339971	193515	126683	49634	157269	186492	133626	248028	43214	68993	85185	81660	96222	0	83353	83880	86937	0	105868
EIC	Negative	225.1857	5.94	unknown	582398	984327	1875498	6036464	2610946	1012962	1864822	1802455	2217190	1154167	648187	1957913	1135144	377372	285885	872949	1064085	1706340	551097
EIC	Negative	225.1759	4.91	unknown	91849	95997	171355	525718	251626	102931	176851	204768	239687	123669	75061	165635	139980	63538	21434	93583	110502	204227	30102
fpHILIC	Negative	363.0737	3.29	unknown	0	1882307	1528286	669310	489902	1107283	278070	277061	534990	974738	776005	381758	344186	166711	261685	871757	0	135239	0
EIC	Negative	307.1893	5.94	unknown	181431	279765	550711	1601777	751235	298061	548732	527243	681096	345517	212903	589736	348861	106790	87387	260564	347616	479809	153245
EIC	Negative	606.4823	6.17	unknown	529997	844187	1159566	2346321	871505	786578	1098510	1094893	1018512	487824	526437	455921	732314	542461	465120	258260	764726	522843	299353
EIC	Negative	608.3946	5.74	unknown	0	2638735	2498923	1875178	0	3141606	2524691	2224656	0	1585312	0	0	0	1668956	0	0	0	1522575	2228265
EIC	Negative	500.3824	5.58	unknown	0	74006	120675	784881	126172	0	49638	174041	397097	89387	22731	74298	117220	0	0	101262	68144	155173	30785
EIC	Negative	297.2435	6.42	unknown	7357680	4455338	9124433	24791343	7649814	5655634	6603349	11832435	11290473	7357959	4908749	7498601	5997666	1725214	1886025	5947397	3545082	6785101	2345326
EIC	Negative	503.2994	4.64	unknown	0	1608051	0	0	0	1539660	0	0	1253375	964298	1227370	1151541	1116240	0	0	0	0	0	1342761
EIC	Negative	431.3379	3.38	unknown	0	64556	195955	1745347	216708	99298	179911	197795	351662	160623	69523	160553	131631	25766	12372	65459	84227	324134	16406
EIC	Negative	324.0088	1.26	unknown	64225	50675	68490	37708	47200	48934	36545	62805	33836	53413	21553	16914	21547	28311	25944	42043	17354	0	25202
fpHILIC	Positive	346.1796	2.25	unknown	487044	325465	734361	607928	293447	331714	248214	419428	432537	441066	180656	387237	330270	138676	223309	122972	78718	354723	62011
EIC	Negative	361.1844	3.00	unknown	0	1928452	4186348	1971698	0	1480467	0	0	0	0	0	15750	13140	12646	61285	48390	0	13726	600186
EIC	Negative	591.3199	1.24	unknown	882388	30917	1882510	329701	37921	141535	77075	692082	65000	1807703	56141	41090	65678	0	114099	70119	0	192437	118788
EIC	Negative	514.2855	0.99	unknown	827221	25418	3973898	4134008	56880	26398	0	4591785	104083	2736873	128528	0	0	0	0	0	0	2279071	68781
EIC	Negative	434.3364	5.44	unknown	230035	46868	451385	143310	150448	56453	29893	292562	38539	149944	0	0	0	35640	0	0	45149	199778	34573
fpHILIC	Negative	175.0246	3.29	unknown	48492	46060900	28378534	12526597	15011176	30954256	8771110	5998194	12093366	17203620	21932962	9510860	11548569	3712577	6604527	26450484	191305	2411713	279011
EIC	Negative	314.2058	3.25	unknown	195623	60077	195075	76469	99783	94270	111159	243450	25127	146822	56507	41583	43911	38327	36670	27084	38205	111376	71081
EIC	Negative	455.4106	6.35	unknown	1906962	2689038	5774895	7195745	4266803	2853962	3673893	6288514	4908992	2492294	1946622	4363194	2684664	1160619	1507694	1908808	1896643	3817885	1773212
EIC	Negative	360.1934	3.65	unknown	1598401	0	1576982	1541572	0	0	1717048	1619934	0	0	0	0	0	0	0	0	0	0	1658272
EIC	Negative	604.3628	4.52	unknown	14242704	358649	13601586	14049583	13489967	13888372	14142702	0	13667018	13044895	13668725	0	0	13376744	0	0	13534026	0	13950299
EIC	Negative	309.1972	5.79	unknown	88587	103437	133735	332788	189079	98631	176167	146955	163426	130137	0	173062	99520	0	23686	93899	94872	112683	61633
fpHILIC	Negative	176.0281	3.30	unknown	0	3411375	1817171	874954	1062814	2256959	585537	421360	857391	1205877	1547080	677466	820245	284790	505144	1921866	0	210662	0
EIC	Negative	450.3313	2.87	unknown	1808221	119067	1419863	414111	282681	44507	309716	2846413	272262	489340	40025	91121	78694	185301	145242	97371	117973	1466158	91118
EIC	Negative	449.3279	2.87	unknown	6184458	443470	4700424	1306997	942393	117953	1115909	9472380	963228	1806695	151452	331643	232723	643531	505695	364959	397634	4853612	340457
fpHILIC	Negative	174.0169	3.29	unknown	0	4672963	3900302	2429738	2532200	4150892	1784201	1287474	2161281	2841997	3273373	1921075	2085565	878590	1433496	3892412	0	725619	54304
EIC	Negative	320.1612	4.91	unknown	93543	96090	145540	323581	189346	102350	157223	158374	188153	109915	72799	122387	118647	26513	25837	96568	85978	144681	55648
EIC	Negative	570.3424	2.38	unknown	2961653	1867378	2478275	2970014	1545272	1823623	1629350	2338913	1560647	2415222	1822304	1924649	179707	1324462	1643549	175863	185344	2385206	208302
EIC	Negative	483.3697	6.29	unknown	307323	157483	441613	294021	317776	89637	167405	158510	322680	232696	126784	155529	123606	89257	99990	171991	101321	113087	60166
EIC	Negative	353.0331	2.09	unknown	1598648	1744286	763808	384351	1268439	1249827	1125323	1041694	1074528	603642	1020121	63640	452364	621803	781828	972701	201674	400997	629436
EIC	Negative	450.3315	4.14	unknown	632445	0	439963	187353	46781	0	0	254500	38447	216840	0	0	0	0	0	0	0	176476	0
EIC	Negative	504.4135	6.06	unknown	81377	164823	419942	1403888	390309	125828	263186	368976	564610	190940	102550	148649	235517	72004	79051	101890	181164	438102	90325
fpHILIC	Negative	535.4731	0.38	unknown	936445	1538268	4560562	4828610	2029125	1374594	2248609	2875323	5285408	1235084	845547	1701612	2276489	734300	825284	1189039	1904173	2176021	1107224
EIC	Negative	292.1674	2.85	unknown	326981	373122	492831	353485	189804	233145	220289	290095	120700	350798	210926	176522	87690	46647	59031	122563	144544	224916	0
EIC	Negative	313.2025	3.25	unknown	1039131	304085	1051867	417496	504288	465678	580816	1250568	124491	729176	260780	223571	192102	181202	193996	128815	216585	658380	331987
EIC	Negative	263.1653	4.11	unknown	102174	66045	107996	162918	76447	69648	68333	99254	82022	66838	85556	45722	52725	15936	42274	35847	37072	53028	38841
EIC	Negative	313.2025	3.19	unknown	621524	157757	630643	258064	261238	250638	359995	836332	73682	496103	143324	109551	123943	85910	111554	72481	100956	437294	212275
EIC	Negative	378.1833	2.33	unknown	1323966	1052057	1265475	846393	742183	743032	1395999	894190	693011	466841	460200	132234	99810	0	830285	663091	62615	727543	66695
EIC	Negative	307.1893	5.79	unknown	3352343	5011576	7017569	13897845	8763551	4446302	7852085	7198028	7573726	5384165	3365444	7696053	5445853	1796872	1557933	4226674	4717192	5718706	2631864
fpHILIC	Negative	228.1320	0.45	unknown	791489	713127	534305	169635	646905	1140929	543834	129096	122784	379336	323054	350900	286316	241797	483374	106174	271733	88954	410522
EIC	Negative	381.2111	2.87	unknown	1350852	693384	1228985	2490419	225704	364921	430624	443861	238518	588488	295785	106828	95322	0	234529	287931	15957	340087	19173
fpHILIC	Negative	380.2808	0.42	unknown	1241908	583740	326747	1108020	371612	633668	635754	580736	443815	557270	526691	235507	430361	616776	551966	105113	414505	209080	128154
EIC	Negative	508.4453	6.42	unknown	1235636	1866042	3199967	6745840	3090872	1754794	2441133	3678815	3826868	1303466	1483912	2219380	1812077	856520	577557	1611223	2458812	2404476	770401
EIC	Negative	341.2334	3.62	unknown	1852854	615546	2065501	1183776	954496	1102113	1259086	1995836	306314	1253086	582128	776060	517063	280026	443938	455030	333598	1258889	503117
EIC	Negative	308.1928	5.79	unknown	603541	919683	1252840	2530640	1549597	814129	1396159	1276047	1391162	942666	561898	1329893	878494	338287	271439	770369	830740	1061127	505107
EIC	Negative	242.1841	4.46	unknown	230948	61924	273660	504460	203069	208932	160319	318751	202639	201300	85524	222389	230559	42283	49711	134708	88154	279655	34222
EIC	Negative	362.1885	3.12	unknown	0	252784	0	1764894	159376	144834	0	0	0	0	0	0	0	0	0	0	0	0	163164
EIC	Negative	379.1778	2.33	unknown	331263	304359	347496	150902	223524	220090	408474	268184	202966	140661	133241	37597	30565	11291	243007	213024	11673	243565	0
fpHILIC	Positive	515.3156	0.39	unknown	827886	0	873565	834696	0	32349	31200	963569	60437	517166	0	36232	0	0	0	0	0	584732	0
fpHILIC	Negative	227.1287	0.45	unknown	4441052	4305397	4420579	1953010	4161703	7803529	3730752	1563769	826316	2549144	2473359	3023552	2343572	2053273	2813180	1250747	1512955	1531815	2673335
EIC	Negative	297.1165	2.47	unknown	148255	64321	96792	33689	86054	140275	95687	66283	9750	112797	97080	23665	10098	10865	72439	65769	13475	51529	12851
EIC	Negative	377.1796	2.33	unknown	5361023	4317150	5204941	3342127	3058092	3030251	5858307	3806652	2876789	1903994	1917801	367333	314609	40719	3517497	2788927	192496	3356069	278423
EIC	Negative	319.1533	4.60	unknown	186751	0	176968	203337	32227	48023	78462	128180	27343	175412	29298	103858	146094	0	0	0	44568	127134	0
EIC	Negative	449.3281	2.21	unknown	254983	39598	191909	174022	67613	7422	106767	375667	75393	100689	7971	43622	21966	37769	54291	41996	19825	199160	34262

EIC	Negative	520.6001	0.64	unknown	86008	33269	95186	69280	83244	59156	56243	23779	43235	159839	10017	16728	23550	124272	0	24414	13996	10545	97895
EIC	Negative	311.1528	3.38	unknown	72344	106987	196700	437436	186102	123125	164532	171939	239196	139133	93677	156598	135848	32907	28430	126518	120588	214879	0
EIC	Negative	417.0284	1.26	unknown	388790	323603	442966	124434	226279	234997	219615	266813	151542	232464	202323	29346	87272	141686	124358	263582	58532	11245	153728
EIC	Negative	311.1659	3.21	unknown	249487	171605	177657	278056	147621	291251	129986	137388	65040	92881	113478	35390	36543	0	90735	175495	25124	114406	24537
EIC	Negative	293.1393	2.23	unknown	139390	33920	225938	177839	54944	37111	84506	222455	137336	231954	34365	43285	25873	24088	29266	64625	45936	117876	42154
EIC	Negative	632.4972	6.28	unknown	1040073	1154995	1348077	2526985	820551	923099	1180800	1404488	1337491	824257	781375	443386	916016	904133	761037	288081	948274	794104	392187
EIC	Negative	420.1383	1.06	unknown	61587	209815	82393	66170	44524	32072	89117	0	146024	0	0	0	0	0	0	0	0	0	175327
EIC	Negative	331.1914	4.93	unknown	1479546	527591	1537848	1038297	804482	557573	918343	1496655	353664	1133039	555673	395217	422736	329631	405640	0	391441	1047878	485105
EIC	Negative	499.0997	1.26	unknown	300790	323412	384699	88994	244446	230891	227318	268846	183821	208518	230900	5295	101058	191860	150622	254024	44493	0	202868
fpHILIC	Negative	215.1650	0.39	unknown	567497	869900	1586733	3955744	1504595	1021144	1235852	1678137	1799905	1219172	630080	1179907	1036495	405216	226076	764388	853346	1629172	619444
EIC	Negative	313.2748	6.01	unknown	230765	213765	498563	562382	195997	126289	176624	516771	270162	136251	120278	117276	160788	114638	84279	122609	126539	280162	116036
EIC	Negative	447.3125	3.59	unknown	335788	43344	335585	82019	55766	56229	39242	169528	30973	50243	11093	13300	10807	35787	29532	30079	30784	92743	16486
EIC	Negative	543.4623	6.35	unknown	331587	164926	224078	1038752	163382	221719	102588	241840	283632	227357	205162	266616	273823	0	0	221137	89603	212780	51579
EIC	Negative	323.2594	6.37	unknown	1285132	1201697	2744475	4563697	2162899	2288295	1614422	3068129	1559122	2143100	970869	1246558	1481866	729217	545568	1390944	983322	2351353	810731
EIC	Negative	244.1999	3.81	unknown	200874	153466	503479	706932	321895	252833	320750	601082	392593	313431	193269	240687	225777	210620	90502	174115	199751	439601	171593
EIC	Negative	225.1857	6.03	unknown	327108	683748	1057805	3575410	1455468	645773	1049499	1237269	1529307	891436	483675	1068429	826506	280773	186438	465316	847766	1504623	384430
EIC	Negative	243.1965	3.81	unknown	1247395	1038218	3217983	4368684	2094064	1631405	2070782	3652059	2542275	2090888	1179852	1555346	1389817	1378662	581796	1086184	1317969	2869693	1032161
fpHILIC	Positive	344.2790	0.61	unknown	1171198	851492	1217631	2625706	1000009	1276559	1152726	1158348	1878660	1346202	697276	1101134	997740	286931	418776	855894	656074	1150715	633640
EIC	Negative	381.1597	4.91	unknown	84402	88276	149389	373144	208434	79850	154944	174545	216483	127962	66908	153610	115067	47862	27389	102852	100048	191338	58051
EIC	Negative	294.2157	5.46	unknown	157020	108450	184398	485042	212382	121073	143006	252332	342235	156774	102636	120461	118779	49629	61648	121951	126780	231524	74641
EIC	Negative	342.2372	3.62	unknown	377817	139078	441558	255611	168932	226375	296789	431936	88325	264956	118213	172785	116528	64197	108200	118058	85721	271610	114325
EIC	Negative	631.4953	6.28	unknown	2266747	2401260	3218288	5892637	1754377	2143084	2785350	3327034	3063606	2040343	1923565	1063144	2082134	1952802	1834182	749130	2289777	1633281	1022300
EIC	Negative	325.2021	3.36	unknown	643010	304443	689686	476506	472459	465142	515333	732678	150722	474376	251589	278350	206747	150070	204240	186619	209016	461801	286380
EIC	Negative	307.1892	6.03	unknown	115819	221521	359887	1035415	494825	220763	343179	362037	475814	313508	156604	354407	300269	90489	68201	166051	319721	434663	105860
EIC	Negative	289.2174	6.36	unknown	864486	897071	1022071	1923639	817340	1074213	949230	1492476	977414	808327	584607	785649	789738	422590	631119	570509	591714	709073	563438
EIC	Negative	594.3425	2.35	unknown	4951037	98086	3606532	3109429	1436694	3825925	1778396	3550396	2704702	4860634	3013112	4806385	2599605	233418	127591	4145678	124732	3087147	208279
EIC	Negative	283.1450	5.79	unknown	97058	91451	138691	222827	150668	59158	143280	114704	161795	110632	59930	151291	98065	29328	21722	85400	96640	113804	46858
EIC	Negative	363.2295	5.83	unknown	1866985	1889151	1811867	1977195	1507343	1863620	2452718	1723024	1656193	1484782	1835641	982559	1568588	2322689	2198221	121404	1845305	0	1192347
EIC	Negative	272.9448	1.21	unknown	193902	122934	156404	124025	111235	35689	27890	54378	21677	37537	23077	22221	19167	22044	30224	48693	16278	35520	15372
EIC	Negative	297.2075	3.74	unknown	575825	262537	568388	296255	331648	427620	430960	576233	230716	374183	180282	308898	296466	200601	191296	253769	190957	400441	128095
EIC	Negative	510.2478	1.12	unknown	1433539	557438	2308064	1068154	813067	945205	1476903	1629763	1021428	1829534	1195980	642184	268915	648411	797991	670002	764162	541228	665767
EIC	Negative	324.2625	6.37	unknown	378463	258988	648807	1007696	523399	351036	485900	503979	513102	336710	233603	353414	333542	230038	162335	333954	297418	363963	233497
EIC	Negative	377.1807	2.55	unknown	1552317	631353	1815120	934546	304691	524814	475235	645367	229066	0	0	0	0	0	0	372762	0	905526	0
EIC	Negative	387.2542	2.82	unknown	298940	64802	527132	78430	79145	53031	83339	304994	79420	158189	45494	54570	35605	74879	55398	29840	45816	179468	42307
EIC	Negative	553.2752	3.12	unknown	236794	12143	215711	162365	33486	0	0	171439	26642	320758	0	0	15441	0	9751	10978	0	99435	16184
fpHILIC	Negative	170.1266	0.43	unknown	421982	311399	508646	954395	527215	305724	368654	513598	647475	393689	366985	511118	353526	125024	192854	232921	250478	465148	251065
EIC	Negative	479.3374	2.97	unknown	260476	12739	230082	77763	9746	12165	33332	342923	28603	64842	0	11865	0	0	0	0	0	252424	0
EIC	Negative	592.2515	1.12	unknown	117580	60196	193737	93662	74617	85083	131216	166348	97853	173434	109143	66786	36067	61530	73809	63283	73460	55988	62125
EIC	Negative	511.3995	4.07	unknown	14178	0	55543	239400	56664	31142	47760	95366	95839	68133	26836	43228	76945	12887	0	51298	19473	123896	0
EIC	Negative	251.1654	4.07	unknown	190738	69498	259950	157640	89506	122954	109641	207146	49355	219097	102325	139143	133204	127870	61964	39703	56687	96124	91146
EIC	Negative	506.6207	0.63	unknown	119210	65056	180909	93973	59421	68037	55682	15615	91574	240845	21613	18798	0	0	4714	19247	0	17773	132062
EIC	Negative	419.1349	1.06	unknown	259073	925806	350428	275921	175547	130775	357981	13795	613290	0	0	0	5953	0	0	16608	5270	0	751193
EIC	Negative	625.2130	2.08	unknown	317348	206166	203476	0	77668	130282	110046	0	49212	0	133687	0	26419	78373	77702	78691	0	0	64304
EIC	Negative	247.1703	4.88	unknown	197800	333338	373881	1600070	501023	243856	404161	568036	964062	414196	210222	317977	492582	113493	100235	404473	406563	545878	155070
EIC	Negative	387.2756	5.99	unknown	7985182	5920876	7484828	7829693	3839223	3923599	6882420	7044663	4831627	4054505	5001692	2690674	5198639	7245136	4998504	1057980	4386268	3035076	2487410
EIC	Negative	625.3606	3.15	unknown	339778	24713	356761	116467	132280	47593	65134	514260	70171	179284	49463	12919	30743	57215	73028	47219	73555	256302	49817
fpHILIC	Positive	456.1912	2.23	unknown	846708	371886	715911	740658	475424	484590	354653	486286	585438	481763	399084	506116	461638	303959	232695	334317	276198	346757	252200
EIC	Negative	363.2546	5.18	unknown	414724	168263	340770	282763	252737	247938	231524	336419	196410	288743	154859	187480	154264	104225	105835	169793	109716	243399	138153
EIC	Negative	251.2015	6.31	unknown	2726161	4512901	7487843	17216134	8571413	4105383	7308736	6881233	8081847	5109964	3454261	6113883	5096427	2331677	1364307	3075162	5543635	8584028	2784392
EIC	Negative	607.3820	5.01	unknown	969810	1346007	1031631	856804	0	0	1168039	0	0	36864	0	995413	0	1054327	0	0	0	714161	0
EIC	Negative	266.2209	6.36	unknown	882589	1306011	1837244	2672205	1632188	1243694	1520619	1858431	1955439	941715	927393	1100437	1331771	836160	714204	1041187	1186285	1231318	848082
EIC	Negative	341.1979	3.13	unknown	2961163	526050	2299429	1018622	791435	0	1191660	1642769	0	1500125	427201	661391	473444	504078	540321	0	0	1678217	0
EIC	Negative	566.0712	1.26	unknown	93648	85194	105836	52516	69107	63120	70721	64164	62991	76015	60599	14247	44733	54665	53124	64961	28239	0	56332
EIC	Negative	327.2906	6.33	unknown	353191	376186	918484	850688	311805	282638	277168	718796	459580	222389	203162	166102	213904	333267	153674	189805	216167	418315	283495
EIC	Negative	511.2536	1.12	unknown	344240	151362	577812	278498	204422	233658	376846	393067	257065	483276	310440	172029	78310	165586	209396	185635	206191	126897	173611
EIC	Negative	388.2789	5.99	unknown	1895840	1369864	1775414	1792756	897327	905809	1577397	1576033	1130130	946390	1157418	642906	1209713	1721249	1186248	261604	1008438	708363	587432
EIC	Negative	241.1808	4.46	unknown	1603743	581580	1750561	3155375	1421675	1282208	1311626	1992437	1048941	1544681	683405	1601970	1356037	387690	408893	766353	962045	1797244	420021
EIC	Negative	290.1797	1.77	unknown	131066	64616	117119	128967	75657	103358	90128	95983	63080	107176	69631	82661	106120	25954	83828	66652	69090	0	46138
EIC	Negative	510.2477	0.98	unknown	235379	115423	427571	193788	152990	191365	275460	307846	209629	419071	232108	126656	55100	125936	161702	141869	157602	109608	136905
EIC	Negative	439.2965	6.20	unknown	882502	430637	600992	1298007	308220	276618	289985	679022	456545	477527	319241	167749	299966	299937	369051	0	295505	366785	221306
EIC	Negative	305.1736	5.52	unknown	65085	100368	134695	436560	184507	82372	152705	145081	237008	145099	83247	166905	161275	0	27690	111169	130362	195704	34923
EIC	Negative	229.2075	6.35	unknown	1348608	1740609	3118412	3923581	2283330	1791981	2188664	3212904	2618241	1676528	1113634	1699259	1884755	1049361	877721	1473205	1535864	2298701	1356268
fpHILIC	Positive	488.1783	2.61	unknown	434468	164405	79239	509955	219712	310866	166746	178081	431494	255835	0	342619	265392	61974	0	345271	167585	208557	93917
fpHILIC	Negative	299.2590	0.38	unknown	1990457	1675967	3150035	2921270	2315958	2490380	2019559	2441058	2449344	1597834	1032319	1395864	1223720	1168937	938283	1413083	1404176	2016019	1551027
EIC	Negative	293.2124	5.45	unknown	773109	577138	935376	2643924	1029855	699748	829103	1264765	1596835	819735	557577	687480	630033	312206	395249	738476	644280	1221313	392492
EIC	Negative	569.0708	1.26	unknown	444318	428432	506270	276059	325465	315758	313285	354531	297778	352514	314766	70778	214709	243842	249845	314914	151034	0	290206
EIC	Negative	469.3303	4.83	unknown	229070	46089	358039	703739	136803	122281	220700	336484	157864	367899	124622	253291	234818	0	0	106337	152745	301646	49311
EIC	Negative	605.4802	6.27	unknown	375783	450409	543006	1274911	315057	335260	538712	615456	521914	349620	366232	305620	479171	258185	335808	118785	412413	432551	157922
EIC	Negative	225.1857	6.08	unknown	284153	493420	922080	2610236	1115124	503961	889086	930290	1313923	649830	421494	879939	666859	293245	171792	412211	776583	1276468	342558
EIC	Negative	294.1435	1.77	unknown	418754	262674	310029	91362	297215	161303	239719	279016	126636	362453	144693	152266	104494	168771	194458	165168	121654	0	212273
EIC	Negative	259.1368	2.38	unknown	136948	74921	123649	173810	98672	111430	81273	117932	102850	103279	49630	44811	48229	17013	63567	85146	31664	122032	19493
fpHILIC	Negative	561.4886	0.37	unknown	1500604	1862788	5051617	7373865	2450753	1863212	2899936	3442293	5122136	1551425	1258425	2309649	2500654	866342	1165880	1386164	2458396	3720379	1190357
fpHILIC	Negative	537.4885	0.38	unknown	960017	1530520	3891231	4925251	2212206	1788975	2446737	2668423	5237539	1663775	1088981	2253948	2504153	965435	1028658	1706401	1833516	3047107	1412226
EIC	Negative	413.2819	5.46	unknown	341040	278559	277279	316542	202401	228626	297048	238922	245640	223428	287474	120832	201356	283536	363301	0	210971	85653	104014
EIC	Negative	364.2329	5.83	unknown	404543	415934	359553	418204	323650	402387	534763	387252	349404	315750	411984	208183	320998	483002	487834	64629	398381	76233	232652
EIC	Negative	249.1860	5.68	unknown	2105667	3079395	5035725	14827454	5948377	3021856	4424745	5865297	7727950	3826335	2290424	4712044	4214877	1476571	1185614	2820618	4301721	6720996	1993403
fpHILIC	Positive	342.2639	0.61	unknown	1492894	266544	1451675	2369282	799709	1997346	1019940	1792632	916603	1085084	294726	727596	766879	259220	517153	913578	678577	1309143	699538
EIC	Negative	477.2476	3.32	unknown	22170	28382	23923	191166	15183	12320	16748	113792	91410	74078	50849	100131	70151	0	0	0	40695	116788	0
fpHILIC	Negative	177.0291	3.30	unknown	0	702690	425624	148272	194735	402747	72650	53573	181963	258055	258002	127268	117330	82605	119988	407620	0	44197	0

EIC	Negative	429.2490	2.39	unknown	1204264	430225	985908	924428	663385	674529	601740	1537044	167531	802583	251475	272106	181409	315064	273546	167390	271477	1226204	363192
EIC	Negative	630.3781	5.13	unknown	756967	219134	498635	0	352655	0	470949	480611	0	0	0	397603	38114	111215	289683	102549	44617	387794	0
EIC	Negative	250.1893	5.26	unknown	249733	258967	406133	1061060	403211	175744	300444	495695	553244	287351	149845	210838	321297	104582	138737	195071	285664	433319	166569
EIC	Negative	285.1987	3.34	unknown	271800	0	280408	229451	90050	75761	91462	236104	75757	432385	0	93850	73397	84044	67558	0	73097	219432	0
EIC	Negative	313.2748	6.12	unknown	86485	103450	231255	271143	134209	72027	130963	234058	179170	128172	47340	98896	133103	51635	45851	107078	91615	180566	49885
EIC	Negative	393.3012	6.43	unknown	4241434	3586228	5813549	4339999	3580637	1690331	3470105	5190064	3092787	3888732	1829920	1327143	1884727	1685378	2099287	2407361	2426744	1844420	1998364
EIC	Negative	619.4931	6.29	unknown	500826	726135	614394	574348	648351	585096	715367	693845	412654	455769	402504	123664	482273	502053	590266	217259	477316	272497	382849
fpHILIC	Positive	352.2451	2.29	unknown	3957881	468558	2196233	1327886	890147	1365189	719873	1047697	163487	976742	252836	362343	425953	161205	793785	491909	117505	1262230	0
EIC	Negative	293.1398	1.77	unknown	2241571	1487055	1659444	425941	1639058	822072	1356702	1436708	631594	1950498	786782	790624	594554	943498	1080391	912639	640768	0	1114013
EIC	Negative	273.2342	4.02	unknown	108045	95188	308756	981330	230632	185277	197622	548725	396435	303401	162526	296751	261378	99265	29020	265403	171613	450329	76465
EIC	Negative	249.1860	5.26	unknown	1336391	1316500	2390404	6189870	2247785	927643	1736339	2813392	3342580	1734210	924756	1274869	1835670	778291	817931	1267583	1661736	2412263	913370
EIC	Negative	270.1793	3.10	unknown	318272	81285	189307	129469	129095	133577	160406	270316	63867	149940	57820	82400	65844	62285	88888	83784	82042	171672	58042
EIC	Negative	291.1025	2.21	unknown	151334	140154	211019	163710	129322	113458	147814	218725	142365	121286	89544	85319	68855	54403	82366	84481	83356	143106	88189
EIC	Negative	648.3875	5.79	unknown	196828	467403	265767	243417	315702	349009	261957	272508	282004	99854	245822	187326	185862	194331	177347	189636	247045	114312	222136
EIC	Negative	268.2002	3.44	unknown	348265	237687	832437	809473	541625	362568	410874	799898	441932	505026	224271	348457	337683	410784	113031	233541	385945	632284	276232
EIC	Negative	412.2788	5.46	unknown	1831550	1544380	1564134	2087475	1089147	1203462	1832124	1261253	1509209	1258184	1541406	666894	1103768	1578534	1770275	280555	1325990	529975	530049
EIC	Negative	354.2350	4.03	unknown	73924	94497	183552	468854	158481	103122	135348	309008	243299	184391	101248	180333	149833	66713	15685	155782	128510	255760	55432
EIC	Negative	339.1795	2.39	unknown	34041	45144	79109	54908	50179	69666	55528	55304	27915	31600	30039	47282	35620	39504	39799	21438	38478	26901	39899
EIC	Negative	333.2049	6.06	unknown	2634557	3205219	5194925	8199724	5165963	2630690	4134294	4939022	5493546	3700031	2655826	3042497	3745162	1951788	2096548	2473519	3151328	4475167	2211760
EIC	Negative	411.2756	5.73	unknown	1674066	1245155	1572654	5128380	636394	704618	1215179	2092323	1155131	1708559	1537990	565680	1346728	1933158	1759501	0	1412605	0	571344
fpHILIC	Negative	402.1010	0.97	unknown	284018	348786	558996	478689	365900	195208	277380	357700	273625	320335	160912	134139	137002	123552	142648	160255	124030	414636	106814
EIC	Negative	279.1590	4.28	unknown	124002	179299	185253	546884	304013	190775	232306	207542	321122	142445	120491	206764	216070	104361	69918	157977	196334	259031	127264
fpHILIC	Negative	280.1401	2.30	unknown	3290731	2185687	4227312	2114630	2345442	3041519	2790065	1936026	1719045	3406129	1543382	1343286	1752765	1212786	1690483	944406	1588260	2682284	732273
EIC	Negative	447.3491	4.83	unknown	265986	39885	588894	1540983	168561	188169	304320	538520	93697	458421	52269	241745	280306	0	26762	64006	177748	522597	43969
fpHILIC	Positive	137.0456	2.43	unknown	5189035	1114892	8228001	648230	3473628	2838702	1535668	605422	2861422	847284	114232	62780	4348725	3770120	0	63538	7187571	57573	0
EIC	Negative	269.1762	3.10	unknown	1867852	462747	1123918	768329	806657	760588	984861	1529475	318028	929586	345514	489913	378560	391629	545073	475271	464391	1049113	310139
EIC	Negative	250.1893	5.68	unknown	371846	541409	910174	2733192	1044401	555202	793390	1010205	1371668	697546	425412	834786	733700	244888	226347	511446	793733	1199591	356595
EIC	Negative	551.3238	3.55	unknown	572068	738631	606387	636138	576886	564782	631893	492578	477927	526974	599559	631434	570603	569719	570468	521857	519432	0	163393
EIC	Negative	342.2371	3.69	unknown	241833	99630	239730	158615	112674	118636	173908	223000	46631	168864	77673	96422	72290	45398	61985	63157	66264	169147	76061
EIC	Negative	397.2228	6.51	unknown	112554	98091	451856	451344	276002	105078	0	83185	602945	304294	384547	109925	281662	54032	85590	254041	222121	63264	65496
EIC	Negative	391.2861	6.39	unknown	5481023	3546600	4986900	4501543	3256071	2675273	2571768	4002503	1571232	3715085	2617304	1659741	1796960	685636	2449632	1491056	1936563	1832682	1400875
EIC	Negative	433.3329	5.44	unknown	856223	133339	1429536	459490	509043	209186	224123	949704	208217	506893	126271	139003	103238	158287	173421	141720	180542	671408	193879
EIC	Negative	448.3171	4.05	unknown	1016422	72224	845051	283758	58867	101883	110639	445861	64080	323784	54129	51054	54235	140981	55563	41639	58102	306682	44684
EIC	Negative	464.3023	2.45	unknown	923957	0	1261741	668299	102113	222587	0	1725151	105171	32247	0	0	0	0	0	0	5622	1392968	0
EIC	Negative	319.2220	4.29	unknown	4640990	1060134	4884373	734656	229632	813950	1089481	490336	0	4512700	458694	1614733	329199	408153	222604	300609	404047	443630	332816
fpHILIC	Positive	455.1818	2.23	unknown	4153546	1478302	2795455	3235968	1676489	2079020	1523825	1868253	2397853	2188290	1830555	2089677	1790433	1095150	964528	1443230	1108372	1484245	1206058
EIC	Negative	415.2110	5.67	unknown	876437	438132	824711	698282	665095	484715	576080	892162	530746	657157	398136	493555	404481	335285	365776	419099	400404	542151	323944
EIC	Negative	365.2275	5.83	unknown	708982	748058	676048	763131	606998	770739	897229	681335	679721	558658	727801	413908	605536	832027	793762	0	731733	369586	473710
EIC	Negative	419.3168	2.97	unknown	152449	0	146585	63186	8930	0	20920	220059	0	51040	0	0	0	0	0	0	0	162830	0
fpHILIC	Positive	69.0454	0.95	unknown	484137	760297	608595	352325	474479	385829	395781	229714	236924	249371	269470	130912	145244	232162	221773	261996	220269	211974	206479
fpHILIC	Positive	460.2698	0.40	unknown	3822787	2645492	2444374	2556330	3177535	2746609	2356125	2215691	3401749	2429663	2093497	2427495	2494771	2131666	1438192	1896389	1796896	2429251	1502495
EIC	Negative	387.2756	5.83	unknown	13678795	15313858	13222057	13518143	11216296	15109049	20294946	13111255	11710459	9827503	13756211	6458564	11337553	18172857	16358779	3752140	13684285	5074958	7774598
EIC	Negative	625.4811	6.41	unknown	387427	308019	485601	1539399	621634	201588	492117	627977	1003796	352021	476775	329088	539693	115642	0	670897	359161	735752	152909
EIC	Negative	251.2015	6.06	unknown	43752752	53050435	86427131	162296814	80868970	41449959	66289329	83264971	99011416	55944545	38210157	48826992	61802482	30006368	31493716	41880441	53735961	80578431	34645108
EIC	Negative	291.1276	3.89	unknown	175781	189018	313106	588796	320132	164527	281982	285232	359780	230683	145690	286220	225493	100296	97818	171777	211853	320199	121491
EIC	Negative	489.3230	4.27	unknown	5520290	1375150	6256163	3442575	1851580	1785572	2250491	5759206	2189070	2279703	1228800	1444905	1244939	2756531	1711338	1091194	1422068	3583729	1240808
EIC	Negative	279.1583	4.60	unknown	355419	541022	1038830	2092610	913920	357076	975211	928517	1180080	809966	351178	883829	732545	218263	200848	561012	680957	1055491	352573
EIC	Negative	355.2133	3.61	unknown	367497	92510	284932	130114	0	0	0	259859	0	149308	78140	102387	67102	56957	73574	0	96573	0	0
EIC	Negative	577.3506	2.91	unknown	313851	170821	293489	435565	116020	277222	117390	118427	0	269678	73259	289792	230582	0	184339	94482	79392	0	53875
EIC	Negative	369.1843	5.67	unknown	151087	69262	154351	127578	104694	83366	103979	156806	83535	114831	74076	73113	79986	68263	66155	60354	63293	101918	57560
EIC	Negative	319.2220	4.23	unknown	1430546	1123440	1236385	0	0	1390692	1376112	0	0	1231881	238279	1527914	1269513	146192	0	0	182751	1417648	215355
EIC	Negative	388.2789	5.83	unknown	3146793	3563068	3130280	3227958	2615587	3575972	4777773	3063818	2763149	2293620	3297964	1532064	2668375	4243672	3856799	859422	3257137	1186814	1875503
EIC	Negative	429.1956	5.67	unknown	362330	186219	341934	280180	252932	198479	245392	341296	185990	262311	149095	180606	173258	128921	151420	143709	137718	236391	135315
EIC	Negative	326.0047	1.26	unknown	987034	804044	965553	566519	616431	587258	568837	637694	510957	701342	509300	175513	355522	433905	409411	552164	259859	252444	448360
EIC	Negative	243.1965	3.00	unknown	1922524	1359178	3602718	9584248	3174503	2232905	2480961	5692461	3749411	3789935	1942122	3671834	2853094	773167	472854	2478432	2260970	5251792	942222
fpHILIC	Positive	237.1698	2.28	unknown	1158562	449135	907108	702919	524646	811009	566876	680140	401380	732125	199533	610770	273859	116741	487238	466222	164953	742752	103486
EIC	Negative	593.4784	6.25	unknown	359418	42725	86797	691961	192893	220946	439462	275910	101656	244714	485370	226461	166781	100152	235786	80835	182210	251335	61260
EIC	Negative	259.1915	5.18	unknown	103273	81730	253886	409151	197311	122776	171134	190789	244286	120532	100291	108151	141983	51359	87687	141333	109006	220473	52667
EIC	Negative	265.1809	4.64	unknown	3802637	2183361	5094691	5861749	2659040	1392650	2740429	3682786	2085691	3670317	3309999	2435533	2591053	1041886	1383096	1508005	1414418	2948349	1034349
EIC	Negative	252.2047	6.06	unknown	7719551	9323212	15479736	28612915	14348083	7338815	11770296	14877398	17401804	9983496	6832129	8798236	11090218	5330267	5642159	7425206	9703655	14408031	6210216
EIC	Negative	419.3166	2.38	unknown	220806	0	201552	98372	0	0	0	185372	0	42773	0	0	0	0	0	0	0	122614	0
EIC	Negative	383.1760	5.79	unknown	159126	192078	261279	479615	306782	179728	300795	287806	310913	245611	139637	298577	223653	78819	92538	181576	214616	297681	130485
fpHILIC	Positive	189.1310	2.57	unknown	585736	250679	229372	502814	350328	329744	216401	167563	697578	401312	226202	220449	218320	107284	246559	373331	277291	148948	119826
fpHILIC	Positive	270.0905	2.43	unknown	496867	64103	759735	40507	306502	303013	175925	32255	353436	122373	0	0	470159	387592	0	0	775156	0	0
EIC	Negative	542.3380	3.93	unknown	190666	0	756402	427133	0	0	0	443430	0	286865	0	0	0	0	0	0	0	293511	0
EIC	Negative	356.1180	1.84	unknown	152334	114766	0	0	75686	119292	92571	0	56250	0	63786	52825	69158	48915	55793	70561	43254	0	58388
EIC	Negative	334.2097	6.06	unknown	552685	631461	984305	1555148	881890	456785	756708	901933	1119214	688013	477853	515442	745624	389687	377013	476918	617607	859304	400162
fpHILIC	Negative	169.1234	0.42	unknown	3403054	2859739	4316185	7428398	4788413	3229232	3804569	4042743	4409701	3411849	2665377	3939183	3260409	1574877	1826241	2564171	3205505	3621240	2266371
EIC	Negative	269.2125	3.46	unknown	1642069	924781	4113793	14845088	3738331	2215153	3002257	8855867	6456694	5248031	2191906	4863171	4624135	505789	389122	4198329	3089039	7780928	674305
fpHILIC	Positive	372.3102	0.60	unknown	1821440	1406724	1685121	4060052	1431795	2268252	1873517	1874497	3230545	1513499	1457639	2014588	1966480	888662	876576	1996821	1045147	2456600	725653
EIC	Negative	313.2385	6.38	unknown	27785648	14373986	29324349	21967054	19049934	14978568	21191498	33526007	10759272	19241948	11987819	11662155	11034566	8987851	11995092	11217807	11889943	20789063	10306425
EIC	Negative	405.2865	4.04	unknown	198348	107659	257376	140440	146105	143955	140092	214972	156024	218186	132924	39869	106913	159786	141241	51569	119016	111255	110406
EIC	Negative	316.2365	6.39	unknown	362660	2560110	710177	2594999	980197	1085115	1256866	613961	2082083	950659	1105058	520667	1254826	741838	601670	1055368	1094990	442455	813724
EIC	Negative	270.2159	3.46	unknown	299681	161978	709398	2635640	644625	384123	537455	1549896	1159408	948433	391271	865608	830193	90761	64839	730048	547104	1391922	114745
EIC	Negative	429.3011	4.27	unknown	8620576	2354657	10218410	5671341	3249426	2883610	3765143	9495966	3547915	3831187	1870544	2432970	2072680	4649228	2994092	1863098	2304941	6086658	2044966
fpHILIC	Negative	164.0353	0.55	unknown	2009571	894651	1106082	943496	1060205	2067086	856875	920424	666181	338723	448039	684260	1144895	1276471	893526	833157	1001337	241640	745127
fpHILIC	Positive	228.2051	0.39	unknown	3959213	4069178	9559486	11246249	7264372	4561091	7636902	8453921	7760632	4539793	2925696	4248880	6267886	2913214	2170135	4613249	5366161	7784434	3868400
EIC	Negative	244.1999	4.65	unknown	327219	467725	725843	2435516	835025	483874	797975	912431	1132446	940644	438125	721205	702103	208645	180123	550630	553204	1246266	247866
EIC	Negative	333.2074	5.67	unknown	13888772	6529039	13155964	10804049	9239118	7128919	9020038	13076860	6820616	9604762	5671732	6234902	5933570	4773288	5548763	5435222	5494387	9069387	4694311

EIC	Negative	411.2754	5.46	unknown	7447854	6089325	6024853	8164242	4144196	4666294	6727550	4875568	5921111	5190127	6070035	2562884	4312465	6123887	7169799	1099346	5214171	1977678	2162121
fpHILIC	Negative	484.2738	0.46	unknown	1051345	0	1832755	1737800	0	0	0	3086059	0	1105677	0	0	0	0	0	0	0	2065543	0
EIC	Negative	244.1999	3.00	unknown	309423	226013	587539	1478437	487949	345106	398921	894556	590726	589178	312789	558758	450707	117745	93665	379437	369646	827267	149494
EIC	Negative	269.1762	2.92	unknown	536759	84222	391151	257495	157422	190487	170942	272160	151195	341147	103833	161263	110207	87974	111897	117615	91097	232649	77725
EIC	Negative	444.1910	3.26	unknown	62286	69077	71960	214389	11271	80466	47937	35421	22303	72517	36065	26601	11584	7747	38746	80097	13526	0	0
EIC	Negative	314.2418	6.38	unknown	5651504	2901304	5818493	4431561	3857876	2982985	4292496	6749938	2101483	3899225	2451557	2389548	2180183	1793369	2476144	2236110	2337400	4207438	2145718
EIC	Negative	393.2589	6.44	unknown	9336188	6207880	2242120	4932759	4130178	5735690	6909588	6744200	3190371	4699390	5866225	2720920	3939876	5349651	4837909	2157774	4578307	3039381	3300660
EIC	Negative	334.2108	5.67	unknown	3071011	1445132	2978681	2452294	2024287	1544492	1995147	2932768	1539717	2140078	1286027	1387463	1321621	1082868	1227417	1184818	1232213	2038025	1050591
EIC	Negative	603.3501	3.90	unknown	0	483808	154653	186224	371838	29464	136965	103287	126779	159495	0	27884	65986	480759	57546	25292	25350	166583	242102
EIC	Negative	437.2907	6.20	unknown	17085576	6287392	11071505	24863419	4900613	5054590	5252207	12912766	8350172	9398981	5302988	2989670	5722761	6128363	5742555	2302379	5788101	7349989	3394032
EIC	Negative	271.2281	4.03	unknown	5272897	4560071	16272474	47940624	11893512	8179565	9415012	28251870	19290084	14830210	7624512	14258891	12701426	3645091	1881558	13639944	9336421	22877605	4218276
EIC	Negative	629.3662	5.12	unknown	3287967	913886	2231067	2504673	1541402	2208850	2055924	2125751	338049	1425826	1145351	1673460	864090	546046	1203068	455352	1082540	1669744	1172598
EIC	Negative	541.3410	3.92	unknown	563949	22128	2397636	1297449	31635	0	0	1525267	22700	905841	23267	21717	21310	72569	70412	29027	20618	946994	0
EIC	Negative	583.4929	6.42	unknown	8661649	12576420	10145866	14521306	13093745	11802531	6733589	10695580	14204331	10874786	935153	7697771	10970569	8084970	6359422	7970176	7424799	8808213	7624692
EIC	Negative	272.2314	4.02	unknown	1009204	832618	2778901	8352971	2077710	1447028	1711976	5102833	3463060	2597694	1350052	2535319	2263260	643973	349358	2408663	1714773	4103245	749733
EIC	Negative	329.2330	4.05	unknown	466155	260107	551525	456398	325984	301919	316141	444117	162140	390284	191617	237701	228801	167506	176182	168080	201080	291184	213264
EIC	Negative	632.3939	6.09	unknown	474487	539017	430424	321722	425380	622953	504798	374344	278045	297188	396299	359671	337405	256364	324174	226361	316583	250460	375935
fpHILIC	Positive	234.1440	2.50	unknown	805893	431872	387927	881939	499159	542072	390254	494925	650407	581174	519862	276746	501078	179006	327535	524188	370705	274047	248955
EIC	Negative	385.2271	3.29	unknown	331145	52474	463893	927893	41981	42938	39877	123823	43736	259708	12091	212575	47587	39357	45049	28236	46161	78659	0
EIC	Negative	502.2946	5.13	unknown	7014199	10381541	6871217	5180213	9041237	9333574	7575243	6026649	5976479	3955581	6508217	5043623	4789132	8016565	5290844	2857627	6436273	4339562	6795107
EIC	Negative	300.2626	5.25	unknown	413202	354940	963202	2182050	603454	465835	547594	1279108	790955	616970	347650	628592	539149	470635	180229	580607	414946	942533	382797
fpHILIC	Negative	559.4728	0.37	unknown	1879888	1921965	3655425	3869433	2747745	1601809	2289407	2355071	3281032	1646995	1520217	2176134	1787894	1316562	1611630	1501437	1603455	2019386	1593312
EIC	Negative	438.2941	6.12	unknown	6342095	4798799	4292784	9507360	4551740	6663674	5693233	5527689	5972761	4976556	4393865	2854197	3705743	4420503	5598564	2161624	5674525	2822176	3023107
EIC	Negative	319.2279	3.36	unknown	3770608	0	0	3187172	432817	481682	0	490994	559257	0	2112354	1553557	0	0	0	0	0	1334321	0
EIC	Negative	300.1684	3.12	unknown	21567	12936	25409	200392	101469	0	9833	110990	9550	16360	8615	0	10724	0	0	14316	11276	132105	0
EIC	Negative	337.2044	3.16	unknown	378833	115512	376422	181219	140645	187853	91046	259348	32868	239015	95438	118159	42823	49359	136807	61484	133435	0	152493
EIC	Negative	279.1605	2.39	unknown	1076035	574560	834057	798949	679713	1035419	856114	970821	241155	853557	458635	578475	474646	399010	580892	350472	574305	585660	485982
EIC	Negative	265.1811	3.69	unknown	319634	374805	363750	1774516	556935	371779	501533	546276	1132793	587357	330087	397268	610660	197402	168038	548558	468196	706077	227950
EIC	Negative	579.2262	2.92	unknown	519117	276374	159170	240021	129627	218625	107095	315651	141443	424278	144741	166045	186365	48420	87478	136239	61733	303192	33878
fpHILIC	Negative	284.1001	2.28	unknown	1330715	1170751	1311107	914257	986548	1216872	1001693	711212	706194	1187374	703220	644900	864211	519406	539380	447314	827952	889617	451514
EIC	Negative	243.1965	4.65	unknown	2055635	2899105	4679192	15185516	5414785	3369931	4962605	5623973	7068209	5586228	2744606	4834370	4539549	1484616	1178040	3496910	3821555	7851520	1704260
EIC	Negative	628.3628	5.12	unknown	9089642	2573010	6143616	6742600	4248086	5903239	5840549	6115889	896207	4008529	3325108	4819518	2436415	1520720	3203284	1333263	3012238	4726264	3435352
EIC	Negative	440.3104	6.35	unknown	597214	820435	816199	1020007	621016	860566	702955	683955	528443	778162	684787	452227	450440	696046	641739	103472	629181	480423	467132
EIC	Negative	269.2125	3.74	unknown	1576778	816803	2601957	2468425	1748236	1273689	1576982	2870583	1483565	1655080	873343	1153833	1157604	952187	628244	1160266	1172623	2248787	913612
EIC	Negative	270.2159	5.19	unknown	423330	581235	825766	2691090	1149418	566138	926112	920231	1330401	989010	603005	1086382	822437	285662	248860	697671	672434	1404129	323460
EIC	Negative	430.3046	4.27	unknown	2594495	745742	3015547	1648681	1021240	957103	1065008	2730953	1069588	1170843	575130	783304	629378	1373701	884466	635292	704759	1866829	590915
EIC	Negative	416.2143	5.67	unknown	218629	113540	214909	174307	161398	119071	143954	226994	140736	166814	90490	118824	110610	86567	98217	108361	114475	124127	97035
EIC	Negative	279.2234	6.29	unknown	7118742	7399854	10917204	15183376	11093691	6563814	8356655	10803921	12520763	6900215	5497453	7724778	9694923	5305494	3870912	6876145	8524935	9097763	5469351
EIC	Negative	447.3129	4.05	unknown	3274078	310686	2561499	960237	273835	427893	351359	1550906	262944	1212910	192501	213167	184910	448108	247515	167004	242809	1139693	170723
EIC	Negative	266.1846	3.69	unknown	40389	64324	91808	390519	102007	69528	88080	96349	202000	106474	65198	70099	108012	30950	30659	92425	85365	133145	41392
EIC	Negative	348.1914	6.06	unknown	153666	188659	275357	453828	234669	125180	228622	240813	297856	185657	118328	160311	210865	106627	97301	139729	171147	247105	118814
EIC	Negative	511.3063	4.27	unknown	1415421	611166	1535189	1032175	681237	724435	845434	1614209	740860	701402	502665	606134	509423	877797	655806	431455	554243	969723	554422
EIC	Negative	325.0019	1.26	unknown	6433679	5156853	6177297	3604459	3888255	3789622	3754061	4135063	3203105	4564356	3397115	1139206	2382322	2803283	2664326	3572733	1687554	1996721	2979245
EIC	Negative	437.2907	6.12	unknown	23079924	17786820	15207782	33502704	15924624	23746981	20359145	19444545	21378163	17619791	15783146	10342978	13124648	15719536	20461872	7549218	20423256	10315032	10667389
EIC	Negative	270.2159	3.74	unknown	301644	141974	473371	434326	293913	232762	282380	512897	273817	319408	168826	201215	198219	173972	113004	219245	208446	389002	170007
EIC	Negative	565.4469	4.14	unknown	57978	46178	282315	334844	144021	75754	131751	372725	133836	150485	57931	59111	65197	192002	19609	43050	115328	298003	115794
fpHILIC	Positive	236.1686	2.28	unknown	9998151	3638727	8072779	6271562	5072919	6234539	4660713	6908636	2362733	5743314	2405324	3130039	3424944	1627727	3845669	3047547	2468077	6459702	1592313
EIC	Negative	463.3444	5.22	unknown	433154	0	219975	46082	0	0	0	102563	0	63498	0	0	0	0	26049	0	0	44750	0
EIC	Negative	266.1844	4.64	unknown	702808	369517	851369	1013546	422130	231967	500570	658028	336586	663036	642114	427589	461384	217500	244564	306217	257705	510128	165735
EIC	Negative	347.2591	6.32	unknown	3435215	2559772	4441307	10105687	3508178	3838336	3964665	5942673	4328239	4122382	2814547	3479598	3577570	1623165	1728107	2894672	2914926	5145786	2226550
fpHILIC	Negative	496.2744	0.40	unknown	7348068	168102	8247751	9897222	0	277172	0	7644149	0	4639755	0	0	0	127691	112321	0	127154	5116748	124668
EIC	Negative	253.2076	6.06	unknown	821224	954026	1602962	3086770	1455359	786119	1189472	1587396	1892265	1100635	828926	909224	1168927	669343	665849	872988	1056869	1479267	712333
EIC	Negative	415.2966	6.17	unknown	14922521	16600908	13910551	18552340	12102019	15051525	18571796	13569892	13189591	12629510	15076953	8349735	11552283	14094845	15490626	6129414	13295204	6926866	8385752
EIC	Negative	322.2474	6.04	unknown	214091	246658	404723	769905	348222	314099	404869	504813	396365	346582	259764	385502	375531	162240	185755	302479	274635	438904	162624
EIC	Negative	351.0080	0.55	unknown	151967	95595	87378	221450	117751	218947	158729	106122	31855	60732	100243	91540	115171	294602	170510	100872	63232	113193	56072
EIC	Negative	361.2387	6.21	unknown	2884346	1250017	2631589	1663775	1695696	1810540	1825622	2466891	441585	1673950	1223182	1370268	973125	634332	1012274	775504	900736	2095926	928271
EIC	Negative	298.2030	3.37	unknown	58310	27488	40016	328883	41257	18749	49451	70576	149439	66631	37007	48895	60311	10392	28146	108062	28876	63401	0
fpHILIC	Negative	563.5036	0.37	unknown	1018803	2044365	4074694	6800932	2060014	1577388	2870729	2684511	3233068	1794647	1280613	2053893	1627921	870277	998470	1738893	1648002	2960563	1271643
EIC	Negative	526.0574	1.26	unknown	324622	304154	348221	192989	224041	214961	222608	262402	206692	264803	225415	62426	157902	176281	177871	215051	106235	93798	191534
EIC	Negative	342.2019	3.07	unknown	1176840	214053	978237	420775	414552	430658	589379	684590	49583	623017	225511	252965	186474	189102	252365	182898	276813	577024	215630
EIC	Negative	319.1535	2.36	unknown	441795	160251	405706	391571	249297	200215	172495	536273	128293	453051	148560	159684	135076	129423	168143	141989	108430	365466	108933
EIC	Negative	416.3008	6.17	unknown	1885900	2132677	1803518	2326292	1605806	2034503	2145693	1743216	1685357	1447108	1892888	1095608	1442655	1777684	2012584	744442	1611709	904932	1124373
EIC	Negative	535.4008	6.42	unknown	169059	3073290	2302702	2792741	2016965	200236	894887	1835152	1454185	105086	173020	958074	1462808	930230	762925	1053220	716157	916356	999334
EIC	Negative	415.2071	6.06	unknown	480694	605627	869866	1163024	890139	497780	665693	799332	968650	712920	536026	553053	687838	369743	359586	532010	569892	788880	405091
EIC	Negative	311.2592	6.38	unknown	339336	302937	385835	302388	199880	207604	281141	285401	260876	160778	157738	199135	188785	123340	153352	150602	174882	241027	138378
EIC	Negative	485.3029	3.74	unknown	252364	28354	379334	113061	59795	0	18025	168677	24121	130804	30267	15869	14013	30635	27049	13151	16814	113006	18074
EIC	Negative	347.1884	6.06	unknown	821254	826337	1237092	1882057	1148831	613301	963899	1127565	1310996	881004	591117	730200	943813	443556	423458	602392	939624	1105085	519942
EIC	Negative	327.2181	3.93	unknown	647888	310885	520095	415637	390635	471167	417535	495483	157371	391832	239935	249468	226475	190865	241839	210500	260298	361899	268784
EIC	Negative	323.0061	1.26	unknown	393971	320497	392042	221648	239902	224217	220296	247326	193602	260317	200252	76885	140981	176599	159565	220272	100032	123564	179734
EIC	Negative	339.2541	6.34	unknown	1323313	806695	2819376	1450962	1311535	844824	1255032	1912105	385623	1387116	781280	1027800	819486	379889	632468	472763	538398	1587969	742074
EIC	Negative	490.3259	4.20	unknown	335211	141561	362085	279814	144848	179272	148176	346118	186574	174509	108821	154998	112689	209752	149498	100986	135493	220755	103071
EIC	Negative	291.1967	5.17	unknown	338121	124232	346284	261473	149407	96650	197300	338915	101711	231676	146731	100286	101377	106224	130255	108744	59345	245122	72130
EIC	Negative	329.2336	3.80	unknown	609873	202946	632450	442365	311734	392075	324889	543370	112336	382689	184589	201636	185013	139833	164416	175086	218780	421517	193298
fpHILIC	Negative	330.2523	0.37	unknown	2191035	2050993	3823049	5390284	2103801	2515214	2667155	2992379	4013653	2455115	1391987	2556304	2600178	1330141	1556555	1599683	2168537	2957818	1679001
EIC	Negative	606.3783	5.96	unknown	1319652	1498930	1405100	1064818	1231384	2027403	1699627	1271753	637153	845952	1109578	1184498	934027	844903	1168937	703963	980964	809233	1129772
EIC	Negative	401.2186	2.05	unknown	1014657	1268045	439535	1279080	752157	1805176	1016191	530615	463938	557419	757305	1161822	1026998	198139	1093738	721154	625413	156511	594527
EIC	Negative	238.1529	2.87	unknown	304379	224310	301471	1095258	361401	273593	340594	393198	719628	461767	248306	335628	433466	139644	153886	345127	301427	505812	153082
EIC	Negative	255.1969	3.60	unknown	318013	163394	368114	622126	346248	268828	332065	413398	289562	344752	202280	291795	272005	86831	118757	234027	225501	455760	120790
EIC	Negative	269.2125	5.19	unknown	2236880	3264349	4435142	15370488	6124002	3243901	5054210	5053613	7218961	5411471	3272934	6127043	4579028	1617758	1350515	3919685	3744527	7805736	1869655

EIC	Negative	273.2073	2.36	unknown	521331	145261	419161	448019	271753	332837	256414	623238	137310	526920	136414	163433	141068	93722	137227	147175	210823	488519	151952
fpHILIC	Negative	498.2898	0.44	unknown	57871648	1114611	73275488	88571296	1108062	1487736	1883424	68525880	3040223	36733076	1181197	1024233	1971105	1894498	1511511	1017314	1363793	48099900	1280809
EIC	Negative	239.2015	6.35	unknown	872575	1001780	1608180	2147466	1451232	1043837	1175207	1541945	1651402	962707	732141	975425	1309844	740311	416684	1022534	1102901	1360512	915022
EIC	Negative	337.2139	5.68	unknown	471921	161366	348239	430822	200350	148718	199566	278477	252398	261983	193385	158411	182321	282696	217666	34562	182411	169698	153866
EIC	Negative	325.0019	1.41	unknown	581634	1347719	0	0	843975	875519	781141	0	542929	0	904486	149623	410718	620614	485298	847242	236082	0	569884
EIC	Negative	597.2296	1.96	unknown	0	124311	0	0	30981	43107	115908	0	0	0	173400	65998	98688	17445	18124	56337	44667	0	14458
EIC	Negative	343.2852	5.82	unknown	106125	90899	135474	465899	184128	66108	218402	120200	148536	141302	72036	169327	198412	27872	86670	70654	122992	177347	61091
EIC	Negative	301.2657	5.38	unknown	110398	83274	280705	320550	130187	57816	125943	254022	177157	130970	66656	127399	108372	156589	58850	44216	96225	214679	111039
EIC	Negative	300.9577	1.25	unknown	416267	261908	382481	266174	193912	159896	156821	198005	151194	297862	138242	68665	95311	150809	110468	126333	70991	150000	148367
EIC	Negative	319.2275	3.96	unknown	438621	0	602687	1774346	506895	346558	435054	806054	616081	715289	359739	682666	570607	0	0	471903	385161	1053992	0
EIC	Negative	540.3376	3.93	unknown	1633091	131894	7543868	4069398	157416	52675	63859	4598456	173784	2730419	157401	159341	163381	312895	251508	171092	150938	2809367	175496
EIC	Negative	429.2860	3.73	unknown	495029	284322	534715	341761	265730	284548	404091	377561	353905	487159	396797	131616	196034	408913	360485	104036	331733	165049	227802
EIC	Negative	251.2015	5.80	unknown	315389	429524	757563	1316072	728961	466874	615802	912301	801089	518200	385913	575703	651712	337943	297663	458053	556449	783665	392152
fpHILIC	Negative	670.3730	0.54	unknown	1021559	298692	501749	0	299914	346945	385285	139086	350929	0	426250	0	65290	269183	107459	579609	0	0	388732
EIC	Negative	568.0678	1.26	unknown	1464789	1394013	1530690	933443	1065096	968716	1046694	1129972	960993	1212833	999706	225377	664374	794870	794670	980412	448756	513317	920207
EIC	Negative	271.2281	6.35	unknown	19613268	11590651	21630409	48464100	15988647	17469753	15688630	20047482	20088957	16965355	12946519	18926555	15136803	6789210	6440425	17145834	11152821	18934760	9491349
EIC	Negative	500.1029	1.26	unknown	74896	87365	114092	23704	65760	70088	69733	0	55618	64264	61926	0	21992	54359	37650	73243	14585	0	59359
EIC	Negative	242.1841	3.82	unknown	140682	202346	201031	907014	341021	216614	262946	289702	534889	346323	203865	294678	276195	107899	92125	241931	231841	494600	104412
EIC	Negative	235.1338	3.97	unknown	161961	127812	234362	631170	204298	131612	212651	265532	297303	270731	123611	201855	211595	75786	75940	180680	150461	296219	83747
EIC	Negative	299.2592	5.25	unknown	1943541	1730384	4699885	11079545	3031889	2102759	2793466	6651657	3891926	3276850	1956483	3245582	2847690	2323289	1064351	3069982	2281325	4683094	1987452
EIC	Negative	272.2315	6.35	unknown	3426097	2062643	3767409	8766704	2815857	3126207	2702680	3582805	3644991	3095700	2375110	3294645	2705496	1239559	1101690	3057113	1973595	3405716	1716096
EIC	Negative	523.3292	2.46	unknown	287624	35573	456726	230300	83668	85076	48064	658544	35212	313987	42242	40601	35552	60539	51976	35846	44109	482406	31341
fpHILIC	Negative	223.1703	0.39	unknown	4339681	3047194	6974932	13867830	5357696	4347734	6212667	6563548	5953082	6400800	2923116	6546050	5557190	1099285	2030941	3235063	5194698	7147808	2581118
fpHILIC	Positive	235.1649	2.28	unknown	94304776	35476624	73098192	55152416	43967456	57581780	41132560	63678384	22889136	54686660	24850214	27720346	28826584	14688727	31592438	26532842	26540024	61609376	16539002
fpHILIC	Negative	187.1341	0.41	unknown	3200682	1387488	2641801	4431181	2200016	3204851	2153333	2360765	2292109	2432291	1229455	2014258	2003321	1401658	1256283	1635022	1688811	2619279	1478905
EIC	Negative	239.1653	2.25	unknown	570532	271265	617823	957294	394176	351602	446720	697603	541798	645050	286737	398079	357646	140118	210400	428939	247145	675838	166308
EIC	Negative	297.2436	4.52	unknown	3508333	2030131	10212497	34816343	6429336	4432925	5626335	18234081	10926511	9071520	4715858	11340752	8816223	2284768	857299	9545858	5565335	14098722	2398361
EIC	Negative	414.2940	6.17	unknown	86087236	96979381	81159144	110162719	69994648	88803355	98296621	80521717	78176140	67518712	89010633	49515251	67273031	83187172	90043301	35802353	79012214	41037989	49915367
EIC	Negative	607.3817	5.85	unknown	877288	1195102	906465	722663	1003986	1435079	1298953	820146	380272	662358	969979	877784	769428	619468	836622	512089	813730	524398	829777
EIC	Negative	237.1495	2.22	unknown	664962	358989	540178	999190	457710	463047	678949	669874	495187	670419	338391	527599	516817	138491	297959	520647	329213	603267	218070
EIC	Negative	252.1320	2.54	unknown	203260	96669	164411	109361	101154	116582	161800	176775	94325	130944	80488	69890	68714	75045	71035	83561	69865	149064	90027
EIC	Negative	291.1967	4.29	unknown	272032	304134	299701	1140857	349769	261396	334599	396791	828668	372601	220320	246944	477969	137374	165504	472312	339625	427757	142235
EIC	Negative	268.2000	4.37	unknown	564406	657497	910964	3624477	1165213	714865	853245	1072625	1592499	1080720	614093	1194814	880242	326967	318856	751369	744261	1707062	329905
EIC	Negative	490.3259	4.27	unknown	1783647	476218	2036975	1090091	641144	646642	747150	1804523	664013	751685	416608	453883	475730	974195	622244	439362	488281	1203727	459471
EIC	Negative	504.2994	5.13	unknown	358642	557697	334336	299862	418689	476564	409844	321693	288158	185915	454760	237656	273962	442625	299508	161746	361540	223933	319821
fpHILIC	Negative	329.2497	0.37	unknown	10399700	7837418	12878024	20952898	7945118	10022139	10094753	11139261	14511024	10029115	6617892	9472610	9150772	3869333	5808069	6386634	8686268	10464304	6992187
EIC	Negative	258.1798	3.00	unknown	500643	156379	240620	196965	212664	246223	250146	289660	67162	277791	117656	180955	138488	120197	189336	117247	132402	210023	112131
EIC	Negative	413.2911	6.17	unknown	335130417	378599278	312913782	426766988	276293192	344477427	377865620	310284522	305331119	261321798	344430175	190371126	263271813	324220719	351150379	138572206	307714424	161009747	196305326
EIC	Negative	270.2159	4.05	unknown	421848	420757	960433	1701542	755695	607398	563711	1304452	863777	668668	438176	585371	604217	579687	251489	653297	600911	978106	447271
fpHILIC	Negative	253.0176	0.58	unknown	1241876	1234839	1546357	600929	1069252	2372199	1156200	1116924	464117	874151	1140837	934608	516597	1028903	1346928	634345	555111	864754	1026148
EIC	Negative	251.1654	2.52	unknown	652635	493366	659447	1303149	635654	589655	510374	456381	545150	616997	489060	1243830	480839	149147	382543	541070	299156	469899	269243
fpHILIC	Negative	195.1390	0.40	unknown	2563306	1263566	3013902	5073973	2173859	1756881	2617738	2973072	2871619	2156031	1605967	2334208	2351653	653425	1001545	1684237	1847344	3188465	1174778
EIC	Negative	263.2017	6.07	unknown	158069	232884	197153	459204	231898	165045	224051	222913	294388	184441	149181	218832	236272	108075	167399	180838	185427	148465	135326
EIC	Negative	273.2342	6.35	unknown	393132	208249	388674	998159	335656	359680	297646	368362	415939	342768	242269	408356	298466	142931	133157	348766	200893	406121	188074
EIC	Negative	502.2945	5.44	unknown	510472	459371	421710	338331	407438	547513	453660	409458	246516	250576	306050	252337	224735	365689	312700	124062	371031	366221	310159
EIC	Negative	254.1762	3.17	unknown	111989	71228	117452	449664	86213	98252	102202	133157	202725	116374	92622	119533	126092	0	62951	139769	98947	146037	0
EIC	Negative	343.2879	5.83	unknown	57248	24262	313962	474327	188867	125408	166413	245627	143291	0	102356	179691	199212	54804	58367	102044	141840	301306	21894
EIC	Negative	378.8434	0.52	unknown	135591	119020	187906	196745	90696	100473	170932	128583	142835	150531	123855	116584	128101	50836	121044	0	99794	123022	112310
EIC	Negative	361.2603	5.68	unknown	4222848	1447864	3099757	3432115	1734684	1814558	1956092	2412773	2489635	2265385	1688584	1393454	1828648	2481128	2221123	919233	1555124	1297216	1502132
EIC	Negative	341.1981	3.07	unknown	5821781	1266326	4671042	2206791	2017809	2272270	2861768	3385739	0	3185918	987136	1239809	958944	937884	1174120	804921	1342656	3105821	1176634
EIC	Negative	237.1495	2.87	unknown	1649142	1485430	1709358	6945740	2200182	1668108	2115705	2503192	4518514	2882650	1596264	2105054	2647285	878910	977142	2303210	1949095	3211960	964367
EIC	Negative	238.1080	2.03	unknown	136624	116720	143592	0	60322	313014	153463	0	0	0	193475	226096	96329	0	111884	53034	153792	0	62353
EIC	Negative	328.2208	3.24	unknown	104076	49232	108407	96845	66863	62790	61488	109178	30717	88100	41770	49843	39946	33984	34321	40586	37832	89554	37184
EIC	Negative	632.3840	5.49	unknown	665936	649599	562709	672216	650559	739015	694453	626136	457791	408527	631952	585014	447995	366903	419137	387059	509963	424559	551354
EIC	Negative	606.3784	5.85	unknown	2527378	3567715	2759816	2134642	2884188	4195593	3773779	2386392	1132682	2006160	2903053	2601376	2367810	1759913	2495852	1610885	2305607	1541327	2453587
fpHILIC	Negative	213.1495	0.40	unknown	1569861	1484956	2108631	8335356	2143821	1799268	2510079	3034848	4539887	2845280	1421699	2509249	2701245	739036	577277	2524505	2179158	3692751	1193164
EIC	Negative	267.1967	4.37	unknown	3005110	3798876	4861857	20344417	6586017	3740537	4868294	6230919	8700693	6068573	3638101	6543751	5131601	1716366	1602822	4555496	4209479	9378205	2096289
EIC	Negative	489.3230	4.20	unknown	1032865	475842	1176793	836950	484923	559108	435374	1072458	525291	569054	359485	394104	331859	664934	427066	354110	381906	713456	355326
fpHILIC	Negative	499.2925	0.45	unknown	17902810	350367	21488100	27496706	467019	516745	489263	20173404	1119898	11062717	0	315504	662575	449933	421837	391255	364190	14897461	443305
EIC	Negative	241.1808	3.81	unknown	895052	1217013	1301824	5846986	2034514	1387466	1650110	1805688	3345915	2183847	1352535	1784417	1790523	719130	578922	1478094	1515925	3107054	739469
fpHILIC	Negative	308.2676	0.37	unknown	1583585	2024034	3188947	3614486	2592832	1801863	2750953	2749032	2477657	2093650	1286971	2580711	1699241	1150398	1186619	1412714	1673582	2920835	1801808
EIC	Negative	505.3167	5.88	unknown	628570	668425	666122	472565	547193	774732	496360	460927	289986	319340	0	386399	248870	605368	391177	163268	312795	398162	602694
EIC	Negative	240.1684	2.24	unknown	99270	41075	74247	160092	72688	52323	76028	115404	75860	104299	62492	58960	61094	19199	39920	71506	38925	104584	30774
EIC	Negative	363.1804	5.19	unknown	267821	266184	365107	563643	344282	205541	286123	336572	445710	271861	215132	257660	277763	247656	0	264738	252078	408517	253296
EIC	Negative	604.3629	5.13	unknown	2314305	2066153	2721514	1803713	1779263	2222887	2132945	2637257	1134182	1651658	1981221	1395461	1468777	1454445	1427106	1258735	1512741	1510187	1506627
fpHILIC	Negative	180.0335	0.93	unknown	630266	486356	468791	519183	612026	722240	584918	336985	348534	310938	370514	303915	328517	293595	340633	507784	373651	301505	374078
fpHILIC	Negative	126.9050	1.09	unknown	5476509	6114743	7696156	3019300	4137769	7871423	3890243	3842337	3106004	5048787	1825125	6877378	4831547	3464229	2607534	2857428	4748559	3899939	2540705
EIC	Negative	647.2349	1.74	unknown	107484	57277	168108	124204	61969	40860	103159	97950	69477	103600	88298	92209	98821	33980	68737	86223	74349	0	43129
EIC	Negative	298.2471	4.52	unknown	765618	477407	2028256	7002668	1323833	830455	1131838	3717236	2075281	1748504	921173	2360276	1734881	446291	194505	1969793	1098761	2882059	484866
EIC	Negative	348.2627	6.32	unknown	822966	682755	1040763	2483589	834204	889110	924037	1400276	1076420	972927	618173	747368	854792	405600	402962	814042	755371	1221550	551536
EIC	Negative	283.2282	4.07	unknown	620353	423796	1176766	1002861	868553	574090	676885	1249428	536989	765684	520251	509639	503508	630263	408387	353731	579443	1010797	507522
EIC	Negative	592.3244	1.24	unknown	323135	0	702588	74450	6859	53779	17391	168080	16010	686979	20455	15755	22270	0	52953	28547	0	61850	48665
EIC	Negative	297.2077	5.28	unknown	277960	148829	626404	221458	191909	162008	224401	541457	94014	407164	195415	239341	163105	248541	154564	0	164960	331414	238263
EIC	Negative	362.2639	5.68	unknown	879642	282645	663219	728111	367938	363696	403194	472867	509019	468453	349590	287607	382529	504911	436890	179826	345665	280572	308995
EIC	Negative	415.2433	6.20	unknown	605783	213449	411216	889458	250390	204012	202554	520574	287458	377983	225741	159408	207846	233236	259966	0	273531	354691	186378
EIC	Negative	413.1941	5.58	unknown	185722	190537	248413	711547	289752	160492	234023	326020	555103	319081	188248	277465	334574	98538	132098	275132	247744	354861	139151
EIC	Negative	251.1654	3.02	unknown	828921	555811	883955	893350	675065	479571	648292	757507	479275	688693	554157	708356	434616	300637	544106	416326	345518	570586	355802
EIC	Negative	313.1487	2.42	unknown	187751	81407	351360	100525	127786	141817	136609	206844	54555	211427	85830	40491	34856	24279	36687	39294	65910	284189	64649
EIC	Negative	504.2715	3.87	unknown	236257	383065	179520	251916	296246	335771	52257	233595	273178	218543	245780	59686	157016	83370	40867	279559	61939	159980	335284

EIC	Negative	247.1703	5.39	unknown	276534	154873	260841	934114	322502	250447	400902	483069	318059	414800	205005	381885	451526	65120	160082	323235	228323	472639	123747
fpHILIC	Positive	401.3458	0.59	unknown	1060111	804567	932055	1778829	811994	832212	888030	832440	1279922	716824	733685	715823	1148636	524882	672790	1040412	696387	811746	202567
EIC	Negative	330.2370	3.80	unknown	126252	40909	116114	91595	57429	94978	62265	115363	22845	82713	40418	47919	43324	32167	38684	44497	50885	82588	43516
EIC	Negative	237.1859	5.85	unknown	226270	337587	377137	1511878	488739	269674	331665	458054	729618	449714	232297	305739	660094	118885	111798	492524	433815	497523	145021
EIC	Negative	570.0723	1.25	unknown	105039	102796	103472	61608	66033	66838	72527	49124	62498	85291	71277	19263	50098	59653	54195	74652	37352	0	67868
EIC	Negative	314.0529	2.41	unknown	144474	99381	173636	125504	82159	81420	91094	183561	50451	120266	117535	82850	45574	16749	70240	72120	33804	140918	44104
fpHILIC	Negative	180.0748	0.48	unknown	4946335	1830360	2280380	639779	1925813	2230611	2386008	2393673	2738540	2506953	2583704	275817	1252534	1310920	1680960	4680027	242095	658542	1379422
EIC	Negative	399.2763	6.25	unknown	483226	283550	364318	406605	261537	303725	343222	372152	259484	312262	387894	222106	278872	275658	341744	172902	279044	176067	170888
EIC	Negative	527.2983	5.23	unknown	2722947	857659	1728621	1861840	1569545	2322310	1595541	1700259	426377	1169962	1302872	1915853	681863	847120	1432006	375553	1118163	1382558	1332313
EIC	Negative	555.2803	1.77	unknown	327313	302425	366539	292538	94762	284825	418863	342728	32954	486120	297860	315061	241276	271942	81008	184833	226701	0	377582
EIC	Negative	623.4355	6.31	unknown	369812	945629	1147921	1083086	675292	833249	423601	910643	1095717	685092	967500	857005	359565	433528	681641	549895	720150	542398	424867
EIC	Negative	364.2129	6.41	unknown	3684288	5843763	4193842	4896897	4992972	4888764	6130560	4585280	4273805	3782903	4070371	4623172	3995011	3058874	3109589	4182504	4175445	3454976	3512249
EIC	Negative	249.1498	3.21	unknown	576318	226536	652365	380270	306622	342820	193375	472675	106240	525557	257714	150243	118387	0	206633	141996	82134	494375	128911
EIC	Negative	605.3660	5.13	unknown	813306	845605	852976	596549	629051	696055	751842	878645	431492	523395	651549	467428	579300	485106	476074	468790	542007	527540	497353
EIC	Negative	523.3288	2.85	unknown	256295	293664	651565	1151813	320118	301476	305022	484209	363841	675137	330591	337443	177263	63632	109585	234334	252340	666470	54139
EIC	Negative	543.4638	4.13	unknown	65876	36564	254447	313060	120051	59855	90529	283131	126580	165842	40846	51746	55041	167319	14104	36519	98759	267397	87955
EIC	Negative	479.3384	3.43	unknown	793132	587026	1277553	18208167	2083086	1226026	2188121	2976608	5490471	3414057	1394849	4046267	3003509	232242	302557	2148050	2045046	5557631	310799
EIC	Negative	399.2544	5.95	unknown	151982	341267	115809	351590	221878	252359	225496	132650	349897	171050	177274	169368	482098	146360	192881	226376	199436	123748	135129
EIC	Negative	241.1808	3.07	unknown	4406545	1894670	5227629	8753114	3743397	3575270	4270157	5716151	3879312	4819262	2890125	4574877	4063113	1770885	1685169	3477505	2935024	6039820	1592872
EIC	Negative	283.1919	3.90	unknown	784722	375015	724799	757021	543276	484738	623969	780275	362820	595024	357556	481618	374174	216892	358083	382136	313367	735127	286849
EIC	Negative	531.3320	3.74	unknown	430668	54299	705504	108617	89678	49794	50138	248010	57085	201956	43465	37108	14360	37189	36076	20455	44382	226383	40127
EIC	Negative	227.1652	3.47	unknown	178601	157783	265079	917313	294091	217673	320156	374107	514669	347219	218857	357046	351788	105844	110504	329555	249704	485028	110856
EIC	Negative	283.1921	4.83	unknown	328979	146382	415945	744323	241421	248188	297606	408934	166536	417041	194677	273073	309414	115680	120081	153160	193492	430964	116917
EIC	Negative	265.1481	2.62	unknown	0	3106860	0	2042635	3169427	0	2233457	0	955941	0	0	2552354	1284163	0	0	1567854	0	0	3170900
fpHILIC	Negative	151.0401	0.52	unknown	687945216	305392160	289129408	81141120	294068128	315022112	384826944	316810048	453634016	323896288	339952544	2264593	97656424	202012784	208686112	669749952	43309780	91504664	302977888
EIC	Negative	300.2626	5.38	unknown	1016646	756812	2093179	2655514	1035847	657689	987514	2125490	1392246	1031886	595276	681874	741265	1208745	563533	561983	762455	1771403	971914
fpHILIC	Negative	197.1546	0.41	unknown	8257199	2778161	5892637	11277799	4074439	4720874	4692571	6156130	3855338	4388535	2276990	4671450	4119327	1531831	2691067	2905592	2923578	6732657	2304286
EIC	Negative	228.1686	3.47	unknown	16159	26359	47750	155458	53022	40418	62604	60009	84734	65015	38636	57585	64242	21457	21375	60858	43197	78066	21285
EIC	Negative	273.1723	3.27	unknown	47891	37843	73809	45741	23841	62864	79667	64848	98374	53100	69105	92617	72966	42079	24669	42050	51170	68301	60416
EIC	Negative	477.0439	1.25	unknown	131302	131678	131533	118222	96945	83351	91666	88697	87714	119531	92629	47783	70509	71417	84947	80352	60123	28950	81484
EIC	Negative	319.1588	4.83	unknown	527185	265660	734990	1002990	464473	451791	588780	726606	346346	742231	312480	504993	535777	132588	207239	403826	406645	714292	199134
fpHILIC	Negative	87.0453	0.92	unknown	1090676	608632	1526227	1146657	751913	842169	663491	1026968	797540	728611	556074	759930	606613	545431	505507	643028	648366	668171	594273
EIC	Negative	338.2702	6.38	unknown	544157	353646	613324	1940430	409697	362123	515873	663017	620715	446325	309257	533838	528092	173080	218038	574590	344124	589024	202053
EIC	Negative	243.1421	4.24	unknown	200333	97196	194591	183268	115434	149724	129002	153267	70118	139379	99405	104569	71996	26954	78247	124054	35409	173438	51854
fpHILIC	Negative	280.2358	0.38	unknown	51006864	65372852	100794704	116234264	76634176	65694072	76400872	83825664	105224600	59180368	49059492	69248824	70039056	42264040	47442832	58727764	68102728	76332488	55239196
fpHILIC	Negative	152.0434	0.52	unknown	61112420	26959610	24602762	6764659	25413560	26704418	34651976	28531902	39216628	27982972	29454980	203175	8502363	17683856	18220174	58960812	3872214	8273880	26590532
EIC	Negative	312.2264	5.98	unknown	509978	178627	674895	422710	301155	201750	357313	718918	113269	471457	238554	202440	157353	139111	180325	150579	178923	588349	172479
EIC	Negative	502.2845	5.13	unknown	6753310	9250428	6552233	0	8697547	0	7271668	0	0	0	6179720	0	0	0	0	0	5895318	0	6095034
EIC	Negative	439.2965	6.12	unknown	1123177	999094	786534	1659243	849237	1074187	1030028	1013070	977710	875688	775481	531343	658933	772663	1241083	389523	1021582	507108	553653
EIC	Negative	288.1616	2.64	unknown	482669	212299	488667	469744	364007	227662	412207	666661	102325	414928	311165	205312	206333	157498	133430	112675	235058	560258	290018
EIC	Negative	242.1841	3.07	unknown	668051	296479	835114	1387558	576344	564311	691824	900378	605264	764669	465848	719913	635809	276577	238463	547956	470723	1002312	249093
EIC	Negative	463.3071	2.46	unknown	2694739	223890	3977161	2131464	319959	675324	230894	5640208	340413	2674304	328972	330303	196156	340475	242923	185948	267375	4573014	148198
EIC	Negative	449.3284	4.15	unknown	2108234	102523	1571228	642255	113456	85415	103031	847495	62908	728369	131823	91334	63866	76448	93452	54144	64424	646669	53046
EIC	Negative	271.1746	5.90	unknown	224557	114159	212862	293060	84797	105929	82877	170757	150485	141905	93155	44990	66856	0	66780	135678	0	200901	44019
EIC	Negative	287.2231	2.96	unknown	154745	154038	598520	2513971	656411	379669	596068	1227881	1068315	804619	331938	767765	870110	97637	109013	1052805	571601	1117016	105248
EIC	Negative	480.3417	3.43	unknown	227011	184157	399309	5759525	649985	477540	691272	872683	1691739	985205	488414	1239721	938414	95100	100616	680854	622621	1795841	99410
EIC	Negative	303.1574	4.59	unknown	67092	68302	140177	387686	105024	49847	131646	132892	202780	149002	48906	160183	134977	42393	41399	112366	86779	227814	0
EIC	Negative	383.2912	5.63	unknown	317124	235843	429943	744944	340344	265248	310842	438256	438098	300339	206334	244485	343447	124257	205831	268869	279009	395112	201753
fpHILIC	Negative	111.0201	1.36	unknown	23259306	19039550	19305102	28579316	21154208	24109976	20827160	16833262	22356468	19530082	16135233	19019420	18213398	12090857	13766662	20548338	16268565	16292566	12483674
EIC	Negative	283.0404	0.56	unknown	854342	98780	120436	427271	66461	554016	147833	24106	70209	151729	136672	154711	95015	106260	96947	162892	97575	121152	171853
fpHILIC	Negative	179.0711	0.48	unknown	48891900	18548934	22636634	5824865	19329314	19329690	21545264	21913010	27045780	23967002	22612894	2109420	11705481	12366389	16223975	44594464	2212182	6738338	12545625
EIC	Negative	225.1759	4.83	unknown	153382	59046	233952	369383	102268	74742	159385	203687	63397	179326	63577	130769	155889	0	44286	76132	89988	200403	44622
fpHILIC	Negative	278.0879	2.27	unknown	1529632	343105	333374	205142	249888	665946	196221	174075	263275	214097	110281	235312	193522	187588	118287	243200	210636	434402	163141
EIC	Negative	244.1999	3.09	unknown	611380	604439	1090620	1237624	999838	613415	740169	1233010	827146	901646	552251	661017	634018	869296	373522	539136	706123	1043766	670796
fpHILIC	Negative	251.2016	0.38	unknown	1769413	2109687	3693946	7253411	3778103	2446308	3865343	3440079	4392146	2511284	1915996	2790384	3084958	1603259	2157221	2188580	3015264	3923593	2251454
EIC	Negative	430.2874	3.73	unknown	99372	74126	64847	93010	71960	73058	107986	81304	78503	105185	101003	49219	52714	127719	105115	15989	84677	52470	53630
EIC	Negative	381.2628	5.38	unknown	511080	254898	803131	943989	365519	279192	436464	849577	621630	408298	257964	275469	305833	473986	296380	253257	295317	706986	376418
EIC	Negative	605.3661	5.31	unknown	2198249	1982349	2532301	1722363	1661193	2068657	2284478	2157228	869707	1705425	1790357	1614160	1505954	1381186	1507330	930996	1586917	1334376	1471207
fpHILIC	Positive	276.1188	3.86	unknown	8705762	3888037	6042492	7145914	4699274	5636940	5256789	4653820	4892048	5495188	4926348	3321729	4892415	3439551	5027145	2423306	4851407	3344036	3357049
fpHILIC	Positive	277.1219	3.86	unknown	966815	415100	692936	793990	508402	653126	604814	549815	531997	654568	565796	409459	522442	372804	569602	295700	506543	397247	384567
EIC	Negative	257.1760	3.00	unknown	3329975	1037720	1724940	1270772	1365828	1488914	1569271	1865432	343918	1625851	771280	1189632	924477	775770	1220747	758049	876994	1407313	754031
EIC	Negative	375.2748	6.14	unknown	1416576	548622	799117	1337336	663564	492702	856861	480379	534864	594180	576910	481639	464339	593548	781157	270168	591096	374353	273920
fpHILIC	Positive	276.1190	3.15	unknown	489060	189525	164631	509749	236704	487056	211194	165673	184370	282312	202990	190098	271863	46313	170292	248625	199118	190727	163352
EIC	Negative	259.1915	3.13	unknown	53916	43298	78276	187881	63482	69754	84095	81977	100756	92596	49935	56374	64226	39738	30051	68804	59405	101017	43400
EIC	Negative	320.1612	4.82	unknown	152059	26076	114066	247055	106270	46820	129017	155148	54135	138623	34668	105845	77782	0	48685	42588	96073	150368	34061
EIC	Negative	401.2917	6.29	unknown	1323813	1285022	1076614	887076	815009	1588049	1507144	897535	578467	794274	1245594	635239	772455	1535433	1596253	313850	1114513	439909	611098
EIC	Negative	399.2765	6.17	unknown	1002650	1039803	873690	1237462	784314	955098	998385	897638	829528	864590	1122126	582664	708594	971313	980375	414595	948043	426753	612043
fpHILIC	Negative	307.2643	0.37	unknown	6283143	8767304	12404810	15912288	9845644	8849903	10747144	11018790	10561629	9258331	6103246	10453756	8633147	5039565	5704009	6342231	8646337	11028073	6939775
EIC	Negative	311.2229	6.08	unknown	857194	568426	919690	1031002	695866	525891	704916	839539	559507	699558	484654	540220	560307	416189	505801	466884	502265	648644	397254
EIC	Negative	296.2310	6.22	unknown	128622	102137	135299	213448	115134	103796	105482	103986	132738	98149	82545	101845	106320	31468	67880	91993	65036	124113	76551
fpHILIC	Negative	117.0559	0.61	unknown	86804544	60721268	84489624	84151648	68448240	105730072	58317576	42691636	64731060	51140840	54627132	52880896	52844632	37880828	51668180	48125796	54580992	46908032	56559148
EIC	Negative	448.3512	4.83	unknown	98008	0	190727	452236	27797	0	0	203934	0	146409	0	0	0	0	0	0	0	175800	0
EIC	Negative	285.2077	5.59	unknown	720181	188046	1121749	693407	287702	341247	318855	656277	241344	664517	235728	371166	275324	271849	194522	149577	222533	479511	356079
EIC	Negative	259.1916	3.08	unknown	26560	40322	87765	269749	66303	64662	74660	108281	145535	105638	61121	65636	88728	44647	28968	74052	67075	141978	37042
EIC	Negative	329.0033	1.25	unknown	92994	79476	99244	62921	76588	91973	81085	85344	79531	73871	86577	34560	69010	82620	72201	93012	54442	12352	83027
fpHILIC	Positive	299.1387	0.38	unknown	718560	629778	1053917	1115410	561010	451327	518153	781857	444553	894586	691995	589922	694734	216290	267637	543854	334763	743493	206599
fpHILIC	Negative	141.0920	0.46	unknown	683570	377717	630110	868516	483817	694197	463013	498208	666450	634727	493703	711673	444079	504285	229924	419342	534736	571136	379676
EIC	Negative	513.4159	4.08	unknown	51748	20081	124409	871954	104187	98727	101307	237179	222158	168810	78397	198926	160198	0	0	126923	105604	336358	12121
EIC	Negative	415.2966	6.26	unknown	7299585	4638120	6029724	7666349	3343434	4607273	4852356	5792502	3779187	4655404	5640000	3450310	4355703	5251044	5198765	1443408	3976359	3533539	2883198

EIC	Negative	239.2015	6.30	unknown	1291484	1604605	2933354	3775831	2445302	1524247	1945382	2699773	2802199	1906016	1381659	1665410	2197511	1552419	735580	1645763	2114524	2627208	1573785
fpHILIC	Negative	279.2328	0.38	unknown	253796960	336813792	502389312	602385344	384532768	321965536	388183744	424285760	537096640	293701344	259603520	354625952	352635232	209661760	247406464	295808992	334460512	402334528	286287328
fpHILIC	Negative	321.1368	0.95	unknown	1153850	407959	904403	414251	438857	501151	347999	452980	216628	505331	246988	400051	310755	298717	347307	319921	234243	336135	254103
EIC	Negative	271.2281	6.04	unknown	4735611	8946325	9836324	12960213	8554290	7446873	7573119	10937572	8355059	7597762	5245246	6156008	6107848	6312923	4430237	5435665	6270455	9676987	6854891
EIC	Negative	526.2948	5.23	unknown	8684689	2914809	5814386	6485497	4951179	7245600	5381287	5400022	1449372	3836345	4388509	5978495	2320487	2740423	4973932	1422449	3402367	4408653	4424681
EIC	Negative	311.2228	5.98	unknown	2544066	845076	3307989	2071803	1546017	930508	1759867	3610089	567904	2447630	1137777	1023887	836444	696208	851207	762396	929010	2963757	912887
fpHILIC	Negative	117.0443	0.57	unknown	800866	637568	1052395	872158	760869	861818	736391	881409	717989	975367	712562	967214	644779	507447	540777	685146	562107	652516	580525
EIC	Negative	270.1795	3.19	unknown	93160	31631	68129	37516	47548	45336	52823	67360	19382	32823	25389	29158	30900	21656	41264	31923	32134	53393	19046
EIC	Negative	429.3011	4.20	unknown	1674073	703860	1707542	1242469	656383	876636	650820	1584147	910073	970877	569782	639803	539354	965572	708815	523409	693155	1064688	588352
EIC	Negative	391.2439	6.17	unknown	8921615	9767381	8988411	11933212	7121046	8684657	10209277	8607936	8611264	7794182	9249382	5612243	7078120	8725456	9821201	4143397	8408180	5086825	5691950
EIC	Negative	304.0911	1.26	unknown	1209324	1241576	1579615	374704	1146838	1217188	1279715	1139018	893325	1115402	1177422	98313	547720	1059859	867920	1871796	290422	195016	1127222
fpHILIC	Negative	565.5183	0.37	unknown	255190	1417692	1597684	2344288	914897	894183	1218398	1055103	1501804	683482	276877	1206422	747038	472632	637129	1021019	739879	1226620	657720
EIC	Negative	242.1842	2.97	unknown	244890	111360	353370	355793	210334	178543	173445	362534	179803	349662	148962	207830	144374	204569	71417	111777	169567	336828	157644
EIC	Negative	323.1844	3.82	unknown	98362	143726	136436	539865	214720	144212	176975	186506	335697	254006	142937	199184	176495	79411	75247	158089	164288	329584	84486
EIC	Negative	309.1341	1.58	unknown	541276	379735	307489	168998	256027	200227	253445	259880	125323	414648	159494	207227	188471	220503	181100	201686	131640	105094	220542
EIC	Negative	508.3386	2.43	unknown	183775	87890	103318	108617	179681	291764	67441	136621	61403	198455	82795	136649	75750	84218	130636	80457	107649	173900	72814
fpHILIC	Negative	199.1708	0.40	unknown	4160819	4705679	9250367	12333026	6646800	4848846	5961417	8372530	9861151	5297360	3607596	4707981	6482524	3684080	3272900	5117215	5746645	8689587	4567012
fpHILIC	Positive	426.3571	0.54	unknown	5270700	3998937	5426003	9724309	4897880	5127436	6209160	4905671	7927162	4807857	4752849	5419543	7032957	2032155	3646239	6650209	3718191	5348352	2955065
EIC	Negative	556.4314	3.43	unknown	141603	96675	155181	580373	221099	160251	301256	215601	297999	130036	162608	253873	236829	86435	99979	234348	188977	309169	86336
EIC	Negative	269.1761	3.19	unknown	480386	159506	340572	227397	253399	243026	306977	390251	98719	265004	134406	210727	172760	131597	198198	202391	170751	299113	122871
EIC	Negative	263.1654	2.44	unknown	546553	232785	486839	319617	333318	313974	416766	443697	155375	384571	215049	214069	210350	202657	280720	184544	230218	382645	200646
EIC	Negative	287.2016	6.08	unknown	312541	223545	370783	831294	364114	274358	464642	517451	395610	408077	264449	399095	372351	107284	177152	316260	287264	603229	166381
EIC	Negative	495.3696	4.04	unknown	827841	475896	635519	690384	547015	666014	555265	778859	260685	515754	436046	340742	304501	277290	442409	381574	465115	565457	306135
EIC	Negative	272.2314	6.04	unknown	813949	1590486	1758358	2230534	1513450	1273605	1332463	1998854	1470934	1312641	938611	1083881	1059102	1105659	763625	986603	1123135	1731351	1236420
EIC	Negative	389.2464	6.17	unknown	26469405	28063694	26032210	34850087	20075796	25117169	29225674	26045001	25053159	22556924	26651464	16134804	20164686	25440308	28978529	12208757	24194902	14889202	16456705
EIC	Negative	606.3783	5.74	unknown	30329745	35645786	33995393	25514561	30059228	41899109	34215516	29569444	17742987	21655307	30234925	27221914	23531058	22652029	24289980	18973357	25526550	20250954	29417013
EIC	Negative	313.2387	2.36	unknown	108477	50718	72788	148388	63729	64932	68793	124236	91705	94178	38572	82767	67975	52904	31918	84437	59048	99122	44631
EIC	Negative	305.1738	4.83	unknown	3175744	2150422	3848622	4524871	2679158	2614881	3328242	3752217	2345700	3573238	2215317	3526396	3444197	983167	1560983	2178991	2516115	3428006	1680588
fpHILIC	Positive	318.1904	2.51	unknown	715903	368218	684847	825873	511068	507790	651004	432580	818895	781357	450013	395551	599512	206132	625989	607202	323566	456189	245763
EIC	Negative	325.2658	5.20	unknown	1137992	1069345	252019	211226	0	83108	1029646	1100449	1013395	209687	1069445	1026417	1068834	995147	1064554	189127	1029198	213652	0
EIC	Negative	507.3334	2.43	unknown	568892	323476	399160	298395	527284	879555	200027	450333	198351	609154	305359	406141	221242	233972	433839	245762	316895	558834	190137
EIC	Negative	300.2277	6.06	unknown	1531232	474419	1755891	1184483	778151	551933	820172	1761593	235995	1231510	635054	526763	507857	414766	545235	288721	513583	1230107	632438
EIC	Negative	369.2278	2.96	unknown	39136	23943	64570	162555	56506	48701	74842	92177	84425	64177	44125	67011	84655	25731	14603	89369	66765	85916	19375
EIC	Negative	305.0933	1.26	unknown	175972	192057	217199	82201	168674	182037	199034	147888	134904	179037	186277	16532	89356	170947	128124	291751	48693	0	175774
EIC	Negative	361.2602	5.49	unknown	11857121	3907217	8736186	8718686	4569348	3832417	6636349	4636219	4013377	6387087	5880662	3352009	4018000	6706681	7870078	957540	5071418	2360946	2079219
EIC	Negative	553.4128	3.44	unknown	22469	34568	89523	1174424	106838	75958	137552	222739	250266	152901	0	178422	144987	30390	0	152334	146376	315628	0
EIC	Negative	347.2594	5.02	unknown	163871	95521	198843	522232	134403	142835	140522	283705	187888	240812	123136	195531	136332	85819	91707	149245	108613	278429	60843
fpHILIC	Negative	202.0722	3.59	unknown	859852	546893	515709	534487	502557	946523	402496	162041	310886	462277	824294	329748	343251	297460	459371	361342	344101	217779	419673
EIC	Negative	301.2030	2.52	unknown	492363	135863	498536	260368	190547	230304	251787	486735	66410	418163	149756	129521	110687	133300	171947	82218	128952	454866	152647
EIC	Negative	283.1921	2.43	unknown	1369418	454350	1248349	956593	707550	734883	861970	1449016	411124	1045033	471330	533690	493089	436257	525515	468868	476188	1380422	379279
EIC	Negative	407.0050	1.26	unknown	384394	320673	347651	270791	253938	262974	239784	237660	232011	284977	234607	127362	204016	203228	194454	222065	163877	166281	204890
EIC	Negative	604.3629	5.31	unknown	6346832	5850611	7696383	5043695	4817834	6213632	6570719	6352991	2614770	4917229	5279159	4684442	4445577	3912302	4261509	2977597	4822700	4028045	4527227
EIC	Negative	239.1652	3.43	unknown	28096702	25116158	36278687	141320581	47522065	35841925	49662071	56314711	78795022	60403521	38144485	66553224	57454444	15147582	17336783	47945978	45733337	79029304	18104438
EIC	Negative	323.1850	3.07	unknown	322414	178788	353514	651348	338671	326450	376968	459130	316784	395191	257244	504886	344640	173468	171497	304350	275654	510995	149152
EIC	Negative	330.1794	1.78	unknown	875428	390033	825989	405919	219476	152613	380413	163725	362975	523399	243581	257308	234049	256383	220696	341756	279960	0	151086
fpHILIC	Positive	276.1432	3.07	unknown	2059296	1142071	1178493	1601884	1597891	1607325	1238590	819993	1213568	1165297	1233994	556113	571424	484809	1029754	964828	1235063	669326	1137260
EIC	Negative	607.3816	5.74	unknown	10265892	12146918	11605992	8495912	10152198	14256099	11617442	10018946	6041495	7401256	10356836	9150479	7991831	7608813	8320524	6505744	8812590	6852878	10023462
EIC	Negative	275.1427	3.43	unknown	172947	146150	215216	900635	290425	202781	307936	344141	466314	388844	232413	403772	336042	101771	97029	286774	270811	478082	121318
EIC	Negative	283.1109	1.71	unknown	168773	116832	101592	277702	88527	85271	109326	133012	77477	203376	116966	121386	51035	61276	64091	146921	101319	34823	37089
EIC	Negative	252.1682	3.02	unknown	135429	99229	161103	195366	110755	85677	101303	165657	84431	112301	107442	122861	90087	65231	107701	74790	73306	102938	68006
EIC	Negative	413.2911	6.25	unknown	167442642	110184826	141568108	171355476	78903462	104219919	110711077	133149797	84584846	107047141	129368581	77880623	98619360	121514671	121351572	35904406	93663021	82429683	65281678
EIC	Negative	506.3541	4.08	unknown	158002	120338	245191	1723058	437557	258719	511792	467885	936323	659911	357068	722906	702795	49252	85234	471671	434500	950120	95497
EIC	Negative	464.3102	2.46	unknown	1055002	67054	1415158	781931	119624	259684	45873	1970039	117589	837821	92769	115132	67597	106655	84486	72357	94158	1661752	46713
EIC	Negative	414.2940	6.25	unknown	43249520	28312258	35709063	44474034	20196474	26946796	28695599	34483883	21603610	27539186	33760695	20191366	25123441	31222552	31675523	9182994	24239641	21250709	16557920
EIC	Negative	608.3850	5.74	unknown	2243158	2812046	2610620	1857450	2236309	3149045	2635520	2272627	1349567	1628625	2255958	2027759	1753655	1830261	1817293	1436449	1896436	1620887	2311479
EIC	Negative	265.1811	3.15	unknown	262742	101365	330485	191694	119355	155813	128074	233697	79381	190893	109258	119859	86048	118953	86564	78299	84124	199696	110823
EIC	Negative	240.1684	3.43	unknown	4446019	3961152	5637867	22335376	7509271	5642117	7853719	8909985	12383226	9424813	6043463	10484544	9015054	2363119	2890771	7622192	7285167	12422805	2845367
EIC	Negative	390.2497	6.17	unknown	6281734	6582419	6091588	8446529	4690259	5744927	7055811	5884918	6075868	5251335	6263493	3767603	4885874	6129784	7025909	2834359	5673505	3597988	3899668
EIC	Negative	366.2097	6.41	unknown	1148651	1781626	1217095	1565647	1534449	1446894	1618689	1368550	1591247	1194156	1200616	1680958	1331651	925208	973057	1223096	1345658	1205498	1164546
fpHILIC	Negative	193.1234	0.42	unknown	1027120	661239	1000538	1642681	774816	696060	896846	812373	713429	683914	702848	736008	848618	343684	584266	764770	544715	774004	377952
EIC	Negative	427.3074	2.85	unknown	0	72145	189440	2386475	401977	87938	27950	347531	880395	536247	154718	397907	316242	47618	0	265564	303076	752426	45405
EIC	Negative	237.1495	3.61	unknown	649829	421189	771318	1140899	456882	519535	615343	745329	334421	564296	517546	645420	571646	251740	310110	429003	367375	720040	265754
EIC	Negative	276.8469	6.42	unknown	326088	380235	827649	567129	684588	437049	519490	759775	558906	554273	322042	485485	646192	293027	344169	489722	559345	683503	241475
EIC	Negative	306.1771	3.34	unknown	186234	41532	224722	135374	61249	53894	69898	134434	51193	242915	39374	62906	54014	36932	56361	31683	38795	128872	34656
EIC	Negative	229.1809	3.23	unknown	158550	108889	218647	429805	169918	158072	171014	245582	182717	180539	135702	185871	177043	93452	84831	157602	145683	232502	105898
EIC	Negative	392.2474	6.17	unknown	2073302	2145685	2113736	2764905	1416217	2053047	2202235	2009433	2018311	1795925	2042713	1215366	1570702	1961900	2295437	985781	1912290	1168280	1238263
EIC	Negative	239.1652	2.17	unknown	1068617	609373	1061390	1809603	706369	824755	1120353	976005	812817	1138376	617249	1242022	1162121	279804	578180	1150457	514090	907107	331972
EIC	Negative	267.1967	3.99	unknown	428790	220374	465848	692756	369638	348855	385102	507114	352543	420231	256914	396770	327014	183938	197722	312415	303402	533417	193371
EIC	Negative	240.1685	2.17	unknown	214966	121786	147779	262061	117173	126604	173246	206530	126702	199311	114678	202352	184271	37448	111706	183148	91654	138367	64106
EIC	Negative	363.2095	6.41	unknown	15336233	23307464	17007175	20662463	20700532	19835993	20226315	18404153	17076389	15972939	16682773	18930416	16548872	12279890	13595119	16652925	17318443	13532604	14519393
fpHILIC	Positive	298.1004	3.85	unknown	1488825	885016	1153500	1443037	895370	1035499	1151253	1103983	1096087	1151499	1079687	729260	1084105	766878	1168334	582524	924217	764072	713780
EIC	Negative	294.2157	5.52	unknown	320021	285672	374594	865819	376346	252258	262993	492714	515940	317131	255781	352484	344538	87106	185570	410195	280357	417196	200928
EIC	Negative	261.1518	2.91	unknown	222833	65850	259052	343864	99883	98441	149294	202921	75611	226211	80161	104357	47718	15416	122570	87604	33113	197256	52257
EIC	Negative	303.0877	1.26	unknown	6658404	6766323	8682866	2149721	6191232	6659443	6990252	5948239	4810082	6296814	6434637	540755	3051957	5890201	4686080	10316996	1600547	1295563	6222113
EIC	Negative	241.1808	2.98	unknown	1565855	664189	2260803	2302340	1308790	1165276	1052049	2166924	1111682	2125725	861098	1295378	990906	1234230	447157	678803	1115462	2140069	982673
EIC	Negative	305.1750	3.56	unknown	200682	84512	172965	126276	125204	128771	156247	192451	57841	160274	71373	100576	81744	83900	93820	90533	85545	184601	66244
EIC	Negative	509.3501	3.74	unknown	377321	50007	770359	112909	78369	44256	44085	224876	31801	191242	38435	34151	15293	16212	39360	45839	31903	177587	39164
EIC	Negative	499.2935	1.97	unknown	35814032	3013236	0	32574738	3334685	4542490	4222523	44917016	9167182	18320148	6082800	5507862	8989997	11746930	10278147	6604462	7819150	22869726	4853566
EIC	Negative	481.3447	3.43	unknown	37677	35679	85594	1050516	131394	89450	115676	166423	315552	188469	91934	222300	189420	14259	0	171372	113696	365027	0

fpHILIC	Negative	159.1027	0.46	unknown	1552329	1423470	1952618	4470849	1747495	1949784	1701601	1821703	2589104	1940717	1347516	1793194	1768703	985250	932679	1853257	1499330	2377687	1129939
EIC	Negative	307.2053	4.14	unknown	203327	169259	488905	484868	294900	195122	243958	528285	308247	311243	170952	224149	223224	367399	157876	142450	280877	448248	218251
EIC	Negative	434.0306	1.25	unknown	235839	227043	237169	223556	166696	153313	162260	219626	157129	189738	155461	82778	127115	132851	135183	142151	106474	148054	150816
EIC	Negative	237.1494	2.96	unknown	168162	102954	184648	362014	137715	140354	172084	185307	186982	183686	118860	174623	156812	92068	93992	132901	130198	200562	96076
EIC	Negative	335.1865	3.72	unknown	714873	231080	728947	519331	334442	409717	398902	743271	116105	493669	239722	313036	223823	152162	235858	217424	227600	685545	225316
EIC	Negative	275.1269	3.07	unknown	367578	259967	290858	494141	363807	312394	386688	289311	248720	361195	290780	342289	312718	133472	248644	338377	272884	273601	139933
EIC	Negative	273.2342	6.04	unknown	102689	178414	202976	249793	176791	156015	157976	209046	159560	143109	114100	132508	117135	143614	87596	119044	129869	194118	153268
EIC	Negative	525.3431	2.23	unknown	394579	61977	612316	220201	103513	82074	71981	609063	89035	226522	47746	46539	28304	60676	55049	83926	64169	505449	51992
fpHILIC	Negative	179.0757	2.29	unknown	612686	309946	522364	466383	376614	474969	408982	486849	256831	370514	293216	342675	275633	220852	306850	315668	274082	488497	210674
EIC	Negative	253.1809	3.52	unknown	688462	408589	1282478	2931266	202452	215163	240308	474605	190356	1104835	259674	809552	336883	234091	149166	174307	132343	303578	294844
fpHILIC	Negative	178.0721	2.28	unknown	9159164	4383589	7423321	6472381	5259473	6924410	5498712	6448726	3415836	6358814	3646678	4426360	4035589	2677725	4028585	4277908	3969034	6724764	2962721
EIC	Negative	550.3207	3.55	unknown	1988513	2378355	1918569	2137040	1936636	1940052	2229575	1727329	1603539	1706589	2060420	2198289	1928652	1973971	2009273	1750661	1745181	267576	1751879
EIC	Negative	252.2048	5.80	unknown	66990	75358	135625	270588	123721	92874	105992	140861	139168	89364	67706	115222	117172	58045	50583	76540	110889	159009	83581
EIC	Negative	291.1220	3.36	unknown	403096	238355	290261	650549	300681	373075	370874	562358	225677	204973	228293	411611	454689	138821	281048	258243	186731	581475	154130
EIC	Negative	299.2240	6.06	unknown	8107041	2606973	9318454	6437823	4083662	2741212	4043261	9761348	1216866	6777465	3491364	2812332	2692497	2200280	2894312	1614638	2839532	6586498	3503161
fpHILIC	Positive	132.0654	1.76	unknown	1756934	841190	855510	1642424	1115388	1277144	934497	912132	1016773	1281959	1208325	1276118	996609	0	821555	1035267	680089	916654	742284
fpHILIC	Negative	101.0608	0.61	unknown	14560357	4010825	12756654	8122239	2454743	8715102	2104053	9375632	7715956	4638486	5114392	4502097	4506900	5520849	5340449	3291441	5939452	4518888	5157558
EIC	Negative	389.2910	6.39	unknown	100402104	37037716	70601856	0	29641972	51374132	33808212	33114106	0	59229984	45922924	41647916	0	62881492	54869252	0	38555592	0	24734394
fpHILIC	Positive	147.1202	2.96	unknown	2088916	1279101	1722420	2213526	1003602	1168451	1139365	1328192	1195042	1090251	1290400	1158861	1138157	861237	888926	1036348	954294	1173159	609277
EIC	Negative	249.1860	3.36	unknown	461824	135356	340893	270085	181736	193032	204223	329886	159481	301688	119540	165081	135458	107410	155354	130034	152085	318637	97818
EIC	Negative	505.3515	4.08	unknown	509963	400015	793815	5526344	1400295	854968	1641754	1496346	2874374	2079756	1155590	2266212	2135097	140444	313088	1555059	1534682	2980214	230768
EIC	Negative	353.2699	6.52	unknown	719529	539217	1189209	2016676	856849	613911	954892	1202744	574703	807234	857924	622249	557554	571444	576621	661725	658060	882005	585586
fpHILIC	Positive	298.1137	2.15	unknown	1019994	479634	571051	875828	578461	986178	691728	456432	671631	624205	651000	720273	681035	396379	546506	727355	491251	542043	402046
EIC	Negative	362.2639	5.49	unknown	2479974	867082	1872867	1811864	956455	782975	1418345	961364	860228	1347178	1247884	692351	819396	1489764	1725325	213853	1081423	494386	471971
EIC	Negative	299.1650	3.24	unknown	333438	357660	417790	305636	114039	217935	197997	137012	127344	115800	185652	278845	124229	36626	160692	192365	102190	100318	80505
EIC	Negative	284.1954	3.56	unknown	491793	196649	427169	315925	295642	281379	357387	493734	126556	344704	180211	284126	216329	193073	234210	225613	222200	403706	159402
EIC	Negative	496.3732	4.04	unknown	242142	161524	199367	229685	154943	206717	166976	244498	85952	184135	155116	90715	83763	98524	128051	116408	165882	189230	88461
EIC	Negative	605.3351	3.04	unknown	289130	50121	387255	239078	68062	57543	89042	375819	91651	213867	51507	43823	53246	130490	77797	45117	59658	307172	56736
EIC	Negative	294.2156	3.98	unknown	67447	34977	122431	434716	100616	69221	72594	205476	125064	119252	70149	111688	110247	54751	30886	93869	83573	208748	42342
fpHILIC	Negative	115.0401	0.57	unknown	136700448	119156136	158913376	165116112	121298592	153127968	149396880	142838592	145474176	146566608	148512864	173978720	128208144	81449856	101780800	121912136	110292504	134556048	102468256
EIC	Negative	276.1278	3.33	unknown	130829	102498	0	110619	98932	100997	98350	0	29249	0	0	0	98365	11703	98354	108829	55499	0	0
EIC	Negative	237.1495	3.01	unknown	248699	128667	201406	300223	174467	197553	193515	187838	158181	185286	127699	170859	150431	89104	138227	155050	131019	213785	93081
fpHILIC	Negative	268.2361	0.38	unknown	589550	755272	1375105	1617879	889190	698638	929172	885977	1289880	815862	572330	722229	1200883	580796	375933	959268	906290	813812	709186
fpHILIC	Negative	167.1078	0.43	unknown	1205889	586381	1029923	1406264	854134	683388	760094	793691	877064	924242	659806	939342	874173	437804	437024	616824	693941	888858	547100
EIC	Negative	439.3071	6.35	unknown	2155618	2859861	2979868	2309789	1529421	3060131	2287794	2897219	1581045	1881877	2717701	1100289	1624602	2486434	2372416	912541	2224670	1575912	2026320
EIC	Negative	258.1503	3.69	unknown	194527	94880	197582	194097	135091	107529	124844	201554	113488	163438	97638	90358	78549	78553	105375	112415	87111	162548	80401
EIC	Negative	480.2999	4.84	unknown	1137421	0	0	0	0	0	1289559	0	0	1131402	0	0	0	0	0	0	0	1035424	0
EIC	Negative	294.2157	5.02	unknown	417377	475778	446293	1430396	606762	580662	732331	685375	790394	590374	473371	776991	638765	334236	413198	619110	496930	882482	316936
EIC	Negative	295.2277	6.22	unknown	463700	485088	620751	1044498	590542	433694	468101	576351	698590	454325	392140	496333	446504	249064	250118	537689	434085	661610	342700
EIC	Negative	647.4915	6.36	unknown	1475341	1103635	1607635	646392	1055105	862671	968037	652506	534715	920225	673756	628669	494388	465984	387828	745341	607423	750259	785947
fpHILIC	Negative	275.1082	3.86	unknown	541205	235200	362697	406864	265584	340408	280502	290420	247348	315812	302684	181159	263994	185574	337755	142292	260995	217995	186860
EIC	Negative	530.3582	2.92	unknown	130381	45340	289191	789274	131922	44959	188299	124949	214897	314288	113670	202684	164392	0	0	187805	150681	229161	0
EIC	Negative	238.1529	2.37	unknown	802100	405374	719164	687835	450572	429263	528758	794598	329042	740677	368488	445480	394068	353186	406221	356683	328075	599699	348547
EIC	Negative	298.1849	5.49	unknown	382731	162350	445766	261255	156457	0	193395	128785	0	181094	165092	0	0	0	157148	0	0	228329	0
EIC	Negative	329.1761	1.78	unknown	3810595	1737842	3613495	1668327	984743	675255	1714254	619718	1575153	2441666	1115582	1140060	1036008	1195551	1005219	1506542	1261594	0	713862
EIC	Negative	310.1745	3.09	unknown	583539	159801	400560	191425	281931	199559	346622	469516	101713	295560	126650	159937	124595	123057	226774	197367	172595	426910	104287
EIC	Negative	269.1370	2.34	unknown	476665	239395	511548	1566398	312593	226133	262402	809728	396075	677536	158466	229400	275764	114738	113862	233342	186015	931988	153041
fpHILIC	Positive	267.0574	2.66	unknown	951409	881091	966345	1643764	1010284	1262583	952660	1031864	1200229	1079550	1075281	1076715	976324	607059	892531	1061108	994921	697142	757354
EIC	Negative	244.1896	4.08	unknown	61309	40505	81324	236418	79882	68177	95276	91087	131418	113710	71925	91602	101257	14639	34182	83502	91480	148327	18366
EIC	Negative	237.1495	2.37	unknown	5283970	2618659	4663625	4470488	2864037	2700475	3329540	5193603	2099087	4995931	2486293	2840139	2501719	2189472	2696707	2192427	2159231	3856832	2157298
EIC	Negative	239.1651	4.30	unknown	308628	172149	302783	508861	257155	154642	238966	284329	182806	252357	201459	255529	219777	89955	128298	197383	146231	331144	99286
EIC	Negative	530.9696	1.19	unknown	117296	95409	79874	67857	46317	50339	44073	56058	35272	55912	33390	23267	26758	21584	22893	21819	17449	101707	23523
EIC	Negative	247.1703	4.97	unknown	351937	253495	524861	1107989	343533	287080	416270	541974	391645	560985	307709	493952	486095	122930	164025	335204	348219	573413	178823
EIC	Negative	237.1859	5.47	unknown	110834	100259	145405	311050	106429	104781	101674	148246	176056	139055	75299	135809	106312	75024	74599	91482	129553	140767	78418
fpHILIC	Negative	274.1044	3.85	unknown	4835103	2119583	3476654	3604663	2317287	2768346	2452076	2501316	2376447	3078523	2599455	1774027	2436031	1777979	2751394	1263122	2330949	1938723	1749255
fpHILIC	Negative	583.2557	0.38	unknown	1501335	1774954	2940027	2612938	940792	693326	865933	1997581	529599	1212540	994489	1357459	1613692	260258	422657	953710	692134	1675529	319167
fpHILIC	Negative	239.1652	0.39	unknown	4123547	3307891	4740493	18524098	5270994	4228046	6536318	7495673	9217262	7758562	4684739	7743227	7663848	1973361	2156071	5819532	5475037	10514447	2526304
fpHILIC	Negative	331.2645	0.37	unknown	5082301	6119285	6367024	9554655	5631266	5777329	5915137	6943868	7273799	6121821	4976199	6468625	5599700	3426908	4225610	4202292	5730002	6976838	4395719
fpHILIC	Negative	204.9946	2.98	unknown	1320045	1041504	1156642	1185269	873333	969443	1120003	657719	915768	1023567	1054343	825494	971743	316309	511984	815863	763074	931458	652031
EIC	Negative	399.2897	3.09	unknown	156820	44349	197115	116485	40883	40358	41688	154883	46542	108046	32422	39739	44875	54832	40967	32910	31611	127772	29507
EIC	Negative	463.3069	2.39	unknown	449240	24926	159457	203658	41547	34796	39623	429853	0	141244	20300	24389	9498	32341	38224	26147	26083	325697	26207
EIC	Negative	293.2124	5.52	unknown	1784632	1526457	1954404	4463522	1810760	1262512	1303129	2319970	2581486	1704642	1226546	1757858	1747295	470818	862281	2091866	1535251	2026687	1004089
EIC	Negative	241.1809	2.65	unknown	1610910	523999	1433470	3725220	1442639	1107345	1370365	2491088	1860300	1923686	1104479	2109604	1519422	792166	618475	1858334	1075270	2505726	650767
EIC	Negative	421.2060	3.26	unknown	149752	106992	157111	420796	34145	115573	65283	31748	12420	148059	55902	27203	10743	0	74616	94141	0	41575	0
EIC	Negative	281.1763	2.45	unknown	2769954	1235082	2387916	1770299	1720862	1674428	2206581	2298720	837331	2133093	1292576	1182765	1085455	1090430	1531667	1073505	1260381	2145441	1080691
EIC	Negative	460.2845	5.13	unknown	291316	272987	209074	275713	291674	334263	184838	257723	269415	172611	218199	204731	262381	215930	200034	184252	277178	198489	226029
fpHILIC	Positive	229.1011	2.22	unknown	447563	366294	417030	563568	378397	450335	393647	437679	481317	391665	517961	471238	471604	349744	278824	428503	309422	364451	357221
EIC	Negative	498.2485	0.99	unknown	190676	163483	183662	110475	124064	176144	208229	198615	110473	156759	140468	125498	129311	110755	168799	112929	123643	122289	136149
EIC	Negative	365.1983	4.39	unknown	653365	281108	131234	238660	219289	198278	177069	115778	144243	386419	222608	212323	122098	127445	163082	153538	103184	149491	124793
EIC	Negative	254.1762	3.47	unknown	308895	145440	258826	1127004	157431	185390	169932	305203	523299	386622	220666	300391	283852	68591	118190	341087	177938	359652	92707
EIC	Negative	258.1503	3.19	unknown	324104	144666	347538	260274	202636	153120	197423	403409	111059	262369	162395	162304	147107	117946	158502	125065	157354	274791	152554
EIC	Negative	303.1591	4.95	unknown	41095	12337	76954	186134	48666	50297	43263	106880	71022	88238	44792	46689	72247	14053	16850	56455	57032	118628	11391
fpHILIC	Negative	241.1810	0.38	unknown	4237457	2990633	6108462	20546686	6446034	5214257	7814245	6728332	11396861	8444426	5691946	10082892	9088126	1989820	2596559	6832601	7027326	11462823	2669842
fpHILIC	Positive	368.2794	0.60	unknown	1277930	780157	1762031	2581870	1267415	1259862	1147293	1428420	1657360	1205221	1066045	1043882	992492	137490	816331	1671342	979757	1426209	606457
EIC	Negative	568.3625	5.29	unknown	683697	705273	689082	664898	597190	780263	729108	702001	418371	507167	516990	631279	575836	553219	599299	490374	505585	479212	551047
EIC	Negative	584.3293	5.74	unknown	326630	365096	395791	310680	317125	431083	346154	312971	207399	234611	345577	277209	274052	246461	271292	204415	312767	218674	306829
EIC	Negative	242.1843	2.66	unknown	193113	97233	230834	612162	234563	195106	220508	410741	296330	303205	178546	344768	248404	127312	107146	323761	182455	389947	101196
fpHILIC	Positive	550.3850	0.53	unknown	2580937	2118863	2727772	2333602	1966685	2871098	2254987	2300349	1437405	1977883	1815329	2073629	1662497	1593572	1763393	1467705	1956486	2031100	1602771
EIC	Negative	335.1499	2.88	unknown	412700	84569	408221	46582	47357	87182	63294	79132	57129	129396	50919	68603	70587	32844	182070	15337	27612	0	47301

EIC	Negative	409.2970	6.18	unknown	659789	477658	697185	788828	727029	351934	442703	580554	429688	626469	405558	366188	368447	462748	471161	449572	358986	417613	366890
EIC	Negative	254.1481	2.89	unknown	555159	149713	353210	226046	164975	202535	226968	255297	111516	344465	113646	188694	134413	159155	166722	100300	112018	270975	133876
EIC	Negative	336.0378	0.55	unknown	346800	157381	146853	456670	110951	366253	238044	126496	112913	148591	196141	268413	169678	119285	138821	219807	114107	177041	128730
fpHILIC	Negative	156.0455	0.96	unknown	2452923	9078899	8808009	3470334	2819608	4445902	2347839	2887544	2513912	5413932	4556717	2461879	2672119	3856049	2757595	3243524	2033733	2811129	1398774
EIC	Negative	284.1955	2.43	unknown	249971	82456	205778	180862	128792	140928	139283	265769	78841	180337	77651	91712	83634	66594	91389	82540	89978	271899	79456
EIC	Negative	463.3423	4.00	unknown	546488	0	315016	71368	0	0	0	118143	0	61340	0	0	0	0	0	0	0	96467	0
EIC	Negative	251.1289	2.55	unknown	1629874	726213	984011	650111	713909	685580	885199	1109477	465120	882996	571086	502685	557331	462091	497842	609370	510846	1023226	509997
EIC	Negative	498.2901	2.62	unknown	351632538	2939172	578220678	343794427	0	0	0	473261571	19366122	287936582	6494129	7558728	12712107	0	9903433	4993071	7478349	373459006	6627883
EIC	Negative	376.2788	6.13	unknown	345415	146790	175794	280996	140802	119088	185730	111126	150153	121874	123915	105074	133119	137300	167385	88165	124595	103457	93996
EIC	Negative	538.3219	3.00	unknown	85056	0	270949	174641	0	0	0	484106	0	94239	0	0	0	16964	23438	0	0	372420	0
fpHILIC	Positive	400.3415	0.56	unknown	3704165	2484644	3622390	6003168	3100800	2944898	3347512	3298396	5027791	3206571	3210918	3604104	3749112	1592937	2390828	4123141	2494704	3409480	2075297
EIC	Negative	271.2281	4.14	unknown	20483139	17188090	46267782	52608585	29540404	19578419	24830462	51674028	31870785	31525479	18419657	20491870	21335665	36995595	13914362	15902611	27298287	47980153	23786252
fpHILIC	Negative	169.0771	0.97	unknown	380975	925663	1023395	385273	287505	507395	233644	258569	292066	600515	416998	253601	296700	391928	300990	344217	238489	266608	142316
EIC	Negative	284.1954	3.79	unknown	224890	67165	179048	126477	106348	108556	129777	218920	57012	155484	60092	98862	70628	76221	103966	58697	81944	190913	72526
EIC	Negative	306.1770	4.83	unknown	561809	390561	664956	825683	461125	465243	593461	644819	402541	642970	359096	662048	596017	188568	328749	386665	468622	637016	280802
EIC	Negative	306.1769	2.36	unknown	206149	113888	223821	191430	10159	17907	130006	136130	67007	115631	11221	84111	160060	49818	16171	45048	9377	133339	91811
EIC	Negative	242.1841	3.20	unknown	354876	193464	567195	606998	276282	345705	293774	417854	318111	452176	248928	395875	330883	396033	157920	205604	284443	465947	238828
fpHILIC	Negative	325.0451	0.98	unknown	320068	593445	546332	1033926	901172	0	712038	273612	814804	851273	363032	898286	1427942	891118	235873	479816	443656	737415	315848
fpHILIC	Positive	586.2732	0.38	unknown	2768523	2669585	4771065	4866851	2254595	1701946	2185452	3547785	1910860	4319211	3085957	2485191	2980788	921793	1141731	2382435	1323067	3683629	976339
EIC	Negative	608.3945	6.34	unknown	8822702	6713557	6877630	4931309	5222408	8494497	7820309	6158472	4351614	5175794	5300742	6147382	5491099	5059835	6555154	4663569	4701749	4531590	5818008
fpHILIC	Positive	239.1640	0.45	unknown	804584	435388	919209	961815	674938	547816	770522	785211	425452	803418	488632	583025	652196	304713	444790	555244	415333	731905	479552
EIC	Negative	283.1920	3.56	unknown	2825429	1084147	2516308	1736024	1637958	1596511	1987343	2654126	708877	1912832	1011525	1522473	1268515	1107477	1302199	1223337	1288648	2304069	922339
EIC	Negative	502.2947	5.24	unknown	1141150	945996	942091	701180	1095803	1193164	858872	924335	462155	628826	653466	618070	513674	899023	817888	351803	774283	833152	941869
EIC	Negative	271.0929	0.55	unknown	290565	74413	101050	139904	59222	201311	87937	0	28707	162538	95873	72248	61138	53372	75392	87812	12511	0	133718
fpHILIC	Negative	110.9758	2.96	unknown	559082	191892	1154153	449197	282699	233574	262266	743088	179769	718740	182055	185741	167167	219030	143750	144466	208824	705421	193360
EIC	Negative	281.1763	3.22	unknown	5330127	1694294	3873392	2424362	2825713	2759169	3688781	4317230	678073	3226671	1666228	2149887	1607625	1741127	2493310	1725374	2242988	3834868	1416117
EIC	Negative	240.2048	6.35	unknown	160569	147746	409506	371416	223847	140373	186411	275468	255933	221367	138096	195155	212762	200516	45732	204690	226582	227327	183867
fpHILIC	Negative	270.2519	0.37	unknown	993410	988294	1517687	1666489	1010472	1294105	1138165	1293452	1430236	997003	796227	1217205	1144643	1009123	835116	1006707	1104447	981113	1135073
EIC	Negative	412.2790	5.73	unknown	431021	321065	413049	1277219	175478	188783	325448	541608	293425	426412	417373	167190	370113	485951	432611	62951	398660	216036	141545
EIC	Negative	269.1761	3.34	unknown	569916	370470	422891	310575	309858	421298	338851	253556	545836	346726	264236	425800	484993	374873	339367	462688	335186	250831	146923
fpHILIC	Positive	113.0539	2.22	unknown	553227	358170	461411	575238	348530	554438	343732	464338	504860	493581	413300	452584	456162	405275	336703	423178	324454	377876	358366
fpHILIC	Positive	548.3698	0.54	unknown	3932834	3349640	4289689	3151042	3320290	4275461	3667317	3491179	2134572	2820715	3218449	2992491	2591387	2648721	2708827	2116701	3085475	2786307	3178772
EIC	Negative	268.2002	3.56	unknown	365563	195592	665419	608809	277287	295165	227458	559469	218369	512057	218263	251919	185582	469497	98413	103465	237672	582278	274950
EIC	Negative	241.1809	4.08	unknown	33809135	28855861	43697679	157642868	58655283	44713267	67818167	62672064	95933225	76524529	53581357	81419807	77506030	16518569	24761770	65180040	62320859	98080484	20901473
EIC	Negative	484.3734	4.08	unknown	257058	210392	528006	6183181	852658	468164	1223381	1002400	2294631	1423005	740033	1719825	1543668	84471	149289	1081085	1015004	2452700	107048
EIC	Negative	322.2477	6.22	unknown	301270	132605	335663	537044	216312	197094	122502	299039	193248	330602	144229	190512	166624	96527	93030	152548	131472	352151	116983
EIC	Negative	328.0002	1.25	unknown	613735	578484	675605	402240	592867	621735	614572	580989	571948	591649	630770	244741	508136	598652	576720	697226	395973	219432	573225
fpHILIC	Positive	440.2058	3.12	unknown	472972	182353	189470	547286	221220	578852	163000	121690	204102	245629	226643	225137	274205	68872	171936	289203	244391	169353	186778
EIC	Negative	479.3374	3.30	unknown	749146	54844	726607	382187	0	0	0	522512	86503	203659	0	0	0	42023	0	0	0	501746	0
EIC	Negative	445.2958	2.86	unknown	946523	397412	763292	715982	392971	451735	437761	869997	507351	711776	345709	381838	312171	660245	424968	255030	391522	794902	325478
EIC	Negative	269.0878	0.65	unknown	519225	171510	215232	664465	86981	482040	250329	105067	114557	212795	248053	296808	216564	44486	136931	237146	122044	209611	150488
EIC	Negative	419.3165	3.07	unknown	272293	0	214921	65197	12056	11409	12085	279571	12096	70999	0	0	0	12082	9087	0	9438	251216	0
EIC	Negative	581.0430	0.55	unknown	548629	496484	318631	1015424	242623	761788	586854	384042	244419	265167	543211	733004	486302	135578	378731	460428	337315	400740	253008
fpHILIC	Positive	232.1399	3.97	unknown	665697	512802	384137	675226	456151	664947	399466	372999	413855	436840	418094	445712	511791	340803	395231	362705	403115	291698	449830
EIC	Negative	283.1919	3.63	unknown	1384541	417067	1212111	866119	646738	700952	774172	1160349	280373	911185	369150	606960	450023	452159	542289	463377	484957	1055023	392063
EIC	Negative	277.1808	2.85	unknown	4889033	10601614	10828848	8134771	5548163	7773199	5889559	7677415	3351593	10988534	9886657	6271581	3206144	2744899	5101374	4602285	5596768	8111170	1532759
EIC	Negative	354.2347	4.14	unknown	295011	250176	548532	585406	409587	272529	321791	631175	445443	423924	248524	241362	246177	482579	199846	198479	359805	630252	328363
EIC	Negative	299.2591	4.63	unknown	0	325435	848726	1110471	587216	0	520390	0	524520	0	0	0	0	0	380319	0	0	966875	0
EIC	Negative	505.0078	0.55	unknown	256973	63267	52204	158842	0	222345	72435	0	0	45938	71142	99259	60060	0	66256	89628	40262	0	47183
fpHILIC	Positive	120.0901	2.28	unknown	4328337	3248620	4821318	3737820	3473255	4119861	3689533	4435763	2908578	0	3635208	3084989	3076544	2345177	2714902	3019094	3233738	3850828	2558248
EIC	Negative	364.7810	0.52	unknown	119980	78025	142713	109235	176003	86556	101097	47739	112225	158312	58401	33528	66432	361933	94726	74897	52461	77302	132929
EIC	Negative	296.2313	5.53	unknown	813764	877681	1218160	3052781	1573608	1027939	1325306	1308017	1674067	1493193	970251	1586631	1282635	599425	618069	924312	1304689	2092627	765030
EIC	Negative	501.0265	1.19	unknown	173221	172359	130983	121195	121256	136767	133074	105559	98198	120442	105571	103817	86424	86671	94076	75618	90003	126894	104598
fpHILIC	Positive	587.2759	0.37	unknown	633779	565647	1010652	1029987	487040	376676	532917	721882	432863	903405	728005	609189	652566	186352	251711	528161	294827	848658	184318
fpHILIC	Negative	353.1733	2.70	unknown	212596	215707	160776	186937	230356	299863	200216	66538	59850	170457	60194	172727	183166	104591	104189	56129	70232	139722	337544
EIC	Negative	375.2165	5.52	unknown	88859	101485	121038	262451	102730	41015	74296	117186	142368	101194	96326	96517	116879	0	57659	125398	69164	113007	29452
EIC	Negative	609.3976	6.34	unknown	3122730	2306454	2262565	1571655	1698126	2839805	2727024	2009614	1432527	1759544	1630057	2283341	1808697	1714583	2385235	1547826	1403755	1478728	2034913
EIC	Negative	272.2315	4.14	unknown	3655602	3040024	8233794	9360104	5281754	3511552	4489419	9177680	5679237	5602734	3243153	3648170	3850989	6624516	2503133	2845881	4847658	8756171	4309078
EIC	Negative	297.2435	5.24	unknown	2003312	1760196	3177616	3934892	2437330	2241974	1888382	3543832	2325505	2265535	1566862	1719147	1783230	2883005	1029646	2087515	1920700	3107030	2136399
EIC	Negative	447.0467	0.55	unknown	690953	66012	77573	568993	0	519799	120180	0	0	202783	127370	155001	92009	102093	122642	180307	0	90483	136212
EIC	Negative	409.3068	5.98	unknown	484022	293713	599715	1170496	375993	265055	417385	517795	488228	396030	296014	365975	440753	155888	254853	367188	323893	570673	192502
EIC	Negative	268.2000	3.99	unknown	93450	43413	76707	130165	66112	63227	70714	83206	88088	86304	52121	69421	65840	39512	51618	64860	54511	102093	34366
EIC	Negative	253.1810	3.79	unknown	234428	91079	255197	187471	82835	103114	94254	219151	50336	182353	79394	110016	102827	81264	87190	56415	79235	135062	97245
EIC	Negative	577.3350	3.72	unknown	594220	1055657	905768	707388	712108	1027654	783709	775702	179364	103914	1009928	749114	197230	1483652	763365	234078	198586	641824	1043371
EIC	Negative	603.0169	1.19	unknown	100021	102351	89621	80943	80454	72540	66587	74860	70840	75995	62867	63429	59066	58435	59402	54579	56729	74276	57742
EIC	Negative	468.2720	4.26	unknown	961819	943727	996701	809285	843369	984191	859667	861429	592558	821925	810050	655542	646581	728567	667118	626832	667691	756848	713849
EIC	Negative	392.2240	2.48	unknown	405057	265213	502948	431152	251175	336554	393144	339287	0	0	314167	0	337957	301471	326400	149073	323531	0	295370
EIC	Negative	235.1705	4.85	unknown	255953	185796	370050	905341	173347	150141	174047	260396	189304	284005	139481	595390	182295	50785	193942	195542	126987	218637	140442
EIC	Negative	243.1863	4.08	unknown	569474	491236	780702	2719562	988443	743245	1187608	1081393	1676383	1346140	894674	1403956	1314658	275067	404468	1119338	1110453	1720794	355203
EIC	Negative	294.2156	3.77	unknown	192064	141642	251720	232121	176779	170762	191462	203733	141210	185962	115144	129584	99609	278976	67461	133138	146654	245439	140859
EIC	Negative	300.1687	3.24	unknown	71956	66720	96145	63449	24935	47691	40775	26539	25414	32279	38132	69621	27822	7398	36199	37273	26269	26152	11331
EIC	Negative	359.1458	3.88	unknown	208754	247423	248714	470300	254467	170032	218838	292118	287088	263675	389565	284365	239664	131425	112362	185703	176294	372360	168121
EIC	Negative	496.2323	0.84	unknown	226774	209766	344460	169388	172935	228773	269114	319770	154054	254982	236123	209995	145220	161715	184023	146157	195289	237479	187332
EIC	Negative	279.1601	2.52	unknown	274660	179785	337188	256379	169207	204902	228053	225892	106120	216482	123337	115601	147280	53219	172709	111501	150817	215544	125144
EIC	Negative	273.2342	4.14	unknown	422624	342499	969903	1100521	611938	418626	515015	1057202	669180	632276	388138	439890	453810	768085	293783	323906	589816	1021404	488486
fpHILIC	Negative	198.1579	0.41	unknown	1143987	343945	910018	1461422	599918	629470	765056	774293	368142	640754	344050	610056	542069	112304	298139	513120	577936	842063	371315
fpHILIC	Positive	193.1543	2.07	unknown	771229	387271	559429	302479	326348	790717	530794	256162	390141	375991	472716	464638	933289	75032	480334	437002	242256	337595	410468
EIC	Negative	263.1654	2.77	unknown	722475	304402	571808	392810	361074	577994	459230	647664	119270	719459	296996	339307	224354	271382	304674	165352	274281	685839	371583
EIC	Negative	636.5272	6.46	unknown	7532524	6445736	4784151	5963228	4116864	5116691	6608417	6299053	4855638	6819803	5408877	3466321	4763700	7490935	5953655	2087454	6457716	4968304	3001276

EIC	Negative	407.2928	5.10	unknown	434356	246145	519508	982252	363621	196858	298304	428281	420919	421815	216820	293058	284806	192867	233049	264331	271717	520673	132194
EIC	Negative	309.1710	2.85	unknown	10594785	21912611	22422740	17471736	12372544	16688764	12892448	16281796	7721092	22924449	20747721	13606738	7514712	6632260	11343385	10136016	12021042	16910406	4574618
EIC	Negative	320.2314	5.66	unknown	184279	163188	207946	643805	223828	219699	231216	317049	389463	256151	177095	295513	277722	98574	157090	267582	205531	390655	130726
fpHILIC	Positive	127.0979	5.42	unknown	3814637	3575607	2296300	2489268	2838797	3635705	3338285	2220289	2708181	1788617	2271663	2461375	2657415	2571175	4330420	2635091	1478353	1679615	2167648
fpHILIC	Negative	298.1419	3.48	unknown	1750416	1297062	1648930	1776570	1627901	1733526	1414783	1727261	1273643	1744138	1681913	1719262	1736758	1267117	1538133	1552561	1669494	1724749	0
EIC	Negative	377.2329	5.53	unknown	261518	285246	394674	851413	472317	332229	395592	395146	546405	521915	310826	485014	458477	175838	205191	300054	426762	628269	225811
EIC	Negative	241.1808	3.20	unknown	2369471	1205752	3579957	3836356	1801267	1962798	1802609	2740905	2015711	2835198	1630621	2505937	2052693	2522212	990185	1331738	1853576	2864884	1575374
EIC	Negative	233.0424	1.25	unknown	192754	173511	157219	149794	168743	173735	184339	149600	157729	175284	192612	106756	158515	153183	164826	175930	148958	32863	173624
fpHILIC	Positive	585.2701	0.37	unknown	7647882	7241932	13204343	13777293	5946409	4896655	6067090	9729743	5517474	11709050	8801712	6898748	8090993	2705250	3044603	6649715	3601867	10785249	2763524
EIC	Negative	239.1652	2.91	unknown	3833906	2511479	2510949	6087932	1996612	4931816	2419888	1970986	1028441	2720491	2169664	4387048	4476897	2401268	2824018	1660132	1922850	2294394	1607812
EIC	Negative	506.3264	6.39	unknown	2661141	3070283	2625516	2099542	2053632	3051310	1950617	2222630	1254871	1472546	1303353	1810528	975198	1744093	1397006	1503886	1187281	1800857	2906315
EIC	Negative	311.1772	2.85	unknown	431869	617307	613289	470748	380212	478513	366823	489028	195942	643132	564690	390978	227751	193073	311244	263537	360070	500449	160179
EIC	Negative	355.0106	1.19	unknown	73059	68705	54211	44104	50499	52703	48809	46536	47346	57670	42695	40725	42187	35193	40452	33239	33569	61025	37114
EIC	Negative	449.3281	3.74	unknown	4709887	616122	8491566	1370123	792086	441779	426406	2541227	584314	2140249	366771	307422	238213	461736	363654	404541	410324	2445461	376166
EIC	Negative	295.2279	5.53	unknown	4026258	4687660	6145499	14975376	7826284	5274498	6434875	6573885	8354439	7517605	4772889	7605876	6316591	2995725	3354541	4784174	6294927	10575399	3801499
EIC	Negative	337.2137	5.49	unknown	1068524	338845	750237	829993	421357	320075	528230	423929	406284	647446	529485	296014	401365	609795	741074	81202	476637	276623	180297
fpHILIC	Positive	510.3555	0.59	unknown	4791543	5345612	4348803	4676826	5232834	5615582	4771569	4593423	3603735	4088493	4559877	4679246	4672960	4266631	5181100	4939741	4693457	5108428	0
EIC	Negative	499.2932	2.60	unknown	104379958	890169	174039083	102404763	33069	38109	21548	140533913	0	86062794	1978188	2230345	0	0	3057384	1540970	0	111478449	1592474
EIC	Negative	445.3329	4.06	unknown	132067	12725	315145	84037	0	0	0	146393	0	126721	17793	20763	0	0	12899	13309	0	81157	15196
EIC	Negative	500.2913	2.61	unknown	32223428	241920	54246976	32116963	30630	12428	48958	44422816	0	27612082	624781	692825	1228702	0	935099	484337	724493	34621887	513609
EIC	Negative	443.2411	3.49	unknown	162474	93860	135038	403726	110715	119272	103210	169231	124983	245519	107409	137595	171995	29248	49585	128427	101909	218090	27711
EIC	Negative	319.2640	5.96	unknown	486264	407742	538045	507159	416263	268652	336716	432086	348758	583833	398536	250283	278596	197970	397608	282668	254661	364973	210991
fpHILIC	Positive	794.6088	0.43	unknown	1531860	1475069	1613438	1592598	1371058	1430519	1212965	1718203	1640698	1232235	1357400	1138156	1089298	1078385	1106300	1236413	1260526	1388046	1193420
EIC	Negative	240.2044	6.30	unknown	241278	273773	445718	773805	437843	226678	318616	443452	425646	341391	235054	280748	371830	279508	81825	296163	387488	533728	254515
EIC	Negative	583.0304	1.19	unknown	71932	84419	71203	53074	63909	70538	62189	49410	54243	60721	52248	50159	51945	42248	47657	41357	51255	48883	62786
EIC	Negative	281.1765	3.45	unknown	812541	284292	743728	464801	465070	422961	571439	806149	188837	548677	283279	452319	337264	341106	400152	308867	390689	715642	243968
EIC	Negative	316.1999	5.64	unknown	686062	429663	635762	687982	568182	398476	421008	624674	472424	584817	487957	464265	473790	449003	422268	393367	417729	491955	360448
fpHILIC	Positive	169.0352	2.98	unknown	741323	451372	662115	870045	391197	524184	533318	186036	340643	379703	466662	637461	509019	133299	238275	426564	390902	331923	270639
EIC	Negative	388.2791	5.91	unknown	625790	244679	436812	446008	252944	312159	365149	265328	193809	344356	367683	213032	239907	390403	473042	19365	331614	194502	147968
fpHILIC	Negative	88.0121	1.03	unknown	9456632	6195359	5128376	3490089	4409488	6244990	6950319	1449020	1237496	4274014	5776139	3473912	2096358	4639194	4380392	4563785	1928944	3358961	2804112
EIC	Negative	407.2809	5.32	unknown	466427	375460	525977	376734	460015	303824	436619	441662	412352	429223	364556	297456	334488	324727	385391	397826	324469	333339	284501
EIC	Negative	346.2470	5.83	unknown	255069	289452	298952	778716	294399	299069	310386	395917	470485	378368	277719	236720	510727	185198	155156	365119	398496	401014	167153
EIC	Negative	285.2435	4.74	unknown	558877	516530	1107911	1266257	842808	560441	635749	1218076	700407	728425	449792	560024	538375	797045	445332	551540	570482	1119219	638131
EIC	Negative	265.1811	3.39	unknown	476989	179090	573285	417047	237038	299346	258698	474504	157539	455441	238903	314267	238598	241779	184633	160607	182074	431354	240543
EIC	Negative	339.2177	3.08	unknown	111275	45115	157463	75039	46966	62266	66419	68661	38929	78722	37843	33435	37272	26215	35905	55127	21373	83115	42522
fpHILIC	Positive	232.0914	3.35	unknown	511481	284197	420332	443531	424594	299245	330076	371318	358665	315427	454135	278605	260289	319851	0	310326	343222	404398	441348
EIC	Negative	278.1840	2.85	unknown	841132	1958731	2027589	1471377	1009541	1440893	1095766	1399664	627439	2013147	1850315	1208933	595471	499769	970134	851081	1038371	1503426	324751
EIC	Negative	419.0227	1.19	unknown	82908	79562	54392	62224	55043	63910	49006	44342	35637	54154	49597	48561	29988	36548	38326	37721	36018	60287	36241
EIC	Negative	526.3154	4.05	unknown	664082	740424	635867	518310	557550	720077	652386	578016	446825	488635	553399	559894	581932	613823	569536	506061	491762	448233	470738
EIC	Negative	526.3154	3.64	unknown	2866034	3124893	3048715	2960253	2423798	3687857	3327332	2629917	2452408	2608236	2753064	3219338	2746947	3808417	2827870	2609321	2303715	2171872	2310521
fpHILIC	Negative	360.1230	3.34	unknown	3706960	3281150	3547248	4130820	3628468	3604150	3898922	3226699	3250642	3310279	3919218	3549484	3597755	1933647	2985622	2839537	3183934	3021542	2793608
EIC	Negative	527.3190	3.64	unknown	725149	828938	931114	782873	661733	1013253	939292	751647	718628	714294	745055	906688	757347	971166	744420	736918	585072	769315	602927
EIC	Negative	635.5241	6.46	unknown	17462892	15294210	10982586	13249806	9367818	11822821	15085570	13927618	11039027	15540230	12567732	8151865	10610017	16816430	13759115	4956909	14789225	11356571	6707061
fpHILIC	Positive	487.1773	2.61	unknown	2119036	797477	410937	2092656	827685	1421382	629770	765837	1984292	929843	333285	1312421	1000247	490242	507349	1695991	712458	1113525	710727
EIC	Negative	501.2928	2.61	unknown	7429882	51751	11733309	6870601	27235	43988	9000	9787516	38137	5742286	143212	156959	218212	254473	194509	117022	123514	7767320	141914
fpHILIC	Negative	232.0512	2.92	unknown	381616	464266	427983	413482	312931	552269	387505	368569	259323	409319	578777	510834	382345	287663	299520	353897	325113	331440	276789
EIC	Negative	644.3552	3.78	unknown	117683	12246	31290	77372	28829	66695	31795	46167	24797	49823	26910	71848	0	14421	41935	33677	39345	0	52578
fpHILIC	Positive	223.1692	0.37	unknown	677144	444011	825577	778168	529108	415831	562194	629559	391482	554673	409070	508678	399223	377868	399861	374182	359271	583597	419515
EIC	Negative	309.1338	1.57	unknown	571753	395423	309331	168998	259189	195973	72853	259579	130134	420142	51051	215323	218729	222421	60111	207652	139892	149475	218536
EIC	Negative	485.3766	4.08	unknown	50276	45852	71196	1150888	126236	105004	217191	199777	427922	377528	136135	314674	320593	0	30688	211311	191838	457454	18733
fpHILIC	Positive	327.1571	2.78	unknown	42024900	37252656	39231060	32755652	32828954	38733512	30178644	33223020	30300522	36420912	36309296	38536832	33682504	39176292	0	33622332	32180150	37565632	36465620
EIC	Negative	309.1710	3.08	unknown	2933524	911432	2327424	1203250	1516864	1126821	1959461	2613763	528399	1665964	763040	971298	757243	756631	1247241	1175645	1095535	2371764	664554
EIC	Negative	435.2405	6.30	unknown	969467	406209	629570	984566	478534	1006700	879185	838171	291629	499455	333645	772865	465520	333083	602041	373527	525541	1095150	361709
EIC	Negative	387.2755	5.91	unknown	2638511	1089298	1846638	1875113	1091879	1404087	1653887	1057901	785211	1473812	1604380	964483	1002266	1673907	2014418	286456	1444123	761498	569327
fpHILIC	Positive	119.0896	2.28	unknown	62104112	45291032	57890460	50040108	47446676	54719660	51146424	55549336	38921320	54920880	42976812	44752084	43087108	31778148	39335592	40162412	42932004	52088844	35378312
EIC	Negative	425.3292	6.35	unknown	1176476	1315774	1573617	1520087	624443	510297	753578	791276	1247295	1072329	516155	837424	753181	393569	692251	882203	720401	597500	434127
EIC	Negative	263.1654	2.86	unknown	512364	258236	532070	297821	314495	341222	329152	554224	124476	470563	282638	264166	200423	252078	251088	181193	205681	490645	280627
EIC	Negative	277.1440	3.88	unknown	3697867	3502330	2176821	3847272	2565687	1894267	2445912	2469826	2816302	2346413	7156702	3694194	3641373	2139689	1714987	2128281	1823810	2553318	2109436
EIC	Negative	338.2089	5.23	unknown	1052824	0	156160	780196	0	36085	0	1038925	28326	181380	192384	0	128450	0	67502	269624	0	191015	406714
fpHILIC	Positive	276.1804	2.00	unknown	322958	170908	240389	508169	169647	305396	241573	194508	465340	233981	241148	275307	316094	99133	181225	322379	168368	313736	180785
EIC	Negative	323.1853	3.34	unknown	356475	141649	334364	180141	230969	195304	273429	358579	97294	216741	127809	210148	164065	148393	184504	167786	189317	316169	121846
fpHILIC	Negative	325.0036	2.05	unknown	539909	792682	691911	666666	570707	970402	486529	435311	378268	723071	728338	597341	465968	310863	600897	455042	395333	436101	522915
EIC	Negative	552.3313	4.17	unknown	52040011	76932112	59737164	47602828	55723738	62640776	73291946	50982850	63981722	42955628	55595646	59436929	67477367	66417462	57415250	59725533	50735008	42066275	50309613
EIC	Negative	249.1860	3.29	unknown	2308330	1538963	2367786	2154134	1625599	1084159	1552105	1718982	1390636	2545455	1505996	1322081	1187518	995140	1632240	1323224	952434	1406656	944793
EIC	Negative	339.2186	3.21	unknown	306973	81863	248839	128716	123328	108127	154408	248522	0	153952	72133	98086	82335	76116	90998	71882	99719	202816	73282
fpHILIC	Negative	371.1662	3.72	unknown	841409	650341	697775	608856	781241	780293	639941	616645	441236	566087	594207	503529	561179	408041	692391	578406	504522	566107	429193
EIC	Negative	334.9594	1.19	unknown	73580	55475	59740	51889	43729	46450	38003	33849	40371	53381	37211	24208	27400	34377	30174	25012	24339	49502	43043
EIC	Negative	475.2666	4.10	unknown	627391	645560	565761	644418	560118	533347	521515	534569	483868	296415	433167	438813	338931	710655	535620	269594	439978	473089	520741
EIC	Negative	321.2439	3.76	unknown	110543	0	154964	0	16341	52588	0	51713	0	118741	16488	32792	12568	0	12815	13570	19352	41308	15548
EIC	Negative	350.0101	1.19	unknown	78955	74537	65653	63738	67168	69013	62671	63677	65013	52840	56078	54622	56984	60578	58024	52589	58691	51282	57961
fpHILIC	Negative	269.2120	0.39	unknown	899332	866161	1672937	2660537	1367858	1003965	1277509	1632500	1839021	1172510	762925	1492392	1283925	777537	469745	1529939	974202	2006570	1078230
EIC	Negative	483.3697	4.08	unknown	869025	631890	1425451	19925838	2810441	1542720	3617446	3171726	7180255	4520018	2254895	5460944	5015909	216123	479508	3523234	3237534	7986568	363569
EIC	Negative	553.3346	4.17	unknown	15387291	22449769	17487377	14153114	16355171	18237346	21878559	14898619	18716561	12514633	16466775	17721874	19842019	19585103	16961809	17569732	14905065	12437508	14832332
EIC	Negative	252.2047	5.75	unknown	130606	83198	192882	362397	118497	107044	87915	189639	115629	151435	78289	99987	116553	57162	64779	96819	90215	207806	67025
fpHILIC	Negative	349.1838	3.71	unknown	3530959	2548119	2407855	2363426	2973918	3430096	2628252	2230754	1615417	2042654	2214189	2013727	2092263	1478066	2687799	2487111	1877823	2306220	1607217
fpHILIC	Negative	252.2051	0.38	unknown	440040	270781	905118	1517892	415830	362258	315330	482397	1303774	663750	220821	578726	785299	179019	247061	315160	585400	817914	486565
EIC	Negative	267.1967	3.77	unknown	2336002	1076260	2610562	1697925	1105368	1163588	1335017	2042877	525544	1820469	1103512	1188534	879208	996286	1022810	715114	652714	1823514	1042535
fpHILIC	Positive	334.1387	3.19	unknown	1485452	982223	1011030	1126981	816845	1412304	980172	716579	851740	726322	764975	607592	734657	827211	820148	983670	648276	974632	643045
EIC	Negative	373.2058	6.30	unknown	922757	772380	951412	1203959	804538	482850	726500	860221	837046	721929	533574	550310	713069	515363	392162	686431	686087	777107	620712

EIC	Negative	359.2206	6.30	unknown	3507741	2702638	3747857	3065577	3190465	2318695	3287806	3120255	2810355	3024320	2263609	2359371	2775485	2303972	1769597	2711978	2828471	2799538	2447737
EIC	Negative	395.2440	3.45	unknown	177586	136463	313433	511463	159164	173696	263834	339695	323953	241438	168115	268115	228112	187000	94282	248385	223499	371863	138001
EIC	Negative	451.3447	6.51	unknown	500494	530994	636321	816199	538223	382243	597150	506168	588937	529470	527419	523725	493471	331368	419384	534712	505565	443618	327453
fpHILIC	Positive	701.5586	0.48	unknown	1840866	2118972	1697155	2033773	1961204	1582951	1338830	1666753	2341282	1639807	1716288	1578410	1712840	1705661	1555111	1608029	1587739	1298444	1546167
EIC	Negative	463.3066	6.09	unknown	475201	0	0	1514992	0	0	0	627713	0	0	0	0	0	0	0	0	536339	0	0
fpHILIC	Positive	231.0978	3.86	unknown	618919	243722	458431	416555	298905	341121	336959	292641	293769	429867	326441	226386	337672	237781	328362	201009	311447	230987	264958
EIC	Negative	563.4309	6.70	unknown	79936	67616	115478	189921	117081	103760	85807	113848	84694	152416	98418	122109	88170	97257	102274	86833	54805	101487	86785
EIC	Negative	412.2787	5.59	unknown	1153241	841378	980192	1727443	476942	561561	868397	831358	721606	906158	1063988	390871	698356	1038334	1149694	188711	918384	332359	372622
EIC	Negative	310.1745	2.85	unknown	1981006	4158429	4240761	3123930	2303837	3172936	2441674	3014742	1431891	4254729	3888174	2551166	1435592	1223133	2246066	1948423	2292605	3237844	860330
EIC	Negative	439.0101	1.19	unknown	600073	555511	472211	422113	397904	421651	429921	379111	351277	397318	345142	326395	288369	296214	317256	284810	295029	461061	336963
EIC	Negative	319.2277	2.15	unknown	387809	0	367196	0	0	0	0	458709	0	0	459152	404120	0	422159	0	0	0	392781	0
EIC	Negative	315.8959	0.52	unknown	98875	115877	147696	138186	107269	123841	120971	107754	136886	99271	103529	109035	106889	40091	94667	106703	97356	124623	88248
EIC	Negative	264.1689	3.06	unknown	448834	367642	395574	314955	317635	407991	353189	411419	215389	309589	303654	329588	328159	252966	291656	286207	266646	344455	273508
EIC	Negative	458.9978	1.19	unknown	199098	199243	196430	175449	149042	137584	143866	166193	144891	154804	127140	119148	124101	116012	117097	114585	116346	173759	114594
EIC	Negative	345.2436	5.83	unknown	1002153	1272729	1256654	3165200	1137116	1189712	1333493	1609336	1985621	1644884	1123025	992800	2026631	812778	700490	1477447	1598912	1718865	731513
EIC	Negative	287.1659	4.94	unknown	37262	50964	77136	197145	54973	22755	24274	68800	78450	103559	39521	24104	84987	0	11428	72939	42014	87565	0
EIC	Negative	358.0100	1.19	unknown	190159	159128	143413	127687	119244	128911	128843	107715	102427	130160	104170	98337	84297	86700	90290	81387	80219	150782	101341
EIC	Negative	297.1241	3.43	unknown	150939	147034	202873	539730	236214	182185	230005	292559	337050	321730	206031	314255	266285	97069	110723	233912	201064	416713	135114
EIC	Negative	323.1848	3.20	unknown	224253	118166	299310	379473	177891	200639	171894	285071	207672	275888	162531	217027	176090	220989	101795	186797	164421	294953	148144
fpHILIC	Negative	285.2708	0.37	unknown	2522831	3181194	3171808	4239154	3300171	3436760	3239482	3130252	2647815	3212719	2130179	3379521	2569985	2673409	2022808	2444328	2643461	3197584	3233073
EIC	Negative	315.1967	5.64	unknown	2955337	1983492	2950138	2956464	2491869	1675094	2023346	2796599	2129182	2839995	2156151	2094183	2155786	1864458	1834671	1906079	1968480	2183444	1661879
EIC	Negative	648.3470	4.77	unknown	297944	237880	264288	199118	229732	228709	268018	236452	152687	263922	192752	119627	197301	199270	244221	241737	133675	145221	194105
EIC	Negative	238.1528	3.60	unknown	110706	69305	120821	174014	78233	85073	96660	112552	59317	90905	82873	104903	100365	43628	63653	83763	61677	111658	50696
EIC	Negative	415.2437	6.12	unknown	431408	221031	289937	617964	222426	329393	320990	301282	377297	429847	277932	154932	243417	240139	344242	144300	382333	285173	178791
fpHILIC	Negative	89.0131	1.03	unknown	1850649	1311747	1069228	820836	870789	1240350	1344269	291761	244090	823595	1316549	633474	472465	976140	913036	977669	369108	560034	614989
EIC	Negative	282.1798	3.22	unknown	967459	330168	685332	405020	502379	483496	638303	771714	132630	569188	276850	392069	335159	302458	445704	340635	431765	689157	271268
EIC	Negative	515.2876	1.50	unknown	0	4735905	287233504	219313072	7610405	9055021	3380116	411822464	49251946	340532512	12225578	6834510	64390080	37629820	19382873	9121758	15079081	359981952	11085849
fpHILIC	Negative	243.0622	2.65	unknown	15198453	12751750	12328033	18522126	13574961	17232436	14633676	11261107	12947646	16155539	16607228	14745769	14172063	8270405	14021023	12366342	13272043	10075137	10297265
EIC	Negative	314.2421	3.32	unknown	1035551	741801	1873716	6594999	1249932	1023141	1428969	2591697	1969386	1960417	1128529	2007560	1834110	745224	495905	1761909	1485396	2715220	664593
fpHILIC	Negative	297.2436	0.39	unknown	2626494	2053406	5514902	8172422	3372114	2677241	3844128	5024866	4361195	3153443	2089918	3817266	3012453	3017793	1759377	4140581	3612331	4834122	2273301
fpHILIC	Positive	160.0965	2.71	unknown	2532251	1549994	2189136	2654107	1332271	2480974	1737643	2029401	2163526	2061402	2237786	2163709	1750247	1426654	1437300	2109033	1418542	2170352	1284984
fpHILIC	Positive	251.1024	0.96	unknown	3293522	1279400	2224174	3685919	2209462	2467441	1756956	1584744	1616479	2343638	2374591	2173848	2192384	1790200	1331280	2068991	1584299	2192880	1151748
EIC	Negative	417.0367	0.55	unknown	2355440	1375011	1251304	3306365	873485	2606050	1754580	969848	840311	1162540	1665319	2023976	1383624	638894	1197553	1568207	930591	1387728	1007590
fpHILIC	Negative	359.1195	3.34	unknown	26471036	23045224	25683850	28673164	24714362	24368254	27524196	25100706	23572940	24712276	27544494	22778162	24767636	13842757	21161274	21713564	23347486	22165980	19968530
EIC	Negative	367.2142	4.14	unknown	91196	81780	147103	189915	113788	77766	92417	187182	124009	130916	79658	83373	84117	146694	70271	71761	106602	165346	101360
EIC	Negative	554.3374	4.17	unknown	3134375	4610465	3604091	2986469	3455003	3809213	4454074	3103455	3987826	2653483	3472368	3663257	4095641	4061012	3558279	3750359	3153154	2697242	3130253
EIC	Negative	325.2657	4.15	unknown	202744	206669	193258	199623	199760	202988	199994	175103	176917	160915	200733	155289	174743	176450	173110	160337	177476	156425	159755
fpHILIC	Positive	831.5723	0.43	unknown	1286176	2364012	2305663	1684181	1977417	2360016	2506298	1773307	1355409	1173067	2053070	2026144	1965964	1885382	1530925	1812416	1860626	1262019	2174319
EIC	Negative	297.2436	6.27	unknown	4976633	5397773	9276877	24278629	9900755	6333294	9557475	10700820	12271779	11446867	6558148	10694572	9027802	3686949	3689234	7209081	9330846	17250776	4772396
fpHILIC	Positive	265.1762	1.66	unknown	56513	817145	418537	467157	336350	732252	466508	245822	502687	384076	603781	508788	343179	543955	579415	585389	230332	272533	311325
EIC	Negative	294.1793	3.29	unknown	1083313	706996	1064864	942994	841938	511683	710452	784011	627932	1124127	699620	630607	552998	464851	759089	598698	485854	717940	452363
EIC	Negative	335.0337	0.55	unknown	3419767	1714355	1566417	4319237	1119758	3577704	2247286	1179519	1050785	1517794	2023742	2504130	1723478	897362	1541518	2089217	1245646	1715035	1369545
EIC	Negative	379.2473	4.51	unknown	315394	187730	608359	1682774	386405	272137	335053	936759	649502	626427	331403	693548	518067	224589	182742	653758	391333	783580	227846
EIC	Negative	606.3688	5.01	unknown	5930138	8106892	6691896	5477187	6968399	7508375	7361821	6144696	5482500	5150421	7583701	6546046	6944077	6360285	5629956	5555910	6641640	4410813	6809658
fpHILIC	Positive	371.1671	1.81	unknown	1113307	512540	872940	1326651	612507	905408	576001	774541	756920	1007823	667791	742738	635435	400051	631077	741339	548894	759902	466924
EIC	Negative	416.3004	6.25	unknown	962211	583426	799156	972343	352732	578678	572163	730222	513494	647776	711794	464246	517103	677969	674870	59125	561663	570867	519202
EIC	Negative	245.1552	4.23	unknown	133068	75090	197344	399605	71362	87329	99757	157149	85692	171793	72680	104285	149291	24273	41998	88834	97292	148559	58937
fpHILIC	Negative	310.2833	0.37	unknown	1577561	2566790	3563026	3715332	3362005	2271494	2432741	2283728	2299301	2185474	1807725	3720714	2539588	1374732	1627766	2572998	2020369	2999940	1773734
EIC	Negative	293.1762	3.29	unknown	5558029	3815555	5678507	5222291	3979354	2653057	3716176	4135393	3299107	6159829	3678664	3209466	2998021	2484963	3977799	3164922	2402811	3469264	2442781
fpHILIC	Positive	584.2625	0.37	unknown	940642	755290	1016696	936069	562332	577254	591871	718493	426285	940026	828911	693022	767285	269270	360542	656767	418381	777100	287546
EIC	Negative	474.2630	4.10	unknown	2360169	2502696	2234798	2407128	2076665	2193132	2051191	2063647	1900099	1187974	1683832	1682001	1295927	2732052	2100053	1045149	1819909	1875431	2102368
fpHILIC	Positive	469.3118	0.61	unknown	1598086	1238227	1551998	1187386	1148122	1075243	1820140	1571378	814161	932110	1205803	1138035	1211568	1067121	1326400	1163350	1161650	1076943	863229
EIC	Negative	376.9945	1.19	unknown	763316	709802	692250	622917	531421	525726	496076	584571	489215	568178	456741	434069	419088	388256	402306	401184	391059	682387	408018
EIC	Negative	411.2755	5.59	unknown	4633781	3265508	3808986	6281981	1883382	2345026	3422322	3209101	2991821	3496438	4131711	1463331	2871279	4171504	4676093	690785	3714557	1406985	1564382
EIC	Negative	298.2470	6.27	unknown	971852	1000426	1819064	5012193	1991729	1322633	1936650	2176090	2422805	2236820	1236010	2260338	1837402	699388	768246	1489626	1825220	3553154	934981
EIC	Negative	323.2231	3.59	unknown	95963	23091	112468	36155	22199	32329	35138	74987	24189	71843	26942	36953	23372	24662	29058	21916	24592	64098	24744
EIC	Negative	405.2868	3.49	unknown	483288	117698	559357	419984	159396	141039	130529	441878	171742	244373	174538	84831	130503	551598	191938	38947	237927	334519	106552
EIC	Negative	514.2847	1.50	unknown	306394624	15746092	1259021184	727230336	25180033	30411880	11334414	1372201984	164298676	821838656	40863749	22925809	213005149	124466184	64697028	30454726	50116817	1215459456	37027415
EIC	Negative	365.1952	4.39	unknown	647797	257920	316429	257429	255387	209734	180834	195910	55527	449267	293039	223343	169463	114553	201475	255816	90158	128018	107331
EIC	Negative	424.7369	0.52	unknown	192857	102628	184794	137311	240101	162643	174807	78948	113492	277822	89973	76701	75156	462633	127166	96379	71891	166560	209943
EIC	Negative	357.0070	1.19	unknown	937770	816816	695639	664805	603327	619030	632684	565056	519737	640975	524154	497762	407631	429833	460612	404827	433446	765445	501735
EIC	Negative	576.3312	3.72	unknown	1860796	3477195	2966701	2253271	2241933	3325194	2426204	2429363	1142697	1705793	3200200	2364659	2139822	4625709	2284722	1393407	2491676	612756	3313323
fpHILIC	Negative	350.1877	3.71	unknown	507241	389713	384074	330472	430888	478598	360736	308305	251208	289013	321784	273102	275403	204667	380440	337879	295272	316891	266783
EIC	Negative	346.7277	0.64	unknown	58692	18857	55125	43656	63527	36256	37949	23238	36624	93198	23501	20614	28546	185280	29828	22827	18053	33860	68027
EIC	Negative	605.3660	5.01	unknown	26129412	37659393	30779574	25035458	31872324	34322963	33433474	28207446	25440656	23623867	35732361	29269536	30224138	28253310	26087204	25972723	30949972	19800051	31118202
EIC	Negative	476.0409	1.26	unknown	541785	537921	578268	514197	398277	347033	389066	406501	370618	449388	381268	178688	283789	305025	317949	318115	244198	446951	343027
fpHILIC	Negative	173.0567	2.58	unknown	527320	354566	485138	507958	352072	615019	442544	409233	294094	403919	378772	349182	373293	394344	432284	386417	378805	313514	335526
fpHILIC	Positive	279.2313	0.36	unknown	973951	864373	1073692	994294	867938	690291	836511	850696	802833	762183	831540	780321	719991	816583	668116	738718	585902	810214	735852
fpHILIC	Negative	87.0088	1.03	unknown	272124576	185068016	154033456	115766776	130144800	185998960	196567760	46073588	36061960	124930120	167800560	100658056	66112556	141711712	138248576	138979920	57315516	99290984	86276120
fpHILIC	Negative	186.1136	0.49	unknown	850958	550371	842205	2046379	546808	1041858	881983	693222	1026243	797408	817513	936001	859301	459251	473716	1189938	879961	615413	504704
EIC	Negative	528.2864	4.17	unknown	1386939	1918537	1568833	1313137	1387573	1573768	1790105	1314494	1647666	1164527	1411636	1500950	1687450	1682874	1450117	1506221	1323649	1167515	1298571
EIC	Negative	604.3629	5.01	unknown	77117921	111168293	90854960	74409409	93387415	101812405	98661372	82965245	74738346	70472903	105181606	87231678	88622314	83879576	77347968	76252441	91252428	58063879	92082567
fpHILIC	Negative	169.0257	2.98	unknown	912811	1076794	898025	1158723	576797	787089	942762	617861	695734	742841	779791	799981	919705	432689	497008	679625	657903	834260	498469
EIC	Negative	605.3659	4.31	unknown	1019040	253242	978818	265781	280030	330728	1562837	152227	250338	171994	431744	1287841	355849	1215482	246593	263069	256096	225843	1262962
EIC	Negative	499.0399	0.55	unknown	1209073	783603	645403	1776944	450625	1338752	974761	597868	409784	573716	908447	1239408	834606	308316	652789	840141	563739	731188	513337
fpHILIC	Positive	146.1174	2.97	unknown	24983140	14038077	20218452	27785266	12661775	15398158	14201982	15419055	14138123	16348365	16601531	14519762	12368083	10011956	10495032	14129445	12672655	16079149	9506158
EIC	Negative	502.2923	2.61	unknown	1262721	0	2122809	1189917	0	0	11178	1598112	32771	987917	31081	30986	48562	39268	34486	8003	13015	1370708	11621
EIC	Negative	271.1918	3.52	unknown	365123	120180	281783	200730	135175	205030	182058	254443	69940	190640	94960	179812	148204	102566	144768	151638	116049	227264	102051
fpHILIC	Negative	263.0018	0.38	unknown	6006738	4490172	7457610	8394642	5599918	4491981	6621937	5120708	7751298	5160591	5496423	6371347	7938933	5544170	1772076	5214371	8500974	4395913	6064658

EIC	Negative	440.0129	1.19	unknown	129139	130245	104637	92693	91933	95468	89509	75418	81892	100218	75873	74614	72339	74703	67484	63542	67015	102625	80197
EIC	Negative	285.2073	4.09	unknown	4433611	1413510	3687500	2464210	2260879	2747064	2489435	3779239	498302	2694798	1282818	2201349	1480683	1456318	2066094	1171766	1717387	3991433	1382333
EIC	Negative	409.2821	4.72	unknown	729597	847945	660784	653227	828966	680887	728679	700041	771356	778709	675765	700269	744617	626931	665347	732490	636990	607676	612168
EIC	Negative	414.2489	6.12	unknown	272745	199991	165093	374027	131906	210826	159893	210662	225872	243842	155457	105665	139748	155910	209156	110524	231503	157654	115304
EIC	Negative	263.1654	3.06	unknown	2552601	1972020	2126071	1738180	1770669	2211085	2055098	2253763	1168019	1736584	1707062	1850547	1840388	1390261	1640969	1587054	1588878	1902949	1580784
EIC	Negative	274.1047	0.61	unknown	117143	38142	66016	52541	35730	47477	36433	25498	39354	70848	37545	27584	33961	27801	38902	26113	34533	28063	34424
fpHILIC	Positive	546.3526	0.55	unknown	17810554	21087640	22580520	18365130	19692816	22227824	22406690	19651786	16206614	17455912	20973064	19385070	21095920	17362990	15795507	16498022	20558690	15442801	19704342
EIC	Negative	313.1197	1.25	unknown	882574	316965	587021	600051	322604	364406	364738	659825	398252	656360	342402	431487	414264	383719	353226	459877	417475	297256	325503
fpHILIC	Positive	119.0836	2.28	unknown	4363163	2826480	4405076	3489530	3203498	3549188	3071149	3781207	2719587	3591710	3237852	2765021	2860915	2170062	2604871	2562060	2953950	3901557	2319109
EIC	Negative	295.2279	4.09	unknown	1196562	1103435	2134152	4575432	1740320	1343387	1553686	2671921	2063285	1754999	1232304	1907209	1635089	1073199	692574	2311623	1756424	2509843	963624
fpHILIC	Negative	144.0456	0.96	unknown	581744	328776	331624	270353	400955	387166	417653	249393	204436	201135	299720	246541	200868	340367	413402	235259	276396	228877	304782
EIC	Negative	516.2731	3.42	unknown	502036	281652	367598	361695	235776	490284	192658	353826	332142	355820	231488	301655	190558	203280	297903	262884	436120	0	413978
EIC	Negative	607.3712	5.01	unknown	979122	1443886	1087342	834884	1123811	1254290	1185797	1005713	897569	848596	1299474	1018607	1013841	1054915	903050	945305	1128784	755783	1074761
EIC	Negative	579.3498	4.74	unknown	16279552	20844888	19387687	14220115	16333479	18007721	18775571	15989137	13928394	13785145	18900562	17538764	15118765	18266269	14875040	13990336	13002162	12841492	18309668
EIC	Negative	570.3420	3.26	unknown	190334	125078	170487	125223	115337	210131	121877	158025	35112	167572	153153	161113	33564	157917	143096	43177	93401	121970	161650
EIC	Negative	418.0401	0.55	unknown	299013	152918	139233	432842	107662	325653	206540	145630	85741	137970	186284	246260	178220	90722	153688	195958	123140	164734	129023
EIC	Negative	598.3734	5.31	unknown	1236132	1203721	1277595	1102161	1128818	1109181	1218104	1413901	744665	1101410	1058855	899160	892448	927971	1074273	781163	1101741	1122816	873723
EIC	Negative	257.1760	2.90	unknown	7921887	2375161	5614553	4182541	2265861	2339581	2626974	3586815	1653946	6731816	2183656	2710230	1890947	2004458	2065136	2116648	1829452	3485172	1514661
EIC	Negative	578.3467	4.74	unknown	52150384	65805283	61475042	45091787	51313156	57415961	59362053	50002208	43359132	43932536	58462167	55592855	48116414	56991638	47504507	44332832	41517968	40431747	57021502
EIC	Negative	433.3331	4.16	unknown	134799	73110	116850	155436	114848	142732	99568	142287	75422	170103	83771	76853	69425	23067	66535	91598	72367	186032	90965
EIC	Negative	265.1811	2.82	unknown	216653	155142	362262	268033	182126	231155	202481	305022	164888	309808	174756	229257	158039	216557	158224	138833	130771	330144	200500
EIC	Negative	442.2965	5.95	unknown	120682	138661	299333	477694	150682	167645	134841	279644	213585	176537	74831	104553	165438	117755	109560	126635	143365	325466	130547
EIC	Negative	567.3505	4.77	unknown	2164042	2915374	2469020	1571531	2112566	2738995	2952724	1979743	1474587	1826877	2107176	2543548	2039730	2242163	2412882	2083728	1769853	1505036	2170694
EIC	Negative	305.1747	3.34	unknown	1029930	202645	1120812	746773	329558	254608	342544	715713	281594	1383140	246417	304022	248158	275510	253470	222989	253447	726551	242621
EIC	Negative	258.2158	3.48	unknown	59117	36106	83432	127189	56036	42449	56558	87437	54558	70534	47602	65369	50163	24782	38394	51156	49558	86932	36338
EIC	Negative	444.6736	0.63	unknown	261384	230052	597112	416672	241935	288919	228283	168150	361000	738335	236454	210222	272173	112970	182264	158825	188939	305972	301968
EIC	Negative	516.6092	0.64	unknown	96457	37555	103033	71644	86595	59573	59748	40589	61132	161686	39415	29912	49775	141335	44611	37985	29839	58512	100842
EIC	Negative	284.1954	3.34	unknown	2118873	360807	2282012	1597890	602932	584463	679281	1484763	501817	2657282	464093	624074	529346	514847	486708	415554	497036	1577522	427635
fpHILIC	Positive	263.0732	3.15	unknown	703539	497336	450766	634709	485874	635624	379759	405452	588694	494117	417008	483941	405435	410662	387552	557846	440652	429141	389725
EIC	Negative	361.1659	3.38	unknown	615647	110554	589561	287085	270293	307532	372766	724445	59747	453200	187735	217781	154332	167699	253911	125054	250077	636577	267303
EIC	Negative	482.6955	0.52	unknown	218060	58213	186904	172013	226878	149460	155833	79093	84299	264344	67255	80906	106662	327606	100968	120290	57276	128388	208075
EIC	Negative	540.3314	3.75	unknown	612907	44224	2100030	781511	60209	55925	31268	1651086	44719	0	63475	65771	49161	100117	76269	72278	63234	1367988	50458
EIC	Negative	344.1546	2.83	unknown	1667411	0	1618741	3598426	49337	0	0	1586646	0	1855515	0	0	0	0	0	0	0	1422654	24341
EIC	Negative	599.2525	0.84	unknown	136099	98748	195129	151738	65694	39778	100837	115140	53140	123556	103106	80128	114235	26938	45530	74773	49019	131755	30360
EIC	Negative	323.1851	2.97	unknown	164180	83234	182846	188366	137874	132737	113196	190609	121099	204870	90509	144500	112370	143621	77982	79865	116695	216029	124969
fpHILIC	Negative	332.2676	0.37	unknown	1212764	1322858	1662320	2762476	1273747	1356110	1691269	1761052	1268422	1776489	1069459	1769554	1531363	879463	1186121	819372	1363731	1805828	1333343
EIC	Negative	265.1811	3.31	unknown	1993312	914886	2209122	2747777	1248859	1426963	1378178	1692349	911771	2071728	1162966	1965839	1497976	776421	914693	1278355	871167	1980432	850627
EIC	Negative	265.1811	2.88	unknown	274099	128277	268375	405137	166773	205727	161524	258243	133637	322906	147045	249741	183429	113404	114398	128314	140515	313883	122843
fpHILIC	Negative	557.4577	0.35	unknown	3504167	3400369	3357100	2683361	3596489	2630053	3269440	3115753	1633711	1980321	2590231	2272241	1885919	2134872	3122715	2723341	1976897	2329101	2472776
fpHILIC	Negative	239.0773	3.34	unknown	959828	858442	918962	895974	871424	858770	922467	842057	781643	784104	749172	651400	811253	722851	710768	729392	922338	757000	673130
fpHILIC	Negative	279.0383	2.65	unknown	630858	566431	526016	840984	558613	689384	595366	488470	570849	674948	681172	637916	580663	370047	565375	549680	605312	438677	416171
EIC	Negative	319.2640	6.03	unknown	253395	203865	275451	247241	234867	178985	161629	255372	186149	296728	212622	139460	166353	161764	214928	163105	176258	210021	137998
EIC	Negative	377.9976	1.19	unknown	131000	119131	113033	101647	89220	88238	90447	94057	75565	90602	73425	70399	72277	66656	70794	64362	69942	109004	74170
EIC	Negative	568.3515	4.77	unknown	459588	573882	518049	367254	458300	540984	610752	440591	315495	395862	468897	517538	460078	487823	528864	495883	350760	301195	423469
EIC	Negative	361.1846	3.26	unknown	3842994	3010442	4329677	10412952	1517784	2864255	2285661	1078949	776382	3733454	1676650	1086254	507043	285262	2037302	2893917	799660	1187297	651100
EIC	Negative	343.2276	6.19	unknown	1001950	777961	857815	689220	814155	782637	725707	1032193	412808	644738	655688	860466	614139	525595	692702	691620	598158	753373	610557
EIC	Negative	296.1951	3.71	unknown	196929	82227	190117	125116	125655	103106	154268	196587	77305	163286	87820	120136	89921	86615	104903	102996	99541	193765	82506
EIC	Negative	293.2124	3.77	unknown	873580	672517	1195816	807419	867500	842618	988319	972912	671503	838162	582594	514235	496421	1448041	339837	641652	815803	1382925	679479
EIC	Negative	426.7345	0.52	unknown	157910	58379	91587	118663	199627	102894	120672	31436	117933	220587	41278	22434	73193	386374	130603	0	92539	117404	168492
EIC	Negative	291.1219	3.45	unknown	594489	202380	484146	534781	209989	333340	276656	362768	170766	381716	177403	308472	245092	125750	254098	183422	217106	366277	182529
EIC	Negative	342.9323	0.52	unknown	401537	464337	489877	546843	329590	378147	467593	516190	459444	369860	436948	470869	510485	141905	329825	403678	444089	467518	267370
EIC	Negative	368.7760	0.52	unknown	157412	78895	161949	106385	149251	95384	147032	0	84163	205277	87146	37132	70377	456011	100931	73686	0	96361	152192
EIC	Negative	267.1241	2.93	unknown	1121163	1486057	764365	536567	938163	1318228	1050475	544088	809062	816877	1007419	903844	862165	1001035	1293106	910657	791467	556855	595760
EIC	Negative	385.2381	5.96	unknown	141908	117032	195954	163756	145946	89749	112170	131473	119425	221001	145469	97677	96897	80582	135766	87455	87075	132893	89847
EIC	Negative	251.2015	5.75	unknown	760929	448507	1020999	2062109	569821	509295	481123	1018556	659367	848387	427540	621698	599909	269041	615619	489700	494624	1238779	338491
fpHILIC	Positive	201.9835	4.07	unknown	2178494	679271	2429389	1833610	562333	615994	511617	1607103	1179067	2395247	673497	499308	541377	592733	743969	760254	415766	1568718	498561
EIC	Negative	295.9940	1.19	unknown	370269	322268	303381	289356	243116	250480	230031	263121	212365	266463	199975	201850	176094	181734	184495	174971	176240	332806	199741
EIC	Negative	258.1799	2.90	unknown	1311603	388505	788793	676617	388179	404793	404051	567641	227171	1047267	337828	422726	352802	299271	334650	343059	285727	547056	264063
EIC	Negative	285.1171	3.15	unknown	24005	276794	45976	50386	110427	73912	221149	43287	126673	16420	204683	283651	189211	140228	192437	137321	95259	0	87737
EIC	Negative	281.1762	2.57	unknown	1232582	608848	676261	853968	793650	769315	991120	767850	668588	894898	682944	577899	552778	503660	661469	535550	854689	931042	573165
EIC	Negative	316.1223	1.65	unknown	262800	183796	484437	123995	148080	151789	278354	139943	143588	430292	440298	495651	168004	350707	78040	255951	170822	166698	96416
EIC	Negative	267.1607	2.51	unknown	179950	139157	170813	233371	130159	128391	103306	155927	210072	178827	95321	113960	132727	113414	99012	172805	121936	172543	82462
EIC	Negative	298.2470	4.63	unknown	3315702	2131854	6915944	8830961	4167099	2381713	3569277	7862114	3873661	5495514	2673286	3342786	3132813	4300653	2986816	2442399	3749851	7389975	2912934
fpHILIC	Negative	168.0182	2.98	unknown	2141942	2185153	2474377	2459591	1272894	2033564	1710026	1298162	1473968	1838794	1874223	1697460	1896295	0	1201265	1375816	1464559	1713422	1188965
EIC	Negative	258.2158	3.58	unknown	142336	103001	204297	185018	148901	132924	123186	195516	147140	146716	110585	124862	114986	110320	92887	133162	120888	201254	118424
EIC	Negative	516.2859	1.50	unknown	29349396	1494041	124566768	69259568	2452025	2908252	1103761	130097736	16127888	78089608	3960172	2220195	20900880	12021160	6197233	2932421	4726466	118727400	3568291
EIC	Negative	279.1873	2.85	unknown	84759	218336	241486	195650	116426	162502	110292	142565	66227	224421	211771	126327	77744	58098	111929	104841	116656	153175	46860
EIC	Negative	343.2216	6.50	unknown	234685	496471	461529	480002	352483	352164	289755	126344	201754	320326	412224	330642	222834	172914	151972	293714	333567	130994	270271
EIC	Negative	462.2951	6.09	unknown	2832925	3565233	2447050	8224223	1393887	2315569	1744121	3270139	3388905	3565852	3498751	1093071	3353828	3313210	2355547	1017095	3013876	2497856	1928041
EIC	Negative	413.2448	6.12	unknown	972719	737964	671065	1177025	433386	713521	640279	658675	748546	1005615	631423	358378	457272	696692	794005	349556	688055	583955	436038
EIC	Negative	632.3195	3.55	unknown	144978	210532	146610	164541	157396	177337	191731	144531	139902	155632	181603	187435	156211	147353	173570	153192	125990	122354	148830
EIC	Negative	314.2420	3.45	unknown	571085	441285	969034	2255679	643757	590815	828023	1093153	891366	856255	529206	889307	784361	626233	302780	831580	763244	1260392	455685
EIC	Negative	530.2830	4.17	unknown	461381	672124	535405	460824	523219	551115	658594	454380	599228	402986	501526	529623	592682	599967	510437	519633	498110	496310	464119
EIC	Negative	253.0304	0.55	unknown	3015648	1221899	1224897	3212292	894422	2817303	1582556	768245	730528	1260661	1474123	1801619	1205480	739478	1158430	1657232	842689	1212578	1196415
EIC	Negative	299.2239	6.18	unknown	1922426	593194	2136656	1457683	735799	488736	866628	2024347	159942	1904260	621404	650641	518363	470996	723075	264131	542841	1483094	806005
EIC	Negative	521.0134	1.19	unknown	178591	173106	153651	135705	132283	129211	135204	124369	109028	139423	117014	114874	104251	95527	108671	93563	102207	142663	115875
fpHILIC	Negative	297.2799	0.37	unknown	544524	465649	780937	1225915	656726	685472	476995	538781	599268	651832	701056	741907	412087	541971	311920	794091	421546	679758	435705
EIC	Negative	566.3470	4.77	unknown	7256620	9348120	7849358	5337399	6816393	8942494	9582214	6244165	4785017	5782964	6823679	8206621	6928442	7555478	8159184	6683046	5951256	4847336	6867030
fpHILIC	Negative	288.0755	3.39	unknown	7550735	2172276	3330265	4646788	2507379	6644201	1183660	4596531	1082104	3430359	1030699	2918120	1189219	931536	779023	3449919	2138053	4631167	3323316

EIC	Negative	315.1601	3.29	unknown	305731	283463	407960	374609	268298	188864	234069	258726	248113	490345	288235	264719	227291	184250	328439	190661	183465	251754	117244
EIC	Negative	311.2010	5.27	unknown	944561	1102304	973924	1452277	0	0	0	1418507	0	1068292	1269499	1268385	1214839	1292630	1268994	0	0	0	0
EIC	Negative	529.2903	4.17	unknown	377817	502932	401987	383199	381389	406321	484387	436300	480595	384620	400659	411777	431020	511681	428763	402280	381411	358460	387445
EIC	Negative	253.1446	2.89	unknown	3550653	1029618	2219306	1357362	1016841	1297303	1413684	1664217	711017	2093735	731958	1156420	833635	892829	1127221	832945	867085	1783823	849799
fpHILIC	Negative	306.2521	0.37	unknown	1634905	3295080	2978566	3556772	2387812	2859768	2985144	2399958	3072660	2392628	2583327	2409165	2781234	1524912	2107034	1995602	2649322	2876523	2636047
fpHILIC	Negative	173.0091	4.58	unknown	31245084	25812978	25301750	29573402	27296468	34122464	27840914	21755594	28733008	21198906	26146386	23911766	25203094	23519164	22635910	29203292	23953534	25643860	23704914
EIC	Negative	227.1652	3.60	unknown	173564	87059	178758	379985	102714	121004	116142	139930	96549	166757	97215	151871	154976	50919	77161	121616	93945	164169	59234
EIC	Negative	280.1613	4.45	unknown	418244	71435	243402	341031	149745	69302	160403	203148	54040	189321	78293	152698	144928	52640	80479	140541	67254	281962	0
fpHILIC	Negative	114.0197	0.59	unknown	5673831	6191681	6757223	3777712	2990360	5329401	6765931	1780297	1220849	5970265	5164596	3364324	1945597	4718623	3980431	4272726	2184622	3005733	2760692
EIC	Negative	330.9579	1.19	unknown	182096	156921	133999	130798	123683	120557	115632	113796	113104	141380	108350	104906	93446	89887	103280	91777	82002	149487	99231
fpHILIC	Negative	558.4603	0.36	unknown	1347358	1402285	1291919	1013406	1594609	1165751	1337556	1166392	857987	877782	1042406	1029916	761083	829229	1408833	1151975	1002633	939356	983755
fpHILIC	Negative	293.1144	2.73	unknown	1769676	998957	1246480	934804	976704	1724352	1101774	913559	993015	1393235	991741	1157706	834179	1238458	1339336	880876	927069	1043278	926392
EIC	Negative	245.1580	5.01	unknown	182378	100242	206976	228194	110995	114949	92070	157947	44418	163456	95199	86216	81356	18465	83706	149976	45753	166790	20456
EIC	Negative	475.9986	0.55	unknown	75587	68696	73184	146487	28026	107943	82309	32038	51241	47577	78360	87185	78572	0	63580	105791	27324	0	73847
fpHILIC	Positive	282.1182	2.82	unknown	1560910	1060067	1023369	1707873	1219409	1919701	1142958	1067519	1128601	1029026	1230531	1248684	1315135	967616	968918	1272828	1185948	1386403	998961
EIC	Negative	471.2196	0.71	unknown	511600	777663	853866	468778	485642	588463	776236	841849	479174	510570	812449	704573	619192	408251	614312	485900	593307	646085	506473
EIC	Negative	235.1339	4.05	unknown	177030	86870	188563	247843	85777	108920	115210	133820	68542	137146	88094	134635	127717	43067	76529	89865	89953	149034	54552
EIC	Negative	479.2835	4.68	unknown	1253006	1314683	1314851	974009	940731	1220656	879110	1122392	624725	921761	1015488	990431	757251	1536079	1103503	0	874893	1154419	1166241
EIC	Negative	450.3313	3.74	unknown	1391368	177326	2566472	401352	232372	128288	119871	752661	175018	663150	120783	99463	67046	135796	117208	138647	127836	765212	115705
EIC	Negative	434.2368	5.63	unknown	2728047	2507030	1969877	3796454	2014609	1841690	2231899	1923235	2670362	3087777	1947143	1848519	2843644	907867	1577038	2494778	1821461	2531017	1168544
fpHILIC	Negative	202.9978	2.98	unknown	3261211	3024775	3406365	3998115	2147717	3004320	2767997	1930420	2354287	2920377	3005346	2510444	3218496	1032832	1617931	2263371	2260157	2779136	2041694
EIC	Negative	308.8195	0.52	unknown	357946	177594	251637	223474	397742	218288	227822	152008	182125	416422	118385	119982	206311	1296397	199582	154677	154266	237239	312472
EIC	Negative	278.1476	3.87	unknown	625024	623407	397247	604688	418423	316640	391169	391368	493151	394287	1253908	631413	624975	360518	315962	387305	296932	444996	361288
EIC	Negative	296.2313	4.09	unknown	232290	217858	410759	911484	341582	272999	303533	507590	378350	376948	240770	364572	337732	211376	147610	442574	364627	490174	176108
EIC	Negative	250.1893	3.29	unknown	382117	278654	409917	399565	277432	185952	267104	275812	227995	437159	268250	223673	207182	184876	288958	226125	175671	240660	177780
EIC	Negative	297.2436	4.63	unknown	16780649	10226183	33665623	43050154	20562076	11438906	17406373	38735321	19154512	26930295	13637844	16002392	15343342	20777271	13976288	11677755	18559176	36960645	15251076
fpHILIC	Negative	244.0656	2.65	unknown	1414005	1278976	1149467	1804889	1334987	1592648	1474101	1060867	1177643	1588415	1569906	1333485	1360969	780222	1333680	1293696	1340947	1015296	985229
fpHILIC	Negative	116.0354	1.92	unknown	5891754	2023804	2380156	9567101	3027250	3340002	3055148	2143355	5849687	4347980	3647000	3771258	4197836	1346921	2728464	6567812	2638655	2363016	1357138
fpHILIC	Negative	384.1849	0.59	unknown	1122620	387118	654646	816048	374094	234871	248429	673075	266138	677182	180026	485412	394961	295502	203627	126842	351386	715708	217836
EIC	Negative	333.1712	3.02	unknown	275281	144785	308712	218961	176458	133840	235881	239481	89078	265244	138194	143348	112062	105774	132158	111307	102042	346842	94321
fpHILIC	Positive	730.5366	0.45	unknown	521224	617479	630720	736033	339225	545891	619792	501387	341939	378903	505618	357553	511398	703168	427100	320586	444602	459489	460480
EIC	Negative	283.1919	3.34	unknown	12176979	2106981	12813028	8969354	3263764	3131823	3893135	8203090	2912104	14713830	2628826	3628918	2998855	2956802	2770778	2302200	2835352	8944455	2407121
EIC	Negative	288.1902	2.42	unknown	191065	74329	214683	122539	70334	70185	102926	197571	44444	187889	78612	82189	74898	75671	73120	55676	80850	151213	76159
EIC	Negative	336.1900	3.72	unknown	161861	55852	157431	116146	70057	88182	80362	152005	22842	106064	53252	62609	54059	32709	57380	55514	51098	161326	47047
fpHILIC	Negative	226.1448	0.41	unknown	1788247	717721	795033	1854133	886273	1647853	1108402	953946	790268	1137472	875792	1376839	1133457	315414	1089913	1016757	1060320	1052539	633751
fpHILIC	Negative	216.0986	2.59	unknown	3714719	2044372	3277821	3407448	2931702	3891591	3038983	3009379	1663132	3043276	2554731	2529257	2379849	2725750	2435910	3078055	2480020	2690623	2412096
EIC	Negative	552.3314	3.96	unknown	8862766	14738162	10187350	8539213	10172000	11885133	13432164	9134254	11335323	8177197	10865253	11726196	12225351	14725538	11900181	11442447	9899300	7863671	9325763
fpHILIC	Negative	245.0778	2.01	unknown	1251306	778007	702747	1387976	926895	1366403	936457	680981	945476	971814	1032496	983239	854363	511407	780397	1170504	970204	764822	690647
fpHILIC	Positive	833.5862	0.43	unknown	2886122	5769390	2673665	2851207	3740967	3959837	4421860	2915395	3190708	2529114	3634152	3527901	3863800	3313674	3787876	3940381	3368258	2259595	3076942
EIC	Negative	344.2314	4.97	unknown	309986	408966	341640	1263398	393952	351232	392368	520399	631761	516303	402075	437150	589041	201924	282486	569084	385295	585647	241997
fpHILIC	Negative	167.0210	2.98	unknown	176452656	153122240	176614080	190393344	110795944	147085120	137182848	93586544	111072248	146013600	136019072	125725304	144646720	55936532	83744568	111944224	120426488	129692488	95659000
EIC	Negative	630.3782	4.43	unknown	309434	0	323566	0	0	0	321882	283765	0	0	0	0	0	0	0	0	0	239749	307341
EIC	Negative	282.7780	0.52	unknown	247423	74278	194854	125979	282072	117760	133025	33644	94753	322551	39186	36551	86671	1248990	108142	88900	34579	98197	267584
EIC	Negative	289.1447	1.76	unknown	229542	98969	324660	108325	28370	84667	81071	99226	29118	147033	88668	101911	101038	75082	47729	81739	79174	0	82891
EIC	Negative	474.9950	0.55	unknown	815523	495150	479513	1148443	299506	949952	607018	324376	316807	368883	618733	689542	464097	79630	441088	653688	344342	485257	335145
fpHILIC	Negative	198.0715	2.92	unknown	2699911	3023496	2741805	2372508	2192178	3180800	2379168	2313855	1571067	2613936	3102919	3268184	2463879	1940955	2057644	2133083	1952776	2220921	2164612
EIC	Negative	311.9930	0.55	unknown	145318	30626	40663	157940	51803	124465	82086	11578	22294	72258	75328	50788	30868	16236	74124	72517	18334	39702	59842
EIC	Negative	360.2676	6.36	unknown	991527	613020	1127793	1540492	845004	815161	972550	1378812	1000562	929129	864607	921490	1065826	473560	726997	969751	819472	1350161	671036
EIC	Negative	470.2164	0.71	unknown	2352896	3553336	3996369	2237849	2170928	2726331	3543560	3800373	2235274	2408851	3753798	3317063	2884067	1887535	2896285	2234976	2679514	2925113	2379811
EIC	Negative	325.2752	6.50	unknown	451415	659676	1038341	1772013	811384	665929	889014	1361301	920758	836029	647082	812863	727790	522720	680091	862397	840413	1120606	709645
EIC	Negative	553.3347	3.96	unknown	2550660	4303541	2995327	2558714	2967438	3486845	3925204	2712908	3407191	2391224	3180287	3483257	3639660	4273653	3568640	3427587	2907621	2280995	2772547
EIC	Negative	270.9480	1.19	unknown	566922	387201	438916	370432	271010	259602	240905	249496	231715	388675	215291	179181	161083	224243	172119	154666	151267	403095	254511
fpHILIC	Negative	117.0193	3.95	unknown	8017695	5075072	4363449	7279424	4929649	7115198	5434855	4182151	3451222	6461286	4815663	5259168	3427686	4671831	4695214	4259113	3703826	5646424	4469996
EIC	Negative	297.2076	3.82	unknown	2264828	620147	2785959	2555772	1107614	1037086	1097776	2203521	1034550	2374473	799822	1024294	929231	998297	780396	783210	961521	2581581	770680
fpHILIC	Negative	146.0824	2.92	unknown	3111067	3380122	3047280	2438326	2324463	3427538	2831702	2536277	1923301	2604832	3540994	3519328	2873755	2038051	2456116	2263370	2398480	2372763	2340702
fpHILIC	Positive	547.3555	0.55	unknown	4640958	5386261	6066130	4686936	5104387	6006734	6017570	4826194	3694581	5114446	5376564	5181613	5744414	4364032	4305484	4278344	5287849	4155372	5240428
EIC	Negative	255.1138	1.31	unknown	596516	299189	0	239018	256665	235350	242890	0	207610	370822	295847	325794	281505	163561	170865	301035	218231	0	164881
fpHILIC	Positive	477.1615	2.60	unknown	960119	560976	500380	1022922	648940	708273	510957	545079	983416	599039	372093	722198	658092	428809	434613	866190	527244	700277	553615
EIC	Negative	286.2108	4.09	unknown	820883	267777	663193	488066	378588	467413	426561	662360	97326	506246	234950	402422	259075	263806	359856	212241	313672	721718	236503
EIC	Negative	294.9912	1.19	unknown	2613219	2259982	2159630	2025552	1676446	1681459	1670256	1772168	1487590	1853314	1481758	1395000	1271238	1247431	1316038	1247241	1242028	2358669	1358095
fpHILIC	Positive	809.5859	0.43	unknown	12201658	11812223	11070800	10727569	9964511	11529494	12720614	10842496	8633570	8651621	9690049	10506141	9536520	9776366	10487595	9570648	10902213	8526663	9246566
fpHILIC	Negative	546.1729	3.36	unknown	2514267	1960776	2352702	2338916	2021861	1792604	1804074	2611677	2302115	2395781	2101505	1885060	1953143	2049772	1852854	1864172	1776051	2020224	2047510
fpHILIC	Positive	571.3567	0.54	unknown	2940965	1534707	2257444	2600795	1681850	2249710	2091211	1940745	1679846	1991044	1679235	2123764	1720976	1157933	1730304	1370207	1903572	1913065	1844557
EIC	Negative	476.1830	2.59	unknown	639216	378875	159317	256621	261052	332595	287628	364924	252018	403706	255852	387020	421023	180244	222312	356941	266557	306185	209470
EIC	Negative	295.1918	3.71	unknown	1084691	386907	1025096	658897	664473	504672	792750	1166159	408221	864380	530606	622733	521907	465888	602405	587918	579685	1031167	412556
EIC	Negative	569.3659	5.28	unknown	202735	237617	203167	190387	214055	205403	204234	207008	114734	157606	195463	211313	182628	192112	211081	149003	164212	157394	177533
EIC	Negative	242.1841	3.15	unknown	175976	129946	260660	326500	197722	193815	253830	223114	194955	242753	175773	287541	301229	205063	142402	190090	224400	261499	148246
fpHILIC	Positive	219.0098	4.07	unknown	557029	142029	648456	409080	112550	185725	100784	437011	221030	573895	169027	133976	178937	155068	210424	135836	99982	366053	148706
EIC	Negative	251.1286	3.15	unknown	175240	173887	481104	255813	142662	156627	380493	299202	92175	270330	127795	123566	159527	176725	266358	146606	117964	335710	107712
fpHILIC	Negative	179.0562	3.34	unknown	65402436	57085644	62174292	66257756	58954976	61593232	67792288	60670072	57065268	59843080	66513696	57249900	66393448	37721760	55652108	55928868	58683892	57242152	50968556
EIC	Negative	646.4879	6.36	unknown	3034028	2332756	3489713	1408463	1562391	1601184	2106082	1498410	1079798	1959104	1394209	998208	1159645	1112018	869988	1496996	1280645	1790859	1705340
fpHILIC	Negative	224.0236	3.88	unknown	493786	251397	150708	479310	209828	545007	254891	94635	394944	193437	234003	247207	446989	154103	288245	412470	338850	182840	142621
EIC	Negative	639.0031	0.55	unknown	272188	250353	143393	550344	117498	395053	283214	194112	150567	117553	317274	380410	237291	0	199537	328311	181495	224490	104535
EIC	Negative	588.3329	3.99	unknown	173828	397042	208022	217831	253809	243665	299979	208575	289679	184158	295190	268089	314128	343873	242016	235593	288850	176349	237082
EIC	Negative	338.2122	5.22	unknown	1126528	481193	993618	786438	607810	743810	604308	1062579	254430	716715	528721	788691	609409	440434	668235	507036	580396	811475	415252
EIC	Negative	323.2590	5.04	unknown	68691	37944	169251	556922	120072	15058	104264	229628	170313	187365	72218	154678	148424	24364	34894	135478	93947	317834	36913
fpHILIC	Positive	570.3499	0.54	unknown	8964624	7614667	7673310	8734748	6656445	8504017	7544466	7198847	6326046	6027218	6912563	8019639	6937372	5540503	5851942	5771395	6842883	6961254	7595288
EIC	Negative	235.1338	4.22	unknown	1420857	538347	1257405	1949549	523235	651557	623970	979826	339577	990856	499497	743492	735277	229699	520841	583341	419748	974854	344673
fpHILIC	Negative	890.5578	0.43	unknown	499738	754259	584712	340139	503555	662788	756994	450342	347362	344957	678101	768736	531798	439046	635257	457831	481672	363584	544038

fpHILIC	Negative	291.1312	3.47	unknown	4312681	2484462	3112150	2798587	2440759	2728824	2215492	2508075	2074561	2708263	2519776	2698675	2368695	1735608	3105560	2387552	2316100	3110728	0
EIC	Negative	239.1653	2.74	unknown	623032	332524	744839	733143	451209	368851	381811	518306	308418	1245433	310821	400032	336418	623141	157696	150347	358496	862950	321531
fpHILIC	Positive	702.5610	0.48	unknown	896253	915284	799491	876262	818434	746471	593818	836674	1068614	805418	803962	790406	761060	928434	811150	745570	749655	629532	772628
EIC	Negative	241.1808	3.15	unknown	1226445	881310	1614630	2131266	1294926	1209266	1606081	1481666	1269282	1592811	1102459	1876485	1905908	1355084	908577	1323824	1438773	1626228	965018
fpHILIC	Negative	289.0793	3.38	unknown	767714	204137	329439	473394	219422	591879	138598	485959	111632	332063	73730	249895	141581	110831	35779	338232	185943	493444	359523
fpHILIC	Negative	195.0100	2.97	unknown	1378661	1100737	1069940	1329413	1068574	1467041	1148016	538766	1213664	1065894	1289841	1514651	1074953	319994	976586	1453548	1069692	692480	773428
EIC	Negative	461.2908	6.09	unknown	9284084	11397159	8013130	27378009	4572642	7219304	5764008	10561882	10613438	11721026	11568468	3401668	10943707	11168729	7796452	3348445	9847656	8166544	6225316
EIC	Negative	391.2439	6.24	unknown	1288832	515099	881693	1259265	431042	395340	563861	787820	659283	873265	695292	372591	586348	662658	742264	188430	506607	813165	341814
EIC	Negative	411.1798	1.78	unknown	327287	191008	298140	111859	111103	75226	188403	0	155128	244016	121723	143063	110588	126502	100632	190508	121584	0	71562
fpHILIC	Positive	808.5810	0.43	unknown	27496506	30193594	23060724	23147580	23454950	26460218	28449452	23144480	21962158	19938434	23742776	25981562	24174590	23259934	23750416	23121598	24505920	18827248	22699072
EIC	Negative	268.2001	3.77	unknown	419883	204580	473235	322367	207286	222081	234135	348105	111213	342365	207781	220610	180080	164953	189770	147917	135811	371729	181669
EIC	Negative	296.1951	3.52	unknown	260428	84641	194861	114733	108266	131500	137073	160074	44382	128235	68631	120957	64089	72228	82544	63672	81140	231824	72472
EIC	Negative	292.9957	1.19	unknown	174737	156455	141994	135273	110761	111170	106913	127468	105080	132111	99392	93932	83801	82757	93259	80963	90534	152898	95837
EIC	Negative	394.9893	0.55	unknown	495896	214433	237950	559251	182776	478325	267411	143929	146649	204724	282509	314306	213703	62576	219924	312945	164144	235528	183274
EIC	Negative	637.2281	1.03	unknown	47633	27499	73836	60373	24157	15038	41582	45765	14406	35621	44185	37248	44408	14389	25736	17127	18207	52997	15008
EIC	Negative	307.2272	5.68	unknown	139205	61802	152852	186604	71443	56721	66248	146140	58912	137159	57849	47072	83591	21156	44015	58037	63769	176883	23294
fpHILIC	Negative	605.4052	0.36	unknown	1162996	1111640	1165415	798051	929489	932968	1069852	1277483	750213	515647	852953	929039	948944	1365283	1159566	955634	708347	770755	950431
EIC	Negative	287.1868	2.42	unknown	1138761	364729	1308404	722691	397429	392557	621962	1083410	145638	1169547	475528	485168	349105	349747	419896	253886	329067	928916	489618
EIC	Negative	389.2464	6.24	unknown	3805716	1603220	2774274	3779675	1040089	1277948	1515361	2275042	2020600	2733352	2182107	1118122	1676590	2119272	2315891	672120	1619670	1815966	1034721
EIC	Negative	556.9982	0.55	unknown	418536	319574	294587	722647	207944	611777	401276	233932	235305	214645	400456	523914	353302	22883	275517	463334	274106	316733	221375
fpHILIC	Positive	468.3080	0.61	unknown	7234198	4351224	5989604	5017381	4396845	5118755	5765714	5340488	4239441	4622721	4370079	4909672	4736678	5007304	5081123	4559819	4092928	5049184	4032947
EIC	Negative	488.2276	0.71	unknown	298064	454624	511094	290494	288885	352639	456162	512771	286358	300708	507526	434810	361288	239432	379843	292898	357348	397617	314496
EIC	Negative	264.8976	0.65	unknown	339207	132638	134492	280475	78552	260845	141489	66248	82385	159122	147271	154866	115389	48532	102888	151912	79219	99236	145170
EIC	Negative	296.2313	4.37	unknown	1504209	1169720	2400576	1945355	1689218	1480899	1649748	2164987	1575914	1307654	1071305	1053210	1086542	2251609	695903	2037536	1594922	2079125	1469544
EIC	Negative	325.2021	3.21	unknown	275197	128979	332299	230169	166357	142863	187627	293158	123045	242169	72466	147301	136097	124137	133919	148164	139099	297624	121458
EIC	Negative	259.1369	2.67	unknown	107551	35758	53275	65856	61453	68623	64733	95017	0	45360	35598	46282	33593	15306	55606	64223	47904	75425	23596
EIC	Negative	437.3276	6.39	unknown	1324331	761978	1286110	1029696	689193	483204	740682	1005303	136322	804286	629579	463257	514425	567226	642339	526491	541158	609629	491960
EIC	Negative	364.2577	6.03	unknown	688242	657227	775802	714836	734046	487303	516053	712154	599114	870155	683400	451363	501109	504029	698679	467903	574052	633422	432866
fpHILIC	Negative	91.0401	1.74	unknown	13006488	29188672	32070286	39799228	27621822	31001936	29773582	24785534	31667878	27058202	23561434	31579332	27761084	18416046	18835110	32436268	25515586	32060856	24521220
fpHILIC	Negative	283.2642	0.37	unknown	105968360	147370176	162834448	168016832	155205648	127696632	145729104	122019248	114836856	124983608	103378312	148941568	103955968	124561416	98332888	99949144	121755968	136244416	156522080
EIC	Negative	283.1921	3.00	unknown	242078	147472	213410	386418	221732	236767	299920	234927	209459	298627	206135	319622	345936	133110	155643	254667	215064	330809	151791
fpHILIC	Negative	78.9189	2.10	unknown	5796470	6049452	4817775	5410248	4954481	7217190	5417956	4496967	5608559	5021212	5959137	5273211	5845199	5543158	5633869	4748074	5264366	4595392	5273593
EIC	Negative	446.3358	6.51	unknown	1033026	1071435	1407103	1585774	1062976	605767	1330381	1427252	1158797	1332717	1254082	833547	840972	691240	1101906	1409357	914299	915385	808476
EIC	Negative	289.1060	3.18	unknown	885899	404932	678942	1094681	430944	505450	520847	691890	262554	753106	381551	552534	560819	175410	452823	425949	339657	710855	294584
fpHILIC	Positive	832.5822	0.43	unknown	6371620	10148059	5222020	5733040	7127114	8004460	8753523	5430243	5499145	5026182	7029384	7180423	7112663	6711797	6966167	7325747	7336474	4121224	6354144
EIC	Negative	512.9608	4.37	unknown	913037	1172910	896282	1212770	983250	986265	1060783	981788	1008940	829499	928028	850564	891140	885448	973487	1043568	807943	1024563	781452
fpHILIC	Negative	193.0132	2.98	unknown	3877479	3428431	2661258	4279092	2846696	4569144	3434367	1589978	3305639	2699042	3719476	3970235	3122459	880594	3402765	4443381	2639755	2015846	2023282
fpHILIC	Positive	204.0560	3.16	unknown	1201577	1466366	1443584	1607818	1442187	1339411	1415418	1395352	1389984	1458607	1381872	1265126	1489422	1203881	1311174	1365104	1248801	1357312	1160573
fpHILIC	Negative	327.0028	2.05	unknown	558437	759178	722347	601743	662971	962223	601880	377203	303827	854489	740091	691365	357115	303140	652323	578346	393822	477118	582601
fpHILIC	Positive	219.1340	3.32	unknown	574890	285942	151035	200574	177154	286545	232125	64936	75586	192060	251556	328709	84512	197369	252758	261021	70597	63469	124330
EIC	Negative	317.2028	2.99	unknown	0	0	0	259666	60671	61492	0	34329	60539	0	0	160159	0	0	0	0	200386	0	0
EIC	Negative	253.1439	3.79	unknown	784164	841211	739287	790191	766020	716975	959678	672467	755328	567084	772228	870556	836769	841281	844206	715195	585021	716849	712385
fpHILIC	Negative	158.0400	2.97	unknown	1154477	1019917	908298	1106067	691285	1089601	927971	445478	851358	707371	1040950	884273	861698	189403	823629	1003489	755204	495608	620815
EIC	Negative	246.9022	1.19	unknown	169444	87205	126284	92965	80587	73446	67761	54754	65516	137587	50943	48213	44305	82853	45951	51243	36507	93112	92091
EIC	Negative	338.9371	0.52	unknown	3146530	4367897	4302741	5004598	3284943	3616312	4145375	4972988	4308380	3635656	4235366	4550498	4546384	1937972	3396167	3667221	4351653	4513530	2863544
EIC	Negative	610.6760	0.52	unknown	295981	252409	479827	545302	269784	329357	309902	154071	539737	414811	261839	266906	333923	0	224070	292003	280091	357218	328582
fpHILIC	Negative	219.0451	2.99	unknown	2656161	2087730	3516449	1956741	1077077	2261593	2407054	856684	751178	1205755	1810837	1809940	1607705	0	1038802	1389205	1138994	1751172	1489913
EIC	Negative	406.2966	5.77	unknown	321842	402427	297738	374064	393856	376067	360369	304995	265054	297504	355241	270608	303354	247092	310538	317940	314639	333534	297570
fpHILIC	Positive	295.1280	2.73	unknown	1556889	839484	1072231	1017889	784802	1264276	986130	740176	979370	936762	813486	743678	845653	1017186	1040134	1029658	639401	1041075	652448
EIC	Negative	435.2400	5.63	unknown	754982	692813	528837	1077259	554109	486199	628149	526325	715858	874822	540396	512233	753027	253399	437078	728445	524463	678494	326674
EIC	Negative	463.2981	6.00	unknown	910224	1568037	857887	2332130	889787	1564594	1030764	1182111	1453468	1406466	1568723	681829	1264731	1363588	1343079	675588	1767491	774895	1106703
EIC	Negative	594.8166	0.52	unknown	277339	394498	494176	529022	309046	364213	427123	376448	440085	457910	409218	439317	411591	0	321452	399900	400965	408319	246018
fpHILIC	Positive	855.5714	0.43	unknown	397281	1071754	768410	532355	598387	918981	1264128	587850	478272	397839	801216	891696	845090	804405	781448	685165	783730	428355	888271
EIC	Negative	339.2539	6.07	unknown	66146	55501	116310	344740	94363	135940	101310	170173	167825	161907	78615	107100	162786	27390	56428	105573	155439	273096	30477
EIC	Negative	441.0108	1.19	unknown	86882	90008	78956	65593	82382	90670	82278	70548	77906	73461	82573	83675	71354	83929	75300	72853	75505	66904	83930
fpHILIC	Positive	756.5541	0.44	unknown	4932607	6964167	5561961	5397581	5152096	4903442	6848245	4842751	5816559	3707218	5538135	6451122	6207905	6863360	5460124	5092536	5599306	3782814	5631003
EIC	Negative	286.9446	0.66	unknown	61888	60780	52820	118779	33472	77065	59836	48369	32585	38600	64773	73071	54741	11794	43913	54418	44295	57118	33466
fpHILIC	Positive	272.1853	0.40	unknown	1053964	622607	756008	908871	874972	844010	719326	838126	750323	1085191	1061828	766142	663753	652712	523637	722321	888722	1004367	598891
EIC	Negative	243.1603	2.54	unknown	143713	111348	84884	65016	101145	106708	100751	63644	76154	102600	97840	67241	68216	68720	109474	92148	86909	55092	72623
EIC	Negative	581.3182	5.01	unknown	589502	739883	614803	538613	611349	672715	630992	582842	511742	589365	663978	587084	619839	578601	520591	571321	618929	473477	609605
fpHILIC	Positive	203.1500	4.46	unknown	15499415	8607508	8817014	15209616	11727286	14167996	10782714	7730340	12954122	13263986	10968052	11399175	12350485	7369152	12129864	12565389	10449254	8276360	9834371
fpHILIC	Positive	218.1386	2.04	unknown	14082830	14618374	14897893	11582367	10357654	17372708	11800831	10063203	9483082	8317458	14016594	12053488	9071088	15315663	15429776	9293815	8135930	9074077	12924724
EIC	Negative	443.2816	3.10	unknown	329187	87160	368864	302872	79380	110363	80599	272702	74280	262344	81534	118047	82583	92520	82086	51051	74161	304943	61009
EIC	Negative	634.7207	0.52	unknown	189533	312860	385265	400638	152506	250035	258474	127138	375667	248992	275037	284827	295155	0	0	262088	274695	286789	257400
EIC	Negative	590.3472	5.01	unknown	358968	421943	398656	334753	394350	379092	423268	339293	337046	314967	431029	381106	363044	349678	335645	344965	363386	272696	423702
fpHILIC	Negative	199.9691	4.08	unknown	9060309	4959400	13043505	6223767	3764154	2986098	3743857	10267384	5344465	10202881	3943023	2432044	3612472	4834358	5694262	3704431	3258588	7115010	4495311
fpHILIC	Negative	332.1361	3.43	unknown	1521500	1761778	1717728	2172609	1977556	1645962	1626006	1981469	1758724	1878176	1954863	2126884	1781625	1679753	1490796	1736633	1773600	1788095	1570525
fpHILIC	Positive	540.3656	0.61	unknown	1575962	1306124	1808695	1174031	1589629	1179980	1055877	1646009	812174	1427558	1374568	844782	1183802	1019296	1272655	994657	1461354	1261860	852800
fpHILIC	Positive	538.3868	0.53	unknown	925378	848414	1031404	907233	849214	930731	750100	1049613	792734	798212	587780	730736	674565	882507	891365	834718	673328	812255	854584
EIC	Negative	430.2970	5.65	unknown	384685	529010	385162	603082	501693	475594	418132	393204	371099	401555	529062	461449	435122	354231	416025	455044	414832	386873	336187
fpHILIC	Negative	180.0593	3.34	unknown	4613967	3804026	4269101	4824165	4383221	4325083	4602778	4106913	3713285	4029242	4416268	3922383	4723249	2713946	4076684	3676839	4252700	4074645	3583024
fpHILIC	Negative	145.0049	4.03	unknown	428294	179239	379708	411163	205545	252993	242856	547554	164545	409179	226300	213887	185231	273853	351088	177479	163277	386522	237323
EIC	Negative	446.3000	2.86	unknown	223240	89816	267822	207554	57453	75068	127197	308833	0	187246	35313	125093	50926	167909	113000	29418	111922	226172	48803
EIC	Negative	239.1652	2.31	unknown	388866	171175	330298	909541	276861	299228	260214	471592	277744	572553	248709	478803	275271	102535	162716	346702	235407	612998	118504
EIC	Negative	327.2907	6.58	unknown	904303	810225	1131412	1825245	909072	762610	927367	1044455	1206086	1252206	926914	833615	1292706	573585	549555	1234821	936529	1211456	583236
fpHILIC	Positive	391.1016	3.16	unknown	5297779	5971487	6637688	6356158	5626895	5483526	5686870	6132066	5353493	5980533	6186588	5790049	5995542	4936129	5484907	5202066	4929073	5638485	5085832
EIC	Negative	529.3329	4.88	unknown	309214	162214	330342	662725	159768	237702	322188	382852	408399	569978	273200	231326	416927	0	108194	316930	301878	648058	32210
EIC	Negative	279.1554	4.08	unknown	107131	83675	129025	519111	157849	118056	179909	187175	250460	212603	156778	227180	211502	38759	81081	214157	170930	295642	70459
fpHILIC	Negative	165.0920	0.44	unknown	5131983	2690268	4214309	5329063	3435696	3095628	3481273	4439755	2187743	3584725	3062117	3554465	3172678	1654706	2796026	2758284	3102658	4569233	2275265

EIC	Negative	237.1495	3.49	unknown	143832	59576	141287	187191	66140	81192	92198	113196	46952	113100	53628	99044	83798	38005	61035	63754	57320	152436	37828
EIC	Negative	553.3346	4.24	unknown	8727865	11114114	8203213	7056937	9153923	10611284	12314262	7587574	6777794	7090394	9332622	10756093	10142709	10646159	10430991	7704811	9165396	7177621	8802981
EIC	Negative	321.2444	6.22	unknown	1323800	640140	1455809	2439109	740506	855566	623081	1135766	776261	1464071	650564	941330	746221	411214	437807	648457	645324	1653038	465467
EIC	Negative	528.2855	3.96	unknown	291913	415463	335445	261841	294228	349381	368429	277474	339700	269708	319272	367381	355645	419970	333650	361462	285407	269052	270419
EIC	Negative	235.1339	4.12	unknown	385000	261277	461175	944065	308646	235499	317458	468147	240496	438856	191164	202482	419371	88626	171803	442662	256119	500297	94212
EIC	Negative	340.9345	0.52	unknown	2002644	2804536	2778677	3276263	2190496	2293557	2658572	3276614	2734855	2322164	2719370	2867401	2804876	775971	2220405	2438131	2851149	2879706	1886522
EIC	Negative	458.6535	0.64	unknown	105684	58350	112544	84236	111613	66877	69757	44200	75357	183705	44299	37286	64190	213679	56647	54267	32426	69534	130414
EIC	Negative	480.2998	5.09	unknown	2226842	2357976	0	0	2379587	0	0	0	0	0	2521151	0	0	0	0	0	0	2221041	0
EIC	Negative	286.7725	0.64	unknown	179674	68314	154883	117266	185946	112789	120161	75122	106175	271091	71836	50979	87107	714953	94392	81579	68096	110953	205464
EIC	Negative	313.2387	6.21	unknown	226707	114451	399453	180524	137917	36397	137414	277450	85142	216292	117270	191093	58059	57692	73315	97375	84794	270539	103761
EIC	Negative	248.1370	3.70	unknown	69704	57525	148090	58287	66337	106960	111254	114598	0	147669	138176	107198	51408	70138	85559	61216	69584	119951	36541
fpHILIC	Negative	171.1391	0.42	unknown	3292160	2507721	5371245	5226317	3423119	3214635	2832643	3166549	3553713	3477521	2710566	3306524	3698037	2934866	2274903	3049538	3276743	3645840	2883775
EIC	Negative	500.2274	0.81	unknown	141018	192947	146473	88786	107523	153246	166548	177936	91794	121839	152560	150941	161683	108796	135989	118736	133085	124949	138718
EIC	Negative	247.1340	3.69	unknown	401288	272334	898997	367853	371615	604657	582305	674663	116650	835995	819168	626695	256174	374743	485747	361107	426731	720511	203159
EIC	Negative	329.2333	6.05	unknown	1048525	415954	1116313	815791	399064	287171	389475	869925	217207	854327	392861	310775	363188	271727	454389	242164	285952	625736	414579
EIC	Negative	238.1460	2.16	unknown	124467	87239	77402	306019	85382	93624	94775	106312	100667	129534	84121	172203	84625	51187	80671	93054	91941	111808	72169
EIC	Negative	285.2072	2.28	unknown	122316	69471	127357	80859	82316	86064	102870	75272	75736	92060	56695	83935	76514	62894	53716	100601	66652	109469	64634
EIC	Negative	311.9939	1.19	unknown	103956	93622	88610	87892	81388	84519	89348	93181	79307	83681	86229	75752	77078	72566	76371	68602	73947	107511	73811
EIC	Negative	634.3348	3.96	unknown	627737	1011643	644641	575851	777513	804317	904796	653712	827527	620285	775910	840355	850881	1043440	919459	828199	716153	550631	698426
EIC	Negative	552.3313	4.24	unknown	29123374	37289486	28179852	23873428	30434928	35287638	41057897	25504885	23156427	23865426	31918226	36428756	34212434	36437380	34950744	25717915	30999377	24043419	29753094
fpHILIC	Positive	141.0539	2.67	unknown	1105104	271498	80679	161878	209947	474059	0	258805	443692	158067	74561	350321	243149	349822	47298	318956	305635	416835	208912
fpHILIC	Negative	168.0244	2.98	unknown	9669978	8683618	9676929	11285640	6089937	8054070	7389834	5297628	5872444	8200062	7409801	7008146	7589817	3042908	4722958	6106222	7066507	7394089	5330230
EIC	Negative	570.3417	4.05	unknown	2314717	2416954	2101681	1989949	1818879	2300406	1890286	2127134	1441722	2101312	1985547	1933761	1896083	1736222	1714070	1828994	1823130	1789030	1814032
EIC	Negative	312.9867	0.55	unknown	582784	211095	226870	560892	144223	495385	241267	118463	140106	250765	258980	264936	185312	46686	216894	293913	136888	199655	219970
EIC	Negative	229.1810	3.83	unknown	123991	90888	135601	305963	94698	78639	95390	109672	118201	146085	76833	98514	145318	54656	58825	143154	93284	116351	53702
EIC	Negative	339.2123	5.49	unknown	358691	130633	289757	325663	152319	125816	218305	186203	151345	250262	179455	113235	180562	228274	284285	64526	202006	116550	114560
fpHILIC	Negative	235.0811	1.89	unknown	737688	213077	510950	499070	224936	488442	320813	410317	455051	404151	366649	616519	208177	280216	202910	514822	168149	466501	428821
EIC	Negative	470.2164	0.82	unknown	76741	98774	110167	56890	60573	79417	101647	85581	66732	78569	108386	96276	75875	69619	82521	65399	70032	70632	80937
EIC	Negative	338.2415	3.54	unknown	140271	103708	138886	390312	121943	119019	119843	178089	156735	177457	109289	196669	152476	61263	82010	136743	111805	245474	76480
EIC	Negative	284.7750	0.64	unknown	269796	97240	229380	179544	277448	158128	174074	105373	162367	419190	105499	83148	128003	1048194	142983	126110	92599	158422	302914
fpHILIC	Positive	186.1123	2.36	unknown	796666	973846	806057	860133	801426	833568	839420	675790	916199	1008676	1068862	941786	1016629	786639	618047	986527	829725	558844	821580
fpHILIC	Negative	239.0774	3.19	unknown	675237	734642	751162	949297	683687	676485	836592	605430	658427	634989	735697	517167	695584	390430	598477	591994	716164	728246	614012
EIC	Negative	616.4305	2.72	unknown	315571	210258	197921	601619	318410	203445	241815	224897	261654	372820	195055	247289	288511	111938	146153	286725	273349	365820	101694
EIC	Negative	578.3390	4.01	unknown	2927340	3890580	2966512	3349669	3146451	3392449	3846098	3178931	2951228	2529015	3820439	3913471	3215422	3450718	3357374	3223110	3111435	2464832	3351693
EIC	Negative	389.2728	5.84	unknown	567579	547377	587352	660432	156887	168027	748928	632102	158895	324067	479579	153958	165460	591454	633745	148171	475930	351034	142422
EIC	Negative	286.2474	4.74	unknown	106223	81729	213382	221660	134786	85428	123429	238945	132187	123817	78983	84009	105892	139304	87230	85319	129490	237256	131988
EIC	Negative	502.2845	5.24	unknown	1100323	924387	944824	0	901846	0	803616	0	0	0	631099	0	0	0	0	0	742354	0	859592
EIC	Negative	316.2572	3.54	unknown	161099	158367	157131	160446	160886	163080	154075	146513	149343	148467	145984	146709	155124	142570	144419	150460	146968	142330	142822
EIC	Negative	248.8995	1.19	unknown	105042	54906	80508	57819	51230	46916	44017	32380	42152	90557	35996	28773	26136	52829	32502	28740	25374	55876	61114
fpHILIC	Positive	157.0749	2.61	unknown	1215391	1367324	1186565	1033447	1074021	1475213	1304342	928573	1003229	1037746	1402534	1205343	1064247	1240779	1228636	1041928	1013428	1089205	1063139
fpHILIC	Positive	760.5779	0.44	unknown	22934636	25387116	23908926	19226456	20901790	19709264	18279486	20215376	27668790	18671904	23210516	21868918	19192314	28839220	22420734	18874560	19589230	16674183	22788944
EIC	Negative	310.9892	0.55	unknown	1811844	605143	742548	1616113	497368	1548781	764959	325703	415703	775726	768826	827610	586817	310001	657389	942879	424698	613052	701164
EIC	Negative	244.1998	4.13	unknown	63976	61845	81879	69393	72334	73160	69175	64409	66388	72101	58653	72765	66076	73575	57762	69401	63803	90554	43284
EIC	Negative	275.1268	3.48	unknown	167447	99159	124951	141880	105874	80923	84668	89587	41479	124802	75782	91498	83242	54787	77460	90793	54340	110477	45406
EIC	Negative	296.9919	1.19	unknown	420892	426183	388577	377124	385254	398955	394003	385620	381116	368645	396640	391100	359705	371675	371343	351831	373675	359733	372240
EIC	Negative	318.8873	0.52	unknown	756665	731796	1095653	1127670	751521	793744	805420	693445	963636	792637	740175	808320	864116	321997	739064	813971	721061	880094	614571
EIC	Negative	251.1323	3.18	unknown	355339	0	0	0	0	282141	0	0	0	0	0	0	410058	0	0	0	265342	0	0
EIC	Negative	581.2410	2.93	unknown	1981133	1179066	1002934	1514658	501999	1031321	604992	1725324	385828	2973568	904377	1140000	1109187	169505	350509	1181576	517431	1594646	352368
fpHILIC	Negative	80.9169	2.10	unknown	5687029	6476060	5036893	5696109	4995333	7158834	5607138	4623500	5662901	5135114	6287215	5867647	6310368	5704582	5838500	4749240	5422366	4861904	5512702
EIC	Negative	283.1920	3.20	unknown	577023	221676	602588	482883	223180	250136	246799	425458	241856	404789	203476	284145	237823	161273	204466	207652	222517	499589	170956
EIC	Negative	609.3233	2.33	unknown	206832	141961	96406	0	113609	136174	138334	87741	101678	167709	121053	176684	89703	164475	116754	128461	120653	0	177472
fpHILIC	Negative	224.9798	3.00	unknown	787261	773025	729251	897146	592975	598586	710831	687870	763682	662377	583890	657085	603047	481905	524688	665410	725122	680648	560046
fpHILIC	Positive	315.1266	3.45	unknown	835254	637817	883977	834028	768235	772255	720182	890002	615744	817957	647932	723085	717453	690390	822607	753210	725867	801608	440750
fpHILIC	Positive	876.5581	0.38	unknown	463399	316846	398836	417032	344419	344000	249359	300113	510610	433949	401605	324851	295307	385504	303294	312596	317336	293753	418587
EIC	Negative	283.1348	3.13	unknown	494032	221449	636158	128416	212433	318722	205418	351227	76057	346121	180540	131368	99406	345189	198175	71509	128087	362640	335048
fpHILIC	Positive	830.5677	0.43	unknown	3108080	5329633	3665800	3155209	3401133	4333566	5149753	3315059	2002284	2317631	3937522	4264914	4001829	3395876	3101216	3577540	3766358	2601771	4095606
EIC	Negative	392.9921	0.55	unknown	1478102	663526	713906	1764928	486161	1493526	855247	408406	437190	667461	876572	958900	666587	199755	668988	964150	491525	701592	640936
EIC	Negative	528.1776	0.71	unknown	166677	224514	292600	150591	160784	184958	218744	181823	179141	212076	235944	192779	192783	157147	172225	154114	164236	155139	226415
fpHILIC	Positive	390.1048	3.16	unknown	1079513	1105518	1341860	1298450	1053681	1003986	1194813	1170572	1093658	1322239	1327078	1187123	1166941	1149054	1084725	969735	994532	1088001	1064231
EIC	Negative	410.2373	5.74	unknown	1017701	457382	811489	1313507	508823	597849	596572	806392	577495	760764	501617	634006	601795	323397	460514	554173	515723	983797	396978
EIC	Negative	445.3328	6.50	unknown	3078538	3178388	4347819	4979758	3491593	2041719	4207586	4337847	3495964	3959653	3843295	2711357	2530421	2062361	3444484	4426439	2879838	3135866	2695186
EIC	Negative	430.3537	5.01	unknown	646125	682977	585964	486416	687667	522036	574325	467508	569776	453037	568391	488941	479735	468780	487017	562628	560964	384085	668123
EIC	Negative	261.1508	2.49	unknown	132551	34883	69070	61953	64957	79411	59122	87587	31499	127668	49164	39732	39858	26500	35143	60841	36799	139398	32320
fpHILIC	Negative	285.2070	0.54	unknown	2596732	1081916	2944703	2585275	1323842	1395341	1532553	2335767	1336792	2719560	1072931	1395057	1103841	1063761	970095	1066795	1118058	3178743	1148021
EIC	Negative	290.1096	3.18	unknown	127553	90591	104763	183187	80711	90129	87702	111243	52550	120498	77027	92051	92819	32016	87215	70913	62398	116611	61662
fpHILIC	Positive	180.0761	2.67	unknown	816135	184247	96247	140391	186070	297595	50527	248380	356918	60170	86898	297755	239761	301353	68909	223404	235926	315903	231893
EIC	Negative	339.0967	0.92	unknown	99839	78264	125829	126137	68183	55986	89427	109138	56469	96846	93512	84046	91290	27814	65054	79038	54931	118984	40120
fpHILIC	Positive	768.5935	0.43	unknown	1029107	1401449	1334450	1133708	1228318	1090541	930542	1191793	1254505	956711	963104	874850	825828	1002808	971999	1109966	952539	1277648	1067868
EIC	Negative	443.1883	3.26	unknown	279402	273943	322587	789003	128964	276940	178792	97824	74482	332071	165724	98948	47249	0	196622	271277	74383	110598	62284
EIC	Negative	443.2996	5.95	unknown	62401	38212	70845	135966	45880	56356	40589	80653	65196	51106	0	50634	52019	30367	35788	43266	50416	92162	51128
EIC	Negative	259.1915	2.98	unknown	85032	43586	81289	74998	27797	45612	59066	50170	39826	48403	51239	62948	74602	41766	48912	42979	40954	42376	34992
fpHILIC	Negative	307.1150	3.47	unknown	1085983	888414	1069743	1136087	702364	908836	1158303	854311	718656	1001701	821862	674895	929001	461837	927483	747286	902899	965483	560448
fpHILIC	Positive	519.3254	0.60	unknown	1856417	1808284	2477730	2204313	1868956	1692954	2297396	2054595	1582192	1634491	2256143	2455529	2107644	2297520	1888986	1686720	1926067	1887800	1587985
fpHILIC	Negative	307.0644	0.43	unknown	9999576	5191436	9862234	5936451	5587365	5400446	6304244	6554013	2046803	7592197	5729293	5546045	0	3636430	6448516	0	4576709	8299232	5155494
fpHILIC	Negative	512.2683	0.44	unknown	37327204	372096	66084580	92799616	110088	402852	572863	34785448	1182364	16765236	143001	103494	488922	352257	277277	257962	275396	34052196	171007
EIC	Negative	279.1603	2.69	unknown	824432	814505	660009	571989	661409	643089	876324	716911	619701	570017	577174	549536	664571	754727	785672	717065	595000	532521	695995
EIC	Negative	398.2347	5.23	unknown	555115	229751	517499	403229	325874	310627	292712	508436	109215	388258	252534	348116	364179	222528	304991	265658	264193	418995	209352
fpHILIC	Negative	284.2681	0.37	unknown	21458350	29351086	33122030	34842604	29520668	25744132	29745978	23439640	23188262	25745026	21906460	29429048	21152104	25981016	19480334	20803250	24480818	28187184	31581034
EIC	Negative	389.2702	5.97	unknown	896070	612252	946231	985073	532779	399090	544288	752142	521389	982764	646483	404520	444356	398331	652607	437749	451678	702754	356848

EIC	Negative	258.9312	0.52	unknown	1349925	1589103	1815562	2094668	1380903	1420654	1506263	1579646	1641895	1388683	1540320	1548420	1610959	695769	1370033	1469530	1472263	1680940	1132168
EIC	Negative	237.1495	4.69	unknown	836325	236083	898131	881413	295790	454997	362364	578814	193648	618998	271617	569425	423423	101005	288649	287156	342796	639182	226676
EIC	Negative	398.8929	0.52	unknown	1218796	1623102	1875928	2108954	1278671	1521618	1569558	1577773	1710791	1329723	1599646	1698650	1634412	497810	1340477	1571236	1572536	1688971	1074655
EIC	Negative	300.2566	4.40	unknown	1906802	1753036	1778316	1739286	1814431	1562566	1527524	1718432	1691683	1833229	1423304	1431283	1608591	1578089	1530097	1500234	1551702	1764503	1522495
fpHILIC	Positive	276.1549	4.03	unknown	957357	344799	511881	1062036	469745	598908	384621	400292	526896	615056	797709	851211	526309	323921	338553	713941	390436	363153	447135
EIC	Negative	268.0978	1.76	unknown	101472	77439	145953	99004	60569	73659	60823	55584	69914	96458	77371	80433	82329	111689	98033	70075	74018	0	61365
EIC	Negative	277.1555	4.08	unknown	0	0	0	1065283	0	0	395001	412024	562194	461453	325784	473162	475657	0	0	394570	350274	551738	0
EIC	Negative	363.2545	6.03	unknown	2868011	2690105	3159541	2975963	2978323	1907768	2061278	2950733	2404241	3584187	2844099	1816399	2113219	2161811	2917969	2001938	2337578	2564535	1831464
fpHILIC	Negative	582.3404	0.58	unknown	18305818	20945290	18954646	12989921	15672723	20518288	21496966	15612538	15128867	12953465	18965544	19751158	17445396	17069846	19563876	16219472	16796432	12638768	18588328
EIC	Negative	343.1543	2.84	unknown	240774	337923	254507	163081	271019	218240	310419	90723	147224	126755	143916	243639	144088	200554	321142	253911	143336	43489	221548
fpHILIC	Positive	131.0531	3.47	unknown	7743326	5660131	6650137	6350943	5745405	6024646	5923773	6157616	6239970	5936638	6140627	5796029	5340475	5018245	6474518	4941637	6099349	6118190	4902959
EIC	Negative	554.3374	4.24	unknown	1837215	2338936	1820487	1493593	1891847	2220444	2529323	1698592	1441188	1533580	1962006	2280375	2119957	2267378	2292480	1571727	1953764	1575904	1884213
fpHILIC	Negative	182.0318	3.03	unknown	2445789	1428632	1440867	1675770	1129646	1148513	956741	1208585	1191205	1563704	1184565	1043965	1116733	1099824	1247846	996897	1169939	1035872	894889
fpHILIC	Positive	358.1633	3.47	unknown	2819409	1888881	2480313	2646259	2230605	2328876	2382898	2768048	2403803	2653747	2659292	2296246	2446606	1994463	2658400	2548778	2300763	2582510	1408020
EIC	Negative	256.9340	0.52	unknown	2003816	2381305	2762636	3142147	2163080	2244063	2324956	2430118	2524547	2142400	2335964	2401761	2410344	1055027	2117815	2363367	2329477	2538843	1744177
EIC	Negative	361.2389	5.27	unknown	696040	455123	715863	726907	490916	316855	374696	542009	417716	859814	497150	322471	368968	264658	494671	349017	336418	622132	231390
EIC	Negative	326.2217	6.21	unknown	765667	2725178	760525	2792022	1736726	1537670	1455990	1041349	2778171	1306993	1861373	1467594	2758086	1173032	1502586	2090335	1901825	1286168	1337695
EIC	Negative	486.6876	0.52	unknown	164807	29643	163930	0	139884	97090	93816	0	0	194677	58594	0	76308	215076	51732	49780	25734	30158	156749
EIC	Negative	256.8834	0.62	unknown	121314	49634	32730	78642	35434	118258	61833	18844	33704	42099	64230	64360	48944	32409	59158	73587	33900	34559	53422
EIC	Negative	251.1268	3.18	unknown	415518	314530	525361	441470	346921	297112	413506	350831	171733	321045	329495	349068	511772	200390	312674	330115	283759	360968	213336
fpHILIC	Positive	761.5842	0.44	unknown	7139867	8331139	7556628	6197559	7121225	5996955	6423026	6861097	8948146	6079820	7426649	7137849	6668295	8859416	7468799	6264244	7091169	5522027	7232476
EIC	Negative	452.9477	0.55	unknown	420307	140309	207775	363636	114508	380095	192690	88725	119859	218637	185941	200905	158925	27528	141938	240323	113367	151547	203836
EIC	Negative	284.1954	2.67	unknown	70267	46871	48063	89450	38644	62927	57632	64413	43919	105926	64860	116089	74560	58963	48088	58943	55899	47102	44104
EIC	Negative	516.2736	3.11	unknown	650860	338521	400735	408015	237716	513106	217289	442291	349512	496790	248320	338722	270523	222139	347255	327469	474814	0	522275
EIC	Negative	400.6941	0.64	unknown	108734	58101	118430	84004	124796	71316	80107	46858	72540	192601	45046	36039	59926	289304	65016	59553	41458	77714	132158
EIC	Negative	571.3451	4.05	unknown	684699	701429	603909	618758	551916	682658	567207	625519	426483	638202	588033	583323	578276	498055	518243	538605	583619	532746	539954
EIC	Negative	265.1811	2.99	unknown	400336	220885	467966	587440	288356	369031	354390	427652	240017	494711	299089	532678	425364	225318	234655	354986	251383	550259	205498
EIC	Negative	596.8132	0.52	unknown	480198	683648	845960	988609	524854	673725	660501	629986	808708	783847	755392	759821	762498	0	565370	746226	672141	733707	418665
fpHILIC	Positive	106.1110	4.07	unknown	648541	498220	471650	395026	373151	531501	528722	472718	372530	385762	267884	321925	349372	297515	524595	526322	334236	412730	370653
EIC	Negative	386.2374	5.75	unknown	4460559	2437845	3925388	6324819	2228678	2518606	2459382	3122789	2634885	3682629	2475708	2751223	2538050	1698842	1941293	2643678	2121551	3979416	1653652
EIC	Negative	287.1868	2.46	unknown	947351	303459	1048549	675531	409984	388276	487121	1090865	173205	1042744	403099	406220	346470	346706	359474	288874	354723	969130	458390
EIC	Negative	571.3450	4.18	unknown	4303352	4012269	3906674	4044177	3339140	3999165	3538346	3975959	2810868	4088616	3881140	3449463	3396931	2952049	2800110	3453160	3388303	3593550	3605994
EIC	Negative	634.3345	4.23	unknown	1367962	1430809	1190528	943500	1299759	1276768	1572033	1089109	1146962	1070817	1340130	1481360	1522019	1593278	1576535	1088792	1166696	1060474	1196477
EIC	Negative	356.2172	3.26	unknown	406547	75297	440672	184017	80608	123363	123222	250064	29235	273826	64753	108472	62408	81035	73915	29541	68710	316360	89979
fpHILIC	Positive	782.5682	0.43	unknown	81311248	94635952	81127816	81318160	72673336	71569192	73059496	72851776	91005296	67151640	78715832	78400448	76300464	90854856	67701664	72443888	77611784	63109884	83137664
EIC	Negative	339.2539	3.83	unknown	94110	81283	178361	460579	60664	94521	76141	170851	110152	164846	99677	155117	136626	43238	36476	127437	88480	185970	50978
EIC	Negative	284.7750	0.52	unknown	408102	109296	311301	183017	397304	240719	209842	91158	223745	579025	85090	104858	146605	2121643	167177	167312	77643	190527	507969
fpHILIC	Negative	182.8247	2.10	unknown	697112	1100155	885346	948344	672406	746892	648829	928430	599982	1147337	966991	858283	612245	1136699	1096492	286640	706222	577699	769535
fpHILIC	Negative	269.2486	0.37	unknown	4060133	5860224	6752783	7580869	6182097	4734293	5562824	5557130	6637801	4712459	4305710	5722385	5475789	4291966	4104772	6051602	5192955	5903772	6147380
EIC	Negative	457.1721	3.26	unknown	133826	135071	173406	442695	62996	117849	82246	40075	33345	159483	77524	49483	19182	8321	88768	126597	43825	47284	15105
EIC	Negative	570.3417	4.18	unknown	14688244	13531302	13232176	13726211	11418494	13335958	11605781	13412004	9566463	13547040	13092752	11570324	11687660	10067954	9627544	11291522	11337871	12275518	12183640
EIC	Negative	337.2328	2.15	unknown	1704757	1502139	1423958	1665160	1533040	1476893	1609414	1505349	1472142	1499886	1476863	1558925	1481742	1487295	1460483	1455236	1517455	1299050	1530374
EIC	Negative	577.3347	4.01	unknown	13705935	18151230	13489229	15338190	13406625	14470475	17307855	15028763	13372471	10766551	17041248	18398447	14754738	15932822	14994885	14096628	14559527	11112301	15586547
EIC	Negative	324.2560	4.01	unknown	377396	356352	326463	293696	356589	0	345996	12597	328680	317674	306069	323828	316982	341410	330217	0	0	351762	316046
fpHILIC	Positive	224.1118	2.72	unknown	620840	520827	828656	972723	547204	547513	768783	578241	394112	924279	413157	473369	548284	277053	484377	490462	584028	716621	436074
EIC	Negative	342.2731	6.20	unknown	754267	541010	1122925	1059777	504176	387694	563398	656356	461810	528246	446348	583139	551801	363604	334721	466301	467764	862095	329911
fpHILIC	Negative	157.0366	2.97	unknown	24738772	21909210	19905114	24463130	17039328	25508022	20372286	9582873	17114652	18164308	21644108	22394824	18968772	5525035	19303838	23779088	16258658	12578800	13776961
EIC	Negative	577.3347	3.91	unknown	3393976	5074447	5172705	3354767	3547035	4673994	3753714	3913633	2424584	2712562	4454041	3703020	3414911	6130119	3351494	2448294	3291049	3370074	5053651
fpHILIC	Positive	734.5675	0.44	unknown	1154345	857268	892966	1062529	702502	707197	786067	884361	1208845	814798	883633	923572	679882	1041164	813027	850858	862808	752900	823472
fpHILIC	Negative	145.0871	0.46	unknown	1417030	1141637	1372776	1374184	901081	1544035	1074461	981394	1531750	1359603	842443	1485216	1321450	1274086	770499	1469655	1282130	1225507	1024988
EIC	Negative	322.2965	3.74	unknown	151162	159833	148014	153485	154348	161021	144353	159681	136796	135021	143937	156320	156781	150207	155595	143356	140358	144462	139855
EIC	Negative	251.1483	4.72	unknown	482127	597601	300020	275195	511472	419303	475873	190395	391316	250579	671228	533497	395124	600769	456435	484527	382122	179384	400781
EIC	Negative	247.0954	2.52	unknown	181962	54718	106782	93649	48755	75672	60928	83221	32212	115839	58770	64538	44579	31789	56135	38149	53466	96894	43396
EIC	Negative	341.2699	6.20	unknown	3250740	2435450	5132303	4649924	2153472	1782287	2591698	2809567	2069773	2557009	1851365	2442118	2292824	1629550	1343229	2175983	1965132	3988965	1453660
fpHILIC	Negative	119.0351	3.34	unknown	14518975	12522964	13698713	15151743	13418145	13583649	14905227	13459388	12470474	13285216	14951040	12612204	15281758	8911568	13496789	13133984	13495849	13039428	11849277
EIC	Negative	254.0340	0.55	unknown	233156	85725	105741	261607	82352	227424	138224	41161	72947	113845	124070	139600	89029	75803	94191	139067	80868	128172	96619
fpHILIC	Positive	523.3589	0.57	unknown	27481524	30890478	30515910	24567314	25668894	28614634	34221980	26974564	20279204	23245148	26016914	31174514	26997380	25857466	28862324	24234024	26707326	24493042	25863632
EIC	Negative	392.2237	2.48	unknown	405057	337509	396125	431152	335833	280360	315170	295184	271855	190544	314167	319158	276459	307747	326400	327640	323531	137617	321479
fpHILIC	Positive	482.3239	0.61	unknown	3438948	3131597	2360477	2374142	2912318	2355332	2839378	2279023	2639233	2754282	2657229	2781856	3068808	2425808	2159290	2688628	2244976	2822462	2574299
EIC	Negative	603.3501	4.52	unknown	64938299	65080952	60525304	63497221	63257625	64227157	65414949	69047226	61990823	60289602	62868533	68180919	64088087	61510990	65101765	59848157	62351825	58954002	64394625
EIC	Negative	278.7578	0.62	unknown	69234	41270	58893	45616	65982	45230	47725	27449	56461	86783	28692	32344	41339	204603	39571	41641	32032	39880	79781
EIC	Negative	518.6043	0.64	unknown	117192	48201	123264	89079	114679	75384	74980	49123	71977	207621	49573	35302	64667	159175	54146	60429	32955	81556	141272
EIC	Negative	237.1859	5.15	unknown	4924905	2569405	9822355	14643249	976431	782265	942270	2007879	1095236	5313379	1285331	10636300	1605567	1115243	491013	802079	572244	1923723	1778160
EIC	Negative	335.1857	3.98	unknown	132324	54283	196728	236833	44568	44447	48960	68825	43487	155429	41581	183465	59780	29791	34698	38975	44636	87086	34680
EIC	Negative	400.8909	0.52	unknown	361164	499984	619746	672982	435909	471362	506215	547705	524306	596406	553590	537767	526265	186453	427657	554278	513739	528927	359669
EIC	Negative	314.8929	0.52	unknown	2483834	2537017	3431420	3545259	2456265	2576527	2455851	2352232	2921534	2555761	2531793	2596503	2574044	909709	2297714	2713737	2349631	2810334	2071797
fpHILIC	Negative	173.1043	5.40	unknown	7656529	7364110	4579762	5170194	5243539	7265918	6051219	4587402	5132858	3844231	5048030	5323667	5860003	5564469	8238523	6316023	3610743	4090861	4876759
EIC	Negative	255.1604	2.09	unknown	452522	292119	468979	287120	279841	381133	284062	321037	302546	390238	290542	386175	331282	268802	312491	343288	301928	294505	252794
EIC	Negative	290.1799	1.70	unknown	76422	61946	145082	226508	98971	102015	109573	122258	81338	156143	82318	87505	113544	35416	100955	93873	100876	148268	49354
EIC	Negative	303.1576	3.51	unknown	66553	19168	54964	289581	90012	35285	31295	127740	11583	45189	25116	33847	7648	32730	28984	25589	27938	195351	6139
EIC	Negative	576.3314	4.01	unknown	43498008	57939055	43269356	48092160	42198365	45342211	54579399	46695649	42177708	34140292	53564278	57237882	46662310	50570690	47493932	44620098	45426858	34813764	49378385
fpHILIC	Positive	132.0537	3.47	unknown	977930	643324	858105	808317	840385	711844	699290	660132	741522	740585	756791	640005	684499	508090	699925	556741	818161	848561	583666
fpHILIC	Positive	163.1100	2.92	unknown	2286222	2448459	2522646	1586502	1351490	2282575	2446374	2057570	1271217	2092401	2221635	2201104	1731914	2023357	2002534	1662494	1692612	1621919	2056020
EIC	Negative	384.7188	0.63	unknown	346885	277308	695848	508509	321791	366391	285939	232152	440809	889517	296609	272633	329096	176690	145752	197071	236962	380767	621388
EIC	Negative	295.1818	3.52	unknown	1257479	0	907324	0	0	0	0	841048	0	659975	343796	0	369821	357307	483989	323802	0	0	0
EIC	Negative	462.2951	6.00	unknown	4362118	8032678	3801507	11883849	4282909	7952345	5146023	5053357	7387864	7297110	8014398	3239664	6296020	7012948	7054344	3120287	8846006	3958131	5674280
EIC	Negative	355.3225	6.73	unknown	298423	513997	451183	1182842	444704	455616	650173	572890	486816	444668	396000	860601	697147	290821	392985	509378	584544	695589	325379
EIC	Negative	635.3397	3.96	unknown	205153	331804	204132	199615	228705	236438	259568	204622	255590	198982	250572	273586	262564	311871	261464	291228	220455	158759	206817
EIC	Negative	324.1877	3.40	unknown	223484	109182	204239	106506	109380	136702	130313	131783	44804	172761	99211	134365	88996	74769	86733	82408	102329	173835	86137

EIC	Negative	335.1415	3.65	unknown	645414	640326	480198	537287	566585	498637	559162	600945	592327	633655	545181	558868	506386	516865	534204	530090	373081	643405	627218
EIC	Negative	570.9313	0.55	unknown	76468	75790	92048	112093	69435	19594	88659	134285	94081	43619	67296	69612	79242	0	51159	107402	92475	139513	0
EIC	Negative	281.1765	2.93	unknown	765906	159623	584982	418063	189567	199301	193190	376429	158275	748107	138988	194440	152912	185972	229459	142532	167075	380019	155148
EIC	Negative	315.2090	6.47	unknown	2987879	2783311	2828772	2342683	2795256	2625800	2391532	2518183	2165869	2890077	2336094	2146091	2143998	2210631	2357846	2388311	2275535	2493436	2508476
EIC	Negative	439.2618	2.35	unknown	848156	385132	684068	899831	351807	337355	359758	1083717	366121	948797	332718	346056	341367	341064	344706	346226	328109	1018329	367153
EIC	Negative	276.1305	3.33	unknown	135692	119295	88791	113945	119319	107780	102202	113138	54186	92622	102554	102447	110180	56630	99932	117564	73113	112194	75288
EIC	Negative	298.2108	3.82	unknown	406598	122447	537934	455722	191213	190185	193993	400694	196598	448812	158407	185189	160780	197812	151106	154353	189816	490847	142795
EIC	Negative	291.1965	3.23	unknown	168864	118128	153272	294289	117249	133480	146587	180613	133700	187899	125768	180008	173967	113929	114247	140180	148624	190008	92039
fpHILIC	Positive	230.0956	3.34	unknown	10496189	6227775	8161470	8978708	8102839	7671957	7716241	8056787	8341155	8371518	6916965	6754102	7313328	8321460	8018217	5750756	8002922	8260671	8375699
EIC	Negative	461.2908	6.00	unknown	11479961	22526932	10015840	35744784	10511666	21765913	14303984	13567986	21345421	21965695	23880029	7868379	17692498	19949010	20805648	7838748	25380814	9623141	14826780
EIC	Negative	343.0627	0.50	unknown	307601	248208	340255	240586	191003	220743	257948	220767	328476	290178	260388	225935	209001	0	196003	276946	216265	253710	231254
EIC	Negative	399.1175	0.83	unknown	65257	65241	121298	113218	51764	30229	80330	84195	40590	77047	77462	69229	82983	15387	34808	61055	54931	99932	25875
EIC	Negative	576.3314	3.91	unknown	10798178	16207639	16495186	10419022	11132266	14839962	11969364	12236321	7624738	8597675	14071757	11612849	10832806	19489138	10731994	7852659	10433132	10780498	15899639
fpHILIC	Negative	218.0519	2.99	unknown	548073	403229	861372	316299	202542	416959	495272	175969	146890	262828	317489	324313	263193	112684	187568	260845	261156	341493	334650
EIC	Negative	280.1887	3.78	unknown	238878	94844	156869	0	102032	149732	106898	160218	61752	157195	99684	135714	0	0	146146	111259	0	200230	102109
EIC	Negative	598.8083	0.52	unknown	292741	444961	558003	559463	322256	422904	380716	412837	493571	498260	434244	454647	480686	0	333780	475729	423655	476757	249422
EIC	Negative	255.1604	2.37	unknown	942665	451621	850489	882906	449287	660900	477582	711340	330318	849121	332740	507186	476026	396335	507810	418536	379015	855032	374970
EIC	Negative	602.3469	4.52	unknown	191798624	191602547	180110021	187803116	183787164	188792978	192400359	203802595	182918750	175485655	185411363	200983154	188987559	180805149	193049840	176330732	183840269	174701506	190179998
EIC	Negative	579.3437	4.01	unknown	470848	641943	529692	562895	480308	557693	610922	542687	488549	387705	537087	730647	556599	638680	534285	482728	555435	383545	629645
fpHILIC	Positive	219.1418	2.04	unknown	1593298	1611733	1797718	1267888	1163626	1907984	1285342	1155871	1167361	959733	1560662	1411571	1012469	1799230	1755527	1085542	959154	1063695	1581435
EIC	Negative	240.1685	3.03	unknown	111473	96200	206970	159854	93468	105908	96149	138918	99704	151863	88236	103599	94542	190511	63859	63254	104613	174584	109192
EIC	Negative	566.3470	4.94	unknown	1075205	1359322	1135022	879899	1092287	1275842	1290881	983052	608592	934633	1192303	1154753	1055865	1000556	1265339	898035	937498	761269	1206779
fpHILIC	Negative	326.1287	3.42	unknown	1361472	1072343	1264023	1534206	1050785	948891	1017536	1162431	1037104	1175491	1097503	1089507	1038580	813665	948471	1052837	941511	1245326	849520
fpHILIC	Negative	197.0780	2.93	unknown	583323	619797	587496	476488	418134	724098	539640	490035	321533	523380	683463	632291	537604	381634	520437	537470	474282	442037	433706
EIC	Negative	604.3530	4.52	unknown	14688276	14098235	13968243	14041059	13689244	14806530	13927909	14985305	14154828	13371341	13790545	15332405	14016320	13658646	14337397	12974265	14599378	12785090	14418239
EIC	Negative	528.3064	4.52	unknown	1788712	1724117	1635815	1508404	1468059	1498451	1516535	1882006	1423908	1563786	1623229	1759758	1711801	1607815	1662720	1520316	1570543	1245254	1524696
EIC	Negative	562.9574	5.02	unknown	556219	674486	582559	699138	553698	644189	598543	596650	616643	500750	597419	590090	539745	569985	630851	639563	549406	597775	490113
EIC	Negative	239.1652	3.02	unknown	711676	608919	1252931	1068547	586159	624768	571787	944013	660897	998888	589158	639720	542225	1265246	401814	415373	663082	1064851	727901
EIC	Negative	371.1350	1.62	unknown	231562	264793	319435	0	127306	360283	125377	245530	103802	262663	234893	193716	122247	527942	300867	125208	117585	116302	269826
EIC	Negative	325.2183	6.21	unknown	3056075	11368396	3033191	11314347	6712417	6202671	5895218	4249401	11108488	5336746	7588260	5930366	11310621	4639158	6129476	8521722	7611819	5180871	5580689
EIC	Negative	402.2952	6.29	unknown	304922	329030	361356	123460	45104	362549	369314	156170	43992	257803	285424	196437	194081	340962	422419	0	311711	61230	149399
EIC	Negative	311.2593	6.50	unknown	1007123	764099	1761020	3977868	974061	726447	1079552	1379291	1028628	1617436	1923516	1633483	1418198	465008	538057	1239946	1084093	1540435	615282
EIC	Negative	264.1322	2.28	unknown	194623	66857	215559	49634	54075	80457	45435	109466	9730	182213	52261	61473	8988	77266	66257	7931	19832	112757	82600
fpHILIC	Positive	231.0982	3.34	unknown	1216125	932554	1003244	1121605	998295	1032319	887469	1020365	877114	1198388	1017033	794673	886664	830985	910644	709476	893316	1070038	1074860
fpHILIC	Positive	290.1347	3.34	unknown	1456075	309511	639439	1330516	623452	375605	390586	567255	766564	879720	517079	391683	299357	270551	330720	550234	655623	610611	487826
EIC	Negative	269.1403	1.69	unknown	219349	437891	220692	255902	275815	150283	254574	158970	424295	175164	204577	208345	287422	270724	281336	397317	182945	185100	176365
EIC	Negative	284.1955	3.11	unknown	150613	83472	119839	146249	96374	88218	91505	136808	120748	119002	104239	107695	103934	94775	89274	110261	98752	138123	80094
fpHILIC	Negative	547.1688	3.36	unknown	8447710	8215375	9611043	10085469	7601032	7534564	7555384	9261601	7906915	9509900	8642050	8465705	7749760	7429106	7256200	7573921	7404467	8393824	7956001
fpHILIC	Positive	204.1537	4.46	unknown	1274488	767103	765238	1203582	1005229	1112426	798602	594739	993844	1118145	905445	878201	1011786	590164	1026834	1010122	796857	726743	800181
EIC	Negative	323.1854	3.40	unknown	1202323	503098	997838	499502	527070	681785	658661	681845	180428	769857	515285	817276	459521	328439	458864	404022	461152	890871	427858
fpHILIC	Positive	732.5513	0.44	unknown	1215050	1341227	1242698	1133975	981706	875534	947022	1020544	1362367	913634	893854	1094690	976773	1467447	1010700	873686	1138713	980392	1018567
EIC	Negative	269.0878	0.55	unknown	12464953	2584103	3960873	7501519	2060505	8009707	3871065	1409182	2047556	7323508	3576368	3724322	2634036	2097377	2629798	3651077	1861220	2774021	5932685
EIC	Negative	572.3480	4.18	unknown	928597	826976	845122	840110	676865	834263	715653	808353	565995	831834	743794	684585	737897	629161	602898	653181	703079	776247	752295
EIC	Negative	237.1495	4.60	unknown	1742922	539403	2024373	3960620	707912	856982	976529	1400408	576298	1610280	711620	1304523	1338371	275358	492795	868609	841328	1733787	439325
EIC	Negative	409.2819	4.45	unknown	577795	433697	477711	528102	397695	240725	587882	582021	534318	533302	446003	468778	543728	385682	398197	551652	432536	543000	343862
fpHILIC	Positive	522.3547	0.58	unknown	98566416	108538352	111105256	87689360	87601248	103948328	116650664	96483872	70867744	85343440	97383304	111940984	95047760	93238736	101401728	85234480	92866672	87395584	93812952
EIC	Negative	336.2550	5.88	unknown	245482	121281	297313	985267	143055	120702	144132	238432	258533	262554	134841	226290	189105	77834	99868	205244	160391	324518	86674
fpHILIC	Negative	254.0157	2.39	unknown	1110878	458791	662465	841263	364345	438799	379654	264883	374186	236556	318402	332740	349990	401152	406657	373397	328385	316437	340542
EIC	Negative	439.2442	6.00	unknown	795945	1347310	713039	1925184	767880	1321852	902144	977130	1290380	1452319	1395488	591107	1109582	1267856	1231374	609755	1506789	735436	1013153
fpHILIC	Positive	675.5420	0.49	unknown	617160	505485	390031	467218	448574	294969	402599	410156	290660	309893	321552	393028	363580	401278	407047	288067	389595	384856	322539
EIC	Negative	314.8426	0.62	unknown	101704	38132	28633	72214	32429	91342	52026	9394	24931	36624	56222	54119	35676	19095	47709	56792	24799	28756	45826
EIC	Negative	540.3314	4.84	unknown	650397	728123	586410	646097	626563	684625	715514	562508	676062	621208	716453	682046	683546	721003	619181	599282	649095	596107	704651
fpHILIC	Negative	111.0201	2.01	unknown	6425276	2660725	2467732	6785184	2716058	2292964	4037129	4973458	8561551	3965824	3120806	3143695	6954948	6385985	4769789	2367227	7516708	4408687	2854244
fpHILIC	Positive	181.0527	1.82	unknown	943102	923284	692990	1197290	922339	1073176	807690	622237	866999	994397	1030925	1018686	950008	674858	818570	1296007	718989	662273	672884
EIC	Negative	360.1875	3.65	unknown	1659341	1688621	1650746	1615101	1547577	1587102	1765872	1711720	1762714	1506237	1607426	1564050	1346710	1581636	1571463	1541180	1542833	1614676	1865609
EIC	Negative	588.3327	4.13	unknown	308434	330127	283658	279855	188772	283452	303228	267581	206415	222216	303024	324171	281506	298982	275020	193817	248198	197392	299375
EIC	Negative	316.8898	0.52	unknown	2371978	2389346	3287807	3454087	2326049	2359823	2380810	2149427	2840124	2423754	2444137	2396144	2477293	872841	2205023	2572628	2284809	2637307	2017300
EIC	Negative	578.3467	4.56	unknown	222760796	235975406	238185239	214192841	224893560	223979777	224694498	238662624	224385656	226266580	220065417	230803600	224514370	231945786	227994400	210844430	218572948	218880110	228592058
EIC	Negative	333.2200	4.63	unknown	373271	218642	630606	768120	376833	255992	302481	744190	351909	431786	279818	275076	269155	416901	297580	188127	374957	756460	357638
EIC	Negative	325.2609	5.21	unknown	1129517	1206116	1005544	1151130	1120010	1159114	1094038	1107306	1063304	1059904	1115407	1037212	1151133	986181	1139724	1069330	1067747	1046703	1066898
fpHILIC	Negative	102.0278	4.03	unknown	652921	511671	611753	729780	476085	521417	500357	747724	468839	686157	516803	380343	376032	603611	692759	361365	411742	655228	518254
fpHILIC	Negative	249.0884	0.96	unknown	1703584	872320	1147146	2079956	1357399	1217689	936159	815039	793443	1399403	1281113	1273157	1224861	945604	910398	1204227	958193	1173903	733023
EIC	Negative	296.8397	0.52	unknown	286056	209417	301917	280557	192778	220101	226123	209004	234562	322254	222169	186166	214639	134164	150489	201617	212499	203408	285079
EIC	Negative	307.2271	5.75	unknown	137046	66964	138090	255182	86384	82361	96458	135019	64997	149827	83704	81645	86840	38148	73327	65704	66551	194032	52941
EIC	Negative	572.7690	0.52	unknown	579262	540205	850476	753637	443017	570098	510947	519754	698991	535979	547520	499383	582264	0	464073	661843	488448	602892	451172
EIC	Negative	250.1448	4.72	unknown	2939820	4087640	2085545	1801518	3467473	2488942	3185912	1244572	2440413	1565869	4333497	3351240	2483949	3850535	2967591	3072370	2487706	1218681	2734925
fpHILIC	Positive	121.0723	1.93	unknown	2483722	2352569	2506716	2686327	2655190	2848071	2067740	2274367	1981311	2283140	2600904	2395694	2319606	2569288	2445502	2168873	2139336	1911221	2843094
EIC	Negative	579.3498	4.56	unknown	70854233	74815512	75493077	68121586	71294431	70606290	71622502	75562506	70066548	71447422	69909403	72805292	70897750	72779941	73159119	66045357	68981393	69428324	71862863
EIC	Negative	582.2446	2.93	unknown	726929	460617	330191	594754	161648	354359	223938	637444	135431	1044120	342431	432046	437973	67268	104901	471423	178092	595029	122750
EIC	Negative	389.0306	0.48	unknown	2047544	2110654	2316733	2219660	1794790	1977383	2169724	2068047	2166057	2151478	2040870	2017255	2131611	966774	1952694	2007280	2024701	1942869	1976625
EIC	Negative	309.1748	2.12	unknown	0	225639	0	0	20401	16933	15698	27857	24636	0	32290	15788	194646	166236	28086	15197	30206	0	29217
EIC	Negative	239.1077	3.13	unknown	121909	114365	122666	131720	124318	118806	122708	110127	111457	101513	100832	106529	125892	115101	111901	129444	113329	103950	113236
fpHILIC	Negative	257.1761	0.93	unknown	1653192	551163	1617632	1463530	739042	640607	795649	1511963	472030	1924943	514574	688177	576692	465124	569603	579426	565263	1754137	580835
EIC	Negative	564.3310	4.57	unknown	967147	950193	1011942	921685	880519	1072410	895773	941675	971946	915578	933024	1022126	903380	1026225	896399	944820	914296	888485	993375
EIC	Negative	291.1602	2.99	unknown	133910	103814	109249	92968	90392	92827	103737	116143	76085	112462	103174	86295	87157	71045	81560	80919	101391	112358	90431
EIC	Negative	524.3003	3.27	unknown	423786	427253	376504	410734	329736	385585	379885	318289	326541	358585	359604	385259	335667	367589	361770	347147	374870	305831	305026
EIC	Negative	580.3524	4.56	unknown	15174236	15843885	16100852	14292655	15526125	14930413	15363928	15924743	14817761	14772327	14788786	15455263	14793763	15587624	15424793	13906831	15088528	14987884	15168639
fpHILIC	Negative	843.5604	0.43	unknown	7290261	6671893	6282438	5763890	6910277	5720509	5836579	5776738	7085921	5811407	5978361	6835612	6726473	6778751	6076467	5722304	6475745	5523493	6726790
EIC	Negative	362.1887	3.26	unknown	933707	739162	1138374	2510269	306062	700878	503645	235606	190161	879457	400338	261821	124110	70843	491348	724265	213479	289272	168716

EIC	Negative	270.0913	0.55	unknown	1304086	270691	409062	771961	210625	833226	432786	142352	214413	761890	362176	405229	253045	204102	280077	391529	211022	292199	613875
EIC	Negative	531.3066	4.84	unknown	790775	869882	811337	858198	775067	737051	808957	852029	828021	792807	803255	780535	800810	797976	808749	856414	758419	711661	768693
EIC	Negative	428.2649	4.05	unknown	201772	172340	189602	147501	149761	131325	170872	210726	164696	160156	178467	158310	201908	151916	182163	115046	187671	184184	138261
EIC	Negative	567.3506	4.94	unknown	328115	405724	330385	268466	333910	384802	393454	273023	188450	268162	387137	363909	315708	304590	371615	278340	300183	240795	344913
EIC	Negative	324.2560	4.99	unknown	359389	329570	293666	379664	326899	0	310370	327405	310179	328890	320895	320339	287721	265673	313761	0	323961	327222	279364
EIC	Negative	488.8957	3.47	unknown	94325	84057	69477	120973	79458	70176	99735	53963	74894	83958	85872	79048	68648	71395	62007	95969	79282	76358	55641
fpHILIC	Negative	304.0706	3.21	unknown	39120944	29145194	29107830	33610020	30432572	31159962	29311688	29121542	31096262	30721932	27480476	29490462	28939432	24656926	28869756	31699112	27787806	31723584	27460000
fpHILIC	Positive	140.0471	2.66	unknown	2110597	500654	353391	441425	339129	926380	115646	509511	1057045	282077	279338	977313	531736	620590	222372	719995	571153	876867	487816
EIC	Negative	471.0346	0.49	unknown	1769743	1902914	2041612	2062111	1583366	1759568	1872895	1922988	2002918	1956563	1885640	1815018	1990247	184027	1797235	1807559	1835875	1754087	1732646
EIC	Negative	605.3556	4.52	unknown	2546160	2358774	2215820	2202315	2217577	2409075	2340597	2375520	2192152	2222565	2302025	2337774	2293288	2247514	2354363	2167757	2367227	2074915	2301300
fpHILIC	Positive	84.0448	3.47	unknown	8691995	5132679	6116416	5537776	5530097	5775566	4965677	5782375	5507454	5666002	5401772	5947497	4696299	4643280	6360982	5270036	5009897	5914243	4362429
fpHILIC	Positive	140.0528	2.66	unknown	21131312	3460528	2651062	2982741	2941855	8233851	885585	3870604	9214042	2405900	2094005	7399680	4404511	5058377	1926418	5674790	4994776	7997674	3966459
EIC	Negative	298.2470	4.41	unknown	2376699	1864580	2171576	2206365	2153341	1999582	1989760	2253953	1919781	2330429	1886215	1921238	2001794	1872396	2020199	1917339	2009002	2286018	1853366
EIC	Negative	354.0044	1.19	unknown	78922	83401	73686	76660	69266	72005	68420	72093	64174	65831	67412	67077	64642	59869	69439	62417	66803	78268	61316
fpHILIC	Positive	762.5909	0.44	unknown	1623236	1880344	1651841	1747138	1789079	1432698	1600246	1725827	2146040	1560964	2023194	1942600	1633828	2190310	1784661	1501094	1951075	1406741	1856524
EIC	Negative	529.3531	2.92	unknown	258725	211821	531167	2219030	298156	198733	394216	361448	598048	685838	236265	478186	618400	122999	104324	555958	385222	600313	163873
EIC	Negative	365.1977	2.53	unknown	125611	89685	146000	92201	57083	88162	117946	66804	89809	93048	79570	125027	125000	69017	55772	103923	85021	82810	83049
fpHILIC	Positive	164.1164	2.92	unknown	4319605	4249476	4162037	2775118	2391258	3998863	3424267	2944918	1977717	3162447	3582388	3558120	2940783	2882563	3236020	2684691	2286770	2648244	3559587
EIC	Negative	336.7163	0.62	unknown	199806	115702	179589	129076	142328	119258	109187	73056	132010	216727	94739	71657	95312	195538	86418	108747	75738	109472	182920
EIC	Negative	261.1507	2.83	unknown	449182	223777	613070	622850	191246	303108	249329	438198	175946	478868	204236	232456	174747	114602	257590	232401	155757	494500	188145
fpHILIC	Positive	131.0499	3.48	unknown	717072	461110	639572	661900	411718	349132	597583	611130	422717	603124	554681	314813	546844	447103	664860	359580	569915	338338	408213
fpHILIC	Positive	133.0277	4.56	unknown	559963	480901	525339	617998	384370	800991	404107	438300	545601	682704	511255	515500	522698	408796	537977	550122	397459	536155	496918
fpHILIC	Negative	147.0185	4.03	unknown	626982	438933	521121	624720	386174	421503	463288	570454	337569	636248	469844	361446	305322	535746	575362	263294	314629	592235	388346
fpHILIC	Negative	325.1254	3.43	unknown	11156044	8638204	9722128	10735243	8073156	7438279	7876377	8992657	7857256	8916621	8414931	9073709	7917995	5756748	8006402	7945318	7748302	8915602	6792405
EIC	Negative	355.2130	3.32	unknown	1144937	224926	1100170	558313	278506	379469	431431	782543	110169	790505	198657	372048	191293	198574	245255	100221	221090	1110620	264422
fpHILIC	Positive	269.1069	1.79	unknown	0	66379	72444	678263	43795	48318	112117	389349	111308	75943	141758	75751	1682405	414073	32113	65545	466810	93859	32156
fpHILIC	Positive	759.5728	0.44	unknown	48908028	56493532	54776980	42440228	41302056	39121400	34176212	39664884	56129168	38882092	52076848	42221552	40172048	63294436	42139096	38566460	36248728	34789060	54420296
EIC	Negative	484.6909	0.52	unknown	221664	64982	262344	168721	285778	144219	130493	0	181808	299130	90994	28900	88717	382388	117903	146056	84629	158455	220445
EIC	Negative	289.1060	3.11	unknown	263729	88288	178524	320539	88303	162973	125852	203508	36552	216441	74431	142491	127387	46567	136313	77214	86855	219393	85229
EIC	Negative	317.2491	5.27	unknown	287044	216429	315672	267111	225424	66922	191090	240951	173589	361709	196427	165949	134801	106973	206937	166619	162586	236654	107596
fpHILIC	Negative	528.2943	0.61	unknown	834334	690635	731400	738472	722677	678302	689138	789204	560355	705952	709136	788018	588797	679243	931336	709689	548649	543279	639614
fpHILIC	Negative	820.5682	0.44	unknown	4450455	4432211	4218020	3057956	3834043	3072688	3455279	3414418	4836706	3468816	3798485	3527177	3685264	4585626	4300450	3353320	3608087	2921241	4076034
EIC	Negative	451.2654	4.27	unknown	868765	1207006	1084930	771775	965701	1247569	940852	802640	660261	615824	810754	867740	713271	1373921	848123	975086	693903	914079	1098384
fpHILIC	Negative	187.1087	2.91	unknown	1468776	1373621	1403085	1355828	1133263	1832202	1082842	829287	1233639	1420335	1314607	1281453	1041744	775008	1164090	1427581	1154411	1387788	862588
EIC	Negative	253.1446	2.27	unknown	7101838	4441075	5966420	9261316	4623431	4015793	3573307	5449246	3886824	7542055	2652676	3236451	4036645	2655756	3129991	4992036	3642228	6841741	2554514
fpHILIC	Negative	763.5598	0.48	unknown	644502	1133564	684964	691384	955096	683962	757358	474505	953438	578996	887119	685119	619644	661766	843941	824087	654363	546765	887931
EIC	Negative	280.1625	2.39	unknown	211638	94034	145034	111844	54657	189138	51640	133231	26849	150061	83936	100801	86190	22932	101357	51890	93534	111727	89049
EIC	Negative	342.7334	0.52	unknown	249506	63893	231834	125472	217194	135043	124496	22169	133234	381772	47138	27760	74119	931890	83324	106798	34201	104729	292224
EIC	Negative	396.1779	0.71	unknown	139738	184717	217957	95769	110402	136431	161944	121919	119214	172323	192252	167085	121797	162652	129476	114059	117212	106355	171784
EIC	Negative	402.6897	0.64	unknown	125154	54212	112429	86723	113852	76869	76006	46477	70722	201420	55353	38324	63664	314795	66535	54598	44727	78033	133538
EIC	Negative	639.4113	4.03	unknown	172744	0	207175	186558	0	0	0	214979	0	223550	0	0	0	0	0	0	0	211505	0
EIC	Negative	241.1808	3.98	unknown	604417	443488	719822	1373013	599141	492858	170941	787318	0	668907	134135	184388	646581	335431	321299	0	539994	809640	346208
EIC	Negative	342.2655	6.06	unknown	197295	144025	240637	270176	141983	135281	165533	160585	231372	166015	143438	168331	170648	134336	128105	202755	180581	204040	105815
EIC	Negative	616.2505	1.12	unknown	157479	81004	269670	187773	82589	51604	113037	141356	61582	135070	135003	113796	133411	40647	75214	89825	72217	169800	35919
EIC	Negative	546.2967	4.18	unknown	397455	316933	341408	360515	279474	345285	281323	324494	251352	361758	347499	282063	268278	263293	240627	277136	307425	330665	302467
fpHILIC	Positive	121.0394	2.66	unknown	4090791	585959	442094	523469	567580	1452889	167295	737743	1645216	510714	403740	1360175	823226	1005642	424213	1054264	930693	1411409	677478
EIC	Negative	438.2487	6.00	unknown	612375	1012401	557255	1451253	573533	956260	693627	749943	989475	1135572	1087317	466430	878818	941996	976667	463213	1170770	550265	739552
fpHILIC	Positive	391.1017	3.37	unknown	3387399	2852449	4964039	4521680	3873378	3146686	3354235	4403659	3185033	4986786	3849763	3354946	3553089	3056458	2942313	3090277	3661121	4604334	3387557
EIC	Negative	362.2373	4.38	unknown	125031	38558	0	0	0	0	57407	99686	0	0	0	0	104074	0	0	0	0	154722	0
fpHILIC	Positive	349.2297	5.39	unknown	1345913	1196815	521299	688837	775702	1276072	888225	541903	757652	404527	750890	815643	966601	848958	1628229	882014	467832	512419	686333
fpHILIC	Negative	265.1479	0.35	unknown	7961089	13577370	30419858	26596520	32983672	15914325	11881831	11229782	7927759	22475976	16426613	12035883	17027810	10630837	12352820	19645576	26557380	9615373	10597961
fpHILIC	Positive	249.1553	3.37	unknown	561340	477811	372612	330851	365788	396749	336180	312478	484359	431183	291194	427431	324424	563725	327772	570797	305216	394503	269899
EIC	Negative	291.0075	0.48	unknown	560997	561313	604283	586139	485293	527976	582398	576772	556584	603122	556158	593821	553455	291836	539204	518743	557592	541195	532946
EIC	Negative	608.3200	2.34	unknown	471569	430675	314927	264116	357849	442797	424852	416704	310211	398073	412420	544601	271703	490216	374720	325083	388585	283471	552781
fpHILIC	Positive	300.1995	0.61	unknown	3192884	883256	1458489	2066633	1294538	1423142	1583081	1619420	1033437	1282742	1220561	916102	1792263	751145	1191518	1513949	1386578	1952566	812189
fpHILIC	Positive	758.5691	0.44	unknown	104576520	114916128	113670808	88386736	86757800	82361664	72169848	82774000	114799272	83139208	106784016	90141856	82662936	132639560	89261856	79978808	75138608	74613120	113813920
EIC	Negative	532.3014	4.84	unknown	1085906	1133592	1091549	1168125	1014893	996433	1079282	1094467	1125659	1108952	1073555	1087222	1088985	1065385	1114911	1098208	999898	1001112	1023471
fpHILIC	Negative	245.0489	0.44	unknown	2770854	2548079	3651036	1389069	1953730	3058262	2048210	2240524	1189354	2653046	3288122	3825794	2165209	4170554	2245888	1764772	1896687	1922371	2516005
fpHILIC	Negative	145.0142	4.03	unknown	59810940	43588808	52578244	61224656	39871804	41059252	42980424	64671968	34603348	59642688	44004364	32206760	29467494	53501992	60509704	28468276	32830010	58310424	40003232
fpHILIC	Positive	518.3215	0.60	unknown	5269109	6605757	7677305	7635282	5688990	5737102	6671247	6069486	6230471	5447778	6766929	6924547	6556797	6519045	5660793	5990823	6321658	5833648	6181758
EIC	Negative	622.4055	6.22	unknown	907889	279720	828878	591814	434133	205205	335731	954532	241485	737756	296870	341636	297891	270142	297212	385954	287311	920750	250704
EIC	Negative	296.2313	4.43	unknown	1183659	775500	2084878	1164748	1039921	891247	1240756	1276731	1078676	1032215	722133	831131	716452	1695829	465104	1475940	1036605	1521936	954001
EIC	Negative	291.1222	3.89	unknown	152277	179581	274222	0	0	165883	0	283462	0	0	0	0	0	0	0	0	0	312197	131218
EIC	Negative	429.2861	3.25	unknown	631652	253679	271604	364229	123317	371427	193674	304099	425217	641655	256122	180106	160137	136897	412950	189035	458027	108712	230518
EIC	Negative	377.2702	5.91	unknown	790996	408759	834374	783459	470065	332429	483577	782102	409603	776608	480774	478386	384944	365217	492267	486666	354342	730134	382131
EIC	Negative	351.0909	0.55	unknown	213757	58874	74234	148080	60956	161246	95321	0	27835	112815	73037	77049	29904	23485	61970	88910	25526	64943	99528
EIC	Negative	356.3251	6.73	unknown	81478	146867	161001	297395	117410	113288	161885	131707	163459	115612	105440	217615	156503	100197	98197	129842	192911	171963	86000
EIC	Negative	274.1849	2.46	unknown	1163381	590306	649458	1538850	713480	831395	924274	1149665	811471	1069554	696928	703934	973732	248184	694120	836225	758156	1680835	406821
EIC	Negative	315.1968	5.28	unknown	317439	147170	296848	265866	109173	0	133969	259215	71774	296512	209419	123487	134458	113898	117202	96889	93969	166317	92824
EIC	Negative	266.1399	2.65	unknown	466934	113519	79331	313949	78945	301756	132570	192057	17534	281046	118623	193345	125147	0	247341	102384	187655	53674	123083
fpHILIC	Positive	386.2056	5.33	unknown	2920127	3120130	2264885	2284211	2492665	2984305	2552761	1873847	2439909	2080619	2622090	2846695	2964111	2840949	3301744	2610504	2058900	1897987	2330920
EIC	Negative	271.1918	3.65	unknown	1187135	256247	704280	502508	383557	542712	572160	897868	51872	598551	266116	507085	359912	391975	513399	227738	420519	987870	270351
EIC	Negative	253.1809	3.98	unknown	1349945	414146	2296022	2901003	299791	402744	351870	626197	301084	1514916	355256	2140183	484535	199466	282823	279295	226524	849190	321047
EIC	Negative	326.2720	4.46	unknown	3252866	2938147	3065991	3190092	3024588	2949290	2886102	3021847	2910973	3044211	2956304	3155136	3083177	2771445	2799381	3014898	2918555	3172143	2725388
EIC	Negative	581.3562	4.56	unknown	2313932	2389813	2576181	2330704	2249354	2431890	2376112	2477844	2305635	2350651	2449075	2492456	2345559	2405462	2502112	2222477	2192958	2398543	2395884
EIC	Negative	554.3473	4.84	unknown	142812413	179169719	144516868	144087094	151250754	148393848	154155632	141415081	166987604	138401384	162097884	160009275	158058200	162719131	160646440	157800537	148565802	125905701	161050157
EIC	Negative	601.4479	5.67	unknown	566535	146885	571427	413207	171694	168944	249612	264444	226174	274303	186029	155408	226737	226760	213618	116005	250779	258124	230726
fpHILIC	Negative	114.0310	2.97	unknown	942432	975666	723275	872537	610569	1015195	793836	373062	596603	623348	896077	817821	680144	201789	789598	965169	587841	401332	610069
EIC	Negative	227.1652	3.74	unknown	161152	119096	168520	277203	141847	142510	147048	156869	108470	162568	110897	162538	155944	73517	100384	155499	140861	213787	79001

fpHILIC	Positive	754.5356	0.45	unknown	389006	450870	338373	555204	296858	442679	488754	380422	253711	367909	396931	507810	489987	357997	408221	445659	419657	264196	305629
fpHILIC	Positive	757.5548	0.44	unknown	2188609	3204867	2617564	2673430	2268996	2463656	2761874	2142992	2548313	1823714	3043772	2693605	2642291	2887643	2420854	2591634	2631639	1940775	2481255
fpHILIC	Positive	308.0910	4.81	unknown	1267750	183896	815134	923619	243092	268159	175056	784271	210259	1076490	198570	251876	185955	247620	203108	266826	210432	734076	249960
fpHILIC	Positive	783.5720	0.43	unknown	38353336	42619752	38150644	36619324	34711652	33258744	33589092	33093590	42237924	31834106	37266668	37687144	35466716	41819272	32539736	34010276	38410740	30606394	39619004
EIC	Negative	324.7629	0.63	unknown	342205	261575	613739	438545	287643	298088	267876	212940	429915	766023	260963	243384	298768	137739	136045	330314	154074	330322	531503
EIC	Negative	361.2389	4.38	unknown	507418	361361	356286	948583	459682	358065	331177	404440	356993	584595	324875	333959	455753	129611	252224	379661	360799	659436	241314
EIC	Negative	614.9564	0.55	unknown	283826	164874	157823	393364	109179	324866	203735	95153	114554	132617	219636	237971	181888	0	168240	255885	139164	144689	132045
fpHILIC	Negative	378.1187	2.03	unknown	2695082	1383988	1669746	2796470	1308619	834807	1940927	2492276	3374672	2278051	1686600	1550645	2720180	3365031	2435274	1309798	2542273	2230011	1347208
EIC	Negative	326.2720	4.98	unknown	4370311	4140923	4136858	4393763	4325612	4063559	4045228	4237585	4030557	4236231	4059342	4064471	3929826	3836354	4129178	4289687	4063449	4127787	3913360
EIC	Negative	299.1879	2.87	unknown	159674	113320	163312	95360	67573	114207	101480	118422	50850	105861	73231	106898	94475	69012	91302	85012	91183	122591	58543
EIC	Negative	598.3730	3.56	unknown	220492	157994	225411	121333	115753	229629	136377	156084	123075	196699	142146	228383	146240	139448	157660	70688	177614	119749	228664
EIC	Negative	539.3253	3.73	unknown	679015	87854	1886343	1030739	114413	104322	67360	1325586	127893	972887	116220	128005	89378	170296	176591	101014	120843	1205914	120593
fpHILIC	Positive	854.5688	0.43	unknown	779488	2549293	1185048	1099487	1267479	1661187	1948884	1108987	889391	670083	1589139	1553163	1640426	1484208	1134893	1476522	1716756	814574	1675433
EIC	Negative	432.8069	0.52	unknown	934043	652328	1128859	993390	663161	772173	600715	595778	875432	996465	634962	603787	697668	134729	607775	815930	540235	736768	777739
EIC	Negative	391.2190	2.15	unknown	1603187	452833	1788456	693566	354329	882487	416714	722597	356341	437375	704556	416764	690209	586851	713400	366351	913754	361181	355379
EIC	Negative	283.1921	3.11	unknown	833956	493311	722518	777728	540765	544743	496326	675669	660193	674674	612699	623081	580471	545594	500883	616686	539227	769087	468622
fpHILIC	Negative	79.9572	3.22	unknown	1726070	1457648	1263707	1603041	1363749	1633274	1342433	1368065	1356275	1358122	1094292	1324793	1363417	1069010	1409829	1441564	1350425	1485854	1320003
fpHILIC	Negative	313.1132	3.46	unknown	2593591	1451597	1849839	2116594	1728290	2097405	1748028	1955707	1384803	2203727	1846022	1997126	1734705	1397421	2170431	1893812	1588521	2318256	873482
fpHILIC	Negative	98.9644	4.56	unknown	705435	580135	614160	639836	443013	606613	439881	518735	666266	624233	551225	545585	524011	450383	518989	673531	446543	492742	522603
fpHILIC	Positive	139.0498	2.66	unknown	327164608	56327996	39920860	48449816	47889216	132200992	13392461	64018216	139777120	39628040	31897404	116170600	66793448	87238664	30747186	89726040	83899752	125996568	63955332
fpHILIC	Positive	192.0648	2.38	unknown	529585	354022	408996	305357	340458	388255	322406	123853	242150	272612	526464	353034	422337	390917	354145	296284	228079	213949	314417
EIC	Negative	344.2314	4.19	unknown	427402	438429	409887	1203459	426132	403415	383742	492364	648551	641101	423443	447469	590828	221537	301494	616212	462909	628241	328586
EIC	Negative	540.3313	4.22	unknown	2910656	3489056	2659927	2624653	2568369	3089512	2561844	2397409	2378645	2366676	2840390	2811667	2664789	2986406	2737423	2898470	2376512	2211985	2678917
EIC	Negative	318.0083	1.09	unknown	125991	76240	71950	43422	46962	58433	42386	58186	38967	55331	40993	41773	36859	63657	60153	37430	41499	57473	46663
EIC	Negative	532.9536	0.55	unknown	399636	181486	241730	515234	125698	424598	247157	113425	144293	201146	270491	247527	221232	18863	198842	299607	137382	216310	195902
fpHILIC	Negative	101.0244	4.03	unknown	14811378	10358392	12806987	15297267	10000989	10305080	10709816	15920651	9080329	14790865	11148243	8467347	7516982	13188776	14808677	7573210	8497445	14559949	10199276
fpHILIC	Negative	174.1077	5.40	unknown	537765	478467	315931	305591	349471	481643	428805	305933	345364	234417	333194	350294	373581	359308	598646	426026	254302	292639	326865
EIC	Negative	275.1269	3.33	unknown	938543	673223	707644	728324	615894	608172	616337	605703	323516	585219	646502	650777	640037	323266	570583	624536	442252	711444	403082
EIC	Negative	266.1841	2.99	unknown	85211	49524	120439	146537	62210	72546	75503	84595	45259	90678	57284	105691	83907	57814	57299	80038	48546	110720	41495
EIC	Negative	562.8989	0.52	unknown	896737	902247	942486	1052788	645690	662726	816282	1163660	915970	488615	943375	1025616	964511	89485	618752	820578	969903	868135	904248
EIC	Negative	552.0356	0.48	unknown	6177334	6752251	7582093	7639255	5530764	6305435	6706789	6964853	7012916	7130745	6816738	6626172	7077701	1995145	6057085	6703712	6646170	6701012	6239280
EIC	Negative	316.2572	3.43	unknown	2625960	2727662	2513702	2255304	2535675	2620587	2533345	2524918	2294803	2246332	2499738	2468950	2484032	2523057	2674585	2416954	2478851	2285993	2586884
fpHILIC	Negative	570.3399	0.62	unknown	2368844	3382528	2860888	2260854	2628805	3071762	2785586	2344852	3289830	2371155	2783035	2849110	3132262	2695608	3286302	2973005	2534508	1983846	3270239
EIC	Negative	575.4344	3.70	unknown	372481	0	579227	83032	0	12581	12566	85541	0	0	0	0	0	0	0	0	0	0	0
EIC	Negative	320.2260	3.35	unknown	379415	12027	455800	116592	26211	17603	21433	22784	27131	0	8647	0	115179	5783	0	0	0	0	0
fpHILIC	Negative	405.2651	0.52	unknown	10022685	1184940	21228290	12954128	547385	792173	1864592	1996047	446677	180555	0	0	0	0	0	0	0	0	0
EIC	Negative	390.2734	3.74	unknown	186109	0	157328	80390	0	0	0	61890	0	0	0	0	0	0	0	0	0	0	0
EIC	Negative	424.2718	2.47	unknown	249352	10460	429341	0	0	0	73737	0	28192	0	0	0	0	0	0	0	0	0	0
EIC	Negative	573.3814	2.45	unknown	995895	11731	682779	246282	42136	0	11280	245833	7044	28709	0	0	0	0	0	0	0	0	0
EIC	Negative	279.1638	2.75	unknown	0	274510	0	396237	108959	0	0	0	0	0	0	0	0	272885	0	0	0	0	0
EIC	Negative	324.2560	6.38	unknown	297378	0	428922	754825	0	0	317955	0	0	0	0	0	0	0	0	0	0	0	0
EIC	Negative	523.2004	2.48	unknown	503538	99390	779672	601902	46415	8659	146452	143711	30137	0	0	0	0	0	0	0	0	0	0
EIC	Negative	442.2448	2.48	unknown	1610821	269008	2614084	1989579	106583	179279	444556	395076	100840	0	0	0	0	0	0	0	0	0	0
EIC	Negative	471.2731	3.00	unknown	309266	0	613215	233658	16453	0	21271	177852	0	0	0	0	0	0	0	0	0	0	0
EIC	Negative	631.4564	3.85	unknown	382644	0	337863	330523	59261	0	0	150979	0	79195	0	0	0	0	0	0	0	0	0
EIC	Negative	443.2398	2.48	unknown	2145477	383828	3543656	2659737	156675	246356	622581	586238	125328	0	0	0	0	0	0	0	0	0	0
EIC	Negative	465.2863	2.36	unknown	20083185	0	29003882	6987824	0	955489	0	9971682	0	0	0	0	0	0	0	0	0	0	0
EIC	Negative	444.2432	2.48	unknown	510533	81663	878279	708616	41926	80015	164604	145930	25932	0	0	0	0	0	0	0	0	0	0
EIC	Negative	454.2848	1.83	unknown	0	0	1341106	0	0	0	0	0	531987	0	0	0	0	0	0	0	0	0	0
EIC	Negative	387.2540	3.31	unknown	639013	0	631140	96853	46375	20054	67473	162001	0	0	0	0	0	0	0	0	0	0	0
EIC	Negative	450.2941	3.00	unknown	1074435	41859	2537482	764603	97588	96526	109926	600320	36817	55851	0	0	0	0	0	0	0	0	0
EIC	Negative	466.2894	2.48	unknown	41371489	8693031	61757083	49607502	3466243	5780126	13247917	11446254	2763146	0	0	0	0	0	0	0	0	0	0
EIC	Negative	465.2861	2.48	unknown	144107535	29878368	213157759	174236957	11939784	19816171	46816419	39749418	9612103	0	0	0	0	0	0	0	0	0	0
EIC	Negative	390.2733	3.00	unknown	417936	31583	949791	357612	44404	54421	56638	239210	0	12318	0	0	0	0	0	0	0	0	0
EIC	Negative	406.2684	2.43	unknown	769785	37773	790732	390186	31330	29327	25633	353198	22781	60150	0	0	0	0	0	0	0	0	0
EIC	Negative	317.2027	5.32	unknown	0	0	0	431476	0	147843	0	0	0	0	131391	142959	0	0	0	0	0	0	0
EIC	Negative	423.2762	2.48	unknown	957522	203550	1477722	1198697	103172	126339	320111	278362	90416	0	0	0	0	0	0	0	0	0	0
EIC	Negative	389.2700	3.74	unknown	653132	0	625682	253101	35130	36253	44901	249272	0	0	0	0	0	0	0	0	0	0	0
EIC	Negative	427.2625	3.69	unknown	2381756	131763	5047015	575363	303449	175352	330385	1004713	157628	90123	0	0	0	0	0	0	0	0	0
EIC	Negative	339.2654	6.34	unknown	0	0	2187856	1126913	977211	0	0	0	0	1081636	0	0	539826	0	0	0	0	0	0
EIC	Negative	504.2428	2.48	unknown	729315	119641	1281994	1133506	48587	109664	275096	310450	71648	19662	0	0	0	0	0	0	0	0	0
EIC	Negative	487.2723	3.05	unknown	846724	111882	1152764	554090	112466	67206	136571	346031	25857	319408	0	0	0	0	0	0	0	0	0
EIC	Negative	317.2027	5.26	unknown	0	0	0	280057	0	128263	0	0	0	0	139709	175348	0	0	0	0	0	0	0
EIC	Negative	387.2538	3.08	unknown	655206	50890	578264	169507	11766	83893	111080	143810	28933	0	0	0	0	0	0	0	0	0	0
EIC	Negative	390.2738	3.54	unknown	1951483	113993	2036598	461391	91794	150805	225822	678833	42536	39315	0	0	0	0	0	0	0	0	0
EIC	Negative	485.2589	3.54	unknown	311811	26339	340980	82878	26140	28997	29717	118634	0	0	0	0	0	0	0	0	0	0	0
EIC	Negative	611.4249	2.19	unknown	18593232	0	24621454	1124351	29646	0	0	11546173	0	0	0	0	0	0	0	0	0	0	0
EIC	Negative	449.2901	3.55	unknown	2473371	63591	3252516	604524	368872	61784	307399	901013	77069	72915	0	0	0	0	0	0	0	0	0
EIC	Negative	611.9265	2.19	unknown	14466105	0	19435045	878984	0	0	0	9353613	11984	0	0	0	0	0	0	0	0	0	0
EIC	Negative	488.2762	3.05	unknown	308108	23887	326174	166685	40427	0	49432	123629	26167	92054	0	0	0	0	0	0	0	0	0
EIC	Negative	612.4278	2.19	unknown	6434823	0	8681503	364448	0	0	0	4259369	0	0	0	0	0	0	0	0	0	0	0
EIC	Negative	450.2941	3.55	unknown	737226	33252	937756	189585	112784	47012	85160	249231	38245	0	0	0	0	0	0	0	0	0	0
EIC	Negative	612.9293	2.20	unknown	2010643	0	2634652	147116	0	0	0	1341388	0	0	0	0	0	0	0	0	0	0	0
EIC	Negative	427.2461	3.61	unknown	288598	0	274510	104013	50991	0	38846	100270	0	44068	0	0	0	0	0	0	0	0	0
EIC	Negative	425.2469	3.20	unknown	126380	0	189309	87811	0	0	41962	72683	7112	12225	0	0	0	0	0	0	0	0	0
EIC	Negative	389.2700	3.45	unknown	1550592	34461	1409767	281391	188465	137805	293491	332528	17564	0	0	0	0	0	0	0	0	0	0
EIC	Negative	471.2722	3.55	unknown	798758	55840	854125	200655	60450	73115	107557	299160	27435	40513	0	0	0	0	0	0	0	0	0
EIC	Negative	472.2747	3.54	unknown	213709	10811	256805	56226	30022	25222	34832	74717	0	0	0	0	0	0	0	0	0	0	0
EIC	Negative	634.4777	4.07	unknown	155039	0	183209	286287	22404	14815	29784	124545	0	39635	0	0	0	0	0	0	0	0	0

EIC	Negative	473.2879	3.05	unknown	1710368	266438	2063756	1215126	280188	189001	344407	734469	125798	717030	0	0	0	0	0	0	0	0	0
EIC	Negative	441.2423	2.57	unknown	443765	37837	562382	86878	18094	26429	37272	242683	29849	0	11372	0	0	0	0	0	0	0	0
EIC	Negative	471.2724	3.20	unknown	421150	29475	509998	263596	45235	0	95081	222016	26980	73735	0	0	0	0	0	0	0	0	0
EIC	Negative	393.2922	3.68	unknown	926837	25440	1534767	241915	125865	83619	143547	428811	59275	0	0	0	0	0	0	0	0	0	0
EIC	Negative	421.2972	3.19	unknown	362546	21404	597474	0	53957	55705	26948	166919	19337	0	0	0	0	0	0	0	0	0	0
EIC	Negative	611.4251	2.13	unknown	21825293	1550829	17049790	4072317	1542391	1955176	2806733	7532083	734795	0	0	0	0	0	0	0	0	0	0
EIC	Negative	611.9264	2.14	unknown	17378682	2189863	14239002	3224536	1948806	1674866	2247638	6227988	506199	0	0	0	0	0	0	0	0	0	0
EIC	Negative	392.2890	2.57	unknown	2254333	179572	1611107	471887	235125	213750	399018	557857	127621	55846	0	0	0	0	0	0	0	0	0
EIC	Negative	613.4324	2.30	unknown	844191	0	932709	237014	77260	0	0	600108	0	100915	0	0	0	0	0	0	0	0	0
EIC	Negative	406.2683	2.23	unknown	3757538	64004	4398889	1073779	129240	139840	54699	2794426	23221	38564	0	0	0	0	0	0	0	0	0
EIC	Negative	453.3134	2.56	unknown	1307774	119071	915240	321572	143545	127833	246367	319599	99059	0	0	0	0	0	0	0	0	0	0
EIC	Negative	612.9294	2.30	unknown	3978191	65024	4035765	1332703	319888	125035	75814	2671715	22200	207204	0	0	0	0	0	0	0	0	0
EIC	Negative	612.4278	2.14	unknown	7543497	808518	6181535	1558967	929133	615839	1405686	2573482	162600	0	0	0	0	0	0	0	0	0	0
EIC	Negative	474.2916	3.05	unknown	466328	65881	572228	319978	93790	61041	106839	266585	23777	222969	0	0	0	0	0	0	0	0	0
EIC	Negative	633.4761	4.08	unknown	361314	18840	425921	658184	75252	51978	128288	285136	25003	134064	0	0	0	0	0	0	0	0	0
EIC	Negative	443.2570	2.19	unknown	5522541	224292	6939967	1171716	410503	269090	235634	3970906	279773	281546	0	0	0	0	0	0	0	0	0
EIC	Negative	426.2941	2.19	unknown	185999	0	242737	63723	12795	11167	0	144959	13214	0	0	0	0	0	0	0	0	0	0
EIC	Negative	445.2549	2.19	unknown	1802478	83580	2484084	402855	150196	95734	106188	1330508	119711	102195	0	0	0	0	0	0	0	0	0
EIC	Negative	472.2748	3.61	unknown	452719	18704	375154	182313	86925	43953	81532	189558	0	61950	0	0	0	0	0	0	0	0	0
EIC	Negative	612.9296	2.13	unknown	2231505	269721	1789329	644271	233940	373040	437491	843740	77392	0	0	0	0	0	0	0	0	0	0
EIC	Negative	389.2691	2.84	unknown	1451124	148825	1432831	935270	249183	282060	288839	618726	161851	136669	0	0	0	0	0	0	0	0	0
EIC	Negative	444.2611	2.19	unknown	1430619	20748	1762675	286743	155449	94533	58121	987565	108145	96519	0	0	9847	0	0	0	0	0	0
EIC	Negative	409.2867	2.19	unknown	1300490	33206	1394493	108612	57492	57387	52851	983080	0	0	0	0	0	0	0	0	0	0	0
EIC	Negative	331.1583	2.78	unknown	686900	0	527818	0	0	0	0	500270	0	0	0	0	0	0	0	0	0	0	0
EIC	Negative	448.3071	4.05	unknown	0	0	762253	282114	0	0	0	433907	0	0	0	0	0	0	0	0	0	0	0
EIC	Negative	488.2769	3.68	unknown	765672	62010	936421	247327	175367	72404	154976	419067	56936	33096	0	0	0	0	0	0	0	0	0
EIC	Negative	504.3133	5.88	unknown	2228029	2351645	2337249	1728641	1969512	2925137	0	0	0	1111713	0	0	0	0	0	0	0	0	0
EIC	Negative	450.2932	3.45	unknown	160476	0	313383	40578	46191	0	81936	94164	0	0	0	0	0	0	0	0	0	0	0
EIC	Negative	387.2541	3.22	unknown	387623	7417	266582	109231	27625	39557	42231	209673	18881	0	0	0	0	0	0	0	0	0	0
EIC	Negative	408.2838	2.20	unknown	7604309	56781	8822016	2199888	698075	492680	398336	6265971	493992	378811	0	0	0	0	0	0	0	0	0
EIC	Negative	466.2894	5.91	unknown	0	2395621	1791914	0	0	2768718	0	0	0	0	0	0	0	0	0	0	0	0	0
EIC	Negative	307.1948	3.87	unknown	0	0	1920336	0	0	0	0	0	859896	2742191	0	950135	0	0	0	0	0	0	0
EIC	Negative	407.2807	2.19	unknown	28800771	144557	32273057	8525553	2649277	1935947	1791816	23463170	1732555	2060805	0	0	0	0	0	0	0	0	0
EIC	Negative	478.3178	1.83	unknown	781409	35687	1329825	0	98946	49022	29637	731612	71454	0	7153	0	16934	0	0	0	0	0	0
EIC	Negative	449.2898	3.45	unknown	622827	59461	1139227	175996	272618	0	289132	310241	59908	57515	0	0	0	0	0	0	0	0	0
EIC	Negative	425.2915	2.19	unknown	654499	40320	763031	173822	77352	54034	26201	547564	51179	40374	0	0	0	0	0	0	0	0	0
EIC	Negative	556.3322	3.60	unknown	0	198900	191452	0	0	0	0	171770	9119	227436	0	0	0	0	0	0	0	0	0
EIC	Negative	445.2542	2.75	unknown	5349053	0	5989949	2094888	564994	622286	327619	4306179	642295	0	0	0	75517	0	0	0	0	0	0
EIC	Negative	410.2892	2.75	unknown	902670	87672	1055663	543921	149144	172870	0	720032	235419	0	0	0	0	0	0	0	0	0	0
EIC	Negative	406.2681	2.56	unknown	1969255	82576	2382130	303586	216583	191088	125509	1536607	206356	0	0	0	14454	0	0	0	0	0	0
EIC	Negative	444.2607	2.75	unknown	3936944	273722	4321161	1675252	422227	562773	279046	3237886	505947	104097	0	0	69049	0	0	0	0	0	0
EIC	Negative	405.2646	2.56	unknown	7197007	347571	8905895	1088866	802715	756977	448675	5874073	616119	0	0	0	20622	0	0	0	0	0	0
EIC	Negative	538.3171	2.60	unknown	90777	0	0	0	78953	51716	0	0	0	0	0	0	0	0	0	0	0	0	0
EIC	Negative	393.2921	2.84	unknown	642279	98920	336232	246143	68698	130058	158307	226161	80189	0	0	0	0	0	0	0	0	0	0
EIC	Negative	429.2602	2.39	unknown	0	359281	0	756491	546500	0	0	0	0	0	0	0	0	0	0	0	0	0	0
EIC	Negative	622.4170	2.19	unknown	494729	0	331814	0	0	0	0	411907	0	0	0	0	0	0	0	0	0	0	0
EIC	Negative	628.3627	4.43	unknown	0	0	0	12262665	0	13402682	0	0	0	0	0	13414769	0	0	0	0	0	0	0
EIC	Negative	561.3108	5.32	unknown	0	41537	232828	179395	0	0	38511	190739	0	0	0	0	0	0	0	0	0	0	0
EIC	Negative	392.2891	2.84	unknown	3894326	499785	2652503	1501271	394457	904534	993017	1907807	430535	170587	0	0	26589	0	0	0	0	0	0
EIC	Negative	453.2861	2.19	unknown	412377	26640	503439	167562	75762	64037	40541	370788	35991	27593	0	0	0	0	0	0	0	0	0
EIC	Negative	583.3138	4.00	unknown	0	196668	345413	765223	0	0	350428	0	348073	0	0	0	0	0	0	0	0	0	0
EIC	Negative	634.4769	3.86	unknown	205987	0	252429	822866	136128	32790	124770	319674	104826	158439	0	0	0	0	0	0	0	0	0
EIC	Negative	421.2602	1.77	unknown	217745	0	223116	24992	6483	0	0	243172	14948	0	0	0	0	0	0	0	0	0	0
EIC	Negative	449.3096	2.09	unknown	265587	24902	605416	272410	13986	11954	28399	599883	44052	0	0	0	0	0	0	0	0	0	0
EIC	Negative	628.3627	4.26	unknown	0	0	0	8275126	0	11180429	0	0	0	0	0	10101656	0	0	0	0	0	0	0
EIC	Negative	553.3364	2.03	unknown	1976068	423476	1150249	713772	310642	584884	798129	1089559	163234	0	0	0	0	0	0	0	0	0	0
fpHILIC	Negative	227.9981	0.52	unknown	1452892	508659	1368080	1189819	482872	976141	632369	929108	282287	0	0	0	0	0	0	0	0	0	0
EIC	Negative	362.1977	5.19	unknown	288123	0	293277	0	0	0	365122	0	0	0	0	0	0	0	0	0	0	0	0
EIC	Negative	279.1865	3.55	unknown	2571821	0	123923	7398345	98057	2448165	0	4714530	0	71552	2251590	6707509	0	0	0	0	0	0	0
EIC	Negative	319.2219	4.76	unknown	0	10610779	0	0	7382805	0	6588661	0	0	5246964	0	0	0	0	0	0	0	0	0
fpHILIC	Negative	324.0720	2.93	unknown	1278229	609442	1189364	592710	549518	739649	639502	644926	311918	0	0	0	0	0	0	0	0	0	0
EIC	Negative	478.2943	4.68	unknown	0	9782468	9976820	0	7447684	0	0	0	5601507	0	0	0	0	0	0	0	0	0	0
EIC	Negative	535.1739	3.85	unknown	209128	19188	186788	156513	54198	32915	81783	187439	21473	82053	0	0	0	0	0	0	0	0	0
EIC	Negative	427.2623	2.84	unknown	826651	149359	470971	0	98275	263189	243326	337279	86343	0	0	0	0	0	0	0	0	0	0
EIC	Negative	381.1716	3.65	unknown	705108	0	645219	802877	677680	0	0	784068	0	0	0	0	0	0	0	0	0	0	0
EIC	Negative	393.2651	6.66	unknown	0	606044	862482	0	0	0	0	0	1564032	0	0	0	0	0	0	0	0	0	0
EIC	Negative	410.2907	2.14	unknown	314665	115753	217367	93284	109172	90300	122319	192709	75263	0	0	0	0	0	0	0	0	0	0
EIC	Negative	570.3413	1.67	unknown	249321	1216484	4205550	0	1290204	69495	2141413	0	579967	320382	26500	0	0	0	0	0	0	0	0
EIC	Negative	506.2598	2.75	unknown	1082125	122106	1496161	847903	333468	318607	199786	1614845	346158	29639	0	0	26108	0	0	0	0	0	0
EIC	Negative	420.2407	2.74	unknown	0	22511	0	1171377	0	0	0	901064	0	99261	1056062	1157289	0	0	0	0	0	0	0
EIC	Negative	280.1889	3.58	unknown	262650	0	205557	0	0	162144	117531	182154	0	168492	0	161643	0	0	0	0	0	0	0
EIC	Negative	321.1688	5.79	unknown	0	0	0	2746616	0	0	1813505	1618152	0	0	0	0	0	0	0	0	0	0	0
EIC	Negative	317.2027	4.89	unknown	0	0	0	0	0	1581928	0	0	0	0	0	4264571	0	0	0	0	0	0	0
fpHILIC	Negative	495.1190	2.74	unknown	797924	271871	430451	333096	360045	433925	467853	378165	274613	0	0	0	0	0	0	0	0	0	0
EIC	Negative	478.3178	2.28	unknown	483471	0	572494	341579	55253	51667	17044	1083041	49917	86384	0	0	0	0	0	0	0	0	0
EIC	Negative	464.3022	1.52	unknown	2445022	472981	3180528	303742	535230	600194	368097	4180102	557468	0	0	0	38703	0	0	0	0	0	0
EIC	Negative	464.3022	3.11	unknown	0	0	231795	175375	0	0	0	373920	0	0	0	0	0	0	0	0	0	0	0
EIC	Negative	474.2926	2.84	unknown	366157	145741	269386	112335	122639	226855	276316	230035	124925	31786	0	0	0	0	0	0	0	0	0
fpHILIC	Negative	423.0908	0.97	unknown	741392	587326	517600	266029	625609	704732	491878	430139	417452	0	0	0	0	0	0	0	0	0	0

EIC	Negative	452.3107	2.57	unknown	7323037	614585	5194416	1710272	774139	730387	1341688	1833731	397724	139921	0	0	24947	0	13130	0	0	0	0
EIC	Negative	473.2881	2.84	unknown	1501401	529307	1148540	809054	446839	944235	867334	980082	399614	0	0	0	0	0	0	0	0	0	11861
fpHILIC	Positive	350.1368	2.66	unknown	1928109	350497	276624	324452	325732	772579	74457	468832	977478	277540	187516	641610	467506	0	102599	577545	653517	879642	392890
EIC	Negative	287.1077	3.54	unknown	231832	252920	239858	245869	224069	242691	248512	230210	218700	241245	236653	233987	249306	220063	233837	223365	219554	249588	232429
EIC	Negative	289.1236	3.59	unknown	458807	422400	396408	430672	384558	427321	419821	408507	380299	396276	398400	395067	408050	365960	395144	417067	394376	373819	430788
fpHILIC	Negative	157.0869	0.40	unknown	2177473	1576344	2945793	2159084	1519634	2400816	1945650	1671270	2139405	2747442	1430857	2171726	2537194	1509349	1557743	2242818	1721933	2836118	1274372
EIC	Negative	254.1761	3.37	unknown	1445566	473680	757957	2372758	296273	953643	407926	650868	682328	1050864	630352	950218	677889	226471	398951	825193	511036	904845	337563
fpHILIC	Negative	245.0430	3.06	unknown	1395337	804292	660967	959999	898925	1065991	1104515	822159	661136	844086	840196	1138825	642539	682571	994547	961252	792428	984796	799759
EIC	Negative	466.1816	0.70	unknown	116913	111947	144862	65375	92542	109015	123764	82260	96100	128926	114411	109940	91333	134647	92794	90176	95187	66559	156833
EIC	Negative	320.2674	5.88	unknown	205221	121435	211985	200245	127614	74269	110950	187089	109939	256196	135634	102079	109938	100644	129193	98089	112094	176501	91476
fpHILIC	Positive	772.5830	0.43	unknown	1481241	1501744	1494854	1274140	1159636	1483145	1082996	1623623	1119049	1156555	1297547	1085264	1081700	1707108	1269876	1069803	1218248	1355940	1528395
EIC	Negative	270.1713	2.44	unknown	8343937	4073950	6041642	6422950	5259518	5567375	4956798	6042432	5899286	7440735	6381234	5767152	5061348	5081903	5429269	4957915	5856595	7190057	4179802
EIC	Negative	328.2820	4.97	unknown	1746609	1790522	1648696	1811969	1769195	1610245	1663982	1741205	1609629	1648811	1663665	1660789	1617385	1581631	1654655	1705488	1708799	1733888	1559073
fpHILIC	Negative	868.5672	0.43	unknown	4692850	4567322	3240507	3678244	4195975	4556507	4252354	3712111	3099361	3201777	3732274	4381594	4359137	3796184	4194804	3960751	4615525	3248379	3722242
EIC	Negative	634.0388	0.48	unknown	5303502	5682192	6324528	6241299	4518598	5207927	5512413	5641357	5874999	5671606	5539480	5382598	5852927	1313956	4918113	5707710	5502145	5497021	5033236
fpHILIC	Negative	386.2373	0.36	unknown	818203	582274	1048942	1740613	500439	695426	483579	689969	692267	715639	514188	657330	554785	312441	446866	604048	566353	1090267	343211
EIC	Negative	312.2542	6.29	unknown	696897	375083	844610	2185234	526931	419027	513254	659903	756659	592218	433559	635762	700370	315646	331628	855393	574556	776366	328450
fpHILIC	Negative	581.3289	0.59	unknown	18144482	20222258	20950244	15457464	14702417	20562982	17475858	16837634	14503083	15450852	19423570	19630196	16665942	21438138	15513204	14834660	16116686	15253073	22098720
EIC	Negative	295.1194	2.22	unknown	152255	96732	165635	199827	85345	78534	95423	98800	81455	201934	100758	108775	85086	55885	108144	81231	59652	120279	68876
EIC	Negative	557.3569	4.84	unknown	1311654	1603385	1382475	1266273	1347625	1257292	1432368	1291819	1479848	1196370	1358135	1492646	1410446	1420673	1436838	1390575	1388334	1107260	1465575
EIC	Negative	339.0966	0.85	unknown	112313	102798	159667	150407	94224	67858	118685	127224	63996	138836	100242	124867	108802	45927	73238	88960	87816	172483	48114
EIC	Negative	295.1919	3.52	unknown	1301278	393871	985338	544825	534975	619055	716437	888954	217396	683837	353166	636446	387736	380156	509914	328949	422459	1358180	348893
fpHILIC	Positive	513.3373	0.61	unknown	1215698	651182	1177969	649705	610787	801237	734221	913318	603316	965432	642176	716159	322580	715138	817044	952009	742574	826378	314719
fpHILIC	Negative	266.1513	0.35	unknown	994435	1580098	4212425	3656420	4233657	2077413	1598028	1420947	1073395	2549510	2199083	1797418	2271352	1338707	1696131	2766095	3205155	1161454	1553690
fpHILIC	Negative	309.2800	0.37	unknown	7106327	10670731	16012353	15284279	11529938	9185068	11277193	10033638	9095214	8774260	7210107	14142572	10404389	6011099	7582101	11684591	8490179	15055205	8209276
fpHILIC	Positive	280.1034	2.97	unknown	527652	349912	538349	327271	289556	318162	405111	263534	256740	409656	480663	221938	391657	199686	551580	55224	428540	169728	326351
EIC	Negative	570.3418	2.42	unknown	5232662	3846191	4067529	4656260	3198353	4033240	3395653	4269972	3161356	4234625	3959994	4005705	3355531	2955193	3335305	3308693	3308772	4484962	3601258
EIC	Negative	382.8119	0.65	unknown	167165	70370	83598	163977	46368	114982	65759	47088	50237	94112	76992	74320	62347	18361	55345	83409	40395	63967	81213
EIC	Negative	338.7139	0.62	unknown	180592	101587	168817	122343	127660	108454	99896	67576	117756	210679	84738	73705	87440	179665	79673	100288	70315	96326	174581
fpHILIC	Negative	146.0177	4.03	unknown	3344886	2393991	2915533	3462054	2239399	2179034	2288745	3456676	1855313	3281470	2419137	1779296	1598735	3007892	3265079	1560865	1855355	3277502	2059926

Supplemental Table 2

		OLZ vs CON								MO vs OLZ								MINO vs CON								
ID	m/z	statistic_OvsC	dm_OvsC	p.value_OvsC	adj_pval_OvsC	fold_change_imputed_OvsC	log2_fold_change_imputed_OvsC	fold_change_imputed_scaled_OvsC	log2_fold_change_imputed_scaled_OvsC	statistic_MOvsO	dm_MOvsO	p.value_MOvsO	adj_pval_MOvsO	fold_change_imputed_MOvsO	log2_fold_change_imputed_MOvsO	fold_change_imputed_scaled_MOvsO	log2_fold_change_imputed_scaled_MOvsO	statistic_MvsC	dm_MvsC	p.value_MvsC	adj_pval_MvsC	fold_change_imputed_MvsC	log2_fold_change_imputed_MvsC	fold_change_imputed_scaled_MvsC	log2_fold_change_imputed_scaled_MvsC	statOvsC*statMOvsO
11d-TXB2 [M-H]	367.2122	-5.335067146	-0.8803872	0.0010815	0.03720363	0.475700298	-1.071875165	0.528203565	-0.920834057	3.642766857	0.623959125	0.006562644	0.096587859	1.53596721	0.619147417	1.651982671	0.724198553	-1.907435199	-0.345607028	0.098127092	0.498091089	0.691622849	-0.531942563	0.832215151	-0.264971541	-19.43440578
12oxo LTB4 [M-H+Acetate]	393.2289	-4.259144517	-1.275490041	0.003750547	0.073225505	0.33509269	-1.577367882	0.374147065	-1.418322638	0.996852655	0.39027217	0.348028482	0.661274396	1.489715534	0.57503687	1.615267267	0.691772898	-1.670667081	-0.780834562	0.138712658	0.55113297	0.539443436	-0.890456404	0.649563507	-0.622457512	-4.245739519
14,15-DiHETE [M-H+Acetate]	395.2439	-3.135762545	-1.06611592	0.016476452	0.164742919	0.40422697	-1.306762515	0.452373517	-1.144413621	2.038948636	0.514735689	0.075797418	0.333518868	1.415417863	0.501228032	1.514543957	0.598883451	-1.854474456	-0.516737054	0.106068304	0.511707754	0.57091287	-0.808657511	0.695830478	-0.523192224	-6.393658763
15(S) HEPE [M-H]	317.2124	-5.216507731	-1.038081753	0.001230507	0.038328302	0.441117278	-1.180765826	0.486823068	-1.038530563	3.684351835	0.811227237	0.006180275	0.094611384	1.718810262	0.781410296	1.845962115	0.884130646	-1.099300252	-0.216554503	0.308001098	0.675344707	0.76849183	-0.379898173	0.91873231	-0.122283528	-19.21944983
15(S) HEPE [M-H+Acetate]	377.2332	-6.325572287	-0.962707732	0.000394353	0.025604563	0.457935038	-1.126785139	0.506469837	-0.981451743	3.789121421	0.717741391	0.005318487	0.087859906	1.631924126	0.706573982	1.750758691	0.807980249	-1.33599067	-0.245215747	0.223349236	0.619430327	0.748771511	-0.41740255	0.896923571	-0.15694304	-23.96836145
15d PGA2 [M-H]	315.1967	-3.351807411	-1.116916326	0.012220498	0.143844529	0.379238021	-1.398824485	0.42570208	-1.232083956	1.736366884	0.569466497	0.120708953	0.415378934	1.556934773	0.638708505	1.670110199	0.739943299	-0.16123188	-0.058848981	0.876465519	0.950269824	0.814449389	-0.296103044	0.993660633	-0.009174886	-5.819967391
15d PGJ2 [M-H]	315.1967	-2.542552217	-0.985783616	0.03852018	0.257630868	0.409306402	-1.288746864	0.461053698	-1.116993307	1.489030938	0.626660193	0.174804573	0.494105167	1.671629163	0.741254833	1.800245022	0.848193277	-0.941287431	-0.327204165	0.377897886	0.706049834	0.625499693	-0.676918918	0.766295351	-0.384027542	-3.785938913
15R-PGD2 [M-H]	351.218	-6.223823192	-1.4156692	0.000435056	0.026405285	0.321548752	-1.636890603	0.358468085	-1.480083417	5.056718873	1.043259105	0.000981035	0.047741268	2.070684819	1.050107976	2.219273772	1.150087651	-0.721738307	-0.277686777	0.493852247	0.768214737	0.788090735	-0.343566354	0.951871979	-0.071160542	-31.47212419
17-HETE [M-H]	319.2279	-3.202885464	-1.527317244	0.015005554	0.157696599	0.282556499	-1.823388724	0.316877739	-1.658001783	2.200449751	0.633384072	0.058952547	0.300146331	1.495451547	0.580581168	1.600549626	0.678567409	-1.066898752	-0.549777266	0.321425602	0.681370734	0.590084376	-0.761006834	0.719561445	-0.474810207	-7.047788522
17(18) EpETE [M-H]	317.2124	-3.641146032	-1.575115524	0.008274505	0.112753035	0.275824207	-1.858179016	0.31092087	-1.685380636	2.925341778	1.044529525	0.019134114	0.172706358	2.067260453	1.047720165	2.225424603	1.154080624	-0.173332348	-0.097616442	0.867295935	0.945603988	0.896328119	-0.157901139	1.092486376	0.127615289	-10.65159661
20cooh AA [M-H]	333.2075	-3.479309858	-0.924878494	0.010275834	0.1291112	0.450055437	-1.151825373	0.504036047	-0.98840118	2.492722261	0.621593511	0.03736338	0.239448539	1.550808495	0.633020543	1.670515057	0.740292986	-0.255310642	-0.081770089	0.805822306	0.926439925	0.835642862	-0.259041601	1.015411685	0.022064767	-8.672953139
6S-LXA4 [M-H+Acetate]	411.2387	-5.87014387	-1.091987584	0.000617851	0.029500467	0.40357408	-1.309094575	0.446755573	-1.162442368	1.287613986	0.404673875	0.233877684	0.556003055	1.377661316	0.46222126	1.497069371	0.582141075	-1.262016632	-0.287696272	0.247363932	0.635224991	0.703845849	-0.5066686	0.844751143	-0.243401697	-7.558479349
6t,12epi LTB4 [M-H]	335.2232	-5.439001783	-0.828760341	0.000967276	0.035647699	0.488741065	-1.032857767	0.543747596	-0.878990977	1.4573679	0.404873226	0.183119858	0.502703267	1.435038426	0.521089369	1.518678619	0.602816601	-2.52292421	-0.465753773	0.039640735	0.365164601	0.63312892	-0.659428799	0.76330363	-0.389671042	-7.926626605
8-iso-15-keto-PGE2 [M-H]	349.2023	-20.81286359	-1.527863498	1.49E-07	0.000668987	0.306464075	-1.70621013	0.337696932	-1.566199021	10.66104313	0.800385386	5.25E-06	0.003806141	1.731300864	0.791856457	1.855401178	0.891731162	-3.620953315	-0.505815765	0.008499416	0.214484777	0.629198175	-0.668413609	0.750588064	-0.413906746	-221.8868364
8-iso-PGA1 [M-H]	335.2232	-8.126951417	-2.403329614	8.24E-05	0.017507673	0.161597602	-2.629522307	0.181300852	-2.463542387	4.191783853	1.452371584	0.003030357	0.071769533	2.819132663	1.49525137	3.059100398	1.613107456	-0.78711221	-0.366496449	0.457033652	0.748635128	0.761067319	-0.393904023	0.921880092	-0.117348982	-34.06642373
8-iso-PGA1 [M-H+Acetate]	395.2441	-9.683481292	-2.375632867	2.64E-05	0.016446553	0.16347599	-2.612849334	0.182596019	-2.453272784	3.301898152	1.363230146	0.010828165	0.127143886	2.844758625	1.508306247	3.109595618	1.63672698	-0.605843848	-0.310120291	0.563738719	0.802056406	0.836808322	-0.257030895	1.012827251	0.018388128	-31.97386898
8-iso-PGA2 [M-H+Acetate]	393.2288	-8.563839231	-2.079593988	5.89E-05	0.016446553	0.198501762	-2.332776284	0.222422877	-2.168622915	2.744266016	1.247752948	0.025281968	0.198153498	2.793097562	1.481865967	3.042395754	1.605207831	-0.526955525	-0.315975308	0.614505615	0.830975563	0.919561004	-0.120982809	1.112123979	0.153317628	-23.50145296
8(9)-EpETE [M-H]	317.2124	-4.249009024	-1.529960053	0.003797778	0.073706276	0.290120013	-1.785278274	0.326021287	-1.616961928	2.24900291	0.740290429	0.054652275	0.290873392	1.702829415	0.767933917	1.83302596	0.874227218	-1.00519912	-0.499266869	0.348276138	0.694790021	0.676051068	-0.564795865	0.821912349	-0.282943546	-9.556033659
9-HETE [M-H+Acetate]	379.2472	2.341887593	0.410709775	0.051701113	0.290640479	1.140241254	0.189339104	1.275879351	0.351491912	-0.035631313	-0.013648003	0.97244937	0.991919654	1.140200873	0.189288011	1.213587079	0.27927763	0.076534175	0.015743995	0.94113566	0.980957362	0.86958264	-0.201604955	1.050914686	0.071645555	-0.08344453
9-oxoODE [M-H]	293.2125	2.510522044	0.411214911	0.040366087	0.261917668	1.1615861	0.216096094	1.291311487	0.368837046	0.215377922	0.120250477	0.834862197	0.943551023	1.581936095	0.661691321	1.701615888	0.766905409	-1.090060278	-0.132886874	0.311782424	0.677273074	0.787253652	-0.34509955	0.946298195	-0.079633221	0.540711021
9(S) HOTrE [M-H]	293.2124	2.74585302	0.552209029	0.028674984	0.222606633	1.301446644	0.380116166	1.437551944	0.523614087	-0.089559551	-0.022550353	0.930838816	0.978092599	1.020155651	0.028789289	1.080791233	0.112087878	0.615233733	0.171460319	0.557863651	0.798945963	1.004609013	0.006634124	1.203680041	0.26745195	-0.245917364
Adrenic Acid [M-H]	331.2646	3.042139579	0.622271808	0.018790164	0.176086663	1.332197811	0.413808316	1.468714144	0.554553631	0.275527338	0.037020091	0.789894074	0.928591897	1.016068141	0.022997158	1.090893263	0.12550995	3.683429849	0.814134952	0.007824159	0.211667678	1.494285342	0.579455665	1.797256858	0.845796609	0.838192621
Adrenic Acid [M-H]	331.2646	2.531524392	0.616556859	0.039145663	0.259183683	1.316696835	0.396923208	1.453037802	0.539072236	-0.144132257	-0.027335137	0.88896074	0.96406336	0.992523583	-0.010826712	1.056471323	0.079253607	2.626595419	0.574063887	0.034080836	0.353324531	1.253293374	0.325724164	1.50445371	0.589239718	-0.364874324
Arachidonic Acid [M-H]	303.2327	2.888440061	0.860474804	0.023368229	0.198158938	1.577260826	0.657421254	1.719680915	0.782140899	0.635475056	0.113640953	0.54286427	0.807854383	1.060327063	0.084509339	1.135338318	0.183122268	2.515936541	0.8020826	0.040047744	0.365164601	1.495674534	0.580796272	1.76781672	0.82196871	1.83553161
ASPARAGINE	131.0463	-3.41888406	-0.731848981	0.011152309	0.137215481	0.517559654	-0.950202938	0.571548608	-0.807051895	2.070487113	0.689703693	0.072174428	0.329125488	1.745930115	0.803995813	1.863104824	0.897708847	-0.157493599	-0.028864135	0.879302534	0.951058521	0.826266299	-0.275321268	0.991865034	-0.011784272	-7.078755386
ASPARAGINE	133.0605	-2.977728398	-0.582348143	0.020581009	0.184860554	0.577697195	-0.791614606	0.633500653	-0.658581989	3.793548215	0.725262041	0.005285026	0.087859906	1.686218963	0.753791889	1.807750773	0.854195794	-0.233530467	-0.055479345	0.82203184	0.931995558	0.828284362	-0.271801944	0.988067249	-0.017318858	-11.29615625
ASPARAGINE (M-H2O-H)	113.0357	-3.254611632	-0.582683569	0.013967975	0.151808603	0.576088853	-0.795636752	0.637549716	-0.649390248	2.136643358	0.712247143	0.065115895	0.314680419	1.780520181	0.832298787	1.901560209	0.92718362	0.701981531	0.111401809	0.505354431	0.773668602	0.915156148	-0.127910171	1.102117433	0.140277954	-6.953944327
CARNOSINE	225.0993	-2.941171661	-0.681160769	0.02167712	0.189267699	0.546238277	-0.872397682	0.607748311	-0.718454115	0.827119366	0.158440226	0.43214669	0.7274209	1.088817242	0.122761818	1.169857548	0.226332866	-0.503454233	-0.096040025	0.630097954	0.838202521	0.806971096	-0.309411095	0.972260044	-0.040585862	-2.432700039
CARNOSINE(M+Na)	249.0955	-3.333592737	-0.497778196	0.012529201	0.14486333	0.611069869	-0.71059075	0.6797213	-0.556984763	1.722204333	0.213231622	0.123332504	0.419536389	1.152755606	0.205086682	1.238642315	0.308759638	0.358328611	0.059044914	0.730660044	0.89483282	0.900006656	-0.151992423	1.085211573	0.117976338	-5.741127856
CITRATE (M+Na-2H)	213.0017	2.321223434	0.256209292	0.053298329	0.296046341	1.050307187	0.070811339	1.155795367	0.208885992	0.946765494	0.110445889	0.371473929	0.679960994	1.073555741	0.1023971	1.15261544	0.204911251	2.950994137	0.382950674	0.021376664	0.30668594	1.132796575	0.179888809	1.354072347	0.437304823	2.197654252
CITRATE(M+2Na-H)	236.9983	4.112487564	0.439979726	0.004501823	0.08020871	1.193280582	0.254933312	1.311893168	0.391650241	2.23179288	0.138930117	0.056139733	0.293574339	1.093755859	0.129290745	1.172550422	0.229649963	4.213757148	0.561651364	0.003967204	0.178414759	1.282236647	0.358662547	1.532173971	0.615580118	9.178220463
CITRATE(M+Na)	215.0161	2.706531204	0.565840414	0.030350892	0.229787583	1.314911491	0.394965693	1.459771847	0.545742903	-1.725740032	-0.423912893	0.122672596	0.417727414	0.754605307	-0.406205849	0.807857627	-0.307827033	1.194314066	0.173179212	0.27125024	0.651607504	0.967512461	-0.047647852	1.167795067	0.223787122	-4.670769247
CITRULLINE	176.1026	4.661421136	0.429668822	0.002310647	0.054200457	1.194316521	0.256185234	1.318489724	0.398886327	-2.770565359	-0.390522801	0.024275488	0.194608996	0.760754241	-0.394497623	0.816080038	-0.293217442	0.643848091	0.043333299	0.540187544	0.790338304	0.904628138	-0.144603224	1.082633114	0.11454442	-12.91477192
CITRULLINE(M+Na)	198.0856	3.731111109	0.284249388	0.007348	0.105542527	1.074194971	0.103255872	1.183211425	0.242707888	-1.02674105	-0.093799869	0.334582993	0.646108478	0.934246147	-0.098125386	1.000779397	0.001123994	2.787023931	0.208212197	0.027023758	0.331244217	1.008540008	0.012268316	1.204504902	0.268440265	-3.830884939
Dihomo-g-linolenic [M-H]	305.2487	2.780538748	0.607888619	0.027277093	0.216919416	1.335717988	0.417615442	1.460116309	0.546083295	0.055434258	0.008417105	0.95715194	0.987493102	1.000425183	0.000613279	1.069690205	0.097193036	2.738047839	0.635342205	0.02899977	0.340734231	1.348155562	0.430986977	1.601227575	0.679178366	0.154137102
Docosadienoic Acid [M-H]	335.2958	2.609069045	0.679911269	0.034960662	0.245666463	1.318881333	0.399314763	1.463346657	0.549271575	-3.78999729	-0.480669639	0.005311849	0.087859906	0.72194382	-0.470041521	0.773238206	-0.371015172	1.746447037	0.51719605	0.124235293	0.535805784	1.186071492	0.246190973	1.427026554	0.513012181	-9.888364611
Docosatrienoic Acid [M-H]	333.2803	4.081418855	0.568939614	0.004681304	0.081404749	1.300461061	0.379023203	1.432994273	0.519032844	-0.977683116	-0.155497794	0.356866028	0.667028245	0.906229783	-0.14205119	0.970454706	-0.043267215	4.54948505	0.523099716	0.002637715	0.142153195	1.236122487	0.305821706	1.482028296	0.567572993	-3.990334303
Eicosadienoic Acid [M-H]	307.2645	3.177886273	0.627795435	0.015536289	0.161046121	1.3731404	0.457479145	1.512793449	0.597215021	-0.173289742	-0.037631869	0.866728016	0.957143674	0.991095464	-0.012904068	1.05123735	0.072088439	3.922519901	0.621012667	0.005730457	0.199830543	1.33650081	0.418460712	1.59898966	0.677160609	-0.550695094
ELAIDATE [M-H]	281.2489	2.719154748	0.479920912	0.029801982	0.227562906	1.234512519	0.303941467	1.365491594	0.449420433	2.437089073	0.428030642	0.040749114	0.249976455	1.344751951	0.427340082	1.429867316	0.515881278	3.239460692	0.886059933	0.014263696	0.258371003	1.653596454	0.7256072	1.987161714	0.990709283	6.626822324
ERUCATE [M-H]	337.3115	2.484662009	0.60602196	0.041922929	0.266102627	1.319435346	0.399920658	1.453785716	0.539814635	0.481602608	0.076217062	0.6429863	0.863315078	1.044456961	0.062753044	1.11188982	0.153013835	2.72039624	0.833433005	0.029748561	0.343393314	1.560281016	0.641805891	1.859925617	0.895244926	1.196619704
Estrone [M-H+Acetate]	329.1762	-6.476663503	-1.351042183	0.000341558	0.024987604	0.34887209	-1.51922991	0.387424219	-1.36801395	4.300431633	0.71705935	0.00261423	0.069017783	1.598081597	0.676341073	1.70855502	0.772776707	-5.410688589	-0.629113581	0.000996999	0.082009739	0.558975912	-0.839141981	0.672961927	-0.571403209	-27.8524486
FAHFA_499.378735	499.3801	3.505909914	0.849559662	0.009913815	0.126488734	1.636798096	0.710876372	1.802173288	0.84973774	0.367372382	0.124987571	0.722869797	0.89815167	1.109285687	0.149630968	1.19505692	0.257079335	3.32847854	0.651905461	0.012617375	0.247080784	1.384249105	0.46910359	1.655060203	0.726883696	1.287974475
FAHFA_501.394385	501.3955	3.767744595	0.827218493	0.007003556	0.103008133	1.609055381	0.686213982	1.770857774	0.824448347	-0.022935884	-0.007177983	0.982263182	0.994854753	1.004493622	0.006468404	1.083759977	0.116045275	2.219157862	0.413901142	0.061954205	0.430248532	1.177477611	0.235699628	1.406345291	0.491950854	-0.086416552
FAHFA_503.410035	503.4104	2.65841201	0.627916169	0.032542012	0.240272198	1.406423855	0.492031446	1.557173656	0.638929843	0.372283027	0.149116006	0.719350861	0.896170831	1.158020398	0.211660666	1.250594777	0.322614397	2.40814552	0.371308819	0.046901567	0.391607065	1.130144443	0.176507174	1.355858772	0.439206913	0.989681671
FAHFA_505.425685	505.427	5.836284765	0.711981138	0.000639451	0.02957347	1.442903576	0.528974893	1.598087183	0.676346116	-0.889826522	-0.253451979	0.399525882	0.700916942	0.868839048	-0.202839152	0.939553966	-0.089952065	3.087620806	0.325067925	0.017625754	0.282401819	1.09037888	0.124829523	1.310024113	0.389593367	-5.193280974
FAHFA_529.425685	529.4263	3.720461244	0.426379995	0.007451556	0.106513536	1.190620546	0.251713696	1.319159917	0.399619468	-0.913950849	-0.23925384	0.38745839	0.693111299	0.870449104	-0.20016815	0.933418329	-0.099404298	1.244396323	0.191528564	0.253401487	0.637758046	1.001292338	0.001863246	1.203173379	0.266844552	-3.400318712
FAHFA_529.425685	529.4263	2.383836906	0.705052232	0.048607153	0.284275558	1.457400878	0.543397765	1.611943323	0.688801019	-0.064234414	-0.021953308	0.95035963	0.98526155	0.977380454	-0.033007841	1.056240459	0.078938309	1.05770783	0.229339893	0.325317789	0.683275848	1.004900606	0.007052812	1.205202151	0.269275153	-0.153124368
FAHFA_555.441335	555.4425	2.647656121	0.458826818	0.033053965	0.241978662	1.225563901	0.293445708	1.351238408	0.434282242	-0.941972185	-0.243293735	0.37377793	0.680839632	0.855633283	-0.224935324	0.915506645	-0.127357738	1.474701377	0.308336763	0.18379273	0.585783182	1.087530415	0.121055749	1.299678628	0.378154931	-2.494018422
FAHFA_557.456985	557.458	5.261985075	0.639336411	0.001170806	0.038328302	1.389997228	0.475082005	1.53122079	0.614682323	-0.730690478	-0.144073674	0.485814297	0.768946333	0.903653856	-0.146157839	0.97387826	-0.038186656	4.023933589	0.260469137	0.005034401	0.192456318	1.049140892	0.069208434	1.255737106	0.328534462	-3.844882388
FAHFA_559.472635	559.4731	3.471616385	0.535425069	0.010383181	0.12995916	1.293243607	0.37099406	1.434008179	0.520053252	-1.304119888	-0.317001194	0.228461923	0.55081462	0.807996586	-0.307578898	0.863167292	-0.212287898	1.36288582	0.259099414	0.215136426	0.614410866	1.052046483	0.073198449	1.265080562	0.33922926	-4.527403971
FAHFA_619.566535	619.5682	2.595708281	0.571954877	0.035647209	0.248881635	1.278553587	0.354512629	1.4226626	0.508593552	-0.070907742	-0.019768426	0.945211737	0.983275944	1.053483886	0.075168246	1.111801642	0.152899418	2.003018384	0.634887869	0.085239031	0.475795095	1.401332796	0.486799615	1.685773852	0.75341101	-0.184055812
GLUCONATE	195.051	2.589158101	0.778031829	0.03598889	0.250195688	1.552904341	0.634968962	1.704714469	0.769530115	-1.179945654	-0.374159399	0.271916392	0.592010918	0.758420933	-0.39892931	0.817742806	-0.290280932	2.045117087	0.473826219	0.080105628	0.469612231	1.206995398	0.271420176	1.43670105	0.522759896	-3.055065849
GLUTAMINE(M+Na)	169.0581	2.514950041	0.20676874	0.040105551	0.261043415	1.013700458	0.019631408	1.120049148	0.16356204	-0.774603937	-0.080866825	0.460851955	0.752368656	0.948287401	-0.076603727	1.01458664	0.020892069	2.382569715	0.208224936	0.048697786	0.393217926	1.002320197	0.00334346	1.203585181	0.267338249	-1.948090203
Gondolic Acid [M-H+Acetate]	369.301	2.580566411	0.596099712	0.036442225	0.251470631	1.29902246	0.377426375	1.419596717	0.505481143	-0.315843907	-0.051736782	0.760203723	0.913954365	0.963193507	-0.054102428	1.040685975	0.057534803	1.166132413	0.271263104	0.281750378	0.659025359	1.021481697	0.030663353	1.216053853	0.28220712	-0.815056177
HEPTADECANOATE [M-H]	269.2488	3.110602289	0.667474342	0.017066806	0.168625219	1.420618803	0.506519486	1.56795754	0.648886492	1.149858095	0.243316019	0.283413544	0.602766393	1.164296685	0.219458731	1.251406459	0.323550455	3.7938037	0.737070517	0.006769236	0.202513001	1.450419482	0.536470209	1.739548002	0.79871249	3.576751222
HISTIDINE	156.0766	-3.068782674	-0.673698534	0.018098458	0.173049922	0.540331262	-0.88808394	0.600148631	-0.736608258	2.147191391	0.471071205	0.064054913	0.313371476	1.402115847	0.487605554	1.496891547	0.581969699	-0.966653626	-0.212533778	0.365919143	0.701728322	0.739125265	-0.436109206	0.893943951	-0.161743716	-6.589263738
Lignoceric Acid [M-H]	367.3584	3.54324518	0.390091012	0.009428733	0.122969541	1.151901766	0.204017689	1.270316424	0.345187904	0.550506659	0.143541832	0.597004156	0.839545421	1.188923675	0.249656101	1.255454441	0.328209676	3.739875443	0.652028139	0.00726395	0.205948786	1.383175704	0.467984433	1.657911295	0.729366819	1.950580065
OLEOYL-GLYCEROL [M-H]	355.2853	-6.124514062	-1.961024911	0.000479377	0.026758206	0.236225887	-2.081761022	0.264753721	-1.917277134	1.825383274	0.714416223	0.105379784	0.388799435	1.714792546	0.778034051	1.795423819	0.844324439	-3.00698307	-1.595195088	0.019746166	0.300852219	0.340439827	-1.554528272	0.408329557	-1.292194096	-11.17958553
PALMITOLEATE [M-H+Acetate]	313.2385	-2.369237985	-2.362110765	0.049661843	0.285863059	0.321130011	-1.638770599	0.365485096	-1.452115517	0.666411303	0.836634582	0.523897295	0.794087209	1.476257304	0.561944197	1.525076466	0.60888158	-1.645869639	-1.427749329	0.143786119	0.553920021	0.439760296	-1.185210738	0.532974244	-0.907862278	-1.578886972
Palmitoleic Acid [M-H+Acetate]	313.2386	3.572389634	0.933298617	0.009067905	0.119217362	1.673192926	0.742603804	1.843632497	0.882551103	-2.688462449	-0.524218237	0.02756221	0.204707484	0.675658631	-0.565633569	0.728937681	-0.456132616	2.926730097	0.592579424	0.022127003	0.309198449	1.276954632	0.35270727	1.533194095	0.616540347	-9.604235383
PGA3 [M-H]	331.1919	-6.208265981	-1.30577215	0.000441685	0.026405285	0.35730134	-1.484786769	0.398289501	-1.328110643	5.217256751	0.971840304	0.000805273	0.04269255	1.904970281	0.929768491	2.043952598	1.031361738	-1.42623892	-0.27001428	0.196837146	0.595768271	0.716117306	-0.481732163	0.86469659	-0.209734095	-32.3901176
PGA3 [M-H+Acetate]	391.2135	-6.402555096	-1.153095019	0.00036639	0.024987604	0.399959106	-1.322075598	0.444468223	-1.169847818	5.400141518	0.922625196	0.000645908	0.040614693	1.851390334	0.888609095	1.980130841	0.985595762	-1.012247539	-0.198271869	0.345122555	0.693683791	0.764331315	-0.387729956	0.921210079	-0.118397899	-34.57470359
SERINE	104.0354	-4.77928356	-0.748540613	0.002013948	0.049746038	0.515449756	-0.956096289	0.569274762	-0.812802953	3.426564039	0.689154187	0.009000343	0.11470906	1.66056085	0.73167059	1.769267239	0.823151976	-1.324831792	-0.186243514	0.226836611	0.619783389	0.751836406	-0.411509319	0.901802162	-0.149117126	-16.37652118
SERINE(M-H2O+H)	88.0398	-2.464654888	-0.386963421	0.043169789	0.2686166	0.675979325	-0.564948972	0.739769966	-0.434851365	2.494844146	0.512321687	0.037240034	0.239448539	1.443930836	0.530001639	1.551789851	0.633933196	0.064922554	0.010082513	0.950050841	0.984471879	0.874268767	-0.193851234	1.04060531	0.057422975	-6.148929818
STEARATE [M-H+Acetate]	343.2852	3.368436982	0.70890002	0.011945791	0.142150548	1.409436227	0.495118202	1.554184547	0.636157823	1.879695955	0.266278475	0.096950852	0.374757856	1.196713374	0.259077653	1.285029941	0.361801975	3.551378616	0.897606869	0.009326499	0.220355443	1.59897386	0.677146354	1.91518245	0.937481837	6.331637368
Tetrahydrocortisol [M-H]	365.2338	2.472126002	1.676380722	0.042699776	0.267357014	3.144396329	1.65278307	3.538025411	1.82294441	-0.726613706	-0.446108047	0.488175535	0.770797256	0.665435094	-0.58763014	0.698155408	-0.518379881	-0.117621492	-0.091435434	0.909671418	0.966667369	0.932222254	-0.101254141	1.1288396	0.174840504	-1.796280636
Tricosenoic Acid [M-H+Acetate]	411.3483	5.225814203	0.872408464	0.00121802	0.038328302	1.604698802	0.682302532	1.752592271	0.809490402	-3.552287783	-0.506353344	0.007484418	0.102318431	0.702560209	-0.509306227	0.755886502	-0.403758469	1.034284955	0.209112605	0.335406619	0.691854374	1.007461682	0.010724968	1.195680909	0.257832429	-18.56359595
Unknown oxylipin (EIC_131)	331.1918	-2.991942009	-1.074423431	0.020170816	0.182940047	0.433418089	-1.206168727	0.475646142	-1.072039419	1.683859842	0.714521137	0.130704363	0.431100891	1.721813613	0.783928979	1.8307959	0.872470966	1.956340443	0.491544739	0.091310691	0.486166819	1.227131608	0.295289984	1.452030577	0.538071834	-5.038010997
Unknown oxylipin (EIC_131)	331.1918	-2.826901572	-0.593138872	0.025519336	0.208014489	0.603430155	-0.728741302	0.66607047	-0.586253273	2.51297556	0.47114801	0.03620273	0.237523793	1.340898564	0.423200104	1.435754438	0.521809021	0.337835295	0.06112597	0.7453925	0.901465967	0.923735282	-0.114448622	1.106689981	0.146251134	-7.103934561
Unknown oxylipin (EIC_134)	331.1918	-7.808531589	-1.67983564	0.000106362	0.019884795	0.267106486	-1.904513085	0.299474812	-1.739493429	4.625281081	0.755365669	0.001698463	0.057594588	1.700017367	0.765594484	1.806041195	0.8528308	-3.285810361	-0.90627586	0.013379512	0.2538783	0.471409559	-1.084947082	0.570890783	-0.808713325	-36.11665342
Unknown oxylipin (EIC_138)	333.2075	-8.393902823	-2.15515849	6.70E-05	0.016446553	0.191645237	-2.383489949	0.213855463	-2.225292034	6.521974863	1.460133671	0.000183803	0.028695533	2.760918192	1.46514814	2.958483845	1.564858017	-0.452867619	-0.22572015	0.664346957	0.85737067	0.872011411	-0.197581081	1.053353572	0.074989777	-54.74482322
Unknown oxylipin (EIC_138;EIC_139;EIC_140;EIC_145	333.2075	-6.387465832	-2.718358818	0.000371691	0.024987604	0.119063842	-3.070192741	0.134260476	-2.896893431	4.798548333	1.626537347	0.001358054	0.055133274	3.223857196	1.68878784	3.461423809	1.791365593	-0.691360397	-0.500497666	0.511608963	0.776764156	0.77836491	-0.361481422	0.947496016	-0.077808218	-30.65056352
Unknown oxylipin (EIC_138;EIC_140)	333.2074	-5.508318642	-2.213862822	0.000898589	0.03487916	0.182234402	-2.456132756	0.204192037	-2.29200149	3.595988275	1.063147627	0.007023104	0.098915041	2.035035111	1.025053686	2.188767928	1.130118996	-1.163337519	-0.757529062	0.282809921	0.659025359	0.67069683	-0.576267311	0.814392558	-0.296203716	-19.80784925
Unknown oxylipin (EIC_143)	333.2074	-3.76211296	-1.207349357	0.007055346	0.103156747	0.365673789	-1.451370874	0.40964694	-1.287547058	2.723969017	0.536702173	0.026088033	0.199662582	1.404447921	0.490003128	1.505459339	0.590203743	-2.849012944	-0.824551222	0.024723357	0.319882961	0.465614874	-1.102790949	0.565682995	-0.821934291	-10.24787914
Unknown oxylipin (EIC_144;EIC_148;EIC_149)	333.2074	-6.528266897	-1.686937824	0.000325382	0.024987604	0.269200608	-1.893246425	0.300252854	-1.735750138	5.493753265	0.995315291	0.000577989	0.039674412	1.937452392	0.95416086	2.086594805	1.061150472	-2.18212369	-0.518934632	0.06543523	0.440354115	0.595450338	-0.747946907	0.722059365	-0.469810641	-35.86468758
Unknown oxylipin (EIC_148)	333.2074	-5.410469799	-1.48947753	0.000997233	0.035647699	0.308238837	-1.697879449	0.344398311	-1.537850027	3.863321737	0.723626902	0.004786361	0.085395354	1.600722488	0.678723215	1.71982441	0.782261276	-2.599905421	-0.675577695	0.035430042	0.358356138	0.540046686	-0.888843963	0.655514159	-0.609301151	-20.90238558
Unknown oxylipin (EIC_156;EIC_159;EIC_160;EIC_162	335.2232	-3.605271458	-1.118154882	0.008678652	0.11644382	0.379021895	-1.399646904	0.424398406	-1.236508857	2.504654405	0.501684095	0.0366751	0.238281789	1.418894048	0.504766864	1.523422238	0.60731586	-1.382993088	-0.507234168	0.209171415	0.607034623	0.606613709	-0.721149993	0.736870362	-0.440517267	-9.029959039
Unknown oxylipin (EIC_170;EIC_172;EIC_173)	335.2231	-3.131460309	-1.404521164	0.016575835	0.165276793	0.280046864	-1.836259821	0.316560215	-1.659448145	2.28060569	0.469658056	0.05202212	0.283143399	1.428293068	0.514292033	1.524257062	0.60810623	-1.51762286	-0.852258127	0.17289884	0.578678376	0.4668765	-1.09888712	0.569489202	-0.812259608	-7.141626198
Unknown oxylipin (EIC_171)	335.2231	-4.847533076	-1.521987172	0.001861583	0.04817216	0.302756266	-1.723771277	0.338557789	-1.562525983	4.321702067	0.961133185	0.002540232	0.068025432	1.871983658	0.90456784	2.00959425	1.006904241	-2.257427824	-0.557028961	0.058553087	0.419027519	0.568868106	-0.813833898	0.691748955	-0.531679535	-20.94959371
Unknown oxylipin (EIC_219)	349.2023	-7.623015857	-2.771651035	0.000123888	0.020000824	0.117861105	-3.084840397	0.131819869	-2.923360252	3.132525828	1.07230783	0.013961913	0.145102942	2.165676462	1.11481773	2.35918701	1.238289784	-1.12853935	-0.75642433	0.296280125	0.666666881	0.666674471	-0.584945612	0.805293697	-0.312413054	-23.87929405
Unknown oxylipin (EIC_232;EIC_237;EIC_238;EIC_240;	351.218	-3.573684203	-1.132991191	0.009052228	0.119217362	0.373348509	-1.421405124	0.418079095	-1.258152188	4.141576418	0.722814581	0.003246339	0.073250429	1.641604	0.715106151	1.750392993	0.807678868	-1.237194538	-0.366786408	0.255905113	0.639505888	0.623198836	-0.682235557	0.757023222	-0.401590539	-14.80068622
Unknown oxylipin (EIC_328;EIC_332;EIC_335;EIC_339	375.2173	-5.230974036	-1.006515716	0.001211157	0.038328302	0.436287656	-1.196648439	0.487436889	-1.036712658	3.25079109	0.602619146	0.011687231	0.1326619	1.512377151	0.596817958	1.617145691	0.693449659	-0.757473011	-0.1047567	0.473488603	0.755930151	0.798363692	-0.324881984	0.96437111	-0.052339662	-17.00480379
Unknown oxylipin (EIC_33)	299.2591	3.493668372	0.613018995	0.010078679	0.127370283	1.35674523	0.440149836	1.492660153	0.577885732	-1.570911276	-0.252554249	0.1548451	0.465964418	0.834633176	-0.260785827	0.892400579	-0.164236646	-0.467089968	-0.084152456	0.654625757	0.852910002	0.822314986	-0.282236974	0.980696448	-0.028121442	-5.48824304
Unknown oxylipin (EIC_62;EIC_66)	311.2229	2.311093175	0.37300653	0.054099599	0.298118003	1.146983316	0.197844406	1.275486591	0.351047732	-0.775043471	-0.231067152	0.460606497	0.752368656	0.909035136	-0.137592036	0.975209851	-0.036215395	-1.79552451	-0.185151867	0.115639339	0.525175795	0.765632206	-0.385276577	0.921710574	-0.117614293	-1.791197676
Unknown oxylipin (EIC_79)	315.1967	-5.49484778	-1.508184197	0.0009115	0.034935692	0.299097213	-1.741313629	0.336301203	-1.572174154	4.058010716	0.765684937	0.00364348	0.075266337	1.674326881	0.743581214	1.791962827	0.84154071	-1.781190319	-0.433722066	0.118089275	0.530709966	0.619704865	-0.690346801	0.753519296	-0.408283638	-22.29815117
Unknown oxylipin (EIC_83;EIC_93;EIC_94)	317.2124	-6.174747661	-2.334963942	0.000456356	0.026405285	0.159606591	-2.647407861	0.180273697	-2.471739179	2.717392762	1.486447759	0.026354828	0.200069657	3.454606307	1.788521308	3.769110077	1.914223929	-0.251780116	-0.136761533	0.80844314	0.927797853	0.862728969	-0.213020695	1.052538795	0.07387341	-16.7792146
Unknown oxylipin (EIC_87)	317.2124	-3.288239351	-1.637862566	0.013334826	0.150183761	0.26702027	-1.904978831	0.300339588	-1.735333444	2.254499977	0.749553679	0.054185462	0.289863328	1.582509313	0.66221399	1.703148732	0.768204428	-0.517114872	-0.24881459	0.621009249	0.834109436	0.708316965	-0.497532999	0.865256977	-0.208799426	-7.413335543
Unknown oxylipin (Novel EIC_1)	269.2125	2.845118121	0.525137123	0.024861647	0.205304457	1.25154402	0.323709036	1.391861058	0.477015202	-3.186344055	-0.485566416	0.012873993	0.139170901	0.71429619	-0.48540567	0.764175693	-0.388023726	-0.510530904	-0.076772286	0.625380983	0.836279273	0.808418939	-0.306824974	0.975833206	-0.035293518	-9.06552521
unknown	636.436	-6.190781204	-4.643492019	0.000449271	0.026405285	0.031040263	-5.009715426	0.035384215	-4.820750268	4.162791704	2.744560251	0.003153115	0.072454858	6.884517047	2.783355452	7.483866104	2.903783747	-0.695039462	-0.774039752	0.509436852	0.775417468	0.930100141	-0.104542039	1.135986042	0.183945109	-25.77093264
unknown	635.4326	-6.394852059	-4.667878873	0.000369086	0.024987604	0.029886024	-5.064385193	0.034156897	-4.871679253	3.844793481	2.676723788	0.004913667	0.086584022	6.988322915	2.804946273	7.579582472	2.922118378	-0.707814985	-0.792067162	0.501940332	0.77156636	0.92113781	-0.118511083	1.125159404	0.170129406	-24.58688551
unknown	375.2558	2.617912283	2.225056009	0.034513817	0.243991691	5.298018421	2.40545286	5.811618468	2.538939993	-4.629524126	-5.145287764	0.001689097	0.057594588	0.042552953	-4.554596952	0.046315178	-4.432371132	-5.991228153	-3.580230304	0.000547025	0.06485728	0.080874016	-3.628179943	0.095589194	-3.387008661	-12.11968807
unknown	410.2258	-7.235455979	-4.076352674	0.000172081	0.022002789	0.052709756	-4.245786168	0.058565098	-4.093815054	5.528267123	2.546504009	0.000554958	0.039241075	5.439272178	2.443413619	5.82269653	2.54168743	-1.147119905	-0.861085056	0.289023382	0.663448842	0.673648819	-0.569931401	0.813402666	-0.297958375	-39.99953341
unknown	334.2108	-3.413513086	-3.692913797	0.011234026	0.137442928	0.021641664	-5.53004473	0.024903634	-5.327499912	1.877053109	2.329422467	0.097345707	0.37556721	30.9040383	4.949723466	30.38074907	4.925085536	-1.765346743	-2.070898491	0.120855035	0.532372766	0.083010579	-3.590560986	0.102323369	-3.288792428	-6.407345351
unknown	444.9849	-3.051398996	-3.367933109	0.018546639	0.175543961	0.277700567	-1.848397973	0.307300869	-1.702276247	1.921868306	2.541057072	0.090853364	0.362136179	2.664754266	1.414002499	2.840138189	1.505961127	-0.41324222	-0.137090629	0.691794486	0.873077577	0.861130203	-0.215696705	1.031434464	0.044652157	-5.864387018
unknown	480.2679	-4.771313766	-3.673357634	0.002032623	0.04982982	0.068357129	-3.870764395	0.076943234	-3.700061715	2.96906059	1.901333401	0.017896799	0.166959983	3.342642943	1.740989256	3.653458235	1.869262715	-0.976018821	-0.927181457	0.361570597	0.699757782	0.681491551	-0.553232324	0.833361118	-0.262986304	-14.16631966
unknown	299.2017	-4.312114141	-3.139989	0.003513983	0.069861832	0.079654495	-3.650100405	0.091350084	-3.452450133	2.93521369	2.218523043	0.018847158	0.171411883	5.714225373	2.514557938	6.180197597	2.627652966	0.015688643	0.013663523	0.98792058	0.996704155	0.942628985	-0.085238052	1.157372354	0.210853088	-12.65697646
unknown	453.2822	3.030554255	2.251401496	0.019099655	0.17651305	3.709445139	1.891203404	3.965578382	1.987531299	-5.772677534	-2.808570001	0.000418001	0.034499987	0.140141804	-2.835040724	0.14673632	-2.768702086	-1.657605415	-2.208288241	0.141363683	0.552326291	0.279125342	-1.841014982	0.321291439	-1.638045555	-17.49441246
unknown	479.265	-5.481006403	-3.460571728	0.000924982	0.035068641	0.060978261	-4.035561176	0.069257263	-3.85189082	3.793657566	1.764255065	0.005284203	0.087859906	3.791289311	1.922688552	4.093617482	2.033376299	-0.914700102	-0.940995469	0.39076852	0.708462886	0.684685625	-0.546486372	0.838638549	-0.253878948	-20.79306141
unknown	409.2225	-8.586507268	-3.489364969	5.79E-05	0.016446553	0.072911205	-3.777715651	0.081569674	-3.615823305	6.390671471	1.683821279	0.000211223	0.028695533	3.129205746	1.645796519	3.357380862	1.747336206	-1.281264318	-0.960336329	0.240910082	0.630154291	0.637405188	-0.649717333	0.768633179	-0.379632842	-54.87354704
unknown	438.2946	3.218413102	1.969141857	0.014685684	0.156123148	3.809802572	1.929716238	4.188423236	2.066407233	-4.094950336	-2.978156453	0.003461826	0.074729546	0.14425144	-2.793342373	0.149274359	-2.743961723	-2.664492157	-1.903014905	0.032256274	0.351590746	0.315663642	-1.663539994	0.369953085	-1.434585765	-13.17924181
unknown	437.2912	3.395744344	2.060757381	0.011508996	0.138981779	4.130691253	2.046383231	4.520112571	2.176358703	-5.482039681	-2.830936279	0.000586043	0.039808166	0.126981393	-2.977310983	0.133091624	-2.909508314	-2.989567022	-1.476871936	0.020238749	0.303668884	0.350679577	-1.511774684	0.410547872	-1.284377636	-18.61560524
unknown	347.2064	-3.305117707	-3.062213771	0.013028698	0.148531711	0.08589972	-3.541202762	0.097427346	-3.359529423	2.705315969	1.901973162	0.026852101	0.201199758	3.569729894	1.835814916	3.910290335	1.96727573	-2.611393365	-1.888907435	0.034842636	0.355538922	0.147325472	-2.762921207	0.183183963	-2.44863489	-8.941387714
unknown	633.4164	-4.75969254	-3.186199873	0.002060198	0.050316665	0.08979995	-3.477141541	0.101905768	-3.294692386	2.829468646	1.814935156	0.022168335	0.185032826	3.63300501	1.861163355	3.919024346	1.970494536	-1.371808525	-0.816734731	0.212471117	0.612512214	0.441314139	-1.180122124	0.542444333	-0.882453002	-13.46740081
unknown	377.2703	3.408390247	2.04978519	0.011312568	0.137835846	4.111893251	2.039802811	4.499647016	2.169811831	-5.360568074	-2.790997482	0.000677211	0.040614693	0.132134888	-2.91991666	0.13813961	-2.855801041	-2.898244249	-1.408053822	0.023043567	0.312521914	0.361222732	-1.469039409	0.424027016	-1.237771908	-18.27090795
unknown	630.3858	-4.131789115	-2.90311956	0.004394124	0.079214656	0.145899377	-2.776954371	0.164462135	-2.604172634	2.959727503	1.968653217	0.018153771	0.167678101	3.272564144	1.71042147	3.493204092	1.804550935	-3.373621884	-1.012530241	0.011861503	0.240213864	0.44366235	-1.172465967	0.536377442	-0.89867953	-12.22896988
unknown	634.4181	-4.66567546	-3.102907467	0.002299133	0.054200457	0.084864935	-3.558687617	0.096435823	-3.374287033	3.800738204	1.835932983	0.005231159	0.087859906	3.565715696	1.834191677	3.870563913	1.952543772	-1.318702522	-0.872043411	0.228772287	0.621510987	0.41432003	-1.271182527	0.510385254	-0.970341448	-17.73301097
unknown	393.2653	3.254667567	2.264886235	0.013966895	0.151808603	5.100654212	2.3506823	5.56305306	2.475876866	-2.550297563	-2.484228137	0.034158843	0.230176907	0.227871927	-2.133704892	0.247964358	-2.011795331	-1.283574196	-1.495556528	0.240145397	0.629669534	0.686121976	-0.54346302	0.806615473	-0.310047015	-8.300370766
unknown	299.1999	-5.20505818	-3.08722063	0.001246067	0.038328302	0.091506365	-3.449984084	0.104242609	-3.261982999	2.471579455	1.820476104	0.038615245	0.243765741	4.32681135	2.11330422	4.681085379	2.226843079	-0.216586876	-0.161316212	0.834707082	0.934210551	0.909990999	-0.13607582	1.11246706	0.153762619	-12.86471486
unknown	378.2738	3.283606795	1.952205853	0.01342019	0.150884589	3.934637305	1.976230654	4.308563117	2.107206819	-3.108571815	-2.831298791	0.01447674	0.147504409	0.185650019	-2.429342628	0.194587197	-2.361511303	-1.267668305	-1.18540099	0.245453674	0.634714093	0.581857617	-0.781261933	0.691560941	-0.532071707	-10.20732754
unknown	396.2471	-7.703237703	-2.867251889	0.000115938	0.019884795	0.119395338	-3.066181591	0.133805168	-2.901794254	4.307651055	1.922704134	0.002588856	0.068625739	3.952974436	1.982938625	4.289665466	2.100865142	-0.63207149	-0.366821162	0.547420641	0.79317667	0.847318473	-0.239023772	1.021390914	0.03053513	-33.18286001
unknown	300.2044	-5.654950558	-3.076732059	0.000770505	0.03200293	0.092154834	-3.439796342	0.104345332	-3.26056204	2.59501205	1.748094699	0.031863882	0.220110567	4.144011658	2.051028062	4.530362025	2.179626342	-0.192509065	-0.140048743	0.85280941	0.940474025	0.927683019	-0.108296161	1.133551365	0.180849766	-14.67466484
unknown	395.2441	-8.981213834	-2.779980448	4.32E-05	0.016446553	0.124019648	-3.011359399	0.138829165	-2.848617416	4.487930092	1.821365664	0.002034317	0.061338495	3.767148553	1.913472925	4.093808704	2.03344369	-0.698574611	-0.404973426	0.507355295	0.775417468	0.825267513	-0.277066245	0.995244892	-0.006876533	-40.30705983
unknown	346.2029	-4.158033147	-2.93184255	0.004252191	0.078232544	0.073407038	-3.767937793	0.08442999	-3.56610064	3.637960455	1.720094064	0.006608438	0.096587859	4.096691638	2.034459304	4.422675479	2.144919386	-2.625225243	-1.862744489	0.034148782	0.353466995	0.154972323	-2.689917511	0.192456393	-2.3773965	-15.12676016
unknown	445.2007	-5.086933338	-4.029281434	0.001420016	0.040613708	0.058205063	-4.10271153	0.064722104	-3.949597682	1.169299006	1.227700987	0.275940448	0.595446358	3.262859905	1.706137045	3.491129561	1.803693898	-1.568650038	-1.982027432	0.160717641	0.569720431	0.489847024	-1.029596819	0.594365354	-0.750578072	-5.948146094
unknown	335.223	-3.050327159	-2.265064804	0.018574656	0.175543961	0.152848494	-2.709825755	0.171893262	-2.540415102	5.669456717	2.150009945	0.000470695	0.035044854	4.03449369	2.012387634	4.351767508	2.121601483	-0.043909599	-0.030795964	0.966202771	0.989673803	0.696809689	-0.521163409	0.85769212	-0.221468229	-17.2936978
unknown	397.2513	-9.752914411	-2.847748985	2.52E-05	0.016446553	0.125497371	-2.994270951	0.140171817	-2.834731789	3.685566003	1.692738694	0.006169475	0.094611384	3.404262905	1.767342458	3.702906939	1.888658292	-1.041623253	-0.561527438	0.332219535	0.688847549	0.7703197	-0.376470774	0.925181954	-0.11219097	-35.94500978
unknown	348.1902	-5.02081422	-2.000120302	0.001529054	0.042105315	0.196542658	-2.34708562	0.219105237	-2.190304129	1.917922697	2.379062909	0.091407896	0.362931802	35.83547887	5.163316728	35.47832379	5.148865944	-0.56187202	-0.318093975	0.591730623	0.816691643	0.747950763	-0.418984792	0.90175034	-0.149200033	-9.62953355
unknown	369.1855	-9.110926887	-3.264310897	3.94E-05	0.016446553	0.091704455	-3.446864364	0.102195824	-3.290591848	2.29940412	1.448833626	0.050517801	0.280124644	3.64737659	1.866859165	3.820521192	1.933769463	-1.168192699	-1.168796253	0.280971437	0.659025359	0.745583492	-0.423558178	0.900000899	-0.152001652	-20.94970282
unknown	336.2265	-8.637280579	-2.703169978	5.57E-05	0.016446553	0.130889016	-2.93358406	0.146559834	-2.770438319	4.353145327	1.739670135	0.002434963	0.066159964	3.534942552	1.821686769	3.841897724	1.941819113	-0.675635876	-0.383892151	0.520959247	0.782493535	0.824513331	-0.278385275	0.99510264	-0.007082755	-37.59933759
unknown	376.2596	2.507243061	2.171727385	0.040560143	0.262654113	5.178032868	2.372404123	5.698641271	2.510617978	-1.974012175	-2.159098153	0.083821776	0.350002253	0.249612456	-2.002238164	0.262508528	-1.929563805	-1.509878057	-1.701084066	0.174819952	0.579563248	0.555492926	-0.848159555	0.657719614	-0.604455401	-4.949328327
unknown	289.1812	-4.085008054	-2.676234293	0.004660174	0.081404749	0.152816234	-2.710130284	0.171466626	-2.544000298	3.029782005	1.744717333	0.016314826	0.157847147	2.764728121	1.467137615	3.02889399	1.598791085	-0.233454696	-0.116648123	0.822088399	0.931995558	0.790651236	-0.338886647	0.968544639	-0.046109552	-12.37668389
unknown	429.196	-6.191166588	-2.917817998	0.000449103	0.026405285	0.103470412	-3.272709811	0.116385909	-3.103011698	3.201011387	1.590742853	0.012593195	0.13808775	3.405856786	1.768017772	3.636031713	1.862364783	-1.054824976	-0.90874974	0.326546338	0.684477232	0.670128863	-0.577489549	0.815782368	-0.293743769	-19.81799475
unknown	335.2232	-8.947900096	-2.686993132	4.43E-05	0.016446553	0.131897552	-2.922510305	0.14767148	-2.759536874	4.364995115	1.724714269	0.002396523	0.066159964	3.519303713	1.815290023	3.825767148	1.935749068	-0.667593259	-0.375790475	0.525783126	0.785661062	0.827069571	-0.273919404	0.998205124	-0.002591786	-39.05754021
unknown	394.2318	-6.505034231	-2.746832424	0.000332556	0.024987604	0.116980056	-3.09566551	0.131865281	-2.922863324	4.491042109	1.64566834	0.002025955	0.061338495	3.317192448	1.729962713	3.55707933	1.83069315	-0.721020264	-0.517831169	0.49426726	0.768214737	0.76832608	-0.38020937	0.935085095	-0.096830435	-29.21438265
unknown	475.268	2.851886005	1.587561407	0.024621866	0.204273774	2.481093182	1.31097592	2.718880352	1.443012665	-7.381270018	-2.824324808	7.76E-05	0.02086171	0.140069275	-2.835787571	0.147956942	-2.756750711	-2.963120367	-1.885057698	0.021011806	0.305218054	0.238359646	-2.068788087	0.281719944	-1.827666395	-21.05054066
unknown	446.2042	-2.783212628	-2.551295936	0.02717234	0.216614709	0.119336086	-3.066897725	0.134042571	-2.899236828	3.308406711	1.757421981	0.010723637	0.126225337	2.9005349	1.536318979	3.159120282	1.659522868	0.046045538	0.040808104	0.964560034	0.989599746	0.708370639	-0.49742368	0.870034938	-0.200854758	-9.207999338
unknown	521.3493	-3.765738423	-2.859237164	0.007021958	0.103008133	0.096478324	-3.373651346	0.109359046	-3.192855528	2.699271749	1.559232179	0.027104585	0.201768261	3.037051001	1.602671136	3.193204872	1.675005118	-1.070327091	-0.936153205	0.31998333	0.680618964	0.474306421	-1.076108695	0.580308118	-0.785108983	-10.16475134
unknown	636.4358	-3.023631178	-2.339070281	0.019287178	0.176645631	0.152622991	-2.711955789	0.17348802	-2.527092054	2.944708661	1.888822638	0.018575377	0.170126451	3.851169204	1.94529651	4.189475958	2.066769795	-0.657124444	-0.829748349	0.532104002	0.786457433	0.972744927	-0.039866544	1.188829852	0.249542248	-8.903712915
unknown	334.2109	-6.371981287	-2.717526698	0.00037722	0.025100554	0.11925436	-3.067886082	0.134485755	-2.89447473	4.713751268	1.621052029	0.001514324	0.05569795	3.215099137	1.684863224	3.451596956	1.78726401	-0.701009572	-0.508260876	0.505924737	0.773942504	0.775814345	-0.366216643	0.944253859	-0.08275332	-30.03593487
unknown	393.2286	-6.467939236	-2.723571951	0.00034438	0.024987604	0.119043893	-3.070434482	0.134154302	-2.898034776	4.573607967	1.605827131	0.00181717	0.059172455	3.198432508	1.677365041	3.432367362	1.779203971	-0.704045951	-0.50581922	0.504144492	0.773197012	0.778897789	-0.360494072	0.947702671	-0.077493591	-29.58181842
unknown	469.2961	-10.46440866	-2.65482297	1.58E-05	0.016446553	0.144781801	-2.788047822	0.160836766	-2.636330862	3.757030355	1.614093391	0.005567931	0.090319604	3.370040416	1.752765893	3.546624379	1.826446543	-3.478440171	-1.127048317	0.010287908	0.222881896	0.43037858	-1.216321819	0.515900276	-0.954835876	-39.31510097
unknown	315.1967	-7.189211047	-2.570006527	0.000179129	0.022463467	0.13821861	-2.854976217	0.155960206	-2.680750127	3.117649447	1.642822332	0.01427934	0.146408135	3.729453611	1.898964282	4.066924056	2.023938051	-0.208853054	-0.11797985	0.840510457	0.935779718	0.932146638	-0.101371168	1.133933691	0.181336278	-22.41343985
unknown	397.2	-8.551468591	-2.448206878	5.94E-05	0.016446553	0.156687194	-2.674040824	0.176176055	-2.504910241	3.185959767	1.713071505	0.012881437	0.139170901	3.981900634	1.99345722	4.341707281	2.118262462	-0.014761547	-0.007378866	0.988634333	0.996704155	1.016691852	0.023882481	1.232193797	0.301229178	-27.24463488
unknown	457.2581	2.494448808	2.03305835	0.04132658	0.264277757	4.457672448	2.156290611	4.876817567	2.285940005	-2.658876384	-2.045527505	0.028855721	0.209170025	0.217117019	-2.203455277	0.230945276	-2.114377058	-0.043781031	-0.041126809	0.966301657	0.989673803	1.093347301	0.128751745	1.278601874	0.354567113	-6.632431025
unknown	333.1356	-2.39372699	-2.245906904	0.047905692	0.28280061	0.118492017	-3.077138235	0.133408979	-2.906072325	2.826987021	1.846242693	0.022253183	0.185329509	4.201751347	2.070990788	4.550435562	2.186004645	-0.695707498	-0.60683229	0.509043081	0.775417468	0.33081166	-1.595918008	0.408920504	-1.290107692	-6.767035132
unknown	316.1999	-7.061016629	-2.526196042	0.000200427	0.022534203	0.144217214	-2.793684719	0.162614626	-2.620471068	3.182197234	1.633480438	0.012954568	0.139170901	3.624076992	1.857613605	3.948727439	1.981387789	-0.226479126	-0.127608255	0.827300165	0.933376377	0.938521024	-0.091539031	1.140924032	0.190202733	-22.46954759
unknown	335.06	-4.396295842	-2.509085765	0.003171072	0.066445461	0.129068865	-2.953787073	0.14580876	-2.777850696	5.324836467	1.636243263	0.000706908	0.040644607	3.068485601	1.617526814	3.274715137	1.711369414	-1.595259522	-0.944275069	0.154684256	0.566017081	0.397533953	-1.33085001	0.487873346	-1.035421428	-23.40955642
unknown	430.1993	-6.692773298	-2.704901202	0.000279288	0.02428314	0.11693669	-3.09620043	0.131912633	-2.922345358	4.18326214	1.513883809	0.003065896	0.071769533	3.19070038	1.67387314	3.387437575	1.76019436	-1.049576397	-1.039149469	0.328792532	0.686230623	0.693289152	-0.528470909	0.843444679	-0.245634649	-27.99762515
unknown	431.2101	-6.073313103	-2.594820617	0.000504189	0.027624042	0.155148639	-2.688277056	0.171413613	-2.544446408	3.183779399	1.559000672	0.012923763	0.139170901	2.897097077	1.534608028	3.189933468	1.673526334	-0.755334705	-0.392876467	0.474691053	0.756466363	0.787571119	-0.344517888	0.951500843	-0.07172316	-19.33608914
unknown	629.3853	-5.260447375	-2.495028886	0.001172771	0.038328302	0.167575709	-2.577115061	0.189169364	-2.40224963	3.540575274	1.619782393	0.007613459	0.103216983	2.890349349	1.531243878	3.085729163	1.625611441	-3.171610484	-0.807744736	0.01567265	0.26754281	0.487267886	-1.037212953	0.591294874	-0.758050325	-18.6250099
unknown	459.2735	2.609817869	1.788682811	0.034922592	0.245666463	2.939306934	1.555476019	3.234727975	1.693644394	-5.165641838	-2.248914749	0.000857705	0.044389646	0.187594642	-2.414309472	0.196802456	-2.345179867	-2.683884157	-1.457363267	0.031362293	0.347221584	0.269230597	-1.893085717	0.318719911	-1.649638943	-13.48138437
unknown	417.2264	-5.208513323	-2.113337941	0.001241348	0.038328302	0.201059069	-2.314308685	0.222812135	-2.166100287	4.570788265	1.897366539	0.001823902	0.059172455	3.777480023	1.917424126	4.085004352	2.030337615	0.391882172	0.192144183	0.706802298	0.880719011	1.095196867	0.131190224	1.307260152	0.386546274	-23.80701157
unknown	398.2029	-4.390020039	-2.389214496	0.003195323	0.066570926	0.188035412	-2.410923708	0.211451966	-2.241598124	2.659224933	1.664616004	0.029123453	0.209836299	3.140143633	1.650830551	3.422698798	1.775134339	-0.519967944	-0.318165391	0.619119889	0.833646914	0.90415675	-0.145355186	1.095158464	0.131139635	-11.64638291
unknown	377.2337	-3.522528612	-2.397119002	0.009694645	0.12495252	0.140563565	-2.830705407	0.161524529	-2.630174824	2.252358354	1.648230916	0.054366856	0.290595304	4.037004554	2.013285214	4.369907621	2.127602782	0.23498228	0.168165233	0.820948346	0.931923674	0.925001795	-0.11247193	1.137854129	0.186315618	-7.933996746
unknown	509.2388	-3.586715059	-2.256013608	0.008896047	0.11766962	0.175836212	-2.507695883	0.197192536	-2.34232315	2.48793642	1.743571125	0.037643104	0.239448539	3.715726371	1.893644264	4.080259226	2.028660812	-0.349949456	-0.364183315	0.736669453	0.897575019	1.285370357	0.362184106	1.566048295	0.647128704	-8.923519024
unknown	432.1882	-3.986279333	-2.050061183	0.005281428	0.087634077	0.181299527	-2.463552931	0.205840517	-2.280401107	4.796731888	1.912958811	0.001361211	0.055133274	4.101496828	2.036150513	4.340931314	2.118004595	0.345857071	0.298241459	0.739611682	0.898976287	1.486069146	0.571501246	1.812332931	0.857848007	-19.12111319
unknown	635.4327	-6.882724922	-2.334194963	0.000234951	0.02428314	0.18687853	-2.419827262	0.207317399	-2.270086894	4.744691056	1.649081319	0.001455148	0.055229968	2.956355857	1.563819938	3.180344759	1.669183166	-1.799868708	-0.663353884	0.114906531	0.523989851	0.599729688	-0.737615704	0.719457541	-0.475018547	-32.65640338
unknown	487.2693	6.725733286	2.263938092	0.000270962	0.02428314	4.42042258	2.144184294	4.916399026	2.297602013	-3.102430669	-1.679576564	0.014611903	0.147912784	0.348101942	-1.522418233	0.370942133	-1.43073395	0.257410014	0.054761019	0.804265133	0.925628827	0.892041441	-0.164817361	1.079187537	0.109945592	-20.86612122
unknown	336.2265	-7.672394401	-2.450932176	0.000118925	0.019884795	0.157710814	-2.66464651	0.176906211	-2.498943391	4.182692917	1.51343299	0.003068286	0.071769533	2.910782903	1.541407242	3.157766616	1.658904549	-0.771096367	-0.36040645	0.465876	0.754027648	0.763160275	-0.389942018	0.923853055	-0.114264695	-32.09126972
unknown	416.2145	-5.898201674	-2.434228491	0.000600569	0.029445934	0.147160949	-2.764533212	0.165977281	-2.59094232	4.309249697	1.51308684	0.002583274	0.068625739	3.051017684	1.609290542	3.231533129	1.692218782	-0.78867874	-0.534205543	0.456175005	0.7482908	0.731212171	-0.451638011	0.889436412	-0.169036627	-25.41682378
unknown	431.1845	-3.714015172	-2.043428698	0.007515005	0.106765467	0.185250804	-2.43244829	0.208824851	-2.259634689	4.899909892	1.779938341	0.001193906	0.052604473	3.496375307	1.805860054	3.744106599	1.904621511	0.238718291	0.215189061	0.818162079	0.931923674	1.516732741	0.600966895	1.848422655	0.886294677	-18.19833968
unknown	347.1869	-6.054039969	-2.420964498	0.000513898	0.027695294	0.146772364	-2.768347747	0.163481483	-2.61280086	6.244541702	1.475924148	0.000247168	0.029691542	2.812679351	1.491945093	3.02705866	1.597916633	-0.633592662	-0.373098501	0.546483052	0.792788356	0.735273553	-0.443647002	0.886059121	-0.174525131	-37.80470505
unknown	314.1848	-4.803573797	-2.207250497	0.001958193	0.049074945	0.205987414	-2.279371906	0.229427828	-2.123887701	2.739019221	1.61081658	0.025487849	0.198573795	3.148112251	1.654486983	3.461712975	1.79148611	0.321528413	0.171140466	0.757197915	0.908054321	1.1305849	0.177069333	1.372936245	0.457264632	-13.15708096
unknown	429.1672	-2.370738736	-1.981123918	0.049552352	0.285863059	0.280564938	-1.833593368	0.312874626	-1.676343434	2.525509245	1.762005387	0.035502847	0.23503966	2.609959209	1.384027259	2.770830242	1.470318325	-0.055466117	-0.032581033	0.957317048	0.988232464	0.81108108	-0.302081954	0.993766519	-0.009021158	-5.987322595
unknown	469.332	-5.837542818	-2.207057291	0.000638633	0.02957347	0.170212453	-2.554591508	0.191958798	-2.38113141	3.626072058	1.581615979	0.006723176	0.096825003	3.36407347	1.750209214	3.655628953	1.870119644	-0.567557048	-0.297100876	0.588067752	0.815912726	0.734949816	-0.444282352	0.897072037	-0.156704253	-21.1673509
unknown	474.3672	2.910262267	1.679149305	0.022652077	0.194873605	2.423981519	1.2773787	2.737388064	1.452799972	-3.907488817	-2.077104336	0.00449693	0.083044401	0.254885558	-1.972078461	0.27265445	-1.874854394	-0.070910259	-0.063892771	0.94545255	0.982937431	1.021198482	0.030263299	1.23103555	0.299872424	-11.37181726
unknown	318.2156	-6.265463747	-2.33870247	0.000417855	0.026388034	0.158399317	-2.658361981	0.178870991	-2.483008662	2.724956951	1.483965	0.026048193	0.199600786	3.469389904	1.794681985	3.786770492	1.920967985	-0.269981211	-0.147551601	0.794960839	0.923395018	0.858266756	-0.220501976	1.047022303	0.066292174	-17.07311899
unknown	320.2215	-6.646795526	-2.39957481	0.000291386	0.024360587	0.15703399	-2.670851227	0.17692024	-2.498828989	3.929084321	1.416353576	0.004362297	0.081386137	2.76875209	1.469235883	2.989599445	1.579952201	-0.649713956	-0.36685475	0.536606733	0.788343521	0.787267861	-0.345073511	0.956087429	-0.064785544	-26.11582009
unknown	470.2991	-5.736271005	-2.402727248	0.000708346	0.030924562	0.18517821	-2.433013746	0.20482374	-2.287545157	2.868309606	1.411644943	0.020883081	0.180499402	2.66850638	1.416032461	2.827386379	1.499469049	-3.295742662	-1.193609421	0.013197794	0.252383616	0.419902506	-1.251873698	0.503401783	-0.990217768	-16.45340123
unknown	533.386	-2.622806221	-2.272828273	0.034269084	0.243608738	0.093793293	-3.414371428	0.107754342	-3.214182092	5.75801599	1.45883282	0.000425074	0.034499987	2.677805428	1.421051137	2.850835548	1.511384819	-0.817926901	-0.864719642	0.440348239	0.738937434	0.368351453	-1.440845162	0.452305716	-1.144629866	-15.10216016
unknown	415.2109	-5.34279343	-2.2557628	0.001072511	0.03720363	0.169166154	-2.563487145	0.189959027	-2.39623982	3.690819362	1.452631004	0.006122975	0.094611384	2.970895427	1.570897824	3.149706365	1.655217338	-0.75640654	-0.489899844	0.474088064	0.755930151	0.737282646	-0.439710296	0.897111754	-0.156640381	-19.71928544
unknown	363.2005	-5.047944487	-3.47242181	0.001483227	0.041563887	0.059798549	-4.063745722	0.067715222	-3.884376011	1.856271091	0.938828653	0.100504477	0.381922076	2.131813031	1.092080913	2.284832907	1.192088663	-1.433601606	-2.238870255	0.1948032	0.594922789	0.565166933	-0.823251036	0.687770377	-0.540001117	-9.37035342
unknown	273.1493	-4.774403683	-2.551567411	0.00202536	0.04982982	0.158729416	-2.655358584	0.177014133	-2.498063543	2.418908075	1.273891411	0.041921448	0.253825223	2.244514624	1.166403496	2.465857628	1.302089505	-1.27015147	-0.508620107	0.24461838	0.634257039	0.611456012	-0.70967938	0.745040079	-0.424610058	-11.54884362
unknown	459.1865	-6.087915537	-2.980500903	0.000496969	0.027463848	0.087533596	-3.514019355	0.098912777	-3.337699297	1.74501165	1.089587772	0.119133251	0.41414368	2.759789068	1.464558005	2.95808773	1.56466484	-0.951398964	-0.873275562	0.373087748	0.7041028	0.586416146	-0.77000327	0.715895534	-0.482179015	-10.62348354
unknown	433.2001	-5.490502488	-1.942459462	0.000915709	0.034935692	0.216044796	-2.210597616	0.243346799	-2.038914296	3.454654691	1.666604844	0.008635433	0.111951608	3.52686581	1.818386685	3.84829737	1.944220285	0.682608736	0.417069529	0.516799686	0.780104341	1.44427541	0.530345878	1.752112145	0.809095119	-18.96779018
unknown	393.2286	-8.584901214	-2.188627095	5.79E-05	0.016446553	0.187082051	-2.418256948	0.209000085	-2.258424567	6.417825279	1.477491964	0.000205202	0.028695533	2.808409949	1.489753544	3.004863099	1.587299263	-0.455024393	-0.225996822	0.662868241	0.857028302	0.871153583	-0.199001008	1.052780583	0.074204785	-55.09639603
unknown	394.2317	-8.588163051	-2.179160451	5.78E-05	0.016446553	0.18887936	-2.404463038	0.210890523	-2.245433828	6.590522869	1.476486149	0.000171069	0.028695533	2.792698565	1.481659862	2.989591384	1.579948311	-0.408590563	-0.204385049	0.695050468	0.873973024	0.888137292	-0.171145384	1.07302465	0.101683218	-56.60048499
unknown	377.2334	-6.589418485	-2.289739949	0.000307317	0.024439191	0.169468953	-2.560907103	0.19118555	-2.386954605	4.116069505	1.403612178	0.003362371	0.073824984	2.731151241	1.449509207	2.945716991	1.55861883	-0.57701476	-0.305254943	0.58200273	0.812511197	0.790278376	-0.339567162	0.961220266	-0.057061028	-27.12250448
unknown	379.1896	-3.822066338	-1.928154336	0.006524706	0.098870669	0.217275401	-2.202403244	0.245374087	-2.026945197	3.790508584	1.660732234	0.005307978	0.087859906	3.154210272	1.657278839	3.41891037	1.773536602	0.299568133	0.145071793	0.773206038	0.915252085	0.919402807	-0.121231025	1.122883219	0.167207894	-14.48757526
unknown	318.2157	-4.51963892	-2.170250068	0.002733331	0.059871995	0.188451174	-2.40773731	0.214878222	-2.21840882	2.803939589	1.458038675	0.023057378	0.189633969	2.88833347	1.530237317	3.075587314	1.620861934	-0.568280664	-0.39017309	0.587602449	0.81544064	0.804072239	-0.314602973	0.979426034	-0.02999155	-12.67279449
unknown	334.2109	-8.389490912	-2.150517855	6.72E-05	0.016446553	0.192951848	-2.373687236	0.215311387	-2.215503473	6.39712229	1.458163077	0.000209775	0.028695533	2.753796733	1.461422073	2.950852339	1.56113173	-0.467348286	-0.231984672	0.654449847	0.85285121	0.869933815	-0.201022451	1.05071156	0.071366677	-53.66859932
unknown	144.1016	3.043234788	1.887639962	0.018761184	0.176086663	4.409882481	2.14074021	4.88233156	2.287570272	-2.813605079	-1.658536642	0.022716538	0.18759541	0.250102029	-1.999411333	0.263984257	-1.9214762	-0.727107004	-0.200389413	0.490756504	0.766064218	0.771510717	-0.374241898	0.924094919	-0.113887049	-8.562460856
unknown	317.2124	-6.792555593	-2.265793126	0.000254926	0.02428314	0.174369961	-2.519776573	0.196472129	-2.347603423	4.133983972	1.376635071	0.003280419	0.073261239	2.660963408	1.411948671	2.868909466	1.520502442	-0.500278654	-0.262580477	0.63222069	0.839541926	0.829578804	-0.269549061	1.007597395	0.010919298	-28.08031595
unknown	318.2156	-6.773645238	-2.265866483	0.000259353	0.02428314	0.174413698	-2.519414746	0.196512281	-2.347308619	4.079079513	1.374711914	0.003538648	0.074959362	2.661011112	1.411974535	2.869393118	1.520745637	-0.519756383	-0.27183327	0.619259883	0.833646914	0.822747511	-0.281478338	0.999237304	-0.001100757	-27.63023752
unknown	350.2055	-7.29179483	-2.692911372	0.000163914	0.021377633	0.123155203	-3.021450515	0.137857251	-2.858752938	5.008410395	1.154762089	0.001041828	0.050324128	2.212893454	1.14593399	2.396931022	1.261188392	-1.162352508	-0.768027256	0.283184125	0.659025359	0.642174887	-0.638961847	0.776367922	-0.365187586	-36.52030103
unknown	313.1815	-8.101234946	-2.099484155	8.41E-05	0.017507673	0.190789052	-2.389949709	0.214283269	-2.222408884	2.822254082	1.475370549	0.022415936	0.185972418	3.412717146	1.770920846	3.740405726	1.903194769	0.457415679	0.244764604	0.661230624	0.85615982	1.17047808	0.227097916	1.425198187	0.511162553	-22.8637434
unknown	433.221	-5.879275837	-2.073236921	0.000612166	0.029500467	0.204767999	-2.287937825	0.225856993	-2.146518513	5.356213854	1.476786819	0.000680756	0.040614693	2.707866865	1.437156809	2.92685248	1.549350033	-1.403689956	-0.587820423	0.203185042	0.6034824	0.606386633	-0.721690142	0.728916505	-0.456174527	-31.49065869
unknown	399.2155	-6.258079164	-2.149534614	0.000420849	0.026388034	0.188017661	-2.411059913	0.211654044	-2.240220039	2.690635873	1.414414631	0.027469563	0.204252018	3.176720304	1.667538073	3.466023285	1.793281347	-0.034469467	-0.016561233	0.973465105	0.992551009	0.93521256	-0.096633789	1.137642846	0.186047705	-16.83821229
unknown	319.2189	-6.697928946	-2.270414956	0.000277967	0.02428314	0.169706206	-2.558889769	0.191338615	-2.385800034	4.321505272	1.337406424	0.002540907	0.068025432	2.607108851	1.382450819	2.808406593	1.48975182	-0.517345155	-0.266254851	0.620856637	0.834109436	0.795616962	-0.32985406	0.968545768	-0.04610787	-28.94513525
unknown	317.2122	-4.553071128	-2.050619688	0.002626477	0.058720754	0.172544009	-2.534963716	0.1959001	-2.351809959	5.689589974	1.450095326	0.00045987	0.035044854	2.87887488	1.525505089	3.07298705	1.619641687	-0.272542632	-0.192615737	0.793069382	0.923171267	0.832704348	-0.264123737	1.021448838	0.030616944	-25.90510784
unknown	413.2003	-6.001171581	-2.280018106	0.000541625	0.028255504	0.162377519	-2.622576191	0.183487188	-2.446248766	2.180390596	1.302721326	0.060825411	0.304640942	3.292934281	1.71937372	3.599915499	1.847963043	-0.053035888	-0.029046225	0.959185139	0.98857673	0.914169481	-0.129466439	1.116447164	0.158914977	-13.08489808
unknown	318.2152	-5.84351119	-2.387733736	0.000634771	0.02957347	0.154492614	-2.694390232	0.175325696	-2.511890637	3.397904987	1.235223459	0.009389512	0.116849249	2.431273191	1.281712011	2.624432685	1.392005594	-0.829854642	-0.445879377	0.434005766	0.736181591	0.685494524	-0.544782953	0.835821087	-0.258733938	-19.85569581
unknown	471.3488	-3.648490988	-1.923422035	0.008194302	0.11202315	0.224459868	-2.155470569	0.25103066	-1.994064516	2.981818155	1.514260013	0.017551649	0.164919746	2.863190851	1.517623839	3.135755641	1.64881314	-0.905451044	-0.489166711	0.3953217	0.711471454	0.63016009	-0.666209708	0.77033717	-0.376438055	-10.87913667
unknown	534.3884	-2.481997812	-2.203581264	0.0420868	0.266102627	0.122278098	-3.031762074	0.141480748	-2.821322339	2.905156361	1.304869339	0.019735071	0.175569435	2.163521036	1.113381148	2.270290582	1.182876965	-0.954302309	-1.017429833	0.371715219	0.7041028	0.383468689	-1.382819311	0.470323684	-1.088274113	-7.210591732
unknown	522.352	-4.606646742	-2.841112188	0.002464725	0.056394626	0.094620096	-3.401709562	0.107280815	-3.220535994	2.011145195	1.007368446	0.079137592	0.341897362	2.339022132	1.225905513	2.442583689	1.288407993	-1.384314744	-1.205881913	0.20878451	0.606699715	0.4568242	-1.130289015	0.556461593	-0.845645978	-9.26463546
unknown	647.3954	-3.430671283	-1.975700677	0.010975207	0.135805171	0.262124957	-1.931673376	0.295261743	-1.759933656	2.949099096	1.429601353	0.018451087	0.169225789	2.205792536	1.141297106	2.341590089	1.227488545	-1.501750386	-0.476836825	0.17685697	0.580707101	0.619429423	-0.690988182	0.749807662	-0.415407527	-10.11738958
unknown	316.1997	-4.265563092	-2.110757423	0.003720971	0.072866223	0.207499821	-2.268818001	0.23629862	-2.081316892	3.191088363	1.33671124	0.012782453	0.139170901	2.340538425	1.22684045	2.498267459	1.320927937	-0.917918649	-0.373065548	0.389193259	0.708203131	0.648122377	-0.62566185	0.79221061	-0.336044072	-13.61178874
unknown	507.1702	-3.427671504	-2.2549119	0.011019989	0.13610104	0.146713183	-2.768929588	0.162528319	-2.621236979	2.996014639	1.22737554	0.017175705	0.163507693	2.396714731	1.261058202	2.540652876	1.345199276	-0.643089509	-0.512344515	0.540651689	0.790338304	0.62509311	-0.677856994	0.749464995	-0.416066998	-10.269354
unknown	477.2491	-2.40280816	-1.805662945	0.04727073	0.281705789	0.223569216	-2.161206543	0.254382426	-1.974929087	2.244176108	1.515273055	0.055065461	0.291226172	3.005740291	1.587720359	3.22547506	1.689511661	-0.301537935	-0.300139062	0.771765153	0.91446756	1.107649752	0.147501761	1.353369108	0.436555363	-5.392324665
unknown	445.2036	-6.431480561	-3.126047555	0.000356464	0.024987604	0.090680759	-3.463059731	0.101357337	-3.302477565	1.274080024	0.873241066	0.238399092	0.559990186	2.458268136	1.297642286	2.628128067	1.394035579	-1.437586118	-1.648301573	0.193710357	0.593881167	0.5335416	-0.906327333	0.648450387	-0.624931897	-8.194220908
unknown	592.3244	-2.536346248	-2.754486032	0.038870892	0.258914285	0.171193367	-2.546301293	0.188289381	-2.408976454	1.164507555	0.98928049	0.27776723	0.596220236	1.165237545	0.220624092	1.288125912	0.365273621	-1.300129969	-1.707164886	0.234725704	0.627057074	0.568125955	-0.815717279	0.688870105	-0.537696123	-2.953594369
unknown	317.2124	-5.405155485	-2.18351726	0.001002926	0.035647699	0.178359702	-2.487138402	0.201501383	-2.311138352	3.293157959	1.216156263	0.010970227	0.128174698	2.410010426	1.269039388	2.60428098	1.380885112	-0.882169383	-0.489205177	0.406956389	0.716458588	0.685252461	-0.54529249	0.835022732	-0.260112622	-17.80003081
unknown	494.2489	-3.124124913	-2.088811101	0.016746763	0.166472013	0.244449283	-2.03239292	0.274392519	-1.865686948	1.952011523	1.264796888	0.086722223	0.356567222	2.064475201	1.045775089	2.20300218	1.139470923	-2.234573567	-1.541412025	0.060560804	0.426016182	0.364631053	-1.455490665	0.442569098	-1.176025374	-6.09832783
unknown	318.2155	-3.703487768	-1.907467006	0.007619892	0.107552635	0.187090842	-2.418189152	0.213120173	-2.230260934	4.579536395	1.378538638	0.001803104	0.059172455	2.75095673	1.459933447	2.924176426	1.548030357	-0.254725514	-0.192511189	0.806256483	0.926612359	0.82834646	-0.271693786	1.017616329	0.025193725	-16.96025702

unknown	318.2156	-3.65786455	-1.927573712	0.00809317	0.111106439	0.227855753	-2.133807301	0.256824142	-1.961147267	3.060130472	1.362715731	0.015579511	0.1535229	2.440935171	1.287433979	2.632723368	1.396555939	-0.301422829	-0.177064672	0.771849325	0.91446756	0.859624622	-0.218221289	1.047756128	0.06730296	-11.19354277
unknown	297.1865	-4.810241117	-1.833266475	0.001943189	0.049074945	0.234481975	-2.092451072	0.264100666	-1.920840158	3.405434364	1.421993269	0.009285574	0.116221162	2.832665366	1.502160181	3.065470026	1.616108298	0.09516216	0.033782371	0.92685291	0.974211448	0.844970457	-0.243027194	1.029845005	0.042427224	-16.3809604
unknown	381.2046	-4.115890784	-2.105875483	0.004482626	0.080085483	0.183767838	-2.444043795	0.207122065	-2.271446839	2.455747159	1.229332715	0.039580383	0.245813029	2.454351692	1.295341992	2.688747028	1.426934025	-0.05148254	-0.033012757	0.960379326	0.98857673	0.944038066	-0.083083061	1.152525642	0.204798849	-10.1075871
unknown	415.2106	-4.337662548	-1.920743724	0.003405786	0.068993027	0.234088715	-2.09487271	0.260721288	-1.939419711	3.130637632	1.334737622	0.01400179	0.14516005	2.5701815	1.361870243	2.717183033	1.442111751	-0.602958158	-0.357110306	0.565551565	0.803302495	0.82684037	-0.274319265	0.99984851	-0.000218571	-13.57964961
unknown	379.2475	2.855609578	0.951298042	0.024490982	0.204004403	1.771179068	0.824710077	1.988425267	0.991626341	-6.311748611	-2.647035704	0.000229861	0.028825404	0.172136858	-2.538372055	0.178659549	-2.484715073	-3.366809371	-2.125715613	0.011972382	0.241708679	0.291520676	-1.778329885	0.349554836	-1.5164093	-18.02388979
unknown	217.0638	-3.675827284	-1.720296761	0.007903129	0.109418909	0.237505676	-2.073966105	0.263316553	-1.925129875	3.272469144	1.458347899	0.011314386	0.130586037	3.025057774	1.596962696	3.215579774	1.685078881	-1.681528858	-0.686351399	0.136543833	0.550207717	0.464047435	-1.107655811	0.557019542	-0.844200153	-12.02903137
unknown	301.2162	-3.854563174	-2.197197767	0.006255364	0.097116206	0.198228345	-2.334764827	0.225681666	-2.147638877	1.857066899	1.134958394	0.100381742	0.381907434	2.179334201	1.123887451	2.316177216	1.211745641	-0.155217669	-0.099023712	0.881030692	0.952295896	0.949040343	-0.075458679	1.157191846	0.210628063	-7.158181681
unknown	475.2337	-2.730819569	-1.616425274	0.029303984	0.225609542	0.251402215	-1.991930736	0.28421911	-1.814924537	2.865122553	1.539848343	0.020985576	0.180499402	3.156353895	1.658258972	3.403100533	1.766849771	-0.259363891	-0.158214437	0.802816735	0.92550737	0.70218004	-0.510087108	0.86359559	-0.211572218	-7.824132736
unknown	298.1897	-4.65083419	-1.767637372	0.002339577	0.054315343	0.24146776	-2.050097515	0.272585034	-1.875221743	3.422145045	1.395148978	0.009059219	0.11470906	2.805171094	1.488088767	3.029391668	1.599028115	0.130621975	0.047738406	0.899748989	0.961050756	0.849014087	-0.236139603	1.036113077	0.051181461	-15.91582918
unknown	306.1774	-2.526951455	-1.88195962	0.039408105	0.259314079	0.318292984	-1.651572737	0.351843656	-1.506993595	1.95519794	1.282371355	0.086296233	0.355998299	1.786575756	0.83719709	1.933051377	0.950879983	-0.685002223	-0.200508935	0.51537676	0.778574303	0.781710199	-0.355294235	0.941985822	-0.086222749	-4.940690279
unknown	418.2306	-6.524285559	-1.784051561	0.000326599	0.024987604	0.241856174	-2.04777873	0.267400979	-1.902923345	3.601829458	1.35259254	0.006963773	0.098504915	2.782601117	1.476434115	3.03107849	1.599831211	0.17919735	0.08656641	0.862859285	0.944236515	1.026674764	0.037979229	1.228623137	0.297042458	-23.49936392
unknown	649.4111	-3.020672811	-1.774346522	0.019367906	0.176888114	0.28240325	-1.824171405	0.318437693	-1.650916979	2.829485356	1.346733704	0.022167765	0.185032826	2.177105096	1.122411053	2.308823578	1.207157938	0.051282202	0.018471661	0.96053335	0.988579226	0.827388756	-0.273362742	1.006744414	0.009697468	-8.546949484
unknown	351.218	-3.5217185	-1.961325631	0.009705207	0.12495252	0.182022756	-2.45780927	0.201000159	-2.314731454	2.886076553	1.200037988	0.020321125	0.178173227	2.462045657	1.299857516	2.68721909	1.42611395	-0.465191221	-0.325420518	0.655919489	0.853123753	0.678811294	-0.558917527	0.810565323	-0.302999639	-10.16394919
unknown	423.2219	-5.690187495	-3.03606325	0.000742852	0.031393339	0.089160295	-3.487454803	0.100336317	-3.317084213	1.197878399	0.774654512	0.265247159	0.587925468	2.293981714	1.197853891	2.440445749	1.287144681	-1.5057495	-1.689518751	0.175851993	0.58055525	0.451735622	-1.146449412	0.55117706	-0.85941225	-6.816152685
unknown	273.1494	-5.213148478	-2.305039219	0.00123505	0.038328302	0.182843293	-2.451320384	0.20481912	-2.287577695	2.438773486	1.019493278	0.040642188	0.249555281	1.93003478	0.948626846	2.096601499	1.068052674	-2.991923495	-0.848317255	0.020171345	0.303668884	0.461861386	-1.114468162	0.562654902	-0.829677763	-12.71368829
unknown	299.1924	-3.315643584	-1.778702855	0.012841611	0.146823641	0.245425403	-2.026643512	0.27535775	-1.860620884	2.456147852	1.312583126	0.039555659	0.245813029	2.583204097	1.369161634	2.823680203	1.497576705	0.510639109	0.221999834	0.625309003	0.836279273	0.920501041	-0.119508742	1.126188863	0.171448789	-8.143710866
unknown	470.3353	-3.031490742	-1.828390956	0.019074438	0.17651305	0.274631063	-1.864433282	0.311388425	-1.683212778	2.053631141	1.274643294	0.074089244	0.331824149	2.246187471	1.167478343	2.415977274	1.272606884	-0.789375914	-0.35900451	0.455793228	0.74810663	0.72730425	-0.459369087	0.884046354	-0.177806078	-6.225563792
unknown	400.2191	-5.208083183	-1.86827183	0.001241935	0.038328302	0.21570144	-2.212892284	0.242907526	-2.041520905	3.23115978	1.246672704	0.012036041	0.134165849	2.626691532	1.393246786	2.852845505	1.51240162	-0.22961648	-0.116421541	0.824954932	0.93329825	0.832853079	-0.263866078	1.016314586	0.023347037	-16.82814891
unknown	429.1955	-5.219784614	-1.959561167	0.001226094	0.038328302	0.214995211	-2.217623568	0.240806271	-2.054055133	3.67367298	1.181289738	0.006276143	0.095068095	2.332973102	1.222169674	2.472104358	1.305739647	-0.754348216	-0.434194295	0.475246486	0.757166463	0.768566735	-0.379757559	0.930463865	-0.103977971	-19.1757817
unknown	364.2036	-5.988010152	-3.089829403	0.000548785	0.028401827	0.083437443	-3.583161237	0.094037265	-3.410623603	1.12628127	0.732539706	0.292695375	0.609019317	2.328908218	1.219653784	2.478490346	1.309461639	-1.733316821	-2.850512541	0.126636521	0.537956721	0.419748472	-1.252403021	0.510794174	-0.969186025	-6.744183676
unknown	362.1976	-3.369554709	-2.745751738	0.011927566	0.142150548	0.08589879	-3.541218384	0.098336382	-3.34613092	1.077657199	0.823511922	0.312606228	0.626241669	2.981131942	1.575860229	3.248762682	1.699890361	-3.836743472	-3.022450445	0.006401526	0.200230902	0.068873294	-3.859911512	0.085621423	-3.545884384	-3.631224889
unknown	375.2173	-5.584616274	-1.975653533	0.000829204	0.03418599	0.206814597	-2.273590083	0.233154499	-2.100641826	2.321848538	1.14082656	0.048778465	0.275335955	2.556223334	1.354013888	2.780793859	1.475496801	-0.293309906	-0.159049909	0.777790194	0.916550662	0.879514605	-0.18522056	1.0704156	0.098171047	-12.96663313
unknown	380.2083	-4.175400169	-2.330597932	0.004161034	0.077748142	0.132642604	-2.914383862	0.151356956	-2.723973117	1.416010357	0.962171637	0.194507006	0.513930383	3.076175847	1.621137976	3.332872168	1.736765984	-2.675599007	-1.445018185	0.031741038	0.349633971	0.23871794	-2.066621101	0.295671394	-1.757933426	-5.912409884
unknown	364.2038	-3.431644855	-2.733083205	0.010960715	0.135805171	0.12440256	-3.006911924	0.144159083	-2.794266358	1.123019784	0.819563022	0.293998401	0.609590962	1.772065072	0.825431582	1.878952281	0.909928428	-0.539888883	-0.556194408	0.606014316	0.825359096	0.748387149	-0.41814331	0.912977444	-0.131348877	-3.853805063
unknown	631.4007	-5.426377103	-1.951874766	0.000980405	0.035647699	0.226437763	-2.142813521	0.255395694	-1.969193893	3.66013038	1.146009991	0.006400007	0.09572121	2.158870589	1.110276765	2.307651157	1.206425151	-1.109847022	-0.308270382	0.303730498	0.673224534	0.673379951	-0.570507328	0.819649264	-0.286921396	-19.86124768
unknown	632.4045	-5.392355645	-1.949509869	0.001016788	0.035647699	0.22544032	-2.149182531	0.254395646	-1.974854118	3.706640082	1.146997793	0.005985182	0.094523898	2.163931048	1.113654529	2.31319166	1.209884806	-1.084799722	-0.307210956	0.313951927	0.677273074	0.671405198	-0.574744389	0.817609094	-0.290516852	-19.98752157
unknown	322.2101	-6.743125162	-1.631826524	0.000266682	0.02428314	0.293305064	-1.769526113	0.325438344	-1.61954385	5.920648132	1.362917519	0.000353434	0.033402052	2.467092921	1.302812055	2.645874768	1.403744779	-3.198106239	-0.461203859	0.015105499	0.265286825	0.635843461	-0.653256465	0.765666367	-0.385212209	-39.92367139
unknown	315.1967	-7.801126412	-2.090625942	0.000107005	0.019884795	0.198219693	-2.334827792	0.222536497	-2.167886129	4.489987813	1.044675736	0.002028784	0.061338495	2.104824837	1.073700178	2.239431998	1.163132858	-2.355284957	-0.960255372	0.050691727	0.397786703	0.47862862	-1.063021427	0.580563982	-0.784473023	-35.02696252
unknown	461.1816	-2.460124053	-1.946255334	0.043457418	0.269122341	0.175334014	-2.511822193	0.19823567	-2.334711512	[illegible]	[illegible]	[illegible]	[illegible]	[illegible]	[illegible]	[illegible]	[illegible]	[illegible]	[illegible]	[illegible]	[illegible]	[illegible]	[illegible]	[illegible]	[illegible]	[illegible]
unknown	352.2213	-2.750464789	-1.657674655	0.028484874	0.222250576	0.236590519	-2.079535831	0.259336375	-1.94710352	[illegible]	[illegible]	[illegible]	[illegible]	[illegible]	[illegible]	[illegible]	[illegible]	[illegible]	[illegible]	[illegible]	[illegible]	[illegible]	[illegible]	[illegible]	[illegible]	[illegible]
unknown	643.4252	7.563074342	1.922673022	0.000130231	0.020000824	3.388200711	1.76051934	3.719251547	1.895012326	[illegible]	[illegible]	[illegible]	[illegible]	[illegible]	[illegible]	[illegible]	[illegible]	[illegible]	[illegible]	[illegible]	[illegible]	[illegible]	[illegible]	[illegible]	[illegible]	[illegible]
unknown	352.2213	-4.560734493	-1.755616719	0.002602638	0.058720754	0.276578626	-1.854238428	0.309167089	-1.693541344	[illegible]	[illegible]	[illegible]	[illegible]	[illegible]	[illegible]	[illegible]	[illegible]	[illegible]	[illegible]	[illegible]	[illegible]	[illegible]	[illegible]	[illegible]	[illegible]	[illegible]
unknown	457.2602	-4.233090236	-1.539720995	0.003873282	0.074726838	0.299005781	-1.741754717	0.337087367	-1.568805534	[illegible]	[illegible]	[illegible]	[illegible]	[illegible]	[illegible]	[illegible]	[illegible]	[illegible]	[illegible]	[illegible]	[illegible]	[illegible]	[illegible]	[illegible]	[illegible]	[illegible]
unknown	481.2182	-6.155036004	-1.749992144	0.000465239	0.026405285	0.248612553	-2.008028951	0.278671173	-1.843364326	[illegible]	[illegible]	[illegible]	[illegible]	[illegible]	[illegible]	[illegible]	[illegible]	[illegible]	[illegible]	[illegible]	[illegible]	[illegible]	[illegible]	[illegible]	[illegible]	[illegible]
unknown	379.213	4.499809392	1.582082513	0.002798969	0.060840261	2.805885783	1.488456284	3.158412113	1.659199428	[illegible]	[illegible]	[illegible]	[illegible]	[illegible]	[illegible]	[illegible]	[illegible]	[illegible]	[illegible]	[illegible]	[illegible]	[illegible]	[illegible]	[illegible]	[illegible]	[illegible]
unknown	110.9758	2.829653007	1.500113008	0.025418836	0.207454607	2.524239648	1.335848884	2.811846478	1.491517828	[illegible]	[illegible]	[illegible]	[illegible]	[illegible]	[illegible]	[illegible]	[illegible]	[illegible]	[illegible]	[illegible]	[illegible]	[illegible]	[illegible]	[illegible]	[illegible]	[illegible]
unknown	593.3857	-4.011359905	-1.551372147	0.005115456	0.085752919	0.313788944	-1.672133571	0.35030244	-1.513327057	[illegible]	[illegible]	[illegible]	[illegible]	[illegible]	[illegible]	[illegible]	[illegible]	[illegible]	[illegible]	[illegible]	[illegible]	[illegible]	[illegible]	[illegible]	[illegible]	[illegible]
unknown	329.1762	-9.064752906	-1.864057412	4.07E-05	0.016446553	0.233609967	-2.097826266	0.262065104	-1.932002832	[illegible]	[illegible]	[illegible]	[illegible]	[illegible]	[illegible]	[illegible]	[illegible]	[illegible]	[illegible]	[illegible]	[illegible]	[illegible]	[illegible]	[illegible]	[illegible]	[illegible]
unknown	441.2527	2.361576951	1.67890808	0.050224637	0.286849409	2.765779313	1.467686045	3.015191911	1.59224983	[illegible]	[illegible]	[illegible]	[illegible]	[illegible]	[illegible]	[illegible]	[illegible]	[illegible]	[illegible]	[illegible]	[illegible]	[illegible]	[illegible]	[illegible]	[illegible]	[illegible]
unknown	330.1795	-9.058420492	-1.848736524	4.09E-05	0.016446553	0.236021363	-2.083010646	0.264331065	-1.919582109	[illegible]	[illegible]	[illegible]	[illegible]	[illegible]	[illegible]	[illegible]	[illegible]	[illegible]	[illegible]	[illegible]	[illegible]	[illegible]	[illegible]	[illegible]	[illegible]	[illegible]
unknown	410.2218	-3.560416883	-2.271309739	0.009214296	0.120898238	0.166411012	-2.587177187	0.185387167	-2.431386715	[illegible]	[illegible]	[illegible]	[illegible]	[illegible]	[illegible]	[illegible]	[illegible]	[illegible]	[illegible]	[illegible]	[illegible]	[illegible]	[illegible]	[illegible]	[illegible]	[illegible]
unknown	417.2266	-8.425919452	-1.634929666	6.54E-05	0.016446553	0.288273092	-1.794491916	0.318725558	-1.649613383	[illegible]	[illegible]	[illegible]	[illegible]	[illegible]	[illegible]	[illegible]	[illegible]	[illegible]	[illegible]	[illegible]	[illegible]	[illegible]	[illegible]	[illegible]	[illegible]	[illegible]
unknown	301.1802	-5.165368831	-1.973507524	0.00130172	0.039665955	0.215919382	-2.21143534	0.241916655	-2.047417998	[illegible]	[illegible]	[illegible]	[illegible]	[illegible]	[illegible]	[illegible]	[illegible]	[illegible]	[illegible]	[illegible]	[illegible]	[illegible]	[illegible]	[illegible]	[illegible]	[illegible]
unknown	378.2377	-5.26248884	-1.690477174	0.001170163	0.038328302	0.25628751	-1.964164923	0.288152123	-1.795097446	[illegible]	[illegible]	[illegible]	[illegible]	[illegible]	[illegible]	[illegible]	[illegible]	[illegible]	[illegible]	[illegible]	[illegible]	[illegible]	[illegible]	[illegible]	[illegible]	[illegible]
unknown	413.2003	-6.453074783	-1.727562607	0.000349251	0.024987604	0.26226777	-1.930887571	0.294097305	-1.765634531	[illegible]	[illegible]	[illegible]	[illegible]	[illegible]	[illegible]	[illegible]	[illegible]	[illegible]	[illegible]	[illegible]	[illegible]	[illegible]	[illegible]	[illegible]	[illegible]	[illegible]
unknown	418.2307	-4.047498139	-1.603842859	0.004886252	0.083630577	0.301374538	-1.73037056	0.337736535	-1.566029844	[illegible]	[illegible]	[illegible]	[illegible]	[illegible]	[illegible]	[illegible]	[illegible]	[illegible]	[illegible]	[illegible]	[illegible]	[illegible]	[illegible]	[illegible]	[illegible]	[illegible]
unknown	414.2027	-8.261157393	-1.806832446	7.42E-05	0.017281626	0.250351867	-1.99797088	0.279371273	-1.839744414	[illegible]	[illegible]	[illegible]	[illegible]	[illegible]	[illegible]	[illegible]	[illegible]	[illegible]	[illegible]	[illegible]	[illegible]	[illegible]	[illegible]	[illegible]	[illegible]	[illegible]
unknown	334.2109	-6.825733441	-1.740480658	0.000247362	0.02428314	0.259247191	-1.947599735	0.28914511	-1.790134393	[illegible]	[illegible]	[illegible]	[illegible]	[illegible]	[illegible]	[illegible]	[illegible]	[illegible]	[illegible]	[illegible]	[illegible]	[illegible]	[illegible]	[illegible]	[illegible]	[illegible]
unknown	301.1117	2.974227927	1.53679397	0.02068338	0.18540303	2.517856886	1.332196283	2.711323246	1.438997122	[illegible]	[illegible]	[illegible]	[illegible]	[illegible]	[illegible]	[illegible]	[illegible]	[illegible]	[illegible]	[illegible]	[illegible]	[illegible]	[illegible]	[illegible]	[illegible]	[illegible]
unknown	400.2192	-8.145581842	-1.506247156	8.12E-05	0.017507673	0.314813092	-1.667432558	0.350077031	-1.514255688	[illegible]	[illegible]	[illegible]	[illegible]	[illegible]	[illegible]	[illegible]	[illegible]	[illegible]	[illegible]	[illegible]	[illegible]	[illegible]	[illegible]	[illegible]	[illegible]	[illegible]

unknown	347.1869	-6.154098508	-1.266529206	0.000465666	0.026405285	0.376930842	-1.407628249	0.413751043	-1.273165145	3.004475791	0.664809517	0.016955639	0.162122762	1.576446948	0.65667662	1.684316375	0.752163154	-2.36002024	-0.403603257	0.050339789	0.396410444	0.677431946	-0.561852072	0.807341041	-0.308749863	-18.48983998
unknown	332.1952	-3.00157094	-1.189519767	0.019897863	0.180967873	0.412488115	-1.277575542	0.453629676	-1.140413072	1.410186115	0.702667459	0.196159594	0.515788996	1.714859322	0.77809023	1.822334284	0.865787628	1.831734065	0.418829172	0.10966621	0.514235711	1.176099014	0.234009524	1.393087389	0.478285762	-4.232773662
unknown	392.2169	-4.915591762	-1.322452937	0.001722258	0.046408457	0.345712884	-1.532353723	0.386449149	-1.371649506	3.55951206	0.631617561	0.007405986	0.101989132	1.502311547	0.587184027	1.609294405	0.686428277	-2.634015699	-0.605140052	0.033715304	0.353324531	0.556089691	-0.846610502	0.672139483	-0.573167442	-17.49710816
unknown	301.1802	-3.417244054	-0.894928677	0.011177193	0.13726266	0.467674717	-1.096422658	0.521886717	-0.938191413	2.9934864	0.929644329	0.017242037	0.16389989	1.986437406	0.990183334	2.138920564	1.096882904	-0.614583422	-0.130628994	0.558269359	0.798945963	0.774358071	-0.368927257	0.936068726	-0.095313638	-10.2294736
unknown	309.0679	-2.854866088	-0.972300404	0.024517057	0.204004403	0.429409834	-1.219572863	0.472255714	-1.082359844	3.879076129	0.853942991	0.004680893	0.084796192	1.794176286	0.843321649	1.927010983	0.946364794	-1.347022343	-0.498100119	0.219947841	0.617273899	0.610358881	-0.712270323	0.726751404	-0.460466142	-11.07424289
unknown	352.2211	-3.034908827	-1.093315588	0.018982696	0.176459747	0.371112027	-1.430073338	0.415868547	-1.265800519	4.949650322	0.759082933	0.001121377	0.051496456	1.680461371	0.74885738	1.794704659	0.84374645	-0.976025448	-0.339214124	0.361567534	0.699757782	0.613681548	-0.704437888	0.747546835	-0.419764126	-15.02173745
unknown	403.2339	2.710031376	1.03293875	0.030197643	0.229352204	1.582929965	0.662597427	1.747980406	0.805689013	-4.272050707	-0.802708873	0.002716614	0.070577854	0.585835041	-0.771433607	0.624836264	-0.678449908	0.504598272	0.223258748	0.629334129	0.838202521	0.926788441	-0.109688044	1.11188528	0.153007944	-11.57739146
unknown	508.3387	2.670168924	1.787565308	0.031991854	0.237877853	2.860934619	1.516486528	3.108294352	1.636123132	-1.169204087	-0.463365999	0.275976541	0.595446358	0.640481286	-0.642771678	0.679727288	-0.556972053	1.081752359	0.693423173	0.315214243	0.677394009	1.271686256	0.34674278	1.489981653	0.575294566	-3.12197242
unknown	569.2634	-3.153024225	-1.018282255	0.016084045	0.163252634	0.439509359	-1.186034208	0.491468788	-1.024828296	2.431923044	0.809019489	0.041078824	0.251289879	1.711503183	0.775263975	1.845162418	0.883747813	-0.604252549	-0.128558299	0.564737979	0.803021447	0.781327235	-0.356001191	0.94590492	-0.08023292	-7.667912269
unknown	318.2156	-4.878831789	-1.014454017	0.001796017	0.047235692	0.449503123	-1.153596956	0.496162379	-1.011115748	3.499678152	0.80782262	0.008082894	0.106866089	1.7129618	0.776492979	1.839763419	0.879520257	-1.101846015	-0.216921028	0.306965832	0.675038254	0.768181138	-0.380481554	0.91853711	-0.122590086	-17.07434102
unknown	430.3048	3.991729837	1.425053979	0.005244865	0.087249393	2.272116241	1.184036645	2.450050961	1.292811758	-1.227691128	-0.568610244	0.254458415	0.576233732	0.771352379	-0.374538014	0.816996793	-0.291597679	-1.006694091	-0.407110529	0.347605393	0.694678138	0.64318874	-0.636685944	0.75912856	-0.397583866	-4.900611308
unknown	311.1691	2.584664905	1.964090302	0.036225236	0.251035882	2.648073904	1.404943386	2.927288676	1.549565025	-1.405134448	-0.410963673	0.197602907	0.517082085	0.709623254	-0.494874807	0.763521615	-0.389259096	1.172489105	0.82960748	0.279352836	0.659025359	1.114089228	0.155864784	1.34055507	0.422830486	-3.631801695
unknown	473.2178	5.034929643	1.856340837	0.001505017	0.041941104	3.058245361	1.612704158	3.362144175	1.749381592	-1.638834101	-0.433852368	0.13988042	0.444622523	0.733914301	-0.446316485	0.782270556	-0.354260431	1.980368597	1.039891868	0.088133898	0.480589586	1.83498455	0.875767916	2.18875702	1.130111806	-8.251414398
unknown	481.2182	-4.734005396	-0.833345473	0.002122629	0.051648011	0.502539261	-0.992691784	0.550680662	-0.860712148	4.178134782	0.962237771	0.003087495	0.071769533	1.968035662	0.976756363	2.11847802	1.08302816	1.977531089	0.328836293	0.088503315	0.481744675	1.096846928	0.133362203	1.305194814	0.38426516	-19.77931261
unknown	133.9917	2.534135314	1.167970863	0.038996632	0.259183683	1.675409713	0.744513942	1.870711028	0.90358672	-2.060840382	-0.685344722	0.073264297	0.330927496	0.669955943	-0.57786187	0.722076845	-0.469775715	1.949070919	1.087622974	0.092293766	0.487534789	1.692018498	0.758745341	2.043714317	1.031193542	-5.222448388
unknown	327.2545	-2.481139903	-1.175338146	0.042139709	0.266102627	0.394658216	-1.341324311	0.442382601	-1.176633451	1.518968772	0.677273568	0.167253622	0.483324721	1.544245433	0.626902064	1.65137544	0.723668154	-1.252994165	-0.534061875	0.250439911	0.63626403	0.59383511	-0.751865702	0.724733696	-0.464477121	-3.768774032
unknown	559.4022	-2.955342376	-1.625189397	0.021245068	0.186842438	0.293724013	-1.76746688	0.331214209	-1.594163528	0.927928375	0.486485807	0.380589179	0.687104662	1.388803166	0.473842142	1.464518516	0.550426433	-1.73885095	-0.910641074	0.125619112	0.537166852	0.483943102	-1.047090658	0.590051223	-0.761087894	-2.742346048
unknown	207.0137	2.601253826	1.141083687	0.035360565	0.247394299	1.682296596	0.750432081	1.867846343	0.901375777	-1.989781914	-0.691128246	0.081800615	0.346828226	0.664507283	-0.589643084	0.716843017	-0.480270881	1.696751794	0.949487547	0.133557971	0.546977463	1.59720416	0.675548735	1.913610892	0.936297507	-5.175927816
unknown	411.1779	-4.158123385	-0.829391879	0.004251712	0.078232544	0.504643538	-0.986663416	0.558916797	-0.839294561	3.521543105	0.945717092	0.007828211	0.10473818	1.939028573	0.955334063	2.081450334	1.057589135	1.030013737	0.190143144	0.337272724	0.692740612	1.018095502	0.025872899	1.217737953	0.284203711	-14.64301074
unknown	391.2134	-4.440312348	-1.248470393	0.00300657	0.063449336	0.371718147	-1.427718974	0.414727899	-1.269762994	2.631800946	0.626844807	0.03009392	0.212649365	1.494666332	0.579823454	1.606123875	0.683583168	-2.967455538	-0.730412292	0.020882972	0.305218054	0.523224659	-0.93449756	0.631100669	-0.664057941	-11.68601824
unknown	325.1842	3.17292649	1.270797292	0.01564395	0.161373551	1.985220223	0.989299056	2.212405055	1.145615544	-2.140743722	-0.614061674	0.064701419	0.313927049	0.650982306	-0.619309765	0.699712436	-0.515165962	2.312216446	1.027466454	0.054010151	0.408856677	1.701534093	0.766836058	2.047907949	1.03415087	-6.792422462
unknown	318.2063	-5.554268013	-1.098032459	0.00085606	0.03433527	0.409520712	-1.287991675	0.456874453	-1.130130321	4.228902384	0.71046286	0.002880663	0.070662721	1.621501226	0.697330115	1.733603334	0.793773833	-0.665426117	-0.116936118	0.527087735	0.78581748	0.795097837	-0.330795699	0.959388685	-0.05981267	-23.48845724
unknown	361.2058	-2.362911449	-0.948422919	0.050126135	0.286849409	0.395877903	-1.336872552	0.438275251	-1.190090882	2.462008794	0.82099204	0.039195792	0.245200668	1.884748015	0.914371653	2.038834745	1.027744844	-0.212779511	-0.17179367	0.837562732	0.934962946	1.101906079	0.140001261	1.31713936	0.397407999	-5.817508768
unknown	311.1685	3.163396324	1.24498118	0.015853063	0.162360704	1.943867165	0.958929635	2.162668413	1.112812484	-2.132739187	-0.624797745	0.065512963	0.31551701	0.654321252	-0.611928965	0.703836602	-0.506687553	2.216731758	0.993221298	0.062176425	0.430416632	1.664660956	0.735228371	2.002348212	1.001692883	-6.746699305
unknown	348.1904	-4.781947342	-0.946023326	0.002007749	0.049746038	0.452311865	-1.144610254	0.503216426	-0.99074908	4.622510737	0.819727791	0.001704609	0.057594588	1.743027234	0.801595111	1.870272295	0.903248329	-0.736586416	-0.138055297	0.485321713	0.763731683	0.779931082	-0.358581448	0.942049738	-0.086124862	-22.10460293
unknown	346.1591	2.643394423	1.081230617	0.033259125	0.242005398	1.870435515	0.903374228	2.036042711	1.025767826	-1.125553899	-0.711274421	0.292985571	0.609040137	0.727293319	-0.459390772	0.785856766	-0.347661711	0.773629218	0.387750599	0.464469892	0.753453241	1.208096688	0.272735923	1.420652963	0.506554176	-2.9752829
unknown	305.1749	-4.802667495	-1.134071665	0.001960242	0.049074945	0.404453575	-1.305953983	0.448556191	-1.15663937	3.56075344	0.673465026	0.007392597	0.101989132	1.540006262	0.622936217	1.658678924	0.730034646	-0.601184232	-0.177662248	0.566667682	0.804362202	0.794270117	-0.33229837	0.956137908	-0.064709375	-17.1011148
[illegible]	[illegible]	[illegible]	[illegible]	[illegible]	[illegible]	[illegible]	[illegible]	[illegible]	[illegible]	[illegible]	[illegible]	[illegible]	[illegible]	[illegible]	[illegible]	[illegible]	[illegible]	[illegible]	[illegible]	[illegible]	[illegible]	[illegible]	[illegible]	[illegible]	[illegible]	[illegible]

unknown	403.271	3.556086357	0.758689196	0.009267873	0.121357028	1.508258412	0.592883629	1.659266585	0.730545695	-1.931798246	-0.526563225	0.089472014	0.360152472	0.694280238	-0.526409988	0.750829176	-0.413443383	0.759094261	0.148142433	0.472578283	0.755825057	0.979842338	-0.029378465	1.171430419	0.228271263	-6.869641387
unknown	594.3425	2.581468562	0.934142347	0.036394346	0.251470631	1.755181929	0.811620577	1.905793362	0.930391702	-1.238350046	-0.426867213	0.250690626	0.570993214	0.715050903	-0.483882147	0.771431826	-0.374389427	1.007464444	0.435622823	0.347260154	0.694413818	1.227500425	0.295723524	1.448658712	0.534717752	-3.196761714
unknown	623.5739	3.273775221	0.921174948	0.013603306	0.150959461	1.646622635	0.719509963	1.775550755	0.828266601	-2.15764832	-0.431144097	0.063019887	0.31029134	0.746182753	-0.422399081	0.799733905	-0.322408043	1.207979165	0.31624055	0.266278002	0.648755913	1.059091765	0.082827597	1.243240611	0.314105536	-7.063655604
unknown	433.3171	4.336212917	0.710571676	0.003411826	0.068993027	1.44099107	0.527061395	1.590588725	0.66956085	-2.327741622	-0.557583246	0.048331782	0.273824113	0.684756919	-0.546336157	0.740063562	-0.43427891	-0.088640247	-0.017434763	0.931850498	0.976309582	0.876960739	-0.18941584	1.051046878	0.071827017	-10.09358329
unknown	349.2389	-10.49142189	-1.715209466	1.56E-05	0.016446553	0.262278809	-1.930826846	0.292185186	-1.775045061	1.17311821	0.229441656	0.274491369	0.594844136	1.216903663	0.283214961	1.291889149	0.369482284	-3.976187732	-1.157030203	0.005349863	0.19274284	0.408791266	-1.290563723	0.49348779	-1.018913704	-12.30767807
unknown	317.2113	-3.599803821	-0.860776394	0.008742103	0.116579257	0.482841134	-1.050379508	0.53618918	-0.899185989	2.068992286	0.45655827	0.072342269	0.329660334	1.366723115	0.450720996	1.468406765	0.554251667	-1.234416022	-0.221960341	0.256876646	0.639505888	0.729723489	-0.454578202	0.882422326	-0.180458803	-7.447966338
unknown	316.1624	-5.252885482	-1.082493997	0.001182489	0.038328302	0.4211261	-1.247675804	0.468796694	-1.092965699	1.201459382	0.356451212	0.263931629	0.587289683	1.333960974	0.41571646	1.405053014	0.490624566	-3.060365697	-1.020981131	0.018314007	0.287772626	0.470468088	-1.087831228	0.56273749	-0.829466015	-6.311128546
unknown	383.1477	-2.402375898	-0.716679154	0.047300758	0.281705789	0.539274289	-0.890908842	0.59498817	-0.749067111	2.469804528	0.535995348	0.03872225	0.243888938	1.388727335	0.473763366	1.494251851	0.57942333	-0.391513968	-0.094925839	0.707062258	0.880719011	0.797883716	-0.325749592	0.960313559	-0.058422548	-5.933398869
unknown	431.302	3.755773784	0.800652772	0.00711414	0.103321402	1.530200352	0.61372056	1.690509166	0.757457838	-1.760321071	-0.478556201	0.116389879	0.409152775	0.723460177	-0.467014489	0.781500608	-0.355681099	1.00626072	0.202834369	0.347799729	0.694790021	1.002411612	0.003475032	1.202847572	0.266453832	-6.611367729
unknown	353.3072	3.030924725	0.881958163	0.019089675	0.17651305	1.519191987	0.603304201	1.684909268	0.752670905	-2.739540676	-0.432094927	0.025467311	0.198573795	0.737676874	-0.438939087	0.793253555	-0.334146013	1.527003345	0.48859666	0.17059785	0.576504405	1.145884493	0.196461625	1.383673612	0.468503673	-8.303341573
unknown	482.3154	3.659125202	0.520864101	0.008079672	0.111106439	1.264931692	0.339059479	1.38653325	0.471482214	-4.699130809	-0.730497376	0.001543194	0.05569795	0.605500224	-0.723800601	0.650238958	-0.620958099	-1.039181483	-0.181833008	0.333277348	0.69059472	0.769493726	-0.378018532	0.916461812	-0.125853329	-17.19470797
unknown	449.1741	4.897954278	1.630660851	0.00175722	0.046956071	2.552244366	1.351766468	2.855620208	1.513804117	-0.945758258	-0.233236071	0.371957201	0.680123646	0.855274178	-0.225541112	0.911654472	-0.133440966	2.64370676	1.121766416	0.033244044	0.353324531	1.832041829	0.873452444	2.223203966	1.152640314	-4.632280706
unknown	357.1557	7.530853654	1.125162063	0.000133791	0.020000824	1.904190683	0.929177955	2.108793943	1.076418132	-2.271896408	-0.333463429	0.052734117	0.285376912	0.791686512	-0.336998822	0.848159156	-0.237593085	3.177347152	0.748175782	0.015547953	0.267156132	1.467995935	0.553847973	1.765865533	0.820375489	-17.10931937
unknown	433.3175	4.433322385	0.706522002	0.003032054	0.063780717	1.438364653	0.524429473	1.588318157	0.667499928	-1.43803657	-0.528013782	0.18836716	0.504867344	0.736030938	-0.442161685	0.80030561	-0.321377073	1.337029252	0.262512101	0.223027057	0.619430327	1.057222256	0.080278701	1.268190315	0.342771264	-6.375279718
unknown	552.5	2.905406176	0.371894608	0.022809413	0.195390534	1.134487992	0.182041339	1.250097963	0.322041155	-12.30530421	-1.001589328	1.77E-06	0.001648794	0.495675854	-1.012531112	0.531751163	-0.911176811	-3.223414544	-0.732892834	0.014584213	0.261273766	0.535778719	-0.900290816	0.640309578	-0.643158504	-35.75190686
unknown	480.3096	2.627435457	0.477990531	0.034039248	0.24312151	1.225889647	0.293829116	1.338394415	0.42050333	-4.232449735	-0.776110916	0.00286678	0.070662721	0.589319517	-0.762878048	0.632387441	-0.661119378	-1.131099494	-0.240467942	0.295271447	0.666250902	0.737764719	-0.438767295	0.875932221	-0.191108855	-11.1204885
unknown	255.1604	-3.085480779	-0.595314761	0.017678786	0.171297715	0.579627107	-0.786803028	0.642270827	-0.638746327	4.248252427	0.621667739	0.002805809	0.070662721	1.529817822	0.61335986	1.63619677	0.710346258	0.124681626	0.033244273	0.904280605	0.963138943	0.90456628	-0.144701878	1.087316666	0.120772166	-13.10790121
unknown	312.1722	3.792786243	0.972039313	0.006778223	0.101214625	1.755723516	0.812065673	1.947322488	0.961491822	-1.676976289	-0.379916202	0.132070242	0.432778917	0.742084202	-0.430345201	0.79721006	-0.32696818	-0.069594413	-0.011882507	0.946462877	0.98311685	0.848094592	-0.237702911	1.02289343	0.032655846	-6.360412598
unknown	117.1021	2.403256142	1.76267609	0.047239631	0.281705789	2.430465004	1.281232361	2.661586671	1.412286546	-0.605532196	-0.20820185	0.56160658	0.819338919	0.830608254	-0.267759886	0.895789641	-0.158768112	-0.332575904	-0.426032896	0.749192229	0.903714859	0.925240832	-0.11209916	1.092602238	0.127768283	-1.455248969
unknown	330.1794	-2.9047587	-0.774812263	0.022830478	0.195390534	0.511131958	-0.968232296	0.56658622	-0.81963258	2.608869621	0.472404746	0.031185024	0.216107911	1.354364437	0.437615996	1.446770912	0.532836498	-1.594693287	-0.368605132	0.154810427	0.566017081	0.659257163	-0.601086754	0.790766072	-0.338677121	-7.578136729
unknown	481.3127	2.753483882	0.495179705	0.028361134	0.221754139	1.239218605	0.309430709	1.352876369	0.436030007	-4.023734198	-0.733163076	0.003821235	0.077146367	0.608283616	-0.717183949	0.652008631	-0.617037033	-0.934347287	-0.194743644	0.381226373	0.706975249	0.760631849	-0.394729746	0.90292985	-0.147314188	-11.07928726
unknown	403.2129	3.102002097	0.980528588	0.017273735	0.170041533	1.644136331	0.717329932	1.763204439	0.818199761	-0.786304166	-0.370208472	0.454347813	0.747280032	0.880119844	-0.18422811	0.958536433	-0.061094826	1.121846306	0.512042708	0.298930476	0.66712034	1.239176574	0.309381776	1.448698572	0.534757447	-2.439117173
unknown	473.2178	4.807165004	1.329075674	0.001950096	0.049074945	2.189550571	1.130634772	2.420742624	1.275449698	-1.136310895	-0.273115734	0.288717388	0.606077089	0.822053412	-0.282695961	0.878737497	-0.186495838	2.663688142	0.962434801	0.032293908	0.351590746	1.71583294	0.778909093	2.056048565	1.039874342	-5.462433969
unknown	227.0648	-3.13923526	-0.620265966	0.016396694	0.164244	0.571016329	-0.808396094	0.630728728	-0.664908448	3.059883053	0.576649852	0.015585364	0.1535229	1.485285937	0.570740696	1.592391183	0.671194789	-1.513788423	-0.454205104	0.173847555	0.578678376	0.673027391	-0.571262874	0.80436371	-0.314080101	-9.605692773
unknown	451.1724	4.208303127	1.123710044	0.003994151	0.075714701	1.855745065	0.891998532	2.062358877	1.044295402	-1.446433744	-0.317622189	0.186071741	0.504311646	0.803725104	-0.31522595	0.856720484	-0.223103511	2.220330331	0.731854562	0.0618471	0.430248532	1.417695426	0.503547621	1.707055524	0.771509984	-6.087031647
unknown	110.0714	-3.119771835	-0.736052632	0.016849085	0.167234216	0.50597646	-0.982857828	0.56417076	-0.825796198	2.509122465	0.48323642	0.036420688	0.237975259	1.416583601	0.502415746	1.510476851	0.595004074	-0.917695878	-0.239894843	0.389302138	0.708203131	0.71777484	-0.478396741	0.869965064	-0.200970629	-7.827889596
unknown	433.318	3.629342969	0.778194834	0.008405175	0.113991122	1.530553921	0.614053871	1.685637491	0.753294307	-1.270048283	-0.454814914	0.23976019	0.560937843	0.761860418	-0.392401392	0.825487515	-0.2766817	1.206343682	0.228535248	0.266869026	0.64888539	1.028889183	0.041087604	1.230396509	0.299123315	-4.609440808
unknown	474.2212	4.661832403	1.307795452	0.002309531	0.054200457	2.148382797	1.103251074	2.374476561	1.247609515	-1.105200942	-0.269677122	0.301199581	0.618231811	0.827003131	-0.274035304	0.884177333	-0.177592346	2.632410249	0.953079622	0.033794043	0.353324531	1.695590098	0.761787447	2.03104232	1.022220301	-5.152261565
unknown	311.169	3.796191225	0.982785636	0.006748198	0.101214625	1.768815948	0.822783938	1.960239191	0.971029705	-1.569231367	-0.358613057	0.155233021	0.466916297	0.754063546	-0.407241989	0.809786499	-0.304386505	0.008888255	0.001536319	0.993156298	0.998152932	0.856294603	-0.223820862	1.031640955	0.044940953	-5.957102346
unknown	412.3521	4.623443138	0.842126992	0.002416303	0.055693055	1.573164979	0.653669975	1.71486983	0.77809907	-2.979687074	-0.416366255	0.017608819	0.165218861	0.74518656	-0.424326441	0.801653084	-0.31895005	1.295268281	0.39867106	0.23630614	0.627248075	1.180626532	0.239552669	1.401808018	0.487288781	-13.77641375
unknown	74.0247	-3.894814522	-0.591642887	0.005938421	0.094652316	0.571418707	-0.807379828	0.631860867	-0.662321176	6.562670417	0.589769944	0.000176122	0.028695533	1.504599143	0.589379174	1.609301346	0.6864345	-1.539694232	-0.227775615	0.167529483	0.573066253	0.728624904	-0.456751789	0.875743396	-0.191419891	-25.56038404
unknown	535.25	2.982424449	0.591151397	0.020444516	0.184141836	1.333202448	0.414895871	1.486125406	0.571555862	-3.221600414	-0.589953052	0.012209841	0.135408793	0.65366492	-0.61337682	0.697442181	-0.519854476	-0.225294591	-0.090392049	0.828186121	0.933376377	0.903419202	-0.146532516	1.090729471	0.12529332	-9.608179838
unknown	449.1738	4.159495389	1.189381619	0.004244432	0.078232544	1.9193926	0.940649836	2.133360678	1.093127896	-1.372557269	-0.289273756	0.207134984	0.527543366	0.817976462	-0.289868765	0.872472061	-0.196819161	2.148875978	0.756798862	0.068727602	0.446832199	1.430207076	0.516224047	1.722894486	0.78483435	-5.709145634
unknown	271.228	-5.248414032	-0.608890872	0.001188277	0.038328302	0.580421708	-0.784826617	0.641583839	-0.640290293	3.440016209	0.562942886	0.008823596	0.113488505	1.478758446	0.564386409	1.584447835	0.663980163	-0.426462064	-0.093473391	0.682579378	0.867322706	0.838017932	-0.25494698	1.004012961	0.005777893	-18.05462934
unknown	345.1711	-4.449037699	-1.084820014	0.002975091	0.063235069	0.415846623	-1.265876578	0.462528243	-1.112386632	1.399098511	0.315380458	0.1993396	0.519334211	1.234637426	0.30408743	1.31719037	0.397463869	-3.804811025	-0.802293746	0.006672834	0.202513001	0.498328964	-1.004829667	0.601397964	-0.733608112	-6.224642018
unknown	375.1144	2.576542079	0.782883	0.036656598	0.252415711	1.529539996	0.613097832	1.680986497	0.749308136	-1.297048984	-0.436174053	0.230768778	0.552164377	0.740233969	-0.433946753	0.799655713	-0.322549104	1.653998438	0.426378317	0.142104109	0.553562027	1.161719534	0.216261811	1.384437356	0.469299775	-3.341901284
unknown	256.1638	-2.500238326	-0.554413762	0.040977926	0.263674223	0.595043289	-0.748933469	0.659050662	-0.601538723	3.985244241	0.615411631	0.004032023	0.078798929	1.517682642	0.601870145	1.622184069	0.697937531	0.201599341	0.056506237	0.845963256	0.937387662	0.913874175	-0.12993255	1.098301636	0.135274329	-9.964060392
unknown	449.1739	4.064835649	1.201502683	0.004780299	0.082466474	1.907877192	0.931968309	2.1231638	1.086215678	-1.230463593	-0.283906199	0.253473878	0.574922837	0.828488264	-0.271446833	0.884256657	-0.177462919	2.383569521	0.887912092	0.048626263	0.393217926	1.552431113	0.634529252	1.872860313	0.905243301	-5.001632278
[illegible]	[illegible]	[illegible]	[illegible]	[illegible]	[illegible]	[illegible]	[illegible]	[illegible]	[illegible]	[illegible]	[illegible]	[illegible]	[illegible]	[illegible]	[illegible]	[illegible]	[illegible]	[illegible]	[illegible]	[illegible]	[illegible]	[illegible]	[illegible]	[illegible]	[illegible]	[illegible]

unknown	344.1325	-2.930522335	-0.813359544	0.02200792	0.190207357	0.512943135	-0.963129198	0.5688727	-0.813822246	0.630846215	0.149217827	0.545737139	0.810348669	1.075026309	0.104371967	1.143628191	0.193618089	-4.348879169	-0.693462566	0.003359446	0.158658761	0.532293005	-0.909707489	0.639429191	-0.645143488	-1.848708922
unknown	300.2261	-2.342304792	-0.37770593	0.051669373	0.290640479	0.677588868	-0.561517922	0.741013586	-0.432428102	2.080859939	0.31562562	0.071020182	0.325920814	1.24413689	0.315145231	1.335231793	0.417090212	-0.540619792	-0.111758226	0.605536368	0.824880438	0.810671055	-0.302811463	0.962528384	-0.05509901	-4.874008208
unknown	522.2481	3.098754317	0.614384716	0.017352573	0.170045191	1.366403135	0.45038319	1.490380218	0.57568043	-1.383127328	-0.192218281	0.203999279	0.523732008	0.86324524	-0.212157621	0.923037789	-0.115538382	1.479949814	0.287425956	0.182427952	0.584576254	1.069995766	0.097605088	1.265782424	0.340029441	-4.285971779
unknown	538.3518	2.393122015	0.479896572	0.047948302	0.28280061	1.218389031	0.284974858	1.326722752	0.407866919	-1.362283526	-0.244971252	0.210222743	0.531548084	0.844955263	-0.243053137	0.909576816	-0.136732612	0.80750641	0.195476409	0.445942346	0.740852511	0.993966637	-0.008730668	1.175133177	0.232824265	-3.260110696
unknown	411.1846	-2.35070617	-0.313262264	0.051034421	0.289639217	0.718428893	-0.477082722	0.791516644	-0.337308407	2.866150529	0.36832087	0.020952459	0.180499402	1.273971435	0.34933293	1.365066593	0.448971333	3.135527934	0.236403665	0.016481855	0.272096653	1.028043464	0.039901261	1.227180498	0.295347461	-6.737477733
unknown	299.2235	-4.029008039	-0.405987235	0.005002088	0.085166099	0.666717164	-0.584853227	0.732747996	-0.448610978	2.120841593	0.28363591	0.066737714	0.318825373	1.215432341	0.281469586	1.305657594	0.384776603	-0.614104908	-0.105788696	0.558568	0.798945963	0.815602836	-0.294061302	0.974362499	-0.037469487	-8.544887828
unknown	603.3501	2.706350773	0.44845113	0.030358814	0.229787583	1.208866279	0.273654667	1.320986054	0.401615236	-1.669182369	-0.253597473	0.133632686	0.434839693	0.830776424	-0.267467819	0.894050641	-0.161571543	1.463095166	0.27239228	0.186843631	0.587692397	1.056407553	0.079166521	1.252182599	0.324444958	-4.517392994
unknown	298.2834	3.602212391	0.872263192	0.008714091	0.11644382	1.552081823	0.634204616	1.711358403	0.77514193	-0.943457157	-0.129223632	0.373063019	0.680297525	0.911004517	-0.134469888	0.976971468	-0.033611665	3.337064695	0.753163756	0.012469718	0.246409183	1.411389368	0.497116047	1.688575075	0.755806324	-3.398533063
unknown	113.0709	3.89007662	0.427036994	0.005974802	0.094685692	1.194837551	0.256814484	1.316578151	0.396793161	-2.074387315	-0.258666395	0.071738296	0.327462229	0.83225991	-0.264893951	0.8896059	-0.168761739	0.587810289	0.051354221	0.575123814	0.808272067	0.912138084	-0.132675852	1.089224552	0.123301408	-8.069525595
unknown	317.1145	-2.499004815	-0.622161032	0.041051955	0.263674223	0.570860455	-0.808789969	0.636559286	-0.65163321	0.564363954	0.17432178	0.587975835	0.834245087	1.199992354	0.263025214	1.263228522	0.337115651	-1.800236448	-0.385439903	0.114844703	0.523989851	0.664029444	-0.59068088	0.803387098	-0.315832802	-1.410348238
unknown	284.2315	-8.716724312	-0.644587442	5.25E-05	0.016446553	0.564775495	-0.824250601	0.622587812	-0.68365076	1.370672433	0.16818617	0.207698509	0.528279765	1.123858789	0.168460775	1.205407201	0.269520588	-2.534452935	-0.483184181	0.038978543	0.365164601	0.638779118	-0.646610944	0.762620236	-0.390963283	-11.94777372
unknown	285.2076	-3.343456336	-0.448137858	0.012361006	0.144014736	0.647535684	-0.626968396	0.714950767	-0.484084198	1.871321747	0.241479367	0.098207276	0.376821298	1.174074517	0.231523978	1.256312305	0.329195147	-0.472886083	-0.102535809	0.650684387	0.850436367	0.822223083	-0.282398221	0.986461653	-0.019665125	-6.256682552
unknown	618.3423	3.010343374	0.689076222	0.019652598	0.179237188	1.414846998	0.500646048	1.536464472	0.619614407	-0.828694026	-0.156694588	0.431305423	0.72675521	0.893764138	-0.162033936	0.963385934	-0.053814235	1.399429624	0.524533396	0.204404699	0.603765516	1.297450004	0.375678946	1.530974429	0.614450186	-2.49465357
unknown	602.3468	2.635798856	0.447654638	0.033628073	0.24205311	1.206812612	0.271201679	1.318058381	0.398414273	-1.573476918	-0.240116311	0.154254342	0.464874765	0.838871676	-0.25347796	0.902704207	-0.147674764	1.521036731	0.289941509	0.172058166	0.57715602	1.068725225	0.095890977	1.266040052	0.340323046	-4.147368661
unknown	606.4074	8.994520942	1.77920496	4.28E-05	0.016446553	3.025544987	1.597195036	3.318503941	1.730532988	-0.195464084	-0.060338148	0.849902931	0.952964086	0.997556087	-0.003530136	1.080557462	0.111775793	3.176418759	1.567755073	0.015568062	0.267156132	2.877283649	1.524707452	3.437019026	1.781157838	-1.758105799
unknown	283.1111	2.53945917	0.531835051	0.038694563	0.258267399	1.289374545	0.366671408	1.419374112	0.505254898	-0.918027937	-0.199324437	0.385445427	0.691240428	0.860754094	-0.216326958	0.923546535	-0.114743439	1.273015431	0.206355023	0.243658026	0.633530297	1.001866687	0.002690549	1.19087308	0.252019663	-2.331294462
unknown	463.2555	2.33079853	1.127218849	0.052552074	0.293652166	1.65606987	0.727763542	1.816115461	0.860855926	-0.394137479	-0.093745511	0.703777351	0.889830118	0.922583117	-0.116249203	0.993245824	-0.009777272	1.966446629	0.98347379	0.08996106	0.483648665	1.489444171	0.574774048	1.77049914	0.824156143	-0.918655057
unknown	281.1277	3.678660389	0.774001097	0.0078736	0.109418909	1.475976242	0.561669499	1.636408012	0.710532506	-0.898748456	-0.13525982	0.395031701	0.698861021	0.898143081	-0.1549828	0.966242015	-0.049543509	3.657564502	0.886915283	0.008096386	0.211667678	1.587415187	0.666679513	1.911534212	0.934731021	-3.306190346
unknown	766.5734	2.515244029	0.53645634	0.040088315	0.261043415	1.288846737	0.366080716	1.41202724	0.49776792	-1.239315992	-0.191673217	0.250351487	0.570993214	0.862129081	-0.214024204	0.923055684	-0.115510413	0.285529104	0.061243398	0.783502879	0.918332199	0.906390384	-0.141795539	1.082472616	0.114330528	-3.117182148
unknown	167.0925	2.710978655	0.663903009	0.030156307	0.229352204	1.384194816	0.469047007	1.535774668	0.618966557	-0.469779479	-0.15476484	0.651051634	0.865736152	0.940613995	-0.088325298	1.020651219	0.029489947	1.200769191	0.350742988	0.268891815	0.649036986	1.100222386	0.137795162	1.334545873	0.416348897	-1.273562139
unknown	283.2282	-7.091712572	-0.66958967	0.000195079	0.022534203	0.555496777	-0.848149555	0.613971673	-0.703756001	1.017062973	0.151369066	0.338892363	0.650800623	1.110218344	0.150843436	1.189962106	0.250915632	-3.128725278	-0.509420762	0.016639348	0.273534928	0.619441978	-0.690958942	0.741396508	-0.431682774	-7.212718275
unknown	414.1385	4.086389233	0.363622241	0.004652071	0.081404749	1.134869662	0.182526616	1.248746934	0.320481135	-2.186400944	-0.277937057	0.060258159	0.303431241	0.82960672	-0.269500515	0.887063377	-0.172890911	1.620836679	0.235531663	0.149083774	0.560468293	1.03872556	0.054814532	1.240962302	0.311459289	-8.934485277
unknown	409.2606	2.384259391	0.974177685	0.048576974	0.284275558	1.762744742	0.817823577	1.930215826	0.94876217	-0.298600269	-0.102267064	0.772854122	0.919944489	0.90764776	-0.139795571	0.966031186	-0.049858331	0.391172472	0.302046443	0.707303398	0.880719011	1.338492732	0.420609305	1.585736394	0.665152963	-0.711940496
unknown	202.0722	3.681409322	0.684052158	0.007845063	0.109418909	1.426369877	0.512348141	1.575957236	0.656228387	-0.844466869	-0.145356299	0.422941486	0.720295704	0.892985004	-0.163292146	0.955456359	-0.065738116	2.894502463	0.551395495	0.02316691	0.312521914	1.287750006	0.364852546	1.540960955	0.623830307	-3.108828205
unknown	116.0434	4.184287977	0.262446929	0.004115214	0.077112954	1.054262987	0.076234794	1.165913631	0.22146092	-8.489645152	-0.378328569	2.84E-05	0.012348446	0.764196938	-0.387983619	0.81927544	-0.287579528	0.624104186	0.114303165	0.552347273	0.796342377	0.9673589	-0.047876851	1.162107025	0.21674294	-35.52312014
unknown	360.2994	4.314083507	0.722918032	0.003505509	0.069861832	1.460662363	0.546622733	1.599891111	0.677973719	-0.625391527	-0.137163484	0.54913412	0.811978334	0.942668003	-0.085178335	1.001732174	0.002496837	3.867700128	0.664660037	0.006149941	0.200230902	1.397805689	0.483163824	1.658401643	0.729793451	-2.697991271
unknown	558.4613	5.463622958	0.646003972	0.00094223	0.035516069	1.396247287	0.481554477	1.536765476	0.619897013	-0.78184099	-0.15248154	0.456821561	0.749605788	0.899872465	-0.152207545	0.969965797	-0.043994219	3.428415543	0.255709534	0.011008863	0.231576769	1.04684145	0.066042955	1.251940784	0.324166325	-4.271684383
unknown	297.2797	3.192023298	0.833925099	0.015233739	0.158942741	1.497950973	0.582990406	1.650710558	0.723087174	-0.915459127	-0.117740275	0.386712822	0.692409202	0.920498824	-0.119524756	0.984968094	-0.021851102	2.796404439	0.711822401	0.026661682	0.331244217	1.364310546	0.44817207	1.630433223	0.705255355	-2.922166861
unknown	287.1972	2.640472986	0.454153744	0.03340053	0.242005398	1.217013059	0.283344649	1.327425975	0.40863141	-1.802629801	-0.212260478	0.109113508	0.395074506	0.851994341	-0.231084247	0.913761393	-0.130110606	0.880469864	0.190249497	0.407815427	0.716489415	1.008374913	0.012032131	1.196019388	0.258240776	-4.759795294
unknown	334.2834	3.827207568	0.588453145	0.006481266	0.098870669	1.333607633	0.415334267	1.468033107	0.553884504	-0.921935382	-0.162849976	0.383523403	0.689348431	0.897141067	-0.156593242	0.960334958	-0.0583904	4.976748411	0.567794106	0.001606875	0.109150303	1.281775171	0.35814323	1.535265952	0.618488593	-3.528438071
unknown	320.1567	-2.588134833	-0.47449129	0.036042574	0.250302051	0.638524792	-0.647185458	0.712142347	-0.48976245	0.767199819	0.191563455	0.46499993	0.75426479	1.171130655	0.227902037	1.240874642	0.311357376	-2.987264116	-0.340890116	0.020304852	0.303668884	0.689556021	-0.53626033	0.829873082	-0.269037383	-1.985616575
unknown	393.1729	3.963109377	0.682228897	0.005439996	0.089131199	1.411583073	0.497314036	1.565197043	0.646344289	-0.700696268	-0.128377759	0.503359061	0.779113325	0.916844552	-0.125250944	0.980776791	-0.028003255	0.003808004	0.000515955	0.99706791	0.998979237	0.866392491	-0.206907356	1.042615266	0.060206889	-2.776935951
unknown	312.2264	2.593294953	0.356408279	0.035772703	0.249490694	1.117758591	0.160608635	1.24448135	0.31554461	-0.870522773	-0.250484635	0.409375119	0.709416728	0.905731991	-0.142843879	0.971991098	-0.040984995	-2.005253749	-0.257876268	0.084958463	0.475795095	0.722085025	-0.469759371	0.870705867	-0.199742651	-2.257522313
unknown	359.1195	3.821634189	0.261300989	0.006528371	0.098870669	1.056015973	0.078631656	1.170244725	0.226810262	-4.04944896	-0.341655023	0.003687031	0.075370308	0.784231582	-0.350648352	0.840007282	-0.251526261	-0.187412862	-0.039685409	0.856653514	0.942135942	0.878717076	-0.186529364	1.055484228	0.077905021	-15.47551259
unknown	326.1588	2.530783142	0.572981266	0.03918808	0.259183683	1.282048619	0.358450974	1.429292892	0.515301585	-0.842457309	-0.154515488	0.424000757	0.721077673	0.900001635	-0.15185621	0.958813666	-0.060677622	3.258182542	0.648191537	0.013899237	0.253884939	1.341883094	0.424258988	1.617403195	0.693679367	-2.132076756
unknown	361.1251	5.818869733	0.29059165	0.000650889	0.029681429	1.072921445	0.101544451	1.187164806	0.247520229	-2.631219657	-0.304033626	0.030121091	0.212649365	0.811280231	-0.301727761	0.871072871	-0.199134681	0.439162953	0.084375075	0.673780335	0.861949842	0.948622091	-0.076094629	1.139431296	0.188313938	-15.31072442
unknown	325.2751	2.959182891	0.673868472	0.02112955	0.186701621	1.377107921	0.461641625	1.531622586	0.61506084	-0.677552904	-0.128704228	0.517166629	0.791263075	0.916059301	-0.126487101	0.975267443	-0.036130198	2.583602452	0.577985392	0.036281357	0.360129185	1.289611671	0.366936705	1.555724201	0.637586322	-2.005002962
unknown	282.252	2.499094009	0.654175736	0.041046598	0.263674223	1.347095074	0.429851676	1.492576527	0.577804903	-0.848994473	-0.13251649	0.420561715	0.7188684	0.900067243	-0.151895308	0.965102545	-0.051245854	1.848167313	0.505659642	0.107054595	0.511805728	1.202654991	0.266222832	1.448964589	0.535022338	-2.121717
unknown	408.2476	-2.503427505	-0.357984993	0.040787166	0.263326094	0.693519232	-0.527992205	0.757910275	-0.39990103	0.909405885	0.241653356	0.389711379	0.694290296	1.244760251	0.315867897	1.337229404	0.419246984	0.566463848	0.219031838	0.588771104	0.816020482	1.088029996	0.121718332	1.300647724	0.379230266	-2.276631707
unknown	333.2709	2.842893382	0.805912377	0.024941003	0.205359962	1.54953236	0.631832884	1.70101166	0.76639303	-0.495910912	-0.107271949	0.633292683	0.857945553	0.8958448	-0.15867928	0.966390191	-0.049322284	3.781518962	0.853585232	0.006878619	0.203725671	1.527983627	0.611629084	1.838486213	0.878518357	-1.40982185
unknown	475.2243	2.698573463	1.029344942	0.030702341	0.230890429	1.84270656	0.881826349	2.048865833	1.034825514	-0.250910449	-0.083743328	0.808207625	0.934619954	0.899383293	-0.15299201	0.957654132	-0.062423392	2.298963541	0.784562857	0.055075149	0.409753683	1.495718599	0.580838775	1.798478638	0.846777023	-0.677100278
unknown	594.4843	3.605231342	0.688334929	0.008679116	0.11644382	1.418225205	0.504086641	1.56020861	0.64173894	-0.690953678	-0.124363376	0.509143205	0.783565312	0.909801847	-0.136375731	0.975108057	-0.036365994	2.448203147	0.603902285	0.044223641	0.387440217	1.330798865	0.412292541	1.595010377	0.67356581	-2.491047854
unknown	923.2365	2.661419159	0.775319238	0.032400362	0.240157349	1.49448591	0.579649296	1.634500959	0.708850224	-0.413859599	-0.109860894	0.675852982	0.878811025	0.918741338	-0.122269352	0.985975543	-0.020376527	2.058281143	0.498250082	0.07856418	0.46499309	1.204260593	0.268158396	1.420625495	0.506526281	-1.15468225
unknown	509.3498	4.362907707	0.559303259	0.003302463	0.067721253	1.305518226	0.384622598	1.438267151	0.524331674	-0.867293074	-0.149871277	0.411039746	0.710118859	0.908388903	-0.138618012	0.970970681	-0.042500361	1.940765535	0.233582752	0.093429624	0.490930619	1.032726823	0.046458682	1.231947627	0.300940926	-3.783919638
unknown	813.6127	2.908766117	0.498758857	0.022700429	0.195032278	1.251521704	0.323683311	1.366735821	0.450734408	-1.068335579	-0.167630018	0.316542843	0.62966224	0.888984354	-0.169770067	0.954390164	-0.06734892	2.327035515	0.385310162	0.052844075	0.407324132	1.134531962	0.182097253	1.34510849	0.427722539	-3.107538333
unknown	244.11	2.570994235	0.618163803	0.036954275	0.253484968	1.333406572	0.415116743	1.449805786	0.535859651	-1.093554516	-0.133985782	0.305981738	0.621784641	0.901216856	-0.150053797	0.965020028	-0.05136921	1.084863424	0.392088595	0.313925583	0.677273074	1.163667845	0.218679317	1.371909148	0.456184945	-2.811522357
unknown	317.1146	-2.504694346	-0.623330104	0.040711645	0.263223187	0.575532365	-0.797031034	0.642318922	-0.638638298	0.376889758	0.132070578	0.716056167	0.894521507	1.193859903	0.255633549	1.252609668	0.324936919	-1.966412207	-0.431748595	0.089965624	0.483648665	0.649079764	-0.623532317	0.785468819	-0.348374089	-0.943993646
unknown	836.6094	3.11747435	0.568600057	0.016903357	0.167263717	1.303447961	0.382332986	1.427328533	0.513317443	-1.032904897	-0.143596064	0.331860495	0.643713746	0.900849373	-0.150642196	0.964443946	-0.049242037	1.484944912	0.307465155	0.181137594	0.583974335	1.07456325	0.103750404	1.275108994	0.350620571	-3.220054522
unknown	605.4044	9.547466038	1.770913319	2.90E-05	0.016446553	2.994020971	1.582084326	3.284213575	1.71554795	-0.163958853	-0.046022933	0.873830178	0.960136707	0.999838838	-0.000235412	1.082735824	0.114681284	3.165029958	1.527699382	0.015817007	0.268600781	2.775730692	1.472867601	3.315614546	1.729276297	-1.565391583
unknown	379.1773	-3.145063176	-0.815479657	0.016263769	0.163542494	0.510098333	-0.971152709	0.565389921	-0.822681929	0.489348039	0.098038524	0.637729715	0.860338681	1.025063334	0.03571305	1.098680893	0.135772423	-2.966044685	-0.57061652	0.020942808	0.305218054	0.582402566	-0.779911383	0.700917145	-0.512684181	-1.539030497

unknown	279.0189	2.52409022	0.213281828	0.039573236	0.259621897	1.023036907	[illegible]
unknown	502.3992	3.359961141	0.797803197	0.012084966	0.143023707	1.584023845	[illegible]
unknown	149.0457	2.32357864	0.703874501	0.053113777	0.295272751	1.503445257	[illegible]
unknown	356.2472	2.773374161	0.543304191	0.027559867	0.217361705	1.265451644	[illegible]
unknown	142.9384	5.398309528	0.459202646	0.001010313	0.035647699	1.211821449	[illegible]
unknown	582.3779	3.317624147	0.221281146	0.012806731	0.146770994	1.028307616	[illegible]
unknown	553.2746	4.090446492	0.972650097	0.004628357	0.081351802	1.743589001	[illegible]
unknown	382.2967	3.32908845	0.30579218	0.012606824	0.145245763	1.085628369	[illegible]
unknown	583.381	3.458256428	0.211767242	0.010572477	0.131822417	1.021221812	[illegible]
unknown	161.1285	2.486907632	0.381767032	0.041785315	0.266075514	1.148963727	[illegible]
unknown	355.1683	2.536671298	0.392703263	0.038852441	0.258914285	1.165872227	[illegible]
unknown	585.3865	4.805311158	0.202377148	0.001954271	0.049074945	1.013806	[illegible]
unknown	254.0157	-3.602823431	-0.840802315	0.008706999	0.11644382	0.474228874	[illegible]
unknown	584.3837	3.602840916	0.217624652	0.008706796	0.11644382	1.025754426	[illegible]
unknown	332.2679	2.970525012	0.598558946	0.020792259	0.185734687	1.312400318	[illegible]
unknown	528.2733	5.023484407	0.973022764	0.001524474	0.042105315	1.748523441	[illegible]
unknown	295.1194	-4.107831399	-0.582329398	0.004528234	0.080459444	0.572294202	[illegible]
unknown	571.428	2.5473203	1.401379161	0.038252951	0.256662025	2.348130594	[illegible]
unknown	168.1028	2.972631959	0.311596918	0.020730233	0.185434638	1.094291643	[illegible]
unknown	392.2237	-2.832939178	-0.20704742	0.02529935	0.207257611	0.763593302	[illegible]
unknown	383.3	3.037408086	0.328909066	0.018915913	0.176215241	1.100181732	[illegible]
unknown	629.3659	2.49851176	0.696456427	0.041081584	0.263674223	1.417616803	[illegible]
unknown	403.3059	2.388862557	0.754969578	0.048249402	0.283709963	1.411741478	[illegible]
unknown	182.0318	-2.490413356	-0.42102606	0.04157141	0.265251629	0.644788242	[illegible]
unknown	239.1077	2.530445763	0.260005505	0.039207402	0.259183683	1.057994863	[illegible]
unknown	431.1393	3.247925357	0.344446363	0.014097662	0.151951826	1.12163654	[illegible]
unknown	338.3148	2.423072327	0.601860013	0.045884749	0.278339023	1.313520582	[illegible]
unknown	254.148	-2.453213808	-0.462763196	0.0438999	0.271347152	0.621906048	[illegible]
[illegible]	[illegible]	[illegible]	[illegible]	[illegible]	[illegible]	[illegible]	[illegible]

unknown	216.0308	4.30844315	3.874317589	0.003529837	0.069963736	7.034728276	2.814494699	8.096393396	3.017279391	1.443305821	0.599616148	0.186923878	0.504479396	1.386287665	0.471226658	1.490735619	0.576024419	1.004572406	1.195336276	0.348557624	0.694790021	1.685860674	0.753485312	2.11196714	1.078587388	6.218401077
unknown	381.1717	-2.312657581	-1.104493099	0.053975064	0.298028277	0.390804585	-1.355480701	0.436320077	-1.196541236	-3.649174966	-2.172998345	0.006502115	0.096364298	0.272739774	-1.874402988	0.298017329	-1.746531872	-4.800145921	-3.380978714	0.001965956	0.121070365	0.10031261	-3.317425119	0.121967694	-3.035429023	8.43929215
unknown	409.2607	2.641648658	1.150928319	0.03334355	0.242005398	1.95934598	0.97037217	2.186117035	1.128370639	5.152318461	2.092986899	0.000871838	0.044765801	4.23506187	2.082383046	4.487652831	2.165961072	4.605897281	2.141129801	0.00246691	0.136328132	3.880277735	1.956159918	4.682237894	2.227198236	13.61061515
unknown	441.2524	5.128302622	2.089839364	0.0013562	0.04019899	3.694585987	1.885412706	3.992317796	1.997226567	5.335893725	1.310439236	0.000697569	0.040614693	2.387385371	1.255431465	2.536749053	1.342980808	3.73640209	1.484540281	0.007297134	0.205993988	2.368961293	1.244254627	2.772864902	1.471377328	27.36407778
unknown	381.2111	-5.298074915	-1.810628796	0.001125718	0.038328302	0.236465567	-2.080297975	0.264061141	-1.921056084	-1.837919344	-1.803440372	0.103374659	0.386125007	0.526920783	-0.92434201	0.555043734	-0.849326643	-4.110946689	-2.454437074	0.004510545	0.186159882	0.180557953	-2.469466127	0.21966136	-2.186646985	9.73743437
unknown	390.2735	-2.327851392	-1.786384685	0.052780625	0.294676762	0.2087529	-2.260131858	0.234622174	-2.091588728	-3.928099823	-1.86694001	0.00436834	0.081386137	0.250933595	-1.994622464	0.274093097	-1.867262099	-4.498436043	-3.289336838	0.002803579	0.146257116	0.069598333	-3.844803439	0.085578928	-3.546600576	9.14403264
unknown	406.2682	-3.089493155	-2.139573167	0.017579494	0.170843338	0.197112513	-2.342908734	0.222143513	-2.170436081	-6.093829483	-3.873241752	0.000291447	0.03028281	0.067551851	-3.887860888	0.072711412	-3.781674374	-2.808628268	-5.107905419	0.026197485	0.331073259	0.221900422	-2.172015685	0.278113177	-1.846255992	18.82684448
unknown	501.293	-2.319088025	-3.198275238	0.053466223	0.296726159	0.213849467	-2.225332484	0.235365239	-2.087026828	-2.093347873	-2.867818483	0.069654487	0.323801609	0.119870765	-3.060448251	0.123951149	-3.012156446	-3.77520245	-4.722784022	0.006935608	0.203725671	0.074989368	-3.737170118	0.091059437	-3.457047655	4.854657985
unknown	405.2649	-3.027378075	-2.195230963	0.019185446	0.17651305	0.188836082	-2.404793639	0.213085032	-2.230498837	-7.735754388	-5.598407665	5.56E-05	0.017254805	0.026813471	-5.220898179	0.027580467	-5.180209285	-6.945737398	-8.309481809	0.000222038	0.043876004	0.005848369	-7.417749925	0.007114937	-7.134933394	23.41905323

Supplemental table 3

ID	m/z	statistic_OLZ vs CON	dm_OvsC	p.value_OvsC	adj_pval_OvsC	fold_change_imputed_OvsC	log2_fold_change_imputed_OvsC	fold_change_imputed_scaled_OvsC	log2_fold_change_imputed_scaled_OvsC	statistic_MOvsO	dm_MOvsO	p.value_MOvsO	adj_pval_MOvsO	fold_change_imputed_MOvsO	log2_fold_change_imputed_MOvsO	fold_change_imputed_scaled_MOvsO	log2_fold_change_imputed_scaled_MOvsO	statistic_MvsC	dm_MvsC	p.value_MvsC	adj_pval_MvsC	fold_change_imputed_MvsC	log2_fold_change_imputed_MvsC	fold_change_imputed_scaled_MvsC	log2_fold_change_imputed_scaled_MvsC	statOvsC*statMOvsO
		OLZ vs CON								MO vs OLZ								MINO vs CON								
11d-TXB2 [M-H]	367.2122	-5.335067146	-0.8803872	0.0010815	0.03720363	0.475700298	-1.071875165	0.528203565	-0.920834057	3.642766857	0.623959125	0.006562644	0.096587859	1.53596721	0.619147417	1.651982671	0.724198553	-1.9074352	-0.345607	0.09812709	0.49809109	0.69162285	-0.5319426	0.83221515	-0.2649715	-19.434406
15(S) HEPE [M-H]	317.2124	-5.216507731	-1.038081753	0.001230507	0.038328302	0.441117278	-1.180765826	0.486823068	-1.038530563	3.684351835	0.811227237	0.006180275	0.094611384	1.718810262	0.781410296	1.845652115	0.884130646	-1.0993003	-0.2165545	0.3080011	0.67534471	0.76849183	-0.3798982	0.91873231	-0.1222835	-19.21945
15(S) HEPE [M-H+Acetate]	377.2332	-6.325572287	-0.962707732	0.000394353	0.025604563	0.457935038	-1.126785139	0.506469837	-0.981451743	3.789121421	0.717741391	0.005318487	0.087859906	1.631924126	0.706573982	1.750758691	0.807980249	-1.3359907	-0.2452157	0.22334924	0.61943033	0.74877151	-0.4174025	0.89692357	-0.156943	-23.968361
15R-PGD2 [M-H]	351.218	-6.223823192	-1.4156692	0.000435056	0.026405285	0.321548752	-1.636890603	0.358468085	-1.480083417	5.056718873	1.043259105	0.000981035	0.047741268	2.070684819	1.050107976	2.219273772	1.150087651	-0.7217383	-0.2776868	0.49385225	0.76821474	0.78809073	-0.3435664	0.95187198	-0.0711605	-31.472124
17(18) EpETE [M-H]	317.2124	-3.641146032	-1.575115524	0.008274505	0.112753035	0.275824207	-1.858179016	0.31092087	-1.685380636	2.925341778	1.044529525	0.019134114	0.172706358	2.067260453	1.047720165	2.225424603	1.154080624	-0.1733323	-0.0976164	0.86729594	0.94560399	0.89632812	-0.1579011	1.09248638	0.12761529	-10.651597
20cooh AA [M-H]	333.2075	-3.479309858	-0.924878494	0.010275834	0.1291112	0.450055437	-1.151825373	0.504036047	-0.98840118	2.492722261	0.621593511	0.03736338	0.239448539	1.550808495	0.633020543	1.670515057	0.740292986	-0.2553106	-0.0817701	0.80582231	0.92643993	0.83564286	-0.2590416	1.01541168	0.02206477	-8.6729531
8-iso-PGA1 [M-H]	335.2232	-8.126951417	-2.403329614	8.24E-05	0.017507673	0.161597602	-2.629522307	0.181300852	-2.463542387	4.191783853	1.452371584	0.003030357	0.071769533	2.819132663	1.49525137	3.059100398	1.613107456	-0.7871122	-0.3664964	0.45703365	0.74863513	0.76106732	-0.393904	0.92188009	-0.117349	-34.066424
8-iso-PGA1 [M-H+Acetate]	395.2441	-9.683481292	-2.375632867	2.64E-05	0.016446553	0.16347599	-2.612849334	0.182596019	-2.453272784	3.301898152	1.363230146	0.010828165	0.127143886	2.844758625	1.508306247	3.109595618	1.63672698	-0.6058438	-0.3101203	0.56373872	0.80205641	0.83680832	-0.2570309	1.01282725	0.01838813	-31.973869
8-iso-PGA2 [M-H+Acetate]	393.2288	-8.563839231	-2.079593988	5.89E-05	0.016446553	0.198501762	-2.332776284	0.222422877	-2.168622915	2.744266016	1.247752948	0.025281968	0.198153498	2.793097562	1.481865967	3.042395754	1.605207831	-0.5269555	-0.3159753	0.61450562	0.83097556	0.919561	-0.1209828	1.11212398	0.15331763	-23.501453
8(9)-EpETE [M-H]	317.2124	-4.249009024	-1.529960053	0.003797778	0.073706276	0.290120013	-1.785278274	0.326021287	-1.616961928	2.24900291	0.740290429	0.054652275	0.290873392	1.702829415	0.767933917	1.83302596	0.874227218	-1.0051991	-0.4992669	0.34827614	0.69479002	0.67605107	-0.5647959	0.82191235	-0.2829435	-9.5560337
ASPARAGINE	133.0605	-2.977728398	-0.582348143	0.020581009	0.184860554	0.577697195	-0.791614606	0.633500653	-0.658581989	3.793548215	0.725262041	0.005285026	0.087859906	1.686218963	0.753791889	1.807750773	0.854195794	-0.2335305	-0.0554793	0.82203184	0.93199556	0.82828436	-0.2718019	0.98806725	-0.0173189	-11.296156
CITRULLINE	176.1026	4.661421136	0.429668822	0.002310647	0.054200457	1.194316521	0.256185234	1.318489724	0.398886327	-2.770565359	-0.390522801	0.024275488	0.194608996	0.760754241	-0.394497623	0.816080038	-0.293217442	0.64384809	0.0433333	0.54018754	0.7903383	0.90462814	-0.1446032	1.08263311	0.11454442	-12.914772
Docosadienoic Acid [M-H]	335.2958	2.609069045	0.679911269	0.034960662	0.245666463	1.318881333	0.399314763	1.463346657	0.549271575	-3.78999729	-0.480669639	0.005311849	0.087859906	0.72194382	-0.470041521	0.773238206	-0.371015172	1.74644704	0.51719605	0.12423529	0.53580578	1.18607149	0.24619097	1.42702655	0.51301218	-9.8883646
Palmitoleic Acid [M-H+Acetate]	313.2386	3.572389634	0.933298617	0.009067905	0.119217362	1.673192926	0.742603804	1.843632497	0.882551103	-2.688462449	-0.524218237	0.02756221	0.204707484	0.675658631	-0.565633569	0.728937681	-0.456132616	2.9267301	0.59257942	0.022127	0.30919845	1.27695463	0.35270727	1.5331941	0.61654035	-9.6042354
PGA3 [M-H]	331.1919	-6.208265981	-1.30577215	0.000441685	0.026405285	0.35730134	-1.484786769	0.398289501	-1.328110643	5.217256751	0.971840304	0.000805273	0.04269255	1.904970281	0.929768491	2.043952598	1.031361738	-1.4262389	-0.2700143	0.19683715	0.59576827	0.71611731	-0.4817322	0.86469659	-0.2097341	-32.390118
PGA3 [M-H+Acetate]	391.2135	-6.402555096	-1.153095019	0.00036639	0.024987604	0.399959106	-1.322075598	0.444468223	-1.169847818	5.400141518	0.922625196	0.000645908	0.040614693	1.851390334	0.888609095	1.980130841	0.985595762	-1.0122475	-0.1982719	0.34512255	0.69368379	0.76433131	-0.38773	0.92121008	-0.1183979	-34.574704
SERINE	104.0354	-4.77928356	-0.748540613	0.002013948	0.049746038	0.515449756	-0.956096289	0.569274762	-0.812802953	3.426564039	0.689154187	0.009000343	0.11470906	1.66056085	0.73167059	1.769267239	0.823151976	-1.3248318	-0.1862435	0.22683661	0.61978339	0.75183641	-0.4115093	0.90180216	-0.1491171	-16.376521
SERINE(M-H2O+H)	88.0398	-2.464654888	-0.386963421	0.043169789	0.2686166	0.675979325	-0.564948972	0.739769966	-0.434851365	2.494844146	0.512321687	0.037240034	0.239448539	1.443930836	0.530001639	1.551789851	0.633933196	0.06492255	0.01008251	0.95005084	0.98447188	0.87426877	-0.1938512	1.04060531	0.05742297	-6.1489298
Tricosenoic Acid [M-H+Acetate]	411.3483	5.225814203	0.872408464	0.00121802	0.038328302	1.604698802	0.682302532	1.752592271	0.809490402	-3.552287783	-0.506353344	0.007484418	0.102318431	0.702560209	-0.509306227	0.755886502	-0.403758469	1.03428496	0.20911261	0.33540662	0.69185437	1.00746168	0.01072497	1.19568091	0.25783243	-18.563596
unknown oxylipin (EIC_131)	331.1918	-2.826901572	-0.593138872	0.025519336	0.208014489	0.603430155	-0.728741302	0.66607047	-0.586253273	2.51297556	0.47114801	0.03620273	0.237523793	1.340898564	0.423200104	1.435754438	0.521809021	0.33783529	0.06112597	0.7453925	0.90146597	0.92373528	-0.1144486	1.10668998	0.14625113	-7.1039346
unknown oxylipin (EIC_138;EIC_139;EIC_140;EIC_145)	333.2075	-6.387465832	-2.718358818	0.000371691	0.024987604	0.119063842	-3.070192741	0.134260476	-2.896893431	4.798548333	1.626537347	0.001358054	0.055133274	3.223857196	1.68878784	3.461423809	1.791365593	-0.6913604	-0.5004977	0.51160896	0.77676416	0.77836491	-0.3614814	0.94749602	-0.0778082	-30.650564
unknown oxylipin (EIC_138;EIC_140)	333.2074	-5.508318642	-2.213862822	0.000898589	0.03487916	0.182234402	-2.456132756	0.204192037	-2.29200149	3.595988275	1.063147627	0.007023104	0.098915041	2.035035111	1.025053686	2.188767928	1.130118996	-1.1633375	-0.7575291	0.28280992	0.65902536	0.67069683	-0.5762673	0.81439256	-0.2962037	-19.807849
unknown oxylipin (EIC_138)	333.2075	-8.393902823	-2.15515849	6.70E-05	0.016446553	0.191645237	-2.383489949	0.213855463	-2.225292034	6.521974863	1.460133671	0.000183803	0.028695533	2.760918192	1.46514814	2.958483845	1.564858017	-0.4528676	-0.2257202	0.66434696	0.85737067	0.87201141	-0.1975811	1.05335357	0.07498978	-54.744823
unknown oxylipin (EIC_143)	333.2074	-3.76211296	-1.207349357	0.007055346	0.103156747	0.365673789	-1.451370874	0.40964694	-1.287547058	2.723969017	0.536702173	0.026088033	0.199662582	1.404447921	0.490003128	1.505459339	0.590203743	-2.8490129	-0.8245512	0.02472336	0.31988296	0.46561487	-1.1027909	0.565683	-0.8219343	-10.247879
unknown oxylipin (EIC_144;EIC_148;EIC_149)	333.2074	-6.528266897	-1.686937824	0.000325382	0.024987604	0.269200608	-1.893226425	0.300252854	-1.735750138	5.493753265	0.995315291	0.000577989	0.039674412	1.937452392	0.95416086	2.086594805	1.061150472	-2.1821237	-0.5189346	0.06543523	0.44035412	0.59545034	-0.7479469	0.72205936	-0.4698106	-35.864688
unknown oxylipin (EIC_148)	333.2074	-5.410469799	-1.48947753	0.000997233	0.035647699	0.308238837	-1.697879449	0.344398311	-1.537850027	3.863321737	0.723626902	0.004786361	0.085395354	1.600722488	0.678723215	1.71982441	0.782261276	-2.5999054	-0.6755777	0.03543004	0.35835614	0.54004669	-0.888844	0.65551416	-0.6093012	-20.902386
unknown oxylipin (EIC_156;EIC_159;EIC_160;EIC_162)	335.2232	-3.605271458	-1.118154882	0.008678652	0.11644382	0.379021895	-1.399646904	0.424398406	-1.236508857	2.504654405	0.501684095	0.0366751	0.238281789	1.418894048	0.504766864	1.523422238	0.60731586	-1.3829931	-0.5072342	0.20917142	0.60703462	0.60661371	-0.72115	0.73687036	-0.4405173	-9.029959
unknown oxylipin (EIC_170;EIC_172;EIC_173)	335.2231	-3.131460309	-1.404521164	0.016575835	0.165276793	0.280046864	-1.836259821	0.316560215	-1.659448145	2.28060569	0.469658056	0.05202212	0.283143399	1.428293068	0.514292033	1.524257062	0.60810623	-1.5176229	-0.8522581	0.17289884	0.57867838	0.4668765	-1.0988871	0.5694892	-0.8122596	-7.1416262
unknown oxylipin (EIC_171)	335.2231	-4.847533076	-1.521987172	0.001861583	0.04817216	0.302756266	-1.723771277	0.338557789	-1.562525983	4.321702067	0.961133185	0.002540232	0.068025432	1.871983658	0.90456784	2.00959425	1.006904241	-2.2574278	-0.557029	0.05855309	0.41902752	0.56886811	-0.8138339	0.69174896	-0.5316795	-20.949594
unknown oxylipin (EIC_219)	349.2023	-7.623015857	-2.771651035	0.000123888	0.020000824	0.117861105	-3.084840397	0.131819869	-2.923360252	3.132525828	1.07230783	0.013961913	0.145102942	2.165676462	1.11481773	2.35918701	1.238289784	-1.1285394	-0.7564243	0.29628013	0.66666688	0.66667447	-0.5849456	0.8052937	-0.3124131	-23.879294
unknown oxylipin (EIC_232;EIC_237;EIC_238;EIC_240;EIC	351.218	-3.573648203	-1.132991191	0.009052228	0.119217362	0.373348509	-1.421405124	0.418079095	-1.258152188	4.141576418	0.722814581	0.003246339	0.073250429	1.641604	0.715106151	1.750392993	0.807678868	-1.2371945	-0.3667864	0.25590511	0.63950589	0.62319884	-0.6822356	0.75702322	-0.4015905	-14.800686
unknown oxylipin (EIC_328;EIC_332;EIC_335;EIC_339)	375.2173	-5.230974036	-1.006515716	0.001211157	0.038328302	0.436287656	-1.196648439	0.487436889	-1.036712658	3.25079109	0.602619146	0.011687231	0.1326619	1.512377151	0.596817958	1.617145691	0.693449659	-0.757473	-0.1047567	0.4734886	0.75593015	0.79836369	-0.324882	0.96437111	-0.0523397	-17.004804
unknown oxylipin (EIC_79)	315.1967	-5.49484778	-1.508184197	0.0009115	0.034935692	0.299097213	-1.741313629	0.336301203	-1.572174154	4.058010716	0.765684937	0.00364348	0.075266337	1.674326881	0.743581214	1.791962827	0.84154071	-1.7811903	-0.4337221	0.11808927	0.53070997	0.61970486	-0.6903468	0.7535193	-0.4082836	-22.298151
unknown oxylipin (EIC_83;EIC_93;EIC_94)	317.2124	-6.174747661	-2.334963942	0.000456356	0.026405285	0.159606591	-2.647407861	0.180273697	-2.471739179	2.717392762	1.486447759	0.026354828	0.200069657	3.454606307	1.788521308	3.769110077	1.914223929	-0.2517801	-0.1367615	0.80844314	0.92779785	0.86272897	-0.2130207	1.0525388	0.07387341	-16.779215
unknown oxylipin (EIC_87)	317.2124	-3.288239351	-1.637862566	0.013334826	0.150183761	0.26702027	-1.904978831	0.300339588	-1.735333444	2.254499977	0.749553679	0.054185462	0.289863328	1.582509313	0.66221399	1.703148732	0.768204428	-0.5171149	-0.2488146	0.62100925	0.83410944	0.70831696	-0.497533	0.86525698	-0.2087994	-7.4133355
unknown oxylipin (Novel EIC_1)	269.2125	2.845118121	0.525137123	0.024861647	0.205304457	1.25154402	0.323709036	1.391861058	0.477015202	-3.186344055	-0.485566416	0.012873993	0.139170901	0.71429619	-0.48540567	0.764175693	-0.388023726	-0.5105309	-0.0767723	0.62538098	0.83627927	0.80841894	-0.306825	0.97583321	-0.0352935	-9.0655252
unknown	636.436	-6.190781204	-4.643492019	0.000449271	0.026405285	0.031040263	-5.009715426	0.035384215	-4.820750268	4.162791704	2.744560251	0.003153115	0.072454858	6.884517047	2.783355452	7.483866104	2.903783747	-0.6950395	-0.7740398	0.50943685	0.77541747	0.93010014	-0.104542	1.13598604	0.18394511	-25.770933
unknown	635.4326	-6.394852059	-4.667878873	0.000369086	0.024987604	0.029886024	-5.064385193	0.034156897	-4.871679253	3.844793481	2.676723788	0.004913667	0.086584022	6.988322915	2.804946273	7.579582472	2.922118378	-0.707815	-0.7920672	0.50194033	0.77156636	0.92113781	-0.1185111	1.1251594	0.17012941	-24.586886
unknown	410.2258	-7.235455979	-4.076352674	0.000172081	0.022002789	0.052709756	-4.245786168	0.058565098	-4.093815054	5.528267123	2.546504009	0.000554958	0.039241075	5.439272178	2.443413619	5.82269653	2.54168743	-1.1471199	-0.8610851	0.28902338	0.66344884	0.67364882	-0.5699314	0.81340267	-0.2979584	-39.999533
unknown	480.2679	-4.771313766	-3.673357634	0.002032623	0.04982982	0.068357129	-3.870764395	0.076943234	-3.700061715	2.96906059	1.901333401	0.017896799	0.166959983	3.342642943	1.740989256	3.653458235	1.869262715	-0.9760188	-0.9271815	0.3615706	0.69975778	0.68149155	-0.5532323	0.83336112	-0.2629863	-14.16632
unknown	299.2017	-4.312114141	-3.139989	0.003513983	0.069861832	0.079654495	-3.650100405	0.091350084	-3.452450133	2.93521369	2.218523043	0.018847158	0.171411883	5.714225373	2.514557938	6.180197597	2.627652966	0.01568864	0.01366352	0.98792058	0.99670416	0.94262898	-0.0852381	1.15737235	0.21085309	-12.656976
unknown	453.2822	3.030554255	2.251401496	0.019099655	0.17651305	3.709445139	1.891203404	3.965578382	1.987531299	-5.772677534	-2.808570001	0.000418001	0.034499987	0.140141804	-2.835040724	0.14673632	-2.768702086	-1.6576054	-2.2082882	0.14136368	0.55232629	0.27912534	-1.841015	0.32129144	-1.6380456	-17.494412
unknown	479.265	-5.481006403	-3.460571728	0.000924982	0.035068641	0.060978261	-4.035561176	0.069257263	-3.85189082	3.793657566	1.764255065	0.005284203	0.087859906	3.791289311	1.922688552	4.093617482	2.033376299	-0.9147001	-0.9409955	0.39076852	0.70846289	0.68468562	-0.5464864	0.83863855	-0.2538789	-20.793061
unknown	409.2225	-8.586507268	-3.489364969	5.79E-05	0.016446553	0.072911205	-3.777715651	0.081569674	-3.615823305	6.390671471	1.683821279	0.000211223	0.028695533	3.129205746	1.645796519	3.357380862	1.747336206	-1.2812643	-0.9603363	0.24091008	0.63015429	0.63740519	-0.6497173	0.76863318	-0.3796328	-54.873547
unknown	438.2946	3.218413102	1.969141857	0.014685684	0.156123148	3.809802572	1.929716238	4.188423236	2.066407233	-4.094950336	-2.978156453	0.003461826	0.074729546	0.14425144	-2.793342373	0.149274359	-2.743961723	-2.6644922	-1.9030149	0.03225627	0.35159075	0.31566364	-1.66354	0.36995309	-1.4345858	-13.179242
unknown	437.2912	3.395744344	2.060757381	0.011508996	0.138981779	4.130691253	2.046383231	4.520112571	2.176358703	-5.482039681	-2.830936279	0.000586043	0.039808166	0.126981393	-2.977310983	0.133091624	-2.909508314	-2.989567	-1.4768719	0.02023875	0.30366888	0.35067958	-1.5117747	0.41054787	-1.2843776	-18.615605
unknown	347.2064	-3.305117707	-3.062213771	0.013028698	0.148531711	0.08589972	-3.541202762	0.097427346	-3.359529423	2.705315969	1.901973162	0.026852101	0.201199758	3.569729894	1.835814916	3.910290335	1.96727573	-2.6113934	-1.8889074	0.03484264	0.35553892	0.14732547	-2.7629212	0.18318396	-2.4486349	-8.9413877
unknown	633.4164	-4.75969254	-3.186199873	0.002060198	0.050316665	0.08979995	-3.477141541	0.101905768	-3.294692386	2.829468646	1.814935156	0.022168335	0.185032826	3.63300501	1.861163355	3.919024346	1.970494536	-1.3718085	-0.8167347	0.21247112	0.61251221	0.44131414	-1.1801221	0.54244433	-0.882453	-13.467401
unknown	377.2703	3.408390247	2.04978519	0.011312568	0.137835846	4.111893251	2.039802811	4.499647016	2.169811831	-5.360568074	-2.790997482	0.000677211	0.040614693	0.132134888	-2.91991666	0.13813961	-2.855801041	-2.8982442	-1.4080538	0.02304357	0.31252191	0.36122273	-1.4690394	0.42402702	-1.2377719	-18.270908
unknown	634.4181	-4.66567546	-3.102907467	0.002299133	0.054200457	0.084864935	-3.558687617	0.096435823	-3.374287033	3.800738204	1.835932983	0.005231159	0.087859906	3.565715696	1.834191677	3.870563913	1.952543772	-1.3187025	-0.8720434	0.22877229	0.62151099	0.41432003	-1.2711825	0.51038525	-0.9703414	-17.733011
unknown	393.2653	3.254667567	2.264886235	0.013966895	0.151808603	5.100654212	2.3506823	5.56305306	2.475876866	-2.550297563	-2.484228137	0.034158843	0.230176907	0.227871927	-2.133704892	0.247964358	-2.011795331	-1.2835742	-1.4955565	0.2401454	0.62966953	0.68612198	-0.543463	0.80661547	-0.310047	-8.3003708
unknown	299.1999	-5.20505818	-3.08722063	0.001246067	0.038328302	0.091506365	-3.449984084	0.104242609	-3.261982999	2.471579455	1.820476104	0.038615245	0.243765741	4.32681135	2.11330422	4.681085379	2.226843079	-0.2165869	-0.1613162	0.83470708	0.93421055	0.909991	-0.1360758	1.11246706	0.15376262	-12.864715
unknown	378.2738	3.283606795	1.952205853	0.01342019	0.150884589	3.934637305	1.976230654	4.308563117	2.107206819	-3.108571815	-2.831298791	0.01447674	0.147504409	0.185650019	-2.429342628	0.194587197	-2.361511303	-1.2676683	-1.185401	0.24545367	0.63471409	0.58185762	-0.7812619	0.69156094	-0.5320717	-10.207328
unknown	396.2471	-7.703237703	-2.867251889	0.000115938	0.019884795	0.119395338	-3.066181591	0.133805168	-2.901794254	4.307651055	1.922704134	0.002588856	0.068625739	3.952974436	1.982938625	4.289665466	2.100865142	-0.6320715	-0.3668212	0.54742064	0.79317667	0.84731847	-0.2390238	1.02139091	0.03053513	-33.18286
unknown	300.2044	-5.654950558	-3.076732059	0.000770505	0.03200293	0.092154834	-3.439796342	0.104345332	-3.26056204	2.59501205	1.748094699	0.031863882	0.220110567	4.144011658	2.051028062	4.530362025	2.179626342	-0.1925091	-0.1400487	0.85280941	0.94047402	0.92768302	-0.1082962	1.13355137	0.18084977	-14.674665
unknown	395.2441	-8.981213834	-2.779980448	4.32E-05	0.016446553	0.124019648	-3.011359399	0.138829165	-2.848617416	4.487930092	1.821365664	0.002034317	0.061338495	3.767148553	1.913472925	4.093808704	2.03344369	-0.6985746	-0.4049734	0.50735529	0.77541747	0.82526751	-0.2770662	0.99524489	-0.0068765	-40.30706
unknown	346.2029	-4.158033147	-2.93184255	0.004252191	0.078232544	0.073407038	-3.767937793	0.08442999	-3.56610064	3.637960455	1.720094064	0.006608438	0.096587859	4.096691638	2.034459304	4.422675479	2.144919386	-2.6252252	-1.8627445	0.03414878	0.353467	0.15497232	-2.6899175	0.19245639	-2.3773965	-15.12676
unknown	335.223	-3.050327159	-2.265064804	0.018574656	0.175543961	0.152848494	-2.709825755	0.171893262	-2.540415102	5.669456717	2.150009945	0.000470695	0.035044854	4.03449369	2.012387634	4.351767508	2.121601483	-0.0439096	-0.030796	0.96620277	0.9896738	0.69680969	-0.5211634	0.85769212	-0.2214682	-17.293698
unknown	397.2513	-9.752914411	-2.847748985	2.52E-05	0.016446553	0.125497371	-2.994270951	0.140171817	-2.834731789	3.685566003	1.692738694	0.006169475	0.094611384	3.404262905	1.767342458	3.702906939	1.888658292	-1.0416233	-0.5615274	0.33221954	0.68884755	0.7703197	-0.3764708	0.92518195	-0.112191	-35.94501
unknown	369.1855	-9.110926887	-3.264310897	3.94E-05	0.016446553	0.091704455	-3.446864364	0.102195824	-3.290591848	2.29940412	1.448833626	0.050517801	0.280124644	3.64737659	1.866859165	3.820521192	1.933769463	-1.1681927	-1.1687963	0.28097144	0.65902536	0.74558349	-0.4235582	0.9000009	-0.1520017	-20.949703
unknown	336.2265	-8.637280579	-2.703169978	5.57E-05	0.016446553	0.130889016	-2.93358406	0.146559834	-2.770438319	4.353145327	1.739670135	0.002434963	0.066159964	3.534942552	1.821686769	3.841897724	1.941819113	-0.6756359	-0.3838922	0.52095925	0.78249353	0.82451333	-0.2783853	0.99510264	-0.0070828	-37.599338
unknown	289.1812	-4.085008054	-2.676234293	0.004660174	0.081404749	0.152816234	-2.710130284	0.171466626	-2.544000298	3.029782005	1.744717333	0.016314826	0.157847147	2.764728121	1.467137615	3.02889399	1.598791085	-0.2334547	-0.1166481	0.8220884	0.93199556	0.79065124	-0.3388866	0.96854464	-0.0461096	-12.376684
unknown	429.196	-6.191166588	-2.917817998	0.000449103	0.026405285	0.103470412	-3.272709811	0.116385909	-3.103011698	3.201011387	1.590742853	0.012593195	0.13808775	3.405856786	1.768017772	3.636031713	1.862364783	-1.054825	-0.9087497	0.32654634	0.68447723	0.67012886	-0.5774895	0.81578237	-0.2937438	-19.817995
unknown	335.2232	-8.947900096	-2.686993132	4.43E-05	0.016446553	0.131897552	-2.922510305	0.14767148	-2.759536874	4.364995115	1.724714269	0.002396523	0.066159964	3.519303713	1.815290023	3.825767148	1.935749068	-0.6675933	-0.3757905	0.52578313	0.78566106	0.82706957	-0.2739194	0.99820512	-0.0025918	-39.05754
unknown	394.2318	-6.505034231	-2.746832424	0.000332556	0.024987604	0.116980056	-3.09566551	0.131865281	-2.922863324	4.491042109	1.64566834	0.002025955	0.061338495	3.317192448	1.729962713	3.55707933	1.83069315	-0.7210203	-0.5178312	0.49426726	0.76821474	0.76832608	-0.3802094	0.9350851	-0.0968304	-29.214383
unknown	475.268	2.851886005	1.587561407	0.024621866	0.204273774	2.481093182	1.31097592	2.718880352	1.443012665	-7.381270018	-2.824324808	7.76E-05	0.02086171	0.140069275	-2.835787571	0.147956942	-2.756750711	-2.9631204	-1.8850577	0.02101181	0.30521805	0.23835965	-2.0687881	0.28171994	-1.8276664	-21.050541
unknown	446.2042	-2.783212628	-2.551295936	0.02717234	0.216614709	0.119336086	-3.066897725	0.134042571	-2.899236828	3.308406711	1.757421981	0.010723637	0.126225337	2.9005349	1.536318979	3.159120282	1.659522868	0.04604554	0.0408081	0.96456003	0.98959975	0.70837064	-0.4974237	0.87003494	-0.2008548	-9.2079993
unknown	521.3493	-3.765738423	-2.859237164	0.007021958	0.103008133	0.096478324	-3.373651346	0.109359046	-3.192855528	2.699271749	1.559232179	0.027104585	0.201768261	3.037051001	1.602671136	3.193204872	1.675005118	-1.0703271	-0.9361532	0.31998333	0.68061866	0.47430642	-1.0761087	0.58030812	-0.785109	-10.164751
unknown	636.4358	-3.023631178	-2.339070281	0.019287178	0.176645631	0.152622991	-2.711955789	0.17348802	-2.527092054	2.944708661	1.888822638	0.018575377	0.170126451	3.851169204	1.94529651	4.189475958	2.066769795	-0.6571244	-0.8297483	0.532104	0.78645743	0.97274493	-0.0398665	1.18882985	0.24954225	-8.9037129
unknown	334.2109	-6.371981287	-2.717526698	0.00037722	0.025100554	0.11925436	-3.067886082	0.134485755	-2.89447473	4.713751268	1.621052029	0.001514324	0.05569795	3.215099137	1.684863224	3.451596956	1.78726401	-0.7010096	-0.5082609	0.50592474	0.7739425	0.77581435	-0.3662166	0.94425386	-0.0827533	-30.035935
unknown	393.2286	-6.467939236	-2.723571951	0.00034438	0.024987604	0.119043893	-3.070434482	0.134154302	-2.898034776	4.573607967	1.605827131	0.00181717	0.059172455	3.198432508	1.677365041	3.432362362	1.779203971	-0.704046	-0.5058192	0.50414449	0.77319701	0.77889779	-0.3604941	0.94770267	-0.0774936	-29.581818
unknown	315.1967	-7.189211047	-2.570006527	0.000179129	0.022463467	0.13821861	-2.854976217	0.155960206	-2.680750127	3.117649447	1.642822332	0.01427934	0.146408135	3.729453611	1.898964282	4.066924056	2.023938051	-0.2088531	-0.1179799	0.84051046	0.93577972	0.93214664	-0.1013712	1.13393369	0.18133628	-22.41344
unknown	397.2	-8.551468591	-2.448206878	5.94E-05	0.016446553	0.156687194	-2.674040824	0.176176055	-2.504910241	3.185959767	1.713071505	0.012881437	0.139170901	3.981900634	1.99345722	4.341707281	2.118262462	-0.0147615	-0.0073789	0.98863433	0.99670416	1.01669185	0.02388248	1.2321938	0.30122918	-27.244635
unknown	457.2581	2.494448808	2.03305835	0.04132658	0.264277757	4.457672448	2.156290611	4.876817567	2.285940005	-2.658876384	-2.045527505	0.028855721	0.209170025	0.217117019	-2.203455277	0.230945276	-2.114377058	-0.043781	-0.0411268	0.96630166	0.9896738	1.0933473	0.12875175	1.27860187	0.35456711	-6.632431
unknown	333.1356	-2.39372699	-2.245906904	0.047905692	0.28280061	0.118492017	-3.077138235	0.133408979	-2.906072325	2.826987021	1.846242693	0.022253183	0.185329509	4.201751347	2.070990788	4.550435562	2.186004645	-0.6957075	-0.6068323	0.50904308	0.77541747	0.33081166	-1.595918	0.4089205	-1.2901077	-6.7670351
unknown	316.1999	-7.061016629	-2.526196042	0.000200427	0.022534203	0.144217214	-2.793684719	0.162614626	-2.620471068	3.182197234	1.633480438	0.012954668	0.139170901	3.624076992	1.857613605	3.948727439	1.981387789	-0.2264791	-0.1276083	0.82730017	0.93337638	0.93852102	-0.091539	1.14092403	0.19020273	-22.469548
unknown	335.06	-4.396295842	-2.509085765	0.003171072	0.066445461	0.129068865	-2.953787073	0.14580876	-2.777850696	5.324836467	1.636243263	0.000706908	0.040644607	3.068485601	1.617526814	3.274715137	1.711369414	-1.5952595	-0.9442751	0.15468426	0.56601708	0.39753395	-1.33085	0.48787335	-1.0354214	-23.409556
unknown	430.1993	-6.692773298	-2.704901202	0.000279288	0.02428314	0.11693669	-3.09620043	0.131912633	-2.922345358	4.18326214	1.513883809	0.003065896	0.071769533	3.19070038	1.67387314	3.387437575	1.76019436	-1.0495764	-1.0391495	0.32879253	0.68623062	0.69328915	-0.5284709	0.84344468	-0.2456346	-27.997625
unknown	431.2101	-6.073313103	-2.594820617	0.000504189	0.027624042	0.155148639	-2.688277056	0.171413613	-2.544446408	3.183779399	1.559000672	0.012923763	0.139170901	2.897097077	1.534608028	3.189933468	1.673526334	-0.7553347	-0.3928765	0.47469105	0.75646636	0.78757112	-0.3445179	0.95150084	-0.0717232	-19.336089
unknown	459.2735	2.609817869	1.788682811	0.034922592	0.245666463	2.939306934	1.555476019	3.234727975	1.693644394	-5.165641838	-2.248914749	0.000857705	0.044389646	0.187594642	-2.414309472	0.196802456	-2.345179867	-2.6838842	-1.4573633	0.03136229	0.34722158	0.2692306	-1.8930857	0.31871991	-1.6496389	-13.481384
unknown	417.2264	-5.208513323	-2.113337941	0.001241348	0.038328302	0.201059069	-2.314308685	0.222812135	-2.166100287	4.570788265	1.897366539	0.001823902	0.059172455	3.777480023	1.917424126	4.085004352	2.030337615	0.39188217	0.19214418	0.7068023	0.88071901	1.09519687	0.13119022	1.30726015	0.38654627	-23.807012
unknown	398.2029	-4.390020039	-2.389214496	0.003195323	0.066570926	0.188035412	-2.410923708	0.211451966	-2.241598124	2.652922493	1.664616004	0.029123455	0.209836299	3.140143633	1.650830551	3.422698798	1.775134339	-0.5199679	-0.3181654	0.61911989	0.83364691	0.90415675	-0.1453552	1.09515846	0.13113964	-11.646383
unknown	377.2337	-3.522528612	-2.397119002	0.009694645	0.12495252	0.140563565	-2.830705407	0.161524529	-2.630174824	2.252358354	1.648230916	0.054366856	0.290595304	4.037004554	2.013285214	4.369907621	2.127602782	0.23498228	0.16816523	0.82094835	0.93192367	0.92500179	-0.1124719	1.13785413	0.18631562	-7.9339967
unknown	509.2388	-3.586715059	-2.256013608	0.008896047	0.11766962	0.175836212	-2.507695883	0.197192536	-2.34232315	2.48793642	1.743571125	0.037643104	0.239448539	3.715726371	1.893644264	4.080259226	2.028660812	-0.3499495	-0.3641833	0.73666945	0.89757502	1.28537036	0.36218411	1.56604829	0.6471287	-8.923519
unknown	432.1882	-3.986279333	-2.050061183	0.005281428	0.087634077	0.181299527	-2.463552931	0.205840517	-2.280401107	4.796731888	1.912958811	0.001361211	0.055133274	4.101496828	2.036150513	4.340931314	2.118004595	0.34585707	0.29824146	0.73961168	0.89897629	1.48606915	0.57150125	1.81233293	0.85784801	-19.121113
unknown	635.4327	-6.882724922	-2.334194963	0.000234951	0.02428314	0.18687853	-2.419827262	0.207317399	-2.270086894	4.744691056	1.649081319	0.001455148	0.055229968	2.956355857	1.563819938	3.180344759	1.669183166	-1.7998687	-0.6633539	0.11490653	0.52398985	0.59972969	-0.7376157	0.71945754	-0.4750185	-32.656403
unknown	487.2693	6.725733286	2.263938092	0.000270962	0.02428314	4.42042258	2.144184294	4.916399026	2.297602013	-3.102430669	-1.679576564	0.014611903	0.147912784	0.348101942	-1.522418233	0.370942133	-1.43073395	0.25741001	0.05476102	0.80426513	0.92562883	0.89204144	-0.1648174	1.07918754	0.10994559	-20.866121
unknown	336.2265	-7.672394401	-2.450932176	0.000118925	0.019884795	0.157710814	-2.66464651	0.176906211	-2.498943391	4.182692917	1.51343299	0.003068286	0.071769533	2.910782903	1.541407242	3.157766616	1.658904549	-0.7710964	-0.3604064	0.465876	0.75402765	0.76316028	-0.389942	0.92385306	-0.1142647	-32.09127
unknown	416.2145	-5.898201674	-2.434228491	0.000600569	0.029445934	0.147160949	-2.764533212	0.165977281	-2.59094232	4.309249697	1.51308684	0.002583274	0.068625739	3.051017684	1.609290542	3.231533129	1.692218782	-0.7886787	-0.5342055	0.45617501	0.7482908	0.73121217	-0.451638	0.88943641	-0.1690366	-25.416824
unknown	431.1845	-3.714015172	-2.043428698	0.007515005	0.106765467	0.185250804	-2.43244829	0.208824851	-2.259634689	4.899909892	1.779938341	0.001193906	0.052604473	3.496375307	1.805860054	3.744106599	1.904621511	0.23871829	0.21518906	0.81816208	0.93192367	1.51673274	0.60096689	1.84842265	0.88629468	-18.19834
unknown	347.1869	-6.054039969	-2.420964498	0.000513898	0.027695294	0.146772364	-2.768347747	0.163481483	-2.61280086	6.244541702	1.475924148	0.000247168	0.029691542	2.812679351	1.491945093	3.02705866	1.597916633	-0.6335927	-0.3730985	0.54648305	0.79278836	0.73527355	-0.443647	0.88605912	-0.1745251	-37.804705
unknown	314.1848	-4.803573797	-2.207250497	0.001958193	0.049074945	0.205987414	-2.279371906	0.229427828	-2.123887701	2.739019221	1.61081658	0.025487849	0.198573795	3.148112251	1.654486983	3.461712975	1.79148611	0.32152841	0.17114047	0.75719791	0.90805432	1.1305849	0.17706933	1.37293624	0.45726463	-13.157081
unknown	429.1672	-2.370738736	-1.981123918	0.049552352	0.285863059	0.280564938	-1.833593368	0.312874626	-1.676343434	2.525509245	1.762005387	0.035502847	0.23503966	2.609959209	1.384027259	2.770830242	1.470318325	-0.0554661	-0.032581	0.95731705	0.98823246	0.81108108	-0.302082	0.99376652	-0.0090212	-5.9873226
unknown	469.332	-5.837542818	-2.207057291	0.000638633	0.02957347	0.170212453	-2.554591508	0.191958798	-2.38113141	3.626072058	1.581615979	0.006723176	0.096825003	3.36407347	1.750209214	3.655628953	1.870119644	-0.567557	-0.2971009	0.58806775	0.81591273	0.73494982	-0.4442824	0.89707204	-0.1567043	-21.167351
unknown	474.3672	2.910262267	1.679149305	0.022652077	0.194873605	2.423981519	1.2773787	2.737388064	1.452799972	-3.907488817	-2.077104336	0.00449693	0.083044401	0.254885558	-1.972078461	0.27265445	-1.874854394	-0.0709103	-0.0638928	0.94545255	0.98293743	1.02119848	0.0302633	1.23103555	0.29987242	-11.371817
unknown	318.2156	-6.265463747	-2.33870247	0.000417855	0.026388034	0.158399317	-2.658361981	0.178870991	-2.483008662	2.724956951	1.483965	0.026048193	0.199600786	3.469389904	1.794681985	3.786770492	1.920967985	-0.2699812	-0.1475516	0.79496084	0.92339502	0.85826676	-0.220502	1.0470223	0.06629217	-17.073119
unknown	320.2215	-6.646795526	-2.39957481	0.000291386	0.024360587	0.15703399	-2.670851227	0.17692024	-2.498828989	3.929084321	1.416353576	0.004362297	0.081386137	2.76875209	1.469235883	2.989599445	1.579952201	-0.649714	-0.3668548	0.53660673	0.78834352	0.78726786	-0.3450735	0.95608743	-0.0647855	-26.11582
unknown	533.386	-2.622806221	-2.272828273	0.034269084	0.243608738	0.093793293	-3.414371428	0.107754342	-3.214182092	5.75801599	1.45883282	0.000425074	0.034499987	2.677805428	1.421051137	2.850835548	1.511384819	-0.8179269	-0.8647196	0.44034824	0.73893743	0.36835145	-1.4408452	0.45230572	-1.1446299	-15.10216
unknown	415.2109	-5.34279343	-2.2557628	0.001072511	0.03720363	0.169166154	-2.563487145	0.189959027	-2.39623982	3.690819362	1.452631004	0.006122975	0.094611384	2.970895427	1.570897824	3.149706365	1.655217338	-0.7564065	-0.4898998	0.47408806	0.75593015	0.73728265	-0.4397103	0.89711175	-0.1566404	-19.719285
unknown	273.1493	-4.774403683	-2.551567411	0.00202536	0.04982982	0.158729416	-2.655358584	0.177014133	-2.498063543	2.418908075	1.273891411	0.041921448	0.253825223	2.244514624	1.166403496	2.465857628	1.302089505	-1.2701515	-0.5086201	0.24461838	0.63425704	0.61145601	-0.7096794	0.74504008	-0.4246101	-11.548844
unknown	433.2001	-5.490502488	-1.942459462	0.000915709	0.034935692	0.216044796	-2.210597616	0.243346799	-2.038914296	3.454654691	1.666604844	0.008635433	0.111951608	3.52686581	1.818386685	3.84829737	1.944220285	0.68260874	0.41706953	0.51679969	0.78010434	1.44427541	0.53034588	1.75211215	0.80909512	-18.96779
unknown	393.2286	-8.584901214	-2.188627095	5.79E-05	0.016446553	0.187082051	-2.418256948	0.209000085	-2.258424567	6.417825279	1.477491964	0.000205202	0.028695533	2.808409949	1.489753544	3.004863099	1.587299263	-0.4550244	-0.2259968	0.66286824	0.8570283	0.87115358	-0.199001	1.05278058	0.07420479	-55.096396
unknown	394.2317	-8.588163051	-2.179160451	5.78E-05	0.016446553	0.18887936	-2.404463038	0.210890523	-2.245433828	6.590522869	1.476486149	0.000171069	0.028695533	2.792598565	1.481659862	2.989591384	1.579948311	-0.4085906	-0.204385	0.69505047	0.87397302	0.88813729	-0.1711454	1.07302465	0.10168322	-56.600485
unknown	377.2334	-6.589418485	-2.289739949	0.000307317	0.024439191	0.169468953	-2.560907103	0.19118555	-2.386954605	4.116069505	1.403612178	0.003362371	0.073824984	2.731151241	1.449509207	2.945716991	1.55861883	-0.5770148	-0.3052549	0.58200273	0.8125112	0.79027838	-0.3395672	0.96122027	-0.057061	-27.122504
unknown	379.1896	-3.822066338	-1.928154336	0.006524706	0.098870669	0.217275401	-2.202403244	0.245374087	-2.026945197	3.790508584	1.660732234	0.005307978	0.087859906	3.154210272	1.657278839	3.41891037	1.773536602	0.29956813	0.14507179	0.77320604	0.91525208	0.91940281	-0.121231	1.12288322	0.16720789	-14.487575
unknown	318.2157	-4.51963892	-2.170250068	0.002733331	0.059871995	0.188451174	-2.40773731	0.214878222	-2.21840882	2.803939589	1.458038675	0.023057398	0.189633969	2.88833347	1.530237317	3.075587314	1.620861934	-0.5682807	-0.3901731	0.58760245	0.81544064	0.80407224	-0.314603	0.97942603	-0.0299916	-12.672794
unknown	334.2109	-8.389490912	-2.150517855	6.72E-05	0.016446553	0.192951848	-2.373687236	0.215311387	-2.215503473	6.39712229	1.458163077	0.000209775	0.028695533	2.753796733	1.461422073	2.950852339	1.56113173	-0.4673483	-0.2319847	0.65444985	0.85285121	0.86993382	-0.2010225	1.05071156	0.07136668	-53.668599
unknown	144.1016	3.043234788	1.887639962	0.018761184	0.176086663	4.409882481	2.14074021	4.88233156	2.287570272	-2.813605079	-1.658536642	0.022716538	0.18759541	0.250102029	-1.999411333	0.263984257	-1.9214762	-0.727107	-0.2003894	0.4907565	0.76606422	0.77151072	-0.3742419	0.92409492	-0.113887	-8.5624609
unknown	317.2124	-6.792555593	-2.265793126	0.000254926	0.02428314	0.174369961	-2.519776573	0.196472129	-2.347603423	4.133983972	1.376635071	0.003280419	0.073261239	2.660963408	1.411948671	2.868909466	1.520502442	-0.5002787	-0.2625805	0.63222069	0.83954193	0.8295788	-0.2695491	1.0075974	0.0109193	-28.080316
unknown	318.2156	-6.773645238	-2.265866483	0.000259353	0.02428314	0.174413698	-2.519414746	0.196512281	-2.347308619	4.079079513	1.374711914	0.003538648	0.074959362	2.661011112	1.411974535	2.869393118	1.520745637	-0.5197564	-0.2718333	0.61925988	0.83364691	0.82274751	-0.2814783	0.9992373	-0.0011008	-27.630238
unknown	350.2055	-7.29179483	-2.692911372	0.000163914	0.021377633	0.123155203	-3.021450515	0.137857251	-2.858752938	5.008410395	1.154762089	0.001041828	0.050324128	2.212893454	1.14593399	2.396931022	1.261188392	-1.1623525	-0.7680273	0.28318413	0.65902536	0.64217489	-0.6389618	0.77636792	-0.3651876	-36.520301
unknown	313.1815	-8.101234946	-2.099484155	8.41E-05	0.017507673	0.190789052	-2.389949709	0.214283269	-2.222408884	2.822254082	1.475370549	0.022415936	0.185972418	3.412717146	1.770920846	3.740405726	1.903194769	0.45741568	0.2447646	0.66123062	0.85615982	1.17047808	0.22709792	1.42519819	0.51116255	-22.863743
unknown	433.221	-5.879275837	-2.073236921	0.000612166	0.029500467	0.204767999	-2.287937825	0.225856993	-2.146518513	5.356213854	1.476786819	0.000680756	0.040614693	2.707866865	1.437156809	2.92685248	1.549350033	-1.40369	-0.5878204	0.20318504	0.6034824	0.60638663	-0.7216901	0.72891651	-0.4561745	-31.490659
unknown	399.2155	-6.258079164	-2.149534614	0.000420849	0.026388034	0.188017661	-2.411059913	0.211654044	-2.240220039	2.690635873	1.414414631	0.027469563	0.204252018	3.176720304	1.667538073	3.466023285	1.793281347	-0.0344695	-0.0165612	0.9734651	0.99255101	0.93521256	-0.0966338	1.13764285	0.18604771	-16.838212
unknown	319.2189	-6.697928946	-2.270414956	0.000277967	0.02428314	0.169706206	-2.558888769	0.191338615	-2.385800034	4.321505272	1.337406424	0.002540907	0.068025432	2.607108851	1.382450819	2.808406593	1.48975182	-0.5173452	-0.2662549	0.62085664	0.83410944	0.79561696	-0.3298541	0.96854577	-0.0461079	-28.945135
unknown	317.2122	-4.553071128	-2.050619688	0.002626477	0.058720754	0.172544009	-2.534963716	0.1959001	-2.351809959	5.689589974	1.450095326	0.00045987	0.035044854	2.87887488	1.525505089	3.07298705	1.619641687	-0.2725426	-0.1926157	0.79306938	0.92317127	0.83270435	-0.2641237	1.02144884	0.03061694	-25.905108
unknown	318.2152	-5.84351119	-2.387733736	0.000634771	0.02957347	0.154492614	-2.694390232	0.175325696	-2.511890637	3.397904987	1.235223459	0.009389512	0.116849249	2.431273191	1.281712011	2.624432685	1.392005594	-0.8298546	-0.4458794	0.43400577	0.73618159	0.68549452	-0.544783	0.83582109	-0.2587339	-19.855696
unknown	471.3488	-3.648490988	-1.923422035	0.008194302	0.11202315	0.224459868	-2.155470569	0.25103066	-1.994064516	2.981818155	1.514260013	0.017551649	0.164919746	2.863190851	1.517623839	3.135755641	1.64881314	-0.905451	-0.4891667	0.3953217	0.71147145	0.63016009	-0.6662097	0.77033717	-0.3764381	-10.879137
unknown	534.3884	-2.481997812	-2.203581264	0.0420868	0.266102627	0.122278098	-3.031762074	0.141480748	-2.821322339	2.905156361	1.304869339	0.019735071	0.175569435	2.163521036	1.113381148	2.270290582	1.182876965	-0.9543023	-1.0174298	0.37171522	0.7041028	0.38346869	-1.3828193	0.47032368	-1.0882741	-7.2105917
unknown	647.3954	-3.430671283	-1.975700677	0.010975207	0.135805171	0.262124957	-1.931673376	0.295261743	-1.759933656	2.949099096	1.429601353	0.018451087	0.169225789	2.205792536	1.141297106	2.341590089	1.227488545	-1.5017504	-0.4768368	0.17685697	0.5807071	0.61942942	-0.6909882	0.74980766	-0.4154075	-10.11739
unknown	316.1997	-4.265563092	-2.110757423	0.003720971	0.072866223	0.207499821	-2.268818001	0.23629862	-2.081316892	3.191088363	1.33671124	0.012782453	0.139170901	2.340538425	1.22684045	2.498267459	1.320927937	-0.9179186	-0.3730655	0.38919326	0.70820313	0.64812238	-0.6256619	0.79221061	-0.3360441	-13.611789
unknown	507.1702	-3.427671504	-2.2549119	0.011019989	0.13610104	0.146713183	-2.768929588	0.162528319	-2.621236979	2.996014639	1.22737564	0.017175705	0.163507693	2.396714731	1.261058202	2.540652876	1.345199276	-0.6430895	-0.5123445	0.54065169	0.7903383	0.62509311	-0.677857	0.749465	-0.416067	-10.269354
unknown	477.2491	-2.40280816	-1.805662945	0.04727073	0.281705789	0.223569216	-2.161206543	0.254382426	-1.974929087	2.244176108	1.515273055	0.055065461	0.291226172	3.005740291	1.587720359	3.22547506	1.689511661	-0.3015379	-0.3001391	0.77176515	0.91446756	1.10764975	0.14750176	1.35336911	0.43655536	-5.3923247
unknown	317.2124	-5.405155485	-2.18351726	0.001002926	0.035647699	0.178359702	-2.487138402	0.201501383	-2.311138352	3.293157959	1.216156263	0.010970227	0.128174698	2.410010426	1.269039388	2.60428098	1.380885112	-0.8821694	-0.4892052	0.40695639	0.71645859	0.68525246	-0.5452925	0.83502273	-0.2601126	-17.800031
unknown	318.2155	-3.703487768	-1.907467006	0.007619892	0.107552635	0.187090842	-2.418189152	0.213120173	-2.230260934	4.579536395	1.378538638	0.001803104	0.059172455	2.75095673	1.459933447	2.924176426	1.548030357	-0.2547255	-0.1925112	0.80625648	0.92661236	0.82834646	-0.2716938	1.01761633	0.02519373	-16.960257
unknown	318.2156	-3.65786455	-1.927573712	0.00809317	0.111106439	0.227855753	-2.133807301	0.256824142	-1.961147267	3.060130472	1.362715731	0.015579511	0.1535229	2.440935171	1.287433979	2.632723368	1.396555939	-0.3014228	-0.1770647	0.77184933	0.91446756	0.85962462	-0.2182213	1.04775613	0.06730296	-11.193543
unknown	297.1865	-4.810241117	-1.833266475	0.001943189	0.049074945	0.234481975	-2.092451072	0.264100666	-1.920840158	3.405434364	1.421993269	0.009285574	0.116221162	2.832665366	1.502160181	3.065470026	1.616108298	0.09516216	0.03378237	0.92685291	0.97421145	0.84497046	-0.2430272	1.02984501	0.04242722	-16.38096
unknown	381.2046	-4.115890784	-2.105875483	0.004482626	0.080085483	0.183767838	-2.444043795	0.207122065	-2.271446839	2.455747159	1.229332715	0.039580383	0.245813029	2.454351692	1.295341992	2.688747028	1.426934025	-0.0514825	-0.0330128	0.96037993	0.98857673	0.94403807	-0.0830831	1.15252564	0.20479885	-10.107587
unknown	415.2106	-4.337662548	-1.920743724	0.003405786	0.068993027	0.234088715	-2.09487271	0.260721288	-1.939419711	3.130637632	1.334737622	0.01400179	0.14516005	2.5701815	1.361870243	2.717183033	1.442111751	-0.6029582	-0.3571103	0.56555156	0.8033025	0.82684037	-0.2743193	0.99984851	-0.0002186	-13.57965
unknown	217.0638	-3.675827284	-1.720296761	0.007903129	0.109418909	0.237505676	-2.073966105	0.263316553	-1.925129875	3.272469144	1.458347899	0.011314386	0.130586037	3.025057774	1.596962696	3.215579774	1.685078881	-1.6815289	-0.6863514	0.13654383	0.55020772	0.46404743	-1.1076558	0.55701954	-0.8442002	-12.029031
unknown	475.2337	-2.730819569	-1.616425274	0.029303984	0.225609542	0.251402215	-1.991930736	0.28421911	-1.814924537	2.865122553	1.539848343	0.020985576	0.180499402	3.156353895	1.658258972	3.403100533	1.766849771	-0.2593639	-0.1582144	0.80281673	0.92550737	0.70218004	-0.5100871	0.86359559	-0.2115722	-7.8241327
unknown	298.1897	-4.65083419	-1.767637372	0.002339577	0.054315343	0.24146776	-2.050097515	0.272585034	-1.875221743	3.422145045	1.395148978	0.009059219	0.11470906	2.805171094	1.488088767	3.029391668	1.599208115	0.13062197	0.04773841	0.89974899	0.96105076	0.84901409	-0.2361396	1.03611308	0.05118146	-15.915829
unknown	418.2306	-6.524285559	-1.784051561	0.000326599	0.024987604	0.241856174	-2.04777873	0.267400979	-1.902923345	3.601829458	1.35259254	0.006963773	0.098504915	2.782601117	1.476434115	3.03107849	1.599831211	0.17919735	0.08656641	0.86285928	0.94423652	1.02667476	0.03797923	1.22862314	0.29704246	-23.499364
unknown	649.4111	-3.020672811	-1.774346522	0.019367906	0.176888114	0.28240325	-1.824171405	0.318437693	-1.650916979	2.829485356	1.346733704	0.022167765	0.185032826	2.177105096	1.122411053	2.308823578	1.207157938	0.0512822	0.01847166	0.96053335	0.98857923	0.82738876	-0.2733627	1.00674441	0.00969747	-8.5469495
unknown	351.218	-3.5217185	-1.961325631	0.009705207	0.12495252	0.182022756	-2.45780927	0.201000159	-2.314531454	2.886076553	1.200037988	0.020321125	0.178173227	2.462045657	1.299857516	2.68721909	1.42611395	-0.4651912	-0.3254205	0.65591949	0.85312375	0.67881129	-0.5589175	0.81056532	-0.3029996	-10.163949
unknown	273.1494	-5.213148478	-2.305039219	0.00123505	0.038328302	0.182843293	-2.451320384	0.20481912	-2.287577695	2.438773486	1.019493278	0.040642188	0.249555281	1.93003478	0.948626846	2.096601499	1.068052674	-2.9919235	-0.8483173	0.02017135	0.30366888	0.46186139	-1.1144682	0.5626459	-0.8296778	-12.713688
unknown	299.1924	-3.315643584	-1.778702855	0.012841611	0.146823641	0.245425403	-2.026643512	0.27535775	-1.860620884	2.456147852	1.312583126	0.039555659	0.245813029	2.583204097	1.369161634	2.823680203	1.497576705	0.51063911	0.22199983	0.625309	0.83627927	0.92050104	-0.1195087	1.12618886	0.17144879	-8.1437109
unknown	400.2191	-5.208083183	-1.86827183	0.001241935	0.038328302	0.21570144	-2.212892284	0.242907526	-2.041520905	3.23115978	1.246672704	0.012036041	0.134165849	2.626691532	1.393246786	2.852845505	1.51240162	-0.2296165	-0.1164215	0.82495493	0.93329825	0.83285308	-0.2638661	1.01631459	0.02334704	-16.828149
unknown	429.1955	-5.219784614	-1.959561167	0.001226094	0.038328302	0.214995211	-2.217623568	0.240806271	-2.054055133	3.67367298	1.181289738	0.006276143	0.095068095	2.332973102	1.222169674	2.472104358	1.305739647	-0.7543482	-0.4341943	0.47524649	0.75716646	0.76856674	-0.3797576	0.93046387	-0.103978	-19.175782
unknown	375.2173	-5.584616274	-1.975653533	0.000829204	0.03418599	0.206814597	-2.273590083	0.233154499	-2.100641826	2.321848538	1.14082656	0.048778465	0.275335955	2.556223334	1.354013888	2.780793859	1.475496801	-0.2933099	-0.1590499	0.77779019	0.91655066	0.87951461	-0.1852206	1.0704156	0.09817105	-12.966633
unknown	631.4007	-5.426377103	-1.951874766	0.000980405	0.035647699	0.226437763	-2.142813521	0.255395694	-1.969193893	3.66013038	1.146009991	0.006400007	0.09572121	2.158870589	1.110276765	2.307651157	1.206425151	-1.109847	-0.3082704	0.3037305	0.67322453	0.67337995	-0.5705073	0.81964926	-0.2869214	-19.861248
unknown	632.4045	-5.392355645	-1.949509869	0.001016788	0.035647699	0.22544032	-2.149182531	0.254395646	-1.974854118	3.706640082	1.146997793	0.005985182	0.094523898	2.163931048	1.113654529	2.31319166	1.209884806	-1.0847997	-0.307211	0.31395193	0.67727307	0.6714052	-0.5747444	0.81760909	-0.2905169	-19.987522
unknown	315.1967	-7.801126412	-2.090625942	0.000107005	0.019884795	0.198219693	-2.334827792	0.222536497	-2.167886129	4.489987813	1.044675736	0.002028784	0.061338495	2.104824837	1.073700178	2.239431998	1.163132858	-2.355285	-0.9602554	0.05069173	0.3977867	0.47862862	-1.0630214	0.58056398	-0.784473	-35.026963
unknown	352.2213	-2.750464789	-1.657674655	0.028484874	0.222250576	0.236590519	-2.079535831	0.259336375	-1.94710352	4.208630461	1.283695176	0.002961402	0.071259419	2.343221955	1.228493616	2.522914758	1.335091463	-0.5719494	-0.4068215	0.58524657	0.81438703	0.65541896	-0.6095107	0.78260281	-0.3536478	-11.57569
unknown	352.2213	-4.560734493	-1.755616719	0.002602638	0.058720754	0.276578626	-1.854238428	0.309167089	-1.693541344	3.389697255	1.204868052	0.009504218	0.117380692	2.143965169	1.100281468	2.298058118	1.200415284	-1.9668319	-0.5712832	0.08991	0.48364867	0.61903815	-0.6918998	0.74445253	-0.4257482	-15.459509
unknown	457.2602	-4.233090236	-1.539720995	0.003873282	0.074726838	0.299005781	-1.741754717	0.337087367	-1.568805534	4.159673068	1.36612988	0.003166636	0.072454858	2.533932583	1.341378141	2.706749509	1.436561382	-0.2338816	-0.0894031	0.82176977	0.93199556	0.82663792	-0.2746726	1.00471752	0.00678994	-17.608271
unknown	481.2182	-6.155036004	-1.749992144	0.000465239	0.026405285	0.248612553	-2.008028951	0.278671173	-1.843364326	2.626990266	1.191789598	0.03031954	0.212932836	2.604929042	1.381244074	2.844361407	1.508104787	0.12525971	0.05676363	0.90383944	0.96290427	0.9881223	-0.0172385	1.19945976	0.26238475	-16.16922
unknown	379.213	4.499809392	1.582082513	0.002798969	0.060840261	2.805885783	1.488456284	3.158412113	1.659199428	-3.98304483	-1.305637348	0.004044439	0.078798929	0.381880721	-1.388806007	0.400962081	-1.318462289	-1.7721362	-0.3277476	0.11966232	0.53237277	0.69202198	-0.5311102	0.83489134	-0.2603397	-17.922943
unknown	110.9758	2.829653007	1.500113008	0.025418836	0.207454607	2.524239648	1.335848884	2.811846478	1.491517828	-3.252399545	-1.375170049	0.011659128	0.1326619	0.36110549	-1.469507739	0.383336203	-1.383317841	-1.4809793	-0.6152261	0.18216133	0.58457625	0.52922672	-0.9180422	0.63216481	-0.6616274	-9.2031622
unknown	593.3857	-4.011359905	-1.551372147	0.005115456	0.085752919	0.313788944	-1.672133571	0.35030244	-1.513327057	2.56433041	1.328767575	0.033421037	0.227256079	2.652463529	1.407332915	2.869639592	1.520869555	1.29838843	0.38936799	0.23529079	0.62724807	1.15941489	0.21339692	1.40737621	0.49300803	-10.286452
unknown	441.2527	2.361576951	1.67890808	0.050224637	0.286849409	2.765779313	1.467686045	3.015191911	1.59224983	-2.376536466	-1.218762545	0.044787139	0.264544322	0.384958241	-1.377226141	0.415175276	-1.268207562	-0.7655236	-0.657362	0.46897898	0.75440126	0.66004806	-0.599357	0.77619709	-0.3655051	-5.6123737
unknown	417.2266	-8.425919452	-1.634929666	6.54E-05	0.016446553	0.288273092	-1.794491916	0.318725558	-1.649613383	3.312640072	1.228587546	0.01065622	0.125886256	2.48742445	1.314652708	2.706456896	1.436405411	0.1803212	0.06015519	0.86200974	0.94399868	1.00197753	0.00285016	1.20010674	0.26316273	-27.912038
unknown	301.1802	-5.165368831	-1.973507524	0.00130172	0.039665955	0.215919382	-2.21143534	0.241916655	-2.047417998	2.706971829	1.01241684	0.026783354	0.201199758	2.057051427	1.040577862	2.235678884	1.160712985	-0.9747575	-0.4094766	0.36215393	0.69994243	0.66827081	-0.5814952	0.81448909	-0.2960327	-13.982508
unknown	378.2377	-5.26248884	-1.690477174	0.001170163	0.038328302	0.25628751	-1.964164923	0.288152123	-1.795097446	4.357334829	1.162776733	0.002421297	0.066159964	2.262242017	1.177753279	2.442529545	1.288376013	-0.1658561	-0.07698	0.87295884	0.94844461	0.88197644	-0.181188	1.07261096	0.1011269	-22.930426
unknown	413.2003	-6.453074783	-1.727562607	0.000349251	0.024987604	0.26226777	-1.930887571	0.294097305	-1.765634531	3.928311516	1.123513338	0.00436704	0.081386137	2.192835131	1.132797347	2.357572724	1.237302274	-0.4173174	-0.1448788	0.68894774	0.87130754	0.8205508	-0.2853354	0.99454124	-0.0078969	-25.349688
unknown	418.2307	-4.047498139	-1.603842859	0.004886252	0.083630577	0.301374538	-1.73037056	0.337736535	-1.566029844	3.280658471	1.20472181	0.011176815	0.129686852	2.246578704	1.167729604	2.359022066	1.238188913	-0.495069	-0.1326068	0.63571118	0.84120061	0.799327	-0.3231423	0.96339412	-0.053802	-13.278459
unknown	414.2027	-8.261157393	-1.806832446	7.42E-05	0.017281626	0.250351867	-1.99797088	0.279371273	-1.839744414	3.55396117	1.064066349	0.007466173	0.102283427	2.148882323	1.10358648	2.318508977	1.213197313	-0.6778476	-0.2234066	0.51963757	0.782113	0.79140931	-0.3375041	0.95614097	-0.0647048	-29.359833
unknown	334.2109	-6.825733441	-1.740480658	0.000247362	0.02428314	0.259247191	-1.947599735	0.28914511	-1.790134393	5.605086664	1.042509114	0.000507229	0.036751549	2.007407988	1.005333862	2.163546003	1.113397797	-2.1798063	-0.5186822	0.06565949	0.44079589	0.59627842	-0.745942	0.72295523	-0.4680218	-38.258827
unknown	400.2192	-8.145581842	-1.506247156	8.12E-05	0.017507673	0.314813092	-1.667432558	0.350077031	-1.514255688	4.268064317	1.157919802	0.002731339	0.070581687	2.254686235	1.17292668	2.427846934	1.279677468	0.05195742	0.01121897	0.96001423	0.98857673	0.90470684	-0.1444777	1.08972489	0.12396396	-34.765867
unknown	399.2155	-7.473457301	-1.491137634	0.000140409	0.020181104	0.31797394	-1.653019563	0.353709294	-1.499363966	4.216262387	1.140617887	0.002930722	0.071129468	2.218596026	1.149646998	2.38881033	1.256292309	0.08358621	0.0185252	0.93572557	0.97865093	0.90715622	-0.1405771	1.09287612	0.12812988	-31.510057
unknown	437.1702	-4.514081878	-1.210770895	0.002751553	0.060009615	0.380670664	-1.3933847	0.424538036	-1.236034278	4.404773071	1.403961451	0.002272215	0.064703543	2.669417547	1.416524987	2.886577577	1.529359998	1.91287693	0.64850209	0.0973447	0.49702986	1.44755854	0.53362169	1.75211388	0.80909654	-19.883505
unknown	306.0526	-2.381862484	-1.449397447	0.048748444	0.284384964	0.273249761	-1.871707859	0.30726901	-1.702425827	3.419188183	1.167911193	0.009098842	0.114987494	2.061958592	1.044015361	2.22339817	1.152766332	-0.4457253	-0.2385257	0.66925525	0.85931616	0.60065288	-0.7353966	0.73565231	-0.442904	-8.1440361

unknown	400.2189	-4.071495609	-1.63874644	0.004740269	0.081992817	0.295095977	-1.760743843	0.328778339	-1.604812844	2.990535406	1.014128224	0.017319799	0.16423272	1.866968117	0.90069729	2.010147997	1.007301723	-0.7335788	-0.2447773	0.4870417	0.76393386	0.75737446	-0.4009213	0.9172254	-0.1246518	-12.175952
unknown	509.2386	-2.823040572	-1.249031391	0.02566107	0.208838473	0.333028859	-1.586280896	0.37499801	-1.415045157	2.917789034	1.326936853	0.019356717	0.173386199	2.742627764	1.45555883	2.947968688	1.559721201	0.11572166	0.09008736	0.91112293	0.96718747	1.28049335	0.35669977	1.54982217	0.63210269	-8.2370368
unknown	395.2353	-3.318618743	-1.770243248	0.012789254	0.146770994	0.251096074	-1.993688625	0.280593241	-1.833447835	2.618418812	0.935340773	0.030725833	0.214126482	1.724542759	0.7862139	1.866900669	0.900645169	-0.6128619	-0.3742748	0.55934421	0.79918571	0.77779658	-0.3625352	0.94614532	-0.0798663	-8.6895337
unknown	394.2319	-6.604136093	-1.50005787	0.000303139	0.024404569	0.308709832	-1.695676665	0.343220405	-1.542792769	6.629341413	1.103316793	0.000164292	0.028695533	2.087691817	1.061908759	2.244035721	1.166095641	-1.5447077	-0.3451187	0.16633136	0.57183885	0.68084474	-0.5546022	0.8209005	-0.2847207	-43.781073
unknown	385.2383	-2.343879911	-1.010094626	0.051549719	0.290444357	0.445370869	-1.166920898	0.496001866	-1.011582546	4.745573263	1.629098212	0.001453498	0.055229968	2.861537175	1.516790349	3.066993774	1.616825238	0.34098481	0.1292621	0.74312071	0.90032917	0.94176009	-0.0865685	1.14509303	0.19546482	-11.123054
unknown	493.2444	-3.968747263	-1.600283643	0.005400939	0.088938195	0.273704482	-1.869309035	0.306780734	-1.70472021	2.96931964	1.009590458	0.017889721	0.166959983	2.005643601	1.004065265	2.15119709	1.105139709	-2.3514476	-1.1950702	0.05097877	0.39831477	0.39001565	-1.3583961	0.47382778	-1.0775653	-11.784479
unknown	351.2179	-7.84559461	-1.616622115	0.000103206	0.019884795	0.286251871	-1.80464297	0.318203052	-1.651980421	4.062304668	0.972765241	0.003621846	0.075216749	1.953747229	0.966243827	2.111828188	1.078492466	-1.5043768	-0.5273133	0.17619636	0.58055525	0.66747892	-0.5832058	0.80127546	-0.3196298	-31.871196
unknown	393.2287	-7.44458578	-1.50084364	0.000143876	0.020181104	0.308823876	-1.695143798	0.342924947	-1.544035235	5.853178159	1.040463991	0.000381402	0.03374818	2.024248847	1.017386656	2.179393405	1.123926642	-1.6246071	-0.3336352	0.14827434	0.56046829	0.69001548	-0.5352994	0.83001444	-0.2687917	-43.574487
unknown	315.1967	-4.27864209	-1.702280382	0.003661494	0.071917478	0.256276048	-1.964229445	0.291269801	-1.779571965	2.771225046	0.916942105	0.024250779	0.194608996	1.881692125	0.912030599	2.012714209	1.009142334	-0.9604429	-0.3936664	0.36882494	0.70263145	0.63882101	-0.6465163	0.78046298	-0.3575979	-11.85708
unknown	394.28	3.792266035	1.099278821	0.006782823	0.101214625	1.842660569	0.881790341	2.049995416	1.035620684	-3.786547263	-1.417336255	0.005338048	0.087859906	0.400898537	-1.318690943	0.432941959	-1.207754467	-0.1539382	-0.0563811	0.88200256	0.95287254	0.84871263	-0.2366519	1.02846343	0.0404905	-14.359595
unknown	499.3105	3.323024625	0.95990435	0.012712141	0.146200828	1.767198688	0.821464253	1.965321633	0.974765435	-4.561515927	-1.622806796	0.00184623	0.059600339	0.332588377	-1.588190343	0.350116904	-1.514091376	-1.5958619	-0.6226463	0.15455014	0.56601708	0.60609623	-0.7223812	0.73310903	-0.4479003	-15.15803
unknown	633.4074	-5.107392627	-1.708623204	0.001388048	0.040613708	0.25881558	-1.950003629	0.29202338	-1.775844215	3.209594861	0.907902191	0.012431861	0.137171175	1.883676691	0.913551366	2.012916622	1.009287415	-0.9713073	-0.2838167	0.3637533	0.70082245	0.67775628	-0.5611615	0.82577479	-0.2761797	-16.392661
unknown	314.1846	-3.360782906	-1.296480361	0.012071396	0.143023707	0.322250043	-1.633747542	0.356304464	-1.488817538	4.487463857	1.193670504	0.002035573	0.061338495	2.324424757	1.216873726	2.508652853	1.326912845	-0.5407747	-0.2030465	0.60543508	0.82488044	0.6793178	-0.5578414	0.81134551	-0.3016117	-15.081392
unknown	351.2179	-6.065022506	-1.390889607	0.00050834	0.027624042	0.330914151	-1.595471107	0.367516693	-1.444118313	6.509311015	1.095083648	0.000186268	0.028695533	2.096093179	1.067702851	2.256143869	1.173859068	-0.9404325	-0.2477903	0.37830672	0.70604983	0.75031254	-0.4144364	0.90622177	-0.142064	-39.479118
unknown	305.0495	-2.369158273	-1.397904281	0.049667665	0.285863059	0.279139977	-1.840939341	0.314090113	-1.670749563	3.315879937	1.080090308	0.010604927	0.125507681	1.980279663	0.985704188	2.131563498	1.091912033	-0.4151048	-0.2228711	0.69049271	0.87207726	0.60857195	-0.7165002	0.74541735	-0.4238797	-7.8558444
unknown	557.2967	-4.580832162	-1.689260752	0.002541246	0.057740301	0.250361119	-1.997917564	0.283911818	-1.81648519	2.795917449	0.871077647	0.023344313	0.190285328	1.897636512	0.924203674	2.033291888	1.023817335	-1.2152947	-0.8216229	0.26364782	0.64599694	0.63728692	-0.649985	0.77458516	-0.3685042	-12.807629
unknown	634.411	-4.55212963	-1.791433179	0.002629422	0.058720754	0.250557337	-1.996787313	0.283470958	-1.818727159	2.249939949	0.818583292	0.054572419	0.290740806	1.758857206	0.814638362	1.873643762	0.905846678	-0.9586293	-0.311444	0.36967678	0.70322704	0.67848823	-0.5596043	0.82519098	-0.2772	-10.242018
unknown	336.2265	-4.665728217	-1.513113385	0.00229899	0.054200457	0.303882119	-1.718416311	0.340162229	-1.555705138	4.279376612	0.950674312	0.002689778	0.070160157	1.847455736	0.885539798	1.985976731	0.989848719	-2.3999993	-0.6032094	0.0474662	0.39230303	0.5478378	-0.8681793	0.66671928	-0.5848487	-19.966408
unknown	316.1998	-5.071698819	-1.570451934	0.001444352	0.040950513	0.281452229	-1.829038019	0.31660751	-1.659232619	4.710591384	0.905194425	0.001520514	0.05569795	1.840861583	0.880381153	1.973801122	0.980976633	-1.5585238	-0.4196401	0.16307027	0.57018188	0.61200035	-0.7083956	0.746263	-0.4222439	-23.890701
unknown	399.2156	-3.94398551	-1.58876308	0.005574794	0.090883087	0.285505064	-1.80841176	0.320841497	-1.640067344	2.308526316	0.886321008	0.049803526	0.278771494	1.854169185	0.89077289	1.995818496	0.996980525	-0.9004634	-0.4358094	0.39779332	0.71197145	0.70299286	-0.5084181	0.85377375	-0.2280743	-9.1047943
unknown	480.3784	-5.09292432	-0.989736268	0.001410571	0.040613708	0.451598117	-1.146888623	0.497485176	-1.007274557	7.820206769	1.398927543	5.14E-05	0.016966841	2.573290975	1.363614599	2.771525341	1.470680199	2.56863301	0.50891086	0.03708173	0.36091037	1.27169206	0.34674937	1.53125424	0.61471384	-39.827721
unknown	336.2264	-5.441216396	-1.576897164	0.000964993	0.035647699	0.277631517	-1.848756739	0.310923316	-1.685369286	5.848628604	0.861084669	0.000383373	0.03374818	1.781121924	0.832786277	1.921884346	0.942521521	-1.5095215	-0.5407363	0.17490887	0.57956337	0.59615844	-0.7462323	0.72600727	-0.4619441	-31.823654
unknown	433.2212	-3.784582579	-1.478734746	0.006851162	0.101472111	0.338481489	-1.562851156	0.377950973	-1.403728991	2.654986343	0.917768928	0.029030361	0.209642287	1.731799051	0.792271537	1.850619527	0.888008319	-1.0773952	-0.2853691	0.31702623	0.67847982	0.74735631	-0.4201319	0.90079562	-0.1507283	-10.048015
unknown	313.1814	-3.294114681	-1.217941851	0.013227397	0.149933511	0.341356137	-1.550650406	0.376774679	-1.408226084	4.264553467	1.112971527	0.002744378	0.070581687	2.201092427	1.138219727	2.378966739	1.250335101	-0.6612816	-0.2387004	0.52958832	0.7860791	0.66479836	-0.5890113	0.7940845	-0.3326356	-14.047928
unknown	315.1965	-5.971322158	-1.550245036	0.000558017	0.028504913	0.287914085	-1.796289726	0.323314637	-1.628989273	5.136813696	0.862066551	0.000888607	0.045270338	1.806865239	0.85348891	1.934448339	0.951922201	-1.6198113	-0.3970869	0.14930462	0.56046829	0.63580083	-0.6533532	0.77292349	-0.3716025	-30.673569
unknown	291.1966	-5.92849379	-1.285096091	0.000582515	0.029052163	0.345471461	-1.53336156	0.384631335	-1.378451794	8.270885406	1.035247818	3.43E-05	0.013993256	2.048616392	1.034649862	2.188199951	1.129744573	-0.0316887	-0.0080066	0.97560491	0.99295285	0.84777847	-0.2382408	1.02374514	0.03385661	-49.033893
unknown	431.21	-5.7910104	-1.44569402	0.000669664	0.02991015	0.316357584	-1.660371914	0.352762464	-1.503231038	4.057251668	0.888061951	0.003647318	0.075266337	1.87704322	0.90846187	1.991432968	0.993806919	-0.97566	-0.2472178	0.36173646	0.69975778	0.72822506	-0.4575437	0.87955664	-0.1851516	-23.495587
unknown	318.2157	-4.143999714	-1.560458284	0.004327451	0.078824883	0.284693365	-1.812519225	0.320076064	-1.643513301	2.370852463	0.799998626	0.045186254	0.265644591	1.763812982	0.8186976	1.898007546	0.924485728	-1.0089712	-0.5163865	0.34658566	0.69419824	0.6751896	-0.5666354	0.82085151	-0.2848068	-9.8248119
unknown	345.171	-4.6202169	-1.220675094	0.002425522	0.055693055	0.384938251	-1.377301059	0.426814599	-1.22831857	3.943222903	1.002999875	0.0042765	0.08061666	1.981923019	0.986900927	2.117737997	1.082524113	-1.7295405	-0.415061	0.12733526	0.53939735	0.65789967	-0.6040605	0.78961317	-0.340782	-18.218545
unknown	335.2232	-4.06064362	-1.515080055	0.004805685	0.082475584	0.282230558	-1.825053891	0.316055787	-1.661748865	3.053211808	0.807710521	0.015744042	0.154385566	1.758400207	0.814263461	1.904950374	0.929753414	-0.6077781	-0.3100618	0.5625255	0.80162342	0.74395356	-0.4267155	0.90421853	-0.1452566	-12.398005
unknown	521.2758	-3.249478681	-1.14511432	0.014067419	0.151876888	0.389537109	-1.360167322	0.437172454	-1.193725595	2.593545937	1.066581029	0.031936581	0.220379304	2.206175916	1.141547833	2.37778989	1.249621239	0.58268894	0.15031071	0.57838126	0.8105923	0.91059007	-0.1351264	1.10753162	0.14734789	-8.4276722
unknown	491.1763	-6.205226928	-1.747462269	0.000442994	0.026405285	0.268096386	-1.899176321	0.297009776	-1.751417677	2.279999281	0.693314157	0.052071383	0.283143399	1.603160125	0.680918531	1.723710493	0.785517486	-1.6672485	-0.4796364	0.13940194	0.55113297	0.64545083	-0.6316209	0.77224049	-0.3728779	-14.147913
unknown	332.1952	-5.560322055	-1.271078017	0.000850626	0.03433527	0.368507082	-1.440235748	0.410350834	-1.285070211	4.652574307	0.951280014	0.001639185	0.057161116	1.866883806	0.900632138	2.004670342	1.003365012	-1.2402193	-0.2459855	0.25485103	0.63869468	0.72979404	-0.4544387	0.88053704	-0.1835444	-25.869812
unknown	495.3694	-2.432420269	-0.673616957	0.045259507	0.276314868	0.554857308	-0.849811293	0.611947729	-0.708519668	5.267138241	1.759755463	0.000757917	0.041884553	3.579076502	1.839587382	3.790640806	1.922441756	2.22268582	1.10163843	0.06163249	0.43021854	2.18530022	1.12783149	2.62761329	1.39375297	-12.811894
unknown	351.2181	-5.073264879	-1.217080972	0.001441829	0.040950513	0.382859541	-1.385112884	0.425565839	-1.232545746	4.820046882	0.968444414	0.001321286	0.054879648	1.900060931	0.926045684	2.040970796	1.029255539	-2.1785673	-0.3687134	0.06577971	0.44079589	0.67346348	-0.5703284	0.81375099	-0.2973407	-24.453375
unknown	299.1653	-4.527112348	-1.429953248	0.002709035	0.059871995	0.30961848	-1.691436511	0.348117269	-1.52235471	3.722766222	0.803822265	0.005848131	0.093241226	1.728605691	0.789608816	1.855743943	0.89199766	-1.249439	-0.3420475	0.2516609	0.63656674	0.63732405	-0.649901	0.77836274	-0.3614855	-16.853381
unknown	335.2232	-6.008251034	-1.374961139	0.000537817	0.028255504	0.337514927	-1.566976786	0.376364278	-1.40979839	4.210747598	0.832307322	0.002952857	0.071259419	1.768889147	0.82284364	1.885397708	0.914868879	-0.9621164	-0.1918419	0.36804026	0.70236772	0.75679003	-0.402035	0.91456895	-0.1288362	-25.299229
unknown	479.3745	-6.172658819	-0.892673177	0.000457289	0.026405285	0.475963319	-1.071077702	0.524782887	-0.930207421	9.370196052	1.270802528	1.38E-05	0.006905016	2.395996972	1.260626085	2.571272478	1.362482501	2.73054005	0.49952306	0.02931582	0.34120727	1.25309595	0.32549688	1.50754031	0.59219658	-57.839023
unknown	417.2263	-5.232410951	-1.266866743	0.001209253	0.038328302	0.360243587	-1.472955346	0.400815255	-1.318990676	3.741497325	0.891619991	0.005693135	0.090992488	1.903341602	0.928534513	2.01450501	1.010425394	-0.4375919	-0.1140684	0.67486579	0.86244885	0.81160316	-0.3011536	0.9776876	-0.0325545	-19.577052
unknown	336.2265	-6.017058578	-1.374329857	0.000533121	0.028255504	0.337866021	-1.565476827	0.376710389	-1.408472275	4.193246182	0.815903812	0.003024304	0.071769533	1.745418026	0.803572602	1.861001376	0.896079122	-0.9650909	-0.1937299	0.36664865	0.70173058	0.75674743	-0.4021162	0.91434692	-0.1291864	-25.231008
unknown	395.2441	-6.648470971	-1.360113202	0.000290935	0.024360587	0.335921539	-1.573803794	0.374688635	-1.416235877	4.917185743	0.824100787	0.001168151	0.051819823	1.775865926	0.828522666	1.895582565	0.922641297	-0.7768751	-0.1786807	0.46267216	0.75199554	0.76985068	-0.3773494	0.9303172	-0.1042054	-32.691767
unknown	432.2126	-5.146399209	-1.365967632	0.001329291	0.039762876	0.332556433	-1.588328915	0.372894854	-1.423159207	3.517383655	0.811386782	0.007875996	0.105029391	1.788259713	0.838556277	1.891396307	0.919451685	-1.2139356	-0.322911	0.26413479	0.64655518	0.68718839	-0.5412224	0.83265257	-0.2642134	-18.10186
unknown	331.1919	-4.462150503	-1.582260637	0.002928463	0.062817448	0.283187988	-1.820168024	0.317329806	-1.65594506	2.404738868	0.693953879	0.042858612	0.256875928	1.64511517	0.718188586	1.736904653	0.79651856	-1.615892	-0.5233286	0.15015153	0.56046829	0.58188307	-0.7811988	0.70628164	-0.5016845	-10.730307
unknown	367.1689	-4.67152525	-1.56074228	0.002283404	0.054200457	0.282171964	-1.825353441	0.316356537	-1.66037669	2.553463601	0.702727614	0.033990947	0.229722469	1.694356841	0.760737746	1.787543308	0.837978195	-1.7009226	-0.6157688	0.13275073	0.54625992	0.55859856	-0.8401162	0.67729773	-0.5621379	-11.92857
unknown	300.1685	-4.481349963	-1.419198938	0.002861637	0.061995799	0.307824803	-1.699818614	0.346374937	-1.529593551	3.752934923	0.771345135	0.005600654	0.090440081	1.702414835	0.767582628	1.829181535	0.871198261	-1.3284672	-0.3806408	0.22569527	0.61943033	0.61639713	-0.6980679	0.75345491	-0.4084069	-16.818215
unknown	415.1206	-5.725929759	-1.23774512	0.00071593	0.030924562	0.367342139	-1.444803693	0.411189093	-1.282126099	3.640065284	0.88217884	0.006588342	0.096587859	1.873513177	0.905746124	2.007420184	1.005342626	0.50378727	0.16571759	0.62987555	0.83820252	0.99291135	-0.0102632	1.20728423	0.27176537	-20.842758
unknown	314.1845	-3.860808095	-1.281154217	0.006205005	0.096801053	0.379343212	-1.398424371	0.424809293	-1.235112769	2.748088957	0.85121332	0.025133035	0.197641266	1.701163905	0.766522149	1.818258906	0.862557643	-0.8858705	-0.2494898	0.4050902	0.7159805	0.75302722	-0.4092261	0.90789259	-0.1394065	-10.609844
unknown	401.2194	-8.074393501	-1.136507245	8.59E-05	0.017507673	0.40116022	-1.317749543	0.44531299	-1.167108399	4.831640042	0.951710602	0.00130191	0.0547726	1.947686579	0.961761538	2.093524708	1.065933945	0.01604311	0.00220324	0.98764768	0.99670416	0.87230088	-0.1971022	1.05068659	0.0713324	-39.012563
unknown	297.172	-2.935037123	-1.022581639	0.02186703	0.189873369	0.458821799	-1.123994158	0.504560794	-0.986899986	2.529889024	1.05194176	0.03526153	0.233917028	2.044556928	1.031788233	2.22498355	1.15379467	1.6682452	0.40958941	0.13920065	0.55113297	1.1654018	0.22082744	1.40086216	0.48631501	-7.4253182
unknown	332.1952	-4.46898588	-1.571201751	0.002904476	0.062715528	0.285962893	-1.806100144	0.320374265	-1.64216983	2.364750294	0.683826495	0.045618705	0.266797825	1.634232503	0.708613251	1.725636491	0.78712859	-1.6265764	-0.5226373	0.14785322	0.56022712	0.58361335	-0.7769152	0.70830752	-0.4975522	-10.568036
unknown	329.1761	-5.725716451	-1.310671993	0.000716088	0.030924562	0.345337919	-1.533919339	0.376144477	-1.410641189	5.080486751	0.80967927	0.000952556	0.047057725	1.795959697	0.844754975	1.92453954	0.944513312	-2.2718469	-0.5831207	0.05732118	0.41532376	0.57705491	-0.7932195	0.68061176	-0.555096	-29.089427
unknown	396.2471	-6.533131794	-1.349725533	0.000323902	0.024987604	0.340307727	-1.555088187	0.379472122	-1.397934193	4.507518321	0.785659582	0.001982299	0.061338495	1.722686771	0.784660406	1.839384474	0.879223068	-0.7271814	-0.1563342	0.49071368	0.76606422	0.78235256	-0.3541092	0.94544629	-0.0809326	-29.448211
unknown	334.2109	-5.45189382	-1.450484725	0.00095407	0.035647699	0.317301857	-1.656072129	0.354344404	-1.49677583	3.867156038	0.731061461	0.00476046	0.085395354	1.616789199	0.693131589	1.735471827	0.795327945	-2.6593327	-0.6492381	0.03249858	0.35261767	0.54836379	-0.8667948	0.66541583	-0.5876719	-21.083324
unknown	399.2155	-3.726238557	-1.430523024	0.007395184	0.1059868	0.314230484	-1.670104948	0.352356499	-1.504892271	2.841072381	0.740237526	0.021776012	0.183464307	1.601165704	0.679122619	1.725145529	0.786718069	-0.4421562	-0.1729722	0.67171455	0.86090414	0.76753713	-0.3816916	0.93385049	-0.0987365	-10.586513
unknown	322.9959	2.584694261	1.169596188	0.036223687	0.251035882	1.863906875	0.898329782	2.037815084	1.027023144	-2.239085224	-0.899464202	0.055504614	0.292145323	0.570686879	-0.809228701	0.605200294	-0.724515406	0.76509748	0.55337589	0.46921782	0.75440126	1.41871034	0.50458007	1.69136605	0.75818893	-5.7873507
unknown	313.1814	-5.087096912	-1.155480196	0.001419757	0.040613708	0.395123545	-1.339624279	0.440642976	-1.182317885	5.082959904	0.90899491	0.000949645	0.047057725	1.816345445	0.861038611	1.950709588	0.963999012	-1.8733089	-0.3145105	0.10317514	0.50777743	0.68472345	-0.5464067	0.82973536	-0.2692768	-25.85751
unknown	433.2002	-2.642274425	-0.882915077	0.033313262	0.242005398	0.461498661	-1.115601634	0.517098415	-0.951489213	4.237040619	1.18160376	0.002848921	0.070662721	2.3008074	1.202140221	2.437878656	1.285626318	1.87660511	0.57625466	0.10267675	0.50777743	1.25600191	0.32883866	1.52578085	0.60954776	-11.195424
unknown	411.1805	-2.607701376	-1.013905162	0.035030305	0.245890869	0.483252089	-1.049152125	0.525550799	-0.928097876	2.66847944	1.015655918	0.028429192	0.207515381	1.853692101	0.890401632	2.005924589	1.00426737	1.92242877	0.44487769	0.09598606	0.49574567	1.19834112	0.26103865	1.41916294	0.50504024	-6.9585975
unknown	275.1656	-3.373704727	-1.214918159	0.011860162	0.142150548	0.361286359	-1.46878531	0.405261215	-1.303075986	3.48516817	0.843830607	0.008256751	0.108117016	1.749255456	0.806740991	1.876923911	0.908370166	0.39231431	0.16200695	0.70649726	0.88071901	0.98158877	-0.0268094	1.19301639	0.25461386	-11.757928
unknown	371.2017	-5.681875318	-1.348256435	0.000749274	0.031393339	0.340651306	-1.553632356	0.381468125	-1.390365582	4.587722759	0.759416458	0.001783875	0.059172455	1.659775649	0.730988247	1.769034569	0.82296224	-0.831559	-0.2110339	0.4331048	0.73548865	0.75759717	-0.4004972	0.91552963	-0.1273215	-26.066869
unknown	367.1687	-5.771385476	-1.166898066	0.000683254	0.030309525	0.389233269	-1.361293068	0.434694554	-1.201926075	4.686761589	0.857123221	0.001568086	0.056184018	1.77372651	0.826783579	1.903938028	0.92898652	-1.5758791	-0.2566833	0.15905733	0.56971676	0.71477728	-0.4844343	0.8634114	-0.2118799	-27.049108
unknown	425.256	4.810816675	1.690492545	0.0019419	0.049074945	2.879564387	1.525850581	3.1183512	1.640783419	-2.224230628	-0.581853659	0.056805973	0.295164739	0.643239144	-0.636572892	0.687963927	-0.539595174	0.00944323	0.0025535	0.992729	0.99811564	0.84779057	-0.2382202	0.99748298	-0.0036359	-10.700366
unknown	336.2266	-4.135326197	-1.461467529	0.004374696	0.079214656	0.297845579	-1.747363548	0.333003753	-1.586389658	2.314166967	0.659877907	0.049366912	0.277071683	1.580907309	0.660752783	1.721291204	0.78349119	-0.6177201	-0.2990546	0.55631412	0.79824884	0.74821992	-0.4184657	0.90838594	-0.1386227	-9.5698353
unknown	330.1793	-7.546109196	-0.98262875	0.000132092	0.020000824	0.447671079	-1.159488977	0.496192412	-1.011028423	5.092206752	0.969170568	0.000938847	0.047057725	1.958321091	0.969617332	2.105288852	1.074018189	-0.2207226	-0.0487438	0.83160815	0.93337638	0.86202095	-0.2142052	1.03534825	0.05011611	-38.426348
unknown	328.2574	-2.565031138	-1.117908725	0.037277027	0.25400574	0.419202434	-1.254281002	0.47054644	-1.087590981	2.39096857	0.847508107	0.04378959	0.261255187	1.66255761	0.733404333	1.771675331	0.825114247	-1.5171047	-0.5217562	0.17302677	0.57867838	0.59919722	-0.7388972	0.72888911	-0.4562288	-6.1329088
unknown	399.1948	4.15226783	1.42638784	0.004282935	0.078232544	2.362011357	1.240015901	2.580999359	1.367929783	-2.712982213	-0.652918272	0.026535331	0.200274183	0.622586298	-0.683654268	0.663029868	-0.592854233	0.33806492	0.18560867	0.74522678	0.90145032	1.08736352	0.12083433	1.28155126	0.35789118	-11.265029
unknown	297.1531	2.928011532	1.273931832	0.022086687	0.190512286	1.947813842	0.961855801	2.162420167	1.112646872	-2.649990736	-0.726483678	0.029256222	0.209878797	0.599482819	-0.738209688	0.645245419	-0.6320801	2.03542588	0.99170184	0.08125955	0.46961223	1.63181265	0.70647543	1.96229959	0.97254532	-7.7592034
unknown	407.2061	-2.532922198	-0.880371441	0.039065804	0.259183683	0.515472031	-0.956033945	0.569860582	-0.811319093	3.390948232	1.050919523	0.00948664	0.117380692	1.912603968	0.935538174	2.045325179	1.03233023	0.55699808	0.17915383	0.59488112	0.8189191	1.05969276	0.08364604	1.26333766	0.33724029	-8.589008
unknown	353.2327	-3.787484713	-1.145642298	0.006825262	0.101472111	0.401086441	-1.318014899	0.445640668	-1.166047199	3.318996937	0.801476781	0.010555826	0.125507681	1.677761885	0.746537977	1.807228664	0.853779058	-2.0439973	-0.4221808	0.08023813	0.46961223	0.63584074	-0.6532626	0.77106955	-0.3750671	-12.57065
unknown	329.1761	-5.04681272	-1.106007322	0.001485107	0.041563887	0.391903712	-1.351428855	0.436496576	-1.195957759	4.478755099	0.821539196	0.002059189	0.061596213	1.810765016	0.856599339	1.94014239	0.956162538	-1.0161783	-0.2175976	0.34337358	0.69368379	0.7195725	-0.474788	0.8672665	-0.2054527	-22.603438
unknown	391.2134	-4.900632816	-1.350697705	0.00175186	0.046956071	0.340123279	-1.555870342	0.380505153	-1.394012104	3.241705912	0.661341269	0.011847316	0.133200597	1.535811336	0.619001002	1.647158151	0.719979081	-2.458844	-0.5750647	0.04353904	0.38295017	0.5715518	-0.8070438	0.6901712	-0.5349738	-15.88641
unknown	481.2183	-4.654339643	-0.981993235	0.002329954	0.054315343	0.454448754	-1.137810478	0.499202085	-1.002304134	3.671260017	0.904773763	0.006298024	0.095068095	1.859572908	0.894971312	2.002784865	1.002007458	-0.5453885	-0.1113994	0.60242315	0.82356423	0.81549317	-0.2942553	0.97109116	-0.0423214	-17.087291
unknown	413.2001	-6.122987888	-1.078861127	0.000480097	0.026758206	0.424328972	-1.236744909	0.470137886	-1.088844149	3.903799395	0.796133231	0.004520374	0.083044401	1.712259269	0.77590117	1.834327132	0.875250951	-1.390079	-0.2218561	0.20710448	0.60621714	0.75758669	-0.4005171	0.90689951	-0.1409854	-23.902916
unknown	363.1534	-3.267958266	-0.97474979	0.013712908	0.151188271	0.425009613	-1.234432621	0.470269136	-1.088441444	3.504565299	0.879375216	0.008025215	0.106583357	1.897434175	0.924049837	2.021599221	1.015497014	-1.2851328	-0.5316303	0.23963061	0.62882544	0.60569074	-0.7233467	0.7287339	-0.456536	-11.452773
unknown	329.1761	-5.930656922	-0.935910196	0.000581249	0.029052163	0.464018129	-1.107746924	0.514518492	-0.958705167	4.255408151	0.910840074	0.002778661	0.070662721	1.876007448	0.907665556	2.018183274	1.013057193	-0.2943475	-0.0642544	0.77702947	0.91655066	0.84932983	-0.2356032	1.02062042	0.02944641	-25.237366
unknown	400.219	-4.878635958	-1.000269607	0.00179642	0.047235692	0.452250016	-1.144807541	0.49836643	-1.004721204	3.405614905	0.847501486	0.009283096	0.116221162	1.781008246	0.832694196	1.915005499	0.937348535	-0.5491582	-0.0945236	0.59996845	0.82235662	0.82618489	-0.2754634	0.98710045	-0.0187312	-16.614755
unknown	399.2154	-4.880754247	-0.984193189	0.001792074	0.047235692	0.454556767	-1.137467619	0.500876044	-0.997474483	3.861401461	0.861167778	0.004799389	0.085395354	1.790558335	0.840409521	1.924321575	0.944349909	-0.7175699	-0.1386667	0.4962647	0.76865587	0.80524363	-0.3125028	0.9607151	-0.0578194	-18.846552
unknown	298.1562	2.836389774	1.192829563	0.02517452	0.206494397	1.819865926	0.863832167	2.026097692	1.018703738	-2.601025113	-0.706971305	0.03156748	0.21852605	0.614557019	-0.702381224	0.661060681	-0.597145388	1.79621119	0.87632559	0.11552321	0.52517579	1.50908489	0.59367397	1.81664687	0.86127801	-7.377521
unknown	392.2172	-3.826784553	-0.949502669	0.006484828	0.098870669	0.476399066	-1.06975751	0.528778167	-0.919265486	3.747757856	0.886867832	0.005642314	0.090848223	1.748958881	0.806496371	1.875720321	0.907444731	-0.213921	-0.0415286	0.83670629	0.9349221	0.85376436	-0.2280902	1.02812313	0.04001305	-14.341862
unknown	347.1869	-6.154098508	-1.266529206	0.000465666	0.026405285	0.376930842	-1.407628249	0.413751043	-1.273165145	3.004475791	0.664809517	0.016955639	0.162122762	1.576446948	0.65667662	1.684316375	0.752163154	-2.3600202	-0.4036033	0.05033979	0.39641044	0.67743195	-0.5618521	0.80734104	-0.3087499	-18.48984
unknown	392.2169	-4.915591762	-1.322452937	0.001722258	0.046408457	0.345712884	-1.532353723	0.386449149	-1.371649506	3.55951206	0.631617561	0.007405986	0.101989132	1.502311547	0.587184027	1.609294405	0.686428277	-2.6340157	-0.6051401	0.0337153	0.35332453	0.55608969	-0.8466105	0.67213948	-0.5731674	-17.497108
unknown	301.1802	-3.417244054	-0.894928677	0.011177193	0.13726266	0.467674717	-1.096422658	0.521886717	-0.938191413	2.9934864	0.929644329	0.017242037	0.16389989	1.986437406	0.990183334	2.138920564	1.096882904	-0.6145834	-0.130629	0.55826936	0.79894596	0.77435807	-0.3689273	0.93606873	-0.0953136	-10.229474
unknown	309.0679	-2.854866088	-0.972300404	0.024517057	0.204004403	0.429409834	-1.219572863	0.472255714	-1.082359844	3.879076129	0.853942991	0.004680893	0.084796192	1.794176286	0.843321649	1.927010983	0.946364794	-1.3470223	-0.4981001	0.21994784	0.6172739	0.61035888	-0.7122703	0.7267514	-0.4604661	-11.074243
unknown	352.2211	-3.034908827	-1.093315588	0.018982696	0.176459747	0.371112027	-1.430073338	0.415868547	-1.265800519	4.949650322	0.759082933	0.001121377	0.051496456	1.680461371	0.74885738	1.794704659	0.84374645	-0.9760254	-0.3392141	0.36156753	0.69975778	0.61368155	-0.7044379	0.74754683	-0.4197641	-15.021737
unknown	403.2339	2.710031376	1.03293875	0.030197643	0.229352204	1.582929965	0.662597427	1.747980406	0.805689013	-4.272050707	-0.802708873	0.002716614	0.070577854	0.585835041	-0.771433607	0.624836264	-0.678449908	0.50459827	0.22325875	0.62933413	0.83820252	0.92678844	-0.109688	1.11188528	0.15300794	-11.577391
unknown	569.2634	-3.153024225	-1.018282255	0.016084045	0.163252634	0.439509359	-1.186034208	0.491468788	-1.024828296	2.431923044	0.809019489	0.041078824	0.251289879	1.711503183	0.775263975	1.845162418	0.883747813	-0.6042525	-0.1285583	0.56473798	0.80302145	0.78132724	-0.3560012	0.94590492	-0.0802329	-7.6679123
unknown	318.2156	-4.878831789	-1.014454017	0.001796017	0.047235692	0.449503123	-1.153596956	0.496162379	-1.011115748	3.499678152	0.80782262	0.008082894	0.106866089	1.7129618	0.776492979	1.839763419	0.879520257	-1.101846	-0.216921	0.30696583	0.67503825	0.76818114	-0.3804816	0.91853711	-0.1225901	-17.074341
unknown	481.2182	-4.734005396	-0.833345473	0.002122629	0.051648011	0.502539261	-0.992691784	0.550680662	-0.860712148	4.178134782	0.962237771	0.003087495	0.071769533	1.968035662	0.976756363	2.11847802	1.08302816	1.97753109	0.32883629	0.08850332	0.48174467	1.09684693	0.1333622	1.30519481	0.38426516	-19.779313
unknown	411.1779	-4.158123385	-0.829391879	0.004251712	0.078232544	0.504643538	-0.986663416	0.558916797	-0.839294561	3.521543105	0.945717092	0.007828211	0.10473818	1.939028573	0.955334063	2.081450334	1.057589135	1.03001374	0.19014314	0.33727272	0.69274061	1.0180955	0.0258729	1.21773795	0.28420371	-14.643011
unknown	391.2134	-4.440312348	-1.248470393	0.00300657	0.063449336	0.371718147	-1.427718974	0.414727899	-1.269762994	2.631800946	0.626844807	0.03009392	0.212649365	1.494666332	0.579823454	1.606123875	0.683583168	-2.9674555	-0.7304123	0.02088297	0.30521805	0.52322466	-0.9344976	0.63110067	-0.6640579	-11.686018
unknown	318.2063	-5.554268013	-1.098032459	0.00085606	0.03433527	0.409520712	-1.287991675	0.456874453	-1.130130321	4.228902384	0.71046286	0.002880663	0.070662721	1.621501226	0.697330115	1.733603334	0.793773833	-0.6654261	-0.1169361	0.52708774	0.78581748	0.79509784	-0.3307957	0.95938868	-0.0598127	-23.488457
unknown	361.2058	-2.362911449	-0.948422919	0.050126135	0.286849409	0.395877903	-1.336872552	0.438275251	-1.190090882	2.462008794	0.82099204	0.039195792	0.245200668	1.884748015	0.914371653	2.038834745	1.027744844	-0.2127795	-0.1717937	0.83756273	0.93496295	1.10190608	0.14000126	1.31713936	0.397408	-5.8175088
unknown	348.1904	-4.781947342	-0.946023326	0.002007749	0.049746038	0.452311865	-1.144610254	0.503216426	-0.99074908	4.622510737	0.819727791	0.001704609	0.057594588	1.743027234	0.801595111	1.870272295	0.903248329	-0.7365864	-0.1380553	0.48532171	0.76373168	0.77993108	-0.3585814	0.94204974	-0.0861249	-22.104603
unknown	305.1749	-4.802667495	-1.134071665	0.001960242	0.049074945	0.404453575	-1.305953983	0.448556191	-1.15663937	3.56075344	0.673465026	0.007392597	0.101989132	1.540006262	0.622936217	1.658678924	0.730034646	-0.6011842	-0.1776622	0.56666768	0.8043622	0.79427012	-0.3322984	0.95613791	-0.0647094	-17.101115
unknown	238.9941	2.437266241	0.949943318	0.04493883	0.275160666	1.699430083	0.765051008	1.869551764	0.902692417	-2.29080153	-0.798316494	0.051200752	0.28247048	0.574768076	-0.798948161	0.610214604	-0.712611388	0.90519448	0.59460728	0.39544856	0.71147145	1.59821071	0.67645763	1.91706264	0.93889748	-5.5832932
unknown	402.1282	-3.76633448	-0.838200159	0.007016485	0.103008133	0.495182988	-1.013966342	0.550590857	-0.860947441	2.56890396	0.900892596	0.033184093	0.226353002	1.954377229	0.96670896	2.111203433	1.078065602	-0.7332569	-0.1806683	0.48722603	0.76393386	0.78158961	-0.3555168	0.94231926	-0.0857122	-9.6753516
unknown	348.1907	-4.098678304	-0.973096102	0.00458065	0.08094964	0.46233758	-1.112981462	0.506215994	-0.982175005	3.690363542	0.770816416	0.006126994	0.094611384	1.653680687	0.725680688	1.779511065	0.831480903	-0.2156631	-0.045676	0.83539967	0.93441531	0.8509627	-0.2328322	1.01434169	0.02054372	-15.125613
unknown	347.1862	-5.253266204	-0.974767502	0.001181997	0.038328302	0.430930019	-1.214474493	0.480612934	-1.057052621	4.824654078	0.768156812	0.001313548	0.054879648	1.724152634	0.785887497	1.851870768	0.888983424	-0.5665647	-0.1369822	0.58870622	0.81602048	0.7919178	-0.3365774	0.95620906	-0.064602	-25.345192
unknown	326.1876	3.068894106	1.25083548	0.018095623	0.173049922	1.933883905	0.951501189	2.155491993	1.108017203	-2.216593291	-0.597253037	0.05748678	0.29672836	0.655474832	-0.609387708	0.704911816	-0.504485307	2.23884491	1.02607766	0.06018035	0.42379707	1.68635448	0.75390783	2.03155395	1.02258368	-6.8024901
unknown	347.187	-3.676280945	-0.928239133	0.007898393	0.109418909	0.464725969	-1.105547829	0.517093618	-0.951502596	3.556397593	0.801488884	0.007439692	0.102135219	1.694348667	0.760730786	1.818112649	0.862441591	-0.3696121	-0.0710728	0.72259966	0.89058258	0.81225078	-0.3000029	0.98219373	-0.0259205	-13.074317
unknown	289.1448	-2.620882845	-0.975844294	0.034365051	0.243608738	0.463677916	-1.108805078	0.51505638	-0.95719773	2.615300117	0.75730191	0.030875037	0.214415462	1.568417198	0.649309366	1.682243398	0.750386459	-0.9823763	-0.3273422	0.35864133	0.69900998	0.69420355	-0.5265694	0.83886664	-0.2534866	-6.8543952
unknown	302.1481	-5.68981941	-1.095080902	0.000743135	0.031393339	0.422830805	-1.241847608	0.465842499	-1.102085831	3.387020773	0.670717323	0.009541943	0.117453374	1.552215699	0.634329051	1.672557312	0.742055647	-0.5437225	-0.157006	0.60350976	0.82396509	0.82322016	-0.2806498	0.98640783	-0.0197438	-19.271537
unknown	347.1869	-3.757610359	-1.123775697	0.007097052	0.103321402	0.408572437	-1.291336214	0.456458878	-1.131443201	2.741887656	0.647855389	0.025375081	0.198406355	1.503917916	0.588725826	1.601750283	0.679649245	-2.6069282	-0.5131085	0.03506974	0.35621457	0.59441181	-0.7504653	0.71921486	-0.4755053	-10.302945
unknown	433.2212	-3.936240909	-0.768013431	0.005630417	0.091333215	0.51394847	-0.960304378	0.558883068	-0.839381628	4.848483427	0.945339271	0.001274308	0.054253644	1.956676238	0.96840506	2.114212143	1.080120146	-0.0009175	-0.0002053	0.99929351	0.99944677	0.86673717	-0.2063335	1.02656047	0.03781861	-19.084799
unknown	348.1906	-7.070162673	-1.165345992	0.000198816	0.022534203	0.400597395	-1.319775054	0.439995889	-1.184438051	3.257469252	0.614001739	0.011571013	0.132581196	1.533957311	0.617258334	1.638737853	0.712585087	-2.2258421	-0.3621325	0.0613461	0.42968627	0.69490933	-0.5251033	0.82972382	-0.2692969	-23.030838
unknown	397.1322	-5.400367973	-1.077118298	0.001008086	0.035647699	0.428951507	-1.221113535	0.471339253	-1.08516226	3.18454577	0.660202411	0.01290887	0.139170901	1.544239993	0.626896982	1.664769228	0.735322203	-0.508206	-0.1525496	0.62692861	0.83654624	0.83054665	-0.2678669	0.99258897	-0.0107317	-17.197719
unknown	329.1761	-3.92375905	-1.022851815	0.005721343	0.092120692	0.426406331	-1.229699237	0.475608539	-1.072153479	3.808202824	0.682898349	0.005175855	0.087859906	1.564984322	0.646148204	1.679586192	0.748105833	0.21194898	0.05156304	0.83818601	0.93496295	0.87837875	-0.1870849	1.06655647	0.09296035	-14.94247
unknown	304.0788	3.263876055	0.911568395	0.013790389	0.151188271	1.60849512	0.685711559	1.790757344	0.840569858	-3.651028769	-0.757852117	0.006484716	0.096325354	0.588346487	-0.765262064	0.628887135	-0.669126971	1.59182667	0.51822619	0.15545064	0.56601708	1.2576355	0.33071384	1.50497011	0.58973484	-11.916505
unknown	301.1448	-5.547332445	-1.069713668	0.000862333	0.03433527	0.430886289	-1.214620901	0.474529238	-1.075431113	3.165534205	0.638336581	0.013283717	0.141310146	1.518297172	0.602454193	1.636331558	0.7104651	-0.5018667	-0.1453163	0.63115865	0.83858712	0.8304498	-0.2680351	0.99474246	-0.007605	-17.560271
unknown	348.1907	-5.097398527	-1.013076914	0.001403563	0.040613708	0.450359719	-1.150850298	0.494768877	-1.015173343	3.878066737	0.671776994	0.004687575	0.084796192	1.539727368	0.622674922	1.652425016	0.724584807	-1.8301038	-0.2062318	0.10992863	0.51423571	0.76212606	-0.3918984	0.91015099	-0.1358222	-19.768052
unknown	258.1799	-4.521805404	-0.974706259	0.002726263	0.059871995	0.446666832	-1.162728966	0.49775105	-1.006503734	4.018033232	0.697098406	0.003851694	0.077342718	1.584914455	0.664404974	1.697015848	0.763000038	-0.9868844	-0.225107	0.35657523	0.69847615	0.7478309	-0.419216	0.90437905	-0.1450005	-18.168764
unknown	275.1656	-4.555781925	-0.977480923	0.002618017	0.058720754	0.449688791	-1.153001171	0.499523132	-1.001376606	3.996856893	0.691683199	0.00396714	0.078637742	1.560686589	0.64218085	1.680179868	0.748615686	-0.7092439	-0.1140912	0.50110633	0.77105517	0.7980512	-0.3254468	0.96303156	-0.054345	-18.208808
unknown	327.1605	-6.187315962	-1.024145856	0.000450792	0.026405285	0.432640872	-1.20875813	0.473990511	-1.077069919	3.991445102	0.657190587	0.003997237	0.078749184	1.587588993	0.666837465	1.702434446	0.767599247	-2.3228629	-0.6710401	0.05316979	0.40838657	0.56670795	-0.8193227	0.67277653	-0.5718007	-24.696332
unknown	306.1773	-5.341473307	-1.106529907	0.001074041	0.03720363	0.411619141	-1.280618024	0.455297213	-1.135119465	3.691874262	0.607577172	0.006113682	0.094611384	1.490562987	0.57585734	1.597065287	0.675423291	-1.6605496	-0.3695639	0.14076201	0.5519659	0.68328837	-0.5494335	0.82091563	-0.2846941	-19.720048
unknown	395.1842	-3.575070334	-0.764702787	0.009035475	0.119217362	0.522492182	-0.936518645	0.571908363	-0.806144092	3.803332312	0.879088873	0.005211868	0.087859906	1.850234185	0.887707885	1.984223126	0.988574266	-0.5614382	-0.1108168	0.59201064	0.81669164	0.80473059	-0.3134222	0.95239217	-0.0703723	-13.597181
unknown	348.1904	-3.399397177	-1.119202787	0.011451882	0.138806178	0.409186575	-1.289169283	0.456935281	-1.129938253	2.303475128	0.598187019	0.050197783	0.278912229	1.450095331	0.536147748	1.544853521	0.627470052	-2.40341	-0.5307838	0.04722896	0.39198715	0.58582104	-0.7714681	0.70919494	-0.4957459	-7.8304268
unknown	401.1247	-2.817507991	-0.760781768	0.02586561	0.209788113	0.513424574	-0.961775744	0.571637088	-0.806828573	2.374830922	0.873739171	0.044906524	0.264769841	1.93104514	0.949381889	2.089952	1.063469809	-0.9669121	-0.6338093	0.36579857	0.70172832	0.64763053	-0.6267571	0.78473083	-0.3497302	-6.6911051
unknown	331.1913	-2.53171879	-0.999711241	0.039134547	0.259183683	0.416189664	-1.26468696	0.468140331	-1.094987035	2.521745601	0.6593471	0.035711555	0.235465167	1.538175422	0.621220046	1.634897796	0.70920045	-1.6380279	-0.522073	0.14542639	0.5552675	0.55491511	-0.849661	0.67541171	-0.5661609	-6.3843507
unknown	333.2075	-5.151359937	-1.244992457	0.001322019	0.039762876	0.379454066	-1.398002841	0.422967142	-1.241382503	2.319598491	0.52293013	0.048950101	0.275650782	1.398756976	0.484145326	1.488889626	0.574236809	-2.5165784	-0.9348671	0.04001018	0.3651646	0.50437924	-0.9874192	0.60684049	-0.7206108	-11.949087
unknown	303.1518	-5.799862587	-1.054756331	0.000663634	0.029845207	0.434870277	-1.201342991	0.479587881	-1.060132892	3.23742799	0.613508902	0.011923492	0.133517315	1.496321808	0.581420483	1.607656646	0.684959317	-0.5409331	-0.137243	0.60533156	0.82488044	0.82571332	-0.2762871	0.99033433	-0.0140124	-18.776637
unknown	367.2128	-5.910412457	-1.124829883	0.000593217	0.029305823	0.400011579	-1.321886333	0.443687292	-1.172384863	4.178037562	0.574469572	0.003087906	0.071769533	1.464312163	0.550223141	1.573089602	0.653600848	-1.7430566	-0.4425308	0.12485115	0.53626544	0.66385906	-0.5910511	0.79918335	-0.3234016	-24.693925
unknown	334.2109	-3.882192739	-1.210827524	0.00603588	0.095072398	0.366920143	-1.446461986	0.411058545	-1.28258421	2.717669116	0.527921501	0.02634356	0.200069657	1.395141074	0.480411012	1.493887956	0.579071948	-2.9415486	-0.8292015	0.02166551	0.30716656	0.46835753	-1.0943178	0.56905887	-0.8133502	-10.550515
unknown	412.2424	-2.653011024	-0.72306437	0.032798045	0.240851406	0.558362261	-0.840726659	0.606274831	-0.721956164	2.787643766	0.880036492	0.023644084	0.191936909	1.846684347	0.884937288	2.005445436	1.003922714	-0.2399468	-0.061526	0.81724645	0.93185244	0.87610657	-0.1908217	1.04055643	0.0573552	-7.3956496
unknown	475.2013	-3.293223663	-0.823032635	0.013243629	0.149933511	0.516308095	-0.953695877	0.571812129	-0.806386874	2.920450697	0.771948533	0.019277964	0.173156481	1.669244221	0.739195045	1.78104541	0.8327243	-0.1064385	-0.0234841	0.91822063	0.9705018	0.8839309	-0.1779945	1.05612839	0.07878523	-9.6176973
unknown	317.2028	-5.543220794	-1.00723814	0.000866076	0.03433527	0.436425706	-1.196192013	0.486279618	-1.040141973	4.066226042	0.627566573	0.003602211	0.075216749	1.524210395	0.60806206	1.634289926	0.708663943	-0.7485262	-0.1025446	0.47853343	0.75972709	0.79935848	-0.3230855	0.96450321	-0.052142	-22.539989
unknown	334.2109	-3.345404994	-0.94349889	0.012328065	0.144014736	0.449035804	-1.155097611	0.502545036	-0.992675206	2.459985229	0.669930988	0.039319665	0.245362238	1.594628251	0.673220134	1.719410822	0.781914291	-0.2934326	-0.0951017	0.77770023	0.91655066	0.83408795	-0.2617286	1.01311897	0.0188036	-8.2296469
unknown	414.203	-4.518803721	-0.865469191	0.002736061	0.059871995	0.496916416	-1.008924891	0.546814897	-0.870875547	3.624236919	0.722130954	0.006741075	0.096825003	1.612335876	0.689152313	1.733286857	0.793510439	-0.9713783	-0.1695484	0.36372035	0.70082245	0.78654763	-0.346394	0.93896692	-0.0908538	-16.377215
unknown	398.2028	-2.34351905	-0.841276511	0.051577107	0.290444357	0.449634539	-1.153175233	0.506363802	-0.981753818	2.317001851	0.742638767	0.049148925	0.276531611	1.746056012	0.80409984	1.87391336	0.906054252	-0.4674552	-0.1525728	0.65437702	0.85285121	0.7027855	-0.5088437	0.85945883	-0.2184996	-5.429938
unknown	305.1748	-5.117983339	-1.083491976	0.001371814	0.040477837	0.422915302	-1.241559333	0.467690669	-1.09637345	3.216239291	0.567262821	0.012308465	0.136039831	1.435740834	0.521795352	1.54072341	0.623607893	-1.5707147	-0.3222162	0.16024182	0.56971676	0.70938972	-0.4953497	0.85208819	-0.2309253	-16.460659
unknown	269.1762	-2.820515868	-0.784710114	0.025754197	0.209144603	0.503169617	-0.990883285	0.560727504	-0.834628258	3.290299046	0.774626568	0.01101712	0.128290433	1.723504202	0.785344816	1.828958402	0.871022263	0.22363096	0.06513617	0.82943086	0.93337638	0.8951703	-0.1597659	1.08255653	0.11444236	-9.2803407
unknown	331.1913	-2.854587671	-0.921695974	0.024526829	0.204004403	0.463787907	-1.108462892	0.520904852	-0.940908221	2.367863876	0.658885908	0.045397534	0.265769216	1.535397714	0.618612405	1.640814881	0.714412481	-0.1610425	-0.0474892	0.87660921	0.95026982	0.8150563	-0.2950284	0.99261953	-0.0106873	-6.759275
unknown	301.1807	-3.257248818	-0.901449221	0.013917175	0.151808603	0.469111902	-1.091995991	0.52341737	-0.933966292	3.192849005	0.666874562	0.012748656	0.139170901	1.540058139	0.622984815	1.650659681	0.723042709	-1.7345108	-0.3325248	0.12641635	0.53768295	0.67046555	-0.5767649	0.81352357	-0.2977439	-10.399904
unknown	105.0386	-5.288304388	-0.833805626	0.001137731	0.038328302	0.486450849	-1.039634051	0.536452919	-0.898476535	3.701727984	0.720386966	0.006027607	0.094611384	1.692630222	0.759266832	1.804046162	0.851236255	-1.6933754	-0.2478294	0.13421486	0.54822576	0.7218095	-0.47031	0.86477447	-0.2096042	-19.575864
unknown	311.1657	-2.961584703	-0.911380816	0.021057645	0.186643306	0.478935596	-1.06209643	0.53264332	-0.908758327	2.219649597	0.648442522	0.05721337	0.29652546	1.51053151	0.59505628	1.625243159	0.700655582	-1.2027792	-0.3640746	0.26816098	0.64888539	0.68774399	-0.5400565	0.82809419	-0.2721332	-6.5736803
unknown	273.0761	3.361370444	0.919202876	0.012061705	0.143023707	1.690716838	0.757635057	1.877377116	0.908718479	-2.247556948	-0.640686803	0.054775728	0.290873392	0.638019461	-0.648327665	0.682955814	-0.550135854	2.61397672	0.90170701	0.03471194	0.35553892	1.74546612	0.80361235	2.08096301	1.05725132	-7.5548715
unknown	261.1505	2.387213815	0.753329961	0.04836647	0.283886363	1.480881385	0.566456089	1.653086988	0.725162644	-2.545620737	-0.779660796	0.0344084	0.230594988	0.574984224	-0.798405722	0.619461182	-0.690914215	-0.3262928	-0.1272039	0.75374136	0.90685376	0.8595758	-0.2183032	1.03966078	0.05611288	-6.076941
unknown	305.1454	2.385466143	0.520596209	0.04849088	0.284105144	1.217780838	0.284254517	1.359853946	0.443451709	-3.520680388	-1.124834483	0.007838097	0.10473818	0.485227633	-1.043266382	0.522728935	-0.935865075	-0.5086969	-0.140731	0.62660162	0.83636851	0.77893086	-0.3604328	0.94425393	-0.0827532	-8.3984639
unknown	315.1967	-5.295731142	-0.968306384	0.001128587	0.038328302	0.447836017	-1.158957533	0.500011011	-0.99996823	3.661702446	0.598064485	0.006385496	0.09572121	1.498140655	0.583173079	1.604248033	0.681897214	-0.7242479	-0.0914947	0.49240356	0.76688884	0.80405711	-0.3146301	0.97169361	-0.0414266	-19.391392
unknown	336.2264	-3.396763543	-1.113056414	0.011493029	0.138981779	0.385022281	-1.37698616	0.430330398	-1.216483342	2.34613415	0.519330151	0.046963817	0.270301015	1.422171177	0.508095122	1.525335111	0.609126232	-1.3850276	-0.5184644	0.2085761	0.60644125	0.60898355	-0.7155248	0.73860711	-0.437121	-7.9692629
unknown	376.2209	-5.8670715	-0.96778822	0.000619777	0.029500467	0.447966184	-1.158538265	0.500141297	-0.999592361	3.943061766	0.596960114	0.004277467	0.08061666	1.503380461	0.588210159	1.606824721	0.684212562	-0.7842006	-0.096812	0.4586325	0.74978864	0.80636542	-0.3104943	0.97382323	-0.0382682	-23.134225
unknown	627.4995	-2.709698854	-0.693055038	0.030212167	0.229352204	0.524296721	-0.931544573	0.572778414	-0.803950972	2.626661744	0.827617526	0.030335011	0.212932836	1.886651107	0.915827654	2.047603609	1.033936454	1.38866115	0.6251425	0.2075166	0.60627947	1.39109628	0.47622228	1.65940745	0.73066817	-7.1174623
unknown	316.1998	-5.255288856	-0.961934058	0.00117939	0.038328302	0.449310443	-1.154215501	0.501752898	-0.994951051	3.672038102	0.592810665	0.00629096	0.095068095	1.49291047	0.578127649	1.598647783	0.676852117	-0.7275147	-0.0943389	0.49052193	0.76606422	0.80215263	-0.3180513	0.96954183	-0.044625	-19.297621
unknown	346.1741	-2.954306602	-0.586604584	0.021276337	0.186842438	0.598129716	-0.741469701	0.658948508	-0.601762361	4.027561709	0.965080056	0.003800931	0.076974762	1.969933875	0.978147203	2.089817425	1.063376908	2.28135668	0.35839739	0.05652311	0.41460874	1.11966564	0.16306797	1.33979176	0.42200879	-11.898652
unknown	299.2235	-5.573658316	-0.820304059	0.000838791	0.03418599	0.49937033	-1.001817988	0.553424041	-0.853542777	4.739322411	0.684418102	0.001465233	0.055229968	1.59237813	0.671182962	1.710927511	0.774778637	0.68045109	0.17004015	0.51808454	0.78077867	1.0013967	0.00201361	1.20308424	0.26673767	-26.415364
unknown	383.1477	-5.540262202	-0.997223144	0.000868781	0.03433527	0.451471688	-1.147292578	0.496271194	-1.010799379	2.917915847	0.561019319	0.019352958	0.173386199	1.446484974	0.532551337	1.558854924	0.640486669	-0.5451425	-0.152929	0.60258356	0.82361086	0.82258501	-0.2817633	0.98385266	-0.0234858	-16.166019
unknown	396.2463	-3.199773683	-1.020292302	0.015070548	0.157998467	0.417625434	-1.259718518	0.467684027	-1.096393937	2.427578031	0.547691646	0.041358217	0.251900907	1.444352673	0.530423053	1.544585682	0.627219903	-1.6999054	-0.4388715	0.13294718	0.54637998	0.60562407	-0.7235056	0.73772022	-0.4388543	-7.7677003
unknown	119.0859	2.49661074	0.761544812	0.041196029	0.264069022	1.585669709	0.665092293	1.742252758	0.800953939	-2.487441092	-0.718865283	0.037672176	0.239448539	0.584433399	-0.774889467	0.623097404	-0.682470389	2.91378301	0.42439102	0.02253872	0.31072941	1.17248522	0.22956974	1.39803385	0.48339929	-6.2101721
unknown	370.0164	-4.083459195	-0.776183836	0.004669279	0.081404749	0.495919221	-1.011822951	0.547443495	-0.869218031	3.639385218	0.696130126	0.006594828	0.096587859	1.66299717	0.733785714	1.792641323	0.842086858	-1.5903164	-0.7536172	0.15578892	0.56601708	0.5865394	-0.7697001	0.69933379	-0.5159469	-14.861281
unknown	383.1218	-2.47042983	-0.680582661	0.042806019	0.267629961	0.528861509	-0.919038116	0.582722576	-0.779118891	2.408460664	0.785717094	0.042610426	0.255858735	1.840642918	0.880209773	1.959153908	0.970230738	-0.5429789	-0.2483185	0.60399514	0.82398584	0.77483315	-0.3680424	0.92799922	-0.1078045	-5.9499331
unknown	381.1467	-3.92770516	-0.881732168	0.005692425	0.091881945	0.488052354	-1.03489218	0.537761853	-0.894960675	3.001241506	0.595688284	0.017039411	0.162685206	1.457374137	0.543371294	1.570712154	0.651418819	-0.4635673	-0.0734488	0.65702699	0.85325864	0.82880042	-0.2709034	0.99445971	-0.0080152	-11.787992
unknown	357.1354	2.856742568	0.715859049	0.024451303	0.204004403	1.444148761	0.530219361	1.614500909	0.691088253	-2.307732867	-0.732227133	0.049865251	0.278877617	0.620712802	-0.688002194	0.661503774	-0.596178708	0.01535501	0.00727325	0.98817743	0.99670416	0.96098387	-0.0574159	1.15683976	0.21018904	-6.5925987
unknown	525.1792	-3.82699446	-1.013936148	0.00648306	0.098870669	0.439487413	-1.186106248	0.47457876	-1.075280562	2.725424223	0.503955517	0.026029372	0.199600786	1.399619779	0.485034957	1.508204195	0.592831768	-2.1636647	-0.5805331	0.0672431	0.44603925	0.59183417	-0.7567351	0.69736629	-0.5200115	-10.430183
unknown	332.1952	-7.357196938	-1.112644697	0.000154975	0.02062434	0.405732347	-1.30139977	0.451749029	-1.146406594	2.859229261	0.452477649	0.021176477	0.180748439	1.361223129	0.44490357	1.459989767	0.545958258	-2.8340026	-0.5640874	0.02526081	0.32426327	0.60495497	-0.7251003	0.72851528	-0.4569689	-21.035913
unknown	257.176	-3.966226334	-0.884755008	0.005418365	0.089000405	0.469231168	-1.091629249	0.523455996	-0.933859831	3.798418639	0.561266713	0.005248473	0.087859906	1.444871686	0.530941378	1.547589798	0.630023124	-0.550348	-0.1244297	0.59919482	0.82190775	0.78711269	-0.3453579	0.95400244	-0.0679351	-15.065388
unknown	439.3798	3.793948696	0.861067728	0.006767957	0.101214625	1.580366345	0.660259028	1.715542187	0.778664604	-2.95511941	-0.568049396	0.01828206	0.168624202	0.685458935	-0.544857857	0.735645839	-0.442916716	0.0697346	0.0159901	0.94635523	0.98311685	0.87231559	-0.1970779	1.03032799	0.04310367	-11.211571
unknown	297.1348	4.444493817	1.150557741	0.002991439	0.063334972	1.897153517	0.923836426	2.107396643	1.075461876	-2.362219151	-0.416489492	0.045799299	0.267097302	0.739052107	-0.436252009	0.790860445	-0.338504955	2.69480307	1.06836122	0.03087034	0.34698251	1.86901181	0.90227568	2.25497122	1.17310902	-10.498868
unknown	276.1688	-3.202308978	-0.845126988	0.015017572	0.157696599	0.47953139	-1.060302838	0.534203832	-0.90453777	3.329022535	0.555459198	0.010399513	0.124431603	1.424264039	0.510216627	1.530865042	0.614347103	-0.3107827	-0.0742567	0.76501571	0.91202558	0.8084215	-0.3068204	0.97833726	-0.0315962	-10.660559
unknown	299.2002	-3.314808009	-0.824802226	0.012856356	0.146823641	0.503527219	-0.989858329	0.560232667	-0.835901985	2.325665614	0.564718085	0.048488671	0.274236446	1.445982085	0.532049679	1.555692547	0.637556968	-0.8494501	-0.2092575	0.42372716	0.7292491	0.75780117	-0.4001087	0.9138775	-0.1299273	-7.709135
unknown	345.1717	-4.140503773	-0.679674446	0.004346426	0.078949978	0.560377507	-0.835529046	0.616357573	-0.698160538	4.293567433	0.665629957	0.002638604	0.069134803	1.552049275	0.634174361	1.66707752	0.737321192	0.36716819	0.09581229	0.72434234	0.89155	0.97004479	-0.0438767	1.15805559	0.21170451	-17.777532
unknown	329.1762	-2.939422614	-0.861543929	0.021731087	0.189449761	0.477635533	-1.066017927	0.52969501	-0.916766178	2.700694229	0.515514774	0.027044945	0.201747615	1.391216283	0.476346724	1.487479681	0.572869961	-1.3994286	-0.3659542	0.20440498	0.60376552	0.65541033	-0.6095297	0.786674	-0.3461622	-7.9384817
unknown	417.2271	-2.953895907	-0.714048751	0.021288749	0.186842438	0.511656215	-0.966753315	0.572991102	-0.803415359	2.334558995	0.617531676	0.047820142	0.272444423	1.609224103	0.686365252	1.733458428	0.793653238	-0.3319232	-0.1083952	0.74966431	0.90398722	0.81383165	-0.2971977	0.98528068	-0.0213933	-6.8960443
unknown	345.1711	-2.580748801	-0.494798301	0.03643254	0.251470631	0.642766108	-0.637634234	0.704195186	-0.50595273	4.550280262	0.859037799	0.001873685	0.059893636	1.771070223	0.824621416	1.898265996	0.924682165	2.53296896	0.44743777	0.03906313	0.3651646	1.20555423	0.26969655	1.43544426	0.52149731	-11.74313
unknown	303.1603	-3.496631534	-0.791084664	0.010038503	0.127356179	0.527158	-0.923692664	0.582101135	-0.780658264	2.207855926	0.525202131	0.058275561	0.298519195	1.400151199	0.485582628	1.509926476	0.594478301	0.54541404	0.10026343	0.60240652	0.82356423	0.94947001	-0.0748057	1.14050238	0.18966946	-7.7200587
unknown	289.1812	-3.806857677	-0.752700673	0.006655075	0.100225732	0.53211534	-0.9101891	0.587772869	-0.766669328	2.729949599	0.54407187	0.025847814	0.199235925	1.420259531	0.506154585	1.532578669	0.615961131	-1.4577153	-0.2528752	0.18827328	0.58827506	0.73576269	-0.4426876	0.88509978	-0.176088	-10.39253
unknown	433.3171	4.336212917	0.710571676	0.003411826	0.068993027	1.44099107	0.527061395	1.590588725	0.66956085	-2.327741622	-0.557583246	0.048331782	0.273824113	0.684756919	-0.546336157	0.740063562	-0.43427891	-0.0886402	-0.0174348	0.9318505	0.97630958	0.87696074	-0.1894158	1.05104688	0.07182702	-10.093583
unknown	383.1477	-2.402375898	-0.716679154	0.047300758	0.281705789	0.539274289	-0.890908842	0.59498817	-0.749067111	2.469804528	0.535995348	0.03872225	0.243888938	1.388727335	0.473763366	1.494251851	0.57942333	-0.391514	-0.0949258	0.70706226	0.88071901	0.79788372	-0.3257496	0.96031356	-0.0584225	-5.9333989
unknown	353.3072	3.030924725	0.881958163	0.019089675	0.17651305	1.519191987	0.603304201	1.684909268	0.752670905	-2.739540676	-0.432094927	0.025467311	0.198573795	0.737676874	-0.438939087	0.793253555	-0.334146013	1.52700334	0.48859666	0.17059785	0.57650441	1.14588449	0.19646162	1.38367361	0.46850367	-8.3033416

unknown	482.3154	3.659125202	0.520864101	0.008079672	0.111106439	1.264931692	0.339059479	1.38653325	0.471482214	-4.699130809	-0.730497376	0.001543194	0.05569795	0.605500224	-0.723800601	0.650238958	-0.620958099	-1.0391815	-0.181833	0.33327735	0.69059472	0.76949373	-0.3780185	0.91646181	-0.1258533	-17.194708
unknown	480.3096	2.627435457	0.477990531	0.034039248	0.24312151	1.225889647	0.293829116	1.338394415	0.42050333	-4.232449735	-0.776110916	0.00286678	0.070662721	0.589319517	-0.762878048	0.632387441	-0.661119378	-1.1310995	-0.2404679	0.29527145	0.6662509	0.73776472	-0.4387673	0.87593222	-0.1911089	-11.120489
unknown	255.1604	-3.085480779	-0.595314761	0.017678786	0.171297715	0.579627107	-0.786803028	0.642270827	-0.638746327	4.248252427	0.621667739	0.002805809	0.070662721	1.529817822	0.61335986	1.63619677	0.710346258	0.12468163	0.03324427	0.9042806	0.96313894	0.90456628	-0.1447019	1.08731667	0.12077217	-13.107901
unknown	330.1794	-2.9047587	-0.774812263	0.022830478	0.195390534	0.511131958	-0.968232296	0.56658622	-0.81963258	2.608869621	0.472404746	0.031185024	0.216107911	1.354364437	0.437615996	1.446770912	0.532836498	-1.5946933	-0.3686051	0.15481043	0.56601708	0.65925716	-0.6010868	0.79076607	-0.3386771	-7.5781367
unknown	481.3127	2.753483882	0.495179705	0.028361134	0.221754139	1.239218605	0.309430709	1.352876369	0.436030007	-4.023734198	-0.733163076	0.003821235	0.077146367	0.608283616	-0.717183949	0.652008631	-0.617037033	-0.9343473	-0.1947436	0.38122637	0.70697525	0.76063185	-0.3947297	0.90292985	-0.1473142	-11.079287
unknown	227.0648	-3.13923526	-0.620265966	0.016396694	0.164244	0.571016329	-0.808396094	0.630728728	-0.664908448	3.059883053	0.576649852	0.015585364	0.1535229	1.485285937	0.570740696	1.592391183	0.671194789	-1.5137884	-0.4542051	0.17384756	0.57867838	0.67302739	-0.5712629	0.80436371	-0.3140801	-9.6056928
unknown	110.0714	-3.119771835	-0.736052632	0.016849085	0.167234216	0.50597646	-0.982857828	0.56417076	-0.825796198	2.509122465	0.48323642	0.036420688	0.237975259	1.416583601	0.502415746	1.510476851	0.595004074	-0.9176959	-0.2398948	0.38930214	0.70820313	0.71777484	-0.4783967	0.86996506	-0.2009706	-7.8278896
unknown	412.3521	4.623443138	0.842126992	0.002416303	0.055693055	1.573164979	0.653669975	1.71486983	0.77809907	-2.979687074	-0.416366255	0.017608819	0.165218861	0.74518656	-0.424326441	0.801653084	-0.31895005	1.29526828	0.39867106	0.23630614	0.62724807	1.18062653	0.23955267	1.40180802	0.48728878	-13.776414
unknown	74.0247	-3.894814522	-0.591642887	0.005938421	0.094652316	0.571418707	-0.807379828	0.631860867	-0.662321176	6.562670417	0.589769944	0.000176122	0.028695533	1.504599143	0.589379174	1.609301346	0.6864345	-1.5396942	-0.2277756	0.16752948	0.57306625	0.7286249	-0.4567518	0.8757434	-0.1914199	-25.560384
unknown	535.25	2.982424449	0.591151397	0.020444516	0.184141836	1.333202448	0.414895871	1.486125406	0.571555862	-3.221600414	-0.589953052	0.012209841	0.135408793	0.65366492	-0.61337682	0.697442181	-0.519854476	-0.2252946	-0.090392	0.82818612	0.93337638	0.9034192	-0.1465325	1.09072947	0.12529332	-9.6081798
unknown	271.228	-5.248414032	-0.608890872	0.001188277	0.038328302	0.580421708	-0.784826617	0.641583839	-0.640290293	3.440016209	0.562942886	0.008823596	0.113488505	1.478758446	0.564386409	1.584447835	0.663980163	-0.4264621	-0.0934734	0.68257938	0.86732271	0.83801793	-0.254947	1.00401296	0.00577789	-18.054629
unknown	256.1638	-2.500238326	-0.554413762	0.040977926	0.263674223	0.595043289	-0.748933469	0.659050662	-0.601538723	3.985244241	0.615411631	0.004032023	0.078798929	1.517682642	0.601870145	1.622184069	0.697937531	0.20159934	0.05650624	0.84596326	0.93738766	0.91387418	-0.1299326	1.09830164	0.13527433	-9.9640604
unknown	423.4205	2.959697997	0.785016731	0.021114107	0.186701621	1.520019921	0.604090232	1.656055614	0.727751123	-2.393969173	-0.431390667	0.043585009	0.260272748	0.731644451	-0.450785366	0.780409974	-0.357695878	1.15297978	0.34227794	0.28676539	0.66244135	1.13953904	0.18845036	1.3396528	0.42185915	-7.0854258
unknown	480.3096	3.176845351	0.461831215	0.015558819	0.161046121	1.212969026	0.278542711	1.327078923	0.408254172	-4.67029774	-0.713345532	0.001601898	0.056464757	0.612786928	-0.706542572	0.658568013	-0.602595653	-1.1441527	-0.2080599	0.29017233	0.66361752	0.75585388	-0.4038207	0.89928389	-0.1531515	-14.836814
unknown	481.3127	3.18626608	0.463830926	0.015356184	0.159709213	1.214474676	0.280332408	1.329376239	0.410749472	-4.462108229	-0.709399396	0.002105163	0.06228907	0.615147833	-0.700994932	0.66110395	-0.597050961	-1.1867452	-0.2149334	0.27403766	0.6550585	0.75217804	-0.4108539	0.89512276	-0.1598426	-14.217464
unknown	482.3155	2.665577079	0.50750385	0.032205563	0.239194167	1.245125767	0.316291473	1.356522852	0.439913351	-4.085427872	-0.645817531	0.003507701	0.074959362	0.642723843	-0.637729101	0.68856149	-0.5383426	-0.6439435	-0.1391593	0.54012921	0.7903383	0.7871438	-0.3453009	0.93251459	-0.1008018	-10.890023
unknown	357.1692	-4.650487228	-0.678262909	0.002340532	0.054315343	0.556588802	-0.845316211	0.612311841	-0.707661513	2.355889704	0.471055635	0.046254043	0.267848154	1.376962344	0.461489107	1.486870692	0.572279187	-0.7667336	-0.1488853	0.46830477	0.75431383	0.79774617	-0.3259983	0.95343602	-0.068792	-10.956035
unknown	158.0813	-2.324053644	-0.64117713	0.053076634	0.295272751	0.554146726	-0.851660073	0.614271581	-0.703051456	2.228146441	0.494800654	0.056460014	0.294305158	1.39236957	0.477542189	1.490541269	0.57583632	-0.2217134	-0.0558868	0.83086623	0.93337638	0.81481978	-0.2954471	0.98437309	-0.0227229	-5.1783319
unknown	157.0796	-3.895431406	-0.687872583	0.005933701	0.094652316	0.533335115	-0.906885777	0.590125778	-0.760905615	2.863427629	0.447421893	0.021040296	0.180499402	1.369420667	0.45356569	1.46591878	0.551805172	-1.6694524	-0.3346273	0.1389572	0.55113297	0.6840235	-0.5478822	0.82306771	-0.280917	-11.154286
unknown	307.1561	2.441405817	0.354368879	0.044666746	0.274525779	1.119978082	0.1634705	1.233963287	0.303299472	-2.951493788	-0.842886862	0.018383659	0.169082992	0.587401821	-0.767580358	0.626527138	-0.674551094	-1.3159222	-0.6420172	0.22965508	0.62241386	0.64435309	-0.6340766	0.76852141	-0.3798427	-7.2057941
unknown	534.2458	3.069483293	0.652954828	0.018080638	0.173049922	1.395496434	0.480778438	1.550026482	0.632292864	-2.286738538	-0.453406544	0.051526503	0.283092644	0.716230687	-0.481503762	0.767988316	-0.380843733	0.04536211	0.01772102	0.96508563	0.9896738	0.96342566	-0.0537547	1.16295119	0.21779054	-7.0191057
unknown	303.2333	2.520963784	0.529158134	0.039754491	0.260280154	1.205112783	0.26916817	1.341145139	0.423465375	-4.133961297	-0.546647274	0.003280522	0.073261239	0.690840795	-0.533574816	0.740632678	-0.433169889	0.06112852	0.01833168	0.95296555	0.98557894	0.86937995	-0.2019413	1.05087373	0.07158933	-10.421567
unknown	156.0767	-3.504397827	-0.646023226	0.009934021	0.126488734	0.549548409	-0.863681524	0.607534598	-0.718961525	2.494133741	0.418522909	0.037281284	0.239448539	1.345681822	0.428337334	1.438863268	0.524929503	-1.5452711	-0.3247739	0.16619722	0.57183885	0.69109092	-0.5330526	0.83057924	-0.2678103	-8.7404369
unknown	479.2028	-2.7578051	-0.641239392	0.028185004	0.220906741	0.563060679	-0.82863769	0.623731487	-0.681003004	2.370404862	0.417483582	0.045217834	0.265644591	1.312391495	0.39219815	1.408935827	0.494605903	0.65615987	0.16217065	0.53268876	0.78698641	0.96240373	-0.0552859	1.16102825	0.21540308	-6.5371146
unknown	315.1602	-2.318266779	-0.454102804	0.053530935	0.296832676	0.647872134	-0.626218988	0.7054951	-0.503292032	2.752425943	0.583942745	0.024965169	0.196957378	1.508605512	0.593215602	1.623544607	0.699147023	0.09102792	0.02939644	0.93002049	0.97559568	0.92681839	-0.1096414	1.09513169	0.13110436	-6.3808576
unknown	332.1952	-2.640514	-0.570670798	0.033398541	0.242005398	0.614779128	-0.701859909	0.678413868	-0.559762434	2.276852991	0.442982956	0.052327727	0.284120825	1.311286897	0.390983368	1.404840484	0.490406326	0.44632049	0.08217434	0.66884557	0.85931616	0.93833601	-0.0918235	1.12431472	0.16904594	-6.0120622
unknown	308.2679	2.37390922	0.665181506	0.049321851	0.285863059	1.302543634	0.381331703	1.444948993	0.531018566	-3.256436247	-0.379139974	0.01158891	0.132581196	0.771595168	-0.374083986	0.825928376	-0.275911417	2.10093138	0.62852733	0.07376967	0.45852206	1.25903847	0.33232237	1.52103899	0.60505714	-7.730484
unknown	445.3321	-4.212913478	-0.467306841	0.00397136	0.075641769	0.639610035	-0.644735522	0.705909611	-0.502444631	3.057750557	0.506160387	0.015635904	0.15378843	1.427525748	0.513516767	1.531323028	0.614778647	-0.7635945	-0.1104952	0.4700576	0.75440126	0.81838317	-0.2891516	0.97820906	-0.0317853	-12.882039
unknown	334.1253	-2.853658007	-0.525352024	0.024559488	0.204015824	0.59511134	-0.748768486	0.659530735	-0.600488202	2.367799846	0.448240043	0.045402071	0.265769216	1.386272938	0.471211332	1.483636658	0.569137819	-0.4963025	-0.0973593	0.63488383	0.84089716	0.79330214	-0.3340577	0.95632078	-0.0644335	-6.756891
unknown	262.0687	-3.244263954	-0.441018988	0.014169225	0.152471148	0.643418557	-0.63617055	0.709140297	-0.495857015	4.263250959	0.513893979	0.002749233	0.070581687	1.44536913	0.531437987	1.538843013	0.621846061	-1.6462957	-0.2711283	0.14369749	0.55392002	0.72928041	-0.4554544	0.87132849	-0.1987114	-13.831111
unknown	246.0969	-2.357816386	-0.403829012	0.050503274	0.287877489	0.667853179	-0.582397121	0.741191778	-0.432081217	2.445172415	0.560454552	0.040238561	0.248029616	1.492010924	0.577258099	1.592197202	0.671019033	-1.0248062	-0.1935601	0.33955897	0.6932229	0.76061179	-0.3947678	0.91881091	-0.1221601	-5.7652676
unknown	119.049	-3.515971782	-0.494906221	0.009780486	0.125548327	0.620208267	-0.689175338	0.682429363	-0.551248372	3.121463489	0.448578744	0.014197243	0.146272419	1.371947681	0.456225465	1.468945018	0.554780397	-0.7881235	-0.2058817	0.45647919	0.7482908	0.77300801	-0.3714447	0.92499216	-0.112487	-10.974978
unknown	335.1296	-3.255560998	-0.527542579	0.013949664	0.151808603	0.603105756	-0.729517092	0.667691152	-0.582747173	2.226493136	0.418028638	0.056605827	0.294829549	1.34567951	0.428334856	1.441601069	0.527671986	-0.0820344	-0.0132965	0.93691578	0.97910702	0.84543464	-0.2422349	1.01986889	0.0283837	-7.2484842
unknown	241.0831	2.764790037	0.416209479	0.027902722	0.219221267	1.176393613	0.234370857	1.303627431	0.382531615	-2.941203799	-0.514167441	0.018675222	0.170379238	0.705404012	-0.503478314	0.752438422	-0.410354574	0.20961146	0.02265711	0.83994088	0.93577972	0.88601353	-0.1745994	1.06119171	0.08568531	-8.131811
unknown	432.2375	2.740721259	0.450444181	0.028888093	0.223728331	1.211637648	0.276958311	1.333627039	0.41535526	-3.201603795	-0.465321485	0.01258199	0.13808775	0.728313634	-0.457368243	0.773360033	-0.370787887	2.43003571	0.27750359	0.04541816	0.39039698	1.05823266	0.08165684	1.25886257	0.33212079	-8.7747036
unknown	285.2073	-2.480253195	-0.500295451	0.042194466	0.266102627	0.621605213	-0.685929491	0.693107303	-0.528849375	2.633026835	0.418781487	0.0300367	0.212649365	1.305167735	0.384235228	1.398326633	0.483701397	0.20330652	0.04579137	0.8446791	0.93691995	0.90060102	-0.15104	1.08942162	0.1235624	-6.5305732
unknown	402.2225	3.098162377	0.449136899	0.017366983	0.170045191	1.222265921	0.289558197	1.342308087	0.424715837	-3.224664098	-0.455536505	0.012153854	0.13524792	0.719523151	-0.474886988	0.772283719	-0.372797137	-0.1001081	-0.0220155	0.92306531	0.9724816	0.89479669	-0.1603682	1.06840504	0.09545869	-9.990533
unknown	242.0864	3.069474202	0.399121704	0.018080869	0.173049922	1.161590408	0.216101445	1.282615125	0.359088325	-3.048690984	-0.509432589	0.01585253	0.154475374	0.711696191	-0.490666581	0.76001638	-0.395897583	0.07085152	0.00721618	0.94549765	0.98293743	0.8776368	-0.1883041	1.04830438	0.06805768	-9.3578783
unknown	183.0841	-2.314874269	-0.409103067	0.053799104	0.297466341	0.653674234	-0.613356264	0.720991685	-0.471945474	2.907946209	0.485706774	0.019650858	0.175059082	1.414845545	0.500644567	1.510552876	0.595076685	-0.9204364	-0.2369902	0.38796429	0.70820313	0.74461302	-0.4254372	0.89254284	-0.1640067	-6.7315299
unknown	455.2875	2.442786897	0.487586874	0.044576348	0.274525779	1.23870943	0.308837808	1.357529254	0.440983288	-2.334070067	-0.40277034	0.047856655	0.272444423	0.749493637	-0.416011865	0.805450918	-0.312131417	0.29374863	0.07146231	0.77746851	0.91655066	0.92081855	-0.1190112	1.0964978	0.13290292	-5.7016358
unknown	433.2413	2.609488513	0.420337006	0.034939331	0.245666463	1.185258314	0.245201513	1.303192714	0.382050443	-3.286836366	-0.443365691	0.011074201	0.128725245	0.737610563	-0.439068779	0.784638714	-0.349899576	2.24806873	0.26322581	0.059367	0.42125378	1.04716846	0.06649355	1.24503137	0.31618209	-8.5769617
unknown	564.3668	2.430584929	0.445811295	0.045381571	0.276314868	1.204302285	0.26819756	1.316320283	0.396510563	-2.487776708	-0.415376281	0.037652476	0.239448539	0.7438248	-0.426965244	0.800242634	-0.321490603	-0.1433134	-0.0347696	0.89008095	0.95731781	0.8625189	-0.213372	1.02494491	0.03554636	-6.0467526
unknown	343.1702	4.305569603	0.809938596	0.003542303	0.069998049	1.530399623	0.613908423	1.662072169	0.732983027	-2.280402854	-0.222097388	0.052038592	0.283143399	0.853656642	-0.22827219	0.916180338	-0.126296493	1.25092728	0.2386224	0.25114915	0.63626403	1.01636567	0.02341955	1.19873809	0.26151648	-9.8184332
unknown	227.1651	2.50816608	0.627520419	0.04050542	0.262560481	1.312681425	0.39251683	1.455339338	0.541355582	-2.251581088	-0.283801877	0.054432839	0.290709702	0.814093943	-0.296732809	0.871876286	-0.197804655	1.37033489	0.4568396	0.21290929	0.61279315	1.20066067	0.26382847	1.44615677	0.53222395	-5.6473393
unknown	136.0753	-2.557864172	-0.39361879	0.037668807	0.255026892	0.662633645	-0.593716636	0.73125837	-0.451546863	2.895041264	0.443675776	0.02004355	0.177828557	1.374675337	0.459090931	1.465960088	0.551845826	-0.8474094	-0.2148761	0.42478942	0.72934467	0.76065956	-0.3946772	0.9120655	-0.1327907	-7.4051223
unknown	160.0794	3.741193244	0.427318525	0.007251403	0.104477332	1.196332451	0.258618358	1.321279449	0.401935628	-3.0226701	-0.38608911	0.016492323	0.159287085	0.757352217	-0.400963693	0.810441688	-0.303219709	1.15145772	0.11003953	0.28735048	0.66265181	0.95102765	-0.0724408	1.13817625	0.18672398	-11.308393
unknown	165.0542	-2.352023401	-0.382774245	0.050935593	0.289577688	0.664343428	-0.58999887	0.733811063	-0.446519441	3.164141422	0.423000926	0.013311624	0.141376381	1.350483815	0.433476349	1.441227535	0.52729812	-0.8595823	-0.2167617	0.41848134	0.72423483	0.75665822	-0.4022863	0.90726894	-0.1403978	-7.4421347
unknown	147.0436	-2.772839251	-0.381418173	0.027581102	0.217361705	0.675454709	-0.566069058	0.744374645	-0.42589918	2.228270128	0.405121101	0.056449121	0.294305158	1.331124365	0.412645366	1.426350668	0.512328712	-1.4827392	-0.2733423	0.18170637	0.58427378	0.72392605	-0.4660858	0.86758124	-0.2049292	-6.1786349
unknown	253.1235	5.490081313	0.647284219	0.000916118	0.034935692	1.376931735	0.461457036	1.509711372	0.59427276	-2.964063099	-0.23821229	0.018033923	0.167281951	0.842775402	-0.246779887	0.904482514	-0.144835483	1.64808558	0.25278403	0.14332576	0.55392002	1.05147411	0.07241333	1.25086409	0.32292505	-16.272947
unknown	177.1061	4.83806601	0.445205636	0.001881934	0.048506299	1.206178016	0.270442846	1.331885169	0.413469703	-2.456609727	-0.346202674	0.039527179	0.245813029	0.784377045	-0.350380779	0.841642366	-0.248720767	0.44554692	0.03226724	0.66937807	0.85931616	0.89937001	-0.1530133	1.07627667	0.10604899	-11.88524
unknown	178.107	4.82539934	0.426191393	0.00190955	0.049024313	1.184874865	0.244734704	1.30887857	0.388331258	-2.883872308	-0.342700684	0.020389984	0.178173227	0.787784148	-0.344127709	0.843584406	-0.245395668	-0.2197063	-0.0193208	0.83236938	0.93390928	0.86219076	-0.213921	1.0324875	0.04612431	-13.915836
unknown	159.0762	4.284673054	0.400457159	0.003634421	0.071601381	1.170362122	0.226954983	1.292665273	0.370348747	-2.30369525	-0.363024281	0.050180537	0.278912229	0.777506325	-0.363073684	0.834412534	-0.261167265	0.06229691	0.0049829	0.95206788	0.98530941	0.8831447	-0.1792783	1.05738912	0.08050639	-9.870581
unknown	219.0261	2.632547287	0.389088503	0.033787315	0.24205311	1.151678307	0.203737791	1.262980454	0.336832312	-2.860558002	-0.313310411	0.021133279	0.180616135	0.803966311	-0.314793046	0.861933774	-0.21435107	0.55748638	0.10475782	0.59456506	0.8189191	0.94589833	-0.080243	1.12596435	0.17116115	-7.5305542
unknown	116.0434	4.184287977	0.262446929	0.004115214	0.077112954	1.054262987	0.076234794	1.165913631	0.22146092	-8.489645152	-0.378328569	2.84E-05	0.012348446	0.764196938	-0.387983619	0.81927544	-0.287579528	0.62410419	0.11430317	0.55234727	0.79634238	0.9673589	-0.0478769	1.16210702	0.21674294	-35.52312
unknown	359.1195	3.821634189	0.261300989	0.006528371	0.098870669	1.056015973	0.078631656	1.170244725	0.226810262	-4.04944896	-0.341655023	0.003687031	0.075370308	0.784231582	-0.350648352	0.840007282	-0.251526261	-0.1874129	-0.0396854	0.85665351	0.94213594	0.87871708	-0.1865294	1.05548423	0.07790502	-15.475513
unknown	361.1251	5.818869733	0.29059165	0.000650889	0.029681429	1.072921445	0.101544451	1.187164806	0.247520229	-2.631219657	-0.304033626	0.030121091	0.212649365	0.811280231	-0.301727761	0.871072871	-0.199134681	0.43916295	0.08437508	0.67378034	0.86194984	0.94862209	-0.0760946	1.1394313	0.18831394	-15.310724
unknown	299.0984	3.293611552	0.253506856	0.01323656	0.149933511	1.049055426	0.069090903	1.160034762	0.214168038	-3.362793642	-0.246535415	0.009890711	0.120454542	0.837113389	-0.256505042	0.896402584	-0.157781287	-0.0087604	-0.0013126	0.99325477	0.99815293	0.88596047	-0.1746858	1.06070562	0.08502432	-11.075736
unknown	115.0401	2.403942372	0.162253792	0.047192033	0.281705789	0.982449819	-0.025544375	1.086865459	0.120173363	-6.291455259	-0.313385471	0.000234941	0.028906646	0.801826585	-0.318637843	0.858301306	-0.2204439	0.4004575	0.0705324	0.70075988	0.87793567	0.93634419	-0.0948892	1.1249833	0.16990358	-15.124296
unknown	359.1195	3.160828975	0.131453322	0.015909905	0.162360704	0.964871545	-0.051591208	1.065942826	0.092130058	-3.480279401	-0.200380226	0.008316218	0.108460111	0.864914095	-0.209371246	0.926975472	-0.10939693	-0.1698287	-0.02819	0.86994881	0.94715613	0.87526133	-0.1922143	1.04881117	0.06875496	-11.000568
unknown	179.0562	3.272304973	0.145333	0.013630919	0.150959461	0.975851044	-0.035267147	1.076585996	0.106463564	-2.26460001	-0.126555834	0.053338071	0.287452529	0.90982542	-0.136338352	0.97522553	-0.0361922	0.31976506	0.04671456	0.75847874	0.90869739	0.91733377	-0.1244813	1.09801243	0.13489439	-7.4104619

Supplemental table 4

ID	m/z	OLZ vs CON							
		statistic_OvsC	dm_OvsC	p.value_OvsC	adj_pval_OvsC	fold_change_imputed_OvsC	log2_fold_change_imputed_OvsC	fold_change_imputed_scaled_OvsC	log2_fold_change_imputed_scaled_OvsC
11d-TXB2 [M-H]	367.2122	-5.335067146	-0.8803872	0.0010815	0.03720363	0.475700298	-1.071875165	0.528203565	-0.920834057
15(S) HEPE [M-H]	317.2124	-5.216507731	-1.038081753	0.001230507	0.038328302	0.441117278	-1.180765826	0.486823068	-1.038530563
15(S) HEPE [M-H+Acetate]	377.2332	-6.325572287	-0.962707732	0.000394353	0.025604563	0.457935038	-1.126785139	0.506469837	-0.981451743
15R-PGD2 [M-H]	351.218	-6.223823192	-1.4156692	0.000435056	0.026405285	0.321548752	-1.636890603	0.358468085	-1.480083417
17(18) EpETE [M-H]	317.2124	-3.641146032	-1.575115524	0.008274505	0.112753035	0.275824207	-1.858179016	0.31092087	-1.685380636
20cooh AA [M-H]	333.2075	-3.479309858	-0.924878494	0.010275834	0.1291112	0.450055437	-1.151825373	0.504036047	-0.98840118
8-iso-PGA1 [M-H]	335.2232	-8.126951417	-2.403329614	8.24E-05	0.017507673	0.161597602	-2.629522307	0.181300852	-2.463542387
8-iso-PGA1 [M-H+Acetate]	395.2441	-9.683481292	-2.375632867	2.64E-05	0.016446553	0.16347599	-2.612849334	0.182596019	-2.453272784
8-iso-PGA2 [M-H+Acetate]	393.2288	-8.563839231	-2.079593988	5.89E-05	0.016446553	0.198501762	-2.332776284	0.222422877	-2.168622915
8(9)-EpETE [M-H]	317.2124	-4.249009024	-1.529960053	0.003797778	0.073706276	0.290120013	-1.785278274	0.326021287	-1.616961928
ASPARAGINE	133.0605	-2.977728398	-0.582348143	0.020581009	0.184860554	0.577697195	-0.791614606	0.633500653	-0.658581989
CITRULLINE	176.1026	4.661421136	0.429668822	0.002310647	0.054200457	1.194316521	0.256185234	1.318489724	0.398886327
Docosadienoic Acid [M-H]	335.2958	2.609069045	0.679911269	0.034960662	0.245666463	1.318881333	0.399314763	1.463346657	0.549271575
Palmitoleic Acid [M-H+Acetate]	313.2386	3.572389634	0.933298617	0.009067905	0.119217362	1.673192926	0.742603804	1.843632497	0.882551103
PGA3 [M-H]	331.1919	-6.208265981	-1.30577215	0.000441685	0.026405285	0.35730134	-1.484786769	0.398289501	-1.328110643
PGA3 [M-H+Acetate]	391.2135	-6.402555096	-1.153095019	0.00036639	0.024987604	0.399959106	-1.322075598	0.444468223	-1.169847818
SERINE	104.0354	-4.77928356	-0.748540613	0.002013948	0.049746038	0.515449756	-0.956096289	0.569274762	-0.812802953
SERINE(M-H2O+H)	88.0398	-2.464654888	-0.386963421	0.043169789	0.2686166	0.675979325	-0.564948972	0.739769966	-0.434851365
Tricosenoic Acid [M-H+Acetate]	411.3483	5.225814203	0.872408464	0.00121802	0.038328302	1.604698802	0.682302532	1.752592271	0.809490402

ID	MOvsO							
	statistic_MOvsO	dm_MOvsO	p.value_MOvsO	adj_pval_MOvsO	fold_change_imputed_MOvsO	log2_fold_change_imputed_MOvsO	fold_change_imputed_scaled_MOvsO	log2_fold_change_imputed_scaled_MOvsO
11d-TXB2 [M-H]	3.64276686	0.62395912	0.00656264	0.09658786	1.53596721	0.61914742	1.65198267	0.72419855
15(S) HEPE [M-H]	3.68435184	0.81122724	0.00618028	0.09461138	1.71881026	0.7814103	1.84565211	0.88413065
15(S) HEPE [M-H+Acetate]	3.78912142	0.71774139	0.00531849	0.08785991	1.63192413	0.70657398	1.75075869	0.80798025
15R-PGD2 [M-H]	5.05671887	1.0432591	0.00098104	0.04774127	2.07068482	1.05010798	2.21927377	1.15008765
17(18) EpETE [M-H]	2.92534178	1.04452952	0.01913411	0.17270636	2.06726045	1.04772016	2.2254246	1.15408062
20cooh AA [M-H]	2.49272226	0.62159351	0.03736338	0.23944854	1.55080849	0.63302054	1.67051506	0.74029299
8-iso-PGA1 [M-H]	4.19178385	1.45237158	0.00303036	0.07176953	2.81913266	1.49525137	3.0591004	1.61310746
8-iso-PGA1 [M-H+Acetate]	3.30189815	1.36323015	0.01082817	0.12714389	2.84475863	1.50830625	3.10959562	1.63672698
8-iso-PGA2 [M-H+Acetate]	2.74426602	1.24775295	0.02528197	0.1981535	2.79309756	1.48186597	3.04239575	1.60520783
8(9)-EpETE [M-H]	2.24900291	0.74029043	0.05465227	0.29087339	1.70282941	0.76793392	1.83302596	0.87422722
ASPARAGINE	3.79354822	0.72526204	0.00528503	0.08785991	1.68621896	0.75379189	1.80775077	0.85419579
CITRULLINE	-2.7705654	-0.3905228	0.02427549	0.194609	0.76075424	-0.3944976	0.81608004	-0.2932174
Docosadienoic Acid [M-H]	-3.7899973	-0.4806696	0.00531185	0.08785991	0.72194382	-0.4700415	0.77323821	-0.3710152
Palmitoleic Acid [M-H+Acetate]	-2.6884624	-0.5242182	0.02756221	0.20470748	0.67565863	-0.5656336	0.72893768	-0.4561326
PGA3 [M-H]	5.21725675	0.9718403	0.00080527	0.04269255	1.90497028	0.92976849	2.0439526	1.03136174
PGA3 [M-H+Acetate]	5.40014152	0.9226252	0.00064591	0.04061469	1.85139033	0.8886091	1.98013084	0.98559576
SERINE	3.42656404	0.68915419	0.00900034	0.11470906	1.66056085	0.73167059	1.76926724	0.82315198
SERINE(M-H2O+H)	2.49484415	0.51232169	0.03724003	0.23944854	1.44393084	0.53000164	1.55178985	0.6339332
Tricosenoic Acid [M-H+Acetate]	-3.5522878	-0.5063533	0.00748442	0.10231843	0.70256021	-0.5093062	0.7558865	-0.4037585

ID	MINO vs CON								
	statistic_MvsC	dm_MvsC	p.value_MvsC	adj_pval_MvsC	fold_change_imputed_MvsC	log2_fold_change_imputed_MvsC	fold_change_imputed_scaled_MvsC	log2_fold_change_imputed_scaled_MvsC	statOvsC*statMOvsO
11d-TXB2 [M-H]	-1.9074352	-0.345607	0.09812709	0.49809109	0.69162285	-0.5319426	0.83221515	-0.2649715	-19.434406
15(S) HEPE [M-H]	-1.0993003	-0.2165545	0.3080011	0.67534471	0.76849183	-0.3798982	0.91873231	-0.1222835	-19.21945
15(S) HEPE [M-H+Acetate]	-1.3359907	-0.2452157	0.22334924	0.61943033	0.74877151	-0.4174025	0.89692357	-0.156943	-23.968361
15R-PGD2 [M-H]	-0.7217383	-0.2776868	0.49385225	0.76821474	0.78809073	-0.3435664	0.95187198	-0.0711605	-31.472124
17(18) EpETE [M-H]	-0.1733323	-0.0976164	0.86729594	0.94560399	0.89632812	-0.1579011	1.09248638	0.12761529	-10.651597
20cooh AA [M-H]	-0.2553106	-0.0817701	0.80582231	0.92643993	0.83564286	-0.2590416	1.01541168	0.02206477	-8.6729531
8-iso-PGA1 [M-H]	-0.7871122	-0.3664964	0.45703365	0.74863513	0.76106732	-0.393904	0.92188009	-0.117349	-34.066424
8-iso-PGA1 [M-H+Acetate]	-0.6058438	-0.3101203	0.56373872	0.80205641	0.83680832	-0.2570309	1.01282725	0.01838813	-31.973869
8-iso-PGA2 [M-H+Acetate]	-0.5269555	-0.3159753	0.61450562	0.83097556	0.919561	-0.1209828	1.11212398	0.15331763	-23.501453
8(9)-EpETE [M-H]	-1.0051991	-0.4992669	0.34827614	0.69479002	0.67605107	-0.5647959	0.82191235	-0.2829435	-9.5560337
ASPARAGINE	-0.2335305	-0.0554793	0.82203184	0.93199556	0.82828436	-0.2718019	0.98806725	-0.0173189	-11.296156
CITRULLINE	0.64384809	0.0433333	0.54018754	0.7903383	0.90462814	-0.1446032	1.08263311	0.11454442	-12.914772
Docosadienoic Acid [M-H]	1.74644704	0.51719605	0.12423529	0.53580578	1.18607149	0.24619097	1.42702655	0.51301218	-9.8883646
Palmitoleic Acid [M-H+Acetate]	2.9267301	0.59257942	0.022127	0.30919845	1.27695463	0.35270727	1.5331941	0.61654035	-9.6042354
PGA3 [M-H]	-1.4262389	-0.2700143	0.19683715	0.59576827	0.71611731	-0.4817322	0.86469659	-0.2097341	-32.390118
PGA3 [M-H+Acetate]	-1.0122475	-0.1982719	0.34512255	0.69368379	0.76433131	-0.38773	0.92121008	-0.1183979	-34.574704
SERINE	-1.3248318	-0.1862435	0.22683661	0.61978339	0.75183641	-0.4115093	0.90180216	-0.1491171	-16.376521
SERINE(M-H2O+H)	0.06492255	0.01008251	0.95005084	0.98447188	0.87426877	-0.1938512	1.04060531	0.05742297	-6.1489298
Tricosenoic Acid [M-H+Acetate]	1.03428496	0.20911261	0.33540662	0.69185437	1.00746168	0.01072497	1.19568091	0.25783243	-18.563596

Supplemental table 5

Method	Polarity	m/z	RT	ID	C1	C3	C5	C7	C8	O1	O3	O4	O7	O8	M3	M4	M5	M7	M8	MO3	MO4	MO6	MO7	MO8	
EIC	Negative	379.2473	4.84	11-HETE [M-H+Acetate]	72853	89685	80417	58936	95698	57633	51243	42503	48360	50825	38532	41536	37873	45162	59025	40361	49784	41398	60789	135614	
EIC	Negative	397.2557	3.81	11,12-diHETrE [M-H+Acetate]	87826	411447	134871	102391	113443	139862	112209	106698	106145	106663	93726	93154	100637	102015	495968	99416	98721	102398	182811	141787	
EIC	Negative	317.2122	4.03	11(S) HEPE [M-H]	21396	16309	30753	21450	33092	82535	84500	64699	77854	76165	103046	80679	80945	98580	82107	83779	71262	65129	67622	76779	
EIC	Negative	379.2481	4.92	12-HETE [M-H+Acetate]	118385	382284	388639	179299	209271	195140	102522	110878	114758	117177	140124	100735	75976	119649	246237	138589	90795	104555	342491	920310	
EIC	Negative	377.2315	5.03	12-oxoETE [M-H+Acetate]	69375	242317	190955	88332	113656	95613	84329	65922	71286	76289	87975	64878	65011	75628	312943	113262	62815	67195	178180	307998	
EIC	Negative	295.2280	5.16	12,13 EpOME [M-H]	2603518	6191585	4951318	2967782	4175196	4316515	3103040	3684227	3570385	3952259	3304364	2997257	2483350	3932456	8182517	3878824	2896632	3092386	5192399	7225997	
EIC	Negative	381.2653	5.71	15 oxoEDE [M-H+Acetate]	0	0	89096	88703	0	0	0	175106	181761	0	150435	115285	16063	25493	7050	61037	121397	0	0	0	
EIC	Negative	379.2485	4.71	15-HETE [M-H+Acetate]	99201	297872	267184	119937	175929	160137	74545	97914	91844	99681	107332	75990	42735	84824	194803	103764	60130	83767	243640	608536	
EIC	Negative	377.2314	4.60	17(18) EpETE [M-H+Acetate]	49020	132953	147362	92713	66846	88021	29908	54399	61501	58401	37389	61742	26379	56329	36440	57815	66721	32397	129602	245270	
EIC	Negative	379.2471	5.17	5-HETE [M-H+Acetate]	76847	127589	128808	65330	108629	96925	55390	55589	48414	58305	63069	59648	32658	61689	109829	51001	43247	38228	82736	184034	
EIC	Negative	377.2314	5.32	5-oxoETE [M-H+Acetate]	35938	254917	170259	78370	209627	79624	75098	38949	71917	70848	83737	74817	9869	77367	326606	100283	25517	68401	155501	293190	
EIC	Negative	379.2482	5.66	5,6-EET [M-H+Acetate]	120729	153311	130309	105172	158670	122258	88244	90569	95147	92300	99359	70010	77410	93083	97091	122724	74785	74722	123860	116741	
EIC	Negative	411.2377	2.01	8-iso-15-keto-PGF2alpha [M-H+Acetate]	119247	513401	278405	182389	239415	279482	142267	142833	112104	110079	118829	125222	79284	132431	167630	117978	192892	147973	31425	920478	
EIC	Negative	397.2558	4.02	8,9-diHETrE [M-H+Acetate]	127336	263984	171217	137106	134679	171700	124435	148426	133216	127176	110454	99145	113747	157117	360071	143808	119892	110575	174371	166417	
EIC	Negative	379.2485	5.03	9-HETE [M-H+Acetate]	191799	502652	493618	256066	321266	363924	219685	255770	270225	304412	283403	221897	180737	298111	367525	318761	182719	212285	536655	1049780	
EIC	Negative	293.2124	4.74	9-oxoODE [M-H]	134374	973111	1422291	366504	781399	903580	162491	284211	200663	323791	340940	226177	113081	207217	152174	447443	236281	258856	1491987	3865348	
EIC	Negative	313.2388	3.54	9,10 diHOME [M-H]	108006	207477	199270	137237	173606	184507	120240	122487	129563	135902	126770	121693	107714	123374	213001	126424	127148	117174	188138	252180	
EIC	Negative	295.2280	5.31	9,10 EpOME [M-H]	539870	2837217	2117611	890988	2161158	1164928	800754	819313	785063	885025	955633	712036	575489	933465	3788075	1248764	631400	792706	1876239	3727707	
EIC	Negative	277.2171	6.33	a-linolenic Acid [M-H]	296577	1790241	1306473	676105	3401971	966618	304126	1323422	1873055	2560654	1812899	363061	759510	1865368	703435	2696818	163546	673146	7193017	2195265	
EIC	Negative	331.2646	6.53	Adrenic Acid [M-H]	2604120	20312897	9236304	5760444	25212705	9106530	4526257	12304979	16768028	15179613	12893152	3510129	6165298	13240123	5412051	14232569	875968	2240357	26006113	18344300	
fpHILIC	Negative	160.0616	3.74	AMINOADIPATE	94215	1275856	2045923	760122	751398	701222	630135	1032869	730111	1311832	1490089	635070	1289992	1902809	868954	1735193	556732	897330	1875495	2418534	
fpHILIC	Positive	144.0655	3.75	AMINOADIPATE (M-H2O+H)	79794	675430	1268907	379015	427329	365803	258303	493992	435991	795591	803064	236118	564559	1118748	397687	1091785	228372	391466	1089135	1349593	
EIC	Negative	311.2956	6.72	ARACHIDATE [M-H]	1019050	5244927	3118107	2381879	4903874	3547641	2668055	2501242	3883590	3146425	3833669	1478859	1936524	3420172	2230422	4937915	3219292	1989558	3282992	3342389	
EIC	Negative	339.3266	6.85	Behenic Acid [M-H]	11645	78911	62174	25996	82152	47962	26181	44475	55249	59814	60980	0	25254	70256	0	97392	0	0	84280	59094	
EIC	Negative	266.0892	0.56	DEOXYGUANOSINE [M-H]	70035	307186	437087	222341	355184	412455	119577	138886	156709	408161	421728	251970	226095	442872	155148	533374	171293	709028	570393	522026	
EIC	Negative	326.1106	0.56	DEOXYGUANOSINE [M-H+Acetate]	477635	1959784	2767308	1442531	2471831	2834228	751925	949506	1045850	2706067	2652892	1624289	1432192	2849081	977248	3465637	1072985	4716424	3854387	3563788	
EIC	Negative	227.0646	0.51	DEOXYURIDINE [M-H]	104903	359664	516847	228858	694806	212375	109508	382064	633853	472271	825059	135338	452141	654290	249119	506464	175990	321064	410451	713180	
EIC	Negative	329.2337	2.79	EIC_124;EIC_127	175253	310787	197088	161930	240804	200864	150533	155313	155477	147740	141157	131659	156251	144558	192331	211265	239186	137908	200007	138884	
EIC	Negative	329.2336	2.31	EIC_125;EIC_126;EIC_129	207393	536960	227639	196762	389644	474346	163754	253922	240761	193660	176253	158690	175295	207769	289999	250502	322300	184865	296327	373987	
EIC	Negative	329.2335	2.45	EIC_125;EIC_128;EIC_129	107957	489605	150974	160753	296242	292622	84727	117278	135184	83090	101389	82757	90356	109296	205273	148386	200926	93565	179333	267611	
EIC	Negative	329.2338	2.55	EIC_128	47076	211638	93055	70578	118752	213615	45033	60861	63746	48107	57181	45824	44929	63130	106687	70397	78840	55285	101953	205110	
EIC	Negative	333.2075	2.32	EIC_138	63725	145973	101568	72616	412852	246238	38561	64233	100313	81813	738446	75711	88311	130499	97348	93078	52041	96176	652929	303141	
EIC	Negative	333.2076	2.44	EIC_138;EIC_139;EIC_140;EIC_145	60062	84622	63834	31176	212057	211566	25771	37810	51126	45094	711267	47017	52588	74966	65893	51722	35635	63271	323140	187422	
EIC	Negative	333.2075	2.39	EIC_138;EIC_140	72345	71194	77737	72308	183909	903896	82973	81598	94457	104473	253141	79944	63174	127974	89094	108566	73041	92709	133311	111253	
EIC	Negative	293.2124	4.53	EIC_16;EIC_18	151579	977471	1494891	399112	774125	1041036	164363	279435	202356	310242	351648	238543	131336	228784	142137	460460	232069	238409	1417452	3741128	
EIC	Negative	295.2280	4.43	EIC_20;EIC_21	539059	2265456	2887137	1178477	1973775	4412013	536993	1197378	1290491	1383503	877999	677831	562587	1200908	914811	950545	1247470	523864	2662350	4084344	unchanged
EIC	Negative	295.2280	4.60	EIC_22	273039	1340805	1600306	679811	799352	1070313	257719	571029	643399	656336	432074	367205	221153	574086	376035	511069	673469	290904	1362655	2628416	
EIC	Negative	295.2280	5.12	EIC_23	2898437	6222040	5105071	3347788	4460287	4703722	3699876	4160508	4134810	4507389	3558122	3421609	2873502	4387129	8466348	4292926	3359108	3523884	5273063	7078857	
EIC	Negative	295.2280	5.02	EIC_23;EIC_30	574799	2705465	2237574	934920	1265391	1144745	832674	811011	778963	851757	929663	690321	597107	892404	3687262	1160253	623268	758863	1968098	3781786	
EIC	Negative	353.2341	3.24	EIC_251;EIC_256;EIC_260;EIC_265	32475	73059	87537	44593	72327	62107	40476	60209	54927	70984	61335	46783	39431	57450	60403	63721	39936	58083	116204	150021	
EIC	Negative	359.2208	2.72	EIC_294;EIC_296	0	121100	21437	123904	94313	238764	102565	156213	143366	80211	241749	52346	118858	98055	124168	152736	79369	165548	163173	139415	
EIC	Negative	297.2436	4.70	EIC_31	781265	3243036	2953832	1401792	1852553	1727388	919853	986997	1139566	1109853	1258871	939854	685476	1041403	2389783	1408168	841677	960507	2881372	7501399	
EIC	Negative	309.2056	3.68	EIC_51;EIC_58	128617	345821	207095	126336	245163	393563	112988	96860	110092	116768	111136	186183	107869	114469	268986	375863	114564	94626	146491	214987	
EIC	Negative	309.2055	3.61	EIC_51;EIC_59	154938	339031	208893	116516	291428	373848	130140	98417	114241	117142	121665	188192	111952	124157	296252	396747	117571	99742	149109	217122	
EIC	Negative	309.2049	3.86	EIC_58	53743	0	151850	74793	116290	52593	50442	55315	0	48983	87109	42370	42764	52526	95450	89911	0	66751	0	594749	
EIC	Negative	309.2071	3.86	EIC_58	53743	170555	155976	77761	122925	117612	52208	62530	60791	56605	97225	61512	52968	57531	99959	99539	56007	71256	174932	676814	
EIC	Negative	309.2056	3.79	EIC_58	93312	239293	210856	129952	172717	201452	105784	119552	127112	111589	142076	117064	90134	112727	245870	128055	116810	115781	284789	642673	
EIC	Negative	309.2056	3.24	EIC_60	53806	85701	98973	60967	85977	117830	82148	72651	32344	78953	70013	77253	65681	75574	196724	74924	72749	70191	85171	103125	unchanged
EIC	Negative	311.2230	2.93	EIC_62	104330	232416	292677	168000	208862	245966	116606	135125	123809	132475	110096	128913	117265	124536	164376	144233	139002	120226	156734	227143	
EIC	Negative	311.2228	3.52	EIC_65	161259	609916	706361	310390	369223	347322	170338	257417	201451	245644	161423	226122	141938	184359	206949	286000	236123	151007	268669	327813	
EIC	Negative	313.2380	5.24	EIC_72	41078	88582	90636	54695	102073	85192	55592	64914	67768	66924	62028	46658	41574	67094	76791	77898	46036	59825	92127	72384	
EIC	Negative	313.2388	3.28	EIC_73;EIC_74	125805	338989	193616	177665	197088	236563	124867	157533	166152	164128	136071	133528	116363	133356	202463	154306	195263	129192	203902	263711	
EIC	Negative	313.2388	3.15	EIC_76	117806	353830	224998	180249	221980	254811	133673	169789	166238	143586	150127	143776	114654	148665	201228	170875	202803	143582	220506	363584	
EIC	Negative	317.2123	3.78	EIC_83;EIC_93;EIC_94	34388	73675	65088	32295	348000	1370688	0	39062	53109	61806	495218	53400	54792	106288	57965	67347	38098	70352	132371	337547	
EIC	Negative	317.2123	3.95	EIC_85;EIC_89;EIC_90	72050	74742	84994	76115	85405	363636	448724	339474	455687	443624	495991	488053	439034	524125	411248	460416	376463	298045	318952	346898	
EIC	Negative	307.2645	6.57	Eicosadienoic Acid [M-H]	265077	2741968	1320763	774100	4078764	1274141	640338	1336955	1904225	2157496	1703645	333868	670803	1675563	560501	2109343	123468	745271	4195849	1924673	
EIC	Negative	367.2859	6.55	Eicosadienoic Acid [M-H+Acetate]	113384	432406	3448388	2430861	2228303	3306547	200789	169891	415481	461300	338629	194338	182066	376545	283491	774867	56861	444107	4118165	1540372	
EIC	Negative	301.2172	6.25	Eicosapentaenoic Acid [M-H]	748097	9737092	8685815	2520300	10530658	6337198	1475544	3740027	6654135	13575136	7007556	1207955	2168161	10145120	2840189	9432030	689406	5070521	12386430	10339401	
EIC	Negative	305.2483	6.54	Eicosatrienoic Acid [M-H]	611361	3777925	3170836	1948071	677251	2697422	1203586	3111382	347151	404910	305704	87074	1812020	4456171	127479	3512982	226589	706149	4462819	397183	
EIC	Negative	301.2171	6.36	EPA [M-H]	219747	482430	614759	328710	1390619	7027203	375602	520661	719622	913643	1676594	646014	546295	1292629	595184	1296992	258982	554921	1579262	1364458	
EIC	Negative	565.5227	6.63	FAHFA_565.519585	12912	317821	127270	92673	389514	104118	68866	125137	175516	197890	187340	19022	84241	191215	66458	173664	0	47387	220557	263020	
EIC	Negative	583.4750	6.47	FAHFA_583.472635	0	188705	250840	0	811365	199001	0	171993	225272	483011	354410	30224	91207	348899	103375	543835	0	94078	774203	383673	
EIC	Negative	593.5525	6.63	FAHFA_593.550885	19266	1228821	847479	302590	1462031	715597	206330	582087	1145466	750502	1514525	65925	167277	1243507	189847	900492	113700	298173	1175012	1698493	
EIC	Negative	607.4733	6.44	FAHFA_607.472635	2213198	13678380	15245515	12042817	26300472	5942751	3016260	8760803	17500012	24003506	21134329	3695162	10146937	26434762	8759174	29635842	1819606	6005813	29357270	38070717	
EIC	Negative	607.4734	6.45	FAHFA_607.472635	2213198	23196654	432035	69543	990226	10299537	6194484	178820	430925	45278504	40845236	6920899	18790216	692532	76813	46598104	1819606	11442851	42580112	67951712	unchanged
EIC	Negative	277.2171	6.30	GAMMA-LINOLENATE [M-H]	660643	1611325	1507548	791231	2575670	1287018	355295	864195	2265440	2191646	1597548	322480	623317	1575782	1395862	4336227	189704	680814	6357167	2262858	
EIC	Negative	369.3013	6.64	Gondolic Acid [M-H+Acetate]	0	1377368	723438	318891	1407271	1327964	250441	850971	60520	996804	54248	138450	223754	1185034	418916	1532206	848826	566886	1647316	75389	
EIC	Negative	269.2488	6.58	HEPTADECANOATE [M-H]	1756744	5468892	4851870	3185299	8731993	4682255	2695877	3593059	6027151	5994532	5776009	3106790	2291722	5985163	2889097	7126445	3156859	3578908	4669736	5850976	
fpHILIC	Negative	212.0026	0.99	Indoxylsulfuric Acid	187664	601909	0	719248	384843	787122	1606524	903415	1017401	469172	212171	89271	81841	213892	264389	322461	0	92259	239015	182983	
fpHILIC	Negative	130.0874	2.30	LEUCINE;NORLEUCINE	1085454	4577192	6408742	3166792	6411518	3578889	2577788	4180216	4235853	6676126	6695029	1975795	4495412	5358025	2601249	6841477	1703614	1672769	10637821	8331556	
EIC	Negative	279.2330	6.47	LINOLEATE [M-H]	7429606	20023740	16416981	9941809	31278073	14858839	7324490	14691701	24332769	23678571	17578820	5483646	10595268	18627898	12367644	27431070	1886658	11620200	46227772	21568801	unchanged
EIC	Negative	279.2330	6.48	Linoleic Acid [M-H]	10635232	25965138	22324520	13344667	49426024	22973960	855712	1567661	2598861	2156559	28309236	7184188	1093107	1643630	1247033	2408350	2511363	1144540	71150640	2170164	
fpHILIC	Negative	148.0439	2.42	METHIONINE	463096	2845219	3325911	1575251	2585900	1593089	1265302	1601478	2927352	2988594	3924835	1018111	2039100	2748777	2075701	2976903	757014	1507397	6375894	6117072	
EIC	Negative	356.1036	0.69	METHYLTHIOADENOSINE [M-H+Acetate]	14184	102984	140288	63413	122534	61326	29433	59884	104327	89018	127229	32804	73331	118884	65099	135832	32005	57986	130891	121621	
EIC	Negative	227.2011	6.35	MYRISTATE [M-H]	3332864	15125716	17458018	8359615	24652979	11560018	5858580	11249977	16402274	17013674	17530169	6365731	7739544	18490734	11409110	26515575	3953228	13224064	22939020	22272051	
EIC	Negative	308.0992	0.59	N-ACETYLNEURAMINATE [M-H]	16793	106181	136901	63498	288389	66324	27877	86531	118284	131127	181015	36932	111916	193337	75927	157462	46384	35804	129280	222522	
EIC	Negative	269.2125	4.75	Novel EIC_1	571513	811252	754933	607701	1091206	646755	527955	571086	633948	606260	589828	497970	455026	577651	887617	719971	513295	529650	733850	737657	
EIC	Negative	269.2125	4.59	Novel EIC_1	576073	812259	760473	618708	1005739	693754	550474	618936	666186	630704	608562	535543	479832	620165	944139	722625	546392	566519	742829	753461	
EIC	Negative	269.2125	4.97	Novel EIC_2	499435	675283	659024	547427	1611779	557754	445871	509789	560191	494756	500324	417967	401447	491805	765667	617926	449322	454201	613327	631400	
EIC	Negative	309.1710	2.61	Novel EIC_6	249595	233745	234916	244321	228793	149924	170140	166628	170197	184366	157698	160958	145426	156113	165050	131651	153432	187518	184871	188749	
fpHILIC	Positive	204.1227	2.32	O-ACETYLCARNITINE	20716736	104811192	205935760	75717240	65881616	40788456	62361644	71169576	101212576	157348736	314936416	58473808	81837136	93426760	21570818	233607888	45419900	121236400	340565248	362772864	
EIC	Negative	355.2850	6.40	OLEOYL-GLYCEROL [M-H]	70868	125205	211346	133848	284874	188339	108023	131065	218413	179611	284862	170249	155917	341075	470179	212601	51649	241240	526758	372074	unchanged
EIC	Negative	329.2486	6.54	osbond acid, all-cis-4,7,10,13,16-DPA [M-H]	77295	289687	200718	62343	356048	154383	79792	203765	253772	210034	200692	48587	85745	227864	95900	238076	0	121336	602819	266891	
EIC	Negative	253.2170	6.42	PALMITOLEATE [M-H]	3329037	18994016	13798641	7298004	24655164	8564533	3330143	10228298	16069192	18514918	14481007	4175670	6859356	17584157	8148617	19528438	1543651	5487867	19756047	22244582	
EIC	Negative	358.2963	6.24	Palmitoyl Ethanolamide [M-H+Acetate]	182497	1576296	3238085	1336265	2115435	1934391	520289	783841	2717478	2012666	1685641	181740	389763	2879649	835965	2529184	553494	955038	2034470	2508226	
EIC	Negative	341.2697	6.60	Petroselenic Acid [M-H+Acetate]	206885	2052261	1107707	905651	1415518	1045349	476882	824016	1183999	1000342	1575451	579704	389475	1409914	790991	2318942	1230997	687281	824726	1635986	
fpHILIC	Positive	130.0862	2.71	PIPECOLATE	214814	901820	1086241	191742	697886	469449	434341	588923	594301	818952	686224	345049	649639	1149139	542546	1155095	265590	519469	1230080	1393620	
EIC	Negative	343.2850	6.63	STEARATE [M-H+Acetate]	253663	1083562	837259	512023	1122436	615108	426176	609134	832152	729883	945806	286138	357374	769533	441136	836164	576528	368284	784277	985468	
EIC	Negative	514.2848	1.52	Taurocholic Acid [M-H]	1014154	553052	5229181	12198966	12530882	698367	443193	455032	24826120	2698379	25802840	4264364	5290125	5022710	9958603	17618088	2465801	3280532	138493521	20261340	
EIC	Negative	498.2904	1.99	Tauroursodeoxycholic acid [M-H]	128242	74341	499557	1202046	1163251	59946	51675	72173	2130392	226765	1599405	419840	393884	418647	838135	1403095	233987	298636	9000360	1685572	
fpHILIC	Negative	118.0510	3.15	THREONINE	1624310	4984548	5756734	3769400	2901026	2874579	4313665	3899778	3063987	3587226	2533048	4149586	4134805	5550118	2984889	4306237	3555905	3298785	3795158	5795451	
fpHILIC	Negative	180.0665	2.91	TYROSINE	486186	2178854	3855568	1279801	3576687	1355542	970968	1578492	3867523	2543966	4836583	858786	1432478	2185845	1202568	2066261	675183	1549916	6859230	3795226	
EIC	Negative	453.2893	2.09		8093	9666	14521	13397	13981	108659	111866	128867	123523	113437	113940	115826	101069	104891	113246	85781	103714	96166	84351	117375	
EIC	Negative	244.2000	3.81		84977	84556	100724	93470	130183	184663	173306	202003	199215	232501	166361	179628	158536	207078	168767	158419	160016	164929	192858	175349	
EIC	Negative	315.1273	2.49		28887	20661	16821	22685	23425	106291	87739	99675	91334	95182	79585	80169	71277	84927	91280	70694	79856	75326	71432	83703	
EIC	Negative	477.3336	2.09		46687	34725	49181	43397	39274	315316	344568	402701	341096	325969	327147	357222	305092	293695	327110	246914	310877	276529	304684	298490	
EIC	Negative	478.3374	2.09		8230	13614	18529	14125	13918	95804	106310	128353	111366	96709	96138	115144	96723	84486	97008	77476	87642	90445	96094	84894	
EIC	Negative	257.1544	3.92		72554	72226	67664	72966	85624	140154	151824	160754	159126	166825	137342	170831	137929	192432	138284	167277	126026	135969	127162	141166	
EIC	Negative	381.2654	3.22		132098	156724	146005	140619	156254	282126	320997	304382	350948	323359	339397	297891	341201	311758	305699	286421	213115	277123	266651	285198	
EIC	Negative	335.1872	1.92		30124	24175	21138	23669	37827	105543	128725	114154	113530	113752	116567	124532	120000	113983	114966	105346	126320	102850	86722	90425	
EIC	Negative	318.2156	3.95		19589	18904	20944	16430	25230	90358	101880	79137	100936	98816	109993	109791	100102	117600	93006	95273	85059	70984	69317	80417	
EIC	Negative	339.1636	3.04		169717	161164	174759	205367	233761	348175	381798	450005	427587	446794	404458	401415	178633	203378	289363	251733	289503	414152	415399	496302	
EIC	Negative	379.2493	3.29		203684	190109	185528	195561	212725	616553	796008	767609	801280	708201	772535	776305	786055	749632	679313	704095	619514	591164	619996	592950	
EIC	Negative	325.1479	2.93		102645	102981	115338	143081	154261	252517	293175	342415	304249	322452	284125	297309	100405	127468	138097	180439	203457	308706	281005	364254	
EIC	Negative	339.1636	3.23		163641	147790	159607	202974	218039	339338	365757	434377	395000	414745	359466	382377	163753	183026	273710	238694	291824	405307	382183	475265	
EIC	Negative	297.1163	3.28		179387	133266	142467	173510	195804	304018	301073	369152	344153	354036	318852	366050	157022	195143	202183	250227	238729	359312	325516	394896	
EIC	Negative	258.2157	4.44		31205	46213	39388	36666	60499	87197	79561	84819	96223	120416	87735	92136	68073	98873	78184	86954	77207	75859	86196	76101	
EIC	Negative	327.1637	2.77		161456	126251	159016	182206	198502	287297	312921	395722	371448	354210	330089	340596	141964	176999	198123	194235	253562	368852	323621	421452	
EIC	Negative	327.1637	2.56		140679	120040	119033	160354	161543	244406	265125	310116	282167	299078	285375	299345	110878	158231	158944	174627	218285	285403	277061	341915	
EIC	Negative	327.1637	3.42		343925	272984	312235	354545	409729	568045	611662	781178	707628	738699	637135	668729	318959	390953	396063	439942	489280	704445	706607	896972	
EIC	Negative	327.1637	3.24		152632	121209	133321	148801	177429	242474	263864	333553	303682	321079	284455	302986	142671	173220	168129	187573	212721	302860	280569	370618	

EIC	Negative	311.1326	2.47	48045	30685	43796	54266	63247	87618	118345	119564	115642	120835	106325	122951	40296	52899	58395	60943	87655	117489	115278	150396
EIC	Negative	263.0485	1.62	60569	37284	42086	32160	53157	91906	86768	97023	109677	107490	105514	126341	58919	117699	93502	81663	93005	100008	77899	80180
EIC	Negative	313.1482	2.88	146294	110140	124675	149490	175220	228217	274044	343012	296295	319194	293050	305261	117962	163540	177228	182640	204516	308727	289628	390825
fpHILIC	Negative	249.0325	0.41	220353	65066	96538	110946	140623	372570	333236	350236	306356	259337	419558	772906	369743	536178	225202	156703	493276	353017	344869	223204
EIC	Negative	227.1288	3.01	34961	27909	91231	87521	91984	170673	128349	151135	140514	156308	159794	174200	119254	174857	104149	142924	161201	122729	104851	117123
EIC	Negative	316.2504	3.92	53091	0	0	0	46510	109273	74847	103213	116511	83144	146330	109146	46328	89831	90331	71969	134257	65553	87671	71654
EIC	Negative	377.1959	5.28	20560	19577	40345	74191	44936	120062	74905	103142	100088	92724	96526	60166	62482	73437	36175	82844	68697	88074	90911	121192
EIC	Negative	401.2308	4.76	25341	108371	45274	117849	107313	188420	189785	86214	214389	163147	102058	7663	91288	92617	94906	89036	30551	37963	366334	148897
fpHILIC	Positive	222.0967	0.48	620159	508587	294196	470612	743834	1106909	709422	1078496	1506236	1049309	1779136	421207	1262293	923069	1202264	631125	662760	998705	717219	894884
EIC	Negative	319.2220	4.76	141346	0	383648	980970	1147411	1824353	1941465	1003107	2099225	1405162	1077761	232179	870087	947798	963590	979635	329374	312417	3810322	1485241
EIC	Negative	320.2314	4.76	32819	244098	83824	214915	272959	404592	420108	225286	516864	317878	235937	49962	201098	208590	217968	211570	72702	68929	831936	326150
EIC	Negative	645.3891	6.86	0	0	20592	9968	46409	40839	18607	25263	35136	60782	139644	27642	19221	86838	39684	36926	26331	23947	44786	29995
EIC	Negative	315.1967	3.94	56324	60145	26640	60742	94872	226023	106571	113720	120877	126810	158464	114831	94818	149855	112556	121512	96736	88126	134282	111534
fpHILIC	Positive	372.2927	0.35	0	371221	91483	77050	657149	788384	396219	655205	1435923	1346939	1220361	131154	367080	421353	435629	329255	187463	405095	502641	333517
fpHILIC	Negative	201.0210	3.26	0	0	0	0	33271	136715	0	83467	233425	244963	582414	0	131844	60496	142026	207388	0	40481	113364	0
EIC	Negative	600.9239	0.57	9294	50280	0	0	0	111241	150255	15080	11795	78045	6761	46140	40503	7458	16607	0	31016	13934	20616	0
fpHILIC	Positive	222.0522	0.61	583448	440558	623146	210774	428500	1427669	682351	1962098	856481	583452	663009	839518	360069	902873	875867	2042023	620422	1923508	379293	372123
fpHILIC	Negative	367.0521	3.26	0	117487	0	0	233028	676696	0	614829	1109501	1425201	1703267	0	836414	426860	745686	1151605	85410	250991	940307	91415
fpHILIC	Negative	352.0600	3.26	0	0	0	0	40427	301702	0	245931	520092	750350	1138951	0	350519	106382	394527	503122	33321	54759	145394	0
EIC	Negative	390.2940	6.44	4362550	1050652	10184536	8787104	988724	10657954	8686089	9941463	11325339	10628843	1077104	6100254	3965236	9978643	8744872	989545	14780992	10860135	8445397	11253012
fpHILIC	Negative	175.0246	3.26	0	7313121	0	238134	11332800	27980168	32963	24736824	37510884	53045596	76634848	577960	29690132	14476465	32868296	39701168	7835449	12922162	20714124	2727503
fpHILIC	Negative	176.0280	3.26	0	515680	0	0	757321	1882957	0	1827977	2569279	3743959	5410390	39283	2032375	1027006	2221324	2826580	578163	847496	1449219	220338
EIC	Negative	338.9362	0.56	110764	206645	68558	68246	58045	272764	478470	70123	132196	222531	55585	149766	69487	70392	117516	60474	97660	66539	97346	66053
fpHILIC	Negative	351.0564	3.26	0	102173	0	0	249496	2255368	0	1739656	3526763	5588879	7506835	0	2621129	628120	2765970	3412200	182861	320452	986145	0
fpHILIC	Positive	370.2770	0.35	0	221708	64802	36524	422321	492157	220648	310965	784479	697891	669378	54217	208678	297645	189290	231430	100405	251918	286430	250666
fpHILIC	Positive	198.0522	0.61	2627271	2210009	3340003	1814947	2205600	7258529	3083406	10542650	4009894	3659157	2261458	3850043	1366544	4940458	5291131	9484924	2893897	10085441	1652198	1738211
EIC	Negative	322.2139	3.52	12981	14924	28865	16371	38297	103265	42297	37423	83370	55653	63035	76778	24428	47140	36118	34861	47244	59710	45390	57398
fpHILIC	Positive	181.0258	0.60	1846913	1577970	2662797	1309551	1383756	5405598	1805450	7166319	2560588	2481370	1695757	2436794	975088	3425691	3976920	7196268	2069210	6803195	1443555	1267392
fpHILIC	Negative	149.0009	0.60	2025389	1295459	2133642	2067746	1470594	7761843	1731741	6728805	3292921	2958584	2703289	2540342	1307681	3833503	4021758	6683137	2625472	8430167	1267380	1324024
fpHILIC	Negative	385.0630	3.26	0	52629	0	0	74953	155742	0	141881	319690	372754	612166	0	185927	149992	148955	332114	30317	75507	318825	34579
EIC	Negative	415.2439	3.43	0	0	255455	0	9042	261690	240367	272181	0	292640	0	215899	27149	0	302893	309918	51378	278581	289017	0
fpHILIC	Negative	432.1275	3.29	0	0	0	0	32819	88429	0	105047	180900	363735	710612	0	123172	44220	117281	269008	0	0	159958	0
fpHILIC	Negative	358.0468	3.26	0	0	0	0	37544	399636	0	219902	787040	1149086	2323056	0	350016	115442	329860	797638	0	69907	512126	0
fpHILIC	Positive	442.1268	3.28	0	0	0	0	93913	230625	0	194667	509069	888992	2267146	0	318445	193360	201835	699940	0	40212	424117	0
fpHILIC	Positive	220.1003	0.48	365065	9595766	3951334	6871979	15567547	25230546	5717024	22949704	30640268	21337226	34727584	4542128	21550938	16194722	21900450	12678521	4619931	18168268	11758351	15247264
EIC	Negative	341.2024	6.54	178382	0	278972	0	783152	0	779070	1449001	1929584	1359641	1763746	0	0	0	0	768378	0	0	0	0
EIC	Negative	349.2755	2.93	0	13274	0	0	0	0	111327	127931	0	128491	0	41809	22987	71326	0	0	94730	0	0	0
fpHILIC	Negative	218.0857	0.47	0	3138522	1542887	2116115	4716567	7023810	1118049	7463762	11300926	7777540	15129379	779069	6696026	5655294	5947234	4882978	967197	5239938	6025747	6733108
fpHILIC	Negative	359.0427	3.25	0	77662	0	0	195577	1657489	0	856011	3784959	5235560	10044542	0	1482826	487650	1547100	3480195	42565	268831	2127076	54430
fpHILIC	Negative	310.0777	3.13	0	474022	133090	56905	306271	807949	33510	968197	987607	1355947	897273	63709	1072956	710105	946863	1281025	260670	380180	472205	389144
fpHILIC	Positive	221.1038	0.47	0	995858	372752	763531	1539036	2289426	599941	2133053	3016552	1839156	3053495	404819	2080530	1762871	2064437	1318421	516193	1779365	1136736	1498504
fpHILIC	Negative	219.0887	0.47	0	347675	184226	210499	434722	625999	116401	659016	1167022	755515	1520175	102133	619589	522627	591085	473803	125514	455463	588489	737617
fpHILIC	Negative	244.1373	0.39	0	654881	419034	633133	1379005	1883704	452733	2095972	3072385	1363731	4978273	345400	1823916	1137925	1529943	862501	503958	1311117	1723235	1403334
fpHILIC	Negative	299.0174	2.98	0	0	0	0	201544	86702	0	45265	249185	267301	710675	0	35320	126515	0	163885	0	0	277008	126250
EIC	Negative	346.2601	3.52	262718	196569	232615	193401	276577	823715	247514	271876	638053	346833	422230	384819	161557	297693	187351	227546	255175	394799	294229	363326
fpHILIC	Positive	274.1836	0.36	0	106682	0	94461	320745	230079	149127	370681	580657	241720	675237	91195	298663	213887	278418	170182	128574	173920	175183	112421
fpHILIC	Positive	246.1525	0.39	0	537448	172190	537066	1343092	1596840	503232	1663477	2986191	1052630	3368330	336073	1578646	840585	1460492	828037	549621	1002949	1294387	900131
fpHILIC	Negative	172.0437	0.53	0	653281	88276	242917	927957	1128435	259113	981306	1880155	1148206	2126181	99953	930001	944735	742535	825941	117790	656789	939655	745669
fpHILIC	Negative	199.0542	0.53	0	197324	0	65112	340612	374291	84927	320853	690815	357519	740830	0	363667	280946	218591	310965	0	175889	285195	180913
fpHILIC	Positive	260.1679	0.38	0	292181	93041	326478	959698	736116	403393	1029952	1852840	626260	2205369	312532	993665	667842	903419	469018	362475	557520	626304	359557
fpHILIC	Positive	288.1992	0.36	0	272246	37903	140711	687956	338179	302744	634968	1020626	519859	1015311	157320	472366	349685	472284	316992	245140	220123	289883	194423
fpHILIC	Positive	146.0268	1.03	0	358972	0	75567	391738	431308	47569	318846	854295	735458	1118956	0	285638	349211	241766	563089	90486	173192	480937	334015
EIC	Negative	241.0507	1.43	0	19852	0	19001	81103	140173	0	188092	0	160211	0	178709	67854	0	0	100092	4429	4264	0	190697
EIC	Negative	526.2848	5.06	0	155231	158039	87604	0	150603	64707	176121	291413	333380	289560	0	0	213436	99421	190874	0	68478	0	0
EIC	Negative	356.9060	0.54	0	295369	0	76364	82803	302200	164027	75939	201840	338042	101384	0	88354	109215	157641	101875	0	70620	144937	131794
fpHILIC	Positive	344.2619	0.35	0	440555	144876	108222	1582632	853314	250772	760875	1303683	1379333	1312555	107258	648300	751909	397924	482912	128609	254573	636101	535076
fpHILIC	Negative	258.1533	0.39	0	442871	139900	389562	1084870	847297	378823	1171115	2417609	686711	2898210	227800	1093349	834541	1064539	675577	367626	656098	779318	480099
fpHILIC	Negative	270.1170	0.42	0	315800	0	242227	672995	260432	211268	631655	916454	560979	1158110	61115	497546	396049	386177	356817	166461	280275	318331	323020
fpHILIC	Positive	302.2148	0.36	0	277084	41467	130204	646064	270773	196742	545253	882440	504768	756736	120911	309928	313392	371364	261090	143369	189472	259973	148285
fpHILIC	Positive	176.0372	0.98	0	562471	0	83733	717583	249483	97187	350541	917856	2234902	541612	0	210882	1021458	91741	1305788	0	47286	448452	267335
EIC	Negative	347.2591	3.53	5115	0	0	0	0	0	0	0	136009	79423	87196	0	0	0	3668	0	0	0	0	0
fpHILIC	Positive	301.2925	0.57	231955	185349	143532	252375	209076	212956	463707	359014	145439	1012045	136188	248787	344842	247486	292042	104198	246905	113564	112677	279415
fpHILIC	Negative	128.0177	1.18	0	131030	0	189451	217854	170030	89527	307119	570032	103037	305425	47547	293059	308928	141224	59654	32580	203037	362905	445445
EIC	Negative	354.9099	0.55	0	421343	98707	101103	113152	455721	273397	101556	218756	527597	131047	69645	113037	140201	198321	54286	60331	89684	219343	195054
fpHILIC	Negative	158.0280	1.18	0	234134	40548	297679	399027	317009	147733	604881	970931	181950	597432	80011	508660	550564	249794	134337	73857	381666	698560	872944
fpHILIC	Positive	356.2615	0.35	0	225267	33325	51148	461509	332934	81066	261532	488652	383931	657737	49458	162489	255210	207381	372560	90176	248146	230351	139011
EIC	Negative	300.2565	5.69	479476	42930	0	0	62108	429922	0	407621	422662	66196	347151	0	0	0	43730	0	0	36676	206870	282427
EIC	Negative	401.2250	4.77	0	97788	0	97310	97429	176535	0	86437	188703	141879	48492	14624	28281	84455	0	0	5405	8561	34577	136641
fpHILIC	Negative	688.1727	3.54	0	138151	156381	67234	194617	1960820	0	47511	84385	1196257	139076	0	137039	295015	192968	150219	0	42298	395813	476483
fpHILIC	Positive	315.0796	3.78	0	341747	1344731	680609	251829	34671	170480	1092020	2581708	4955216	0	255747	215296	359391	0	2394239	178690	296389	229263	325293
fpHILIC	Negative	245.0075	0.59	95864	147045	0	553123	0	776650	319715	421630	289582	0	453510	227013	425780	83929	457199	33056	225045	724172	218458	376647
fpHILIC	Positive	293.0978	3.78	0	88401	480791	185968	84305	0	64186	306816	813081	1842333	0	114138	63852	143376	0	897455	56144	58374	106199	175860
fpHILIC	Negative	128.0176	0.60	0	2237806	583311	4508372	11571137	9347873	1661900	7578276	19484534	3889938	8167485	701886	7637602	7033160	2639409	3570489	1516905	5080965	5799842	11395566
fpHILIC	Positive	210.0794	0.93	381513	0	0	413754	629227	9937199	422540	1986619	65664	0	64553	97565	117282	27416566	104168	0	5883635	205415	736821	700102
EIC	Negative	410.2904	4.98	0	338319	0	40259	0	0	251633	318316	0	348467	300576	0	254737	9779	278297	0	29798	267352	0	311859
EIC	Negative	272.9082	0.55	0	247510	59882	45850	91362	254597	103440	53866	109343	392820	79735	23950	50731	84045	103276	68864	15581	49626	111016	101280
fpHILIC	Positive	360.1493	3.53	241685	81207	95341	87230	264029	5805657	128046	52983	68760	707465	0	0	188748	104397	425347	67795	47154	54769	124281	161980
fpHILIC	Negative	208.0647	0.93	285434	0	0	304175	311805	6364376	224852	936107	0	0	54548	69208	51090	16181872	45765	0	3715522	101959	460449	467063
EIC	Negative	335.2230	2.19	95480	75871	92980	82884	188022	900777	115806	118588	130522	126388	209232	138145	99310	162248	106789	145741	110630	104203	134361	103675
EIC	Negative	397.3079	4.78	0	0	0	0	52926	126285	0	0	117086	0	0	0	0	0	0	0	36840	165855	0	0
EIC	Negative	322.9959	1.20	0	0	0	102436	0	127195	62619	0	0	162586	0	0	0	159936	156526	0	0	162012	0	0
fpHILIC	Positive	333.1678	2.87	1180699	429048	600431	76284	268586	5915440	724475	463333	233613	183648	42865	82481	97465	4265672	43044	148809	732594	104596	292590	336656
fpHILIC	Negative	487.1784	3.51	137912	270299	295885	193102	651462	8774249	204803	147360	152164	1299970	152446	46056	408751	376956	725850	202240	79562	112448	525726	450715
EIC	Negative	348.8377	0.55	4682	74841	0	0	0	82273	12375	0	15430	257735	9812	0	0	16413	5089	0	0	0	15230	11370
fpHILIC	Positive	365.1048	3.51	615917	435807	271533	288851	488440	7733450	467312	198601	0	1139295	0	62345	395293	312432	1203452	135410	211639	162692	274694	276584
fpHILIC	Negative	494.1890	3.51	590124	204987	135442	205505	379158	8575222	378327	146963	35129	690599	40434	90530	325863	217830	926821	109946	184394	113503	152032	152297
fpHILIC	Negative	341.1092	3.52	793123	515470	619874	393448	1122364	21341424	474255	255429	237844	2508803	211649	100123	649448	664331	1502962	331213	245117	276702	933462	1073582
fpHILIC	Positive	167.1105	2.60	155866	0	85259	0	71489	3390581	55434	36028	0	119712	0	0	0	5923724	0	64331	823908	0	79368	188206
fpHILIC	Positive	123.0844	2.87	250047	81710	164242	35247	33303	1646048	129762	117752	47870	35731	0	0	0	1073463	0	0	234972	0	41875	64093
fpHILIC	Positive	185.0551	3.09	0	0	36844	0	0	2512301	0	0	0	56894	0	0	0	4276548	0	41670	477943	0	121908	61191
fpHILIC	Positive	166.1071	2.60	2094816	419819	1313879	325078	1054388	52163552	820049	574451	226690	1856836	127904	188831	169496	90621000	162436	1054574	12723133	235897	1102989	2936042
EIC	Negative	291.1606	1.65	87775	80466	91394	98097	133303	775954	92680	105904	103435	106492	380543	110706	85075	164675	130554	168041	87565	96175	147157	110996
fpHILIC	Positive	165.0947	2.22	215976	171272	133450	34491	137941	5035184	272298	93281	51821	82992	38274	55255	47354	6348454	71046	194878	628246	78054	93275	101116
fpHILIC	Negative	162.0771	2.22	200722	110523	209868	134261	137301	5432234	254413	121389	60849	90479	45069	55356	49737	4261914	80974	140149	930520	38710	54518	79059
EIC	Negative	297.1864	5.49	17295	28280	52118	31645	73419	367493	35184	42832	50792	51779	66099	38878	32736	78730	38926	59164	31000	35469	56204	55260
EIC	Negative	332.8662	0.55	0	103495	0	0	0	122272	0	0	18477	267726	0	0	0	0	9851	14145	0	0	5699	17244
EIC	Negative	372.8797	0.54	0	242769	54169	0	84567	236351	47221	45384	110727	346710	94282	0	57757	122676	106937	77079	0	43319	136682	127418
EIC	Negative	417.2290	3.02	0	56534	52276	55014	149389	680787	70021	70193	70250	83081	96424	59621	79655	91078	66389	62649	71718	94071	32986	55007
EIC	Negative	302.2206	6.36	51478	97936	146126	65231	294766	1588884	88105	100117	157345	200496	366367	143392	133309	273624	116485	294956	68137	122461	346599	259847
fpHILIC	Negative	110.9758	0.97	4862222	3387500	0	2475914	5857972	1946617	2247471	2038868	1521199	78505728	1727507	3607454	1704671	4087532	4782350	1846416	2839570	3072650	14160928	3049488
EIC	Negative	331.0824	0.89	0	0	21569	0	134938	2695175	36750	0	0	0	62252	6735	0	0	0	0	0	0	5014	0
EIC	Negative	302.0750	1.40	0	3948	12063	0	80523	1147679	30657	4700	4360	5503	69833	0	0	0	0	6284	0	4307	4523	0
EIC	Negative	301.0721	1.40	23651	40629	62926	35774	449678	6470443	166900	33885	32032	48442	359329	28102	22080	22666	20924	53380	29483	33780	22729	45270
fpHILIC	Positive	164.1281	4.45	532253	459301	448114	346433	517471	599884	337503	337475	278322	4840731	382659	309896	300802	461362	518228	382918	383877	283927	746586	385150
EIC	Negative	275.1657	2.14	76075	65637	100996	90624	215528	1769347	88975	95547	93314	101897	179967	103084	59604	131631	86045	125338	69458	99048	118876	94661
fpHILIC	Positive	211.1370	1.73	295727	113080	246140	1098714	194595	17768824	191741	160981	52442	242872	47211	0	56312	16607734	0	281646	3838273	57357	84817	377246
EIC	Negative	361.1660	0.99	0	0	0	0	73937	1084456	4519	0	0	0	79299	0	0	0	0	0	0	0	0	0
EIC	Negative	417.2295	3.57	0	0	0	0	144485	1135666	50005	27732	0	0	0	0	11444	39013	18981	27582	0	46728	64460	42940
EIC	Negative	511.1973	1.39	11889	11597	20737	0	87365	1284253	16816	5906	0	10408	78113	10646	4925	4502	5391	11152	4499	4725	4748	0
EIC	Negative	315.0872	1.45	0	0	13156	0	79705	1070140	20460	0	0	0	95813	0	10024	0	0	0	0	0	0	0
EIC	Negative	370.8851	0.55	0	318534	61794	40032	102198	340299	61858	50001	124407	471759	114984	10719	82195	124031	127504	93301	11045	62896	178861	171788
fpHILIC	Positive	310.1672	1.73	0	0	30823	113576	0	1362499	0	0	0	39047	0	0	0	1913787	0	0	449211	0	0	44394

EIC	Negative	320.2262	4.76	0	0	77470	0	255893	0	0	0	466107	301644	0	0	0	0	0	0	68592	0	0	0
fpHILIC	Negative	208.1187	1.72	160664	55634	81814	708447	87797	7133564	80709	72000	0	92224	0	0	36063	5567896	0	96660	1697350	0	0	136837
EIC	Negative	417.2301	3.42	0	75373	141567	108173	484450	3803867	119208	69345	95966	101019	100423	64021	61674	122294	58077	92187	45066	170437	196045	161764
fpHILIC	Negative	404.1042	3.54	90460	216977	263182	158531	512682	4893753	62799	44232	30897	447962	63980	0	136804	220760	312287	129678	70535	130527	357476	459572
EIC	Negative	247.9723	0.64	0	40539	0	0	0	23600	0	0	22542	67947	43422	0	0	50370	0	26402	0	0	19837	63013
EIC	Negative	511.1744	3.46	30152	23865	74845	21857	21665	36996	19510	19275	25529	366764	9813	103746	16403	27130	152482	19742	20894	185378	17294	46912
EIC	Negative	511.1741	3.28	103587	74664	232676	67912	78181	118892	58369	75839	63869	1070893	53773	345192	37358	74764	529847	60825	51323	683912	53556	159687
EIC	Negative	397.2002	3.62	0	29353	31339	19676	58261	191982	18930	23786	26441	30742	70349	17004	16517	45366	23446	32909	0	26645	59619	38136
EIC	Negative	512.1776	3.15	44216	26520	108210	18958	27051	38952	23308	29803	19919	387072	24102	148316	22228	19715	233236	21733	25560	254894	19379	75634
EIC	Negative	511.1740	3.15	158189	99056	393295	75584	88450	137836	60573	83528	75582	1526681	79229	578793	89505	84780	856359	73487	78882	930269	70227	269500
EIC	Negative	457.3660	6.71	0	41304	0	0	9152	0	0	0	188240	0	134375	0	9858	155763	0	0	0	0	22140	41184
EIC	Negative	307.1906	3.13	35952	83475	105659	43077	97606	701646	27983	56311	17769	29885	67152	40756	32671	38576	34422	74514	42838	59811	104750	425512
EIC	Negative	511.1740	2.95	37158	26793	90175	29311	7468	28970	18855	20351	27549	304280	23222	104477	16219	29198	167772	24762	26035	181794	26931	62613
EIC	Negative	461.1822	4.13	0	988782	0	0	0	1130964	0	0	0	930224	0	0	1977710	0	0	0	0	0	957919	0
EIC	Negative	307.1945	3.03	8824	76868	0	0	175579	635975	0	0	0	0	0	18045	0	0	15707	0	0	0	0	0
EIC	Negative	307.1945	3.14	0	72053	0	0	174660	598855	0	0	0	0	15887	0	0	0	0	0	0	0	0	0
fpHILIC	Negative	245.9359	0.40	2139373	300621	110222	2118296	127493	94459	9465712	597522	0	0	0	4366766	291240	104281	343756	97094	1320323	983413	0	504888
fpHILIC	Negative	165.9966	0.58	0	947704	8940959	730067	1557482	0	0	0	0	0	0	348850	0	610904	0	229936	0	0	80818	83500
EIC	Negative	309.1744	2.72	1755553	1730938	1633216	1914706	0	0	0	0	0	0	0	0	0	0	0	0	0	0	344645	0
EIC	Negative	283.8419	2.43	0	59407	505061	60263	168371	0	0	0	0	0	0	5114	3993	44485	0	0	0	4822	30670	83407
fpHILIC	Positive	87.1003	4.46	150398	0	608618	0	0	0	0	0	0	0	0	0	0	0	0	248232	0	0	0	0
EIC	Negative	342.2655	6.60	0	0	0	194699	309361	0	0	0	0	0	0	0	87767	0	176834	0	0	161835	0	0
EIC	Negative	370.2061	2.53	0	24295	157052	54751	66513	0	0	0	0	0	19464	17954	0	0	0	49325	0	24895	128958	312095
EIC	Negative	321.2386	5.05	0	0	179497	122595	0	0	0	0	0	0	0	0	0	273305	0	115616	0	0	0	0
EIC	Negative	321.2386	4.85	0	0	119795	122609	0	0	0	0	0	0	0	0	0	179491	0	114662	0	0	0	0
EIC	Negative	370.2059	2.67	0	8288	134585	44482	51233	0	0	0	0	0	12701	24829	0	0	0	42790	0	7901	119826	282612
EIC	Negative	365.2074	2.01	0	86425	53920	34013	57379	0	0	0	0	0	0	0	0	0	11659	0	0	0	37480	181329
EIC	Negative	403.3066	3.99	0	0	0	0	159705	0	0	0	0	0	0	0	0	0	0	0	144037	0	155181	0
EIC	Negative	349.2791	5.99	0	0	0	62488	0	0	0	0	0	0	135524	127237	62433	39020	0	50919	0	0	49507	0
EIC	Negative	628.3534	4.93	0	0	0	31685	0	0	0	0	0	0	61675	0	0	0	0	0	0	0	186311	93265
EIC	Negative	356.2269	4.87	0	0	8167	0	0	0	0	0	0	0	231911	0	0	0	0	0	0	188676	1318568	1747113
fpHILIC	Negative	150.0018	0.52	76530	1011929	12446676	1241035	2354853	0	105238	0	0	0	0	1262004	0	1043950	0	34898	0	0	93344	37692
EIC	Negative	232.0047	1.54	0	80145	521600	137594	220802	0	10185	2651	0	0	0	135818	11254	71240	0	5139	0	5198	0	26336
EIC	Negative	323.9641	1.54	12114	120292	428722	152624	185016	4104	15787	3904	0	4200	3527	136153	14201	63685	5985	7622	3881	10057	17881	34768
EIC	Negative	331.0087	2.44	0	95872	172162	94254	129757	0	0	0	0	16411	0	16986	11223	76995	0	14020	0	0	15970	10690
EIC	Negative	309.0264	2.44	0	144942	313797	136465	234426	0	3853	3219	5812	24940	0	23273	18763	131324	0	35253	0	2778	20672	12642
fpHILIC	Negative	343.9953	3.59	44996	3664486	32114998	2780369	3587524	88331	0	432084	610571	921113	60404	492392	303352	3728501	0	1802911	42169	1080498	1835438	3097086
EIC	Negative	250.0081	2.44	0	99499	186577	91696	147223	0	0	4177	4639	16956	0	13609	10052	92698	0	3123	0	0	15520	7958
EIC	Negative	249.0051	2.44	20364	594898	1300428	609174	954676	0	24069	23731	36819	98235	0	101406	74591	552694	0	119096	4859	14975	79534	65885
fpHILIC	Negative	344.9972	3.59	0	531928	4369998	361416	496989	0	87983	52205	88769	115687	0	77270	36691	482202	0	243363	0	140225	240369	455927
fpHILIC	Negative	112.0042	0.96	0	231466	574110	106602	195459	0	73039	0	0	0	0	61306	0	153166	0	53962	0	38170	114031	118095
fpHILIC	Negative	274.0569	2.26	0	175737	1728963	187261	239358	0	80178	44341	0	34761	0	158006	72661	225950	0	76700	73857	116144	111012	203609
fpHILIC	Positive	346.0085	3.59	38390	451837	5601130	457239	499513	0	296521	37808	48480	102179	0	238293	37839	405979	0	187949	0	191023	224208	253809
fpHILIC	Negative	192.0337	0.53	1639636	0	326070	162624	2055021	0	61013	103967	127660	0	269781	1037711	104756	270273	314682	2537689	240695	363152	0	533805
EIC	Negative	359.0148	0.45	3807911	16326	19711	77064	103128	31090	236127	51637	16469	0	15040	320063	9534	9261	46586	11498	1218019	108764	5665	0
EIC	Negative	280.2363	6.48	2145597	5374782	359736	2703811	10223324	327891	168225	308683	523237	445097	5957762	1406798	224168	331953	237645	459406	512078	239877	14594722	437023
fpHILIC	Negative	144.0301	0.97	85453	1006980	4305427	676419	1107882	64979	353242	74266	107222	69133	46955	252249	96906	586082	40002	272136	92971	150350	378624	469908
EIC	Negative	401.2340	5.47	84901	96911	96623	69005	118789	23857	0	0	22385	0	0	0	39216	16740	15457	13013	31783	19882	50621	54102
EIC	Negative	355.1428	3.16	208118	135466	109166	151025	153647	47375	0	0	27888	0	0	0	0	0	32658	32064	0	76631	62883	26838
EIC	Negative	279.1636	2.70	90784	143493	52736	267093	26704	0	0	0	64925	0	0	51989	0	0	0	325251	89454	74898	412795	177869
EIC	Negative	333.2213	4.92	0	55416	56060	26632	27089	0	0	5589	0	13369	0	0	0	0	30691	0	0	0	0	141088
EIC	Negative	359.1027	1.68	184533	133885	94254	149270	136830	0	37694	11444	33829	0	0	26984	30727	0	34266	17486	53528	75359	79739	50113
EIC	Negative	441.0173	0.45	1723786	33582	24085	66012	46686	47717	103170	41227	35116	0	20396	246503	33690	13855	46323	22292	544680	63518	29206	21305
fpHILIC	Negative	152.0025	0.93	0	0	0	596963	5767088	0	136084	250414	384271	0	0	4253413	0	1039390	0	47040	0	1213480	1218612	1878520
fpHILIC	Negative	235.0457	0.60	51116	1610516	16563011	2343729	1138981	233906	704144	658032	436305	637020	140785	396283	378112	1715466	90898	685676	255701	989173	2032199	3538738
EIC	Negative	416.1663	3.15	101378	75264	65927	73160	74258	23903	6851	0	18235	0	0	0	19035	0	0	12422	24741	31990	33104	22079
EIC	Negative	338.1344	3.16	153209	104671	89617	108082	107073	33229	23235	7963	15174	0	12040	0	19007	5475	14815	18019	36170	44016	46223	27610
EIC	Negative	523.0216	0.46	1429692	21235	14530	55903	33058	33007	125761	33514	12850	16691	12581	169887	22144	12489	34274	11211	394597	37565	17475	8732
fpHILIC	Negative	230.0560	1.01	0	379616	0	79508	672798	0	0	0	0	164458	0	0	0	428965	0	421903	0	0	154658	36118
EIC	Negative	365.0325	0.55	1036628	0	0	0	0	0	137897	0	15602	0	0	185415	0	0	30858	0	158799	70882	11442	0
fpHILIC	Negative	259.0649	1.71	0	406719	723478	60708	397584	0	30484	38302	34321	132444	0	37976	0	394074	0	363976	0	0	175046	117411
fpHILIC	Negative	258.0613	1.70	0	3656690	5905584	494847	3363494	58881	232362	315112	230566	1241176	50963	247257	172462	3302884	65089	2873230	136411	0	1620207	846106
EIC	Negative	337.1334	3.16	494282	359938	256573	379960	383568	105236	74440	37801	75639	0	38367	66622	80797	44495	86856	82624	116332	157142	156842	105971
EIC	Negative	399.2177	5.98	78874	118473	68022	103324	93735	15616	26487	7449	12224	11044	6394	0	20925	8608	8690	38193	9135	27999	24984	41890
EIC	Negative	415.1637	3.16	294613	207083	153747	219551	229140	63121	45833	24191	42741	0	17590	34798	46275	17086	40642	40717	75132	23749	94072	55538
EIC	Negative	281.0707	2.19	260373	118887	87583	195350	137637	47512	47597	20157	14150	0	18151	30663	22626	11262	35086	32406	109593	80247	77866	38433
EIC	Negative	282.0714	1.68	147204	100582	73307	119923	96750	33716	24304	0	28519	5436	8451	23837	29883	0	25919	20894	36347	41245	59542	41446
EIC	Negative	559.3029	5.52	0	0	150258	24308	193640	0	12518	0	19376	33029	0	0	125978	22728	26810	13644	0	0	24861	177181
fpHILIC	Negative	237.0995	0.38	620700	317488	190637	118520	1088729	421230	0	0	0	0	182737	863955	92107	0	137635	1408874	122905	314319	450969	385191
EIC	Negative	281.0705	1.68	614822	425625	272572	496939	430458	140569	104857	49063	104778	34299	58251	80416	100846	50655	112044	92324	163658	203768	217626	123886
EIC	Negative	359.2221	5.74	143586	199114	128149	171383	200743	45658	39059	15579	33586	34703	23126	20261	33785	37579	29189	50860	25641	56221	57124	63455
EIC	Negative	285.0830	0.87	820146	550020	407112	712351	668123	214641	137605	99897	198415	0	0	99752	180165	0	153918	180075	256234	289256	336356	270282
EIC	Negative	332.1406	2.27	93899	136414	114113	107283	99390	24375	30029	18746	18123	23697	11220	20176	20223	54090	80441	177212	31734	55117	101035	40966
EIC	Negative	463.2919	1.75	67986	83070	333077	55942	97700	33782	27283	24207	17569	31814	36582	83782	17125	22204	56221	85827	25208	44544	113141	559010
EIC	Negative	649.1267	4.87	257944	497906	356624	356451	354064	87302	84619	50711	75024	90969	53317	48084	66725	153821	210532	529825	70460	172987	417058	146323
EIC	Negative	286.0845	0.87	157185	101474	81059	138920	168454	37748	31073	20030	34596	15163	12009	0	41480	22305	35079	27380	62256	66551	75933	46765
EIC	Negative	300.2561	3.00	262415	215498	178115	203543	226934	73216	59552	18386	59869	28938	21820	22619	81408	39350	79549	80879	68176	87877	94643	104273
fpHILIC	Negative	307.1573	0.35	369483	482739	56268	172986	224968	103916	61589	0	88873	39822	107950	277195	222803	72704	343647	365333	258318	0	2040165	147332
EIC	Negative	409.2352	1.81	105110	116774	36284	115589	110020	0	109252	0	0	0	0	0	0	105876	0	31311	106724	109100	247798	108403
fpHILIC	Positive	588.1074	3.09	0	129560	122943	650729	77803	0	87200	0	38643	99116	323146	123300	70009	101020	44386	172485	0	133066	213026	575811
fpHILIC	Negative	273.0531	0.97	1098579	564000	0	629905	555383	158546	205083	102512	141772	49647	149099	108997	207588	127618	159994	138958	238929	296846	233372	244560
EIC	Negative	225.0615	0.87	437660	287687	210484	385287	358827	135583	94294	46174	90648	30112	45121	53418	97619	55394	73222	85835	138270	155923	179324	112336
EIC	Negative	361.2003	5.73	84484	94819	67437	86784	81027	15924	25761	18960	21719	15612	10641	18160	29497	21898	22951	27853	24957	26856	34433	26909
EIC	Negative	365.0314	0.46	4305957	146843	76028	350048	269011	92399	662027	309730	108134	78525	88002	1009743	260324	52113	281003	64182	1445254	330270	105067	68232
EIC	Negative	529.0395	0.47	1759442	35915	51931	114558	63316	66742	256924	88713	55078	27566	35042	419700	80295	23152	81100	27453	496806	113319	38167	27739
EIC	Negative	277.1447	3.59	1466104	1408687	1032195	1526018	1546142	353857	393102	260611	347410	353600	267505	286873	385154	287663	325821	431099	294833	448797	500371	488521
EIC	Negative	621.0043	0.46	2457320	130842	44097	119555	89167	96627	313415	165700	88684	33740	85006	534835	146449	79699	55992	95027	1535899	118058	53039	37059
EIC	Negative	449.0366	0.47	1076844	36883	29332	77178	50235	45854	164968	56465	24765	27004	27572	239034	37087	19332	46751	0	343536	28540	33551	20025
EIC	Negative	374.9916	0.45	6815556	587857	391239	592426	625871	495810	772454	376558	363160	309291	370020	1162442	334223	387199	338279	492289	5205658	570684	251883	355825
EIC	Negative	362.2916	3.27	178466	177196	128883	193741	168323	79360	39045	27534	48908	27537	39965	27611	50201	32974	46780	51286	38539	82783	72011	66472
EIC	Negative	582.3778	5.41	401557	1085582	0	591279	1096645	839503	0	0	0	0	1372728	205824	0	1299834	0	0	232739	0	2767961	1981701
EIC	Negative	447.0347	0.47	6087946	157926	188003	414852	252707	282154	961213	339566	191399	104492	141442	1438801	251568	113097	278911	115278	1965749	378380	178651	120222
EIC	Negative	448.0368	0.47	1547857	44526	48998	123260	75740	72152	238698	102470	56982	26445	38831	357145	66759	36243	84289	43240	509593	116233	41378	29546
EIC	Negative	343.2600	2.60	28658	63184	88546	35911	108238	24390	16846	18603	0	27937	27443	56894	0	28698	103205	27177	0	18617	48705	20194
EIC	Negative	257.1542	4.11	75676	52872	33389	60588	51431	12498	22535	11029	12765	15322	11319	17873	19681	11708	17744	16822	16764	23658	26541	21094
fpHILIC	Positive	230.0657	1.00	0	406161	0	57728	659092	0	0	32507	42917	230613	0	51585	32275	465788	0	372616	0	57673	148099	185057
fpHILIC	Negative	228.0514	1.01	82614	25246178	0	4314606	43000012	851870	880658	2168330	3236586	13172682	558946	755557	1152385	30797732	372615	25111780	478015	975300	9288936	5260917
EIC	Negative	538.9981	0.46	3206101	248803	186398	319887	221501	226718	474403	194648	146832	145224	130255	718848	178005	135502	155962	163422	2197583	300454	121596	157624
EIC	Negative	614.5452	6.57	57759	7604880	4973136	4069528	563494	297555	2906189	802867	520228	419030	559670	1609590	1983912	475156	171623	854413	285894	3884448	308898	451776
EIC	Negative	283.0277	0.46	4724108	164813	170923	416692	295067	298456	694993	322333	196643	152224	143131	1082916	253409	135346	316621	122499	1635427	362391	206831	139118
EIC	Negative	314.2449	2.89	0	99553	101751	71035	73215	99956	0	0	0	0	58919	0	0	0	68484	59069	53697	0	97893	208551
fpHILIC	Positive	144.1018	2.10	232268	209294	1037456	254352	1188606	260316	195656	190401	104204	94838	438791	136542	135918	250875	218614	309974	166015	138140	220823	192380
EIC	Negative	294.1793	3.53	74050	73457	70621	77760	78592	23940	22716	17947	18414	25680	20881	18843	26516	23302	24099	19624	24933	32563	34539	27842
EIC	Negative	367.0314	0.46	720510	20227	0	65080	44576	38757	116685	48512	33798	9269	17020	163153	48473	0	51067	9110	247418	58789	28636	15713
fpHILIC	Negative	586.0963	3.10	0	111077	123932	775046	80289	47055	84565	31245	47814	107369	416766	124299	63559	115222	58989	182197	0	127127	240324	576169
EIC	Negative	375.9937	0.45	973259	90844	61791	107192	108669	85665	134090	69566	54746	47720	53069	169012	60987	65860	54634	71466	652034	103876	14554	55249
EIC	Negative	445.2982	6.04	29336	90213	125954	81334	92084	0	0	30266	48063	44566	51436	44779	30097	49802	138178	82809	62635	15103	74709	86618
EIC	Negative	597.3047	1.52	9758	0	77790	157729	26580	24834	9844	7861	0	37480	44069	34731	80812	71369	148895	0	57689	52521	1282521	64010
fpHILIC	Negative	229.0547	1.00	0	2528921	0	431686	4159773	98728	88864	212219	324136	1383368	61022	85497	147892	3064284	0	2552121	41913	117409	880372	517698
EIC	Negative	284.0305	0.46	1040846	40030	44623	84691	79113	76893	142334	89665	46045	27383	33267	214602	57352	33501	67127	31519	349793	97390	46788	27210
EIC	Negative	331.1509	5.29	0	182026	139038	97215	162686	0	19313	23448	65403	64537	50388	26296	62772	62099	31617	74758	20416	63175	118442	94430
EIC	Negative	285.0266	0.46	740937	27225	33681	73800	61846	60068	103531	66869	39067	17751	21167	166724	47093	16408	57079	21439	244068	62369	32901	9218

EIC	Negative	366.0332	0.46	1020705	29981	45203	86767	65317	57167	180970	76324	44416	23640	28558	231316	57914	26206	71235	22142	351044	72509	42792	30325	
EIC	Negative	293.1764	3.53	341469	365492	361122	370711	421395	140485	107642	98358	117257	113060	99627	104302	132670	141627	118014	106415	135667	152196	176322	148981	
fpHILIC	Negative	236.0960	0.38	6768137	4046786	2914703	1320254	8708157	3701038	305864	984807	1458147	969840	1782127	8584721	790574	1685831	2259657	12868488	1745943	2740349	3603307	3795114	
EIC	Negative	293.1423	3.59	142426	406620	71639	42029	44547	44186	31075	41787	51925	52442	51168	23254	26162	56664	38697	28209	51070	73687	44894	32752	
EIC	Negative	332.2680	6.54	1043274	346148	3068085	2187315	10121717	3180012	1456339	190628	221518	257502	210629	1384374	2446998	4297860	2096679	4360874	300840	759371	393754	264627	
EIC	Negative	582.3778	5.17	431299	1263529	0	0	1127824	0	0	898180	0	0	1792617	0	0	1409184	0	0	349637	0	1862199	0	
EIC	Negative	354.2663	4.13	109976	41296	46072	239473	22659	20213	20144	35393	36189	35899	22582	17416	19195	18822	43851	39607	54607	15853	18180	34527	
EIC	Negative	456.9955	0.46	3158431	329542	243764	359980	315427	278735	501824	237474	218744	184187	209572	721252	209508	226051	221641	269734	2566216	347381	140159	204829	
EIC	Negative	481.2967	2.62	1502357	429184	256996	142815	164116	169347	239776	159871	104601	143307	341684	221717	81324	202625	119187	197795	290020	274593	121464	84664	
EIC	Negative	540.0005	0.46	540802	50998	38381	73363	53865	50769	102107	43203	35901	20855	13475	130487	35813	33510	18826	22501	341083	65140	28277	23655	
EIC	Negative	245.0798	2.48	8930	185057	170057	59020	216909	41145	5176	30202	61680	76422	56156	0	35592	70544	39930	83586	0	57007	103262	95866	
EIC	Negative	349.2053	6.30	597112	540960	1021557	1112480	1410183	425147	139670	142955	316333	553129	2198211	562796	6806920	1147634	15554593	399784	265912	196087	593161	422000	
EIC	Negative	350.2083	6.30	120290	115258	196898	214045	273212	82229	31750	25178	66016	107760	424819	113097	1337424	224472	3061603	68747	50240	37863	112447	79831	
EIC	Negative	375.1792	2.98	369628	351967	323283	335792	355757	111043	128666	110161	121717	120939	99203	113815	150519	142691	121515	117841	122425	160752	169509	156590	
fpHILIC	Negative	244.0463	1.00	0	447543	0	76757	521061	0	0	49334	65564	242535	0	42215	0	455744	0	822118	32697	44306	847567	394078	
EIC	Negative	313.0724	2.48	63541	11695	26322	0	12909	26252	0	0	3604	9923	6223	3363	0	580936	3045	16135	8509	0	82867	329629	
fpHILIC	Positive	259.1282	2.73	0	593686	0	37609	1391433	39327	67202	155264	203708	249524	30511	80792	127987	1120004	0	311321	34190	112154	900980	348527	
EIC	Negative	293.1762	2.98	4425642	4860063	4662392	4511112	4934434	1787847	1655975	1413145	1698772	1737077	1434033	1439577	1775877	1855027	1685269	1482515	1661152	2189211	2231404	2269758	
EIC	Negative	358.2630	1.99	6461904	110173	6370551	6998364	104670	105877	105958	110596	6677284	109797	6261158	6121117	6492679	106454	6218146	102522	104024	6480877	4890546	6524903	
EIC	Negative	317.1398	3.76	766567	877728	529911	785092	746448	231885	334946	205748	266782	279050	203992	185953	272343	235234	244291	326641	189010	327433	382092	365026	
EIC	Negative	425.0648	1.68	179767	140948	135398	50575	337115	84362	57781	57709	75027	27057	50912	63319	44505	97528	63574	540079	52967	57514	60719	152550	
EIC	Negative	294.1792	2.98	839283	876555	858289	843485	921602	323518	315633	263763	320983	336515	268657	279555	338702	355899	332043	270075	322429	421122	419999	410884	
EIC	Negative	279.1637	2.83	33205	16904	36150	67853	0	0	0	28856	0	29136	0	0	0	0	0	135854	0	32208	154412	0	
EIC	Negative	318.1431	3.75	144376	172102	102749	148385	149204	53142	67886	44647	53241	55054	43908	40719	55621	47775	51267	60085	41555	62038	82389	78131	
EIC	Negative	354.2371	2.25	13181	116319	290398	118496	183839	47312	31383	64774	65893	69570	85234	28892	51383	92039	60632	0	22945	34921	189318	293844	
EIC	Negative	530.2800	1.95	0	0	18285	57938	29313	0	0	0	28994	11927	43761	22320	26939	24922	54447	58657	23150	14901	589848	99486	
EIC	Negative	457.9984	0.46	441475	51578	35004	65219	54258	50532	83714	42289	50946	23985	33721	109364	35198	38377	29365	42476	343565	67967	25429	38077	
EIC	Negative	251.1323	2.08	14589	86855	69522	0	89282	61548	40198	0	0	0	72229	0	0	76839	72844	0	54018	0	108753	106079	
EIC	Negative	249.1496	5.30	1411172	3578094	2417678	1937607	3344360	983568	808046	679311	1388786	1196807	920194	489383	974304	1086303	937161	1449974	384553	998903	1834143	1661494	
EIC	Negative	250.1528	5.30	220678	632852	415476	320329	563865	179372	135909	112309	237426	207942	157428	77548	161605	181189	155191	231074	64183	161055	326737	282556	
fpHILIC	Negative	137.0355	2.46	977452	440180	258611	245315	199935	101647	268760	206374	175946	111058	225599	295124	487849	151805	356051	272430	183260	170279	399362	234577	
fpHILIC	Positive	399.2504	0.41	4759942	10904219	3435685	2793995	11748206	3493370	3827315	1940588	2695096	1874697	11196831	3486720	1803751	4981869	3021510	1804810	15656010	2536488	1746812	2229853	
fpHILIC	Negative	199.0377	1.81	404307	1413543	1239968	6144711	2491083	971251	1366127	397579	953310	1120748	3076861	779036	1217496	1734626	719781	1778391	615792	2316944	1682207	3834290	
EIC	Negative	375.2155	4.74	12400	81983	128384	30821	68732	78520	0	13825	0	40491	15680	15109	0	10310	0	40034	9482	11728	111686	336766	
fpHILIC	Positive	400.2534	0.41	824700	2337831	876185	577882	2257986	670051	820508	413305	495348	479930	2255089	817910	380732	1152605	562064	424560	3213185	560051	332266	364480	
EIC	Negative	238.0912	1.68	299918	284011	220143	96224	565496	203003	95248	91792	136531	95298	133449	256551	100859	157258	152101	813515	104588	137648	225602	247894	
EIC	Negative	237.0990	1.68	705353	683481	519636	239408	1365834	484104	238224	215345	330108	226590	315560	632042	213904	380861	377974	1933286	275302	328334	547973	613382	
EIC	Negative	431.3015	3.03	176649	136652	142767	142633	128303	0	153934	0	155535	0	0	0	139298	142605	148795	105981	0	0	156639	136210	
EIC	Negative	236.0961	1.68	6359486	6332068	4836475	2173892	12119326	4341319	2226264	1990571	3044355	2004742	2846670	5781088	2058615	3463563	3477596	17758403	2471153	3059012	4956082	5649585	
EIC	Negative	357.1084	5.36	69992	79620	43456	17248	109560	0	37686	99709	0	0	79867	0	63141	10716	69382	18250	0	19343	85353	71606	
fpHILIC	Positive	453.3253	0.40	3762601	0	2226632	1273224	2060752	0	1952391	1044409	1075638	0	2140110	210386	2221187	2297865	2128115	1939929	783539	1352832	0	2203942	
fpHILIC	Positive	201.0519	1.81	103971	533535	433646	1807803	742534	316565	501520	146975	299885	329072	816742	284695	392279	595574	280094	617104	205782	756624	648034	1364233	
EIC	Negative	513.2727	1.70	60792	24269	247657	424928	610261	29529	7298	11007	489887	68826	214501	31443	66579	35344	140191	146200	27325	76007	1391889	237880	
EIC	Negative	255.0660	2.42	331906	44924	99718	56140	54461	98979	30556	36989	46972	46985	124688	25366	26896	1554493	46011	99541	41162	44700	118949	104034	
fpHILIC	Positive	140.0530	2.47	1091436	493661	192809	62621	196310	93982	327486	235194	158202	97989	131703	438557	455078	202831	300339	355124	91802	210197	472354	226104	
EIC	Negative	512.2692	1.70	178801	58799	836946	1392076	2046265	80165	42394	48829	1652694	222217	710345	100309	208581	175156	460672	472026	75005	269975	4700221	780016	
EIC	Negative	379.2480	5.96	127829	551741	411270	160667	345832	165550	137371	139691	138966	143247	138434	110912	107937	143899	566556	221972	108930	103760	210696	173104	
EIC	Negative	362.2915	3.47	218031	316163	209734	402667	290248	227000	99888	95479	137315	94046	138700	71220	117061	112992	132747	110063	92829	221005	167133	139562	
fpHILIC	Positive	176.0912	2.63	77148	517331	2023359	72465	550045	195726	178417	391155	289340	420115	102368	240646	266014	714589	139540	483304	154739	161342	486592	686513	
EIC	Negative	298.2469	5.96	219958	1267610	918494	345046	812844	374140	277603	310819	314307	346377	324304	250140	206939	370262	1337244	531569	210035	244441	460717	424019	
EIC	Negative	297.1345	0.91	960823	440597	936092	372335	379976	359645	287436	334598	315867	111092	311254	1348410	254741	318721	1089460	265439	396405	1699906	270600	532244	
EIC	Negative	459.2344	5.02	23712	54762	45310	28273	33158	26017	17758	17974	0	23393	17023	7072	4848	7186	73049	16891	12970	22027	37798	61531	
EIC	Negative	297.2436	5.96	1119673	6364680	4673910	1718675	4056776	1961727	1405240	1519545	1559271	1811249	1655696	1275544	1088939	1758339	6709557	2656309	1064005	1232617	2261881	2094143	
fpHILIC	Positive	129.1020	2.54	1760672	448355	470963	1585173	758617	526875	301393	730847	337630	417090	264117	1048225	7531163	1121895	263261	383771	1201377	472551	584897	1724281	
EIC	Negative	398.2898	3.36	28490	0	63955	0	50488	0	0	0	0	66083	0	0	39888	0	55910	0	35286	57435	74157	43315	
fpHILIC	Negative	323.1895	0.35	1502103	396596	428491	246081	1410177	284666	536265	214243	365067	444854	462154	309195	182962	305052	268450	642313	510501	244397	1124973	264492	
EIC	Negative	331.0413	2.58	166474	119691	135985	134309	144830	60657	75520	66323	77700	45484	70829	59554	105458	68699	78354	87559	63625	80254	72593	86258	
fpHILIC	Negative	202.0722	0.93	41117	34527	0	343248	35624	41059	0	121558	48756	0	0	0	0	80285	0	0	0	132977	124654	630092	
EIC	Negative	357.2074	6.35	282720	505140	323207	317642	466781	173072	179487	113780	206873	212137	159674	116367	187567	191393	170555	333345	102240	206429	243856	391584	
EIC	Negative	241.1075	0.90	31443	30216	22221	38446	216005	28746	28431	33645	32618	35050	38958	37031	30937	30019	52517	29610	30932	66168	28964	35002	
EIC	Negative	433.3342	6.55	559684	562027	921000	1007703	0	63527	35343	142566	697149	491779	907433	386984	263936	1546984	317631	407816	301489	385820	487045	522276	
fpHILIC	Negative	319.2225	0.41	418046	753910	751812	744450	436257	0	606020	270403	258720	323523	336629	0	645549	530894	435207	472492	300542	731600	83931	0	
fpHILIC	Negative	163.0247	2.53	0	221323	537074	253636	172419	170147	67843	30108	161341	127574	120095	104814	188039	364133	258470	161349	96217	247847	124858	0	
EIC	Negative	331.2388	2.01	18772	148162	86585	35407	92589	77286	14662	38379	42299	7789	14365	46325	0	34890	57775	39956	61364	53093	71389	331539	
fpHILIC	Positive	452.3215	0.40	14164791	0	8016941	5448517	7742874	0	8259502	4158653	4344562	0	8262117	954996	8309548	9827305	8180210	7132252	3237988	6456014	36402	8703116	
fpHILIC	Negative	395.2471	0.35	1742053	387340	451223	169813	1644828	97445	793417	265429	427571	511721	456683	272634	142303	514571	634079	678914	746502	482672	1551099	244427	
fpHILIC	Negative	280.1667	0.34	721913	204049	212028	82326	571948	73082	284047	67228	98594	335050	152534	181176	82838	176781	188654	248288	318311	162356	493125	102026	
EIC	Negative	368.2887	6.55	6851	76809	869552	602565	520504	780742	36126	0	28782	149738	37924	24917	29457	0	96113	159888	0	116826	970448	375438	
EIC	Negative	413.2237	2.57	200580	147699	146672	0	161224	0	137931	179673	0	0	0	178473	115527	155126	0	0	161735	160074	0	123515	
EIC	Negative	439.2618	2.66	45403	164710	43573	177371	12220	23213	0	0	60406	131177	222905	121412	91319	0	24644	37108	14694	78198	29664	105744	
EIC	Negative	407.2690	3.73	133314	54254	52264	64426	63294	55832	51359	13778	42095	15325	64149	30209	45526	69331	172345	93993	12689	29388	54021	221634	
EIC	Negative	363.2955	3.47	46375	69262	41677	83454	61840	44029	26273	21281	28537	28321	27585	10473	25686	25575	26819	21595	20435	48528	35984	29030	
EIC	Negative	403.2701	3.66	88205	76045	67202	65048	75401	42422	31270	40927	33995	34462	36387	42579	32141	26038	54591	38554	48062	36816	45017	38987	
EIC	Negative	383.2207	6.24	478910	1085352	738008	624756	989669	332874	143115	245606	439731	768055	381508	75630	216697	634090	207170	632100	72691	410290	756986	647229	
EIC	Negative	441.0594	1.80	54965	46144	45165	13351	121745	5946	52622	46770	33454	0	10881	6538	24182	68627	34170	99950	46303	12032	0	27489	
EIC	Negative	300.2564	6.30	0	9170	0	0	0	161149	0	150386	0	0	172358	0	0	0	0	0	0	0	153713	183389	unchanged
EIC	Negative	341.2680	3.32	0	13976	8365	0	0	16308	35838	72328	171635	58716	35251	67356	5240	33406	67561	156914	32113	172489	35818	39691	unchanged
EIC	Negative	417.3224	6.44	89113	0	0	23947	0	0	0	679362	840891	25431	172277	25135	0	676579	37975	993110	70950	336176	295858	331666	unchanged
EIC	Negative	461.1774	2.46	0	0	48263	0	0	0	0	574541	0	0	0	558190	0	542578	0	586836	0	0	0	563057	unchanged

Supplemental table 6

		OLZ vs CON								MO vs OLZ								MINO vs CON							
ID	m/z	statistic_OvsC	dm_OvsC	p.value_OvsC	adj_pval_OvsC	fold_change_imputed_OvsC	log2_fold_change_imputed_OvsC	fold_change_imputed_scaled_OvsC	log2_fold_change_imputed_scaled_OvsC	statistic_MOvsO	dm_MOvsO	p.value_MOvsO	adj_pval_MOvsO	fold_change_imputed_MOvsO	log2_fold_change_imputed_MOvsO	fold_change_imputed_scaled_MOvsO	log2_fold_change_imputed_scaled_MOvsO	statistic_MvsC	dm_MvsC	p.value_MvsC	adj_pval_MvsC	fold_change_imputed_MvsC	log2_fold_change_imputed_MvsC	fold_change_imputed_scaled_MvsC	log2_fold_change_imputed_scaled_MvsC
	359.0427	4.32407319	3.4186067	0.003462873	0.020546381	8.60769764	3.1056274	6.42994966	2.684807442	-2.046832584	-2.702325681	0.074875483	0.667479319	0.460691138	-1.11812825	0.291942187	-1.776245394	3.022870301	3.06428895	0.019307907	0.107400231	9.485682862	3.245751635	5.94276948	2.57113542
	351.0564	4.15752529	3.23318696	0.00425489	0.023667823	6.33830615	2.66409735	4.49400964	2.168003218	-2.975549607	-2.086330111	0.017720368	0.524885812	0.374468236	-1.417084748	0.259945438	-1.943719261	2.879737009	2.888062335	0.023660472	0.123869529	6.186811514	2.629196082	4.220426032	2.077388639
Indoxylsulfuri	212.0026	3.26995577	1.97532396	0.013675165	0.052916942	2.94820047	1.55983463	3.89616608	1.962055175	-5.311385382	-2.587271061	0.000718454	0.255769771	0.189866323	-2.396944058	0.105917601	-3.23898574	-0.906264443	-0.438989151	0.394919704	0.677497555	0.530990329	-0.91324251	0.573053136	-0.803259176
	258.0613	-3.2496269	-3.1722993	0.014064537	0.053838443	0.12029923	-3.0553007	0.13121486	-2.929996952	1.489102382	1.468502328	0.174786197	0.673879826	2.875012764	1.523568361	1.959027953	0.970137983	-2.559910887	-3.34623795	0.037556491	0.173637801	0.222216406	-2.169962763	0.214279164	-2.222436523
	259.0649	-5.0760408	-2.605211	0.001437369	0.011253588	0.17222413	-2.5376408	0.15870921	-2.655542244	2.749840878	1.422032086	0.025065087	0.524885812	2.360537812	1.239115593	2.22741327	1.155369258	-2.241391625	-1.303335671	0.059954666	0.229503884	0.388207138	-1.365101451	0.388868033	-1.362647453
	192.0337	-2.6195488	-2.0503575	0.034431776	0.110429839	0.22254011	-2.1678627	0.16704203	-2.581716945	2.354255747	1.667260998	0.046372169	0.56925835	3.406854717	1.768440426	2.817668896	1.494502091	-1.421740675	-1.201194039	0.198089131	0.454966004	0.346562546	-1.528812348	0.443665751	-1.172454908
	236.096	-3.2917461	-1.9635867	0.013270593	0.051915726	0.26454293	-1.9184262	0.22441794	-2.155740097	2.80595456	1.728177726	0.022985906	0.524885812	3.3361476	1.738183119	2.669877532	1.416773566	-1.162285141	-0.883500479	0.283209733	0.57565101	0.538481364	-0.893031678	0.746541964	-0.421704737
	280.2363	-2.9544761	-2.6818871	0.021271216	0.074975773	0.07835563	-3.6738192	0.08493334	-3.557525205	1.20675063	1.21683369	0.261997656	0.705179471	9.160681122	3.195454871	4.643177109	2.215112312	-1.138570784	-1.530812384	0.292343915	0.579626539	0.360520496	-1.471846816	0.327790902	-1.609152284
	352.06	3.176532	1.98224141	0.015565607	0.0569775	3.31568156	1.72930545	2.78242259	1.476341553	-2.476806151	-1.619396432	0.038301877	0.524885812	0.462297473	-1.113106619	0.323438124	-1.628438355	2.42200998	1.940030315	0.045956364	0.204505818	3.612050694	1.852818141	3.00953642	1.589541276
	463.2919	-3.7817764	-1.8835776	0.006876307	0.032210068	0.18514591	-2.4332654	0.22794386	-2.133249533	2.696787884	1.702613651	0.027209048	0.524885812	6.147042442	2.619892446	3.407800034	1.768840682	-1.875343091	-1.298634592	0.102867296	0.312997927	0.296874189	-1.752076428	0.451640374	-1.146753635
	332.1406	-7.3881078	-2.0150826	0.000150943	0.002704972	0.20723904	-2.2706323	0.23831087	-2.069083338	3.232415111	1.469264146	0.012013412	0.524885812	3.531912673	1.820449673	2.256892013	1.17433739	-2.681989929	-1.557398902	0.031448467	0.151292627	0.33554446	-1.575424158	0.387114292	-1.369168523
	367.0521	4.55516139	2.19820885	0.002619951	0.016081076	4.3853634	2.1326964	3.85317531	1.946047826	-1.566912453	-1.314089644	0.155769948	0.673879826	0.621332618	-0.686562301	0.438303204	-1.189998871	3.814500154	2.144380291	0.006589212	0.045110761	4.220711834	2.077486334	3.787868482	1.92138624
	144.0301	-2.2835409	-2.6678668	0.0563414	0.165764779	0.08224636	-3.6039044	0.15027646	-2.734309034	1.712384009	1.064634609	0.125182769	0.668242279	2.039329169	1.02809466	0.972887505	-0.0396551	-1.782816518	-2.343494063	0.117808849	0.335519602	0.125697453	-2.991972677	0.171924065	-2.540156595
	344.9972	-3.587709	-2.7653588	0.008884254	0.037652316	0.07100182	-3.8160002	0.08909986	-3.488433083	3.619943095	1.022942931	0.006783155	0.524885812	2.317783931	1.212746081	1.613896826	0.690548353	-2.253650719	-2.180492842	0.058880196	0.229041029	0.133587188	-2.904146441	0.142285933	-2.813135058
	649.1267	-8.7648373	-1.9634857	5.07E-05	0.002064111	0.21199587	-2.2378919	0.23751207	-2.07392727	2.953130197	1.369625148	0.01833773	0.524885812	3.439441621	1.782174368	2.179865107	1.124238862	-3.633778227	-1.634127859	0.008355813	0.053119095	0.290468573	-1.783546014	0.333353189	-1.584876565
	372.2927	3.18484211	1.83865174	0.015386631	0.0569775	2.65650556	1.40952973	2.58642415	1.370958882	-4.173504528	-1.396250972	0.003107142	0.524885812	0.380293423	-1.394815105	0.323376945	-1.628711267	1.352080993	0.90532035	0.218403369	0.477003677	1.480104489	0.565699028	1.324066772	0.404975879
	307.1573	-1.7652648	-1.2595287	0.120869493	0.284963838	0.28536362	-1.8091267	0.27886111	-1.842381326	2.534086559	1.935591419	0.035031818	0.524885812	7.19127718	2.846248018	3.975395747	1.991098486	0.069694536	0.059029912	0.946385997	0.962609757	0.722955063	-0.468022119	0.857739931	-0.221387809
	433.3342	-1.9652591	-1.8201997	0.090118628	0.234849422	0.40807441	-1.2930959	0.36672293	-1.447237623	1.574292091	1.272164167	0.154067071	0.673879826	1.471266055	0.557058159	1.398845427	0.484236553	0.051198257	0.025446151	0.96059789	0.968761611	0.97655257	-0.034230386	0.902274836	-0.148361145
	309.0264	-4.9431228	-4.0026633	0.001669216	0.011913752	0.09400178	-3.4111681	0.08317139	-3.587768882	0.570156442	0.563464087	0.584224388	0.905583237	1.137105322	0.185365887	1.398178653	0.483548713	-3.837768395	-2.087342606	0.006393019	0.044625778	0.228304008	-2.130971909	0.230651105	-2.116215892
	282.0714	-3.5914522	-2.1881827	0.008839995	0.037652316	0.20858289	-2.2613073	0.20964197	-2.254000513	1.705498086	0.948858831	0.126495524	0.668242279	1.830983685	0.872618936	1.423557647	0.509500916	-3.745321197	-2.021923283	0.007212253	0.048444564	0.201222469	-2.313136688	0.227570671	-2.135613456
	399.2177	-5.8421346	-2.4585006	0.000635659	0.006857417	0.16222598	-2.6239232	0.19903538	-2.328903226	1.882979392	0.839882034	0.096462416	0.668242279	1.952773963	0.965524965	1.180735713	0.239686079	-4.6292607	-2.565693113	0.002399778	0.021944774	0.143581485	-2.800058367	0.187978874	-2.41135756
	281.0705	-4.3283424	-2.1889945	0.003444826	0.020546381	0.19894315	-2.3295719	0.19874458	-2.331012606	1.85294164	0.91546342	0.101019513	0.668242279	1.84807388	0.886022432	1.547772159	0.630193114	-4.786365663	-2.073753449	0.001997512	0.019753179	0.184556263	-2.437867397	0.201686698	-2.309812162
	281.0707	-3.7263757	-1.9996363	0.007393851	0.033319127	0.21275391	-2.2327424	0.20646386	-2.276038847	1.691782875	0.995777609	0.129148418	0.668242279	2.105944381	1.074467335	1.971313971	0.979157572	-4.193601911	-2.297860058	0.00406779	0.031481155	0.155886713	-2.681430134	0.166662521	-2.584998388
	220.1003	2.44337304	2.53660112	0.04453804	0.137874279	2.8731561	1.52263638	3.43745144	1.781339333	-1.727507813	-0.784750214	0.12234389	0.668242279	0.590058766	-0.761069449	0.500891906	-0.997428795	2.322746922	2.474052264	0.053178875	0.222725641	2.684309046	1.424550779	3.215081514	1.684855316
	401.225	3.33490893	0.90540884	0.012506615	0.049470612	1.62357803	0.69917672	1.68401196	0.751902382	-2.813516652	-2.175596613	0.022719632	0.524885812	0.406683106	-1.298023034	0.250884186	-1.994906556	-1.064910185	-0.480472482	0.322264554	0.610245644	0.643296588	-0.636444058	0.696194684	-0.522437297
	300.2561	-4.9000519	-2.0972673	0.001753021	0.012236777	0.21741457	-2.2014795	0.22508403	-2.151464369	2.586695296	0.937132062	0.032278524	0.524885812	1.816328487	0.861025141	1.404402647	0.48995662	-3.849359371	-2.019635933	0.006297667	0.044625778	0.221749973	-2.172994164	0.245890236	-2.023913648
	288.1992	2.14864828	1.75611288	0.068750714	0.192718537	1.95777699	0.96921644	2.22707218	1.15514832	-2.693106774	-1.116198647	0.027364623	0.524885812	0.449713035	-1.152923392	0.38863111	-1.363526701	1.918302593	1.568588643	0.096570675	0.305387914	1.714887954	0.778114318	1.774051699	0.827048053
	259.1282	-3.854074	-2.612583	0.006259328	0.030212186	0.15423086	-2.6968367	0.16705727	-2.58158535	0.883563893	0.74955402	0.402702415	0.787264591	2.387569665	1.255542829	1.366694755	0.45069106	-2.204104374	-2.169456548	0.063346079	0.237381096	0.334120395	-1.581560044	0.325958834	-1.617238318
	338.1344	-6.071081	-2.4056724	0.000505303	0.006203025	0.17094359	-2.5484078	0.16735395	-2.579025479	1.83705962	0.812905924	0.103511008	0.668242279	1.771172157	0.824704448	1.550406069	0.632646123	-4.585456545	-2.666226355	0.002527348	0.021944774	0.133840186	-2.90141674	0.159808638	-2.645582706
	273.0531	-4.1969763	-2.3343028	0.004050756	0.022889989	0.17569951	-2.5088179	0.18936201	-2.400781149	1.670539116	0.832711841	0.133359477	0.668242279	1.752942697	0.809778836	1.372198202	0.456488881	-5.062941626	-1.9172705	0.00145855	0.017904958	0.201280091	-2.312723613	0.200882254	-2.315577975
	320.2314	3.24051959	1.90627718	0.014242812	0.053940861	2.37933154	1.25055632	3.07684896	1.621453626	-1.722451897	-0.974160682	0.123286191	0.668242279	0.801859473	-0.318578671	0.423521839	-1.23949173	1.274090377	0.8390831	0.243298409	0.519652811	1.153296513	0.205763478	1.354744775	0.438021083
	370.277	2.86716825	1.5516491	0.024089343	0.083260252	2.22740962	1.15536689	2.12892389	1.090124372	-3.712850032	-1.17579938	0.005932004	0.524885812	0.447241176	-1.160875078	0.372096133	-1.426252696	0.798243275	0.570456487	0.450956644	0.719912848	1.261365107	0.334985929	1.069396932	0.096797443
	245.0075	7.95443312	1.80750778	9.45E-05	0.002427078	3.04085791	1.60447841	3.3429132	1.741105897	-1.235229234	-1.005346116	0.251788948	0.705179471	0.728977183	-0.456054436	0.701709627	-0.51105394	2.424356462	1.377205335	0.045798337	0.204505818	2.315162702	1.211113585	2.785239112	1.477801188
	319.222	3.41864155	1.94703614	0.011155985	0.045131029	2.48212565	1.31157615	3.24580041	1.698574287	-1.631564664	-0.923856549	0.141416311	0.668242279	0.836060456	-0.258320827	0.436375289	-1.196358687	1.431467777	0.909317148	0.195390723	0.451682451	1.227489802	0.295711039	1.43629388	0.522350969
	286.0845	-5.1137833	-1.9482201	0.001378227	0.011253588	0.21820958	-2.1962136	0.22659752	-2.141796047	2.122904234	0.922122845	0.066523785	0.667479319	2.01201212	1.008638996	1.718255785	0.780944817	-3.642932838	-1.868407224	0.008254916	0.053119095	0.2199645	-2.184657387	0.256959957	-1.960384539
	338.9362	1.83271568	1.24919846	0.109508496	0.267020716	2.119013	1.08339244	2.50974491	1.327540734	-2.629478138	-1.431058181	0.030202644	0.524885812	0.329969628	-1.599594857	0.251418071	-1.991839747	0.337312354	0.192636533	0.745769969	0.884980363	0.833754043	-0.262306243	1.031521616	0.044774054
	302.2148	1.81526693	1.47370501	0.112344846	0.271105517	1.72897204	0.78991454	1.90865693	0.932557811	-2.680969241	-1.196877372	0.027884089	0.524885812	0.417582926	-1.259865371	0.347884905	-1.523318015	1.541608141	1.185769205	0.167071164	0.424838103	1.348850132	0.431730062	1.382042606	0.466802092
	385.063	2.88044985	1.45214069	0.023636391	0.082495639	2.28679667	1.1933281	2.00611419	1.004403726	-1.620783112	-1.211069919	0.143722737	0.668242279	0.689148678	-0.537112828	0.445116885	-1.167743866	2.752814053	1.536999299	0.028388538	0.144375995	2.487693178	1.31480856	2.041580645	1.029686557
	401.2308	3.85152052	1.67799145	0.006280061	0.030212186	2.35265928	1.2342924	2.89195067	1.532042943	-1.723569931	-0.93575522	0.123077236	0.668242279	0.799070022	-0.323606165	0.428830745	-1.22151975	0.369205186	0.299276939	0.722889729	0.877916933	1.085667781	0.1185827	1.18741408	0.247823126
	225.0615	-3.8132884	-1.9041883	0.006599607	0.031326136	0.24519896	-2.0279752	0.24799487	-2.011617818	1.741506787	0.822414291	0.11976976	0.668242279	1.692715172	0.759339236	1.406165436	0.491766338	-4.952095141	-1.949065791	0.001652323	0.018382094	0.200685586	-2.316991091	0.214676001	-2.219767178
	176.028	2.05305878	1.35371292	0.079172141	0.211919414	2.18523855	1.12779078	1.56273949	0.644077299	-1.470557049	-1.130440682	0.179614408	0.673879826	0.562573376	-0.829886817	0.474930891	-1.074210497	0.469803404	0.644692011	0.652779134	0.829962042	2.227612305	1.155498167	1.509673946	0.594236995
	390.294	2.77786584	2.2347568	0.02738223	0.091962963	2.47139808	1.30532741	2.60344712	1.380423104	-0.842004693	-0.659097682	0.424239594	0.787264591	0.904163995	-0.145343626	0.912484356	-0.132128269	1.154430838	1.219672658	0.286208508	0.57565101	1.440505347	0.526575016	1.699954244	0.765495915
	301.2925	1.81307093	1.14848691	0.112706788	0.271105517	2.27886927	1.18831817	2.22795518	1.155720213	-1.90995804	-1.249197193	0.092537179	0.668242279	0.390650299	-1.356050377	0.365986328	-1.450138339	1.834414834	0.729252428	0.109236014	0.326790091	1.318950737	0.399390681	1.49014151	0.575449342
	229.0547	-3.1245415	-3.0144915	0.016737005	0.060185593	0.13750526	-2.8624413	0.13716283	-2.866038533	0.407446536	0.471488032	0.694365069	0.956913237	1.950118041	0.963561453	1.416187682	0.502012473	-2.355194037	-2.884512511	0.050698509	0.217453844	0.244174529	-2.034015379	0.242743229	-2.042497042
	359.1027	-4.8042645	-1.965232	0.001956633	0.013395407	0.22601292	-2.1455228	0.22181232	-2.172588603	1.309578609	0.698865538	0.226694484	0.691850039	1.779305096	0.83131391	1.527480917	0.611154356	-6.24768304	-1.842432231	0.000425105	0.007566866	0.211032131	-2.244465423	0.221155587	-2.176866405
	230.0657	-3.2262873	-2.3107559	0.014526269	0.054435281	0.17451486	-2.5185782	0.20496998	-2.286515462	0.948871061	0.592831641	0.370465182	0.771260846	1.48508624	0.570546712	1.125720016	0.170848051	-2.275740398	-1.824045093	0.056993059	0.22543921	0.267399476	-1.902931454	0.284437056	-1.813818666
	172.0437	2.12458933	1.62017564	0.071237095	0.196592294	2.19487091	1.13413609	2.19584405	1.134775597	-1.442524181	-0.84204518	0.187137356	0.673879826	0.608803614	-0.715951171	0.453867392	-1.139657253	1.361123864	1.294530322	0.215666215	0.47430188	1.969654485	0.977942576	1.801893322	0.849513601
	453.2893	15.2524611	3.64729253	1.25E-06	0.000446455	10.1398932	3.34197055	11.8309591	3.56449513	-1.317759305	-0.358708879	0.224067544	0.691850039	0.831219131	-0.266699235	0.700752328	-0.513023462	18.7678827	3.659507558	3.03E-07	9.33E-05	9.493473985	3.246936115	11.54762141	3.529523809
	260.1679	1.92033514	1.45387989	0.096282263	0.246593422	1.89246783	0.92026878	1.79123437	0.840954119	-2.104000504	-0.895074714	0.068509836	0.667479319	0.510883691	-0.968933215	0.437558257	-1.192452984	2.092653598	1.604048787	0.074676863	0.264075916	2.069261134	1.04911572	1.782757702	0.834110637
	478.3374	11.1549618	3.30332088	1.04E-05	0.000614906	7.93563574	2.98834581	9.57586382	3.259402636	-1.360943013	-0.382130986	0.210628555	0.691850039	0.810616442	-0.302908656	0.662813928	-0.593324177	12.02795923	3.273162663	6.26E-06	0.000445695	7.212967158	2.850592855	9.187472529	3.199668031
	425.0648	-3.5329678	-1.4436091	0.009559652	0.039963863	0.30451371	-1.7154209	0.34006595	-1.556113533	1.294276094	0.863622852	0.231678808	0.691850039	2.860967225	1.51650297	1.806694078	0.853352239	-3.941236486	-1.209458793	0.005594469	0.041492315	0.322568543	-1.632322343	0.362251169	-1.46493775
Linoleic Acid	279.233	-3.4337502	-2.8024159	0.010929448	0.044722797	0.22263035	-2.1672778	0.21573409	-2.212673956	0.372627548	0.441918056	0.719104245	0.959250658	2.632763151	1.396577739	1.540697709	0.623583827	-2.292212614	-2.416259853	0.055625828	0.222899049	0.291476592	-1.778548065	0.278400124	-1.844768241
	246.1525	2.72387107	1.75287271	0.029599573	0.098480823	2.53416316	1.34150941	2.66664569	1.415026148	-1.657812869	-0.680394957	0.135942548	0.668242279	0.586376319	-0.770101254	0.484790434	-1.044566862	2.312905519	1.671622511	0.053955354	0.222899049	2.463278808	1.300579929	2.416227056	1.272756033
	477.3336	11.341699	3.29987631	9.28E-06	0.000614906	8.14590238	3.02607453	8.73040915	3.126049268	-1.267584326	-0.350676422	0.24059523	0.696356927	0.831089527	-0.266924199	0.689071146	-0.537275148	13.07450751	3.304910994	3.57E-06	0.000317644	7.583655448	2.922893419	8.466470113	3.081760599
	337.1334	-5.9668635	-1.9812533	0.000560513	0.006436858	0.2201557	-2.1834039	0.20258415	-2.30340676	1.672438819	0.565239568	0.132977801	0.668242279	1.504986949	0.589750976	1.369286815	0.45342467	-5.170869879	-2.22079378	0.001293844	0.016450297	0.169777985	-2.558278694	0.191602556	-2.383811291
	359.2221	-7.6177937	-2.1084151	0.000124427	0.002605647	0.20081835	-2.316037	0.21821834	-2.19615573	1.969779777	0.523385053	0.084372386	0.667479319	1.502512086	0.587376596	1.110531566	0.151250401	-9.615065524	-2.181976494	2.77E-05	0.001006338	0.171461244	-2.544045576	0.189643105	-2.398641176
	258.1533	2.08135915	1.42434312	0.075932614	0.206351225	2.11800855	1.08270841	2.18523815	1.127790518	-1.619951795	-0.755110645	0.143901998	0.668242279	0.537796677	-0.894867253	0.446467846	-1.163371818	2.061390211	1.538452833	0.078204442	0.272948836	2.355498781	1.236032585	2.138668686	1.096713003
	236.0961	-4.6743178	-1.1607221	0.002275937	0.014657568	0.36717612	-1.4454559	0.38962902	-1.35982697	2.042495999	0.916115674	0.075381237	0.667479319	2.490895112	1.316664273	1.70055387	0.766004708	-1.796362867	-0.695036662	0.11549757	0.334285651	0.47565881	-1.072000995	0.604960263	-0.725087714
	237.099	-4.5736761	-1.1622787	0.00256292	0.016007012	0.36511478	-1.453578	0.38498143	-1.377139244	2.052276934	0.912558431	0.074245211	0.667479319	2.474805119	1.307314923	1.701811073	0.767070884	-1.786444909	-0.710993695	0.117185459	0.335519602	0.469190643	-1.091753852	0.595481578	-0.747871218
	238.0912	-4.7426701	-1.1752542	0.002101339	0.014114657	0.36325148	-1.4609594	0.379724	-1.396976915	1.93450988	0.881194331	0.089098318	0.668242279	2.459102516	1.29813188	1.685392825	0.753084889	-1.863618795	-0.70127985	0.104653954	0.315735659	0.467427977	-1.097184008	0.588284276	-0.76541462
	285.083	-6.0189873	-1.6270683	0.000532099	0.00631424	0.27035817	-1.8870562	0.25155064	-1.991079262	2.567119401	0.627798115	0.033276342	0.524885812	1.612021444	0.688870935	1.419311724	0.505191484	-5.449763087	-1.674685478	0.000956239	0.01427546	0.240066563	-2.058493619	0.24002616	-2.058736444
	415.2439	1.98689766	2.06287497	0.087289626	0.230185976	2.09672741	1.06813931	2.14932392	1.103882927	-1.214094131	-0.494377605	0.259332983	0.705179471	0.886747169	-0.173405275	0.604550505	-0.726065226	1.133517475	1.392376873	0.294321368	0.579626539	1.603677989	0.681384485	1.663290925	0.734040532
	315.1273	9.61183092	2.38691117	2.78E-05	0.001411662	4.27842395	2.09707945	4.25108378	2.087830693	-1.83953591	-0.417640987	0.103118739	0.668242279	0.793407619	-0.333865844	0.676346439	-0.564165681	9.518950014	2.253820112	2.96E-05	0.001006338	3.628197875	1.85925314	3.789237992	1.921907754
	331.1509	-5.2019088	-1.7000516	0.001250385	0.010729234	0.28990284	-1.7863586	0.26067291	-1.939687414	1.432918092	0.563046933	0.189778536	0.673879826	1.595850203	0.674325237	1.206746208	0.271122294	-4.899491839	-1.491421555	0.001754141	0.018923464	0.290595212	-1.782917161	0.29384736	-1.766861159
	270.117	1.84359321	1.20811015	0.107775424	0.264607247	1.68541179	0.75310112	1.96664067	0.97573338	-1.723057506	-0.760743448	0.123172964	0.668242279	0.559869311	-0.836837993	0.44692774	-1.161886501	1.182413052	0.968355386	0.275643824	0.570518612	1.63199728	0.706638652	1.633521985	0.707985871
	163.0247	-2.0424001	-1.1291937	0.080427489	0.213673031	0.40176415	-1.3155793	0.40965825	-1.287507214	1.683969195	0.774148543	0.13068277	0.668242279	1.654713445	0.7265814	1.466517156	0.552393948	-0.061710076	-0.030780674	0.952518735	0.964899385	0.746925598	-0.420963554	0.851289499	-0.23227826
	244.1373	2.79871373	1.51985645	0.026573329	0.090096241	2.47960637	1.31011111	2.64544129	1.403508402	-1.224053035	-0.572409491	0.255755169	0.705179471	0.654465652	-0.611610616	0.517793936	-0.949550025	2.291006953	1.534926273	0.055724762	0.222899049	2.744365005	1.456472375	2.585821779	1.370622845
EIC_58	309.2049	-2.5029541	-0.769137	0.040815427	0.127458703	0.45377441	-1.1399529	0.55308053	-0.854438545	2.187023118	1.12915561	0.060199738	0.667479319	3.612128961	1.852849401	2.088294045	1.062324867	-1.515086984	-0.47544504	0.173525734	0.426035596	0.548686975	-0.865944765	0.653401648	-0.613958001
	361.2003	-5.4119011	-1.8143824	0.000995706	0.009089005	0.23913573	-2.0640984	0.27041348	-1.886761006	1.515538569	0.458697188	0.168103722	0.673879826	1.439209602	0.525276717	1.071619782	0.099793119	-4.316887831	-1.684253964	0.003493483	0.02826545	0.251756888	-1.989896843	0.28948664	-1.788431324
	221.1038	2.26921651	1.21491656	0.057543934	0.167915086	1.93754746	0.95423165	1.77162517	0.825073402	-1.606576998	-0.679623865	0.146814325	0.670075636	0.632631912	-0.660561764	0.536726618	-0.897740656	1.911177931	1.16191206	0.097588318	0.305387914	1.837125825	0.877450441	1.668500648	0.738552247
	323.9641	-2.5517909	-3.2642354	0.038004139	0.12079887	0.08890627	-3.491571	0.12256174	-3.028419382	0.278099725	0.248871703	0.787988133	0.98492983	0.894935065	-0.160145089	0.556425312	-0.845740046	-1.36056664	-2.062534333	0.215834001	0.47430188	0.239686709	-2.060778182	0.450718022	-1.149702958
	362.2916	-5.8879785	-1.717885	0.000606802	0.006750675	0.27250103	-1.8756664	0.27193019	-1.878691742	1.617021296	0.469804773	0.144535549	0.668242279	1.398891107	0.484283664	1.139164217	0.187975735	-6.745020473	-1.697086148	0.00026622	0.005265246	0.242047091	-2.046640341	0.259138836	-1.94820285
	318.2156	12.4003451	2.42255458	5.10E-06	0.00060519	4.45157617	2.15431624	4.89941455	2.292609366	-1.211033372	-0.320027065	0.260440877	0.705179471	0.851256668	-0.232333902	0.710336939	-0.493424584	16.0976834	2.701541099	8.68E-07	0.000102976	5.0125031	2.325531224	5.642924779	2.496443118
	244.0463	-2.1275423	-1.2077577	0.070927142	0.196592294	0.42832295	-1.2232291	0.45959136	-1.121576421	0.648592997	0.632380402	0.534771873	0.863071616	2.572259365	1.363036119	1.514789257	0.599117095	-1.272681846	-0.746339244	0.243769717	0.519652811	0.540867912	-0.886651786	0.550295434	-0.861721736
	416.1663	-5.6221165	-1.9432561	0.000797309	0.008109773	0.23337342	-2.0992879	0.20456165	-2.289392383	0.95474489	0.391298138	0.367661874	0.770899441	1.345281922	0.42790854	1.314945083	0.395002548	-5.97501926	-1.320231378	0.000555957	0.008996396	0.309031731	-1.694173116	0.310310236	-1.688216808
	297.1864	1.77443025	1.14440054	0.119261867	0.283048164	2.56243864	1.35751746	2.65225346	1.40721865	-1.309137653	-0.653943993	0.226836824	0.691850039	0.432595606	-1.208909082	0.38311001	-1.384169373	1.992172477	0.751034453	0.086613361	0.286612995	1.193926785	0.255714369	1.389244532	0.474300562
	335.223	1.86728627	1.05037618	0.104091858	0.262609325	2.46193747	1.29979412	2.3275667	1.218822512	-1.427850621	-0.708842952	0.191185007	0.673879826	0.430010897	-1.217554874	0.410678744	-1.283917818	2.723494099	0.749287038	0.029615699	0.146433177	1.265779601	0.340026223	1.369370889	0.453513248
EIC_85;EIC_8	317.2123	11.8671824	2.62995359	6.85E-06	0.000609757	5.17327415	2.37107765	5.71369523	2.514424085	-0.99361217	-0.278026205	0.349510636	0.754095675	0.877935982	-0.187812351	0.723463109	-0.467008642	17.32713711	2.942730038	5.24E-07	9.33E-05	5.948342797	2.572487791	6.695802899	2.743257061
	222.0967	4.23715429	1.31371176	0.00385385	0.02212856	2.0123435	1.00887659	1.9640266	0.973814471	-2.054836384	-0.547933804	0.073950702	0.667479319	0.716408531	-0.481145577	0.631814622	-0.662426769	3.342099953	1.349854066	0.01238399	0.073478343	2.063145983	1.044845906	1.951575664	0.964639398
	257.1542	-3.7683254	-1.5701205	0.006998238	0.032355491	0.27801357	-1.8467728	0.29248776	-1.773551824	1.363305697	0.440036266	0.209913757	0.691850039	1.414435798	0.500226694	1.038891852	0.055045478	-3.309421582	-1.374825226	0.01295185	0.075587843	0.293671028	-1.767727148	0.320682726	-1.64078145
	322.2139	5.51601154	1.73797671	0.000891307	0.008575823	2.70973524	1.4381519	2.99562732	1.582858151	-1.383644793	-0.397121641	0.203846833	0.689049618	0.759617773	-0.396654433	0.711267402	-0.49153605	3.34394112	1.461494132	0.012352802	0.073478343	2.08273323	1.058478062	2.716776596	1.441895936
	379.2493	8.22681148	2.15869672	7.62E-05	0.002427078	3.72670375	1.89790014	4.06900276	2.024675258	-1.138852466	-0.318632611	0.287716285	0.72152734	0.847700501	-0.238373456	0.683443649	-0.549105705	11.63815821	2.298407046	7.81E-06	0.000463197	3.801637784	1.92662108	4.21597982	2.075867962
	218.0857	1.90649662	1.21914004	0.098262657	0.249867898	2.25621452	1.17390424	2.08579209	1.060595361	-0.863326961	-0.555834163	0.4130905	0.787264591	0.687605472	-0.54034707	0.528529019	-0.91994541	1.519233703	1.134078036	0.172501699	0.426035596	2.225179938	1.153922004	1.926665218	0.946105907
	149.0009	3.17105336	1.46167888	0.015684817	0.0569775	2.59623063	1.37641855	2.50172186	1.322921402	-0.586572866	-0.449551719	0.573665351	0.899669008	0.904613148	-0.14462713	0.876807538	-0.189667894	2.942688087	1.03393379	0.021630444	0.114931913	1.664277054	0.734895621	1.73657474	0.796244504
	198.0522	2.85870482	1.26220343	0.024382742	0.083464001	2.20005453	1.13753928	2.33854519	1.225611308	-0.672249946	-0.508510853	0.520363622	0.859999021	0.905477362	-0.143249522	0.793649957	-0.333425254	1.522942599	0.673147585	0.17159047	0.426035596	1.364525364	0.448399211	1.556073373	0.637910089
	335.1872	8.72477653	2.32179524	5.22E-05	0.002064111	4.06628055	2.02370976	4.42543687	2.145819881	-0.800516152	-0.263999826	0.446531054	0.802853814	0.888760544	-0.170133325	0.772257782	-0.372845592	10.79105015	2.465785145	1.29E-05	0.000657295	4.167594293	2.059214841	4.65306725	2.218182038
	219.0887	2.16752001	1.32233347	0.066861408	0.190421291	2.37560008	1.24829199	2.40106642	1.263675312	-0.776703314	-0.453678741	0.459680345	0.822342727	0.716281488	-0.481401439	0.550250675	-0.861839085	2.026527322	1.326445297	0.08233358	0.281834178	2.39822435	1.261966627	2.284050883	1.191594791
	315.1967	4.97802282	1.56585535	0.001604563	0.01190051	2.33296272	1.22216325	2.38159691	1.251929257	-2.504593323	-0.371263813	0.03667859	0.524885812	0.795661678	-0.32977298	0.657972745	-0.603900269	4.961008203	1.566338324	0.001635729	0.018382094	2.119577613	1.083776795	2.247056882	1.168036645
	346.2601	3.61065203	1.05393454	0.008616697	0.037409077	1.92300793	0.94336471	1.79872901	0.846977849	-1.755820583	-0.548701403	0.117190142	0.668242279	0.659399027	-0.600776337	0.614088035	-0.703482601	1.55576909	0.546851148	0.163715736	0.424838103	1.200769454	0.263959182	1.283318251	0.35987899
	415.1637	-7.0619617	-2.1270729	0.00020026	0.003240568	0.19413415	-2.3648741	0.1891097	-2.402704725	0.664206275	0.258107268	0.525235648	0.861676916	1.34728587	0.430055998	1.123512814	0.168016578	-4.982014926	-2.532795828	0.001597347	0.018382094	0.141437039	-2.821768121	0.163391089	-2.613598788
	227.1288	4.6799012	1.74040641	0.002261089	0.014657568	2.42836093	1.27998287	2.88936964	1.53075478	-1.025673181	-0.297250617	0.335056404	0.748391487	0.868602732	-0.203231605	0.791895122	-0.336618722	4.255268175	1.793377038	0.003768534	0.029813292	2.380491294	1.251259352	2.988836548	1.579584002
	325.1479	6.74294228	1.62919865	0.000266727	0.003956447	2.55004766	1.35052421	2.85792397	1.514967538	-1.080429367	-0.304033838	0.311442977	0.739157999	0.883188496	-0.179206714	0.692395298	-0.530332168	2.30546594	0.923837642	0.054549977	0.222899049	1.594872324	0.673440935	1.897949294	0.924441449
	316.2504	5.19075743	1.67947131	0.001265809	0.010729234	2.56070418	1.3565406	2.45903796	1.298094005	-0.836195383	-0.291842399	0.427313408	0.788205042	0.885245632	-0.175850274	0.850841463	-0.233037754	4.324217927	1.696586213	0.00346226	0.02826545	2.534297258	1.341585754	2.493466478	1.318152808
12-HETE [M-	379.2481	-2.0942778	-0.7197692	0.074497985	0.204009866	0.46640251	-1.1003526	0.5412861	-0.885536748	1.379176539	0.675458439	0.205166459	0.689049618	2.493055935	1.317915252	1.509869969	0.594424309	-1.443350234	-0.596904493	0.192139163	0.451682451	0.497166613	-1.00819868	0.623248048	-0.682121635
	158.028	1.85107181	1.51941599	0.106599301	0.263537161	2.2464313	1.16763494	2.48317743	1.312187351	-0.401467689	-0.307156799	0.698586509	0.956913237	0.972490488	-0.040243956	0.62686147	-0.673781437	1.922297683	1.509336137	0.096004583	0.305387914	2.007847081	1.005649397	1.994737772	0.996199103
	381.2654	6.4906563	1.36327316	0.000337084	0.004800076	2.14090725	1.0982223	2.35736413	1.237174623	-1.544396943	-0.340383413	0.161071291	0.673879826	0.839864662	-0.251771229	0.663330815	-0.592199548	9.446757724	1.483878239	3.11E-05	0.001006338	2.160036949	1.111055991	2.433890685	1.283264373
	222.0522	2.67759187	1.21836671	0.031649503	0.10336902	2.12446056	1.08709656	2.22956627	1.156763081	-0.517212335	-0.378695959	0.61900032	0.92287782	0.968309074	-0.046460481	0.825548206	-0.276575635	2.392364395	0.806350155	0.048001717	0.208397697	1.403447591	0.488975191	1.53601973	0.619196748
	277.1447	-7.4997332	-1.7269904	0.000137335	0.002704972	0.25065687	-1.9962143	0.26644017	-1.908116469	1.32071234	0.245344064	0.223125687	0.691850039	1.266327008	0.340650005	0.951893156	-0.071128446	-6.936911155	-1.757704355	0.000223798	0.004944958	0.227834887	-2.133939422	0.253717199	-1.978706772
	377.1959	7.9424481	1.9967528	9.54E-05	0.002427078	3.13142292	1.64681837	3.57030163	1.836045962	-1.440175871	-0.212076945	0.187780011	0.673879826	0.920143974	-0.120068479	0.73901461	-0.436325208	4.119627402	1.471276888	0.004461651	0.033794631	2.097217305	1.068476356	2.369819652	1.244777271
	401.234	-7.0762471	-1.7806283	0.000197753	0.003240568	0.24264221	-2.0430976	0.27973318	-1.837876693	0.533032299	0.223745391	0.608495795	0.92287782	1.406060883	0.491659066	1.017732477	0.025358382	-4.752679254	-1.77325344	0.002077039	0.019984486	0.237644273	-2.073124457	0.280792642	-1.832422962
	294.1793	-8.3158674	-1.518931	7.11E-05	0.002427078	0.29306282	-1.7707181	0.31087638	-1.685587104	1.042847351	0.262224493	0.327505162	0.748391487	1.283393286	0.359963341	1.06240955	0.08734002	-7.205833739	-1.343836992	0.000176559	0.004489642	0.306392559	-1.706546836	0.338661202	-1.562085376
	258.2157	7.8261909	1.37060128	0.000104845	0.00248832	2.11259017	1.07901292	2.33205695	1.221603018	-1.336296711	-0.289835324	0.218211052	0.691850039	0.85925513	-0.218841535	0.732001306	-0.450081873	8.073878047	1.340735095	8.59E-05	0.002549824	1.917604128	0.93930492	2.271734174	1.183794028
	181.0258	2.39243739	1.16832623	0.047996568	0.144803204	2.0793138	1.0561075	2.1874562	1.129254133	-0.458215811	-0.336315931	0.658987563	0.945512523	0.96705833	-0.048325184	0.843008266	-0.246381317	1.485941617	0.671164425	0.180881114	0.44105258	1.339528305	0.421725066	1.515425391	0.599722826
	249.0325	3.94742587	1.71165989	0.005550278	0.028227128	2.48265911	1.31188618	2.36443048	1.241492723	-0.498597547	-0.227559285	0.631480831	0.932809858	0.968758151	-0.045791551	0.922842127	-0.115844231	3.913611831	2.231356035	0.005796443	0.042112936	3.55710054	1.830701752	3.682337415	1.880621828
	244.2	6.81077308	1.26768756	0.000250741	0.003881038	1.98119668	0.98637211	2.16152323	1.112048338	-1.432076327	-0.300506904	0.190011535	0.673879826	0.858708586	-0.219759478	0.714278619	-0.485441158	7.365574615	1.20298237	0.00015387	0.00421368	1.758805314	0.814595797	2.030293027	1.021687963
	274.0569	-2.2085677	-1.9997716	0.062930138	0.180670396	0.18216294	-2.4566986	0.19700841	-2.343670885	0.342016493	0.189714928	0.741149815	0.961972151	0.975815693	-0.035319409	0.790493456	-0.339174575	-1.533713745	-1.156276436	0.168969065	0.426035596	0.249108994	-2.005150986	0.274262645	-1.866369956
	349.2053	-3.3969442	-1.3377127	0.011490202	0.045960807	0.35346041	-1.5003795	0.34507823	-1.535004625	0.849597352	0.278554439	0.420245544	0.787264591	1.190022533	0.250988891	1.006921041	0.009950557	2.142365049	2.093940548	0.06939155	0.257326998	5.887179269	2.557576559	7.768832093	2.957697731
	339.1636	6.2363969	1.42653706	0.00042978	0.005464344	2.22272786	1.15233132	2.43137512	1.281772491	-0.983796772	-0.259398995	0.354029289	0.754697166	0.908842612	-0.137897616	0.717456133	-0.47903747	3.141075649	0.984140947	0.016354593	0.093907019	1.598317557	0.676554074	1.866685922	0.900479209
	327.1637	5.24432396	1.3680527	0.001193599	0.010623034	2.13457073	1.09394597	2.3020711	1.202932392	-0.889877501	-0.267537904	0.399500098	0.787264591	0.907135115	-0.140610643	0.730087035	-0.453859635	2.518625198	0.861506438	0.03989063	0.182064928	1.472690612	0.558454376	1.688725802	0.755935097
	263.0485	5.10181405	1.31558389	0.001396685	0.011253588	2.0419439	1.02994323	2.03136506	1.022449531	-0.966610608	-0.272607998	0.362047196	0.767195248	0.878041407	-0.187639118	0.780987839	-0.35662801	4.615980235	1.406321608	0.002437687	0.021944774	2.079690931	1.056369141	2.210698933	1.144502563
	339.1636	6.30856199	1.45440269	0.00040085	0.00528528	2.2629889	1.17822951	2.46625594	1.302322526	-0.886860579	-0.240862945	0.401028017	0.787264591	0.919996594	-0.120299576	0.73486607	-0.444446754	2.954124573	0.966746492	0.021281837	0.11479294	1.58163108	0.661413127	1.857186809	0.893118939
	311.1326	5.00386154	1.57743032	0.001558486	0.011804704	2.42017516	1.27511147	2.65044876	1.406236649	-0.675630215	-0.218177611	0.518324544	0.859999021	0.946187216	-0.079802427	0.740052048	-0.434301356	2.335676199	1.001230398	0.052176023	0.221126955	1.640135003	0.713814571	1.916202606	0.93825011
	144.1018	-2.1584488	-1.3893859	0.06776297	0.191457279	0.25353348	-1.9797518	0.34790312	-1.52324248	0.674736714	0.247183201	0.518863049	0.859999021	1.21518071	0.281170874	0.875433055	-0.191931236	-1.436306789	-0.839049692	0.194060641	0.451682451	0.35409488	-1.497792112	0.404059631	-1.307359875
	257.1544	6.34314556	1.33186258	0.000387765	0.00528528	2.08994726	1.06346654	2.25508489	1.173181742	-1.013342735	-0.247885395	0.340560171	0.748391487	0.895871619	-0.15863609	0.727635146	-0.458712865	6.94080726	1.422815028	0.000223019	0.004944958	2.084941691	1.060007037	2.34562029	1.229969488
15-HETE [M-	379.2485	-2.2237624	-0.6640672	0.061534652	0.178100293	0.49905235	-1.0027369	0.56452796	-0.824883057	1.052065491	0.487266466	0.32350687	0.748391487	2.098441009	1.069317907	1.296563542	0.374692911	-1.705540099	-0.736553163	0.13186242	0.36389939	0.481497192	-1.054400709	0.597324946	-0.743412122
	293.1764	-13.114321	-1.4310324	3.50E-06	0.00060519	0.30980088	-1.6905868	0.3311607	-1.594396627	1.096024684	0.225911098	0.304962456	0.728635129	1.247535549	0.319080927	1.044595374	0.06294422	-6.877142431	-1.279828157	0.000236136	0.004944958	0.320241051	-1.64276984	0.360748187	-1.47093595
11(S) HEPE [I	317.2122	7.685847	1.91933952	0.000117611	0.002605647	3.03892073	1.60355904	3.45625531	1.789209794	-0.739800746	-0.165265396	0.48056448	0.842763325	0.945089215	-0.081477571	0.767418601	-0.381914361	10.53173033	2.230706541	1.52E-05	0.000675924	3.508474643	1.810843936	3.950973154	1.982208044
	327.1637	5.52390984	1.32174004	0.0008839	0.008575823	2.07043036	1.04993068	2.20738052	1.142335354	-0.84827796	-0.227690589	0.420937696	0.787264591	0.926046405	-0.110843605	0.741875655	-0.430750696	2.412183581	0.861668832	0.046624235	0.204916389	1.496814861	0.581895787	1.691341396	0.758167896
	357.2074	-3.9826588	-0.8813931	0.005305869	0.027375211	0.44888937	-1.1555682	0.4963066	-1.010696456	1.280911921	0.338955892	0.236107556	0.694663552	1.442881847	0.528953167	1.00288555	0.004156974	-4.792672588	-0.864585946	0.001983001	0.019753179	0.418573145	-1.256448341	0.474452602	-1.075664127
	327.1637	4.94097948	1.27315528	0.00167328	0.011913752	2.00434423	1.0031303	2.12531312	1.087675404	-0.841336915	-0.229525812	0.424592139	0.787264591	0.924684498	-0.112966892	0.73617916	-0.441871184	2.726896315	0.871669441	0.029470498	0.146433177	1.466270948	0.55215172	1.659928155	0.7311208
	313.1482	4.6632516	1.3443952	0.002305685	0.014657568	2.10057286	1.07078283	2.24944875	1.1695715	-0.715932315	-0.211244339	0.494397149	0.852583727	0.942204137	-0.085888428	0.732888233	-0.448334894	2.577099883	0.90469621	0.036626807	0.171567673	1.520022864	0.604093025	1.719233329	0.781765357
	297.1163	5.46318903	1.31810497	0.000942665	0.008831281	2.05545594	1.03945844	2.14507573	1.101028581	-0.858937787	-0.199466915	0.415368444	0.787264591	0.937963397	-0.092396471	0.747823362	-0.419230553	2.703838661	0.922492947	0.030469334	0.148590176	1.523065673	0.60697815	1.725213431	0.786774853
	375.1792	-5.6778849	-1.2984921	0.000752379	0.007877847	0.33843278	-1.5630588	0.36264056	-1.463387785	0.848553565	0.19796086	0.420793048	0.787264591	1.227147838	0.295309065	0.971535352	-0.041661601	-4.591552831	-1.122845932	0.002509153	0.021944774	0.358547659	-1.479763198	0.395873264	-1.336889458
	327.1637	5.06566663	1.29679489	0.001454115	0.011253588	2.03586867	1.0256445	2.16932447	1.117245859	-0.735472345	-0.196378047	0.483054113	0.842976785	0.950115813	-0.073824715	0.744997125	-0.424693237	2.766717975	0.852401133	0.027825332	0.143562581	1.441115924	0.527186391	1.635584393	0.709806202
	350.2083	-3.5256404	-1.3430404	0.009654192	0.039963863	0.35477016	-1.4950434	0.34868926	-1.519986181	0.555036935	0.181591631	0.594044283	0.915496817	1.115663736	0.157902261	0.937030424	-0.093832204	2.134831907	2.068626649	0.070167756	0.257522899	5.851463457	2.548797489	7.690459307	2.943069765
	431.3015	1.77566178	0.47069381	0.11904742	0.283048164	1.20860447	0.27334219	1.18770548	0.248177126	-2.197100291	-0.478440223	0.059261265	0.667479319	0.727106184	-0.459762029	0.60592462	-0.722789769	1.769639282	0.374981233	0.12009964	0.336594452	1.001536444	0.002214919	1.023141406	0.033005551
	317.1398	-4.0375076	-1.2002399	0.004948471	0.025906703	0.36112761	-1.4694194	0.39328395	-1.346356792	0.698824045	0.164986612	0.504467363	0.857503199	1.206150434	0.270409855	0.859877932	-0.217796225	-5.405561105	-1.294548952	0.00100249	0.01427546	0.312755431	-1.676893159	0.339402722	-1.558929958
	343.26	-4.1154126	-1.2477464	0.004485318	0.024524702	0.32004047	-1.6436738	0.37496353	-1.415177796	0.568852326	0.154789089	0.585067822	0.905583237	1.228721546	0.297158008	1.015171653	0.02172369	-0.547109886	-0.30310593	0.601301551	0.808592193	0.664134208	-0.590453284	0.978264365	-0.031703704
	293.1762	-8.1286229	-1.2471364	8.23E-05	0.002427078	0.35134341	-1.5090463	0.37740945	-1.405797533	0.708903864	0.145042283	0.498518594	0.853233747	1.1858505	0.245922141	0.964049062	-0.052821526	-6.64074158	-1.162338464	0.000293022	0.005490307	0.346978188	-1.527083119	0.388619534	-1.363569676

	294.1792	-7.3801856	-1.2243773	0.000151965	0.002704972	0.35710137	-1.4855944	0.38235694	-1.38700802	0.622684386	0.140247993	0.550824655	0.868511102	1.182065378	0.24130983	0.971434809	-0.041810912	-6.11470097	-1.108671	0.000484025	0.008205368	0.360406885	-1.472301523	0.403555308	-1.309161683
	413.2237	2.06352337	0.45805035	0.077958568	0.210251897	1.18441047	0.24416915	1.13382754	0.181201216	-1.451351151	-0.368561273	0.184739029	0.673879826	0.790093881	-0.339904006	0.714963982	-0.48405753	1.473811675	0.348711005	0.184024998	0.444733227	0.990522711	-0.013738041	1.044814146	0.063246335
	331.0413	-2.6673686	-0.8715955	0.032122008	0.103958497	0.45953508	-1.1217531	0.49725076	-1.007954524	0.6698784	0.192528464	0.521797159	0.859999021	1.198367129	0.261069956	0.911532794	-0.133633533	-1.914922158	-0.533915808	0.097052218	0.305387914	0.540257505	-0.888280887	0.579387174	-0.787400347
	383.2207	-2.4147858	-0.9819672	0.046446409	0.142542427	0.46887406	-1.0927276	0.45481405	-1.136651268	0.27741246	0.160981517	0.788497196	0.98492983	1.305753503	0.384882574	0.959549005	-0.059571607	-2.666482351	-1.328990981	0.032163314	0.152668528	0.368195158	-1.441457441	0.349710163	-1.515768372
	399.2504	-2.5360411	-1.0859189	0.038888219	0.122515096	0.35868887	-1.4791951	0.44146284	-1.1796361	0.17023376	0.134120776	0.869052679	0.998232252	1.73334239	0.793556661	2.137983503	1.096250721	-0.718763587	-0.376581077	0.495573061	0.759867583	0.635130698	-0.654874593	0.613770644	-0.70422845
	318.1431	-4.0642705	-1.0979472	0.004783713	0.025417935	0.38558066	-1.3748954	0.42209784	-1.244350653	0.643203404	0.131731606	0.53808786	0.863071616	1.183333942	0.242857266	0.845120954	-0.24277026	-5.182528895	-1.182653116	0.001277325	0.016450297	0.336772625	-1.570153223	0.369765186	-1.435318695
	249.1496	-4.2818706	-1.1037321	0.003646973	0.021283976	0.37625337	-1.4102236	0.42023388	-1.250735609	0.3355847	0.099913421	0.745815255	0.96199359	1.251665079	0.323848577	0.855411758	-0.225309057	-4.821064288	-1.198771399	0.001919104	0.019753179	0.327948684	-1.608458008	0.376610692	-1.408854136
EIC_294;EIC_	359.2208	2.35427673	1.2928961	0.050766984	0.150608721	1.83597997	0.87655032	1.86592397	0.899890204	-0.254505641	-0.073288173	0.805524838	0.995041444	0.971047774	-0.042385819	0.758052236	-0.39963083	2.009443467	1.146281439	0.084435051	0.283775793	1.617167781	0.693469366	1.498694528	0.583706356
	403.2701	-3.7268014	-0.8047314	0.007389717	0.033319127	0.47729955	-1.0670331	0.48612593	-1.040597992	0.396642022	0.09852928	0.702001898	0.956913237	1.133059494	0.180223616	0.982576168	-0.025358848	-2.21283214	-0.667617141	0.062535298	0.236835809	0.49987714	-1.000354543	0.567143484	-0.81821432
	362.2915	-3.8863805	-0.7857362	0.006003352	0.029830525	0.50569961	-0.9836474	0.52443929	-0.93115232	0.339330943	0.098749438	0.743096465	0.961972151	1.117577953	0.160375465	0.954573921	-0.067071173	-5.249702293	-0.817234746	0.001186606	0.016247376	0.443034841	-1.174507935	0.47623202	-1.070263468
	400.2534	-2.7169473	-1.0595655	0.029897215	0.098550078	0.36579683	-1.4508855	0.46178645	-1.114702244	0.088789046	0.071284517	0.931432065	0.998232252	1.700000208	0.765534923	2.105555273	1.074200748	-0.655846602	-0.338482932	0.532878763	0.78715701	0.656648512	-0.606806757	0.657828282	-0.60421706
11-HETE [M-	379.2473	-3.6558141	-0.4903534	0.008115176	0.0356667	0.59190735	-0.7565567	0.65003606	-0.621408349	0.521734437	0.146465871	0.615987951	0.92287782	1.308831277	0.388279129	0.959995488	-0.05890047	-3.598714612	-0.570725258	0.008754804	0.054679124	0.524732986	-0.93034461	0.610892861	-0.711008713
	250.1528	-3.8825445	-1.0717615	0.00603314	0.029830525	0.38102321	-1.3920492	0.42644287	-1.229575617	0.192197711	0.057231365	0.852376432	0.998232252	1.220683011	0.287688608	0.831332828	-0.266501913	-4.375859086	-1.222477403	0.003250797	0.027554378	0.319918205	-1.644225004	0.368116198	-1.441766863
	459.2344	-1.9386917	-0.4685516	0.093715381	0.24175852	0.58434949	-0.7750966	0.64218088	-0.638948395	0.377959939	0.110006417	0.715291685	0.959250658	1.319103134	0.399557366	0.917511262	-0.124202229	-1.598605591	-1.207311335	0.153940622	0.41206366	0.556526615	-0.845477411	0.702389502	-0.509656813
	298.2469	-1.8581068	-0.799066	0.105504296	0.262654051	0.403428	-1.3096169	0.49432345	-1.016472737	0.224833841	0.036247231	0.827745053	0.998232252	1.15249383	0.204759027	0.823359921	-0.280404871	-0.703781839	-0.444442959	0.504299181	0.763959611	0.618567675	-0.692996652	0.839464432	-0.252458895
5-HETE [M-H	379.2471	-2.9885613	-0.5625354	0.02026759	0.072152622	0.56961706	-0.8119357	0.63218472	-0.66158192	0.118811397	0.037576049	0.908354	0.998232252	1.26896635	0.343653813	0.863947613	-0.21098426	-1.57807512	-0.459737915	0.15855613	0.416827631	0.59183159	-0.756741391	0.711894474	-0.490264692
[truncated: 3,376 more chars]
